# Genome Evolutionary Dynamics Meets Functional Genomics: A Case Story on the Identification of SLC25A44

**DOI:** 10.3390/ijms22115669

**Published:** 2021-05-26

**Authors:** Behrooz Darbani

**Affiliations:** 1 The Novo Nordisk Foundation Center for Biosustainability, Technical University of Denmark, 2800 Kgs. Lyngby, Denmark; bd@agro.au.dk or behroozdarbani@gmail.com; Tel.: +45-(53)-578055; 2 Research Center Flakkebjerg, Department of Agroecology, Aarhus University, 4200 Slagelse, Denmark

**Keywords:** electron transfer chains, candidate gene, flavonoids, gene clusters, gene organization, genomic co-localization, *para*-coumaric acid, resveratrol, SLC25 subfamily 44, ubiquinone

## Abstract

Gene clusters are becoming promising tools for gene identification. The study reveals the purposive genomic distribution of genes toward higher inheritance rates of intact metabolic pathways/phenotypes and, thereby, higher fitness. The co-localization of co-expressed, co-interacting, and functionally related genes was found as genome-wide trends in humans, mouse, golden eagle, rice fish, *Drosophila*, peanut, and *Arabidopsis*. As anticipated, the analyses verified the co-segregation of co-localized events. A negative correlation was notable between the likelihood of co-localization events and the inter-loci distances. The evolution of genomic blocks was also found convergent and uniform along the chromosomal arms. Calling a genomic block responsible for adjacent metabolic reactions is therefore recommended for identification of candidate genes and interpretation of cellular functions. As a case story, a function in the metabolism of energy and secondary metabolites was proposed for *Slc*25A44, based on its genomic local information. *Slc*25A44 was further characterized as an essential housekeeping gene which has been under evolutionary purifying pressure and belongs to the phylogenetic ETC-clade of SLC25s. Pathway enrichment mapped the *Slc*25A44s to the energy metabolism. The expression of peanut and human *Slc*25A44s in oocytes and *Saccharomyces cerevisiae* strains confirmed the transport of common precursors for secondary metabolites and ubiquinone. These results suggest that SLC25A44 is a mitochondrion-ER-nucleus zone transporter with biotechnological applications. Finally, a conserved three-amino acid signature on the cytosolic face of transport cavity was found important for rational engineering of SLC25s.

## 1. Introduction

Knockout, RNAi, and overexpression libraries, classical genetics, transcriptomics, and proteomics are the mostly used, but also very laborious, approaches to inferring the functions of genes. The application of gene clusters, although it goes back to the late 1980s [1], nowadays is becoming a promising approach for functional mapping of genes located within biosynthetic gene clusters [2,3,4,5]. This study generalizes the approach by revealing sharp genome-wide co-localization patterns for co-expressed and co-interacting genes in plant and animal genomes. It also highlights the genomic co-localization of functionally related genes in several species. Co-localization of genes which are responsible for different steps of a metabolic pathway, leads to a higher frequency of the intact pathway among the offspring. This is due to the co-segregation of the co-localized active alleles to the next generations. Individuals with co-localized genes responsible for a decisive phenotype in the struggle for life, therefore, outcompete those with the randomly distributed responsible genes.

As a case story, the study follows the same concept and reports the functional identification of solute carrier family 25 subfamily 44 (*Slc*25A44). Known as mitochondrial carriers, the SLC25 transporters are only present in eukarya, as recently confirmed by genome-wide transportome analysis of 249 species [6]. The SLC25s, as key players of energy metabolism, are not restricted to the mitochondria [7] and participate in the metabolite trafficking in and out of ER [8], peroxisomes [9], and plastids [10]. Despite their importance, the function for nearly half of SLC25s is still unknown. The analyses found the flavonoid and stilbenoid biosynthetic pathway genes and the genes coding for subunits of mitochondrial electron transfer chains (ETC), such as ubiquinone oxidoreductases co-localized with *Slc*25A44. It was hypothesized that SLC25A44 may be transporting the common precursors of the ubiquinone which is the electron shuttle in ETC and the secondary metabolites such as resveratrol. This was further confirmed by downstream functional analyses of the peanut and human SLC25A44s in *Xenopus* oocytes and *S. cerevisiae* strains.

## 2. Results

### 2.1. Genome-Wide Evidence for Co-Localization of Co-Interacting, Co-Expressed, and Functionally Related Genes

Genomic co-localization facilitates co-segregation of genes, and this can be evolutionary decisive in the struggle for life when the co-localized genes are cooperatively required for a higher fitness [2,11]. We have previously used the information of biosynthetic gene clusters for identification of membrane transporters [2,3]. To establish a common approach, two strategies were carried out to investigate the presence of an evolutionary genome-wide pattern for co-localization of co-interacting, co-expressed, and functionally related genes. In the first approach, visual genome scanning found several co-localized functionally related genes coding for membrane transporters and other proteins in two plant and three animal species of *Arachis duranensis*, *Arabidopsis thaliana*, *Homo sapiens*, *Oryzias latipes*, and *Aquila chrysaetos* (Table S1 and S2). The co-localized events with genes engaged in energy metabolism was the prominent type of genomic blocks (Table S1). Other more-interesting events were involved in the transport and metabolism of (i) amino acids, (ii) nucleotides, and (iii) minerals (Tables S1 and S2). For example, the co-localization of magnesium transporters and DNA/RNA polymerases agrees with the essentiality and the transient co-factor role of magnesium in DNA/RNA polymerization. One of the genomic blocks in the animal genomes had the co-localized genes coding for the metal chelating CNNM1, aspartate aminotransferase GOT1, mitochondrial iron transporter SLC25A28, copper homeostasis protein CUTC, cytochrome C oxidase assembly protein COX15, and the bilirubin exporter ABCC2. This remarkable co-localization has potential neuropathological importance for the transition metal overloading disease of Alzheimer’s that has characteristics of hampered oxidative energy metabolism and glutamate/GABA-glutamine cycle. GOT1 is a neuroprotectant enzyme by being involved in the production of hydrogen sulfide and the neurotransmitter “glutamate” [12,13]. Among the others, the co-localization of genes coding for auxin transporter and auxin response factors in plants is also notable.

**Figure 1:**
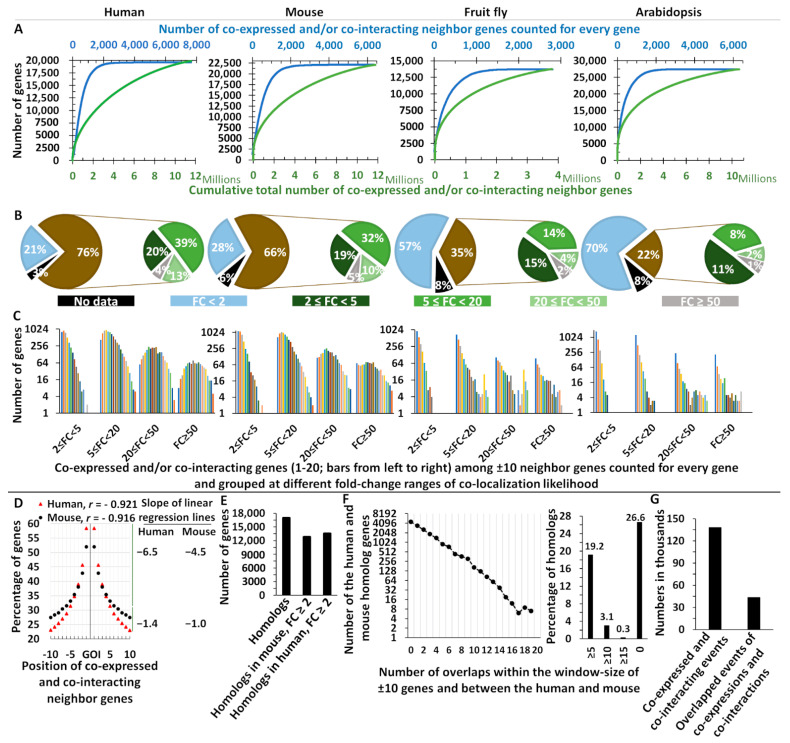
Genome-wide trends in co-localization of co-expressed and co-interacting genes. (**A**) Number of co-expressed and co-interacting genes counted for every gene. The blue color is for the total number and the green color is for the cumulative number of events. (**B**) Percentage of genes with different fold-changes in likelihoods of co-localization with co-expressed and co-interacting partner genes. For every gene, the co-localization likelihood, i.e., the observed likelihood for co-expressed and co-interacting gene(s) located among the ±10 neighbor genes, was compared to its observed genome-wide likelihood of co-expressed and co-interacting genes, and fold-changes of ≥2 were considered as non-random distributions (see the methods). Homologs and duplicated events were excluded from the analyses. (**C**) Genes with co-localization likelihood fold-change of ≥2 had 1–20 co-expressed and co-interacting partner genes within the genomic window size of ±10 genes. (**D**) The co-localization likelihood of co-expressed and co-interacting genes across the inter-loci distances. GOI is the gene of interest and represents every gene in the genome. (**E**) Homolog genes between the human and mouse with co-localization likelihood fold-change of ≥2. The analyses were based on 16,974 homologs, and 254 homologs with missing data were excluded. (**F**) The frequency of overlapped co-expressed and co-interacting neighbor genes with identical physical gene order positions when comparing the human and mouse. The analyses were based on the 16,974 homolog genes between the human and mouse and within the genomic window size of ±10 genes from the homolog genes. (**G**) Total number of neighbor events and the number of overlapped neighbor events with identical physical gene order positions, both within the genomic window size of ±10 genes from the homolog genes, when comparing the human and mouse. (**E**–**G**) To exclude the effect of possible segmental inversions and differences in chromosomal orientations between the human and mouse, inverted but exactly same events were extracted and included in the analyses as overlapped events. FC: Fold change in the co-localization likelihood against the genome-wide likelihood, GOI: gene of interest.

In the second approach, publicly available gene co-expression and protein co-interaction data [14] of *H. sapiens*, *M. musculus*, *Drosophila melanogaster,* and *A. thaliana* were used to determine whether the co-expressed and co-interacting genes are randomly distributed along the chromosomes or the genomic co-localization of them has evolutionary been shaped. The analyses revealed sharp co-localization trends in all four species (Figure 1). More than 98% of the genes had less than 2000 co-expressed and co-interacting genes, accounting for 90–99% of the total number of paired co-expressions and co-interactions (Figure 1A). In addition, it was only 4–14% of the genes that had no more than 10 co-expressed and co-interacting genes. The genomic co-localization trends of co-expressed and co-interacting partner genes were found for considerable number of the genes, from 22% of the *Arabidopsis* genes to 76% of the human genes (Figure 1B and Table S3). The co-localization likelihood for these genes was at least two times higher than the random distribution; the observed probability of the co-expressed and co-interacting gene(s) being located within the genomic window size of ±10 genes from a gene of interest was ≥2 times higher than the observed probability of the co-expressed and co-interacting gene(s) being found in the genome for the same gene of interest. Of these genes that were co-localized with their co-expressed and co-interacting genes, 50% in *Arabidopsis* to 75% in humans had co-localization likelihood fold-change of ≥5 when compared to random distribution (Figure 1B and Table S3). Overall, 97–98% of the genes with co-localization likelihood fold-change of ≥2 had 10–2000 co-expression and co-interaction gene partners. As expected, there was a bias toward the lower number of co-expressed and co-interacting genes located within the genomic window size of ±10 neighbor gene, and this was more pronounced for the co-localization likelihood fold-change of 2 ≤ FC < 5 (Figure 1C and Table S3). However, co-localization events with 1–20 co-expressed and co-interacting neighbor genes were found in all the higher co-localization likelihood fold-changes of 5 ≤ FC < 20, 20 ≤ FC < 50, and 50 ≤ FC (Figure 1C). Approximately, 43% and 30% of genes had more than four co-expressed and co-interacting neighbor genes within the window size of ±10 genes in the human and mouse genomes, respectively. The distribution of genes with co-localization likelihood fold-change of ≥2 was also examined in human. There was an even distribution along the chromosomal arms (Figure S1).

It is worth noting that the simple roll of “the shorter distance between the genes of a given pathway the higher fitness for the organism” applies because recombination rate positively depends on the inter-loci distance (Figure 1D). Accordingly, the likelihood of co-expressed and co-interacting genes sharply declined from 58.4% to 31.5% and from 52% to 33.2% by moving from the nearest neighbor gene to the fifth in the human and mouse, respectively (Figure 1D). This smoothly continued to the levels of 23.1–27.5% by moving toward the 10th neighbor gene-position in the human and mouse, respectively (Figure 1D). The higher recombination rates at distant neighborhood in the human genome are consistent with the lower gene density within the human genome (157 kb per gene) in comparison with mouse (123 kb per gene). Therefore, shorter inter-loci distances lessen recombination rates and thereby increase the likelihood of intact pathways to be passed down across generations. As proposed for the gene clusters [11], the analyses also revealed convergent evolution for the genomic blocks. The homolog genes between the human and mouse were further analyzed. Up to 80% and 76% of the 16974 homolog genes had co-localization likelihood fold-change of ≥2 in humans and mouse, respectively (Figure 1E). There were 12,451 homolog genes with one to 19 overlaps, i.e., co-expressed and co-interacting neighbor genes were located at similar physical gene order positions within the genomic window size of ±10 genes, when comparing the human with mouse (Figure 1F). This also includes 19.18% of the homolog genes having at least five overlaps in their neighborhood when comparing the human with mouse (Figure 1F). Taken together, there were only 15 homolog genes each with all their co-expressed and co-interacting neighbor genes, completely overlapped when comparing the human with mouse (Figure 1F). Considering all the analyzed co-expression and co-interaction neighbor events of 16,974 homolog genes, 68.6% (94,689) of the events were not found similar between the human and mouse (Figure 1G). These results shed light on the convergent evolution of the co-localization of co-expressed and co-interacting genes.

**Figure 2:**
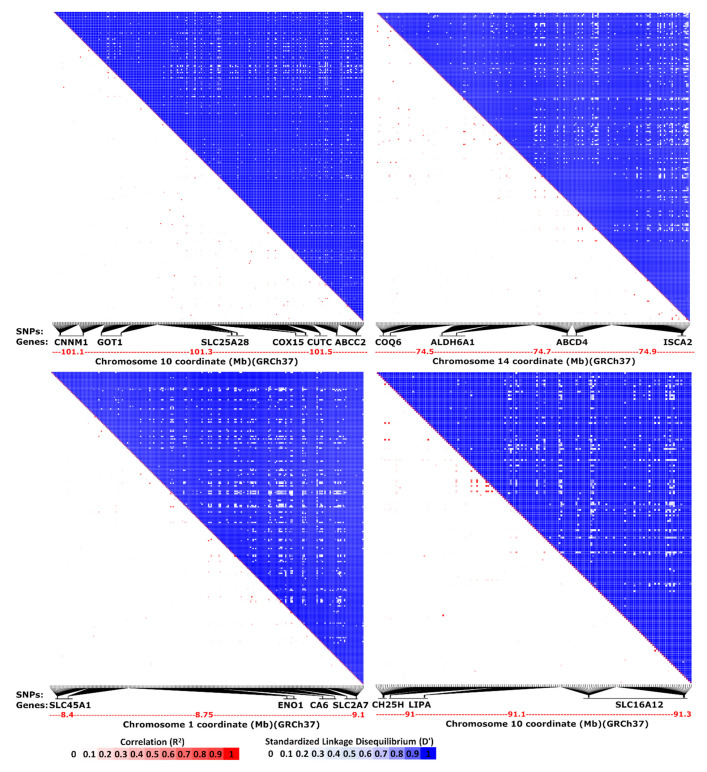

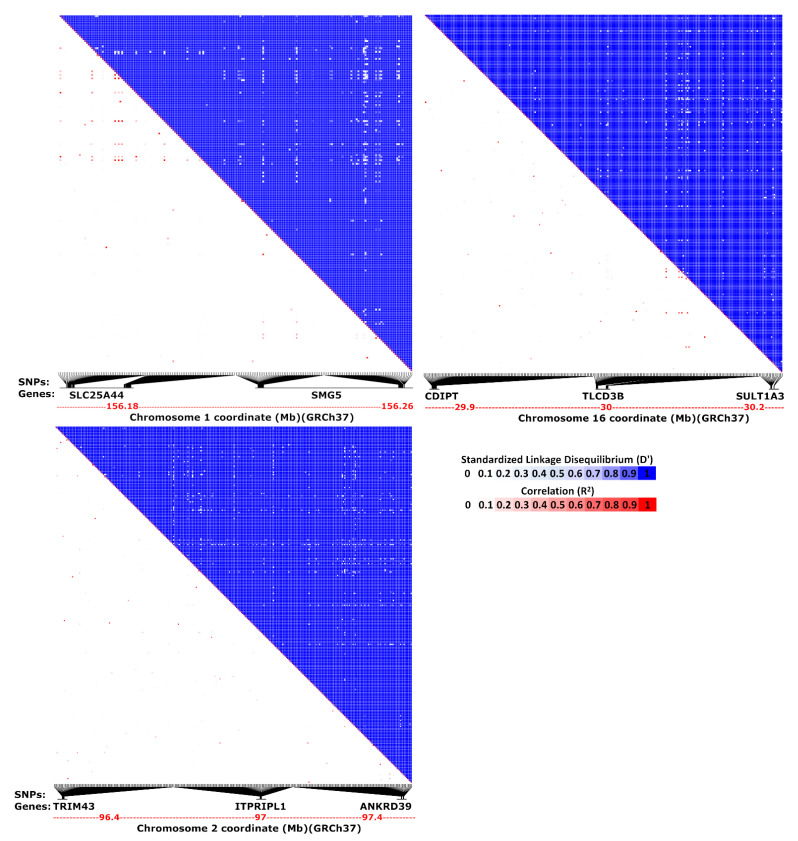
Co-segregation of the co-localized events. The heatmap matrices of pairwise linkage disequilibrium statistics are shown. The analysis includes the SNPs in the flanking regions of the genes. The TLCD3B and ITPRIPL1 genes had interactions with all the 20 neighbor genes, and the co-segregation is demonstrated for the most distant neighbors. Complete linkage: D’ = 1, Random segregation: D’ = 0.

To confirm the co-segregation of the co-localized events, five clusters from functionally related genes and two clusters with 20 + 1 interacting neighbor genes, all identified within the human genome, were analyzed for linkage disequilibrium. The human 1000 genomes project data [15], which includes more than 84 million SNPs, were applied. For every gene in the analyses, 60–100 SNPs from the 5′- and 3′-flanking regions were considered. The results confirmed the co-segregation of the clustered alleles (Figure 2). As expected, the correlation did not show any considerable co-occurrence, which was due to the lower frequency of the rare variants. However, the standardized linkage disequilibrium patterns clearly demonstrated the co-segregation of the variants (Figure 2).

### 2.2. A Case Story on the Identification of Slc25A44

**Figure 3:**
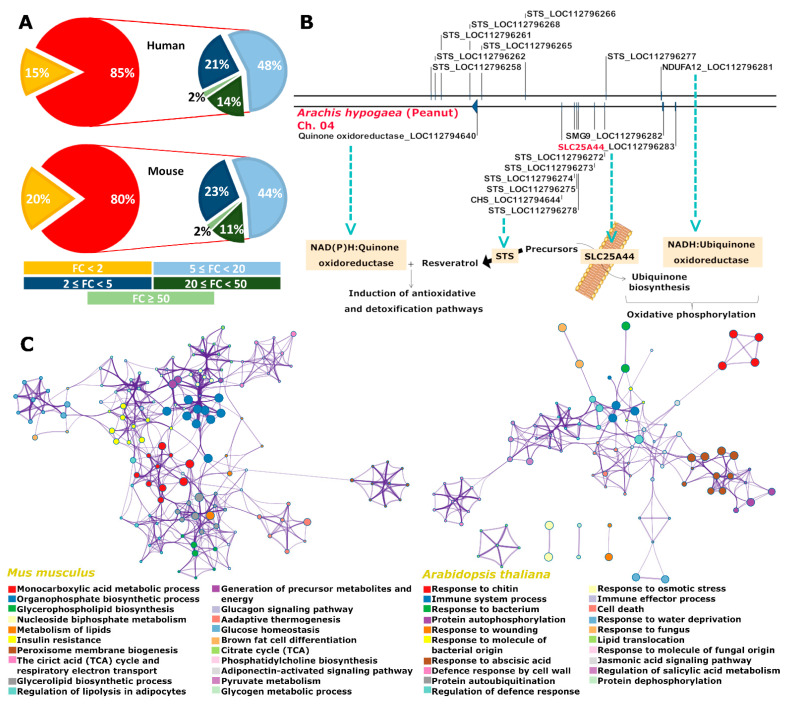

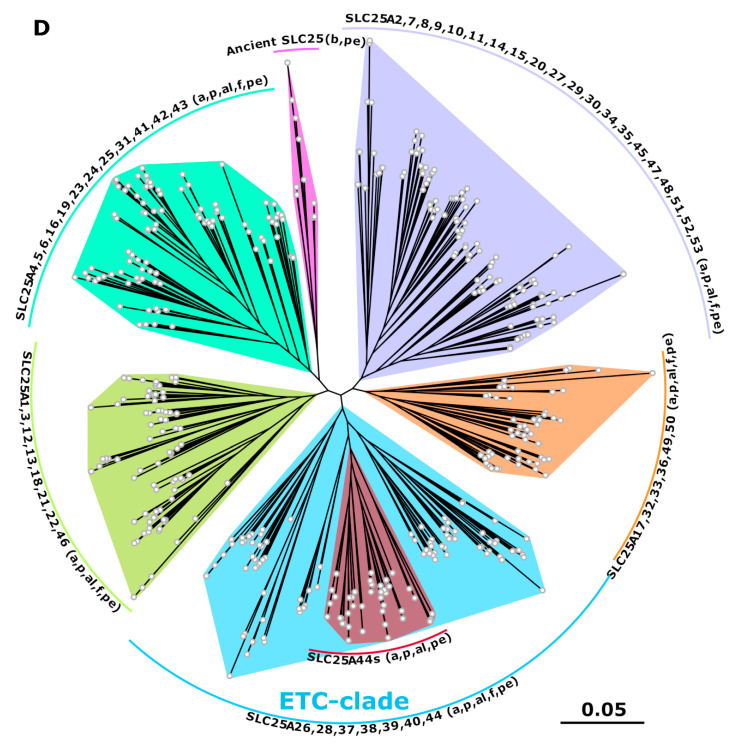
Identification of SLC25A44 as a candidate transporter for the common precursors of ubiquinone and resveratrol. (**A**) The co-localization of membrane transporter genes with their co-expressed and co-interacting partner genes within the genomic window size of ±10 genes. (**B**) The identified genomic block in peanut. The metabolic relation among the genes is shown. (**C**) Metabolic pathway enrichment for the genes co-expressed with *Slc*25A44. (**D**) The phylogenetic tree of mitochondrial carriers. The tree includes SLC25 members from four primitive eukaryotes, four fungal species, two animals, one plant and one algal species, ancient SLC25 members from five bacterial species, and finally members of SLC25A44 from additional 18 animals and seven plant and algal species. See Table 1 and Table S5 for the species names and accession number of sequences. The clades are labeled by letters “a” for animal, “p” for plant, “f” for fungi, “pe” for primitive eukaryotes, and “b” for bacteria when there is at least one representative transporter member from the corresponding domains of life. CHS: chalcone synthase, ETC: electron transfer chains, FC: fold change in co-localization likelihood, NDUF: NADH ubiquinone oxidoreductase, and STS: resveratrol synthase.

There has been increasing evidence for the biosynthetic gene clusters with non-biosynthetic transporter genes. These transporters are responsible for the transport of precursors, intermediates, or final products of the metabolic pathways governed by the biosynthetic gene clusters [2,16]. To generalize the concept, the membrane transporter coding genes have been found under evolutionary selection to be co-localized together with their co-expressed and co-interacting genes (Figure 3A). More than 80% of the membrane transporter coding genes had co-localization likelihood fold-change of ≥2 for their co-expressed and co-interacting neighbor genes (Figure 3A). By employing genomic local functions, *Slc*25A44 was therefore proposed as a candidate transporter gene for the common precursors of ubiquinone, flavonoids, and stilbenoids.

Resveratrol synthases, chalcone synthases, mitochondrial complex I subunit *Nduf*A12, quinone oxidoreductase, and *Slc*25A44 genes were found as neighbors in the genomes of peanut (*Arachis* *hypogaea*) and its diploid ancestors *A. duranensis* and *A. ipaensis* (Figure 3B and Figure S2). In grape (*Vitis* *vinifera*) and other plant species from Fabaceae, Cucurbitaceae, and Rosaceae families, *Slc*25A44 only co-localized with the genes coding for the mitochondrial ETC subunits (Table 1). Co-localization of the flavonoid pathway genes (resveratrol synthases, chalcone synthases, and 4-coumarate-CoA ligases) and ETC subunit coding genes was also observed on different chromosomes of grape and four species from Fabaceae (Table 1). In contrast, there was not any similar genomic arrangement in the plant species *Oryza sativa*, *Gossypium hirsutum*, *Arabidopsis thaliana*, *Brassica oleracea*, and *Capsella rubella*. Evolving of such genomic blocks was also investigated in nine animal species including *Homo sapiens*, *Mus musculus*, *Gorilla gorilla gorilla*, *Bos taurus*, *Xenopus laevis*, *Drosophila melanogaster*, *Cimex lectularius*, *Halyomorpha halys*, and *Caenorhabditis elegans*. *Slc*25A44 was only found adjacent to the mitochondrial complex III subunit UQCRQ coding gene in the genome of the insect *H. halys* (Table 1). The existence of genomic blocks with different combinations of genes, all implicated in the same metabolic pathway, again highlights the convergent evolution of the co-localization events.

Using the grape berries transcriptome data [17], the co-upregulation of *Slc*25A44 and the resveratrol biosynthesis pathway genes of phenylalanine ammonia-lyase (*Pal*) and resveratrol synthases were further noticed. Therefore, co-expression and pathway enrichment analyses were applied for *Slc*25A44 using the *A. thaliana* and mouse public data. AtSlc25A44 was mainly associated with biotic and abiotic stress responses and energy metabolism (Figure 3C and Table S4). *MmSlc*25A44 was also implicated in energy metabolism (Figure 3C and Table S4).

### 2.3. Slc25A44 Is a Highly Conserved Housekeeping Transporter Gene

**Figure 4:**
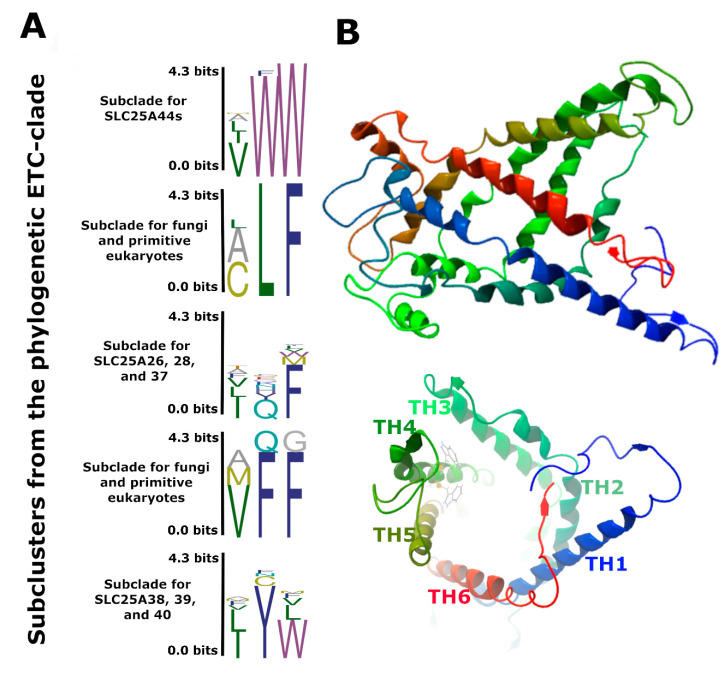

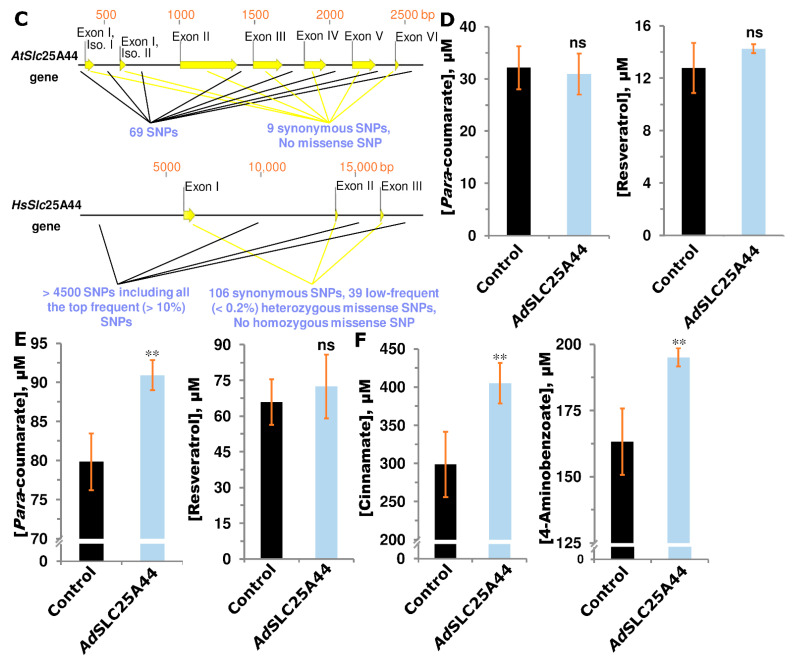
The highly conserved *Slc*25A44 gene has transport activity when expressed in *X. laevis* oocytes. (**A**) The conserved three-amino acid signature in SLC25A44s and four other subclades within the phylogenetic ETC-clade. (**B**) The predicted whole and cytosolic face structure of the *Ad*SLC25A44. Transmembrane helices (TH) and conserved tryptophan residues on TH4 are illustrated. (**C**) The structure of locus and polymorphism distribution for *Arabidopsis* and human *Slc*25A44s. (**D**–**F**) Functional transport studies by expressing *Ad*SLC25A44 in oocytes. (**D**) Export assay: resveratrol and *para*-coumaric acid were injected into the oocytes and were quantified in the medium after 210 min. (**E**,**F**) Import assay: resveratrol and *para*-coumaric acid (**E**) or cinnamic and 4-aminobanzoic acids (**F**) were added into the medium and intracellular levels of these compounds in oocytes were quantified after 210 min. (**D**–**F**) Bars represent mean ± Std. *n* = 3–4 biological independent samples each with 20 (**D**) or 30 (**E**,**F**) oocytes. The two-tailed Student’s *t*-test was used to find the significant transport activities at 1% level (marked by two asterisks) when compared to the control with no heterologous expression (RNA-free solution was injected). ns: not significant.

The SLC25s have diverged massively, and in humans, they consist of 53 subfamilies. We have previously found the bacterial ancient SLC25s as the origin of the eukaryotic SLC25s [6]. In agreement, primitive eukaryotes code for homologs of the ancient SLC25s (Figure 3D). Phylogenetic analysis identified five major clades of eukaryotic SLC25s (Figure 3D). The SLC25A44 was in a clade with the S-adenosylmethionine transporter (SLC25A26) [18], the iron transporters of SLC25A28 and A37 [19], and the uncharacterized transporters of SLC25A38, A39, and A40 (Figure 3D). The SLC25A38 and A39 have been reported to be associated with heme biosynthesis [20,21]. The clade was called ETC-clade. This is due to the structural and functional dependency of ETC on the S-adenosylmethionine [22] as well as iron and heme [23,24,25]. Interestingly, the *Slc*25A28 and *Slc*25A38 also co-localized with the genes coding for ETC and ATP-synthase subunits (Table 1). Further analyses identified a conserved three-amino acid signature (V/I/L/AWW) for SLC25A44s (Figure 4A). This signature was distinguishable among the phylogenetic sub-clusters of the ETC-clade, thereby promising motif for transport engineering (Figure 3D and Figure 4A). The signature is situated onto the cytosolic face of transport cavity at the C-terminal end of transmembrane helix 4 (Figure 4B). Finally, *Slc*25A44 had no clear fungal homolog, and thereby, highly diversified orthologues are conceivably functioning in fungi (Figure 3D and Figure S3A). The yeast *Sc*AGC1 was the most immediate blast hit for SLC25A44 members (Figure S3B). The *Agc*1 was also found as neighbor to the genes coding for mitochondrial respiratory subunits in fungal species (Table 1).

Inspection of the human transcriptome [26] revealed the ubiquitous expression of *HsSlc*25A44 (Figure S4). Analyzing the *A. thaliana* proteome [27] and transcriptome data [28] also revealed the constitutive expression of *AtSlc*25A44 at levels similar to the housekeeping genes *Ubc*9 and *Gapdh* across hundreds of different conditions and tissues (Figure S5A–G). In addition, *Slc*25A44 was found highly conserved at the intra-species level with the majority of polymorphism in its non-coding regions (Figure 4C). There was no missense mutation for *Slc*25A44 in different lines of *A. thaliana*, which is a self-fertilizing homozygote plant (Figure 4C). In addition, the human *Slc*25A44 had only 3% of the variants within the coding regions; less than 0.8% were missense variants, all as very low-frequent and heterozygous-only SNPs (Figure 4C). Similar patterns were also observed for the mouse and rice *Slc*25A44s (Table S6). The data indicate that SLC25A44 is essential in higher eukaryotes. Accordingly, the non-synonymous/synonymous substitution rate ratio was 0.0528 (Log(L) = −22,531) for SLC25A44 orthologs from 129 examined species within the phylogenetic branch of vertebrates. There was only one amino acid site with asymptotic diversification *p*-value of 0.01. The gene-wide analysis of selection also showed a similar evolutionary pattern of purifying selection (Table 2).

### 2.4. Functional Characterization of AdSlc25A44 in Xenopus Oocytes

The *AdSlc*25A44 was expressed in *X. laevis* oocytes for functional analysis. The heterologous vacuolar and mitochondrial transporters are expressed, at least partially, in the plasma membrane of oocytes due to the very high-level translational capacity of oocytes, and this allows for their characterization [2,3,29]. For the export assay, a solution consisting of resveratrol and *para*-coumaric acid was injected into the oocytes to a final intracellular concentration of ca. 2 mM each, and the exported compounds into the Kulori buffer were quantified after 3.5 h. The expression of *AdSlc*25A44 had no significant efflux activity for resveratrol and *para*-coumaric acid (Figure 4D). In contrast, the import assay with Kulori buffer containing resveratrol and *para*-coumaric acid (0.2 mM each) showed *para*-coumaric acid transport activity for *AdSlc*25A44. Here, oocytes expressing *AdSlc*25A44 contained 14% more *para*-coumaric acid than the control oocytes with no heterologous transporter (*p* = 9.6 × 10^−3^; Figure 4E). *AdSlc*25A44 was also examined for the import of cinnamic and 4-aminobenzoic acids, and similar transport patterns were found (Figure 4E,F). Oocytes expressing *AdSlc*25A44 had 35% (*p* = 5.4 × 10^−3^) and 20% (*p* = 2.7 × 10^−3^) higher intracellular levels of cinnamic and 4-aminobenzoic acids, respectively (Figure 4F). There was no significant resveratrol-influx activity for *AdSlc*25A44 (Figure 4E). The lack of export activity could be explained due to the absence of a co-substrate in the buffer and the conceivable antiport activity, which is a common mechanism for the SLC25 members [30].

### 2.5. SLC25A44 Localized onto the Mitochondria, ER, and Nucleus of Yeast and Had Impact on the Production of Para-Coumaric, 4-Aminobenzoic, and 4-Hydroxybenzoic Acids

**Figure 5:**
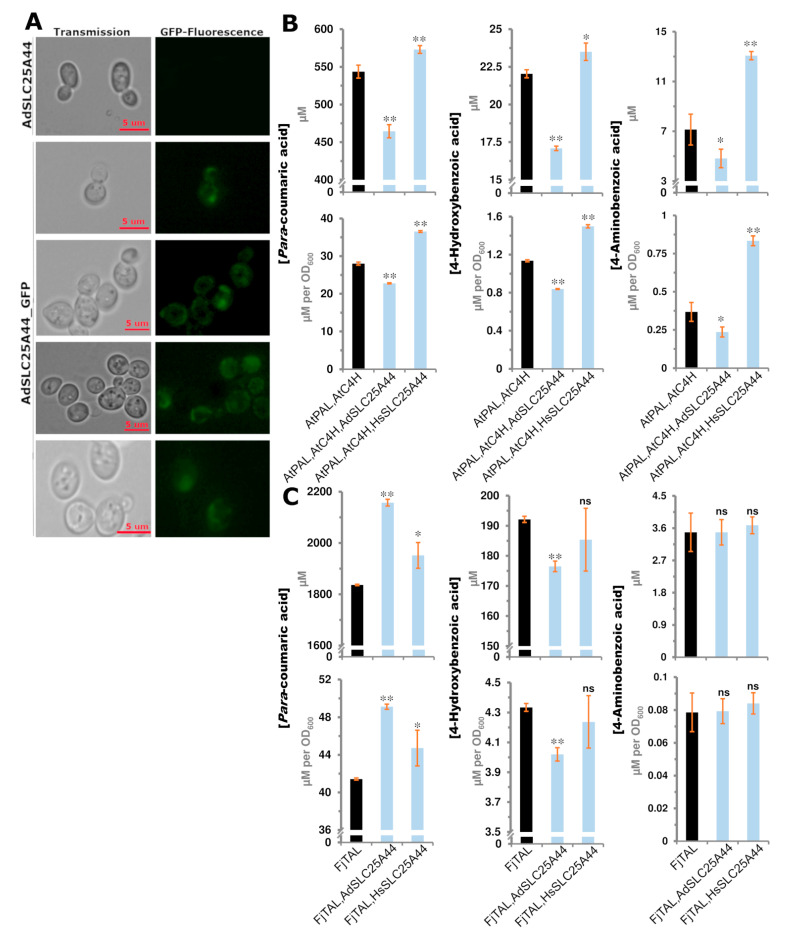
The transport activities of *Ad*SLC25A44 and *Hs*SLC25A44 in *S. cerevisiae*. (**A**) Subcellular localization of *Ad*SLC25A44_GFP in yeast. (**B**,**C**) *Para*-coumarate, 4-aminobenzoate, and 4-hydroxybenzoate concentrations in the fermentation broth after 72 h growth of the yeast strains producing *para*-coumaric acid from phenylalanine by the enzymes PAL and C4H (**B**) or from tyrosine by the enzyme TAL (**C**). Bars represent mean ± Std; *n* = 3 biological independent samples. The two-tailed Student’s *t*-test was used to compare the strains with and without the heterologous transporters. The significant events at 5% and 1% levels are labeled by one and two asterisks, respectively. C4H: Cinnamic acid hydroxylase, PAL: Phenylalanine ammonia-lyase, TAL: Tyrosine ammonia-lyase, and ns: not significant.

The human SLC25A44 has previously been mapped on mitochondrion, ER, and nucleus [26]. To examine the localization of *Ad*SLC25A44, a C-terminal fused GFP version of *Ad*SLC25A44 was expressed in *S. cerevisiae*. The observed sub-cellular localization pattern (Figure 5A) was similar to the previously reported yeast mitochondrial, ER, and nucleus membrane proteins such as CTP1, OXA1, YHM2, APQ12, ERD2, and BRR1 [31]. *S. cerevisiae* strains, called PAL and TAL, were built to produce *para*-coumaric acid via different pathways for functional analysis of SLC25A44. In the PAL strain, phenylalanine ammonia-lyase (*AtPal*2) and cinnamic acid hydroxylase (*AtC*4h) genes were expressed to produce *para*-coumaric acid from phenylalanine as reported previously [32]. The expression of *AdSlc*25A44 in the PAL strain reduced the extracellular titers and specific yield of *para*-coumaric acid by 15% (*p* = 3 × 10^−4^) and 19% (*p* = 3 × 10^−5^), respectively (Figure 5B). The production of related shikimate pathway intermediates including cinnamic, 4-aminobenzoic, and 4-hydroxybenzoic acids [33,34] were additionally examined. As a result of *AdSlc*25A44 expression, the titer and specific yield decreased up to 23% (*p* = 9.6 × 10^−6^) and 27% (*p* = 1.9 × 10^−6^) for 4-hydroxybenzoic acid and up to 33% (*p* = 4.9 × 10^−2^) and 36% (*p* = 

3.1 × 10^−2^) for 4-aminobenzoic acid, respectively (Figure 5B). There was no detectable export for cinnamic acid from the PAL strain. The *AdSlc*25A44 was further examined in the TAL strain which was designed for the biosynthesis of tyrosine-derived *para-*coumaric acid by expressing *Flavobacterium johnsoniae* tyrosine ammonia-lyase (*FjTal*) as reported previously [35]. In the TAL strain, *AdSlc*25A44 improved the *para*-coumaric acid extracellular titer and specific yield by 18% (*p* = 2 × 10^−6^) and 19% (*p* = 1.5 × 10^−6^), respectively (Figure 5C). However, the expression of *AdSlc*25A44 decreased the extracellular titer and specific yield of 4-hydroxybenzoic acid by 9% (*p* = 1.8 × ^−4^) and 8% (*p* = 4.6 × 10^−4^), respectively (Figure 5C). Here, *AdSlc*25A44 had no significant effect on the extracellular level of 4-aminobenzoic acid (Figure 5C).

To examine whether these transport activities are common or not, the human *HsSlc*25A44 was expressed in the PAL and TAL strains. The expression of *HsSlc*25A44 had a similar effect as the expression of *AdSlc*25A44 in the TAL strain. Here, the titer and specific yield of *para*-coumaric acid increased by 6% (*p* = 1.6 × 10^−2^) and 8% (*p* = 4 × 10^−2^), respectively (Figure 5C). In contrast to the *AdSlc*25A44, the expression of *HsSlc*25A44 in the PAL strain improved the titers and specific yields of *para*-coumaric acid up to 6% (*p* = 7 × 10^−3^) and 30% (*p* = 6 × 10^−6^), 4-hydroxybenzoic acid up to 7% (*p* = 1.6 × 10^−2^) and 32% (*p* = 6.5 × 10^−6^), and 4-aminobenzoic acid up to 83% (*p* = 1 × 10^−3^) and 127% (*p* = 3 × 10^−4^), respectively, in the fermentation broth (Figure 5B). Overall, the transporters had similar effects, except for *Ad*SLC25-44 when it was expressed in PAL strain. This difference could be attributed to the interactions of *para*-coumaric acid biosynthesis in ER of PAL strain or cytosol of TAL strain [36] with the expected different distribution rates of *Ad*SLC25A44 and *Hs*SLC25A44 among mitochondria, ER, and nucleus.

### 2.6. Rational Transporter Engineering toward Higher Production of Para-Courmaric Acid

**Figure 6:**
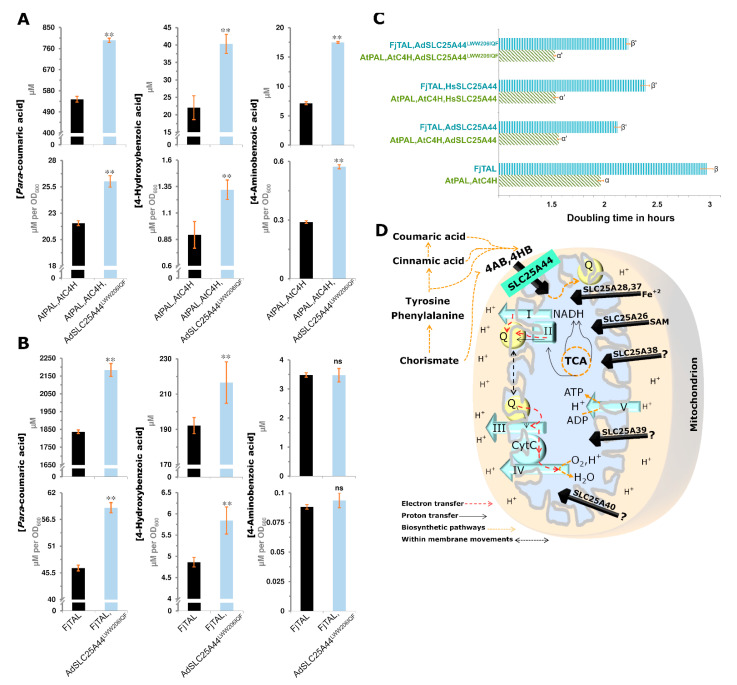
The SLC25A44 is a transporter of ubiquinone precursors with potential to boost the bio-based production of *para*-coumaric acid. (**A**,**B**) The effect of *Ad*SLC25A44^LWW206IQF^ expression on the production rate of *para*-coumaric, 4-hydroxybenzoic, and 4-aminobenzoic acids in the PAL and TAL yeast strains. Bars represent mean ±Std. *n* = 6–9 biological independent samples. The two-tailed Student’s *t*-test was used to compare the strains with and without *Ad*SLC25A44^LWW206IQF^. The significant events at 1% level are marked by two asterisks. (**C**) Logarithmic cell growth was measured spectrophotometrically at *A*_600_ nm. Significant differences, i.e., α vs. α′ and β vs. β′ (*p* = 10^−4^), were determined through one-way ANOVA followed by the least significant difference test. Bars represent mean ±Std. *n* = 3 biological independent samples. (**D**) A simplistic roadmap for the involvement of SLC25A44 and the other transporter members of ETC-clade in the mitochondrial electron transfer chains. 4HB: 4-hydroxybenzoate, 4AB: 4-aminobenzoate, I-IV: electron transfer chains complexes, CytC: cytochrome C, Q: ubiquinone, SAM: S-adenosylmethionine, and ns: not significant.

Here, the focus was on the identified conserved motif in the cytosolic face of SLC25A44s (Figure 4A,B). The VWW residues in *Hs*SLC25A44 occupy a smaller space than the LWW residues of *Ad*SLC25A44 due to the short side chain of valine. The corresponding residues in human ADP/ATP antiporter (SLC25A6) are AYF, all with smaller side chains. The LWW residues of the *Ad*SLC25A44 were then mutated into the IQF with smaller side chains. The IQF signature does also exist in the *Arabidopsis* plastid S-adenosylmethionine/S-adenosylhomocysteine antiporter (SLC25A26), which is the most immediate neighbor of SLC25A44 within the ETC-clade (Figure 3D and Figure 4A). The expression of *Ad*SLC25A44^LWW206IQF^ in the PAL and TAL yeast strains resulted in higher extracellular levels of *para*-coumaric acid (Figure 6A,B). *Para*-coumaric acid is a valuable chemical building block and a precursor of flavonoids and stilbenoids [35]. The expression of SLC25A44^LWW206IQF^ in PAL strain improved the titer and specific yield of *para*-coumaric acid by 46% (*p* = 2.1 × 10^−16^) and 18% (*p* = 3.6 × 10^−10^), respectively (Figure 6A). The titer and specific yield of *para*-coumaric acid also increased by 19% (*p* = 6.1 × 10^−12^) and 27% (*p* = 1.2 × 10^−12^), respectively, in the TAL strain expressing SLC25A44^LWW206IQF^ (Figure 6B). Furthermore, the expression of SLC25A44^LWW206IQF^ enhanced the production of 4-hydroxybenzoic acid in the PAL (83%, *p* = 4.2 × 10^−9^) and TAL (20%, *p* = 5.8 × 10^−6^) strains (Figure 6A,B). The production of 4-aminobenzoic acid was improved only in the PAL strain (Figure 6A,B). Importantly, the expression of the SLC25A44 transporters not only enhanced the production but also improved the growth rate of yeast PAL and TAL strains (Figure 6C).

## 3. Discussion

The evolutionary dynamics of gene organization opposes the random distribution hypothesis. In prokaryotes, not only the co-localization of genes within operons but also the order of genes has been under evolutionary pressures [37,38]. Some patterns have also been reported in eukaryotes [39]. In *S. cerevisiae*, 25% of 416 cell cycle dependent genes were found adjacent [40], and 18% of the 2081 examined adjacent gene-pairs fell into the same functional category [41]. In *Drosophila*, 35% of the 1661 testes-specific genes were also found within 187 clusters [42]. In contrast, the clustering of tissue-specific genes was not the case in humans but the clustering of highly expressed housekeeping genes [43]. Furthermore, gene co-localization preference was found for 30–98% of the 79–98 examined metabolic pathways in five eukaryotic species [44]. Despite these original discoveries, the lack of genome-wide analyses considering all genes, the lack of gene-pair analysis for distant neighbors, relying on correlation-based analyses which were not capable of comparing the genes for their co-localization rates, and finally, the lack of access to the interacting neighbor genes were among the things to be improved. Additionally, no application was conceived for such genomic blocks.

Here, the existence of co-localization patterns at genome scales by considering all genes, developmental stages, and tissues and, thereby, the application of such an evolutionary phenomenon for identification of candidate genes are addressed. The genomic local information within gene clusters, which has been shaped by the evolutionary preference for co-segregation of the genes [2,11], has recently been employed for functional identification of candidate membrane transporters [2,3]. Here, the study generalizes the concept by demonstrating several cases for co-localization of functionally related genes, including membrane transporters, in two plants and three animal species (Table S1 and Table S2). Furthermore, the analyses reveal genome-wide evolutionary tendencies for co-localization of co-expressed and co-interacting genes in humans, mouse, fruit fly, and *Arabidopsis* (Figure 1 and Table S3). While confirming the co-segregation of the events, the frequency of co-localization was negatively correlated with inter-loci distances (Figure 1D and Figure 2). The results, based on the human and mouse data, also confirmed that the genomic distances up to ±6 neighbor genes can secure 30% hunting-chance for a related gene (Figure 1D). Therefore, calling a genomic block involved in adjacent metabolic reactions is a promising approach and can bypass the laborious experiments required for the identification of candidate genes through the classical genetics or screening of knockdown/out libraries.

By applying the same strategy and based on the genomic neighborhood of *Slc*25A44 (Figure 3B and Figure S2), the *Slc*25A44 was proposed to link the primary metabolism of energy to the secondary metabolites. The downstream analyses and experiments confirmed the hypothesis by introducing the SLC25A44 as a transporter for the common precursors of ubiquinone, stilbenoids, and flavonoids (Figure 3, Figure 4, Figure 5 and Figure 6). A pivotal role in respiration is therefore proposed for SLC25A44s through the intracellular transport of ubiquinone precursors (Figure 6D). The biosynthesis of ubiquinone requires mitochondrial import of hydroxybenzoic acid or aminobenzoic acid, which are biosynthesized from tyrosine, phenylalanine, cinnamic acid, *para*-coumaric acid, or chorismate [45]. It is fatal to have defects in the biosynthesis of ubiquinone, which is essential for electron shuttling from the complex I (NADH oxidoreductase) and complex II toward the complex III of the ETC [46]. The essentiality of SLC25A44 for the indispensable biosynthesis of ubiquinone in higher eukaryotes has conceivably been the main evolutionary force for the observed purifying selection and the ubiquitous expression of *Slc*25A44s (Figure 4C, Figure S4 and Figure S5, Table 2 and Table S6). In agreement, down-regulation of *Slc*25A44 orthologue in *Trypanosoma brucei* led to lower levels of oxidative phosphorylation and ATP, growth arrest, and death [47]. Accordingly, the expression of SLC25A44 improved the growth rate in yeast (Figure 6C). Down-regulation of *Slc*25A44 orthologue also reduced ETC activity in *Caenorhabditis elegans* [48]. Additionally, SLC25A44 was found to be co-expressed with FOXK transcriptional regulators in mouse (Figure S6) and human (Figure S7), likely to improve the efficiency of ETC while glycolytic intermediates are mainly utilized for anabolic reactions. FOXK1 and FOXK2 favor aerobic glycolysis by uncoupling the glycolysis from oxidative phosphorylation [49]. It is therefore not surprising that *Slc*25A44 has the highest expression levels in brain, kidney, and liver (Figure S4), which are known as the most sensitive organs to mitochondrial disorders [30,50] and have the highest resting metabolic rates [51]. SLC25A44 has recently been reported to be involved in thermogenesis through the transport of the branched chain amino acids (BCAAs: leucine, isoleucine, and valine) to be catabolized within mitochondria specifically in the mouse fat cells and not muscle or liver [52]. The contribution of SLC25A44 to the metabolism of energy has likely evolved through both tissue-specific and common mechanisms of the BCAA transport and the ubiquinone precursor transport, respectively. The common mechanism which is introduced in this study is also in line with the ubiquitous expression (Figures S4 and S5) and purifying selection (Figure 4C, Table 2 and Table S6) as well as the *Slc*25A44 knockdown studies in *Trypanosoma brucei* [47] and *Caenorhabditis elegans* [48]. SLC25A44 could also play a role in the biosynthesis of secondary metabolites such as resveratrol. The health-related properties of resveratrol seem to be dependent on the interaction with quinone oxidoreductase [53]. Interestingly, the genomic blocks in the peanut genomes also contained a gene coding for quinone oxidoreductase (Figure 3B and Figure S2). Taken together, the mitochondrion-ER-nucleus partitioning of SLC25A44 fits its function due to the ubiquinone biosynthesis on the ER-mitochondrion encounter zone as well as the *para*-coumaric acid and flavonoid biosynthesis on ER, nucleus, and plasma membranes.

In summary, the study extends our knowledge by revealing a common evolutionary force that has shaped the organization of genes to meet their cooperation through their co-localizations. The co-localization of functionally related genes can facilitate functional genomics and precise functional interpretations. The likelihood of co-localization events drops as the inter-loci distance increases. Applying genomic local information can provide a considerable chance of 43% for hunting a group of at least six related genes within genomic window size ±10 genes in human. Based on one of the identified genomic blocks in peanut plants, *Slc*25A44 was characterized as the transporter of the common precursors of ubiquinone, flavonoids, and stilbenoids. The results also introduce the SLC25A44^LWW206IQF^ as a promising candidate transporter to enhance the bio-based production of *para*-coumaric acid.

## 4. Materials and Methods

### 4.1. DNA Constructs

The integrative plasmids (Table S7) were constructed using gene and promoter BioBricks (Table S8). Specific primers (Table S8) were used to amplify the fragments using Phusion U polymerase (ThermoFisher Scientific, Waltham, MA, USA). The native genes were amplified from *S. cerevisiae* CEN.PK genomic DNA, and the heterologous genes were synthetized by GeneArt (ThermoFisher Scientific, Waltham, MA, USA). The empty integrative vectors were digested with FastDigest SfaAI (ThermoFisher Scientific, Waltham, MA, USA) restriction endonuclease, nicked with Nb.BsmI (New England BioLabs, Ipswich, MA, USA), and finally assembled together with the PCR-amplified genes and promoters. To express the transporter coding gene in the oocytes, it was cloned downstream of the T7 promoter in the USER compatible *Xenopus* expression vector pNB1u as described previously [2,3]. The empty vector was digested by PacI and nicked by Nt.BbvCI (New England BioLabs, Ipswich, MA, USA) for USER-cloning of the amplified transporter ORF. The reaction was transformed into chemically competent *E. coli* cells. Finally, plasmid DNAs from single colonies were sequenced and confirmed.

### 4.2. Yeast Strain Construction

All yeast strains constructed in this study are derived from CEN.PK strain [32,35] and listed in Table S7. The native and heterologous genes under the control of strong constitutive promoters were integrated into the genome of the parental yeast strain. Before yeast transformation, the integrative vectors were linearized by FastDigest NotI (ThermoFisher Scientific, Waltham, MA, USA). Yeast cells were transformed by the standard PEG/LiAc method [54]. The cells were plated on selective plates with the appropriate selection. The plates were incubated for 3–5 days.

### 4.3. Media and Yeast Cultivation Conditions

Yeast cells were grown in standard YPD medium at 30 °C. For selection, drop-out agar plates without leucine, uracil, or histidine or a combination of these were used. Yeast strains expressing candidate transporter genes were selected by adding G418 (G8168, Sigma-Aldrich, St. Louis, MO, USA) at the final concentration of 200 µg/mL. The strains were confirmed for integration of the genes of interest by colony PCR using OneTaq^®^ Hot Start Quick-Load^®^ 2X Master Mix (New England Biolabs, Ipswich, MA, USA) using the manufacturer’s protocol and primers listed in Table S9. For production of *para*-coumaric acid, the 24-deep-well plates were used with 3 mL working volume per well. Cells from the seed cultures (in mineral medium containing 200 µg/mL of G418) were washed with sterile water and resuspended in the mineral medium to OD_600_ of 0.03. The mineral medium contained 7.5 g/L (NH_4_)_2_SO_4_, 14.4 g/L KH_2_PO_4_, 0.5 g/L MgSO_4_·7H_2_O, 1 mL/L of vitamin solution, 2 mL/L trace elements, and 20 g/L glucose [32]. The pH was set to 6 (using 2N NaOH) before filter sterilization. The experiment was carried out with three replicates per strain. Incubation of strains at 30 °C was for 72 h and with 300 rpm agitation. After OD measurement, sample collection from yeast cultures was performed by adding 1:1 vol. 99.9% ethanol to solubilize the secreted compounds in the fermentation broth. This was followed by centrifugation at 2000 g for 2 min to exclude the yeast cells. The supernatants were filtered through Whatman 0.45 µm PTFE membrane (CAT: 6784-0404, Life Sciences, Amsterdam, The Netherlands) before analysis. To measure the doubling times, yeast strains from overnight cultures were aerobically cultivated (OD_600_ ≈ 0.4) using YPD medium and 24-deep-well plates with 2 mL working volume at 30°C and 300 rpm agitation. Logarithmic cell growth was measured spectrophotometrically at *A*_600_ nm. ODs for T_1_ and T_2_ were measured after 2 and 6 h, respectively. Doubling time was measured as a function of [T_2_ − T_1(h)_]/[(OD_600_,t_2_∕OD_600_,t_1_)^1/2^].

### 4.4. Transport Assays in Xenopus Oocytes

The *Xenopus laevis* oocytes were obtained from Ecocyte Bioscience (Dortmund, North Rhine–Westphalia, Germany) and kept at 18 °C. Linear cassette including T7 promoter, *AdSlc*25A44 ORF, and 3ʹUTR was amplified with Phusion Hot Start polymerase (ThermoFisher Scientific, Ipswich, MA, USA) and used as template for in vitro transcription. Capped complementary RNAs were synthesized using the mMESSAGE mMACHINE^®^ T7 transcription kit (AM1344; ThermoFisher, Ipswich, MA, USA). The quality and quantity of the cRNA was determined using Agilent 2100 Bioanalyzer. For expression, oocytes were kept 3 days after microinjection of 25 ng in vitro transcribed cRNA of the transporter [2]. RNA-free solution was injected into the control oocytes, i.e., with no heterologous transporter. RoboInject (Multi Channel Systems, Reutlingen, BW, Germany) was used for micro-injection of cRNAs and compounds [3]. In all of the experiments, injection needles (Multi Channel Systems, Reutlingen, BW, Germany) with an opening of 25 μm were used. For efflux assay, the stock solution of 10 nL containing 200 mM resveratrol and 200 mM *para*-coumaric acid was used for micro-injection into the oocytes with and without the heterologous transporter to obtain an estimated internal concentration of 2 mM assuming 100X dilution after injection [2]. Following four washing steps, batches with 20 oocytes in each were incubated for 210 min in 90 μL Kulori buffer, pH 5. After incubation, 80 μL of the medium was collected from each batch with intact oocytes and added onto 60 μL of 100% MeOH before LC-MS analysis. The oocyte samples with 30 oocytes per replicate, after feeding with resveratrol and *para*-coumaric acid (200 μM each) or with cinnamic acid and 4-aminobenzoic acid (200 μM each) in Kulori buffer (pH 5) for 210 min, were washed four times with Kulori buffer (pH 7), and oocyte extracts were prepared in 90 μL of 60% MeOH. Finally, 30 μL water was added before analysis [2]. Statistically significant differences were determined through Student’s *t*-test.

### 4.5. Chemicals and HPLC/LC-MS Analyses

Resveratrol (34092) was obtained from Fluka and *trans para*-coumaric acid (C0393) was obtained from TCI. *Trans* cinnamic acid (C80857), hydroxybenzoic acid (H20059), and aminobenzoic acids (A9878) were obtained from Sigma-Aldrich. LC-MS was applied to analyze the concentrations of resveratrol and *para*-coumaric acid in the samples. LC-MS measurements were carried out on a Dionex UltiMate 3000 UHPLC (Thermo Fisher Scientific, San Jose, CA, USA) connected to an Orbitrap Fusion Mass Spectrometer (Thermo Fisher Scientific, San Jose, CA, USA) and using a Hypersil GOLD PFP, 15 cm × 2.1 mm, 3 μm column [32]. To quantify cinnamic acid, 4-hydroxybenzoic acid, and 4-aminobenzoic acid, 10 µl of each sample was analyzed using Dionex Ultimate 3000 HPLC with a Zorbax Eclipse Plus C18 4.6 × 100 mm, 3.5 µm (Agilent 959961-902, Santa Clara, CA, USA) column and a DAD-3000 diode array detector at 280 nm (Dionex, Sunnyvale, CA, USA). The mobile phase consisted of 0.05% acetic acid (A) and acetonitrile (B). The flow rate was 1 mL/min, and the column was kept at 30 °C. The mobile phase was introduced as a gradient of 5–12% B in 1.5 min and was held at this composition for an additional minute. The gradient was then ramped to 30% B in 2 min, and this gradient was held for an additional minute which followed by a linear gradient to 70% B in 2.5 min. This composition was kept for half minute and then changed to 5% B in 9.5 min. Column was equilibrated with 5% B for 11 min. Samples were held at 5 °C during the analysis.

### 4.6. Bioinformatics and Polymorphism Analysis

To investigate the genomic co-localization of co-expressed and co-interacting genes, the publicly available data from STRING [14] was used. STRING collects data from different sources such as BIND, DIP, GRID, HPRD, IntAct, MINT, PID, Biocarta, BioCyc, GO, KEGG, and Reactome [14]. First, low confidence paired genes or protein networks with scores below 150 [14] were excluded. Gene aliases were converted to be compatible with those in the reference genomes. Total number of paired gene and protein networks (total networks) were counted for every gene in the genome. The number of local networks were also counted for every gene and within their genomic window size of ±10 genes after positional sorting of the genes along the chromosomes. The KEGG Orthology groups (https://www.genome.jp/kegg/ko.html) (accessed on 24 May 2021) were used for identification of the homolog/duplicated genes within the genomic window size of ±10 for every gene in order to exclude the co-expression and co-interacting events between these homolog/duplicated genes from the analyses. The likelihood of total and local networks measured as per gene, i.e., total networks/total number of genes and local networks/number of genes within the defined genomic window size. The total network likelihood indicates the genome-wide probability of a network between two genes. Therefore, the ratio of local network likelihood over the total network likelihood were measured for every gene to find the co-localization likelihood fold-changes for co-expressed and co-interacting genes. Equal genome-wide and local likelihoods indicate random distribution of co-expressed and co-interacting genes. In theory, the ratios bigger than one represent the evolutionary preference toward genomic co-localization. For this study, every gene with ratio ≥2 was considered as positively selected for co-localization with its co-expressed and co-interacting genes. The human and mouse homolog genes were retrieved from the Mouse Genome Database (http://www.informatics.jax.org/) (accessed on 24 May 2021) to examine the overlap events, i.e., homolog genes with co-expressed and co-interacting neighbor genes located at the same physical gene order positions, between the human and mouse. To exclude the effect of segmental inversions and differences in chromosomal orientations between the human and mouse, inverted but exactly the same events were extracted and included in the analyses as overlapped events. The 1000 Genomes Project data [15] was also analyzed by LDlink [55] to calculate the pairwise linkage disequilibrium statistics. The Botany Array Resource [56] and COEXPEDIA [57] were used for co-expression analysis in *A. thaliana* and mouse. Pathway enrichment was performed using the Metascape [58]. The *Arabidopsis* Genome Encyclopedia [59] (http://rarge-v2.psc.riken.jp/) (accessed on 24 May 2021), TAIR and its associated Salk SNP database [60] (https://www.arabidopsis.org/) (accessed on 24 May 2021), and Gramene Release 39 [61] (https://www.gramene.org/) (accessed on 24 May 2021) were used for inspection of the *Slc*25A44 polymorphism in *A. thaliana*, which is a self-pollinating homozygote plant. The human allele database including HapMap genotypes [62] (https://bioinfo.ut.ee/HAD/) (accessed on 24 May 2021), and the dbSNP Entrez data (https://www.ncbi.nlm.nih.gov/SNP/) (accessed on 24 May 2021) were used to examine the polymorphism of human *Slc*25A44 locus. Three dimensional structure of *Ad*SLC25A44 was built using the Phyre2 [63]. The maximum likelihood phylogenetic trees were built on the WAG substitutional matrix-based model for amino acid sequences and a bootstrap value of 1000. The imprint of natural selection on SLC25A44 was analyzed as per amino acid site (including 503 amino acid positions) and using the mixed effects model of evolution (MEME) [64]. Gene-wide selection analysis was performed by the branch-site unrestricted statistical test for episodic diversification (BUSTED) [65]. BUSTED uses the unconstrained (ω1 ≤ ω2 ≤ 1 ≤ ω3) and constrained (ω3 = 1; disallowing positive selection) models. RevTrans-2.0 [66] was used for the codon-based multiple alignment of SLC25A44 orthologs from 129 vertebrate species including Mammalia, Aves, Crocodylia, Testudines, Lepidosauria, Amphibia, and Chondrichthyes. The detection of selection pressure was performed through the Datamonkey [67].

## Figures and Tables

**Table 1:** The identified genomic co-localizations for *Slc25A44, Slc25A28, Slc25A38*, and fungal *Agc*1.

Species	Chr./Contig	Co-Localized Loci
*Vitis* *vinifera*	Chr. 14	*Slc*25A44 (XP_010660575.1), *Nduf*A4 (XP_002281305.1)
	Chr. 16	4*Cl*2 (XP_002274994.1), *Nduf*S6 (XP_002276949.1), *Smg*9 (XP_010662114.1), *Nduf*A12 (XP_002276966.1), *Cox*11 (XP_002277478.1)
	Chr. 13	*Slc*25A38 (XP_002277407.1), *Atp*5H/*ATP* synthase d (XP_002277452.1), *Tim*14-1 (XP_002277365.1)
*Cajanus* *cajan*	Chr. 8	*Slc25A44 (XP_020221994.1), Lyrm (XP_020221999.1)*
	NW_017984070.1	Chalcone synthase *(XP_020230031.1), NdufA12 (XP_020230179.1), Smg9 (XP_020230144.1), 4Cl2 (XP_020230139.1*
	Chr. 2	*Slc*25A38 (XP_020234579.1), *Atp*5H/*ATP* synthase d (XP_020202519.1)
*Glycine max*	Chr. 9	*Slc*25A44 (XP_025979445.1), *Lyrm* (XP_025979447.1)
	Chr. 11	*4Cl*3 (NP_001237270.1), *Smg*9 (XP_014619235.1); *Nduf*A12 (NP_001235085.1), Chalcone synthase (NP_001304585.2)
	Chr. 20	*Slc*25A38 (XP_003556216.1), *Atp*5H/*ATP* synthase d (NP_001235072.1)
*Phaseolus vulgaris*	Chr. 6	*Slc*25A44 (XP_007147410.1), *Lyrm* (XP_007147403.1)
	Chr. 2	Chalcone synthase (XP_007157053.1), *Ndu*fA12 (XP_007157055.1), *Smg*9 (XP_007157056.1), 4*Cl* (XP_007157067.1)
	Chr. 4	*Slc*25A38 (XP_007143676.1), *Atp*5H/*ATP* synthase d (XP_007143675.1)
*Vigna radiata*	Chr. 10	*Slc*25A44 (XP_022642859.1), *Lyrm* (XP_014517948.1)
	Chr. 11	4*Cl*2 (XP_014521169.1), *Smg*9 (XP_014519466), *Nduf*A12 (XP_014519966.1, XP_022643154.1), Chalcone synthase (XP_014520574.1)
	Chr. 8	*Slc*25A38 (XP_014514167.1), *Atp*5H/*ATP* synthase d (XP_014511180.1)
*Cicer arietinum*	Chr. 1	*Slc*25A44 (XP_027189994.1), *Lyrm* (XP_004486516.1)
	Chr. 8	4*Cl*2 (XP_004511423.1), *Smg*9 (XP_004511426.1), Chalcone synthase (XP_012574354.1)
	Chr. 4	*Slc*25A38 (XP_004496329.1), *Atp*5H/*ATP* synthase d (XP_004496331.1)
*Medicago truncatula*	Chr. 2	*Slc*25A44 (XP_024632094.1), *Lyrm* (XP_003594632.1)
	Chr. 5	4*Cl*2 (XP_003610843.1), *Smg*9 (XP_003610849.1), Chalcone synthase (XP_013453346.1)
	Chr. 1	*Slc*25A38 (XP_003591971.1), *Atp*5H/*ATP* synthase d (XP_003591975.1)
*Lupinus angustifolius*	Chr. 16	*Slc*25A44 (XP_019418829.1), *Lyrm* (XP_019418830.1)
	Chr. 17	*Smg*9 (XP_019420313.1), Chalcone synthase (XP_019420991.1)
	Chr. 20	*Slc*25A38 (XP_019427565.1), *Atp*5H/*ATP* synthase d (XP_019427572.1)
*Cucumis* *sativus*	Chr. 3	*Slc*25A44 (XP_004147664.2), *Lyrm* (XP_004147626.1)
	Chr. 5	*Slc*25A38 (XP_004142487.1), *Atp*5H/*ATP* synthase d (XP_004142418.1), *Tim*14-1 (XP_004142436.1)
*Prunus* *persica*	Chr. 1	*Slc*25A44 (XP_020410285.1), *Lyrm* (XP_020410759.1)
	Chr. 2	*Slc*25A38 (XP_007220258.1), *Atp*5H/*ATP* synthase d (XP_007218513.1), *Tim*14-1 (XP_007219829.1)
*Homo* *sapiens*	Chr. 1	*Slc*25A44 (NP_001273113.1), *Smg*5 (NP_001310543.1)
	Chr. 10	*Slc*25A28 (XP_011538541.1), *Cox*15 (NP_510870.1), *Cut*C (NP_057044.2)
*Gorilla gorilla gorilla*	Chr. 1	*Slc*25A44 (XP_004026998.1), *Smg*5 (XP_018879956.1)
	Chr. 10	*Slc*25A28 (XP_018890384.2), *Cox*15 (XP_004049980.3), *Cut*C (XP_030870896.1)
*Mus musculus*	Chr. 3	*Slc*25A44 (NP_001139348.1), *Smg*5 (NP_839977.2)
	Chr. 19	*Slc*25A28 (NP_660138.1), *Cox*15 (NP_659123.2), *Cut*C (NP_001107034.1)
*Bos Taurus*	Chr. 3	*Slc*25A44 (NP_001071325.1), *Smg*5 (NP_001077148.1)
	Chr. 26	*Slc*25A28 (NP_001192481.1), *Cox*15 (NP_001070329.1), *Cut*C (NP_001193825.1)
*Xenopus laevis*	Chr. 8S	*Slc*25A44 (NP_001090232.1), *Smg*5 (XP_018089021.1)
	Chr. 7S	*Slc*25A28 (XP_018083007.1, NP_001086329.1), *ATP* synthase 6v0e2 (NP_001165047.1), *Cox*15 (XP_018083005.1)
*Halyomorpha halys*	NW_020110602.1	*Slc*25A44 (XP_014285646.1), *Uqcrq* (Complex III Subunit, XP_024215595.1)
*Saccharomyces cerevisiae*	Chr. 16	*Agc*1 (Aspartate-glutamate carrier, YPR021C), *Atp*20 (F1-F0 ATPase assembly protein, YPR020W)
*Pichia membranifaciens*	NW_017566982.1	*Agc*1 like (XP_019020020.1), *Sqr* (Complex II subunit, XP_019020021.1)
*Schizosaccharomyces pombe*	Chr. 1	*Agc*1 like (NP_593068.1), *Atp*10 (F1-F0 ATPase assembly protein, NP_593071.1)
*Candida tenuis*	NW_006281231.1	*Agc*1 like (XP_006684445.1), *Nduf*A8 (XP_006684447.1)
*Scheffersomyces stipites*	Chr. 1	*Agc*1 like (XP_001387183.2), *Cox*19 (Complex IV chaperone, XP_001386938.2)

Chr.: chromosome, 4CL: 4-coumarate-CoA ligase, COX: Cytochrome C Oxidase/Complex IV chaperone, CUTC: Copper homeostasis protein, LYRM: LYR motif containing protein/Complex I subunit, NDUF: NADH ubiquinone oxidoreductase/Complex I subunit, SLC25A44: Solute carrier family 25 subfamily 44, SQR: Succinate quinone oxidoreductase/Complex II subunit, UQCRQ: Ubiquinol-cytochrome C reductase/Complex III Subunit.

**Table 2:** Gene-wide evolutionary selection analysis of SLC25A44s across the vertebrate phylogeny.

Model	Log Likelihood	cv(svr) ^1^	ω1 ^2^	ω2 ^2^	ω3 ^2^
Unconstrained	−22,033	0.65	0.02 (83.38%) ^3^	0.20 (16.53%) ^3^	33.86 (0.08%) ^3^
Constrained	−22,046	0.651	0.02 (80.89%) ^3^	0.12 (17.25%) ^3^	1.00 (1.86%) ^3^

^1^ Coefficient variation of synonymous rates, ^2^ d*N*/d*S* rate classes, ^3^ percentage of amino acid/codon sites.

## Data Availability

The data underlying this article are available in the article and in its online supplementary material.

## Supplementary Materials

The following are available online at https://www.mdpi.com/article/10.3390/ijms22115669/s1.
