# Supplementary material for: Genome Evolutionary Dynamics Meets Functional Genomics: A Case Story on the Identification of SLC25A44
Source: Int J Mol Sci. 2021 May 26;22(11):5669. doi: 10.3390/ijms22115669 (PMC8199184; doi:10.3390/ijms22115669)
Supplement: Supplementary file 1 [file ijms-22-05669-s001.zip › ijms-1198289-supplementary.pdf]

Supplementary Information

Genome evolutionary dynamics meets functional genomics: A case story on the identification of SLC25A44

Behrooz Darbani
The Novo Nordisk Foundation Center for Biosustainability, Technical University of Denmark, 2800 Kgs. Lyngby, Denmark; and Department of Agroecology, Research Center Flakkebjerg, Aarhus University, DK-4200 Slagelse, Denmark
behroozdarbani@gmail.com, bd@agro.au.dk

This PDF file includes:

Figures S1 to S7
Tables S1 to S9

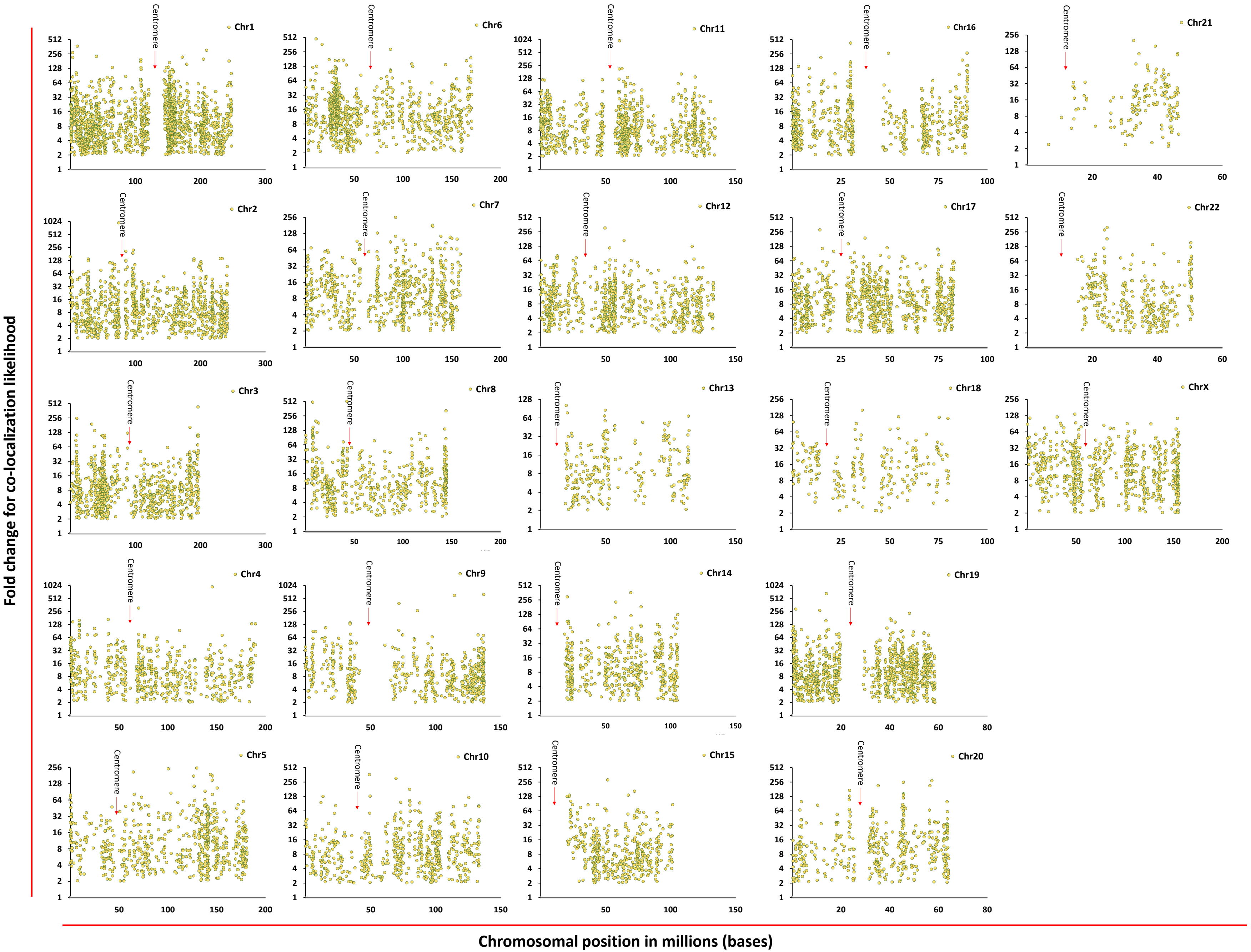

Fig. S1. Chromosomal distribution of the genes having co-localization likelihood of ≥ 2 in humans.

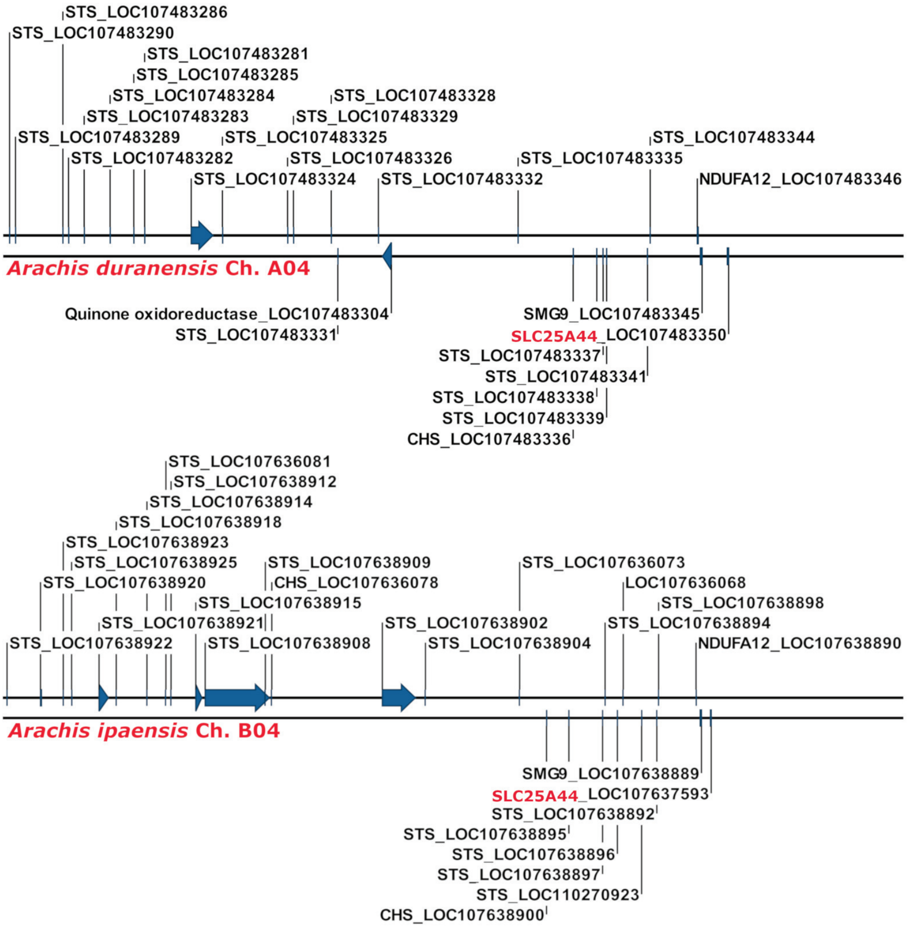

Fig. S2. The identified genomic blocks in peanut ancestors *A. ipaensis* and *A. duranensis.*

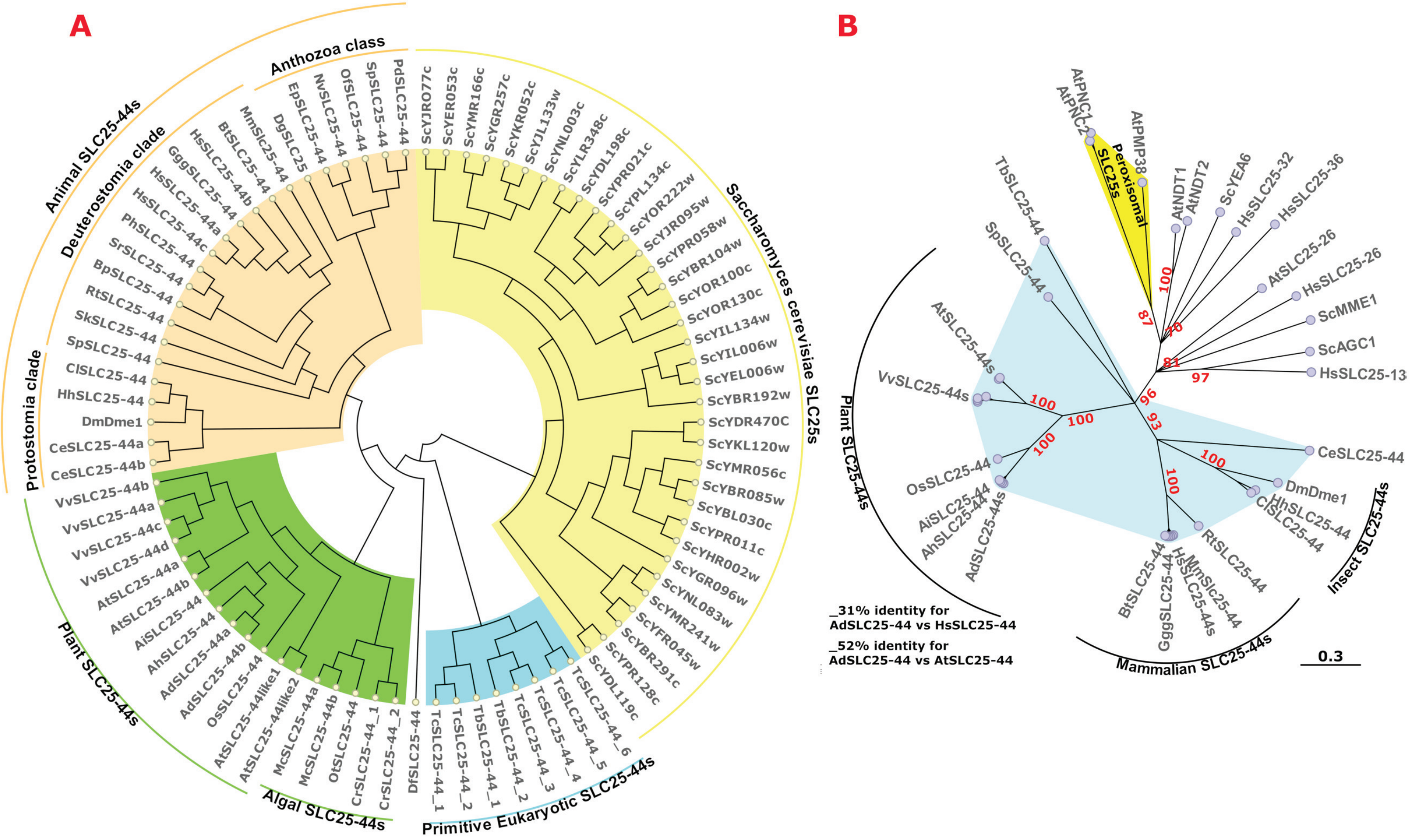

Fig. S3. There is no eukaryotic orthologue for members of SLC25A44 (SLC25-44) in fungi. (A) Members of the SLC25A44 have animal, plant, and primitive eukaryotic specific phylogenetic clusters which are completely different from any member of *S. cerevisiae* SLC25s. **(B)** *S. cerevisiae* AGC1, MME1, and YEA6 are the most immediate blast hits for SLC25A44s. **(A, B)** The maximum likelihood phylogenetic tree was built on the WAG matrix for amino acid sequences and bootstraps are shown as percentage of 1000. See Table S5 for protein sequences.

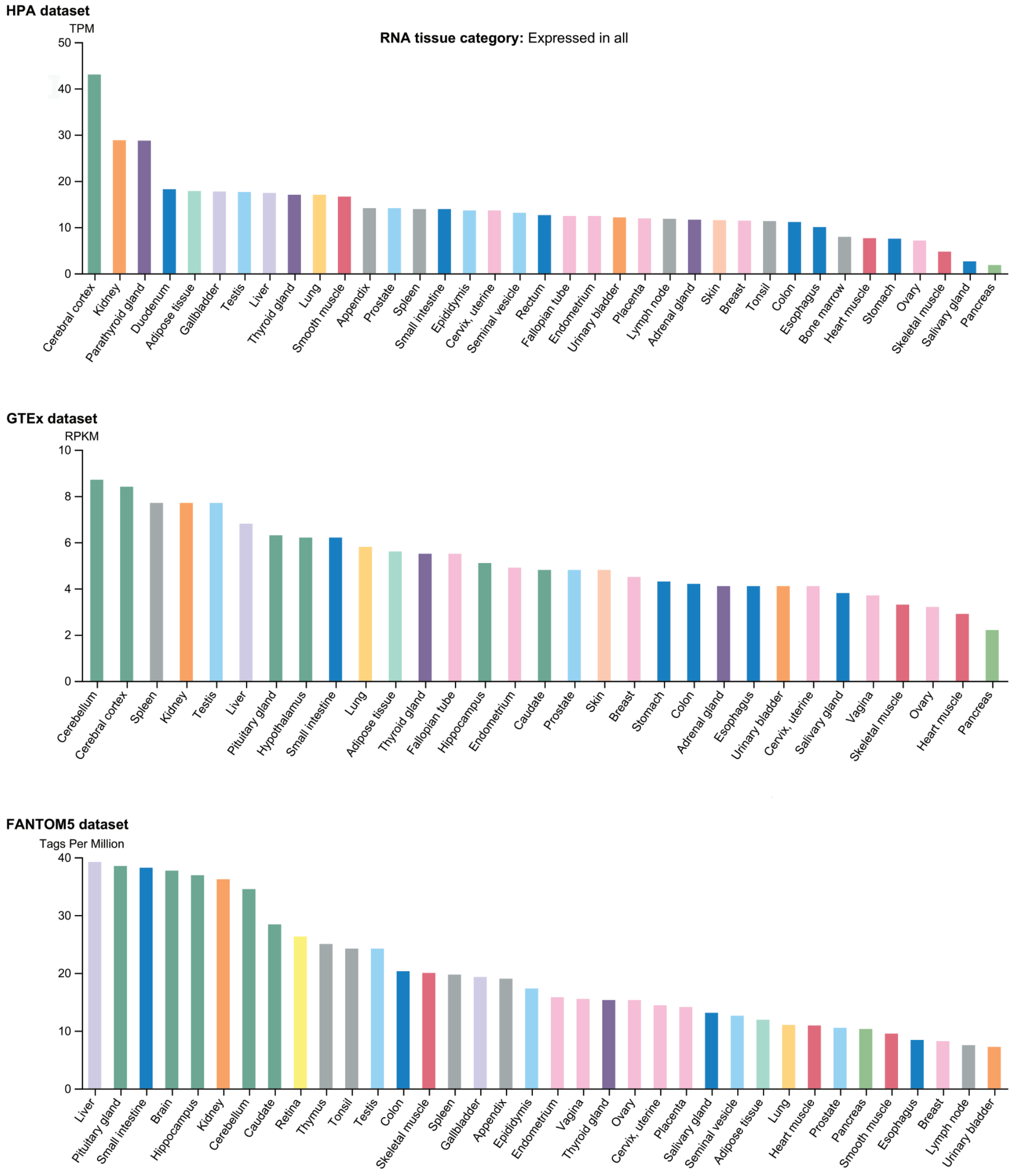

Fig. S4. Transcript levels of *Slc*25A44 in human (From the human protein atlas at https://www.proteinatlas.org/; Nat. Biotechnol. 2010, 28:1248-50).

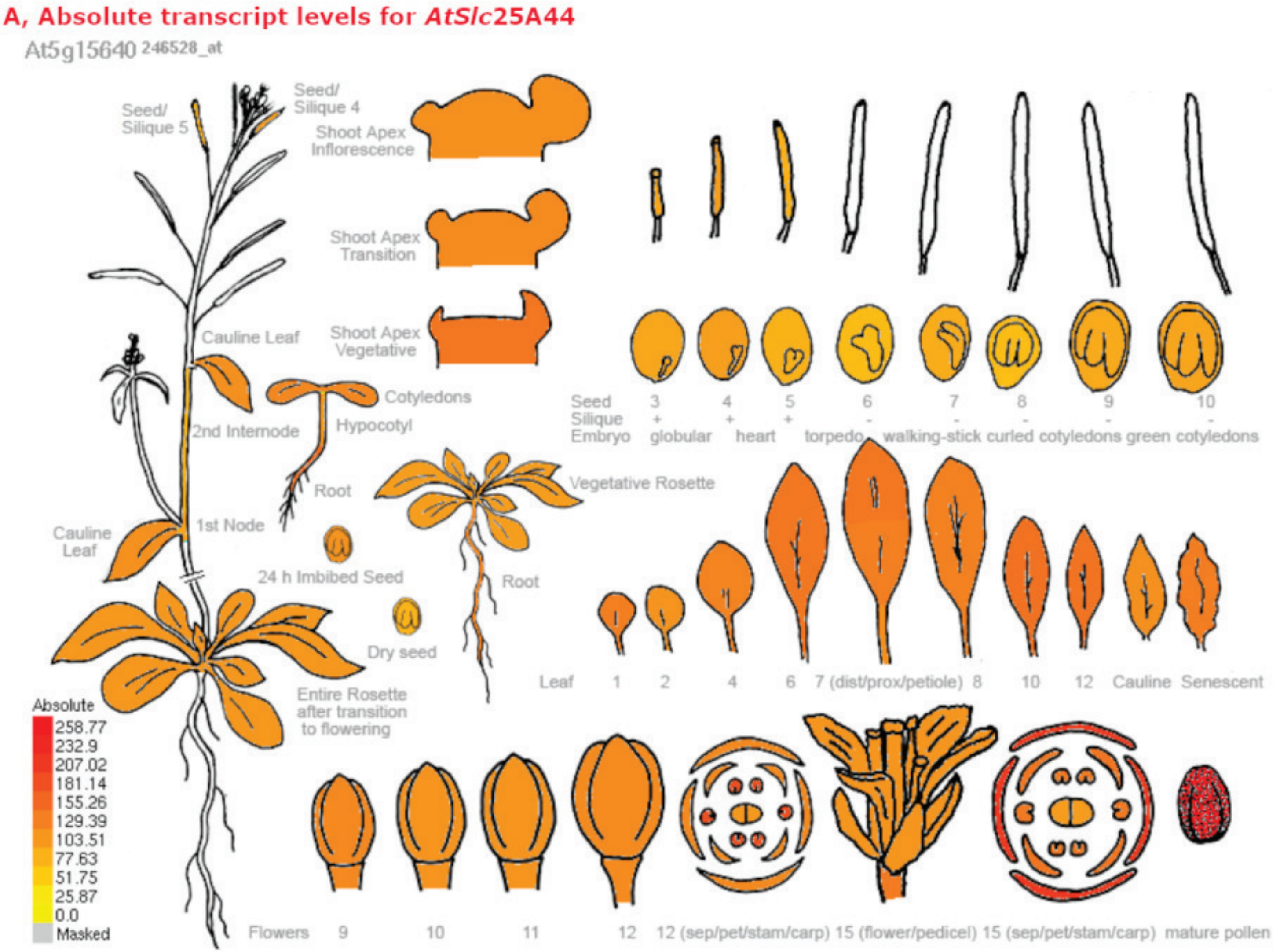

Fig. S5. Constitutive expression of *Arabidopsis thaliana Slc*25A44. (A-F) *AtSlc*25A44 absolute and relative transcript levels in comparison with the housekeeping genes *Ubc*9 and *Gapdh*. At5g15640: *Slc*25A44, At4g27960: *Ubc*9, At1g13440: *Gapdh* (Data from Nat. Genet. 37:501-6). **(G)** *At*SLC25A44 protein levels (Data from Science 320, 938-41).

B, Hormone treatments

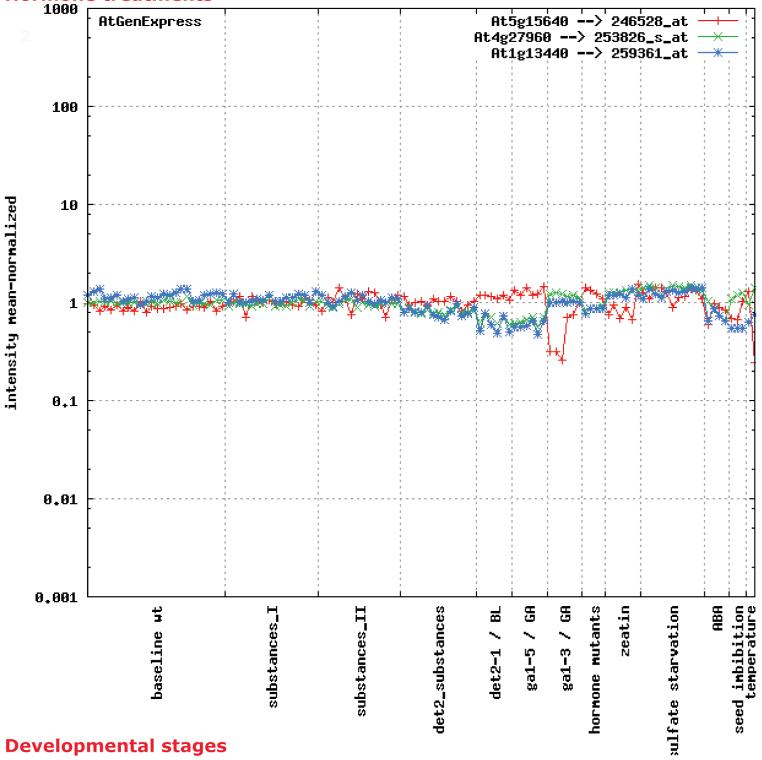

C, Developmental stages

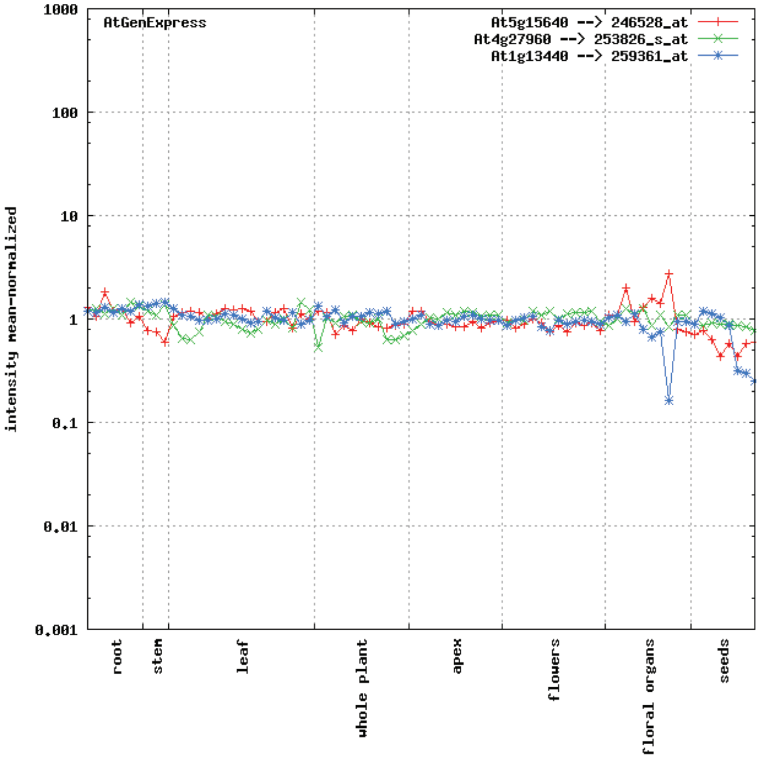

Fig. S5. Constitutive expression of *Arabidopsis thaliana Slc*25A44. (A-F) *AtSlc*25A44 absolute and relative transcript levels in comparison with the housekeeping genes *Ubc*9 and *Gapdh*. At5g15640: *Slc*25A44, At4g27960: *Ubc*9, At1g13440: *Gapdh* (Data from Nat. Genet. 37:501-6). **(G)** *At*SLC25A44 protein levels (Data from Science 320, 938-41).

D, Biotic stress

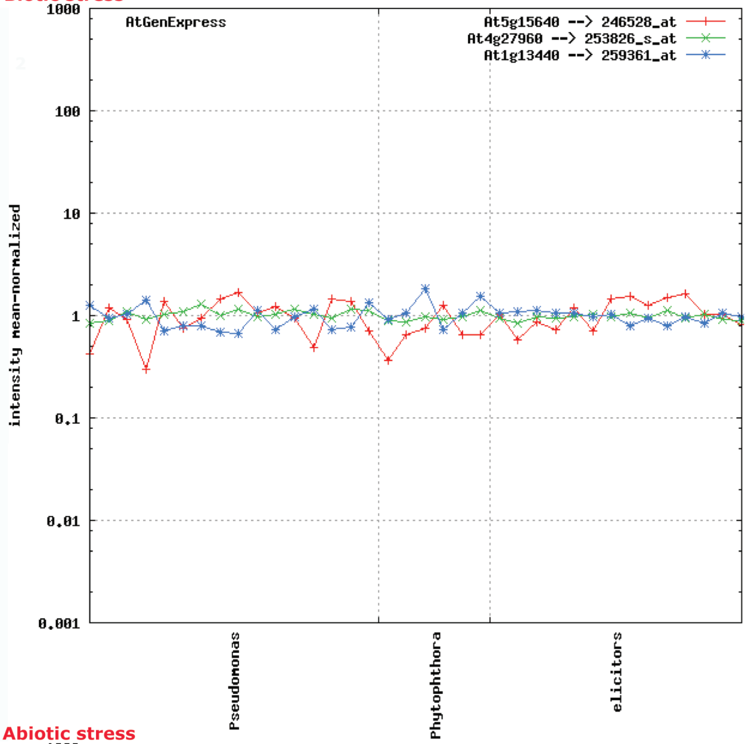

E, Abiotic stress

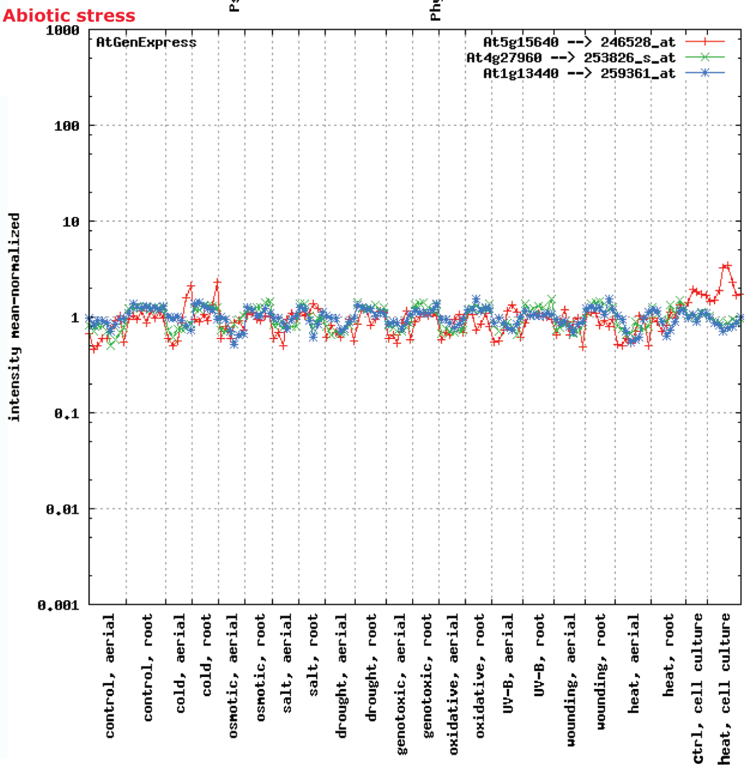

Fig. S5. Constitutive expression of *Arabidopsis thaliana* *Slc*25A44. (A-F) *AtSlc*25A44 absolute and relative transcript levels in comparison with the housekeeping genes *Ubc*9 and *Gapdh*. At5g15640: *Slc*25A44, At4g27960: *Ubc*9, At1g13440: *Gapdh* (Data from Nat. Genet. 37:501-6). **(G)** *At*SLC25A44 protein levels (Data from Science 320, 938-41).

F, Light stress

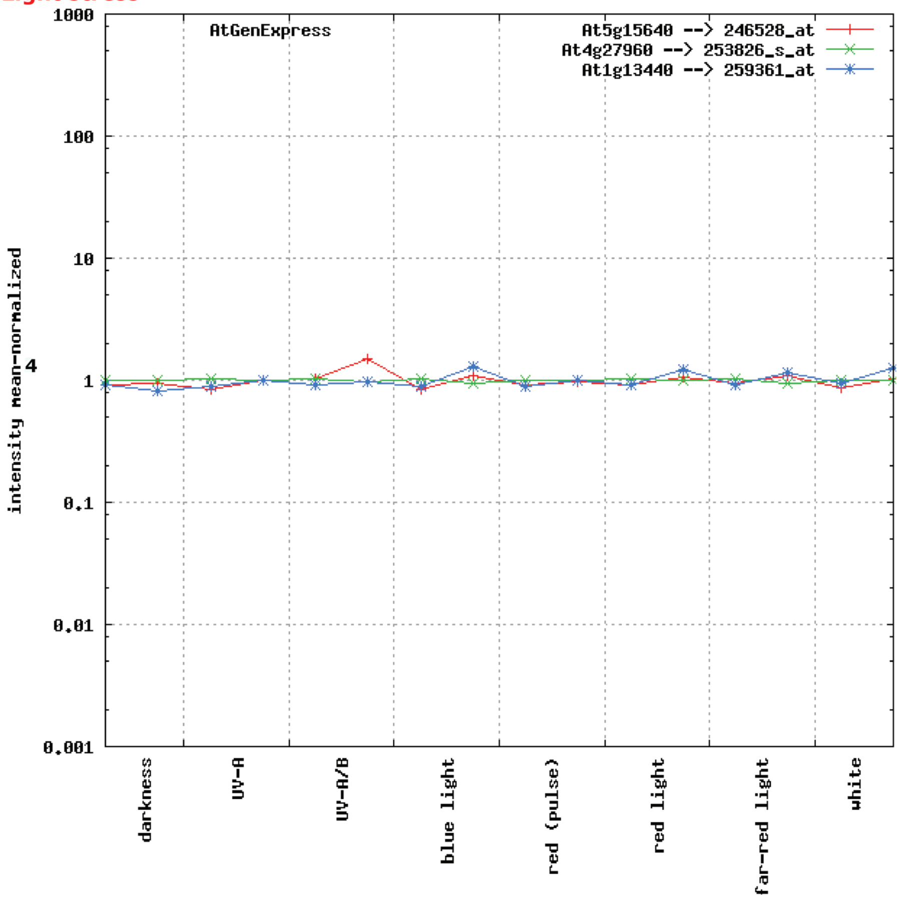

G, Protein levels

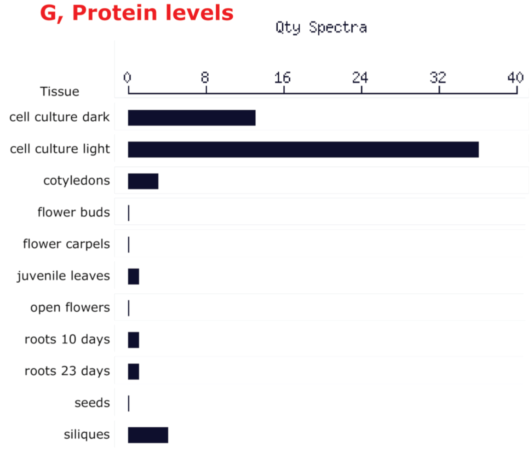

Fig. S5. Constitutive expression of *Arabidopsis thaliana Slc*25A44. (A-F) *AtSlc*25A44 absolute and relative transcript levels in comparison with the housekeeping genes *Ubc*9 and *Gapdh*. At5g15640: *Slc*25A44, At4g27960: *Ubc*9, At1g13440: *Gapdh* (Data from Nat. Genet. 37:501-6). **(G)** *AtSLC*25A44 protein levels (Data from Science 320, 938-41).

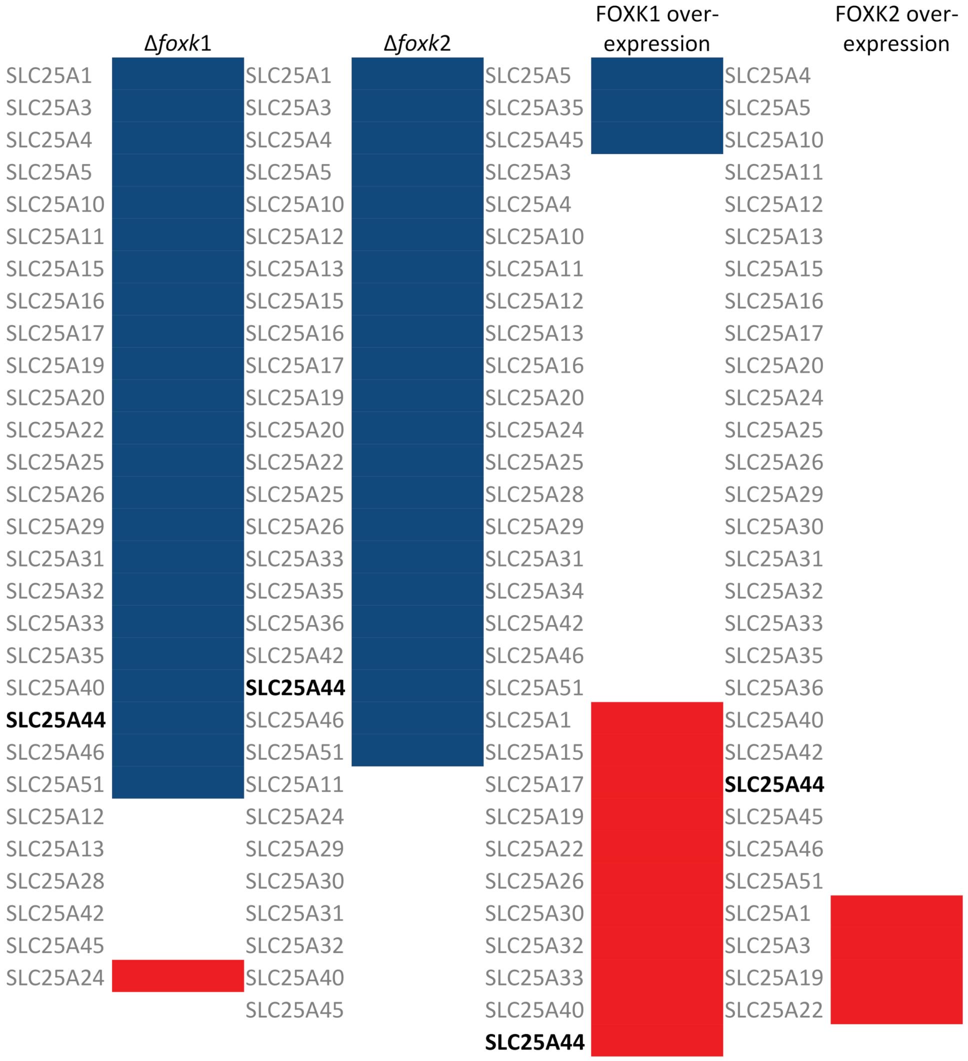

Fig. S6. The effects of FOXK1 and FOXK2 on the cellular levels of SLC25 transporter proteins (Data from *Nature,* 566:279–83). Blue: downregulation (p < 0.05), White: no significant changes, Red: upregulation (p < 0.05).

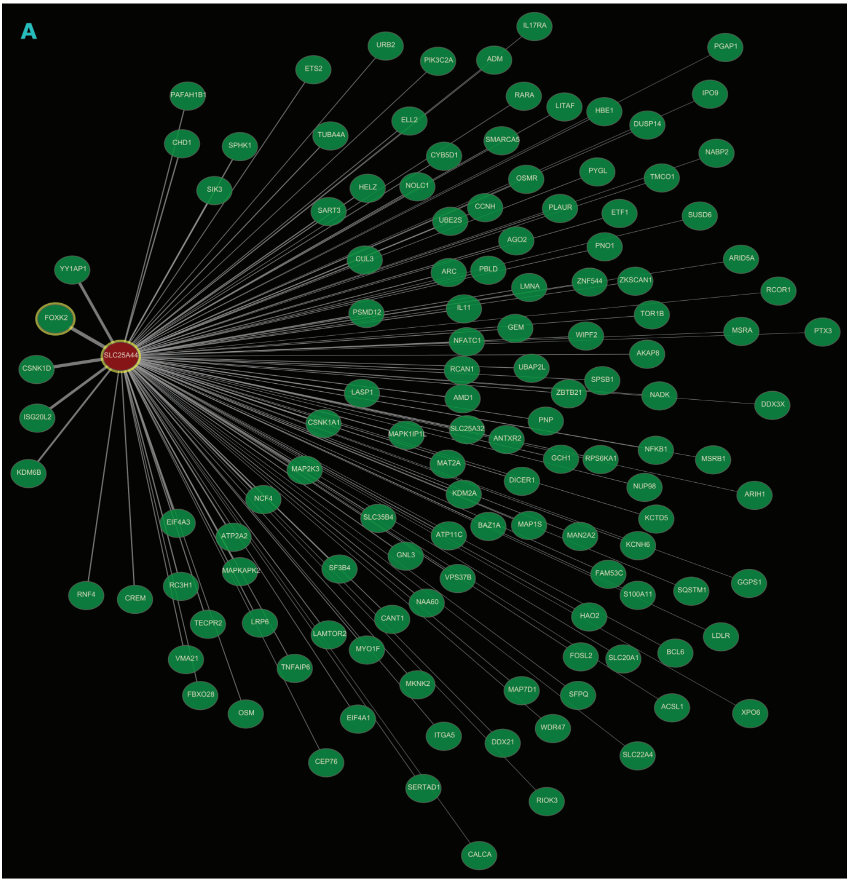

Fig. S7. The *Slc*25A44 (A) and *Foxk*2 (B) co-expressed genes in human (Data from Nucleic Acids Res. 45,D389-D396).

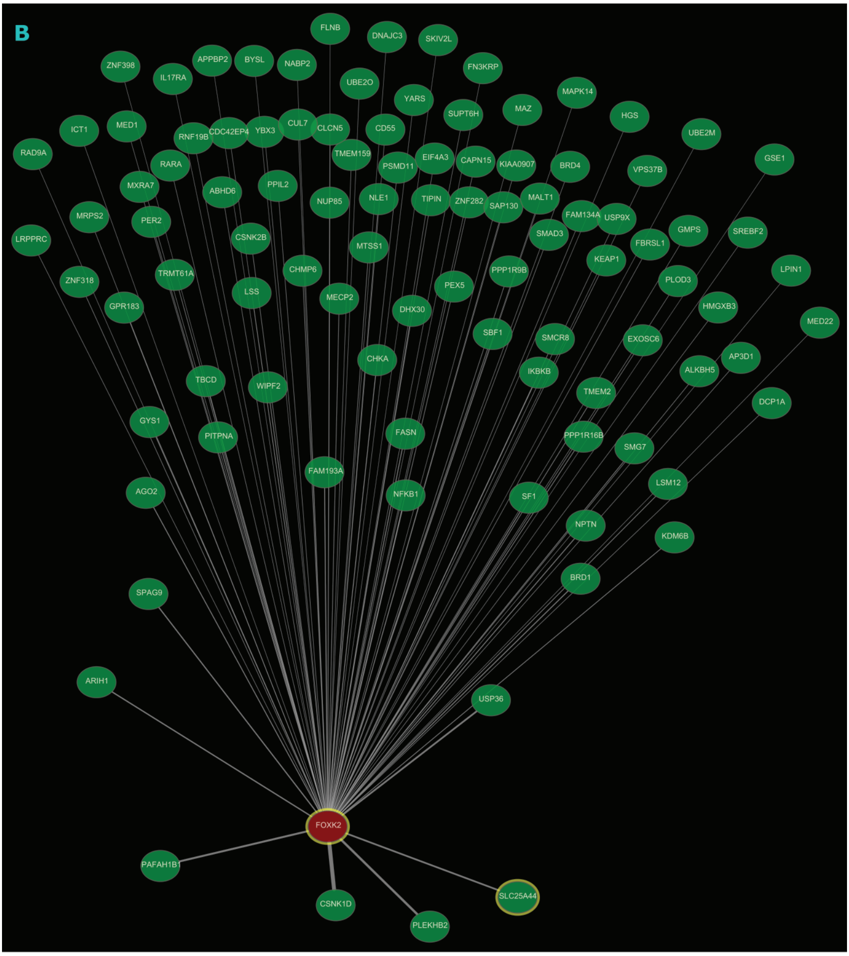

Fig. S7.The *Slc*25A44 (A) and *Foxk*2 (B) co-expressed genes in human
(Data from Nucleic Acids Res. 45,D389-D396).

Table S1. Visual scanning of the genomes found several events of the co-localized functionally-related-genes with at least one membrane transporter coding gene

	Arachis duranensis	*Arabidopsis thaliana*	*Homo sapiens*	*Oryzias latipes*	*Aquila chrysaetos*
**Co-localized genes involved in amino acid transport and production/usage**	7	6	7	4	5
**Co-localized transporter and enzyme coding genes involved in energy/heme metabolism**	19	7	19	25	22
**Nucleotide and Mg transporters co-localized with DNA/RNA polymerization enzymes and factors**	8	6	-	4	-
**Co-localized transporter and enzyme coding genes involved in nitrogen metabolism**	3	2	1	-	1
**Different transporter genes involved in cell wall formation**	3	-	-	-	-
**Auxin transporter co-localized with auxin response genes**	2	1	-	-	-
**Trace metal transporters co-localized with genes coding for mineral containing factors**	2	6	2	2	1
**Sulfate transporters co-localized with genes involved in sulfur metabolism**	4	-	1	2	2
**Nitrate/phosphate transporters co-localized with RUBisCO subunit and carbonic anhydrase genes**	2	-	-	-	-
**Inositol transporters co-localized with inositol phosphatase and kinase genes**	3	-	-	-	-
**Co-localized transporter and enzyme coding genes involved in cholesterol metabolism**	-	-	1	1	1
**Choline metabolism and transport**	-	-	2	2	1
**Glutathione metabolism and transport**	-	-	1	-	1
**Thiamine transporter co-localized with the coagulation factor V coding gene**	-	-	1	-	1

Table S2. The co-localized functionally-related-genes with at least one membrane transporter coding gene and found by visual genome scanning

Arabidopsis thaliana

#Name	GeneID	Protein product	Protein Name
**Amino acid transporters co-localized with genes involved in amino acid production or usage (6 clusters)**			
chromosome 1	837342	NP_001320253.1	Eukaryotic aspartyl protease family protein
chromosome 1	837343	NP_172300.2	ATPase complex subunit
chromosome 1	837344	NP_001077487.1	Transmembrane amino acid transporter family protein
chromosome 1	837345	NP_563809.1	Arogenate dehydratase 6, converts the prephenate produced from the shikimate-chorismate pathway into phenylalanine
chromosome 1	838281	NP_001185019.1	Ubiquitin-specific protease 15
chromosome 1	838282	NP_173155.1	Cationic amino acid transporter 8
chromosome 1	840071	NP_564385.1	Ribosomal L18p/L5e family protein
chromosome 1	840072	NP_174465.1	Amino acid permease family protein
chromosome 1	840073	NP_174466.1	Amino acid permease family protein
chromosome 2	818715	NP_850346.1	Ubiquitin-associated (UBA) protein
chromosome 2	818718	NP_030664.1	Transmembrane amino acid transporter family protein
chromosome 2	818721	NP_181655.1	Glutamate synthase 2
chromosome 3	820363	NP_001327533.1	Aromatic and neutral transporter 1
chromosome 3	820364	NP_187797.3	Ubiquitin-specific protease 13
chromosome 3	820560	NP_001326490.1	Ribosomal protein L30/L7 family protein
chromosome 3	820565	NP_566460.1	Amino acid permease family protein
**Co-localized transporter and enzyme coding genes involved in energy/heme metabolism (5 clusters)**			
chromosome 4	828724	NP_001320068.1	Mitochondrial substrate carrier family protein, mitochondrial CoA transporter
chromosome 4	828725	NP_001328352.1	Haloacid dehalogenase-like hydrolase (HAD) superfamily protein
chromosome 4	828726	NP_194350.1	1-amino-cyclopropane-1-carboxylate synthase 7
chromosome 4	828727	NP_194351.1	Mitochondrial ATP synthase subunit G protein
chromosome 4	829382	NP_194973.1	Cytochrome bd ubiquinol oxidase
chromosome 4	829383	NP_194974.1	sugar phosphate exchanger, putative (DUF506)
chromosome 4	829386	NP_001328113.1	HCO3- transporter family
chromosome 4	829388	NP_001119099.1	ATPase, F0/V0 complex, subunit C protein
chromosome 5	830867	NP_195752.1	Thiamine pyrophosphate dependent pyruvate decarboxylase family protein
chromosome 5	831414	NP_195753.1	pyruvate decarboxylase-3
chromosome 5	831479	NP_195754.1	Mitochondrial substrate carrier family protein, SUCCINATE-FUMARATE CARRIER 1
chromosome 5	831184	NP_568288.1	Ubiquinol-cytochrome C reductase iron-sulfur subunit
chromosome 5	831186	NP_196849.1	delta subunit of Mt ATP synthase
chromosome 5	831193	NP_196853.1	ADP/ATP carrier 2
chromosome 5	835177	NP_001190509.1	succinate dehydrogenase assembly factor
chromosome 5	835178	NP_199918.2	Mitochondrial substrate carrier family protein, ATP-Mg/Pi transporter
chromosome 5	835179	NP_199919.1	NADPH/respiratory burst oxidase protein D
**Sugar transporters co-localized with enzymes involved in carbohydrate production or usage (2 clusters)**			
chromosome 1	837159	NP_001318934.1	Nucleotide/sugar transporter family protein, UDP-glucose transmembrane transport
chromosome 1	837160	NP_172136.2	glucan synthase-like 7
chromosome 1	837235	NP_172204.1	UDP-glucosyl transferase 71C5
chromosome 1	837240	NP_001318940.1	golgi nucleotide sugar transporter 2
**Nucleotide and Mg transporters co-localized with DNA/RNA polymerization enzymes and factors (6 clusters)**			
chromosome 1	3766805	NP_001319095.1	DEAD-box ATP-dependent RNA helicase-like protein
chromosome 1	839716	NP_174143.1	purine permease 3
chromosome 1	839717	NP_174144.1	purine permease 1
chromosome 1	839720	NP_174147.1	Telomerase activating protein Est1
chromosome 1	839720	NP_973927.1	Telomerase activating protein Est1
chromosome 1	841419	NP_175418.1	Xanthine/uracil permease family protein
chromosome 1	841421	NP_564561.1	photosystem I reaction center subunit N
chromosome 1	841422	NP_175420.3	DNA/RNA polymerases superfamily protein
chromosome 1	842866	NP_176733.2	Xanthine/uracil permease family protein
chromosome 1	842869	NP_176736.2	Endonuclease/exonuclease/phosphatase family protein

chromosome 1	842869	NP_001321558.1	Endonuclease/exonuclease/phosphatase family protein
chromosome 3	821960	NP_001189957.1	nuclear RNA polymerase D2A
chromosome 3	821969	NP_001327822.1	GTP-binding protein-like protein
chromosome 3	821970	NP_001326607.1	magnesium transporter NIPA (DUF803)
chromosome 3	821972	NP_189031.1	topoisomerase II
chromosome 3	821973	NP_001325936.1	RNA recognition motif (RRM)-containing protein
chromosome 3	821974	NP_189033.1	reverse transcriptase-like protein
chromosome 3	822279	NP_001326342.1	magnesium transporter, putative (DUF803)
chromosome 3	822280	NP_001319649.1	DNA repair metallo-beta-lactamase family protein
chromosome 5	835095	NP_001332453.1	Xanthine/uracil permease family protein
chromosome 5	835096	NP_568723.1	Galactose oxidase/kelch repeat superfamily protein
chromosome 5	835098	NP_568725.1	radical SAM domain-containing protein / GCN5-related N-acetyltransferase (GNAT) family protein
chromosome 5	835099	NP_199844.3	Protein kinase superfamily protein
chromosome 5	835100	NP_568726.1	hypothetical protein AT5G50335
chromosome 5	835101	NP_001318773.1	DNA repair protein RadA-like protein
**Co-localized transporter and enzyme coding genes involved in nitrogen metabolism (2 clusters)**			
chromosome 1	837324	NP_172285.2	alpha carbonic anhydrase 5, it produces bicarbonate which is used for TCA and urea cycle.
chromosome 1	837325	NP_172286.1	Tetratricopeptide repeat (TPR)-like superfamily protein
chromosome 1	837326	NP_172287.1	alpha carbonic anhydrase 7, it produces bicarbonate
chromosome 1	837327	NP_172288.1	nitrate transporter 2:1, nitrate is a precursor of NH4 which is used together with bicarbonate for urea cycle
chromosome 1	837328	NP_172289.1	nitrate transporter 2.2
chromosome 2	818408	NP_850294.1	AMP deaminase, putative / myoadenylate deaminase, releases ammonia
chromosome 2	818409	NP_181363.1	ammonium transporter 2
**Auxin transporter co-localized with auxin response factors (1 clusters)**			
chromosome 1	838916	NP_564189.1	Auxin efflux carrier family protein
chromosome 1	838924	NP_173729.1	Auxin-responsive GH3 family protein
**Trace metal transporters co-localized with genes coding for mineral containing factors (6)**			
chromosome 1	842386	NP_176291.1	2Fe-2S ferredoxin-like superfamily protein
chromosome 1	842387	NP_564766.1	iron regulated transporter 3
chromosome 1	842387	NP_001321604.1	iron regulated transporter 3
chromosome 1	842387	NP_001321605.1	iron regulated transporter 3
chromosome 1	843131	NP_176970.2	RING/FYVE/PHD zinc finger superfamily protein
chromosome 1	843138	NP_564921.1	ZIP metal ion transporter family
chromosome 1	843142	NP_001319342.1	zinc finger/BTB domain protein, putative (DUF1644)
chromosome 4	827332	NP_567493.5	oligopeptide transporter, loads iron into the phloem, facilitates iron recirculation from the xylem to the phloem
chromosome 4	2745712	NP_001329947.1	Heavy metal transport/detoxification superfamily protein
chromosome 4	827338	NP_567496.4	ferredoxin hydrogenase, iron–sulfur protein that mediate electron transfer.
chromosome 4	827343	NP_567500.2	NADH-ubiquinone oxidoreductase, Fe-S protein
chromosome 4	828976	NP_194587.2	magnesium transport 5, mitochondria
chromosome 4	828980	NP_194591.1	ABC transporter of the mitochondrion 2, cellular iron ion homeostasis
chromosome 4	828981	NP_567813.1	ABC transporter of the mitochondrion 1, cellular iron ion homeostasis
chromosome 4	828984	NP_001190860.1	photosystem II reaction center PSB28 protein
chromosome 4	828984	NP_567814.1	photosystem II reaction center PSB28 protein
chromosome 5	831889	NP_195948.1	NRAMP metal ion transporter family protein, transports Mn2+
chromosome 5	831884	NP_568113.1	isocitrate dehydrogenase V, it has Mg2+ and Mn2+
chromosome 5	831867	NP_001318466.1	membrane magnesium transporter-like protein
chromosome 5	835081	NP_199827.2	ferric reduction oxidase 8
chromosome 5	835085	NP_199831.1	nitrate transmembrane transporter
chromosome 5	835086	NP_199832.1	quinolinate synthase, iron-sulfur protein involved in the de novo synthesis of NAD+

Arachis duranensis

#Name	GeneID	Protein product	Protein name
**Amino acid transporters co-localized with genes involved in amino acid production or usage (7 clusters)**			
chromosome A01	107472969	XP_015948014.1	E3 ubiquitin-protein ligase RNF185
chromosome A01	107473107	XP_015948163.1	probable vacuolar amino acid transporter YPQ2
chromosome A01	107457692	XP_015931373.1	probable amino acid permease 7

chromosome A01	107472733	XP_015947745.2	probable amino acid permease 7
chromosome A01	107496916	XP_015973756.1	proteasome activator subunit 4
chromosome A03	107476563	XP_015951880.1	E3 ubiquitin-protein ligase UPL3
chromosome A03	107476554	XP_015951871.1	amino acid permease 4
chromosome A03	110278388	XP_020992283.1	amino acid permease 5-like
chromosome A03	107477011	XP_015952465.2	subtilisin-like protease SBT1.6
chromosome A03	107476553	XP_015951870.1	actin-depolymerizing factor 5
chromosome A02	107474523	XP_020991080.1	vacuolar amino acid transporter 1
chromosome A02	107474520	XP_015949631.1	serine carboxypeptidase-like
chromosome A02	107474956	XP_020991233.1	serine carboxypeptidase-like 19
chromosome A02	107474951	XP_015950078.1	serine carboxypeptidase-like 11
chromosome A02	107474957	XP_015950084.1	serine carboxypeptidase-like 18
chromosome A02	107474959	XP_020991238.1	lysine histidine transporter-like 8
chromosome A02	110277905	XP_020991237.1	serine carboxypeptidase-like 16
chromosome A02	107475056	XP_015950193.1	serine carboxypeptidase-like 11
chromosome A02	107475055	XP_020991245.1	serine carboxypeptidase-like 11
chromosome A06	107493160	XP_020999805.1	tryptophan synthase alpha chain
chromosome A06	107493157	XP_015969732.2	lysine histidine transporter 2-like
chromosome A06	107493088	XP_020981395.1	lysine histidine transporter 2-like
chromosome A06	107493087	XP_020981396.1	lysine histidine transporter-like 2
chromosome A06	107493151	XP_020999818.1	lysine histidine transporter 2-like
chromosome A06	107494363	XP_015970902.1	valine--tRNA ligase, chloroplastic/mitochondrial 2 isoform X1
chromosome A06	107494330	XP_020980620.1	cationic amino acid transporter 2, vacuolar-like
chromosome A06	107494361	XP_015970901.1	cationic amino acid transporter 4, vacuolar-like

Co-localized transporter and enzyme coding genes involved in energy/heme metabolism (14 clusters)

chromosome A04	107483290	XP_015959407.1	stilbene synthase 3
chromosome A04	107483289	XP_015959406.1	stilbene synthase 3
chromosome A04	107483286	XP_015959405.1	stilbene synthase 3
chromosome A04	107483282	XP_015959400.1	stilbene synthase 3-like
chromosome A04	107483283	XP_015959401.1	stilbene synthase 3
chromosome A04	107483284	XP_015959402.1	putative stilbene synthase 2
chromosome A04	107483285	XP_015959403.1	stilbene synthase 1
chromosome A04	107483281	XP_015959399.1	stilbene synthase 1
chromosome A04	107483324	XP_020995724.1	stilbene synthase 3 isoform X2
chromosome A04	107483325	XP_015959435.1	stilbene synthase 1
chromosome A04	107483326	XP_015959436.1	stilbene synthase 1
chromosome A04	107483329	XP_015959438.1	stilbene synthase 1
chromosome A04	107483328	XP_015959437.1	stilbene synthase 1
chromosome A04	107483331	XP_015959439.2	putative stilbene synthase 2
chromosome A04	107483332	XP_015959440.1	stilbene synthase 1
chromosome A04	107483304	XP_020995730.1	quinone oxidoreductase-like protein 2 homolog
chromosome A04	107483333	XP_015959441.1	stilbene synthase 3
chromosome A04	107483335	XP_015959445.1	stilbene synthase 3
chromosome A04	107483336	XP_015959446.1	chalcone synthase
chromosome A04	107483338	XP_015959448.1	stilbene synthase 3
chromosome A04	107483337	XP_015959447.1	stilbene synthase 3
chromosome A04	107483339	XP_015959449.1	stilbene synthase 3
chromosome A04	107483341	XP_020995733.1	stilbene synthase 3 isoform X2
chromosome A04	107483341	XP_015959450.1	stilbene synthase 3 isoform X1
chromosome A04	107483344	XP_015959453.1	stilbene synthase 3
chromosome A04	107483346	XP_015959455.1	NADH dehydrogenase, alpha subcomplex subunit 12
chromosome A04	107483345	XP_015959454.1	protein SMG9
chromosome A04	107483350	XP_015959458.1	solute carrier family 25 member 44
chromosome A08	107461061	XP_015935003.1	NADH dehydrogenase, NADH dehydrogenase flavoprotein 2
chromosome A08	107461041	XP_015934969.1	NADP-dependent malic enzyme
chromosome A08	107461092	XP_015935035.1	Solute carrier family 25 member 44-like 2
chromosome A08	107461402	XP_020984731.1	alcohol dehydrogenase 1, NAD to NADH
chromosome A08	107461451	XP_015935428.1	Solute carrier family 25 member 44-like 3
chromosome A02	107475879	XP_020991624.1	SLC25A30
chromosome A02	107475907	XP_015951065.1	3-dehydroquinate dehydratase/shikimate dehydrogenase
chromosome A03	107480364	XP_015955979.1	SLC25A26, S-adenosylmethionine carrier 1

chromosome A03	107480360	XP_015955974.1	probable ATP synthase 24 kDa subunit, mitochondrial
chromosome A03	107480379	XP_015955994.1	iron-sulfur assembly protein IscA, chloroplastic
chromosome A04	107484901	XP_015960945.1	alcohol dehydrogenase-like 4
chromosome A04	107484882	XP_020996276.1	thylakoid ADP,ATP carrier protein, chloroplastic
chromosome A04	110280406	XP_020997080.1	NADH dehydrogenase
chromosome A04	107483117	XP_015959215.1	sugar carrier protein C-like
chromosome A04	107486787	XP_020997017.1	plastidic glucose transporter 4
chromosome A04	107486787	XP_020997018.1	ATP synthase gamma chain, chloroplastic isoform X2
chromosome A08	107462656	XP_015936765.1	SLC25A4, ADP,ATP carrier protein 1, mitochondrial
chromosome A08	107462784	XP_015936928.1	ATP synthase subunit O, mitochondrial
chromosome A10	107471024	XP_015945954.1	ATP synthase subunit gamma, mitochondrial
chromosome A10	107471021	XP_020988454.1	SLC25A45
chromosome A09	107467792	XP_015942464.2	ATP-dependent 6-phosphofructokinase 2
chromosome A09	107467872	XP_015942576.1	ATP synthase subunit d, mitochondrial
chromosome A09	107467808	XP_015942487.1	SLC25A38, associated with heme biosynthesis
chromosome A05	107490917	XP_015967205.1	probable folate-biopterin transporter 9, chloroplastic
chromosome A05	107491028	XP_020999660.1	uroporphyrinogen decarboxylase 1
chromosome A05	107491277	XP_015967585.1	probable transmembrane ascorbate ferrireductase 4 isoform X2
chromosome A06	107494840	XP_015971359.1	protoporphyrinogen oxidase, chloroplastic/mitochondrial
chromosome A06	107494865	XP_015971391.1	mitochondrial pyruvate carrier 1
chromosome A05	107490448	XP_020998717.1	ABC transporter B family member 28
chromosome A05	107490446	XP_015966713.1	fe-S cluster assembly factor HCF101, chloroplastic isoform X1

Sugar transporters co-localized with enzymes involved in carbohydrate production or usage (5 clusters)

chromosome A03	107479690	XP_020994226.1	probable trehalase: releases glucose
chromosome A03	107479692	XP_020994227.1	beta-amylase 3, chloroplastic isoform X1
chromosome A03	107479692	XP_015955281.1	beta-amylase 3, chloroplastic isoform X2
chromosome A03	107479705	XP_015955290.1	bidirectional sugar transporter SWEET4
chromosome A07	107459705	XP_015933416.1	alpha,alpha-trehalose-phosphate synthase
chromosome A07	107459692	XP_015933401.1	bidirectional sugar transporter SWEET2
chromosome A08	107461287	XP_015935246.1	polyol transporter 5
chromosome A08	107461291	XP_015935252.1	protein CELLULOSE SYNTHASE INTERACTIVE 3 isoform X1
chromosome A09	107464934	XP_015939388.1	bidirectional sugar transporter SWEET6a-like
chromosome A09	107464957	XP_015939405.1	galactinol synthase 1, adds galactose on myo-inositol
chromosome A10	107471096	XP_015946030.1	UDP-galactose/UDP-glucose transporter 3
chromosome A10	107471043	XP_015945973.1	glucose-6-phosphate isomerase, cytosolic 2B

Nucleotide and Mg transporters co-localized with DNA/RNA polymerization enzymes and factors (8 clusters)

chromosome A03	107476420	XP_020992823.1	DNA polymerase epsilon catalytic subunit A
chromosome A03	107476423	XP_015951713.1	histone deacetylase 9
chromosome A03	107476432	XP_020992830.1	nucleobase-ascorbate transporter 12
chromosome A03	107477868	XP_020993444.1	replication protein A 70 kDa DNA-binding subunit B-like
chromosome A03	107477982	XP_015953557.1	equilibrative nucleotide transporter 3-like
chromosome A03	107478800	XP_015954422.1	nucleotide-sugar uncharacterized transporter 1
chromosome A03	107478799	XP_015954420.1	DNA-directed RNA polymerase III subunit 2
chromosome A03	107480120	XP_020994402.1	DNA polymerase epsilon catalytic subunit A-like
chromosome A03	107480125	XP_015955750.1	magnesium transporter MRS2-3
chromosome A04	107483672	XP_015959764.1	transcription factor GTE1-like
chromosome A04	107483687	XP_015959775.1	DNA replication licensing factor MCM6
chromosome A04	107483689	XP_015959778.1	probable purine permease 4
chromosome A04	107483657	XP_020995858.1	high affinity nitrate transporter 2.5
chromosome A04	107483690	XP_015959779.1	probable purine permease 4

chromosome A04	107484292	XP_015960391.1	signal transducer and activator of transcription A-like
chromosome A04	107484299	XP_015960396.1	probable purine permease 5
chromosome A04	107486096	XP_015962131.1	probable purine permease 11
chromosome A04	107486034	XP_015962058.2	transcription factor NAI1-like
chromosome A05	107488664	XP_015964905.1	magnesium transporter MRS2-3
chromosome A05	110281472	XP_020999420.1	ATP-dependent DNA helicase PIF1-like
chromosome A05	107488608	XP_015964852.1	DNA polymerase kappa
chromosome A05	107488668	XP_015964914.1	histone deacetylase 6

Transporter genes involved in cell wall formation (3 clusters)

chromosome A01	107491793	XP_020997455.1	probable boron transporter 2, is needed for cell wall/membrane
chromosome A01	107491758	XP_015968168.1	GDSL esterase/lipase At5g62930
chromosome A01	107491775	XP_020997468.1	GDSL esterase/lipase At5g62930-like
chromosome A01	107469510	XP_015944370.1	ABCG32: involved in cuticle formation
chromosome A01	107469463	XP_020983755.1	palmitoyl-acyl carrier protein thioesterase, chloroplastic
chromosome A01	107471942	XP_015946979.1	feruloyl CoA ortho-hydroxylase 1
chromosome A01	107471935	XP_015946967.1	palmitoyl-acyl carrier protein thioesterase, chloroplastic
chromosome A01	107471251	XP_015946193.1	palmitoyl-acyl carrier protein thioesterase, chloroplastic-like
chromosome A05	107489726	XP_015965962.1	ABCG11, cell wall development by cutin and fatty acid transport
chromosome A05	107489698	XP_015965938.2	secoisolariciresinol dehydrogenase-like; cell wall lignification

Co-localized transporter and enzyme coding genes involved in nitrogen metabolism (3 clusters)

chromosome A01	107475786	XP_015950947.1	glutamate receptor 3.6, non-selective cation (Ca) channel
chromosome A01	107475817	XP_015950983.1	ferredoxin--NADP reductase, involved in nitrite assimilation
chromosome A01	107475844	XP_015951009.1	glutamate receptor 3.6-like, non-selective cation (Ca) channel
chromosome A01	107475853	XP_015951017.1	glutamate receptor 3.6-like, non-selective cation (Ca) channel
chromosome A01	107475874	XP_020992401.1	glutamate receptor 3.3-like, non-selective cation (Ca) channel
chromosome A01	110274728	XP_020985497.1	glutamate receptor 3.1-like, non-selective cation (Ca) channel
chromosome A01	107477904	XP_015953487.1	amino acid transporter ANTL1-like
chromosome A02	107473910	XP_020990840.1	dihydroorotase, mitochondrial
chromosome A02	107473919	XP_015949002.1	ammonium transporter 1 member 4-like
chromosome A05	107487394	XP_015963508.1	glutamate receptor 3.6, non-selective cation (Ca) channel
chromosome A05	107487395	XP_020997658.1	glutamate receptor 3.6
chromosome A05	107487398	XP_015963515.1	amino acid transporter ANTL2

Auxin transporter co-localized with auxin response factors (2 clusters)

chromosome A02	107475253	XP_015950371.1	auxin response factor 17-like
chromosome A02	107475182	XP_015950287.1	auxin transporter-like protein 2
chromosome A06	107492319	XP_015968807.1	auxin-responsive protein IAA30 isoform X1
chromosome A06	107492327	XP_015968820.2	ABCB21, facultative transporter of auxin

Trace metal transporters co-localized with genes coding for mineral containing factors (2)

chromosome A09	107465190	XP_015939669.1	solute carrier family 40 member 3, iron transporter
chromosome A09	107465178	XP_015939659.2	nicotianamine synthase
chromosome A09	107465203	XP_020986978.1	putative pyridoxal kinase
chromosome A06	107494778	XP_015971299.1	probable metal-nicotianamine transporter YSL6
chromosome A06	107494779	XP_015971300.1	ABCI11, is involved in transition metal homeostasis

Sulfate transporters co-localized with enzymes and factors involved in sulfur metabolism (4 clusters)

chromosome A02	107474488	XP_020991062.1	glutathione S-transferase L3 isoform X1
chromosome A02	107474505	XP_020991066.1	sulfite exporter TauE/SafE family protein 3-like
chromosome A02	107474495	XP_020991067.1	hydroxyethylthiazole kinase, thiamine diphosphate biosynthesis
chromosome A03	107478700	XP_020993850.1	probable sulfate transporter 3.5
chromosome A03	110278517	XP_020992435.1	thiol-disulfide oxidoreductase LTO1-like
chromosome A04	107486334	XP_015962363.1	putative glutaredoxin-C14, glutathione-disulfide oxidoreductase
chromosome A04	107486419	XP_015962439.1	probable sulfate transporter 4.2

chromosome A06	107493005	XP_015969568.1	selenocysteine methyltransferase
chromosome A06	107493179	XP_015969756.1	sulfate transporter 3.1 isoform X2

Nitrate/phosphate transporters co-localized with RUBisCO large subunit genes (2 clusters)

chromosome A03	107476993	XP_015952449.2	high affinity nitrate transporter 2.4
chromosome A03	107476988	XP_015952441.1	ruBisCO large subunit-binding protein subunit alpha
chromosome A03	107477105	XP_015952556.1	ABCG28: critical for localizing polyamines
chromosome A03	107476982	XP_015952436.1	alpha carbonic anhydrase 7 isoform X2
chromosome A03	107476984	XP_020992890.1	alpha carbonic anhydrase 7-like isoform X1
chromosome A03	107481201	XP_015956907.1	phosphate transporter PHO1 homolog 3
chromosome A03	107481199	XP_015956905.1	ribulose-1,5 bisphosphate carboxylase/oxygenase large subunit N-methyltransferase, chloroplastic

Inositol transporters co-localized with inositol phosphatase and kinases (3 clusters)

chromosome A01	107466189	XP_015940667.1	inositol-phosphate phosphatase
chromosome A01	107467104	XP_015941631.1	inositol transporter 4-like
chromosome A03	107479329	XP_015954962.1	probable inositol transporter 2 isoform X1
chromosome A03	107479342	XP_015954973.1	phosphatidylinositol 4-kinase gamma 4-like
chromosome A04	107482925	XP_015958999.1	inositol-phosphate phosphatase
chromosome A04	107482922	XP_020995590.1	inositol transporter 4 isoform X2

Nicotianamine transporter co-localized with enzymes involved in nicotianamine biosynthesis (1 clusters)

chromosome A01	107495712	XP_015972366.1	probable metal-nicotianamine transporter YSL7
chromosome A01	107495914	XP_015972614.1	thioredoxin-like 3-3
chromosome A01	107495899	XP_015972602.1	S-adenosylmethionine synthase 3-like, S-adenosylmethionine is the precusor for nicotianamine.
chromosome A01	107495923	XP_015972621.1	thioredoxin-like 3-3
chromosome A01	107495906	XP_015972606.1	S-adenosylmethionine synthase 3-like

Chlorophyll catabolite transporter co-localized with chlorophyll synthase (1 cluster)

chromosome A01	107463694	XP_015938060.1	ABCC3: transpors glutathione-conjugates as well as chlorophyll catabolites
chromosome A01	107472986	XP_015948008.1	chlorophyll synthase, chloroplastic-like

Carotenoid biosynthesis (1 cluster)

chromosome A01	107465538	XP_015940004.1	prolycopene isomerase, lycopene isomerase, chloroplastic, lycopene is a carotenoid.
chromosome A01	107467559	XP_015942177.1	ABC transporter B family member 11-like?
chromosome A01	107470514	XP_015945405.1	prolycopene isomerase, lycopene isomerase, chloroplastic, lycopene is a carotenoid.

Aquila chrysaetos

#Name	GeneID	Protein product	Protein Name
**Amino acid transporters co-localized with genes involved in amino acid production or usage (5 clusters)**			
chromosome 10	115347410	XP_029884658.1	probable cationic amino acid transporter
chromosome 10	115347046	XP_029883786.1	60S ribosomal protein L22-like 1
chromosome 10	115347045	XP_029883784.1	eukaryotic translation initiation factor 5A-2 isoform X1
chromosome 10	115347053	XP_029883813.1	monocarboxylate transporter 14,a member of SLC16 family which can transport lactate, pyruvate, ketone bodies, and amino acids
chromosome 10	115347054	XP_029883816.1	F-box only protein 36 isoform X1
chromosome 10	115347050	XP_029883793.1	E3 ubiquitin-protein ligase TRIP12 isoform X1
chromosome 19	115353217	XP_029898125.1	SUMO-specific isopeptidase USPL1 isoform X1
chromosome 19	115353335	XP_029898454.1	high mobility group protein B1
chromosome 19	115353429	XP_029898689.1	ubiquitin-like protein 3
chromosome 19	115353409	XP_029898631.1	high affinity cationic amino acid transporter 1
chromosome 19	115353190	XP_029898035.1	proteasome maturation protein
chromosome 2	115337116	XP_029861028.1	tRNA (guanine(37)-N1)-methyltransferase isoform X2
chromosome 2	115337116	XP_029861021.1	tRNA (guanine(37)-N1)-methyltransferase isoform X1
chromosome 2	115352450	XP_029896523.1	probable sodium-coupled neutral amino acid transporter 6 isoform X5
chromosome 6	115342474	XP_029872542.1	E3 ubiquitin-protein ligase RNF186
chromosome 6	115342471	XP_029872534.1	transmembrane and coiled-coil domain-containing protein 4
chromosome 6	115342472	XP_029872537.1	5-hydroxytryptamine receptor 6
chromosome 6	115342475	XP_029872544.1	neuroblastoma suppressor of tumorigenicity 1 isoform X1
chromosome 6	115342442	XP_029872442.1	MICOS complex subunit MIC10
chromosome 6	115342438	XP_029872437.1	F-actin-capping protein subunit beta isoform X2
chromosome 6	115342440	XP_029872439.1	lysosomal amino acid transporter 1 homolog

chromosome 6	115342439	XP_029872438.1	aflatoxin B1 aldehyde reductase member 2
chromosome 6	115342441	XP_029872440.1	mRNA turnover protein 4 homolog
chromosome 6	115342436	XP_029872431.1	ER membrane protein complex subunit 1 isoform X1
chromosome 6	115342435	XP_029872426.1	E3 ubiquitin-protein ligase UBR4 isoform X1

Co-localized transporter and enzyme coding genes involved in energy/heme metabolism (22 clusters)

chromosome 1	115341470	XP_029870462.1	electrogenic sodium bicarbonate cotransporter 1 isoform X1
chromosome 1	115345729	XP_029880897.1	cytochrome c oxidase assembly protein COX18, mitochondrial
chromosome 10	115347317	XP_029884417.1	acetyl-coenzyme A transporter 1 isoform X1
chromosome 10	115347085	XP_029883886.1	GMP synthase, ATP + H2O + L-glutamine + XMP = AMP + diphosphate + GMP + 2 H+ + L-glutamate
chromosome 10	115347094	XP_029883918.1	solute carrier family 25 member 36, a member of clade 4 (see fig. 1) that transports pyrimidine nucleotides and folate
chromosome 10	115347186	XP_029884141.1	nicotinamide/nicotinic acid mononucleotide adenylyltransferase 3 isoform X1, catalyzes the formation of NAD+
chromosome 10	115346998	XP_029883640.1	28S ribosomal protein S22, mitochondrial isoform X2
chromosome 11	115347957	XP_029885553.1	ATP synthase membrane subunit DAPIT, mitochondrial
chromosome 11	115348123	XP_029886087.1	metal transporter CNNM2 isoform X2
chromosome 11	115348123	XP_029886086.1	metal transporter CNNM2 isoform X1
chromosome 11	115347990	XP_029885656.1	sideroflexin-2 isoform X2, SLC56A1, mitochondrial, sideroflexin-1 transports pyridoxin and serine
chromosome 11	115348152	XP_029886172.1	39S ribosomal protein L43, mitochondrial
chromosome 11	115348151	XP_029886170.1	twinkle protein, mitochondrial isoform X1, involved in mitochondrial DNA (mtDNA) metabolism
chromosome 11	115348208	XP_029886426.1	sideroflexin-3
chromosome 11	115347993	XP_029885667.1	solute carrier family 2, facilitated glucose transporter member 5-like isoform X1
chromosome 11	115347996	XP_029885680.1	metal transporter CNNM1, a metal storage protein
chromosome 11	115347998	XP_029885684.1	aspartate aminotransferase
chromosome 11	115348273	XP_029886604.1	homeobox protein Nkx-2.3 isoform X2
chromosome 11	115348414	XP_029886922.1	mitoferrin-2, mitochondrial iron transporter
chromosome 11	115348537	XP_029887253.1	ectonucleoside triphosphate diphosphohydrolase 7
chromosome 11	115348540	XP_029887260.1	V-type proton ATPase subunit e 2
chromosome 11	115348538	XP_029887254.1	cytochrome c oxidase assembly protein COX15 homolog isoform X1
chromosome 11	115348539	XP_029887258.1	copper homeostasis protein cutC homolog isoform X1
chromosome 11	115348535	XP_029887240.1	canalicular multispecific organic anion transporter 1
chromosome 12	115348885	XP_029888162.1	ATP synthase mitochondrial F1 complex assembly factor 1
chromosome 12	115348884	XP_029888159.1	EF-hand calcium-binding domain-containing protein 14 isoform X1
chromosome 12	115349129	XP_029888803.1	cytochrome P450 4B1-like
chromosome 12	115349004	XP_029888520.1	PDZK1-interacting protein 1
chromosome 12	115349003	XP_029888519.1	LOW QUALITY PROTEIN: T-cell acute lymphocytic leukemia protein 1
chromosome 12	115349067	XP_029888630.1	SCL-interrupting locus protein isoform X1
chromosome 12	115349068	XP_029888633.1	UMP-CMP kinase
chromosome 12	115349687	XP_029890138.1	LOW QUALITY PROTEIN: forkhead box protein D2
chromosome 12	115349464	XP_029889660.1	metalloprotease TIKI2
chromosome 12	115349514	XP_029889756.1	sodium/glucose cotransporter 4
chromosome 13	115350034	XP_029890933.1	cytochrome c oxidase assembly factor 6 homolog
chromosome 13	115350305	XP_029891661.1	putative thiamine transporter SLC35F3 isoform X2
chromosome 15	115351232	XP_029893655.1	coenzyme Q-binding protein COQ10 homolog A, mitochondrial
chromosome 15	115351231	XP_029893653.1	citrate synthase, mitochondrial
chromosome 16	115351592	XP_029894338.1	NADH dehydrogenase
chromosome 16	115351588	XP_029894333.1	mitochondrial carrier homolog 2
chromosome 16	115351601	XP_029894351.1	NADH dehydrogenase
chromosome 18	115353062	XP_029897885.1	magnesium transporter MRS2 homolog, mitochondrial isoform X1
chromosome 18	115353059	XP_029897878.1	phosphatidylinositol-glycan-specific phospholipase D isoform X1
chromosome 18	115353060	XP_029897881.1	succinate-semialdehyde dehydrogenase, mitochondrial
chromosome 2	115335315	XP_029856659.1	iron-sulfur cluster assembly 2 homolog, mitochondrial isoform X1
chromosome 2	115348660	XP_029887504.1	ATP-binding cassette sub-family D member 4, lysosomal cobalamin transporter
chromosome 2	115348652	XP_029887481.1	acetyl-coenzyme A synthetase 2-like, mitochondrial
chromosome 2	115352741	XP_029897226.1	methylmalonate-semialdehyde dehydrogenase, involved in inositol metabolism, valine, leucine and isoleucine (BCAA) degradation
chromosome 2	115352752	XP_029897235.1	ubiquinone biosynthesis monooxygenase COQ6, mitochondrial isoform X1
chromosome 20	115333237	XP_029851762.1	solute carrier family 26 member 6, transports Cl-, HCO3-, oxalate, OH-, and formate
chromosome 20	115333272	XP_029851842.1	cytochrome b-c1 complex subunit 1, mitochondrial
chromosome 20	115353800	XP_029899604.1	S-adenosylmethionine mitochondrial carrier protein isoform X3
chromosome 20	115353873	XP_029899795.1	monocarboxylate transporter 2-like isoform X2
chromosome 20	115333264	XP_029851838.1	succinate--CoA ligase

chromosome 21	115333684	XP_029852833.1	copper-transporting ATPase 1 isoform X1
chromosome 21	115333844	XP_029853203.1	cytochrome c oxidase subunit 7B, mitochondrial
chromosome 21	115333843	XP_029853201.1	magnesium transporter protein 1 isoform X1
chromosome 21	115333842	XP_029853196.1	transcriptional regulator ATRX isoform X1
chromosome 21	115333915	XP_029853362.1	fibroblast growth factor 16
chromosome 21	115333564	XP_029852513.1	palmitoyltransferase ZDHHC15
chromosome 21	115333565	XP_029852514.1	uracil phosphoribosyltransferase homolog
chromosome 21	115333473	XP_029852271.1	ABCB7, mitochondrial, could be involved in the transport of heme from the mitochondria to the cytosol
chromosome 27	115336529	XP_029859548.1	solute carrier family 49 member A3, slc49S transport heme.
chromosome 27	115336528	XP_029859541.1	neuronal acetylcholine receptor subunit alpha-7-like isoform X1
chromosome 27	115336590	XP_029859708.1	S-adenosylmethionine synthase-like isoform X1
chromosome 27	115336591	XP_029859713.1	ATP synthase subunit e, mitochondrial
chromosome 27	115336567	XP_029859663.1	sulfate anion transporter 1 isoform X2
chromosome 27	115336869	XP_029860400.1	neuronal acetylcholine receptor subunit alpha-6 isoform X1
chromosome 3	115338914	XP_029863828.1	COX assembly mitochondrial protein homolog isoform X1
chromosome 3	115339974	XP_029866576.1	eomesodermin homolog isoform X1
chromosome 3	115338868	XP_029863676.1	sodium bicarbonate cotransporter 3 isoform X1
chromosome 3	115339615	XP_029865691.1	asparagine synthetase
chromosome 3	115339596	XP_029865643.1	succinate dehydrogenase assembly factor 3, mitochondrial Complex II
chromosome 3	115339240	XP_029864779.1	calcium-binding mitochondrial carrier protein Aralar2, exchange cytoplasmic glutamate with mitochondrial aspartate
chromosome 3	115339369	XP_029865134.1	pyruvate dehydrogenase kinase, isozyme 4
chromosome 5	115340991	XP_029868946.1	mitochondrial thiamine pyrophosphate carrier isoform X1
chromosome 5	115341006	XP_029868996.1	28S ribosomal protein S7, mitochondrial
chromosome 5	115341013	XP_029869007.1	ATP synthase subunit d, mitochondrial
chromosome 6	115342950	XP_029874091.1	alpha-enolase isoform X1
chromosome 6	115342947	XP_029874084.1	carbonic anhydrase 6 isoform X1
chromosome 6	115342948	XP_029874086.1	solute carrier family 2, facilitated glucose transporter member 5-like isoform X1
chromosome 6	115342423	XP_029872396.1	solute carrier family 25 member 33 isoform X1
chromosome 7	115344072	XP_029876753.1	gamma-aminobutyric acid receptor subunit rho-3 isoform X1
chromosome 7	115343656	XP_029875742.1	DDB1- and CUL4-associated factor 6 isoform X8
chromosome 7	115343545	XP_029875436.1	mitochondrial pyruvate carrier 2
chromosome 9	115346210	XP_029881873.1	solute carrier family 2, facilitated glucose transporter member 11-like isoform X2
chromosome 9	115346216	XP_029881891.1	iron-sulfur cluster co-chaperone protein HscB
chromosome 19	115353404	XP_029898623.1	hemoglobin subunit epsilon
chromosome 19	115353402	XP_029898620.1	hemoglobin subunit beta
chromosome 19	115353403	XP_029898622.1	hemoglobin subunit beta-like
chromosome 19	115353405	XP_029898624.1	hemoglobin subunit rho
chromosome 19	115353399	XP_029898618.1	folate receptor gamma-like

Co-localized transporter and enzyme coding genes involved in nitrogen metabolism (1 clusters)

chromosome 27	115336968	XP_029860679.1	tubulin polyglutamylase complex subunit 2 isoform X1
chromosome 27	115336968	XP_029860680.1	tubulin polyglutamylase complex subunit 2 isoform X1
chromosome 27	115336968	XP_029860681.1	tubulin polyglutamylase complex subunit 2 isoform X2
chromosome 27	115336968	XP_029860682.1	tubulin polyglutamylase complex subunit 2 isoform X2
chromosome 27	115337348	XP_029861447.1	urea transporter 2-like, renal tubular urea transporter, influx (FEBS Letters 386 (1996) 156-160)

Trace metal transporters co-localized with genes coding for mineral containing factors (1)

chromosome 4	115340393	XP_029867814.1	zinc transporter 8
chromosome 4	115340375	XP_029867778.1	double-strand-break repair protein rad21 homolog
chromosome 4	115340634	XP_029868385.1	rRNA-processing protein UTP23 homolog isoform X1
chromosome 4	115340529	XP_029868105.1	eukaryotic translation initiation factor 3 subunit H
chromosome 4	115340519	XP_029868061.1	zinc finger transcription factor Trps1 isoform X1

Sulfate transporters co-localized with enzymes and factors involved in sulfur metabolism (2 clusters)

chromosome 22	115334255	XP_029854116.1	5-hydroxytryptamine receptor 4 isoform X1, serotonin receptor, serotonin is found as sulfate conjugates.
chromosome 22	115334043	XP_029853639.1	sulfate transporter-like isoform X1
chromosome 22	115334042	XP_029853633.1	sulfate transporter isoform X1
chromosome 5	115341962	XP_029871505.1	sodium-independent sulfate anion transporter isoform X2
chromosome 5	115341963	XP_029871507.1	N-sulphoglucosamine sulphohydrolase, catalyzes a step in lysosomal heparan sulfate degradation

Co-localized genes coding for transporter and enzymes involved in cholesterol metabolism (1 cluster)

chromosome 11	115348114	XP_029886051.1	monocarboxylate transporter 12, a member of SLC16 which can transport pyruvate, lactate, ketone bodies, and amino acids
chromosome 11	115348113	XP_029886049.1	interferon-induced protein with tetratricopeptide repeats 5-like isoform X3
chromosome 11	115348297	XP_029886656.1	putative lysosomal acid lipase/cholesteryl ester hydrolase
chromosome 11	115348512	XP_029887182.1	cholesterol 25-hydroxylase, ketones like Acetyl-CoA, acetoacetyl-CoA and 3-hydroxy- 3-methylglutaryl-CoA are precursors
chromosome 11	115348640	XP_029887466.1	lysosomal acid lipase/cholesteryl ester hydrolase-like

Choline metabolism and transport (1cluster)

chromosome 11	115348594	XP_029887375.1	LOW QUALITY PROTEIN: vesicular acetylcholine transporter
chromosome 11	115348381	XP_029886843.1	choline O-acetyltransferase isoform X1

Glutathione metabolism and transport (1 cluster)

chromosome 17	115352857	XP_029897413.1	solute carrier family 15 member 5, a member of di,tri peptide transporter family
chromosome 17	115352599	XP_029896884.1	microsomal glutathione S-transferase 1 isoform X1

Microtubule assembly (1 cluster)

chromosome 19	115353423	XP_029898674.1	microtubule-associated tumor suppressor candidate 2 isoform X5
chromosome 19	115353423	XP_029898673.1	Microtubule-associated tumor suppressor candidate 2 isoform X4
chromosome 19	115353189	XP_029898034.1	Solute carrier family 46 member 3; prabable lysosomal export of maytansine conjugates

Thiamine transporters co-localized with the coagulation factor V coding gene (1 cluster)

chromosome 7	115343608	XP_029875650.1	coagulation factor V isoform X1
chromosome 7	115343608	XP_029875651.1	coagulation factor V isoform X2
chromosome 7	115343611	XP_029875662.1	thiamine transporter 1

Homo sapiens

#Name	GeneID	Protein product	Protein Name
**Amino acid transporters co-localized with genes involved in amino acid production or usage (7 clusters)**			
chromosome 1	23352	NP_065816.2	E3 ubiquitin-protein ligase UBR4
chromosome 1	54896	XP_005245973.1	lysosomal amino acid transporter 1 homolog, mediates the pH-dependent export of the cationic amino acids arginine, histidine and lysine from lysosomes
chromosome 1	164153	NP_981957.1	ubiquitin-like protein 4B
chromosome 1	388662	NP_001010898.1	sodium-dependent neutral amino acid transporter SLC6A17
chromosome 2	57162	XP_016860009.1	E3 ubiquitin-protein ligase pellino homolog
chromosome 2	6509	NP_003029.2	neutral amino acid transporter A
chromosome 11	347862	XP_024304258.1	glutamine amidotransferase-like class 1 domain-containing protein 1
chromosome 11	79751	NP_078974.1	mitochondrial glutamate carrier 1, involved in the transport of glutamate across the inner mitochondrial membrane. Glutamate is cotransported with H+
chromosome 14	9056	NP_003973.3	Y+L amino acid transporter 1
chromosome 14	122704	NP_848026.1	39S ribosomal protein L52, mitochondrial
chromosome 14	57570	NP_001337182.1	tRNA (guanine(37)-N1)-methyltransferase
chromosome 14	145389	NP_001166173.1	probable sodium-coupled neutral amino acid transporter 6
chromosome X	92745	XP_005272751.2	sodium-coupled neutral amino acid transporter 5
chromosome X	24140	XP_024308127.1	putative tRNA (cytidine(32)/guanosine(34)-2\\'-O)-methyltransferase

Co-localized transporter and enzyme coding genes involved in energy/heme metabolism (19 clusters)

chromosome 1	50651	NP_001366543.1	proton-associated sugar transporter A
chromosome 1	2023	NP_001419.1	alpha-enolase; involved in glycolysis by producing PEP
chromosome 1	765	XP_011540386.1	carbonic anhydrase 6, involved in reversible hydration of carbon dioxide producing bicarbonate as precursor for citrate and OAA (TCA)
chromosome 1	155184	XP_011539126.1	solute carrier family 2, facilitated glucose transporter member 7
chromosome 1	148641	NP_775779.1	putative thiamine transporter SLC35F3
chromosome 1	388753	NP_001193570.2	cytochrome c oxidase assembly factor 6
chromosome 1	25874	NP_001137146.1	mitochondrial pyruvate carrier 2
chromosome 1	10560	NP_001306596.1	thiamine transporter 1
chromosome 2	8604	NP_003696.2	calcium-binding mitochondrial carrier protein Aralar1, aspartate/glutamate transporter
chromosome 2	254042	XP_016859239.1	methionine aminopeptidase 1D, mitochondrial, release of N-terminal amino acids, preferentially methionine, from peptides and arylamides
chromosome 2	5163	NP_001265478.1	[Pyruvate dehydrogenase (acetyl-transferring)] kinase, mitochondrial

chromosome 4	521	NP_009031.1	ATP synthase subunit e, mitochondrial
chromosome 4	84179	XP_024310012.1	solute carrier family 49 member A3, Unknown substrate, SLC4949A1 and 49A2 are BCAA transporters
chromosome 4	8671	XP_024310036.1	electrogenic sodium bicarbonate cotransporter 1, bicarbonate is a precursor for citrate and OAA (TCA)
chromosome 4	285521	NP_001287658.1	cytochrome c oxidase assembly protein COX18, mitochondrial
chromosome 6	57380	NP_001273193.1	magnesium transporter MRS2 homolog, mitochondrial, Mitochondrial Mg2+ homeostasis decides cellular energy metabolism
chromosome 6	7915	NP_733936.1	succinate-semialdehyde dehydrogenase, mitochondrial
chromosome 7	5166	NP_002603.1	pyruvate dehydrogenase kinase, isozyme 4
chromosome 7	10165	NP_055066.1	calcium-binding mitochondrial carrier protein Aralar2, exchange cytoplasmic glutamate with mitochondrial aspartate
chromosome 7	57001	NP_064571.1	succinate dehydrogenase assembly factor 3, mitochondrial Complex II
chromosome 7	440	NP_001339425.1	asparagine synthetase
chromosome 9	92014	NP_219480.1	solute carrier family 25 member 51, a member of slc25 clade 5 (see Fig. 1) in which members transport carnitine/acylcarnitine or amino acids
chromosome 9	219	NP_000683.3	aldehyde dehydrogenase X, mitochondrial precursor, an aldehyde + H2O + NAD+ = a carboxylate + 2 H+ + NADH
chromosome 10	118980	XP_024303560.1	
chromosome 10	54805	NP_060119.3	
chromosome 10	84833	XP_024304005.1	
chromosome 11	2542	NP_001157750.1	glucose-6-phosphate exchanger SLC37A4
chromosome 11	10525	XP_016872584.1	hypoxia up-regulated protein 1, has a pivotal role in cytoprotective cellular mechanisms triggered by oxygen deprivation
chromosome 11	3145	NP_000181.2	porphobilinogen deaminase, involved in the third step of the heme biosynthetic
chromosome 10	26507	NP_001332816.1	metal transporter CNNM1, a metal storage protein
chromosome 10	2805	NP_002070.1	aspartate aminotransferase
chromosome 10	81894	NP_112489.3	mitoferrin-2, mitochondrial iron transporter
chromosome 10	1355	NP_001307903.1	cytochrome c oxidase assembly protein COX15 homolog isoform 2, involved in the biosynthesis of heme A
chromosome 10	51076	NP_057044.2	copper homeostasis protein cutC homolog
chromosome 10	1244	NP_000383.2	canalicular multispecific organic anion transporter 1
chromosome 12	93058	NP_653177.3	coenzyme Q-binding protein COQ10 homolog A, mitochondrial isoform a
chromosome 12	1431	NP_004068.2	citrate synthase, mitochondrial
chromosome 17	8402	XP_024306762.1	mitochondrial 2-oxoglutarate/malate carrier protein isoform X1
chromosome 17	2027	NP_001361453.1	beta-enolase isoform 3, produces phosphoenolpyruvate
chromosome 17	55065	NP_001098047.1	solute carrier family 52, riboflavin transporter
chromosome 20	64849	NP_073740.2	solute carrier family 13 member 3, plasma membrane high-affinity sodium-dicarboxylate cotransporter
chromosome 20	81031	XP_011527362.1	solute carrier family 2, facilitated glucose transporter member 10
chromosome X	5207	XP_016885065.1	6-phosphofructo-2-kinase/fructose-2,6-bisphosphatase 1 isoform X1
chromosome X	212	NP_001033057.1	5-aminolevulinate synthase, erythroid-specific, mitochondrial
chromosome X	84061	NP_001354845.1	magnesium transporter protein 1
chromosome X	1349	NP_001857.1	cytochrome c oxidase subunit 7B, mitochondrial precursor
chromosome X	441531	NP_001025062.1	phosphoglycerate mutase 4, is an important enzyme in the glycolytic pathway and catalyzes the transfer of phosphate groups
chromosome X	538	NP_000043.4	copper-transporting ATPase 1
chromosome 14	51004	XP_011535109.1	ubiquinone biosynthesis monooxygenase COQ6, mitochondrial isoform X1
chromosome 14	4329	NP_001265522.1	methylmalonate-semialdehyde dehydrogenase, involved in inositol metabolism, valine, leucine and isoleucine (BCAA) degradation
chromosome 14	5826	NP_005041.1	lysosomal cobalamin transporter ABCD4
chromosome 14	122961	NP_919255.2	iron-sulfur cluster assembly 2 homolog, mitochondrial isoform 1 precursor
chromosome 18	25941	NP_001258880.1	tubulin polyglutamylase complex subunit 2 isoform 4
chromosome 18	8170	XP_024307038.1	urea transporter 2, renal tubular urea transporter, influx (FEBS Letters 386 (1996) 156-160)
chromosome 18	498	NP_001244263.1	ATP synthase subunit alpha, mitochondrial isoform b precursor

Co-localized iron transporter and hypoxia/nutrient-deprivation related enzyme asparagine synthetase genes (1 cluster)

chromosome 2	30061	NP_055400.1	solute carrier family 40 member 1, ferroportin (FPN1), iron transporters
chromosome 2	54529	NP_001340426.1	asparagine synthetase domain-containing protein 1

Co-localized transporter and enzyme coding genes involved in nitrogen metabolism (urea cycle), and polyamine biosynthesis (1 cluster)

chromosome 1	79814	NP_079034.3	agmatinase, mitochondrial precursor, agmatine + H2O = putrescine + urea
chromosome 1	6248	NP_006502.1	regulatory solute carrier protein family 1 member 1
chromosome 1	284723	NP_997231.1	solute carrier family 25 member 34, a member of slc25 clade 5 (see Fig. 1) in which members transport carnitine/acylcarnitine or amino acids

Co-localized genes coding for heme degrading enzyme with myoglobin (1 cluster)

chromosome 22	3162	NP_002124.1	heme oxygenase 1
chromosome 22	4151	NP_001349775.1	myoglobin

Trace metal transporters co-localized with genes coding for mineral containing factors (2)

chromosome 2	51374	NP_001164266.1	all-trans retinoic acid-induced differentiation factor
chromosome 2	7781	XP_005264605.1	zinc transporter 3
chromosome 6	6257	NP_001257330.1	retinoic acid receptor RXR-beta, zinc binding
chromosome 6	7922	NP_008910.2	zinc transporter SLC39A7

Co-localized transporter and enzyme coding genes involved in sulfur metabolism (1 cluster)

chromosome 17	6448	NP_000190.1	N-sulphoglucosamine sulphohydrolase isoform 1 precursor, catalyzes a step in lysosomal heparan sulfate degradation by releasing glucosamine and sulfate
chromosome 17	284129	XP_016879994.1	sodium-independent sulfate anion transporter

Co-localized genes coding for transporter and enzymes involved in cholesterol metabolism (1 cluster)

chromosome 10	9023	NP_003947.1	cholesterol 25-hydroxylase, ketones like Acetyl-CoA, acetoacetyl-CoA and 3-hydroxy- 3-methylglutaryl-CoA are precursors
chromosome 10	3988	XP_024303791.1	lysosomal acid lipase/cholesteryl ester hydrolase
chromosome 10	387700	XP_016871726.1	monocarboxylate transporter 12, unknown substrate, a member of SLC16 which can transport pyruvate, lactate, ketone bodies, and amino acids

Choline transport and metabolism (2 clusters)

chromosome 10	6572	NP_003046.2	vesicular acetylcholine transporter
chromosome 10	1103	NP_065574.4	choline O-acetyltransferase
chromosome 20	1137	NP_000735.1	neuronal acetylcholine receptor subunit alpha-4
chromosome 20	3785	NP_001369164.1	potassium voltage-gated channel subfamily KQT member 2 isoform c

Co-localized transporter and enzyme coding genes involved in sugar metabolism (1 cluster)

chromosome 1	55974	NP_061333.2	sugar transporter SWEET1
chromosome 1	54344	NP_061846.2	dolichol-phosphate mannosyltransferase subunit 3 isoform 1, inolved in protein glycosylation

Glutathione metabolism and transport (1 cluster)

chromosome 12	729025	NP_001164269.1	solute carrier family 15 member 5, unknown substrate, a member of di,tri peptide transporter family,
chromosome 12	4257	NP_665707.1	microsomal glutathione S-transferase 1, glutathione is tri peptide glutamate cysteine glycine

Thiamine transporter co-localized with the coagulation factor V coding gene (1 cluster)

chromosome 1	10560	NP_001306596.1	thiamine transporter 1
chromosome 1	2153	NP_000121.2	coagulation factor V preproprotein

Oryzias latipes

#Name	GeneID	Protein product	Protein Name
**Amino acid transporters co-localized with genes involved in amino acid production or usage (4 clusters)**			
chromosome 17	101159534	XP_004079220.1	calcium-binding mitochondrial carrier protein Aralar1 isoform X3, aspartate/glutamate transporter
chromosome 17	101167074	XP_023821182.1	methionine aminopeptidase 1D, mitochondrial, release of N-terminal amino acids, preferentially methionine, from peptides and arylamides
chromosome 19	101166749	XP_011486466.1	E3 ubiquitin-protein ligase BRE1B isoform X1
chromosome 19	101167994	XP_004080585.1	vesicular glutamate transporter 1-like
chromosome 19	101168248	XP_004080586.1	sodium-dependent neutral amino acid transporter B(0)AT2
chromosome 22	101165114	XP_023807184.1	mitochondrial basic amino acids transporter isoform X1
chromosome 22	101165363	XP_011488599.1	tryptophan--tRNA ligase, cytoplasmic isoform X1
chromosome 23	101165455	XP_023808136.1	sodium-coupled neutral amino acid transporter 2
chromosome 23	111946956	XP_023808295.1	28S ribosomal protein S33, mitochondrial isoform X1

Co-localized transporter and enzyme coding genes involved in energy/heme metabolism (25 clusters)

chromosome 1	101160958	XP_011487388.2	mitoferrin-2
chromosome 1	101156301	XP_004065522.1	aspartate aminotransferase, cytoplasmic-like
chromosome 1	101161214	XP_011487452.3	metal transporter CNNM1
chromosome 1	101161460	XP_023813815.1	4-hydroxy-2-oxoglutarate aldolase, mitochondrial
chromosome 1	101157032	XP_004065525.1	hexokinase-1 isoform X2
chromosome 1	101158169	XP_004065918.1	succinate--CoA ligase
chromosome 1	101158419	XP_004065919.1	CDGSH iron-sulfur domain-containing protein 2

chromosome 10	101175414	XP_004073344.1	solute carrier family 25 member 51, a member of slc25 clade 5 (see Fig. 1) in which members transport carnitine/acylcarnitine, ornithine, citrulline, lysine
chromosome 10	101154959	XP_023814961.1	glyoxylate reductase/hydroxypyruvate reductase
chromosome 10	101164409	XP_004073379.1	ATP synthase subunit O, mitochondrial
chromosome 10	101164654	XP_004073380.1	sideroflexin-1, SLC56A1, mitochondrial, transports pyridoxin and serine
chromosome 10	101164902	XP_023815009.1	copper-transporting ATPase 1 isoform X1
chromosome 10	101160919	XP_011478582.1	probable phospholipid-transporting ATPase VB
chromosome 10	101165154	XP_004073382.1	gamma-aminobutyric acid receptor subunit beta-2
chromosome 10	101166394	XP_004073387.1	cytochrome b-c1 complex subunit 8, component of the ubiquinol-cytochrome c oxidoreductase
chromosome 11	101163911	XP_023816060.1	cytochrome c oxidase subunit 6B1-like isoform X1
chromosome 11	101173640	XP_011479277.1	solute carrier family 2, facilitated glucose transporter member 1
chromosome 12	101167557	XP_020563378.1	solute carrier family 2, facilitated glucose transporter member 5
chromosome 12	101175176	XP_004074866.1	sodium/glucose cotransporter 1-like isoform X1
chromosome 12	101168801	XP_023816192.1	NAD(P) transhydrogenase, mitochondrial-like
chromosome 12	101156420	XP_004074874.1	NADH dehydrogenase
chromosome 14	101156598	XP_023818374.1	argininosuccinate lyase, 2-(Nω-L-arginino)succinate = fumarate + L-arginine
chromosome 14	101161258	XP_004076308.2	carbonic anhydrase 4 isoform X1, H^+ + hydrogencarbonate = CO_2 + H_2O
chromosome 14	101156839	XP_004076205.1	fatty aldehyde dehydrogenase
chromosome 14	101157076	XP_023818375.1	vitronectin
chromosome 14	101161499	XP_011481551.1	solute carrier family 13 member 2, succinate, citrate, ketoglutarate transporters
chromosome 15	101157390	XP_020565237.1	sideroflexin-1, SLC56A1, mitochondrial, transports pyridoxin and serine
chromosome 15	101157390	XP_011482752.1	sideroflexin-1
chromosome 15	101160342	XP_004077179.1	S-adenosylmethionine synthase
chromosome 15	101163514	XP_004077193.1	mitoferrin-2, mitochondrial iron transporter
chromosome 15	101160836	XP_004077432.1	homeobox protein zampogna
chromosome 15	101163760	XP_004077194.1	homeobox protein Nkx-2.3
chromosome 15	101163996	XP_004077195.1	aspartate aminotransferase, cytoplasmic
chromosome 15	101161081	XP_004077433.2	metal transporter CNNM1, a metal storage protein
chromosome 15	101165337	XP_020565260.1	canalicular multispecific organic anion transporter 1, conjugated bilirubin exporter
chromosome 15	101165753	XP_004077203.1	copper homeostasis protein cutC homolog
chromosome 15	101165997	XP_004077204.1	cytochrome c oxidase assembly protein COX15 homolog
chromosome 16	101171357	XP_004077929.1	peroxisomal carnitine O-octanoyltransferase
chromosome 16	101171435	XP_023819737.1	multidrug resistance protein 1
chromosome 16	101171859	XP_011483557.1	solute carrier family 25 member 40, unknown substrate
chromosome 16	101168982	XP_004078004.1	cytochrome c oxidase subunit 6B1
chromosome 16	101167147	XP_023820222.1	solute carrier family 2, facilitated glucose transporter member 3
chromosome 16	101170049	XP_004078009.1	gamma-aminobutyric acid receptor-associated protein-like 1
chromosome 16	101173578	XP_023820299.1	gamma-aminobutyric acid type B receptor subunit 2-like isoform X2
chromosome 16	101165503	XP_004078479.1	ribonuclease P protein subunit p25-like protein
chromosome 16	101159164	XP_023820453.1	succinate-semialdehyde dehydrogenase, mitochondrial
chromosome 18	101158482	XP_004079842.1	gamma-aminobutyric acid receptor-associated protein
chromosome 18	101158976	XP_004079844.1	solute carrier family 2, facilitated glucose transporter member 4
chromosome 21	101155546	XP_023806522.1	NADP-dependent malic enzyme, (S)-malate + $NADP^+$ = CO_2 + NADPH + pyruvate (in cytosol)
chromosome 21	101172615	XP_004081547.1	mitochondrial pyruvate carrier 2
chromosome 21	101171400	XP_023806482.1	glucose-6-phosphate exchanger SLC37A1
chromosome 21	110014890	XP_020557787.1	NADH dehydrogenase
chromosome 5	101159554	XP_023810430.1	sodium- and chloride-dependent GABA transporter 2
chromosome 5	101166609	XP_023810452.1	solute carrier family 2, facilitated glucose transporter member 11 isoform X1
chromosome 5	100049362	XP_004086202.4	pyruvate dehydrogenase E1 component subunit beta, mitochondrial
chromosome 5	101162907	XP_020557101.1	electroneutral sodium bicarbonate exchanger 1 isoform X2, bicarbonate is a precursor for citrate and OAA (TCA)
chromosome 5	101172797	XP_020557127.1	NADH dehydrogenase
chromosome 5	101172312	XP_004086151.1	solute carrier family 25 member 34, a member of slc25 clade 5 (see Fig. 1) in which members transport carnitine/acylcarnitine, ornithine, citrulline, lysine
chromosome 5	101171309	XP_004086147.3	succinate dehydrogenase
chromosome 6	101175646	XP_020559925.1	NADH dehydrogenase
chromosome 6	101160478	XP_023811844.1	mitochondrial glutamate carrier 1 isoform X2

chromosome 7	101163325	XP_023812312.1	enolase isoform X1
chromosome 7	101155193	XP_004070439.1	carbonic anhydrase 6
chromosome 7	101154948	XP_020560239.1	solute carrier family 2, facilitated glucose transporter member 5 isoform X1
chromosome 7	101174649	XP_004086393.1	NADH dehydrogenase
chromosome 7	101161039	XP_004086422.1	solute carrier family 2, facilitated glucose transporter member 1-like
chromosome 15	101164916	XP_023819233.1	phytanoyl-CoA hydroxylase-interacting protein-like isoform X1
chromosome 15	101165170	XP_023819454.1	monocarboxylate transporter 9, unknown substrate, a member of SLC16 which can transport pyruvate, lactate, ketone bodies, and amino acids
chromosome 24	101175286	XP_011490258.1	ATP-binding cassette sub-family D member 4, lysosomal cobalamin transporter
chromosome 24	101167010	XP_023808739.1	acetyl-coenzyme A synthetase 2-like, mitochondrial
chromosome 24	101168512	XP_004083815.1	cytochrome c oxidase subunit 7A2, mitochondrial
chromosome 12	101160426	XP_011479961.2	urea transporter 1, renal tubular urea transporter, influx (FEBS Letters 386 (1996) 156-160)
chromosome 12	101169376	XP_004074612.1	ATP synthase subunit alpha, mitochondrial

Nucleotide metabolism and transport (2 clusters)

chromosome 1	101169415	XP_004065721.1	UTP--glucose-1-phosphate uridylyltransferase isoform X1
chromosome 1	101169661	XP_004065722.1	E3 ubiquitin-protein ligase pellino homolog 1
chromosome 1	101174399	XP_011471832.3	equilibrative nucleoside transporter 2
chromosome 15	101162945	XP_004077105.1	adenylosuccinate synthetase isozyme 2, plays an important role in the de novo pathway and in the salvage pathway of purine nucleotide biosynthesis.
chromosome 15	101163194	XP_004077106.1	equilibrative nucleoside transporter 1

Nucleotide and Mg transporters co-localized with DNA/RNA polymerization enzymes and factors (2 clusters)

chromosome 10	101155359	XP_023814762.1	mediator of RNA polymerase II transcription subunit 12 isoform X1
chromosome 10	101155608	XP_004073114.1	methionine--tRNA ligase, mitochondrial
chromosome 10	111947970	XP_023814763.1	uncharacterized protein LOC111947970
chromosome 10	101155856	XP_004073115.1	magnesium transporter NIPA2
chromosome 12	101158782	XP_023816390.1	metal transporter CNNM4 isoform X1
chromosome 12	101164170	NP_001265813.1	sialidase-3-like
chromosome 12	101159030	XP_023816293.1	metal transporter CNNM3 isoform X1
chromosome 12	101159280	XP_023816856.1	interleukin-1 beta
chromosome 12	101164417	XP_004074508.2	transcriptional activator protein Pur-beta
chromosome 12	101159520	XP_023816292.1	homeobox protein MSX-3
chromosome 12	101159763	XP_023816289.1	DNA-directed DNA/RNA polymerase mu isoform X1

Trace metal transporters co-localized with genes coding for mineral containing factors (2)

chromosome 22	101164605	XP_004082140.2	retinol dehydrogenase 12 isoform X1
chromosome 22	101172541	XP_023807457.1	zinc transporter ZIP9, (Zn is fundamental for the synthesis of retinol-binding protein and is required for the conversion of retinol to retinal for dark adaptation)
chromosome 10	101174600	XP_023814746.1	glutathione reductase, mitochondrial isoform X1
chromosome 10	101167048	XP_004073076.1	zinc transporter 9 isoform X1
chromosome 10	101174839	XP_011478175.1	glutaredoxin domain-containing cysteine-rich protein 1

Sulfate transporters co-localized with enzymes and factors involved in sulfur metabolism (2 clusters)

chromosome 1	101155010	XP_011477359.1	thioredoxin domain-containing protein 2 isoform X2
chromosome 1	101170906	XP_020560983.1	sodium-independent sulfate anion transporter
chromosome 1	101155495	XP_004066210.3	N-sulphoglucosamine sulphohydrolase isoform X1
chromosome 10	101169619	XP_020562484.1	sulfate transporter
chromosome 10	101173638	XP_011478864.1	5-hydroxytryptamine receptor 4 isoform X1

Choline metabolism and transport (2 clusters)

chromosome 15	101157241	XP_004077167.1	probable vesicular acetylcholine transporter-A
chromosome 15	101156191	XP_011482737.1	choline O-acetyltransferase isoform X1
chromosome 19	101163117	XP_023805494.1	probable vesicular acetylcholine transporter-B
chromosome 19	101173179	XP_020567830.1	choline O-acetyltransferase isoform X1

Co-localized genes coding for transporter and enzymes involved in cholesterol metabolism (1 cluster)

chromosome 19	101156001	XP_023805079.1	lysosomal acid lipase/cholesteryl ester hydrolase-like
chromosome 19	101164274	XP_004084712.1	cholesterol 25-hydroxylase, ketones like Acetyl-CoA, acetoacetyl-CoA and 3-hydroxy- 3-methylglutaryl-CoA are precursors
chromosome 19	101161384	XP_004086853.1	monocarboxylate transporter 12, unknown substrate, a member of SLC16 which can transport pyruvate, lactate, ketone bodies, and amino acids

Table S3. Enrichment rates of genes for co-localization with their co-expressed and co-interacting genes

Homo sapiens

Locus	Protein product	Co-expressions and co-interactions		Co-localization likelihood fold-change (FC)	Locus	Protein product	Co-expressions and co-interactions		Co-localization likelihood fold-change (FC)
		Total	witihin ±10 neighbor genes				Total	witihin ±10 neighbor genes	
OR4F5	NP_001005484.2	30	0	0.00	APOC4	NP_001637.1	116	4	32.76
OR4F29	NP_001005221.2	38	0	0.00	APOC2	NP_000474.2	527	8	14.42
OR4F16	XP_016857897.1	14	0	0.00	CLPTM1	NP_001269104.1	308	10	30.84
SAMD11	NP_001372569.1	216	7	47.36	RELB	NP_006500.2	1179	5	4.03
NOC2L	NP_056473.3	1128	6	7.22	CLASRP	XP_011524698.1	433	6	13.16
KLHL17	XP_006710663.1	334	6	22.75	ZNF296	NP_660331.1	315	6	18.09
PLEKHN1	XP_011540550.2	120	7	69.27	GEMIN7	XP_016882801.1	328	5	14.48
PERM1	NP_001278296.2	147	6	45.62	PPP1R37	NP_061994.1	93	9	91.93
HES4	XP_005244828.1	335	7	22.06	NKPD1	XP_011525107.1	771	8	9.86
ISG15	NP_005092.1	2612	2	0.77	TRAPPC6A	NP_001257820.1	327	12	34.86
AGRN	XP_011539731.1	1248	9	6.85	BLOC1S3	NP_997715.1	220	9	38.86
RNF223	NP_001192181.1	188	0	0.00	EXOC3L2	NP_001369351.1	550	9	15.54
C1orf159	NP_060361.4	55	0	0.00	MARK4	NP_001186796.1	388	11	26.93
TTLL10	XP_016856401.1	320	3	8.91	CKM	NP_001815.2	662	2	2.87
TNFRSF18	NP_683699.1	521	3	5.47	KLC3	XP_024307137.1	917	13	13.47
TNFRSF4	XP_016857721.1	747	1	1.27	ERCC2	XP_011524913.1	980	12	11.63
SDF4	XP_024303241.1	509	9	16.80	PPP1R13L	XP_016881666.1	223	10	42.60
B3GALT6	NP_542172.2	226	5	21.02	POLR1G	NP_001284519.1	314	15	45.38
C1QTNF12	XP_011539738.1	173	1	5.49	ERCC1	NP_001356346.1	883	9	9.68
UBE2J2	NP_477515.2	629	9	13.59	FOSB	NP_006723.2	1036	7	6.42
SCNN1D	NP_001123885.2	264	3	10.79	RTN2	NP_996783.1	663	14	20.06
ACAP3	XP_005244772.1	737	8	10.31	PPM1N	NP_001073870.1	432	13	28.59
PUSL1	XP_024308825.1	513	2	3.70	VASP	NP_003361.1	981	9	8.72
INTS11	NP_001243392.1	662	5	7.17	OPA3	NP_001017989.2	379	11	27.57
CPTP	XP_005244858.1	257	6	22.18	GPR4	XP_016882096.1	362	3	7.87
TAS1R3	XP_016857924.1	626	5	7.59	EML2	NP_001338981.1	716	11	14.59
DVL1	NP_004412.2	1588	8	4.79	GIPR	XP_011525011.1	484	2	3.93
MXRA8	XP_016857006.1	556	12	20.50	SNRPD2	NP_001371576.1	1697	6	3.36
AURKAIP1	NP_001120701.1	484	1	1.96	QPCTL	NP_060129.2	284	5	16.72
CCNL2	NP_112199.2	565	3	5.04	FBXO46	NP_001316563.1	123	4	30.89
MRPL20	NP_060441.2	830	2	2.29	MEIOSIN	XP_011525873.1	116	8	65.51
ANKRD65	XP_016856813.1	928	6	6.14	SIX5	NP_787071.3	498	6	11.45
TMEM88B	NP_001140157.1	510	1	1.86	DMPK	NP_001075031.1	1244	7	5.35
VWA1	NP_073745.2	764	7	8.70	DMWD	NP_004934.1	724	6	7.87
ATAD3C	NP_001034300.2	398	2	4.77	RSPH6A	NP_110412.1	287	6	19.86
ATAD3B	XP_005244863.1	429	5	11.07	SYMPK	NP_004810.2	1071	6	5.32
ATAD3A	NP_060658.3	693	7	9.60	FOXA3	NP_004488.2	759	1	1.25
TMEM240	NP_001108220.1	291	4	13.06	IRF2BP1	NP_056464.1	347	1	2.74
SSU72	NP_054907.1	819	1	1.16	MYPOP	XP_016882231.1	48	1	19.79
FNDC10	NP_001229588.1	22	0	0.00	NANOS2	NP_001025032.1	547	0	0.00
MIB2	XP_016855838.1	2913	4	1.30	NOVA2	NP_002507.1	570	1	1.67
MMP23B	XP_016858104.1	214	4	17.76	CCDC61	XP_005259248.1	150	0	0.00
CDK11B	NP_001278274.1	950	4	4.00	PGLYRP1	NP_005082.1	483	2	3.93
SLC35E2B	XP_024305278.1	271	6	21.03	IGFL4	NP_001002923.1	33	0	0.00
CDK11A	NP_277071.2	635	3	4.49	IGFL3	XP_011525255.1	31	0	0.00
SLC35E2A	NP_001186716.1	272	6	20.95	IGFL2	NP_001128585.1	77	0	0.00
NADK	NP_001340570.1	599	3	4.76	IGFL1	NP_940943.1	113	1	8.41
GNB1	XP_016856548.1	2088	13	5.91	HIF3A	XP_016882621.1	542	3	5.26
CALML6	XP_005244786.2	1052	8	7.22	PPP5C	XP_016882424.1	2147	4	1.77
TMEM52	NP_848640.1	103	5	46.11	CCDC8	NP_114429.2	376	3	7.58
CFAP74	NP_001291289.1	133	6	42.85	PNMA8A	NP_001096619.1	348	2	5.46
GABRD	NP_000806.2	1164	14	11.43	PPP5D1	NP_001192210.1	1818	3	1.57
PRKCZ	XP_016857279.1	1707	11	6.12	PNMA8B	NP_065760.1	2191	3	1.30
FAAP20	NP_001139782.1	113	0	0.00	CALM3	NP_005175.2	2211	4	1.72
SKI	XP_005244832.1	289	4	13.15	PTGIR	NP_000951.1	543	3	5.25
MORN1	NP_079124.1	160	5	29.69	GNG8	XP_011525826.1	772	4	4.92
RER1	NP_008964.3	525	1	1.81	DACT3	NP_659493.2	468	7	14.21
PEX10	NP_002608.1	449	7	14.81	PRKD2	NP_001073349.1	343	6	16.62

PLCH2	XP_024306827.1	902	8	8.43	STRN4	NP_037535.2	453	6	12.58
PANK4	NP_060686.3	376	11	27.79	FKRP	XP_016882786.1	350	5	13.57
HES5	NP_001010926.1	755	1	1.26	SLC1A5	NP_005619.1	669	1	1.42
TNFRSF14	NP_003811.2	670	2	2.84	AP2S1	NP_067586.1	687	3	4.15
PRXL2B	XP_006710417.1	211	3	13.51	ARHGAP35	NP_004482.4	765	6	7.45
MMEL1	XP_016857801.1	337	6	16.91	NPAS1	NP_002508.2	479	5	9.92
TTC34	NP_001229601.2	60	1	15.83	TMEM160	NP_060324.1	308	4	12.34
ACTRT2	NP_536356.3	1014	11	10.31	ZC3H4	XP_016882019.1	791	3	3.60
PRDM16	NP_071397.3	924	8	8.22	SAE1	NP_001139185.1	1172	8	6.48
ARHGEF16	XP_016856538.1	794	9	10.77	BBC3	NP_001120714.1	506	1	1.88
MEGF6	XP_016856022.1	314	15	45.38	CCDC9	XP_016882067.1	84	2	22.62
TPRG1L	NP_877429.2	112	10	84.82	INAFM1	NP_848606.3	40	0	0.00
WRAP73	XP_005244811.1	272	13	45.40	C5AR1	NP_001727.2	885	2	2.15
TP73	NP_005418.1	895	10	10.61	C5AR2	NP_001258678.1	243	1	3.91
CCDC27	XP_016855892.1	42	12	271.41	DHX34	NP_055496.2	960	6	5.94
SMIM1	NP_001366620.1	109	2	17.43	MEIS3	NP_001333077.1	228	4	16.67
LRRC47	NP_065761.1	791	14	16.81	SLC8A2	XP_005259229.1	1163	3	2.45
CEP104	XP_024306871.1	243	13	50.82	KPTN	XP_016881717.1	226	0	0.00
DFFB	NP_001269598.1	301	9	28.40	NAPA	NP_003818.2	531	1	1.79
C1orf174	NP_997239.2	44	10	215.90	ZNF541	XP_005259371.1	489	4	7.77
AJAP1	NP_001035943.1	402	14	33.08	BICRA	NP_056526.3	218	3	13.07
NPHP4	XP_011539517.1	551	7	12.07	EHD2	NP_055416.2	568	2	3.34
KCNAB2	NP_001186789.1	780	10	12.18	NOP53	NP_056525.2	968	4	3.93
CHD5	NP_056372.1	1752	7	3.80	SELENOW	NP_003000.1	224	4	16.96
RPL22	NP_000974.1	1215	8	6.25	TPRX1	NP_940881.2	201	3	14.18
RNF207	XP_011539741.1	232	8	32.76	CRX	NP_000545.1	693	7	9.60
ICMT	NP_036537.1	523	9	16.35	SULT2A1	NP_003158.2	552	7	12.05
HES3	NP_001019769.1	340	5	13.97	BSPH1	XP_016881607.1	110	4	34.54
GPR153	XP_016856739.1	326	6	17.48	ELSPBP1	XP_016882619.1	181	6	31.49
ACOT7	NP_009205.3	504	10	18.85	CABP5	XP_016882481.1	1089	8	6.98
HES2	NP_061962.2	148	2	12.84	PLA2G4C	NP_001152794.1	295	5	16.10
ESPN	XP_016857922.1	1242	3	2.29	LIG1	NP_001275992.1	1472	2	1.29
TNFRSF25	NP_683871.1	945	2	2.01	ZSWIM9	XP_006723267.1	41	10	231.70
PLEKHG5	NP_001036128.2	684	1	1.39	CARD8	XP_016881974.1	454	2	4.18
NOL9	NP_078930.4	488	5	9.73	ZNF114	XP_016881903.1	212	1	4.48
TAS1R1	NP_619642.2	497	2	3.82	CCDC114	NP_001351100.1	220	0	0.00
ZBTB48	NP_005332.1	325	3	8.77	EMP3	NP_001416.1	371	1	2.56
KLHL21	NP_055666.2	577	6	9.88	TMEM143	NP_001290469.1	107	0	0.00
PHF13	NP_722519.2	612	6	9.31	SYNGR4	NP_036583.2	159	3	17.92
THAP3	NP_612359.2	133	9	64.28	KDELR1	NP_006792.1	781	1	1.22
DNAJC11	NP_060668.2	600	6	9.50	GRIN2D	NP_000827.2	622	4	6.11
CAMTA1	XP_011539385.1	424	12	26.89	GRWD1	NP_113673.3	903	1	1.05
VAMP3	NP_004772.1	1127	12	10.11	KCNJ14	NP_037480.1	194	4	19.59
PER3	NP_001364204.1	413	10	23.00	CYTH2	NP_059431.1	483	1	1.97
UTS2	XP_016855605.1	408	15	34.92	LMTK3	XP_011524714.1	524	2	3.63
TNFRSF9	NP_001552.2	782	11	13.36	SULT2B1	NP_814444.1	1222	2	1.55
PARK7	NP_001116849.1	2353	13	5.25	FAM83E	XP_024307329.1	128	5	37.11
ERRFI1	NP_061821.1	525	16	28.95	SPACA4	NP_598005.1	258	7	25.77
SLC45A1	NP_001366543.1	626	17	25.80	RPL18	NP_001257419.1	1139	2	1.67
RERE	NP_036234.3	791	17	20.42	SPHK2	NP_064511.2	676	0	0.00
ENO1	NP_001419.1	2245	11	4.65	DBP	NP_001343.2	234	3	12.18
CA6	XP_011540386.1	395	18	43.29	CA11	NP_001208.2	451	9	18.96
SLC2A7	XP_011539126.1	183	10	51.91	NTN5	NP_665806.1	218	5	21.79
SLC2A5	NP_001315548.1	653	17	24.73	FUT2	NP_001091107.1	560	4	6.79
GPR157	XP_005263554.1	175	19	103.14	MAMSTR	XP_016882133.1	72	0	0.00
H6PD	NP_001269516.1	1877	19	9.62	RASIP1	NP_060275.2	1083	3	2.63
SPSB1	NP_079382.2	609	17	26.52	IZUMO1	NP_872381.2	216	6	26.39
SLC25A33	NP_115691.1	512	17	31.54	FUT1	NP_001316806.1	421	4	9.03
TMEM201	XP_016856038.1	342	14	38.89	FGF21	NP_061986.1	956	3	2.98
PIK3CD	XP_016856965.1	1749	2	1.09	BCAT2	NP_001158245.1	960	5	4.95
CLSTN1	NP_055759.3	598	13	20.65	HSD17B14	NP_057330.2	371	9	23.04
CTNNBIP1	XP_016857342.1	268	14	49.62	PLEKHA4	XP_011525460.1	172	0	0.00
LZIC	XP_005263563.1	130	13	95.00	PPP1R15A	NP_055145.3	609	3	4.68
NMNAT1	XP_016857597.1	617	13	20.02	TULP2	XP_011525555.1	236	3	12.08
RBP7	NP_443192.1	230	10	41.30	NUCB1	XP_016882334.1	611	0	0.00
UBE4B	XP_005263479.1	1082	16	14.05	DHDH	NP_055290.1	463	4	8.21

KIF1B	NP_001352880.1	1534	13	8.05	BAX	NP_001278357.1	609	1	1.56
PGD	NP_002622.2	841	5	5.65	FTL	XP_024307215.1	757	2	2.51
CENPS-CORT	NP_940946.1	522	6	10.92	GYS1	NP_001155059.1	1178	4	3.23
CENPS	NP_001257446.1	522	6	10.92	RUVBL2	NP_006657.1	2083	8	3.65
CORT	NP_001293.3	858	5	5.54	LHB	NP_000885.1	569	3	5.01
DFFA	NP_004392.1	433	7	15.36	CGB3	NP_000728.1	265	2	7.17
PEX14	NP_004556.1	349	3	8.17	CGB2	NP_203696.2	79	1	12.02
CASZ1	XP_005263536.1	351	5	13.53	CGB1	NP_203695.2	94	1	10.11
C1orf127	NP_001164225.1	23	9	371.72	CGB5	NP_149032.1	70	1	13.57
TARDBP	XP_016856352.1	1165	8	6.52	CGB8	NP_149439.1	259	2	7.34
MASP2	NP_006601.2	377	9	22.68	CGB7	NP_149133.1	229	3	12.44
SRM	NP_003123.2	965	8	7.88	NTF4	XP_011525310.1	877	6	6.50
EXOSC10	NP_002676.1	1528	8	4.97	KCNA7	NP_114092.2	605	11	17.27
MTOR	XP_024301955.1	3751	3	0.76	SNRNP70	NP_003080.2	1555	1	0.61
ANGPTL7	NP_066969.1	426	9	20.07	LIN7B	NP_071448.1	436	2	4.36
UBIAD1	NP_001317278.1	307	8	24.75	C19orf73	NP_060581.2	0	0	#DIV/0!
DISP3	NP_065831.1	297	9	28.79	PPFIA3	NP_003651.1	758	5	6.27
FBXO2	NP_036300.2	745	5	6.38	HRC	NP_002143.1	511	1	1.86
FBXO44	XP_024306742.1	528	4	7.20	TRPM4	NP_060106.2	365	2	5.21
FBXO6	XP_016856496.1	579	4	6.56	SLC6A16	XP_011525162.1	182	1	5.22
MAD2L2	NP_006332.3	614	5	7.74	CD37	NP_001765.1	478	3	5.96
DRAXIN	XP_011539707.1	226	1	4.20	TEAD2	XP_005259391.1	680	8	11.18
AGTRAP	XP_011540102.1	277	5	17.15	DKKL1	XP_011525027.1	242	4	15.70
C1orf167	XP_011539578.1	40	0	0.00	KASH5	XP_005258606.1	149	3	19.13
MTHFR	XP_011539798.1	1502	6	3.79	PTH2	NP_848544.1	210	0	0.00
CLCN6	NP_001277.2	390	6	14.61	GFY	NP_001372116.1	90	1	10.56
NPPA	NP_006163.1	708	7	9.39	SLC17A7	NP_064705.1	1395	2	1.36
NPPB	NP_002512.1	549	3	5.19	PIH1D1	NP_060386.1	573	2	3.32
KIAA2013	NP_612355.1	280	8	27.14	ALDH16A1	NP_699160.2	557	3	5.12
PLOD1	NP_001303249.1	530	8	14.34	FLT3LG	XP_011524977.1	595	2	3.19
MFN2	XP_024307067.1	1409	5	3.37	RPL13A	NP_036555.1	1753	6	3.25
MIIP	NP_068752.2	83	6	68.67	RPS11	NP_001006.1	1476	5	3.22
TNFRSF8	NP_001234.3	643	1	1.48	FCGRT	NP_001129491.1	553	6	10.31
TNFRSF1B	XP_011540362.1	1325	6	4.30	RCN3	XP_024307388.1	246	7	27.03
VPS13D	NP_056193.2	696	5	6.82	NOSIP	XP_011525319.1	345	13	35.80
DHRS3	XP_006711099.1	528	2	3.60	PRRG2	NP_000942.1	159	12	71.69
AADACL4	NP_001013652.1	233	0	0.00	PRR12	NP_065770.1	276	12	41.30
AADACL3	NP_001096640.2	153	0	0.00	RRAS	NP_006261.1	2081	13	5.93
C1orf158	NP_689503.3	44	0	0.00	SCAF1	XP_011525496.1	505	12	22.57
PRAMEF12	NP_001074299.2	125	0	0.00	IRF3	NP_001184051.1	1251	15	11.39
PRAMEF1	NP_075389.2	94	1	10.11	BCL2L12	NP_001269448.1	379	11	27.57
PRAMEF11	NP_001139816.2	90	0	0.00	PRMT1	NP_001527.3	1898	11	5.51
HNRNPCL1	NP_001013653.1	1153	0	0.00	ADM5	NP_001094810.1	92	0	0.00
PRAMEF2	NP_075390.1	144	3	19.79	CPT1C	XP_024307116.1	539	8	14.10
PRAMEF4	NP_001009611.2	130	0	0.00	TSKS	NP_068379.1	144	13	85.76
PRAMEF10	NP_001034450.3	85	0	0.00	AP2A1	NP_055018.2	1033	5	4.60
PRAMEF7	NP_001012277.2	75	0	0.00	FUZ	NP_001165408.1	304	9	28.12
PRAMEF6	NP_001010889.1	98	0	0.00	MED25	NP_001365284.1	572	8	13.29
PRAMEF27	NP_001287820.1	12	0	0.00	PTOV1	NP_001292037.1	298	3	9.56
HNRNPCL3	NP_001369287.1	1028	2	1.85	PNKP	NP_009185.2	624	2	3.04
PRAMEF25	NP_001297063.1	97	0	0.00	AKT1S1	NP_115751.3	453	2	4.19
HNRNPCL2	NP_001130033.3	1046	1	0.91	TBC1D17	NP_078958.2	554	7	12.00
PRAMEF26	NP_001293001.1	91	0	0.00	IL4I1	NP_758962.1	938	2	2.03
HNRNPCL4	NP_001289480.1	1028	1	0.92	NUP62	NP_036478.2	730	5	6.51
PRAMEF9	NP_001010890.2	97	0	0.00	ATF5	NP_036200.2	433	0	0.00
PRAMEF13	NP_001278309.1	96	0	0.00	SIGLEC11	XP_005258533.2	202	0	0.00
PRAMEF18	NP_001093320.2	75	0	0.00	VRK3	XP_005259029.1	646	1	1.47
PRAMEF5	NP_001013425.2	99	0	0.00	ZNF473	NP_056243.1	328	3	8.69
PRAMEF8	XP_016856758.1	78	0	0.00	IZUMO2	XP_011524739.1	90	3	31.67
PRAMEF15	NP_001091846.1	107	0	0.00	MYH14	XP_011525622.1	1263	4	3.01
PRAMEF14	NP_001019832.2	99	0	0.00	KCNC3	NP_004968.2	966	6	5.90
PRAMEF19	NP_001093260.3	86	0	0.00	NAPSA	XP_016883001.1	505	0	0.00
PRAMEF17	NP_001093321.1	70	0	0.00	NR1H2	NP_009052.4	609	2	3.12
PRAMEF20	NP_001093322.2	97	0	0.00	POLD1	NP_001295561.1	1559	4	2.44
LRRC38	NP_001010847.1	489	1	1.94	SPIB	NP_003112.2	482	3	5.91
PDPN	NP_938203.2	666	1	1.43	MYBPC2	NP_004524.3	330	5	14.39

PRDM2	XP_016857753.1	536	2	3.54	FAM71E1	XP_011524707.2	31	1	30.64
KAZN	XP_005245852.1	303	1	3.14	EMC10	XP_011525120.1	177	4	21.47
TMEM51	XP_016857079.1	176	1	5.40	JOSD2	NP_001257570.1	205	0	0.00
FHAD1	XP_011538882.1	146	1	6.51	ASPDH	NP_001019827.2	172	2	11.05
EFHD2	NP_077305.2	384	1	2.47	LRRC4B	NP_001335497.1	1334	4	2.85
CTRC	XP_011538852.1	382	2	4.97	SYT3	NP_115674.1	828	7	8.03
CELA2A	NP_254275.1	310	2	6.13	C19orf81	NP_001182005.1	18	0	0.00
CELA2B	NP_056933.3	279	3	10.21	SHANK1	XP_011525316.1	2362	7	2.82
CASP9	NP_001264983.1	1681	0	0.00	CLEC11A	NP_002966.1	317	0	0.00
DNAJC16	NP_056106.1	532	4	7.14	GPR32	NP_001497.1	210	0	0.00
AGMAT	NP_079034.3	612	2	3.10	ACP4	NP_149059.1	264	9	32.38
DDI2	NP_115717.3	345	1	2.75	C19orf48	NP_116101.1	180	3	15.83
RSC1A1	NP_006502.1	390	2	4.87	KLK1	NP_002248.1	564	8	13.47
PLEKHM2	XP_016856246.1	143	0	0.00	KLK15	NP_001264010.1	357	7	18.63
SLC25A34	NP_997231.1	154	0	0.00	KLK3	NP_001639.1	1114	8	6.82
TMEM82	NP_001013663.1	124	1	7.66	KLK2	NP_005542.1	435	6	13.10
FBLIM1	XP_016857014.1	684	4	5.56	KLK4	NP_004908.4	721	11	14.49
UQCRHL	NP_001083060.1	415	0	0.00	KLK5	NP_001070959.1	448	12	25.45
SPEN	NP_055816.2	694	5	6.84	KLK6	NP_002765.1	772	7	8.61
ZBTB17	NP_001311067.1	464	9	18.43	KLK7	NP_001230055.1	511	9	16.73
SRARP	NP_849162.1	99	4	38.38	KLK8	NP_653089.1	490	8	15.51
HSPB7	NP_001336611.1	556	7	11.96	KLK9	NP_036447.1	388	8	19.59
CLCNKA	NP_004061.3	298	5	15.94	KLK10	XP_016882482.1	469	14	28.36
CLCNKB	NP_000076.2	307	4	12.38	KLK11	XP_005258496.1	468	5	10.15
FAM131C	NP_872429.2	223	1	4.26	KLK12	NP_062544.1	360	10	26.39
EPHA2	NP_004422.2	1846	7	3.60	KLK13	NP_001335107.1	378	9	22.62
ARHGEF19	NP_694945.2	453	3	6.29	KLK14	NP_001298111.2	275	10	34.54
CPLANE2	NP_112169.2	1323	4	2.87	CTU1	NP_660275.2	811	7	8.20
FBXO42	XP_006710761.1	583	5	8.15	SIGLEC9	XP_006723209.1	484	6	11.78
SZRD1	NP_001108072.1	370	0	0.00	SIGLEC7	NP_055200.1	524	5	9.06
SPATA21	NP_940948.1	920	4	4.13	CD33	XP_016882997.1	849	5	5.59
NECAP2	NP_001138749.1	373	0	0.00	SIGLECL1	NP_001372395.1	2	0	0.00
NBPF1	NP_060410.3	193	1	4.92	IGLON5	NP_001094842.1	429	1	2.21
CROCC	XP_011540770.2	458	1	2.07	VSIG10L	NP_001157394.1	57	0	0.00
MFAP2	NP_001128719.1	324	2	5.86	ETFB	NP_001976.1	645	3	4.42
ATP13A2	XP_006710575.1	903	3	3.16	CLDND2	XP_011524727.1	86	1	11.05
SDHB	NP_002991.2	1813	1	0.52	NKG7	XP_006723291.1	601	7	11.06
PADI2	NP_031391.2	334	1	2.84	LIM2	NP_001155220.1	263	2	7.22
PADI1	NP_037490.2	237	1	4.01	C19orf84	NP_001180552.1	0	0	#DIV/0!
PADI3	NP_057317.2	229	0	0.00	SIGLEC10	NP_001164632.1	285	8	26.67
PADI4	NP_036519.2	462	1	2.06	SIGLEC8	NP_001350477.1	194	3	14.69
PADI6	NP_997304.3	205	2	9.27	CEACAM18	NP_001265321.1	104	5	45.67
RCC2	NP_001129676.1	949	2	2.00	SIGLEC12	NP_443729.1	235	5	20.21
ARHGEF10L	XP_024303827.1	269	3	10.59	SIGLEC6	NP_001171018.1	210	2	9.05
ACTL8	NP_110439.2	1018	0	0.00	ZNF175	NP_009078.1	260	1	3.65
IGSF21	NP_116269.3	708	7	9.39	SIGLEC5	NP_001371638.1	248	5	19.15
KLHDC7A	NP_689588.2	133	1	7.14	SIGLEC14	XP_016881602.1	314	5	15.13
PAX7	NP_001128726.1	1200	1	0.79	HAS1	NP_001284365.1	763	0	0.00
TAS1R2	NP_689418.2	494	5	9.61	FPR1	NP_001180235.1	863	5	5.50
ALDH4A1	NP_001306147.1	712	2	2.67	FPR2	NP_001453.1	955	2	1.99
IFFO2	XP_011538932.1	107	3	26.63	FPR3	NP_002021.3	611	1	1.55
UBR4	XP_016856319.2	1126	1	0.84	ZNF577	NP_001357386.1	228	1	4.17
EMC1	NP_001258358.1	390	6	14.61	ZNF649	NP_075562.2	207	0	0.00
MRTO4	NP_057267.2	1476	4	2.57	ZNF613	NP_001026891.2	167	0	0.00
AKR7A3	XP_011539348.1	321	6	17.76	ZNF350	XP_016882587.1	296	0	0.00
AKR7A2	NP_001307908.1	464	5	10.24	ZNF615	NP_001308246.1	176	0	0.00
SLC66A1	XP_005245973.1	251	0	0.00	ZNF614	NP_079316.2	195	0	0.00
CAPZB	NP_004921.1	1258	3	2.27	ZNF432	NP_001309213.1	245	0	0.00
MICOS10	NP_001027535.1	457	0	0.00	ZNF841	NP_001356758.1	168	0	0.00
MICOS10-NBL	NP_001191017.1	244	1	3.89	ZNF616	NP_848618.2	242	0	0.00
NBL1	NP_877421.2	142	0	0.00	ZNF836	NP_001096127.1	157	0	0.00
HTR6	NP_000862.1	364	0	0.00	PPP2R1A	NP_055040.2	1669	1	0.57
TMCO4	XP_016856405.1	213	0	0.00	ZNF766	XP_016882930.1	165	1	5.76
RNF186	NP_061935.1	326	4	11.66	ZNF480	NP_653285.2	194	0	0.00
OTUD3	XP_005245849.1	206	7	32.28	ZNF610	NP_001154897.1	137	0	0.00
PLA2G2E	NP_055404.1	178	3	16.01	ZNF880	XP_016882301.1	157	0	0.00

PLA2G2A	NP_001155199.1	723	2	2.63	ZNF528	XP_006723481.1	191	0	0.00
PLA2G5	XP_005245948.1	308	1	3.08	ZNF534	NP_001338608.1	162	0	0.00
PLA2G2D	NP_036532.1	265	1	3.58	ZNF578	NP_001353111.1	150	0	0.00
PLA2G2F	XP_011540257.1	189	3	15.08	ZNF808	NP_001308353.1	162	0	0.00
PLA2G2C	NP_001303651.1	167	0	0.00	ZNF701	NP_001166126.1	140	0	0.00
UBXN10	NP_689589.1	133	0	0.00	ZNF83	NP_001334944.1	246	0	0.00
VWA5B1	NP_001034589.2	62	0	0.00	ZNF611	NP_112234.3	170	0	0.00
CAMK2N1	NP_061054.2	399	4	9.52	ZNF600	NP_001308796.1	178	0	0.00
MUL1	NP_078820.2	391	8	19.44	ZNF28	NP_001356692.1	182	0	0.00
FAM43B	NP_997217.1	437	4	8.70	ZNF468	NP_001008801.1	124	0	0.00
CDA	NP_001776.1	915	0	0.00	ZNF320	XP_024307164.1	147	0	0.00
PINK1	NP_115785.1	1099	5	4.32	ZNF816-ZNF3	NP_001189402.1	164	0	0.00
DDOST	NP_005207.3	835	4	4.55	ZNF816	NP_001189385.1	184	0	0.00
KIF17	NP_001116291.1	1145	0	0.00	ERVV-1	NP_689686.2	115	0	0.00
SH2D5	XP_011539764.1	207	0	0.00	ERVV-2	NP_001177984.1	75	0	0.00
HP1BP3	NP_001358981.1	589	2	3.23	ZNF160	NP_001309064.1	231	0	0.00
EIF4G3	XP_016858191.1	1047	2	1.81	ZNF415	XP_016882458.1	180	0	0.00
ECE1	NP_001106819.1	551	2	3.45	ZNF347	NP_115973.2	251	0	0.00
NBPF3	NP_001243345.1	48	1	19.79	ZNF665	XP_011525627.1	266	0	0.00
ALPL	NP_000469.3	1229	3	2.32	ZNF677	NP_001372537.1	213	2	8.92
RAP1GAP	XP_016857474.1	654	1	1.45	VN1R2	NP_776255.2	150	1	6.33
USP48	NP_001337093.1	589	1	1.61	VN1R4	XP_016882217.1	180	2	10.56
LDLRAD2	NP_001013715.2	57	1	16.67	ZNF845	NP_001308452.1	174	1	5.46
HSPG2	NP_005520.4	1331	4	2.85	ZNF525	NP_001335085.1	136	0	0.00
CELA3B	NP_031378.1	309	0	0.00	ZNF765	NP_001035275.1	168	0	0.00
CELA3A	NP_005738.4	260	0	0.00	ZNF761	NP_001337425.1	206	0	0.00
CDC42	NP_001782.1	3734	5	1.27	ZNF813	NP_001004301.2	157	0	0.00
WNT4	NP_110388.2	1249	5	3.80	ZNF331	XP_016882425.1	395	2	4.81
ZBTB40	NP_055685.3	337	1	2.82	DPRX	NP_001012746.1	411	2	4.62
EPHA8	NP_065387.1	1112	3	2.56	NLRP12	XP_016882951.1	220	3	12.95
C1QA	NP_001334394.1	833	3	3.42	MYADM	NP_612382.1	333	0	0.00
C1QC	NP_001334548.1	711	1	1.34	PRKCG	NP_001303258.1	1381	7	4.82
C1QB	NP_000482.3	933	1	1.02	CACNG7	NP_001371730.1	969	3	2.94
EPHB2	NP_001296122.1	1849	4	2.06	CACNG8	NP_114101.4	405	3	7.04
LACTBL1	NP_001276903.1	89	0	0.00	CACNG6	NP_665813.1	150	2	12.67
TEX46	NP_001229450.1	0	0	#DIV/0!	VSTM1	NP_001275722.1	72	6	79.16
KDM1A	NP_001009999.1	1810	3	1.57	TARM1	NP_001129158.2	174	4	21.84
LUZP1	XP_016857741.1	254	2	7.48	OSCAR	NP_573398.2	348	8	21.84
HTR1D	NP_000855.1	509	1	1.87	NDUFA3	XP_016882322.1	470	10	20.21
HNRNPR	XP_016855497.1	1124	1	0.85	TFPT	NP_037474.1	226	12	50.44
ZNF436	NP_001070663.1	669	3	4.26	PRPF31	NP_056444.3	1395	11	7.49
TCEA3	XP_016857693.1	711	3	4.01	CNOT3	XP_005278336.1	851	7	7.81
ASAP3	XP_016857176.1	514	3	5.54	LENG1	NP_077292.2	127	11	82.28
E2F2	XP_011539172.1	1127	4	3.37	TMC4	NP_653287.2	99	1	9.60
ID3	NP_002158.3	691	3	4.12	MBOAT7	NP_077274.3	301	13	41.03
RPL11	NP_000966.2	1906	7	3.49	TSEN34	NP_001269262.1	290	8	26.21
ELOA	NP_003189.2	486	4	7.82	RPS9	NP_001308631.1	1545	9	5.53
PITHD1	NP_065095.2	152	2	12.50	LILRB3	XP_016881712.1	521	8	14.59
LYPLA2	XP_024308564.1	564	1	1.68	LILRA6	NP_077294.3	221	3	12.90
GALE	NP_001121093.1	804	0	0.00	LILRB5	NP_001074912.2	131	2	14.50
HMGCL	NP_001159531.1	775	0	0.00	LILRB2	NP_001074447.2	742	2	2.56
FUCA1	NP_000138.2	544	3	5.24	LILRA5	NP_871714.1	214	5	22.20
CNR2	XP_016855750.1	778	2	2.44	LILRA4	NP_036408.4	366	6	15.57
PNRC2	XP_016857180.1	424	3	6.72	LAIR1	NP_001275956.2	514	12	22.18
SRSF10	NP_001177938.1	1475	3	1.93	TTYH1	NP_001005367.1	624	1	1.52
MYOM3	NP_689585.3	227	0	0.00	LENG8	NP_443157.1	479	9	17.85
IL22RA1	NP_067081.2	388	1	2.45	LENG9	NP_001288711.1	60	0	0.00
IFNLR1	NP_775087.1	300	1	3.17	CDC42EP5	NP_659494.2	169	0	0.00
GRHL3	NP_937817.3	403	0	0.00	LAIR2	NP_002279.2	175	9	48.85
STPG1	XP_011540705.1	94	0	0.00	LILRA2	NP_001124389.2	279	3	10.21
NIPAL3	XP_011540110.1	180	5	26.39	LILRA1	NP_006854.1	529	8	14.37
RCAN3	NP_001238908.1	398	4	9.55	LILRB1	XP_016881676.1	496	9	17.24
NCMAP	XP_005245929.1	108	0	0.00	LILRB4	XP_016881704.1	400	2	4.75
SRRM1	NP_001353524.1	1276	5	3.72	KIR3DL3	NP_703144.3	155	2	12.26
CLIC4	NP_039234.1	604	4	6.29	KIR2DL3	NP_056952.2	375	6	15.20
RUNX3	XP_011540653.1	1259	4	3.02	KIR2DL1	NP_055033.2	319	7	20.85

SYF2	NP_997053.1	647	7	10.28	KIR2DL4	NP_001074239.1	250	9	34.20
RSRP1	NP_001308701.1	136	5	34.92	KIR3DL1	NP_037421.2	318	8	23.90
RHD	NP_001269800.1	265	4	14.34	KIR3DL2	NP_006728.2	264	7	25.19
TMEM50A	NP_055128.1	146	5	32.53	FCAR	NP_001991.1	418	10	22.73
RHCE	XP_016857503.1	260	4	14.61	NCR1	XP_011525832.1	541	10	17.56
MACO1	NP_060672.2	246	6	23.17	NLRP7	XP_011524903.1	258	5	18.41
LDLRAP1	XP_006710624.1	452	1	2.10	NLRP2	NP_001167552.1	327	5	14.53
MAN1C1	NP_001275939.1	271	1	3.51	GP6	NP_001077368.2	384	0	0.00
SELENON	NP_065184.2	239	0	0.00	RDH13	XP_011524710.1	502	6	11.35
MTFR1L	NP_062457.3	59	0	0.00	EPS8L1	XP_005259077.1	319	2	5.96
AUNIP	NP_001274419.1	236	1	4.03	PPP1R12C	NP_001258547.1	1222	6	4.66
PAQR7	NP_848509.1	241	2	7.88	TNNT1	NP_001119605.1	1085	6	5.25
STMN1	NP_001138926.1	1248	3	2.28	TNNI3	NP_000354.4	803	3	3.55
PAFAH2	XP_011539830.1	285	1	3.33	DNAAF3	NP_849159.2	148	2	12.84
EXTL1	NP_004446.2	523	3	5.45	SYT5	NP_001284703.1	546	6	10.44
SLC30A2	NP_115902.1	328	3	8.69	PTPRH	NP_001154912.2	602	5	7.89
TRIM63	NP_115977.2	1061	4	3.58	TMEM86B	NP_776165.3	153	0	0.00
PDIK1L	XP_006710444.1	505	4	7.52	PPP6R1	NP_055746.3	542	3	5.26
FAM110D	NP_079145.2	38	0	0.00	HSPBP1	XP_016882032.1	1272	4	2.99
ZNF593	NP_056955.2	709	2	2.68	BRSK1	NP_115806.1	1460	6	3.90
CNKSR1	NP_001284576.1	306	1	3.10	TMEM150B	XP_011525152.1	101	1	9.41
CATSPER4	NP_937770.1	325	1	2.92	KMT5C	NP_116090.2	347	2	5.48
CEP85	XP_024304966.1	145	1	6.55	COX6B2	NP_001356728.1	433	0	0.00
SH3BGRL3	NP_112576.1	239	0	0.00	FAM71E2	NP_001138874.1	51	0	0.00
UBXN11	NP_663320.2	315	0	0.00	IL11	NP_000632.1	706	1	1.35
CD52	NP_001794.2	723	1	1.31	TMEM190	NP_631911.1	40	1	23.75
CRYBG2	XP_011539975.1	179	2	10.61	TMEM238	NP_001177693.1	13	0	0.00
ZNF683	XP_005245885.1	133	0	0.00	RPL28	NP_001350626.1	900	2	2.11
LIN28A	NP_078950.1	1146	0	0.00	UBE2S	NP_055316.2	2243	1	0.42
DHDDS	NP_079163.2	731	0	0.00	SHISA7	NP_001138648.1	741	1	1.28
HMGN2	NP_005508.1	231	0	0.00	ISOC2	NP_001129674.1	483	7	13.77
RPS6KA1	NP_002944.2	947	0	0.00	ZNF628	XP_024307520.1	378	1	2.51
ARID1A	NP_006006.3	1072	2	1.77	NAT14	NP_065111.1	161	3	17.70
PIGV	NP_001361407.1	332	0	0.00	SSC5D	NP_001138422.1	97	0	0.00
ZDHHC18	NP_115659.1	215	1	4.42	SBK2	NP_001357025.1	102	0	0.00
SFN	NP_006133.1	2242	4	1.69	SBK3	NP_001186753.1	49	1	19.39
GPN2	NP_060536.3	1170	1	0.81	ZNF579	XP_016881899.1	193	2	9.84
GPATCH3	NP_071361.2	138	0	0.00	FIZ1	XP_011525728.1	300	4	12.67
NUDC	XP_016855583.1	1037	0	0.00	ZNF524	XP_011524789.1	304	3	9.37
NR0B2	NP_068804.1	957	1	0.99	ZNF865	NP_001182534.1	493	3	5.78
KDF1	NP_689578.2	113	1	8.41	ZNF784	NP_976308.1	180	1	5.28
TRNP1	NP_001013664.2	176	0	0.00	ZNF580	NP_001156895.1	204	1	4.66
TENT5B	NP_443175.2	179	0	0.00	ZNF581	XP_016882356.1	131	1	7.25
SLC9A1	NP_003038.2	751	1	1.26	CCDC106	NP_001357399.1	146	3	19.52
WDTC1	NP_001263181.1	3649	6	1.56	U2AF2	NP_009210.1	1588	3	1.79
TMEM222	NP_115501.2	106	0	0.00	EPN1	NP_001123543.1	774	6	7.36
SYTL1	XP_024306203.1	360	2	5.28	NLRP9	NP_789790.2	189	5	25.13
MAP3K6	NP_001284538.1	484	1	1.96	RFPL4A	XP_011525217.1	237	5	20.04
FCN3	NP_775628.1	341	1	2.79	RFPL4AL1	NP_001264326.1	167	1	5.69
CD164L2	XP_011539743.1	62	1	15.32	NLRP11	NP_001372380.1	505	9	16.93
GPR3	NP_005272.1	407	2	4.67	NLRP4	NP_604393.2	219	5	21.69
WASF2	NP_008921.1	683	3	4.17	NLRP13	NP_001307986.1	136	5	34.92
AHDC1	XP_024302229.1	110	4	34.54	NLRP8	NP_789781.2	118	9	72.45
FGR	XP_011539313.1	1559	6	3.66	NLRP5	NP_703148.4	228	10	41.66
IFI6	NP_002029.3	322	1	2.95	ZNF787	NP_001002836.2	397	6	14.36
FAM76A	NP_001137384.1	192	1	4.95	ZNF444	XP_024307341.1	293	8	25.94
STX12	NP_803173.1	888	5	5.35	GALP	NP_149097.1	255	4	14.90
PPP1R8	NP_054829.2	578	6	9.86	ZSCAN5B	NP_001372567.1	248	3	11.49
THEMIS2	NP_001099026.1	458	3	6.22	ZSCAN5C	NP_001345342.1	183	0	0.00
RPA2	XP_024304630.1	975	3	2.92	ZSCAN5A	NP_001309007.1	273	3	10.44
SMPDL3B	XP_011539561.1	341	0	0.00	EDDM13	NP_001341587.1	0	0	#DIV/0!
XKR8	NP_060523.2	165	0	0.00	ZNF582	NP_001307300.1	202	0	0.00
EYA3	NP_001269490.1	524	6	10.88	ZNF583	XP_016881835.1	175	0	0.00
PTAFR	NP_001158193.1	752	3	3.79	ZNF667	NP_001308285.1	173	0	0.00
DNAJC8	NP_055095.2	624	4	6.09	ZNF471	NP_065864.2	246	2	7.72
ATP5IF1	NP_057395.1	361	3	7.89	ZFP28	XP_011524765.2	254	2	7.48

SESN2	NP_113647.1	512	0	0.00	ZNF470	NP_001001668.3	180	0	0.00
MED18	NP_001120822.1	310	5	15.32	ZNF71	NP_001357144.1	258	4	14.73
PHACTR4	NP_001337087.1	377	4	10.08	SMIM17	NP_001180557.1	70	0	0.00
RCC1	NP_001041659.1	1722	4	2.21	ZNF835	NP_001005850.2	133	5	35.71
TRNAU1AP	NP_060316.1	1231	2	1.54	ZIM2	NP_001356701.1	362	9	23.62
RAB42	NP_001372117.1	1102	1	0.86	PEG3	NP_001139656.1	729	8	10.42
TAF12	XP_016857675.1	682	1	1.39	USP29	NP_065954.1	516	7	12.89
GMEB1	XP_016855576.1	279	1	3.40	ZIM3	NP_443114.1	176	5	26.99
YTHDF2	NP_057342.2	771	4	4.93	DUXA	NP_001012747.1	486	9	17.59
OPRD1	NP_000902.3	981	4	3.87	ZNF264	NP_003408.1	277	5	17.15
EPB41	XP_016856073.1	751	3	3.79	AURKC	NP_001015879.1	1081	2	1.76
TMEM200B	XP_011539759.1	70	4	54.28	ZNF805	NP_001018857.2	150	2	12.67
SRSF4	XP_011540253.1	960	6	5.94	ZNF460	NP_006626.3	181	1	5.25
MECR	NP_057095.4	761	4	4.99	ZNF543	NP_998763.2	163	1	5.83
PTPRU	XP_016855481.1	887	9	9.64	ZNF304	NP_001277247.1	182	0	0.00
MATN1	NP_002370.1	421	2	4.51	TRAPPC2B	NP_001342133.1	389	0	0.00
LAPTM5	XP_011540400.1	704	5	6.75	ZNF547	NP_775902.2	120	0	0.00
SDC3	XP_011540765.1	526	7	12.64	ZNF548	NP_001166244.1	132	0	0.00
PUM1	NP_055491.1	1422	10	6.68	ZNF17	NP_008890.2	162	0	0.00
NKAIN1	XP_016857809.1	331	8	22.96	ZNF749	NP_001018855.2	128	0	0.00
SNRNP40	NP_004805.2	1021	6	5.58	VN1R1	NP_065684.1	198	2	9.60
ZCCHC17	NP_001269495.1	833	7	7.98	ZNF772	NP_001137540.1	186	1	5.11
FABP3	XP_011539309.1	652	7	10.20	ZNF419	NP_001278674.1	201	0	0.00
SERINC2	NP_001185967.1	282	8	26.95	ZNF773	NP_001291264.1	190	1	5.00
TINAGL1	XP_011540248.1	353	1	2.69	ZNF549	NP_001186224.2	195	0	0.00
HCRTR1	XP_016856596.1	421	0	0.00	ZNF550	NP_001264022.1	145	0	0.00
PEF1	NP_036524.1	354	0	0.00	ZNF416	NP_060349.1	174	0	0.00
COL16A1	XP_016855828.1	531	3	5.37	ZIK1	NP_001010879.2	231	0	0.00
ADGRB2	XP_016857401.1	270	0	0.00	ZNF530	XP_006723257.1	155	0	0.00
SPOCD1	XP_016858271.1	246	0	0.00	ZNF134	NP_003426.3	179	1	5.31
PTP4A2	NP_001356787.1	405	2	4.69	ZNF211	NP_001252526.1	201	0	0.00
KHDRBS1	NP_006550.1	1289	2	1.47	ZSCAN4	XP_016881947.1	394	1	2.41
TMEM39B	NP_060526.2	169	0	0.00	ZNF551	NP_612356.2	186	0	0.00
KPNA6	NP_036448.1	962	2	1.97	ZNF154	NP_001078853.1	325	0	0.00
TXLNA	XP_016856050.1	211	0	0.00	ZNF671	NP_079109.2	204	0	0.00
CCDC28B	XP_016857796.1	178	2	10.67	ZNF776	NP_001334936.1	146	0	0.00
IQCC	NP_001153514.1	19	0	0.00	ZNF586	NP_060122.2	114	0	0.00
DCDC2B	NP_001092904.1	101	0	0.00	ZNF552	NP_079038.2	167	0	0.00
TMEM234	XP_016857303.1	112	1	8.48	ZNF587B	NP_001191747.1	164	0	0.00
EIF3I	NP_003748.1	1189	5	3.99	ZNF587	NP_116217.1	275	0	0.00
FAM167B	NP_116037.2	45	0	0.00	ZNF814	NP_001138461.1	211	1	4.50
LCK	XP_024302814.1	2252	4	1.69	ZNF417	NP_001284663.1	241	1	3.94
HDAC1	NP_004955.2	4050	7	1.64	ZNF418	XP_016881797.1	169	0	0.00
MARCKSL1	NP_075385.1	381	2	4.99	ZNF256	NP_005764.2	220	1	4.32
FAM229A	NP_001161148.1	14	1	67.85	C19orf18	NP_689687.1	25	0	0.00
TSSK3	NP_443073.1	1141	6	5.00	ZNF606	NP_001334951.1	242	0	0.00
BSDC1	NP_001137362.1	132	0	0.00	ZSCAN1	XP_006723212.1	461	1	2.06
ZBTB8B	NP_001139192.1	267	4	14.23	ZNF135	NP_009065.1	243	1	3.91
ZBTB8A	NP_001035531.2	207	5	22.95	ZSCAN18	XP_011525539.1	324	5	14.66
ZBTB8OS	NP_001353199.1	314	1	3.03	ZNF329	XP_011525612.1	181	1	5.25
RBBP4	NP_005601.1	2118	3	1.35	ZNF274	NP_598009.1	515	1	1.84
SYNC	NP_001155180.2	167	1	5.69	ZNF544	XP_016882089.1	241	1	3.94
KIAA1522	NP_065939.2	165	1	5.76	ZNF8	NP_066575.2	266	0	0.00
YARS1	NP_003671.1	2246	3	1.27	ZSCAN22	NP_001308045.1	209	1	4.55
S100PBP	XP_024304943.1	136	2	13.97	A1BG	NP_570602.2	409	1	2.32
FNDC5	NP_001165411.2	464	2	4.09	ZNF497	NP_940860.2	168	1	5.65
HPCA	XP_016856607.1	1862	2	1.02	ZNF837	XP_016881731.1	228	2	8.33
TMEM54	NP_001316654.1	143	3	19.93	RPS5	NP_001000.2	1689	2	1.12
RNF19B	NP_001287755.1	591	1	1.61	RNF225	NP_001182064.1	191	0	0.00
AK2	NP_001306071.1	1100	2	1.73	ZNF584	NP_775819.1	136	3	20.95
AZIN2	XP_016855656.1	543	1	1.75	ZNF132	NP_003424.3	257	2	7.78
TRIM62	XP_016857118.1	422	3	6.75	ZNF324B	NP_997278.2	237	1	4.45
ZNF362	NP_001357141.1	393	4	9.67	ZNF324	XP_005258770.1	216	2	10.35
A3GALT2	NP_001073907.1	206	4	18.45	ZNF446	NP_001291382.1	251	5	23.65
PHC2	NP_001372052.1	494	3	5.77	SLC27A5	NP_001308125.1	530	2	4.78
ZSCAN20	XP_016857726.1	273	4	13.92	ZBTB45	NP_116181.1	229	0	0.00

CSMD2	XP_016855677.1	440	2	4.32	TRIM28	XP_024307077.1	1575	6	5.57
HMGB4	NP_001366230.1	436	1	2.18	CHMP2A	NP_940818.1	784	1	2.02
C1orf94	NP_001128206.1	18	0	0.00	UBE2M	NP_003960.1	830	2	4.16
GJB5	NP_005259.1	216	3	13.19	MZF1	XP_011525566.1	617	0	0.00
GJB4	NP_694944.1	231	3	12.34	FAM110C	NP_001071178.2	110	9	155.45
GJB3	NP_076872.1	405	3	7.04	SH3YL1	NP_001153069.1	582	10	29.68
GJA4	XP_016856532.1	371	3	7.68	ACP1	NP_004291.1	588	5	13.46
SMIM12	XP_011538856.2	94	5	50.53	ALKAL2	XP_011508644.1	168	6	52.20
DLGAP3	NP_001073887.1	940	3	3.03	TMEM18	NP_690047.2	216	8	50.26
TMEM35B	NP_001182085.1	12	0	0.00	SNTG2	NP_061841.2	626	11	22.26
ZMYM6	NP_009098.3	181	2	10.50	TPO	XP_011508682.2	483	2	4.92
ZMYM1	XP_024305591.1	192	2	9.90	PXDN	XP_005264764.1	547	9	18.39
SFPQ	XP_005271172.1	1123	6	5.08	MYT1L	XP_016859107.1	941	9	10.10
ZMYM4	NP_001362582.1	361	3	7.89	EIPR1	NP_003301.1	324	13	40.12
KIAA0319L	NP_079150.3	232	0	0.00	TRAPPC12	NP_001308031.1	190	9	45.00
NCDN	NP_055099.1	475	4	8.00	ADI1	NP_060739.2	432	8	17.59
TFAP2E	NP_848643.2	302	2	6.29	RNASEH1	NP_001365201.1	664	8	11.45
PSMB2	NP_001186708.1	1493	3	1.91	RPS7	NP_001002.1	1276	2	1.49
C1orf216	NP_001335620.1	20	0	0.00	COLEC11	NP_001242914.1	273	9	31.32
CLSPN	XP_011540242.1	712	1	1.33	ALLC	XP_016859984.1	492	7	13.52
AGO4	NP_060099.2	1019	1	0.93	DCDC2C	NP_001274373.1	110	8	69.09
AGO1	NP_001304051.1	1386	1	0.69	SOX11	NP_003099.1	819	5	5.80
AGO3	XP_016856012.1	909	1	1.05	CMPK2	NP_001243407.1	769	6	7.41
TEKT2	XP_016856544.1	369	0	0.00	RSAD2	XP_011508717.1	570	3	5.00
ADPRS	NP_060295.1	192	0	0.00	RNF144A	XP_016860892.1	306	4	12.42
COL8A2	XP_005270534.1	431	1	2.20	ID2	NP_002157.2	1010	1	0.94
TRAPPC3	NP_001257826.1	521	2	3.65	KIDINS220	NP_001335670.1	1288	4	2.95
MAP7D1	NP_060537.3	321	1	2.96	MBOAT2	NP_620154.2	550	3	5.18
THRAP3	NP_001308400.1	612	0	0.00	ASAP2	XP_011508705.1	735	4	5.17
SH3D21	XP_016857829.1	315	2	6.03	ITGB1BP1	NP_001305998.1	254	1	3.74
EVA1B	NP_001291691.1	128	1	7.42	CPSF3	NP_057291.1	1382	4	2.75
STK40	NP_114406.1	1218	0	0.00	IAH1	NP_001034702.1	242	3	11.78
LSM10	NP_116270.1	805	2	2.36	ADAM17	NP_003174.3	1206	4	3.15
OSCP1	NP_659484.4	177	1	5.37	YWHAQ	NP_006817.1	2620	4	1.45
MRPS15	NP_112570.2	1174	0	0.00	TAF1B	NP_005671.3	322	6	17.70
CSF3R	NP_758519.1	871	1	1.09	GRHL1	XP_006711945.1	361	5	13.16
GRIK3	NP_000822.2	991	0	0.00	KLF11	NP_003588.1	668	5	7.11
ZC3H12A	NP_001310479.1	555	0	0.00	CYS1	NP_001032237.1	211	1	4.50
MEAF6	NP_001257805.1	375	1	2.53	RRM2	NP_001159403.1	1437	8	5.29
SNIP1	NP_078976.2	526	4	7.22	HPCAL1	XP_016859439.1	1129	12	10.10
DNALI1	NP_003453.3	310	3	9.19	ODC1	NP_002530.1	1163	2	1.63
GNL2	NP_037417.1	898	5	5.29	NOL10	NP_001248321.1	706	9	12.11
RSPO1	NP_001229838.1	774	0	0.00	ATP6V1C2	XP_011508641.1	500	6	11.40
C1orf109	NP_001337685.1	154	5	30.84	PDIA6	NP_001269634.1	1135	8	6.70
CDCA8	NP_001243804.1	760	2	2.50	KCNF1	NP_002227.2	1251	8	6.07
EPHA10	XP_016856569.1	1028	3	2.77	C2orf50	NP_001358250.1	3	0	0.00
MANEAL	NP_001106954.1	265	2	7.17	SLC66A3	NP_689604.1	124	6	45.97
YRDC	NP_078916.3	540	4	7.04	ROCK2	NP_004841.2	1615	3	1.76
C1orf122	NP_940848.2	75	0	0.00	E2F6	NP_937987.2	938	10	10.13
MTF1	NP_005946.2	297	0	0.00	GREB1	XP_011508720.1	425	2	4.47
INPP5B	NP_001352751.1	867	1	1.10	NTSR2	NP_036476.2	1222	2	1.55
SF3A3	NP_001307759.1	1552	2	1.22	LPIN1	NP_001248357.1	926	2	2.05
FHL3	XP_024309867.1	597	2	3.18	TRIB2	NP_067675.1	1297	6	4.39
UTP11	NP_057121.2	894	1	1.06	LRATD1	NP_001356293.1	131	7	50.76
POU3F1	NP_002690.3	643	1	1.48	NBAS	XP_016859806.1	316	7	21.04
RRAGC	NP_001258780.1	532	5	8.93	DDX1	NP_004930.1	1747	9	4.89
MYCBP	NP_036465.2	234	2	8.12	MYCN	NP_001280160.1	1541	6	3.70
GJA9	NP_110399.2	173	3	16.47	CYRIA	NP_110424.1	227	6	25.11
RHBDL2	NP_001291675.1	416	9	20.55	RAD51AP2	XP_011531386.1	36	1	26.39
AKIRIN1	NP_078871.1	246	2	7.72	VSNL1	NP_001353735.1	1614	9	5.30
NDUFS5	NP_001171908.1	806	6	7.07	SMC6	XP_016860405.1	1112	9	7.69
MACF1	NP_036222.3	1001	5	4.75	GEN1	NP_001123481.3	990	3	2.88
KIAA0754	NP_055853.1	1001	5	4.75	MSGN1	NP_001099039.1	157	4	24.20
BMP8A	NP_861525.2	368	3	7.74	KCNS3	NP_002243.3	829	6	6.88
PABPC4	NP_003810.1	1100	9	7.77	RDH14	NP_065956.1	191	1	4.97
HEYL	NP_055386.2	382	1	2.49	NT5C1B-RDH1	NP_001186032.1	483	1	1.97

NT5C1A	NP_115915.1	387	8	19.64	NT5C1B	NP_150278.2	271	3	10.52
HPCAL4	NP_001269326.1	1707	12	6.68	OSR1	NP_660303.1	267	0	0.00
PPIE	NP_982281.1	1149	14	11.57	TTC32	NP_001008238.1	682	3	4.18
BMP8B	NP_001711.2	525	9	16.28	WDR35	NP_065830.2	375	6	15.20
OXCT2	NP_071403.1	205	9	41.71	MATN3	NP_002372.1	581	8	13.08
TRIT1	NP_001299621.1	812	12	14.04	LAPTM4A	NP_055528.1	246	6	23.17
MYCL	NP_001028254.2	561	8	13.55	SDC1	NP_002988.4	1268	2	1.50
MFSD2A	NP_001129965.1	374	8	20.32	PUM2	NP_001339854.1	1093	6	5.21
CAP1	XP_016855558.1	1118	1	0.85	RHOB	NP_004031.1	2265	7	2.94
PPT1	NP_001136076.1	745	15	19.13	HS1BP3	XP_016860187.1	110	7	60.45
RLF	NP_036553.2	255	3	11.18	GDF7	NP_878248.2	436	2	4.36
TMCO2	NP_001008740.1	63	15	226.18	LDAH	NP_001269649.1	371	6	15.36
ZMPSTE24	NP_005848.2	970	8	7.83	APOB	NP_000375.3	1989	6	2.87
COL9A2	NP_001843.1	512	9	16.70	TDRD15	XP_011531514.1	643	2	2.95
SMAP2	NP_073570.1	589	8	12.90	KLHL29	XP_016858753.1	704	3	4.05
ZFP69B	XP_016857636.1	199	5	23.87	ATAD2B	XP_006712093.1	2659	5	1.79
ZFP69	XP_005270866.1	236	5	20.13	UBXN2A	NP_859064.2	242	1	3.93
EXO5	XP_016857588.1	145	3	19.65	MFSD2B	XP_016859552.1	190	1	5.00
ZNF684	NP_689586.3	147	5	32.31	WDCP	NP_079479.1	104	0	0.00
RIMS3	XP_011540781.1	786	3	3.63	FKBP1B	XP_011531001.1	991	5	4.79
NFYC	XP_024303124.1	972	5	4.89	SF3B6	NP_057131.1	695	1	1.37
KCNQ4	NP_004691.2	812	3	3.51	TP53I3	NP_004872.2	415	2	4.58
CITED4	NP_597724.1	260	1	3.65	PFN4	NP_955378.1	587	5	8.09
CTPS1	XP_024309320.1	1586	0	0.00	FAM228B	NP_001139182.1	70	0	0.00
SLFNL1	XP_024309637.1	41	1	23.17	FAM228A	NP_001035800.1	30	2	63.33
SCMH1	XP_011539341.1	436	1	2.18	ITSN2	NP_001335111.1	1480	7	4.49
FOXO6	NP_001278210.2	451	0	0.00	NCOA1	NP_003734.3	912	9	9.37
EDN2	XP_016856001.1	583	0	0.00	PTRHD1	NP_001013685.1	165	6	34.54
HIVEP3	XP_016857483.1	604	1	1.57	CENPO	NP_001186732.1	377	5	12.60
GUCA2B	NP_009033.1	281	0	0.00	ADCY3	NP_001364058.1	1481	6	3.85
GUCA2A	NP_291031.2	349	0	0.00	DNAJC27	NP_057628.1	1322	11	7.90
FOXJ3	NP_001185781.1	530	1	1.79	EFR3B	NP_055786.1	378	6	15.08
RIMKLA	NP_775913.2	287	1	3.31	POMC	NP_000930.1	2014	4	1.89
ZMYND12	NP_115633.3	61	1	15.57	DNMT3A	XP_016859015.1	1298	5	3.66
PPCS	NP_001274440.1	660	1	1.44	DTNB	NP_001338323.1	328	1	2.90
CCDC30	XP_024305270.1	88	1	10.79	ASXL2	NP_060733.4	421	6	13.54
PPIH	XP_016855546.1	1355	2	1.40	KIF3C	XP_005264356.2	1258	6	4.53
YBX1	NP_004550.2	1529	2	1.24	RAB10	NP_057215.3	939	4	4.05
CLDN19	NP_683763.2	238	1	3.99	GAREM2	XP_011530869.1	53	3	53.77
P3H1	NP_071751.3	327	1	2.91	HADHA	NP_000173.2	1024	3	2.78
C1orf50	NP_077002.2	211	1	4.50	HADHB	NP_000174.1	904	2	2.10
SVBP	XP_016856715.1	42	1	22.62	ADGRF3	XP_011530923.1	299	0	0.00
ERMAP	NP_061008.2	338	2	5.62	SELENOI	NP_277040.1	356	2	5.34
ZNF691	XP_006710734.1	184	0	0.00	DRC1	NP_659475.2	151	0	0.00
SLC2A1	NP_006507.2	1814	2	1.05	OTOF	NP_001274418.1	539	0	0.00
FAM183A	XP_016856811.1	14	0	0.00	FAM166C	NP_001098989.1	20	0	0.00
EBNA1BP2	NP_001153408.1	1348	4	2.82	CIB4	NP_001025052.1	946	4	4.02
CFAP57	XP_016855911.1	147	1	6.46	KCNK3	NP_002237.1	479	0	0.00
TMEM125	NP_653227.1	167	2	11.38	SLC35F6	NP_060347.2	216	2	8.80
C1orf210	NP_001158301.1	139	1	6.83	CENPA	NP_001800.1	1751	1	0.54
TIE1	NP_005415.1	791	2	2.40	DPYSL5	XP_024308775.1	544	1	1.75
MPL	XP_016856809.1	490	2	3.88	MAPRE3	NP_036458.2	1214	2	1.56
CDC20	NP_001246.2	2409	4	1.58	TMEM214	XP_005264438.1	331	1	2.87
ELOVL1	NP_001243330.1	412	1	2.31	AGBL5	NP_068603.4	176	3	16.19
MED8	NP_443109.2	327	1	2.91	OST4	NP_001128165.1	156	0	0.00
SZT2	XP_005270743.1	128	1	7.42	EMILIN1	NP_008977.1	285	1	3.33
HYI	XP_006711000.1	211	0	0.00	KHK	XP_005264351.1	370	8	20.54
PTPRF	NP_001316067.1	1212	5	3.92	CGREF1	NP_001159712.1	234	7	28.42
KDM4A	XP_005271411.1	982	7	6.77	ABHD1	NP_115993.3	346	8	21.96
ST3GAL3	NP_777623.2	407	6	14.00	PREB	NP_001317414.1	624	3	4.57
ARTN	NP_476431.2	307	0	0.00	PRR30	NP_848648.2	100	1	9.50
IPO13	NP_055467.3	350	4	10.86	TCF23	XP_005264216.1	211	10	45.02
DPH2	NP_001375.2	528	8	14.39	SLC5A6	XP_024308974.1	353	6	16.15
ATP6V0B	NP_004038.1	528	3	5.40	ATRAID	NP_001164266.1	171	8	44.44
B4GALT2	NP_085076.2	431	10	22.04	CAD	NP_004332.2	1171	10	8.11
CCDC24	NP_001336056.1	176	12	64.77	SLC30A3	XP_016860362.1	497	3	5.73

SLC6A9	NP_001315558.1	544	5	8.73	DNAJC5G	NP_001290057.1	369	0	0.00
KLF17	XP_005270522.2	371	3	7.68	TRIM54	NP_115935.3	586	1	1.62
DMAP1	NP_061973.1	895	7	7.43	UCN	NP_003344.1	438	2	4.34
ERI3	XP_016857793.1	357	7	18.63	MPV17	NP_002428.1	456	10	20.83
RNF220	NP_001363415.1	510	8	14.90	GTF3C2	NP_001305838.1	278	13	44.42
TMEM53	NP_001287677.1	158	4	24.05	EIF2B4	NP_056451.3	560	14	23.75
ARMH1	XP_011539647.1	27	2	70.37	SNX17	NP_001253990.1	459	14	28.97
KIF2C	NP_006836.2	1543	5	3.08	ZNF513	NP_653232.3	277	14	48.01
RPS8	NP_001003.1	1479	1	0.64	PPM1G	NP_817092.1	1328	16	11.45
BEST4	XP_016856511.1	255	0	0.00	NRBP1	NP_001308288.1	280	16	54.28
PLK3	NP_004064.2	1604	2	1.18	KRTCAP3	NP_001308254.1	278	16	54.67
TCTEX1D4	NP_001364463.1	76	0	0.00	IFT172	XP_005264311.1	478	17	33.78
BTBD19	XP_016855936.1	73	0	0.00	FNDC4	XP_005264556.1	277	16	54.87
PTCH2	NP_001159764.1	577	4	6.59	GCKR	NP_001477.2	417	15	34.17
EIF2B3	NP_065098.1	851	3	3.35	C2orf16	NP_115642.3	117	17	138.03
HECTD3	NP_078878.3	872	0	0.00	ZNF512	NP_115810.2	403	16	37.72
UROD	NP_000365.3	1694	4	2.24	CCDC121	NP_001136155.1	113	15	126.10
ZSWIM5	NP_065934.1	206	0	0.00	GPN1	NP_001138519.1	646	15	22.06
HPDL	NP_116145.1	269	4	14.13	SUPT7L	NP_001269661.1	176	13	70.17
MUTYH	NP_001280119.1	815	8	9.32	SLC4A1AP	NP_060628.2	230	12	49.56
TOE1	XP_005270469.1	564	7	11.79	MRPL33	NP_663303.1	601	8	12.64
TESK2	NP_009101.2	1006	7	6.61	RBKS	NP_071411.1	760	12	15.00
MMACHC	NP_056321.2	274	3	10.40	BABAM2	NP_001248769.1	214	2	8.88
PRDX1	NP_001189360.1	1672	11	6.25	FOSL2	XP_006712039.1	864	1	1.10
AKR1A1	NP_697021.1	606	6	9.41	PLB1	XP_011530891.1	404	2	4.70
NASP	NP_002473.2	1387	9	6.16	PPP1CB	NP_002700.1	2903	6	1.96
CCDC17	XP_016855943.1	64	5	74.21	SPDYA	NP_001136106.1	491	2	3.87
GPBP1L1	XP_016857508.1	102	6	55.88	TRMT61B	XP_016859890.1	661	1	1.44
TMEM69	NP_057570.2	101	5	47.03	WDR43	NP_055946.1	851	1	1.12
IPP	NP_001138821.1	495	1	1.92	TOGARAM2	XP_016858996.1	401	3	7.11
MAST2	XP_011539361.1	655	8	11.60	PCARE	NP_001025054.1	189	0	0.00
P3R3URF-PIK3	NP_001290356.1	855	2	2.22	CLIP4	XP_016860447.1	1233	3	2.31
PIK3R3	NP_001290357.1	217	0	0.00	ALK	NP_004295.2	1910	3	1.49
P3R3URF	NP_001315584.1	133	0	0.00	YPEL5	XP_016859807.1	389	9	21.98
TSPAN1	XP_011538762.1	362	0	0.00	LBH	NP_112177.2	90	1	10.56
POMGNT1	NP_001230695.2	311	0	0.00	LCLAT1	NP_872357.2	416	4	9.13
LURAP1	NP_001013633.1	89	4	42.69	CAPN13	XP_016860755.1	694	4	5.48
RAD54L	NP_003570.2	1605	4	2.37	GALNT14	NP_078848.2	301	3	9.47
LRRC41	NP_006360.3	360	2	5.28	CAPN14	NP_001138594.1	562	4	6.76
UQCRH	NP_005995.2	868	1	1.09	EHD3	NP_055415.1	636	2	2.99
NSUN4	NP_950245.2	794	1	1.20	XDH	XP_011531397.1	848	4	4.48
FAAH	NP_001432.2	647	2	2.94	SRD5A2	XP_011531374.1	494	3	5.77
DMBX1	NP_671725.1	597	4	6.36	MEMO1	NP_001372125.1	330	6	17.27
KNCN	NP_001091080.1	204	2	9.31	DPY30	NP_001308139.1	773	4	4.92
MKNK1	NP_001364267.1	371	3	7.68	SPAST	NP_055761.2	1402	5	3.39
MOB3C	NP_660322.3	241	0	0.00	SLC30A6	NP_001180442.1	268	5	17.72
ATPAF1	NP_001036011.2	444	1	2.14	NLRC4	NP_001289433.1	818	4	4.65
TEX38	XP_011539723.1	68	0	0.00	YIPF4	XP_005264656.1	301	1	3.16
EFCAB14	NP_055589.1	116	0	0.00	BIRC6	XP_005264511.2	2218	4	1.71
CYP4B1	NP_001093242.1	503	2	3.78	TTC27	NP_060205.3	666	4	5.71
CYP4A11	NP_001350516.1	532	1	1.79	LTBP1	XP_011531155.1	690	2	2.75
CYP4X1	NP_001307218.1	404	5	11.76	RASGRP3	XP_016859248.1	767	4	4.95
CYP4Z1	NP_835235.1	332	4	11.45	FAM98A	NP_056290.3	443	1	2.14
CYP4A22	NP_001010969.2	490	3	5.82	CRIM1	XP_011531200.1	560	1	1.70
PDZK1IP1	NP_005755.1	387	5	12.27	FEZ2	NP_005093.2	360	1	2.64
TAL1	XP_016857676.1	848	3	3.36	VIT	NP_444506.2	324	1	2.93
STIL	NP_001269868.1	559	6	10.20	STRN	XP_005264576.1	660	3	4.32
CMPK1	NP_057392.1	876	5	5.42	HEATR5B	XP_006712098.1	298	1	3.19
FOXE3	NP_036318.1	546	0	0.00	GPATCH11	NP_001358789.2	151	4	25.16
FOXD2	NP_004465.3	557	1	1.71	EIF2AK2	NP_001129124.1	966	1	0.98
TRABD2B	XP_016856751.1	86	1	11.05	SULT6B1	NP_001354480.1	111	1	8.56
SLC5A9	XP_011539226.1	248	1	3.83	CEBPZOS	NP_001309303.1	38	0	0.00
SPATA6	XP_006710762.1	198	0	0.00	CEBPZ	NP_005751.2	905	1	1.05
AGBL4	XP_016858084.1	240	0	0.00	NDUFAF7	NP_653337.1	247	0	0.00
BEND5	NP_078879.2	114	0	0.00	PRKD3	NP_005804.1	314	2	6.05
ELAVL4	NP_001311137.1	1527	3	1.87	QPCT	NP_036545.1	546	1	1.74

DMRTA2	NP_115486.1	294	1	3.23	CDC42EP3	NP_001358499.1	350	0	0.00
FAF1	NP_008982.1	653	4	5.82	RMDN2	NP_001309141.1	116	1	8.19
CDKN2C	NP_523240.1	1395	5	3.40	CYP1B1	NP_000095.2	910	4	4.18
C1orf185	XP_024302293.1	39	3	73.07	ATL2	NP_001129145.1	338	1	2.81
RNF11	NP_055187.1	644	8	11.80	HNRNPLL	XP_005264697.2	351	1	2.71
TTC39A	NP_001138304.1	134	4	28.36	GALM	NP_620156.1	608	1	1.56
EPS15	XP_016856107.1	1473	7	4.51	SRSF7	XP_005264542.1	996	4	3.82
OSBPL9	XP_011538901.1	235	2	8.08	GEMIN6	NP_079051.9	415	1	2.29
NRDC	NP_001095132.1	393	0	0.00	DHX57	NP_945314.1	1122	2	1.69
RAB3B	XP_016857447.1	644	1	1.48	MORN2	NP_001138922.1	129	0	0.00
TXNDC12	NP_056997.1	546	3	5.22	ARHGEF33	NP_001138923.2	102	0	0.00
KTI12	NP_612426.1	197	1	4.82	SOS1	NP_001369323.1	1297	3	2.20
BTF3L4	NP_689478.1	580	1	1.64	CDKL4	XP_011531119.1	127	2	14.96
ZFYVE9	NP_004790.2	529	2	3.59	MAP4K3	NP_001257354.1	479	5	9.92
CC2D1B	NP_001317514.1	294	2	6.46	TMEM178A	XP_024308470.1	530	2	3.58
ORC1	NP_001177748.1	1384	0	0.00	THUMPD2	XP_011531421.1	200	1	4.75
PRPF38A	XP_011540617.1	785	2	2.42	SLC8A1	XP_016860234.1	714	1	1.33
TUT4	XP_016856297.1	460	0	0.00	PKDCC	NP_612379.2	1211	2	1.57
GPX7	NP_056511.2	726	2	2.62	EML4	XP_005264324.1	861	3	3.31
SHISAL2A	XP_011539679.1	27	0	0.00	COX7A2L	NP_001305967.1	348	0	0.00
COA7	NP_075565.2	344	2	5.52	KCNG3	NP_758847.1	642	3	4.44
ZYG11B	NP_078922.1	174	0	0.00	MTA3	NP_001317371.1	398	1	2.39
ZYG11A	NP_001004339.2	151	0	0.00	OXER1	NP_683765.1	469	1	2.03
ECHDC2	XP_024303929.1	610	2	3.11	HAAO	XP_011531031.1	564	1	1.68
SCP2	NP_002970.2	672	2	2.83	ZFP36L2	NP_008818.3	779	2	2.44
PODN	XP_016855783.1	647	2	2.94	THADA	NP_001332853.1	663	3	4.30
SLC1A7	NP_006662.3	868	1	1.09	PLEKHH2	NP_742066.2	206	2	9.22
CPT2	NP_000089.1	779	2	2.44	DYNC2LI1	NP_001335842.1	292	3	9.76
CZIB	NP_001291689.1	208	0	0.00	ABCG5	XP_006712136.1	794	2	2.39
MAGOH	NP_002361.1	1185	2	1.60	ABCG8	XP_011531331.1	858	3	3.32
LRP8	XP_011540397.1	765	3	3.73	LRPPRC	XP_011530775.1	871	7	7.63
DMRTB1	NP_149056.1	231	1	4.11	PPM1B	XP_016859885.1	997	6	5.72
GLIS1	XP_016855898.1	446	1	2.13	SLC3A1	NP_000332.2	619	8	12.28
NDC1	NP_001162023.1	666	2	2.85	PREPL	NP_001035844.1	326	4	11.66
YIPF1	NP_061855.1	309	0	0.00	CAMKMT	XP_016860460.1	433	6	13.16
DIO1	NP_000783.2	232	0	0.00	SIX3	NP_005404.1	735	1	1.29
HSPB11	NP_001369190.1	767	1	1.24	SIX2	NP_058628.3	652	2	2.91
LRRC42	NP_443172.1	9	0	0.00	SRBD1	NP_060549.4	868	1	1.09
LDLRAD1	NP_001263321.1	75	2	25.33	PRKCE	NP_005391.1	1473	5	3.22
TMEM59	NP_001291979.1	221	2	8.60	EPAS1	NP_001421.2	1196	2	1.59
TCEANC2	NP_694580.1	522	3	5.46	TMEM247	NP_001138523.1	45	1	21.11
CDCP2	NP_001340584.1	62	1	15.32	ATP6V1E2	XP_016860714.1	336	0	0.00
CYB5RL	NP_001340282.1	305	0	0.00	RHOQ	NP_036381.2	1752	7	3.80
MRPL37	NP_001317531.1	353	0	0.00	PIGF	NP_002634.1	111	5	42.79
SSBP3	XP_006710608.1	259	0	0.00	CRIPT	NP_054890.1	214	7	31.07
ACOT11	NP_056362.1	409	1	2.32	SOCS5	NP_659198.1	570	8	13.33
FAM151A	NP_788954.2	268	0	0.00	MCFD2	NP_001164982.1	343	6	16.62
MROH7	NP_001034553.3	76	0	0.00	TTC7A	NP_001275882.1	215	7	30.93
TTC4	NP_004614.3	1293	0	0.00	STPG4	NP_001157033.1	25	2	76.00
PARS2	NP_689481.2	885	0	0.00	CALM2	NP_001292553.1	1880	7	3.54
TTC22	XP_011539973.1	94	1	10.11	EPCAM	NP_002345.2	1196	4	3.18
LEXM	NP_001104003.1	188	0	0.00	MSH2	XP_011531169.1	2165	10	4.39
DHCR24	NP_055577.1	798	2	2.38	KCNK12	NP_071338.1	437	8	17.39
TMEM61	NP_872338.1	78	0	0.00	MSH6	XP_024308587.1	1559	6	3.66
BSND	NP_476517.1	274	3	10.40	FBXO11	XP_016860504.1	729	14	18.24
PCSK9	NP_777596.2	1316	6	4.33	FOXN2	NP_001362372.1	554	7	12.00
USP24	XP_016856321.1	815	3	3.50	PPP1R21	NP_001129101.1	145	3	19.65
PLPP3	NP_003704.3	648	6	8.80	STON1-GTF2A	NP_001185522.1	423	8	17.97
PRKAA2	NP_006243.2	1752	8	4.34	STON1	NP_006864.2	329	11	31.76
FYB2	XP_005270641.1	127	11	82.28	GTF2A1L	NP_006863.2	278	7	23.92
C8A	NP_000553.1	527	7	12.62	LHCGR	NP_000224.2	701	10	13.55
C8B	XP_016857724.1	590	10	16.10	FSHR	NP_852111.2	697	10	13.63
DAB1	NP_001340915.1	374	0	0.00	NRXN1	NP_001317025.1	1566	8	4.85
OMA1	NP_660286.1	580	5	8.19	GPR75-ASB3	NP_001157637.1	979	3	2.91
TACSTD2	NP_002344.2	499	7	13.33	ASB3	NP_057199.1	979	3	2.91
MYSM1	XP_006710377.1	1911	9	4.47	CHAC2	NP_001008708.1	249	3	11.45

JUN	NP_002219.1	4160	7	1.60	ERLEC1	XP_024308560.1	699	8	10.87
FGGY	XP_016857132.1	982	14	13.54	GPR75	NP_006785.1	164	7	40.55
HOOK1	NP_056972.1	356	15	40.03	PSME4	NP_055429.2	814	13	15.17
CYP2J2	NP_000766.2	467	6	12.20	ACYP2	XP_016860900.1	257	5	18.48
C1orf87	NP_689590.1	172	8	44.18	TSPYL6	NP_001003937.2	425	5	11.18
NFIA	XP_016856851.1	690	4	5.51	C2orf73	XP_024308455.1	118	3	24.15
TM2D1	NP_114416.1	216	17	74.76	SPTBN1	XP_016860268.1	1126	8	6.75
PATJ	XP_011538764.1	1812	9	4.72	EML6	XP_016859587.1	640	2	2.97
L1TD1	NP_061952.3	247	14	53.84	RTN4	NP_722550.1	1250	2	1.52
KANK4	XP_016855973.1	892	15	15.97	CLHC1	NP_001340709.1	812	0	0.00
USP1	NP_003359.3	1088	11	9.60	RPS27A	NP_001129064.1	3220	6	1.77
DOCK7	NP_001258930.1	590	16	25.76	MTIF2	XP_016859653.1	1124	4	3.38
ANGPTL3	NP_055310.1	681	12	16.74	CCDC88A	NP_001241872.1	373	2	5.09
ATG4C	XP_024306182.1	789	14	16.86	CFAP36	NP_001269690.1	239	2	7.95
FOXD3	NP_036315.1	1000	13	12.35	PPP4R3B	XP_016860025.1	768	2	2.47
ALG6	NP_037471.2	321	19	56.23	PNPT1	NP_149100.2	1176	2	1.62
ITGB3BP	NP_001334074.1	460	17	35.11	EFEMP1	XP_005264262.1	851	2	2.23
EFCAB7	XP_011540603.1	1033	20	18.39	CCDC85A	NP_001335441.1	466	1	2.04
PGM1	NP_002624.2	1078	8	7.05	VRK2	NP_001123952.1	789	2	2.41
ROR1	NP_005003.2	1030	5	4.61	FANCL	NP_060532.2	521	5	9.12
UBE2U	NP_001353161.1	1514	19	11.92	BCL11A	NP_001350793.1	863	10	11.01
CACHD1	NP_065976.3	223	19	80.94	PAPOLG	NP_075045.2	705	12	16.17
RAVER2	NP_001353094.1	114	16	133.33	REL	NP_002899.1	1692	8	4.49
JAK1	NP_001308786.1	1907	11	5.48	PUS10	XP_016858917.1	352	13	35.08
AK4	NP_982289.1	799	16	19.02	PEX13	NP_002609.1	389	12	29.30
DNAJC6	NP_055602.1	2096	18	8.16	KIAA1841	NP_001317364.1	119	14	111.76
LEPROT	NP_059996.1	214	13	57.71	C2orf74	NP_001137431.2	115	14	115.65
LEPR	NP_002294.2	987	5	4.81	USP34	NP_055524.3	866	11	12.07
PDE4B	NP_001284370.1	916	12	12.44	XPO1	XP_005264601.1	3232	15	4.41
SGIP1	XP_006711024.1	974	13	12.68	FAM161A	NP_115556.2	334	13	36.97
TCTEX1D1	XP_016856055.1	202	14	65.84	CCT4	NP_001243650.1	1825	18	9.37
INSL5	NP_005469.2	349	10	27.22	COMMD1	NP_689729.1	709	18	24.12
DNAI4	XP_016857843.1	704	11	14.84	B3GNT2	NP_001306004.1	373	14	35.65
MIER1	NP_001071171.2	442	8	17.19	TMEM17	XP_011530996.1	199	11	52.51
SLC35D1	NP_055954.1	466	12	24.46	EHBP1	NP_001341141.1	1112	15	12.81
C1orf141	NP_001263280.1	143	10	66.43	OTX1	NP_055377.1	611	14	21.77
IL23R	NP_653302.2	768	3	3.71	WDPCP	XP_005264405.1	320	17	50.47
IL12RB2	NP_001361188.1	541	2	3.51	MDH1	NP_001303303.1	1322	12	8.62
SERBP1	NP_056455.3	1246	6	4.57	UGP2	NP_006750.3	996	13	12.40
GADD45A	NP_001915.1	1261	1	0.75	VPS54	NP_001005739.1	534	12	21.35
GNG12	XP_016857298.1	1030	3	2.77	PELI1	XP_016860009.1	377	13	32.76
DIRAS3	NP_004666.1	1442	1	0.66	LGALSL	NP_054900.2	169	13	73.07
WLS	NP_001002292.3	527	1	1.80	AFTPH	NP_001362898.1	198	16	76.76
RPE65	NP_000320.1	555	2	3.42	SERTAD2	XP_005264726.1	205	12	55.61
DEPDC1	NP_060249.2	477	0	0.00	SLC1A4	NP_003029.2	424	6	13.44
LRRC7	NP_001357714.1	1062	1	0.89	CEP68	NP_055962.2	112	13	110.26
LRRC40	NP_060238.3	587	2	3.24	RAB1A	NP_056358.1	1360	12	8.38
SRSF11	NP_001337537.1	818	2	2.32	ACTR2	NP_001005386.1	1716	9	4.98
ANKRD13C	NP_110443.3	131	0	0.00	SPRED2	XP_005264259.1	520	10	18.27
CTH	NP_001893.2	934	1	1.02	MEIS1	NP_002389.1	1032	4	3.68
PTGER3	XP_011540112.1	787	1	1.21	ETAA1	NP_061875.2	171	9	50.00
ZRANB2	NP_976225.1	623	2	3.05	C1D	NP_775269.1	558	0	0.00
NEGR1	NP_776169.2	849	5	5.59	WDR92	NP_612467.1	79	7	84.17
LRRIQ3	NP_001099129.1	555	3	5.13	PNO1	NP_064528.1	1095	6	5.21
FPGT-TNNI3K	NP_001106279.3	751	2	2.53	PPP3R1	NP_000936.1	1540	8	4.93
FPGT	NP_003829.4	259	4	14.67	CNRIP1	NP_001104571.1	237	7	28.06
TNNI3K	NP_057062.1	958	4	3.97	PLEK	NP_002655.2	2073	8	3.67
LRRC53	XP_011540814.2	386	1	2.46	FBXO48	XP_016859926.1	65	7	102.30
ERICH3	XP_016855764.1	326	0	0.00	APLF	NP_775816.1	253	8	30.04
CRYZ	XP_016855856.1	762	5	6.23	PROKR1	NP_620414.1	181	0	0.00
TYW3	NP_612476.1	223	5	21.30	ARHGAP25	NP_001007232.2	478	2	3.97
LHX8	NP_001001933.1	851	1	1.12	BMP10	NP_055297.1	571	1	1.66
SLC44A5	XP_016856097.1	216	6	26.39	GKN2	NP_872342.2	252	0	0.00
ACADM	NP_001120800.1	1058	7	6.29	GKN1	NP_062563.4	272	2	6.98
RABGGTB	NP_004573.2	741	5	6.41	ANTXR1	NP_115584.1	617	3	4.62
MSH4	NP_002431.2	674	5	7.05	GFPT1	NP_002047.2	1037	4	3.66

ASB17	XP_006710409.1	322	2	5.90	NFU1	NP_001002755.1	550	7	12.09
ST6GALNAC3	NP_001336038.1	332	8	22.89	AAK1	NP_001358506.1	1204	6	4.73
ST6GALNAC5	NP_112227.1	435	4	8.74	ANXA4	NP_001307627.1	683	4	5.56
PIGK	NP_005473.1	349	3	8.17	GMCL1	NP_848526.1	278	8	27.34
AK5	NP_777283.1	804	0	0.00	SNRNP27	NP_006848.1	733	5	6.48
ZZZ3	NP_001363085.1	386	2	4.92	MXD1	NP_002348.1	372	7	17.88
USP33	NP_001364359.1	671	0	0.00	ASPRV1	NP_690005.2	240	0	0.00
MIGA1	NP_001257313.1	158	8	48.10	PCBP1	NP_006187.2	1232	5	3.86
NEXN	XP_005271379.1	492	1	1.93	C2orf42	NP_001335688.1	45	1	21.11
FUBP1	NP_001362985.1	1089	1	0.87	TIA1	NP_001338439.1	1413	7	4.71
DNAJB4	NP_001304029.1	785	1	1.21	PCYOX1	NP_057381.3	222	2	8.56
GIPC2	NP_001291654.1	384	6	14.84	SNRPG	NP_001304095.1	1511	4	2.51
PTGFR	NP_000950.1	505	4	7.52	FAM136A	NP_116211.2	405	1	2.35
IFI44L	NP_006811.2	416	0	0.00	TGFA	NP_001093161.1	600	1	1.58
IFI44	XP_011538818.1	416	1	2.28	ADD2	NP_001171983.1	681	4	5.58
ADGRL4	NP_071442.2	674	5	7.05	FIGLA	NP_001004311.2	365	2	5.21
ADGRL2	XP_016856273.1	734	5	6.47	CLEC4F	NP_001308237.1	218	2	8.72
TTLL7	NP_001337143.1	415	5	11.45	CD207	XP_011531178.1	510	1	1.86
PRKACB	NP_002722.1	2722	3	1.05	VAX2	NP_036608.1	331	3	8.61
SAMD13	NP_001010971.1	36	1	26.39	ATP6V1B1	NP_001683.2	772	3	3.69
DNASE2B	NP_067056.2	264	0	0.00	ANKRD53	NP_001108588.1	912	2	2.08
RPF1	NP_079341.2	952	0	0.00	TEX261	NP_653183.2	92	0	0.00
GNG5	NP_005265.1	812	5	5.85	NAGK	XP_016859929.1	366	2	5.19
CTBS	NP_004379.1	322	2	5.90	MCEE	XP_005264670.1	756	5	6.28
SSX2IP	XP_016855724.1	362	0	0.00	MPHOSPH10	NP_005782.1	819	1	1.16
LPAR3	XP_024301895.1	671	2	2.83	PAIP2B	XP_011531144.1	211	3	13.51
MCOLN2	NP_694991.2	295	1	3.22	ZNF638	NP_055312.2	338	1	2.81
MCOLN3	XP_011540042.1	330	1	2.88	DYSF	XP_005264642.1	729	4	5.21
DNAI3	NP_660155.2	272	0	0.00	CYP26B1	NP_001264671.1	651	6	8.76
SYDE2	NP_115560.1	317	3	8.99	EXOC6B	NP_001308662.1	687	5	6.91
C1orf52	NP_932343.1	55	5	86.36	SPR	NP_003115.1	389	1	2.44
BCL10	NP_001307644.1	661	2	2.87	EMX1	XP_011530999.1	691	7	9.62
DDAH1	XP_005270764.1	411	6	13.87	SFXN5	NP_001358668.1	154	2	12.34
CCN1	NP_001545.2	907	4	4.19	RAB11FIP5	NP_056285.1	437	4	8.70
ZNHIT6	NP_001164141.1	409	4	9.29	NOTO	NP_001127934.1	255	0	0.00
COL24A1	NP_690850.2	397	2	4.79	SMYD5	NP_006053.2	1670	4	2.28
ODF2L	NP_001171695.1	144	4	26.39	PRADC1	NP_115695.1	44	0	0.00
CLCA2	NP_006527.1	218	3	13.07	CCT7	NP_006420.1	1694	3	1.68
CLCA1	NP_001276.3	508	4	7.48	FBXO41	XP_016858910.1	458	0	0.00
CLCA4	NP_036260.2	396	5	11.99	EGR4	NP_001956.4	548	2	3.47
SH3GLB1	XP_006710735.1	412	3	6.92	ALMS1	NP_055935.4	607	3	4.69
SELENOF	NP_004252.2	373	1	2.55	NAT8	NP_003951.3	458	3	6.22
HS2ST1	NP_036394.1	207	1	4.59	TPRKB	NP_001317321.1	586	4	6.48
LMO4	NP_001356420.1	573	0	0.00	DUSP11	NP_003575.2	463	3	6.16
PKN2	NP_006247.1	1140	8	6.67	C2orf78	NP_001073943.1	64	1	14.84
GTF2B	NP_001505.1	1278	2	1.49	STAMBP	NP_001340897.1	1058	1	0.90
KYAT3	NP_001336377.1	429	9	19.93	ACTG2	NP_001606.1	1921	2	0.99
RBMXL1	NP_062556.2	264	1	3.60	DGUOK	NP_550438.1	656	2	2.90
GBP3	XP_011539536.1	314	3	9.08	TET3	XP_024308513.1	502	3	5.68
GBP1	NP_002044.2	611	2	3.11	BOLA3	NP_001030582.1	542	0	0.00
GBP2	NP_004111.2	593	6	9.61	MOB1A	NP_001304039.1	474	0	0.00
GBP7	NP_997281.2	236	4	16.10	MTHFD2	NP_006627.2	1000	0	0.00
GBP4	NP_443173.2	403	4	9.43	SLC4A5	NP_597812.1	419	2	4.53
GBP5	NP_443174.1	568	5	8.36	DCTN1	NP_001128512.1	1329	3	2.14
GBP6	NP_940862.2	286	3	9.96	C2orf81	NP_001303694.1	164	0	0.00
LRRC8B	XP_016856372.1	541	1	1.76	WDR54	XP_016860553.1	64	0	0.00
LRRC8C	XP_006711023.1	536	1	1.77	RTKN	NP_001015055.1	392	0	0.00
LRRC8D	XP_016857090.1	568	1	1.67	INO80B	NP_112578.2	305	2	6.23
ZNF326	NP_892021.1	362	1	2.62	WBP1	NP_036609.1	80	0	0.00
BARHL2	NP_064447.1	792	2	2.40	MOGS	NP_006293.2	677	0	0.00
ZNF644	XP_016857977.1	464	2	4.09	MRPL53	NP_444278.1	390	2	4.87
HFM1	XP_011539159.1	912	5	5.21	CCDC142	NP_116168.3	1	1	949.95
CDC7	XP_024305857.1	1198	4	3.17	TTC31	XP_011531337.1	735	11	14.22
TGFBR3	NP_001182613.1	604	1	1.57	LBX2	NP_001009812.1	260	4	14.61
BRDT	NP_001229735.2	1412	2	1.35	PCGF1	NP_116062.2	422	9	20.26
EPHX4	NP_775838.3	263	0	0.00	TLX2	NP_057254.1	372	10	25.54

SETSIP	NP_001274666.1	537	1	1.77	DQX1	NP_598376.2	952	10	9.98
BTBD8	NP_001363060.1	81	1	11.73	AUP1	NP_853553.1	584	10	16.27
C1orf146	NP_001012425.1	54	1	17.59	HTRA2	NP_001308656.1	1081	8	7.03
GLMN	XP_016855629.1	533	5	8.91	LOXL3	NP_001276093.1	249	10	38.15
RPAP2	XP_005271280.1	531	1	1.79	DOK1	NP_001372.1	397	9	21.54
GFI1	NP_001120687.1	1062	4	3.58	M1AP	XP_011530854.1	121	9	70.66
EVI5	NP_005656.4	424	5	11.20	SEMA4F	XP_011530778.1	722	6	7.89
RPL5	NP_000960.2	1668	4	2.28	HK2	XP_005264337.1	1116	0	0.00
DIPK1A	NP_001239202.1	176	3	16.19	POLE4	NP_063949.2	690	1	1.38
MTF2	NP_031384.1	529	3	5.39	TACR1	NP_001049.1	721	2	2.64
TMED5	NP_057124.3	424	4	8.96	EVA1A	NP_115557.1	312	2	6.09
CCDC18	XP_011539663.2	250	1	3.80	MRPL19	NP_055578.2	970	4	3.92
DR1	NP_001929.1	666	3	4.28	GCFC2	XP_011531376.1	514	2	3.70
FNBP1L	XP_011539927.1	709	1	1.34	LRRTM4	NP_001317299.1	699	1	1.36
BCAR3	XP_016857969.1	308	3	9.25	REG3G	NP_940850.1	356	0	0.00
DNTTIP2	NP_055412.2	660	1	1.44	REG1B	NP_006498.1	258	0	0.00
GCLM	NP_001295182.1	521	0	0.00	REG1A	NP_002900.2	468	0	0.00
ABCA4	NP_000341.2	736	6	7.74	REG3A	NP_620354.1	423	0	0.00
ARHGAP29	NP_001315594.1	578	3	4.93	CTNNA2	NP_001269527.1	1440	2	1.32
ABCD3	XP_006710865.2	496	5	9.58	LRRTM1	XP_016859475.1	1318	1	0.72
F3	NP_001984.1	943	3	3.02	SUCLG1	NP_003840.2	1696	1	0.56
SLC44A3	NP_001107578.1	240	2	7.92	DNAH6	NP_001361.1	628	0	0.00
CNN3	NP_001272984.1	712	7	9.34	TRABD2A	NP_001074293.1	82	0	0.00
ALG14	NP_659425.1	221	3	12.90	TMSB10	NP_066926.1	219	0	0.00
TLCD4	NP_689700.1	196	6	29.08	KCMF1	NP_064507.3	460	0	0.00
TLCD4-RWDD:	NP_001186620.1	98	5	48.47	TCF7L1	XP_006712172.1	896	5	5.30
RWDD3	NP_056300.3	114	6	50.00	TGOLN2	NP_001355025.1	862	6	6.61
PTBP2	XP_011540178.1	659	6	8.65	RETSAT	NP_060220.3	403	10	23.57
DPYD	XP_016855996.1	1148	3	2.48	ELMOD3	NP_001316720.1	146	12	78.08
SNX7	XP_006710740.1	510	7	13.04	CAPG	NP_001243069.1	381	4	9.97
PLPPR5	NP_001010861.1	231	6	24.67	SH2D6	XP_016859332.1	59	13	209.31
PLPPR4	NP_001159724.1	294	7	22.62	MAT2A	NP_005902.1	1347	9	6.35
PALMD	NP_060204.1	190	8	40.00	GGCX	XP_016859292.1	266	10	35.71
FRRS1	NP_001013682.2	371	7	17.92	VAMP8	NP_003752.2	1139	11	9.17
AGL	NP_000019.2	852	3	3.34	VAMP5	NP_006625.1	832	13	14.84
SLC35A3	NP_001258614.1	517	4	7.35	RNF181	XP_005264416.1	368	13	33.56
MFSD14A	NP_149044.2	203	4	18.72	TMEM150A	NP_001356846.1	96	13	128.64
SASS6	NP_919268.1	586	0	0.00	C2orf68	NP_001013671.2	92	12	123.91
TRMT13	NP_061956.2	212	5	22.40	USP39	NP_001243656.1	699	10	13.59
LRRC39	NP_653221.1	505	10	18.81	SFTPB	NP_001354210.1	424	4	8.96
DBT	NP_001909.4	642	10	14.80	GNLY	XP_005264141.1	471	1	2.02
RTCA	NP_001124313.1	775	9	11.03	ATOH8	NP_116216.2	342	0	0.00
CDC14A	XP_016858135.1	1231	8	6.17	ST3GAL5	NP_001350776.1	377	2	5.04
GPR88	NP_071332.2	610	11	17.13	POLR1A	NP_056240.2	2034	5	2.34
VCAM1	NP_001069.1	1746	9	4.90	PTCD3	NP_060422.4	415	2	4.58
EXTL2	XP_016856140.1	232	1	4.09	IMMT	XP_005264171.1	1065	1	0.89
SLC30A7	XP_016855889.1	340	11	30.73	MRPL35	NP_001350711.1	397	2	4.79
DPH5	NP_001070863.1	709	10	13.40	REEP1	NP_001158204.1	1080	2	1.76
S1PR1	NP_001307659.1	768	10	12.37	KDM3A	NP_060903.2	545	0	0.00
OLFM3	NP_477518.2	1038	5	4.58	RNF103-CHM	NP_001185883.1	729	1	1.30
COL11A1	NP_001177638.1	774	3	3.68	CHMP3	NP_001180446.1	685	1	1.39
RNPC3	NP_060089.1	704	3	4.05	RNF103	NP_001185880.1	452	2	4.20
AMY2B	NP_066188.1	473	2	4.02	RMND5A	NP_073617.1	344	0	0.00
AMY2A	NP_000690.1	556	2	3.42	CD8A	NP_741969.1	1172	1	0.81
AMY1A	NP_004029.2	408	1	2.33	CD8B	NP_742100.1	485	3	5.88
AMY1B	NP_001008219.1	408	1	2.33	RGPD1	XP_016859595.1	341	1	2.79
AMY1C	XP_016856547.1	408	1	2.33	PLGLB1	NP_001027564.1	359	1	2.65
PRMT6	NP_060607.2	1183	2	1.61	LOC10272464	XP_024309048.1	1032	1	0.92
NTNG1	XP_016856172.1	909	5	5.23	PLGLB2	NP_002656.1	364	1	2.61
VAV3	XP_016855542.1	906	3	3.15	RGPD2	XP_016860334.1	461	0	0.00
SLC25A24	NP_037518.3	402	7	16.54	KRCC1	XP_016859781.1	74	7	89.86
NBPF4	NP_001137461.1	38	3	75.00	SMYD1	NP_938015.1	1846	8	4.12
NBPF6	XP_016857637.1	27	5	175.92	FABP1	NP_001434.1	907	8	8.38
FAM102B	NP_001010883.2	147	11	71.08	THNSL2	XP_024308743.1	425	7	15.65
HENMT1	XP_005270468.1	326	10	29.14	FOXI3	NP_001129121.1	471	8	16.14
PRPF38B	NP_060531.2	638	9	13.40	TEX37	XP_005264245.3	163	7	40.80

FNDC7	NP_001138409.1	58	12	196.54	EIF2AK3	NP_004827.4	1097	4	3.46
STXBP3	NP_009200.2	702	5	6.77	RPIA	NP_653164.2	933	6	6.11
AKNAD1	NP_689976.2	100	14	132.99	LOC10050962	XP_006712950.1	314	1	3.03
GPSM2	NP_001307968.1	732	13	16.87	TEKT4	XP_011508972.1	372	8	20.43
CLCC1	NP_001364398.1	222	14	59.91	MAL	NP_002362.1	287	0	0.00
WDR47	XP_016856186.1	211	12	54.03	MRPS5	NP_114108.1	1323	10	7.18
TAF13	NP_005636.1	459	11	22.77	ZNF514	NP_001304934.1	238	10	39.91
TMEM167B	NP_001309177.1	94	12	121.27	ZNF2	NP_001278534.1	409	10	23.23
C1orf194	NP_001353131.1	157	12	72.61	PROM2	NP_001159450.1	273	13	45.24
ELAPOR1	NP_065826.3	336	8	22.62	KCNIP3	NP_038462.1	1372	11	7.62
SARS1	NP_001317598.1	888	1	1.07	FAHD2A	XP_011509584.1	219	16	69.40
CELSR2	NP_001399.1	847	11	12.34	TRIM43B	XP_011509971.1	312	14	42.63
PSRC1	XP_016858065.1	487	6	11.70	TRIM43	NP_620155.1	312	15	45.67
MYBPHL	XP_016856664.1	180	5	26.39	ANKRD36C	NP_001297083.1	1002	18	17.06
SORT1	XP_005271157.1	899	6	6.34	GPAT2	NP_001308459.1	337	13	36.64
PSMA5	NP_002781.2	1611	5	2.95	ADRA2B	NP_000673.2	758	4	5.01
SYPL2	XP_011539585.1	198	9	43.18	ASTL	XP_011509508.1	310	18	55.16
ATXN7L2	NP_001337104.1	69	4	55.07	DUSP2	NP_004409.1	936	11	11.16
CYB561D1	XP_011539589.2	102	1	9.31	STARD7	NP_064536.2	270	17	59.81
AMIGO1	NP_065754.2	227	4	16.74	TMEM127	XP_016859939.1	169	15	84.32
GPR61	NP_114142.3	302	5	15.73	CIAO1	XP_024309015.1	521	18	32.82
GNAI3	NP_006487.1	1295	8	5.87	SNRNP200	NP_054733.2	1355	16	11.22
GNAT2	NP_001364224.1	947	3	3.01	ITPRIPL1	XP_016858916.1	101	20	188.11
AMPD2	NP_631895.1	539	6	10.57	NCAPH	NP_056156.2	1190	14	11.18
GSTM4	NP_671489.1	489	3	5.83	NEURL3	NP_001272414.1	229	13	53.93
GSTM2	NP_001135840.1	572	2	3.32	ARID5A	NP_001306014.1	470	17	34.36
GSTM1	NP_000552.2	844	1	1.13	KANSL3	XP_005264044.1	305	6	18.69
GSTM5	NP_000842.2	483	0	0.00	FER1L5	XP_011510412.1	204	18	83.82
GSTM3	NP_000840.2	712	1	1.33	LMAN2L	NP_110432.1	475	18	36.00
EPS8L3	NP_078802.2	383	4	9.92	CNNM4	XP_005263971.1	615	15	23.17
CSF1	NP_000748.4	1379	2	1.38	CNNM3	XP_011509259.1	388	17	41.62
AHCYL1	XP_011538837.1	950	1	1.00	ANKRD23	NP_659431.5	1051	14	12.65
STRIP1	NP_149079.2	334	4	11.38	ANKRD39	NP_057550.3	872	18	19.61
ALX3	NP_006483.2	795	7	8.36	SEMA4C	XP_016859882.1	461	8	16.49
UBL4B	NP_981957.1	1774	6	3.21	FAM178B	XP_011509596.1	73	17	221.22
SLC6A17	XP_006710706.1	1012	6	5.63	FAHD2B	XP_016858960.1	193	17	83.67
KCNC4	XP_006710688.1	825	4	4.61	ANKRD36	XP_016859498.1	1007	15	14.15
RBM15	NP_001188474.1	670	1	1.42	ANKRD36B	XP_016860072.1	910	2	2.09
SLC16A4	NP_001188475.1	418	2	4.55	COX5B	NP_001853.2	830	10	11.45
LAMTOR5	NP_006393.2	382	2	4.97	ACTR1B	NP_005726.1	1317	12	8.66
PROK1	NP_115790.1	318	3	8.96	ZAP70	XP_016860358.1	1945	3	1.47
KCNA10	NP_005540.1	597	2	3.18	TMEM131	XP_005263969.1	286	6	19.93
KCNA2	NP_001191198.1	1435	4	2.65	VWA3B	NP_659429.4	123	8	61.79
KCNA3	NP_002223.3	974	4	3.90	CNGA3	XP_011508856.1	505	6	11.29
LRIF1	XP_016857258.1	93	1	10.21	INPP4A	XP_006712571.1	314	1	3.03
CD53	NP_000551.1	725	5	6.55	COA5	NP_001008216.1	190	1	5.00
DRAM2	NP_001336813.1	166	2	11.45	UNC50	NP_001317283.1	297	2	6.40
CEPT1	XP_024305848.1	542	2	3.51	MGAT4A	NP_001153626.1	351	1	2.71
DENND2D	NP_001258762.1	273	8	27.84	CRACDL	XP_016859460.1	192	0	0.00
CHI3L2	XP_024308520.1	160	3	17.81	TSGA10	XP_016860524.1	200	2	9.50
CHIA	NP_970615.2	524	3	5.44	C2orf15	NP_001304921.2	21	1	45.24
PIFO	XP_005270529.1	351	3	8.12	MITD1	XP_016858803.1	262	6	21.75
OVGP1	NP_002548.3	240	3	11.87	LIPT1	NP_660198.1	317	3	8.99
WDR77	NP_001303993.1	1306	3	2.18	MRPL30	NP_660213.1	345	6	16.52
ATP5PB	NP_001679.2	966	1	0.98	LYG2	XP_016859240.1	235	6	24.25
C1orf162	XP_016855813.1	221	4	17.19	LYG1	XP_016858800.1	84	9	101.78
TMIGD3	NP_001289609.1	940	2	2.02	TXNDC9	XP_016858636.1	1283	9	6.66
RAP1A	NP_001357145.1	1923	3	1.48	EIF5B	NP_056988.3	1515	7	4.39
INKA2	NP_945120.1	106	0	0.00	REV1	XP_016859798.1	832	2	2.28
DDX20	NP_009135.4	1347	3	2.12	AFF3	XP_011509471.2	488	4	7.79
KCND3	NP_001365899.1	1082	0	0.00	LONRF2	NP_940863.3	3665	11	2.85
CTTNBP2NL	XP_016857295.1	249	2	7.63	CHST10	XP_016860869.1	338	4	11.24
WNT2B	NP_004176.2	1046	2	1.82	NMS	NP_001011717.1	438	0	0.00
ST7L	XP_011539930.1	192	4	19.79	PDCL3	NP_076970.1	426	6	13.38
CAPZA1	XP_016857913.1	991	5	4.79	NPAS2	XP_005264016.1	702	9	12.18
MOV10	NP_001308253.1	977	3	2.92	RPL31	NP_001092047.1	1157	3	2.46

RHOC	NP_001036143.1	2253	6	2.53	TBC1D8	XP_011508809.1	554	7	12.00
PPM1J	NP_005158.5	107	0	0.00	CNOT11	NP_060016.3	229	3	12.44
TAFA3	NP_001004440.1	78	0	0.00	RNF149	XP_005263978.1	264	9	32.38
SLC16A1	NP_003042.3	559	0	0.00	CREG2	NP_722578.1	312	2	6.09
LRIG2	NP_055628.1	606	4	6.27	RFX8	XP_011510073.1	413	1	2.30
MAGI3	NP_001136254.1	1876	10	5.06	MAP4K4	XP_005264105.1	656	10	14.48
PHTF1	NP_001309970.1	209	8	36.36	IL1R2	XP_006712799.1	720	9	11.87
RSBN1	NP_060834.2	257	8	29.57	IL1R1	NP_001307907.1	1139	10	8.34
PTPN22	NP_001295226.1	1365	7	4.87	IL1RL2	XP_016860663.1	195	6	29.23
BCL2L15	NP_001010922.1	236	8	32.20	IL1RL1	NP_057316.3	599	7	11.10
AP4B1	XP_024308209.1	741	10	12.82	IL18R1	XP_024308970.1	668	7	9.95
DCLRE1B	NP_073747.1	592	5	8.02	IL18RAP	XP_024308965.1	650	7	10.23
HIPK1	XP_024309676.1	766	5	6.20	SLC9A4	NP_001011552.2	547	5	8.68
OLFML3	NP_064575.1	469	10	20.25	SLC9A2	NP_003039.2	501	0	0.00
SYT6	NP_001353153.1	645	6	8.84	MFSD9	NP_116107.3	87	0	0.00
TRIM33	NP_148980.2	1095	6	5.21	TMEM182	NP_001308272.2	132	1	7.20
BCAS2	NP_005863.1	1034	6	5.51	POU3F3	NP_006227.1	900	0	0.00
DENND2C	NP_940861.3	243	10	39.09	MRPS9	NP_872578.1	958	3	2.97
AMPD1	NP_001166097.1	630	8	12.06	GPR45	NP_009158.3	367	3	7.77
NRAS	NP_002515.1	2686	7	2.48	TGFBRAP1	NP_001315575.1	837	3	3.40
CSDE1	NP_001123995.1	576	8	13.19	C2orf49	XP_016860381.1	298	1	3.19
SIKE1	NP_079349.2	172	6	33.14	FHL2	NP_001361328.1	1284	5	3.70
SYCP1	XP_016857673.1	502	3	5.68	NCK2	XP_016860592.1	689	3	4.14
TSHB	NP_000540.2	473	9	18.08	ECRG4	NP_115787.1	214	1	4.44
TSPAN2	XP_016855485.1	371	6	15.36	UXS1	NP_001364434.1	502	2	3.78
NGF	XP_011539820.1	2619	7	2.54	RGPD3	XP_016860227.1	460	3	6.20
VANGL1	NP_620409.1	593	1	1.60	ST6GAL2	NP_001309291.1	387	2	4.91
CASQ2	NP_001223.2	497	2	3.82	RGPD4	NP_872394.2	327	1	2.91
NHLH2	NP_001104531.1	348	1	2.73	SLC5A7	NP_068587.1	509	2	3.73
SLC22A15	NP_060890.2	341	0	0.00	SULT1C3	XP_016859644.1	159	0	0.00
MAB21L3	NP_689580.2	172	0	0.00	SULT1C2	XP_005264070.1	387	0	0.00
ATP1A1	NP_000692.2	1122	5	4.23	SULT1C4	NP_006579.2	256	1	3.71
CD58	XP_016858358.1	589	7	11.29	GCC2	XP_006712933.1	613	5	7.75
IGSF3	NP_001007238.1	335	4	11.34	LIMS1	NP_001358425.1	643	1	1.48
CD2	NP_001315538.1	1136	6	5.02	RANBP2	XP_005264059.1	2248	4	1.69
PTGFRN	NP_065173.2	281	6	20.28	CCDC138	NP_001338486.1	125	0	0.00
CD101	XP_024306770.1	258	4	14.73	EDAR	XP_011508805.2	672	1	1.41
TTF2	XP_016858039.1	1002	3	2.84	SH3RF3	NP_001092759.1	866	11	12.07
TRIM45	NP_001139107.1	368	0	0.00	SEPTIN10	XP_011509001.1	354	7	18.78
VTCN1	NP_001240779.1	306	2	6.21	SOWAHC	NP_075392.2	86	1	11.05
MAN1A2	XP_006710365.1	458	5	10.37	RGPD5	XP_006712857.2	428	8	17.76
TENT5C	NP_060179.2	383	4	9.92	LIMS3	NP_277049.1	542	9	15.77
GDAP2	NP_060156.1	241	6	23.65	MALL	NP_001358488.1	207	9	41.30
WDR3	NP_006775.1	1045	6	5.45	NPHP1	NP_001121651.1	565	14	23.54
SPAG17	XP_006710490.1	461	5	10.30	LIMS4	XP_016858594.1	542	11	19.28
TBX15	NP_001317606.1	655	6	8.70	RGPD6	NP_001371292.1	421	7	15.79
WARS2	NP_001365158.1	688	4	5.52	BUB1	NP_001265545.1	1597	15	8.92
HAO2	XP_016856910.1	822	5	5.78	ACOXL	XP_011509706.1	452	10	21.02
HSD3B2	NP_001159592.1	537	3	5.31	BCL2L11	XP_005263608.1	1413	3	2.02
HSD3B1	NP_001315544.1	637	3	4.47	ANAPC1	XP_016860200.1	1032	12	11.05
ZNF697	NP_001073939.1	215	4	17.67	MERTK	NP_006334.2	1198	9	7.14
PHGDH	XP_011539528.1	1343	6	4.24	TMEM87B	NP_116213.1	300	11	34.83
HMGCS2	NP_001159579.1	964	11	10.84	FBLN7	XP_016858806.1	341	10	27.86
REG4	NP_114433.1	426	4	8.92	ZC3H8	NP_115883.2	540	10	17.59
ADAM30	NP_068566.2	276	10	34.42	ZC3H6	XP_006712582.1	511	6	11.15
NOTCH2	NP_077719.2	1571	7	4.23	RGPD8	NP_001157935.1	459	5	10.35
SEC22B	NP_004883.3	1014	3	2.81	TTL	NP_001358641.1	239	0	0.00
NBPF8	NP_001032590.2	145	3	19.65	POLR1B	NP_001269701.1	1762	0	0.00
NBPF26	NP_001338301.1	144	7	46.18	CHCHD5	NP_115685.1	106	1	8.96
PPIAL4A	NP_001137355.1	966	1	0.98	SLC20A1	NP_005406.3	692	1	1.37
FAM72B	NP_001307078.1	202	8	37.62	NT5DC4	XP_016860966.1	212	0	0.00
SRGAP2C	NP_001316913.1	498	8	15.26	CKAP2L	NP_689728.3	415	9	20.60
H3-2	NP_001342338.1	3743	2	0.51	IL1A	NP_001358483.1	1155	10	8.22
FAM72C	NP_001332996.1	161	6	35.40	IL1B	NP_000567.1	2695	11	3.88
PPIAL4E	NP_001137504.2	920	0	0.00	IL37	NP_055254.2	366	10	25.95
NBPF15	NP_001372373.1	77	6	74.02	IL36G	NP_062564.1	416	10	22.84

PPIAL4F	NP_001157734.2	926	0	0.00	IL36A	XP_016859295.1	410	8	18.54
SRGAP2B	NP_001372156.1	428	5	11.10	IL36B	NP_055253.2	364	8	20.88
FAM72D	NP_001332871.1	180	4	21.11	IL36RN	NP_775262.1	432	9	19.79
PPIAL4D	NP_001157733.1	896	0	0.00	IL1F10	NP_115945.4	355	10	26.76
NBPF20	NP_001265196.1	6	0	0.00	IL1RN	NP_776213.1	1068	11	9.78
GPR89A	NP_001091081.1	236	11	44.28	PSD4	XP_005263691.1	442	8	17.19
PDZK1	XP_024303387.1	950	9	9.00	PAX8	NP_003457.1	933	5	5.09
CD160	NP_008984.1	285	7	23.33	CBWD2	NP_742000.1	111	4	34.23
RNF115	NP_055270.1	655	13	18.85	FOXD4L1	NP_036316.1	317	0	0.00
POLR3C	NP_001290385.1	698	13	17.69	RABL2A	NP_001341346.1	1640	3	1.74
NUDT17	NP_001012776.1	127	15	112.20	SLC35F5	NP_001317245.1	233	2	8.15
PIAS3	NP_006090.2	798	11	13.09	ACTR3	NP_005712.1	1506	3	1.89
ANKRD35	NP_001267728.1	703	15	20.27	DPP10	NP_065919.3	720	0	0.00
ITGA10	NP_001289970.1	357	12	31.93	DDX18	NP_006764.3	1840	1	0.52
PEX11B	NP_003837.1	411	14	32.36	CCDC93	XP_011509661.1	117	2	16.24
RBM8A	NP_005096.1	1330	10	7.14	INSIG2	NP_001308258.1	655	1	1.45
LIX1L	XP_016855805.1	244	18	70.08	EN1	NP_001417.3	718	2	2.65
ANKRD34A	NP_001034977.1	200	13	61.75	MARCO	NP_006761.1	476	0	0.00
POLR3GL	NP_115681.1	238	17	67.85	C1QL2	NP_872334.2	609	1	1.56
TXNIP	NP_006463.3	1108	8	6.86	STEAP3	NP_878919.2	366	5	12.98
HJV	NP_001366281.1	365	7	18.22	C2orf76	NP_001309261.1	79	2	24.05
NBPF10	NP_001034792.4	121	2	15.70	DBI	NP_001171514.1	654	4	5.81
NOTCH2NLA	NP_001350935.1	866	14	15.36	TMEM37	XP_011508961.1	237	3	12.02
PPIAL4H	NP_001355057.1	919	2	2.07	SCTR	XP_016860160.1	446	7	14.91
NBPF12	NP_001265070.1	267	7	24.91	CFAP221	NP_001257978.2	180	5	26.39
PRKAB2	NP_005390.1	711	15	20.04	TMEM177	NP_001098668.1	111	0	0.00
FMO5	NP_001138301.1	443	13	27.88	PTPN4	NP_002821.1	720	9	11.87
CHD1L	NP_004275.4	1416	16	10.73	EPB41L5	XP_016860056.1	469	7	14.18
BCL9	XP_005273028.1	518	12	22.01	TMEM185B	NP_077026.2	126	5	37.70
ACP6	XP_011507903.1	353	15	40.37	RALB	NP_001356329.1	1571	6	3.63
GJA5	XP_016856533.1	620	11	16.85	INHBB	NP_002184.2	678	8	11.21
GJA8	XP_011507719.1	338	9	25.29	GLI2	NP_001358200.1	1369	6	4.16
GPR89B	NP_001337109.1	224	14	59.37	TFCP2L1	NP_055368.1	346	2	5.49
NBPF11	NP_001372404.1	79	11	132.27	CLASP1	XP_006712446.1	611	0	0.00
PPIAL4G	NP_001116540.1	952	1	1.00	NIFK	NP_115766.3	1095	2	1.74
NBPF14	NP_056198.2	144	7	46.18	TSN	NP_004613.1	660	5	7.20
NOTCH2NLB	XP_024307798.1	866	10	10.97	CNTNAP5	NP_001354427.1	485	2	3.92
NUDT4B	NP_001342336.1	557	0	0.00	GYPC	NP_002092.1	542	1	1.75
PDE4DIP	XP_011508474.1	624	6	9.13	BIN1	NP_647601.1	1291	6	4.41
NBPF9	NP_001032764.2	145	5	32.76	CYP27C1	NP_001354431.1	206	4	18.45
NOTCH2NLC	NP_001350942.1	866	6	6.58	ERCC3	NP_000113.1	1016	6	5.61
NBPF19	NP_001338294.1	79	2	24.05	MAP3K2	NP_001358840.1	1425	4	2.67
PPIAL4C	NP_001129261.2	919	2	2.07	PROC	XP_024308770.1	534	0	0.00
FCGR1A	NP_001365733.1	917	5	5.18	IWS1	XP_005263764.1	781	4	4.87
H2BC18	NP_001154806.1	838	9	10.20	MYO7B	XP_011509520.1	1093	5	4.35
H3C13	NP_001116847.1	1295	9	6.60	LIMS2	XP_006712690.1	694	3	4.11
H4C14	NP_003539.1	2041	11	5.12	GPR17	XP_016859322.1	660	1	1.44
H3C14	NP_066403.2	1326	9	6.45	SFT2D3	NP_116129.3	51	0	0.00
H2AC18	NP_003507.1	1530	7	4.35	WDR33	NP_060853.3	1091	2	1.74
H2AC19	NP_001035807.1	1530	7	4.35	POLR2D	NP_004796.1	1053	3	2.71
H3C15	NP_001005464.1	1346	9	6.35	AMMECR1L	XP_005263864.1	183	2	10.38
H4C15	XP_024307476.1	2041	11	5.12	SAP130	XP_005263824.1	480	5	9.90
H2BC21	NP_003519.1	2158	10	4.40	UGGT1	NP_064505.1	565	2	3.36
H2AC20	NP_003508.1	1774	8	4.28	HS6ST1	NP_004798.3	297	2	6.40
H2AC21	NP_778235.1	938	8	8.10	RAB6C	NP_115520.2	706	1	1.35
BOLA1	NP_001307954.1	321	0	0.00	POTEF	NP_001093241.1	3064	1	0.31
SV2A	NP_001315603.1	1001	0	0.00	CCDC74B	NP_001245236.1	73	0	0.00
SF3B4	NP_005841.1	849	1	1.12	SMPD4	NP_001164554.1	217	1	4.38
MTMR11	NP_870988.2	129	2	14.73	MZT2B	NP_001317213.1	520	3	5.48
OTUD7B	NP_064590.2	524	1	1.81	TUBA3E	NP_997195.2	1359	5	3.50
VPS45	NP_009190.2	653	2	2.91	CCDC115	NP_001308047.1	205	0	0.00
PLEKHO1	NP_057358.2	264	3	10.79	IMP4	NP_001358654.1	904	0	0.00
ANP32E	XP_005245571.1	642	5	7.40	PTPN18	XP_006712480.1	754	0	0.00
CA14	XP_005245117.1	334	0	0.00	POTEI	XP_024308814.1	1499	2	1.27
APH1A	XP_016856906.1	446	1	2.13	CFC1B	XP_005263794.1	224	0	0.00
C1orf54	NP_001287971.1	50	0	0.00	CFC1	NP_001257349.1	367	0	0.00

CIART	NP_653298.1	202	0	0.00	POTEJ	XP_016860230.1	1458	3	1.95
MRPS21	NP_061870.1	395	2	4.81	GPR148	NP_997247.2	220	6	25.91
PRPF3	NP_004689.1	967	0	0.00	AMER3	NP_001098665.1	302	4	12.58
RPRD2	NP_056018.2	400	1	2.37	ARHGEF4	NP_001354422.1	827	9	10.34
TARS2	NP_079426.2	799	1	1.19	FAM168B	NP_001308675.1	124	4	30.64
ECM1	NP_004416.2	462	5	10.28	PLEKHB2	XP_011509687.1	147	4	25.85
ADAMTSL4	XP_011507946.1	382	3	7.46	POTEE	XP_016859648.1	1555	2	1.22
MCL1	NP_877495.1	1552	1	0.61	RAB6D	NP_001071105.1	1053	0	0.00
ENSA	NP_004427.1	313	1	3.03	MZT2A	XP_005263799.1	147	0	0.00
GOLPH3L	XP_006711491.1	362	2	5.25	TUBA3D	NP_525125.2	1391	6	4.10
HORMAD1	NP_001186758.1	497	2	3.82	CCDC74A	XP_016860723.1	40	0	0.00
CTSS	NP_001186668.1	1291	3	2.21	ANKRD30BL	NP_001345345.1	832	1	1.14
CTSK	NP_000387.1	933	3	3.05	ZNF806	NP_001342390.1	142	0	0.00
ARNT	XP_016856785.1	638	2	2.98	GPR39	NP_001499.1	636	2	2.99
SETDB1	NP_001353347.1	2160	3	1.32	LYPD1	NP_001308163.1	345	3	8.26
CERS2	XP_011507753.1	535	8	14.20	NCKAP5	XP_016859468.1	240	2	7.92
ANXA9	NP_003559.2	447	5	10.63	MGAT5	XP_016859636.1	406	2	4.68
MINDY1	XP_016857266.1	347	12	32.85	TMEM163	NP_112185.1	401	7	16.58
PRUNE1	NP_067045.1	286	7	23.25	ACMSD	NP_612199.2	233	10	40.77
BNIPL	NP_612122.2	180	8	42.22	CCNT2	NP_490595.1	791	6	7.21
C1orf56	NP_060330.2	107	6	53.27	MAP3K19	NP_001018054.1	1185	9	7.21
CDC42SE1	XP_016857336.1	188	7	35.37	RAB3GAP1	XP_011509125.1	560	10	16.96
MLLT11	NP_006809.1	354	5	13.42	ZRANB3	XP_011510268.2	1034	8	7.35
GABPB2	NP_001310835.1	926	1	1.03	R3HDM1	NP_001269729.1	605	10	15.70
SEMA6C	XP_016855570.1	635	0	0.00	UBXN4	NP_055422.1	315	8	24.13
TNFAIP8L2	NP_078851.2	453	2	4.19	LCT	NP_002290.2	570	6	10.00
LYSMD1	NP_001130015.1	28	4	135.71	MCM6	NP_005906.2	1643	9	5.20
SCNM1	NP_076946.1	188	7	35.37	DARS1	NP_001340.2	1019	7	6.53
TMOD4	XP_016856579.1	512	3	5.57	CXCR4	NP_003458.1	2403	2	0.79
VPS72	NP_005988.1	591	4	6.43	THSD7B	NP_001303278.1	258	4	14.73
PIP5K1A	XP_006711631.2	854	2	2.22	HNMT	NP_008826.1	525	5	9.05
PSMD4	NP_001317621.1	2007	6	2.84	SPOPL	NP_001001664.1	674	7	9.87
ZNF687	XP_005245423.1	321	1	2.96	NXPH2	NP_009157.1	300	8	25.33
PI4KB	NP_001356554.1	2170	7	3.06	LRP1B	NP_061027.2	1004	7	6.62
RFX5	NP_001366349.1	667	1	1.42	KYNU	XP_024308976.1	622	5	7.64
SELENBP1	NP_001245217.1	587	1	1.62	ARHGAP15	XP_016859989.1	809	7	8.22
PSMB4	NP_002787.2	1349	4	2.82	GTDC1	XP_016860438.1	121	7	54.96
POGZ	XP_024310073.1	954	4	3.98	ZEB2	NP_001165124.1	970	5	4.90
CGN	NP_065821.1	310	2	6.13	ACVR2A	NP_001607.1	1248	8	6.09
TUFT1	NP_064512.1	226	5	21.02	ORC4	NP_001361199.1	586	7	11.35
SNX27	NP_001317652.1	356	1	2.67	MBD5	XP_011509772.1	331	8	22.96
CELF3	NP_001278036.1	1139	1	0.83	EPC2	NP_056445.3	615	7	10.81
RIIAD1	NP_001138428.1	87	1	10.92	KIF5C	XP_016859551.1	1970	11	5.30
MRPL9	NP_001287662.1	728	1	1.30	LYPD6B	XP_011508922.1	218	7	30.50
OAZ3	NP_001128411.1	250	1	3.80	LYPD6	XP_024308466.1	258	6	22.09
TDRKH	NP_001077433.1	343	1	2.77	MMADHC	NP_056517.1	523	6	10.90
LINGO4	NP_001004432.1	407	9	21.01	RND3	NP_001241667.1	1934	4	1.96
RORC	XP_006711547.2	747	0	0.00	RBM43	NP_940959.1	454	1	2.09
C2CD4D	XP_016855478.1	142	1	6.69	NMI	XP_016860736.1	307	0	0.00
THEM5	XP_011507723.1	221	8	34.39	TNFAIP6	NP_009046.2	734	1	1.29
THEM4	NP_444283.2	280	1	3.39	RIF1	XP_005246722.1	378	2	5.03
S100A10	NP_002957.1	707	7	9.41	NEB	XP_011509529.1	622	1	1.53
S100A11	NP_005611.1	558	9	15.32	ARL5A	NP_036229.1	1431	3	1.99
LOC10013110	NP_001297071.1	5	0	0.00	CACNB4	XP_011510099.1	648	5	7.33
TCHHL1	NP_001008536.1	146	14	91.09	STAM2	NP_005834.4	536	1	1.77
TCHH	NP_009044.2	415	14	32.05	FMNL2	XP_011508832.1	511	4	7.44
RPTN	NP_001116437.1	237	14	56.12	PRPF40A	NP_001341360.1	1397	3	2.04
HRNR	NP_001009931.1	464	14	28.66	ARL6IP6	NP_689735.1	106	3	26.89
FLG	NP_002007.1	614	14	21.66	RPRM	NP_062819.1	300	1	3.17
FLG2	XP_011507833.1	296	13	41.72	GALNT13	NP_443149.2	575	7	11.56
CRNN	NP_057274.1	238	15	59.87	KCNJ3	NP_002230.1	552	4	6.88
LCE5A	NP_848525.1	104	8	73.07	NR4A2	XP_006712616.1	967	4	3.93
CRCT1	XP_011507958.1	232	14	57.32	GPD2	XP_016859319.1	1086	3	2.62
LCE3E	NP_848522.1	167	8	45.51	GALNT5	XP_016858726.1	300	2	6.33
LCE3D	NP_115952.1	200	9	42.75	ERMN	NP_001009959.1	802	3	3.55
LCE3C	NP_848521.1	167	7	39.82	CYTIP	NP_004279.3	1097	2	1.73

LCE3B	NP_848520.1	179	7	37.15	ACVR1C	NP_001104503.1	1150	4	3.30
LCE3A	NP_848518.1	161	6	35.40	ACVR1	NP_001334592.1	1378	2	1.38
LCE2D	NP_848517.1	125	4	30.40	UPP2	NP_001128570.1	490	3	5.82
LCE2C	NP_848516.1	106	3	26.89	CCDC148	NP_620158.3	94	3	30.32
LCE2B	NP_055172.1	163	3	17.48	PKP4	NP_001364147.1	873	3	3.26
LCE2A	NP_848515.1	90	1	10.56	DAPL1	NP_001017920.2	176	5	26.99
LCE4A	NP_848133.1	90	4	42.22	TANC1	XP_011510350.1	1086	5	4.37
C1orf68	NP_001019850.1	140	9	61.07	WDSUB1	NP_001317205.1	487	3	5.85
KPRP	NP_001020402.1	378	19	47.75	BAZ2B	XP_024308593.1	1449	8	5.24
LCE1F	NP_848131.1	108	6	52.78	MARCHF7	NP_001363173.1	900	4	4.22
LCE1E	NP_848130.1	90	7	73.89	CD302	NP_001185693.1	670	6	8.51
LCE1D	NP_848129.1	93	8	81.72	LY75-CD302	NP_001185689.1	354	1	2.68
LCE1C	NP_001263260.1	77	7	86.36	LY75	NP_002340.2	669	5	7.10
LCE1B	NP_848126.1	114	9	75.00	PLA2R1	NP_001182570.1	386	4	9.84
LCE1A	NP_848125.1	118	11	88.55	ITGB6	NP_001269283.1	843	7	7.89
LCE6A	XP_016856816.1	62	11	168.54	RBMS1	XP_016860115.1	536	1	1.77
SMCP	NP_109588.2	186	9	45.97	TANK	XP_005246264.2	359	2	5.29
IVL	NP_005538.2	511	19	35.32	PSMD14	NP_005796.1	1882	5	2.52
SPRR4	XP_016855971.1	169	10	56.21	TBR1	NP_006584.1	1091	5	4.35
SPRR1A	NP_001186757.1	334	9	25.60	SLC4A10	NP_001341369.1	921	7	7.22
SPRR3	NP_001091058.1	268	9	31.90	DPP4	NP_001366534.1	1149	5	4.13
SPRR1B	NP_003116.2	309	9	27.67	GCG	NP_002045.1	1836	5	2.59
SPRR2D	NP_001369177.1	155	10	61.29	FAP	NP_001278736.1	647	3	4.40
SPRR2A	NP_005979.1	239	9	35.77	IFIH1	NP_071451.2	1343	6	4.24
SPRR2B	XP_016857663.1	163	8	46.62	GCA	XP_006712461.1	400	5	11.87
SPRR2E	NP_001019380.2	159	7	41.82	KCNH7	XP_016860709.1	458	9	18.67
SPRR2F	NP_001369184.1	98	3	29.08	FIGN	NP_060556.2	885	6	6.44
SPRR2G	XP_016857666.1	178	6	32.02	GRB14	NP_004481.2	583	4	6.52
LELP1	NP_001010857.1	114	13	108.33	COBLL1	NP_001352600.1	397	5	11.96
PRR9	NP_001182500.1	107	0	0.00	SLC38A11	XP_016858946.1	135	4	28.15
LORICRIN	NP_000418.2	480	18	35.62	SCN3A	NP_001075145.1	755	8	10.07
PGLYRP3	NP_443123.1	193	9	44.30	SCN2A	XP_016860146.1	1652	8	4.60
PGLYRP4	XP_011508094.1	119	6	47.90	CSRNP3	XP_005246922.1	373	7	17.83
S100A9	NP_002956.1	863	6	6.60	GALNT3	XP_016859259.1	385	6	14.80
S100A12	NP_005612.1	729	14	18.24	TTC21B	NP_079029.3	351	5	13.53
S100A8	NP_001306127.1	1074	10	8.84	SCN1A	NP_001340889.1	1132	9	7.55
S100A7A	NP_789793.1	129	13	95.73	SCN9A	XP_011509920.1	721	7	9.22
S100A7	NP_002954.2	482	16	31.53	SCN7A	XP_016860156.1	462	6	12.34
S100A6	XP_016857522.1	626	13	19.73	XIRP2	NP_001186072.1	340	3	8.38
S100A5	XP_016857518.1	206	10	46.11	B3GALT1	NP_066191.1	244	2	7.79
S100A4	NP_002952.1	927	12	12.30	STK39	XP_005246522.1	544	1	1.75
S100A3	NP_002951.1	338	12	33.73	CERS6	NP_001243055.1	471	2	4.03
S100A2	NP_005969.2	381	10	24.93	NOSTRIN	NP_001165102.1	217	3	13.13
S100A16	NP_525127.1	243	6	23.46	SPC25	XP_011509818.1	562	0	0.00
S100A14	NP_065723.1	293	11	35.66	G6PC2	NP_066999.1	385	2	4.93
S100A13	XP_016857523.1	536	13	23.04	ABCB11	XP_016860654.1	873	4	4.35
S100A1	NP_006262.1	620	13	19.92	DHRS9	NP_001276692.1	320	1	2.97
CHTOP	NP_001193541.1	661	4	5.75	LRP2	XP_011509485.1	993	0	0.00
SNAPIN	NP_036569.1	483	1	1.97	BBS5	NP_689597.1	364	3	7.83
ILF2	NP_004506.2	1307	6	4.36	KLHL41	NP_006054.2	625	1	1.52
NPR1	NP_000897.3	900	2	2.11	FASTKD1	NP_001308978.1	167	0	0.00
INTS3	NP_001311404.1	397	3	7.18	PPIG	NP_004783.2	1804	3	1.58
SLC27A3	NP_077306.3	277	0	0.00	CCDC173	NP_001078916.1	36	0	0.00
GATAD2B	XP_024304389.1	397	1	2.39	PHOSPHO2	NP_001186214.1	128	1	7.42
DENND4B	NP_001354395.1	197	4	19.29	KLHL23	NP_001186219.1	226	4	16.81
CRTC2	NP_859066.1	472	4	8.05	SSB	NP_003133.1	1244	5	3.82
SLC39A1	NP_055252.2	399	4	9.52	METTL5	NP_001280116.1	247	1	3.85
CREB3L4	XP_024309111.1	379	3	7.52	UBR3	XP_005246356.1	381	5	12.47
JTB	NP_006685.1	200	2	9.50	MYO3B	NP_620482.3	1638	5	2.90
RAB13	NP_002861.1	687	3	4.15	SP5	NP_001003845.1	257	4	14.79
RPS27	NP_001021.1	1235	3	2.31	ERICH2	XP_016859364.1	82	2	23.17
NUP210L	XP_011508424.1	228	3	12.50	GAD1	XP_016859245.1	1722	6	3.31
TPM3	NP_001351608.1	1434	6	3.97	GORASP2	NP_056345.3	597	0	0.00
C1orf189	NP_001010979.1	43	1	22.09	TLK1	XP_011510540.1	604	1	1.57
C1orf43	NP_620077.1	100	6	57.00	METTL8	NP_001308087.1	303	0	0.00
UBAP2L	XP_005245725.1	636	6	8.96	DCAF17	XP_016860484.1	198	1	4.80

HAX1	NP_006109.2	479	3	5.95	CYBRD1	NP_001243838.1	272	0	0.00
AQP10	XP_011508406.1	428	1	2.22	DYNC1I2	NP_001369.1	1023	2	1.86
ATP8B2	NP_001357526.1	560	0	0.00	SLC25A12	NP_003696.2	544	7	12.22
IL6R	XP_016856688.1	969	6	5.88	HAT1	NP_003633.2	1104	7	6.02
SHE	XP_005244948.1	147	2	12.92	METAP1D	NP_954697.1	789	6	7.22
TDRD10	NP_872305.3	77	4	49.35	DLX1	NP_835221.2	838	9	10.20
UBE2Q1	NP_060052.3	525	10	18.09	DLX2	NP_004396.1	774	8	9.82
CHRNB2	NP_000739.1	1140	5	4.17	ITGA6	NP_001073286.1	1102	5	4.31
ADAR	NP_001351974.1	946	6	6.03	PDK1	NP_001265478.1	903	1	1.05
KCNN3	NP_002240.3	594	8	12.79	RAPGEF4	NP_008954.2	1074	9	7.96
PMVK	NP_006547.1	289	2	6.57	MAP3K20	XP_005246697.1	798	0	0.00
PBXIP1	NP_065385.2	220	10	43.18	CDCA7	NP_114148.3	706	6	8.07
PYGO2	NP_612157.1	260	1	3.65	SP3	NP_001166183.1	1027	4	3.70
SHC1	XP_016857571.1	1180	8	6.44	OLA1	NP_001315617.1	1145	5	4.15
CKS1B	NP_001817.1	1117	1	0.85	SP9	NP_001138722.1	355	7	18.73
FLAD1	NP_079483.3	614	0	0.00	CIR1	NP_004873.3	241	0	0.00
LENEP	NP_061125.1	114	0	0.00	SCRN3	NP_001180457.1	267	2	7.12
ZBTB7B	XP_016856888.1	717	3	3.97	GPR155	NP_001253980.1	270	1	3.52
DCST2	NP_653223.2	152	3	18.75	WIPF1	NP_001362764.1	670	0	0.00
DCST1	NP_689707.2	141	7	47.16	CHRNA1	NP_000070.1	793	9	10.78
ADAM15	NP_001248393.1	503	8	15.11	CHN1	NP_001358443.1	1014	0	0.00
EFNA4	NP_872631.1	565	3	5.04	ATF2	NP_001871.2	903	1	1.05
EFNA3	NP_004943.1	608	0	0.00	ATP5MC3	NP_001680.1	809	4	4.70
EFNA1	NP_004419.2	689	2	2.76	LNPK	NP_001291939.1	175	12	65.14
SLC50A1	NP_001274521.1	338	1	2.81	EVX2	NP_001073927.1	362	11	28.87
DPM3	NP_061846.2	308	1	3.08	HOXD13	XP_011509370.1	627	12	18.18
KRTCAP2	NP_776251.2	523	6	10.90	HOXD12	NP_067016.3	364	12	31.32
TRIM46	XP_024305683.1	361	6	15.79	HOXD11	NP_067015.2	453	13	27.26
MUC1	NP_001191219.1	1189	4	3.20	HOXD10	NP_002139.2	540	12	21.11
THBS3	NP_001239537.1	407	10	23.34	HOXD9	NP_055028.3	499	12	22.84
MTX1	NP_002446.3	391	8	19.44	HOXD8	NP_062458.1	369	11	28.32
GBA	NP_001165283.1	1255	9	6.81	HOXD4	NP_055436.2	475	11	22.00
FAM189B	NP_937995.1	80	5	59.37	HOXD3	NP_008829.3	442	12	25.79
SCAMP3	NP_443069.1	363	10	26.17	HOXD1	NP_078777.1	440	11	23.75
CLK2	NP_001350633.1	701	0	0.00	MTX2	NP_006545.1	541	10	17.56
HCN3	NP_065948.1	529	4	7.18	HNRNPA3	NP_001317178.1	1681	5	2.83
PKLR	NP_000289.1	1561	5	3.04	NFE2L2	NP_001300832.1	1020	5	4.66
FDPS	XP_024309834.1	1118	3	2.55	AGPS	NP_003650.1	553	4	6.87
RUSC1	XP_016856380.1	126	1	7.54	TTC30B	NP_689730.2	240	1	3.96
ASH1L	XP_006711515.1	1749	2	1.09	TTC30A	NP_689488.3	147	2	12.92
MSTO1	NP_060586.2	250	1	3.80	PDE11A	NP_001070665.1	552	8	13.77
YY1AP1	NP_620829.1	176	3	16.19	RBM45	XP_016858809.1	300	3	9.50
DAP3	XP_024305465.1	676	1	1.41	OSBPL6	XP_016858755.1	353	8	21.53
GON4L	XP_011507960.1	198	3	14.39	PRKRA	NP_003681.1	461	1	2.06
SYT11	XP_016856248.1	1087	2	1.75	PJVK	XP_005246686.1	151	4	25.16
RIT1	NP_001243750.1	1431	3	1.99	FKBP7	NP_001128684.1	1116	1	0.85
KHDC4	NP_055764.2	147	0	0.00	PLEKHA3	NP_061964.3	215	4	17.67
RXFP4	NP_871001.1	392	6	14.54	TTN	NP_003310.4	1693	1	0.56
ARHGEF2	XP_016858283.1	810	6	7.04	CCDC141	XP_016859357.1	368	1	2.58
SSR2	NP_003136.1	447	9	19.13	SESTD1	XP_016860739.1	361	0	0.00
UBQLN4	XP_024304237.1	2139	8	3.55	ZNF385B	NP_689733.4	385	4	9.87
LAMTOR2	NP_054736.1	642	9	13.32	CWC22	NP_001362961.1	1238	1	0.77
RAB25	NP_065120.2	1862	7	3.57	UBE2E3	NP_872619.1	1819	1	0.52
MEX3A	NP_001087194.1	167	9	51.19	ITGA4	NP_000876.3	1035	3	2.75
LMNA	NP_733821.1	2006	3	1.42	CERKL	NP_001025483.1	641	8	11.86
SEMA4A	XP_011508173.1	684	3	4.17	NEUROD1	NP_002491.3	1313	4	2.89
SLC25A44	NP_001364314.1	112	10	84.82	ITPRID2	NP_006742.2	192	0	0.00
PMF1-BGLAP	NP_001186590.1	296	9	28.88	PPP1R1C	NP_001248353.1	153	2	12.42
PMF1	NP_001186583.1	229	7	29.04	PDE1A	NP_001245241.1	650	5	7.31
BGLAP	NP_954642.1	1060	3	2.69	DNAJC10	NP_061854.1	1952	2	0.97
PAQR6	XP_024305646.1	475	2	4.00	FRZB	NP_001454.2	942	4	4.03
SMG5	NP_001310544.1	634	1	1.50	NCKAP1	XP_006712264.1	807	4	4.71
TMEM79	NP_115699.1	205	5	23.17	DUSP19	NP_543152.1	619	4	6.14
GLMP	NP_001243537.1	161	2	11.80	NUP35	XP_016858797.1	657	2	2.89
VHLL	NP_001004319.1	200	1	4.75	ZNF804A	NP_919226.1	441	3	6.46
CCT3	NP_001008800.1	1885	6	3.02	FSIP2	NP_775922.3	230	2	8.26

TSACC	NP_001291755.1	79	0	0.00	ZC3H15	NP_060941.2	890	1	1.07
RHBG	XP_011508101.1	378	2	5.03	ITGAV	NP_002201.2	1156	5	4.11
MEF2D	XP_006711397.1	1516	4	2.51	FAM171B	NP_803237.3	269	0	0.00
IQGAP3	NP_839943.3	943	3	3.02	ZSWIM2	NP_872327.2	396	0	0.00
TTC24	NP_001099139.2	689	1	1.38	CALCRL	XP_005246289.2	501	2	3.79
NAXE	XP_016855808.1	510	5	9.31	TFPI	NP_001316169.1	740	2	2.57
GPATCH4	NP_056405.2	501	5	9.48	GULP1	NP_001362877.1	459	7	14.49
HAPLN2	XP_016857509.1	1048	6	5.44	COL3A1	NP_000081.2	1226	7	5.42
BCAN	XP_016857536.1	927	5	5.12	COL5A2	NP_000384.2	779	8	9.76
NES	NP_006608.1	1516	4	2.51	WDR75	NP_115544.1	721	10	13.18
CRABP2	NP_001186652.1	498	3	5.72	SLC40A1	NP_055400.1	590	6	9.66
ISG20L2	NP_001357079.1	524	1	1.81	ASNSD1	NP_001340426.1	687	14	19.36
RRNAD1	XP_005245294.1	65	0	0.00	ANKAR	XP_011508978.1	905	16	16.79
MRPL24	NP_078816.2	956	4	3.97	OSGEPL1	NP_001363027.1	553	14	24.05
HDGF	NP_001119522.1	1397	2	1.36	ORMDL1	NP_001358313.1	261	14	50.96
PRCC	XP_005245370.1	543	6	10.50	PMS1	NP_001307974.1	800	11	13.06
SH2D2A	XP_016858253.1	206	6	27.67	MSTN	NP_005250.1	1144	14	11.63
NTRK1	NP_001007793.1	2169	4	1.75	C2orf88	XP_016860585.1	105	14	126.66
INSRR	NP_055030.1	1443	10	6.58	HIBCH	NP_055177.2	770	14	17.27
PEAR1	XP_016856723.1	330	8	23.03	INPP1	XP_024308643.1	478	12	23.85
LRRC71	XP_016855948.1	71	9	120.42	MFSD6	NP_060164.3	286	12	39.86
ARHGEF11	NP_001364347.1	735	9	11.63	NEMP2	XP_016858589.1	82	12	139.02
ETV3L	XP_024302934.1	148	4	25.67	NAB1	NP_001308242.1	290	10	32.76
ETV3	NP_001138784.1	432	4	8.80	GLS	XP_006712498.1	852	4	4.46
FCRL5	NP_001182317.1	261	5	18.20	STAT1	NP_001371813.1	2412	5	1.97
FCRL4	XP_011508336.1	194	4	19.59	STAT4	NP_001230764.1	1294	4	2.94
FCRL3	NP_001307262.1	360	6	15.83	MYO1B	XP_024308682.1	1307	2	1.45
FCRL2	XP_016857808.1	212	7	31.37	NABP1	NP_001026886.1	270	3	10.56
FCRL1	XP_005244926.1	131	2	14.50	CAVIN2	NP_004648.1	455	4	8.35
CD5L	XP_016858295.1	289	7	23.01	TMEFF2	XP_011509192.1	743	4	5.11
KIRREL1	XP_005245362.1	674	12	16.91	SLC39A10	NP_001120729.1	248	7	26.81
CD1D	NP_001358691.1	1013	7	6.56	DNAH7	XP_011509790.1	769	6	7.41
CD1A	NP_001307581.1	553	2	3.44	STK17B	NP_004217.1	866	9	9.87
CD1B	XP_016858274.1	586	2	3.24	HECW2	NP_001335697.1	965	1	0.98
CD1C	XP_005245636.1	879	7	7.57	CCDC150	NP_001074008.1	72	0	0.00
CD1E	NP_112155.2	511	7	13.01	GTF3C3	NP_036218.1	558	9	15.32
OR10T2	NP_001004475.1	65	4	58.46	C2orf66	NP_998773.2	33	0	0.00
OR10K2	NP_001004476.1	46	0	0.00	PGAP1	NP_079265.2	396	9	21.59
OR10K1	NP_001004473.1	81	0	0.00	ANKRD44	XP_005247005.1	1655	11	6.31
OR10R2	NP_001004472.1	62	7	107.25	SF3B1	NP_036565.2	1463	5	3.25
OR6Y1	NP_001372979.1	140	1	6.79	COQ10B	NP_079423.1	315	1	3.02
OR6P1	NP_001153797.1	123	0	0.00	HSPD1	NP_002147.2	2398	6	2.38
OR10X1	NP_001004477.1	71	1	13.38	HSPE1	NP_002148.1	1681	6	3.39
OR10Z1	NP_001004478.1	50	1	19.00	HSPE1-MOB4	NP_001189414.1	1025	2	1.85
SPTA1	XP_011508219.1	985	10	9.64	MOB4	NP_056202.2	450	12	25.33
OR6K2	NP_001005279.1	81	0	0.00	RFTN2	XP_016858821.1	126	12	90.47
OR6K3	NP_001005327.2	96	1	9.90	MARS2	NP_612404.1	701	13	17.62
OR6K6	NP_001005184.1	52	6	109.61	BOLL	NP_001271290.1	1259	12	9.05
OR6N1	XP_016855814.1	44	0	0.00	PLCL1	NP_006217.3	604	9	14.15
OR6N2	NP_001005278.1	51	0	0.00	SATB2	NP_001165980.1	687	10	13.83
MNDA	NP_002423.1	804	6	7.09	FTCDNL1	XP_024308633.1	137	15	104.01
PYHIN1	XP_011507545.1	240	4	15.83	C2orf69	NP_710156.3	235	8	32.34
IFI16	XP_016856639.1	511	6	11.15	TYW5	NP_001034782.1	447	16	34.00
AIM2	XP_016858337.1	416	6	13.70	MAIP1	NP_078796.2	153	8	49.67
CADM3	XP_024304528.1	983	10	9.66	SPATS2L	NP_001269673.1	197	18	86.80
ACKR1	NP_002027.2	732	7	9.08	KCTD18	NP_001308476.1	198	8	38.38
FCER1A	NP_001992.1	638	11	16.38	SGO2	XP_016858941.1	450	9	19.00
OR10J3	NP_001004467.1	105	2	18.09	AOX1	XP_011509364.1	854	11	12.24
OR10J1	XP_016856504.1	68	4	55.88	BZW1	NP_001193998.1	761	15	18.72
OR10J5	NP_001004469.1	119	3	23.95	CLK1	NP_001155879.1	815	6	6.99
APCS	NP_001630.1	597	7	11.14	PPIL3	XP_016859844.1	1029	14	12.92
CRP	NP_001369632.1	1652	4	2.30	NIF3L1	NP_001356371.1	467	16	32.55
DUSP23	NP_060293.2	772	0	0.00	ORC2	NP_006181.1	1101	1	0.86
FCRL6	XP_005245185.1	262	7	25.38	FAM126B	NP_001308548.1	112	14	118.74
SLAMF8	XP_011508077.1	406	6	14.04	NDUFB3	XP_016859675.1	588	9	14.54
VSIG8	NP_001013683.1	215	11	48.60	CFLAR	XP_016860679.1	1173	7	5.67

CFAP45	NP_036469.2	188	13	65.69	CASP10	NP_116756.2	591	4	6.43
TAGLN2	NP_001264153.1	705	13	17.52	CASP8	NP_001073594.1	2228	7	2.98
IGSF9	NP_065840.2	299	15	47.66	FLACC1	NP_001120863.1	115	13	107.39
SLAMF9	XP_016858245.1	179	13	68.99	TRAK2	NP_055864.2	363	9	23.55
PIGM	NP_660150.1	379	11	27.57	STRADB	NP_061041.2	323	10	29.41
KCNJ10	NP_002232.2	1000	12	11.40	C2CD6	XP_024308496.1	103	8	73.78
KCNJ9	NP_004974.2	1242	12	9.18	TMEM237	NP_001037850.1	181	6	31.49
IGSF8	XP_024306713.1	388	13	31.83	MPP4	XP_016860109.1	503	1	1.89
ATP1A2	NP_000693.1	1517	15	9.39	ALS2	XP_016860058.1	986	7	6.74
ATP1A4	NP_653300.2	900	14	14.78	CDK15	NP_001353315.1	1728	1	0.55
CASQ1	NP_001222.3	413	16	36.80	FZD7	NP_003498.1	785	1	1.21
PEA15	NP_001284505.1	667	15	21.36	KIAA2012	NP_001264301.2	391	0	0.00
DCAF8	NP_056541.2	254	13	48.62	SUMO1	NP_001005782.1	3059	2	0.62
PEX19	NP_002848.1	561	11	18.63	NOP58	NP_057018.1	1657	3	1.72
COPA	NP_004362.2	658	1	1.44	BMPR2	NP_001195.2	1423	3	2.00
NCSTN	NP_056146.1	651	8	11.67	FAM117B	NP_775782.2	188	2	10.11
NHLH1	NP_005589.1	501	6	11.38	ICA1L	NP_612477.3	312	3	9.13
VANGL2	XP_005245414.1	810	7	8.21	WDR12	NP_060726.3	1456	8	5.22
SLAMF6	XP_016855706.1	541	6	10.54	CARF	NP_001309358.1	144	4	26.39
CD84	NP_001171811.1	498	9	17.17	NBEAL1	NP_001364955.1	249	2	7.63
SLAMF1	XP_005245513.1	1024	9	8.35	CYP20A1	NP_001358624.1	349	4	10.89
CD48	XP_016858356.1	917	8	8.29	ABI2	NP_001362641.1	711	3	4.01
SLAMF7	XP_024304525.1	529	8	14.37	RAPH1	NP_998754.1	398	1	2.39
LY9	NP_002339.2	479	11	21.82	CD28	XP_011510496.1	1482	2	1.28
CD244	XP_011507925.1	796	8	9.55	CTLA4	NP_005205.2	1879	3	1.52
ITLN1	NP_060095.2	302	2	6.29	ICOS	NP_036224.1	397	2	4.79
ITLN2	XP_024309089.1	148	2	12.84	PARD3B	NP_001289698.1	910	1	1.04
F11R	NP_001369663.1	568	4	6.69	NRP2	NP_957718.1	362	1	2.62
TSTD1	NP_001106677.1	391	2	4.86	INO80D	XP_011509671.1	299	2	6.35
USF1	NP_001263302.1	984	4	3.86	NDUFS1	NP_001186913.1	1004	3	2.84
ARHGAP30	NP_001274531.1	505	8	15.05	EEF1B2	NP_066944.1	1166	1	0.81
NECTIN4	XP_005245565.1	279	4	13.62	GPR1	NP_001091669.1	390	1	2.44
KLHDC9	NP_689579.3	478	4	7.95	ZDBF2	NP_065974.1	152	3	18.75
PFDN2	NP_036526.2	980	7	6.79	ADAM23	XP_005246989.1	538	4	7.06
NIT1	XP_005245272.1	460	7	14.46	DYTN	NP_001087199.1	411	5	11.56
DEDD	XP_016858291.1	367	7	18.12	MDH1B	XP_005246373.1	719	2	2.64
UFC1	XP_016856939.1	357	9	23.95	FASTKD2	NP_001129665.1	311	8	24.44
USP21	XP_016856525.1	537	2	3.54	CPO	XP_016858861.1	188	2	10.11
PPOX	XP_011507965.1	622	5	7.64	KLF7	NP_001257872.1	579	8	13.13
B4GALT3	XP_024306308.1	327	9	26.15	CREB1	NP_001358355.1	2938	4	1.29
ADAMTS4	NP_001307265.1	546	2	3.48	METTL21A	XP_011509029.1	201	10	47.26
NDUFS2	NP_001364227.1	878	9	9.74	CCNYL1	NP_001317147.1	227	9	37.66
FCER1G	NP_004097.1	862	5	5.51	FZD5	XP_024308898.1	703	7	9.46
APOA2	NP_001634.1	613	2	3.10	PLEKHM3	XP_016859562.1	198	11	52.78
TOMM40L	XP_016857970.1	795	6	7.17	CRYGD	NP_008822.2	233	3	12.23
NR1I3	NP_001070943.1	454	2	4.18	CRYGC	NP_066269.1	137	0	0.00
PCP4L1	NP_001096036.1	354	6	16.10	CRYGB	NP_005201.2	183	3	15.57
MPZ	XP_016856810.1	368	2	5.16	CRYGA	NP_055432.2	127	2	14.96
SDHC	NP_001030588.1	2066	7	3.22	C2orf80	XP_016859566.1	64	0	0.00
CFAP126	NP_001013647.2	105	2	18.09	IDH1	NP_005887.2	1670	11	6.26
FCGR2A	XP_016856153.1	1154	8	6.59	PIKFYVE	XP_011509080.1	1271	5	3.74
HSPA6	NP_002146.2	1947	11	5.37	PTH2R	NP_005039.1	348	4	10.92
FCGR3A	NP_001121064.2	808	7	8.23	MAP2	NP_001362466.1	1470	6	3.88
FCGR3B	NP_001257964.2	633	6	9.00	UNC80	XP_011509306.1	499	4	7.61
FCGR2B	NP_003992.3	1070	6	5.33	RPE	NP_001305855.1	751	2	2.53
FCRLA	XP_011508367.1	369	6	15.45	KANSL1L	XP_005246389.1	90	5	52.78
FCRLB	NP_001002901.1	158	3	18.04	ACADL	NP_001599.1	602	4	6.31
DUSP12	NP_009171.1	1662	3	1.71	MYL1	NP_524144.1	1364	7	4.88
ATF6	XP_011507610.1	997	5	4.76	LANCL1	XP_005246300.1	290	2	6.55
OLFML2B	NP_056256.1	404	8	18.81	CPS1	NP_001356185.1	1117	4	3.40
NOS1AP	NP_055512.1	660	7	10.08	ERBB4	NP_001036064.1	2057	6	2.77
SPATA46	XP_005245160.1	44	2	43.18	IKZF2	XP_011509121.1	446	3	6.39
C1orf226	NP_001128712.1	83	2	22.89	SPAG16	NP_078808.3	471	4	8.07
SH2D1B	NP_444512.2	561	3	5.08	VWC2L	NP_001073969.1	532	6	10.71
UHMK1	NP_001171692.1	742	4	5.12	BARD1	NP_001269477.1	1923	7	3.46
UAP1	NP_001311045.1	782	3	3.64	ABCA12	NP_775099.2	489	3	5.83

DDR2	NP_001341911.1	524	3	5.44	ATIC	XP_016859676.1	1409	3	2.02
HSD17B7	NP_057455.1	604	2	3.15	FN1	NP_997639.2	3984	9	2.15
CCDC190	XP_005245182.1	42	0	0.00	MREG	NP_060470.2	230	2	8.26
RGS4	NP_001095915.1	1143	2	1.66	PECR	NP_060911.2	537	3	5.31
RGS5	NP_003608.1	618	0	0.00	TMEM169	NP_612399.1	209	3	13.64
NUF2	NP_113611.2	947	1	1.00	XRCC5	NP_066964.1	1497	5	3.17
PBX1	XP_011507892.1	1009	5	4.71	MARCHF4	NP_065865.1	491	1	1.93
LMX1A	NP_796372.1	802	2	2.37	SMARCAL1	NP_001120679.1	1060	2	1.79
RXRG	NP_008848.1	732	4	5.19	RPL37A	NP_000989.1	1136	2	1.67
LRRC52	NP_001005214.2	543	6	10.50	IGFBP2	NP_000588.3	825	5	5.76
MGST3	NP_004519.1	416	4	9.13	IGFBP5	NP_000590.1	941	5	5.05
ALDH9A1	NP_000687.3	898	5	5.29	TNP1	NP_003275.1	499	4	7.61
TMCO1	NP_001243093.1	435	7	15.29	TNS1	XP_024308851.1	2171	5	2.19
UCK2	NP_036606.2	1174	7	5.66	RUFY4	XP_016859384.1	289	10	32.87
FAM78B	NP_001307231.1	96	7	69.27	CXCR2	XP_016859479.1	1012	10	9.39
POGK	XP_016857403.1	242	8	31.40	CXCR1	NP_000625.1	877	8	8.67
TADA1	NP_444281.1	277	7	24.01	ARPC2	NP_690601.1	928	9	9.21
ILDR2	NP_955383.1	211	6	27.01	GPBAR1	NP_001308879.1	415	10	22.89
MAEL	XP_006711646.1	417	1	2.28	AAMP	NP_001078.2	515	9	16.60
GPA33	NP_005805.1	261	1	3.64	PNKD	NP_056303.3	517	10	18.37
STYXL2	NP_001073895.1	721	1	1.32	TMBIM1	NP_001308359.1	467	15	30.51
POU2F1	XP_011507955.1	974	2	1.95	CATIP	NP_001307794.1	142	7	46.83
CD247	NP_000725.1	787	4	4.83	SLC11A1	NP_000569.3	1280	18	13.36
CREG1	NP_003842.1	416	2	4.57	CTDSP1	NP_001193807.1	552	11	18.93
RCSD1	NP_443094.3	382	3	7.46	VIL1	NP_009058.2	660	12	17.27
MPZL1	NP_003944.1	371	4	10.24	USP37	XP_005246781.1	559	10	16.99
ADCY10	XP_011508064.1	1871	2	1.02	CNOT9	NP_001258563.1	502	9	17.03
MPC2	NP_001137146.1	458	5	10.37	PLCD4	XP_016860603.1	583	9	14.66
DCAF6	NP_001185885.1	514	3	5.54	ZNF142	NP_001353220.1	290	9	29.48
GPR161	NP_001254543.1	447	6	12.75	BCS1L	NP_001307646.1	1143	3	2.49
TIPRL	NP_690866.1	433	4	8.78	RNF25	NP_071898.2	513	3	5.56
SFT2D2	NP_955376.1	316	3	9.02	STK36	NP_001356352.1	385	13	32.08
TBX19	NP_005140.1	400	2	4.75	TTLL4	XP_016860876.1	256	2	7.42
XCL2	NP_003166.1	397	2	4.79	CYP27A1	NP_000775.1	748	1	1.27
XCL1	NP_002986.1	534	4	7.12	PRKAG3	XP_016859832.1	1258	13	9.82
DPT	NP_001928.2	557	1	1.71	WNT6	NP_006513.1	804	4	4.73
ATP1B1	NP_001668.1	837	9	10.21	WNT10A	XP_011510231.1	801	5	5.93
NME7	NP_037462.1	717	7	9.27	CDK5R2	NP_003927.1	1078	7	6.17
BLZF1	NP_001307902.1	344	7	19.33	FEV	NP_059991.1	342	3	8.33
CCDC181	XP_016857429.1	145	8	52.41	CRYBA2	NP_476434.1	245	4	15.51
SLC19A2	NP_001306596.1	474	7	14.03	CFAP65	XP_016859242.1	175	7	38.00
F5	NP_000121.2	628	5	7.56	IHH	NP_002172.2	1196	6	4.77
SELP	XP_005245497.1	1233	6	4.62	NHEJ1	NP_079058.1	327	3	8.72
SELL	NP_000646.3	1581	8	4.81	SLC23A3	NP_653313.3	250	3	11.40
SELE	NP_000441.2	1290	5	3.68	CNPPD1	XP_024308558.1	580	4	6.55
METTL18	NP_219486.1	203	8	37.44	RETREG2	NP_077269.3	128	3	22.26
C1orf112	NP_001353698.1	277	10	34.29	ZFAND2B	XP_006712348.1	347	0	0.00
SCYL3	XP_016857351.1	230	5	20.65	ABCB6	NP_001336757.1	634	2	3.00
KIFAP3	XP_024309954.1	745	9	11.48	ATG9A	NP_001070666.1	751	4	5.06
METTL11B	NP_001129579.1	195	5	24.36	ANKZF1	NP_060559.2	349	2	5.44
GORAB	XP_011508451.1	238	4	15.97	GLB1L	XP_016860384.1	163	4	23.31
PRRX1	NP_073207.1	996	3	2.86	STK16	NP_001317142.1	426	0	0.00
MROH9	NP_001157101.1	107	0	0.00	TUBA4A	NP_005991.1	1699	1	0.56
FMO3	NP_001002294.1	524	0	0.00	DNAJB2	NP_001034639.1	781	3	3.65
FMO2	NP_001451.2	501	1	1.90	PTPRN	XP_016860101.1	1311	5	3.62
FMO1	NP_001269621.1	363	0	0.00	RESP18	XP_024308649.1	720	1	1.32
FMO4	NP_002013.1	385	0	0.00	DNPEP	NP_001306046.1	455	4	8.35
PRRC2C	XP_005245072.1	400	6	14.25	DES	NP_001369641.1	564	4	6.74
MYOC	NP_000252.1	694	7	9.58	SPEG	XP_011508785.2	1149	11	9.09
VAMP4	NP_001172056.1	1019	7	6.53	SPEGNB	NP_001273740.1	1149	11	9.09
EEF1AKNMT	NP_057019.3	275	6	20.73	GMPPA	NP_001361223.1	431	7	15.43
DNM3	XP_016856468.1	1944	9	4.40	ASIC4	NP_061144.4	344	6	16.57
C1orf105	XP_011508454.1	13	3	219.22	CHPF	NP_078812.3	305	7	21.80
PIGC	NP_714969.1	426	7	15.61	TMEM198	XP_016858856.1	124	1	7.66
SUCO	NP_057311.3	364	8	20.88	OBSL1	XP_016859189.1	581	5	8.18
FASLG	NP_000630.1	1444	2	1.32	INHA	NP_002182.1	657	10	14.46

TNFSF18	NP_005083.3	266	2	7.14	STK11IP	NP_443134.3	288	0	0.00
TNFSF4	XP_016857718.1	620	3	4.60	SLC4A3	NP_001313488.2	519	9	16.47
PRDX6	NP_004896.1	1272	3	2.24	EPHA4	NP_004429.1	1630	5	2.91
SLC9C2	NP_848622.2	1414	4	2.69	PAX3	NP_852124.1	1394	3	2.04
ANKRD45	XP_016856612.1	1426	7	4.66	SGPP2	NP_689599.2	287	2	6.62
KLHL20	XP_016856541.1	545	0	0.00	FARSB	NP_005678.3	954	6	5.97
CENPL	NP_001164653.1	326	3	8.74	MOGAT1	NP_477513.2	438	2	4.34
DARS2	NP_060592.2	788	5	6.03	ACSL3	NP_001341087.1	1107	2	1.72
ZBTB37	XP_016858045.1	159	4	23.90	KCNE4	NP_542402.4	194	3	14.69
SERPINC1	NP_000479.1	1026	2	1.85	SCG2	NP_003460.2	866	0	0.00
RC3H1	XP_005244978.1	417	3	6.83	AP1S3	XP_011508902.1	359	2	5.29
RABGAP1L	NP_001353375.1	519	4	7.32	WDFY1	NP_065881.1	715	0	0.00
GPR52	NP_005675.3	266	1	3.57	MRPL44	XP_011509970.1	1547	1	0.61
CACYBP	NP_055227.1	790	5	6.01	SERPINE2	NP_001130002.1	905	2	2.10
MRPS14	NP_071383.1	1035	1	0.92	FAM124B	NP_001116251.1	138	2	13.77
TNN	XP_016857537.1	498	2	3.82	CUL3	NP_001244126.1	2022	1	0.47
KIAA0040	XP_016858400.1	141	4	26.95	DOCK10	NP_055504.2	422	1	2.25
TNR	XP_011508251.1	837	3	3.40	NYAP2	NP_001358202.1	156	4	24.36
COP1	NP_001001740.1	952	7	6.98	IRS1	NP_005535.1	1650	3	1.73
PAPPA2	XP_016857512.1	250	3	11.40	RHBDD1	XP_016860572.1	322	4	11.80
ASTN1	NP_001273093.1	1034	9	8.27	COL4A4	XP_011508867.1	512	4	7.42
BRINP2	XP_024304490.1	867	5	5.48	COL4A3	NP_000082.2	516	1	1.84
SEC16B	NP_149118.2	453	4	8.39	MFF	NP_064579.3	378	0	0.00
RASAL2	XP_011508469.1	751	11	13.91	TM4SF20	NP_079071.2	162	0	0.00
TEX35	NP_001164193.1	121	1	7.85	AGFG1	NP_001128659.1	1218	4	3.12
RALGPS2	NP_689876.2	661	5	7.19	C2orf83	NP_064546.3	72	0	0.00
ANGPTL1	NP_001363692.1	442	4	8.60	SLC19A3	XP_016860520.1	341	1	2.79
FAM20B	XP_016858490.1	204	6	27.94	CCL20	NP_004582.1	1017	0	0.00
TOR3A	NP_071766.2	285	13	43.33	DAW1	NP_849143.1	600	0	0.00
ABL2	NP_001161710.1	1467	6	3.89	SPHKAP	NP_085126.2	938	1	1.01
SOAT1	NP_003092.4	719	9	11.89	PID1	NP_001317086.1	311	1	3.05
AXDND1	XP_011507471.1	61	10	155.73	DNER	XP_005247007.1	881	4	4.31
NPHS2	XP_005245540.1	604	10	15.73	TRIP12	NP_001271145.1	1953	6	2.92
TDRD5	NP_001186014.1	864	9	9.90	FBXO36	NP_777559.3	167	2	11.38
FAM163A	XP_016855875.1	62	4	61.29	SLC16A14	XP_005246410.1	244	6	23.36
TOR1AIP2	NP_001336863.1	194	9	44.07	SP110	XP_024308618.1	474	2	4.01
TOR1AIP1	NP_001254507.1	257	9	33.27	SP140	XP_006712286.1	601	3	4.74
CEP350	XP_011508493.1	701	9	12.20	SP140L	XP_016860783.1	345	2	5.51
QSOX1	NP_001004128.1	534	9	16.01	SP100	NP_001073860.1	921	2	2.06
LHX4	NP_203129.1	472	4	8.05	CAB39	NP_001124321.1	662	5	7.17
ACBD6	NP_115736.1	296	7	22.47	ITM2C	NP_112188.1	286	2	6.64
XPR1	NP_004727.2	391	3	7.29	GPR55	NP_005674.2	507	4	7.49
KIAA1614	NP_066001.1	419	4	9.07	SPATA3	XP_016858852.1	89	3	32.02
STX6	NP_005810.1	820	7	8.11	C2orf72	NP_001138466.1	20	3	142.49
MR1	NP_001372090.1	146	1	6.51	PSMD1	NP_002798.2	1471	5	3.23
IER5	NP_057629.2	279	5	17.02	HTR2B	XP_006712545.1	483	2	3.93
CACNA1E	XP_016857733.1	1094	9	7.81	ARMC9	XP_011510214.1	193	2	9.84
ZNF648	XP_024309026.1	189	8	40.21	B3GNT7	NP_660279.1	249	1	3.82
GLUL	NP_002056.2	1955	4	1.94	NCL	NP_005372.2	1848	3	1.54
TEDDM1	NP_741997.3	93	1	10.21	NMUR1	XP_006712259.1	573	2	3.32
RGSL1	NP_001353863.1	148	4	25.67	TEX44	NP_689827.2	23	0	0.00
RNASEL	NP_066956.1	4158	13	2.97	PTMA	NP_001092755.1	815	1	1.17
RGS16	XP_024304564.1	430	3	6.63	PDE6D	NP_002592.1	494	1	1.92
RGS8	XP_016858120.1	1037	8	7.33	COPS7B	NP_001269878.1	421	1	2.26
NPL	NP_001186985.1	1339	4	2.84	NPPC	NP_077720.1	296	1	3.21
DHX9	NP_001348.2	1860	7	3.58	DIS3L2	NP_001244210.1	1059	2	1.79
SHCBP1L	NP_112195.2	50	0	0.00	ALPP	NP_001623.3	891	2	2.13
LAMC1	NP_002284.3	797	2	2.38	ALPG	NP_112603.2	786	2	2.42
LAMC2	NP_005553.2	556	4	6.83	ALPI	NP_001622.2	913	3	3.12
NMNAT2	NP_055854.1	903	6	6.31	ECEL1	NP_001277716.1	271	5	17.53
SMG7	NP_963863.2	597	4	6.36	PRSS56	NP_001356777.1	224	3	12.72
NCF2	NP_001177718.1	1222	8	6.22	CHRND	NP_000742.1	604	6	9.44
ARPC5	NP_005708.1	680	0	0.00	CHRNG	NP_005190.4	619	7	10.74
APOBEC4	NP_982279.1	98	2	19.39	TIGD1	NP_663748.1	147	4	25.85
RGL1	XP_011507641.1	340	1	2.79	EIF4E2	NP_001263265.1	1018	11	10.26
COLGALT2	NP_001290349.1	356	1	2.67	EFHD1	NP_001230181.1	472	2	4.03

TSEN15	NP_001350572.1	187	1	5.08	GIGYF2	NP_001096617.1	839	3	3.40
C1orf21	NP_110433.1	70	0	0.00	KCNJ13	NP_002233.2	293	2	6.48
EDEM3	NP_079467.3	323	0	0.00	SNORC	NP_001333049.1	63	0	0.00
NIBAN1	NP_443198.1	230	1	4.13	NGEF	NP_062824.2	1051	6	5.42
RNF2	NP_009143.1	982	3	2.90	NEU2	NP_005374.2	270	6	21.11
TRMT1L	NP_112196.3	843	3	3.38	INPP5D	NP_001017915.1	1106	6	5.15
SWT1	XP_024303520.1	303	5	15.68	ATG16L1	NP_001350671.1	723	6	7.88
IVNS1ABP	NP_006460.2	499	5	9.52	SAG	NP_000532.2	1460	6	3.90
HMCN1	NP_114141.2	789	2	2.41	DGKD	NP_690618.2	320	5	14.84
PRG4	XP_024304475.1	298	0	0.00	USP40	XP_006712675.1	536	8	14.18
TPR	NP_003283.2	1684	4	2.26	UGT1A8	NP_061949.3	587	1	1.62
ODR4	NP_060317.3	149	1	6.38	UGT1A10	NP_061948.1	332	1	2.86
PDC	NP_002588.3	380	0	0.00	UGT1A9	NP_066307.1	362	0	0.00
PTGS2	NP_000954.1	2250	4	1.69	UGT1A7	NP_061950.2	291	0	0.00
PLA2G4A	NP_077734.2	1059	3	2.69	UGT1A6	NP_001063.2	527	0	0.00
BRINP3	XP_016856614.1	430	7	15.46	UGT1A5	NP_061951.1	352	0	0.00
RGS18	NP_570138.1	873	7	7.62	UGT1A4	NP_009051.1	370	0	0.00
RGS21	NP_001034241.1	292	0	0.00	UGT1A3	NP_061966.1	368	0	0.00
RGS1	NP_002913.3	935	3	3.05	UGT1A1	NP_000454.1	527	0	0.00
RGS13	NP_658912.1	409	1	2.32	MROH2A	XP_011509378.1	47	7	141.48
RGS2	NP_002914.1	843	5	5.63	HJURP	NP_001269892.1	575	6	9.91
UCHL5	NP_001337773.1	1269	7	5.24	TRPM8	XP_011510112.1	484	3	5.89
RO60	NP_001035828.1	417	6	13.67	SPP2	XP_005246159.1	371	4	10.24
GLRX2	NP_057150.2	632	6	9.02	ARL4C	NP_001269360.1	1459	8	5.21
CDC73	NP_078805.3	1232	7	5.40	SH3BP4	NP_001358235.1	303	2	6.27
B3GALT2	NP_003774.1	269	6	21.19	AGAP1	XP_006712297.1	603	9	14.18
KCNT2	NP_001274749.1	350	3	8.14	GBX2	NP_001476.2	682	3	4.18
CFH	NP_000177.2	934	8	8.14	ASB18	NP_997721.2	1195	6	4.77
CFHR3	NP_066303.2	185	5	25.67	IQCA1	NP_001257513.1	536	4	7.09
CFHR1	NP_002104.2	268	4	14.18	ACKR3	XP_016860005.1	707	2	2.69
CFHR4	NP_001188479.1	109	7	61.01	COPS8	NP_006701.1	613	3	4.65
CFHR2	NP_005657.1	121	5	39.25	COL6A3	XP_016858793.1	741	1	1.28
CFHR5	XP_011508322.1	153	5	31.04	MLPH	NP_077006.1	318	4	11.95
F13B	NP_001985.2	450	9	19.00	PRLH	NP_056977.1	233	3	12.23
ASPM	NP_001193775.1	1957	1	0.49	RAB17	XP_006712752.1	807	6	7.06
ZBTB41	NP_919290.2	265	11	39.43	LRRFIP1	XP_016860742.1	338	5	14.05
CRB1	XP_016856341.1	1071	7	6.21	RBM44	XP_016859543.1	86	2	22.09
DENND1B	XP_011507550.1	418	3	6.82	RAMP1	NP_005846.1	298	4	12.75
C1orf53	NP_001019765.1	23	0	0.00	UBE2F	NP_001265235.1	509	3	5.60
LHX9	NP_001014434.1	859	6	6.64	SCLY	NP_057594.5	579	4	6.56
NEK7	XP_016855833.1	570	8	13.33	ESPNL	NP_919288.2	909	3	3.14
ATP6V1G3	NP_001363790.1	325	1	2.92	KLHL30	NP_940984.3	272	0	0.00
PTPRC	NP_002829.3	2808	1	0.34	ERFE	NP_001278761.1	137	0	0.00
NR5A2	NP_995582.1	891	4	4.26	ILKAP	NP_110395.1	1155	1	0.82
ZNF281	NP_001268223.1	385	3	7.40	HES6	NP_001136325.1	524	2	3.63
KIF14	XP_011508537.1	1187	8	6.40	PER2	NP_073728.1	748	2	2.54
DDX59	NP_001336731.1	1252	3	2.28	TRAF3IP1	XP_011509246.1	399	4	9.52
CAMSAP2	NP_001284636.1	375	3	7.60	ASB1	NP_001035535.1	1188	5	4.00
GPR25	NP_005289.2	294	3	9.69	TWIST2	NP_476527.1	742	0	0.00
INAVA	XP_011508058.1	266	4	14.28	HDAC4	XP_011510528.1	2619	6	2.18
KIF21B	NP_001239032.1	973	4	3.91	NDUFA10	NP_004535.1	627	0	0.00
CACNA1S	XP_005245535.1	946	6	6.03	OR6B2	NP_001005853.1	68	0	0.00
ASCL5	NP_001257530.1	67	0	0.00	OR6B3	NP_775486.1	59	0	0.00
TMEM9	NP_001275494.1	165	1	5.76	OTOS	XP_016858898.1	238	0	0.00
IGFN1	NP_001158058.1	501	2	3.79	GPC1	NP_002072.2	839	0	0.00
PKP1	NP_000290.2	676	2	2.81	ANKMY1	XP_024308723.1	113	3	25.22
TNNT2	XP_011508246.1	868	4	4.38	DUSP28	NP_001357394.1	557	4	6.82
LAD1	NP_005549.2	338	3	8.43	RNPEPL1	NP_060696.4	510	7	13.04
TNNI1	NP_003272.3	550	3	5.18	CAPN10	NP_075571.2	683	4	5.56
PHLDA3	NP_036528.1	264	0	0.00	GPR35	NP_001182310.1	301	4	12.62
CSRP1	NP_001180499.1	826	7	8.05	AQP12B	XP_011509974.1	100	0	0.00
NAV1	NP_065176.3	352	3	8.10	AQP12A	NP_945349.1	122	1	7.79
IPO9	NP_060555.2	499	4	7.61	KIF1A	NP_001366569.1	1963	6	2.90
SHISA4	NP_937792.2	170	1	5.59	AGXT	NP_000021.1	1099	0	0.00
LMOD1	NP_036266.2	615	4	6.18	MAB21L4	NP_001078906.3	132	8	57.57
TIMM17A	NP_006326.1	575	4	6.61	CROCC2	NP_001338234.1	39	0	0.00

RNPEP	NP_064601.3	708	4	5.37	SNED1	XP_011509233.1	462	10	20.56
ELF3	NP_001107781.1	602	5	7.89	MTERF4	NP_001317109.1	255	1	3.73
GPR37L1	XP_011508460.1	684	3	4.17	PASK	NP_001239051.1	3199	2	0.59
ARL8A	NP_620150.1	1407	7	4.73	PPP1R7	NP_001269341.1	882	7	7.54
PTPN7	NP_001186726.1	819	1	1.16	ANO7	XP_016859718.1	201	7	33.08
LGR6	NP_001017403.1	758	3	3.76	HDLBP	NP_001307896.1	990	4	3.84
UBE2T	NP_054895.1	1651	3	1.73	SEPTIN2	XP_024308688.1	1055	8	7.20
PPP1R12B	NP_001317958.1	1101	4	3.45	FARP2	XP_024309027.1	366	9	23.36
SYT2	XP_016855799.1	1136	3	2.51	STK25	NP_001258908.1	807	10	11.77
KDM5B	NP_001334520.1	2206	3	1.29	BOK	NP_115904.1	475	1	2.00
RABIF	NP_002862.2	290	2	6.55	THAP4	XP_016859745.1	103	10	92.23
KLHL12	NP_001289980.1	380	3	7.50	ATG4B	XP_005247050.2	836	3	3.41
ADIPOR1	NP_057083.2	518	1	1.83	DTYMK	NP_001307831.1	1125	10	8.44
CYB5R1	NP_057327.2	516	2	3.68	ING5	XP_016860590.1	565	2	3.36
TMEM183A	NP_612400.3	132	0	0.00	D2HGDH	XP_011510036.1	317	2	5.99
PPFIA4	XP_006711649.1	619	6	9.21	GAL3ST2	NP_071417.2	51	2	37.25
MYOG	NP_002470.2	1235	2	1.54	NEU4	NP_001161071.1	191	2	9.95
ADORA1	NP_001041695.1	1189	2	1.60	PDCD1	XP_016859782.1	785	0	0.00
MYBPH	NP_004988.2	388	3	7.34	RTP5	NP_776182.2	636	3	4.48
CHI3L1	NP_001267.2	673	4	5.65	DEFB125	NP_697020.2	74	0	0.00
CHIT1	NP_001243054.2	489	1	1.94	DEFB126	NP_112193.1	171	0	0.00
BTG2	NP_006754.1	656	0	0.00	DEFB127	NP_620713.1	79	0	0.00
FMOD	NP_002014.2	1034	4	3.67	DEFB128	NP_001032821.1	94	0	0.00
PRELP	NP_958505.1	725	5	6.55	DEFB129	NP_543021.1	127	0	0.00
OPTC	NP_055174.1	607	3	4.69	DEFB132	NP_997352.1	79	0	0.00
ATP2B4	NP_001675.3	1160	1	0.82	C20orf96	NP_695001.2	134	0	0.00
LAX1	NP_001129662.1	281	6	20.28	ZCCHC3	NP_149080.2	397	0	0.00
ZBED6	NP_001167579.1	276	1	3.44	SOX12	NP_008874.2	279	2	7.57
ZC3H11A	XP_016858448.1	438	6	13.01	NRSN2	XP_016883565.1	130	2	15.38
SNRPE	NP_003085.1	1673	9	5.11	TRIB3	XP_016883478.1	1497	4	2.54
SOX13	NP_005677.2	616	4	6.17	RBCK1	NP_006453.1	659	3	4.32
ETNK2	NP_001284691.1	584	12	19.52	TBC1D20	XP_016883134.1	332	2	5.72
REN	NP_000528.1	1627	7	4.09	CSNK2A1	NP_001349700.1	2232	5	2.13
KISS1	NP_002247.3	513	5	9.26	TCF15	NP_004600.2	460	0	0.00
GOLT1A	XP_016855803.1	642	15	22.20	SRXN1	NP_542763.1	803	2	2.37
PLEKHA6	XP_016856177.1	266	11	39.28	SCRT2	NP_149120.1	517	1	1.84
PPP1R15B	XP_005245608.2	414	13	29.83	SLC52A3	XP_024307589.1	267	1	3.56
PIK3C2B	NP_001364264.1	1703	8	4.46	FAM110A	NP_001035812.1	126	1	7.54
MDM4	XP_016856800.1	821	4	4.63	ANGPT4	XP_011527541.1	375	1	2.53
LRRN2	XP_016855539.1	587	13	21.04	RSPO4	NP_001035096.1	398	1	2.39
NFASC	XP_011507620.1	1027	4	3.70	PSMF1	NP_001310337.1	437	1	2.17
CNTN2	XP_016857687.1	1665	13	7.42	TMEM74B	XP_011527583.1	57	0	0.00
TMEM81	NP_976310.1	48	10	197.91	C20orf202	NP_001009612.1	4	0	0.00
RBBP5	NP_001180201.1	900	7	7.39	RAD21L1	XP_006723665.1	698	0	0.00
DSTYK	XP_011507694.1	904	16	16.81	SNPH	XP_011527704.1	561	0	0.00
TMCC2	NP_055673.2	373	10	25.47	SDCBP2	NP_536737.3	151	1	6.29
NUAK2	NP_112214.3	303	5	15.68	FKBP1A	NP_001186715.1	1391	1	0.68
KLHDC8A	XP_024303889.1	197	1	4.82	NSFL1C	NP_061327.2	455	5	10.44
LEMD1	NP_001185980.1	176	3	16.19	SIRPB2	NP_001128308.1	190	2	10.00
CDK18	XP_016856912.1	1846	9	4.63	SIRPD	NP_848555.2	237	3	12.02
MFSD4A	NP_857595.3	192	2	9.90	SIRPB1	NP_001077379.1	254	2	7.48
ELK4	NP_001964.2	538	5	8.83	SIRPG	NP_543006.2	337	2	5.64
SLC45A3	NP_149093.1	471	9	18.15	SIRPA	NP_001317657.1	609	2	3.12
NUCKS1	XP_005245510.1	305	7	21.80	PDYN	XP_016883367.1	1168	3	2.44
RAB29	XP_016858237.1	506	5	9.39	STK35	XP_011527476.1	508	1	1.87
SLC41A1	NP_776253.3	341	6	16.71	TGM3	NP_003236.3	337	0	0.00
PM20D1	NP_689704.4	584	5	8.13	TGM6	NP_945345.2	214	3	13.32
SLC26A9	NP_599152.2	342	4	11.11	SNRPB	NP_937859.1	1708	3	1.67
RAB7B	NP_001291768.1	1368	3	2.08	ZNF343	NP_001308734.1	198	0	0.00
CTSE	NP_683865.1	647	4	5.87	TMC2	NP_542789.2	268	1	3.54
RHEX	NP_001356419.1	76	3	37.50	NOP56	NP_006383.2	1734	5	2.74
AVPR1B	NP_000698.1	398	0	0.00	IDH3B	NP_777280.1	985	1	0.96
FAM72A	NP_001372173.1	226	3	12.61	EBF4	XP_016883473.1	431	1	2.20
SRGAP2	XP_011507656.1	985	1	0.96	CPXM1	NP_001171628.1	280	1	3.39
IKBKE	XP_005273413.1	1281	2	1.48	C20orf141	NP_542777.1	0	0	#DIV/0!
RASSF5	NP_872604.1	591	1	1.61	TMEM239	NP_001305136.1	6	0	0.00

EIF2D	XP_011507559.1	460	6	12.39	PCED1A	NP_001258097.1	100	0	0.00
DYRK3	NP_003573.2	837	6	6.81	VPS16	NP_072097.2	527	0	0.00
MAPKAPK2	NP_116584.2	534	3	5.34	PTPRA	NP_001372245.1	790	10	12.02
IL10	NP_000563.1	2852	9	3.00	GNRH2	NP_001492.1	467	9	18.31
IL19	NP_715639.1	443	6	12.87	MRPS26	NP_110438.1	417	5	11.39
IL20	NP_001372095.1	103	4	36.89	OXT	NP_000906.1	900	6	6.33
IL24	NP_001172085.1	509	6	11.20	AVP	NP_000481.2	1226	5	3.87
FCMR	NP_001135945.1	224	6	25.45	UBOX5	NP_955447.1	757	11	13.80
PIGR	NP_002635.2	654	10	14.53	FASTKD5	NP_068598.1	187	2	10.16
FCAMR	XP_016857931.1	113	6	50.44	LZTS3	XP_011527709.1	143	10	66.43
C1orf116	NP_076427.2	197	5	24.11	DDRGK1	NP_076424.1	186	6	30.64
YOD1	NP_001263249.1	498	1	1.91	ITPA	NP_001311169.1	1135	10	8.37
PFKFB2	XP_024303425.1	609	3	4.68	SLC4A11	XP_011527685.1	455	7	14.61
C4BPB	NP_000707.1	400	6	14.25	C20orf194	XP_005260741.1	79	1	12.02
C4BPA	NP_000706.1	551	9	15.52	ATRN	NP_001193976.1	461	7	14.42
CD55	NP_001108224.1	658	7	10.11	GFRA4	NP_071422.1	161	7	41.30
CR2	NP_001006659.1	748	7	8.89	ADAM33	XP_011527669.1	529	5	8.98
CR1	NP_000642.3	634	6	8.99	SIGLEC1	NP_001354018.1	1007	5	4.72
CR1L	NP_783641.1	281	1	3.38	HSPA12B	XP_016883121.1	1407	9	6.08
CD46	NP_758868.1	628	6	9.08	C20orf27	NP_001245358.1	67	7	99.25
CD34	NP_001020280.1	2251	6	2.53	SPEF1	NP_056232.2	180	6	31.67
PLXNA2	XP_005273221.1	722	3	3.95	CENPB	NP_001801.1	502	6	11.35
CAMK1G	XP_016857355.1	1407	7	4.73	CDC25B	NP_001274445.1	1098	2	1.73
LAMB3	XP_016856761.1	633	6	9.00	AP5S1	NP_001191375.1	91	4	41.76
G0S2	NP_056529.1	418	7	15.91	MAVS	NP_065797.2	731	1	1.30
HSD11B1	NP_861420.1	822	2	2.31	PANK2	NP_705902.2	369	7	18.02
TRAF3IP3	NP_079504.2	498	7	13.35	RNF24	XP_011527448.1	325	10	29.23
C1orf74	NP_689698.1	25	8	303.98	SMOX	NP_001257620.1	802	6	7.11
IRF6	NP_006138.1	817	6	6.98	ADRA1D	NP_000669.1	1041	3	2.74
UTP25	NP_055203.4	733	4	5.18	PRNP	NP_001073590.1	1043	5	4.55
SYT14	NP_001139733.1	505	10	18.81	PRND	NP_036541.2	297	4	12.79
SERTAD4	NP_001341102.1	172	6	33.14	RASSF2	XP_016883639.1	583	4	6.52
HHAT	NP_001164058.1	442	0	0.00	SLC23A2	NP_005107.4	528	3	5.40
KCNH1	NP_002229.1	925	11	11.30	TMEM230	XP_024307647.1	282	1	3.37
RCOR3	XP_016857247.1	477	8	15.93	PCNA	NP_872590.1	1881	2	1.01
TRAF5	XP_011508259.1	699	8	10.87	CDS2	NP_003809.1	518	3	5.50
RD3	XP_016856640.1	341	8	22.29	PROKR2	XP_016883135.1	392	2	4.85
SLC30A1	NP_067017.2	482	8	15.77	GPCPD1	NP_062539.1	498	4	7.63
NEK2	NP_001191111.1	1342	7	4.96	SHLD1	XP_011527479.1	53	2	35.85
LPGAT1	NP_001362774.1	447	5	10.63	CHGB	NP_001810.2	1055	4	3.60
INTS7	NP_001186738.1	344	2	5.52	TRMT6	NP_057023.2	689	1	1.38
DTL	NP_057532.4	805	4	4.72	MCM8	NP_115874.3	1032	5	4.60
PPP2R5A	NP_006234.1	962	4	3.95	CRLS1	NP_061968.1	642	8	11.84
PACC1	NP_001364407.1	44	0	0.00	LRRN4	NP_689824.2	492	1	1.93
NENF	NP_037481.1	498	3	5.72	FERMT1	NP_060141.3	404	5	11.76
ATF3	NP_001665.1	1356	3	2.10	BMP2	NP_001191.1	1781	4	2.13
FAM71A	NP_705834.2	95	1	10.00	HAO1	NP_060015.1	829	8	9.17
BATF3	NP_061134.1	570	1	1.67	TMX4	NP_066979.2	610	9	14.02
NSL1	NP_001284668.1	297	3	9.60	PLCB1	NP_056007.1	1186	11	8.81
TATDN3	NP_001139643.1	208	0	0.00	PLCB4	XP_024307666.1	1121	9	7.63
SPATA45	NP_001019772.1	72	0	0.00	LAMP5	NP_036393.1	596	8	12.75
FLVCR1	NP_054772.1	360	4	10.56	PAK5	XP_016883449.1	1199	8	6.34
VASH2	NP_001287985.1	125	0	0.00	ANKEF1	NP_071379.3	971	11	10.76
ANGEL2	NP_653168.2	430	1	2.21	SNAP25	NP_001309831.1	2088	5	2.27
RPS6KC1	NP_036556.2	324	1	2.93	MKKS	NP_740754.1	1279	5	3.71
PROX1	XP_016857321.1	931	4	4.08	SLX4IP	NP_001009608.1	136	12	83.82
SMYD2	NP_064582.2	1749	2	1.09	JAG1	NP_000205.1	1733	6	3.29
PTPN14	XP_016857430.1	734	1	1.29	BTBD3	NP_852108.2	303	11	34.49
CENPF	XP_016855575.1	854	4	4.45	SPTLC3	NP_001336874.1	643	4	5.91
KCNK2	XP_016856737.1	403	0	0.00	ISM1	XP_016883169.1	185	2	10.27
KCTD3	NP_057205.2	284	4	13.38	TASP1	NP_001310531.1	302	1	3.15
USH2A	NP_996816.3	1317	7	5.05	ESF1	XP_016883363.1	700	2	2.71
ESRRG	XP_011507568.1	580	5	8.19	NDUFAF5	NP_001034464.1	695	1	1.37
GPATCH2	XP_011507993.1	317	4	11.99	SEL1L2	XP_011527676.1	430	0	0.00
SPATA17	NP_001362584.1	171	5	27.78	MACROD2	NP_001338590.1	489	1	1.94
RRP15	NP_057136.2	845	3	3.37	FLRT3	NP_938205.1	777	4	4.89

TGFB2	NP_001129071.1	1278	2	1.49	KIF16B	NP_001186794.1	1229	3	2.32
LYPLAL1	XP_016855760.1	421	4	9.03	SNRPB2	NP_937863.1	1379	6	4.13
SLC30A10	NP_001363858.1	543	1	1.75	OTOR	NP_064542.1	201	2	9.45
EPRS1	NP_004437.2	2694	3	1.06	PCSK2	NP_002585.2	1103	2	1.72
BPNT1	XP_016855532.1	298	3	9.56	BFSP1	NP_001265536.1	219	0	0.00
IARS2	NP_060530.3	1039	4	3.66	DSTN	NP_006861.1	1403	2	1.35
RAB3GAP2	NP_036546.2	413	0	0.00	RRBP1	NP_004578.3	454	5	10.46
MARK1	NP_001273053.1	420	2	4.52	BANF2	NP_001152967.1	190	1	5.00
C1orf115	NP_078985.3	113	2	16.81	SNX5	NP_689413.1	490	8	15.51
MTARC2	NP_001317971.1	207	1	4.59	MGME1	XP_024307785.1	308	2	6.17
MTARC1	XP_011508202.1	287	1	3.31	OVOL2	NP_067043.2	264	10	35.98
HLX	NP_068777.1	487	4	7.80	PET117	NP_001158283.1	86	3	33.14
DUSP10	NP_009138.1	912	4	4.17	KAT14	NP_001371121.1	334	7	19.91
HHIPL2	NP_079022.2	127	0	0.00	ZNF133	NP_001269936.1	194	7	34.28
TAF1A	XP_016858247.1	311	3	9.16	DZANK1	NP_001338612.1	188	7	35.37
MIA3	NP_001310991.1	504	3	5.65	POLR3F	NP_006457.2	551	10	17.24
AIDA	NP_073742.2	115	1	8.26	RBBP9	NP_006597.2	212	12	53.77
BROX	XP_016855864.1	134	1	7.09	SEC23B	XP_016883082.1	986	8	7.71
FAM177B	XP_016856774.1	63	0	0.00	SMIM26	NP_001335886.1	99	0	0.00
DISP1	XP_016858100.1	262	5	18.13	DTD1	NP_543010.3	295	6	19.32
TLR5	XP_016857697.1	1804	1	0.53	SCP2D1	NP_848578.1	131	5	36.26
SUSD4	XP_016857073.1	202	9	42.32	SLC24A3	NP_065740.2	428	8	17.76
CCDC185	NP_689823.2	78	2	24.36	RIN2	XP_016883376.1	365	7	18.22
CAPN8	NP_001137434.1	696	7	9.55	NAA20	NP_057184.1	841	5	5.65
CAPN2	NP_001139540.1	898	2	2.12	CRNKL1	NP_001265554.1	970	5	4.90
TP53BP2	NP_001026855.2	455	8	16.70	CFAP61	XP_011527513.1	193	4	19.69
FBXO28	NP_055991.1	249	11	41.97	INSM1	NP_002187.1	498	3	5.72
DEGS1	NP_003667.1	456	6	12.50	RALGAPA2	XP_005260825.1	297	4	12.79
NVL	NP_001230076.1	999	3	2.85	KIZ	NP_001263318.1	201	2	9.45
CNIH4	NP_001264129.1	375	7	17.73	XRN2	NP_036387.2	1324	6	4.30
WDR26	NP_001366332.1	522	8	14.56	NKX2-4	NP_149416.1	409	5	11.61
CNIH3	NP_001309231.1	449	7	14.81	NKX2-2	NP_002500.1	907	4	4.19
DNAH14	NP_001354408.1	182	1	5.22	PAX1	NP_006183.2	659	3	4.32
LBR	XP_005273182.1	926	4	4.10	FOXA2	NP_068556.2	1482	4	2.56
ENAH	XP_016857241.1	796	2	2.39	SSTR4	NP_001043.2	781	3	3.65
SRP9	NP_003124.1	1231	3	2.32	THBD	NP_000352.1	859	4	4.42
EPHX1	NP_001365355.1	790	2	2.40	CD93	NP_036204.2	735	4	5.17
TMEM63A	XP_011542633.1	390	6	14.61	NXT1	NP_037380.1	425	10	22.35
LEFTY1	NP_066277.1	617	5	7.70	GZF1	NP_071927.1	321	13	38.47
PYCR2	NP_001258610.1	505	4	7.52	NAPB	NP_001269949.1	597	4	6.36
LEFTY2	NP_001165896.1	684	6	8.33	CSTL1	NP_612140.1	79	9	108.22
SDE2	NP_689821.3	195	4	19.49	CST11	NP_543020.2	139	10	68.34
H3-3A	NP_001365972.1	1694	1	0.56	CST8	NP_005483.1	365	7	18.22
ACBD3	NP_073572.2	441	2	4.31	CST9L	NP_542177.1	61	11	171.30
MIXL1	NP_001269331.1	661	2	2.87	CST9	NP_001008693.2	88	12	129.54
LIN9	XP_016856573.1	507	0	0.00	CST3	NP_000090.1	762	8	9.97
PARP1	NP_001609.2	1688	2	1.13	CST4	NP_001890.1	296	9	28.88
STUM	NP_001003665.1	109	0	0.00	CST1	NP_001889.2	158	10	60.12
ITPKB	NP_002212.3	2451	5	1.94	CST2	NP_001313.1	153	8	49.67
PSEN2	XP_016857324.1	1300	5	3.65	CST5	NP_001891.2	172	6	33.14
COQ8A	XP_016857341.1	458	0	0.00	GGTLC1	XP_016883615.1	125	0	0.00
CDC42BPA	NP_055641.3	1378	3	2.07	SYNDIG1	NP_001310536.1	594	0	0.00
ZNF678	NP_848644.2	178	0	0.00	CST7	NP_003641.3	561	10	16.93
JMJD4	XP_011542564.1	184	1	5.16	APMAP	NP_065392.1	772	2	2.46
SNAP47	XP_016855720.1	361	2	5.26	ACSS1	NP_001239606.1	723	8	10.51
PRSS38	NP_898885.1	118	0	0.00	VSX1	NP_001243201.1	423	1	2.25
WNT9A	NP_003386.1	936	1	1.01	ENTPD6	NP_001107561.2	378	4	10.05
WNT3A	XP_011542621.1	1605	2	1.18	PYGB	NP_002853.2	937	4	4.06
ARF1	NP_001649.1	2192	5	2.17	ABHD12	NP_056415.1	399	3	7.14
C1orf35	NP_077295.1	138	2	13.77	GINS1	NP_066545.3	832	3	3.43
MRPL55	NP_001308213.1	485	3	5.88	NINL	XP_011527489.1	376	4	10.11
GUK1	NP_001229769.1	1763	4	2.16	NANP	NP_689880.1	378	1	2.51
GJC2	NP_065168.2	409	0	0.00	ZNF337	XP_016883293.1	200	1	4.75
IBA57	NP_001010867.1	306	3	9.31	DEFB115	NP_001032819.1	64	1	14.84
OBSCN	NP_001373054.1	1175	7	5.66	DEFB116	NP_001032820.1	59	0	0.00
TRIM11	XP_011542587.1	587	7	11.33	DEFB118	NP_473453.1	94	0	0.00

TRIM17	XP_006711842.1	596	5	7.97	DEFB119	NP_695021.2	117	0	0.00
H3-4	NP_003484.1	1454	9	5.88	DEFB121	NP_001165303.1	74	0	0.00
H2AW	NP_254280.1	923	5	5.15	DEFB123	NP_697019.1	121	0	0.00
H2BU1	NP_778225.1	851	5	5.58	DEFB124	XP_016883271.1	101	0	0.00
RNF187	NP_001010858.2	287	4	13.24	REM1	XP_016883322.1	2002	3	1.42
RHOU	NP_067028.1	1813	3	1.57	HM13	NP_848696.1	442	4	8.60
RAB4A	NP_004569.2	1657	2	1.15	ID1	NP_002156.2	979	2	1.94
CCSAP	NP_660300.3	207	0	0.00	COX4I2	NP_115998.2	604	8	12.58
ACTA1	NP_001091.1	2450	5	1.94	BCL2L1	NP_001182.1	2354	6	2.42
NUP133	NP_060700.2	773	1	1.23	TPX2	NP_036244.2	937	4	4.06
ABCB10	NP_036221.2	524	2	3.63	MYLK2	NP_149109.1	801	9	10.67
TAF5L	XP_005273156.1	924	3	3.08	FOXS1	NP_004109.1	463	9	18.47
URB2	NP_055592.2	290	1	3.28	DUSP15	XP_016883145.1	747	7	8.90
GALNT2	NP_001278795.1	493	2	3.85	TTLL9	NP_001008409.1	349	4	10.89
PGBD5	NP_001245240.1	384	2	4.95	PDRG1	NP_110442.1	268	3	10.63
COG2	NP_031383.1	753	3	3.78	XKR7	NP_001011718.1	448	8	16.96
AGT	NP_001369746.2	1473	2	1.29	CCM2L	XP_011526868.1	155	8	49.03
CAPN9	XP_011542319.1	678	2	2.80	HCK	NP_002101.2	1787	5	2.66
C1orf198	NP_116189.1	28	0	0.00	TM9SF4	NP_055557.2	364	8	20.88
TTC13	NP_001363443.1	282	1	3.37	PLAGL2	NP_002648.1	536	7	12.41
ARV1	XP_024304970.1	233	1	4.08	POFUT1	NP_056167.1	555	11	18.83
FAM89A	NP_940954.1	68	1	13.97	KIF3B	NP_004789.1	1093	13	11.30
TRIM67	NP_001004342.3	611	2	3.11	ASXL1	NP_056153.2	476	14	27.94
C1orf131	NP_001287759.1	66	0	0.00	NOL4L	NP_001243727.1	252	13	49.01
GNPAT	NP_055051.1	435	1	2.18	COMMD7	NP_001092809.1	426	11	24.53
EXOC8	NP_787072.2	717	0	0.00	DNMT3B	NP_787046.1	1491	5	3.19
SPRTN	NP_114407.3	503	0	0.00	MAPRE1	NP_036457.1	1228	12	9.28
EGLN1	NP_071334.1	789	3	3.61	EFCAB8	NP_001137439.1	214	0	0.00
TSNAX	NP_005990.1	480	2	3.96	SUN5	XP_011526876.1	293	15	48.63
DISC1	NP_001158009.1	785	2	2.42	BPIFB2	NP_079503.1	317	8	23.97
SIPA1L2	XP_016857386.1	461	1	2.06	BPIFB6	XP_016883152.1	138	8	55.07
MAP10	NP_061963.3	119	0	0.00	BPIFB3	NP_001363861.1	119	8	63.86
NTPCR	NP_001316381.1	301	1	3.16	BPIFB4	NP_872325.2	106	8	71.69
PCNX2	NP_055616.3	155	1	6.13	BPIFA2	NP_542141.1	160	6	35.62
MAP3K21	NP_115811.2	919	2	2.07	BPIFA3	NP_848561.2	106	6	53.77
KCNK1	NP_002236.1	484	1	1.96	BPIFA1	NP_570913.1	249	3	11.45
SLC35F3	NP_775779.1	304	1	3.12	BPIFB1	XP_024307786.1	214	5	22.20
COA6	NP_001193570.2	486	4	7.82	CDK5RAP1	XP_011527158.1	600	11	17.42
TARBP1	XP_005273291.1	555	3	5.13	SNTA1	XP_005260574.1	623	11	16.77
IRF2BP2	NP_001070865.1	387	2	4.91	CBFA2T2	NP_001028171.1	337	10	28.19
TOMM20	NP_055580.1	1288	3	2.21	NECAB3	XP_005260567.1	142	2	13.38
RBM34	XP_011542435.1	1441	4	2.64	C20orf144	NP_543015.1	37	0	0.00
ARID4B	XP_024303394.1	1098	4	3.46	ACTL10	NP_001019846.1	946	1	1.00
GGPS1	NP_001358407.1	662	5	7.17	E2F1	NP_005216.1	1307	0	0.00
TBCE	NP_001274730.1	547	5	8.68	PXMP4	NP_009169.3	220	2	8.64
B3GALNT2	XP_006711812.1	158	0	0.00	ZNF341	NP_001269862.1	166	2	11.45
GNG4	NP_001092191.1	857	1	1.11	CHMP4B	NP_789782.1	739	4	5.14
LYST	XP_011542336.1	586	7	11.35	RALY	XP_016883220.1	1444	6	3.95
NID1	XP_011542497.1	910	4	4.18	EIF2S2	NP_001303292.1	1220	7	5.45
GPR137B	NP_003263.1	256	2	7.42	ASIP	NP_001372147.1	241	5	19.71
ERO1B	NP_063944.3	430	1	2.21	AHCY	XP_016883198.1	1534	8	4.95
EDARADD	NP_665860.2	567	6	10.05	ITCH	XP_016883579.1	1535	9	5.57
LGALS8	NP_006490.3	387	2	4.91	DYNLRB1	NP_001369294.1	540	3	5.28
HEATR1	NP_060542.4	979	3	2.91	MAP1LC3A	XP_011527385.1	1055	4	3.60
ACTN2	NP_001265272.1	1655	5	2.87	PIGU	XP_016883153.1	154	10	61.69
MTR	XP_011542496.1	539	1	1.76	TP53INP2	NP_001316358.1	198	5	23.99
MT1HL1	NP_001263616.1	250	0	0.00	NCOA6	XP_016883232.1	612	4	6.21
RYR2	XP_006711865.1	1261	5	3.77	GGT7	NP_821158.2	337	5	14.09
ZP4	NP_067009.1	305	2	6.23	ACSS2	XP_011527211.1	848	5	5.60
CHRM3	XP_016855641.1	670	3	4.25	GSS	NP_001309423.1	506	3	5.63
FMN2	NP_001292353.1	1387	11	7.53	MYH7B	XP_011527243.1	1374	5	3.46
GREM2	XP_005273283.1	329	5	14.44	TRPC4AP	NP_955400.1	285	8	26.67
RGS7	NP_001361736.1	1101	13	11.22	EDEM2	NP_001138497.1	336	12	33.93
FH	NP_000134.2	1185	6	4.81	PROCR	XP_011526798.1	432	5	10.99
KMO	XP_005273394.1	950	9	9.00	MMP24	NP_006681.1	455	4	8.35
OPN3	NP_055137.2	329	12	34.65	EIF6	NP_852133.1	1212	6	4.70

CHML	NP_001368783.1	580	5	8.19	FAM83C	NP_848563.1	40	9	213.74
WDR64	NP_001354411.1	734	10	12.94	UQCC1	NP_001171906.1	443	5	10.72
EXO1	NP_006018.4	1642	9	5.21	GDF5	NP_000548.2	794	3	3.59
MAP1LC3C	NP_001004343.1	583	9	14.66	CEP250	XP_005260320.1	447	6	12.75
PLD5	NP_001358991.1	223	13	55.38	C20orf173	XP_006723773.1	4	0	0.00
CEP170	XP_016858440.1	374	4	10.16	ERGIC3	NP_938408.1	652	6	8.74
SDCCAG8	XP_011542328.1	466	10	20.39	SPAG4	NP_003107.1	437	2	4.35
AKT3	NP_859029.1	1919	11	5.45	CPNE1	NP_003906.2	331	5	14.35
ZBTB18	NP_991331.1	504	12	22.62	RBM12	NP_006038.2	655	3	4.35
C1orf100	XP_016856060.1	47	9	181.91	NFS1	NP_001185918.1	820	3	3.48
ADSS2	NP_001352002.1	883	6	6.45	ROMO1	XP_016883167.1	616	2	3.08
CATSPERE	XP_011542440.1	104	7	63.94	RBM39	XP_016883627.1	945	2	2.01
DESI2	NP_057160.2	351	8	21.65	PHF20	NP_057520.2	959	1	0.99
COX20	NP_001299801.1	223	15	63.90	SCAND1	NP_361012.3	370	2	5.13
HNRNPU	NP_004492.2	1385	12	8.23	CNBD2	XP_011526895.1	638	1	1.49
EFCAB2	NP_001137415.1	1064	15	13.39	EPB41L1	XP_011526969.1	699	2	2.72
KIF26B	NP_060482.2	927	12	12.30	AAR2	NP_056326.2	500	0	0.00
SMYD3	NP_001362891.1	1732	16	8.78	DLGAP4	NP_055717.2	516	2	3.68
TFB2M	XP_011542550.1	1441	8	5.27	MYL9	NP_006088.2	1703	1	0.56
CNST	NP_689822.2	117	8	64.95	TGIF2-RAB5IF	NP_001186464.1	355	0	0.00
SCCPDH	NP_057086.2	344	7	19.33	TGIF2	NP_068581.1	253	0	0.00
AHCTF1	NP_056261.4	523	8	14.53	RAB5IF	NP_955777.1	209	0	0.00
ZNF695	NP_001191150.2	198	6	28.79	SLA2	NP_115590.1	269	5	17.66
ZNF670	NP_001191149.1	155	3	18.39	NDRG3	NP_071922.2	306	2	6.21
ZNF669	NP_001136044.1	173	1	5.49	DSN1	NP_001138789.1	552	1	1.72
ZNF124	NP_001230669.1	226	5	21.02	SOGA1	NP_954650.2	145	1	6.55
ZNF496	NP_116141.1	256	4	14.84	TLDC2	XP_016883161.1	133	0	0.00
NLRP3	XP_016855670.1	1341	1	0.71	SAMHD1	NP_001350658.1	840	3	3.39
OR2B11	NP_001004492.1	143	2	13.29	RBL1	NP_002886.2	1050	2	1.81
OR2C3	NP_932340.4	71	2	26.76	MROH8	NP_689716.4	88	1	10.79
GCSAML	NP_660321.1	103	6	55.34	RPN2	NP_001311232.1	625	1	1.52
OR2G2	NP_001001915.1	94	0	0.00	GHRH	NP_001171660.1	575	1	1.65
OR2G3	NP_001001914.1	86	0	0.00	MANBAL	XP_016883495.1	95	1	10.00
OR13G1	NP_001005487.1	102	2	18.63	SRC	XP_016883513.1	4587	5	1.04
OR6F1	NP_001005286.1	104	2	18.27	BLCAP	XP_016883088.1	298	5	15.94
OR14A2	NP_001342221.1	19	2	99.99	NNAT	NP_001309731.1	533	5	8.91
OR14K1	NP_001004732.2	35	0	0.00	CTNNBL1	NP_110517.2	977	4	3.89
OR1C1	NP_036485.2	70	0	0.00	VSTM2L	NP_542174.1	457	4	8.31
OR14A16	NP_001001966.1	61	0	0.00	TTI1	NP_055472.1	387	0	0.00
OR11L1	NP_001001959.1	68	0	0.00	RPRD1B	NP_067038.1	365	1	2.60
TRIM58	NP_056246.3	513	9	16.67	TGM2	NP_001310247.1	1175	1	0.81
OR2W3	NP_001001957.2	151	1	6.29	KIAA1755	XP_024307781.1	257	0	0.00
OR2T8	NP_001005522.1	59	1	16.10	BPI	NP_001716.3	401	1	2.37
OR2AJ1	NP_001342164.2	24	0	0.00	LBP	NP_004130.2	346	5	13.73
OR2L8	NP_001001963.1	94	0	0.00	RALGAPB	XP_005260519.1	269	6	21.19
OR2AK2	NP_001004491.1	121	1	7.85	ADIG	NP_001018092.1	157	6	36.30
OR2L5	NP_001245213.1	35	0	0.00	ARHGAP40	NP_001157903.1	168	0	0.00
OR2L2	NP_001372784.1	41	1	23.17	SLC32A1	NP_542119.1	1581	5	3.00
OR2L3	NP_001004687.1	59	0	0.00	ACTR5	NP_079131.3	377	7	17.64
OR2L13	NP_787107.1	311	1	3.05	PPP1R16B	NP_056383.1	978	8	7.77
OR2M5	NP_001004690.1	31	0	0.00	FAM83D	NP_112181.3	440	6	12.95
OR2M2	NP_001004688.1	37	0	0.00	DHX35	NP_068750.2	994	8	7.65
OR2M3	NP_001004689.1	84	0	0.00	MAFB	NP_005452.2	863	4	4.40
OR2M4	NP_059974.1	59	0	0.00	TOP1	NP_003277.1	1811	4	2.10
OR2T33	NP_001004695.1	65	0	0.00	PLCG1	XP_005260495.1	1199	6	4.75
OR2T12	NP_001004692.1	54	0	0.00	ZHX3	NP_001371253.1	247	6	23.08
OR2M7	NP_001004691.1	58	0	0.00	LPIN3	XP_011527298.1	615	3	4.63
OR14C36	NP_001001918.1	70	0	0.00	EMILIN3	NP_443078.1	11	0	0.00
OR2T4	NP_001004696.1	106	0	0.00	CHD6	NP_115597.3	1253	8	6.07
OR2T6	NP_001005471.1	60	0	0.00	PTPRT	XP_024307588.1	1446	8	5.26
OR2T1	NP_112166.1	125	0	0.00	SRSF6	NP_006266.2	1364	6	4.18
OR2T7	NP_001372980.1	21	0	0.00	L3MBTL1	NP_001364235.1	523	6	10.90
OR2T2	NP_001004136.1	77	0	0.00	SGK2	NP_057360.2	1032	4	3.68
OR2T3	NP_001005495.1	34	0	0.00	IFT52	NP_057088.2	311	4	12.22
OR2T5	NP_001004697.1	67	0	0.00	MYBL2	NP_002457.1	1826	2	1.04
OR2G6	NP_001013373.1	34	0	0.00	GTSF1L	XP_005260355.1	77	0	0.00

OR2T29	NP_001004694.2	49	0	0.00	TOX2	NP_001092267.1	553	8	13.74
OR2T34	NP_001001821.1	38	0	0.00	JPH2	NP_065166.2	444	1	2.14
OR2T10	NP_001004693.1	47	0	0.00	OSER1	XP_011527156.1	162	0	0.00
OR2T11	NP_001001964.1	43	0	0.00	GDAP1L1	NP_001243666.1	557	3	5.12
OR2T35	NP_001001827.1	76	0	0.00	FITM2	NP_001073941.1	199	3	14.32
OR2T27	NP_001372989.1	45	0	0.00	R3HDML	NP_848586.1	168	4	22.62
OR14I1	NP_001004734.1	287	0	0.00	HNF4A	NP_787110.2	2096	5	2.27
LYPD8	NP_001078943.2	119	0	0.00	TTPAL	XP_011527345.1	232	0	0.00
SH3BP5L	NP_085148.1	235	0	0.00	SERINC3	NP_945179.1	261	2	7.28
ZNF672	NP_079112.1	215	0	0.00	PKIG	XP_016883103.1	196	4	19.39
ZNF692	XP_011542525.1	285	0	0.00	ADA	NP_001308980.1	1266	2	1.50
PGBD2	NP_733843.1	215	0	0.00	CCN5	XP_016883606.1	326	1	2.91
TUBB8	NP_817124.1	1309	8	11.61	KCNK15	XP_016883492.1	183	3	15.57
ZMYND11	NP_001357045.1	402	9	38.67	RIMS4	NP_892015.1	759	4	5.01
DIP2C	NP_055789.1	476	10	33.26	YWHAB	XP_016883528.1	2734	3	1.04
LARP4B	NP_055970.1	400	8	29.23	PABPC1L	NP_001359108.1	841	1	1.13
GTPBP4	NP_036473.2	1497	5	4.53	TOMM34	NP_006800.2	879	0	0.00
IDI2	NP_150286.1	267	9	42.69	STK4	NP_006273.1	655	7	10.15
IDI1	XP_016871680.1	449	3	7.93	KCNS1	NP_001309728.1	854	7	7.79
WDR37	NP_054742.2	771	10	14.50	WFDC5	NP_663627.1	166	7	40.06
ADARB2	NP_061172.1	580	7	12.74	WFDC12	NP_543145.1	77	6	74.02
PFKP	XP_005252522.1	1130	8	7.08	PI3	NP_002629.1	1807	6	3.15
PITRM1	NP_001229238.1	685	8	11.09	SEMG1	NP_002998.1	239	5	19.87
KLF6	NP_001153596.1	904	3	3.15	SEMG2	NP_002999.1	209	6	27.27
AKR1E2	XP_011518017.1	170	0	0.00	SLPI	NP_003055.1	655	11	15.95
AKR1C1	NP_001344.2	453	2	4.19	MATN4	NP_085095.1	545	5	8.72
AKR1C2	NP_001307956.1	448	3	6.36	RBPJL	NP_055091.2	535	3	5.33
AKR1C3	NP_001240837.1	576	3	4.95	SDC4	NP_002990.2	731	3	3.90
AKR1C4	NP_001809.4	318	3	8.96	SYS1	NP_001093261.1	308	9	27.76
UCN3	NP_444277.2	227	2	8.37	TP53TG5	NP_055292.1	88	8	86.36
TUBAL3	NP_001165335.1	1273	8	5.97	DBNDD2	NP_060948.3	142	3	20.07
NET1	NP_001040625.1	600	3	4.75	PIGT	NP_057021.2	381	6	14.96
CALML5	NP_059118.2	1162	2	1.64	WFDC2	NP_006094.3	327	13	37.77
CALML3	NP_005176.1	1156	2	1.64	SPINT3	NP_006643.1	229	10	41.48
ASB13	NP_078977.2	1216	2	1.56	WFDC6	NP_543017.1	212	8	35.85
TASOR2	XP_011517820.1	115	0	0.00	EPPIN-WFDC(	NP_001185915.1	390	13	31.67
GDI2	NP_001108628.1	1290	6	4.42	EPPIN	NP_065131.1	357	7	18.63
ANKRD16	NP_061919.1	826	6	6.90	WFDC8	NP_570966.2	193	13	63.99
FBH1	XP_016872333.1	1064	4	3.57	WFDC9	NP_671731.1	34	9	251.46
IL15RA	XP_011517776.1	482	4	7.88	WFDC10A	NP_542791.1	72	9	118.74
IL2RA	NP_001295172.1	1175	4	3.23	WFDC11	NP_671730.1	28	4	135.71
RBM17	NP_116294.1	802	3	3.55	WFDC10B	NP_742143.1	62	9	137.90
PFKFB3	XP_016871816.1	740	5	6.42	WFDC13	NP_742002.1	206	12	55.34
PRKCQ	XP_005252554.1	1314	12	8.68	WFDC3	XP_016883157.1	99	15	143.93
SFMBT2	XP_016871955.1	355	6	16.06	SPINT4	NP_848550.1	210	12	54.28
ITIH5	NP_085046.5	389	8	19.54	DNTTIP1	NP_443183.1	243	9	35.18
ITIH2	NP_002207.2	583	7	11.41	UBE2C	NP_861515.1	2037	4	1.87
KIN	XP_011517729.1	478	0	0.00	TNNC2	NP_003270.1	1244	7	5.35
ATP5F1C	NP_005165.1	1282	4	2.96	SNX21	NP_690857.1	131	7	50.76
TAF3	NP_114129.1	1082	4	3.51	ACOT8	NP_005460.2	469	7	14.18
GATA3	NP_001002295.1	2257	7	2.95	ZSWIM3	NP_542790.2	182	6	31.32
CELF2	XP_024303540.1	727	7	9.15	ZSWIM1	XP_005260667.1	3	0	0.00
USP6NL	NP_055503.1	485	3	5.88	SPATA25	XP_024307594.1	61	5	77.86
ECHDC3	NP_078969.3	221	4	17.19	NEURL2	NP_542787.1	424	2	4.48
PROSER2	XP_016871517.1	20	2	95.00	CTSA	NP_001161066.1	1202	1	0.79
UPF2	NP_056357.1	982	3	2.90	PLTP	NP_001229849.1	502	3	5.68
DHTKD1	NP_061176.4	556	2	3.42	PCIF1	XP_016883502.1	222	2	8.56
SEC61A2	NP_001136099.1	1182	3	2.41	ZNF335	XP_005260561.1	327	4	11.62
NUDT5	NP_001308576.1	628	2	3.03	MMP9	NP_004985.2	2541	3	1.12
CDC123	NP_006014.2	428	2	4.44	SLC12A5	NP_001128243.1	1112	5	4.27
CAMK1D	NP_705718.1	1414	8	5.37	NCOA5	NP_066018.1	744	9	11.49
CCDC3	NP_113643.1	256	7	25.98	CD40	XP_016883624.1	1711	2	1.11
OPTN	NP_068815.2	853	5	5.57	CDH22	NP_067071.1	1078	9	7.93
MCM10	NP_877428.1	817	6	6.98	SLC35C2	NP_001268387.1	376	6	15.16
UCMA	NP_001290048.1	109	0	0.00	ELMO2	NP_573403.1	649	6	8.78
PHYH	NP_001310012.1	485	3	5.88	ZNF334	XP_016883426.1	134	10	70.89

SEPHS1	NP_001182533.1	439	7	15.15	OCSTAMP	XP_011526834.2	97	1	9.79
BEND7	XP_011517695.1	52	7	127.88	SLC13A3	NP_001180268.1	439	10	21.64
PRPF18	NP_003666.1	544	1	1.75	TP53RK	NP_291028.3	659	3	4.32
FRMD4A	XP_016871883.1	397	0	0.00	SLC2A10	XP_011527364.1	2412	10	3.94
FAM107B	NP_113641.2	236	1	4.03	EYA2	NP_005235.3	512	6	11.13
CDNF	NP_001025125.2	231	0	0.00	ZMYND8	NP_001268710.1	510	7	13.04
HSPA14	NP_057383.2	1928	4	1.97	NCOA3	NP_001167559.1	1149	3	2.48
SUV39H2	NP_001180353.1	1826	5	2.60	SULF2	XP_006723893.1	539	8	14.10
DCLRE1C	NP_001337894.1	710	3	4.01	PREX1	NP_065871.3	743	7	8.95
MEIG1	XP_024303904.1	176	1	5.40	ARFGEF2	NP_006411.2	921	10	10.31
OLAH	XP_016871865.1	178	0	0.00	CSE1L	NP_001307.2	1211	6	4.71
ACBD7	NP_001034933.1	260	2	7.31	STAU1	NP_001309858.1	681	3	4.18
RPP38	XP_016870968.1	1339	3	2.13	DDX27	NP_001335116.2	1448	7	4.59
NMT2	XP_016872439.1	473	4	8.03	ZNFX1	NP_066363.1	578	4	6.57
FAM171A1	XP_016871393.1	159	3	17.92	KCNB1	NP_004966.1	961	2	1.98
ITGA8	NP_001278423.1	710	3	4.01	PTGIS	NP_000952.1	569	1	1.67
MINDY3	XP_006717571.1	217	2	8.76	B4GALT5	NP_004767.1	320	3	8.91
PTER	XP_016872417.1	926	3	3.08	SLC9A8	XP_011527038.1	637	3	4.47
C1QL3	NP_001010908.1	265	1	3.58	SPATA2	NP_001129245.1	132	3	21.59
RSU1	XP_005252609.1	715	1	1.33	RNF114	NP_061153.1	513	12	22.22
CUBN	NP_001072.2	547	6	10.42	SNAI1	NP_005976.2	1587	4	2.39
TRDMT1	XP_005252431.1	767	3	3.72	PEDS1-UBE2V	NP_954673.2	831	1	1.14
VIM	XP_006717563.1	1639	6	3.48	UBE2V1	NP_001244322.1	832	2	2.28
ST8SIA6	XP_016871663.1	293	0	0.00	PEDS1	NP_001155977.2	197	5	24.11
HACD1	XP_005252698.1	520	1	1.83	CEBPB	NP_005185.2	1522	5	3.12
STAM	NP_003464.1	548	1	1.73	PTPN1	NP_002818.1	1439	3	1.98
TMEM236	XP_011517928.1	55	2	34.54	RIPOR3	XP_006723776.1	52	0	0.00
MRC1	NP_002429.1	999	6	5.71	PARD6B	NP_115910.1	646	5	7.35
SLC39A12	NP_001138667.1	787	6	7.24	BCAS4	XP_011527189.1	273	9	31.32
CACNB2	NP_963890.2	723	10	13.14	ADNP	XP_011527050.1	666	8	11.41
NSUN6	NP_001338045.1	817	8	9.30	DPM1	NP_001303965.1	1091	2	1.74
ARL5B	NP_848930.1	1426	8	5.33	MOCS3	NP_055299.1	641	3	4.45
MALRD1	XP_011517755.1	797	2	2.38	KCNG1	NP_002228.2	825	1	1.15
PLXDC2	NP_116201.7	332	5	14.31	NFATC2	NP_001245221.1	1021	4	3.72
NEBL	NP_998734.1	562	6	10.14	ATP9A	NP_006036.1	477	9	17.92
SKIDA1	XP_016871714.1	183	3	15.57	SALL4	NP_001304960.1	715	1	1.33
MLLT10	XP_024303947.1	694	10	13.69	ZFP64	XP_016883434.1	403	8	18.86
DNAJC1	NP_071760.2	868	6	6.57	TSHZ2	XP_016883130.1	418	10	22.73
EBLN1	NP_001186867.1	67	4	56.71	ZNF217	XP_024307765.1	772	11	13.54
COMMD3	NP_036203.1	1317	4	2.89	BCAS1	NP_001353226.1	471	7	14.12
COMMD3-BM	NP_001190991.1	404	5	11.76	CYP24A1	XP_016883182.1	618	12	18.45
BMI1	NP_005171.4	1458	3	1.95	PFDN4	NP_002614.2	722	11	14.47
SPAG6	NP_036575.1	916	5	5.19	DOK5	NP_060901.2	530	12	21.51
PIP4K2A	XP_006717513.1	770	11	13.57	CBLN4	NP_542184.1	950	10	10.00
ARMC3	NP_775104.2	330	8	23.03	MC3R	NP_063941.3	524	10	18.13
MSRB2	NP_036360.3	374	5	12.70	FAM210B	NP_543011.2	129	9	66.28
PTF1A	NP_835455.1	595	8	12.77	AURKA	XP_016883523.1	2181	12	5.23
C10orf67	NP_001352791.1	58	5	81.89	CSTF1	NP_001315.1	556	13	22.21
OTUD1	NP_001138845.1	321	9	26.63	CASS4	NP_001157588.1	217	8	35.02
KIAA1217	XP_011517854.2	326	5	14.57	RTF2	NP_001269964.1	279	12	40.86
ARHGAP21	NP_001354380.1	476	2	3.99	FAM209A	NP_001012989.2	58	13	212.92
PRTFDC1	NP_064585.1	407	1	2.33	FAM209B	XP_016883338.1	41	1	23.17
ENKUR	NP_001257312.1	276	4	13.77	TFAP2C	NP_003213.1	619	8	12.28
THNSL1	XP_016872154.1	349	3	8.17	BMP7	NP_001710.1	1470	6	3.88
GPR158	NP_065803.2	761	5	6.24	SPO11	XP_005260436.1	1212	10	7.84
MYO3A	XP_011517806.1	1731	3	1.65	RAE1	XP_005260640.1	1005	7	6.62
GAD2	NP_001127838.1	1847	4	2.06	MTRNR2L3	NP_001177401.1	7	2	271.41
APBB1IP	NP_061916.3	722	2	2.63	RBM38	NP_001278709.1	371	9	23.04
PDSS1	NP_055132.2	759	2	2.50	CTCFL	NP_001255972.1	834	5	5.70
ABI1	NP_001171596.1	858	6	6.64	PCK1	NP_002582.3	1056	12	10.79
ANKRD26	XP_016871418.1	920	4	4.13	ZBP1	NP_001153890.1	286	2	6.64
YME1L1	NP_001240795.1	1469	10	6.47	PMEPA1	NP_954638.1	329	6	17.32
MASTL	XP_016872342.1	699	5	6.80	C20orf85	NP_848551.1	91	1	10.44
ACBD5	XP_016872382.1	394	3	7.23	ANKRD60	NP_001291298.1	901	2	2.11
PTCHD3	NP_001030014.2	421	2	4.51	RAB22A	NP_065724.1	889	8	8.55
RAB18	NP_001243340.1	598	4	6.35	VAPB	NP_004729.1	1191	7	5.58

MKX	XP_016871594.1	444	3	6.42	APCDD1L	NP_699191.1	101	4	37.62
ARMC4	NP_001276950.1	338	2	5.62	STX16	NP_001001433.1	911	10	10.43
MPP7	XP_016871230.1	570	2	3.33	NPEPL1	NP_001191801.1	700	3	4.07
WAC	NP_057712.2	924	6	6.17	GNAS	XP_016883310.1	1735	11	6.02
BAMBI	NP_036474.1	402	1	2.36	NELFCD	NP_945327.3	660	7	10.08
LYZL1	XP_005252684.1	99	2	19.19	CTSZ	NP_001327.2	1010	13	12.23
SVIL	XP_024303906.1	554	2	3.43	TUBB1	NP_110400.1	1240	9	6.89
JCAD	NP_001336951.1	167	4	22.75	ATP5F1E	NP_008817.1	717	12	15.90
MTPAP	NP_060579.3	527	1	1.80	PRELID3B	NP_001243332.1	237	9	36.07
MAP3K8	NP_001307890.1	977	2	1.94	ZNF831	XP_016883131.1	372	7	17.88
LYZL2	XP_011517608.1	100	2	19.00	EDN3	XP_011526957.1	673	9	12.70
ZNF438	XP_016871352.1	258	0	0.00	PHACTR3	NP_001186434.1	594	9	14.39
ZEB1	NP_001167567.1	1379	4	2.76	SYCP2	XP_011526792.1	372	6	15.32
ARHGAP12	XP_016872443.1	582	1	1.63	PPP1R3D	NP_006233.1	245	6	23.26
KIF5B	NP_004512.1	1611	2	1.18	FAM217B	XP_011527287.1	77	3	37.01
EPC1	NP_001269320.1	554	2	3.43	CDH26	XP_016883483.1	312	3	9.13
CCDC7	NP_001308044.1	130	0	0.00	CDH4	NP_001785.2	586	6	9.73
ITGB1	NP_002202.2	1775	3	1.61	TAF4	NP_003176.2	950	8	8.00
NRP1	XP_006717588.1	887	2	2.14	LSM14B	XP_011526907.1	539	9	15.86
PARD3	NP_001171719.1	1166	4	3.26	PSMA7	NP_002783.1	1855	6	3.07
CUL2	XP_011518045.1	1563	1	0.61	SS18L1	NP_945173.1	296	10	32.09
CREM	XP_011517626.1	891	2	2.13	MTG2	XP_024307637.1	569	9	15.03
CCNY	NP_659449.3	430	4	8.84	HRH3	XP_005260323.1	1168	8	6.51
GJD4	NP_699199.2	147	3	19.39	OSBPL2	NP_653081.1	390	12	29.23
FZD8	NP_114072.1	695	5	6.83	ADRM1	XP_016883091.1	891	11	11.73
ANKRD30A	XP_011518059.1	941	2	2.02	LAMA5	XP_011527121.1	688	12	16.57
MTRNR2L7	NP_001177418.1	36	0	0.00	RPS21	XP_024307728.1	1117	7	5.95
ZNF248	NP_001339405.1	293	0	0.00	CABLES2	NP_112492.2	262	6	21.75
ZNF25	NP_001316584.1	323	0	0.00	RBBP8NL	NP_543023.2	173	6	32.95
ZNF33A	NP_001311107.1	229	1	4.15	GATA5	NP_536721.1	1036	3	2.75
ZNF37A	NP_001311187.1	232	0	0.00	MIR1-1HG	NP_848558.1	43	0	0.00
ZNF33B	NP_001291962.1	172	1	5.52	SLCO4A1	XP_005260260.1	338	2	5.62
BMS1	NP_055568.3	988	2	1.92	NTSR1	NP_002522.2	694	3	4.11
RET	NP_066124.1	1466	4	2.59	MRGBP	NP_060740.1	595	4	6.39
CSGALNACT2	NP_061060.3	258	1	3.68	OGFR	NP_031372.2	170	4	22.35
RASGEF1A	NP_001269791.1	219	4	17.35	COL9A3	NP_001844.3	411	2	4.62
FXYD4	NP_775183.1	203	1	4.68	TCFL5	XP_024307581.1	274	1	3.47
HNRNPF	NP_001091678.1	954	2	1.99	DIDO1	NP_149072.2	1077	3	2.65
ZNF487	NP_001342377.1	135	0	0.00	GID8	NP_060366.1	333	2	5.71
ZNF239	NP_001311282.1	270	0	0.00	SLC17A9	NP_071365.4	267	2	7.12
ZNF485	NP_660355.2	167	0	0.00	BHLHE23	NP_542173.2	193	2	9.84
ZNF32	NP_008904.1	185	1	5.13	YTHDF1	NP_060268.2	406	2	4.68
CXCL12	NP_001264919.1	1962	2	0.97	BIRC7	NP_647478.1	550	1	1.73
TMEM72	NP_001116848.1	167	0	0.00	NKAIN4	XP_005260249.1	176	3	16.19
RASSF4	XP_006718076.1	272	1	3.49	ARFGAP1	XP_016883439.1	934	5	5.09
DEPP1	NP_008952.1	219	1	4.34	COL20A1	XP_011527239.1	283	7	23.50
ZNF22	NP_008894.2	323	2	5.88	CHRNA4	NP_000735.1	1535	6	3.71
OR13A1	XP_016872121.1	68	0	0.00	KCNQ2	NP_004509.2	1604	6	3.55
ALOX5	NP_000689.1	953	4	3.99	EEF1A2	NP_001949.1	2072	10	4.58
MARCHF8	NP_659458.2	377	3	7.56	PPDPF	NP_077275.1	142	5	33.45
ZFAND4	XP_011538668.1	2319	6	2.46	PTK6	NP_005966.1	954	8	7.97
WASHC2C	NP_001317003.1	171	4	22.22	SRMS	NP_543013.1	813	9	10.52
FAM25E	NP_001338163.1	0	0	#DIV/0!	FNDC11	XP_016883549.1	77	8	98.70
AGAP4	NP_597703.2	388	2	4.90	HELZ2	NP_001032412.2	1833	4	2.07
TIMM23	NP_006318.1	749	5	6.34	GMEB2	XP_005260259.1	335	12	34.03
NCOA4	NP_001138733.1	500	3	5.70	STMN3	NP_056978.2	754	14	17.64
MSMB	NP_002434.1	426	5	11.15	RTEL1	NP_001269938.1	846	15	16.84
ANTXRL	NP_001265617.1	91	0	0.00	TNFRSF6B	NP_003814.1	230	6	24.78
ANXA8L1	NP_001265853.1	355	1	2.68	ARFRP1	XP_016883065.1	448	14	29.69
NPY4R	XP_016871875.1	641	4	5.93	ZGPAT	NP_115916.3	285	13	43.33
GPRIN2	XP_016872463.1	283	4	13.43	LIME1	NP_060276.2	155	10	61.29
SYT15	XP_024303996.1	314	5	15.13	SLC2A4RG	NP_064446.2	359	11	29.11
PTPN20	NP_001035817.1	600	3	4.75	ZBTB46	NP_001356670.1	351	10	27.06
GDF10	NP_004953.1	816	5	5.82	ABHD16B	NP_542189.1	290	10	32.76
GDF2	NP_057288.1	556	2	3.42	TPD52L2	NP_001230824.1	302	7	22.02
RBP3	NP_002891.1	411	3	6.93	DNAJC5	NP_079495.1	858	10	11.07

ZNF488	XP_016871132.1	456	4	8.33	UCKL1	XP_024307686.1	1056	3	2.70
ANXA8	XP_006718014.1	440	2	4.32	ZNF512B	XP_011527232.1	377	3	7.56
FAM25G	NP_001131021.1	60	0	0.00	SAMD10	XP_011526867.1	149	0	0.00
AGAP9	NP_001177739.1	444	0	0.00	PRPF6	NP_036601.2	1151	3	2.48
LOC10272448	XP_011538737.1	313	3	9.10	SOX18	NP_060889.1	541	1	1.76
NPY4R2	XP_016870952.1	641	2	2.96	TCEA2	NP_003186.1	764	3	3.73
FAM25C	NP_001131020.1	52	0	0.00	RGS19	XP_011526786.1	511	1	1.86
FRMPD2	NP_001018081.4	948	4	4.01	LKAAEAR1	XP_024307617.1	10	0	0.00
MAPK8	NP_001310258.1	3887	2	0.49	OPRL1	XP_016883342.1	929	3	3.07
ARHGAP22	XP_024303868.1	312	7	21.31	NPBWR2	NP_005277.2	558	2	3.40
WDFY4	XP_011538288.2	1538	6	3.71	MYT1	NP_004526.1	671	1	1.42
LRRC18	XP_016871765.1	465	7	14.30	PCMTD2	NP_060727.2	294	2	6.46
VSTM4	NP_001026916.2	291	4	13.06	LOC1027239	XP_011544380.1	409	0	0.00
FAM170B	NP_001157956.1	21	8	361.89	GATD3B	NP_001350689.1	347	0	0.00
TMEM273	XP_016871282.1	54	1	17.59	LOC1027241	NP_001355167.1	730	0	0.00
C10orf71	NP_001128668.1	50	3	57.00	LOC1027242	XP_011544384.3	191	0	0.00
DRGX	NP_001263380.1	504	9	16.96	LOC1027242	XP_011544387.2	0	0	#DIV/0!
ERCC6	NP_001333369.1	1318	10	7.21	LOC1027243	XP_006723981.1	1139	0	0.00
PGBD3	NP_736609.2	1	0	0.00	SIK1B	NP_001307572.1	234	0	0.00
SLC18A3	NP_003046.2	753	8	10.09	CBSL	NP_001308002.1	1675	2	1.33
CHAT	NP_065574.4	922	2	2.06	U2AF1L5	XP_016883708.1	655	1	1.61
C10orf53	NP_872360.2	22	3	129.54	CRYAA2	NP_001300979.1	420	1	2.38
OGDHL	NP_001334749.1	896	5	5.30	LOC1027248	XP_016883710.1	0	0	#DIV/0!
PARG	XP_011538607.1	329	2	5.77	LOC1027249	XP_011544409.2	0	0	#DIV/0!
TIMM23B	NP_001277046.1	547	1	1.74	LOC1027233	NP_001309904.1	0	0	#DIV/0!
AGAP6	NP_001071133.2	337	0	0.00	SMIM11B	XP_016883693.1	50	0	0.00
WASHC2A	NP_001005751.1	159	2	11.95	FAM243B	NP_001351640.1	54	0	0.00
ASAH2	XP_011538274.1	243	2	7.82	KCNE1B	XP_006724009.1	88	0	0.00
SGMS1	NP_671512.1	370	3	7.70	TPTE	NP_954870.3	882	7	7.54
ASAH2B	NP_001308886.1	198	1	4.80	POTED	NP_778146.2	995	5	4.77
A1CF	NP_001357059.1	479	1	1.98	LIPI	NP_001289930.1	265	8	28.68
PRKG1	NP_001091982.1	2061	3	1.38	RBM11	NP_001307531.1	252	9	33.93
CSTF2T	NP_056050.1	980	3	2.91	HSPA13	NP_008879.3	1609	12	7.08
DKK1	NP_036374.1	1210	1	0.79	SAMSN1	NP_001243299.1	663	12	17.19
MBL2	NP_001365302.1	735	1	1.29	LOC388813	NP_001243508.2	180	5	26.39
PCDH15	NP_001341349.1	731	2	2.60	NRIP1	XP_016883962.1	637	7	10.44
MTRNR2L5	NP_001177407.1	58	0	0.00	USP25	NP_001269970.1	1045	12	10.91
ZWINT	NP_001005413.1	695	2	2.73	CXADR	XP_011527778.1	398	8	19.09
IPMK	NP_689416.1	765	2	2.48	BTG3	XP_024307819.1	518	6	11.00
CISD1	NP_060934.1	416	1	2.28	C21orf91	NP_059143.3	195	7	34.10
UBE2D1	NP_003329.1	1979	3	1.44	CHODL	XP_011527755.1	388	7	17.14
TFAM	NP_003192.1	1393	4	2.73	TMPRSS15	NP_002763.3	418	7	15.91
BICC1	XP_011538487.1	785	2	2.42	NCAM2	NP_001339520.1	729	4	5.21
PHYHIPL	NP_115815.2	718	2	2.65	MRPL39	NP_059142.3	1019	7	6.53
FAM13C	XP_005269676.2	177	1	5.37	JAM2	NP_067042.1	249	4	15.26
SLC16A9	NP_001310910.1	386	2	4.92	ATP5PF	NP_001003701.1	962	5	4.94
CCDC6	NP_005427.2	446	3	6.39	GABPA	XP_016883802.1	838	8	9.07
ANK3	NP_001191332.1	2273	4	1.67	APP	NP_001129601.1	3501	6	1.63
CDK1	NP_001307847.1	3505	7	1.90	CYYR1	NP_443186.3	159	3	17.92
RHOBTB1	XP_016872489.1	1729	6	3.30	ADAMTS1	NP_008919.3	757	4	5.02
TMEM26	NP_848600.2	163	4	23.31	ADAMTS5	XP_024307822.1	515	1	1.84
CABCOCO1	NP_001353835.1	126	4	30.16	N6AMT1	NP_877426.4	579	4	6.56
ARID5B	NP_115575.1	735	4	5.17	LTN1	NP_001307695.2	810	5	5.86
RTKN2	XP_016871332.1	270	6	21.11	RWDD2B	NP_058636.1	106	2	17.92
ZNF365	XP_016871426.1	419	4	9.07	USP16	XP_016883746.1	553	4	6.87
ADO	NP_116193.2	266	4	14.28	CCT8	NP_006576.2	1481	3	1.92
EGR2	XP_011537729.1	1428	9	5.99	MAP3K7CL	NP_001358298.1	58	0	0.00
NRBF2	NP_001269334.1	136	5	34.92	BACH1	NP_001177.1	249	1	3.82
JMJD1C	NP_001309181.1	618	5	7.69	GRIK1	NP_001307550.1	745	1	1.28
REEP3	NP_001001330.1	738	4	5.15	CLDN17	NP_036263.1	230	1	4.13
CTNNA3	XP_016871642.1	1120	2	1.70	CLDN8	NP_955360.1	400	1	2.37
LRRTM3	NP_821079.3	991	2	1.92	KRTAP24-1	NP_001078924.1	188	0	0.00
DNAJC12	NP_068572.1	416	7	15.98	KRTAP25-1	NP_001122070.1	143	0	0.00
SIRT1	NP_036370.2	3006	10	3.16	KRTAP26-1	NP_981950.1	233	0	0.00
HERC4	XP_016871530.1	891	9	9.60	KRTAP27-1	NP_001071179.1	283	0	0.00
MYPN	XP_016872322.1	409	9	20.90	KRTAP23-1	NP_853655.1	149	0	0.00

ATOH7	NP_660161.1	605	9	14.13	KRTAP13-2	NP_853652.1	165	0	0.00
PBLD	XP_005270085.1	311	13	39.71	KRTAP13-1	NP_853630.2	197	0	0.00
HNRNPH3	NP_001309366.1	809	11	12.92	KRTAP13-3	NP_853653.1	172	0	0.00
RUFY2	XP_005270014.1	255	12	44.70	KRTAP13-4	NP_853631.1	161	0	0.00
DNA2	XP_006717743.1	1301	12	8.76	KRTAP15-1	NP_853654.1	175	0	0.00
SLC25A16	NP_689920.1	643	8	11.82	KRTAP19-1	NP_853638.1	152	0	0.00
TET1	XP_011538506.1	683	5	6.95	KRTAP19-2	NP_853639.1	95	0	0.00
CCAR1	NP_060707.2	754	11	13.86	KRTAP19-3	NP_853640.1	150	0	0.00
STOX1	NP_001123633.1	295	15	48.30	KRTAP19-4	NP_853641.1	149	0	0.00
DDX50	XP_016872115.1	1521	15	9.37	KRTAP19-5	NP_853642.1	169	0	0.00
DDX21	NP_004719.2	2035	10	4.67	KRTAP19-6	NP_853643.1	136	0	0.00
KIFBP	NP_056449.1	237	1	4.01	KRTAP19-7	NP_853645.1	131	0	0.00
SRGN	NP_001307982.1	787	3	3.62	KRTAP22-2	NP_001157906.1	138	0	0.00
VPS26A	NP_004887.2	863	11	12.11	KRTAP6-3	NP_853636.3	129	0	0.00
SUPV3L1	NP_003162.2	382	11	27.35	KRTAP6-2	NP_853635.1	153	0	0.00
HKDC1	NP_079406.4	734	7	9.06	KRTAP22-1	NP_853651.1	144	0	0.00
HK1	NP_001309294.1	1507	2	1.26	KRTAP6-1	NP_853633.1	151	0	0.00
TACR2	NP_001048.2	394	8	19.29	KRTAP20-1	NP_853646.1	131	0	0.00
TSPAN15	XP_016871499.1	173	10	54.91	KRTAP20-4	NP_001337906.1	126	0	0.00
NEUROG3	XP_016871769.1	548	4	6.93	KRTAP20-2	NP_853647.1	134	0	0.00
FAM241B	XP_005269663.1	22	7	302.26	KRTAP20-3	NP_001121549.1	129	0	0.00
COL13A1	NP_001355811.1	386	9	22.15	KRTAP21-3	NP_001157907.1	130	0	0.00
MACROH2A2	NP_061119.1	978	3	2.91	KRTAP21-2	NP_853648.1	138	0	0.00
AIFM2	NP_116186.1	596	3	4.78	KRTAP21-1	NP_853650.1	139	0	0.00
TYSND1	NP_775826.2	501	2	3.79	KRTAP8-1	NP_787053.1	270	1	3.52
SAR1A	NP_001136120.1	766	0	0.00	KRTAP7-1	NP_853637.2	179	0	0.00
PPA1	NP_066952.1	1499	1	0.63	KRTAP11-1	NP_787054.1	219	0	0.00
NPFFR1	NP_071429.1	468	6	12.18	KRTAP19-8	NP_001092689.1	135	0	0.00
LRRC20	NP_060709.2	435	5	10.92	TIAM1	NP_001340616.1	1002	7	6.64
EIF4EBP2	NP_004087.1	593	4	6.41	SOD1	NP_000445.1	2824	3	1.01
NODAL	NP_060525.3	636	4	5.97	SCAF4	NP_001138916.1	779	3	3.66
PALD1	XP_016871561.1	166	3	17.17	HUNK	NP_055401.1	1247	9	6.86
PRF1	NP_001076585.1	1179	2	1.61	MIS18A	XP_016883891.1	344	4	11.05
ADAMTS14	NP_631894.2	318	6	17.92	MRAP	XP_016883896.1	1382	5	3.44
TBATA	XP_016871349.1	133	2	14.28	URB1	NP_055640.2	435	6	13.10
SGPL1	NP_003892.2	721	6	7.91	EVA1C	NP_478067.2	398	4	9.55
PCBD1	NP_001309933.1	407	3	7.00	TCP10L	NP_653260.1	82	0	0.00
UNC5B	NP_734465.2	532	6	10.71	CFAP298	NP_001337265.1	210	4	18.09
SLC29A3	NP_060814.4	390	5	12.18	SYNJ1	XP_016883986.1	1096	11	9.53
CDH23	NP_071407.4	738	2	2.57	PAXBP1	XP_006724129.1	511	13	24.17
C10orf105	NP_001157847.1	35	1	27.14	C21orf62	NP_001155967.2	33	7	201.50
VSIR	NP_071436.1	256	1	3.71	OLIG2	NP_005797.1	1261	6	4.52
PSAP	NP_002769.1	710	0	0.00	OLIG1	NP_620450.2	939	12	12.14
CHST3	NP_004264.2	327	2	5.81	IFNAR2	NP_001371984.1	397	10	23.93
SPOCK2	XP_011538706.1	615	3	4.63	IL10RB	NP_000619.3	605	12	18.84
ASCC1	NP_001356028.1	950	3	3.00	IFNAR1	NP_001371427.1	736	8	10.33
ANAPC16	NP_001353720.1	325	0	0.00	IFNGR2	NP_001316057.1	460	14	28.91
DDIT4	NP_061931.1	641	1	1.48	TMEM50B	XP_011528048.1	205	16	74.14
DNAJB12	NP_001352009.1	497	0	0.00	DNAJC28	NP_001307675.1	196	1	4.85
MICU1	NP_001182447.1	447	3	6.38	GART	NP_001129477.1	2498	14	5.32
MCU	NP_001257608.1	571	3	4.99	SON	NP_620305.3	629	7	10.57
OIT3	NP_689848.1	110	3	25.91	DONSON	NP_060083.1	515	16	29.51
PLA2G12B	XP_024304003.1	454	3	6.28	CRYZL1	NP_665857.2	591	13	20.90
P4HA1	NP_001136068.1	620	10	15.32	ITSN1	XP_016883917.1	1432	13	8.62
NUDT13	NP_056985.3	175	8	43.43	ATP5PO	NP_001688.1	1086	10	8.75
ECD	NP_001129225.1	253	0	0.00	MRPS6	NP_115865.1	838	11	12.47
FAM149B1	NP_775483.1	89	9	96.06	SLC5A3	NP_008864.4	416	10	22.84
DNAJC9	NP_056005.1	816	9	10.48	KCNE2	NP_751951.1	334	5	14.22
MRPS16	NP_057149.1	1105	10	8.60	SMIM11A	NP_478062.1	50	6	113.99
CFAP70	XP_016871116.1	127	8	59.84	FAM243A	NP_001269466.1	54	0	0.00
ANXA7	XP_016871652.1	862	4	4.41	SMIM34A	NP_001354277.1	46	0	0.00
MSS51	NP_001019764.1	227	10	41.85	KCNE1	NP_001121140.1	464	5	10.24
PPP3CB	XP_005270001.1	2976	10	3.19	RCAN1	NP_004405.3	731	13	16.89
USP54	NP_001337924.1	306	9	27.94	CLIC6	NP_001303938.1	642	7	10.36
MYOZ1	NP_067068.1	381	5	12.47	RUNX1	XP_011528070.1	800	5	5.94
SYNPO2L	NP_001107605.1	676	4	5.62	SETD4	XP_011527938.1	368	7	18.07

AGAP5	NP_001137472.1	390	3	7.31	CBR1	NP_001748.1	633	10	15.01
SEC24C	XP_011538682.1	632	1	1.50	CBR3	NP_001227.1	499	13	24.75
FUT11	XP_006717719.1	217	3	13.13	DOP1B	NP_001307643.1	523	13	23.61
CHCHD1	NP_976043.1	482	0	0.00	MORC3	NP_056173.1	365	7	18.22
ZSWIM8	XP_005269705.1	128	0	0.00	CHAF1B	XP_016883966.1	945	10	10.05
NDST2	NP_003626.1	274	2	6.93	CLDN14	NP_652763.1	328	10	28.96
CAMK2G	NP_001354445.1	1081	8	7.03	SIM2	NP_005060.1	484	13	25.52
PLAU	NP_002649.2	1236	3	2.31	HLCS	NP_001339443.1	696	14	19.11
VCL	NP_054706.1	1795	7	3.70	RIPPLY3	NP_061835.1	163	11	64.11
AP3M1	NP_001307194.1	418	7	15.91	PIGP	NP_710148.1	242	18	70.66
ADK	NP_006712.2	1037	8	7.33	TTC3	XP_016883947.1	589	19	30.64
KAT6B	NP_036462.2	1064	6	5.36	VPS26C	NP_001317947.1	274	16	55.47
DUSP29	NP_001371838.1	737	8	10.31	DYRK1A	NP_001387.2	1438	19	12.55
DUSP13	NP_001007273.1	748	8	10.16	KCNJ6	NP_002231.1	750	15	19.00
SAMD8	XP_016871227.1	212	8	35.85	KCNJ15	XP_016883832.1	455	13	27.14
VDAC2	NP_001311017.1	1106	6	5.15	ERG	NP_001230361.1	369	1	2.57
COMTD1	XP_024303559.1	559	7	11.90	ETS2	NP_001243224.1	1111	16	13.68
ZNF503	NP_116161.2	269	5	17.66	PSMG1	NP_982257.1	762	19	23.69
LRMDA	NP_001292510.1	674	6	8.46	BRWD1	XP_011527913.1	950	13	13.00
KCNMA1	XP_024303758.1	1311	2	1.45	HMGN1	XP_016883822.1	426	15	33.45
DLG5	XP_005270333.1	1512	5	3.14	GET1	NP_004618.2	429	9	19.93
POLR3A	NP_008986.2	1886	4	2.01	LCA5L	NP_001371216.1	66	11	158.33
RPS24	NP_001135757.1	1149	2	1.65	SH3BGR	NP_031367.2	300	16	50.66
ZMIZ1	XP_005270044.1	932	4	4.08	B3GALT5	NP_149362.2	355	14	37.46
PPIF	NP_005720.1	1629	7	4.08	IGSF5	XP_011527774.1	160	10	59.37
ZCCHC24	NP_699198.2	212	7	31.37	PCP4	NP_006189.2	645	9	13.26
EIF5AL1	NP_001093162.1	914	8	8.31	DSCAM	NP_001258463.1	998	15	14.28
SFTPA2	XP_005270185.1	416	7	15.98	BACE2	NP_036237.2	494	14	26.92
SFTPA1	NP_001087239.2	390	9	21.92	FAM3B	XP_011527951.1	259	12	44.01
NUTM2B	NP_001265424.1	40	0	0.00	MX2	NP_002454.1	1397	8	5.44
NUTM2E	NP_001342192.1	16	0	0.00	MX1	NP_001269849.1	1851	9	4.62
SFTPD	XP_011538390.1	514	8	14.79	TMPRSS2	NP_001369649.1	729	15	19.55
TMEM254	NP_079401.2	74	14	179.72	RIPK4	NP_065690.2	3798	13	3.25
PLAC9	NP_001318054.1	127	14	104.72	PRDM15	XP_011527976.1	303	12	37.62
ANXA11	NP_665875.1	626	10	15.17	C2CD2	NP_056315.1	182	11	57.41
MAT1A	NP_000420.1	1351	10	7.03	ZBTB21	NP_001307658.1	428	10	22.20
DYDC1	XP_016871238.1	95	10	99.99	UMODL1	NP_775839.3	229	12	49.78
DYDC2	NP_001256971.1	113	10	84.07	ABCG1	NP_997510.1	849	12	13.43
PRXL2A	XP_016872264.1	198	8	38.38	TFF3	NP_003217.4	529	12	21.55
TSPAN14	XP_024303979.1	385	8	19.74	TFF2	NP_005414.1	328	11	31.86
SH2D4B	NP_997255.2	136	9	62.86	TFF1	NP_003216.1	653	13	18.91
NRG3	XP_016871062.1	907	2	2.09	TMPRSS3	NP_001243246.1	389	17	41.51
GHITM	NP_055209.2	420	5	11.31	UBASH3A	NP_061834.1	664	15	21.46
C10orf99	XP_005269868.1	73	5	65.07	RSPH1	NP_001273435.1	472	14	28.18
CDHR1	NP_001165442.1	475	8	16.00	SLC37A1	NP_001307466.1	402	13	30.72
LRIT2	NP_001017924.1	478	5	9.94	PDE9A	NP_001001570.1	486	16	31.27
LRIT1	NP_056428.1	891	7	7.46	WDR4	XP_016883751.1	755	18	22.65
RGR	NP_002912.2	251	6	22.71	NDUFV3	XP_011527888.1	400	13	30.87
CCSER2	XP_016871829.1	60	0	0.00	PKNOX1	NP_004562.2	528	15	26.99
GRID1	NP_060021.1	806	5	5.89	CBS	XP_016883980.1	1657	14	8.03
WAPL	NP_001305257.1	579	2	3.28	U2AF1	XP_011528045.1	683	12	16.69
OPN4	NP_001025186.1	847	7	7.85	CRYAA	NP_000385.1	411	12	27.74
LDB3	NP_001165081.1	1052	3	2.71	SIK1	XP_011527776.1	429	1	2.21
BMPR1A	NP_004320.2	1666	7	3.99	HSF2BP	XP_016883758.1	203	10	46.80
MMRN2	NP_079032.2	177	6	32.20	H2BS1	NP_059141.1	1139	3	2.50
SNCG	NP_003078.2	575	5	8.26	RRP1B	NP_055871.1	998	11	10.47
ADIRF	NP_006820.1	219	4	17.35	PDXK	NP_003672.1	742	10	12.80
AGAP11	NP_597704.1	390	4	9.74	CSTB	NP_000091.1	424	13	29.13
FAM25A	NP_001139629.1	60	4	63.33	RRP1	NP_003674.1	797	8	9.54
GLUD1	NP_005262.1	1144	1	0.83	AGPAT3	XP_016883897.1	573	3	4.97
SHLD2	XP_016871838.1	42	0	0.00	TRAPPC10	XP_011528016.1	530	5	8.96
NUTM2A	XP_016872096.1	38	0	0.00	PWP2	NP_005040.2	850	6	6.71
NUTM2D	NP_001009610.1	24	0	0.00	GATD3A	NP_004640.4	425	8	17.88
MINPP1	NP_004888.2	309	7	21.52	ICOSLG	XP_011527816.1	405	2	4.69
PAPSS2	NP_001015880.1	679	10	13.99	DNMT3L	NP_787063.1	550	3	5.18
ATAD1	NP_001308897.1	991	12	11.50	AIRE	NP_000374.1	1400	10	6.79

KLLN	NP_001119521.1	112	11	93.30	PFKL	XP_005261192.1	1069	9	8.00
PTEN	NP_001291646.4	4165	7	1.60	CFAP410	XP_016883959.1	251	3	11.35
RNLS	XP_016871871.1	178	11	58.70	TRPM2	XP_016883945.1	470	2	4.04
LIPJ	XP_016871229.1	321	8	23.67	LRRC3	NP_112153.1	475	2	4.00
LIPF	NP_001185758.1	526	7	12.64	TSPEAR	NP_659428.2	267	5	17.79
LIPK	XP_011538375.1	290	11	36.03	KRTAP10-1	NP_941964.2	227	1	4.18
LIPN	NP_001095939.1	499	8	15.23	KRTAP10-2	NP_941966.1	174	0	0.00
LIPM	XP_011538050.1	390	13	31.67	KRTAP10-3	NP_941969.2	191	0	0.00
ANKRD22	NP_653191.2	1115	17	14.48	KRTAP10-4	NP_941960.2	194	0	0.00
STAMBPL1	XP_016871942.1	900	17	17.94	KRTAP10-5	NP_941967.3	207	1	4.59
ACTA2	NP_001307784.1	2419	9	3.53	KRTAP10-6	NP_941961.3	190	0	0.00
FAS	XP_011538066.1	1476	7	4.51	KRTAP10-7	NP_941962.1	79	0	0.00
CH25H	NP_003947.1	591	18	28.93	KRTAP10-8	NP_941968.2	201	0	0.00
LIPA	XP_024303791.1	536	7	12.41	KRTAP10-9	NP_941963.2	195	0	0.00
IFIT2	NP_001538.4	1127	8	6.74	KRTAP10-10	NP_859016.1	67	0	0.00
IFIT3	NP_001540.2	1175	11	8.89	KRTAP10-11	NP_941965.2	197	0	0.00
IFIT1B	NP_001010987.1	856	10	11.10	KRTAP12-4	NP_941971.1	183	0	0.00
IFIT1	NP_001539.3	1229	8	6.18	KRTAP12-3	NP_941970.2	197	0	0.00
IFIT5	NP_036552.1	951	8	7.99	KRTAP12-2	NP_859012.1	200	0	0.00
SLC16A12	XP_016871726.1	302	12	37.75	KRTAP12-1	NP_859014.1	224	0	0.00
PANK1	NP_683878.1	368	8	20.65	KRTAP10-12	NP_941972.1	186	0	0.00
KIF20B	NP_001271188.1	1351	11	7.73	UBE2G2	NP_003334.2	679	0	0.00
HTR7	NP_062873.1	826	1	1.15	SUMO3	NP_008867.2	2040	3	1.40
RPP30	NP_001098016.1	476	0	0.00	PTTG1IP	NP_001273751.1	254	2	7.48
ANKRD1	NP_055206.2	1484	3	1.92	ITGB2	NP_000202.3	1764	3	1.62
PCGF5	XP_016872265.1	372	1	2.55	FAM207A	NP_478070.1	107	0	0.00
HECTD2	NP_001271203.2	739	2	2.57	ADARB1	XP_016883735.1	483	5	9.83
PPP1R3C	NP_005389.1	571	2	3.33	POFUT2	NP_598368.2	455	9	18.79
TNKS2	XP_005270242.2	1375	4	2.76	COL18A1	NP_001366429.1	897	11	11.65
FGFBP3	NP_689642.3	203	1	4.68	SLC19A1	XP_016883934.1	395	9	21.64
BTAF1	NP_003963.1	864	1	1.10	PCBP3	XP_024307864.1	521	11	20.06
CPEB3	XP_011537819.1	364	0	0.00	COL6A1	NP_001839.2	921	7	7.22
MARCHF5	NP_060294.1	321	0	0.00	COL6A2	NP_001840.3	748	10	12.70
IDE	NP_001309723.1	841	4	4.52	FTCD	XP_011527737.1	536	15	26.58
KIF11	NP_004514.2	1979	5	2.40	SPATC1L	XP_016883969.1	130	15	109.61
HHEX	NP_002720.1	1049	3	2.72	LSS	NP_001138908.1	739	7	9.00
EXOC6	NP_001306123.1	727	4	5.23	MCM3AP	NP_003897.2	807	12	14.13
CYP26C1	NP_899230.2	430	7	15.46	YBEY	XP_016883883.1	431	11	24.24
CYP26A1	NP_000774.2	729	5	6.52	C21orf58	XP_006724081.1	99	12	115.15
MYOF	NP_579899.1	737	6	7.73	PCNT	XP_011527896.1	953	9	8.97
CEP55	NP_060601.4	724	2	2.62	DIP2A	XP_016883780.1	439	10	21.64
FFAR4	NP_859529.2	547	0	0.00	S100B	XP_016883913.1	1539	6	3.70
RBP4	NP_001310447.1	466	4	8.15	PRMT2	NP_001273605.1	1081	9	7.91
PDE6C	NP_006195.3	508	6	11.22	OR11H1	NP_001005239.1	84	3	67.85
FRA10AC1	NP_001334641.1	99	2	19.19	POTEH	NP_001129685.1	945	5	9.14
LGI1	NP_005088.1	652	4	5.83	CCT8L2	NP_055221.1	559	4	11.33
SLC35G1	XP_011537675.1	243	2	7.82	XKR3	XP_016884089.1	113	6	77.60
PLCE1	XP_016871799.1	788	6	7.23	GAB4	NP_001353786.1	237	3	17.18
NOC3L	NP_071896.8	954	5	4.98	IL17RA	NP_055154.3	544	1	2.33
TBC1D12	NP_056003.1	334	6	17.06	TMEM121B	XP_011544427.1	114	6	62.50
HELLS	XP_024303736.1	1703	8	4.46	HDHD5	NP_060299.4	216	0	0.00
CYP2C18	NP_000763.1	478	5	9.94	ADA2	NP_001269154.1	718	7	10.29
CYP2C19	NP_000760.1	525	5	9.05	CECR2	NP_001276976.1	1264	8	6.33
CYP2C9	NP_000762.2	618	5	7.69	SLC25A18	XP_011544451.1	630	4	6.03
CYP2C8	NP_000761.3	498	3	5.72	ATP6V1E1	NP_001034455.1	633	3	4.50
ACSM6	NP_997204.2	251	7	26.49	BCL2L13	NP_001257655.1	318	1	2.99
PDLIM1	NP_066272.1	877	3	3.25	BID	NP_932070.1	251	1	3.78
SORBS1	NP_006425.3	889	8	8.55	MICAL3	NP_056056.2	687	3	4.15
ALDH18A1	NP_001310346.1	1946	4	1.95	PEX26	NP_001121121.1	169	2	11.24
TCTN3	XP_024303703.1	219	0	0.00	TUBA8	NP_061816.1	1448	5	3.28
ENTPD1	NP_001091645.1	802	8	9.48	USP18	NP_059110.2	859	6	6.64
CC2D2B	NP_001335937.1	63	2	30.16	GGTLC3	NP_001342408.1	1213	2	1.57
CCNJ	XP_016871843.1	189	1	5.03	TMEM191B	XP_024308033.1	58	2	32.76
ZNF518A	XP_016872475.1	209	0	0.00	RIMBP3	NP_056487.1	228	5	20.83
BLNK	NP_001245371.1	791	2	2.40	DGCR6	NP_005666.2	197	10	48.22
DNTT	NP_004079.3	1356	3	2.10	PRODH	NP_057419.5	717	13	17.22

OPALIN	NP_001035191.1	964	1	0.99	DGCR2	NP_001167005.1	320	10	29.69
TLL2	NP_036597.1	358	3	7.96	TSSK2	NP_443732.3	1186	1	0.80
TM9SF3	NP_064508.3	463	1	2.05	ESS2	NP_073210.1	586	12	19.45
PIK3AP1	XP_011537550.1	619	2	3.07	GSC2	NP_005306.1	445	13	27.75
LCOR	NP_001333445.1	237	1	4.01	SLC25A1	NP_005975.1	686	14	19.39
SLIT1	NP_003052.2	1416	0	0.00	CLTCL1	XP_016884443.1	1359	15	10.49
ARHGAP19	NP_001191229.1	437	1	2.17	HIRA	NP_003316.3	971	14	13.70
FRAT1	NP_005470.2	364	2	5.22	MRPL40	NP_003767.2	756	15	18.85
FRAT2	NP_036215.1	395	2	4.81	C22orf39	NP_001159714.2	64	3	44.53
RRP12	XP_024303688.1	904	2	2.10	UFD1	NP_005650.2	869	17	18.58
PGAM1	NP_002620.1	858	1	1.11	CDC45	XP_011528718.1	1228	18	13.92
EXOSC1	NP_001305293.1	809	2	2.35	CLDN5	NP_001349996.1	735	15	19.39
ZDHHC16	XP_006718084.1	203	3	14.04	SEPTIN5	NP_002679.2	1217	17	13.27
MMS19	XP_016872007.1	690	3	4.13	SEPT5-GP1BB	NP_001009939.1	1217	17	13.27
UBTD1	NP_079230.1	88	2	21.59	GP1BB	NP_000398.1	588	13	21.00
ANKRD2	NP_001278147.1	1140	0	0.00	TBX1	XP_016884417.1	1355	17	11.92
HOGA1	NP_612422.2	582	4	6.53	GNB1L	NP_443730.1	702	16	21.65
C10orf62	NP_001009997.2	63	0	0.00	RTL10	NP_078903.3	325	0	0.00
MORN4	XP_011537552.1	213	3	13.38	TXNRD2	NP_001339229.1	1059	15	13.46
PI4K2A	NP_060895.1	815	3	3.50	COMT	NP_001349757.1	1341	17	12.04
AVPI1	XP_016871983.1	159	4	23.90	ARVCF	XP_011528481.1	706	16	21.53
MARVELD1	NP_113672.1	241	5	19.71	TANGO2	NP_001309071.1	254	12	44.88
ZFYVE27	NP_001372805.1	288	1	3.30	DGCR8	NP_073557.3	723	15	19.71
SFRP5	NP_003006.2	596	0	0.00	TRMT2A	NP_892029.2	683	12	16.69
GOLGA7B	NP_001010917.1	311	1	3.05	RANBP1	NP_001265568.1	1168	13	10.57
CRTAC1	XP_011538219.1	719	2	2.64	ZDHHC8	NP_001171953.1	394	15	36.17
R3HCC1L	NP_001243550.2	348	0	0.00	CCDC188	XP_011528472.1	0	0	#DIV/0!
LOXL4	NP_115587.6	272	4	13.97	RTN4R	NP_075380.1	1081	12	10.55
PYROXD2	NP_116098.2	413	1	2.30	DGCR6L	NP_150282.2	216	15	65.97
HPS1	NP_001309411.1	110	0	0.00	USP41	XP_016884653.1	574	1	1.65
HPSE2	NP_001159717.1	269	4	14.13	ZNF74	NP_003417.2	235	16	64.68
CNNM1	NP_001332816.1	472	2	4.03	SCARF2	NP_878315.2	365	12	31.23
GOT1	NP_002070.1	702	1	1.35	KLHL22	XP_016884507.1	523	12	21.80
NKX2-3	NP_660328.2	743	3	3.84	MED15	NP_001003891.1	779	15	18.29
SLC25A28	NP_112489.3	342	6	16.67	PI4KA	XP_005261692.1	1915	15	7.44
ENTPD7	NP_001336892.1	162	7	41.05	SERPIND1	NP_000176.2	556	13	22.21
COX15	NP_001307903.1	576	3	4.95	SNAP29	NP_004773.1	756	14	17.59
CUTC	NP_057044.2	202	5	23.51	CRKL	NP_005198.1	972	13	12.71
ABCC2	NP_000383.2	953	3	2.99	AIFM3	NP_001139760.1	689	13	17.92
DNMBP	XP_006717798.1	862	1	1.10	LZTR1	NP_006758.2	265	13	46.60
CPN1	NP_001299.1	379	4	10.03	THAP7	NP_085050.2	170	12	67.06
ERLIN1	NP_001334786.1	406	4	9.36	P2RX6	XP_011528800.1	120	11	87.08
CHUK	NP_001269.3	1778	7	3.74	SLC7A4	NP_004164.2	509	12	22.40
CWF19L1	NP_001290333.1	286	4	13.29	LRRC74B	XP_011528475.1	53	0	0.00
BLOC1S2	NP_001269368.1	520	4	7.31	GGT2	XP_016884675.1	1221	0	0.00
PKD2L1	NP_057196.2	459	10	20.70	RIMBP3B	NP_001122107.1	164	2	11.58
SCD	NP_005054.3	1303	3	2.19	HIC2	NP_055909.2	389	5	12.21
WNT8B	NP_003384.2	768	8	9.90	TMEM191C	NP_001193981.1	58	2	32.76
SEC31B	NP_056305.1	620	7	10.73	RIMBP3C	NP_001122105.1	157	2	12.10
NDUFB8	NP_004995.1	710	5	6.69	UBE2L3	NP_001243284.1	1624	8	4.68
HIF1AN	XP_011538242.1	697	6	8.18	YDJC	NP_001017964.1	128	1	7.42
PAX2	NP_001291498.1	1282	8	5.93	CCDC116	XP_006724222.1	127	0	0.00
SLF2	NP_060591.3	220	3	12.95	SDF2L1	NP_071327.2	444	4	8.56
SEMA4G	NP_001190173.1	441	4	8.62	PPIL2	XP_011528345.1	835	5	5.69
MRPL43	NP_789762.1	523	2	3.63	YPEL1	NP_037445.1	280	6	20.36
TWNK	NP_068602.2	504	4	7.54	MAPK1	NP_620407.1	4704	9	1.82
LZTS2	NP_115805.1	275	2	6.91	PPM1F	NP_055449.1	1023	8	7.43
PDZD7	XP_011538480.1	649	1	1.46	TOP3B	NP_001336774.1	942	4	4.03
SFXN3	NP_112233.2	239	2	7.95	VPREB1	NP_001290438.1	783	10	12.13
KAZALD1	XP_016872204.1	152	9	56.25	ZNF280B	NP_542942.2	329	6	17.32
TLX1	XP_011538046.1	655	9	13.05	ZNF280A	NP_542778.2	260	6	21.92
LBX1	NP_006553.2	744	8	10.21	PRAME	NP_996836.1	555	5	8.56
BTRC	XP_006718117.1	1334	9	6.41	GGTLC2	XP_005261872.2	158	5	30.06
POLL	XP_024303713.1	495	10	19.19	IGLL5	NP_001171597.1	1190	6	4.79
DPCD	NP_001316671.1	220	10	43.18	RSPH14	XP_016884263.1	218	6	26.15
FBXW4	NP_071322.2	524	10	18.13	GNAZ	NP_002064.1	1117	4	3.40

FGF8	NP_149355.1	1355	12	8.41	RAB36	XP_011528848.1	630	3	4.52
NPM3	NP_008924.1	574	3	4.96	BCR	NP_004318.3	937	6	6.08
OGA	NP_001135906.1	662	10	14.35	IGLL1	NP_064455.1	272	10	34.92
KCNIP2	XP_005269787.1	1324	7	5.02	DRICH1	XP_011528509.1	81	10	117.28
ARMH3	XP_011538456.1	157	8	48.41	RGL4	NP_001316353.1	332	10	28.61
HPS6	NP_079023.2	388	5	12.24	ZNF70	XP_024308037.1	170	11	61.47
LDB1	XP_016872357.1	715	11	14.61	VPREB3	NP_037510.1	165	10	57.57
PPRC1	NP_055877.3	560	4	6.79	C22orf15	NP_001317970.1	44	13	280.67
NOLC1	XP_005270330.1	1184	7	5.62	CHCHD10	NP_998885.1	374	9	22.86
ELOVL3	NP_689523.1	442	4	8.60	MMP11	NP_005931.2	563	4	6.75
PITX3	NP_005020.1	526	5	9.03	SMARCB1	NP_001349806.1	1605	8	4.73
GBF1	XP_006718110.1	887	5	5.35	DERL3	NP_001129223.1	738	10	12.87
NFKB2	XP_016871767.1	1921	2	0.99	SLC2A11	NP_001020109.1	181	15	78.73
PSD	XP_016871922.1	1025	4	3.71	MIF	NP_002406.1	881	3	3.23
FBXL15	XP_005270206.1	772	6	7.38	GSTT2B	NP_001350733.1	777	16	19.56
CUEDC2	NP_076945.2	134	5	35.45	DDTL	NP_001077862.1	155	4	24.51
C10orf95	NP_001350509.1	1	0	0.00	DDT	NP_001077861.1	690	7	9.64
MFSD13A	XP_016872146.1	118	3	24.15	CABIN1	XP_024307952.1	593	7	11.21
ACTR1A	NP_005727.1	1463	4	2.60	SUSD2	NP_062547.1	268	9	31.90
SUFU	XP_011538165.1	1281	2	1.48	GGT5	NP_001289394.1	337	7	19.73
TRIM8	NP_112174.2	672	4	5.65	SPECC1L	NP_056145.5	568	11	18.40
ARL3	NP_004302.1	1449	7	4.59	ADORA2A	NP_001265426.1	913	4	4.16
SFXN2	XP_011537563.1	255	2	7.45	UPB1	XP_011528524.1	476	8	15.97
WBP1L	NP_001077382.1	166	8	45.78	GUCD1	NP_001271182.1	52	10	182.68
CYP17A1	NP_000093.1	880	6	6.48	SNRPD3	NP_004166.1	1575	7	4.22
BORCS7	NP_653192.2	135	7	49.26	LRRC75B	NP_997527.2	30	10	316.65
AS3MT	NP_065733.2	333	13	37.09	GGT1	NP_001275762.1	1368	4	2.78
CNNM2	NP_060119.3	588	13	21.00	PIWIL3	NP_001242904.1	360	0	0.00
NT5C2	NP_001338098.1	588	9	14.54	SGSM1	NP_001035037.1	556	1	1.71
RPEL1	NP_001137381.1	505	0	0.00	TMEM211	XP_011528396.1	142	0	0.00
INA	NP_116116.1	719	1	1.32	KIAA1671	NP_001138678.1	91	0	0.00
PCGF6	NP_115530.2	446	6	12.78	CRYBB3	XP_016884088.1	164	0	0.00
TAF5	NP_008882.2	985	6	5.79	CRYBB2	NP_000487.1	170	1	5.59
ATP5MD	XP_024304005.1	589	12	19.35	LRP5L	XP_016884577.1	312	1	3.04
PDCD11	XP_005269704.1	1318	7	5.05	GRK3	NP_005151.2	1082	3	2.63
CALHM2	XP_016871795.1	96	6	59.37	MYO18B	XP_016884501.1	1038	3	2.75
CALHM1	NP_001001412.3	286	11	36.54	SEZ6L	NP_066938.2	847	2	2.24
CALHM3	NP_001123214.1	80	2	23.75	ASPHD2	NP_065170.2	118	1	8.05
NEURL1	XP_011538633.1	832	4	4.57	HPS4	NP_001336833.1	228	1	4.17
SH3PXD2A	NP_055446.2	1436	2	1.32	SRRD	NP_001013716.2	82	0	0.00
STN1	NP_079204.2	728	1	1.30	TFIP11	NP_001333791.1	770	0	0.00
SLK	NP_055535.2	470	0	0.00	TPST2	XP_024308062.1	830	2	2.29
COL17A1	NP_000485.3	562	0	0.00	CRYBB1	NP_001878.1	248	0	0.00
SFR1	NP_001002759.1	80	0	0.00	CRYBA4	XP_006724203.1	185	0	0.00
CFAP43	NP_079421.5	85	0	0.00	MN1	NP_002421.3	361	2	5.26
GSTO1	NP_004823.1	674	2	2.82	PITPNB	NP_001271206.1	553	2	3.44
GSTO2	NP_899062.1	476	2	3.99	TTC28	XP_005261462.1	1025	4	3.71
ITPRIP	XP_005270314.1	97	0	0.00	CHEK2	XP_011528144.1	2516	6	2.27
CFAP58	NP_001008723.1	55	1	17.27	HSCB	NP_741999.3	480	1	1.98
SORCS3	NP_055793.1	1069	1	0.89	CCDC117	NP_775781.1	52	4	73.07
SORCS1	XP_016871106.1	651	1	1.46	XBP1	NP_001073007.1	1253	2	1.52
XPNPEP1	NP_001311062.1	688	3	4.14	ZNRF3	XP_016884479.1	500	4	7.60
ADD3	NP_001307520.1	554	4	6.86	C22orf31	NP_056185.1	3	0	0.00
MXI1	NP_569157.2	680	6	8.38	KREMEN1	NP_114434.3	415	4	9.16
SMNDC1	NP_005862.1	666	3	4.28	EMID1	XP_011528172.1	182	1	5.22
DUSP5	NP_004410.3	1191	3	2.39	RHBDD3	XP_006724287.1	263	0	0.00
SMC3	NP_005436.1	1901	3	1.50	EWSR1	XP_011528297.1	1308	13	9.44
RBM20	XP_016871592.1	313	3	9.10	GAS2L1	XP_016884022.1	540	8	14.07
PDCD4	NP_055271.2	810	0	0.00	RASL10A	XP_011528125.1	1317	9	6.49
BBIP1	NP_001182236.1	157	1	6.05	AP1B1	NP_001365495.1	944	5	5.03
SHOC2	NP_001311265.1	804	7	8.27	RFPL1	NP_066306.2	538	11	19.42
ADRA2A	NP_000672.3	870	1	1.09	NEFH	NP_066554.2	900	9	9.50
GPAM	XP_024303857.1	527	8	14.42	THOC5	XP_024308064.1	574	6	9.93
TECTB	NP_478129.1	252	9	33.93	NIPSNAP1	NP_001189431.1	350	5	13.57
ACSL5	NP_057318.2	1010	11	10.35	NF2	NP_000259.1	1301	13	9.49
ZDHHC6	XP_016872055.1	199	11	52.51	CABP7	NP_872333.1	993	0	0.00

VTI1A	NP_001352640.1	625	12	18.24	ZMAT5	NP_001003692.1	529	12	21.55
TCF7L2	NP_001354872.1	1624	4	2.34	UQCR10	NP_037519.2	601	8	12.64
HABP2	NP_004123.1	667	12	17.09	ASCC2	XP_024308059.1	217	10	43.78
NRAP	NP_006166.3	349	2	5.44	MTMR3	NP_066576.1	343	7	19.39
CASP7	NP_001253986.1	1158	12	9.84	HORMAD2	XP_016884110.1	386	9	22.15
PLEKHS1	XP_005270219.1	133	11	78.57	LIF	XP_024308007.1	1050	3	2.71
DCLRE1A	NP_055696.3	629	11	16.61	OSM	NP_065391.1	878	3	3.25
NHLRC2	NP_940916.2	353	14	37.68	CASTOR1	NP_001032755.1	157	3	18.15
ADRB1	NP_000675.1	855	11	12.22	TBC1D10A	NP_114143.1	292	6	19.52
CCDC186	NP_001308758.1	349	13	35.38	SF3A1	NP_005868.1	1310	5	3.63
TDRD1	NP_001372294.1	948	5	5.01	CCDC157	XP_011528568.1	75	0	0.00
VWA2	XP_016871668.1	181	6	31.49	RNF215	NP_001017981.1	273	2	6.96
AFAP1L2	XP_016872293.1	203	4	18.72	SEC14L2	NP_036561.1	418	1	2.27
ABLIM1	NP_001309814.1	510	8	14.90	MTFP1	NP_001003704.1	217	2	8.76
FAM160B1	NP_001128523.1	194	6	29.38	SEC14L3	XP_011528430.1	387	0	0.00
TRUB1	NP_631908.1	1037	6	5.50	SEC14L4	NP_001154840.1	233	0	0.00
ATRNL1	XP_011537890.1	591	4	6.43	SEC14L6	XP_016884420.1	143	0	0.00
GFRA1	NP_001369490.1	526	5	9.03	GAL3ST1	XP_011528820.1	279	2	6.81
CCDC172	XP_016871695.1	42	0	0.00	PES1	NP_001230154.1	1343	1	0.71
PNLIPRP3	NP_001011709.2	208	2	9.13	TCN2	NP_000346.2	315	2	6.03
PNLIP	NP_000927.1	500	4	7.60	SLC35E4	NP_001305300.1	186	3	15.32
PNLIPRP1	NP_006220.1	269	4	14.13	DUSP18	XP_016884116.1	506	1	1.88
C10orf82	XP_011537645.1	77	0	0.00	OSBP2	NP_001269667.1	577	5	8.23
HSPA12A	NP_001317093.1	1752	8	4.34	MORC2	NP_001290186.1	408	2	4.66
ENO4	XP_006717898.1	861	2	2.21	SMTN	NP_001369571.1	602	1	1.58
SHTN1	NP_001120683.1	241	5	19.71	SELENOM	NP_536355.1	107	0	0.00
VAX1	NP_954582.1	447	2	4.25	INPP5J	XP_016884261.1	675	4	5.63
KCNK18	NP_862823.1	346	0	0.00	PLA2G3	NP_056530.2	247	1	3.85
SLC18A2	NP_003045.2	660	1	1.44	RNF185	NP_689480.2	358	2	5.31
PDZD8	XP_005269575.1	132	3	21.59	LIMK2	NP_005560.1	1183	5	4.02
EMX2	NP_004089.1	756	3	3.77	PIK3IP1	NP_443112.2	212	0	0.00
RAB11FIP2	NP_055719.1	429	0	0.00	PATZ1	NP_114439.1	513	0	0.00
FAM204A	XP_005270081.1	62	0	0.00	DRG1	NP_004138.1	957	1	0.99
PRLHR	NP_004239.2	385	2	4.93	EIF4ENIF1	XP_016884352.1	469	1	2.03
CACUL1	NP_722517.3	995	3	2.86	SFI1	XP_011528872.1	520	4	7.31
NANOS1	NP_955631.1	531	0	0.00	PISD	NP_001313341.1	868	2	2.19
EIF3A	NP_003741.1	1289	2	1.47	PRR14L	NP_775837.2	90	4	42.22
DENND10	XP_016871753.1	74	0	0.00	DEPDC5	NP_001351247.1	429	2	4.43
SFXN4	XP_005269582.1	332	1	2.86	C22orf24	NP_001289748.1	35	2	54.28
PRDX3	NP_006784.1	1554	2	1.22	YWHAH	NP_003396.1	2278	8	3.34
GRK5	NP_005299.1	748	2	2.54	SLC5A1	NP_000334.1	663	4	5.73
RGS10	NP_001005339.1	387	0	0.00	C22orf42	XP_016884119.1	26	0	0.00
TIAL1	NP_003243.1	1178	2	1.61	RFPL2	NP_001091997.2	271	7	24.54
BAG3	NP_004272.2	1467	3	1.94	SLC5A4	XP_016884409.1	328	5	14.48
INPP5F	XP_011537826.1	330	3	8.64	RFPL3	XP_016884023.1	272	7	24.45
MCMBP	XP_016872152.1	406	2	4.68	RTCB	NP_055121.1	688	3	4.14
SEC23IP	NP_009121.1	447	1	2.13	BPIFC	XP_011528393.1	101	9	84.65
PLPP4	NP_001025230.1	382	5	12.43	FBXO7	NP_036311.3	902	5	5.27
WDR11	NP_060587.8	229	3	12.44	SYN3	NP_001356839.1	583	7	11.41
FGFR2	NP_001138387.1	1804	7	3.69	TIMP3	NP_000353.1	1082	3	2.63
ATE1	NP_001275665.1	318	6	17.92	LARGE1	XP_024308070.1	322	1	2.95
NSMCE4A	NP_001161337.1	240	5	19.79	ISX	NP_001290437.1	540	3	5.28
TACC2	XP_024303524.1	361	6	15.79	HMGXB4	XP_006724164.1	275	7	24.18
BTBD16	XP_016871125.1	100	4	38.00	TOM1	NP_001129204.1	684	8	11.11
PLEKHA1	XP_016871969.1	301	4	12.62	HMOX1	NP_002124.1	1384	5	3.43
ARMS2	NP_001093137.1	167	2	11.38	MCM5	XP_006724305.1	1846	8	4.12
HTRA1	NP_002766.1	947	4	4.01	RASD2	XP_016884190.1	1975	12	5.77
DMBT1	NP_001364459.1	431	1	2.20	MB	NP_001349775.1	1027	3	2.77
C10orf120	NP_001010912.1	25	0	0.00	APOL6	NP_085144.1	315	3	9.05
CUZD1	NP_071317.2	186	0	0.00	APOL5	XP_006724384.1	96	6	59.37
FAM24B	NP_689857.2	42	0	0.00	RBFOX2	XP_016884177.1	817	9	10.46
FAM24A	XP_016871127.1	36	0	0.00	APOL3	NP_663615.1	441	6	12.92
C10orf88	NP_079218.2	57	5	83.33	APOL4	NP_663693.1	217	7	30.64
PSTK	NP_001350460.1	210	2	9.05	APOL2	XP_016884213.1	313	5	15.17
IKZF5	XP_016872040.1	323	1	2.94	APOL1	NP_663318.1	555	9	15.40
ACADSB	NP_001600.1	590	6	9.66	MYH9	NP_002464.1	2061	15	6.91

HMX3	NP_001099044.1	443	1	2.14	TXN2	NP_036605.2	1276	6	4.47
HMX2	XP_005269800.1	375	2	5.07	FOXRED2	NP_001349970.1	270	7	24.63
BUB3	NP_001007794.1	1587	6	3.59	EIF3D	NP_003744.1	1069	4	3.55
GPR26	NP_703143.1	379	6	15.04	CACNG2	NP_006069.1	790	7	8.42
CPXM2	NP_937791.2	251	7	26.49	IFT27	NP_006851.1	385	0	0.00
CHST15	XP_005269951.1	304	3	9.37	PVALB	NP_002845.1	2206	3	1.29
OAT	NP_001309900.1	982	4	3.87	NCF4	NP_000622.2	1129	5	4.21
NKX1-2	NP_001139812.1	206	2	9.22	CSF2RB	XP_011528205.1	895	7	7.43
LHPP	NP_071409.3	345	5	13.77	TEX33	NP_001157329.1	106	2	17.92
FAM53B	NP_055476.3	221	2	8.60	TST	NP_003303.2	661	3	4.31
EEF1AKMT2	NP_001291397.1	167	3	17.06	MPST	NP_066949.2	471	1	2.02
ABRAXAS2	NP_115558.3	188	7	35.37	KCTD17	XP_005261801.1	321	4	11.84
ZRANB1	XP_006717970.1	364	1	2.61	TMPRSS6	XP_011528291.1	561	2	3.39
CTBP2	NP_001307941.1	1205	3	2.37	IL2RB	NP_001333151.1	1024	8	7.42
TEX36	NP_001305062.1	47	6	121.27	C1QTNF6	NP_114116.3	236	5	20.13
EDRF1	NP_001189367.1	93	0	0.00	SSTR3	XP_016884412.1	837	5	5.67
MMP21	NP_671724.1	129	3	22.09	RAC2	NP_002863.1	2865	10	3.32
UROS	XP_011538429.1	459	5	10.35	CYTH4	NP_037517.1	758	6	7.52
BCCIP	NP_057651.1	1104	4	3.44	ELFN2	NP_443138.2	402	4	9.45
DHX32	NP_060650.2	978	8	7.77	MFNG	NP_002396.2	529	3	5.39
FANK1	NP_001337868.1	1027	10	9.25	CARD10	NP_055365.2	802	2	2.37
ADAM12	NP_001275902.1	722	7	9.21	CDC42EP1	NP_689449.1	192	1	4.95
C10orf90	NP_001337851.1	111	1	8.56	LGALS2	NP_006489.1	253	2	7.51
DOCK1	NP_001364473.1	872	8	8.72	GGA1	NP_001350700.1	707	2	2.69
INSYN2A	XP_016872026.1	92	4	41.30	SH3BP1	NP_001336984.1	279	2	6.81
NPS	NP_001025184.1	1291	0	0.00	PDXP	NP_064711.1	437	1	2.17
FOXI2	NP_997309.2	468	6	12.18	LGALS1	NP_002296.1	1012	2	1.88
CLRN3	XP_011537576.1	232	2	8.19	NOL12	NP_077289.1	393	3	7.25
PTPRE	XP_011538297.1	853	7	7.80	TRIOBP	NP_001034230.1	350	2	5.43
MKI67	NP_001139438.1	1255	1	0.76	H1-0	NP_005309.1	1264	2	1.50
MGMT	NP_002403.3	1076	6	5.30	GCAT	XP_016884163.1	578	2	3.29
EBF3	NP_001362321.1	501	7	13.27	GALR3	NP_003605.1	441	3	6.46
GLRX3	NP_001186797.1	794	6	7.18	ANKRD54	NP_001336782.1	928	2	2.05
TCERG1L	NP_777597.2	600	5	7.92	EIF3L	XP_006724323.1	681	0	0.00
PPP2R2D	XP_006717977.1	1006	11	10.39	MICALL1	XP_011528773.1	457	0	0.00
BNIP3	NP_004043.3	806	5	5.89	C22orf23	NP_001193991.1	110	0	0.00
JAKMIP3	NP_001310015.1	433	6	13.16	POLR2F	NP_001288059.1	1562	4	2.43
DPYSL4	NP_006417.2	390	9	21.92	SOX10	NP_008872.1	1529	1	0.62
STK32C	NP_001305807.1	884	2	2.15	PICK1	NP_036539.1	760	1	1.25
LRRC27	NP_001137229.1	316	0	0.00	SLC16A8	XP_016884174.1	323	1	2.94
PWWP2B	XP_011537689.1	72	0	0.00	BAIAP2L2	XP_011528681.1	247	4	15.38
INPP5A	NP_005530.3	388	12	29.38	PLA2G6	NP_001336794.1	698	4	5.44
NKX6-2	XP_016872278.1	841	1	1.13	MAFF	NP_001155044.1	487	4	7.80
CFAP46	NP_001186978.2	121	1	7.85	TMEM184B	XP_016884244.1	174	3	16.38
ADGRA1	NP_001077378.1	588	11	17.77	CSNK1E	NP_689407.1	1245	2	1.53
KNDC1	NP_689856.6	688	9	12.43	KCNJ4	NP_690607.1	1057	2	1.80
UTF1	NP_003568.2	418	2	4.55	KDELR3	NP_006846.1	631	4	6.02
VENTX	NP_055283.1	306	6	18.63	DDX17	NP_006377.2	1912	3	1.49
ADAM8	NP_001157962.1	787	5	6.04	DMC1	XP_011528140.1	1224	0	0.00
TUBGCP2	NP_006650.1	806	4	4.71	FAM227A	XP_011528611.1	48	7	138.53
ZNF511	NP_665805.2	59	5	80.50	CBY1	NP_001002880.2	372	3	7.66
CALY	NP_056537.1	935	5	5.35	TOMM22	NP_064628.1	696	1	1.36
PRAP1	NP_660203.3	138	5	38.24	JOSD1	NP_001347164.1	191	5	24.87
FUOM	XP_011537977.1	57	4	78.43	GTPBP1	XP_011528839.1	339	1	2.80
ECHS1	NP_004083.3	1040	4	4.57	SUN2	XP_011528407.1	651	4	5.84
PAOX	NP_997011.1	527	6	14.42	DNAL4	NP_005731.1	333	2	5.71
MTG1	NP_612393.2	701	7	13.55	NPTXR	NP_055108.2	491	2	3.87
SPRN	NP_001012526.2	177	5	41.28	CBX6	NP_001290423.1	561	10	16.93
CYP2E1	NP_000764.1	808	4	7.84	APOBEC3A	NP_663745.1	327	3	8.72
SYCE1	NP_001137235.1	313	7	38.63	APOBEC3B	NP_004891.5	416	2	4.57
FRG2B	NP_001074467.1	61	0	0.00	APOBEC3C	NP_055323.2	249	1	3.82
SCGB1C1	XP_005252861.2	129	4	58.91	APOBEC3D	XP_016884085.1	210	1	4.52
ODF3	NP_444510.2	206	5	41.92	APOBEC3F	XP_024307946.1	264	2	7.20
BET1L	NP_001092257.1	479	5	16.53	APOBEC3G	XP_016884392.1	699	3	4.08
RIC8A	XP_005253109.1	389	6	22.54	APOBEC3H	NP_861438.3	72	4	52.78
SIRT3	NP_001357245.1	1452	4	3.74	CBX7	NP_001333673.1	612	8	12.42

PSMD13	NP_002808.3	811	8	12.49	PDGFB	NP_002599.1	1137	4	3.34
NLRP6	NP_612202.2	340	4	13.97	RPL3	NP_001029025.1	1599	2	1.19
PGGHG	XP_016873844.1	208	11	59.10	SYNGR1	NP_004702.2	650	3	4.38
IFITM5	NP_001020466.1	241	3	13.14	TAB1	NP_705717.1	1177	1	0.81
IFITM2	NP_006426.2	316	3	9.49	MGAT3	NP_002400.3	298	1	3.19
IFITM1	NP_003632.4	578	5	8.22	MIEF1	NP_001291493.1	301	1	3.16
IFITM3	NP_066362.2	440	4	8.64	ATF4	XP_016884296.1	1306	2	1.45
B4GALNT4	NP_848632.2	272	10	34.92	RPS19BP1	NP_919307.1	215	0	0.00
PKP3	NP_001289958.1	774	7	8.59	CACNA1I	NP_066919.2	716	3	3.98
SIGIRR	XP_005253105.1	233	6	24.46	ENTHD1	XP_011528230.1	361	1	2.63
ANO9	NP_001012302.2	154	9	55.52	GRAP2	NP_004801.1	1271	2	1.49
PTDSS2	XP_024304467.1	267	3	10.67	FAM83F	NP_612444.2	147	0	0.00
RNH1	XP_016873595.1	525	2	3.62	TNRC6B	NP_001020014.1	464	1	2.05
HRAS	NP_001123914.1	5632	9	1.52	ADSL	NP_001350769.1	1126	0	0.00
LRRC56	XP_016872656.1	586	3	4.86	SGSM3	XP_024307988.1	525	2	3.62
LMNTD2	NP_775844.2	250	3	11.40	MRTFA	NP_001269590.2	472	3	6.04
RASSF7	NP_003466.1	270	9	31.67	MCHR1	NP_005288.4	870	1	1.09
PHRF1	NP_001273510.1	435	7	15.29	SLC25A17	NP_001269656.1	468	2	4.06
IRF7	XP_011518368.1	1109	4	3.43	ST13	NP_001265518.1	667	1	1.42
CDHR5	NP_112554.3	373	6	15.28	XPNPEP3	NP_071381.1	663	1	1.43
SCT	XP_016873598.1	556	3	5.13	DNAJB7	NP_660157.1	495	1	1.92
DRD4	NP_000788.2	1036	6	5.50	RBX1	NP_055063.1	1791	4	2.12
DEAF1	NP_001280563.1	394	9	21.70	EP300	NP_001420.2	3446	6	1.65
TMEM80	XP_006718269.3	156	2	12.18	L3MBTL2	XP_011528722.1	506	4	7.51
EPS8L2	XP_016873620.1	510	6	11.18	CHADL	XP_011528235.1	593	4	6.41
TALDO1	NP_006746.1	1190	0	0.00	RANGAP1	XP_011528595.1	1331	4	2.85
GATD1	NP_001305753.1	85	6	67.06	ZC3H7B	NP_060060.3	158	3	18.04
CEND1	NP_057648.2	919	0	0.00	TEF	NP_001138870.1	746	5	6.37
SLC25A22	NP_078974.1	473	2	4.02	TOB2	XP_016884028.1	262	2	7.25
PANO1	XP_034607700.1	29	1	32.76	PHF5A	NP_116147.1	1134	6	5.03
PIDD1	XP_011518511.1	2115	1	0.45	ACO2	XP_024308018.1	1194	2	1.59
RPLP2	NP_000995.1	1241	1	0.77	POLR3H	NP_001018062.1	533	3	5.35
PNPLA2	XP_006718328.1	896	1	1.06	CSDC2	NP_055275.1	638	0	0.00
CRACR2B	XP_016873079.1	99	3	28.79	PMM1	XP_011528533.1	581	4	6.54
CD151	XP_024304548.1	527	3	5.41	DESI1	NP_056519.1	306	1	3.10
POLR2L	NP_066951.1	1381	3	2.06	XRCC6	NP_001460.1	1682	3	1.69
TSPAN4	XP_011518638.1	484	1	1.96	SNU13	NP_001003796.1	1725	4	2.20
CHID1	XP_005253129.1	118	3	24.15	MEI1	XP_011528247.1	264	1	3.60
AP2A2	NP_001229766.1	947	4	4.01	CCDC134	NP_079097.1	104	0	0.00
MUC6	NP_005952.2	482	5	9.85	SREBF2	NP_004590.2	911	0	0.00
MUC5AC	NP_001291288.1	966	3	2.95	SHISA8	NP_001193949.1	208	0	0.00
MUC5B	NP_002449.2	839	3	3.40	TNFRSF13C	NP_443177.1	696	2	2.73
TOLLIP	NP_001305441.1	613	2	3.10	CENPM	XP_011528670.1	513	3	5.56
BRSK2	NP_003948.2	1481	3	1.92	SEPTIN3	NP_001350774.1	1042	5	4.56
MOB2	NP_001165694.1	463	6	12.31	WBP2NL	NP_689826.2	208	5	22.84
DUSP8	NP_004411.2	1003	8	7.58	NAGA	NP_001349777.1	383	6	14.88
KRTAP5-1	NP_001005922.1	32	4	118.74	PHETA2	NP_001002034.2	73	7	91.09
KRTAP5-2	NP_001004325.1	305	4	12.46	SMDT1	NP_201575.3	344	7	19.33
KRTAP5-3	NP_001012726.1	26	0	0.00	NDUFA6	NP_002481.3	814	7	8.17
KRTAP5-4	NP_001334603.1	186	0	0.00	CYP2D6	NP_001020332.2	709	3	4.02
KRTAP5-5	NP_001001480.2	59	0	0.00	TCF20	NP_852469.1	1849	5	2.57
KRTAP5-6	NP_001012416.1	55	0	0.00	NFAM1	NP_666017.1	406	0	0.00
IFITM10	NP_001164291.2	132	5	35.98	RRP7A	NP_056518.2	647	1	1.47
CTSD	NP_001900.1	1806	6	3.16	SERHL2	XP_024307964.1	273	5	17.40
SYT8	XP_011518757.1	460	5	10.33	POLDIP3	NP_835237.1	854	3	3.34
TNNI2	NP_001139301.1	562	6	10.14	CYB5R3	NP_000389.1	725	1	1.31
LSP1	NP_002330.1	715	4	5.31	ATP5MGL	NP_001159349.1	437	3	6.52
TNNT3	XP_016873695.1	672	6	8.48	A4GALT	XP_016884320.1	304	4	12.50
MRPL23	XP_011518577.1	680	9	12.57	ARFGAP3	NP_001135765.1	894	2	2.13
IGF2	NP_001121070.1	1926	13	6.41	PACSIN2	NP_001171900.1	605	4	6.28
INS-IGF2	NP_001035835.1	498	5	9.54	TTLL1	XP_016884240.1	377	7	17.64
INS	NP_001172026.1	6053	13	2.04	BIK	NP_001188.1	312	1	3.04
TH	NP_000351.2	1596	12	7.14	MCAT	NP_775738.3	1154	4	3.29
ASCL2	NP_005161.1	555	13	22.25	TSPO	NP_001243459.1	690	2	2.75
C11orf21	NP_001136418.1	76	1	12.50	TTLL12	NP_055955.1	399	3	7.14
TSPAN32	XP_011518124.1	357	13	34.59	SCUBE1	NP_766638.2	395	2	4.81

CD81	NP_004347.1	865	13	14.28	MPPED1	NP_001037835.1	813	3	3.51
TSSC4	NP_005697.2	373	14	35.65	EFCAB6	XP_011528620.1	1023	1	0.93
TRPM5	XP_016873117.1	385	10	24.67	SULT4A1	XP_011528423.1	972	6	5.86
KCNQ1	NP_000209.2	1402	16	10.84	PNPLA5	NP_001171146.1	211	3	13.51
CDKN1C	NP_001349404.1	1010	14	13.17	PNPLA3	NP_079501.2	516	3	5.52
SLC22A18	NP_001302430.1	640	14	20.78	SAMM50	NP_056195.3	652	6	8.74
PHLDA2	NP_003302.1	297	14	44.78	PARVB	NP_001003828.1	590	6	9.66
NAP1L4	NP_001356313.1	862	12	13.22	PARVG	XP_011528604.1	832	5	5.71
CARS1	NP_001742.1	1353	8	5.62	SHISAL1	NP_001092764.1	110	1	8.64
OSBPL5	NP_663613.1	485	13	25.46	RTL6	NP_115663.2	104	0	0.00
MRGPRG	NP_001157849.1	81	10	117.28	PRR5	NP_001185650.1	287	3	9.93
MRGPRE	NP_001034254.2	168	3	16.96	PRR5-ARHGAI	NP_851851.3	263	5	18.06
ZNF195	NP_009083.2	201	5	23.63	ARHGAP8	NP_001017526.1	265	4	14.34
ART5	NP_001073004.1	138	8	55.07	PHF21B	NP_001129334.1	958	6	5.95
ART1	XP_016873252.1	174	1	5.46	NUP50	XP_024307914.1	683	0	0.00
CHRNA10	NP_065135.2	436	7	15.25	KIAA0930	NP_001009880.1	140	0	0.00
NUP98	NP_001352057.1	1313	7	5.06	UPK3A	NP_008884.1	334	3	8.53
PGAP2	NP_001243165.1	281	6	20.28	FAM118A	XP_011528556.1	129	0	0.00
RHOG	XP_016873208.1	1893	6	3.01	SMC1B	NP_001278430.1	1168	2	1.63
STIM1	NP_001369497.1	680	8	11.18	RIBC2	NP_056468.3	218	1	4.36
RRM1	NP_001024.1	2017	6	2.83	FBLN1	NP_006477.3	785	4	4.84
OR52B4	NP_001005161.2	84	0	0.00	ATXN10	NP_037368.1	405	3	7.04
TRIM21	NP_003132.2	1179	5	4.03	WNT7B	NP_478679.1	1066	2	1.78
OR52K2	NP_001005172.2	68	0	0.00	PPARA	NP_001349802.1	1307	2	1.45
OR52K1	NP_001372665.1	86	0	0.00	CDPF1	XP_011528262.1	4	0	0.00
OR52M1	NP_001004137.1	100	0	0.00	PKDREJ	NP_006062.1	979	3	2.91
C11orf40	XP_035716991.1	65	1	14.61	TTC38	NP_060401.3	191	1	4.97
OR52I2	NP_001005170.1	77	0	0.00	GTSE1	NP_057510.5	661	1	1.44
OR52I1	NP_001005169.1	50	0	0.00	TRMU	NP_060476.2	676	0	0.00
TRIM68	NP_060543.5	403	1	2.36	CELSR1	NP_055061.1	627	8	12.12
OR51D1	NP_001004751.1	85	0	0.00	GRAMD4	XP_006724232.1	154	10	61.69
OR51E1	NP_689643.2	211	0	0.00	CERK	NP_073603.2	553	7	12.02
OR51E2	NP_110401.1	250	0	0.00	TBC1D22A	XP_016884231.1	479	10	19.83
OR51F1	NP_001004752.2	85	0	0.00	TAFA5	NP_001076436.1	480	10	19.79
OR52R1	NP_001005177.3	56	0	0.00	BRD1	XP_016884203.1	920	7	7.23
OR51F2	NP_001004753.2	97	0	0.00	ALG12	XP_016884425.1	301	12	37.87
OR51S1	NP_001004758.1	36	0	0.00	ZBED4	XP_024308080.1	268	15	53.17
OR51H1	NP_001335220.1	31	0	0.00	CRELD2	XP_005261794.1	345	12	33.04
OR51T1	NP_001004759.2	38	0	0.00	PIM3	NP_001001852.2	1358	10	7.00
OR51A7	NP_001004749.1	144	1	6.60	IL17REL	NP_001358346.1	252	15	56.54
OR51G2	NP_001005238.1	54	0	0.00	TTLL8	NP_001337246.1	141	0	0.00
OR51G1	NP_001005237.1	80	0	0.00	MLC1	NP_001363409.1	465	0	0.00
OR51A4	NP_001005329.1	46	0	0.00	MOV10L1	XP_011528998.1	643	16	23.64
OR51A2	NP_001004748.1	75	0	0.00	PANX2	NP_443071.2	412	12	27.67
MMP26	NP_068573.2	80	1	11.87	TRABD	XP_011529014.2	138	18	123.91
OR51L1	NP_001004755.1	141	0	0.00	SELENOO	NP_113642.1	162	11	64.50
OR52J3	NP_001001916.2	196	0	0.00	TUBGCP6	NP_065194.3	660	16	23.03
OR52E2	NP_001005164.2	75	0	0.00	HDAC10	NP_001152758.1	2160	14	6.16
OR52A5	NP_001005160.1	62	4	61.29	MAPK12	NP_001290181.1	2179	9	3.92
OR52A1	NP_036507.2	39	2	48.72	MAPK11	NP_002742.3	2755	11	3.79
OR51V1	NP_001004760.2	63	4	60.31	PLXNB2	XP_011528984.1	521	12	21.88
HBB	NP_000509.1	1232	11	8.48	DENND6B	XP_024308013.1	119	19	151.67
HBD	NP_000510.1	483	4	7.87	PPP6R2	XP_016884606.1	540	16	28.15
HBG1	NP_000550.2	409	11	25.55	SBF1	XP_005261992.1	792	15	17.99
HBG2	NP_000175.1	321	10	29.59	ADM2	NP_001356811.1	309	7	21.52
HBE1	NP_005321.1	628	15	22.69	MIOX	NP_060054.4	361	16	42.10
OR51B4	NP_149419.2	154	4	24.67	LMF2	XP_016884566.1	227	19	79.51
OR51B2	NP_149420.4	77	1	12.34	NCAPH2	NP_001171940.1	431	12	26.45
OR51B5	NP_001005567.2	101	4	37.62	SCO2	NP_005129.2	733	9	11.66
OR51B6	NP_001004750.1	60	4	63.33	TYMP	NP_001107227.1	752	7	9.31
OR51M1	NP_001004756.2	56	4	67.85	ODF3B	NP_001369736.1	289	15	54.78
OR51J1	NP_001335153.1	36	0	0.00	KLHDC7B	NP_612442.3	206	13	70.53
OR51Q1	NP_001004757.1	90	3	31.67	SYCE3	XP_024308029.1	222	8	42.79
OR51I1	NP_001005288.1	68	2	27.94	CPT1B	NP_001138606.1	599	5	10.57
OR51I2	NP_001004754.1	24	0	0.00	CHKB	NP_005189.2	696	10	19.50
OR52D1	NP_001005163.1	63	2	30.16	MAPK8IP2	XP_011528981.1	931	7	10.99

UBQLN3	XP_011518447.1	1622	2	1.17	ARSA	NP_000478.3	565	2	5.60
UBQLNL	NP_659490.4	1608	5	2.95	ACR	NP_001088.2	498	3	10.40
OR52H1	NP_001005289.1	122	6	46.72	RABL2B	XP_016884045.1	1540	4	4.93
OR52B6	NP_001005162.2	93	4	40.86	CHL1	XP_016861055.1	1282	3	4.45
TRIM6-TRIM3[illegible]	NP_001003819.1	326	3	8.74	CNTN6	XP_016861660.1	1022	5	8.45
TRIM6	NP_001003818.1	383	2	4.96	CNTN4	XP_016861271.1	1026	7	10.80
TRIM34	NP_067629.2	278	4	13.67	IL5RA	NP_000555.2	406	3	10.80
TRIM5	XP_016873951.1	619	6	9.21	TRNT1	NP_886552.3	650	0	0.00
TRIM22	NP_006065.2	637	4	5.97	CRBN	NP_001166953.1	399	9	28.57
OR56B1	NP_001005180.1	114	0	0.00	LRRN1	NP_001311118.1	786	12	18.13
OR52N4	XP_016873200.1	108	0	0.00	SUMF1	XP_016861743.1	286	8	31.26
OR52N5	NP_001372591.1	61	0	0.00	SETMAR	NP_006506.3	1666	12	7.60
OR52N1	NP_001001913.1	30	0	0.00	ITPR1	XP_011531983.1	1507	11	7.30
OR52N2	NP_001005174.1	75	1	12.67	BHLHE40	NP_003661.1	963	6	5.92
OR52E6	NP_001005167.1	44	0	0.00	ARL8B	NP_060654.1	1530	8	4.97
OR52E8	NP_001005168.1	90	0	0.00	EDEM1	NP_055489.1	481	5	9.87
OR52E4	NP_001005165.1	79	0	0.00	GRM7	NP_000835.1	1005	10	9.45
OR52E5	NP_001005166.2	20	0	0.00	LMCD1	NP_055398.1	779	10	12.19
OR56A3	NP_001003443.2	81	1	11.73	SSUH2	XP_016862013.1	132	12	86.36
OR56A5	NP_001139505.1	44	1	21.59	CAV3	NP_203123.1	933	6	6.11
OR52L1	NP_001005173.3	104	1	9.13	OXTR	NP_001341585.1	688	4	5.52
OR56A4	NP_001005179.3	72	1	13.19	RAD18	NP_064550.3	956	9	8.94
OR56A1	NP_001001917.2	73	1	13.01	SRGAP3	XP_024309610.1	1100	11	9.50
OR56B4	NP_001005181.1	72	1	13.19	THUMPD3	XP_016861602.1	326	14	40.80
OR52B2	NP_001004052.1	118	1	8.05	SETD5	XP_016862257.1	784	14	16.96
OR52W1	NP_001005178.1	102	1	9.31	LHFPL4	XP_016861848.1	651	15	21.89
C11orf42	XP_011518228.1	18	0	0.00	MTMR14	NP_001070993.1	383	14	34.72
FAM160A2	NP_001092264.1	119	0	0.00	CPNE9	NP_705899.2	584	16	26.03
CNGA4	XP_016872706.1	328	10	28.96	BRPF1	XP_024309509.1	697	9	12.27
CCKBR	NP_001350481.1	813	2	2.34	OGG1	XP_016861986.1	876	2	2.17
CAVIN3	NP_659477.2	306	1	3.10	CAMK1	XP_005265574.1	1260	8	6.03
SMPD1	NP_001352064.1	770	1	1.23	TADA3	NP_001265199.1	823	13	15.01
APBB1	NP_663722.1	869	3	3.28	ARPC4-TTLL3	NP_001185722.1	330	0	0.00
HPX	NP_000604.1	775	0	0.00	ARPC4	NP_001185709.1	561	12	20.32
TRIM3	XP_016872587.1	685	2	2.77	TTLL3	NP_001021100.3	277	15	51.44
ARFIP2	NP_001357342.1	428	0	0.00	RPUSD3	XP_024309240.1	427	17	37.82
TIMM10B	NP_036324.1	464	3	6.14	CIDEC	NP_001186552.1	492	6	11.58
DNHD1	NP_653267.2	607	1	1.56	JAGN1	NP_115881.3	195	14	68.20
RRP8	XP_011518257.1	713	2	2.66	IL17RE	XP_011531663.1	247	8	30.77
ILK	XP_024304262.1	1612	3	1.77	IL17RC	XP_016862815.1	317	6	17.98
TAF10	NP_006275.1	567	1	1.68	CRELD1	NP_056328.3	270	14	49.26
TPP1	NP_000382.3	289	1	3.29	PRRT3	NP_997234.3	144	17	112.15
DCHS1	NP_003728.1	524	1	1.81	EMC3	NP_060917.1	365	11	28.63
MRPL17	NP_071344.1	1062	1	0.89	FANCD2	NP_001018125.1	1106	4	3.44
OR2AG2	NP_001372982.1	32	0	0.00	FANCD2OS	XP_011531632.1	61	16	249.17
OR2AG1	NP_001004489.1	80	0	0.00	BRK1	NP_060932.2	467	10	20.34
OR6A2	NP_003687.2	153	0	0.00	VHL	NP_000542.1	873	2	2.18
OR10A5	NP_835462.1	76	0	0.00	IRAK2	NP_001561.3	1114	8	6.82
OR10A2	NP_001004460.1	34	0	0.00	TATDN2	NP_055575.3	308	16	49.35
OR10A4	NP_997069.2	158	3	18.04	GHRL	NP_001128418.1	694	2	2.74
OR2D2	NP_003691.1	112	3	25.45	SEC13	XP_016862510.1	1522	7	4.37
OR2D3	NP_001004684.1	138	3	20.65	ATP2B2	XP_016861976.1	1895	8	4.01
ZNF215	XP_024304451.1	341	5	13.93	SLC6A11	NP_055044.1	655	5	7.25
ZNF214	NP_001341759.1	290	8	26.21	SLC6A1	XP_016862560.1	1594	6	3.58
NLRP14	XP_011518346.1	160	9	53.43	HRH1	NP_001091681.1	475	1	2.00
RBMXL2	NP_055284.3	168	0	0.00	ATG7	XP_016861036.1	1611	5	2.95
SYT9	XP_011518204.1	843	0	0.00	VGLL4	XP_024309603.1	212	3	13.44
OLFML1	NP_001357427.1	312	7	21.31	TAMM41	XP_016861214.1	314	4	12.10
PPFIBP2	XP_011518717.1	210	9	40.71	SYN2	NP_598328.1	1305	8	5.82
CYB5R2	XP_016873409.1	512	6	11.13	TIMP4	NP_003247.1	337	5	14.09
OVCH2	XP_016873148.1	175	6	32.57	PPARG	NP_056953.2	2548	6	2.24
OR5P2	NP_703145.1	88	0	0.00	TSEN2	NP_001308207.1	392	7	16.96
OR5P3	NP_703146.1	55	0	0.00	MKRN2OS	NP_001364940.1	48	0	0.00
OR10A6	NP_001004461.1	42	2	45.24	MKRN2	NP_054879.3	259	6	22.01
OR10A3	NP_001003745.1	100	2	19.00	RAF1	XP_016862455.1	1975	7	3.37
NLRP10	NP_789791.1	152	5	31.25	TMEM40	NP_001271335.1	149	4	25.50

EIF3F	NP_003745.1	1382	5	3.44	CAND2	NP_001155971.1	405	4	9.38
TUB	XP_005253166.1	920	4	4.13	RPL32	NP_000985.1	1110	4	3.42
RIC3	NP_001128581.1	231	4	16.45	IQSEC1	NP_055684.3	559	3	5.10
LMO1	NP_001257357.1	743	5	6.39	NUP210	XP_024309179.1	471	3	6.05
STK33	XP_016873648.1	632	6	9.02	HDAC11	NP_079103.2	1371	3	2.08
TRIM66	XP_011518813.1	345	2	5.51	FBLN2	NP_001158507.1	669	6	8.52
RPL27A	NP_000981.1	1451	5	3.27	WNT7A	NP_004616.2	1027	2	1.85
DENND2B	XP_011518626.2	264	4	14.39	CHCHD4	NP_001091972.1	574	0	0.00
AKIP1	XP_016873500.1	120	0	0.00	TMEM43	NP_077310.1	355	2	5.35
C11orf16	XP_016873502.1	5	0	0.00	XPC	NP_001341658.1	668	2	2.84
ASCL3	NP_065697.1	238	0	0.00	LSM3	NP_055278.1	1314	1	0.72
TMEM9B	NP_065695.1	98	0	0.00	SLC6A6	NP_001127839.2	551	0	0.00
NRIP3	NP_065696.1	495	0	0.00	GRIP2	XP_011532444.1	782	0	0.00
SCUBE2	XP_005253093.2	380	2	5.00	CCDC174	NP_057558.3	164	0	0.00
DENND5A	NP_001335678.1	321	1	2.96	C3orf20	XP_006713404.1	123	1	7.72
TMEM41B	NP_055827.1	360	0	0.00	FGD5	XP_011531724.1	343	4	11.08
IPO7	NP_006382.1	1243	1	0.76	NR2C2	XP_016862607.1	571	1	1.66
ZNF143	XP_005253178.1	863	1	1.10	MRPS25	NP_071942.1	338	3	8.43
WEE1	NP_003381.1	1378	0	0.00	RBSN	XP_016862512.1	586	2	3.24
SWAP70	NP_055870.2	325	0	0.00	CAPN7	XP_011531857.1	682	1	1.39
SBF2	XP_011518696.1	521	1	1.82	SH3BP5	XP_011532553.1	371	0	0.00
ADM	NP_001115.1	607	2	3.13	METTL6	XP_016861212.1	363	3	7.85
AMPD3	NP_000471.1	559	0	0.00	EAF1	NP_149074.3	214	1	4.44
MTRNR2L8	NP_001177631.1	31	0	0.00	COLQ	NP_536800.2	223	3	12.78
RNF141	NP_057506.2	159	0	0.00	HACL1	NP_001271342.1	513	3	5.56
LYVE1	NP_006682.2	526	2	3.61	BTD	NP_001357681.1	366	2	5.19
IRAG1	NP_001193809.1	319	2	5.96	ANKRD28	XP_011531844.1	1085	3	2.63
CTR9	NP_055448.1	1561	2	1.22	GALNT15	XP_005264909.1	332	1	2.86
EIF4G2	NP_001036024.3	1455	1	0.65	DPH3	NP_001040899.1	222	2	8.56
ZBED5	NP_001137139.1	127	0	0.00	OXNAD1	XP_005265616.1	376	2	5.05
GALNT18	NP_001350393.1	345	1	2.75	RFTN1	NP_055965.1	289	2	6.57
CSNK2A3	NP_001243615.1	1130	2	1.68	DAZL	NP_001342.2	1537	1	0.62
USP47	NP_001317137.1	796	2	2.39	PLCL2	XP_016861511.1	568	1	1.67
DKK3	NP_056965.3	1037	5	4.58	TBC1D5	XP_016863046.1	601	4	6.32
MICAL2	NP_001333221.1	740	2	2.57	SATB1	NP_001309801.1	656	3	4.34
MICALCL	NP_116256.2	109	0	0.00	KCNH8	XP_016861187.1	545	4	6.97
PARVA	XP_005253072.1	823	3	3.46	EFHB	XP_011531685.3	422	0	0.00
TEAD1	NP_068780.2	878	5	5.41	RAB5A	NP_004153.2	2095	2	0.91
RASSF10	NP_001073990.2	122	3	23.36	PP2D1	NP_001239586.1	637	1	1.49
ARNTL	XP_016873227.1	1005	4	3.78	KAT2B	NP_003875.3	2089	6	2.73
BTBD10	XP_024304481.1	252	3	11.31	SGO1	NP_612493.1	667	2	2.85
PTH	NP_001303281.1	1514	7	4.39	ZNF385D	XP_016862681.1	240	0	0.00
FAR1	XP_011518702.1	472	0	0.00	UBE2E2	XP_011532378.1	2038	2	0.93
SPON1	NP_006099.2	1413	2	1.34	UBE2E1	NP_003332.1	1703	1	0.56
RRAS2	NP_001170785.1	1917	7	3.47	NKIRAS1	XP_024309228.1	275	4	13.82
COPB1	NP_001137533.1	1421	10	6.69	RPL15	NP_001240313.1	1304	3	2.19
PSMA1	NP_683877.1	1780	7	3.74	NR1D2	NP_005117.3	511	2	3.72
PDE3B	NP_001350499.1	550	6	10.36	THRB	NP_001361756.1	908	5	5.23
CYP2R1	NP_078790.2	301	8	25.25	RARB	NP_001277145.1	921	6	6.19
CALCA	NP_001365879.1	1116	6	5.11	TOP2B	NP_001059.2	1892	6	3.01
CALCB	NP_000719.1	256	4	14.84	NGLY1	NP_001138766.1	419	2	4.53
INSC	NP_001027024.3	362	7	18.37	OXSM	XP_006713279.1	1011	3	2.82
SOX6	NP_001354801.1	1010	3	2.82	LRRC3B	XP_016861164.1	1025	4	3.71
C11orf58	NP_055082.1	325	5	14.61	NEK10	XP_006713062.1	379	1	2.51
PLEKHA7	NP_001316559.1	834	4	4.56	SLC4A7	XP_016863016.1	650	2	2.92
RPS13	NP_001008.1	1709	13	7.23	EOMES	NP_005433.2	1136	3	2.51
PIK3C2A	XP_011518488.1	2058	12	5.54	CMC1	XP_024309133.1	102	3	27.94
NUCB2	XP_024304295.1	394	11	26.52	AZI2	XP_011532311.1	264	4	14.39
NCR3LG1	NP_001189368.1	165	1	5.76	ZCWPW2	NP_001311098.1	54	0	0.00
KCNJ11	NP_000516.3	845	11	12.37	RBMS3	NP_001003793.1	393	3	7.25
ABCC8	NP_001338226.1	1397	10	6.80	TGFBR2	NP_001020018.1	2221	4	1.71
USH1C	NP_710142.1	945	6	6.03	GADL1	NP_997242.2	577	4	6.59
OTOG	NP_001264198.1	376	10	25.26	STT3B	NP_849193.1	494	4	7.69
MYOD1	NP_002469.2	1791	11	5.83	OSBPL10	NP_001167531.1	447	6	12.75
KCNC1	NP_001106212.1	1581	12	7.21	ZNF860	XP_016861788.1	196	5	24.23
SERGEF	NP_036271.1	504	13	24.50	GPD1L	NP_055956.1	955	5	4.97

TPH1	NP_004170.1	996	11	10.49	CMTM8	XP_011531718.1	250	10	38.00
SAAL1	NP_612430.2	246	1	3.86	CMTM7	NP_612419.1	191	6	29.84
MRGPRX3	NP_001357393.1	126	0	0.00	CMTM6	NP_060271.1	309	8	24.59
MRGPRX4	NP_473373.2	63	0	0.00	DYNC1LI1	NP_001316064.1	725	7	9.17
SAA4	NP_006503.2	262	4	14.50	CNOT10	NP_056257.1	250	9	34.20
SAA2-SAA4	NP_001186673.1	127	5	37.40	TRIM71	NP_001034200.1	770	5	6.17
SAA2	NP_001372596.1	224	1	4.24	CCR4	XP_016861176.1	580	2	3.28
SAA1	NP_954630.2	982	8	7.74	GLB1	NP_001129074.2	571	6	9.98
HPS5	XP_016872641.1	223	4	17.04	TMPPE	NP_001034859.2	30	6	189.99
GTF2H1	XP_024304225.1	803	4	4.73	CRTAP	NP_006362.1	299	8	25.42
LDHA	NP_001158886.1	1685	6	3.38	SUSD5	XP_005265091.1	149	4	25.50
LDHC	NP_002292.1	896	5	5.30	FBXL2	NP_001336245.1	381	3	7.48
LDHAL6A	XP_011518224.1	620	0	0.00	UBP1	NP_001121632.1	325	4	11.69
TSG101	NP_006283.1	1159	3	2.46	CLASP2	NP_001362634.1	584	7	11.39
UEVLD	NP_001248311.1	1095	2	1.74	PDCD6IP	NP_001155901.1	800	2	2.37
SPTY2D1	NP_919261.2	200	0	0.00	ARPP21	XP_016861063.1	1054	4	3.61
TMEM86A	NP_699178.1	189	1	5.03	STAC	NP_003140.1	182	0	0.00
IGSF22	XP_016873097.1	359	2	5.29	DCLK3	XP_011532470.1	679	7	9.79
PTPN5	XP_016873926.1	1506	4	2.52	TRANK1	XP_016863059.1	188	4	20.21
MRGPRX1	NP_671732.3	134	0	0.00	EPM2AIP1	NP_055620.1	117	8	64.95
MRGPRX2	NP_473371.1	144	0	0.00	MLH1	NP_000240.1	1835	8	4.14
ZDHHC13	NP_061901.2	476	0	0.00	LRRFIP2	NP_001335240.1	360	11	29.03
CSRP3	NP_001356333.1	801	3	3.56	GOLGA4	XP_005265127.1	554	10	17.15
E2F8	XP_011518669.1	814	2	2.33	C3orf35	NP_848029.2	48	7	138.53
NAV2	XP_016874009.1	530	3	5.38	ITGA9	NP_002198.2	609	8	12.48
DBX1	NP_001025036.2	393	3	7.25	CTDSPL	XP_016861008.1	672	11	15.55
HTATIP2	NP_001091990.1	569	1	1.67	VILL	NP_001371967.1	417	10	22.78
PRMT3	NP_005779.1	1263	1	0.75	PLCD1	NP_006216.2	692	9	12.35
SLC6A5	NP_004202.4	616	4	6.17	DLEC1	XP_011532619.1	267	10	35.58
NELL1	NP_001275642.1	865	7	7.69	ACAA1	NP_001123882.1	937	7	7.10
ANO5	NP_998764.1	282	3	10.11	MYD88	NP_001166038.2	1977	4	1.92
SLC17A6	NP_065079.1	1240	4	3.06	OXSR1	XP_011532633.1	407	6	14.00
FANCF	NP_073562.1	318	1	2.99	SLC22A13	NP_004247.2	385	8	19.74
GAS2	XP_016873021.1	604	2	3.15	SLC22A14	XP_006713479.2	246	11	42.48
SVIP	NP_001307270.1	226	0	0.00	XYLB	XP_011532631.1	559	6	10.20
CCDC179	XP_011518104.1	1	0	0.00	ACVR2B	NP_001097.2	1232	4	3.08
LUZP2	XP_016873137.2	194	2	9.79	EXOG	NP_005098.2	253	7	26.28
ANO3	NP_001300655.1	671	3	4.25	SCN5A	XP_016862506.1	958	4	3.97
MUC15	NP_001128564.1	218	1	4.36	SCN10A	NP_001280236.2	768	4	4.95
SLC5A12	XP_006718218.1	368	2	5.16	SCN11A	XP_016861136.1	489	4	7.77
FIBIN	NP_976249.1	297	4	12.79	WDR48	NP_001333154.1	1016	3	2.80
BBOX1	NP_001363190.1	1065	4	3.57	GORASP1	NP_001265719.1	595	2	3.19
CCDC34	NP_110398.1	213	7	31.22	TTC21A	NP_001353828.1	195	5	24.36
LGR4	NP_001333361.1	939	5	5.06	CSRNP1	NP_001307488.1	280	5	16.96
LIN7C	NP_060832.1	550	5	8.64	XIRP1	NP_919269.2	412	4	9.22
BDNF	NP_733927.1	2776	7	2.40	CX3CR1	NP_001164645.1	1061	2	1.79
KIF18A	XP_016873868.1	1317	4	2.89	CCR8	NP_005192.1	587	1	1.62
METTL15	XP_016872786.1	352	1	2.70	SLC25A38	NP_060345.2	304	2	6.25
KCNA4	NP_002224.1	1059	5	4.49	RPSA	NP_001291217.1	1698	4	2.24
FSHB	NP_001369218.1	616	11	16.96	MOBP	NP_891980.1	665	2	2.86
ARL14EP	NP_689529.1	164	3	17.38	MYRIP	NP_001271352.1	529	2	3.59
MPPED2	NP_001364885.1	303	11	34.49	EIF1B	NP_005866.1	574	2	3.31
DCDC1	XP_024304246.1	158	9	54.11	ENTPD3	NP_001239.2	375	1	2.53
DNAJC24	NP_859057.4	537	10	17.69	RPL14	NP_003964.3	1309	3	2.18
IMMP1L	XP_016872795.1	519	10	18.30	ZNF619	NP_001138555.1	110	0	0.00
ELP4	NP_001275654.1	305	10	31.15	ZNF620	XP_016861553.1	171	0	0.00
PAX6	NP_001355840.1	2014	10	4.72	ZNF621	XP_005265136.1	184	1	5.16
RCN1	NP_002892.1	509	10	18.66	CTNNB1	XP_006713048.1	3939	3	0.72
WT1	NP_000369.4	1711	16	8.88	ULK4	NP_060356.2	424	4	8.96
EIF3M	NP_006351.2	1097	14	12.12	TRAK1	XP_016861395.1	374	1	2.54
CCDC73	NP_001008392.2	66	8	115.15	CCK	NP_001167609.1	1539	5	3.09
PRRG4	XP_006718376.3	147	9	58.16	LYZL4	XP_011531657.1	123	0	0.00
QSER1	NP_001070254.2	160	10	59.37	VIPR1	NP_004615.2	482	2	3.94
DEPDC7	NP_001070710.1	214	10	44.39	SEC22C	NP_001188513.1	584	4	6.51
TCP11L1	XP_016873478.1	136	8	55.88	SS18L2	NP_057389.1	273	3	10.44
CSTF3	NP_001317.1	1320	11	7.92	NKTR	NP_001336053.1	1131	5	4.20

HIPK3	XP_016872565.1	1058	11	9.88	ZBTB47	NP_660149.2	413	3	6.90
KIAA1549L	NP_036326.3	274	7	24.27	KLHL40	NP_689606.2	343	2	5.54
C11orf91	XP_016872542.1	30	0	0.00	HHATL	XP_011532272.1	935	3	3.05
CD59	NP_000602.1	1000	4	3.80	CCDC13	XP_011531720.1	151	1	6.29
FBXO3	NP_036307.2	363	3	7.85	HIGD1A	NP_001093138.1	537	1	1.77
LMO2	NP_005565.2	1220	6	4.67	ACKR2	NP_001287.2	655	0	0.00
CAPRIN1	NP_005889.3	658	5	7.22	CYP8B1	NP_004382.2	525	0	0.00
NAT10	NP_078938.3	1149	3	2.48	ZNF662	NP_001128128.1	162	2	11.73
ABTB2	NP_665803.2	262	1	3.63	KRBOX1	NP_001192201.1	123	0	0.00
CAT	NP_001743.1	3276	4	1.16	GASK1A	NP_001123380.2	88	2	21.59
ELF5	NP_001230010.1	467	5	10.17	POMGNT2	XP_016862842.1	209	1	4.55
EHF	NP_001364982.1	554	5	8.57	SNRK	NP_060189.3	266	2	7.14
APIP	NP_057041.2	458	12	24.89	ANO10	NP_001191762.1	286	3	9.96
PDHX	NP_001128496.1	1058	15	13.47	ABHD5	NP_001342115.1	571	2	3.33
CD44	XP_005253288.1	2695	9	3.17	TOPAZ1	XP_011531996.1	142	2	13.38
SLC1A2	XP_011518587.1	1430	6	3.99	TCAIM	NP_776187.2	85	2	22.35
PAMR1	NP_001269605.1	379	9	22.56	ZNF445	NP_001356383.1	242	0	0.00
FJX1	NP_055159.2	263	7	25.28	ZNF852	NP_001274278.1	212	1	4.48
TRIM44	NP_060053.2	202	7	32.92	ZKSCAN7	NP_079445.1	339	1	2.80
LDLRAD3	NP_777562.1	198	6	28.79	ZNF660	NP_775929.2	164	0	0.00
COMMD9	NP_001095123.1	559	7	11.90	ZNF197	NP_001310223.1	276	0	0.00
PRR5L	NP_001153640.1	180	2	10.56	ZNF35	NP_003411.3	291	1	3.26
TRAF6	NP_665802.1	1723	10	5.51	ZNF502	NP_001127912.1	160	0	0.00
RAG1	NP_000439.2	1079	6	5.28	ZNF501	NP_001245209.1	613	1	1.55
RAG2	NP_000527.2	1120	4	3.39	KIAA1143	NP_065747.1	70	0	0.00
IFTAP	NP_620142.2	137	2	13.87	KIF15	XP_016862373.1	1245	0	0.00
LRRC4C	XP_016873559.1	1000	2	1.90	TMEM42	NP_653239.1	70	0	0.00
API5	NP_001136402.1	873	2	2.18	TGM4	XP_011532344.1	236	0	0.00
TTC17	NP_001363454.1	100	2	19.00	ZDHHC3	NP_001336306.1	254	6	22.44
HSD17B12	NP_057226.1	728	2	2.61	EXOSC7	NP_055819.2	887	5	5.35
ALKBH3	NP_631917.1	274	1	3.47	CLEC3B	NP_003269.2	688	5	6.90
C11orf96	NP_001138505.1	37	0	0.00	CDCP1	XP_011532326.1	221	5	21.49
ACCSL	NP_001027025.2	398	0	0.00	TMEM158	NP_056259.2	230	5	20.65
ACCS	XP_011518708.1	403	2	4.71	LARS2	NP_001355192.1	967	6	5.89
EXT2	XP_011518252.1	472	3	6.04	LIMD1	NP_055055.1	799	4	4.76
ALX4	NP_068745.2	846	3	3.37	SACM1L	NP_001306000.1	1130	4	3.36
CD82	NP_002222.1	825	1	1.15	SLC6A20	NP_071800.1	318	3	8.96
TSPAN18	XP_005253274.1	140	0	0.00	LZTFL1	XP_016862134.1	216	4	17.59
TP53I11	NP_001305316.1	182	1	5.22	CCR9	NP_112477.1	754	8	10.08
PRDM11	NP_001371577.1	158	1	6.01	FYCO1	NP_078789.2	419	5	11.34
SYT13	NP_065877.1	708	1	1.34	CXCR6	XP_005264866.1	599	8	12.69
CHST1	XP_016873948.1	753	2	2.52	XCR1	XP_016861686.1	589	8	12.90
SLC35C1	NP_060859.4	331	7	20.09	CCR1	NP_001286.1	1181	9	7.24
CRY2	NP_001120929.1	823	5	5.77	CCR3	XP_016861174.1	897	7	7.41
MAPK8IP1	NP_005447.1	1030	6	5.53	CCR2	NP_001116513.2	1570	8	4.84
C11orf94	NP_001073915.2	17	0	0.00	CCR5	NP_001093638.1	1832	8	4.15
PEX16	NP_476515.2	410	5	11.58	CCRL2	NP_001124382.1	1049	7	6.34
LARGE2	XP_011518193.1	179	4	21.23	LTF	NP_001308051.1	696	6	8.19
PHF21A	XP_016873396.1	1214	4	3.13	RTP3	NP_113628.1	583	5	8.15
CREB3L1	NP_443086.1	411	0	0.00	LRRC2	NP_078788.2	620	5	7.66
DGKZ	XP_024304501.1	418	4	9.09	TDGF1	NP_003203.1	645	1	1.47
MDK	XP_016873253.1	808	3	3.53	ALS2CL	NP_001177636.1	214	5	22.20
CHRM4	NP_001353621.1	663	3	4.30	TMIE	NP_671729.2	200	1	4.75
AMBRA1	NP_001354398.1	786	3	3.63	PRSS50	NP_037402.1	303	0	0.00
HARBI1	XP_016873093.1	130	0	0.00	MYL3	NP_000249.1	1436	3	1.98
ATG13	XP_016874088.1	450	1	2.11	PTH1R	XP_011532270.1	683	2	2.78
ARHGAP1	XP_024304288.1	770	3	3.70	CCDC12	XP_024309132.1	286	6	19.93
ZNF408	NP_079017.1	306	3	9.31	NBEAL2	NP_055990.1	479	6	11.90
F2	NP_000497.1	2039	1	0.47	SETD2	NP_054878.5	2592	7	2.57
CKAP5	NP_055571.2	1230	5	3.86	KIF9	XP_006713354.1	769	8	9.88
LRP4	NP_002325.2	475	0	0.00	KLHL18	XP_005265058.2	249	6	22.89
C11orf49	XP_011518666.1	176	6	32.38	PTPN23	NP_056281.1	1427	8	5.33
ARFGAP2	NP_001229761.1	850	6	6.71	SCAP	XP_005265024.1	458	1	2.07
PACSIN3	NP_057307.2	711	4	5.34	ELP6	NP_001026873.2	232	4	16.38
DDB2	NP_000098.1	811	3	3.51	CSPG5	NP_006565.2	805	4	4.72
ACP2	NP_001343945.1	444	5	10.70	SMARCC1	NP_003065.3	1541	4	2.47

NR1H3	NP_001238863.1	808	3	3.53	DHX30	XP_016861403.1	1158	4	3.28
MADD	NP_001363500.1	458	3	6.22	MAP4	NP_001371610.1	709	3	4.02
MYBPC3	NP_000247.2	456	6	12.50	CDC25A	XP_006713498.1	1626	3	1.75
SPI1	NP_003111.2	1755	5	2.71	CAMP	NP_004336.4	836	1	1.14
SLC39A13	XP_011518768.1	349	12	32.66	ZNF589	NP_057173.2	230	0	0.00
PSMC3	NP_002795.2	1600	7	4.16	NME6	XP_024309066.1	674	0	0.00
RAPSN	XP_011518554.1	337	6	16.91	SPINK8	NP_001073994.1	97	0	0.00
CELF1	NP_001363313.1	977	13	12.64	FBXW12	NP_001153401.1	417	0	0.00
PTPMT1	NP_783859.1	428	12	26.63	PLXNB1	XP_016862120.1	652	1	1.46
KBTBD4	NP_060565.4	192	13	64.32	CCDC51	XP_011532415.1	90	3	31.67
NDUFS3	NP_004542.1	992	11	10.53	TMA7	NP_057017.1	601	2	3.16
FAM180B	NP_001157851.1	56	7	118.74	ATRIP-TREX1	NP_569055.1	426	4	8.92
C1QTNF4	XP_016872654.1	427	10	22.25	TREX1	NP_338599.1	364	0	0.00
MTCH2	XP_011518262.1	593	11	17.62	SHISA5	NP_001258994.1	325	0	0.00
AGBL2	XP_011518675.1	215	2	8.84	PFKFB4	XP_011532131.1	555	1	1.71
FNBP4	NP_056123.2	497	6	11.47	UCN2	NP_149976.1	144	1	6.60
NUP160	NP_056046.1	661	8	11.50	COL7A1	XP_016861177.1	658	3	4.33
PTPRJ	XP_016873572.1	830	7	8.01	UQCRC1	NP_003356.2	1169	3	2.44
OR4B1	NP_001005470.1	79	0	0.00	TMEM89	NP_001008270.1	26	3	109.61
OR4X2	NP_001004727.1	64	0	0.00	SLC26A6	NP_001268662.1	440	2	4.32
OR4X1	NP_001004726.1	226	0	0.00	CELSR3	NP_001398.2	722	4	5.26
OR4S1	NP_001004725.1	140	1	6.79	NCKIPSD	XP_016862084.1	352	3	8.10
OR4C3	NP_001004702.2	164	0	0.00	IP6K2	XP_006713263.1	810	3	3.52
OR4C5	NP_001335152.1	38	0	0.00	PRKAR2A	NP_001308918.1	1151	3	2.48
OR4A47	NP_001005512.2	66	2	28.79	SLC25A20	NP_000378.1	640	4	5.94
TRIM49B	NP_001193555.1	191	2	9.95	ARIH2	NP_001336142.1	509	1	1.87
TRIM64C	NP_001193560.1	162	0	0.00	P4HTM	NP_808807.2	337	1	2.82
FOLH1	NP_001014986.1	705	0	0.00	WDR6	NP_060501.4	484	3	5.89
OR4C13	NP_001001955.2	50	0	0.00	DALRD3	NP_001009996.1	660	4	5.76
OR4C12	NP_001005270.3	132	0	0.00	NDUFAF3	NP_951032.1	504	4	7.54
OR4C46	NP_001004703.1	135	0	0.00	IMPDH2	XP_016861839.1	1405	3	2.03
OR4A5	NP_001005272.3	151	0	0.00	QRICH1	NP_001307513.1	427	0	0.00
TRIM48	NP_077019.2	298	0	0.00	QARS1	NP_001259002.1	1183	2	1.61
OR4A16	NP_001005274.1	76	0	0.00	USP19	NP_001338031.1	707	5	6.72
OR4A15	NP_001005275.1	123	0	0.00	LAMB2	NP_002283.3	582	3	4.90
OR4C15	NP_001001920.2	160	0	0.00	CCDC71	NP_075054.3	61	0	0.00
OR4C16	NP_001004701.2	169	0	0.00	KLHDC8B	XP_006713078.1	119	2	15.97
OR4C11	NP_001004700.2	114	0	0.00	C3orf84	XP_011532319.1	12	0	0.00
OR4P4	NP_001004124.1	164	0	0.00	IHO1	NP_835467.2	110	4	34.54
OR4S2	NP_001004059.2	161	0	0.00	C3orf62	NP_940964.1	41	2	46.34
OR4C6	NP_001004704.1	124	0	0.00	USP4	NP_955475.1	808	4	4.70
OR5D13	NP_001001967.1	118	0	0.00	GPX1	NP_001316384.1	1069	1	0.89
OR5D14	NP_001004735.1	90	0	0.00	RHOA	NP_001300873.1	3746	3	0.76
OR5L1	NP_001004738.1	99	0	0.00	TCTA	NP_071503.1	172	2	11.05
OR5D18	NP_001001952.1	118	0	0.00	AMT	NP_001158184.1	563	4	6.75
OR5L2	NP_001004739.1	124	0	0.00	NICN1	NP_115692.1	120	1	7.92
OR5D16	XP_024304287.1	96	0	0.00	DAG1	NP_004384.5	867	6	6.57
TRIM51	NP_116070.2	267	0	0.00	BSN	NP_003449.2	1639	3	1.74
OR5W2	NP_001001960.1	131	0	0.00	APEH	XP_024309267.1	761	7	8.74
OR5I1	NP_006628.1	144	0	0.00	MST1	XP_006713229.1	317	5	14.98
OR10AG1	NP_001005491.1	35	0	0.00	RNF123	XP_016862507.1	864	9	9.90
OR5F1	NP_003688.1	54	0	0.00	AMIGO3	NP_942015.1	74	1	12.84
OR5AS1	NP_001001921.1	140	0	0.00	GMPPB	NP_068806.2	716	2	2.65
OR8I2	NP_001003750.1	62	0	0.00	IP6K1	NP_695005.1	762	8	9.97
OR8H2	NP_001372993.1	72	0	0.00	CDHR4	XP_016861858.1	198	8	38.38
OR8H3	NP_001005201.1	130	0	0.00	INKA1	NP_976248.2	49	2	38.77
OR8J3	NP_001004064.1	55	0	0.00	UBA7	XP_011532372.1	1257	10	7.56
OR8K5	NP_001004058.2	121	0	0.00	TRAIP	XP_016861015.1	755	8	10.07
OR5J2	NP_001005492.1	138	0	0.00	CAMKV	NP_001307076.1	1152	10	8.25
OR5T2	NP_001004746.1	55	0	0.00	MST1R	NP_001305842.1	1518	13	8.14
OR5T3	NP_001004747.1	40	0	0.00	MON1A	NP_001135973.1	484	7	13.74
OR5T1	NP_001004745.1	122	0	0.00	RBM6	NP_005768.1	392	4	9.69
OR8H1	NP_001005199.1	53	0	0.00	RBM5	XP_016860992.1	872	2	2.18
OR8K3	NP_001005202.1	147	0	0.00	SEMA3F	NP_004177.3	640	5	7.42
OR8K1	NP_001002907.1	61	0	0.00	GNAT1	NP_000163.2	979	4	3.88
OR8J1	NP_001005205.2	71	0	0.00	SLC38A3	XP_006713017.1	513	1	1.85

OR8U1	NP_001005204.1	196	0	0.00	GNAI2	NP_001269549.1	1423	4	2.67
OR8U3	NP_001004744.1	112	0	0.00	SEMA3B	NP_001276990.1	555	9	15.40
OR5M9	NP_001004743.1	84	0	0.00	LSMEM2	XP_006713042.1	30	0	0.00
OR5M3	NP_001004742.2	109	0	0.00	IFRD2	NP_006755.5	288	4	13.19
OR5M8	NP_001005282.1	38	0	0.00	HYAL3	NP_001186961.1	130	3	21.92
OR5M11	NP_001005245.1	61	0	0.00	NAA80	NP_036323.2	25	1	38.00
OR5M10	NP_001004741.1	52	0	0.00	HYAL1	NP_695014.1	385	9	22.21
OR5M1	NP_001004740.1	61	0	0.00	HYAL2	NP_003764.3	327	11	31.96
OR5AP2	NP_001002925.1	88	0	0.00	TUSC2	NP_009206.1	176	9	48.58
OR5AR1	NP_001004730.1	151	0	0.00	RASSF1	NP_009113.3	931	10	10.20
OR9G1	NP_001005213.1	106	0	0.00	ZMYND10	NP_001295308.1	282	8	26.95
OR9G4	NP_001005284.1	137	0	0.00	NPRL2	NP_006536.3	320	10	29.69
OR5AK2	NP_001005323.1	40	0	0.00	CYB561D2	NP_008953.1	89	10	106.74
LRRC55	NP_001005210.2	589	1	1.61	TMEM115	NP_008955.1	281	6	20.28
APLNR	NP_005152.1	880	0	0.00	CACNA2D2	NP_006021.2	490	11	21.33
TNKS1BP1	NP_203754.2	302	0	0.00	C3orf18	XP_016862034.1	112	6	50.89
SSRP1	XP_016873669.1	1794	0	0.00	HEMK1	XP_011532112.1	370	0	0.00
P2RX3	NP_002550.2	342	0	0.00	CISH	NP_659508.1	1178	4	3.23
PRG3	NP_006084.2	292	0	0.00	MAPKAPK3	NP_001230854.1	446	6	12.78
PRG2	NP_001230174.1	281	2	6.76	DOCK3	XP_005264971.1	893	7	7.45
SLC43A3	NP_060081.1	288	2	6.60	MANF	NP_006001.5	535	2	3.55
RTN4RL2	NP_848665.1	626	1	1.52	RBM15B	NP_037418.3	362	5	13.12
SLC43A1	XP_011543622.1	240	2	7.92	DCAF1	NP_001165375.1	795	4	4.78
TIMM10	XP_024304204.1	874	1	1.09	RAD54L2	NP_001309182.1	867	2	2.19
SMTNL1	NP_001099035.2	1062	1	0.89	TEX264	NP_001123356.1	230	0	0.00
UBE2L6	NP_004214.1	1513	1	0.63	GRM2	XP_016861760.1	755	1	1.26
SERPING1	NP_000053.2	724	1	1.31	IQCF6	NP_001355297.1	37	0	0.00
YPEL4	NP_001350416.1	240	1	3.96	IQCF3	NP_001078948.1	1	0	0.00
CLP1	NP_006822.1	530	0	0.00	IQCF2	NP_982248.1	32	0	0.00
ZDHHC5	NP_056272.2	332	2	5.72	IQCF5	NP_001138531.1	5	0	0.00
MED19	NP_001304007.2	267	0	0.00	IQCF1	NP_689610.2	74	0	0.00
TMX2	NP_001334819.1	184	0	0.00	RRP9	NP_004695.1	1077	3	2.65
SELENOH	NP_001308264.1	153	0	0.00	PARP3	XP_016860979.1	486	1	1.95
BTBD18	XP_016873617.1	127	0	0.00	GPR62	NP_543141.3	550	1	1.73
CTNND1	NP_001078927.1	1081	1	0.88	PCBP4	XP_024309446.1	451	1	2.11
OR6Q1	NP_001005186.2	96	0	0.00	ABHD14B	NP_001241682.1	375	0	0.00
OR9I1	NP_001005211.1	31	0	0.00	ABHD14A	NP_056222.2	279	2	6.81
OR9Q1	NP_001005212.1	57	0	0.00	ABHD14A-AC\	NP_001303260.1	394	0	0.00
OR9Q2	NP_001005283.1	197	0	0.00	ACY1	NP_001185824.1	665	3	4.29
OR1S2	NP_001004459.1	56	0	0.00	RPL29	NP_000983.1	1019	4	3.73
OR1S1	NP_001004458.1	65	0	0.00	DUSP7	NP_001938.2	741	2	2.56
OR10Q1	NP_001004471.1	41	1	23.17	POC1A	XP_011531863.1	472	5	10.06
OR10W1	NP_997257.2	40	0	0.00	ALAS1	NP_001291373.1	823	5	5.77
OR5B17	NP_001005489.1	45	0	0.00	TLR9	NP_059138.1	2080	0	0.00
OR5B3	NP_001005469.1	105	0	0.00	TWF2	NP_009215.1	434	8	17.51
OR5B2	NP_001005566.1	29	0	0.00	PPM1M	NP_653242.3	144	4	26.39
OR5B12	NP_001004733.1	23	0	0.00	WDR82	NP_079498.2	1090	6	5.23
OR5B21	NP_001005218.1	26	0	0.00	GLYCTK	XP_024309119.1	284	4	13.38
LPXN	NP_001137467.1	845	1	1.12	DNAH1	XP_016861618.1	839	7	7.93
ZFP91	NP_444251.1	421	3	6.77	BAP1	XP_016862792.1	720	1	1.32
CNTF	NP_000605.1	845	1	1.12	PHF7	NP_001308055.1	1522	6	3.74
GLYAT	NP_964011.2	217	2	8.76	SEMA3G	NP_064548.1	455	0	0.00
GLYATL2	XP_016872826.1	71	0	0.00	TNNC1	NP_003271.1	1096	0	0.00
GLYATL1	NP_542392.2	224	0	0.00	NISCH	NP_009115.3	614	3	4.64
FAM111B	NP_945185.1	235	1	4.04	STAB1	XP_016861487.1	697	8	10.90
FAM111A	NP_001356386.1	151	2	12.58	NT5DC2	NP_075059.1	227	10	41.85
DTX4	NP_055992.1	387	1	2.45	SMIM4	XP_011532031.1	153	1	6.21
MPEG1	NP_001034485.1	668	0	0.00	PBRM1	NP_001353003.1	1701	12	6.70
OR5AN1	NP_001004729.1	90	0	0.00	GNL3	NP_055181.3	1243	11	8.41
OR5A2	NP_001001954.1	106	0	0.00	GLT8D1	NP_690909.1	168	12	67.85
OR5A1	NP_001004728.1	51	0	0.00	SPCS1	NP_054760.4	672	12	16.96
OR4D6	NP_001004708.1	104	1	9.13	NEK4	NP_001180462.1	313	13	39.45
OR4D10	NP_001004705.1	126	0	0.00	ITIH1	NP_002206.2	408	12	27.94
OR4D11	NP_001004706.1	89	0	0.00	ITIH3	XP_005265162.1	590	13	20.93
OR4D9	NP_001004711.1	77	1	12.34	ITIH4	NP_001159921.1	993	12	11.48
OSBP	NP_002547.1	741	4	5.13	STIMATE-MU!	NP_001185903.2	207	7	32.12

PATL1	NP_689929.2	623	0	0.00	MUSTN1	NP_995325.4	94	11	111.16
OR10V1	NP_001005324.1	37	0	0.00	STIMATE	NP_940965.1	96	10	98.95
STX3	XP_005274255.1	769	2	2.47	SFMBT1	XP_005265278.1	442	7	15.04
MRPL16	NP_060310.1	1221	2	1.56	RFT1	XP_011532517.1	124	0	0.00
CBLIF	XP_011543241.1	270	2	7.04	PRKCD	NP_001341605.1	1553	7	4.28
TCN1	NP_001053.2	363	6	15.70	TKT	NP_001128527.1	1149	2	1.65
OOSP2	NP_776162.2	62	4	61.29	DCP1A	NP_001277133.1	836	2	2.27
MS4A3	NP_006129.4	296	9	28.88	CACNA1D	NP_001122312.1	1060	5	4.48
MS4A2	XP_005273903.1	276	8	27.53	CHDH	XP_016862286.1	492	7	13.52
MS4A6A	XP_006718723.1	429	8	17.71	IL17RB	XP_005265367.1	293	3	9.73
MS4A4E	NP_001338164.1	89	6	64.04	ACTR8	NP_075050.3	840	5	5.65
MS4A4A	NP_683876.1	487	6	11.70	SELENOK	NP_067060.2	244	3	11.68
MS4A6E	NP_001363838.1	177	11	59.04	CACNA2D3	NP_060868.2	499	4	7.61
MS4A7	NP_067024.1	312	6	18.27	LRTM1	NP_065729.1	471	7	14.12
MS4A14	NP_001248757.1	391	12	29.15	WNT5A	XP_016862616.1	1764	5	2.69
MS4A5	NP_076434.2	85	11	122.93	ERC2	XP_016861645.1	753	6	7.57
MS4A1	NP_068769.2	573	12	19.89	CCDC66	NP_001340076.1	179	2	10.61
MS4A12	NP_060186.2	189	5	25.13	TASOR	NP_001350869.1	191	1	4.97
MS4A13	NP_001012417.2	2	0	0.00	ARHGEF3	NP_001364336.1	772	4	4.92
MS4A8	NP_113645.1	155	7	42.90	SPATA12	XP_024309278.1	40	0	0.00
MS4A18	NP_001341400.1	18	4	211.10	IL17RD	NP_060033.3	303	1	3.14
MS4A15	NP_001092305.1	60	4	63.33	HESX1	NP_001362988.1	669	5	7.10
MS4A10	NP_996776.2	2	2	949.95	APPL1	NP_036228.1	2501	6	2.28
CCDC86	NP_077003.1	336	2	5.65	ASB14	XP_016861225.1	1261	9	6.78
PTGDR2	NP_004769.2	508	2	3.74	DNAH12	XP_011531769.1	640	4	5.94
ZP1	NP_997224.2	131	0	0.00	PDE12	NP_001309105.1	515	2	3.69
PRPF19	NP_055317.1	1797	0	0.00	ARF4	NP_001651.1	1886	4	2.01
TMEM109	NP_076997.1	192	0	0.00	DENND6A	XP_006713082.1	141	0	0.00
TMEM132A	NP_060340.2	316	0	0.00	SLMAP	XP_005265513.1	918	3	3.10
SLC15A3	NP_057666.1	634	2	3.00	FLNB	NP_001157789.1	1082	4	3.51
CD6	NP_006716.3	515	2	3.69	DNASE1L3	NP_001243489.1	322	3	8.85
CD5	NP_055022.2	1034	4	3.67	ABHD6	XP_005265391.1	403	4	9.43
VPS37C	XP_005274134.1	298	0	0.00	HTD2	NP_001092253.1	379	1	2.51
PGA3	NP_001073275.1	295	0	0.00	PXK	NP_060241.2	348	3	8.19
PGA4	NP_001073276.1	267	1	3.56	PDHB	NP_001302465.1	1017	3	2.80
PGA5	NP_055039.1	348	0	0.00	KCTD6	NP_699162.3	475	1	2.00
VWCE	XP_016872830.1	671	1	1.42	ACOX2	NP_003491.1	737	3	3.87
DDB1	NP_001914.3	1613	0	0.00	FAM107A	NP_001269643.1	699	4	5.44
TKFC	NP_001338906.1	393	0	0.00	FAM3D	XP_006713030.1	178	1	5.34
CYB561A3	XP_011543123.1	178	1	5.34	CFAP20DC	NP_001338459.1	205	0	0.00
TMEM138	XP_011543401.1	145	2	13.10	FHIT	NP_001341519.1	802	5	5.92
TMEM216	NP_001167462.1	347	1	2.74	PTPRG	XP_016862450.1	1049	4	3.62
CPSF7	XP_016873834.1	701	1	1.36	C3orf14	NP_065736.1	162	7	41.05
SDHAF2	NP_060311.1	271	1	3.51	FEZF2	NP_060478.3	825	3	3.45
PPP1R32	NP_659454.2	123	1	7.72	CADPS	NP_899630.1	900	8	8.44
LRRC10B	NP_001138549.1	486	1	1.95	SYNPR	XP_016861220.1	1212	6	4.70
SYT7	XP_011543645.1	749	6	7.61	SNTN	NP_001335685.1	79	0	0.00
DAGLA	XP_016873727.1	349	9	24.50	C3orf49	NP_001342165.1	0	0	#DIV/0!
MYRF	XP_005274279.1	419	8	18.14	THOC7	NP_079351.2	625	9	13.68
TMEM258	NP_055021.1	359	9	23.81	ATXN7	NP_001170858.1	757	12	15.06
FEN1	NP_004102.1	1989	9	4.30	PSMD6	NP_001258709.1	1277	8	5.95
FADS1	NP_037534.5	716	8	10.61	PRICKLE2	XP_011531737.1	811	10	11.71
FADS2	NP_001268430.1	699	8	10.87	ADAMTS9	NP_001305710.1	530	8	14.34
FADS3	XP_016873213.1	399	7	16.67	MAGI1	NP_001028229.1	740	8	10.27
RAB3IL1	NP_001258615.1	461	8	16.49	SLC25A26	NP_001337920.1	302	8	25.16
BEST1	XP_011543532.1	612	10	15.52	LRIG1	NP_001364273.1	918	10	10.35
FTH1	NP_002023.2	1042	8	7.29	KBTBD8	NP_115894.2	618	1	1.54
INCENP	XP_011543297.1	829	2	2.29	SUCLG2	NP_001171070.1	1367	0	0.00
SCGB1D1	NP_006543.1	221	0	0.00	TAFA1	XP_016861875.1	491	1	1.93
SCGB2A1	NP_002398.1	207	0	0.00	TAFA4	XP_011531673.1	217	1	4.38
SCGB1D2	NP_006542.1	180	0	0.00	EOGT	NP_775925.1	274	7	24.27
SCGB2A2	NP_002402.1	174	0	0.00	TMF1	NP_009045.2	525	6	10.86
SCGB1D4	NP_996881.1	274	0	0.00	UBA3	NP_001350790.1	1192	8	6.38
ASRGL1	XP_011543567.1	370	0	0.00	ARL6IP5	NP_006398.1	376	5	12.63
SCGB1A1	NP_003348.1	669	4	5.68	LMOD3	NP_938012.2	294	6	19.39
AHNAK	NP_076965.2	754	5	6.30	FRMD4B	NP_055938.2	395	8	19.24

EEF1G	NP_001395.1	1993	6	2.86	MITF	NP_001341533.1	1283	3	2.22
TUT1	NP_073741.3	510	1	1.86	FOXP1	NP_001231741.1	1302	11	8.03
MTA2	NP_004730.2	822	0	0.00	EIF4E3	XP_011531953.1	527	7	12.62
EML3	NP_001287723.1	617	5	7.70	GPR27	NP_061844.1	374	8	20.32
ROM1	NP_000318.2	475	4	8.00	PROK2	NP_068754.1	617	8	12.32
B3GAT3	NP_036332.2	281	1	3.38	RYBP	NP_036366.3	521	9	16.41
GANAB	NP_001265122.1	713	5	6.66	SHQ1	NP_060600.2	316	9	27.06
INTS5	NP_085131.1	288	9	29.69	GXYLT2	XP_011532369.1	147	9	58.16
C11orf98	NP_001273015.1	12	0	0.00	PPP4R2	NP_777567.1	659	10	14.42
LBHD1	NP_001354870.1	51	2	37.25	EBLN2	NP_060499.3	55	3	51.82
CSKMT	XP_005274289.1	123	2	15.45	PDZRN3	XP_016861431.1	1056	8	7.20
UQCC3	NP_001078841.1	113	1	8.41	CNTN3	XP_016861996.1	851	5	5.58
UBXN1	NP_001273007.1	450	0	0.00	FRG2C	NP_001118231.1	70	3	40.71
LRRN4CL	NP_981967.1	22	5	215.90	ZNF717	XP_011531551.1	284	4	13.38
BSCL2	NP_001116427.1	469	9	18.23	ROBO2	NP_001365125.1	1032	9	8.28
GNG3	XP_006718563.1	1274	2	1.49	ROBO1	XP_016862471.1	1038	9	8.24
HNRNPUL2	NP_001073027.1	386	7	17.23	GBE1	NP_000149.4	897	10	10.59
TTC9C	NP_776171.1	161	3	17.70	CADM2	XP_016861551.1	1062	10	8.94
ZBTB3	NP_001350038.1	323	4	11.76	VGLL3	NP_001307423.1	299	7	22.24
POLR2G	NP_002687.1	1313	6	4.34	CHMP2B	NP_054762.2	526	7	12.64
TAF6L	NP_006464.1	483	0	0.00	POU1F1	NP_000297.1	529	8	14.37
TMEM179B	NP_001350530.1	110	0	0.00	HTR1F	NP_001309139.1	438	6	13.01
TMEM223	NP_001073970.1	140	0	0.00	CGGBP1	XP_024309565.1	268	8	28.36
NXF1	NP_006353.2	1244	2	1.53	ZNF654	NP_001337063.1	174	11	60.05
STX5	NP_003155.2	730	5	6.51	C3orf38	NP_776185.2	85	11	122.93
WDR74	NP_001294906.1	604	1	1.57	EPHA3	NP_005224.2	1551	8	4.90
SLC3A2	NP_002385.3	1141	2	1.67	PROS1	NP_000304.2	492	2	3.86
CHRM1	NP_000729.2	1388	3	2.05	ARL13B	NP_001167621.1	1800	5	2.64
SLC22A6	NP_695009.1	545	4	6.97	STX19	NP_001001850.1	474	2	4.01
SLC22A8	NP_004245.2	397	7	16.75	DHFR2	NP_789785.1	822	0	0.00
SLC22A24	NP_001129978.2	201	1	4.73	NSUN3	NP_071355.1	722	0	0.00
SLC22A25	XP_016873181.1	166	1	5.72	EPHA6	XP_006713655.1	1304	3	2.19
SLC22A10	NP_001034841.3	202	2	9.41	ARL6	NP_001310442.1	1323	4	2.87
SLC22A9	NP_543142.2	300	2	6.33	CRYBG3	NP_705833.3	74	0	0.00
PLAAT5	XP_011543054.1	130	0	0.00	RIOX2	NP_116167.3	211	1	4.50
LGALS12	XP_016873947.1	113	0	0.00	GABRR3	NP_001099050.1	1053	1	0.90
PLAAT4	NP_004576.2	272	1	3.49	OR5AC2	NP_473447.1	161	0	0.00
PLAAT2	XP_011543422.1	165	1	5.76	OR5H1	NP_001005338.1	65	0	0.00
PLAAT3	XP_011543043.1	288	0	0.00	OR5H14	NP_001005514.1	37	0	0.00
ATL3	XP_006718556.1	309	1	3.07	OR5H15	NP_001005515.1	75	0	0.00
RTN3	NP_001252520.1	665	3	4.29	OR5H6	NP_001005479.2	61	0	0.00
C11orf95	XP_024304430.1	103	3	27.67	OR5H2	NP_001005482.1	48	0	0.00
SPINDOC	XP_011543072.1	26	3	109.61	OR5K4	NP_001005517.1	105	1	9.05
MARK2	NP_001034558.2	973	3	2.93	OR5K3	NP_001005516.1	121	0	0.00
RCOR2	NP_001350577.1	537	0	0.00	OR5K1	NP_001004736.2	44	0	0.00
NAA40	XP_011543556.1	174	1	5.46	OR5K2	NP_001004737.1	44	0	0.00
COX8A	NP_004065.1	592	1	1.60	CLDND1	NP_001035290.1	98	1	9.69
OTUB1	NP_060140.2	658	1	1.44	GPR15	NP_005281.1	409	4	9.29
MACROD1	XP_011543272.1	358	1	2.65	CPOX	XP_005247182.1	872	1	1.09
FLRT1	NP_001371395.1	711	0	0.00	ST3GAL6	NP_001258074.1	387	1	2.45
STIP1	NP_001269581.1	1589	2	1.20	DCBLD2	XP_011510721.1	345	1	2.75
FERMT3	NP_001369291.1	702	1	1.35	COL8A1	NP_001841.2	531	4	7.16
TRPT1	NP_001153864.1	96	1	9.90	CMSS1	NP_115735.2	709	2	2.68
NUDT22	XP_024304482.1	153	2	12.42	FILIP1L	NP_878913.2	274	2	6.93
DNAJC4	NP_005519.2	266	4	14.28	TBC1D23	NP_001186127.1	249	0	0.00
VEGFB	NP_001230662.1	429	3	6.64	NIT2	NP_064587.1	571	1	1.66
PPP1R14B	NP_619634.1	525	2	3.62	TOMM70	NP_055635.3	782	3	3.64
PLCB3	XP_011543403.1	953	1	1.00	LNP1	NP_001078920.1	115	0	0.00
BAD	NP_004313.1	316	1	3.01	TMEM45A	NP_001350805.1	378	0	0.00
GPR137	XP_011543470.1	190	7	35.00	ADGRG7	NP_116176.2	378	3	7.54
KCNK4	NP_201567.1	637	5	7.46	TFG	NP_006061.2	483	3	5.90
CATSPERZ	NP_001034585.1	95	4	40.00	ABI3BP	XP_011510958.1	247	2	7.69
ESRRA	XP_016872802.1	744	6	7.66	IMPG2	NP_057331.2	254	2	7.48
TRMT112	NP_001273011.1	905	4	4.20	SENP7	XP_005247719.1	534	2	3.56
PRDX5	NP_036226.2	719	4	5.28	TRMT10C	NP_060289.2	619	2	3.07
CCDC88B	XP_011543259.1	265	5	17.92	PCNP	NP_065090.1	467	3	6.10

RPS6KA4	NP_003933.1	575	3	4.96	ZBTB11	NP_055230.2	412	0	0.00
SLC22A11	NP_060954.1	247	1	3.85	RPL24	NP_000977.1	1240	1	0.77
SLC22A12	XP_006718493.1	331	2	5.74	CEP97	NP_078824.2	832	4	4.57
NRXN2	XP_016874058.1	1259	7	5.28	NXPE3	NP_001335919.1	81	0	0.00
RASGRP2	XP_011543020.1	846	5	5.61	NFKBIZ	NP_113607.1	1367	1	0.69
PYGM	NP_001158188.1	1033	5	4.60	ZPLD1	NP_778226.1	122	2	15.57
SF1	NP_001333293.1	1066	5	4.46	ALCAM	NP_001618.2	648	1	1.47
MAP4K2	XP_016873582.1	333	6	17.12	CBLB	XP_016862887.1	832	1	1.14
MEN1	NP_001357192.1	967	7	6.88	CCDC54	NP_115989.1	200	1	4.75
CDC42BPG	XP_011543458.1	1046	9	8.17	BBX	XP_024309430.1	292	3	9.76
EHD1	NP_001269374.1	705	3	4.04	CD47	NP_942088.1	858	3	3.32
ATG2A	NP_001354900.1	436	2	4.36	IFT57	NP_060480.1	597	8	12.73
PPP2R5B	XP_011543434.1	933	9	9.16	HHLA2	NP_001269488.1	131	7	50.76
GPHA2	XP_011543076.1	266	1	3.57	MYH15	NP_055796.1	1015	7	6.55
MAJIN	NP_001287732.1	30	0	0.00	CIP2A	XP_006713779.1	520	7	12.79
BATF2	NP_612465.3	480	1	1.98	DZIP3	XP_016863029.1	664	8	11.45
ARL2	NP_001658.2	418	3	6.82	RETNLB	NP_115968.1	335	1	2.84
SNX15	NP_037438.2	301	1	3.16	TRAT1	NP_057472.2	748	8	10.16
SAC3D1	NP_037431.3	608	2	3.12	GUCA1C	NP_005450.3	869	7	7.65
NAALADL1	XP_011543010.1	318	1	2.99	MORC1	NP_055244.3	292	8	26.03
CDCA5	XP_011543045.1	652	2	2.91	DPPA2	XP_011510745.1	249	1	3.82
ZFPL1	NP_006773.2	238	0	0.00	DPPA4	NP_060659.3	502	0	0.00
TMEM262	NP_001229560.1	4	0	0.00	NECTIN3	XP_016861612.1	432	1	2.20
VPS51	NP_037397.2	588	3	4.85	CD96	NP_937839.1	352	3	8.10
TM7SF2	NP_003264.2	589	2	3.23	ZBED2	NP_078784.2	109	0	0.00
ZNHIT2	NP_055020.1	340	4	11.18	PLCXD2	NP_695000.1	158	1	6.01
FAU	NP_001988.1	2630	10	3.61	PHLDB2	NP_001127909.1	222	1	4.28
MRPL49	NP_004918.1	411	1	2.31	ABHD10	NP_060864.1	371	0	0.00
SYVN1	NP_115807.1	759	2	2.50	TAGLN3	NP_037391.2	1197	0	0.00
SPDYC	NP_001008778.1	22	0	0.00	TMPRSS7	XP_011511056.1	177	0	0.00
CAPN1	NP_005177.2	1074	2	1.77	C3orf52	NP_001165218.1	81	0	0.00
POLA2	XP_011543179.1	822	3	3.47	GCSAM	NP_001177189.1	182	1	5.22
CDC42EP2	NP_006770.1	235	10	40.42	SLC9C1	XP_016861735.1	488	1	1.95
DPF2	XP_024304405.1	499	11	20.94	CD200	NP_001004196.2	607	4	6.26
TIGD3	NP_663771.1	95	10	99.99	BTLA	NP_001078826.1	436	4	8.72
SLC25A45	NP_001265179.3	205	11	50.97	ATG3	NP_071933.2	712	1	1.33
FRMD8	NP_114110.1	187	10	50.80	SLC35A5	NP_001335834.1	320	3	8.91
SCYL1	XP_024304387.1	342	3	8.33	CCDC80	NP_955805.1	380	2	5.00
LTBP3	NP_066548.2	555	11	18.83	CD200R1L	NP_001008784.2	78	1	12.18
ZNRD2	NP_006387.1	300	10	31.67	CD200R1	NP_740750.1	351	2	5.41
FAM89B	NP_001092255.1	104	10	91.34	GTPBP8	NP_054889.2	261	6	21.84
EHBP1L1	XP_006718549.1	1042	11	10.03	NEPRO	NP_001306038.1	157	0	0.00
KCNK7	NP_203133.1	127	11	82.28	BOC	XP_016862941.1	471	0	0.00
MAP3K11	NP_002410.1	1252	13	9.86	CFAP44	NP_001157968.1	526	1	1.81
PCNX3	XP_011543326.1	188	5	25.26	SPICE1	NP_001318008.1	536	7	12.41
SIPA1	NP_006738.3	551	9	15.52	SIDT1	NP_001309223.1	265	4	14.34
RELA	NP_001138610.1	1987	8	3.82	USF3	XP_016861360.1	220	4	17.27
KAT5	NP_874369.1	1798	5	2.64	NAA50	NP_001295374.1	1176	6	4.85
RNASEH2C	NP_115569.2	166	3	17.17	ATP6V1A	NP_001681.2	1292	4	2.94
AP5B1	NP_612377.4	488	3	5.84	GRAMD1C	NP_060047.3	131	4	29.01
OVOL1	NP_004552.2	521	8	14.59	ZDHHC23	XP_006713625.1	241	9	35.48
SNX32	XP_024304185.1	168	7	39.58	CCDC191	NP_001340696.2	8	0	0.00
CFL1	NP_005498.1	2177	7	3.05	QTRT2	NP_001243764.1	473	0	0.00
MUS81	NP_001337212.1	748	9	11.43	DRD3	NP_387512.3	1016	5	4.67
EFEMP2	NP_058634.4	612	3	4.66	ZNF80	NP_009067.2	195	3	14.61
CTSW	NP_001326.3	475	3	6.00	TIGIT	XP_024309156.1	305	2	6.23
FIBP	NP_004205.2	277	5	17.15	ZBTB20	NP_001335729.1	644	6	8.85
CCDC85B	NP_006839.2	198	3	14.39	GAP43	NP_002036.1	1572	5	3.02
FOSL1	NP_001287786.1	1151	6	4.95	LSAMP	NP_002329.2	741	5	6.41
C11orf68	NP_113638.2	232	2	8.19	IGSF11	NP_001340251.1	364	0	0.00
DRAP1	NP_006433.2	335	4	11.34	TEX55	NP_689752.2	124	0	0.00
TSGA10IP	XP_011543189.1	29	0	0.00	UPK1B	NP_008883.2	365	0	0.00
SART1	NP_005137.1	1088	6	5.24	B4GALT4	XP_016862890.1	263	0	0.00
EIF1AD	XP_016873901.1	209	1	4.55	ARHGAP31	NP_065805.2	275	3	10.36
BANF1	XP_016874003.1	1138	3	2.50	TMEM39A	XP_016862277.1	104	3	27.40
CST6	NP_001314.1	251	5	18.92	POGLUT1	NP_689518.1	295	4	12.88

CATSPER1	NP_444282.3	524	7	12.69	TIMMDC1	NP_057673.2	257	3	11.09
GAL3ST3	NP_149025.1	195	8	38.97	CD80	NP_005182.1	1529	2	1.24
SF3B2	NP_006833.2	1145	9	7.47	ADPRH	NP_001358021.1	168	1	5.65
PACS1	NP_060496.2	521	11	20.06	PLA1A	NP_056984.1	275	1	3.45
KLC2	XP_024304425.1	1097	3	2.60	POPDC2	NP_071418.2	265	1	3.58
RAB1B	XP_016873867.1	1007	9	8.49	COX17	NP_001368932.1	590	1	1.61
CNIH2	NP_872359.1	707	5	6.72	CFAP91	NP_203528.3	160	2	11.87
YIF1A	NP_001287790.1	554	5	8.57	NR1I2	NP_071285.1	897	2	2.12
TMEM151A	NP_694998.1	687	1	1.38	GSK3B	NP_001341525.1	3475	4	1.09
CD248	NP_065137.1	548	0	0.00	GPR156	NP_001161743.1	169	1	5.62
RIN1	NP_001350488.1	440	6	12.95	LRRC58	NP_001093148.1	457	3	6.24
BRMS1	XP_024304194.1	457	6	12.47	FSTL1	NP_009016.1	746	2	2.55
B4GAT1	NP_006867.1	138	0	0.00	NDUFB4	NP_004538.2	449	1	2.12
SLC29A2	XP_024304232.1	354	1	2.68	HGD	XP_005247469.1	706	1	1.35
NPAS4	XP_016873026.1	458	1	2.07	RABL3	NP_001350893.1	180	0	0.00
MRPL11	NP_733934.1	995	1	0.95	GTF2E1	NP_005504.2	910	0	0.00
PELI3	NP_659502.2	217	0	0.00	STXBP5L	NP_001335272.1	997	3	2.86
DPP3	NP_005691.2	454	2	4.18	POLQ	NP_955452.3	710	1	1.34
BBS1	NP_078925.3	623	2	3.05	ARGFX	NP_001012677.1	249	0	0.00
ZDHHC24	XP_005273931.1	76	0	0.00	FBXO40	NP_057382.2	528	2	3.60
ACTN3	NP_001245300.2	1120	2	1.70	HCLS1	NP_001278970.2	821	2	2.31
CTSF	NP_003784.2	556	0	0.00	GOLGB1	XP_006713654.1	677	1	1.40
CCDC87	NP_060689.2	22	0	0.00	IQCB1	NP_001018865.2	1203	1	0.79
CCS	NP_005116.1	437	0	0.00	EAF2	NP_060926.2	236	0	0.00
RBM14	NP_006319.1	897	1	1.06	SLC15A2	NP_066568.3	493	0	0.00
RBM14-RBM4	NP_001185775.1	324	1	2.93	ILDR1	NP_001186729.1	287	0	0.00
RBM4	NP_001185773.1	497	1	1.91	CD86	NP_787058.5	1631	2	1.16
RBM4B	NP_001273064.1	215	1	4.42	CASR	NP_001171536.2	1043	2	1.82
SPTBN2	XP_016873663.1	1155	3	2.47	CSTA	NP_005204.1	458	6	12.44
C11orf80	NP_078926.3	77	0	0.00	CCDC58	NP_001017928.1	477	5	9.96
RCE1	NP_005124.1	344	2	5.52	FAM162A	NP_055182.3	290	3	9.83
PC	XP_016873357.1	1026	3	2.78	WDR5B	NP_061942.2	498	4	7.63
LRFN4	NP_001350453.1	739	4	5.14	KPNA1	XP_024309282.1	973	1	0.98
C11orf86	NP_001340483.1	7	1	135.71	PARP9	XP_016862794.1	440	6	12.95
SYT12	XP_011543648.1	524	0	0.00	DTX3L	NP_612144.1	527	3	5.41
RHOD	NP_055393.1	2080	6	2.74	PARP15	NP_001106995.1	264	2	7.20
KDM2A	NP_036440.1	1087	2	1.75	PARP14	NP_060024.2	499	3	5.71
GRK2	NP_001610.2	1470	1	0.65	HSPBAP1	XP_016862668.1	513	2	3.70
ANKRD13D	XP_011543287.1	123	0	0.00	SLC49A4	NP_116228.1	279	1	3.40
SSH3	NP_060327.3	936	3	3.04	SEMA5B	NP_001243276.1	705	1	1.35
POLD4	NP_001243799.1	187	0	0.00	PDIA5	NP_006801.1	804	2	2.36
CLCF1	NP_037378.1	222	0	0.00	SEC22A	NP_036562.2	678	3	4.20
RAD9A	NP_004575.1	591	1	1.61	ADCY5	NP_899200.1	1707	4	2.23
PPP1CA	NP_996756.1	3116	10	3.05	HACD2	NP_940684.1	462	5	10.28
TBC1D10C	NP_001356427.1	574	1	1.65	MYLK	XP_024309302.1	1303	6	4.37
CARNS1	NP_001159694.1	684	3	4.17	CCDC14	XP_011511383.1	166	1	5.72
RPS6KB2	NP_003943.2	1089	4	3.49	ROPN1	NP_060048.2	314	1	3.03
PTPRCAP	NP_005599.1	350	5	13.57	KALRN	XP_006713873.1	876	2	2.17
CORO1B	NP_065174.1	836	6	6.82	UMPS	NP_000364.1	1614	2	1.18
GPR152	NP_996880.1	113	3	25.22	ITGB5	XP_016861843.1	812	4	4.68
CABP4	XP_011543483.1	1134	2	1.68	MUC13	NP_149038.3	452	3	6.30
TMEM134	XP_006718756.1	152	0	0.00	HEG1	NP_065784.1	273	3	10.44
AIP	XP_024304529.1	238	2	7.98	SLC12A8	NP_078904.4	329	5	14.44
PITPNM1	XP_011543698.1	544	5	8.73	ZNF148	NP_001335363.1	618	2	3.07
CDK2AP2	NP_005842.1	244	4	15.57	SNX4	NP_003785.1	778	1	1.22
CABP2	NP_001305425.1	1039	0	0.00	OSBPL11	NP_073613.2	404	1	2.35
GSTP1	NP_000843.1	1130	5	4.20	ALG1L	NP_001371995.1	400	0	0.00
NDUFV1	NP_009034.2	930	6	6.13	ROPN1B	XP_006713576.1	150	0	0.00
NUDT8	XP_016872963.1	453	4	8.39	SLC41A3	XP_024309378.1	156	1	6.09
TBX10	NP_005986.2	401	2	4.74	ALDH1L1	XP_016861102.1	643	0	0.00
ACY3	NP_542389.1	290	2	6.55	KLF15	XP_011511045.1	657	2	2.89
ALDH3B2	NP_001026786.2	720	5	6.60	CFAP100	NP_872434.2	181	3	15.75
UNC93B1	NP_112192.2	638	1	1.49	ZXDC	XP_011511421.1	233	1	4.08
ALDH3B1	NP_001154945.1	853	7	7.80	UROC1	NP_653240.1	307	0	0.00
NDUFS8	NP_002487.1	1023	9	8.36	CHST13	NP_690849.1	195	1	4.87
TCIRG1	XP_024304088.1	826	3	3.45	C3orf22	XP_016861242.1	0	0	#DIV/0!

CHKA	NP_997634.1	790	3	3.61	TXNRD3	NP_001166984.1	94	0	0.00
KMT5B	XP_005274093.2	526	2	3.61	CHCHD6	NP_001307539.1	290	1	3.28
C11orf24	NP_001287842.1	92	3	30.98	PLXNA1	XP_011511210.1	643	4	5.91
LRP5	XP_011543331.1	1000	6	5.70	C3orf56	NP_001007535.1	34	1	27.94
PPP6R3	NP_001157636.1	509	4	7.47	TPRA1	NP_001339936.1	222	0	0.00
GAL	NP_057057.2	683	3	4.17	MCM2	NP_004517.2	1660	1	0.57
TESMIN	XP_016874077.1	347	3	8.21	PODXL2	NP_056535.1	188	3	15.16
CPT1A	XP_005273820.1	791	6	7.21	ABTB1	NP_742024.1	1034	1	0.92
MRPL21	NP_852615.1	917	8	8.29	MGLL	NP_001243514.1	972	3	2.93
IGHMBP2	NP_002171.2	861	7	7.72	KBTBD12	NP_997218.2	168	1	5.65
MRGPRD	NP_944605.2	232	7	28.66	SEC61A1	NP_037468.1	1475	6	3.86
MRGPRF	XP_016872659.1	298	11	35.07	RUVBL1	XP_016862845.1	2018	4	1.88
TPCN2	NP_620714.2	460	16	33.04	EEFSEC	XP_024309463.1	910	4	4.18
MYEOV	NP_620123.2	147	15	96.93	DNAJB8	NP_699161.1	439	3	6.49
CCND1	NP_444284.1	3321	14	4.00	GATA2	NP_001139134.1	1965	2	0.97
LTO1	NP_703152.1	273	17	59.15	RPN1	NP_002941.1	809	5	5.87
FGF19	NP_005108.1	1082	12	10.54	RAB7A	NP_004628.4	1752	5	2.71
FGF4	NP_001998.1	1140	11	9.17	ACAD9	NP_054768.2	632	2	3.01
FGF3	NP_005238.1	1130	11	9.25	CFAP92	XP_016862434.1	39	1	24.36
ANO1	NP_001365021.1	539	14	24.67	EFCC1	NP_001364429.1	70	0	0.00
FADD	NP_003815.1	999	11	10.46	GP9	NP_000165.1	743	5	6.39
PPFIA1	XP_011543611.1	623	16	24.40	RAB43	NP_001191812.1	641	4	5.93
CTTN	NP_001171669.1	1204	11	8.68	ISY1-RAB43	NP_001191819.1	544	1	1.75
SHANK2	XP_016872878.1	2099	15	6.79	ISY1	NP_065752.1	544	1	1.75
DHCR7	NP_001351.2	846	6	6.74	CNBP	NP_001120668.1	1218	2	1.56
NADSYN1	NP_060631.2	573	8	13.26	COPG1	NP_057212.1	1012	6	5.63
KRTAP5-7	NP_001012521.1	183	8	41.53	HMCES	NP_001357273.1	175	0	0.00
KRTAP5-8	NP_066384.2	241	7	27.59	H1-10	NP_006017.1	563	0	0.00
KRTAP5-9	NP_005544.4	270	0	0.00	EFCAB12	NP_997190.1	54	0	0.00
KRTAP5-10	NP_001012728.1	215	9	39.77	MBD4	NP_001263202.1	550	0	0.00
KRTAP5-11	NP_001005405.1	170	0	0.00	IFT122	NP_443711.2	918	1	1.03
ZNF705E	NP_001265642.1	115	0	0.00	RHO	NP_000530.1	1807	2	1.05
DEFB108B	NP_001002035.1	114	11	91.66	H1-8	NP_722575.1	646	0	0.00
DEFB131B	NP_001229782.1	3	0	0.00	PLXND1	XP_011510891.1	638	1	1.49
RNF121	NP_060790.2	174	12	65.51	TMCC1	NP_001336200.1	228	1	4.17
IL18BP	NP_766632.2	361	7	18.42	TRH	NP_009048.1	756	1	1.26
NUMA1	XP_016873319.1	788	6	7.23	ALG1L2	NP_001129624.1	442	1	2.15
LRTOMT	NP_001192067.1	383	10	24.80	COL6A5	NP_001265227.1	284	0	0.00
LAMTOR1	NP_060377.1	409	0	0.00	COL6A6	XP_011510725.1	409	0	0.00
ANAPC15	NP_001317250.1	298	4	12.75	PIK3R4	NP_055417.1	852	1	1.11
FOLR3	NP_000795.2	463	7	14.36	ATP2C1	NP_001365440.1	1054	1	0.90
FOLR1	NP_000793.1	627	4	6.06	ASTE1	NP_054784.2	142	4	26.76
FOLR2	XP_005273913.1	584	5	8.13	NEK11	NP_001308150.1	261	3	10.92
INPPL1	XP_024304269.1	1099	11	9.51	NUDT16	NP_689608.2	420	2	4.52
PHOX2A	NP_005160.2	440	6	12.95	MRPL3	NP_009139.1	1403	3	2.03
CLPB	NP_001245322.1	1772	4	2.14	CPNE4	NP_001276041.1	503	1	1.89
PDE2A	NP_002590.1	722	1	1.32	ACP3	NP_001127666.1	487	1	1.95
ARAP1	NP_001035207.1	688	6	8.28	DNAJC13	NP_001316055.1	585	2	3.25
STARD10	NP_006636.2	227	4	16.74	ACAD11	NP_115545.3	366	1	2.60
ATG16L2	XP_005274433.1	315	1	3.02	ACKR4	NP_848540.1	415	0	0.00
FCHSD2	NP_055639.2	450	8	16.89	UBA5	NP_079094.1	860	3	3.31
P2RY2	XP_016873328.1	568	4	6.69	NPHP3	NP_694972.3	867	3	3.29
P2RY6	XP_011543378.1	469	4	8.10	TMEM108	NP_001129941.1	184	5	25.81
ARHGEF17	XP_016874112.1	396	4	9.60	BFSP2	NP_003562.1	252	4	15.08
RELT	NP_116260.2	237	4	16.03	CDV3	XP_005247648.1	274	2	6.93
FAM168A	XP_016872904.1	146	3	19.52	TOPBP1	NP_001350818.1	1137	9	7.52
PLEKHB1	NP_067023.1	531	2	3.58	TF	NP_001054.2	738	2	2.57
RAB6A	NP_001230647.1	1053	3	2.71	SRPRB	NP_001366242.1	623	8	12.20
MRPL48	NP_001305428.1	516	2	3.68	RAB6B	NP_057661.3	1269	9	6.74
COA4	XP_016873372.1	105	0	0.00	SLCO2A1	NP_005621.2	443	1	2.14
PAAF1	NP_079431.1	667	6	8.55	RYK	NP_002949.2	853	6	6.68
DNAJB13	NP_705842.2	671	8	11.33	AMOTL2	NP_001265612.1	507	1	1.87
UCP2	NP_001368879.1	998	4	3.81	ANAPC13	NP_056206.1	485	1	1.96
UCP3	NP_003347.1	712	4	5.34	CEP63	XP_005247852.1	363	1	2.62
C2CD3	XP_016872999.1	335	2	5.67	KY	XP_006713675.1	362	3	7.87
PPME1	NP_001258522.1	542	11	19.28	EPHB1	XP_016861355.1	1593	7	4.17

P4HA3	NP_001275677.1	357	0	0.00	PPP2R3A	NP_002709.2	617	5	7.70
PGM2L1	NP_775853.2	776	1	1.22	MSL2	NP_060603.2	339	1	2.80
KCNE3	XP_011543015.1	287	9	29.79	PCCB	NP_001171485.1	703	3	4.05
LIPT2	NP_001138341.1	245	1	3.88	STAG1	NP_005853.2	761	1	1.25
POLD3	XP_005273773.1	542	10	17.53	SLC35G2	XP_016862778.1	154	3	18.51
CHRDL2	NP_056239.3	227	8	33.48	NCK1	XP_024309306.1	1054	3	2.70
RNF169	XP_011543191.1	202	1	4.70	IL20RB	NP_653318.2	281	2	6.76
XRRA1	XP_011543063.1	483	7	13.77	SOX14	NP_004180.1	502	5	9.46
SPCS2	NP_055567.2	392	7	16.96	CLDN18	NP_001002026.1	260	2	7.31
NEU3	NP_001354794.1	272	6	20.95	DZIP1L	XP_005247255.1	194	3	14.69
OR2AT4	NP_001005285.1	123	1	7.72	A4GNT	XP_016862032.1	188	2	10.11
SLCO2B1	NP_009187.1	424	3	6.72	DBR1	NP_057300.2	344	1	2.76
TPBGL	NP_001182457.1	403	0	0.00	ARMC8	NP_001350870.1	367	2	5.18
ARRB1	NP_064647.1	1579	1	0.60	NME9	NP_001335950.1	844	1	1.13
RPS3	NP_001247435.1	1944	7	3.42	MRAS	XP_016861376.1	1524	3	1.87
KLHL35	NP_001034637.2	224	0	0.00	ESYT3	NP_001309763.1	338	2	5.62
GDPD5	NP_110419.5	422	3	6.75	CEP70	NP_001275896.1	420	0	0.00
SERPINH1	XP_024304524.1	894	2	2.13	FAIM	XP_011511252.1	225	1	4.22
MAP6	NP_149052.1	748	5	6.35	PIK3CB	XP_016862108.1	1800	2	1.06
MOGAT2	NP_079374.2	657	4	5.78	FOXL2	NP_075555.1	933	3	3.05
DGAT2	NP_115953.2	663	6	8.60	FOXL2NB	NP_001035150.1	0	0	#DIV/0!
UVRAG	XP_011543528.1	640	2	2.97	PRR23A	NP_001128131.1	33	1	28.79
WNT11	NP_004617.2	1134	0	0.00	PRR23B	NP_001013672.1	40	0	0.00
THAP12	NP_004696.2	263	0	0.00	PRR23C	NP_001128129.1	60	0	0.00
GVQW3	NP_001334813.1	0	0	#DIV/0!	MRPS22	NP_064576.1	764	4	4.97
EMSY	XP_011543475.1	422	4	9.00	COPB2	NP_004757.1	988	3	2.88
LRRC32	NP_001357120.1	713	2	2.66	RBP2	NP_004155.2	330	1	2.88
TSKU	NP_001305406.1	705	7	9.43	RBP1	NP_002890.2	413	1	2.30
ACER3	NP_060837.3	374	8	20.32	NMNAT3	NP_001186976.1	320	4	11.87
B3GNT6	NP_619651.3	140	0	0.00	CLSTN2	NP_071414.2	569	3	5.01
CAPN5	XP_011543527.1	652	2	2.91	TRIM42	NP_689829.3	307	1	3.09
OMP	NP_006180.1	299	1	3.18	SLC25A36	NP_001098117.1	501	8	15.17
MYO7A	NP_000251.3	1418	5	3.35	SPSB4	NP_543138.1	555	1	1.71
GDPD4	XP_011543136.1	355	5	13.38	PXYLP1	NP_689495.1	282	3	10.11
PAK1	NP_001363217.1	1220	4	3.11	ZBTB38	XP_005247313.1	496	4	7.66
AQP11	NP_766627.1	256	9	33.40	RASA2	NP_001290174.1	411	2	4.62
CLNS1A	NP_001298131.1	902	10	10.53	RNF7	NP_055060.1	989	3	2.88
RSF1	XP_016873412.1	486	13	25.41	GRK7	NP_631948.1	530	0	0.00
AAMDC	XP_016873109.1	232	12	49.14	ATP1B3	NP_001670.1	466	3	6.12
INTS4	XP_016874049.1	326	13	37.88	TFDP2	XP_016862589.1	518	5	9.17
KCTD14	NP_076419.2	178	13	69.38	GK5	NP_001034636.1	822	1	1.16
NDUFC2-KCTD	NP_001190190.1	244	3	11.68	XRN1	XP_016862129.1	1409	1	0.67
THRSP	NP_003242.1	501	13	24.65	ATR	NP_001341508.1	2155	2	0.88
NDUFC2	NP_001190983.1	618	11	16.91	PLS1	XP_016862115.1	1044	2	1.82
ALG8	NP_076984.2	282	13	43.79	TRPC1	NP_001238774.1	583	3	4.89
KCTD21	XP_006718579.1	212	9	40.33	PCOLCE2	NP_037495.1	242	4	15.70
USP35	NP_065849.1	526	12	21.67	PAQR9	NP_001362229.1	234	0	0.00
GAB2	NP_536739.1	480	7	13.85	U2SURP	NP_001073884.1	676	1	1.41
NARS2	XP_011543555.1	638	9	13.40	CHST2	NP_004258.2	301	4	12.62
TENM4	XP_016873014.1	390	0	0.00	SLC9A9	NP_775924.1	702	7	9.47
FAM181B	NP_787081.2	196	6	29.08	DIPK2A	NP_775823.1	186	4	20.43
PRCP	NP_005031.1	468	5	10.15	PLOD2	NP_000926.2	649	6	8.78
DDIAS	XP_024304168.1	296	4	12.84	PLSCR4	XP_016862410.1	452	6	12.61
RAB30	NP_055303.2	350	5	13.57	PLSCR2	XP_016862392.1	427	3	6.67
PCF11	NP_001333342.1	894	1	1.06	PLSCR1	XP_016862118.1	620	2	3.06
ANKRD42	NP_001287902.1	1050	5	4.52	PLSCR5	NP_001308174.1	294	2	6.46
CCDC90B	NP_001273045.1	205	2	9.27	ZIC4	NP_001161851.1	1008	2	1.88
DLG2	NP_001338204.1	1641	1	0.58	ZIC1	NP_003403.2	1325	2	1.43
TMEM126B	NP_060950.3	408	3	6.98	AGTR1	NP_114038.4	1261	2	1.51
TMEM126A	NP_115649.1	495	2	3.84	CPB1	NP_001862.2	663	2	2.87
CREBZF	XP_016873575.1	224	0	0.00	CPA3	NP_001861.2	520	1	1.83
CCDC89	NP_689936.1	124	0	0.00	GYG1	NP_001171650.1	482	1	1.97
SYTL2	XP_016873423.1	295	3	9.66	HLTF	XP_016862568.1	1449	4	2.62
CCDC83	NP_775827.2	76	1	12.50	HPS3	NP_115759.2	804	0	0.00
PICALM	XP_016873877.1	927	4	4.10	CP	XP_006713562.1	975	1	0.97
EED	XP_011543632.1	1555	1	0.61	TM4SF18	NP_620141.1	134	0	0.00

HIKESHI	NP_057485.2	467	4	8.14	TM4SF1	XP_016861874.1	430	1	2.21
CCDC81	NP_001149946.1	114	0	0.00	TM4SF4	NP_004608.1	386	3	7.38
ME3	NP_001338863.1	547	1	1.74	WWTR1	XP_016861611.1	1038	5	4.58
PRSS23	NP_009104.3	462	3	6.17	COMMD2	NP_057178.2	236	3	12.08
FZD4	NP_036325.2	837	4	4.54	ANKUB1	NP_001138432.1	46	3	61.95
TMEM135	NP_075069.3	164	3	17.38	RNF13	XP_024309100.1	307	3	9.28
RAB38	XP_016872944.1	454	5	10.46	PFN2	NP_002619.1	1065	1	0.89
CTSC	NP_001805.4	1171	1	0.81	TMEM183B	NP_001073277.1	132	1	7.20
GRM5	NP_001371197.1	1559	3	1.83	TSC22D2	NP_055594.1	336	0	0.00
TYR	NP_000363.1	1559	5	3.05	SERP1	NP_055260.1	454	1	2.09
NOX4	NP_001137308.2	749	2	2.54	EIF2A	NP_114414.2	780	1	1.22
FOLH1B	XP_016872808.1	705	1	1.35	SELENOT	NP_057359.2	316	1	3.01
TRIM77	NP_001139634.1	216	0	0.00	ERICH6	NP_689607.2	30	1	31.67
TRIM49	XP_024304385.1	226	1	4.20	SIAH2	NP_005058.3	897	1	1.06
TRIM64B	NP_001157869.1	160	0	0.00	CLRN1	NP_443721.1	221	2	8.60
TRIM49D1	NP_001371840.1	179	0	0.00	MED12L	XP_016861165.1	437	4	8.70
TRIM49D2	NP_001098992.1	193	0	0.00	GPR171	XP_016861763.1	196	5	24.23
TRIM64	NP_001129958.1	202	0	0.00	P2RY14	XP_016863072.1	512	5	9.28
TRIM49C	XP_024304424.1	188	0	0.00	GPR87	NP_076404.3	340	4	11.18
UBTFL1	NP_001137447.1	445	0	0.00	P2RY13	XP_006713727.1	655	5	7.25
NAALAD2	XP_016872532.1	313	1	3.03	P2RY12	NP_073625.1	681	6	8.37
CHORDC1	NP_001137545.1	493	0	0.00	IGSF10	XP_011511010.1	674	4	5.64
FAT3	XP_016872667.1	833	3	3.42	AADACL2	NP_997248.2	197	2	9.64
MTNR1B	NP_005950.1	653	2	2.91	AADAC	NP_001077.2	437	4	8.70
SLC36A4	NP_689526.2	341	2	5.57	SUCNR1	NP_149039.2	508	10	18.70
DEUP1	XP_011540931.1	200	0	0.00	MBNL1	NP_001363747.1	1060	7	6.27
SMCO4	XP_011541211.1	77	0	0.00	P2RY1	NP_002554.1	549	11	19.03
CEP295	XP_011541349.1	187	0	0.00	RAP2B	NP_002877.2	1768	4	2.15
TAF1D	NP_077021.1	321	0	0.00	ARHGEF26	NP_056410.3	489	6	11.66
C11orf54	NP_001338917.1	95	0	0.00	DHX36	NP_001107869.1	1203	4	3.16
MED17	NP_004259.3	686	1	1.38	GPR149	NP_001033794.1	1472	6	3.87
VSTM5	NP_001138343.1	42	0	0.00	MME	NP_009218.2	1037	1	0.92
HEPHL1	NP_001092142.1	413	0	0.00	PLCH1	XP_016861416.1	662	2	2.87
PANX1	NP_056183.2	444	1	2.14	C3orf33	XP_011511012.1	7	0	0.00
IZUMO1R	NP_001186135.1	289	0	0.00	SLC33A1	NP_001350812.1	749	4	5.07
GPR83	NP_001317274.1	459	0	0.00	GMPS	XP_011511565.1	2112	6	2.70
MRE11	NP_005581.2	1061	1	0.90	KCNAB1	XP_016862660.1	612	5	7.76
ANKRD49	XP_016873430.1	979	1	0.97	SSR3	NP_009038.1	486	4	7.82
FUT4	NP_002024.1	773	1	1.23	TIPARP	NP_001171646.1	430	3	6.63
PIWIL4	NP_689644.2	852	1	1.11	LEKR1	XP_006713694.1	815	5	5.83
AMOTL1	XP_005273855.1	313	1	3.03	CCNL1	NP_001295114.1	563	2	3.37
CWC15	NP_057487.2	814	5	5.84	VEPH1	NP_001161383.1	154	2	12.34
KDM4D	NP_060509.2	538	1	1.77	PTX3	NP_002843.2	509	0	0.00
KDM4E	NP_001155102.1	414	1	2.29	SHOX2	NP_001157150.1	742	2	2.56
SRSF8	NP_115285.1	279	1	3.40	RSRC1	NP_057709.2	1095	5	4.34
ENDOD1	NP_055851.1	339	0	0.00	MLF1	NP_001365780.1	454	1	2.09
SESN3	NP_653266.2	293	0	0.00	GFM1	NP_001295093.1	1840	1	0.52
FAM76B	NP_001317286.1	340	2	5.59	LXN	NP_064554.3	308	1	3.08
CEP57	NP_055494.2	410	2	4.63	RARRES1	XP_005247743.3	298	3	9.56
MTMR2	NP_057240.3	480	2	3.96	MFSD1	XP_006713793.1	268	1	3.54
MAML2	NP_115803.1	324	1	2.93	IQCJ-SCHIP1	NP_001184042.1	261	7	25.48
CCDC82	NP_001350523.1	135	0	0.00	IQCJ	NP_001036170.1	66	0	0.00
JRKL	NP_003763.2	216	2	8.80	SCHIP1	NP_055390.1	154	6	37.01
CNTN5	XP_016873415.1	766	5	6.20	IL12A	NP_000873.2	261	1	3.64
ARHGAP42	NP_689645.2	409	6	13.94	C3orf80	NP_001161686.1	0	0	#DIV/0!
PGR	NP_000917.3	1801	7	3.69	IFT80	NP_065851.1	785	7	8.47
TRPC6	XP_016873710.1	607	7	10.95	SMC4	NP_001002800.1	1716	8	4.43
ANGPTL5	XP_016872955.1	340	4	11.18	TRIM59	NP_775107.1	490	3	5.82
CEP126	NP_065853.3	149	6	38.25	KPNA4	NP_002259.1	1059	9	8.07
CFAP300	XP_005271770.1	129	6	44.18	ARL14	NP_079323.1	1333	6	4.28
YAP1	XP_005271435.1	1811	6	3.15	PPM1L	NP_640338.2	1086	6	5.25
BIRC3	XP_024304235.1	1213	7	5.48	B3GALNT1	NP_001336091.1	301	3	9.47
BIRC2	NP_001243092.1	1223	5	3.88	NMD3	NP_001307156.1	1141	3	2.50
TMEM123	NP_443164.2	123	6	46.34	SPTSSB	NP_001307608.1	313	3	9.10
MMP7	NP_002414.1	1003	10	9.47	OTOL1	NP_001073909.1	111	2	17.12
MMP20	NP_004762.2	435	2	4.37	SI	NP_001032.2	533	1	1.78

MMP27	XP_011541250.1	376	1	2.53	SLITRK3	NP_055741.2	656	1	1.45
MMP8	NP_002415.1	844	1	1.13	BCHE	NP_000046.1	996	4	3.82
MMP10	NP_002416.1	603	6	9.45	ZBBX	XP_024309521.1	97	3	29.38
MMP1	NP_002412.1	1122	8	6.77	SERPINI2	NP_001012303.2	166	5	28.61
MMP3	NP_002413.1	1411	9	6.06	WDR49	NP_001335880.1	413	2	4.60
MMP12	NP_002417.2	897	7	7.41	PDCD10	XP_016861133.1	491	5	9.67
MMP13	NP_002418.1	947	6	6.02	SERPINI1	XP_016862107.1	661	4	5.75
DCUN1D5	NP_115675.1	483	1	1.97	GOLIM4	NP_001295084.1	330	3	8.64
DYNC2H1	NP_001073932.1	883	1	1.08	MECOM	NP_001353402.1	854	4	4.45
PDGFD	NP_149126.1	419	2	4.53	ACTRT3	NP_115876.3	980	3	2.91
DDI1	NP_001001711.1	382	0	0.00	MYNN	NP_001172047.1	394	10	24.11
CASP4	NP_001216.1	785	5	6.05	LRRC34	XP_006713571.1	167	2	11.38
CASP5	NP_001129582.1	782	6	7.29	LRRIQ4	NP_001073929.1	418	2	4.55
CASP1	XP_016873884.1	1791	9	4.77	LRRC31	NP_001264057.1	123	1	7.72
CARD16	XP_011540885.1	679	3	4.20	SAMD7	NP_872416.1	82	0	0.00
CARD17	XP_024304291.1	385	3	7.40	SEC62	NP_003253.1	649	5	7.32
CARD18	NP_067546.1	334	3	8.53	GPR160	XP_016861650.1	216	8	35.18
GRIA4	XP_005271575.1	1133	5	4.19	PHC3	XP_016862724.1	622	5	7.64
MSANTD4	XP_016873905.1	59	6	96.61	PRKCI	NP_002731.4	1429	7	4.65
KBTBD3	NP_689646.2	103	6	55.34	SKIL	NP_005405.2	458	5	10.37
AASDHPPT	NP_056238.2	600	3	4.75	CLDN11	NP_005593.2	533	0	0.00
GUCY1A2	NP_000846.1	1255	6	4.54	SLC7A14	NP_066000.2	681	3	4.18
CWF19L2	NP_689647.2	452	6	12.61	RPL22L1	NP_001307380.1	928	2	2.05
ALKBH8	XP_016874042.1	1023	6	5.57	EIF5A2	NP_065123.1	987	5	4.81
ELMOD1	NP_061182.3	658	9	12.99	SLC2A2	NP_000331.1	1086	3	2.62
SLN	NP_003054.1	207	4	18.36	TNIK	NP_001155038.1	687	2	2.77
SLC35F2	NP_059985.2	284	10	33.45	PLD1	NP_001123553.1	1516	4	2.51
RAB39A	NP_059986.1	1209	2	1.57	TMEM212	NP_001157908.1	185	2	10.27
CUL5	XP_016873852.1	1395	9	6.13	FNDC3B	XP_024309484.1	532	1	1.79
ACAT1	NP_000010.1	893	9	9.57	GHSR	NP_940799.1	506	4	7.51
NPAT	NP_002510.2	462	8	16.45	TNFSF10	NP_003801.1	1175	3	2.43
ATM	XP_016873278.1	2749	8	2.76	NCEH1	NP_065843.4	473	2	4.02
C11orf65	NP_001317297.1	53	9	161.31	ECT2	XP_006713587.1	860	7	7.73
POGLUT3	NP_001350431.1	187	10	50.80	SPATA16	NP_114161.3	183	0	0.00
EXPH5	NP_001294948.1	209	10	45.45	NLGN1	NP_001352853.1	930	3	3.06
DDX10	NP_004389.2	1608	6	3.54	NAALADL2	NP_996898.2	309	3	9.22
C11orf87	XP_011541119.1	79	1	12.02	TBL1XR1	NP_001308122.1	1497	7	4.44
ZC3H12C	XP_005271772.1	271	0	0.00	KCNMB2	NP_852006.1	41	0	0.00
RDX	NP_001247425.1	1134	1	0.84	ZMAT3	NP_001362757.1	232	2	8.19
FDX1	NP_004100.1	590	1	1.61	PIK3CA	NP_006209.2	2797	10	3.40
ARHGAP20	NP_001371586.1	398	5	11.93	KCNMB3	NP_001157149.1	207	3	13.77
C11orf53	NP_940900.2	73	5	65.07	ZNF639	XP_016862039.1	332	7	20.03
COLCA2	XP_016872685.1	81	6	70.37	MFN1	NP_284941.2	1145	8	6.64
POU2AF1	NP_006226.2	468	3	6.09	GNB4	NP_067642.1	1394	9	6.13
BTG4	XP_011541178.1	330	2	5.76	ACTL6A	NP_004292.1	2325	8	3.27
HOATZ	NP_997313.2	19	0	0.00	MRPL47	NP_065142.2	752	4	5.05
LAYN	NP_001245319.1	305	2	6.23	NDUFB5	NP_002483.1	628	7	10.59
SIK2	NP_056006.1	309	9	27.67	USP13	NP_003931.2	590	1	1.61
PPP2R1B	XP_024304368.1	1053	6	5.41	PEX5L	NP_001243682.1	837	1	1.13
ALG9	NP_001339346.1	484	9	17.66	TTC14	NP_001275511.1	173	8	43.93
FDXACB1	NP_612387.1	644	0	0.00	CCDC39	NP_852091.1	343	3	8.31
C11orf1	NP_073598.1	45	2	42.22	FXR1	XP_005247870.2	1315	11	7.95
CRYAB	NP_001355174.1	1441	3	1.98	DNAJC19	NP_660304.1	579	16	26.25
HSPB2	NP_001532.1	1234	1	0.77	SOX2	NP_003097.1	2722	7	2.44
C11orf52	NP_542390.2	85	2	22.35	ATP11B	NP_055431.1	628	11	16.64
DIXDC1	XP_016873955.1	267	2	7.12	DCUN1D1	XP_011511214.1	454	11	23.02
DLAT	NP_001358960.1	1064	9	8.04	MCCC1	NP_064551.3	539	9	15.86
PIH1D2	XP_016872694.1	214	7	31.07	LAMP3	NP_055213.2	415	0	0.00
NKAPD1	NP_060665.3	110	5	43.18	MCF2L2	NP_055893.4	413	14	32.20
TIMM8B	NP_036591.3	651	4	5.84	B3GNT5	XP_016862798.1	304	14	43.75
SDHD	NP_002993.1	987	4	3.85	KLHL6	NP_569713.2	549	14	24.22
IL18	NP_001230140.1	1472	4	2.58	KLHL24	XP_016862142.1	770	10	12.34
TEX12	NP_112565.1	186	1	5.11	YEATS2	NP_001338299.1	1033	16	14.71
BCO2	NP_114144.5	322	3	8.85	MAP6D1	NP_079147.1	267	14	49.81
PTS	NP_000308.1	820	0	0.00	PARL	XP_016862291.1	632	13	19.54
PLET1	NP_001138496.1	177	0	0.00	ABCC5	XP_005247115.1	644	16	23.60

NCAM1	NP_001229536.1	2244	6	2.54	HTR3D	NP_001138615.1	352	16	43.18
TTC12	XP_016873437.1	833	6	6.84	HTR3C	NP_570126.2	349	2	5.44
ANKK1	XP_011541038.1	981	5	4.84	HTR3E	NP_001243542.1	363	14	36.64
DRD2	NP_057658.2	1743	7	3.82	EIF2B5	NP_003898.2	796	17	20.29
TMPRSS5	NP_001275681.1	231	3	12.34	DVL3	XP_005247229.1	1079	13	11.45
ZW10	XP_016874047.1	611	3	4.66	AP2M1	NP_001298127.1	1105	16	13.75
CLDN25	NP_001094859.1	35	0	0.00	ABCF3	NP_060828.2	1069	19	16.88
USP28	NP_001333188.1	1197	4	3.17	VWA5B2	XP_011511590.1	397	18	43.07
HTR3B	NP_006019.1	532	6	10.71	ALG3	NP_001006942.1	472	18	36.23
HTR3A	NP_998786.3	799	4	4.76	EEF1AKMT4	NP_115707.2	322	12	35.40
ZBTB16	XP_016873748.1	1511	3	1.89	EEF1AKMT4-E	NP_055508.3	322	12	35.40
NNMT	NP_001358974.1	637	2	2.98	CAMK2N2	NP_150284.1	546	13	22.62
C11orf71	NP_061894.2	150	1	6.33	ECE2	NP_001093590.1	322	11	32.45
RBM7	NP_001272974.1	380	0	0.00	PSMD2	NP_002799.3	1521	14	8.74
REXO2	NP_056338.2	412	2	4.61	EIF4G1	NP_001181875.2	1848	16	8.22
NXPE1	NP_001354882.1	88	1	10.79	FAM131A	NP_653236.3	76	13	162.49
NXPE4	XP_011541184.1	104	1	9.13	CLCN2	NP_001164559.1	492	12	23.17
NXPE2	XP_016872701.1	103	1	9.22	POLR2H	NP_001265627.1	1285	3	2.22
CADM1	NP_001091987.1	831	0	0.00	THPO	NP_001276932.1	612	4	6.21
BUD13	NP_001153208.1	648	9	13.19	CHRD	XP_016862877.1	938	4	4.05
ZPR1	NP_001304015.1	783	9	10.92	EPHB3	NP_004434.2	1448	1	0.66
APOA5	NP_001358833.1	691	9	12.37	MAGEF1	NP_071432.2	104	0	0.00
APOA4	NP_000473.2	1091	8	6.97	VPS8	NP_001336224.1	454	1	2.09
APOC3	NP_000031.1	808	8	9.41	C3orf70	NP_001020437.1	77	3	37.01
APOA1	NP_000030.1	1654	9	5.17	EHHADH	NP_001957.2	872	2	2.18
SIK3	XP_016872913.1	275	12	41.45	MAP3K13	NP_001229246.1	424	5	11.20
PAFAH1B2	XP_016873329.1	373	9	22.92	TMEM41A	XP_016862926.1	108	3	26.39
SIDT2	NP_001035545.1	233	9	36.69	LIPH	XP_016861341.1	261	5	18.20
PCSK7	XP_024304532.1	761	9	11.23	SENP2	NP_067640.2	324	3	8.80
TAGLN	NP_003177.2	1031	4	3.69	IGF2BP2	XP_016861046.1	586	3	4.86
RNF214	NP_997226.2	107	2	17.76	TRA2B	NP_004584.1	1733	4	2.19
BACE1	NP_620429.1	1977	5	2.40	ETV5	NP_004445.1	654	3	4.36
CEP164	XP_006718851.1	495	1	1.92	DGKG	NP_001074214.1	512	8	14.84
DSCAML1	NP_065744.3	809	4	4.70	CRYGS	NP_060011.1	205	1	4.63
FXYD6-FXYD2	NP_001191197.1	321	8	23.67	TBCCD1	NP_001273678.1	93	1	10.21
FXYD2	NP_067614.1	0	0	#DIV/0!	DNAJB11	NP_057390.1	700	0	0.00
FXYD6	NP_071286.1	499	9	17.13	AHSG	NP_001341500.1	998	7	6.66
TMPRSS13	NP_001193718.1	271	9	31.55	FETUB	NP_001295008.1	493	5	9.63
IL10RA	NP_001549.2	1000	7	6.65	HRG	XP_005247472.1	510	4	7.45
TMPRSS4	XP_005271670.1	510	9	16.76	KNG1	NP_000884.1	1395	6	4.09
SCN4B	NP_001135820.1	822	11	12.71	EIF4A2	NP_001958.2	1881	3	1.52
SCN2B	NP_004579.1	783	8	9.71	RFC4	NP_853551.1	1762	2	1.08
JAML	XP_016872702.1	264	13	46.78	ADIPOQ	NP_001171271.1	1561	4	2.43
MPZL3	NP_001273081.1	207	11	50.48	ST6GAL1	NP_001340845.1	548	1	1.73
MPZL2	NP_658911.1	368	8	20.65	RPL39P19	NP_443201.1	906	2	2.10
CD3E	NP_000724.1	826	6	6.90	RTP1	NP_714919.2	580	0	0.00
CD3D	NP_001035741.1	951	8	7.99	MASP1	XP_016862359.1	440	4	8.64
CD3G	NP_000064.1	725	9	11.79	RTP4	NP_071430.2	749	1	1.27
UBE4A	NP_004779.2	1008	11	10.37	SST	NP_001039.1	1763	3	1.62
ATP5MG	NP_006467.4	671	4	5.66	RTP2	XP_016861790.1	542	1	1.75
KMT2A	XP_011541131.1	2263	5	2.10	BCL6	NP_001128210.1	1446	7	4.60
TTC36	NP_001073910.1	150	1	6.33	LPP	XP_011511125.1	1152	6	4.95
TMEM25	NP_001137509.1	139	3	20.50	TPRG1	XP_005247439.1	121	3	23.55
IFT46	XP_016873507.1	265	1	3.58	TP63	NP_001316893.1	1079	7	6.16
ARCN1	NP_001646.2	991	10	9.59	P3H2	NP_060662.2	331	8	22.96
PHLDB1	XP_011541005.1	195	12	58.46	CLDN1	NP_066924.1	681	5	6.97
TREH	NP_001287994.1	770	10	12.34	CLDN16	NP_006571.2	258	8	29.46
DDX6	XP_011540947.1	2028	9	4.22	TMEM207	NP_997199.1	111	6	51.35
CXCR5	NP_001707.1	933	4	4.07	IL1RAP	NP_001351808.1	283	0	0.00
BCL9L	XP_016873063.1	414	11	25.24	GMNC	NP_001140158.1	139	3	20.50
UPK2	NP_006751.1	548	7	12.13	OSTN	XP_016861792.1	227	5	20.92
FOXR1	NP_859072.1	531	13	23.26	UTS2B	NP_937795.2	278	5	17.09
CENATAC	NP_940891.1	110	16	138.17	CCDC50	NP_848018.1	265	8	28.68
RPS25	NP_001019.1	1168	8	6.51	PYDC2	NP_001076777.1	64	2	29.69
TRAPPC4	NP_057230.1	544	10	17.46	FGF12	NP_001364221.1	1041	4	3.65
SLC37A4	NP_001157749.1	441	14	30.16	MB21D2	NP_848591.2	197	2	9.64

HYOU1	XP_016872585.1	1452	7	4.58	PLAAT1	XP_011511337.1	260	2	7.31
VPS11	NP_001365147.1	788	9	10.85	ATP13A5	NP_940907.2	595	0	0.00
HMBS	NP_000181.2	1121	7	5.93	ATP13A4	NP_115655.2	692	0	0.00
H2AX	NP_002096.1	2173	4	1.75	OPA1	NP_570850.2	1128	2	1.68
DPAGT1	NP_001373.2	741	11	14.10	HES1	NP_005515.1	560	1	1.70
C2CD2L	NP_055622.3	156	13	79.16	CPN2	XP_005269337.1	514	4	7.39
HINFP	XP_011541046.1	435	7	15.29	LRRC15	NP_001128529.2	717	2	2.65
ABCG4	NP_001335120.1	1054	6	5.41	GP5	NP_004479.1	661	3	4.31
NLRX1	XP_006718967.1	371	3	7.68	ATP13A3	NP_001361765.1	845	9	10.12
PDZD3	XP_011541307.1	646	10	14.71	TMEM44	XP_016863007.1	61	3	46.72
CCDC153	NP_001138490.1	55	0	0.00	LSG1	NP_060855.2	936	4	4.06
CBL	NP_005179.2	1244	6	4.58	FAM43A	NP_710157.2	104	3	27.40
MCAM	XP_016873248.1	676	3	4.22	XXYLT1	NP_689744.3	145	2	13.10
RNF26	NP_114404.1	286	10	33.22	ACAP2	NP_036419.3	666	11	15.69
C1QTNF5	NP_056460.1	60	0	0.00	PPP1R2	NP_001278434.1	334	0	0.00
USP2	NP_001230688.1	789	8	9.63	APOD	NP_001638.1	861	6	6.62
THY1	NP_001358979.1	1509	7	4.41	MUC20	NP_001269435.1	392	7	16.96
NECTIN1	NP_976030.1	508	3	5.61	MUC4	NP_612154.2	592	6	9.63
TRIM29	NP_036233.2	440	3	6.48	TNK2	XP_024309060.1	700	9	12.21
OAF	NP_848602.1	128	0	0.00	TFRC	XP_024309499.1	2091	7	3.18
POU2F3	NP_001231611.1	452	1	2.10	ZDHHC19	XP_006713557.1	133	15	107.14
TLCD5	NP_001185599.1	112	3	25.45	SLC51A	NP_689885.4	430	16	35.35
ARHGEF12	NP_056128.1	930	2	2.04	PCYT1A	NP_005008.2	744	15	19.15
GRIK4	NP_055434.2	715	2	2.66	TCTEX1D2	NP_001338557.1	231	10	41.12
TBCEL	NP_001350573.1	178	5	26.68	TM4SF19	NP_001191826.1	163	12	69.93
TECTA	NP_005413.2	374	2	5.08	UBXN7	NP_056377.1	2255	13	5.48
SC5D	NP_008849.2	546	3	5.22	RNF168	NP_689830.2	606	4	6.27
SORL1	NP_003096.2	875	4	4.34	SMCO1	XP_024309203.1	33	15	431.80
BLID	NP_001001786.2	76	4	50.00	WDR53	NP_001332840.1	123	15	115.85
UBASH3B	NP_116262.2	549	2	3.46	FBXO45	NP_001099043.1	304	15	46.87
CRTAM	NP_062550.2	301	2	6.31	NRROS	NP_940967.1	757	13	16.31
JHY	NP_001350018.1	55	2	34.54	CEP19	NP_001366397.1	127	14	104.72
BSX	NP_001091639.1	379	4	10.03	PIGX	NP_001159776.1	47	0	0.00
HSPA8	NP_694881.1	3404	1	0.28	PAK2	XP_016861990.1	1292	1	0.74
CLMP	NP_079045.1	276	1	3.44	SENP5	NP_689912.2	460	9	18.59
GRAMD1B	NP_001354349.1	243	2	7.82	NCBP2	NP_001036005.1	1445	12	8.30
SCN3B	XP_011541199.1	701	3	4.07	PIGZ	XP_016862729.1	179	9	53.07
ZNF202	XP_016873757.1	367	1	2.59	MELTF	XP_006713706.2	493	7	15.87
OR6X1	NP_001005188.1	65	0	0.00	DLG1	NP_001353148.1	1334	3	2.67
OR6M1	NP_001005325.1	65	0	0.00	BDH1	XP_016862496.1	509	2	4.98
TMEM225	NP_001350534.1	51	0	0.00	RUBCN	NP_055502.1	363	2	6.98
OR8D4	NP_001005197.1	48	0	0.00	FYTTD1	NP_115664.2	397	1	3.42
OR4D5	NP_001001965.1	74	1	12.84	LRCH3	XP_016862839.1	543	2	5.38
OR6T1	NP_001005187.1	60	0	0.00	IQCG	NP_001309956.1	218	1	7.26
OR10S1	NP_001004474.1	87	0	0.00	RPL35A	NP_000987.2	1096	1	1.58
OR10G6	NP_001342148.1	47	0	0.00	LMLN	NP_001129521.2	252	2	15.08
OR10G4	NP_001004462.1	50	0	0.00	ZNF595	NP_872330.1	217	5	43.78
OR10G9	NP_001001953.1	114	0	0.00	ZNF718	NP_001034216.2	186	6	55.72
OR10G8	NP_001004464.1	45	0	0.00	ZNF732	NP_001131080.1	156	0	0.00
OR10G7	NP_001004463.1	72	0	0.00	ZNF141	NP_001335207.1	305	7	33.54
VWA5A	XP_011541130.1	301	2	6.31	ABCA11P	NP_597731.2	191	7	49.74
OR10D3	NP_001342142.1	28	0	0.00	PIGG	NP_001120650.1	343	10	36.93
OR8G1	NP_001002905.1	47	1	20.21	PDE6B	XP_011511775.1	976	12	14.60
OR8G5	NP_001005198.2	90	1	10.56	ATP5ME	NP_009031.1	668	4	6.69
OR8D1	NP_001002917.1	53	0	0.00	MYL5	XP_006713949.1	1094	7	6.75
OR8D2	NP_001002918.1	46	0	0.00	SLC49A3	XP_024310018.1	327	15	45.87
OR8B2	XP_016873024.1	29	0	0.00	PCGF3	NP_006306.2	370	13	33.38
OR8B3	XP_016873205.1	45	0	0.00	CPLX1	NP_006642.1	1186	17	13.62
OR8B4	NP_001005196.1	26	0	0.00	GAK	XP_016863481.1	2893	18	5.91
OR8B8	NP_036510.1	58	0	0.00	TMEM175	XP_016864190.1	104	7	63.94
OR8B12	NP_001005195.1	24	0	0.00	DGKQ	NP_001338.2	616	14	21.59
OR8A1	NP_001005194.2	40	1	23.75	SLC26A1	NP_602297.1	382	10	24.87
PANX3	NP_443191.1	231	2	8.22	IDUA	NP_000194.2	344	11	30.38
TBRG1	NP_116200.2	1625	1	0.58	FGFRL1	XP_024309860.1	534	11	19.57
SIAE	NP_733746.1	276	3	10.33	RNF212	NP_001353847.1	270	11	38.70
SPA17	XP_024304351.1	365	3	7.81	SPON2	NP_036577.2	434	8	17.51

NRGN	NP_001119653.1	771	4	4.93	CTBP1	NP_001319.1	1377	16	11.04
VSIG2	NP_055127.2	186	4	20.43	MAEA	NP_001017405.1	684	8	11.11
ESAM	NP_620411.2	388	2	4.90	UVSSA	XP_016863985.1	230	6	24.78
MSANTD2	NP_078907.2	18	0	0.00	NKX1-1	NP_001277008.1	195	7	34.10
ROBO3	NP_071765.2	652	2	2.91	FAM53A	XP_011511706.1	105	6	54.28
ROBO4	XP_006718924.1	628	3	4.54	SLBP	NP_001293004.1	1050	11	9.95
HEPN1	NP_001032647.2	517	3	5.51	TMEM129	NP_001120738.1	189	11	55.29
HEPACAM	XP_005271506.1	352	4	10.79	TACC3	XP_005247986.1	773	10	12.29
CCDC15	NP_079280.2	133	1	7.14	FGFR3	NP_001341739.1	1527	8	4.98
SLC37A2	NP_001138762.1	360	1	2.64	LETM1	XP_006713947.1	462	9	18.51
TMEM218	NP_001245168.1	106	0	0.00	NSD2	NP_001035889.1	2148	11	4.86
PKNOX2	NP_001369252.1	401	1	2.37	NELFA	XP_016864078.1	683	7	9.74
FEZ1	NP_005094.1	680	2	2.79	C4orf48	XP_011511771.1	13	0	0.00
EI24	NP_004870.3	248	3	11.49	NAT8L	NP_848652.2	733	3	3.89
STT3A	NP_689926.1	669	2	2.84	POLN	NP_861524.2	476	1	2.00
CHEK1	NP_001317356.1	2091	1	0.45	HAUS3	NP_078787.2	337	3	8.46
ACRV1	NP_064493.1	269	6	21.19	MXD4	NP_006445.1	298	3	9.56
PATE1	NP_612151.1	105	1	9.05	ZFYVE28	XP_006713963.1	287	5	16.55
PATE2	NP_997720.1	94	2	20.21	CFAP99	XP_024309814.1	21	0	0.00
PATE3	NP_001123355.3	39	2	48.72	RNF4	NP_001171938.1	843	3	3.38
PATE4	NP_001138346.1	94	2	20.21	FAM193A	NP_001353245.1	78	12	146.15
PUS3	NP_112597.4	637	2	2.98	TNIP2	NP_001278945.1	307	4	12.38
HYLS1	XP_016872809.1	142	1	6.69	SH3BP2	NP_001139327.1	454	6	12.55
DDX25	NP_037396.3	514	5	9.24	ADD1	XP_016863192.1	607	6	9.39
CDON	NP_058648.4	378	1	2.51	MFSD10	NP_001111.3	375	5	12.67
RPUSD4	NP_001138299.1	426	2	4.46	NOP14	NP_001278908.1	774	10	12.27
FAM118B	NP_078832.1	81	0	0.00	GRK4	XP_011511749.1	610	8	12.46
SRPRA	XP_016873668.1	1004	2	1.89	HTT	NP_002102.4	1842	1	0.52
FOXRED1	NP_060017.1	288	0	0.00	MSANTD1	NP_001317549.1	177	1	5.37
TIRAP	NP_001034750.1	494	0	0.00	RGS12	XP_016864018.1	539	9	15.86
DCPS	NP_001337165.1	463	1	2.05	HGFAC	NP_001284368.1	750	4	5.07
ST3GAL4	NP_001335325.1	399	1	2.38	DOK7	NP_001288000.1	335	1	2.84
KIRREL3	NP_001288026.1	489	1	1.94	LRPAP1	NP_002328.1	700	4	5.43
ETS1	XP_016872806.1	1478	3	1.93	ADRA2C	NP_000674.2	839	2	2.26
FLI1	NP_002008.2	535	2	3.55	OTOP1	NP_819056.1	188	5	25.26
KCNJ1	NP_722449.3	495	0	0.00	TMEM128	XP_005248091.1	85	5	55.88
KCNJ5	NP_001341098.1	532	1	1.79	LYAR	NP_001139197.1	889	9	9.62
TP53AIP1	XP_016873606.1	232	1	4.09	ZBTB49	NP_001317554.1	269	9	31.78
ARHGAP32	NP_001136157.1	659	2	2.88	NSG1	NP_001274692.1	296	10	32.09
BARX2	NP_003649.2	313	5	15.17	STX18	NP_058626.1	536	11	19.50
TMEM45B	XP_016872677.1	170	1	5.59	MSX1	NP_002439.2	1125	10	8.44
NFRKB	XP_016873284.1	332	4	11.45	CYTL1	NP_061129.1	266	0	0.00
PRDM10	NP_001354820.1	1758	0	0.00	STK32B	NP_060871.1	928	7	7.17
APLP2	NP_001369457.1	926	3	3.08	EVC2	NP_667338.3	317	8	23.97
ST14	NP_068813.1	414	1	2.29	EVC	NP_714928.1	311	8	24.44
ZBTB44	NP_001357152.1	346	0	0.00	CRMP1	NP_001014809.1	877	7	7.58
ADAMTS8	XP_016872634.1	345	3	8.26	C4orf50	XP_016864382.1	0	0	#DIV/0!
ADAMTS15	NP_620686.1	267	2	7.12	JAKMIP1	XP_016863286.1	400	0	0.00
SNX19	XP_011541126.1	205	8	37.07	WFS1	XP_016864075.1	746	1	1.27
NTM	NP_001338930.1	917	7	7.25	PPP2R2C	NP_001193923.1	1445	5	3.29
OPCML	XP_006718909.1	1366	9	6.26	MAN2B2	NP_056089.1	307	3	9.28
SPATA19	NP_777587.1	148	0	0.00	MRFAP1	NP_150638.1	187	1	5.08
IGSF9B	NP_001264214.1	489	1	2.16	S100P	NP_005971.1	276	0	0.00
JAM3	NP_116190.3	435	2	5.14	MRFAP1L1	NP_982287.1	49	0	0.00
NCAPD3	NP_001358994.1	633	5	9.38	BLOC1S4	NP_060836.1	97	0	0.00
VPS26B	NP_443107.1	431	5	14.69	KIAA0232	XP_016864349.1	113	0	0.00
THYN1	NP_954994.1	174	6	0.00	TBC1D14	XP_011511809.1	423	1	2.25
ACAD8	NP_055199.1	486	3	9.02	CCDC96	NP_699207.1	54	0	0.00
GLB1L3	NP_001073876.2	272	1	5.82	TADA2B	NP_689506.2	655	0	0.00
GLB1L2	NP_001357389.1	275	0	0.00	GRPEL1	NP_079472.1	1129	1	0.84
B3GAT1	XP_016873040.1	954	1	1.99	SORCS2	XP_005248044.2	628	6	9.08
IQSEC3	NP_001164209.1	963	10	19.73	PSAPL1	NP_001078851.1	132	4	28.79
SLC6A12	NP_003035.3	561	7	21.55	AFAP1	XP_006713972.1	325	4	11.69
SLC6A13	NP_001177926.1	635	5	12.47	ABLIM2	NP_001123558.1	491	6	11.61
KDM5A	NP_001036068.1	2192	6	4.00	SH3TC1	XP_016863789.1	167	4	22.75
CCDC77	NP_115734.1	188	9	64.97	HTRA3	NP_444272.1	380	1	2.50

B4GALNT3	NP_775864.3	233	6	32.62	ACOX3	NP_001362716.1	564	1	1.68
NINJ2	NP_057617.3	283	6	25.18	TRMT44	NP_689757.2	131	0	0.00
WNK1	NP_998820.3	544	7	14.38	GPR78	NP_543009.2	269	2	7.06
RAD52	XP_016875259.1	1022	4	4.13	CPZ	NP_001014447.2	368	5	12.91
ERC1	XP_016874541.1	863	14	16.22	HMX1	NP_001293071.1	524	2	3.63
WNT5B	XP_024304972.1	1013	4	3.75	FAM90A26	NP_001345347.1	1	0	0.00
FBXL14	XP_016874364.1	827	14	16.08	USP17L10	NP_001243781.1	44	6	129.54
ADIPOR2	NP_001362292.1	438	7	15.18	USP17L11	NP_001243783.1	77	6	74.02
CACNA2D4	NP_758952.4	471	7	14.12	USP17L12	NP_001243782.1	53	7	125.47
LRTM2	XP_016875337.1	1004	9	8.52	USP17L13	NP_001243784.1	48	6	118.74
DCP1B	NP_689853.3	539	5	8.81	USP17L15	NP_001243823.2	42	7	158.33
CACNA1C	XP_016875415.1	1430	8	5.31	USP17L17	NP_001243786.1	52	5	91.34
FKBP4	NP_002005.1	1582	5	3.00	USP17L18	NP_001243788.1	75	5	63.33
ITFG2	NP_060933.3	140	4	27.14	USP17L19	NP_001243789.1	40	5	118.74
NRIP2	XP_005253855.1	405	9	21.11	USP17L20	NP_001243790.1	40	5	118.74
FOXM1	NP_001230018.1	1856	6	3.07	USP17L21	NP_001243791.1	39	5	121.79
RHNO1	NP_001239428.1	274	2	6.93	USP17L22	NP_001243792.1	51	5	93.13
TULP3	NP_001153880.1	501	2	3.79	USP17L24	NP_001229256.1	176	1	5.40
TEAD4	NP_003204.2	834	2	2.28	USP17L25	NP_001229255.1	176	0	0.00
TSPAN9	NP_001161792.1	412	10	23.06	USP17L26	NP_001229257.1	173	0	0.00
PRMT8	NP_001243465.1	1288	2	1.48	USP17L5	NP_001229258.1	70	1	13.57
CRACR2A	NP_001138430.1	476	7	13.97	USP17L27	NP_001229259.1	173	0	0.00
PARP11	NP_065100.2	204	11	51.22	USP17L28	NP_001229260.1	176	0	0.00
CCND2	NP_001750.1	1474	9	5.80	USP17L29	NP_001229261.1	176	0	0.00
TIGAR	NP_065108.1	617	8	12.32	USP17L30	NP_001243796.1	176	0	0.00
FGF23	NP_065689.1	800	4	4.75	DEFB131A	NP_001035538.2	41	0	0.00
FGF6	NP_066276.2	656	7	10.14	SLC2A9	XP_011512159.1	533	4	7.13
C12orf4	NP_001333082.1	146	3	19.52	DRD5	NP_000789.1	1100	2	1.73
RAD51AP1	NP_001124334.1	635	5	7.48	WDR1	NP_005103.2	624	4	6.09
DYRK4	NP_001358230.1	759	5	6.26	ZNF518B	XP_016864275.1	267	3	10.67
AKAP3	XP_024304565.1	378	2	5.03	CLNK	XP_011512077.1	295	3	9.66
NDUFA9	NP_004993.1	875	2	2.17	HS3ST1	NP_005105.1	272	1	3.49
GALNT8	NP_059113.1	200	1	4.75	RAB28	NP_001017979.1	654	2	2.91
KCNA6	XP_016874759.1	570	0	0.00	NKX3-2	NP_001180.1	518	1	1.83
KCNA1	NP_000208.2	1754	4	2.17	BOD1L1	XP_016863499.1	301	2	6.31
KCNA5	NP_002225.2	881	2	2.16	CPEB2	XP_005248192.2	326	2	5.83
NTF3	NP_001096124.1	1073	5	4.43	C1QTNF7	NP_001128642.1	137	0	0.00
ANO2	XP_011519277.1	414	2	4.59	CC2D2A	NP_001365544.1	403	2	4.71
VWF	NP_000543.3	1968	6	2.90	FBXL5	XP_011512133.1	539	2	3.52
CD9	NP_001760.1	997	5	4.76	FAM200B	XP_016863537.1	31	0	0.00
PLEKHG6	NP_001371533.1	384	1	2.47	BST1	XP_011512180.1	350	1	2.71
TNFRSF1A	NP_001056.1	1723	8	4.41	CD38	NP_001766.2	998	2	1.90
SCNN1A	NP_001153047.1	666	3	4.28	FGFBP1	NP_005121.1	400	5	11.87
LTBR	NP_001257916.1	624	5	7.61	FGFBP2	NP_114156.1	183	1	5.19
CD27	XP_016875721.1	887	7	7.50	PROM1	NP_001358337.1	1229	3	2.32
TAPBPL	NP_060479.3	224	3	12.72	TAPT1	XP_011512114.1	311	7	21.38
VAMP1	NP_058439.1	995	5	4.77	LDB2	XP_016864302.1	867	6	6.57
MRPL51	NP_057581.2	450	1	2.11	QDPR	NP_001293069.1	629	7	10.57
NCAPD2	NP_055680.3	904	4	4.20	CLRN2	NP_001073296.1	17	0	0.00
GAPDH	NP_001276674.1	7642	11	1.37	LAP3	NP_056991.2	925	9	9.24
IFFO1	NP_001317253.1	111	0	0.00	MED28	NP_079481.2	444	8	17.12
NOP2	NP_006161.2	1117	5	4.25	FAM184B	NP_056503.1	168	13	73.51
CHD4	NP_001284482.1	1792	5	2.65	DCAF16	NP_001332814.1	177	5	26.83
LPAR5	NP_001136433.1	572	2	3.32	NCAPG	XP_016864032.1	1050	8	7.24
ACRBP	NP_115878.2	383	1	2.48	LCORL	XP_016863454.1	411	13	30.05
ING4	XP_011519266.1	608	3	4.69	SLIT2	XP_005248268.1	1243	11	8.41
ZNF384	NP_001372728.1	587	4	6.47	PACRGL	XP_011512088.1	175	7	38.00
PIANP	XP_011519228.1	504	3	5.65	KCNIP4	NP_671710.1	1400	11	7.46
COPS7A	NP_001157567.1	483	3	5.90	ADGRA3	NP_660333.2	752	8	10.11
MLF2	NP_001369154.1	310	5	15.32	PPARGC1A	NP_001317681.1	1578	9	5.42
PTMS	NP_002815.3	150	1	6.33	DHX15	NP_001349.2	1939	11	5.39
LAG3	NP_002277.4	495	2	3.84	SOD3	NP_003093.2	1344	5	3.53
CD4	NP_000607.1	1637	5	2.90	CCDC149	NP_001124198.1	37	4	102.70
GPR162	NP_055264.1	467	2	4.07	LGI2	XP_016863845.1	346	9	24.71
P3H3	NP_055077.2	256	5	18.55	SEPSECS	NP_058651.3	405	13	30.49
GNB3	NP_002066.1	1460	5	3.25	PI4K2B	NP_060793.2	463	13	26.67

CDCA3	NP_001284532.1	512	5	9.28	ZCCHC4	XP_011512137.1	120	9	71.25
USP5	NP_001092006.1	798	9	10.71	ANAPC4	NP_001273685.1	885	7	7.51
TPI1	NP_001152759.1	2354	4	1.61	SLC34A2	NP_006415.3	371	8	20.48
SPSB2	NP_116030.1	433	1	2.19	SEL1L3	NP_001284521.1	584	10	16.27
LRRC23	NP_008923.1	598	1	1.59	SMIM20	NP_001138904.1	102	0	0.00
ENO2	NP_001966.1	1988	3	1.43	RBPJ	XP_016863659.1	1426	7	4.66
ATN1	NP_001931.2	1015	1	0.94	CCKAR	NP_000721.1	517	11	20.21
C12orf57	NP_001288765.1	354	2	5.37	TBC1D19	NP_060787.2	153	12	74.51
PTPN6	NP_536858.1	1417	2	1.34	STIM2	NP_001162589.1	341	8	22.29
PHB2	NP_001254629.1	1269	4	2.99	PCDH7	XP_011512144.1	603	5	7.88
EMG1	NP_006322.4	1314	4	2.89	ARAP2	XP_016863191.1	537	3	5.31
LPCAT3	NP_005759.4	472	5	10.06	DTHD1	XP_011511995.1	148	2	12.84
C1S	NP_958850.1	801	4	4.74	NWD2	NP_001138462.1	877	0	0.00
C1R	NP_001724.4	759	5	6.26	C4orf19	NP_001098099.1	77	0	0.00
C1RL	NP_001284569.1	285	2	6.67	RELL1	NP_001078868.1	216	1	4.40
RBP5	XP_016875491.1	193	0	0.00	PGM2	NP_060760.2	872	1	1.09
CLSTN3	NP_055533.2	438	1	2.17	TBC1D1	XP_011511961.1	543	11	19.24
PEX5	XP_016875234.1	678	1	1.40	PTTG2	NP_006598.2	297	1	3.20
ACSM4	NP_001073923.1	353	0	0.00	KLF3	NP_057615.3	542	10	17.53
CD163L1	XP_011518918.1	131	0	0.00	TLR10	NP_001182035.1	1723	10	5.51
CD163	NP_001357075.1	940	5	5.05	TLR1	XP_024309967.1	2096	5	2.27
APOBEC1	NP_001635.2	385	6	14.80	TLR6	XP_024309641.1	1822	5	2.61
GDF3	NP_065685.1	754	7	8.82	FAM114A1	NP_001362721.1	187	15	76.20
DPPA3	NP_954980.1	262	5	18.13	TMEM156	XP_011512055.1	91	16	167.02
CLEC4C	NP_987099.1	464	7	14.33	KLHL5	XP_016863765.1	616	14	21.59
NANOGNB	NP_001138937.1	104	5	45.67	WDR19	XP_011512026.1	323	14	41.17
NANOG	NP_079141.2	2009	7	3.31	RFC1	NP_001350424.1	743	13	16.62
SLC2A14	NP_001273166.1	612	4	6.21	KLB	NP_783864.1	362	12	31.49
SLC2A3	NP_008862.1	793	10	11.98	RPL9	NP_000652.2	1451	15	9.82
FOXJ2	NP_060886.1	623	3	4.57	LIAS	NP_006850.2	627	12	18.18
C3AR1	NP_001313404.1	1086	8	7.00	UGDH	XP_005262724.1	840	12	13.57
NECAP1	NP_056324.2	380	4	10.00	SMIM14	NP_777581.1	133	11	78.57
CLEC4A	NP_057268.1	647	6	8.81	UBE2K	NP_005330.1	2040	12	5.59
ZNF705A	XP_024304748.1	115	0	0.00	PDS5A	XP_016863417.1	1087	11	9.61
FAM90A1	XP_016875036.1	108	1	8.80	N4BP2	XP_011512018.1	245	1	3.88
CLEC6A	NP_001007034.1	439	4	8.66	RHOH	XP_016863677.1	1859	3	1.53
CLEC4D	NP_525126.2	641	7	10.37	CHRNA9	NP_060051.2	553	2	3.44
CLEC4E	XP_011518916.1	559	6	10.20	RBM47	XP_016863799.1	398	3	7.16
AICDA	NP_001317272.1	902	4	4.21	NSUN7	NP_078953.4	633	6	9.00
MFAP5	NP_001284639.1	339	3	8.41	APBB2	NP_775098.2	406	0	0.00
RIMKLB	XP_024304860.1	288	4	13.19	UCHL1	NP_004172.2	1316	2	1.44
A2ML1	XP_011518868.1	333	1	2.85	LIMCH1	XP_011511944.1	869	4	4.37
PHC1	XP_016874444.1	494	0	0.00	PHOX2B	NP_003915.2	752	3	3.79
M6PR	NP_001193953.1	536	1	1.77	TMEM33	NP_060596.2	265	6	21.51
KLRG1	XP_016874171.1	557	3	5.12	DCAF4L1	NP_001025126.2	66	3	43.18
A2M	NP_001334352.2	1036	4	3.67	SLC30A9	NP_006336.3	448	8	16.96
PZP	XP_011519107.1	353	3	8.07	BEND4	XP_016863674.1	255	6	22.35
KLRB1	NP_002249.1	710	13	17.39	SHISA3	NP_001073974.1	119	0	0.00
CLEC2D	NP_037401.1	573	11	18.24	ATP8A1	XP_016863134.1	928	3	3.07
CLECL1	NP_001254630.1	396	10	23.99	GRXCR1	NP_001073945.1	241	2	7.88
CD69	NP_001772.1	1257	12	9.07	KCTD8	NP_938167.1	699	5	6.80
KLRF1	NP_057607.1	445	16	34.16	YIPF7	NP_872398.2	291	1	3.26
CLEC2B	NP_005118.2	677	13	18.24	GUF1	XP_024309946.1	1016	0	0.00
KLRF2	NP_001177694.1	153	8	49.67	GNPDA2	NP_001257809.1	542	5	8.76
CLEC2A	XP_011518959.1	211	10	45.02	GABRG1	NP_775807.2	1132	10	8.39
CLEC12A	NP_001193939.1	738	14	18.02	GABRA2	NP_000798.2	728	11	14.35
CLEC1B	XP_011518988.1	565	19	31.95	COX7B2	NP_570972.2	242	11	43.18
CLEC12B	XP_011518960.1	355	12	32.11	GABRA4	NP_001191196.1	840	11	12.44
CLEC9A	NP_997228.1	445	9	19.21	GABRB1	NP_000803.2	896	14	14.84
CLEC1A	NP_001284678.1	289	16	52.59	COMMD8	NP_001316597.1	556	14	23.92
CLEC7A	NP_922941.1	848	13	14.56	ATP10D	NP_065186.3	589	14	22.58
OLR1	NP_002534.1	743	14	17.90	CORIN	NP_001265514.1	557	2	3.41
TMEM52B	NP_001371823.1	114	3	25.00	NFXL1	NP_001265552.1	282	13	43.79
GABARAPL1	NP_113600.1	1215	6	4.69	CNGA1	XP_005248106.2	494	9	17.31
KLRD1	XP_011518953.1	767	13	16.10	NIPAL1	NP_997213.1	268	13	46.08
KLRK1	NP_031386.2	807	10	11.77	TXK	NP_003319.2	1174	12	9.71

KLRC4	NP_038459.1	223	9	38.34	TEC	NP_003206.2	874	1	1.09
KLRC3	NP_002252.2	182	4	20.88	SLAIN2	XP_005248178.1	188	11	55.58
KLRC2	NP_002251.2	267	6	21.35	SLC10A4	NP_689892.1	299	10	31.77
KLRC1	XP_024304741.1	427	8	17.80	ZAR1	NP_783318.1	292	7	22.77
EIF2S3B	NP_001344660.1	830	0	0.00	FRYL	XP_011511984.2	493	8	15.42
MAGOHB	NP_060518.1	969	3	2.94	OCIAD1	XP_024309873.1	213	3	13.38
STYK1	NP_060893.2	380	5	12.50	OCIAD2	NP_001014446.1	197	1	4.82
YBX3	XP_016875612.1	622	2	3.05	CWH43	XP_011512057.1	185	4	20.54
TAS2R7	NP_076408.1	311	1	3.05	DCUN1D4	NP_001035492.1	491	10	19.35
TAS2R8	NP_076407.1	265	0	0.00	LRRC66	NP_001019782.1	437	4	8.70
TAS2R9	NP_076406.1	247	1	3.85	SGCB	NP_000223.1	326	6	17.48
TAS2R10	NP_076410.1	306	2	6.21	SPATA18	NP_660306.1	262	8	29.01
PRR4	NP_001092008.2	111	2	17.12	USP46	NP_001273696.1	531	7	12.52
PRH1	NP_001278244.1	157	4	24.20	ERVMER34-1	NP_078810.1	139	1	6.83
TAS2R13	NP_076409.1	339	0	0.00	RASL11B	NP_076429.1	1510	9	5.66
PRH2	NP_001103683.1	156	5	30.45	SCFD2	NP_689753.2	261	10	36.40
TAS2R14	NP_076411.1	323	0	0.00	FIP1L1	NP_001363695.1	754	9	11.34
TAS2R50	NP_795371.2	293	0	0.00	LNX1	XP_016864265.1	1668	12	6.83
TAS2R20	NP_795370.2	304	0	0.00	LOC10192887	XP_016864373.1	318	1	2.99
TAS2R19	NP_795369.1	271	2	7.01	CHIC2	XP_006714100.1	259	14	51.35
TAS2R31	NP_795366.2	258	0	0.00	GSX2	NP_573574.2	423	10	22.46
TAS2R46	NP_795368.2	311	2	6.11	PDGFRA	NP_001334759.1	1595	9	5.36
TAS2R43	NP_795365.2	324	0	0.00	KIT	NP_001372213.1	2409	6	2.37
TAS2R30	NP_001091112.1	240	0	0.00	KDR	NP_002244.1	2284	7	2.91
TAS2R42	NP_852094.2	253	0	0.00	SRD5A3	NP_078868.1	308	13	40.10
PRB3	NP_006240.4	78	1	12.18	TMEM165	XP_011532696.1	356	14	37.36
PRB4	NP_001248328.1	106	0	0.00	CLOCK	XP_016864343.1	795	9	10.75
PRB2	NP_006239.3	87	1	10.92	PDCL2	NP_689614.2	324	13	38.12
ETV6	NP_001978.1	923	7	7.20	NMU	NP_001278974.1	581	4	6.54
BCL2L14	XP_024304956.1	445	7	14.94	EXOC1	XP_005265804.1	601	10	15.81
LRP6	NP_002327.2	825	7	8.06	CEP135	NP_079285.2	433	9	19.74
MANSC1	NP_001350542.1	140	11	74.64	CRACD	XP_024309926.1	190	8	40.00
BORCS5	NP_001287671.1	217	12	52.53	AASDH	XP_016863229.1	1429	13	8.64
DUSP16	NP_085143.1	885	12	12.88	PPAT	NP_002694.3	950	5	5.00
CREBL2	NP_001301.1	254	13	48.62	PAICS	NP_001072993.1	1712	13	7.21
GPR19	XP_016874701.1	163	3	17.48	SRP72	NP_008878.3	725	11	14.41
CDKN1B	NP_004055.1	1829	12	6.23	ARL9	NP_001350723.1	1307	14	10.18
APOLD1	NP_001123887.1	264	10	35.98	THEGL	XP_011532659.1	0	0	#DIV/0!
DDX47	NP_057439.2	1545	11	6.76	HOPX	XP_016864217.1	721	3	3.95
GPRC5A	NP_003970.1	383	7	17.36	SPINK2	NP_001258651.1	257	9	33.27
GPRC5D	XP_016875071.1	120	9	71.25	REST	NP_001350382.1	853	2	2.23
HEBP1	NP_057071.2	383	0	0.00	NOA1	NP_115689.1	734	8	10.35
FAM234B	NP_065904.1	118	10	80.50	POLR2B	NP_000929.1	2411	9	3.55
GSG1	NP_001193771.1	202	0	0.00	IGFBP7	NP_001544.1	843	5	5.63
EMP1	XP_024304645.1	547	4	6.95	ADGRL3	XP_016863418.1	893	4	4.26
GRIN2B	XP_016874708.1	1896	2	1.00	TECRL	XP_005265721.1	327	0	0.00
ATF7IP	NP_851997.1	440	0	0.00	EPHA5	XP_016863369.1	1747	2	1.09
PLBD1	NP_079105.4	273	2	6.96	CENPC	NP_001349410.1	613	1	1.55
GUCY2C	NP_004954.2	795	2	2.39	STAP1	NP_036240.1	362	0	0.00
H4-16	NP_778224.1	2047	1	0.46	UBA6	NP_060697.4	1166	0	0.00
H2AJ	NP_808760.1	1068	2	1.78	GNRHR	NP_000397.1	774	1	1.23
WBP11	NP_057396.1	694	6	8.21	TMPRSS11D	NP_004253.1	336	4	11.31
SMCO3	XP_016874801.1	8	0	0.00	TMPRSS11A	NP_001107859.1	264	3	10.79
C12orf60	XP_016874362.1	49	1	19.39	TMPRSS11F	NP_997290.2	211	3	13.51
ART4	NP_066549.2	173	0	0.00	TMPRSS11B	NP_872308.2	145	1	6.55
MGP	NP_000891.2	499	1	1.90	YTHDC1	NP_588611.2	482	1	1.97
ERP27	NP_689534.1	629	2	3.02	TMPRSS11E	NP_054777.2	295	2	6.44
ARHGDIB	NP_001308349.1	908	5	5.23	UGT2B17	NP_001068.1	467	1	2.03
PDE6H	XP_016874920.1	298	4	12.75	UGT2B15	NP_001067.2	348	3	8.19
RERG	NP_001177655.1	1434	7	4.64	UGT2B10	NP_001066.1	399	1	2.38
PTPRO	NP_109592.1	759	3	3.75	UGT2A3	NP_079019.3	438	2	4.34
EPS8	XP_024304646.1	728	7	9.13	UGT2B7	NP_001065.2	400	2	4.75
STRAP	NP_009109.3	721	0	0.00	UGT2B11	XP_011529852.1	258	1	3.68
DERA	NP_057038.2	536	3	5.32	UGT2B28	NP_444267.1	263	2	7.22
SLC15A5	NP_001164269.1	191	1	4.97	UGT2B4	NP_001284545.1	332	2	5.72
MGST1	XP_011518976.1	535	1	1.78	UGT2A1	NP_001288168.1	265	2	7.17

LMO3	NP_001230541.1	934	7	7.12	UGT2A2	NP_001288162.1	239	1	3.97
RERGL	NP_001273130.1	1274	10	7.46	SULT1B1	XP_005265733.1	384	9	22.26
PIK3C2G	XP_016874959.1	1501	3	1.90	SULT1E1	NP_005411.1	466	13	26.50
PLCZ1	XP_016875672.1	699	4	5.44	CSN1S1	NP_001881.1	280	5	16.96
CAPZA3	NP_201585.1	544	1	1.75	CSN2	NP_001289699.1	280	3	10.18
PLEKHA5	NP_001372855.1	701	7	9.49	STATH	NP_003145.1	173	9	49.42
AEBP2	NP_001107648.1	469	2	4.05	HTN3	NP_000191.1	526	2	3.61
PDE3A	NP_000912.3	479	4	7.93	HTN1	NP_002150.1	111	5	42.79
SLCO1C1	XP_005253451.1	745	6	7.65	PRR27	NP_999876.2	31	10	306.44
SLCO1B3	NP_062818.1	332	1	2.86	ODAM	NP_060325.3	164	9	52.13
SLCO1B7	NP_001009562.3	212	2	8.96	FDCSP	NP_694542.1	75	6	76.00
SLCO1B1	NP_006437.3	459	1	2.07	CSN3	XP_016863250.1	388	2	4.90
SLCO1A2	NP_066580.1	461	3	6.18	CABS1	NP_149113.3	249	3	11.45
IAPP	NP_001316130.1	498	1	1.91	SMR3A	XP_016863510.1	35	2	54.28
PYROXD1	NP_079130.2	305	1	3.11	SMR3B	NP_006676.1	72	3	39.58
RECQL	NP_116559.1	908	2	2.09	OPRPN	NP_067048.4	144	3	19.79
GOLT1B	XP_024304767.1	812	5	5.85	MUC7	NP_689504.2	320	6	17.81
SPX	NP_085049.1	350	7	19.00	AMTN	NP_997722.1	243	7	27.36
GYS2	NP_068776.2	1109	14	11.99	AMBN	NP_057603.1	333	7	19.97
LDHB	NP_001302466.1	1297	7	5.13	ENAM	NP_114095.2	430	6	13.26
KCNJ8	XP_016874772.1	407	6	14.00	JCHAIN	NP_653247.1	508	7	13.09
ABCC9	XP_005253347.1	678	11	15.41	UTP3	NP_065101.1	862	1	1.10
CMAS	NP_061156.1	385	3	7.40	RUFY3	XP_011530052.1	272	0	0.00
ST8SIA1	NP_003025.1	445	10	21.35	GRSF1	NP_002083.4	471	0	0.00
C2CD5	NP_001372259.1	275	5	17.27	MOB1B	XP_011530714.1	403	0	0.00
ETNK1	XP_016875069.1	768	13	16.08	DCK	NP_000779.1	634	4	5.99
SOX5	NP_001248344.1	1088	4	3.49	SLC4A4	XP_024310036.1	759	7	8.76
BCAT1	NP_001171563.1	995	11	10.50	GC	NP_001191236.1	619	7	10.74
C12orf77	XP_016874455.1	0	0	#DIV/0!	NPFFR2	NP_001138228.1	490	8	15.51
IRAG2	NP_001353477.1	466	12	24.46	ADAMTS3	NP_055058.2	527	7	12.62
CFAP94	NP_001338996.2	289	13	42.73	COX18	NP_001287658.1	960	4	3.96
ETFRF1	XP_016874339.1	179	12	63.68	ANKRD17	XP_005265728.1	1385	7	4.80
KRAS	NP_004976.2	3894	10	2.44	ALB	NP_000468.1	5426	15	2.63
LMNTD1	NP_001139199.1	218	6	26.15	AFP	NP_001125.1	1707	7	3.90
RASSF8	NP_009142.2	289	13	42.73	AFM	NP_001124.1	405	5	11.73
BHLHE41	NP_110389.1	566	9	15.11	RASSF6	XP_011529991.1	334	14	39.82
SSPN	NP_005077.2	374	12	30.48	CXCL8	NP_000575.1	2779	13	4.44
ITPR2	NP_002214.2	888	13	13.91	CXCL6	NP_002984.1	584	10	16.27
INTS13	XP_024304823.1	488	9	17.52	PF4V1	NP_002611.1	154	3	18.51
FGFR1OP2	NP_056448.1	328	13	37.65	CXCL1	NP_001502.1	1492	13	8.28
TM7SF3	XP_016874954.1	190	11	55.00	PF4	NP_002610.1	833	12	13.68
MED21	NP_004255.2	573	12	19.89	PPBP	NP_002695.1	848	15	16.80
C12orf71	NP_001073875.1	31	0	0.00	CXCL5	NP_002985.1	855	12	13.33
STK38L	XP_024304657.1	1784	14	7.45	CXCL3	NP_002081.2	674	10	14.09
ARNTL2	XP_011519068.1	344	4	11.05	CXCL2	NP_002080.1	1018	10	9.33
SMCO2	NP_001138482.1	39	3	73.07	MTHFD2L	XP_016863708.1	674	1	1.41
PPFIBP1	XP_016875545.1	322	11	32.45	EPGN	NP_001257918.1	220	8	34.54
REP15	NP_001025045.3	94	0	0.00	EREG	NP_001423.1	525	9	16.28
MRPS35	NP_068593.2	562	8	13.52	AREG	NP_001648.1	832	10	11.42
MANSC4	XP_006719087.1	41	0	0.00	BTC	NP_001303892.1	342	8	22.22
KLHL42	NP_065833.1	516	5	9.20	PARM1	XP_011530135.1	263	0	0.00
PTHLH	XP_016875164.1	795	3	3.58	RCHY1	NP_001265466.1	1151	1	0.83
CCDC91	XP_016875058.1	268	6	21.27	THAP6	XP_006714172.1	95	0	0.00
FAR2	XP_011519049.1	339	4	11.21	ODAPH	NP_001193910.1	71	0	0.00
ERGIC2	XP_024304777.1	710	4	5.35	CDKL2	XP_016864300.1	291	0	0.00
OVCH1	XP_024304736.1	178	2	10.67	G3BP2	XP_016864367.1	1033	4	3.68
TMTC1	XP_016875494.1	1091	4	3.48	USO1	NP_001276978.1	1124	3	2.54
IPO8	NP_006381.2	1085	0	0.00	PPEF2	NP_006230.2	1651	2	1.15
CAPRIN2	XP_016875343.1	237	1	4.01	NAAA	XP_006714243.1	331	2	5.74
TSPAN11	NP_001357230.1	240	1	3.96	SDAD1	NP_001275912.1	1232	8	6.17
DDX11	XP_016874407.1	871	5	5.45	CXCL9	NP_002407.1	1152	2	1.65
SINHCAF	XP_011519105.1	283	3	10.07	CXCL10	NP_001556.2	1642	4	2.31
DENND5B	NP_659410.3	212	0	0.00	CXCL11	NP_001289052.1	843	4	4.51
ETFBKMT	XP_024304691.1	188	0	0.00	ART3	NP_001364102.1	494	5	9.61
AMN1	NP_001106873.1	507	3	5.62	NUP54	NP_001265532.1	887	9	9.64
H3-5	NP_001013721.2	1343	3	2.12	SCARB2	NP_001191184.1	635	6	8.98

RESF1	XP_016875039.1	109	1	8.72	FAM47E	NP_001229865.1	67	5	70.89
BICD1	XP_011519114.1	442	3	6.45	FAM47E-STBI	NP_001229868.1	67	5	70.89
FGD4	NP_001371055.1	594	5	8.00	STBD1	NP_003934.1	236	3	12.08
DNM1L	NP_001265393.1	1837	6	3.10	CCDC158	XP_011530217.1	130	9	65.77
YARS2	NP_001035526.1	893	5	5.32	SHROOM3	NP_065910.3	438	2	4.34
PKP2	NP_001005242.2	769	6	7.41	SOWAHB	NP_001025041.1	108	1	8.80
SYT10	NP_945343.1	562	3	5.07	SEPTIN11	XP_016863893.1	529	5	8.98
ALG10	NP_116223.3	214	5	22.20	CCNI	NP_001335063.1	1277	2	1.49
ALG10B	NP_001295269.2	220	7	30.23	CCNG2	NP_004345.1	1474	3	1.93
CPNE8	NP_705898.1	316	6	18.04	CXCL13	NP_001358487.1	873	0	0.00
KIF21A	NP_001166936.1	1016	3	2.80	CNOT6L	XP_011530109.1	636	2	2.99
ABCD2	XP_016874481.1	468	3	6.09	MRPL1	NP_064621.3	1209	0	0.00
C12orf40	NP_001026918.2	1	0	0.00	FRAS1	NP_079350.5	1272	2	1.49
SLC2A13	XP_011536149.1	2822	8	2.69	ANXA3	NP_005130.1	679	2	2.80
LRRK2	NP_940980.4	4828	8	1.57	BMP2K	NP_942595.1	869	3	3.28
CNTN1	NP_001834.2	1286	2	1.48	PAQR3	XP_005262826.1	212	8	35.85
PDZRN4	NP_001158067.1	945	4	4.02	NAA11	XP_016864266.1	406	2	4.68
GXYLT1	NP_001093120.1	162	8	46.91	GK2	NP_149991.2	857	0	0.00
YAF2	NP_001177906.1	365	6	15.62	ANTXR2	NP_477520.2	441	7	15.08
ZCRB1	NP_149105.3	1097	10	8.66	PRDM8	XP_011530437.1	467	6	12.20
PPHLN1	XP_011536761.1	152	7	43.75	FGF5	NP_004455.2	789	5	6.02
PRICKLE1	XP_016874327.1	887	6	6.43	CFAP299	XP_016863461.1	188	6	30.32
ADAMTS20	NP_079279.3	297	7	22.39	BMP3	NP_001192.4	770	11	13.57
PUS7L	XP_011537093.1	449	8	16.93	PRKG2	NP_001269414.1	994	13	12.42
IRAK4	XP_016874879.1	1321	3	2.16	RASGEF1B	NP_001287665.1	614	12	18.57
TWF1	NP_002813.3	352	6	16.19	HNRNPD	NP_001003810.1	1782	6	3.20
TMEM117	NP_115632.1	50	1	19.00	HNRNPDL	NP_001193929.1	2041	5	2.33
NELL2	NP_001138582.1	907	4	4.19	ENOPH1	NP_067027.1	550	11	19.00
DBX2	NP_001004329.2	552	1	1.72	TMEM150C	NP_001340383.1	159	6	35.85
ANO6	NP_001136151.1	400	1	2.37	SCD5	NP_001032671.2	1130	8	6.73
ARID2	NP_689854.2	836	4	4.55	SEC31A	NP_001177978.1	837	4	4.54
SCAF11	XP_016875707.1	581	1	1.64	THAP9	NP_078948.3	187	6	30.48
SLC38A1	NP_109599.3	423	2	4.49	LIN54	NP_001108479.1	439	2	4.33
SLC38A2	NP_061849.2	777	2	2.45	COPS4	NP_057213.2	982	4	3.87
SLC38A4	NP_060488.2	483	2	3.93	PLAC8	NP_001124187.1	309	1	3.07
AMIGO2	NP_001357228.1	348	5	13.65	COQ2	NP_056512.5	721	11	14.49
PCED1B	XP_016875696.1	171	1	5.56	HPSE	NP_001186759.1	553	6	10.31
RPAP3	NP_001139547.1	1164	1	0.82	HELQ	NP_001284684.2	518	5	9.17
ENDOU	NP_001165911.1	198	0	0.00	MRPS18C	NP_057151.1	497	6	11.47
RAPGEF3	NP_001092001.2	750	0	0.00	ABRAXAS1	NP_620775.2	265	5	17.92
SLC48A1	XP_024304813.1	163	1	5.83	GPAT3	XP_016864269.1	512	7	12.99
HDAC7	NP_001295019.1	2180	7	3.05	NKX6-1	NP_006159.2	582	0	0.00
VDR	NP_001351014.1	1380	2	1.38	CDS1	NP_001254.2	564	2	3.37
TMEM106C	NP_076961.1	109	2	17.43	WDFY3	XP_011530067.1	1193	2	1.59
COL2A1	XP_016874319.1	1087	4	3.50	ARHGAP24	NP_001020787.2	496	6	11.49
SENP1	XP_011536547.1	673	2	2.82	MAPK10	NP_001304998.1	1963	5	2.42
PFKM	XP_024304788.1	1161	1	0.82	PTPN13	XP_011530467.1	997	5	4.76
ASB8	NP_077000.1	1168	2	1.63	SLC10A6	NP_932069.1	271	0	0.00
CCDC184	NP_001013657.3	367	0	0.00	C4orf36	XP_016863235.1	21	0	0.00
OR10AD1	NP_001004134.1	51	0	0.00	AFF1	NP_001160165.1	414	2	4.59
H1-7	NP_861453.1	185	0	0.00	KLHL8	NP_001278936.1	196	2	9.69
ZNF641	XP_005268697.1	175	1	5.43	HSD17B13	NP_001129702.1	219	1	4.34
ANP32D	NP_036536.2	350	1	2.71	HSD17B11	NP_057329.3	363	0	0.00
C12orf54	XP_016874285.1	19	0	0.00	NUDT9	NP_076952.1	391	2	4.86
OR8S1	NP_001005203.2	79	0	0.00	SPARCL1	NP_001121782.1	1042	6	5.47
LALBA	NP_001371279.1	220	2	8.64	DSPP	NP_055023.2	482	5	9.85
KANSL2	NP_060292.3	177	2	10.73	DMP1	XP_011530007.1	542	5	8.76
CCNT1	NP_001231.2	1247	2	1.52	IBSP	NP_004958.2	952	6	5.99
ADCY6	XP_016874232.1	1229	3	2.32	MEPE	XP_006714341.1	469	6	12.15
CACNB3	NP_001193846.1	371	1	2.56	SPP1	NP_001035147.1	1624	6	3.51
DDX23	NP_004809.2	1332	0	0.00	PKD2	NP_000288.1	645	0	0.00
RND1	NP_055285.1	1720	4	2.21	ABCG2	XP_016864341.1	1370	7	4.85
CCDC65	NP_149115.2	263	0	0.00	PPM1K	XP_016863292.1	1062	9	8.05
FKBP11	NP_057678.1	881	1	1.08	HERC6	XP_005263140.1	880	7	7.56
ARF3	XP_006719454.1	1849	7	3.60	HERC5	XP_011530324.2	962	9	8.89
WNT10B	NP_003385.2	864	5	5.50	PIGY	NP_001036081.1	147	9	58.16

WNT1	NP_005421.1	1824	5	2.60	HERC3	XP_005263384.1	1116	11	9.36
DDN	NP_055901.2	659	3	4.32	NAP1L5	NP_715638.1	973	10	9.76
PRKAG1	XP_005269076.1	1359	6	4.19	FAM13A	XP_011529819.1	370	12	30.81
KMT2D	XP_006719679.1	2169	9	3.94	TIGD2	NP_001369309.1	177	12	64.40
RHEBL1	NP_653194.1	1362	5	3.49	GPRIN3	XP_016863532.1	270	12	42.22
DHH	NP_066382.1	801	6	7.12	SNCA	NP_009292.1	2928	10	3.24
LMBR1L	NP_001339091.1	198	8	38.38	MMRN1	NP_001358332.1	374	9	22.86
TUBA1B	NP_006073.2	1856	2	1.02	CCSER1	XP_016863683.1	232	8	32.76
TUBA1A	NP_006000.2	1963	5	2.42	GRID2	XP_024309792.1	1045	8	7.27
TUBA1C	NP_001290043.1	1981	3	1.44	ATOH1	NP_005163.1	963	3	2.96
PRPH	XP_005269082.1	141	3	20.21	SMARCAD1	XP_024309922.1	814	5	5.84
TROAP	XP_006719244.1	438	3	6.51	HPGDS	XP_005262989.1	2153	4	1.76
C1QL4	XP_011536572.1	99	2	19.19	PDLIM5	NP_001243356.2	981	5	4.84
DNAJC22	NP_079178.2	424	3	6.72	BMPR1B	NP_001243722.1	1477	5	3.22
SPATS2	NP_075559.2	179	0	0.00	UNC5C	NP_003719.3	620	8	12.26
KCNH3	XP_011536387.1	772	1	1.23	PDHA2	NP_005381.1	780	2	2.44
MCRS1	XP_011536062.1	409	0	0.00	STPG2	XP_024309780.1	201	1	4.73
FAM186B	XP_006719689.1	31	10	306.44	RAP1GDS1	NP_001093896.1	376	2	5.05
PRPF40B	NP_001350536.2	988	5	4.81	TSPAN5	XP_005262737.1	324	3	8.80
FMNL3	NP_944489.2	357	5	13.30	EIF4E	NP_001959.1	1827	4	2.08
TMBIM6	NP_001092046.1	582	3	4.90	METAP1	NP_055958.2	1003	13	12.31
NCKAP5L	NP_001354977.1	131	8	58.01	ADH5	NP_000662.3	748	2	2.54
BCDIN3D	NP_859059.1	225	6	25.33	ADH4	NP_000661.2	611	3	4.66
FAIM2	NP_036438.2	1451	10	6.55	ADH6	NP_001095940.1	565	5	8.41
AQP2	NP_000477.1	795	5	5.97	ADH1A	NP_000658.1	635	1	1.50
AQP5	NP_001642.1	647	5	7.34	ADH1B	NP_000659.2	573	2	3.32
AQP6	NP_001643.2	381	8	19.95	ADH1C	NP_000660.1	659	3	4.32
RACGAP1	XP_024304726.1	1091	6	5.22	ADH7	NP_000664.2	517	5	9.19
ASIC1	XP_011536652.1	610	7	10.90	C4orf17	NP_115525.2	56	3	50.89
SMARCD1	NP_003067.3	844	2	2.25	TRMT10A	NP_001362811.1	411	4	9.25
GPD1	NP_005267.2	1099	5	4.32	MTTP	NP_000244.2	568	10	16.72
COX14	NP_116290.1	275	4	13.82	C4orf54	XP_373030.5	3	0	0.00
CERS5	NP_671723.1	405	7	16.42	DAPP1	XP_011530142.1	582	9	14.69
LIMA1	NP_057441.1	698	4	5.44	LAMTOR3	NP_001230665.1	561	4	6.77
FAM186A	XP_006719294.1	90	1	10.56	DNAJB14	NP_001026893.1	584	8	13.01
LARP4	NP_001317344.1	908	8	8.37	H2AZ1	NP_002097.1	2287	10	4.15
DIP2B	NP_775873.2	425	7	15.65	DDIT4L	NP_660287.1	403	11	25.93
ATF1	XP_016874820.1	461	2	4.12	EMCN	NP_001153166.1	362	2	5.25
TMPRSS12	NP_872365.2	176	1	5.40	PPP3CA	NP_001124164.1	3373	11	3.10
METTL7A	NP_054752.3	685	1	1.39	BANK1	NP_060405.5	438	13	28.19
HIGD1C	XP_016875272.1	141	0	0.00	SLC39A8	NP_001128619.1	459	11	22.77
SLC11A2	NP_001366375.1	897	0	0.00	NFKB1	XP_024309837.1	2721	10	3.49
LETMD1	XP_011536465.1	168	0	0.00	MANBA	NP_005899.3	547	10	17.37
CSRNP2	NP_110436.1	144	0	0.00	UBE2D3	NP_871622.1	1951	7	3.41
TFCP2	NP_001166924.1	469	0	0.00	CISD2	NP_001008389.1	366	8	20.76
POU6F1	XP_016875005.1	488	2	3.89	SLC9B1	NP_001094344.2	338	2	5.62
DAZAP2	NP_001129741.1	250	0	0.00	SLC9B2	NP_001357133.1	416	10	22.84
SMAGP	NP_001026798.1	150	1	6.33	BDH2	XP_005263197.1	552	6	10.33
BIN2	NP_001276936.1	1015	0	0.00	CENPE	NP_001273663.1	1548	8	4.91
CELA1	NP_001962.3	439	0	0.00	TACR3	NP_001050.1	533	3	5.35
GALNT6	XP_016874234.1	262	2	7.25	CXXC4	XP_016864141.1	176	7	37.78
SLC4A8	XP_016875730.1	501	2	3.79	TET2	XP_024309870.1	765	2	2.48
SCN8A	NP_055006.1	1010	2	1.88	PPA2	NP_789843.2	1085	5	4.38
ANKRD33	NP_872414.3	88	0	0.00	ARHGEF38	NP_001229658.1	426	5	11.15
ACVRL1	XP_011537310.1	1325	3	2.15	INTS12	NP_065128.2	419	4	9.07
ACVR1B	NP_064733.3	1442	3	1.98	GSTCD	NP_001026890.2	549	6	10.38
TAMALIN	NP_859062.1	1040	3	2.74	NPNT	XP_011530122.1	540	5	8.80
NR4A1	XP_005268879.1	1074	4	3.54	TBCK	NP_149106.3	313	4	12.14
ATG101	XP_024304888.1	361	0	0.00	AIMP1	XP_016864324.1	1250	2	1.52
KRT80	NP_872313.2	424	0	0.00	DKK2	NP_055236.1	493	2	3.85
KRT7	XP_011536627.1	649	0	0.00	PAPSS1	NP_005434.4	583	3	4.89
KRT86	XP_005268923.1	230	0	0.00	SGMS2	NP_001362835.1	347	4	10.95
KRT81	NP_002272.2	377	0	0.00	CYP2U1	XP_005262774.1	446	7	14.91
KRT83	NP_002273.3	205	0	0.00	HADH	NP_001171634.3	847	2	2.24
KRT85	NP_002274.1	299	0	0.00	LEF1	NP_001124186.1	1965	2	0.97
KRT84	NP_149034.2	217	1	4.38	RPL34	NP_000986.2	1028	3	2.77

KRT82	NP_149022.3	238	0	0.00	OSTC	NP_067050.1	386	4	9.84
KRT75	NP_004684.2	290	0	0.00	ETNPPL	NP_001140062.1	678	2	2.80
KRT6B	NP_005546.2	384	0	0.00	COL25A1	XP_016864224.1	314	6	18.15
KRT6C	NP_775109.2	307	0	0.00	SEC24B	XP_005262745.1	801	4	4.74
KRT6A	NP_005545.1	483	0	0.00	MCUB	NP_060388.2	184	0	0.00
KRT5	NP_000415.2	995	0	0.00	CASP6	NP_116787.1	738	3	3.86
KRT71	NP_258259.1	387	0	0.00	PLA2G12A	NP_110448.2	316	10	30.06
KRT74	NP_778223.2	228	0	0.00	CFI	NP_001362211.1	755	7	8.81
KRT72	XP_016874324.1	344	0	0.00	GAR1	NP_127460.1	961	6	5.93
KRT73	XP_011536559.1	344	1	2.76	RRH	NP_006574.1	236	6	24.15
KRT2	NP_000414.2	473	1	2.01	LRIT3	NP_940908.3	879	3	3.24
KRT1	NP_006112.3	646	1	1.47	EGF	XP_016863334.1	4481	4	0.85
KRT77	NP_778253.2	352	1	2.70	ELOVL6	NP_076995.1	732	6	7.79
KRT76	NP_056932.2	295	1	3.22	ENPEP	NP_001968.3	780	4	4.87
KRT3	XP_024304743.1	330	1	2.88	PITX2	NP_700476.1	1149	6	4.96
KRT4	NP_002263.3	615	1	1.54	FAM241A	NP_689613.2	61	6	93.44
KRT79	NP_787028.1	315	1	3.02	AP1AR	NP_061039.3	158	4	24.05
KRT78	XP_011536312.1	341	1	2.79	TIFA	NP_443096.1	195	2	9.74
KRT8	NP_001243211.1	1046	1	0.91	ALPK1	NP_079420.3	244	8	31.15
KRT18	NP_954657.1	1053	17	15.34	NEUROG2	NP_076924.1	642	4	5.92
EIF4B	NP_001287750.1	1749	8	4.35	ZGRF1	XP_016863860.1	717	4	5.30
TNS2	XP_016874578.1	1102	11	9.48	LARP7	NP_001253968.1	831	2	2.29
SPRYD3	NP_116229.1	240	13	51.46	ANK2	NP_001373115.1	2387	5	1.99
IGFBP6	NP_002169.1	428	10	22.20	CAMK2D	NP_001308506.1	1044	2	1.82
SOAT2	NP_003569.1	608	12	18.75	ARSJ	NP_001341139.1	523	4	7.27
CSAD	XP_024304780.1	575	12	19.83	UGT8	NP_001309041.1	458	4	8.30
ZNF740	XP_006719407.1	312	14	42.63	NDST4	NP_072091.1	332	4	11.45
ITGB7	NP_000880.1	975	14	13.64	TRAM1L1	NP_689615.2	120	5	39.58
RARG	XP_024304880.1	669	14	19.88	NDST3	XP_016864328.1	230	4	16.52
MFSD5	NP_001164261.1	145	18	117.92	PRSS12	NP_003610.2	375	4	10.13
ESPL1	NP_036423.4	1360	14	9.78	METTL14	NP_066012.1	350	2	5.43
PFDN5	NP_002615.2	1229	17	13.14	SEC24D	XP_024310061.1	653	3	4.36
MYG1	NP_067653.4	552	16	27.53	SYNPO2	NP_001273683.1	860	3	3.31
AAAS	NP_001166937.1	401	3	7.11	MYOZ2	NP_057683.1	424	2	4.48
SP7	NP_690599.1	944	6	6.04	USP53	XP_016863802.1	248	2	7.66
SP1	NP_612482.2	1563	3	1.82	C4orf3	NP_001163801.1	169	0	0.00
AMHR2	XP_011536475.1	987	4	3.85	FABP2	NP_000125.2	539	0	0.00
PRR13	NP_060927.1	144	10	65.97	PDE5A	NP_001074.2	733	0	0.00
PCBP2	NP_005007.2	1412	8	5.38	MAD2L1	NP_002349.1	1849	3	1.54
MAP3K12	XP_016875445.1	439	8	17.31	PRDM5	XP_011529868.1	487	1	1.95
TARBP2	NP_599150.1	434	3	6.57	NDNF	NP_078850.3	183	0	0.00
NPFF	NP_003708.1	346	2	5.49	TNIP3	XP_011530558.1	318	0	0.00
ATF7	NP_001353490.1	570	3	5.00	QRFPR	NP_937822.2	320	0	0.00
ATP5MC2	XP_016874950.1	610	1	1.56	ANXA5	NP_001145.1	2251	5	2.11
CALCOCO1	NP_001137154.1	229	2	8.30	SMIM43	NP_001371262.1	286	0	0.00
HOXC13	NP_059106.2	515	11	20.29	EXOSC9	NP_001029366.1	868	2	2.19
HOXC12	NP_776272.1	346	8	21.96	CCNA2	NP_001228.2	2681	6	2.13
HOXC11	NP_055027.1	373	8	20.37	BBS7	XP_016863846.1	363	5	13.08
HOXC10	NP_059105.2	368	8	20.65	TRPC3	NP_001353408.1	600	2	3.17
HOXC9	NP_008828.1	440	8	17.27	KIAA1109	NP_001371054.1	518	4	7.34
HOXC8	NP_073149.1	543	10	17.49	ADAD1	XP_024309656.1	365	7	18.22
HOXC6	NP_004494.1	475	8	16.00	IL2	NP_000577.2	2713	6	2.10
HOXC5	NP_061826.1	342	8	22.22	IL21	NP_068575.1	575	5	8.26
HOXC4	NP_055435.2	488	10	19.47	BBS12	XP_016863320.1	200	3	14.25
SMUG1	XP_016874608.1	874	0	0.00	FGF2	NP_001997.5	2793	6	2.04
CBX5	NP_001120793.1	1204	5	3.94	NUDT6	NP_009014.2	297	3	9.60
HNRNPA1	NP_112420.1	2219	2	0.86	SPATA5	XP_016863314.1	1162	8	6.54
NFE2	NP_001129495.1	625	3	4.56	SPRY1	NP_001362339.1	650	3	4.38
COPZ1	NP_057141.1	734	1	1.29	ANKRD50	XP_016863960.1	1074	6	5.31
GPR84	XP_011536797.1	757	2	2.51	FAT4	NP_001278232.1	922	2	2.06
ZNF385A	XP_011536472.1	191	2	9.95	INTU	XP_011530148.1	360	7	18.47
ITGA5	NP_002196.4	983	0	0.00	SLC25A31	NP_112581.1	724	5	6.56
GTSF1	XP_016874289.1	400	1	2.37	HSPA4L	NP_001304310.1	1467	6	3.89
NCKAP1L	NP_005328.2	723	1	1.31	PLK4	NP_055079.3	1769	8	4.30
PDE1B	NP_000915.1	491	1	1.93	MFSD8	NP_001350450.1	244	5	19.47
PPP1R1A	XP_006719534.1	374	0	0.00	ABHD18	NP_001345383.1	50	1	19.00

LACRT	NP_150593.1	161	3	17.70	LARP1B	XP_011530359.2	443	6	12.87
DCD	NP_444513.1	166	2	11.45	PGRMC2	NP_006311.3	571	1	1.66
MUCL1	NP_477521.1	167	3	17.06	JADE1	XP_024309987.1	563	1	1.69
TESPA1	XP_016875751.1	195	3	14.61	SCLT1	XP_016863206.1	350	2	5.43
NEUROD4	NP_067014.2	220	1	4.32	C4orf33	XP_016863212.1	104	1	9.13
OR9K2	NP_001005243.2	73	0	0.00	PCDH10	XP_011530452.1	726	1	1.31
OR10A7	NP_001005280.1	150	0	0.00	PABPC4L	NP_001108206.3	628	2	3.03
OR6C74	XP_016874615.1	102	0	0.00	PCDH18	NP_001287757.1	485	0	0.00
OR6C6	NP_001005493.1	128	0	0.00	SLC7A11	NP_055146.1	697	0	0.00
OR6C1	NP_001005182.1	75	0	0.00	NOCT	NP_036250.2	584	5	8.13
OR6C3	NP_473445.1	153	0	0.00	ELF2	XP_005262862.1	288	3	9.90
OR6C75	NP_001005497.1	135	0	0.00	MGARP	NP_116012.2	268	6	21.27
OR6C65	NP_001005518.1	95	0	0.00	NDUFC1	NP_001171915.1	455	6	12.53
OR6C76	NP_001005183.1	73	0	0.00	NAA15	NP_476516.1	748	0	0.00
OR6C2	NP_473446.1	134	0	0.00	RAB33B	XP_011530601.1	733	5	6.48
OR6C70	NP_001005499.1	37	0	0.00	SETD7	NP_001293128.1	1977	2	0.96
OR6C68	NP_001005519.2	23	0	0.00	MGST2	XP_016863700.1	282	3	10.11
OR6C4	NP_001372904.1	71	0	0.00	MAML3	NP_061187.3	352	1	2.70
OR2AP1	NP_001245214.1	64	1	14.84	SCOC	NP_115936.2	233	2	8.15
OR10P1	NP_996782.1	74	0	0.00	CLGN	NP_001124147.1	710	2	2.68
METTL7B	NP_689850.2	616	0	0.00	MGAT4D	XP_011529960.1	90	2	21.11
ITGA7	NP_001138469.1	726	2	2.62	ELMOD2	XP_005262942.1	223	3	12.78
BLOC1S1	NP_001478.2	362	4	10.50	UCP1	XP_005263263.1	1006	5	4.72
RDH5	NP_001186700.1	491	2	3.87	TBC1D9	NP_055945.2	663	6	8.60
CD63	NP_001244321.1	1029	4	3.69	RNF150	NP_065775.1	432	4	8.80
GDF11	NP_005802.1	635	1	1.50	ZNF330	XP_016863522.1	176	2	10.79
SARNP	NP_149073.1	773	2	2.46	IL15	NP_000576.1	1197	2	1.59
ORMDL2	NP_054901.1	186	1	5.11	INPP4B	NP_001372269.1	394	14	33.75
DNAJC14	NP_115740.5	312	5	15.22	USP38	NP_115946.2	516	10	18.41
MMP19	XP_011536661.1	334	3	8.53	GAB1	NP_997006.1	473	10	20.08
PYM1	NP_115721.1	439	6	12.98	SMARCA5	NP_003592.3	2230	10	4.26
DGKA	XP_016874389.1	503	6	11.33	FREM3	NP_001161707.1	431	11	24.24
PMEL	NP_001186982.1	773	9	11.06	GYPE	XP_016863628.1	218	10	43.58
CDK2	XP_011536034.1	3338	6	1.71	GYPB	XP_011530205.1	200	9	42.75
RAB5B	NP_001238965.1	1108	9	7.72	GYPA	XP_016863625.1	387	9	22.09
SUOX	XP_016875394.1	802	8	9.48	HHIP	XP_006714351.1	681	10	13.95
IKZF4	XP_016875303.1	357	9	23.95	ANAPC10	XP_011529831.1	1287	13	9.60
RPS26	NP_001020.2	1062	9	8.05	ABCE1	NP_002931.2	1545	14	8.61
ERBB3	NP_001973.2	2078	8	3.66	OTUD4	NP_001352986.1	405	6	14.07
PA2G4	NP_006182.2	1705	4	2.23	SMAD1	NP_001341740.1	776	0	0.00
RPL41	NP_066927.1	183	2	10.38	MMAA	NP_001362573.1	198	4	19.19
ZC3H10	NP_116175.1	210	2	9.05	C4orf51	NP_001074000.1	5	5	949.95
ESYT1	NP_001171725.1	371	3	7.68	ZNF827	XP_016863257.1	294	5	16.16
MYL6B	NP_001186558.1	1206	2	1.58	LSM6	XP_016863161.1	1197	3	2.38
MYL6	NP_524147.2	1388	2	1.37	SLC10A7	NP_001304745.1	321	1	2.96
SMARCC2	XP_005269160.1	1458	3	1.95	POU4F2	NP_004566.2	648	2	2.93
RNF41	NP_001229755.1	838	5	5.67	TTC29	XP_006714402.1	99	1	9.60
NABP2	XP_005269204.1	296	3	9.63	EDNRA	NP_001948.1	871	4	4.36
SLC39A5	NP_775867.2	286	3	9.96	TMEM184C	NP_060711.2	105	3	27.14
ANKRD52	NP_775866.2	1202	7	5.53	PRMT9	NP_612373.2	453	3	6.29
COQ10A	NP_653177.3	316	0	0.00	ARHGAP10	NP_078881.3	346	1	2.75
CS	NP_004068.2	1821	1	0.52	NR3C2	NP_001341748.1	987	5	4.81
CNPY2	NP_055070.1	354	2	5.37	DCLK2	NP_001035351.4	876	3	3.25
PAN2	NP_001159751.2	620	1	1.53	LRBA	NP_001186211.2	515	4	7.38
IL23A	XP_011536779.1	458	1	2.07	MAB21L2	NP_006430.1	394	1	2.41
STAT2	NP_001372039.1	1055	2	1.80	RPS3A	NP_000997.1	1844	0	0.00
APOF	NP_001629.1	280	0	0.00	SH3D19	NP_001365051.1	611	3	4.66
TIMELESS	NP_001317224.1	1130	4	3.36	PRSS48	NP_001340540.1	133	0	0.00
MIP	XP_011536656.1	454	2	4.18	FAM160A1	NP_001335623.1	118	0	0.00
SPRYD4	NP_997227.1	258	2	7.36	GATB	NP_001350270.1	492	0	0.00
GLS2	NP_037399.2	893	5	5.32	FBXW7	NP_001336727.1	1702	4	2.23
RBMS2	XP_006719604.1	417	3	6.83	TMEM154	NP_689893.1	159	2	11.95
BAZ2A	NP_038477.2	1228	3	2.32	TIGD4	NP_663772.1	155	2	12.26
ATP5F1B	NP_001677.2	2738	2	0.69	ARFIP1	NP_001274361.1	480	4	7.92
PTGES3	NP_001269532.1	1350	4	2.81	FHDC1	NP_203751.2	859	1	1.11
NACA	NP_001106672.1	948	3	3.01	TRIM2	NP_001362417.1	759	1	1.25

PRIM1	NP_000937.1	1159	6	4.92	MND1	NP_115493.1	548	0	0.00
HSD17B6	NP_003716.2	789	3	3.61	TMEM131L	XP_024309724.1	122	4	31.15
SDR9C7	NP_683695.1	212	6	26.89	TLR2	XP_016864062.1	2694	1	0.35
RDH16	NP_003699.3	290	6	19.65	RNF175	XP_016863536.1	123	2	15.45
GPR182	NP_009195.1	265	4	14.34	SFRP2	NP_003004.1	962	4	3.95
ZBTB39	NP_055645.1	281	10	33.81	DCHS2	NP_001345164.1	456	1	2.08
TAC3	NP_001171525.1	437	7	15.22	PLRG1	NP_001188493.1	1051	2	1.81
MYO1A	NP_001242970.1	1122	6	5.08	FGB	NP_005132.2	925	3	3.08
NEMP1	XP_011536360.1	220	7	30.23	FGA	NP_000499.1	1142	3	2.50
NAB2	NP_005958.1	481	3	5.92	FGG	NP_000500.2	1048	3	2.72
STAT6	NP_003144.3	1407	3	2.03	LRAT	NP_004735.2	366	3	7.79
LRP1	XP_016874792.1	1011	1	0.94	RBM46	NP_659416.1	262	8	29.01
NXPH4	XP_016874236.1	262	2	7.25	NPY2R	NP_001357109.1	763	7	8.72
SHMT2	NP_005403.2	1506	4	2.52	MAP9	XP_016864105.1	272	6	20.95
NDUFA4L2	NP_064527.1	308	2	6.17	GUCY1A1	NP_001366595.1	1293	7	5.14
STAC3	NP_659501.1	368	0	0.00	GUCY1B1	NP_001278880.1	1127	9	7.59
R3HDM2	XP_016874518.1	429	3	6.64	ASIC5	XP_016863780.1	106	8	71.69
INHBC	NP_005529.1	457	7	14.55	TDO2	NP_005642.1	561	9	15.24
INHBE	NP_113667.1	512	4	7.42	CTSO	NP_001325.1	447	10	21.25
GLI1	NP_005260.1	1584	5	3.00	PDGFC	XP_016863945.1	425	1	2.24
ARHGAP9	XP_011536960.1	850	4	4.47	GLRB	NP_001159532.1	950	6	6.00
MARS1	NP_004981.2	1813	1	0.52	GRIA2	NP_001077088.2	1763	4	2.16
DDIT3	NP_001181982.1	1118	6	5.10	GASK1B	XP_024309846.1	195	0	0.00
MBD6	XP_005268674.1	137	3	20.80	TMEM144	XP_016863855.1	137	0	0.00
DCTN2	NP_001248342.1	859	2	2.21	RXFP1	NP_001240656.1	920	5	5.16
KIF5A	NP_004975.2	2196	12	5.19	C4orf46	NP_001008394.1	94	0	0.00
PIP4K2C	NP_079055.3	617	10	15.40	ETFDH	NP_004444.2	737	6	7.73
DTX3	XP_005268754.1	208	6	27.40	PPID	NP_005029.1	1302	3	2.19
ARHGEF25	NP_001104740.2	895	9	9.55	FNIP2	XP_005263215.1	283	1	3.36
B4GALNT1	NP_001263397.1	609	11	17.16	C4orf45	XP_011529973.1	46	2	41.30
OS9	XP_005268638.1	822	15	17.33	RAPGEF2	XP_005263415.1	484	1	1.96
AGAP2	NP_055585.1	757	17	21.33	FSTL5	NP_001121900.1	746	2	2.55
TSPAN31	NP_005972.1	307	14	43.32	NAF1	NP_001122403.1	395	0	0.00
CDK4	NP_000066.1	3074	13	4.02	NPY1R	NP_000900.1	773	4	4.92
MARCHF9	NP_612405.2	344	8	22.09	NPY5R	XP_016863744.1	641	4	5.93
CYP27B1	NP_000776.1	569	8	13.36	TKTL2	NP_115512.3	748	3	3.81
METTL1	NP_005362.3	1061	13	11.64	TMA16	NP_060822.2	149	3	19.13
EEF1AKMT3	NP_056248.2	177	12	64.40	MARCHF1	XP_016863824.1	524	3	5.44
TSFM	NP_001166168.1	841	9	10.17	APELA	XP_016863112.1	165	2	11.51
AVIL	XP_016874199.1	418	11	25.00	TRIM61	NP_001012414.1	256	5	18.55
CTDSP2	NP_005721.3	539	4	7.05	TRIM60	NP_689833.1	267	3	10.67
ATP23	NP_150592.1	391	10	24.30	TMEM192	XP_011530020.1	102	1	9.31
LRIG3	NP_700356.2	890	0	0.00	KLHL2	XP_011529874.1	783	6	7.28
SLC16A7	XP_011537291.1	353	1	2.69	MSMO1	XP_005263233.1	631	2	3.01
TAFA2	XP_024304730.1	180	2	10.56	CPE	NP_001864.1	1008	4	3.77
USP15	NP_001239007.1	887	4	4.28	TLL1	NP_036596.3	505	4	7.52
MON2	XP_016874530.1	1228	4	3.09	SPOCK3	XP_016863746.1	892	3	3.19
PPM1H	NP_065751.1	174	2	10.92	ANXA10	XP_011529873.1	439	4	8.66
AVPR1A	NP_000697.1	553	4	6.87	DDX60	XP_024309900.1	1604	0	0.00
DPY19L2	XP_016874681.1	297	7	22.39	DDX60L	XP_011530707.1	1432	2	1.33
RXYLT1	NP_055069.1	180	7	36.94	PALLD	XP_011530070.1	657	4	5.78
SRGAP1	NP_065813.1	527	6	10.82	CBR4	XP_005263372.1	541	1	1.76
C12orf66	NP_001287869.2	121	1	7.85	SH3RF1	NP_065921.2	645	3	4.42
C12orf56	NP_001093146.1	17	3	167.64	NEK1	NP_001361350.1	500	3	5.70
XPOT	XP_016874237.1	864	12	13.19	CLCN3	NP_776297.2	506	0	0.00
TBK1	XP_005268866.1	1841	8	4.13	HPF1	NP_060337.2	371	1	2.56
RASSF3	NP_835463.1	230	12	49.56	MFAP3L	XP_005263423.1	156	1	6.09
GNS	NP_002067.1	564	8	13.47	AADAT	XP_006714294.1	473	7	14.06
TBC1D30	XP_024304669.1	696	10	13.65	GALNTL6	NP_001030017.2	283	4	13.43
WIF1	NP_009122.2	907	4	4.19	GALNT7	NP_001362528.1	292	2	6.51
LEMD3	NP_055134.2	673	12	16.94	HMGB2	NP_002120.1	1217	2	1.56
MSRB3	XP_024304690.1	580	12	19.65	SAP30	NP_003855.1	452	4	8.41
HMGA2	NP_003474.1	1022	6	5.58	SCRG1	NP_001316526.1	343	5	13.85
LLPH	NP_115714.1	216	4	17.59	HAND2	NP_068808.1	860	2	2.21
TMBIM4	NP_057140.2	443	7	15.01	FBXO8	NP_036312.2	668	7	9.95
IRAK3	NP_009130.2	1324	9	6.46	CEP44	XP_024309997.1	113	2	16.81

HELB	NP_001357214.1	681	10	13.95	HPGD	NP_001243235.1	649	3	4.39
GRIP1	XP_005268814.1	984	1	0.97	GLRA3	NP_001036008.1	612	9	13.97
CAND1	NP_060918.2	883	6	6.45	ADAM29	NP_001124175.1	307	8	24.75
DYRK2	NP_006473.2	1075	10	8.84	GPM6A	NP_001248376.1	1032	10	9.20
IFNG	NP_000610.2	2769	10	3.43	WDR17	XP_024309653.1	900	11	11.61
IL26	NP_060872.1	312	6	18.27	SPATA4	NP_653245.2	303	10	31.35
IL22	NP_065386.1	475	2	4.00	ASB5	NP_543150.1	1240	9	6.89
MDM1	NP_001191957.1	275	9	31.09	SPCS3	NP_068747.1	551	8	13.79
RAP1B	NP_001010942.1	1911	11	5.47	VEGFC	NP_005420.1	985	2	1.93
NUP107	NP_065134.1	1034	13	11.94	NEIL3	NP_060718.3	644	5	7.38
SLC35E3	NP_061126.2	254	9	33.66	AGA	NP_001165459.1	501	1	1.90
MDM2	NP_002383.2	2279	14	5.84	TENM3	XP_016863874.1	527	8	14.42
CPM	NP_938079.1	334	6	17.06	DCTD	NP_001012750.1	377	3	7.56
CPSF6	XP_005268645.1	839	11	12.45	WWC2	XP_011530571.1	586	3	4.86
LYZ	NP_000230.1	1045	10	9.09	CLDN22	NP_001104789.1	97	0	0.00
YEATS4	NP_006521.1	1306	14	10.18	CLDN24	NP_001172078.1	41	0	0.00
FRS2	XP_016874206.1	598	11	17.47	CDKN2AIP	NP_060102.1	248	2	7.66
CCT2	NP_006422.1	2022	13	6.11	ING2	NP_001555.1	648	6	8.80
LRRC10	NP_963844.2	539	8	14.10	RWDD4	NP_001294851.1	458	1	2.07
BEST3	NP_001269543.1	340	14	39.12	TRAPPC11	XP_024309947.1	407	2	4.67
RAB3IP	XP_006719289.3	695	5	6.83	STOX2	XP_011530431.1	184	11	56.79
MYRFL	NP_872336.2	89	6	64.04	ENPP6	NP_699174.1	334	6	17.06
CNOT2	NP_001186231.1	849	7	7.83	IRF2	XP_024309807.1	681	6	8.37
KCNMB4	NP_055320.4	234	5	20.30	CASP3	NP_001341706.1	3959	6	1.44
PTPRB	NP_001317133.1	844	7	7.88	PRIMPOL	NP_001332820.1	257	5	18.48
PTPRR	NP_002840.2	1003	6	5.68	CENPU	NP_078905.2	576	7	11.54
TSPAN8	NP_001356689.1	671	1	1.42	ACSL1	NP_001368819.1	1073	8	7.08
LGR5	NP_003658.1	1302	2	1.46	HELT	NP_001287710.1	331	12	34.44
ZFC3H1	NP_659419.3	521	5	9.12	SLC25A4	NP_001142.2	1156	10	8.22
THAP2	NP_113623.1	114	1	8.33	CFAP97	XP_016863972.1	370	7	17.97
TMEM19	NP_060749.2	102	1	9.31	SNX25	XP_011530604.1	418	9	20.45
RAB21	NP_055814.1	712	2	2.67	LRP2BP	XP_016863900.1	425	7	15.65
TBC1D15	NP_073608.4	709	3	4.02	ANKRD37	NP_859077.1	1086	8	7.00
TPH2	NP_775489.2	575	1	1.65	UFSP2	NP_060829.2	254	2	7.48
TRHDE	NP_037513.2	641	2	2.96	C4orf47	XP_016863725.1	7	0	0.00
ATXN7L3B	NP_001129734.1	80	5	59.37	CCDC110	NP_001138883.1	116	4	32.76
KCNC2	XP_005268912.1	1203	4	3.16	PDLIM3	NP_001244892.1	883	8	8.61
CAPS2	NP_115995.2	426	0	0.00	SORBS2	XP_016864261.1	784	10	12.12
GLIPR1L1	XP_016874623.1	77	4	49.35	TLR3	NP_003256.1	2088	1	0.45
GLIPR1L2	NP_001257325.1	78	2	24.36	FAM149A	NP_001354697.1	114	16	133.33
GLIPR1	NP_006842.2	481	8	15.80	CYP4V2	NP_997235.3	553	12	20.61
KRR1	NP_008974.5	877	10	10.83	KLKB1	XP_016863670.1	666	6	9.01
PHLDA1	NP_031376.3	380	3	7.50	F11	XP_005262878.1	538	3	5.89
NAP1L1	XP_016874828.1	1148	6	4.96	MTNR1A	NP_005949.1	555	3	6.04
BBS10	NP_078961.3	1676	10	5.67	FAT1	XP_006714202.1	956	10	12.42
OSBPL8	XP_016874257.1	462	5	10.28	ZFP42	NP_001291287.1	534	7	16.60
ZDHHC17	NP_056151.2	835	10	11.38	TRIML2	NP_775824.2	303	9	40.31
CSRP2	NP_001312.1	778	4	4.88	TRIML1	XP_006714270.1	288	9	45.67
E2F7	XP_011536268.1	958	7	6.94	FRG1	NP_004468.1	291	8	43.53
NAV3	XP_016875655.1	465	9	18.39	FRG2	NP_001005217.1	104	8	132.86
SYT1	NP_001129277.1	1741	8	4.37	DUX4	NP_001350749.1	540	7	24.63
PAWR	NP_001341661.1	393	8	19.34	PLEKHG4B	NP_443141.4	512	3	11.13
PPP1R12A	NP_001231921.1	1696	6	3.36	LRRC14B	NP_001073947.1	146	6	70.98
OTOGL	XP_005268859.1	293	2	6.48	CCDC127	NP_660308.1	120	6	79.16
PTPRQ	XP_016874763.1	981	10	9.68	SDHA	XP_011512374.1	1669	5	4.38
MYF6	NP_002460.1	693	3	4.11	PDCD6	NP_037364.1	552	10	24.58
MYF5	NP_005584.2	860	3	3.31	AHRR	NP_001364168.1	282	4	17.97
LIN7A	XP_011537230.2	432	4	8.80	EXOC3	NP_009208.2	798	9	13.39
ACSS3	NP_078836.1	484	1	1.96	SLC9A3	NP_001271280.1	847	11	14.51
PPFIA2	XP_016875588.1	998	6	5.71	CEP72	NP_060610.2	342	15	46.29
CCDC59	NP_054886.2	329	0	0.00	TPPP	XP_016864482.1	988	14	14.17
METTL25	NP_115606.2	94	4	40.42	ZDHHC11B	NP_001338232.1	127	1	7.48
TMTC2	NP_689801.1	847	6	6.73	ZDHHC11	NP_079062.1	200	12	57.00
SLC6A15	NP_877499.1	625	6	9.12	BRD9	NP_001362808.1	370	12	30.81
TSPAN19	XP_016874352.1	111	4	34.23	TRIP13	NP_004228.1	1486	16	10.23
LRRIQ1	XP_011537121.1	604	4	6.29	NKD2	XP_005248439.1	186	7	35.75

ALX1	NP_008913.2	687	6	8.30	SLC12A7	XP_011512243.2	474	8	16.03
RASSF9	NP_005438.2	136	0	0.00	SLC6A19	NP_001003841.1	457	11	22.87
NTS	NP_006174.1	1025	3	2.78	SLC6A18	NP_872438.2	355	13	34.79
MGAT4C	XP_016874632.1	490	2	3.88	TERT	NP_001180305.1	1996	10	4.76
C12orf50	XP_016874375.2	85	2	22.35	CLPTM1L	XP_011512446.1	430	14	30.93
C12orf29	NP_001009894.2	128	3	22.26	SLC6A3	NP_001035.1	999	4	3.80
CEP290	XP_016875472.1	732	3	3.89	LPCAT1	NP_079106.3	669	9	12.78
TMTC3	NP_861448.2	788	0	0.00	MRPL36	XP_011512382.1	807	7	8.24
KITLG	NP_003985.2	1444	3	1.97	NDUFS6	NP_004544.1	688	6	8.28
DUSP6	NP_073143.2	1140	3	2.50	IRX4	NP_001265561.1	524	4	7.25
POC1B	NP_758440.1	343	5	13.85	IRX2	NP_001127694.1	551	6	10.34
POC1B-GALNT	NP_001186711.1	134	3	21.27	C5orf38	NP_001352617.1	79	2	24.05
GALNT4	NP_003765.2	160	3	17.81	IRX1	NP_077313.3	660	3	4.32
ATP2B1	XP_024304759.1	1020	5	4.66	ADAMTS16	NP_620687.2	271	3	10.52
CCER1	NP_689851.1	118	5	40.25	ICE1	NP_056140.1	284	7	23.41
EPYC	NP_004941.2	627	5	7.58	MED10	NP_115662.2	361	6	15.79
KERA	NP_008966.1	717	5	6.62	UBE2QL1	XP_024310129.1	789	7	8.43
LUM	NP_002336.1	1283	5	3.70	NSUN2	NP_001180384.1	1024	9	8.35
DCN	NP_598012.1	1302	3	2.19	SRD5A1	NP_001311251.1	382	4	9.95
BTG1	NP_001722.1	280	0	0.00	TENT4A	NP_008930.2	870	9	9.83
PLEKHG7	NP_001364258.1	326	1	2.91	ADCY2	NP_065433.2	1398	3	2.04
EEA1	XP_016875507.1	1419	0	0.00	C5orf49	NP_001083053.1	16	0	0.00
NUDT4	NP_950241.1	557	6	10.23	FASTKD3	NP_076996.2	256	4	14.84
UBE2N	NP_003339.1	2483	3	1.15	MTRR	XP_024301832.1	636	4	5.97
MRPL42	NP_751917.1	602	7	11.05	SEMA5A	NP_003957.2	866	3	3.29
SOCS2	XP_011537237.1	911	5	5.21	TAS2R1	NP_062545.1	395	2	4.81
CRADD	NP_001307029.1	373	3	7.64	ATPSCKMT	NP_001245317.1	413	4	9.20
PLXNC1	NP_005752.1	528	5	9.00	CCT5	NP_001293082.1	1954	6	2.92
CEP83	NP_001354970.1	352	3	8.10	CMBL	NP_620164.1	476	3	5.99
TMCC3	NP_065749.3	303	1	3.14	MARCHF6	NP_005876.2	525	2	3.62
NDUFA12	NP_061326.1	575	4	6.61	ROPN1L	XP_016865436.1	330	4	11.51
NR2C1	XP_011537019.1	449	2	4.23	ANKRD33B	NP_001157912.1	29	0	0.00
FGD6	NP_060821.3	352	2	5.40	DAP	NP_001278892.1	349	1	2.72
VEZT	NP_001339017.1	340	1	2.79	CTNND2	XP_016864563.1	1439	8	5.28
METAP2	NP_006829.1	1401	4	2.71	DNAH5	XP_016864666.1	856	7	7.77
USP44	XP_016875499.1	575	0	0.00	TRIO	NP_009049.2	1049	3	2.72
NTN4	NP_001316629.1	345	3	8.26	OTULINL	NP_061891.1	270	9	31.67
SNRPF	NP_003086.1	1488	6	3.83	OTULIN	NP_612357.4	244	3	11.68
CCDC38	NP_872302.2	125	3	22.80	ANKH	NP_473368.1	251	2	7.57
AMDHD1	NP_689648.2	315	5	15.08	FBXL7	NP_036436.1	1108	15	12.86
HAL	NP_002099.1	648	2	2.93	MARCHF11	NP_001096032.1	493	15	28.90
LTA4H	NP_001243572.1	755	3	3.77	ZNF622	NP_219482.1	814	10	11.67
ELK3	NP_005221.2	289	0	0.00	RETREG1	NP_001030022.1	307	12	37.13
CDK17	XP_016874896.1	1762	2	1.08	MYO10	XP_006714538.1	1350	15	10.56
CFAP54	NP_001354814.1	143	0	0.00	BASP1	NP_006308.3	429	6	13.29
NEDD1	NP_001128647.1	495	1	1.92	CDH18	XP_016864413.1	496	12	22.98
TMPO	NP_001027454.1	848	3	3.36	CDH12	NP_001304157.1	440	11	23.75
SLC25A3	NP_005879.1	1222	2	1.55	PRDM9	NP_064612.2	577	5	8.23
IKBIP	NP_963906.1	209	4	18.18	CDH10	NP_001304153.1	707	11	14.78
APAF1	NP_863651.1	1821	1	0.52	CDH9	NP_057363.3	555	11	18.83
ANKS1B	XP_006719577.1	1473	3	1.93	CDH6	XP_016864399.1	533	12	21.39
FAM71C	NP_699195.1	100	0	0.00	DROSHA	XP_016864890.1	2589	3	1.10
UHRF1BP1L	XP_016874536.1	187	2	10.16	C5orf22	XP_016865096.1	204	7	32.60
ACTR6	NP_071941.1	573	2	3.32	PDZD2	XP_005248326.1	1569	11	6.66
DEPDC4	XP_016874273.1	49	1	19.39	GOLPH3	NP_071413.1	1608	6	3.54
SCYL2	NP_001317182.1	221	2	8.60	MTMR12	NP_001281273.1	213	6	26.76
SLC17A8	NP_647480.1	547	4	6.95	ZFR	NP_057191.2	866	8	8.78
NR1H4	NP_001193906.1	1084	1	0.88	SUB1	XP_016864475.1	1018	6	5.60
GAS2L3	XP_016874695.1	569	2	3.34	NPR3	XP_011512349.1	694	1	1.37
ANO4	XP_011536213.1	315	4	12.06	TARS1	NP_001245367.1	1136	10	8.36
SLC5A8	NP_666018.3	479	1	1.98	ADAMTS12	NP_001311441.1	414	3	6.88
UTP20	NP_055318.2	523	0	0.00	RXFP3	NP_057652.1	372	1	2.55
ARL1	NP_001287997.1	1428	1	0.67	SLC45A2	NP_057264.4	628	3	4.54
SPIC	NP_689536.1	233	1	4.08	AMACR	NP_001161067.1	537	3	5.31
MYBPC1	XP_016874805.1	492	2	3.86	C1QTNF3-AM	NP_112207.1	177	2	10.73
CHPT1	NP_064629.2	453	6	12.58	RAI14	NP_056392.2	1071	4	3.55

SYCP3	XP_024304762.1	523	1	1.82	TTC23L	NP_001373100.1	18	0	0.00
GNPTAB	NP_077288.2	305	5	15.57	RAD1	NP_002844.1	555	2	3.42
DRAM1	NP_060840.2	298	0	0.00	BRIX1	NP_060791.3	1341	5	3.54
WASHC3	XP_024304766.1	341	7	19.50	DNAJC21	XP_011512267.2	756	3	3.77
NUP37	NP_076962.2	1374	6	4.15	AGXT2	XP_005248394.1	630	3	4.52
PARPBP	NP_001306917.1	441	6	12.92	PRLR	NP_001191247.1	546	3	5.22
PMCH	NP_002665.2	876	5	5.42	SPEF2	XP_011512438.1	339	6	16.81
IGF1	XP_016874752.1	3204	7	2.08	IL7R	NP_002176.2	858	0	0.00
PAH	NP_001341233.1	820	4	4.63	CAPSL	XP_006714507.1	483	5	9.83
ASCL1	NP_004307.2	1063	2	1.79	UGT3A1	XP_011512259.1	327	3	8.72
C12orf42	XP_016874770.1	34	1	27.94	UGT3A2	NP_001161788.1	263	2	7.22
STAB2	NP_060034.9	495	3	5.76	LMBRD2	NP_001007528.1	56	1	16.96
NT5DC3	NP_001026871.1	186	3	15.32	SKP2	NP_116026.1	1425	4	2.67
HSP90B1	NP_003290.1	2323	5	2.04	NADK2	NP_001078880.1	119	1	7.98
C12orf73	XP_016875405.1	59	1	16.10	RANBP3L	XP_006714518.1	218	4	17.43
TDG	NP_003202.3	633	0	0.00	SLC1A3	XP_024301949.1	1128	6	5.05
GLT8D2	XP_016875488.1	195	0	0.00	NIPBL	NP_597677.2	1098	6	5.19
HCFC2	NP_037452.1	456	2	4.17	CPLANE1	XP_011512391.1	163	4	23.31
NFYB	XP_024304752.1	1583	2	1.20	NUP155	NP_001265241.1	942	7	7.06
TXNRD1	NP_001087240.1	2203	4	1.72	WDR70	NP_060504.1	213	6	26.76
EID3	NP_001008395.1	178	1	5.34	GDNF	NP_001177398.1	1298	3	2.20
CHST11	NP_060883.1	329	1	2.89	EGFLAM	NP_001192230.1	1098	6	5.19
SLC41A2	XP_016875502.1	271	0	0.00	LIFR	XP_016864951.1	593	7	11.21
C12orf45	NP_689531.2	57	0	0.00	OSMR	XP_011512463.1	408	8	18.63
ALDH1L2	NP_001029345.2	516	2	3.68	RICTOR	XP_016864801.1	1023	2	1.86
WASHC4	NP_001280569.1	446	0	0.00	FYB1	XP_011512313.1	875	7	7.60
APPL2	XP_016875043.1	543	1	1.75	C9	NP_001728.1	307	3	9.28
C12orf75	NP_001138671.1	69	1	13.77	DAB2	NP_001231800.1	1045	6	5.45
NUAK1	NP_055655.1	462	4	8.22	PTGER4	XP_016865148.1	1033	8	7.36
CKAP4	NP_006816.2	778	3	3.66	TTC33	NP_036514.1	776	8	9.79
TCP11L2	XP_016874616.1	218	3	13.07	PRKAA1	NP_006242.5	1755	6	3.25
POLR3B	NP_060552.4	1740	6	3.28	RPL37	NP_000988.1	885	2	2.15
RFX4	NP_998759.1	756	2	2.51	CARD6	NP_115976.2	289	8	26.30
RIC8B	NP_001317074.1	484	4	7.85	C7	NP_000578.2	240	5	19.79
TMEM263	NP_689474.1	70	0	0.00	MROH2B	NP_775760.3	182	1	5.22
MTERF2	XP_016875474.1	274	0	0.00	C6	XP_011512421.1	316	2	6.01
CRY1	XP_024304612.1	944	4	4.03	PLCXD3	NP_001005473.1	448	3	6.36
BTBD11	NP_001018082.1	245	3	11.63	OXCT1	NP_001351230.1	555	3	5.13
PWP1	NP_008993.1	1087	5	4.37	C5orf51	NP_787117.3	19	0	0.00
PRDM4	NP_036538.3	352	5	13.49	FBXO4	XP_011512328.1	469	0	0.00
ASCL4	NP_982260.3	141	4	26.95	GHR	NP_001229328.1	576	3	4.95
WSCD2	XP_016875732.1	919	8	8.27	CCDC152	NP_001128320.1	80	4	47.50
CMKLR1	NP_001135815.1	485	2	3.92	SELENOP	NP_001087195.1	369	3	7.72
FICD	NP_009007.2	208	0	0.00	ANXA2R	NP_001369281.1	71	0	0.00
SART3	NP_055521.1	649	5	7.32	ZNF131	NP_001317641.1	391	4	9.72
ISCU	NP_998760.1	767	0	0.00	NIM1K	NP_699192.1	1192	4	3.19
TMEM119	NP_859075.2	183	0	0.00	HMGCS1	NP_001311151.1	833	1	1.14
SELPLG	NP_001193538.1	685	1	1.39	CCL28	NP_683513.1	380	1	2.50
CORO1C	XP_016874610.1	866	3	3.29	TMEM267	NP_001364324.1	110	2	17.27
SSH1	NP_061857.3	830	3	3.43	C5orf34	NP_940968.1	100	1	9.50
DAO	NP_001908.3	899	2	2.11	PAIP1	NP_877590.1	949	3	3.00
SVOP	NP_061181.1	619	3	4.60	NNT	XP_016864782.1	447	0	0.00
USP30	XP_024304995.1	513	1	1.85	FGF10	NP_004456.1	1070	5	4.44
ALKBH2	NP_001138846.1	234	1	4.06	MRPS30	NP_057724.2	414	6	13.77
UNG	NP_550433.1	1556	1	0.61	HCN1	NP_066550.2	537	2	3.54
ACACB	XP_011536566.1	995	6	5.73	EMB	NP_940851.1	256	2	7.42
FOXN4	NP_998761.2	633	4	6.00	PARP8	XP_005248653.1	220	9	38.86
MYO1H	XP_011536525.1	926	6	6.16	ISL1	NP_002193.2	1412	3	2.02
KCTD10	NP_001304328.1	276	6	20.65	ITGA1	NP_852478.1	674	5	7.05
UBE3B	XP_011537263.1	1138	6	5.01	PELO	NP_057030.3	507	4	7.49
MMAB	NP_443077.1	242	4	15.70	ITGA2	NP_002194.2	946	4	4.02
MVK	XP_016874803.1	617	3	4.62	MOCS2	NP_004522.1	386	5	12.31
FAM222A	XP_016875544.1	86	0	0.00	FST	XP_005248457.1	1032	2	1.84
TRPV4	XP_011536935.2	678	2	2.80	NDUFS4	NP_002486.1	758	3	3.76
GLTP	NP_057517.1	307	1	3.09	ARL15	NP_061960.1	1553	2	1.22
TCHP	NP_115676.1	390	1	2.44	HSPB3	NP_006299.1	1408	2	1.35

GIT2	NP_001317082.1	630	2	3.02
ANKRD13A	XP_005254037.1	198	0	0.00
C12orf76	NP_997318.1	10	0	0.00
IFT81	XP_016874706.1	383	0	0.00
ATP2A2	NP_001672.1	1595	3	1.79
ANAPC7	NP_001372140.1	778	0	0.00
ARPC3	NP_001274151.1	799	4	4.76
GPN3	NP_001157844.1	595	4	6.39
FAM216A	NP_037432.2	138	1	6.88
VPS29	NP_057310.1	756	3	3.77
RAD9B	NP_001354981.1	301	0	0.00
PPTC7	NP_644812.1	694	2	2.74
TCTN1	XP_011537036.1	260	0	0.00
HVCN1	XP_016875516.1	424	0	0.00
PPP1CC	XP_011536807.1	3078	6	1.85
CCDC63	NP_689804.1	279	4	13.62
MYL2	NP_000423.2	1591	4	2.39
CUX2	NP_056082.2	1109	10	8.57
PHETA1	NP_001171467.1	154	10	61.69
SH2B3	XP_011536021.1	617	11	16.94
ATXN2	NP_002964.4	1334	13	9.26
BRAP	XP_016875481.1	474	6	12.02
ACAD10	NP_001130010.1	946	14	14.06
ALDH2	NP_000681.2	1083	14	12.28
MAPKAPK5	NP_001358408.1	365	11	28.63
TMEM116	XP_011537250.1	786	14	16.92
ERP29	NP_006808.1	403	6	14.14
NAA25	NP_079229.2	527	13	23.43
TRAFD1	NP_001137378.1	282	17	57.27
HECTD4	NP_001103132.4	562	16	27.04
RPL6	XP_016875270.1	1493	10	6.36
PTPN11	NP_001317366.1	1973	7	3.37
RPH3A	NP_001334882.1	836	6	6.82
OAS1	NP_001307080.1	556	3	5.13
OAS3	XP_005253946.1	364	3	7.83
OAS2	NP_058197.2	477	3	5.97
DTX1	NP_004407.2	604	4	6.29
RASAL1	NP_001180450.1	382	4	9.95
CFAP73	XP_024304746.1	59	0	0.00
DDX54	NP_076977.3	1464	5	3.24
RITA1	NP_001273144.1	40	1	23.75
IQCD	XP_005253891.1	80	0	0.00
TPCN1	NP_001137291.1	464	3	6.14
SLC8B1	NP_001317395.1	265	2	7.17
PLBD2	NP_775813.2	111	0	0.00
SDS	NP_006834.2	558	3	5.11
SDSL	XP_016874252.1	639	3	4.46
LHX5	NP_071758.1	689	6	8.27
RBM19	XP_016875769.1	735	7	9.05
TBX5	XP_016875401.1	945	5	5.03
TBX3	NP_005987.3	1091	6	5.22
MED13L	XP_016874579.1	651	8	11.67
MAP1LC3B2	NP_001078950.1	571	1	1.66
SPRING1	XP_024304957.1	60	8	126.66
RNFT2	NP_116203.2	538	2	3.53
HRK	XP_016875635.1	239	4	15.90
FBXW8	NP_699179.2	750	6	7.60
TESC	XP_016875021.1	1174	12	9.71
FBXO21	XP_016874527.1	492	5	9.65
NOS1	NP_000611.1	1364	1	0.70
KSR2	XP_011536526.1	1219	4	3.12
RFC5	NP_031396.1	1301	3	2.19
WSB2	NP_001265486.1	807	9	10.59
VSIG10	XP_006719530.1	69	5	68.84
PEBP1	NP_002558.1	1199	3	2.38
TAOK3	NP_001333422.1	501	7	13.27
SNX18	XP_016864486.1	495	1	1.92
ESM1	NP_001129076.1	471	1	2.02
GZMK	NP_002095.1	690	3	4.13
GZMA	NP_006135.2	973	5	4.88
CDC20B	NP_001139206.2	922	2	2.06
GPX8	XP_006714694.1	705	3	4.04
MCIDAS	NP_001177716.1	422	4	9.00
CCNO	NP_066970.3	1295	7	5.13
DHX29	NP_001332893.1	1032	8	7.36
MTREX	NP_056175.3	1739	3	1.64
PLPP1	NP_003702.2	560	7	11.87
SLC38A9	XP_011541486.1	284	5	16.72
DDX4	NP_077726.1	1656	7	4.02
IL31RA	NP_001229566.1	267	7	24.91
IL6ST	NP_001351205.1	762	2	2.49
ANKRD55	XP_016865342.1	989	10	9.61
MAP3K1	NP_005912.1	1840	6	3.10
SETD9	NP_001165461.1	78	4	48.72
MIER3	NP_689835.3	431	10	22.04
GPBP1	NP_001120708.1	270	8	28.15
ACTBL2	NP_001017992.1	1066	4	3.56
PLK2	NP_001239155.1	1761	5	2.70
GAPT	NP_689900.1	443	0	0.00
RAB3C	NP_001304844.1	974	3	2.93
PDE4D	XP_016865055.1	928	5	5.12
DEPDC1B	NP_001138680.1	451	6	12.64
ELOVL7	XP_016865374.1	439	3	6.49
ERCC8	NP_000073.1	482	3	5.91
NDUFAF2	NP_777549.1	387	3	7.36
SMIM15	NP_001041714.1	74	1	12.84
ZSWIM6	NP_065979.1	241	0	0.00
KIF2A	NP_001091981.1	1314	2	1.45
DIMT1	NP_055288.1	1343	2	1.41
IPO11	NP_001128251.1	541	9	15.80
LRRC70	NP_852607.3	399	2	4.76
HTR1A	NP_000515.2	1244	5	3.82
RNF180	XP_016864873.1	251	11	41.63
RGS7BP	NP_001025046.1	916	11	11.41
SHISAL2B	NP_001157914.1	54	12	211.10
SREK1IP1	XP_011541646.1	229	13	53.93
CWC27	NP_005860.2	1380	14	9.64
ADAMTS6	XP_011541419.1	364	10	26.10
CENPK	XP_016865182.1	517	4	7.35
PPWD1	NP_056157.1	573	13	21.55
TRIM23	NP_150230.1	1828	11	5.72
TRAPPC13	NP_001087224.1	117	10	81.19
SGTB	NP_061945.1	1032	11	10.13
NLN	NP_065777.1	565	2	3.36
ERBIN	XP_016865124.1	1114	8	6.82
SREK1	NP_001070667.1	818	5	5.81
MAST4	XP_024301812.1	481	2	3.95
CD180	NP_005573.2	840	0	0.00
PIK3R1	XP_016865074.1	1613	1	0.59
SLC30A5	NP_075053.2	426	2	4.46
CCNB1	NP_114172.1	2900	8	2.62
CENPH	NP_075060.1	573	1	1.66
MRPS36	NP_150597.1	348	0	0.00
CDK7	NP_001790.1	1220	4	3.11
CCDC125	XP_011541560.1	58	0	0.00
AK6	NP_001015891.1	804	1	1.18
TAF9	NP_003178.1	804	4	4.73
RAD17	NP_579917.1	984	8	7.72
MARVELD2	NP_001033692.2	278	3	10.25
OCLN	NP_001192183.1	1121	10	8.47
GTF2H2C	XP_011541917.1	345	8	22.03
SERF1B	NP_075267.1	137	8	55.47

SUDS3	XP_011536973.1	646	3	4.41	SMN2	NP_075013.1	856	7	7.77
SRRM4	NP_919262.2	602	1	1.58	SERF1A	NP_068802.1	144	8	52.78
HSPB8	NP_055180.1	729	2	2.61	SMN1	NP_001284644.1	1039	8	7.31
CCDC60	NP_848594.2	103	1	9.22	NAIP	NP_075043.1	683	9	12.52
TMEM233	XP_011536632.1	17	0	0.00	GTF2H2	NP_001351501.1	625	9	13.68
PRKAB1	NP_006244.2	670	1	1.42	BDP1	XP_016865119.1	586	5	8.11
CIT	XP_011536090.1	1591	1	0.60	MCCC2	NP_071415.1	534	3	5.34
BICDL1	XP_006719757.1	305	3	9.34	CARTPT	NP_004282.1	1131	1	0.84
RAB35	XP_024304569.1	618	5	7.69	MAP1B	NP_005900.2	1453	12	7.85
GCN1	NP_006827.1	1027	6	5.55	MRPS27	NP_055899.2	338	6	16.86
RPLP0	NP_444505.1	2220	5	2.14	PTCD2	NP_079030.3	152	7	43.75
PXN	NP_002850.2	1666	2	1.14	ZNF366	NP_689838.1	342	8	22.22
SIRT4	NP_001372662.1	1143	5	4.16	TNPO1	NP_002261.3	1204	9	7.10
PLA2G1B	NP_000919.1	1102	0	0.00	FCHO2	NP_620137.2	683	9	12.52
MSI1	XP_011536665.1	692	3	4.12	TMEM171	NP_775761.4	146	7	45.55
COX6A1	NP_004364.2	538	4	7.06	TMEM174	NP_694949.1	106	8	71.69
TRIAP1	NP_057483.1	364	0	0.00	FOXD1	NP_004463.1	661	3	4.31
GATC	NP_789788.1	73	2	26.03	BTF3	NP_001032726.1	903	3	3.16
SRSF9	NP_003760.1	1132	5	4.20	ANKRA2	NP_075526.1	951	3	3.00
DYNLL1	NP_003737.1	1285	2	1.48	UTP15	NP_115551.2	878	4	4.33
COQ5	NP_115690.3	497	6	11.47	ARHGEF28	NP_001073948.2	905	6	6.30
RNF10	NP_001317403.1	477	4	7.97	ENC1	NP_001243503.1	496	2	3.83
POP5	NP_937845.1	280	1	3.39	HEXB	NP_000512.2	398	1	2.39
CABP1	XP_016875724.1	1592	1	0.60	GFM2	NP_001268231.1	633	0	0.00
MLEC	NP_001290557.1	392	0	0.00	NSA2	NP_055701.1	1091	3	2.61
UNC119B	NP_001074002.1	358	3	7.96	FAM169A	XP_016864821.1	92	0	0.00
ACADS	NP_000008.1	663	0	0.00	GCNT4	NP_001353666.1	314	1	3.03
SPPL3	NP_620584.2	284	1	3.34	ANKRD31	NP_001157915.1	60	0	0.00
HNF1A	XP_024304936.1	808	0	0.00	HMGCR	XP_011541659.1	1395	1	0.68
C12orf43	NP_001273126.1	64	1	14.84	CERT1	NP_001365933.1	416	0	0.00
OASL	XP_016875629.1	2282	4	1.67	POLK	XP_005248591.1	636	1	1.49
P2RX7	NP_002553.3	634	3	4.50	ANKDD1B	XP_016865303.1	919	1	1.03
P2RX4	NP_001248327.1	421	4	9.03	POC5	XP_016864526.1	213	1	4.46
CAMKK2	XP_016874191.1	952	3	2.99	SV2C	NP_001284645.1	454	5	10.46
ANAPC5	NP_001317418.1	1008	2	1.88	IQGAP2	NP_006624.3	876	6	6.51
RNF34	NP_079402.2	436	2	4.36	F2RL2	NP_004092.1	550	3	5.18
KDM2B	XP_011537169.1	1088	4	3.49	F2R	NP_001983.2	827	6	6.89
ORAI1	NP_116179.2	453	5	10.49	F2RL1	NP_005233.4	544	7	12.22
MORN3	XP_011536516.1	165	5	28.79	S100Z	XP_016864660.1	93	5	51.07
TMEM120B	NP_001074294.2	167	8	45.51	CRHBP	NP_001873.2	352	5	13.49
RHOF	NP_061907.2	1641	6	3.47	AGGF1	NP_060516.2	457	3	6.24
SETD1B	NP_001340274.1	2030	9	4.21	ZBED3	NP_001316493.1	166	1	5.72
HPD	NP_002141.2	600	1	1.58	PDE8B	NP_001336679.1	431	4	8.82
PSMD9	NP_002804.2	881	4	4.31	WDR41	XP_011541807.1	214	1	4.44
CFAP251	NP_653269.3	273	4	13.92	OTP	NP_115485.1	697	0	0.00
BCL7A	NP_066273.1	342	5	13.89	TBCA	NP_001284669.1	671	0	0.00
MLXIP	XP_006719353.1	283	1	3.36	AP3B1	NP_003655.3	1072	6	5.32
IL31	NP_001014358.1	243	0	0.00	SCAMP1	NP_004857.4	488	5	9.73
LRRC43	XP_016874613.1	472	1	2.01	LHFPL2	XP_024310089.1	196	2	9.69
B3GNT4	NP_110392.1	132	0	0.00	ARSB	NP_000037.2	480	2	3.96
DIABLO	NP_001265271.1	677	3	4.21	DMGDH	NP_037523.2	423	4	8.98
VPS33A	NP_001337949.1	549	5	8.65	BHMT2	NP_060084.2	314	2	6.05
CLIP1	XP_016875281.1	962	4	3.95	BHMT	NP_001704.2	536	2	3.54
ZCCHC8	NP_001337864.1	373	6	15.28	JMY	NP_689618.4	239	2	7.95
RSRC2	XP_016875319.1	424	6	13.44	HOMER1	NP_001264006.1	825	0	0.00
KNTC1	XP_006719769.1	706	3	4.04	TENT2	XP_016864640.1	578	4	6.57
HCAR2	NP_808219.1	562	3	5.07	CMYA5	NP_705838.3	626	6	9.10
HCAR3	NP_006009.2	554	3	5.14	MTX3	NP_001350747.1	256	6	22.26
HCAR1	NP_115943.1	465	4	8.17	THBS4	NP_003239.2	631	4	6.02
DENR	NP_003668.2	884	5	5.37	SERINC5	NP_001167542.1	301	2	6.31
CCDC62	XP_006719707.1	172	2	11.05	SPZ1	NP_115956.3	293	1	3.24
HIP1R	XP_011537265.1	875	2	2.17	ZFYVE16	XP_016865580.1	306	11	34.15
VPS37B	NP_078943.1	233	1	4.08	FAM151B	NP_991111.2	287	5	16.55
ABCB9	NP_001229943.1	439	4	8.66	ANKRD34B	NP_001004441.2	77	4	49.35
OGFOD2	NP_001291762.1	143	4	26.57	DHFR	NP_000782.1	1792	9	4.77
ARL6IP4	NP_061164.4	315	1	3.02	MTRNR2L2	NP_001177399.1	57	2	33.33

PITPNM2	NP_001371593.1	480	5	9.90	MSH3	NP_002430.3	863	8	8.81
MPHOSPH9	XP_011536042.1	344	6	16.57	RASGRF2	NP_008840.1	844	4	4.50
C12orf65	XP_024305041.1	600	2	3.17	CKMT2	NP_001816.2	634	1	1.50
CDK2AP1	NP_004633.1	295	2	6.44	ZCCHC9	NP_115656.1	417	2	4.56
SBNO1	NP_060653.3	375	6	15.20	ACOT12	XP_016864536.1	349	1	2.72
KMT5A	NP_065115.3	1937	0	0.00	SSBP2	NP_001243665.1	339	8	22.42
RILPL2	XP_011536314.1	249	1	3.82	ATG10	NP_113670.1	340	1	2.79
SNRNP35	XP_016874213.1	601	1	1.58	RPS23	NP_001016.1	1536	3	1.86
RILPL1	NP_847884.2	180	0	0.00	ATP6AP1L	NP_001373044.1	149	2	12.75
TMED2	NP_001308374.1	851	2	2.23	TMEM167A	NP_777569.1	204	3	13.97
DDX55	XP_016875199.1	2090	6	2.73	XRCC4	XP_016865316.1	664	9	12.88
EIF2B1	NP_001405.1	600	4	6.33	VCAN	NP_004376.2	1136	4	3.34
GTF2H3	NP_001507.2	541	1	1.76	HAPLN1	XP_016864542.1	471	5	10.08
TCTN2	XP_006719668.1	262	3	10.88	EDIL3	NP_001265571.1	626	5	7.59
ATP6V0A2	NP_036595.2	783	7	8.49	COX7C	NP_001858.1	573	2	3.32
DNAH10	NP_001359035.1	667	5	7.12	RASA1	NP_002881.1	968	3	2.94
CCDC92	NP_079416.1	201	6	28.36	CCNH	NP_001351004.1	1047	3	2.72
ZNF664	NP_689650.1	298	6	19.13	TMEM161B	NP_001336336.1	165	1	5.76
RFLNA	NP_001352085.1	215	1	4.42	MEF2C	NP_001180277.1	1908	11	5.48
NCOR2	NP_001070729.2	2311	4	1.64	CETN3	NP_001284697.1	1757	9	4.87
SCARB1	NP_001354916.1	766	2	2.48	MBLAC2	NP_981951.2	189	6	30.16
UBC	NP_066289.3	3319	4	1.14	POLR3G	XP_016864446.1	317	6	17.98
DHX37	NP_116045.2	1250	5	3.80	LYSMD3	XP_005248478.1	154	6	37.01
BRI3BP	XP_011536242.1	125	6	45.60	ADGRV1	XP_016865452.1	570	7	11.67
AACS	NP_076417.2	712	3	4.00	ARRDC3	NP_065852.1	613	8	12.40
TMEM132B	NP_001353783.1	361	6	15.79	NR2F1	NP_005645.1	1152	4	3.30
TMEM132C	NP_001129575.2	498	6	11.45	FAM172A	XP_005272162.1	137	1	6.93
SLC15A4	NP_663623.1	500	4	7.60	POU5F2	NP_694948.1	185	0	0.00
GLT1D1	XP_016874344.1	171	4	22.22	KIAA0825	NP_001139150.1	55	0	0.00
TMEM132D	NP_597705.2	505	5	9.41	SLF1	XP_016865468.1	1762	6	3.23
FZD10	NP_009128.1	602	3	4.73	MCTP1	XP_016865345.1	307	4	12.38
PIWIL1	XP_016875718.1	1078	2	1.76	FAM81B	NP_689761.2	89	3	32.02
RIMBP2	XP_016874595.1	576	3	4.95	TTC37	NP_055454.1	713	1	1.33
STX2	NP_001337978.2	747	1	1.27	ARSK	NP_937793.1	216	0	0.00
RAN	NP_006316.1	1933	1	0.49	GPR150	NP_954713.1	294	0	0.00
ADGRD1	NP_001317426.1	411	0	0.00	RFESD	XP_016864902.1	102	3	27.94
SFSWAP	NP_001248340.1	385	5	12.34	SPATA9	XP_016865438.1	64	2	29.69
MMP17	NP_057239.4	436	2	4.36	RHOBTB3	NP_055714.3	1599	5	2.97
ULK1	XP_011537100.1	899	1	1.06	GLRX	NP_001112362.1	860	1	1.10
PUS1	NP_079491.2	873	4	4.35	ELL2	NP_036213.2	493	4	7.71
EP400	NP_056224.3	1458	3	1.95	PCSK1	NP_000430.3	1158	8	6.56
DDX51	NP_778236.2	1187	5	4.00	CAST	NP_001741.4	458	4	8.30
NOC4L	NP_076983.1	964	4	3.94	ERAP1	NP_001336173.1	864	5	5.50
GALNT9	NP_001116108.1	580	3	4.91	ERAP2	NP_071745.1	656	6	8.69
FBRSL1	XP_011533109.1	151	2	12.58	LNPEP	XP_024301813.1	723	9	11.83
LRCOL1	XP_016874149.1	14	0	0.00	LIX1	NP_694966.3	339	10	28.02
P2RX2	XP_005266212.1	391	1	2.43	RIOK2	NP_060813.2	912	14	14.58
POLE	NP_006222.2	1345	6	4.24	RGMB	NP_001353438.1	318	12	35.85
PXMP2	NP_061133.1	231	0	0.00	CHD1	NP_001363123.1	1922	9	4.45
PGAM5	NP_001164014.1	404	0	0.00	FAM174A	NP_940909.1	59	15	241.51
ANKLE2	NP_055929.1	218	1	4.36	ST8SIA4	XP_011541932.1	459	8	16.56
GOLGA3	XP_011533095.1	339	7	20.65	SLCO4C1	XP_011541672.1	407	12	28.01
CHFR	NP_001154819.1	749	7	9.86	SLCO6A1	XP_011541450.1	262	10	36.26
ZNF605	NP_899061.1	185	3	18.12	PAM	XP_024301834.1	367	0	0.00
ZNF26	NP_001243208.1	238	2	9.98	GIN1	XP_016865088.1	274	9	31.20
ZNF84	NP_003419.3	218	2	11.62	PPIP5K2	NP_001268400.1	539	10	17.62
ZNF140	XP_011533135.1	183	3	22.25	MACIR	NP_149988.1	100	9	85.50
ZNF891	XP_016874155.1	145	0	0.00	NUDT12	XP_005272154.1	1163	8	6.53
ZNF10	NP_056209.2	199	2	15.91	EFNA5	XP_006714628.1	950	3	3.00
ZNF268	NP_003406.1	194	2	17.81	FBXL17	NP_001156787.2	576	1	1.65
ANHX	XP_011533124.1	94	0	0.00	FER	XP_016864721.1	1002	3	2.84
TUBA3C	NP_005992.1	1459	2	2.60	PJA2	XP_016865588.1	794	4	4.79
TPTE2	NP_570141.3	833	8	16.59	MAN2A1	NP_002363.2	465	3	6.13
MPHOSPH8	NP_059990.2	2763	8	4.58	TMEM232	XP_011541867.1	66	4	57.57
PSPC1	NP_001341837.1	565	5	12.93	SLC25A46	NP_001290179.1	328	7	20.27
ZMYM5	XP_011533612.1	120	9	101.78	TSLP	NP_149024.1	615	3	4.63

ZMYM2	XP_024305174.1	664	5	9.54	WDR36	NP_644810.2	959	5	4.95
GJA3	NP_068773.2	288	6	24.74	CAMK4	NP_001310303.1	1303	5	3.65
GJB2	NP_003995.2	572	6	11.72	STARD4	NP_631903.1	211	0	0.00
GJB6	NP_001103690.1	632	4	6.68	NREP	NP_001135946.1	242	3	11.78
CRYL1	NP_001350576.1	584	13	22.26	EPB41L4A	XP_011541833.1	390	2	4.87
IFT88	NP_001340494.1	1337	10	7.11	APC	NP_001341826.1	1038	4	3.66
IL17D	NP_001372154.1	349	4	10.89	SRP19	NP_001191128.1	904	6	6.30
EEF1AKMT1	XP_016875921.1	173	14	76.87	REEP5	NP_005660.4	1074	5	4.42
XPO4	NP_001358990.1	337	7	19.73	DCP2	NP_689837.2	935	5	5.08
LATS2	NP_055387.2	783	5	6.07	MCC	NP_001078846.2	190	7	35.00
SAP18	NP_005861.2	924	5	5.14	TSSK1B	NP_114417.1	1281	5	3.71
SKA3	NP_001159489.1	550	1	1.73	YTHDC2	XP_011541883.1	1518	3	1.88
MRPL57	XP_016876229.1	354	7	18.78	KCNN2	NP_001359162.1	426	2	4.46
ZDHHC20	NP_694983.2	271	9	31.55	TRIM36	NP_061170.2	853	3	3.34
MICU2	NP_689939.1	421	3	6.77	PGGT1B	XP_011541792.1	327	2	5.81
FGF9	NP_002001.1	904	2	2.10	CCDC112	NP_001362825.1	95	4	40.00
SGCG	NP_001365173.1	384	4	9.90	FEM1C	NP_064562.1	1073	2	1.77
SACS	NP_055178.3	475	5	10.00	TMED7-TICAN	NP_001157940.1	260	2	7.31
TNFRSF19	NP_001341914.1	424	1	2.24	TICAM2	NP_067681.1	245	2	7.75
MIPEP	NP_005923.3	510	6	11.18	TMED7	NP_861974.1	678	4	5.60
C1QTNF9B	NP_001007538.1	79	3	36.07	CDO1	NP_001310495.1	694	0	0.00
SPATA13	NP_001273721.1	502	4	7.57	ATG12	NP_004698.3	733	1	1.30
C1QTNF9	NP_848635.2	88	3	32.38	AP3S1	XP_016864514.1	495	3	5.76
PARP4	XP_011533233.1	621	3	4.59	LVRN	NP_776161.3	518	2	3.67
ATP12A	NP_001172014.1	1404	1	0.68	ARL14EPL	XP_016865211.1	59	0	0.00
RNF17	XP_011533454.1	1038	3	2.75	COMMD10	NP_057228.1	537	8	14.15
CENPJ	NP_060921.3	789	2	2.41	SEMA6A	XP_005272099.2	742	1	1.28
PABPC3	NP_112241.2	941	3	3.03	DTWD2	NP_775937.1	162	5	29.32
AMER2	NP_954589.1	659	4	5.77	DMXL1	XP_005271966.1	628	5	7.56
MTMR6	NP_001372160.1	411	2	4.62	TNFAIP8	NP_001071122.1	335	2	5.67
NUP58	XP_011533630.1	287	1	3.31	HSD17B4	NP_000405.1	747	3	3.82
ATP8A2	NP_057613.4	1045	3	2.73	FAM170A	NP_001354885.1	124	2	15.32
SHISA2	NP_001007539.1	200	0	0.00	PRR16	NP_001287712.1	386	3	7.38
RNF6	NP_898866.1	606	3	4.70	FTMT	NP_803431.1	284	4	13.38
CDK8	NP_001251.1	1068	4	3.56	SRFBP1	XP_016864600.2	183	0	0.00
WASF3	NP_006637.2	661	4	5.75	LOX	NP_002308.2	1781	1	0.53
GPR12	NP_005279.1	459	3	6.21	ZNF474	NP_997200.1	37	2	51.35
USP12	NP_872294.2	550	5	8.64	SNCAIP	XP_016865567.1	1202	3	2.37
RPL21	NP_000973.2	1104	0	0.00	SNX2	NP_003091.2	766	5	6.20
RASL11A	NP_996563.1	1289	1	0.74	SNX24	XP_016864884.1	257	2	7.39
GTF3A	NP_002088.2	642	2	2.96	PPIC	NP_000934.1	1132	1	0.84
MTIF3	XP_016875906.1	826	3	3.45	PRDM6	NP_001129711.1	350	2	5.43
LNX2	XP_011533297.1	811	5	5.86	CEP120	NP_001362335.1	377	3	7.56
POLR1D	NP_689918.1	819	5	5.80	CSNK1G3	XP_016864546.1	797	2	2.38
GSX1	NP_663632.1	349	4	10.89	ZNF608	NP_001372550.1	313	9	27.31
PDX1	NP_000200.1	590	4	6.44	GRAMD2B	XP_011541895.1	186	10	51.07
CDX2	NP_001256.4	1156	7	5.75	ALDH7A1	NP_001189333.2	1243	6	4.59
URAD	NP_001099047.1	254	4	14.96	PHAX	NP_115553.2	752	11	13.90
FLT3	XP_016875975.1	1434	5	3.31	TEX43	NP_997291.1	75	14	177.32
PAN3	NP_787050.6	415	2	4.58	LMNB1	NP_005564.1	1518	4	2.50
FLT1	XP_016875974.1	1547	6	3.68	MARCHF3	XP_011541430.1	468	16	32.48
POMP	NP_057016.1	639	2	2.97	C5orf63	XP_016864953.1	107	13	115.41
SLC46A3	NP_001129391.1	235	0	0.00	MEGF10	XP_016865476.1	630	11	16.59
MTUS2	XP_011533321.1	205	1	4.63	PRRC1	NP_001273737.1	215	14	61.86
SLC7A1	NP_003036.1	764	2	2.49	CTXN3	NP_001041717.1	763	12	14.94
UBL3	NP_009037.1	356	0	0.00	SLC12A2	NP_001037.1	872	9	9.80
KATNAL1	XP_024305191.1	864	7	7.70	FBN2	XP_016864717.1	1031	14	12.90
HMGB1	XP_024305109.1	1766	2	1.08	SLC27A6	NP_054750.1	502	13	24.60
USPL1	NP_005791.3	227	5	20.92	ISOC1	NP_057132.2	447	15	31.88
ALOX5AP	NP_001191335.1	724	5	6.56	ADAMTS19	NP_598377.4	285	13	43.33
MEDAG	NP_116238.3	233	6	24.46	MINAR2	NP_001244237.1	38	10	249.99
TEX26	NP_689538.1	24	0	0.00	CHSY3	NP_787052.3	230	9	37.17
HSPH1	XP_016875851.1	1507	3	1.89	HINT1	NP_005331.1	985	5	4.82
B3GLCT	NP_919299.3	473	8	16.07	LYRM7	NP_859056.2	276	8	27.53
RXFP2	NP_570718.1	900	6	6.33	CDC42SE2	NP_001362562.1	228	10	41.66
FRY	NP_075463.2	386	5	12.31	RAPGEF6	NP_057424.3	385	6	14.80

ZAR1L	NP_001130043.1	163	2	11.66	FNIP1	NP_001333043.1	286	9	29.89
BRCA2	NP_000050.3	1745	6	3.27	ACSL6	NP_001192179.1	1046	7	6.36
N4BP2L1	NP_001273389.1	247	0	0.00	IL3	NP_000579.2	1385	8	5.49
N4BP2L2	XP_016875822.1	285	1	3.33	CSF2	NP_000749.2	1846	6	3.09
PDS5B	XP_016875937.1	990	6	5.76	P4HA2	XP_024302023.1	535	7	12.43
KL	NP_004786.2	652	0	0.00	PDLIM4	NP_003678.2	762	11	13.71
STARD13	XP_016876324.1	553	5	8.59	SLC22A4	NP_003050.2	605	10	15.70
RFC3	XP_011533477.1	1435	5	3.31	SLC22A5	NP_001295051.1	530	10	17.92
NBEA	NP_001371941.1	621	7	10.71	IRF1	NP_001341853.1	1276	13	9.68
MAB21L1	NP_005575.1	440	3	6.48	IL5	XP_005272045.1	1307	9	6.54
DCLK1	NP_001317000.1	1588	5	2.99	RAD50	NP_005723.2	771	0	0.00
SOHLH2	NP_060296.2	211	2	9.00	IL13	NP_002179.2	1705	9	5.01
CCDC169-SOH	NP_001185839.1	211	2	9.00	IL4	NP_000580.1	2510	10	3.78
CCDC169	NP_001185837.1	29	0	0.00	KIF3A	XP_016864485.1	1503	10	6.32
SPART	XP_024305102.1	503	4	7.55	CCNI2	NP_001274182.1	1024	1	0.93
CCNA1	NP_003905.1	1841	4	2.06	SEPTIN8	XP_016864743.1	432	7	15.39
SERTM1	NP_982276.2	351	1	2.71	SOWAHA	NP_787069.4	355	2	5.35
RFXAP	NP_000529.1	166	4	22.89	SHROOM1	NP_597713.2	221	6	25.79
SMAD9	NP_001365550.1	868	6	6.57	GDF9	NP_005251.1	680	1	1.40
ALG5	NP_001135836.1	729	4	5.21	UQCRQ	NP_055217.2	730	3	3.90
EXOSC8	XP_006719826.1	851	1	1.12	LEAP2	NP_443203.1	188	3	15.16
SUPT20H	XP_024305147.1	470	2	4.04	AFF4	XP_006714650.1	548	2	3.47
CSNK1A1L	NP_660204.2	601	1	1.58	ZCCHC10	NP_001295058.1	132	0	0.00
POSTN	NP_001273596.1	1140	1	0.83	HSPA4	NP_002145.3	3903	7	1.70
TRPC4	NP_001129430.1	565	3	5.04	FSTL4	XP_011541586.1	207	2	9.18
UFM1	NP_057701.1	564	4	6.74	C5orf15	NP_064584.1	218	1	4.36
FREM2	NP_997244.4	862	7	7.71	VDAC1	XP_016865310.1	1677	5	2.83
STOML3	NP_660329.1	515	3	5.53	TCF7	NP_001333354.1	1210	5	3.93
PROSER1	NP_733837.2	40	1	23.75	SKP1	NP_733779.1	2281	5	2.08
NHLRC3	NP_001012772.1	284	1	3.34	PPP2CA	NP_002706.1	2931	6	1.94
LHFPL6	NP_005771.1	304	6	18.75	CDKL3	XP_024301860.1	223	2	8.52
COG6	NP_001138551.1	504	4	7.54	UBE2B	NP_003328.1	1872	5	2.54
FOXO1	NP_002006.2	2104	1	0.45	CDKN2AIPNL	NP_542387.1	139	0	0.00
MRPS31	NP_005821.2	489	4	7.77	JADE2	NP_001276914.1	434	1	2.19
SLC25A15	NP_055067.1	307	1	3.09	SAR1B	NP_001028675.1	838	3	3.40
ELF1	NP_001138825.1	500	0	0.00	SEC24A	XP_016864450.1	739	2	2.57
WBP4	NP_009118.1	427	4	8.90	CAMLG	NP_001736.1	255	5	18.63
KBTBD6	NP_690867.3	441	0	0.00	DDX46	NP_001287789.1	1636	6	3.48
KBTBD7	NP_115514.2	445	0	0.00	C5orf24	NP_001287823.1	140	5	33.93
MTRF1	XP_016876349.1	570	6	10.00	TXNDC15	NP_078991.3	135	5	35.18
NAA16	NP_078837.3	258	4	14.73	PCBD2	NP_115527.3	276	5	17.21
RGCC	NP_054778.2	501	1	1.90	CATSPER3	NP_821138.1	337	8	22.55
VWA8	NP_055873.1	606	4	6.27	PITX1	NP_002644.4	757	6	7.53
DGKH	NP_821077.1	367	4	10.35	MACROH2A1	XP_005272189.1	1119	5	4.24
AKAP11	XP_016875870.1	327	2	5.81	DCANP1	NP_570900.1	49	2	38.77
TNFSF11	NP_003692.1	1555	3	1.83	TIFAB	NP_001092691.1	286	4	13.29
FAM216B	NP_872314.1	26	0	0.00	NEUROG1	NP_006152.2	741	1	1.28
EPSTI1	XP_005266653.1	265	0	0.00	CXCL14	NP_004878.3	487	4	7.80
DNAJC15	NP_037370.2	460	0	0.00	SLC25A48	NP_660325.4	279	1	3.40
ENOX1	NP_001334892.1	189	2	10.05	IL9	NP_000581.1	695	5	6.83
CCDC122	XP_016875888.1	130	2	14.61	LECT2	NP_002293.2	268	1	3.54
LACC1	NP_001337572.1	307	2	6.19	TGFBI	NP_000349.1	972	2	1.95
SMIM2	NP_076963.1	32	0	0.00	SMAD5	XP_016864959.1	1058	3	2.69
SERP2	NP_001333909.1	327	2	5.81	TRPC7	NP_001161048.1	255	0	0.00
TSC22D1	NP_001230728.1	430	4	8.84	SPOCK1	NP_004589.1	723	10	13.14
NUFIP1	NP_036477.2	367	0	0.00	KLHL3	NP_059111.2	642	10	14.80
GPALPP1	NP_061029.2	101	1	9.41	HNRNPA0	NP_006796.1	1484	9	5.76
GTF2F2	XP_011533354.1	1032	4	3.68	MYOT	NP_006781.1	418	9	20.45
KCTD4	NP_940686.2	241	0	0.00	PKD2L2	XP_016864832.1	173	9	49.42
TPT1	NP_003286.1	957	3	2.98	FAM13B	NP_001372926.1	177	12	64.40
SLC25A30	XP_016876013.1	219	2	8.68	WNT8A	XP_016865313.1	834	5	5.70
COG3	NP_113619.3	501	0	0.00	NME5	NP_003542.1	650	15	21.92
ERICH6B	XP_016875907.1	56	0	0.00	BRD8	NP_631938.2	1143	15	12.47
CBY2	XP_011533273.1	166	0	0.00	KIF20A	NP_005724.1	1595	11	6.55
SIAH3	NP_942146.2	326	1	2.91	CDC23	NP_004652.2	1617	7	4.11
ZC3H13	NP_001317495.1	842	4	4.51	GFRA3	NP_001487.2	260	14	51.15

CPB2	NP_001265470.1	631	1	1.51	CDC25C	XP_006714802.1	1512	6	3.77
LCP1	NP_002289.2	1489	2	1.28	FAM53C	XP_024301876.1	164	16	92.68
LRRC63	XP_016875911.1	370	0	0.00	KDM3B	NP_057688.3	655	13	18.85
RUBCNL	NP_001273691.1	331	2	5.74	REEP2	NP_001258732.1	1014	11	10.31
LRCH1	XP_016875972.1	696	2	2.73	EGR1	NP_001955.1	2239	6	2.55
ESD	NP_001975.1	701	11	14.91	ETF1	XP_016864710.1	1450	14	9.17
HTR2A	NP_000612.1	1008	5	4.71	HSPA9	NP_004125.3	2479	12	4.60
SUCLA2	NP_003841.1	1123	3	2.54	CTNNA1	NP_001310912.1	1160	12	9.83
NUDT15	NP_060753.1	229	8	33.19	LRRTM2	NP_056379.1	914	10	10.39
MED4	NP_054885.1	939	6	6.07	SIL1	XP_011541872.1	1492	9	5.73
ITM2B	NP_068839.1	464	10	20.47	MATR3	NP_001181885.1	1017	9	8.41
RB1	NP_000312.2	1418	8	5.36	PAIP2	XP_016865011.1	358	7	18.57
LPAR6	XP_024305072.1	437	14	30.43	SLC23A1	XP_011542068.1	492	5	9.65
RCBTB2	NP_001339358.1	605	12	18.84	MZB1	NP_057543.2	180	2	10.56
CYSLTR2	NP_001295397.1	381	14	34.91	PROB1	NP_001155018.1	56	2	33.93
FNDC3A	XP_016875929.1	686	13	18.00	SPATA24	XP_005271973.1	110	6	51.82
MLNR	NP_001498.1	372	15	38.30	DNAJC18	NP_689899.1	463	6	12.31
CDADC1	NP_001180407.1	235	17	68.72	ECSCR	NP_001280668.1	136	0	0.00
CAB39L	XP_016876274.1	588	18	29.08	STING1	NP_938023.1	444	1	2.14
SETDB2	NP_114121.2	1544	16	9.84	UBE2D2	NP_003330.1	1861	1	0.51
PHF11	NP_001035533.1	1585	19	11.39	CXXC5	NP_057547.5	287	0	0.00
RCBTB1	NP_001339429.1	740	14	17.97	PSD2	XP_011535998.1	1334	0	0.00
ARL11	NP_612459.1	1373	15	10.38	NRG2	NP_001171864.1	392	4	9.69
EBPL	NP_115954.1	232	14	57.32	PURA	NP_005850.1	577	6	9.88
KPNA3	NP_002258.2	966	15	14.75	IGIP	NP_001007190.1	80	4	47.50
SPRYD7	NP_001120954.1	156	14	85.25	CYSTM1	NP_115788.1	205	6	27.80
TRIM13	NP_001007279.1	512	13	24.12	PFDN1	NP_002613.2	800	5	5.94
KCNRG	NP_775876.1	324	13	38.12	HBEGF	NP_001936.1	1035	2	1.84
DLEU7	NP_945340.2	306	13	40.36	SLC4A9	XP_016865422.1	309	4	12.30
RNASEH2B	XP_011533531.1	460	10	20.65	ANKHD1-EIF4	NP_065741.3	1163	3	2.45
FAM124A	NP_659456.3	93	2	20.43	ANKHD1	NP_060217.1	1412	7	4.71
SERPINE3	NP_001094790.1	127	1	7.48	EIF4EBP3	NP_003723.1	203	4	18.72
INTS6	NP_036273.1	408	6	13.97	SRA1	NP_001030312.3	346	1	2.75
WDFY2	XP_011533216.1	1779	4	2.14	APBB3	NP_573420.2	174	1	5.46
DHRS12	NP_001257353.1	300	9	28.50	SLC35A4	NP_542401.1	370	4	10.27
CCDC70	NP_001333004.2	51	2	37.25	CD14	NP_001167575.1	679	1	1.40
ATP7B	NP_001230111.1	1034	7	6.43	TMCO6	XP_011535965.1	5	0	0.00
ALG11	NP_001004127.2	720	6	7.92	NDUFA2	NP_002479.1	770	1	1.23
UTP14C	NP_067677.4	727	2	2.61	IK	NP_006074.2	351	2	5.41
NEK5	NP_001352481.1	208	3	13.70	WDR55	NP_060176.2	451	7	14.74
NEK3	NP_002489.1	302	2	6.29	DND1	NP_919225.1	327	3	8.72
THSD1	NP_954872.1	198	2	9.60	HARS1	NP_001276023.1	1231	5	3.86
VPS36	NP_001269097.1	452	5	10.51	HARS2	NP_001350464.1	961	3	2.97
CKAP2	NP_001091995.1	596	8	12.75	ZMAT2	NP_653324.1	642	6	8.88
HNRNPA1L2	NP_001011724.1	1220	1	0.78	PCDHA@	NP_061723.1	332	7	20.03
SUGT1	NP_001124384.1	1500	4	2.53	PCDHA2	NP_061728.1	200	1	4.75
CNMD	XP_011533202.1	364	2	5.22	PCDHA3	NP_061729.1	196	1	4.85
PCDH8	NP_116567.1	906	3	3.15	PCDHA4	NP_061730.1	237	1	4.01
OLFM4	NP_006409.3	630	3	4.52	PCDHA5	NP_061731.1	174	0	0.00
PRR20A	NP_940843.1	80	4	47.50	PCDHA6	NP_061732.1	287	1	3.31
PRR20B	NP_001123876.1	20	0	0.00	PCDHA7	NP_061733.1	241	2	7.88
PRR20C	NP_001123877.1	70	3	40.71	PCDHA8	NP_061734.1	228	4	16.67
PRR20D	NP_001123878.1	70	3	40.71	PCDHA9	NP_114063.1	171	2	11.11
PRR20E	NP_001123879.1	80	4	47.50	PCDHA10	NP_061724.1	175	1	5.43
PCDH17	XP_005266414.1	705	10	13.47	PCDHA11	NP_061725.1	173	6	32.95
DIAPH3	NP_001245297.1	795	4	4.78	PCDHA12	NP_061726.1	149	7	44.63
TDRD3	XP_005266613.1	504	5	9.42	PCDHA13	NP_061727.1	194	6	29.38
PCDH20	NP_073754.2	330	5	14.39	PCDHAC1	NP_061721.2	219	8	34.70
PCDH9	NP_001305302.1	808	7	8.23	PCDHAC2	NP_061722.1	346	8	21.96
KLHL1	NP_001273654.1	786	8	9.67	PCDHB1	NP_037472.2	265	3	10.75
DACH1	NP_004383.4	623	1	1.52	PCDHB2	NP_061759.1	222	6	25.67
MZT1	NP_001065243.1	269	7	24.72	PCDHB3	NP_061760.2	242	8	31.40
BORA	NP_001273675.2	570	5	8.33	PCDHB4	NP_061761.1	316	5	15.03
DIS3	NP_055768.3	1154	5	4.12	PCDHB5	NP_056484.2	273	5	17.40
PIBF1	NP_001336584.1	206	6	27.67	PCDHB6	NP_061762.2	297	5	15.99
KLF5	NP_001721.2	963	3	2.96	PCDHB7	NP_061763.1	293	3	9.73

KLF12	NP_009180.3	507	6	11.24	PCDHB8	NP_061993.3	287	4	13.24
TBC1D4	XP_011533633.1	547	2	3.47	PCDHB16	NP_066008.2	259	3	11.00
COMMD6	NP_987091.1	312	4	12.18	PCDHB9	NP_061992.3	219	0	0.00
UCHL3	XP_011533514.1	764	5	6.22	PCDHB10	NP_061753.1	241	3	11.83
LMO7	NP_005349.3	1138	9	7.51	PCDHB11	NP_061754.1	219	3	13.01
KCTD12	NP_612453.1	342	6	16.67	PCDHB12	NP_061755.1	274	5	17.33
ACOD1	NP_001245335.1	449	2	4.23	PCDHB13	NP_061756.1	187	1	5.08
CLN5	NP_006484.2	285	5	16.67	PCDHB14	NP_061757.1	298	5	15.94
FBXL3	XP_016876027.1	951	5	4.99	PCDHB15	NP_061758.1	290	7	22.93
MYCBP2	XP_016875954.1	915	7	7.27	SLC25A2	NP_114153.1	160	8	47.50
SCEL	NP_659001.2	732	1	1.30	TAF7	NP_005633.2	900	2	2.11
LOC10012930	NP_001297069.1	5	0	0.00	PCDHG	NP_061735.1	217	3	13.13
SLAIN1	NP_001229797.1	176	1	5.40	PCDHGA2	NP_061738.1	222	10	42.79
EDNRB	NP_003982.1	1149	8	6.61	PCDHGA3	NP_061739.2	255	10	37.25
POU4F1	NP_006228.3	679	4	5.60	PCDHGB1	NP_061745.1	227	8	33.48
OBI1	NP_078822.3	70	4	54.28	PCDHGA4	NP_061740.2	216	8	35.18
RBM26	XP_016876186.1	363	1	2.62	PCDHGB2	NP_061746.1	214	7	31.07
NDFIP2	NP_061953.2	458	4	8.30	PCDHGA5	NP_061741.1	186	0	0.00
SPRY2	NP_005833.1	840	3	3.39	PCDHGB3	NP_061747.2	193	6	29.53
SLITRK1	NP_443142.1	1020	1	0.93	PCDHGA6	NP_061742.1	174	2	10.92
SLITRK6	NP_115605.2	422	4	9.00	PCDHGA7	NP_061743.1	158	6	36.07
SLITRK5	NP_001371538.1	366	3	7.79	PCDHGB4	NP_003727.1	336	3	8.48
GPC5	NP_004457.1	667	9	12.82	PCDHGA8	NP_114477.1	198	0	0.00
GPC6	NP_005699.1	689	11	15.17	PCDHGB5	NP_061748.1	155	7	42.90
DCT	NP_001309115.1	755	1	1.26	PCDHGA9	NP_061744.1	177	4	21.47
TGDS	NP_055120.1	418	14	31.82	PCDHGB6	NP_061749.1	219	0	0.00
GPR180	NP_851320.1	311	9	27.49	PCDHGA10	NP_061736.1	196	0	0.00
SOX21	NP_009015.1	697	10	13.63	PCDHGB7	NP_061750.1	263	6	21.67
ABCC4	NP_001288758.1	746	10	12.73	PCDHGA11	NP_061737.1	210	4	18.09
CLDN10	XP_016876332.1	328	14	40.55	PCDHGA12	NP_003726.1	243	6	23.46
DZIP1	NP_055749.1	327	18	52.29	PCDHGC3	NP_002579.2	296	5	16.05
DNAJC3	XP_011519406.1	874	13	14.13	PCDHGC4	NP_061751.1	489	3	5.83
UGGT2	NP_064506.3	469	17	34.43	PCDHGC5	NP_061752.1	565	6	10.09
HS6ST3	NP_703157.2	520	17	31.06	DIAPH1	NP_001073280.1	766	2	2.48
OXGR1	NP_001333123.1	437	14	30.43	HDAC3	NP_003874.2	2726	0	0.00
MBNL2	NP_001369612.1	593	17	27.23	RELL2	XP_011535927.1	75	0	0.00
RAP2A	NP_066361.1	1655	13	7.46	FCHSD1	XP_005268581.1	388	1	2.45
IPO5	XP_016876051.1	1390	11	7.52	ARAP3	XP_006714855.1	539	2	3.52
RNF113B	NP_849192.1	389	20	48.84	PCDH1	NP_115796.2	458	5	10.37
FARP1	NP_001273768.1	427	14	31.15	DELE1	XP_024302042.1	454	2	4.18
STK24	NP_001273578.1	875	8	8.69	PCDH12	XP_024301874.1	321	4	11.84
SLC15A1	NP_005064.1	589	6	9.68	RNF14	NP_001188294.1	682	3	4.18
DOCK9	XP_005254092.1	462	16	32.90	GNPDA1	XP_005268405.1	560	1	1.70
UBAC2	XP_011519384.1	280	16	54.28	NDFIP1	NP_085048.1	370	4	10.27
GPR18	XP_024305107.1	505	7	13.17	SPRY4	NP_112226.2	528	1	1.80
GPR183	XP_016875894.1	868	7	7.66	FGF1	NP_001341887.1	1478	5	3.21
TM9SF2	NP_004791.1	459	12	24.84	ARHGAP26	NP_055886.1	596	10	15.94
CLYBL	NP_996531.1	384	6	14.84	NR3C1	NP_001018661.1	2271	8	3.35
ZIC5	NP_149123.2	539	11	19.39	HMHB1	NP_067005.1	60	12	189.99
ZIC2	NP_009060.2	989	8	7.68	YIPF5	NP_110426.4	422	15	33.77
PCCA	NP_000273.2	509	6	11.20	KCTD16	NP_001357416.1	596	15	23.91
GGACT	NP_001182016.1	138	0	0.00	PRELID2	XP_016864617.1	89	14	149.43
TMTC4	NP_001337505.1	769	0	0.00	GRXCR2	XP_016865197.1	188	16	80.85
NALCN	XP_011519371.1	768	4	4.95	SH3RF2	XP_005268437.1	335	15	42.54
ITGBL1	NP_004782.1	550	2	3.45	PLAC8L1	NP_001025040.1	91	17	177.46
FGF14	NP_001308868.1	981	3	2.91	LARS1	NP_057544.2	1285	15	11.09
TPP2	XP_005254127.1	546	3	5.22	RBM27	XP_005268523.1	600	15	23.75
METTL21C	XP_016875896.1	200	0	0.00	POU4F3	NP_002691.1	590	11	17.71
CCDC168	XP_011519408.1	93	0	0.00	TCERG1	NP_001369477.1	1237	14	10.75
TEX30	XP_016876341.1	251	1	3.78	GPR151	NP_919227.2	315	13	39.20
POGLUT2	XP_005254132.1	164	5	28.96	PPP2R2B	NP_001258828.1	1559	12	7.31
BIVM	NP_060163.2	49	1	19.39	STK32A	XP_011535879.1	797	11	13.11
BIVM-ERCC5	NP_001191354.2	741	3	3.85	DPYSL3	NP_001184223.1	755	3	3.77
ERCC5	NP_000114.3	1124	4	3.38	JAKMIP2	NP_055605.2	253	8	30.04
SLC10A2	NP_000443.2	516	2	3.68	SPINK1	NP_001341895.1	374	5	12.70
DAOA	NP_758958.3	196	4	19.39	C5orf46	XP_016864948.1	77	0	0.00

EFNB2	NP_001358985.1	972	6	5.86	SCGB3A2	NP_473364.1	257	1	3.70
ARGLU1	NP_060481.3	497	6	11.47	SPINK5	NP_001121170.1	409	8	18.58
FAM155A	NP_001073865.1	258	5	18.41	SPINK14	XP_016864958.1	121	7	54.96
LIG4	NP_001339533.1	1530	7	4.35	SPINK6	NP_001182219.1	153	4	24.84
ABHD13	NP_116248.2	158	1	6.01	SPINK13	NP_001035218.1	216	6	26.39
TNFSF13B	NP_006564.1	1099	0	0.00	SPINK7	NP_115955.1	191	5	24.87
MYO16	NP_001185879.1	1008	3	2.83	SPINK9	XP_016865198.1	114	5	41.66
IRS2	NP_003740.2	1091	4	3.48	FBXO38	XP_024301991.1	199	4	19.09
COL4A1	NP_001836.3	1089	3	2.62	HTR4	NP_001035259.1	365	4	10.41
COL4A2	NP_001837.2	1032	8	7.36	ADRB2	NP_000015.2	1439	5	3.30
RAB20	NP_060287.1	812	4	4.68	SH3TC2	NP_078853.2	242	2	7.85
NAXD	NP_001229812.1	465	3	6.13	ABLIM3	NP_001287957.1	479	2	3.97
CARS2	NP_078813.1	852	5	5.57	AFAP1L1	NP_689619.1	133	6	42.85
ING1	NP_937860.1	708	4	5.37	GRPEL2	NP_689620.2	779	0	0.00
ANKRD10	NP_060134.2	915	6	6.23	PCYOX1L	NP_076933.3	325	3	8.77
ARHGEF7	XP_006720019.1	646	5	7.35	IL17B	XP_016864835.1	240	3	11.87
TEX29	XP_016875876.1	40	0	0.00	CSNK1A1	NP_001258670.1	1047	2	1.81
SOX1	NP_005977.2	904	2	2.10	ARHGEF37	XP_011535944.1	382	4	9.95
SPACA7	XP_011535768.1	30	0	0.00	PPARGC1B	XP_011535855.1	607	3	4.69
TUBGCP3	NP_001273206.1	717	4	5.30	PDE6A	XP_016865061.1	586	3	4.86
ATP11A	XP_005268356.1	1102	9	7.76	SLC26A2	NP_000103.2	606	8	12.54
MCF2L	XP_011535784.1	606	8	12.54	TIGD6	NP_112215.1	245	0	0.00
F7	XP_011535776.2	468	3	6.09	HMGXB3	NP_055798.3	74	2	25.67
F10	NP_000495.1	979	2	1.94	CSF1R	NP_001336665.1	1833	11	5.70
PROZ	XP_016876301.1	375	5	12.67	PDGFRB	NP_002600.1	2088	8	3.64
PCID2	NP_001307588.1	469	6	12.15	CDX1	NP_001795.2	735	7	9.05
CUL4A	NP_001008895.1	1450	10	6.55	SLC6A7	XP_016865256.1	1251	3	2.28
LAMP1	NP_005552.3	1782	6	3.20	CAMK2A	NP_001350919.1	1776	3	1.60
GRTP1	NP_001273662.1	737	11	14.18	ARSI	NP_001012301.1	383	8	19.84
ADPRHL1	NP_612439.2	265	6	21.51	TCOF1	XP_005268559.1	940	10	10.11
DCUN1D2	XP_016876138.1	365	13	33.83	CD74	NP_001351012.1	1203	14	11.06
TMCO3	NP_001336673.1	414	5	12.08	RPS14	NP_005608.1	1526	13	8.09
TFDP1	XP_016876206.1	740	9	12.84	NDST1	NP_001534.1	367	14	36.24
ATP4B	NP_000696.1	355	0	0.00	SYNPO	XP_016864496.1	569	11	18.36
GRK1	NP_002920.1	602	0	0.00	MYOZ3	NP_588612.2	220	7	30.23
TMEM255B	XP_016876047.1	74	4	68.46	RBM22	NP_060517.1	701	7	9.49
GAS6	NP_000811.1	658	8	16.50	DCTN4	NP_057305.1	569	10	16.70
RASA3	NP_031394.2	543	7	18.84	SMIM3	NP_116565.3	107	12	106.54
CDC16	NP_001072113.1	1300	6	7.31	IRGM	NP_001333486.1	488	4	7.79
UPF3A	NP_075387.1	526	2	6.57	ZNF300	NP_001166302.1	304	4	12.50
CHAMP1	NP_001157616.1	143	3	39.86	GPX3	NP_001316719.1	918	5	5.17
OR11H12	NP_001013372.1	77	2	49.35	TNIP1	XP_006714815.1	499	3	5.71
POTEM	NP_001138914.1	1026	7	11.78	ANXA6	NP_001350043.1	750	6	7.60
POTEG	NP_001005356.1	979	7	11.32	CCDC69	NP_056436.2	103	1	9.22
OR11H2	NP_001184216.1	37	0	0.00	GM2A	NP_001161079.1	442	2	4.30
OR4Q3	XP_024305386.1	172	2	15.78	SLC36A3	XP_011535929.1	217	1	4.38
OR4M1	XP_016876827.1	111	2	22.82	SLC36A2	XP_006714819.1	419	1	2.27
OR4N2	NP_001004723.1	107	2	22.20	SLC36A1	XP_011535897.2	562	3	5.07
OR4K2	NP_001005501.1	96	2	23.28	FAT2	XP_016864713.1	617	4	6.16
OR4K5	NP_001005483.1	136	2	15.52	SPARC	NP_001296373.1	1177	7	5.65
OR4K1	NP_001004063.2	131	2	15.27	ATOX1	NP_004036.1	500	1	1.90
OR4K15	NP_001005486.1	74	0	0.00	G3BP1	NP_938405.1	1164	2	1.63
OR4K14	NP_001004712.1	119	0	0.00	GLRA1	NP_000162.2	631	4	6.02
OR4K13	NP_001372958.1	83	0	0.00	NMUR2	NP_064552.3	831	2	2.29
OR4L1	NP_001004717.1	111	0	0.00	GRIA1	NP_000818.2	1786	2	1.06
OR4K17	NP_001004715.2	142	0	0.00	FAM114A2	NP_001304924.1	239	4	15.90
OR4N5	NP_001004724.1	181	0	0.00	MFAP3	NP_001229265.1	162	3	17.59
OR11G2	NP_001372962.1	152	0	0.00	GALNT10	NP_938080.1	345	3	8.26
OR11H6	NP_001004480.1	40	0	0.00	SAP30L	NP_078908.1	248	4	15.32
OR11H4	NP_001004479.2	36	0	0.00	HAND1	XP_005268588.1	315	0	0.00
TTC5	NP_612385.2	148	1	6.42	LARP1	NP_056130.2	654	3	4.36
CCNB1IP1	NP_067001.3	310	5	15.32	FAXDC2	XP_016864454.1	358	1	2.65
PARP2	NP_005475.2	845	3	3.37	CNOT8	XP_016865537.1	641	2	2.96
TEP1	NP_001305964.1	1090	1	0.87	GEMIN5	NP_001239085.1	421	0	0.00
KLHL33	NP_001352719.1	192	0	0.00	MRPL22	NP_054899.2	963	1	0.99
OSGEP	NP_060277.1	766	4	4.96	KIF4B	NP_001092763.1	894	0	0.00

APEX1	NP_001632.2	1639	4	2.32	SGCD	XP_016865212.1	566	1	1.68
PIP4P1	NP_653169.2	236	2	8.05	TIMD4	XP_016865510.1	432	4	8.80
PNP	NP_000261.2	703	0	0.00	HAVCR1	XP_016864828.1	466	4	8.15
RNASE10	NP_001373135.1	88	3	32.38	HAVCR2	NP_116171.3	691	4	5.50
RNASE9	NP_001103828.1	36	1	26.39	MED7	NP_001094286.1	1065	4	3.57
RNASE11	NP_660293.1	74	7	89.86	FAM71B	NP_570969.2	51	1	18.63
RNASE12	NP_001019993.1	16	5	296.86	ITK	NP_005537.3	1397	7	4.76
OR6S1	NP_001001968.1	38	0	0.00	CYFIP2	NP_001278651.1	862	3	3.31
ANG	NP_001091046.1	681	8	11.16	FNDC9	NP_001001343.2	189	0	0.00
RNASE4	NP_919412.1	140	6	40.71	NIPAL4	NP_001165763.1	249	0	0.00
EDDM3A	XP_016876423.1	49	0	0.00	ADAM19	NP_150377.1	470	5	10.11
EDDM3B	NP_071755.1	100	0	0.00	SOX30	NP_001295094.1	521	3	5.47
RNASE6	XP_016877055.1	523	8	14.53	C5orf52	XP_016864398.1	57	0	0.00
RNASE1	NP_937875.1	695	8	10.93	THG1L	NP_060342.2	249	10	38.15
RNASE3	NP_002926.2	681	8	11.16	LSM11	NP_775762.1	205	0	0.00
RNASE2	NP_002925.1	617	7	10.78	CLINT1	NP_055481.1	666	3	4.28
METTL17	NP_073571.1	320	0	0.00	EBF1	NP_874367.1	1085	5	4.38
SLC39A2	NP_055394.2	295	0	0.00	RNF145	NP_001186309.1	502	7	13.25
NDRG2	NP_001269140.1	565	3	5.04	UBLCP1	NP_659486.2	890	10	10.67
TPPP2	XP_011534718.1	260	3	10.96	IL12B	NP_002178.2	262	0	0.00
RNASE13	NP_001012264.1	94	9	90.95	ADRA1B	XP_006714884.1	888	0	0.00
RNASE7	NP_115961.3	168	1	5.65	TTC1	NP_003305.1	596	8	12.75
RNASE8	NP_612204.1	69	2	27.53	PWWP2A	XP_011532726.1	151	0	0.00
ARHGEF40	XP_011535239.1	270	5	17.59	FABP6	NP_001124430.1	399	2	4.76
ZNF219	XP_005267796.1	467	2	4.07	CCNJL	XP_016865336.1	985	2	1.93
TMEM253	NP_001140155.1	31	0	0.00	C1QTNF2	XP_011532728.1	140	4	27.14
OR5AU1	NP_001004731.2	23	0	0.00	ZBED8	NP_071373.2	72	0	0.00
HNRNPC	XP_016876741.1	2205	5	2.15	SLU7	NP_001351446.1	760	8	10.00
RPGRIP1	NP_065099.3	200	0	0.00	PTTG1	NP_004210.1	1025	9	8.34
SUPT16H	NP_009123.1	1564	8	4.86	ATP10B	XP_016864741.1	644	7	10.33
CHD8	NP_065971.2	1862	9	4.59	GABRB2	NP_000804.1	1288	8	5.90
RAB2B	NP_116235.2	1489	8	5.10	GABRA6	NP_000802.2	1074	2	1.77
TOX4	NP_055643.1	646	10	14.71	GABRA1	NP_001121115.1	1225	6	4.65
METTL3	XP_011535270.1	424	10	22.40	GABRG2	NP_001362276.1	1320	9	6.48
SALL2	NP_001278375.1	474	10	20.04	CCNG1	NP_001350949.1	1629	7	4.08
OR10G3	NP_001005465.1	69	6	82.60	NUDCD2	NP_660309.1	588	2	3.23
OR10G2	NP_001005466.2	62	6	91.93	HMMR	NP_001136028.1	837	4	4.54
OR4E2	NP_001001912.2	81	0	0.00	MAT2B	NP_877725.1	599	1	1.59
DAD1	NP_001335.1	689	6	8.27	TENM2	XP_016865149.1	586	5	8.11
ABHD4	NP_071343.2	394	4	9.64	WWC1	XP_011532787.1	740	1	1.28
OR6J1	NP_001335162.1	47	1	20.21	RARS1	NP_002878.2	1443	1	0.66
OXA1L	NP_005006.4	1226	2	1.55	FBLL1	NP_001342203.1	1440	3	1.98
SLC7A7	NP_003973.3	588	0	0.00	PANK3	NP_078870.1	287	0	0.00
MRPL52	NP_848026.1	341	5	13.93	SLIT3	NP_003053.2	1446	6	3.94
MMP14	NP_004986.1	1150	1	0.83	SPDL1	NP_001316570.1	555	3	5.13
LRP10	NP_001316155.1	340	2	5.59	DOCK2	NP_004937.1	979	9	8.73
REM2	XP_005267440.1	1282	2	1.48	INSYN2B	NP_001333233.1	83	1	11.45
RBM23	XP_016876900.1	505	1	1.88	FOXI1	NP_036320.2	676	7	9.84
PRMT5	NP_001269884.1	1648	7	4.03	C5orf58	XP_016864521.1	17	0	0.00
HAUS4	NP_001159742.1	208	7	31.97	LCP2	NP_005556.1	1106	3	2.58
AJUBA	NP_116265.1	814	5	5.84	KCNIP1	XP_016864896.1	1462	10	6.50
C14orf93	NP_001269899.1	70	5	67.85	KCNMB1	NP_004128.1	359	4	10.58
PSMB5	NP_002788.1	1262	7	5.27	GABRP	XP_024301780.1	372	10	25.54
PSMB11	NP_001093250.1	638	3	4.47	RANBP17	XP_011532929.1	233	11	44.85
CDH24	NP_659422.2	295	1	3.22	TLX3	NP_066305.2	556	9	15.38
ACIN1	NP_001158287.1	723	6	7.88	NPM1	NP_002511.1	2007	7	3.31
C14orf119	XP_016876879.1	142	1	6.69	FGF18	NP_003853.1	708	10	13.42
CEBPE	NP_001796.2	512	0	0.00	SMIM23	XP_011532925.1	45	0	0.00
SLC7A8	NP_036376.2	434	1	2.19	FBXW11	NP_001365909.1	997	9	8.58
RNF212B	NP_001357147.1	153	0	0.00	STK10	NP_005981.3	339	5	14.01
HOMEZ	NP_065885.2	184	0	0.00	EFCAB9	NP_001164654.1	870	3	3.28
PPP1R3E	NP_001263247.1	227	0	0.00	UBTD2	NP_689490.2	109	6	52.29
BCL2L2-PABPI	NP_001186793.2	608	1	1.56	SH3PXD2B	NP_001295104.1	1336	2	1.42
BCL2L2	NP_004041.2	989	3	2.88	NEURL1B	NP_001136123.1	730	1	1.30
PABPN1	NP_004634.1	1284	4	2.96	DUSP1	NP_004408.1	1595	3	1.79
SLC22A17	XP_005267805.1	737	4	5.16	ERGIC1	NP_001026881.1	650	3	4.38

EFS	NP_001264103.1	354	1	2.68	RPL26L1	XP_016865008.1	1225	3	2.33
IL25	NP_758525.1	165	0	0.00	ATP6V0E1	NP_003936.1	256	8	29.69
CMTM5	NP_001275675.1	583	2	3.26	CREBRF	NP_705835.2	185	4	20.54
MYH6	NP_002462.2	1887	1	0.50	BNIP1	NP_053582.2	464	8	16.38
MYH7	XP_016876829.1	1550	1	0.61	NKX2-5	NP_004378.1	1239	6	4.60
NGDN	NP_001036100.1	887	0	0.00	STC2	NP_003705.1	675	9	12.67
ZFHX2	XP_016877204.1	145	0	0.00	BOD1	NP_001153123.1	194	2	9.79
THTPA	NP_077304.1	162	0	0.00	CPEB4	NP_085130.2	439	7	15.15
AP1G2	NP_003908.1	547	0	0.00	C5orf47	XP_011532733.1	63	0	0.00
JPH4	NP_115828.2	843	3	3.38	NSG2	NP_057064.1	197	0	0.00
DHRS2	NP_878912.1	412	1	2.31	MSX2	NP_002440.2	976	6	5.84
DHRS4	NP_066284.2	523	3	5.45	DRD1	NP_000785.1	1503	7	4.42
DHRS4L2	NP_001180564.1	439	4	8.66	SFXN1	NP_001309906.1	387	3	7.36
DHRS4L1	XP_006720309.1	523	3	5.45	HRH2	XP_016864915.1	441	1	2.15
CARMIL3	NP_612369.3	172	3	16.57	CPLX2	XP_016864453.1	1000	1	0.95
CPNE6	XP_024305512.1	938	1	1.01	THOC3	XP_016865474.1	494	1	1.92
NRL	XP_011535108.2	646	1	1.47	FAM153B	NP_001252544.1	209	1	4.55
PCK2	NP_004554.3	1102	1	0.86	SIMC1	NP_001295124.1	132	5	35.98
DCAF11	NP_001156956.1	654	3	4.36	KIAA1191	XP_016865141.1	57	0	0.00
FITM1	NP_981947.1	186	5	25.54	ARL10	XP_011532831.1	1215	1	0.78
PSME1	NP_006254.1	536	5	8.86	NOP16	NP_001243469.2	703	5	6.76
EMC9	NP_057133.2	199	3	14.32	HIGD2A	NP_620175.1	265	5	17.92
PSME2	NP_002809.2	688	7	9.67	CLTB	NP_001825.1	754	1	1.26
RNF31	NP_060469.4	455	3	6.26	FAF2	NP_055428.1	880	6	6.48
IRF9	NP_001372329.1	627	4	6.06	RNF44	XP_006714895.1	361	1	2.63
REC8	XP_024305560.1	911	3	3.13	CDHR2	NP_001165447.1	291	2	6.53
IPO4	NP_078934.3	1220	3	2.34	GPRIN1	NP_443131.2	392	8	19.39
TM9SF1	NP_006396.2	243	1	3.91	SNCB	NP_001304963.1	1252	5	3.79
TSSK4	NP_001171668.1	1172	2	1.62	EIF4E1B	NP_001092878.1	1090	3	2.61
CHMP4A	NP_054888.3	566	2	3.36	TSPAN17	NP_036303.1	223	3	12.78
MDP1	NP_612485.2	47	1	20.21	UNC5A	XP_006714991.1	934	7	7.12
NEDD8-MDP1	NP_001186752.1	1705	0	0.00	HK3	NP_002106.2	1031	1	0.92
NEDD8	NP_006147.1	2535	5	1.87	UIMC1	XP_016865065.1	543	3	5.25
GMPR2	NP_001269951.1	893	2	2.13	ZNF346	NP_001295148.1	335	3	8.51
TINF2	NP_001350597.1	333	1	2.85	FGFR4	NP_001341913.1	940	4	4.04
TGM1	NP_000350.1	663	3	4.30	NSD1	XP_024301918.1	1976	6	2.88
RABGGTA	NP_878256.1	385	2	4.93	RAB24	NP_001026847.1	922	5	5.15
DHRS1	NP_612461.1	327	1	2.91	PRELID1	NP_037369.1	341	2	5.57
NOP9	XP_005267442.1	689	1	1.38	MXD3	NP_001136407.1	381	8	19.95
CIDEB	XP_016876710.1	426	5	11.15	LMAN2	NP_006807.1	476	1	2.00
LTB4R2	NP_001158164.1	398	6	14.32	RGS14	XP_024310096.1	552	5	8.60
LTB4R	NP_001137391.1	649	6	8.78	SLC34A1	XP_016865262.1	383	3	7.44
ADCY4	NP_640340.2	1200	6	4.75	PFN3	NP_001025057.1	116	3	24.57
RIPK3	NP_006862.2	1448	8	5.25	F12	NP_000496.2	692	1	1.37
NFATC4	NP_001306972.1	522	4	7.28	GRK6	XP_011532839.1	646	5	7.35
NYNRIN	NP_079357.2	283	9	30.21	PRR7	XP_016865385.1	141	6	40.42
KHNYN	XP_011534892.1	174	6	32.76	DBN1	XP_011532749.1	771	6	7.39
CBLN3	NP_001034860.1	332	0	0.00	PDLIM7	NP_976227.1	1011	5	4.70
SDR39U1	NP_064580.2	224	13	55.13	DOK3	XP_016865373.1	602	4	6.31
CMA1	NP_001827.1	595	6	9.58	DDX41	NP_057306.2	1730	7	3.84
CTSG	NP_001902.1	1139	7	5.84	FAM193B	XP_011532874.1	95	6	60.00
GZMH	NP_001257710.1	623	6	9.15	TMED9	NP_059980.2	510	6	11.18
GZMB	NP_004122.2	1341	7	4.96	B4GALT7	NP_009186.1	390	7	17.05
STXBP6	XP_016876721.1	687	11	15.21	FAM153A	XP_016864854.1	189	0	0.00
NOVA1	NP_001353321.1	815	3	3.50	PROP1	NP_006252.4	628	4	6.05
FOXG1	NP_005240.3	1525	2	1.25	N4BP3	XP_006714897.1	73	3	39.04
PRKD1	NP_002733.2	879	3	3.24	RMND5B	NP_001275724.1	317	4	11.99
G2E3	NP_060239.2	1671	7	3.98	NHP2	NP_060308.1	1760	3	1.62
SCFD1	NP_057190.2	710	7	9.37	HNRNPAB	NP_112556.2	1736	7	3.83
COCH	XP_016876560.1	472	5	10.06	PHYKPL	XP_011532976.1	326	4	11.66
STRN3	NP_055389.3	596	7	11.16	COL23A1	XP_011532993.1	283	2	6.71
AP4S1	NP_001121598.1	364	7	18.27	CLK4	NP_065717.1	797	6	7.15
HECTD1	XP_016876641.1	825	0	0.00	ZNF354A	XP_016865280.1	270	6	21.11
HEATR5A	NP_056288.2	281	3	10.14	ZNF354B	NP_478137.1	273	6	20.88
DTD2	NP_542395.1	261	9	32.76	ZFP2	NP_085116.2	230	0	0.00
GPR33	NP_001184113.2	280	7	23.75	ZNF454	NP_001310235.1	189	0	0.00

NUBPL	NP_079428.2	619	6	9.21	GRM6	NP_000834.2	711	2	2.67
ARHGAP5	XP_005267692.1	551	0	0.00	ZNF879	NP_001340301.1	157	0	0.00
AKAP6	XP_016877297.1	616	4	6.17	ZNF354C	XP_016864898.1	282	2	6.74
NPAS3	XP_016877071.1	675	7	9.85	ADAMTS2	NP_055059.2	542	3	5.26
EGLN3	NP_001295032.1	644	3	4.43	RUFY1	NP_079434.3	515	10	18.45
SPTSSA	NP_612145.2	173	1	5.49	HNRNPH1	NP_001351169.1	1105	6	5.16
EAPP	NP_060923.2	247	4	15.38	C5orf60	NP_001135778.1	0	0	#DIV/0!
SNX6	NP_001353448.1	570	7	11.67	CBY3	NP_001157916.1	318	0	0.00
CFL2	NP_619579.1	1250	1	0.76	CANX	NP_001350923.1	1999	9	4.28
BAZ1A	XP_011534676.1	1209	5	3.93	MAML1	NP_055572.1	411	2	4.62
SRP54	NP_003127.1	1730	6	3.29	LTC4S	NP_665874.1	293	5	16.21
FAM177A1	NP_775878.2	154	4	24.67	MGAT4B	NP_055090.1	298	4	12.75
PPP2R3C	XP_016876877.1	628	11	16.64	SQSTM1	NP_003891.1	1841	7	3.61
PRORP	XP_005268294.1	336	4	11.31	MRNIP	NP_001017987.1	45	1	21.11
PSMA6	NP_001269163.1	1810	8	4.20	TBC1D9B	NP_055858.2	621	11	16.83
NFKBIA	NP_065390.1	2212	3	1.29	RNF130	XP_011532895.1	663	9	12.90
INSM2	NP_115983.3	297	1	3.20	RASGEF1C	NP_778232.2	563	4	6.75
RALGAPA1	NP_919277.2	403	3	7.07	MAPK9	NP_001351537.1	1862	7	3.57
BRMS1L	XP_016877195.1	438	2	4.34	GFPT2	NP_005101.1	974	0	0.00
MBIP	XP_005267811.1	235	9	36.38	CNOT6	NP_001357401.1	691	8	11.00
NKX2-1	NP_001073136.1	1068	6	5.34	SCGB3A1	NP_443095.2	197	1	4.82
NKX2-8	NP_055175.2	381	6	14.96	FLT4	NP_891555.2	949	5	5.01
PAX9	NP_001359005.1	578	8	13.15	OR2Y1	NP_001001657.1	96	0	0.00
SLC25A21	NP_001164641.1	317	6	17.98	MGAT1	NP_001351324.1	397	0	0.00
MIPOL1	XP_016876495.1	294	7	22.62	ZFP62	XP_016865205.1	231	2	8.22
FOXA1	NP_004487.2	1609	5	2.95	BTNL8	NP_001035552.1	263	1	3.80
SSTR1	NP_001040.1	691	1	1.37	BTNL3	NP_932079.1	238	4	17.74
CLEC14A	NP_778230.1	360	1	2.64	BTNL9	XP_024310148.1	274	0	0.00
SEC23A	NP_006355.2	1078	5	4.41	OR2V1	NP_001245212.1	83	0	0.00
GEMIN2	XP_016877198.1	628	4	6.05	OR2V2	NP_996763.1	71	0	0.00
TRAPPC6B	NP_803235.1	424	7	15.68	TRIM7	XP_024301992.1	423	4	12.83
PNN	NP_002678.3	690	6	8.26	TRIM41	XP_006714994.1	635	3	6.90
MIA2	XP_024305360.1	143	3	19.93	RACK1	NP_006089.1	2178	1	0.73
FBXO33	NP_976046.1	234	4	16.24	TRIM52	XP_016865480.1	273	1	6.33
LRFN5	XP_016876537.1	1038	5	4.58	OR4F3	NP_001005224.1	39	0	0.00
FSCB	NP_115511.3	131	1	7.25	DUSP22	NP_064570.1	947	7	14.04
C14orf28	XP_024305236.1	62	5	76.61	IRF4	NP_002451.2	1125	9	13.82
KLHL28	NP_001295041.1	182	5	26.10	EXOC2	XP_016866507.1	854	9	16.69
TOGARAM1	NP_001295049.1	452	7	14.71	HUS1B	NP_683762.2	220	7	46.50
PRPF39	NP_060392.3	765	10	12.42	FOXQ1	NP_150285.3	564	10	24.06
FKBP3	NP_002004.1	1405	11	7.44	FOXF2	NP_001443.1	788	10	16.07
FANCM	XP_011535336.1	1004	8	7.57	FOXC1	NP_001444.2	1122	8	8.47
MIS18BP1	XP_016876914.1	407	5	11.67	GMDS	NP_001491.1	671	10	16.66
RPL10L	NP_542784.1	1101	2	1.73	MYLK4	NP_001334801.1	677	8	12.47
MDGA2	NP_001106970.3	390	1	2.44	WRNIP1	NP_064520.2	685	0	0.00
RPS29	NP_001025172.1	1332	6	4.28	SERPINB1	NP_109591.1	635	6	8.98
LRR1	NP_689542.2	1142	8	6.65	SERPINB9	NP_004146.1	587	6	9.71
RPL36AP42	NP_000992.1	796	4	4.77	SERPINB6	NP_001258751.1	608	5	7.81
MGAT2	NP_002399.1	214	0	0.00	NQO2	NP_000895.2	299	1	3.18
DNAAF2	NP_001077377.1	430	2	4.42	RIPK1	NP_001341859.1	1343	1	0.71
POLE2	NP_001184259.1	860	4	4.42	BPHL	NP_004323.2	284	3	10.03
KLHDC1	XP_024305239.1	528	7	12.59	TUBB2A	NP_001060.1	1686	4	2.25
KLHDC2	XP_006720157.1	745	6	7.65	TUBB2B	NP_821080.1	1648	4	2.31
NEMF	NP_001288661.2	740	7	8.99	PSMG4	XP_011512897.1	163	2	11.66
ARF6	NP_001654.1	2218	4	1.71	SLC22A23	NP_056297.1	197	6	28.93
VCPKMT	XP_016877130.1	189	2	10.05	PXDC1	NP_899229.2	139	3	20.50
SOS2	NP_008870.2	897	8	8.47	FAM50B	XP_016866218.1	180	3	15.83
L2HGDH	NP_079160.1	496	4	7.66	PRPF4B	XP_016866899.1	806	3	3.54
DMAC2L	XP_011534958.1	441	9	19.39	FAM217A	XP_024302124.1	46	0	0.00
CDKL1	XP_016877219.1	338	7	19.67	C6orf201	XP_016866350.1	45	1	21.11
MAP4K5	XP_011534679.1	325	9	26.31	ECI2	XP_006715020.1	1112	6	5.13
ATL1	NP_056999.2	442	3	6.45	CDYL	NP_001355054.1	1473	9	5.80
SAV1	NP_068590.1	333	2	5.71	RPP40	XP_024302077.1	377	11	27.72
NIN	NP_065972.4	473	4	8.03	LYRM4	XP_016866573.1	370	6	15.40
ABHD12B	NP_001193602.1	284	5	16.72	PPP1R3G	NP_001138587.1	173	12	65.89
PYGL	NP_001157412.1	909	5	5.23	FARS2	NP_001361808.1	597	9	14.32

TRIM9	XP_016876439.1	1109	4	3.43	NRN1	NP_001265639.1	471	5	10.08
TMX1	NP_110382.3	886	4	4.29	F13A1	NP_000120.2	859	7	7.74
FRMD6	XP_024305240.1	536	5	8.86	LY86	NP_004262.1	944	10	10.06
GNG2	XP_024305397.1	965	9	8.86	RREB1	NP_001003699.1	600	11	17.42
RTRAF	NP_057123.1	836	10	11.36	SSR1	NP_001278937.1	575	17	28.09
NID2	XP_005267464.1	699	12	16.31	CAGE1	NP_001164163.1	239	12	47.70
PTGDR	NP_000944.1	704	7	9.45	RIOK1	NP_113668.2	629	13	19.63
PTGER2	NP_000947.2	598	11	17.47	DSP	NP_004406.2	1142	2	1.66
TXNDC16	NP_001153519.1	608	12	18.75	SNRNP48	NP_689764.3	118	15	120.76
GPR137C	NP_001340290.1	129	11	81.00	BMP6	NP_001709.1	827	3	3.45
ERO1A	NP_001369403.1	584	11	17.89	TXNDC5	NP_110437.2	1010	6	5.64
PSMC6	NP_002797.4	2029	14	6.55	BLOC1S5	NP_958437.1	241	11	43.36
STYX	NP_001124173.1	652	11	16.03	EEF1E1	NP_001129122.1	1241	12	9.19
GNPNAT1	NP_932332.1	508	2	3.74	SLC35B3	XP_006715166.1	301	15	47.34
FERMT2	NP_006823.1	567	10	16.75	OFCC1	XP_016867101.1	194	13	63.66
DDHD1	XP_016877158.1	401	11	26.06	TFAP2A	XP_016866721.1	1041	8	7.30
BMP4	NP_001334841.1	2162	5	2.20	GCNT2	NP_001361676.1	451	9	18.96
CDKN3	NP_005183.2	1903	6	3.00	C6orf52	XP_011512876.1	50	0	0.00
CNIH1	NP_005767.1	584	8	13.01	PAK1IP1	XP_011513022.1	887	11	11.78
GMFB	NP_004115.1	491	5	9.67	TMEM14C	NP_001158730.1	424	11	24.64
CGRRF1	NP_006559.1	230	4	16.52	TMEM14B	NP_001273413.1	368	5	12.91
SAMD4A	XP_024305282.1	516	4	7.36	MAK	XP_011512923.1	411	4	9.25
GCH1	NP_001019242.1	814	0	0.00	GCM2	NP_004743.1	399	12	28.57
WDHD1	XP_006720075.1	881	4	4.31	SYCP2L	NP_001035364.2	137	13	90.14
SOCS4	NP_543143.1	433	2	4.39	ELOVL2	NP_060240.3	693	8	10.97
MAPK1IP1L	XP_011535664.2	153	3	18.63	SMIM13	NP_001129047.1	24	12	474.98
LGALS3	NP_001344607.1	1429	1	0.66	ERVFRD-1	NP_997465.1	151	3	18.87
DLGAP5	XP_016877329.1	951	3	3.00	NEDD9	NP_001135865.1	743	6	7.67
FBXO34	XP_016876880.1	120	2	15.83	TMEM170B	NP_001094299.1	67	9	127.61
ATG14	NP_055739.2	465	2	4.09	ADTRP	NP_116133.1	203	7	32.76
TBPL2	NP_950248.1	988	3	2.88	HIVEP1	NP_002105.3	584	5	8.13
KTN1	XP_016876767.1	605	5	7.85	EDN1	XP_016865820.1	1837	1	0.52
PELI2	NP_067078.1	352	1	2.70	PHACTR1	NP_001309239.1	528	1	1.80
TMEM260	XP_016876868.1	44	1	21.59	TBC1D7	NP_001137438.1	361	0	0.00
OTX2	NP_758840.1	1170	4	3.25	GFOD1	NP_061861.1	341	1	2.79
EXOC5	NP_006535.1	846	6	6.74	SIRT5	NP_001363738.1	1472	4	2.58
AP5M1	XP_011535242.1	505	7	13.17	NOL7	NP_057251.2	399	0	0.00
NAA30	NP_001011713.2	295	7	22.54	RANBP9	XP_011512507.1	482	1	1.97
CCDC198	NP_001269986.1	52	6	109.61	MCUR1	XP_011513104.1	301	0	0.00
SLC35F4	XP_011535025.1	133	6	42.85	RNF182	XP_016865939.1	583	2	3.26
ARMH4	XP_016876519.1	62	15	229.83	CD83	NP_004224.1	687	0	0.00
ACTR10	XP_011535262.1	1554	11	6.72	JARID2	XP_011512881.1	2223	5	2.14
PSMA3	NP_002779.1	1821	10	5.22	DTNBP1	XP_016866838.1	699	5	6.80
ARID4A	XP_016877049.1	1039	8	7.31	MYLIP	XP_016866278.1	612	3	4.66
TOMM20L	XP_011535045.1	452	2	4.20	GMPR	NP_006868.3	971	4	3.91
TIMM9	NP_001291419.1	683	11	15.30	ATXN1	NP_001121636.1	788	7	8.44
KIAA0586	NP_001231118.1	246	10	38.62	STMND1	NP_001177695.1	13	5	365.37
DACT1	NP_057735.2	547	6	10.42	RBM24	NP_001137414.1	693	3	4.11
DAAM1	NP_055807.1	1134	13	10.89	CAP2	NP_006357.1	1092	1	0.87
GPR135	XP_016877085.1	266	10	35.71	FAM8A1	NP_057339.1	152	7	43.75
L3HYPDH	NP_653182.1	105	3	27.14	NUP153	XP_016867083.1	1183	7	5.62
JKAMP	NP_057559.2	195	10	48.72	KIF13A	NP_001099038.1	880	8	8.64
CCDC175	XP_011535426.1	13	0	0.00	NHLRC1	NP_940988.2	506	6	11.26
RTN1	NP_066959.1	1460	11	7.16	TPMT	NP_001333747.1	371	4	10.24
LRRC9	NP_001342201.1	514	1	1.85	KDM1B	XP_016865929.1	732	9	11.68
PCNX4	NP_001317106.1	34	0	0.00	DEK	NP_001128181.1	1268	12	8.99
DHRS7	NP_001309211.1	353	5	13.46	RNF144B	NP_877434.2	541	1	1.76
PPM1A	NP_808821.2	956	7	6.96	ID4	NP_001537.1	602	7	11.05
C14orf39	XP_024305323.1	82	2	23.17	MBOAT1	NP_001073949.1	368	10	25.81
SIX6	NP_031400.2	657	4	5.78	E2F3	NP_001940.1	1237	8	6.14
SIX1	NP_005973.1	938	2	2.03	CDKAL1	NP_060244.2	886	8	8.58
SIX4	XP_005267816.1	431	2	4.41	SOX4	NP_003098.1	1092	6	5.22
MNAT1	XP_005267745.1	745	1	1.28	PRL	NP_001157030.1	1381	1	0.69
TRMT5	NP_001337182.1	395	3	7.21	HDGFL1	NP_612641.2	916	7	7.26
SLC38A6	NP_001166173.1	262	1	3.63	NRSN1	NP_542454.3	263	10	36.12
TMEM30B	NP_001017970.1	408	1	2.33	DCDC2	NP_057440.2	401	10	23.69

PRKCH	NP_006246.2	1227	8	6.19	KAAG1	NP_851854.1	54	10	175.92
HIF1A	NP_001521.1	2006	3	1.42	MRS2	NP_001273193.1	405	11	25.80
SNAPC1	NP_003073.1	278	6	20.50	GPLD1	XP_016866242.1	476	9	17.96
SYT16	NP_001354581.1	579	1	1.64	ALDH5A1	NP_733936.1	824	10	11.53
KCNH5	NP_647479.2	608	2	3.12	KIAA0319	XP_016867035.1	517	11	20.21
RHOJ	NP_065714.1	1788	5	2.66	TDP2	NP_057698.2	529	9	16.16
GPHB5	NP_660154.3	176	0	0.00	ACOT13	NP_060943.1	534	11	19.57
PPP2R5E	NP_001269109.1	1006	3	2.83	C6orf62	XP_011513230.1	109	11	95.87
WDR89	XP_011534685.1	231	0	0.00	GMNN	NP_001238918.1	1077	9	7.94
SGPP1	NP_110418.1	303	3	9.41	ARMH2	NP_001269421.1	0	0	#DIV/0!
SYNE2	XP_016876590.1	918	6	6.21	RIPOR2	XP_006715338.1	357	10	26.61
ESR2	XP_016876573.1	1071	1	0.89	CARMIL1	XP_016866497.1	349	6	16.33
MTHFD1	NP_001351766.1	911	2	2.09	SCGN	NP_008929.2	1407	6	4.05
ZBTB25	NP_001291437.1	372	3	7.66	H2AC1	NP_734466.1	927	7	7.17
AKAP5	NP_004848.3	468	3	6.09	H2BC1	NP_733759.1	1595	10	5.96
ZBTB1	NP_055765.2	508	4	7.48	SLC17A4	XP_024302062.1	213	6	26.76
HSPA2	NP_068814.2	1756	3	1.62	SLC17A1	XP_011513120.2	299	3	9.53
PPP1R36	NP_758953.1	31	0	0.00	SLC17A3	NP_006623.2	242	6	23.55
PLEKHG3	XP_011534929.1	189	7	35.18	SLC17A2	NP_005826.1	325	0	0.00
SPTB	XP_016877101.1	836	5	5.68	TRIM38	XP_024302071.1	482	17	33.50
CHURC1-FNTE	NP_001189488.1	309	5	15.37	H1-1	NP_005316.1	726	12	15.70
CHURC1	NP_660148.3	331	2	5.74	H3C1	NP_003520.1	1420	12	8.03
GPX2	NP_002074.2	663	3	4.30	H4C1	NP_003529.1	2039	13	6.06
RAB15	NP_001295083.1	493	0	0.00	H4C2	NP_003535.1	2048	14	6.49
FNTB	NP_002019.1	354	5	13.42	H3C2	NP_003528.1	1465	14	9.08
MAX	NP_001257998.1	530	3	5.38	H2AC4	NP_003504.2	825	17	19.57
FUT8	XP_016876625.1	409	4	9.29	H2BC3	NP_066406.1	1140	15	12.50
GPHN	XP_011534642.1	857	4	4.43	H3C3	NP_003522.1	1445	16	10.52
FAM71D	XP_011534802.1	61	1	15.57	H1-2	NP_005310.1	764	16	19.89
MPP5	NP_071919.2	839	3	3.40	HFE	NP_001371093.1	1899	20	10.00
ATP6V1D	NP_057078.1	712	2	2.67	H4C3	NP_003533.1	2059	17	7.84
EIF2S1	NP_004085.1	1689	3	1.69	H1-6	NP_005314.2	603	18	28.36
PLEK2	NP_057529.1	221	6	25.79	H2BC4	NP_001368918.1	896	15	15.90
TMEM229B	NP_001335470.1	65	0	0.00	H2AC6	NP_003503.1	1099	17	14.69
PLEKHH1	XP_011535311.1	218	2	8.72	H1-4	NP_005312.1	671	18	25.48
PIGH	NP_001350623.1	168	5	28.27	H2BC5	XP_005249096.1	1192	15	11.95
ARG2	NP_001163.1	876	3	3.25	H2BC6	NP_003514.2	896	15	15.90
VTI1B	NP_006361.1	695	6	8.20	H4C4	NP_003530.1	2053	16	7.40
RDH11	NP_001239579.1	532	5	8.93	H3C4	NP_001363866.1	1455	18	11.75
RDH12	NP_689656.2	567	4	6.70	H2AC7	NP_066409.1	961	18	17.79
ZFYVE26	XP_016876614.1	372	8	20.43	H2BC7	NP_003513.1	885	15	16.10
RAD51B	NP_001308747.1	1578	7	4.21	H4C5	NP_003536.1	2037	17	7.93
ZFP36L1	NP_001231630.1	860	4	4.42	H2BC8	NP_003509.1	876	14	15.18
ACTN1	NP_001123477.1	1461	7	4.55	H2AC8	NP_066390.1	770	17	20.97
DCAF5	NP_001271135.1	215	12	53.02	H3C6	NP_001368928.1	1421	15	10.03
EXD2	NP_001180289.1	333	12	34.23	H1-3	NP_005311.1	683	17	23.64
GALNT16	XP_016876987.1	372	10	25.54	H4C6	NP_003531.1	2039	12	5.59
ERH	NP_004441.1	941	10	10.10	H4C7	NP_003538.1	2111	11	4.95
SLC39A9	NP_001239079.1	224	11	46.65	H3C7	NP_066298.1	1425	11	7.33
PLEKHD1	NP_001154970.1	42	16	361.89	H2BC9	NP_003515.1	1110	10	8.56
CCDC177	NP_001258436.1	456	14	29.17	H3C8	NP_003525.1	1416	10	6.71
SUSD6	NP_055549.1	119	15	119.74	H2BC10	NP_003516.1	879	9	9.73
SRSF5	NP_001034554.1	1195	10	7.95	H4C8	NP_003534.1	2045	9	4.18
SLC10A1	NP_003040.1	445	2	4.27	BTN3A2	NP_001184177.1	244	0	0.00
SMOC1	XP_005268052.1	413	13	29.90	BTN2A2	NP_001184167.1	177	0	0.00
SLC8A3	XP_016877098.1	527	9	16.22	BTN3A1	NP_008979.3	211	0	0.00
SYNJ2BP-COX:	NP_001189478.1	158	11	66.14	BTN3A3	NP_008925.1	237	0	0.00
COX16	NP_001191019.1	469	13	26.33	BTN2A1	NP_001184163.1	205	0	0.00
SYNJ2BP	NP_060843.2	766	10	12.40	BTN1A1	NP_001723.2	235	1	4.04
ADAM21	NP_003804.2	243	10	39.09	HMGN4	XP_016865661.1	228	5	20.83
ADAM20	XP_005268208.1	232	2	8.19	ABT1	NP_037507.1	662	5	7.17
MED6	NP_001271140.1	468	0	0.00	ZNF322	NP_001229727.1	254	6	22.44
TTC9	NP_056166.1	238	3	11.97	H2BC11	NP_066402.2	1083	7	6.14
MAP3K9	XP_005267740.1	1107	5	4.29	H2AC11	NP_066408.1	899	6	6.34
PCNX1	XP_005267477.1	278	3	10.25	H4C9	NP_003486.1	2040	8	3.73
SIPA1L1	XP_016876686.1	550	4	6.91	H2BC12	NP_542160.1	1137	6	5.01

RGS6	XP_016877314.1	608	3	4.69	H2AC12	NP_542163.1	1040	11	10.05
DPF3	NP_001267471.1	417	4	9.11	PRSS16	NP_005856.1	241	8	31.53
DCAF4	XP_016876698.1	178	3	16.01	POM121L2	NP_258443.2	98	6	58.16
ZFYVE1	NP_001268663.1	751	3	3.79	ZNF391	NP_001309217.1	160	0	0.00
RBM25	NP_067062.1	1522	3	1.87	ZNF184	NP_009080.2	257	5	18.48
PSEN1	NP_000012.1	2115	2	0.90	H2BC13	NP_003510.1	1070	14	12.43
PAPLN	XP_011535597.1	552	0	0.00	H2AC13	NP_003500.1	900	11	11.61
NUMB	NP_001005745.1	1265	2	1.50	H3C10	NP_003527.1	1408	15	10.12
HEATR4	XP_024305348.1	84	1	11.31	H2AC14	NP_066544.1	971	12	11.74
RIOX1	NP_078920.2	257	0	0.00	H2BC14	NP_003512.1	1029	13	12.00
ACOT1	XP_016877079.1	413	3	6.90	H4C11	NP_068803.1	2039	14	6.52
ACOT2	NP_006812.3	461	2	4.12	H4C12	NP_003532.1	2049	13	6.03
ACOT4	NP_689544.3	350	1	2.71	H2AC15	NP_003501.1	893	13	13.83
ACOT6	NP_001352717.1	186	0	0.00	H2BC15	NP_003511.1	1115	14	11.93
DNAL1	NP_113615.2	645	1	1.47	H2AC16	NP_003502.1	877	13	14.08
PNMA1	NP_006020.4	175	0	0.00	H1-5	NP_005313.1	676	17	23.89
MIDEAS	NP_001354639.1	359	1	2.65	H3C11	NP_003524.1	1480	14	8.99
PTGR2	NP_001358254.1	324	7	20.52	H4C13	NP_003537.1	2115	11	4.94
ZNF410	NP_067011.1	356	4	10.67	H3C12	NP_003526.1	1563	15	9.12
FAM161B	XP_011534777.1	107	5	44.39	H2AC17	NP_003505.1	882	12	12.92
COQ6	NP_872286.2	697	3	4.09	H2BC17	NP_003518.2	1148	11	9.10
ENTPD5	NP_001369187.1	434	4	8.76	OR2B2	NP_149046.2	81	8	93.82
BBOF1	XP_011535474.1	79	3	36.07	OR2B6	NP_036499.1	75	8	101.33
ALDH6A1	NP_001265522.1	537	4	7.08	ZNF165	XP_016866751.1	297	2	6.40
LIN52	XP_011535623.1	242	7	27.48	ZSCAN16	NP_001307484.1	187	0	0.00
VSX2	NP_878314.1	649	2	2.93	ZKSCAN8	XP_016866755.1	197	4	19.29
ABCD4	XP_011535343.1	510	6	11.18	ZSCAN9	XP_011513177.1	165	2	11.51
VRTN	NP_060698.2	177	9	48.30	ZKSCAN4	XP_006715158.1	229	4	16.59
SYNDIG1L	XP_016877089.1	392	4	9.69	NKAPL	NP_001007532.1	222	4	17.12
NPC2	NP_001362369.1	617	1	1.54	ZSCAN26	NP_001018854.2	245	3	11.63
ISCA2	NP_919255.2	486	3	5.86	PGBD1	XP_016866850.1	310	9	27.58
LTBP2	XP_011535067.1	666	5	7.13	ZSCAN31	XP_024302289.1	220	0	0.00
AREL1	XP_006720407.1	922	10	10.30	ZKSCAN3	NP_077819.2	334	1	2.84
FCF1	NP_057046.1	663	7	10.03	ZSCAN12	XP_011513319.1	229	2	8.30
YLPM1	NP_062535.2	228	10	41.66	ZSCAN23	XP_016865968.1	202	1	4.70
PROX2	NP_001073877.2	284	11	36.79	GPX6	NP_874360.1	625	1	1.52
DLST	NP_001924.2	822	3	3.47	GPX5	NP_001500.1	575	0	0.00
RPS6KL1	NP_001357187.1	137	7	48.54	ZBED9	NP_443155.1	597	4	6.36
PGF	NP_001280572.1	755	1	1.26	TRIM27	NP_006501.1	710	8	10.70
EIF2B2	NP_055054.1	797	8	9.54	ZNF311	XP_016866247.1	200	9	42.75
MLH3	XP_005267590.1	735	5	6.46	OR2W1	NP_112165.1	94	2	20.21
ACYP1	NP_001289546.1	272	2	6.98	OR2B3	NP_001005226.1	76	3	37.50
ZC2HC1C	NP_078919.2	72	0	0.00	OR2J3	NP_001005216.2	100	3	28.50
NEK9	NP_149107.4	1012	4	3.75	OR2J2	NP_112167.2	76	3	37.50
TMED10	NP_006818.3	822	3	3.47	OR14J1	NP_112208.1	122	3	23.36
FOS	NP_005243.1	3252	8	2.34	OR5V1	XP_024302330.1	129	3	22.09
JDP2	XP_016876461.1	551	4	6.90	OR12D3	NP_112221.1	63	2	30.16
BATF	NP_006390.1	886	2	2.14	OR12D2	NP_039224.2	102	0	0.00
FLVCR2	NP_060261.2	269	1	3.53	OR11A1	NP_039225.1	69	2	27.53
ERG28	NP_009107.1	329	0	0.00	OR10C1	NP_039229.3	136	8	55.88
TTLL5	NP_055887.3	406	3	7.02	OR2H1	XP_016866226.1	90	7	73.89
TGFB3	NP_001316868.1	1044	6	5.46	MAS1L	NP_443199.1	175	8	43.43
IFT43	NP_001096034.1	472	4	8.05	UBD	NP_006389.2	2326	9	3.68
GPATCH2L	XP_016876917.1	267	3	10.67	OR2H2	XP_011513214.1	118	7	56.35
ESRRB	XP_011534856.1	765	3	3.73	GABBR1	XP_011512755.1	1084	7	6.13
VASH1	NP_055724.1	215	4	17.67	MOG	NP_996534.2	1005	13	12.29
ANGEL1	NP_001357677.1	301	7	22.09	ZFP57	NP_001103279.2	494	6	11.54
LRRC74A	XP_016876523.1	46	0	0.00	HLA-F	XP_011512866.1	415	13	29.76
IRF2BPL	NP_078772.1	357	3	7.98	HLA-G	NP_001350496.1	639	9	13.38
CIPC	NP_219494.2	119	0	0.00	HLA-A	NP_002107.3	1047	16	14.52
ZDHHC22	NP_001351101.1	601	1	1.58	POLR1H	NP_740753.1	928	13	13.31
TMEM63C	NP_065164.2	351	4	10.83	PPP1R11	XP_006715237.1	608	12	18.75
NGB	NP_067080.1	1052	5	4.51	RNF39	NP_739575.3	354	13	34.89
POMT2	XP_011534979.1	447	8	17.00	TRIM31	XP_011512567.1	594	14	22.39
GSTZ1	NP_665877.1	771	7	8.62	TRIM40	NP_001273562.1	387	11	27.00
TMED8	NP_998766.1	176	14	75.56	TRIM10	XP_011512527.1	479	14	27.76

SAMD15	NP_001010860.1	57	11	183.32	TRIM15	XP_011513289.1	494	4	7.69
NOXRED1	XP_011534731.1	274	11	38.14	TRIM26	XP_016866752.1	555	16	27.39
VIPAS39	NP_001180245.1	207	9	41.30	TRIM39	NP_067076.2	676	9	12.65
AHSA1	NP_036243.1	734	8	10.35	TRIM39-RPP2	NP_001186048.1	514	9	16.63
ISM2	NP_954993.1	146	11	71.57	RPP21	NP_001186049.1	373	9	22.92
SPTLC2	NP_004854.1	693	6	8.22	HLA-E	NP_005507.3	604	10	15.73
ALKBH1	NP_006011.2	455	6	12.53	GNL1	NP_005266.2	841	16	18.07
SLIRP	NP_112487.1	1516	8	5.01	PRR3	NP_079539.2	91	16	167.02
SNW1	NP_001305773.1	1509	8	5.04	ABCF1	NP_001020262.1	1008	11	10.37
ADCK1	NP_001353416.1	278	10	34.17	PPP1R10	XP_016866484.1	543	14	24.49
NRXN3	XP_016877279.1	1152	5	4.12	MRPS18B	XP_024302176.1	411	9	20.80
DIO2	NP_054644.1	512	1	1.86	ATAT1	NP_079185.2	236	5	20.13
CEP128	XP_011534795.1	252	5	18.85	C6orf136	NP_001154848.1	76	10	124.99
TSHR	XP_005268094.1	724	6	7.87	DHX16	NP_001157711.1	1381	16	11.01
GTF2A1	NP_056943.1	632	5	7.52	PPP1R18	NP_597728.1	264	14	50.38
STON2	XP_024305504.1	438	6	13.01	NRM	NP_001257638.1	270	9	31.67
SEL1L	XP_005268045.1	803	5	5.92	MDC1	XP_016867011.1	1011	9	8.46
FLRT2	XP_016876619.1	372	3	7.66	TUBB	NP_821133.1	2038	13	6.06
GALC	NP_001188331.1	479	11	21.82	FLOT1	XP_016865647.1	590	1	1.61
GPR65	NP_003599.2	786	11	13.29	IER3	NP_003888.2	489	13	25.25
KCNK10	NP_066984.1	359	10	26.46	DDR1	XP_011513184.1	488	2	3.89
SPATA7	XP_006720268.1	227	12	50.22	GTF2H4	NP_001508.1	805	9	10.62
PTPN21	NP_008970.2	747	13	16.53	VARS2	NP_001161206.1	925	12	12.32
ZC3H14	NP_079100.2	762	13	16.21	SFTA2	NP_995326.1	159	9	53.77
EML5	XP_016876554.1	668	14	19.91	MUCL3	NP_543146.2	403	10	23.57
TTC8	NP_653197.2	770	13	16.04	MUC21	NP_001010909.2	150	6	38.00
FOXN3	NP_005188.2	789	16	19.26	MUC22	NP_001305413.1	55	6	103.63
EFCAB11	NP_660274.1	1040	10	9.13	C6orf15	NP_054789.2	186	8	40.86
TDP1	XP_016876929.1	661	12	17.25	CDSN	NP_001255.4	508	15	28.05
KCNK13	NP_071337.2	194	16	78.35	PSORS1C1	NP_054787.2	157	15	90.76
PSMC1	NP_002793.2	1697	18	10.08	PSORS1C2	NP_054788.2	141	8	53.90
NRDE2	XP_016876883.1	297	6	19.19	CCHCR1	NP_001099033.1	312	11	33.49
CALM1	NP_008819.1	2523	6	2.26	TCF19	NP_001070979.1	528	16	28.79
TTC7B	NP_001010854.1	334	19	54.04	POU5F1	NP_002692.2	2074	8	3.66
RPS6KA5	NP_001309161.1	582	10	16.32	HLA-C	NP_002108.4	866	15	16.45
DGLUCY	NP_001345241.1	133	5	35.71	HLA-B	NP_005505.2	1004	17	16.08
GPR68	XP_005268167.1	394	11	26.52	MICA	NP_001170990.1	639	16	23.79
CCDC88C	NP_001073883.2	329	12	34.65	MICB	NP_005922.2	386	14	34.45
PPP4R3A	NP_001271210.1	789	15	18.06	MCCD1	NP_001011700.2	125	14	106.39
CATSPERB	NP_079040.2	215	16	70.69	DDX39B	NP_542165.1	1911	13	6.46
TC2N	NP_001276063.2	273	14	48.72	ATP6V1G2	NP_001191007.1	1175	14	11.32
FBLN5	NP_006320.2	606	6	9.41	NFKBIL1	NP_004998.3	411	16	36.98
TRIP11	NP_001308780.1	533	14	24.95	LTA	NP_001153212.1	1227	13	10.06
ATXN3	NP_001158251.1	898	6	6.35	TNF	NP_000585.2	4767	16	3.19
NDUFB1	NP_004536.3	280	1	3.39	LTB	NP_002332.1	820	13	15.06
CPSF2	NP_001309201.1	1041	10	9.13	LST1	NP_009092.3	317	13	38.96
SLC24A4	XP_011534738.1	513	11	20.37	NCR3	XP_011512761.1	593	13	20.83
RIN3	NP_079108.3	653	11	16.00	AIF1	NP_001614.3	1846	9	4.63
LGMN	NP_001350628.1	586	7	11.35	PRRC2A	NP_542417.2	530	20	35.85
GOLGA5	NP_005104.4	377	2	5.04	BAG6	XP_016866782.1	733	10	12.96
CHGA	NP_001266.1	1221	3	2.33	APOM	NP_061974.2	411	9	20.80
ITPK1	NP_001136066.1	300	12	38.00	C6orf47	NP_067007.3	106	13	116.50
MOAP1	NP_071434.2	202	6	28.22	GPANK1	XP_024302316.1	139	10	68.34
TMEM251	NP_001092091.2	74	2	25.67	CSNK2B	NP_001311.3	1909	12	5.97
GON7	NP_115879.2	56	0	0.00	LY6G5B	NP_067044.2	134	16	113.43
UBR7	NP_786924.2	291	1	3.26	LY6G5C	NP_079538.3	213	14	62.44
BTBD7	NP_001276062.1	236	6	24.15	ABHD16A	NP_066983.1	319	16	47.65
COX8C	NP_892016.1	191	6	29.84	LY6G6F	NP_001003693.1	161	8	47.20
UNC79	XP_011535323.1	721	3	3.95	LY6G6F-LY6G(	NP_001340263.1	118	5	40.25
PRIMA1	NP_821092.1	340	7	19.56	LY6G6D	NP_067069.2	204	9	41.91
FAM181A	NP_612353.3	254	7	26.18	LY6G6C	NP_079537.1	210	15	67.85
ASB2	XP_016876858.1	1429	7	4.65	MPIG6B	XP_011513222.1	173	13	71.38
OTUB2	NP_075601.1	351	7	18.94	DDAH2	NP_001289936.1	348	12	32.76
DDX24	NP_065147.1	1338	7	4.97	CLIC1	NP_001279.2	856	6	6.66
IFI27L1	XP_016876450.1	120	4	31.67	MSH5	NP_079535.4	675	12	16.89
IFI27	NP_001353923.1	418	5	11.36	SAPCD1	NP_001034740.1	85	6	67.06

IFI27L2	NP_114425.1	156	5	30.45	VWA7	NP_079534.2	374	12	30.48
PPP4R4	NP_478144.1	548	6	10.40	VARS1	NP_006286.1	2125	14	6.26
SERPINA10	XP_016876842.1	502	3	5.68	LSM2	NP_067000.1	1227	10	7.74
SERPINA6	NP_001747.3	436	3	6.54	HSPA1L	NP_005518.3	1848	6	3.08
SERPINA1	XP_016876859.1	1600	8	4.75	HSPA1A	NP_005336.3	2520	5	1.88
SERPINA11	NP_001073920.1	337	3	8.46	HSPA1B	NP_005337.2	1812	10	5.24
SERPINA9	NP_001035983.2	249	2	7.63	NEU1	NP_000425.1	321	7	20.72
SERPINA12	XP_011534757.1	364	2	5.22	SLC44A4	NP_001171515.1	371	16	40.97
SERPINA4	NP_001275961.1	853	2	2.23	EHMT2	NP_079532.5	2246	6	2.54
SERPINA5	NP_000615.3	719	8	10.57	ZBTB12	XP_011512685.2	429	17	37.64
SERPINA3	NP_001371602.1	705	2	2.69	C2	NP_001171534.1	433	11	24.13
GSC	NP_776248.1	477	1	1.99	CFB	NP_001701.2	873	16	17.41
DICER1	XP_016876609.1	3331	4	1.14	NELFE	XP_006715268.1	494	16	30.77
CLMN	NP_079010.2	476	0	0.00	SKIV2L	NP_008860.4	1297	16	11.72
SYNE3	XP_011534817.1	125	0	0.00	DXO	NP_005501.2	305	13	40.49
GLRX5	NP_057501.2	720	1	1.32	STK19	NP_115830.1	285	17	56.66
TCL1B	NP_004909.1	345	3	8.26	C4A	NP_009224.2	1010	8	7.52
TCL1A	XP_016877165.1	462	2	4.11	C4B	NP_001002029.3	1011	10	9.40
BDKRB2	NP_001366621.1	625	1	1.52	CYP21A2	NP_000491.4	557	15	25.58
BDKRB1	NP_001372936.1	577	3	4.94	TNXB	NP_061978.6	685	18	24.96
ATG2B	NP_060506.6	408	3	6.98	ATF6B	NP_001129625.1	448	18	38.17
GSKIP	NP_001258833.1	126	1	7.54	FKBPL	NP_071393.2	833	18	20.53
AK7	NP_689540.2	669	2	2.84	PRRT1	XP_024302328.1	572	15	24.91
PAPOLA	NP_116021.2	1221	2	1.56	PPT2	NP_619731.2	516	14	25.77
VRK1	XP_016877114.1	1278	4	2.97	EGFL8	NP_085155.1	258	12	44.18
BCL11B	NP_001269167.1	978	4	3.89	AGPAT1	NP_001358366.1	576	13	21.44
SETD3	XP_016877190.1	460	3	6.20	RNF5	NP_008844.1	579	10	16.41
CCNK	NP_001092872.1	857	3	3.33	AGER	NP_001193861.1	621	12	18.36
CCDC85C	NP_001138467.1	188	3	15.16	PBX2	NP_002577.2	555	14	23.96
HHIPL1	XP_006720340.1	160	3	17.81	GPSM3	NP_071390.1	622	17	25.96
CYP46A1	NP_006659.1	555	4	6.85	NOTCH4	NP_004548.3	873	20	21.76
EML1	XP_005267455.1	805	8	9.44	TSBP1	NP_001273404.1	229	12	49.78
EVL	NP_001317150.1	442	1	2.15	BTNL2	XP_016866546.1	327	16	46.48
DEGS2	NP_996801.2	303	5	15.68	HLA-DRA	NP_061984.2	1018	8	7.47
YY1	NP_003394.1	1809	10	5.25	HLA-DRB5	NP_002116.2	705	5	6.74
SLC25A29	NP_001034444.1	286	9	29.89	HLA-DRB1	NP_002115.2	1238	10	7.67
SLC25A47	NP_997000.2	183	5	25.95	HLA-DQA1	XP_006715142.1	1015	8	7.49
WARS1	XP_016877116.1	960	6	5.94	HLA-DQB1	NP_002114.3	992	9	8.62
WDR25	XP_005268114.1	642	11	16.28	HLA-DQA2	NP_064440.1	688	8	11.05
BEGAIN	NP_001372022.1	296	13	41.72	HLA-DQB2	XP_011512863.1	508	8	14.96
DLK1	NP_003827.4	983	7	6.76	HLA-DOB	NP_002111.1	445	8	17.08
RTL1	NP_001128360.1	343	7	19.39	TAP2	NP_061313.2	404	16	37.62
DIO3	NP_001353.4	364	9	23.49	PSMB8	NP_004150.1	1256	20	15.13
PPP2R5C	XP_005267881.1	1037	8	7.33	TAP1	NP_000584.3	446	14	29.82
DYNC1H1	NP_001367.2	1433	6	3.98	PSMB9	NP_002791.1	1019	20	18.64
HSP90AA1	XP_011535020.1	4266	8	1.78	HLA-DMB	NP_002109.2	547	7	12.16
WDR20	XP_011535641.1	681	3	4.18	HLA-DMA	NP_006111.2	624	8	12.18
MOK	XP_011535354.1	346	2	5.49	BRD2	NP_001186384.1	1689	20	11.25
ZNF839	XP_011535247.1	13	1	73.07	HLA-DOA	NP_002110.1	336	8	22.62
CINP	NP_116019.1	161	1	5.90	HLA-DPA1	NP_291032.2	709	8	10.72
TECPR2	NP_055659.2	237	0	0.00	HLA-DPB1	NP_002112.3	656	10	14.48
ANKRD9	NP_001335580.1	1047	3	2.72	COL11A2	NP_542410.2	664	18	25.75
RCOR1	NP_055971.2	914	2	2.08	RXRB	NP_001278918.1	609	16	24.96
TRAF3	XP_016877106.1	1123	4	3.38	SLC39A7	NP_008910.2	452	15	31.52
AMN	XP_024305482.1	222	2	8.56	HSD17B8	NP_055049.1	763	16	19.92
CDC42BPB	XP_005268286.1	1333	7	4.99	RING1	NP_002922.2	975	19	18.51
EXOC3L4	XP_011535635.1	526	2	3.61	VPS52	NP_072047.4	743	16	20.46
TNFAIP2	XP_011535417.1	856	4	4.44	RPS18	NP_072045.1	1636	17	9.87
EIF5	NP_001960.2	931	2	2.04	B3GALT4	NP_003773.1	238	10	39.91
MARK3	NP_001122390.2	463	0	0.00	WDR46	XP_016866973.1	972	18	17.59
CKB	NP_001814.2	728	2	2.61	PFDN6	NP_001172110.1	1023	18	16.71
TRMT61A	NP_689520.2	679	0	0.00	RGL2	NP_004752.1	401	18	42.64
BAG5	NP_001015049.1	362	2	5.25	TAPBP	XP_011513130.1	485	17	33.30
COA8	NP_001357524.1	142	1	6.69	ZBTB22	NP_005444.4	608	12	18.75
KLC1	NP_001123579.1	1098	6	5.19	DAXX	NP_001135442.1	1069	15	13.33
XRCC3	NP_001093588.1	1377	2	1.38	KIFC1	NP_002254.2	1429	13	8.64

ZFYVE21	NP_001185882.1	115	6	49.56	PHF1	NP_077084.2	509	11	20.53
PPP1R13B	XP_005267544.1	284	1	3.34	CUTA	NP_001014840.1	301	11	34.72
ATP5MPL	NP_001120865.1	365	0	0.00	SYNGAP1	NP_006763.2	1148	12	9.93
TDRD9	NP_694591.2	979	0	0.00	ZBTB9	NP_689948.1	244	10	38.93
RD3L	NP_001244197.1	27	3	105.55	BAK1	NP_001179.1	947	1	1.00
ASPG	XP_005267647.1	667	4	5.70	ITPR3	NP_002215.2	1320	4	2.88
KIF26A	NP_056471.1	906	8	8.39	UQCC2	NP_115716.1	388	3	7.34
C14orf180	XP_005267695.1	55	0	0.00	IP6K3	XP_024302091.1	824	3	3.46
TMEM179	NP_001273318.1	802	2	2.37	LEMD2	NP_001335639.1	185	2	10.27
INF2	NP_001026884.3	1109	9	7.71	MLN	NP_001171627.1	432	0	0.00
ADSS1	XP_006720089.1	822	6	6.93	GRM4	NP_001243738.1	1018	9	8.40
SIVA1	NP_006418.2	347	11	30.11	HMGA1	NP_665906.1	568	8	13.38
AKT1	NP_001369359.1	6506	9	1.31	SMIM29	NP_001274325.2	40	7	166.24
ZBTB42	NP_001357271.1	171	4	22.22	NUDT3	NP_006694.1	947	10	10.03
CEP170B	XP_005267607.1	241	5	19.71	RPS10-NUDT3	NP_001189399.1	734	12	15.53
PLD4	XP_011534713.1	466	10	20.39	RPS10	NP_001190174.1	1147	14	11.59
AHNAK2	NP_612429.2	351	4	10.83	PACSIN1	NP_065855.1	1105	13	11.18
CLBA1	XP_005267375.1	29	2	65.51	SPDEF	XP_005249045.2	482	12	23.65
CDCA4	NP_060425.2	356	7	18.68	ILRUN	NP_073595.2	242	14	54.96
GPR132	NP_001265624.1	572	7	11.63	SNRPC	NP_003084.1	1192	13	10.36
JAG2	NP_660142.1	1425	6	4.00	UHRF1BP1	NP_060224.3	286	14	46.50
NUDT14	NP_803877.2	517	14	27.08	TAF11	NP_005634.1	434	9	19.70
BRF1	NP_001229717.1	515	1	2.05	ANKS1A	XP_011512733.1	1009	16	15.06
BTBD6	XP_005268245.1	533	7	14.68	TCP11	NP_001353254.1	408	13	30.27
PACS2	XP_006720153.1	437	9	24.46	SCUBE3	XP_005249000.1	361	14	36.84
TEX22	NP_001182011.1	90	6	84.44	ZNF76	XP_016866737.1	346	11	30.20
MTA1	XP_016877246.1	666	8	16.30	DEF6	NP_071330.3	265	1	3.58
CRIP2	NP_001257766.1	568	9	23.16	PPARD	NP_001165290.1	922	8	8.24
CRIP1	NP_001302.1	506	2	6.26	FANCE	NP_068741.1	342	8	22.22
TEDC1	NP_001185912.1	9	0	0.00	RPL10A	NP_009035.3	1540	4	2.47
TMEM121	XP_006720324.1	106	7	125.47	TEAD3	NP_003205.2	617	7	10.78
GOLGA6L6	NP_001138476.2	119	8	127.72	TULP1	NP_001276324.1	416	6	13.70
POTEB2	NP_001264232.1	928	8	14.89	FKBP5	NP_001139247.1	1637	2	1.16
POTEB3	NP_997238.2	873	7	12.70	ARMC12	XP_011512683.1	204	6	27.94
POTEB	NP_001264233.1	928	9	14.17	CLPSL2	NP_001273479.1	0	0	#DIV/0!
OR4M2	NP_001004719.2	70	7	135.71	CLPSL1	NP_001335702.1	43	0	0.00
OR4N4	NP_001005241.2	89	9	128.08	CLPS	NP_001239527.1	479	3	5.95
GOLGA6L22	XP_011542184.1	99	0	0.00	LHFPL5	NP_872354.1	377	2	5.04
NIPA1	NP_653200.2	446	15	37.59	SRPK1	NP_003128.3	1026	8	7.41
NIPA2	XP_016878134.1	360	16	46.91	SLC26A8	NP_619732.2	268	8	28.36
CYFIP1	NP_001311048.1	1046	17	16.25	MAPK14	XP_016865788.1	3726	8	2.04
TUBGCP5	NP_001341303.1	302	18	56.62	MAPK13	NP_002745.1	2137	4	1.78
GOLGA6L1	NP_001001413.3	128	9	66.79	BRPF3	NP_056510.2	571	3	4.99
LOC10272362	NP_001369375.1	129	9	66.28	PNPLA1	XP_016866265.1	281	0	0.00
GOLGA8S	NP_001342394.1	66	7	100.75	BNIP5	XP_011512899.1	56	1	16.96
GOLGA6L2	NP_001291317.1	302	9	28.31	ETV7	NP_001193968.1	258	2	7.36
MKRN3	NP_005655.1	265	14	50.19	PXT1	XP_011512702.1	122	4	31.15
MAGEL2	NP_061939.3	395	15	36.07	KCTD20	NP_775833.2	275	4	13.82
NDN	NP_002478.1	520	16	29.23	STK38	XP_016865715.1	1955	7	3.40
NPAP1	NP_061831.2	166	15	85.84	SRSF3	NP_003008.1	1240	3	2.30
SNURF	NP_005669.2	1424	13	8.67	CDKN1A	NP_001361439.1	2279	2	0.83
UBE3A	NP_001341479.1	2038	14	6.53	RAB44	XP_024302203.1	724	2	2.62
ATP10A	XP_011520130.1	805	15	17.70	CPNE5	NP_001363818.1	343	1	2.77
GABRB3	NP_068712.1	1158	14	11.48	PPIL1	NP_057143.1	1062	2	1.79
GABRA5	NP_001158509.1	1001	17	16.13	C6orf89	NP_689947.2	130	0	0.00
GABRG3	XP_011519732.1	434	14	30.64	PI16	NP_001186088.1	257	0	0.00
OCA2	XP_016877753.1	805	16	18.88	MTCH1	NP_055156.1	347	3	8.21
HERC2	XP_006720790.1	1099	12	10.37	FGD2	XP_016865918.1	444	0	0.00
GOLGA8F	XP_024305576.1	86	5	55.23	PIM1	NP_001230115.1	1432	2	1.33
GOLGA8G	XP_024305673.1	80	5	59.37	TMEM217	XP_011512671.1	92	0	0.00
GOLGA8M	NP_001269397.1	67	3	42.54	TBC1D22B	NP_060242.2	379	2	5.01
APBA2	XP_024305677.1	854	14	15.57	RNF8	NP_898901.1	1095	1	0.87
FAM189A1	NP_056122.1	340	4	11.18	CMTR1	XP_005249012.1	146	0	0.00
NSMCE3	NP_619649.1	711	9	12.02	CCDC167	NP_612502.1	31	1	30.64
TJP1	XP_005254677.2	1746	4	2.18	MDGA1	NP_705691.1	410	3	6.95
GOLGA8J	NP_001269401.1	104	0	0.00	ZFAND3	NP_068762.1	313	3	9.10

GOLGA8T	NP_001342398.1	56	0	0.00	BTBD9	NP_443125.1	374	4	10.16
CHRFAM7A	NP_647536.1	302	9	28.31	GLO1	NP_006699.2	1294	5	3.67
GOLGA8R	NP_001269413.1	57	0	0.00	DNAH8	NP_001193856.1	795	11	13.14
GOLGA8Q	NP_001342405.1	56	0	0.00	GLP1R	NP_002053.3	631	7	10.54
GOLGA8H	NP_001269419.1	56	0	0.00	SAYSD1	NP_060792.1	39	7	170.50
ARHGAP11B	NP_001034930.1	631	9	13.55	KCNK5	XP_005249513.1	347	5	13.69
FAN1	XP_011519672.1	410	10	23.17	KCNK17	NP_113648.2	217	6	26.27
MTMR10	XP_011520039.1	152	12	75.00	KCNK16	XP_016866835.1	240	7	27.71
TRPM1	NP_001238949.1	465	12	24.51	KIF6	NP_001275949.1	1090	7	6.10
KLF13	NP_001289390.1	513	11	20.37	DAAM2	XP_016866119.1	1191	4	3.19
OTUD7A	NP_570971.1	610	9	14.02	MOCS1	NP_001345458.1	468	3	6.09
CHRNA7	NP_001177384.1	585	13	21.11	LRFN2	XP_016866599.1	1215	2	1.56
GOLGA8K	NP_001269422.1	68	1	13.97	UNC5CL	NP_775832.2	146	1	6.51
GOLGA8O	XP_011520290.1	63	0	0.00	TSPO2	NP_001153198.1	185	0	0.00
GOLGA8N	NP_001269423.1	58	0	0.00	APOBEC2	NP_006780.1	299	0	0.00
ARHGAP11A	NP_055598.1	772	12	14.77	OARD1	NP_001316617.1	162	0	0.00
SCG5	NP_001138229.1	769	12	14.82	NFYA	NP_002496.1	835	5	5.69
GREM1	NP_001355648.1	750	9	11.40	TREML1	XP_016866311.1	306	5	15.52
FMN1	XP_011519811.1	2079	5	2.28	TREM2	NP_001258750.1	593	7	11.21
RYR3	NP_001027.3	787	9	10.86	TREML2	XP_011513219.1	277	5	17.15
AVEN	XP_011520120.1	209	4	18.18	TREML4	NP_937796.1	231	3	12.34
CHRM5	NP_001307846.1	440	9	19.43	TREM1	XP_011512998.1	661	7	10.06
EMC7	NP_064539.1	463	3	6.16	NCR2	XP_016866989.1	374	3	7.62
PGBD4	NP_689808.2	103	1	9.22	FOXP4	XP_016865722.1	711	4	5.34
KATNBL1	XP_016878064.1	117	0	0.00	MDFI	NP_005577.1	307	0	0.00
EMC4	NP_001273349.1	441	2	4.31	TFEB	NP_001161299.2	700	3	4.07
SLC12A6	NP_001035962.1	471	5	10.08	PGC	NP_002621.1	854	5	5.56
NOP10	NP_061118.1	938	4	4.05	FRS3	NP_006644.1	352	7	18.89
NUTM1	NP_001271222.2	221	3	12.90	PRICKLE4	NP_037529.3	479	7	13.88
LPCAT4	NP_705841.2	266	6	21.43	TOMM6	NP_001369223.1	251	0	0.00
GOLGA8A	NP_001355000.1	146	5	32.53	USP49	NP_001371471.1	493	6	11.56
GOLGA8B	NP_001018861.3	132	3	21.59	MED20	NP_004266.2	301	6	18.94
GJD2	NP_065711.1	410	2	4.63	BYSL	NP_004044.3	1238	7	5.37
ACTC1	NP_005150.1	2170	4	1.75	CCND3	XP_011513273.1	918	5	5.17
AQR	NP_055506.1	892	4	4.26	TAF8	XP_016865730.1	609	1	1.56
ZNF770	NP_054825.2	174	1	5.46	C6orf132	NP_001157918.1	7	1	135.71
DPH6	XP_016878197.1	188	6	30.32	GUCA1A	NP_001371839.1	988	1	0.96
CDIN1	NP_001308690.1	67	6	85.07	GUCA1B	NP_002089.4	1035	1	0.92
MEIS2	NP_733776.1	771	5	6.16	MRPS10	XP_005249263.1	1168	3	2.44
TMCO5A	NP_001357665.1	130	6	43.84	TRERF1	XP_016866543.1	413	1	2.30
SPRED1	XP_005254259.1	537	7	12.38	UBR2	NP_056070.1	1317	0	0.00
FAM98B	NP_775882.2	174	6	32.76	PRPH2	NP_000313.2	456	2	4.17
RASGRP1	NP_001122074.1	991	7	6.71	TBCC	NP_003183.2	245	0	0.00
THBS1	NP_003237.2	1710	3	1.67	BICRAL	XP_024302157.1	46	0	0.00
FSIP1	NP_001311267.1	222	4	17.12	RPL7L1	NP_001353410.1	982	3	2.90
GPR176	NP_009154.1	450	3	6.33	C6orf226	NP_001008739.1	18	0	0.00
EIF2AK4	NP_001013725.2	1127	1	0.84	PTCRA	NP_001230098.1	740	0	0.00
SRP14	NP_003125.3	991	2	1.92	CNPY3	NP_001305771.1	232	4	16.38
BMF	NP_001003942.1	213	2	8.92	GNMT	NP_061833.1	509	2	3.73
BUB1B	NP_001202.5	1576	3	1.81	PEX6	NP_001303242.1	908	3	3.14
PAK6	NP_001122100.1	983	4	3.87	MEA1	XP_016866357.1	203	5	23.40
ANKRD63	NP_001177408.1	740	0	0.00	PPP2R5D	NP_006236.1	1077	6	5.29
PLCB2	XP_016877808.1	1241	7	5.36	KLHDC3	NP_476502.1	233	4	16.31
CCDC9B	NP_997263.2	58	0	0.00	RRP36	NP_149103.1	478	4	7.95
PHGR1	NP_001139115.1	130	0	0.00	CUL7	XP_005249560.1	687	1	1.38
DISP2	XP_016878176.1	354	2	5.37	MRPL2	NP_057034.2	1769	0	0.00
KNSTRN	NP_150628.3	478	2	3.97	KLC4	NP_958931.1	819	2	2.32
IVD	XP_016877638.1	631	1	1.51	PTK7	NP_002812.2	2032	3	1.40
BAHD1	XP_011519668.2	348	5	13.65	SRF	NP_003122.1	1719	1	0.55
CHST14	NP_569735.1	345	0	0.00	CUL9	XP_016866078.1	380	2	5.00
CCDC32	NP_001369368.1	232	1	4.09	DNPH1	NP_006434.1	229	0	0.00
RPUSD2	NP_689473.1	503	9	17.00	TTBK1	XP_016866853.1	1385	4	2.74
KNL1	NP_733468.3	680	7	9.78	SLC22A7	XP_016865687.1	467	3	6.10
RAD51	NP_597994.3	2586	6	2.20	CRIP3	NP_001352997.1	435	4	8.74
RMDN3	NP_001310823.1	290	6	19.65	ZNF318	NP_055160.2	146	0	0.00
GCHFR	NP_005249.1	197	5	24.11	ABCC10	XP_016866934.1	415	2	4.58

DNAJC17	NP_060633.1	619	11	16.88	DLK2	XP_011513125.1	256	0	0.00
C15orf62	NP_001123920.1	22	1	43.18	TJAP1	XP_016866978.1	248	0	0.00
ZFYVE19	NP_116239.3	649	7	10.25	LRRC73	NP_001012992.1	156	0	0.00
PPP1R14D	NP_001123615.1	131	0	0.00	YIPF3	NP_056203.2	262	1	3.63
SPINT1	NP_857593.1	525	2	3.62	POLR1C	NP_001350587.1	1625	1	0.58
RHOV	NP_598378.3	1622	0	0.00	XPO5	NP_065801.1	1892	2	1.00
VPS18	NP_065908.1	723	4	5.26	POLH	NP_006493.1	621	1	1.53
DLL4	NP_061947.1	884	3	3.22	GTPBP2	XP_024302244.1	298	3	9.56
CHAC1	XP_024305813.1	369	6	15.45	MAD2L1BP	NP_001003690.1	209	1	4.55
INO80	NP_060023.1	1192	3	2.39	RSPH9	XP_005248958.1	325	1	2.92
EXD1	NP_689809.2	250	0	0.00	MRPS18A	NP_060605.1	572	3	4.98
CHP1	NP_009167.1	1163	1	0.82	VEGFA	NP_001028928.1	3666	7	1.81
OIP5	NP_001304789.1	652	7	10.20	MRPL14	NP_001305698.1	450	1	2.11
NUSAP1	XP_006720622.1	647	3	4.40	TMEM63B	XP_016866488.1	262	5	18.13
NDUFAF1	NP_057097.2	424	5	11.20	CAPN11	XP_006715048.1	578	6	9.86
RTF1	NP_055953.3	1083	4	3.51	SLC29A1	XP_011512643.1	502	2	3.78
ITPKA	NP_002211.1	806	4	4.71	HSP90AB1	NP_001358167.1	2715	8	2.80
LTK	NP_001129157.1	776	1	1.22	SLC35B2	NP_835361.1	470	2	4.04
RPAP1	NP_056355.2	379	2	5.01	NFKBIE	NP_004547.3	1462	1	0.65
TYRO3	NP_006284.2	1407	3	2.03	TMEM151B	NP_001131032.1	785	1	1.21
MGA	XP_005254300.1	414	1	2.29	TCTE1	XP_024302115.1	596	8	12.75
MAPKBP1	NP_001122080.1	271	2	7.01	AARS2	XP_011513066.1	1018	6	5.60
JMJD7	NP_001108104.1	542	2	3.51	SPATS1	NP_001359010.1	44	0	0.00
JMJD7-PLA2G4	NP_005081.1	206	0	0.00	CDC5L	NP_001244.1	2281	10	4.16
PLA2G4B	NP_001108105.1	352	2	5.40	SUPT3H	NP_001337254.1	642	8	11.84
SPTBN5	XP_016877789.1	753	3	3.78	RUNX2	NP_001019801.3	1778	6	3.21
EHD4	NP_644670.1	458	0	0.00	CLIC5	NP_001357579.1	524	6	10.88
PLA2G4E	NP_001193599.1	403	2	4.71	ENPP4	NP_055751.1	286	5	16.61
PLA2G4D	NP_828848.3	338	1	2.81	ENPP5	NP_067547.1	336	6	16.96
PLA2G4F	NP_998765.3	366	4	10.38	RCAN2	XP_024302069.1	788	5	6.03
VPS39	NP_056104.2	920	2	2.07	CYP39A1	NP_001265667.1	383	3	7.44
TMEM87A	NP_056312.2	239	1	3.97	SLC25A27	NP_001190981.1	234	3	12.18
GANC	NP_937784.2	408	2	4.66	TDRD6	NP_001010870.1	920	5	5.16
CAPN3	NP_000061.1	1024	3	2.78	PLA2G7	NP_005075.3	564	6	10.11
ZNF106	NP_001368926.1	401	1	2.37	ANKRD66	XP_016865637.1	752	3	3.79
SNAP23	XP_006720788.1	830	6	6.87	MEP1A	XP_011512931.1	474	8	16.03
LRRC57	XP_011519725.1	544	9	15.72	ADGRF5	XP_016865911.1	445	5	10.67
HAUS2	NP_060567.1	328	6	17.38	ADGRF1	NP_722582.2	407	3	7.00
STARD9	XP_011520133.1	710	9	12.04	TNFRSF21	NP_055267.1	474	2	4.01
CDAN1	NP_612486.2	334	6	17.06	CD2AP	NP_036252.1	692	2	2.75
TTBK2	XP_006720465.1	836	8	9.09	ADGRF2	NP_001355044.1	299	4	12.71
UBR1	NP_777576.1	1268	9	6.74	ADGRF4	NP_001334784.1	340	4	11.18
TMEM62	NP_079232.3	29	0	0.00	OPN5	XP_016865905.1	200	1	4.75
CCNDBP1	XP_006720511.1	171	5	27.78	PTCHD4	NP_001371182.1	195	0	0.00
EPB42	XP_005254282.1	297	6	19.19	MMUT	NP_000246.2	689	2	2.76
TGM5	NP_004236.1	477	3	5.97	CENPQ	XP_011513025.2	326	2	5.83
TGM7	NP_443187.1	89	2	21.35	GLYATL3	NP_001010904.1	89	0	0.00
LCMT2	NP_055608.2	243	0	0.00	C6orf141	XP_016865785.1	115	2	16.52
ADAL	NP_001311295.1	497	1	1.91	RHAG	NP_000315.2	557	2	3.41
ZSCAN29	NP_689668.3	227	0	0.00	CRISP2	XP_011513145.1	434	1	2.19
TUBGCP4	NP_001273343.1	270	0	0.00	CRISP3	NP_006052.2	403	1	2.36
TP53BP1	XP_011520287.1	1715	3	1.66	PGK2	NP_620061.2	1625	6	3.51
MAP1A	NP_002364.5	832	1	1.14	CRISP1	NP_001122.2	283	4	13.43
PPIP5K1	NP_001341323.1	462	4	8.22	DEFB114	NP_001032588.1	111	1	8.56
CKMT1B	XP_011519496.1	798	5	5.95	DEFB113	NP_001032818.1	154	0	0.00
STRC	NP_714544.1	198	5	23.99	DEFB110	NP_001032817.1	120	2	15.83
CATSPER2	NP_001269239.1	447	5	10.63	DEFB112	NP_001355986.1	77	0	0.00
CKMT1A	NP_001308856.1	736	5	6.45	TFAP2D	NP_758438.2	193	6	29.53
PDIA3	NP_005304.3	1760	2	1.08	TFAP2B	XP_016866722.1	569	3	5.01
ELL3	NP_079441.1	269	1	3.53	PKHD1	XP_016866434.1	218	2	8.72
SERF2-C15ORI	NP_001186804.1	590	7	11.27	IL17A	NP_002181.1	1801	3	1.58
SERINC4	NP_001244960.1	44	4	86.36	IL17F	NP_443104.1	608	5	7.81
HYPK	NP_001186814.1	548	5	8.67	MCM3	NP_001353304.1	1707	6	3.34
MFAP1	NP_005917.2	969	6	5.88	PAQR8	NP_588608.1	243	9	35.18
WDR76	NP_079184.2	444	4	8.56	EFHC1	NP_060570.2	387	7	17.18
FRMD5	NP_001309880.1	347	5	13.69	TRAM2	NP_036420.1	300	10	31.67

GOLM2	NP_612432.2	173	1	5.49	TMEM14A	XP_024302177.1	222	10	42.79
CTDSPL2	NP_057480.2	351	0	0.00	GSTA2	NP_000837.3	442	4	8.60
EIF3J	NP_003749.2	633	4	6.00	GSTA1	NP_665683.1	494	2	3.85
SPG11	XP_006720763.1	421	5	11.28	GSTA5	NP_714543.1	296	4	12.84
PATL2	XP_011519648.2	501	4	7.58	GSTA3	NP_000838.3	335	8	22.69
B2M	NP_004039.1	2043	5	2.32	GSTA4	NP_001503.1	536	4	7.09
TRIM69	NP_892030.3	674	4	5.64	CILK1	NP_001362328.1	535	15	26.63
TERB2	NP_689661.1	49	1	19.39	FBXO9	NP_258441.1	662	7	10.04
SORD	NP_003095.2	1136	3	2.51	GCM1	XP_016866879.1	381	6	14.96
DUOX2	NP_001350640.1	500	5	9.50	ELOVL5	NP_001229759.1	724	10	13.12
DUOXA2	XP_016877669.1	229	3	12.44	GCLC	NP_001184044.1	779	11	13.41
DUOXA1	NP_001263194.1	228	3	12.50	KLHL31	XP_016866347.1	440	4	8.64
DUOX1	NP_059130.2	482	5	9.85	LRRC1	XP_011513028.1	784	10	12.12
SHF	NP_612365.3	284	1	3.34	MLIP	NP_612636.2	291	5	16.32
SLC28A2	NP_004203.2	317	4	11.99	TINAG	NP_055279.3	340	10	27.94
GATM	NP_001473.1	511	7	13.01	FAM83B	XP_016865967.1	161	2	11.80
SPATA5L1	NP_076968.2	998	1	0.95	HCRTR2	XP_016866287.1	353	0	0.00
C15orf48	NP_115789.1	332	1	2.86	GFRAL	NP_997293.2	121	1	7.85
SLC30A4	XP_016878049.1	360	6	15.83	HMGCLL1	NP_001274682.1	807	3	3.53
BLOC1S6	NP_036520.1	322	2	5.90	BMP5	NP_001316683.1	727	8	10.45
SQOR	NP_067022.1	564	4	6.74	COL21A1	NP_001305681.1	397	9	21.54
SEMA6D	XP_016878106.1	702	1	1.35	DST	NP_001138241.1	1188	4	3.20
SLC24A5	NP_995322.1	513	6	11.11	BEND6	XP_011512648.1	57	5	83.33
MYEF2	XP_005254481.2	593	4	6.41	KIAA1586	XP_011513069.1	162	4	23.46
CTXN2	XP_016877667.1	385	4	9.87	ZNF451	NP_001026794.1	456	6	12.50
SLC12A1	NP_001371065.1	542	4	7.01	BAG2	NP_004273.1	553	11	18.90
DUT	NP_001317215.1	1685	6	3.38	RAB23	NP_057361.3	570	5	8.33
FBN1	NP_000129.3	1364	8	5.57	PRIM2	XP_016866493.1	1000	10	9.50
CEP152	XP_011519680.1	568	1	1.67	MTRNR2L9	NP_001177635.1	58	1	16.38
SHC4	NP_976224.3	435	2	4.37	KHDRBS2	XP_016865833.1	668	10	14.22
EID1	NP_055150.1	209	1	4.55	LGSN	XP_016866418.1	1205	6	4.73
SECISBP2L	NP_055516.2	138	4	27.53	PTP4A1	NP_001372194.1	458	9	18.67
COPS2	NP_004227.1	1094	4	3.47	PHF3	XP_011533950.1	568	9	15.05
GALK2	XP_005254337.1	688	3	4.14	EYS	NP_001136272.1	794	6	7.18
FAM227B	XP_016877481.1	8	0	0.00	LOC441155	XP_016866378.1	438	0	0.00
FGF7	NP_002000.1	1093	2	1.74	ADGRB3	NP_001695.2	639	0	0.00
DTWD1	XP_016877914.1	106	8	71.69	LMBRD1	NP_060838.3	226	0	0.00
ATP8B4	XP_024305833.1	758	7	8.77	COL19A1	XP_011533739.1	335	5	14.18
SLC27A2	NP_003636.2	770	2	2.47	COL9A1	NP_001842.3	435	6	13.10
HDC	XP_016877586.1	654	3	4.36	FAM135A	NP_001317925.1	202	6	28.22
GABPB1	XP_024305656.1	986	1	0.96	SDHAF4	NP_660310.2	143	0	0.00
USP8	NP_005145.3	1066	4	3.56	SMAP1	NP_001037770.1	769	4	4.94
USP50	NP_987090.2	347	0	0.00	B3GAT2	NP_542780.1	424	3	6.72
TRPM7	NP_001288141.1	583	3	4.89	OGFRL1	XP_016866792.1	119	5	39.91
SPPL2A	NP_116191.2	262	6	21.75	RIMS1	NP_055804.2	1125	10	8.44
AP4E1	NP_031373.2	709	10	13.40	KCNQ5	NP_001153605.1	924	11	11.31
TNFAIP8L3	NP_997264.2	149	10	63.76	KHDC1L	NP_001119535.1	54	1	17.59
CYP19A1	NP_001334177.1	1363	6	4.18	KHDC1	NP_001238803.1	137	3	20.80
GLDN	XP_016877614.1	423	8	17.97	DPPA5	NP_001020461.1	215	6	26.51
DMXL2	NP_001365389.1	562	11	18.59	KHDC3L	NP_001017361.1	215	6	26.51
SCG3	NP_037375.2	733	7	9.07	OOEP	NP_001073976.1	161	8	47.20
LYSMD2	NP_699205.1	37	11	282.42	DDX43	NP_061135.2	1055	8	7.20
TMOD2	XP_016877578.1	520	8	14.61	CGAS	NP_612450.2	387	6	14.73
TMOD3	XP_016877576.1	465	12	24.51	MTO1	NP_598400.1	717	0	0.00
LEO1	NP_001273359.1	1182	3	2.41	EEF1A1	NP_001393.1	2366	5	2.01
MAPK6	NP_002739.1	2131	7	3.12	SLC17A5	NP_001369559.1	555	3	5.13
BCL2L10	NP_001293097.1	609	0	0.00	CD109	NP_598000.2	502	1	1.89
GNB5	NP_057278.2	1378	4	2.76	COL12A1	XP_011533738.1	810	5	5.86
MYO5C	XP_016877897.1	1208	7	5.50	COX7A2	XP_024302098.1	645	4	5.89
MYO5A	NP_001135967.2	1518	9	5.63	TMEM30A	NP_001137430.1	568	3	5.02
ARPP19	NP_006619.1	379	4	10.03	FILIP1	NP_001287795.1	206	6	27.67
FAM214A	NP_001371950.1	122	4	31.15	SENP6	NP_056386.2	682	6	8.36
ONECUT1	NP_004489.1	670	1	1.42	MYO6	XP_024302215.1	1311	5	3.62
WDR72	XP_011519739.1	518	2	3.67	IMPG1	NP_001269297.1	211	6	27.01
UNC13C	XP_016877709.1	749	3	3.80	HTR1B	NP_000854.1	690	2	2.75
RSL24D1	NP_057388.1	1299	4	2.93	MEI4	NP_001309176.1	67	5	70.89

RAB27A	XP_024305777.1	762	5	6.23	IRAK1BP1	XP_016865754.1	237	9	36.07
PIGBOS1	XP_024305583.1	0	0	#DIV/0!	PHIP	XP_005248786.1	1081	9	7.91
PIGB	NP_004846.4	200	1	4.75	HMGN3	NP_001305814.1	250	7	26.60
CCPG1	NP_001191380.1	225	0	0.00	LCA5	NP_859065.2	333	7	19.97
C15orf65	NP_001185713.1	16	0	0.00	SH3BGRL2	NP_113657.1	175	7	38.00
DNAAF4	NP_001028732.1	934	5	5.09	ELOVL4	NP_073563.1	722	7	9.21
PYGO1	NP_056432.1	289	1	3.29	TTK	NP_003309.2	1200	4	3.17
PRTG	NP_776175.2	384	2	4.95	BCKDHB	NP_898871.1	798	7	8.33
NEDD4	NP_006145.2	1772	3	1.61	TENT5A	NP_060103.2	433	3	6.58
RFX7	NP_001357483.1	541	2	3.51	IBTK	NP_001287835.1	589	4	6.45
TEX9	XP_016877649.1	124	1	7.66	TPBG	NP_001363851.1	720	0	0.00
MNS1	NP_060835.1	380	1	2.50	UBE3D	XP_016866947.1	362	9	23.62
ZNF280D	XP_016877833.1	385	3	7.40	DOP1A	XP_016866048.1	459	10	20.70
TCF12	XP_011520261.1	932	8	8.15	PGM3	NP_001186848.1	913	13	13.53
CGNL1	XP_016878174.1	370	1	2.57	RWDD2A	NP_219479.2	251	11	41.63
MYZAP	NP_001018110.1	99	2	19.19	ME1	NP_002386.1	704	2	2.70
GCOM1	NP_001272829.1	38	1	25.00	PRSS35	NP_699193.2	172	14	77.32
POLR2M	NP_056347.1	158	2	12.02	SNAP91	NP_001363669.1	1548	13	7.98
ALDH1A2	NP_733797.1	1091	4	3.48	RIPPLY2	NP_001009994.1	399	14	33.33
AQP9	NP_066190.2	992	5	4.79	CYB5R4	NP_057314.2	1327	16	11.45
LIPC	XP_024305684.1	657	5	7.23	MRAP2	XP_016865710.1	218	14	61.01
ADAM10	NP_001307499.1	1296	2	1.47	CEP162	NP_055710.2	312	18	54.80
MINDY2	XP_011519989.1	195	5	24.36	TBX18	XP_016866962.1	633	12	18.01
SLTM	XP_016878067.1	488	1	1.95	NT5E	NP_002517.1	1149	10	8.27
RNF111	XP_006720638.1	726	3	3.93	SNX14	NP_001337466.1	363	18	47.10
CCNB2	NP_004692.1	2222	0	0.00	SYNCRIP	NP_001153146.1	1252	15	11.38
MYO1E	NP_004989.2	1345	6	4.24	HTR1E	NP_000856.1	835	16	18.20
LDHAL6B	NP_149972.1	649	1	1.46	CGA	NP_000726.1	488	1	1.95
FAM81A	XP_016877420.1	182	4	20.88	ZNF292	NP_055836.1	533	16	28.52
GCNT3	NP_004742.1	348	1	2.73	GJB7	NP_940970.1	142	16	107.04
GTF2A2	NP_001307858.1	715	2	2.66	SMIM8	NP_001274374.1	85	9	100.58
BNIP2	NP_001354988.1	291	0	0.00	C6orf163	NP_001010868.2	43	13	287.19
FOXB1	NP_036314.2	597	3	4.77	CFAP206	NP_001026913.1	112	12	101.78
ANXA2	NP_001002858.1	1387	3	2.05	SLC35A1	NP_006407.1	498	6	11.45
ICE2	XP_016878058.1	333	4	11.41	RARS2	NP_001337434.1	768	12	14.84
RORA	NP_599023.1	938	5	5.06	ORC3	XP_016866121.1	970	15	14.69
VPS13C	NP_060154.3	661	4	5.75	AKIRIN2	NP_060534.1	289	14	46.02
C2CD4A	NP_997205.2	195	1	4.87	SPACA1	NP_112222.1	202	0	0.00
C2CD4B	XP_016877659.1	188	1	5.05	CNR1	NP_149421.2	1376	1	0.69
TLN2	XP_016878154.1	748	2	2.54	RNGTT	NP_001273355.1	804	10	11.82
TPM1	NP_001352707.1	1650	5	2.88	PNRC1	NP_006804.1	231	9	37.01
LACTB	NP_116246.2	398	4	9.55	SRSF12	NP_542781.3	1061	10	8.95
RPS27L	NP_057004.1	921	2	2.06	PM20D2	NP_001010853.1	144	11	72.57
RAB8B	NP_057614.1	856	5	5.55	GABRR1	NP_001243632.1	387	11	27.00
APH1B	NP_112591.2	278	0	0.00	GABRR2	NP_002034.3	285	8	26.67
CA12	NP_001280571.1	479	1	1.98	UBE2J1	XP_011534189.1	1540	11	6.79
USP3	NP_006528.2	668	3	4.27	RRAGD	NP_067067.1	554	11	18.86
FBXL22	NP_976307.2	752	2	2.53	ANKRD6	NP_001229738.1	1103	11	9.47
HERC1	NP_003913.3	838	4	4.53	LYRM2	NP_065199.1	174	0	0.00
DAPK2	NP_001371926.1	633	2	3.00	MDN1	NP_055426.1	793	11	13.18
CIAO2A	NP_115607.1	278	0	0.00	CASP8AP2	NP_001131140.1	390	5	12.18
SNX1	NP_001229862.1	797	3	3.58	GJA10	NP_115991.1	162	7	41.05
SNX22	NP_079074.2	234	1	4.06	BACH2	XP_024302278.1	656	6	8.69
PPIB	NP_000933.1	1433	1	0.66	MAP3K7	NP_003179.1	1604	7	4.15
CSNK1G1	NP_001316536.1	663	4	5.73	EPHA7	NP_001363395.1	1581	5	3.00
PCLAF	NP_001025160.1	834	1	1.14	MANEA	NP_078917.2	296	6	19.26
TRIP4	NP_057297.2	317	2	5.99	FUT9	XP_016865677.1	603	6	9.45
ZNF609	XP_016877510.1	190	0	0.00	UFL1	NP_056138.1	557	11	18.76
OAZ2	NP_001288231.1	196	0	0.00	FHL5	NP_001309395.1	689	1	1.38
RBPMS2	NP_919248.1	408	0	0.00	GPR63	XP_016866823.1	308	3	9.25
PIF1	NP_001273426.1	847	1	1.12	NDUFAF4	NP_054884.1	634	7	10.49
PLEKHO2	NP_079477.2	196	1	4.85	KLHL32	NP_001310181.1	257	4	14.79
ANKDD1A	NP_874362.3	1011	2	1.88	MMS22L	NP_940870.2	395	9	21.64
SPG21	NP_001121362.1	347	0	0.00	POU3F2	NP_005595.2	817	10	11.63
MTFMT	NP_640335.2	650	1	1.46	FBXL4	XP_016866216.1	873	11	11.97
RASL12	XP_005254491.1	1541	5	3.08	FAXC	NP_115900.1	137	8	55.47

SLC51B	NP_849190.2	177	0	0.00	COQ3	XP_005267075.1	928	9	9.21
KBTBD13	NP_001094832.1	454	5	10.46	PNISR	NP_001309345.1	537	12	21.23
UBAP1L	XP_011519849.1	26	0	0.00	USP45	NP_001332954.1	470	7	14.15
PDCD7	NP_005698.1	199	0	0.00	CCNC	NP_005181.2	1029	9	8.31
CLPX	XP_011519466.1	954	7	6.97	PRDM13	NP_067633.2	338	10	28.11
CILP	XP_016878167.1	364	0	0.00	MCHR2	NP_115892.2	574	10	16.55
PARP16	XP_006720653.1	189	6	30.16	SIM1	NP_001361698.1	649	5	7.32
IGDCC3	NP_004875.2	1021	2	1.86	ASCC3	NP_006819.2	1026	7	6.48
IGDCC4	NP_066013.1	402	7	16.54	GRIK2	NP_068775.1	1087	5	4.37
DPP8	XP_011520033.1	235	4	16.17	HACE1	NP_001337484.1	3276	7	2.03
HACD3	NP_057479.2	441	3	6.46	LIN28B	XP_006715540.2	769	5	6.18
INTS14	NP_001193987.2	275	10	34.54	BVES	NP_671488.1	276	1	3.44
SLC24A1	XP_011520524.1	341	0	0.00	POPDC3	XP_016866683.1	193	5	24.61
DENND4A	NP_001363848.1	284	4	13.38	PREP	NP_002717.3	398	0	0.00
RAB11A	NP_004654.1	2713	6	2.10	PRDM1	NP_001189.2	1062	6	5.37
MEGF11	XP_016878159.1	388	1	2.45	ATG5	NP_004840.1	1262	3	2.26
DIS3L	NP_001137160.1	851	8	8.93	CRYBG1	NP_001358171.1	154	0	0.00
TIPIN	XP_005254578.2	799	4	4.76	RTN4IP1	XP_011534494.1	423	8	17.97
MAP2K1	NP_002746.1	2539	6	2.24	QRSL1	NP_060762.3	812	8	9.36
SNAPC5	NP_001316542.1	165	5	28.79	CD24	NP_001346013.1	967	2	1.96
RPL4	NP_000959.2	1598	0	0.00	MTRES1	XP_016866409.1	138	9	61.95
ZWILCH	NP_060445.3	625	4	6.08	BEND3	NP_001073919.1	231	8	32.90
LCTL	NP_997221.2	296	4	12.84	PDSS2	XP_011534260.1	658	8	11.55
SMAD6	NP_005576.3	880	9	9.72	SOBP	XP_005267098.1	246	12	46.34
SMAD3	NP_005893.1	2176	7	3.06	SCML4	XP_016866174.1	191	6	29.84
AAGAB	NP_078942.3	151	3	18.87	SEC63	NP_009145.1	885	12	12.88
IQCH	NP_001026885.2	87	5	54.59	OSTM1	NP_054747.2	316	13	39.08
C15orf61	NP_001137408.1	28	4	135.71	NR2E1	NP_003260.1	641	8	11.86
MAP2K5	NP_660143.1	1306	5	3.64	SNX3	NP_001287858.1	623	12	18.30
SKOR1	NP_001352844.1	305	8	24.92	AFG1L	NP_660358.2	248	12	45.97
PIAS1	NP_057250.1	1095	5	4.34	FOXO3	NP_963853.1	2106	4	1.80
CALML4	NP_001026903.3	1056	3	2.70	ARMC2	XP_011534470.1	359	8	21.17
CLN6	NP_060352.1	236	2	8.05	SESN1	NP_055269.1	528	6	10.79
FEM1B	NP_056137.1	1057	3	2.70	CEP57L1	NP_001337583.1	73	11	143.14
ITGA11	XP_011519665.1	436	3	6.54	CD164	NP_001135876.1	456	6	12.50
CORO2B	NP_006082.3	1143	3	2.49	PPIL6	NP_001273289.1	894	10	10.63
ANP32A	NP_006296.1	808	0	0.00	SMPD2	XP_024302294.1	536	2	3.54
SPESP1	NP_663633.1	176	0	0.00	MICAL1	NP_001273542.1	623	6	9.15
NOX5	NP_001171709.1	300	0	0.00	ZBTB24	NP_055612.2	525	4	7.24
GLCE	XP_016877562.1	291	2	6.53	AK9	XP_011533856.1	746	0	0.00
PAQR5	XP_016877849.1	226	2	8.41	FIG4	NP_055660.1	662	4	5.74
KIF23	NP_001354734.1	1662	6	3.43	GPR6	NP_001273028.1	898	5	5.29
RPLP1	NP_000994.1	1159	1	0.82	WASF1	NP_003922.1	788	2	2.41
TLE3	XP_011520285.1	929	4	4.09	CDC40	NP_056975.1	879	9	9.73
UACA	XP_016877884.1	1007	2	1.89	METTL24	NP_001116836.1	30	4	126.66
LARP6	NP_060827.2	452	4	8.41	DDO	NP_001355102.1	554	5	8.57
LRRC49	XP_024305733.1	702	4	5.41	SLC22A16	XP_011534513.1	304	4	12.50
THAP10	NP_064532.1	62	1	15.32	CDK19	XP_011533933.1	780	0	0.00
THSD4	XP_006720755.1	453	2	4.19	AMD1	NP_001625.2	658	6	8.66
NR2E3	NP_055064.1	645	2	2.95	GTF3C6	NP_612417.1	198	7	33.58
MYO9A	XP_016877719.1	1389	7	4.79	RPF2	NP_115570.1	1099	7	6.05
SENP8	NP_660205.3	305	5	15.57	SLC16A10	NP_061063.2	324	5	14.66
GRAMD2A	XP_011519633.1	169	8	44.97	MFSD4B	NP_699200.2	249	6	22.89
PKM	XP_011519972.1	2157	4	1.76	REV3L	NP_002903.3	1073	7	6.20
PARP6	NP_001310455.1	167	9	51.19	TRAF3IP2	NP_671733.2	277	1	3.43
CELF6	NP_001166155.1	323	5	14.71	FYN	NP_694593.1	2378	3	1.20
HEXA	NP_000511.2	329	0	0.00	CCN6	XP_011534524.2	173	4	21.96
TMEM202	NP_001073931.1	29	5	163.78	TUBE1	NP_057346.1	1086	5	4.37
ARIH1	NP_005735.2	582	10	16.32	FAM229B	XP_016866663.1	22	0	0.00
GOLGA6B	NP_061122.4	130	0	0.00	LAMA4	XP_016866343.1	804	3	3.54
HIGD2B	NP_001337861.1	78	0	0.00	RFPL4B	NP_001013756.2	268	1	3.54
BBS4	NP_149017.2	1174	5	4.05	MARCKS	NP_002347.5	643	6	8.86
ADPGK	NP_001352157.1	424	8	17.92	HDAC2	NP_001518.3	3412	5	1.39
NEO1	XP_005254465.1	777	2	2.45	HS3ST5	XP_016865959.1	308	7	21.59
HCN4	NP_005468.1	649	0	0.00	FRK	XP_011533958.1	1653	4	2.30
REC114	NP_001035826.1	145	1	6.55	NT5DC1	NP_689942.2	126	3	22.62

NPTN	NP_001154836.1	475	3	6.00	COL10A1	XP_016865737.1	512	4	7.42
CD276	XP_016878127.1	720	3	3.96	TSPYL4	NP_067680.3	536	5	8.86
INSYN1	NP_001371280.1	66	0	0.00	TSPYL1	NP_003300.1	582	5	8.16
TBC1D21	XP_011519583.1	394	2	4.82	DSE	NP_001309868.1	369	5	12.87
LOXL1	NP_005567.2	488	5	9.73	CALHM6	NP_001263389.1	371	0	0.00
STOML1	NP_001243603.1	514	5	9.24	TRAPPC3L	NP_001132916.1	232	0	0.00
PML	NP_150241.2	947	0	0.00	CALHM5	NP_714922.1	27	0	0.00
GOLGA6A	NP_001033729.2	123	8	61.79	CALHM4	XP_016865879.1	119	0	0.00
ISLR2	XP_016877935.1	659	6	8.65	RWDD1	NP_057036.2	299	6	19.06
ISLR	NP_958934.1	799	7	8.32	RSPH4A	NP_001010892.1	316	4	12.02
STRA6	NP_001185969.1	419	4	9.07	ZUP1	NP_001348118.1	241	7	27.59
CCDC33	XP_011520387.1	186	9	45.97	KPNA5	NP_001353237.1	853	10	11.14
CYP11A1	NP_000772.2	744	5	6.38	FAM162B	NP_001078949.1	133	4	28.57
SEMA7A	NP_001139501.1	606	5	7.84	GPRC6A	NP_001273283.1	436	5	10.89
UBL7	NP_001273671.1	1843	10	5.15	RFX6	NP_775831.2	672	5	7.07
ARID3B	NP_001294868.1	488	11	21.41	VGLL2	NP_872586.1	315	1	3.02
CLK3	NP_003983.2	685	9	12.48	ROS1	XP_016866662.1	1127	5	4.21
EDC3	NP_001338307.1	667	3	4.27	DCBLD1	XP_011534075.1	119	3	23.95
CYP1A1	NP_001306145.1	1133	6	5.03	GOPC	NP_001017408.1	877	6	6.50
CYP1A2	NP_000752.2	664	5	7.15	NUS1	NP_612468.1	572	1	1.66
CSK	NP_001341917.1	1487	2	1.28	SLC35F1	NP_001025029.2	607	4	6.26
LMAN1L	NP_068591.2	470	7	14.15	CEP85L	XP_011534112.1	134	5	35.45
CPLX3	NP_001025176.1	607	6	9.39	PLN	NP_002658.1	366	1	2.60
ULK3	NP_001271293.2	629	8	12.08	MCM9	NP_001365293.1	834	1	1.14
SCAMP2	NP_005688.2	400	6	14.25	ASF1A	NP_054753.1	1411	2	1.35
MPI	NP_002426.1	658	5	7.22	FAM184A	NP_078857.5	101	1	9.41
FAM219B	NP_001308851.1	70	5	67.85	MAN1A1	XP_005267043.1	388	1	2.45
COX5A	NP_004246.2	950	6	6.00	TBC1D32	XP_016865889.1	249	1	3.82
RPP25	NP_060263.2	338	4	11.24	GJA1	NP_000156.1	1621	4	2.34
SCAMP5	XP_006720483.1	503	6	11.33	HSF2	NP_004497.1	788	2	2.41
PPCDC	XP_016877954.1	663	1	1.43	SERINC1	NP_065806.1	543	2	3.50
C15orf39	NP_056307.3	55	0	0.00	PKIB	NP_001257323.1	214	2	8.88
GOLGA6C	NP_001157876.1	55	0	0.00	FABP7	NP_001305971.1	722	6	7.89
GOLGA6D	NP_001138696.1	55	0	0.00	SMPDL3A	NP_006705.1	392	0	0.00
COMMD4	XP_024305736.1	383	3	7.44	CLVS2	NP_001010852.2	1064	4	3.57
NEIL1	NP_001243481.1	328	2	5.79	TRDN	NP_006064.2	523	4	7.27
MAN2C1	NP_001243425.1	443	5	10.72	NKAIN2	NP_001035304.1	807	5	5.89
SIN3A	XP_024305664.1	2374	1	0.40	RNF217	NP_001273327.1	432	3	6.60
PTPN9	NP_002824.1	677	6	8.42	TPD52L1	NP_001305832.1	292	3	9.76
SNUPN	NP_001036046.1	277	3	10.29	HDDC2	NP_057147.2	244	3	11.68
IMP3	NP_060755.1	1174	0	0.00	HEY2	NP_036391.1	615	2	3.09
SNX33	NP_695003.1	355	0	0.00	NCOA7	XP_016865758.1	387	5	12.27
CSPG4	NP_001888.2	796	2	2.39	HINT3	NP_612638.3	151	0	0.00
ODF3L1	XP_006720477.1	47	0	0.00	TRMT11	NP_001026882.2	551	4	6.90
UBE2Q2	XP_005254844.1	496	2	3.83	CENPW	NP_001273453.1	493	3	5.78
FBXO22	NP_671717.1	513	2	3.70	RSPO3	NP_116173.2	748	5	6.35
NRG4	XP_016877433.1	172	1	5.52	RNF146	XP_016866825.1	331	6	17.22
TMEM266	XP_016877404.1	48	3	59.37	ECHDC1	NP_001132982.1	724	5	6.56
ETFA	NP_001121188.1	980	5	4.85	KIAA0408	NP_055517.3	0	0	#DIV/0!
ISL2	NP_665804.1	719	10	13.21	SOGA3	NP_001012279.1	86	3	33.14
SCAPER	XP_011519958.1	192	6	29.69	C6orf58	NP_001010905.1	199	2	9.55
RCN2	NP_001258766.1	701	3	4.07	THEMIS	XP_016866338.1	231	5	20.56
PSTPIP1	NP_001308066.1	946	6	6.03	PTPRK	NP_001278913.1	980	6	5.82
TSPAN3	NP_001161884.1	336	9	25.45	LAMA2	XP_005267038.1	916	5	5.19
PEAK1	XP_011520342.1	184	4	20.65	ARHGAP18	NP_277050.2	302	4	12.58
HMG20A	NP_001291433.1	690	2	2.75	TMEM244	NP_001010876.1	83	5	57.23
LINGO1	NP_116197.4	1302	4	2.92	L3MBTL3	XP_006715639.1	345	2	5.51
TBC1D2B	XP_011519689.1	772	8	9.84	SAMD3	NP_001264114.1	164	3	17.38
SH2D7	XP_016877976.1	14	0	0.00	TMEM200A	XP_016865719.1	159	2	11.95
CIB2	NP_001258818.1	973	4	3.91	SMLR1	NP_001182526.1	80	0	0.00
IDH3A	NP_005521.1	1065	5	4.46	EPB41L2	NP_001129026.1	554	2	3.43
ACSBG1	XP_016877514.1	647	0	0.00	AKAP7	XP_016866998.1	345	4	11.01
DNAJA4	NP_061072.3	1116	7	5.96	ARG1	NP_001231367.1	1207	2	1.57
WDR61	NP_001290177.1	1047	4	3.63	MED23	NP_001257450.1	754	2	2.52
CRABP1	NP_004369.1	528	3	5.40	ENPP3	NP_005012.2	598	1	1.59
IREB2	NP_004127.2	1028	9	8.32	OR2A4	NP_112170.1	73	0	0.00

HYKK	NP_001077081.1	167	6	34.13	CTAGE9	NP_001139131.1	27	0	0.00
PSMA4	NP_001317605.1	1636	14	8.13	ENPP1	NP_006199.2	795	2	2.39
CHRNA5	NP_000736.2	724	7	9.18	CCN2	NP_001892.2	1494	4	2.54
CHRNA3	NP_001160166.1	882	7	7.54	MOXD1	XP_016866203.1	459	5	10.35
CHRNB4	NP_001243496.1	783	7	8.49	STX7	NP_001313507.1	912	13	13.54
ADAMTS7	NP_055087.2	385	9	22.21	TAAR9	NP_778227.3	310	4	12.26
MORF4L1	NP_996670.1	1276	4	2.98	TAAR8	NP_444508.1	339	4	11.21
CTSH	NP_004381.2	703	5	6.76	TAAR6	NP_778237.1	379	5	12.53
RASGRF1	NP_001139120.1	1323	3	2.15	TAAR5	NP_003958.2	409	5	11.61
ANKRD34C	NP_001139813.1	343	3	8.31	TAAR2	NP_001028252.1	329	4	11.55
TMED3	NP_001317305.1	462	6	12.34	TAAR1	NP_612200.1	373	4	10.19
MINAR1	XP_016877516.1	112	7	59.37	VNN1	NP_004657.2	672	9	12.72
MTHFS	NP_006432.1	223	0	0.00	VNN3	NP_001355081.1	386	7	17.23
ST20-MTHFS	NP_001186689.1	158	0	0.00	VNN2	NP_001229279.2	745	7	8.93
BCL2A1	NP_004040.1	1266	2	1.50	SLC18B1	NP_439896.1	98	0	0.00
ZFAND6	XP_024305723.1	373	5	12.73	RPS12	NP_001007.2	1559	7	4.27
FAH	NP_001361306.1	538	1	1.77	EYA4	XP_005266908.1	749	6	7.61
ARNT2	NP_055677.3	640	5	7.42	TCF21	NP_003197.2	596	7	11.16
ABHD17C	NP_067037.1	200	0	0.00	TBPL1	XP_016867002.1	1230	7	5.41
CEMIP	XP_024305769.1	314	4	12.10	SLC2A12	NP_660159.1	2471	6	2.31
MESD	NP_055969.1	282	4	13.47	SGK1	NP_001137148.1	1691	10	5.62
TLNRD1	NP_072088.1	336	4	11.31	ALDH8A1	NP_001180409.1	702	10	13.53
CFAP161	NP_775799.2	114	4	33.33	HBS1L	NP_001138630.1	1330	14	10.00
IL16	NP_001339615.1	1282	5	3.70	MYB	NP_001123645.1	1743	7	3.82
STARD5	NP_871629.1	157	1	6.05	AHI1	NP_001337433.1	929	12	12.27
TMC3	NP_001074001.1	139	7	47.84	PDE7B	NP_061818.1	568	12	20.07
MEX3B	NP_115622.2	280	2	6.79	MTFR2	NP_001092756.1	337	5	14.09
EFL1	NP_001035700.1	1475	2	1.29	BCLAF1	XP_016867020.1	902	8	8.43
SAXO2	NP_001335628.1	17	0	0.00	MAP7	NP_001185546.1	275	4	13.82
GOLGA6L10	NP_001157937.2	80	0	0.00	MAP3K5	XP_016866364.1	1133	6	5.03
GOLGA6L9	NP_937824.3	97	0	0.00	PEX7	NP_000279.1	555	6	10.27
RPS17	NP_001012.1	1003	3	2.84	SLC35D3	NP_001008783.1	319	3	8.93
CPEB1	NP_085097.3	572	1	1.66	IL20RA	NP_055247.3	318	9	26.89
AP3B2	NP_001265440.1	1019	6	5.59	IL22RA2	NP_851826.1	300	7	22.17
FSD2	XP_011519537.1	279	4	13.62	IFNGR1	NP_000407.1	731	4	5.20
WHAMM	NP_001073904.1	209	3	13.64	OLIG3	NP_786923.1	374	4	10.16
HOMER2	XP_005272506.1	514	4	7.39	TNFAIP3	XP_024302300.1	1154	7	5.76
C15orf40	XP_011519515.1	72	0	0.00	PERP	NP_071404.2	603	3	4.73
RAMAC	NP_113640.1	107	0	0.00	ARFGEF3	NP_065073.3	167	2	11.38
BTBD1	NP_079514.1	627	2	3.03	PBOV1	NP_067648.1	37	1	25.67
TM6SF1	XP_016877811.1	170	1	5.59	HEBP2	NP_001313309.1	249	0	0.00
HDGFL3	XP_006720617.1	971	5	4.89	NHSL1	NP_065197.1	129	2	14.73
BNC1	NP_001708.3	351	4	10.83	CCDC28A	NP_056254.2	130	3	21.92
SH3GL3	NP_003018.3	788	3	3.62	ECT2L	XP_016866317.1	740	2	2.57
ADAMTSL3	XP_016877923.1	411	2	4.62	REPS1	XP_005267235.1	306	1	3.10
GOLGA6L4	NP_001254465.2	33	0	0.00	ABRACL	XP_016866640.1	203	1	4.68
ZSCAN2	XP_024305746.1	301	5	15.78	HECA	NP_057301.1	269	1	3.53
WDR73	NP_116245.2	1314	5	3.61	TXLNB	XP_016865811.1	228	2	8.33
NMB	NP_995580.1	325	3	8.77	CITED2	NP_001161861.2	629	1	1.51
SEC11A	NP_001258851.1	730	4	5.21	NMBR	NP_002502.2	413	3	6.90
ZNF592	XP_016878223.1	520	7	12.79	GJE1	NP_001345339.1	71	3	40.14
ALPK3	NP_065829.4	223	6	25.56	VTA1	NP_057569.2	603	4	6.30
SLC28A1	XP_011520510.1	362	2	5.25	ADGRG6	XP_006715579.1	678	2	2.80
PDE8A	NP_002596.1	327	2	5.81	HIVEP2	XP_016866294.1	528	6	10.79
GOLGA6L3	NP_001297082.1	80	0	0.00	AIG1	NP_001353274.1	164	1	5.79
AKAP13	NP_009131.2	845	3	3.37	ADAT2	NP_872309.2	695	7	9.57
KLHL25	NP_071925.2	448	2	4.24	PEX3	NP_003621.1	478	7	13.91
AGBL1	NP_689549.3	183	2	10.38	FUCA2	NP_114409.2	460	10	20.65
NTRK3	NP_001362741.1	2066	1	0.46	PHACTR2	NP_055536.2	478	8	15.90
MRPL46	NP_071446.2	614	1	1.55	LTV1	NP_116249.2	822	7	8.09
MRPS11	NP_073750.2	1030	1	0.92	ZC2HC1B	NP_001013645.1	62	9	137.90
DET1	NP_001308523.1	540	1	1.76	PLAGL1	NP_001074420.1	562	4	6.76
AEN	XP_016877978.1	671	2	2.83	SF3B5	NP_112577.1	1159	9	7.38
ISG20	XP_005254956.1	800	2	2.37	STX11	XP_011534515.1	921	9	9.28
ACAN	NP_001356197.1	1108	2	1.71	UTRN	NP_009055.2	1107	5	4.29
HAPLN3	XP_011519563.1	271	4	14.02	EPM2A	XP_024302318.1	839	6	6.79

MFGE8	NP_001297248.1	587	1	1.62	FBXO30	XP_016866842.1	351	3	8.12
ABHD2	XP_024305597.1	434	3	6.57	SHPRH	XP_006715502.1	1256	5	3.78
RLBP1	NP_000317.1	527	3	5.41	GRM1	XP_016866272.1	1404	8	5.41
FANCI	NP_001106849.1	733	3	3.89	RAB32	NP_006825.1	584	7	11.39
POLG	NP_001119603.1	934	5	5.09	ADGB	NP_078970.3	493	3	5.78
RHCG	NP_001307970.1	341	6	16.71	STXBP5	NP_001121187.1	674	7	9.87
TICRR	NP_689472.3	368	4	10.33	SAMD5	XP_016866339.1	318	8	23.90
KIF7	XP_011519833.1	982	2	1.93	SASH1	XP_016866088.1	545	5	8.72
PLIN1	NP_002657.3	618	6	9.22	UST	NP_005706.1	260	3	10.96
PEX11A	NP_001258501.1	444	1	2.14	TAB2	NP_001278964.1	851	4	4.47
WDR93	XP_006720677.1	65	4	58.46	SUMO4	NP_001002255.1	1803	5	2.63
MESP1	NP_061140.1	275	1	3.45	ZC3H12D	NP_997243.2	292	0	0.00
MESP2	NP_001035047.1	459	3	6.21	PPIL4	NP_624311.1	968	4	3.93
ANPEP	NP_001141.2	1219	1	0.78	GINM1	NP_620140.1	36	0	0.00
AP3S2	NP_005820.1	402	2	4.73	KATNA1	XP_005266862.1	971	6	5.87
ARPIN-AP3S2	NP_001185987.1	273	0	0.00	LATS1	NP_004681.1	745	5	6.38
ARPIN	NP_872422.1	89	4	42.69	NUP43	NP_942590.1	693	3	4.11
ZNF710	NP_940928.2	290	0	0.00	PCMT1	NP_001238978.1	679	10	13.99
IDH2	NP_002159.2	1310	1	0.73	LRP11	XP_011534496.1	244	11	42.83
SEMA4B	NP_001310960.1	507	1	1.87	RAET1E	XP_016865774.1	170	6	33.53
CIB1	NP_006375.2	1270	1	0.75	RAET1G	NP_001001788.2	127	2	14.96
GDPGP1	NP_001309740.1	170	1	5.59	ULBP2	XP_016866810.1	297	4	12.79
NGRN	NP_001028260.2	177	0	0.00	ULBP1	XP_016866811.1	189	5	25.13
ZNF774	NP_001004309.2	125	0	0.00	RAET1L	NP_570970.2	158	1	6.01
IQGAP1	NP_003861.1	1323	1	0.72	ULBP3	NP_078794.1	165	5	28.79
CRTC3	NP_073606.3	309	0	0.00	PPP1R14C	NP_112211.1	247	13	50.00
BLM	NP_000048.1	1122	1	0.85	IYD	NP_981932.1	622	9	13.75
FURIN	NP_001369549.1	1811	3	1.57	PLEKHG1	NP_001316728.1	245	14	54.28
FES	XP_016877494.1	1574	2	1.21	MTHFD1L	XP_016866191.1	828	10	11.47
MAN2A2	NP_001307906.1	361	7	18.42	AKAP12	XP_016867006.1	515	10	18.45
HDDC3	NP_001273380.1	559	8	13.59	ZBTB2	NP_065912.1	283	13	43.64
UNC45A	NP_001310548.1	634	6	8.99	RMND1	NP_060379.2	294	15	48.47
RCCD1	NP_291022.2	500	8	15.20	ARMT1	NP_078849.1	329	15	43.31
PRC1	XP_011520494.1	871	3	3.27	CCDC170	NP_079335.2	298	14	44.63
VPS33B	NP_001276077.1	588	7	11.31	ESR1	NP_001278159.1	3698	9	2.31
SV2B	NP_001309965.1	952	8	7.98	SYNE1	XP_006715477.1	1284	12	8.88
SLCO3A1	NP_001138516.1	305	10	31.15	MYCT1	XP_016866808.1	273	14	48.72
ST8SIA2	NP_006002.1	603	7	11.03	VIP	NP_003372.1	859	2	2.21
FAM174B	NP_997329.2	178	4	21.35	FBXO5	NP_036309.1	795	7	8.36
CHD2	NP_001262.3	1283	8	5.92	MTRF1L	NP_001288799.1	625	12	18.24
RGMA	NP_064596.2	374	5	12.70	RGS17	NP_036551.3	423	13	29.19
MCTP2	NP_001371931.1	353	10	26.91	OPRM1	NP_001138753.1	1114	9	7.67
NR2F2	NP_001138627.1	1209	5	3.93	IPCEF1	NP_056368.1	288	6	19.79
ARRDC4	NP_899232.2	621	3	4.59	CNKSR3	NP_775786.2	268	12	42.54
FAM169B	NP_872368.2	47	1	20.21	SCAF8	NP_001273117.1	896	11	11.66
IGF1R	NP_000866.1	2728	5	1.74	TIAM2	NP_036586.3	700	12	16.28
PGPEP1L	XP_011519556.1	63	2	30.16	TFB1M	XP_011534172.1	1121	8	6.78
SYNM	XP_016877524.1	329	2	5.77	CLDN20	NP_001001346.1	136	10	69.85
TTC23	XP_011520243.1	108	1	8.80	NOX3	NP_056533.1	397	5	11.96
LRRC28	NP_001308607.1	457	0	0.00	ARID1B	NP_001358585.1	679	4	5.60
MEF2A	NP_001339545.1	1492	11	7.00	TMEM242	NP_060922.2	68	3	41.91
LYSMD4	XP_011519547.1	97	3	29.38	ZDHHC14	NP_078906.2	235	4	16.17
ADAMTS17	NP_620688.2	258	9	33.14	SNX9	NP_057308.1	643	6	8.86
CERS3	XP_016877491.1	458	9	18.67	SYNJ2	NP_003889.1	920	7	7.23
LINS1	NP_001339437.1	455	6	12.53	SERAC1	XP_011534498.1	298	4	12.75
ASB7	NP_937886.1	1288	12	8.85	GTF2H5	XP_016866351.1	505	6	11.29
ALDH1A3	NP_000684.2	915	6	6.23	TULP4	XP_011534248.1	382	5	12.43
LRRK1	XP_011520314.1	2493	10	4.01	TMEM181	XP_011534302.1	170	7	39.12
CHSY1	NP_055733.2	347	11	33.46	DYNLT1	NP_001278531.1	582	5	8.16
SELENOS	NP_060915.2	289	5	19.34	SYTL3	XP_024302356.1	268	7	24.81
SNRPA1	NP_003081.2	1527	7	5.44	EZR	NP_001104547.1	1577	7	4.22
PCSK6	NP_001278238.1	767	6	9.91	RSPH3	NP_001333347.1	489	7	13.60
TM2D3	NP_001294889.1	158	10	85.89	TAGAP	NP_473455.2	895	7	7.43
TARS3	NP_689547.2	861	1	1.70	FNDC1	NP_115921.2	554	6	10.29
OR4F6	NP_001005326.1	107	0	0.00	SOD2	NP_001309743.1	2922	5	1.63
OR4F15	NP_001001674.1	33	0	0.00	WTAP	XP_016867003.1	591	3	4.82

OR4F4	NP_001004195.2	52	0	0.00	ACAT2	NP_005882.2	831	3	3.43
POLR3K	NP_057394.3	761	6	14.98	TCP1	NP_110379.2	1983	7	3.35
SNRNP25	NP_078847.2	308	7	39.25	MRPL18	NP_054880.2	440	1	2.16
RHBDF1	NP_071895.3	548	11	31.78	PNLDC1	XP_016865801.1	258	4	14.73
MPG	NP_001015052.1	361	2	8.10	MAS1	NP_001353633.1	316	5	15.03
NPRL3	NP_001230177.1	432	13	40.84	IGF2R	NP_000867.3	1173	11	8.91
HBZ	NP_005323.1	558	9	20.43	SLC22A1	XP_005267159.1	386	2	4.92
HBM	NP_001003938.1	211	7	39.39	SLC22A2	NP_003049.2	614	7	10.83
HBA2	NP_000508.1	609	11	20.19	SLC22A3	NP_068812.1	554	5	8.57
HBA1	NP_000549.1	592	10	17.83	LPA	NP_005568.2	725	3	3.93
HBQ1	NP_005322.1	205	8	39.02	PLG	NP_000292.1	1531	4	2.48
LUC7L	XP_011520863.1	677	15	21.05	MAP3K4	NP_005913.3	561	1	1.69
FAM234A	XP_016879249.1	138	13	89.49	AGPAT4	XP_006715577.1	444	3	6.42
RGS11	NP_899180.1	706	8	10.76	PRKN	NP_054643.2	1813	7	3.67
ARHGDIG	NP_001167.2	624	7	10.66	PACRG	NP_689623.2	521	9	16.41
PDIA2	NP_006840.2	1521	10	6.25	QKI	NP_006766.1	1053	6	5.41
AXIN1	XP_011520985.1	1539	9	5.56	C6orf118	XP_005266895.1	57	10	166.66
MRPL28	NP_006419.2	350	2	5.43	PDE10A	XP_011533689.2	779	10	12.19
PGAP6	NP_067082.2	251	9	34.06	TBXT	NP_001257413.1	544	0	0.00
NME4	NP_005000.1	733	3	3.89	PRR18	NP_787118.2	696	13	17.74
DECR2	NP_065715.1	579	2	3.28	SFT2D1	NP_660152.1	285	13	43.33
RAB11FIP3	NP_001357330.1	546	5	8.70	MPC1	NP_057182.1	356	11	29.35
CAPN15	XP_016879085.1	637	2	2.98	RPS6KA2	NP_001006933.3	757	7	8.78
PRR35	XP_016878448.1	471	2	4.03	RNASET2	NP_003721.2	512	14	25.98
NHLRC4	NP_788850.1	153	1	6.21	CEP43	NP_001265619.1	414	2	4.59
PIGQ	NP_683721.1	399	6	14.28	CCR6	NP_004358.2	809	7	8.22
RAB40C	NP_066991.3	594	6	9.60	GPR31	NP_005290.2	431	15	33.06
WFIKKN1	NP_444514.1	224	1	4.24	UNC93A	XP_016866447.1	207	17	78.02
METTL26	NP_001035255.1	53	0	0.00	TTLL2	NP_114155.4	213	15	66.90
MCRIP2	NP_612427.2	111	0	0.00	AFDN	XP_016866375.1	928	14	14.33
WDR90	XP_016878512.1	1025	4	3.71	KIF25	NP_085118.2	842	17	19.18
RHOT2	XP_024306246.1	1661	9	5.15	FRMD1	XP_011534440.1	271	18	63.10
RHBDL1	NP_001265649.1	537	6	10.61	DACT2	NP_001273280.1	334	16	45.51
STUB1	NP_005852.2	1724	5	2.76	SMOC2	NP_071421.1	546	17	29.58
JMJD8	NP_001310849.2	107	2	17.76	THBS2	NP_001368868.1	903	15	15.78
WDR24	NP_115635.1	203	3	14.04	WDR27	XP_011533989.1	548	18	32.84
FBXL16	XP_024305928.1	1358	8	5.60	C6orf120	NP_001304271.1	147	18	129.24
METRN	NP_076947.1	251	7	26.49	PHF10	NP_579866.2	325	17	58.46
ANTKMT	NP_076422.1	460	5	10.33	TCTE3	XP_006715617.1	274	16	69.34
CCDC78	XP_011520672.1	209	11	50.00	ERMARD	NP_001265460.1	99	15	191.91
HAGHL	NP_001310565.1	218	6	26.15	DLL1	XP_005266991.1	1103	14	17.22
CIAO3	NP_071938.1	332	0	0.00	FAM120B	NP_001273308.1	191	13	99.47
MSLN	NP_037536.2	573	3	4.97	PSMB1	NP_002784.1	1730	10	9.15
RPUSD1	NP_001356587.1	495	4	7.68	TBP	NP_003185.1	2387	9	6.51
CHTF18	XP_011520874.1	855	3	3.33	PDCD2	NP_001186391.1	581	10	32.70
GNG13	NP_057625.1	1475	9	5.80	FAM20C	XP_016867939.1	434	0	0.00
PRR25	NP_001013660.1	18	0	0.00	PDGFA	XP_011513719.1	762	0	0.00
LMF1	NP_001338949.1	316	6	18.04	PRKAR1B	NP_001158230.1	1269	5	6.24
SOX8	NP_055402.2	1066	6	5.35	DNAAF5	NP_060272.3	148	0	0.00
SSTR5	NP_001166031.1	732	6	7.79	SUN1	NP_001165416.1	681	1	1.99
C1QTNF8	NP_997302.2	59	0	0.00	GET4	NP_057033.2	448	1	2.83
CACNA1H	XP_006721026.1	741	3	3.85	ADAP1	NP_001271237.1	797	6	8.94
TPSG1	NP_036599.4	205	3	13.90	COX19	NP_001026788.1	340	4	13.15
TPSB2	NP_077078.5	250	2	7.60	C7orf50	XP_016868209.1	353	2	5.98
TPSAB1	NP_003285.2	372	2	5.11	CYP2W1	XP_011513742.1	242	5	20.66
TPSD1	NP_036349.1	187	1	5.08	GPR146	XP_006715705.1	218	5	21.79
UBE2I	XP_016879129.1	2736	3	1.04	GPER1	NP_001091671.1	552	2	3.44
BAIAP3	XP_011521030.1	340	5	13.97	ZFAND2A	NP_001352312.1	306	1	3.10
TSR3	NP_001001410.1	268	1	3.54	UNCX	NP_001073930.1	846	0	0.00
GNPTG	XP_016879271.1	414	2	4.59	MICALL2	XP_011513824.1	532	4	7.14
UNKL	XP_011520914.1	596	0	0.00	INTS1	NP_001073922.2	538	2	3.53
C16orf91	NP_001258980.1	36	0	0.00	MAFK	NP_002351.1	560	3	5.09
CCDC154	NP_001137452.1	37	0	0.00	TMEM184A	XP_016867304.1	219	0	0.00
CLCN7	NP_001107803.1	499	5	9.52	PSMG3	XP_024302744.1	249	3	11.45
PTX4	NP_001315537.1	152	2	12.50	ELFN1	XP_016867692.1	214	6	26.63
TELO2	XP_011521075.1	533	3	5.35	MAD1L1	NP_003541.2	1017	10	9.34

IFT140	XP_011521068.1	476	4	7.98	MRM2	NP_037525.1	761	5	6.24
TMEM204	NP_078876.2	373	2	5.09	NUDT1	NP_945187.1	797	5	5.96
CRAMP1	NP_065876.3	152	4	25.00	SNX8	NP_037453.1	473	5	10.04
JPT2	NP_653171.1	128	0	0.00	EIF3B	NP_001349720.1	1342	3	2.12
MAPK8IP3	XP_024305969.1	723	8	10.51	CHST12	NP_001230723.1	277	9	30.86
NME3	NP_002504.2	768	10	12.37	GRIFIN	XP_011513707.1	133	7	50.00
MRPS34	NP_076425.1	636	9	13.44	LFNG	NP_001159827.1	521	8	14.59
EME2	NP_001244299.1	197	9	43.40	BRAT1	XP_016867323.1	193	9	44.30
SPSB3	NP_001311010.1	327	5	14.53	IQCE	NP_689771.3	170	8	44.70
NUBP2	NP_036357.1	617	9	13.86	TTYH3	NP_079526.1	225	9	38.00
IGFALS	NP_004961.1	812	9	10.53	AMZ1	XP_016867263.1	133	6	42.85
HAGH	NP_005317.2	617	5	7.70	GNA12	NP_001280021.1	1196	5	3.97
FAHD1	NP_001135870.1	373	3	7.64	CARD11	NP_001311210.1	1258	1	0.76
MEIOB	NP_689977.2	703	1	1.35	SDK1	NP_689957.3	458	1	2.07
HS3ST6	XP_011520910.1	279	8	27.24	FOXK1	NP_001032242.1	1016	3	2.80
MSRB1	NP_001369194.1	307	6	18.57	AP5Z1	NP_055670.1	258	4	14.73
RPL3L	NP_005052.1	1194	11	8.75	RADIL	NP_060529.4	903	5	5.26
NDUFB10	NP_004539.1	632	0	0.00	PAPOLB	NP_064529.4	648	5	7.33
RPS2	NP_002943.2	1674	3	1.70	MMD2	NP_940685.3	917	5	5.18
RNF151	XP_005255186.1	361	0	0.00	RBAK-RBAKDI	NP_001191442.1	119	0	0.00
TBL3	NP_006444.2	820	6	6.95	RBAK	NP_066986.1	180	3	15.83
NOXO1	NP_653204.1	173	3	16.47	WIPI2	NP_056425.1	598	1	1.59
GFER	NP_005253.3	818	6	6.97	SLC29A4	NP_694979.2	260	0	0.00
SYNGR3	NP_004200.2	609	6	9.36	TNRC18	XP_016868222.1	300	1	3.17
ZNF598	NP_835461.2	461	2	4.12	FBXL18	NP_001350370.1	388	6	14.69
NPW	NP_001092926.2	381	1	2.49	ACTB	NP_001092.1	4607	5	1.03
SLC9A3R2	NP_001123484.1	1072	3	2.66	FSCN1	NP_003079.1	756	6	7.54
NTHL1	NP_001305122.2	760	3	3.75	RNF216	NP_001364085.1	349	10	27.22
TSC2	XP_011520940.2	1587	6	3.59	OCM	NP_001091091.1	1081	6	5.27
PKD1	XP_024306067.1	580	4	6.55	CCZ1	NP_056437.4	336	4	11.31
RAB26	NP_055168.2	316	5	15.03	RSPH10B	XP_011513511.1	96	7	69.27
TRAF7	NP_115647.2	1311	5	3.62	PMS2	NP_001308935.1	1008	10	9.42
CASKIN1	XP_024306129.1	1453	6	3.92	AIMP2	NP_006294.2	735	11	14.22
MLST8	XP_016879039.1	963	3	2.96	EIF2AK1	NP_001127807.1	892	1	1.06
BRICD5	NP_872369.2	1	0	0.00	ANKRD61	NP_001258629.1	878	7	7.57
PGP	NP_001035830.1	451	2	4.21	USP42	XP_024302736.1	257	3	11.09
E4F1	XP_011520705.1	616	6	9.25	CYTH3	NP_004218.1	577	10	16.46
DNASE1L2	NP_001365.1	232	4	16.38	FAM220A	NP_001032240.1	24	0	0.00
ECI1	NP_001171500.1	889	5	5.34	RAC1	NP_061485.1	2232	6	2.55
RNPS1	NP_001273556.1	1001	2	1.90	DAGLB	NP_001136408.1	308	6	18.51
ABCA3	NP_001080.2	654	5	7.26	KDELR2	NP_001094073.1	686	6	8.31
CCNF	NP_001752.2	1604	3	1.78	GRID2IP	NP_001138590.1	1759	9	4.86
TEDC2	NP_079384.2	151	1	6.29	ZDHHC4	XP_016867880.1	231	1	4.11
NTN3	NP_006172.1	291	1	3.26	C7orf26	XP_005249902.1	74	0	0.00
TBC1D24	XP_016878982.1	405	2	4.69	ZNF853	XP_011513740.1	273	0	0.00
ATP6V0C	NP_001185498.1	1037	2	1.83	ZNF316	XP_024302386.1	399	1	2.38
AMDHD2	NP_001139287.1	292	1	3.25	ZNF12	NP_008887.2	284	7	23.41
CEMP1	NP_001041677.1	47	0	0.00	RSPH10B2	XP_024302670.1	120	1	7.92
PDPK1	NP_002604.1	1012	1	0.94	CCZ1B	NP_932765.1	341	0	0.00
KCTD5	NP_061865.1	275	0	0.00	C1GALT1	XP_016867931.1	468	2	4.06
PRSS27	NP_114154.1	286	1	3.32	COL28A1	XP_016867620.1	479	5	9.92
SRRM2	NP_057417.3	1161	0	0.00	MIOS	NP_061878.3	234	2	8.12
ELOB	NP_996896.1	1381	1	0.69	RPA3	NP_002938.1	862	3	3.31
PRSS33	NP_690851.2	244	1	3.89	UMAD1	NP_001289277.1	2	0	0.00
PRSS21	NP_006790.1	511	3	5.58	GLCCI1	NP_612435.1	235	2	8.08
ZG16B	NP_660295.3	130	0	0.00	ICA1	XP_024302508.1	484	6	11.78
PRSS22	NP_071402.1	357	1	2.66	NXPH1	NP_689958.1	937	3	3.04
FLYWCH2	NP_001135971.1	71	0	0.00	NDUFA4	NP_002480.1	725	3	3.93
FLYWCH1	XP_006721025.1	71	3	40.14	PHF14	NP_001007158.1	454	4	8.37
KREMEN2	NP_757384.1	454	3	6.28	THSD7A	XP_006715723.1	225	4	16.89
PAQR4	NP_689554.2	248	4	15.32	TMEM106B	NP_001127704.1	319	4	11.91
PKMYT1	NP_001245379.1	1210	4	3.14	VWDE	NP_001333901.1	226	0	0.00
CLDN9	NP_066192.1	213	0	0.00	SCIN	NP_001106177.1	502	2	3.78
CLDN6	NP_067018.2	417	2	4.56	ARL4A	NP_001182325.1	1469	6	3.88
TNFRSF12A	NP_057723.1	575	1	1.65	ETV1	NP_001357485.1	1039	5	4.57
HCFC1R1	NP_001002017.1	97	0	0.00	DGKB	NP_001337650.1	741	7	8.97

THOC6	NP_001334633.1	426	0	0.00	AGMO	XP_016867693.1	484	9	17.66
BICDL2	NP_001096645.1	113	0	0.00	MEOX2	NP_005915.2	590	11	17.71
MMP25	NP_071913.1	561	0	0.00	CRPPA	NP_001094887.1	464	9	18.43
IL32	NP_001356517.1	344	5	13.81	SOSTDC1	NP_056279.1	324	5	14.66
ZSCAN10	XP_016879281.1	386	5	12.31	LRRC72	XP_011513359.1	23	1	41.30
ZNF205	NP_003447.2	339	6	16.81	ANKMY2	NP_064715.1	804	13	15.36
ZNF213	NP_001128127.1	173	9	49.42	BZW2	NP_001349646.1	368	11	28.40
OR1F1	NP_001357568.1	61	9	140.16	TSPAN13	NP_055214.1	395	13	31.26
ZNF200	NP_932353.1	207	8	36.71	AGR2	XP_005249638.1	499	7	13.33
MEFV	XP_016878725.1	711	8	10.69	AGR3	XP_011513454.1	278	9	30.75
ZNF263	XP_011520646.1	449	6	12.69	AHR	NP_001612.1	1103	7	6.03
TIGD7	NP_149985.2	174	9	49.14	SNX13	XP_016867382.1	352	12	32.38
ZNF75A	XP_016879134.1	285	4	13.33	PRPS1L1	NP_787082.1	1025	5	4.63
OR2C1	NP_036500.2	45	0	0.00	HDAC9	NP_001308797.1	2252	11	4.64
MTRNR2L4	NP_001177405.1	54	0	0.00	TWIST1	NP_000465.1	1606	7	4.14
ZSCAN32	XP_016878861.1	208	5	22.84	FERD3L	NP_690862.1	260	12	43.84
ZNF174	NP_001334797.1	290	7	22.93	POLR1F	NP_001002926.1	890	10	10.67
ZNF597	NP_689670.1	234	2	8.12	TMEM196	NP_689987.3	447	10	21.25
NAA60	NP_001304022.1	348	5	13.65	MACC1	NP_877439.3	178	11	58.70
C16orf90	NP_001340311.1	51	0	0.00	ITGB8	XP_011513695.1	762	11	13.71
CLUAP1	NP_001317383.1	341	6	16.71	ABCB5	NP_001157413.1	702	9	12.18
NLRC3	XP_016878527.1	344	0	0.00	SP8	NP_874359.2	737	11	14.18
SLX4	XP_011521017.1	347	0	0.00	SP4	NP_003103.2	445	8	17.08
DNASE1	XP_016878489.1	908	3	3.14	DNAH11	NP_001264044.1	853	14	15.59
TRAP1	NP_001258978.1	1759	5	2.70	CDCA7L	NP_001120843.1	360	14	36.94
CREBBP	NP_001073315.1	3056	6	1.87	RAPGEF5	NP_036426.4	543	14	24.49
ADCY9	XP_011520655.1	1134	3	2.51	STEAP1B	NP_997225.1	99	1	9.60
SRL	NP_001092284.1	400	0	0.00	IL6	NP_000591.1	4118	3	0.69
TFAP4	NP_003214.1	316	2	6.01	TOMM7	NP_061932.1	596	1	1.59
GLIS2	NP_115964.2	697	3	4.09	FAM126A	XP_011513892.1	166	11	62.95
CORO7-PAM16	NP_001188408.1	375	1	2.53	KLHL7	XP_006715816.1	482	8	15.77
PAM16	NP_057153.8	323	2	5.88	NUP42	NP_001357372.1	408	10	23.28
CORO7	NP_001188402.1	181	1	5.25	GPNMB	XP_016867165.1	556	5	8.54
VASN	NP_612449.2	738	2	2.57	MALSU1	NP_612455.1	596	13	20.72
DNAJA3	NP_001128582.1	742	4	5.12	IGF2BP3	NP_006538.2	757	8	10.04
NMRAL1	XP_016878974.1	365	0	0.00	TRA2A	NP_001349688.1	1610	8	4.72
HMOX2	NP_001273196.1	1227	1	0.77	CCDC126	XP_016868264.1	133	8	57.14
CDIP1	NP_001185985.1	236	2	8.05	FAM221A	NP_954587.2	134	3	21.27
C16orf96	NP_001138483.1	42	0	0.00	STK31	XP_011513751.2	288	7	23.09
UBALD1	NP_001317396.1	132	0	0.00	NPY	NP_000896.1	1463	2	1.30
MGRN1	NP_056061.1	543	2	3.50	MPP6	XP_016867804.1	865	3	3.29
NUDT16L1	XP_005255690.1	164	0	0.00	GSDME	XP_024302438.1	247	6	23.08
ANKS3	XP_016878424.1	890	0	0.00	OSBPL3	NP_663162.1	438	4	8.68
C16orf71	XP_016878464.1	37	0	0.00	CYCS	NP_061820.1	3790	3	0.75
ZNF500	XP_011520755.1	355	1	2.68	C7orf31	NP_001358281.1	71	0	0.00
SEPTIN12	XP_016878427.1	505	1	1.88	NPVF	NP_071433.3	168	2	11.31
SMIM22	NP_001240720.1	4	0	0.00	NFE2L3	NP_004280.5	383	7	17.36
ROGDI	NP_078865.1	180	0	0.00	HNRNPA2B1	XP_016867598.1	2128	5	2.23
GLYR1	XP_005255696.3	1414	1	0.67	CBX3	NP_009207.2	1440	3	1.98
UBN1	XP_011520767.1	485	2	3.92	SNX10	NP_037454.2	453	4	8.39
PPL	NP_002696.4	1231	1	0.77	SKAP2	XP_016868260.1	435	4	8.74
SEC14L5	XP_024306265.1	414	3	6.88	HOXA1	NP_005513.2	403	11	25.93
NAGPA	XP_011520819.1	240	1	3.96	HOXA2	NP_006726.1	602	13	20.51
C16orf89	XP_005255200.2	60	0	0.00	HOXA3	NP_109377.1	563	13	21.93
ALG1	NP_061982.3	582	2	3.26	HOXA4	NP_002132.3	421	12	27.08
EEF2KMT	NP_001275958.1	186	2	10.21	HOXA5	NP_061975.2	823	11	12.70
RBFOX1	XP_016878808.1	1673	10	5.68	HOXA6	NP_076919.1	283	10	33.57
TMEM114	NP_001277027.1	82	5	57.92	HOXA7	NP_008827.2	635	12	17.95
METTL22	XP_016879150.1	204	9	41.91	HOXA9	NP_689952.1	891	12	12.79
ABAT	NP_001120920.1	723	8	10.51	HOXA10	NP_061824.3	703	14	18.92
TMEM186	NP_056236.2	67	9	127.61	HOXA11	NP_005514.1	555	16	27.39
PMM2	NP_000294.1	698	7	9.53	HOXA13	NP_000513.2	473	15	30.13
CARHSP1	XP_005255285.3	483	9	17.70	EVX1	NP_001980.1	552	13	22.37
USP7	NP_003461.2	1583	5	3.00	HIBADH	NP_689953.1	748	9	11.43
C16orf72	NP_054836.2	102	9	83.82	TAX1BP1	NP_006015.4	562	9	15.21
GRIN2A	XP_016878661.1	1636	5	2.90	JAZF1	NP_778231.2	730	8	10.41

ATF7IP2	XP_016879194.1	97	1	9.79	CREB5	XP_011513920.1	738	7	9.01
EMP2	NP_001415.1	354	2	5.37	TRIL	NP_055632.2	819	1	1.16
TEKT5	NP_653275.1	280	6	20.36	CPVL	XP_016867855.1	837	1	1.13
NUBP1	NP_002475.2	694	4	5.48	CHN2	XP_016867210.1	678	4	5.60
TVP23A	XP_016879138.1	72	0	0.00	PRR15	NP_001316925.1	149	2	12.75
CIITA	XP_006720943.2	996	5	4.77	WIPF3	NP_001073998.2	504	2	3.77
DEXI	NP_054734.2	229	6	24.89	SCRN1	NP_001138986.1	440	0	0.00
CLEC16A	NP_056041.1	738	6	7.72	FKBP14	NP_060416.1	952	6	5.99
SOCS1	NP_003736.1	1416	9	6.04	PLEKHA8	NP_001337903.1	350	6	16.28
TNP2	NP_005416.1	368	6	15.49	MTURN	NP_690006.2	305	7	21.80
PRM3	NP_067070.2	146	2	13.01	ZNRF2	NP_667339.1	472	7	14.09
PRM2	NP_001273287.1	448	3	6.36	NOD1	XP_011513385.1	779	1	1.22
PRM1	NP_002752.1	156	4	24.36	GGCT	NP_001186746.1	416	6	13.70
RMI2	NP_689521.1	452	8	16.81	GARS1	NP_002038.2	1179	9	7.25
LOC400499	XP_016879425.1	258	0	0.00	CRHR2	NP_001189404.1	518	11	20.17
LITAF	XP_011521056.1	471	4	8.07	INMT	NP_006765.4	361	3	7.89
SNN	XP_016879230.1	258	6	22.09	MINDY4	NP_115598.2	268	3	10.63
TXNDC11	NP_056998.4	906	3	3.15	AQP1	NP_001316801.1	1228	7	5.42
ZC3H7A	NP_054872.2	206	1	4.61	GHRHR	NP_000814.2	439	4	8.66
RSL1D1	NP_056474.2	952	5	4.99	ADCYAP1R1	XP_006715708.1	1030	5	4.61
GSPT1	NP_001123478.2	1400	3	2.04	NEUROD6	NP_073565.2	1095	7	6.07
TNFRSF17	NP_001183.2	492	1	1.93	ITPRID1	NP_001244897.2	92	0	0.00
SNX29	NP_115543.3	323	2	5.88	PPP1R17	NP_006649.2	245	1	3.88
CPPED1	NP_001092925.1	428	2	4.44	PDE1C	NP_001177987.2	526	8	14.45
SHISA9	XP_011520944.1	296	2	6.42	LSM5	NP_036454.1	1735	1	0.55
ERCC4	XP_011520726.1	896	0	0.00	AVL9	XP_011513529.1	222	1	4.28
MRTFB	XP_016878990.1	372	2	5.11	KBTBD2	NP_056298.2	289	6	19.72
PARN	NP_001229921.1	515	0	0.00	FKBP9	NP_001271270.1	997	5	4.76
BFAR	NP_057645.1	336	4	11.31	NT5C3A	NP_001361264.1	453	5	10.49
PLA2G10	NP_003552.1	334	2	5.69	RP9	NP_976033.1	262	4	14.50
NPIPA3	XP_016879042.1	20	0	0.00	BBS9	NP_001334970.1	428	5	11.10
LOC10065277	XP_011521092.1	334	2	5.69	BMPER	NP_001352237.1	476	1	2.00
NPIPA2	XP_024306149.1	31	7	214.50	NPSR1	NP_997056.1	1385	1	0.69
NOMO1	NP_055102.3	174	7	38.22	DPY19L1	NP_056098.1	330	1	2.88
NPIPA1	NP_008916.2	76	3	37.50	TBX20	NP_001071121.1	601	0	0.00
PDXDC1	XP_024305963.1	241	15	59.13	HERPUD2	XP_006715828.1	213	0	0.00
NTAN1	NP_775745.1	248	11	42.13	SEPTIN7	XP_011513963.1	1119	1	0.85
RRN3	NP_001287993.1	465	13	26.56	EEPD1	NP_085139.2	814	2	2.33
NPIPA5	XP_024305900.1	20	0	0.00	KIAA0895	NP_001093895.1	24	0	0.00
MPV17L	XP_016878597.1	252	11	41.47	ANLN	XP_006715809.1	934	3	3.05
BMERB1	NP_149978.1	223	10	42.60	AOAH	XP_011513636.1	509	3	5.60
MARF1	XP_016879395.1	196	13	63.01	ELMO1	XP_016868328.1	708	5	6.71
NDE1	XP_006720960.1	785	12	14.52	GPR141	XP_011513676.1	180	4	21.11
MYH11	NP_002465.1	1806	12	6.31	NME8	NP_057700.3	2158	8	3.52
CEP20	NP_001291429.1	268	12	42.54	SFRP4	NP_003005.2	785	3	3.63
ABCC1	NP_004987.2	940	12	12.13	EPDR1	NP_060019.2	249	4	15.26
ABCC6	XP_016878701.1	679	12	16.79	STARD3NL	XP_016868182.1	123	6	46.34
NOMO3	XP_005255375.1	146	11	71.57	AMPH	XP_006715753.1	1571	4	2.42
NPIPA7	XP_016878322.1	0	0	#DIV/0!	VPS41	NP_542198.2	532	0	0.00
XYLT1	NP_071449.1	320	5	14.84	POU6F2	NP_001357888.1	416	2	4.57
NPIPA8	NP_001269440.1	0	0	#DIV/0!	YAE1	NP_001269375.1	86	0	0.00
LOC10272372	XP_006721059.1	146	8	52.05	RALA	XP_011513769.1	1691	5	2.81
NOMO2	NP_001004060.1	160	3	17.81	CDK13	XP_016868239.1	965	2	1.97
RPS15A	NP_001010.2	1352	3	2.11	MPLKIP	NP_619646.1	213	3	13.38
ARL6IP1	NP_055976.1	350	2	5.43	SUGCT	XP_011513827.1	447	1	2.13
SMG1	XP_005255241.1	2143	5	2.22	INHBA	XP_016867664.1	905	3	3.15
TMC7	NP_079123.3	91	1	10.44	GLI3	NP_000159.3	1821	4	2.09
COQ7	NP_057222.2	484	2	3.93	C7orf25	NP_001093328.1	137	3	20.80
ITPRIPL2	NP_001030013.1	104	0	0.00	PSMA2	NP_002778.1	1529	7	4.35
SYT17	NP_057608.2	399	0	0.00	MRPL32	NP_114109.1	735	4	5.17
CLEC19A	NP_001243649.1	175	0	0.00	HECW1	XP_006715733.1	917	3	3.11
TMC5	NP_001098718.1	231	1	4.11	STK17A	NP_004751.2	659	2	2.88
GDE1	NP_001310996.1	385	2	4.93	COA1	NP_001308127.1	127	1	7.48
CCP110	NP_001310500.1	520	1	1.83	BLVRA	XP_016868009.1	480	0	0.00
VPS35L	NP_064710.5	158	1	6.01	MRPS24	NP_114403.1	607	2	3.13
KNOP1	XP_024306046.1	154	3	18.51	URGCP-MRPS	NP_001191800.1	0	0	#DIV/0!

IQCK	NP_694940.1	190	3	15.00	UBE2D4	XP_006715797.1	1440	5	3.30
GPRC5B	NP_001291700.1	589	2	3.23	SPDYE1	XP_016867504.1	107	1	8.88
GPR139	NP_001002911.1	283	1	3.36	DBNL	NP_001116428.1	733	0	0.00
GP2	NP_001007243.2	485	3	5.88	PGAM2	NP_000281.2	732	3	3.89
UMOD	NP_001365161.1	573	2	3.32	POLM	NP_001271259.1	453	1	2.10
PDILT	NP_777584.1	597	2	3.18	AEBP1	NP_001120.3	424	3	6.72
ACSM5	NP_001311301.1	473	1	2.01	POLD2	XP_024302570.1	854	3	3.34
ACSM2A	XP_016878412.1	443	0	0.00	MYL7	NP_067046.1	1297	4	2.93
ACSM2B	NP_872423.3	379	0	0.00	GCK	NP_001341729.1	1319	3	2.16
ACSM1	XP_011544032.1	525	2	3.62	YKT6	NP_006546.1	1121	2	1.69
THUMPD1	XP_016878922.1	479	0	0.00	CAMK2B	NP_742081.1	1430	5	3.32
ACSM3	XP_024306136.1	629	2	3.02	NUDCD3	XP_011513549.1	312	3	9.13
ERI2	NP_542394.2	244	1	3.89	NPC1L1	XP_011513628.1	555	1	1.71
REXO5	NP_112203.2	503	0	0.00	DDX56	NP_001244118.1	1649	4	2.30
DCUN1D3	NP_775746.1	293	0	0.00	TMED4	NP_001289988.1	405	2	4.69
LYRM1	XP_016878964.1	69	0	0.00	OGDH	NP_001350452.1	1366	2	1.39
DNAH3	NP_060009.1	708	1	1.34	ZMIZ2	XP_016868163.1	584	0	0.00
TMEM159	XP_005255496.1	43	0	0.00	PPIA	NP_066953.1	1930	5	2.46
ZP2	NP_001363160.1	443	0	0.00	H2AZ2	NP_619541.1	1961	5	2.42
ANKS4B	NP_665872.2	879	0	0.00	PURB	NP_150093.1	347	3	8.21
CRYM	NP_001363185.1	753	1	1.26	MYO1G	NP_149043.2	1718	2	1.11
NPIPB3	NP_569731.2	69	0	0.00	CCM2	NP_001350387.1	379	2	5.01
METTL9	NP_001275588.1	94	1	10.11	NACAD	XP_006715737.1	919	0	0.00
IGSF6	NP_005840.2	531	1	1.79	TBRG4	NP_001248763.1	437	5	10.87
OTOA	NP_653273.3	270	1	3.52	RAMP3	XP_006715694.1	302	2	6.29
NPIPB4	XP_024306057.1	48	0	0.00	ADCY1	NP_066939.1	1498	2	1.27
UQCRC2	NP_003357.2	1221	3	2.33	IGFBP1	NP_000587.1	1245	2	1.53
PDZD9	XP_016878598.1	58	6	98.27	IGFBP3	NP_000589.2	1268	3	2.25
MOSMO	NP_001158051.1	106	6	53.77	TNS3	XP_011513779.1	1064	3	2.68
VWA3A	XP_016878436.1	114	6	50.00	C7orf65	NP_001116537.1	0	0	#DIV/0!
SDR42E2	XP_016879466.1	293	0	0.00	PKD1L1	XP_016867287.1	242	0	0.00
EEF2K	XP_016878682.1	463	10	20.52	HUS1	NP_004498.1	543	2	3.50
POLR3E	NP_060589.1	531	4	7.16	SUN3	XP_016867419.1	354	3	8.05
CDR2	NP_001793.1	510	8	14.90	C7orf57	NP_001093629.1	45	2	42.22
NPIPB5	NP_001129337.1	13	0	0.00	UPP1	NP_001349703.1	578	3	4.93
HS3ST2	NP_006034.1	356	10	26.68	ABCA13	XP_011513432.1	635	5	7.48
USP31	XP_016878985.1	306	1	3.10	VWC2	NP_940972.2	448	10	21.20
SCNN1G	NP_001030.2	648	12	17.59	ZPBP	XP_011513399.1	252	10	37.70
SCNN1B	XP_016879014.1	502	10	18.92	SPATA48	NP_001155306.3	161	7	41.30
COG7	NP_705831.1	473	13	26.11	IKZF1	XP_011513360.1	1531	8	4.96
GGA2	NP_055859.1	646	12	17.65	FIGNL1	XP_016867990.1	1064	11	9.82
EARS2	NP_001077083.1	1019	16	14.92	DDC	NP_001229817.2	740	3	3.85
UBFD1	XP_011544196.1	560	5	8.48	GRB10	NP_001337744.1	699	12	16.31
NDUFAB1	NP_004994.1	2060	12	5.53	COBL	NP_001333372.1	396	15	35.98
PALB2	XP_016879161.1	497	10	19.11	POM121L12	NP_872401.3	72	7	92.36
DCTN5	NP_115875.1	450	13	27.44	VSTM2A	NP_001287938.1	688	10	13.81
PLK1	NP_005021.2	2913	8	2.61	SEC61G	NP_001012474.1	1074	16	14.15
ERN2	NP_001295149.2	1125	13	10.98	EGFR	NP_005219.2	5157	12	2.21
CHP2	NP_071380.1	878	2	2.16	LANCL2	NP_061167.1	235	16	64.68
PRKCB	NP_997700.1	1137	8	6.68	VOPP1	XP_011513841.1	156	13	79.16
CACNG3	NP_006530.1	1011	11	10.34	SEPTIN14	NP_997249.2	228	16	66.66
RBBP6	NP_008841.2	950	8	8.00	ZNF713	NP_872439.2	207	14	64.25
TNRC6A	XP_024305999.1	508	1	1.87	MRPS17	NP_057053.1	461	12	24.73
SLC5A11	XP_016878392.1	471	1	2.02	NIPSNAP2	NP_001474.1	507	14	26.23
ARHGAP17	NP_060524.4	433	2	4.39	PSPH	XP_016867957.1	867	14	15.34
LCMT1	NP_057393.2	309	2	6.15	CCT6A	NP_001753.1	1728	13	7.15
AQP8	XP_011544124.1	575	3	4.96	SUMF2	XP_011513556.1	427	13	28.92
ZKSCAN2	NP_001012999.3	245	2	7.75	PHKG1	XP_005271829.1	411	12	27.74
HS3ST4	NP_006031.2	581	3	4.91	CHCHD2	NP_001307256.1	544	11	19.21
KDM8	NP_001138820.1	496	1	1.92	NUPR2	NP_001139184.1	107	9	79.90
NSMCE1	XP_006721086.1	392	3	7.27	ZNF479	XP_011513911.1	232	0	0.00
IL4R	NP_001244335.1	932	5	5.10	ZNF716	NP_001152751.1	200	0	0.00
IL21R	NP_851565.4	653	4	5.82	ZNF727	NP_001152994.1	147	0	0.00
GTF3C1	XP_016878677.1	439	2	4.33	ZNF735	NP_001152996.1	140	0	0.00
KATNIP	XP_005255258.1	294	3	9.69	ZNF679	XP_016867286.1	175	0	0.00
GSG1L	NP_001310830.1	223	2	8.52	ZNF736	NP_001164376.1	139	0	0.00

XPO6	NP_055986.1	1581	3	1.80	ZNF680	NP_848653.2	200	1	4.75
SBK1	XP_005255372.1	276	2	6.88	ZNF107	NP_001269288.1	228	1	4.17
NPIPB6	NP_001269453.1	0	0	#DIV/0!	ZNF138	NP_001258568.2	134	1	7.09
EIF3CL	NP_001093131.1	699	1	1.36	ZNF273	NP_066971.2	199	1	4.77
CLN3	NP_001273033.1	465	8	16.34	ZNF117	NP_001334979.1	256	1	3.71
APOBR	NP_061160.3	554	10	17.15	ERV3-1	NP_001007254.2	81	5	58.64
IL27	XP_011544082.1	230	2	8.26	ZNF92	NP_689839.1	192	1	4.95
NUPR1	NP_036517.1	270	3	10.56	VKORC1L1	XP_011514133.1	847	1	1.12
SGF29	NP_612423.1	543	9	15.75	GUSB	NP_001271219.1	1056	1	0.90
SULT1A2	NP_001045.2	228	13	54.16	ASL	NP_000039.2	601	0	0.00
SULT1A1	NP_803880.1	408	10	23.28	CRCP	NP_001135886.1	248	2	7.66
NPIPB8	XP_016879111.1	41	3	69.51	TPST1	XP_016868215.1	825	1	1.15
EIF3C	NP_001186071.1	1010	12	11.29	KCTD7	NP_001161433.1	520	3	5.48
ATXN2L	XP_005255128.1	990	13	12.47	RABGEF1	NP_001273990.1	1806	0	0.00
TUFM	NP_001352289.1	1762	13	7.01	TMEM248	XP_024302587.1	105	1	9.05
SH2B1	NP_001139267.1	448	12	25.45	SBDS	NP_057122.2	642	2	2.96
ATP2A1	NP_775293.1	1352	9	6.32	TYW1	NP_060734.2	352	2	5.40
RABEP2	NP_079092.2	459	13	26.90	AUTS2	XP_011514312.1	661	4	5.75
CD19	NP_001171569.1	1516	4	2.51	GALNT17	NP_071924.1	654	6	8.72
NFATC2IP	NP_116204.3	1983	8	3.83	CALN1	XP_016868166.1	1465	8	5.19
SPNS1	XP_016879246.1	364	11	28.71	TYW1B	NP_001138912.2	320	1	2.97
LAT	NP_001014989.2	443	3	6.43	POM121	XP_005250783.1	246	1	3.86
NPIPB11	NP_001297066.2	16	0	0.00	TRIM74	XP_011514492.1	266	2	7.14
BOLA2-SMG1F	NP_001307553.1	1604	4	2.37	NSUN5	NP_001161819.1	819	12	13.92
BOLA2	NP_001026997.1	2036	11	5.13	TRIM50	XP_011514091.2	616	11	16.96
SLX1B	NP_076949.1	332	9	25.75	FKBP6	NP_001128683.1	1192	14	11.16
SULT1A4	NP_001017390.1	182	5	26.10	FZD9	NP_003499.1	755	12	15.10
NPIPB12	NP_001342330.1	0	0	#DIV/0!	BAZ1B	NP_115784.1	1493	14	8.91
SPN	NP_003114.1	822	2	2.31	BCL7B	NP_001184173.1	259	16	58.68
QPRT	XP_005255280.2	371	12	30.73	TBL2	NP_036585.1	723	14	18.39
C16orf54	NP_787096.2	71	12	160.55	MLXIPL	NP_116572.1	658	13	18.77
ZG16	NP_689551.3	183	12	62.29	VPS37D	XP_016867268.1	186	2	10.21
KIF22	XP_024306038.1	1286	15	11.08	DNAJC30	NP_115693.2	241	12	47.30
MAZ	NP_001263204.1	672	15	21.20	BUD23	NP_001189489.1	1041	15	13.69
PRRT2	XP_016878376.1	821	17	19.67	STX1A	NP_004594.1	1275	17	12.67
PAGR1	NP_078792.1	110	16	138.17	ABHD11	XP_024302715.1	363	9	23.55
MVP	NP_059447.2	423	5	11.23	CLDN3	NP_001297.1	491	13	25.15
CDIPT	NP_001273514.1	1179	17	13.70	CLDN4	NP_001296.1	643	14	20.68
SEZ6L2	NP_001230262.1	504	19	35.81	METTL27	NP_689772.2	684	8	11.11
ASPHD1	XP_024305990.1	258	20	73.64	TMEM270	NP_872310.2	80	11	130.62
KCTD13	NP_849194.1	325	20	58.46	ELN	XP_011514170.1	1232	11	8.48
TMEM219	NP_001356619.1	104	19	173.55	LIMK1	NP_002305.1	1469	12	7.76
TAOK2	XP_011544286.1	357	19	50.56	EIF4H	NP_071496.1	1592	13	7.76
HIRIP3	NP_003600.2	239	19	75.52	LAT2	NP_115853.2	274	3	10.40
INO80E	NP_775889.1	318	20	59.75	RFC2	NP_002905.2	1402	14	9.49
DOC2A	XP_024306241.1	330	19	54.69	CLIP2	NP_003379.4	615	14	21.62
C16orf92	NP_001340308.1	42	19	429.74	GTF2IRD1	NP_001186136.1	397	12	28.71
TLCD3B	XP_024306232.1	174	20	109.19	GTF2I	NP_127492.1	809	11	12.92
ALDOA	NP_001230106.1	1249	16	12.17	NCF1	NP_000256.4	1302	12	8.76
PPP4C	NP_002711.1	2643	19	6.83	GTF2IRD2	NP_001355229.1	180	8	42.22
TBX6	XP_016879103.1	574	16	26.48	CASTOR2	XP_016868063.1	120	4	31.67
YPEL3	NP_113665.3	167	16	91.01	RCC1L	NP_683682.1	753	8	10.09
GDPD3	NP_077283.2	408	15	34.92	GTF2IRD2B	NP_001355231.1	209	5	22.73
MAPK3	NP_001103361.1	5269	13	2.34	TRIM73	NP_944606.2	270	2	7.04
CORO1A	NP_001180262.1	1350	14	9.85	POM121C	NP_001092885.2	209	0	0.00
BOLA2B	NP_001034271.2	1965	13	6.28	SPDYE5	NP_001293070.1	19	0	0.00
SLX1A	NP_001014999.1	343	8	22.16	HIP1	NP_001230127.1	755	1	1.26
SULT1A3	NP_808220.1	251	7	26.49	CCL26	NP_001358867.1	462	1	2.06
NPIPB13	NP_001308821.1	0	0	#DIV/0!	CCL24	NP_001358122.1	502	1	1.89
CD2BP2	NP_001230575.1	568	1	1.67	RHBDD2	NP_001035546.1	286	0	0.00
TBC1D10B	XP_011544091.1	283	1	3.36	POR	NP_001369584.1	799	2	2.38
MYLPF	NP_001311388.1	1324	3	2.15	TMEM120A	NP_001304732.1	155	0	0.00
SEPTIN1	XP_016878480.1	512	1	1.86	STYXL1	XP_016867786.1	679	2	2.80
ZNF48	NP_689865.2	213	0	0.00	MDH2	NP_005909.2	1362	3	2.09
ZNF771	NP_057727.2	180	0	0.00	SRRM3	NP_001103669.1	938	0	0.00
DCTPP1	NP_077001.1	462	1	2.06	HSPB1	NP_001531.1	1459	2	1.30

SEPHS2	NP_036380.2	291	1	3.26	YWHAG	NP_036611.2	2667	4	1.42
ITGAL	NP_002200.2	1059	3	2.69	SSC4D	NP_542782.1	68	0	0.00
ZNF768	XP_016879154.1	352	1	2.70	ZP3	NP_001103824.1	411	1	2.31
ZNF747	NP_001291948.1	116	0	0.00	DTX2	XP_016867212.1	318	1	2.99
ZNF764	NP_001166150.1	162	1	5.86	UPK3B	NP_001334613.1	312	1	3.04
ZNF688	XP_024305933.1	146	0	0.00	POMZP3	NP_694537.1	154	0	0.00
ZNF785	XP_016878456.1	176	0	0.00	CCDC146	NP_065930.2	106	0	0.00
ZNF689	NP_612456.1	204	1	4.66	FGL2	NP_006673.1	859	1	1.11
PRR14	NP_001307393.1	110	1	8.64	GSAP	NP_001337827.1	106	0	0.00
FBRS	NP_001098549.2	131	1	7.25	PTPN12	NP_002826.3	962	3	2.96
SRCAP	NP_006653.2	1447	4	2.63	RSBN1L	NP_940869.2	105	1	9.05
TMEM265	NP_001243758.1	1447	4	2.63	TMEM60	NP_116325.1	161	1	5.90
PHKG2	NP_001165903.1	395	1	2.40	PHTF2	NP_001353018.1	267	1	3.56
CCDC189	XP_016879342.1	54	1	17.59	MAGI2	XP_016868334.1	1089	2	1.74
RNF40	XP_011544299.1	863	6	6.60	GNAI1	NP_002060.4	1368	4	2.78
ZNF629	NP_001073886.1	657	4	5.78	GNAT3	XP_011514426.1	1118	5	4.25
BCL7C	NP_001273455.1	222	4	17.12	CD36	XP_024302770.1	763	2	2.49
CTF1	XP_011544061.1	386	3	7.38	SEMA3C	NP_001337049.1	596	1	1.59
FBXL19	NP_001369709.1	2424	12	4.70	HGF	NP_001010932.1	1516	5	3.13
ORAI3	NP_689501.1	253	0	0.00	CACNA2D1	XP_005250631.1	778	8	9.77
SETD1A	XP_016879398.1	2546	9	3.36	PCLO	XP_016867495.1	938	8	8.10
HSD3B7	NP_079469.2	473	0	0.00	SEMA3E	NP_036563.1	506	4	7.51
STX1B	NP_443106.1	1307	8	5.81	SEMA3A	XP_016867162.1	910	4	4.18
STX4	NP_001259025.1	757	4	5.02	SEMA3D	NP_001371831.1	470	4	8.08
ZNF668	NP_001166140.1	296	8	25.67	GRM3	NP_001350451.1	1199	8	6.34
ZNF646	NP_055514.3	205	5	23.17	ELAPOR2	XP_011514220.1	297	6	19.19
PRSS53	XP_011544121.1	215	5	22.09	DMTF1	XP_016868354.1	1074	9	7.96
VKORC1	NP_076869.1	272	6	20.95	TMEM243	NP_001316404.1	117	11	89.31
BCKDK	XP_016878348.1	729	6	7.82	CROT	NP_001137407.1	438	10	21.69
KAT8	NP_115564.2	996	3	2.86	ABCB4	XP_011514612.3	852	8	8.92
PRSS8	NP_002764.1	508	3	5.61	ABCB1	NP_001335874.1	1651	8	4.60
PRSS36	NP_001245219.1	122	1	7.79	RUNDC3B	NP_612147.1	235	8	32.34
FUS	NP_004951.1	1361	1	0.70	SLC25A40	NP_061331.2	271	9	31.55
PYCARD	NP_660183.1	752	3	3.79	DBF4	NP_006707.1	768	8	9.90
TRIM72	NP_001008275.2	590	1	1.61	ADAM22	XP_016867826.1	647	7	10.28
PYDC1	NP_690865.1	600	2	3.17	SRI	XP_011514830.1	383	2	4.96
ITGAM	NP_001139280.1	2176	4	1.75	STEAP4	NP_001192245.1	351	0	0.00
ITGAX	NP_001273304.1	1577	3	1.81	ZNF804B	NP_857597.1	145	1	6.55
ITGAD	XP_011544137.1	315	2	6.03	TEX47	NP_689919.1	49	0	0.00
COX6A2	NP_005196.1	549	0	0.00	STEAP1	NP_036581.1	247	3	11.54
ZNF843	NP_001340310.1	118	0	0.00	STEAP2	XP_016867447.1	249	3	11.45
ARMC5	NP_001275696.1	150	0	0.00	CFAP69	XP_011514879.1	106	0	0.00
TGFB1I1	NP_001035919.1	948	0	0.00	GTPBP10	NP_149098.2	574	5	8.27
SLC5A2	XP_006721135.2	409	1	2.32	CLDN12	NP_001172001.1	231	3	12.34
RUSF1	NP_073581.2	186	2	10.21	LOC10272389	NP_001352372.1	231	3	12.34
AHSP	NP_057717.1	238	0	0.00	CDK14	NP_001274064.1	1897	6	3.00
ZNF720	NP_001124385.1	171	0	0.00	FZD1	NP_003496.1	945	1	1.01
LOC10798399	XP_005255809.1	171	0	0.00	MTERF1	XP_005250650.1	414	10	22.95
ZNF267	NP_003405.4	244	0	0.00	AKAP9	XP_016867133.1	849	13	14.55
TP53TG3D	NP_001230651.1	81	0	0.00	CYP51A1	NP_000777.1	656	8	11.58
TP53TG3	NP_057296.1	85	0	0.00	LRRD1	NP_001371861.1	363	0	0.00
TP53TG3C	NP_001192188.1	81	0	0.00	KRIT1	NP_001337598.1	416	10	22.84
TP53TG3E	NP_001316990.1	81	0	0.00	ANKIB1	NP_061877.1	304	13	40.62
TP53TG3B	NP_001093157.1	81	0	0.00	GATAD1	NP_066990.3	205	14	64.87
TP53TG3F	NP_001316995.1	81	0	0.00	ERVW-1	NP_001124397.1	1049	3	2.72
SHCBP1	NP_001311247.1	524	6	10.88	PEX1	NP_001269606.1	692	8	10.98
VPS35	NP_060676.2	1269	4	2.99	RBM48	NP_115496.2	52	14	255.76
ORC6	NP_055136.1	605	7	10.99	FAM133B	NP_690002.2	117	14	113.67
MYLK3	NP_872299.2	676	2	2.81	CDK6	NP_001138778.1	2568	7	2.59
C16orf87	NP_001335589.1	63	0	0.00	SAMD9	NP_001180236.1	357	9	23.95
GPT2	NP_597700.1	763	7	8.72	SAMD9L	NP_001337011.1	463	8	16.41
DNAJA2	NP_005871.1	1216	11	8.59	HEPACAM2	NP_001275739.1	182	11	57.41
NETO2	XP_006721354.1	247	8	30.77	VPS50	NP_060137.2	302	9	28.31
ITFG1	NP_110417.2	199	12	57.28	CALCR	NP_001158210.1	637	14	20.88
PHKB	NP_000284.1	380	1	2.50	TFPI2	NP_001257932.1	734	11	14.24
ABCC12	NP_150229.2	474	8	16.03	GNGT1	NP_001316355.1	1237	4	3.07

ABCC11	XP_016879290.1	374	1	2.54	GNG11	NP_004117.1	927	7	7.17
LONP2	NP_001335007.1	731	2	2.60	BET1	NP_001304668.1	497	3	5.73
SIAH1	NP_001006611.1	1074	2	1.77	COL1A2	NP_000080.2	1299	5	3.66
N4BP1	XP_011521784.1	314	8	24.20	CASD1	XP_011514797.1	163	12	69.93
CBLN1	NP_004343.1	1028	10	9.24	SGCE	NP_001092870.1	437	12	26.09
C16orf78	NP_653203.1	18	5	263.88	PEG10	NP_001165908.1	480	11	21.77
ZNF423	NP_001366215.1	775	8	9.81	PPP1R9A	XP_011514682.1	728	13	16.96
CNEP1R1	NP_694993.2	133	6	42.85	PON1	NP_000437.3	616	6	9.25
HEATR3	NP_891552.1	343	7	19.39	PON3	NP_000931.1	291	9	29.38
TENT4B	XP_005256150.2	648	10	14.66	PON2	NP_001018171.1	400	10	23.75
ADCY7	XP_011521137.1	1166	7	5.70	ASB4	XP_016867792.1	1388	12	8.21
BRD7	XP_016878669.1	449	10	21.16	PDK4	NP_002603.1	1234	4	3.08
NKD1	NP_149110.1	577	9	14.82	DYNC1I1	NP_001265351.1	1063	11	9.83
SNX20	NP_001138444.1	412	6	13.83	SLC25A13	NP_055066.1	506	11	20.65
NOD2	NP_071445.1	1165	4	3.26	SEM1	XP_024302702.1	467	8	16.27
CYLD	NP_056062.1	851	5	5.58	DLX6	NP_005213.3	552	8	13.77
SALL1	NP_002959.2	772	10	12.31	DLX5	NP_005212.1	957	10	9.93
TOX3	XP_005255949.1	827	8	9.19	SDHAF3	NP_064571.1	249	1	3.82
CHD9	XP_016879207.1	1436	7	4.63	TAC1	NP_003173.1	1469	3	1.94
RBL2	NP_001310537.1	1058	11	9.88	ASNS	NP_001339425.1	997	3	2.86
AKTIP	XP_016879053.1	665	7	10.00	OCM2	NP_006179.2	1039	1	0.91
RPGRIP1L	NP_001121369.1	528	7	12.59	LMTK2	NP_055731.2	280	0	0.00
FTO	NP_001350820.1	772	8	9.84	BHLHA15	NP_803238.1	391	0	0.00
IRX3	NP_077312.2	757	6	7.53	TECPR1	XP_016867426.1	214	1	4.44
IRX5	NP_005844.4	496	6	11.49	BRI3	XP_016867420.1	136	0	0.00
IRX6	NP_077311.2	638	6	8.93	BAIAP2L1	NP_061330.2	357	0	0.00
MMP2	NP_004521.1	1950	0	0.00	NPTX2	NP_002514.1	595	2	3.19
LPCAT2	NP_060309.2	406	2	4.68	TMEM130	NP_001127923.1	666	1	1.43
CAPNS2	NP_115706.1	222	1	4.28	TRRAP	NP_001362453.1	2324	2	0.82
SLC6A2	NP_001165975.1	515	3	5.53	SMURF1	NP_001186776.1	1478	5	3.21
CES1	NP_001257.4	799	2	2.38	KPNA7	XP_016867700.1	794	2	2.39
CES5A	NP_001177087.1	490	1	1.94	ARPC1A	NP_006400.2	1139	2	1.67
GNAO1	NP_066268.1	1903	1	0.50	ARPC1B	XP_024302396.1	637	1	1.49
AMFR	NP_001135.3	822	1	1.16	PDAP1	NP_055706.1	312	1	3.04
NUDT21	NP_008937.1	1004	0	0.00	BUD31	NP_001357339.1	824	1	1.15
OGFOD1	NP_060703.3	386	0	0.00	PTCD1	NP_056360.2	438	0	0.00
BBS2	NP_114091.4	696	0	0.00	ATP5MF-PTCI	NP_001185808.1	587	0	0.00
MT4	NP_116324.2	350	0	0.00	CPSF4	NP_001305090.1	913	2	2.08
MT3	NP_005945.1	1168	1	0.81	ATP5MF	NP_001034267.1	728	0	0.00
MT2A	NP_005944.1	637	0	0.00	ZNF789	XP_016867507.1	232	0	0.00
MT1E	NP_783316.2	389	0	0.00	ZNF394	NP_001332897.1	272	0	0.00
MT1M	NP_789846.2	295	0	0.00	ZKSCAN5	NP_055384.1	268	6	21.27
MT1A	NP_005937.2	308	0	0.00	FAM200A	XP_024302449.1	18	0	0.00
MT1B	NP_005938.1	272	0	0.00	ZNF655	NP_001350261.1	288	0	0.00
MT1F	NP_005940.1	315	0	0.00	ZSCAN25	NP_001337914.1	225	4	16.89
MT1G	NP_005941.1	342	0	0.00	CYP3A5	NP_000768.1	599	2	3.17
MT1H	NP_005942.1	299	0	0.00	CYP3A7-CYP3	NP_001243426.2	466	3	6.12
MT1X	NP_005943.1	364	0	0.00	CYP3A7	NP_000756.3	466	3	6.12
NUP93	NP_055484.3	785	3	3.63	CYP3A4	NP_001189784.1	987	6	5.77
SLC12A3	NP_000330.3	497	1	1.91	CYP3A43	NP_001265850.1	357	3	7.98
HERPUD1	NP_055500.1	410	2	4.63	OR2AE1	NP_001005276.1	64	0	0.00
CETP	NP_000069.2	530	2	3.58	TRIM4	NP_149082.1	676	4	5.62
NLRC5	NP_001371879.1	551	2	3.45	GJC3	NP_853516.1	218	1	4.36
CPNE2	NP_689940.3	183	1	5.19	AZGP1	NP_001176.1	636	4	5.97
PSME3IP1	NP_001341027.1	240	2	7.92	ZKSCAN1	NP_001333508.1	335	4	11.34
RSPRY1	XP_024306250.1	158	2	12.02	ZSCAN21	NP_001349710.1	278	2	6.83
ARL2BP	NP_036238.1	189	3	15.08	ZNF3	NP_060185.2	342	3	8.33
PLLP	NP_057077.1	325	1	2.92	COPS6	NP_006824.2	1172	2	1.62
CCL22	NP_002981.2	586	2	3.24	MCM7	NP_005907.3	1962	3	1.45
CX3CL1	NP_002987.1	914	2	2.08	AP4M1	NP_001350600.1	604	0	0.00
CCL17	XP_016879019.1	543	2	3.50	TAF6	NP_001177344.1	707	3	4.03
CIAPIN1	NP_001295276.1	411	3	6.93	CNPY4	NP_689968.1	155	0	0.00
COQ9	NP_064708.1	651	0	0.00	MBLAC1	XP_005250307.1	63	1	15.08
POLR2C	NP_116558.1	1872	1	0.51	LAMTOR4	XP_024302528.1	247	1	3.85
DOK4	NP_001356550.1	349	0	0.00	MAP11	NP_060745.3	144	1	6.60
CCDC102A	NP_149989.2	141	2	13.47	GAL3ST4	NP_078913.3	100	1	9.50

ADGRG5	NP_722579.1	298	2	6.38	GPC2	NP_689955.1	485	2	3.92
ADGRG1	XP_005256294.1	696	4	5.46	STAG3	XP_016867172.1	630	5	7.54
ADGRG3	XP_011521256.1	479	2	3.97	PVRIG	NP_076975.2	118	5	40.25
DRC7	NP_115645.4	236	2	8.05	SPDYE3	XP_011514536.1	51	0	0.00
KATNB1	NP_005877.2	337	6	16.91	PILRB	NP_001358860.1	208	8	36.54
KIFC3	XP_011521377.1	1105	3	2.58	PILRA	XP_024302507.1	408	9	20.95
CNGB1	NP_001273059.1	502	1	1.89	ZCWPW1	NP_001372944.1	161	8	47.20
TEPP	NP_950247.3	111	0	0.00	MEPCE	NP_062552.2	529	9	16.16
ZNF319	NP_001371296.1	179	0	0.00	PPP1R35	XP_011514216.1	138	7	48.19
USB1	NP_078874.2	218	3	13.07	C7orf61	NP_001004323.1	69	7	96.37
MMP15	NP_002419.1	546	1	1.74	TSC22D4	NP_112197.1	274	7	24.27
CFAP20	NP_037374.1	329	1	2.89	NYAP1	NP_775835.2	746	12	15.28
CSNK2A2	XP_005255857.1	1950	3	1.46	AGFG2	XP_005250363.1	481	8	15.80
CCDC113	XP_011521340.1	180	3	15.83	SAP25	NP_001162153.2	110	1	8.64
PRSS54	NP_001073961.1	168	0	0.00	ZASP	NP_001276862.1	1052	1	0.90
GINS3	NP_001119601.1	432	5	10.99	LRCH4	NP_001276863.1	827	5	5.74
NDRG4	NP_001365261.1	902	4	4.21	FBXO24	XP_016867450.1	326	5	14.57
SETD6	NP_001153777.1	302	3	9.44	PCOLCE	NP_002584.2	409	1	2.32
CNOT1	NP_001252541.1	1423	4	2.67	MOSPD3	NP_076438.1	699	3	4.08
SLC38A7	NP_001356539.1	301	3	9.47	TFR2	NP_003218.2	353	3	8.07
GOT2	NP_001273149.1	1388	0	0.00	ACTL6B	NP_057272.1	2137	4	1.78
CDH8	XP_005255817.1	611	5	7.77	GNB2	NP_005264.2	1565	8	4.86
CDH11	XP_024305902.1	933	4	4.07	GIGYF1	NP_001362697.1	428	6	13.32
CDH5	XP_024305901.1	1331	2	1.43	POP7	NP_005828.2	587	2	3.24
BEAN1	XP_011521196.1	118	6	48.30	EPO	NP_000790.2	1384	3	2.06
TK2	NP_001166114.1	357	3	7.98	ZAN	NP_775082.2	399	1	2.38
CKLF	NP_001035228.1	132	4	28.79	EPHB4	NP_004435.3	1456	8	5.22
CKLF-CMTM1	NP_001191028.1	45	1	21.11	SLC12A9	NP_001350423.1	316	5	15.03
CMTM1	NP_443725.3	119	2	15.97	TRIP6	NP_003293.2	1024	7	6.49
CMTM2	NP_653274.1	244	5	19.47	SRRT	XP_024302561.1	1109	4	3.43
CMTM4	XP_016878443.1	223	7	29.82	UFSP1	NP_001015072.2	167	3	17.06
CMTM3	NP_001350847.1	278	5	17.09	ACHE	NP_001354848.1	1213	2	1.57
DYNC1LI2	NP_001310884.1	545	5	8.72	MUC3A	NP_005951.1	310	2	6.13
TERB1	NP_001129977.1	129	6	44.18	MUC12	NP_001157934.1	253	2	7.51
NAE1	NP_003896.1	1188	1	0.80	MUC17	NP_001035194.1	371	1	2.56
CA7	NP_005173.1	381	0	0.00	TRIM56	NP_112223.1	458	0	0.00
PDP2	NP_001316858.1	705	0	0.00	SERPINE1	NP_000593.1	1549	1	0.61
CDH16	NP_001191675.1	381	0	0.00	AP1S1	NP_001274.1	601	4	6.32
RRAD	NP_001122322.1	1536	0	0.00	VGF	NP_003369.2	599	1	1.59
CIAO2B	NP_057146.1	682	2	2.79	NAT16	NP_001356623.1	43	1	22.09
CES2	NP_001352334.1	600	0	0.00	MOGAT3	NP_001274076.1	235	0	0.00
CES3	NP_079198.2	424	0	0.00	PLOD3	NP_001075.1	382	2	4.97
CES4A	XP_011521323.1	332	1	2.86	ZNHIT1	NP_006340.1	496	2	3.83
CBFB	NP_074036.1	636	0	0.00	CLDN15	NP_001172009.1	197	1	4.82
C16orf70	XP_016879218.1	138	0	0.00	FIS1	NP_057152.2	761	4	4.99
B3GNT9	NP_171608.2	93	0	0.00	IFT22	NP_001124292.1	332	1	2.86
TRADD	NP_001310481.1	714	2	2.66	COL26A1	NP_001265492.1	285	2	6.67
FBXL8	NP_060848.2	340	2	5.59	MYL10	NP_612412.2	1165	1	0.82
HSF4	NP_001529.2	832	2	2.28	CUX1	NP_001904.2	1224	2	1.55
NOL3	NP_001263248.1	449	2	4.23	SH2B2	NP_066189.3	330	2	5.76
KIAA0895L	NP_001035805.1	5	0	0.00	SPDYE6	NP_001139682.2	9	0	0.00
EXOC3L1	NP_848611.2	452	7	14.71	PRKRIP1	NP_078929.1	241	4	15.77
E2F4	NP_001941.2	1274	2	1.49	ORAI2	NP_116220.1	269	6	21.19
ELMO3	NP_078988.3	420	5	11.31	ALKBH4	NP_060091.1	157	4	24.20
LRRC29	XP_016878619.1	479	6	11.90	LRWD1	NP_690852.1	566	1	1.68
TMEM208	NP_054906.2	275	0	0.00	POLR2J	XP_016867842.1	1259	0	0.00
FHOD1	NP_037373.2	510	4	7.45	RASA4B	NP_001264264.1	211	0	0.00
SLC9A5	NP_004585.1	417	0	0.00	UPK3BL2	NP_001350435.1	20	0	0.00
PLEKHG4	NP_001123199.1	348	7	19.11	SPDYE2	XP_016867712.1	121	8	62.81
KCTD19	NP_001094385.1	1416	7	4.70	POLR2J3	NP_001091084.2	616	8	12.34
LRRC36	NP_060766.5	104	0	0.00	RASA4	NP_001073346.2	281	0	0.00
TPPP3	XP_024306062.1	319	1	2.98	UPK3BL1	NP_001107875.1	20	0	0.00
ZDHHC1	XP_024306014.1	181	4	20.99	SPDYE2B	NP_001159811.1	7	0	0.00
HSD11B2	NP_000187.3	581	8	13.08	POLR2J2	NP_116581.3	595	0	0.00
ATP6V0D1	NP_004682.2	743	5	6.39	FAM185A	NP_001138740.2	37	1	25.67
AGRP	NP_001129.1	609	2	3.12	FBXL13	XP_016867339.1	867	13	14.24

RIPOR1	XP_011521623.1	180	1	5.28	LRRC17	XP_005250165.1	655	12	17.40
CTCF	XP_016878357.1	1834	2	1.04	ARMC10	XP_011514903.1	165	14	80.60
CARMIL2	XP_011521177.2	204	1	4.66	NAPEPLD	NP_001373134.1	323	12	35.29
ACD	XP_005256172.1	266	5	17.86	PMPCB	XP_006716244.1	1095	11	9.54
PARD6A	XP_005256034.1	678	3	4.20	DNAJC2	NP_001123359.1	1180	12	9.66
ENKD1	XP_024306237.1	51	0	0.00	PSMC2	NP_002794.1	1709	12	6.67
C16orf86	XP_005256009.1	0	0	#DIV/0!	SLC26A5	NP_996768.1	605	6	9.42
GFOD2	NP_110446.3	280	5	16.96	RELN	NP_774959.1	1316	6	4.33
RANBP10	NP_001307168.1	418	5	11.36	ORC5	NP_002544.1	1029	11	10.15
TSNAXIP1	XP_011521540.1	91	2	20.88	LHFPL3	NP_945351.1	442	12	25.79
CENPT	NP_079358.3	389	3	7.33	KMT2E	XP_024302605.1	977	9	8.75
THAP11	NP_065190.2	272	1	3.49	SRPK2	XP_011514838.1	808	11	12.93
NUTF2	NP_001308969.1	987	4	3.85	PUS7	NP_061915.2	569	4	6.68
EDC4	NP_055144.3	436	0	0.00	RINT1	XP_024302623.1	429	7	15.50
NRN1L	NP_001335611.1	123	0	0.00	EFCAB10	NP_001342460.1	36	1	26.39
PSKH1	NP_006733.1	542	3	5.26	ATXN7L1	NP_065776.1	417	1	2.28
CTRL	NP_001898.1	659	2	2.88	CDHR3	NP_689963.2	348	6	16.38
PSMB10	NP_002792.1	966	8	7.87	SYPL1	NP_001368842.1	258	4	14.73
LCAT	NP_000220.1	838	5	5.67	NAMPT	NP_005737.1	920	2	2.07
SLC12A4	NP_001139434.1	389	3	7.33	CCDC71L	NP_787080.2	140	2	13.57
DPEP3	NP_001123230.2	244	0	0.00	PIK3CG	NP_002640.2	2442	9	3.50
DPEP2	XP_024306144.1	519	1	1.83	PRKAR2B	NP_002727.2	1270	9	6.73
DDX28	NP_060850.2	1474	7	4.51	HBP1	NP_001231191.1	138	6	41.30
DUS2	NP_001258691.1	573	6	9.95	COG5	NP_001366445.1	470	10	20.21
NFATC3	NP_775188.1	566	2	3.36	GPR22	XP_011514358.1	386	7	17.23
ESRP2	XP_005256210.1	406	2	4.68	DUS4L	NP_853559.1	510	5	9.31
PLA2G15	XP_011521281.1	555	7	11.98	BCAP29	NP_001358286.1	555	9	15.40
SLC7A6	NP_003974.3	357	5	13.30	SLC26A4	NP_000432.1	527	1	1.80
SLC7A6OS	NP_115554.2	268	1	3.54	CBLL1	NP_079090.2	274	6	20.80
PRMT7	XP_016878781.1	683	6	8.35	SLC26A3	NP_000102.1	669	7	9.94
SMPD3	XP_016878894.1	286	0	0.00	DLD	NP_000099.2	1455	3	1.96
ZFP90	XP_016878441.1	284	7	23.41	LAMB1	NP_002282.2	884	6	6.45
CDH3	XP_011521102.1	960	3	2.97	LAMB4	XP_011514277.1	288	0	0.00
CDH1	NP_004351.1	3224	2	0.59	NRCAM	XP_024302547.1	1008	6	5.65
TANGO6	NP_078838.1	220	2	8.64	PNPLA8	XP_011514577.1	391	8	19.44
HAS3	NP_619515.1	623	0	0.00	THAP5	NP_001123947.1	119	4	31.93
CHTF8	NP_001353531.1	117	1	8.12	DNAJB9	NP_036460.1	690	5	6.88
UTP4	NP_116219.2	748	5	6.35	C7orf66	NP_001019778.1	0	0	#DIV/0!
SNTB2	NP_006741.1	527	2	3.61	IMMP2L	NP_001337888.1	649	10	14.64
VPS4A	NP_037377.1	446	1	2.13	LRRN3	NP_001093130.1	739	5	6.43
COG8	NP_001366194.1	768	1	1.24	DOCK4	NP_055520.3	1660	7	4.01
PDF	NP_071736.1	342	2	5.56	ZNF277	NP_068834.2	252	4	15.08
NIP7	NP_057185.1	1182	4	3.21	IFRD1	NP_001541.2	479	4	7.93
TMED6	NP_653277.2	214	4	17.76	LSMEM1	XP_016867517.1	142	2	13.38
TERF2	XP_005256181.1	509	2	3.73	TMEM168	XP_016868012.1	95	8	80.00
CYB5B	NP_085056.2	1128	2	1.68	BMT2	NP_689769.2	237	8	32.07
NFAT5	NP_619727.2	813	4	4.67	GPR85	XP_016867841.1	358	7	18.57
NQO1	NP_001273066.1	984	1	0.97	PPP1R3A	NP_002702.2	517	9	16.54
NOB1	NP_054781.1	754	4	5.04	FOXP2	XP_016868290.1	1318	13	9.37
WWP2	XP_016878368.1	1136	5	4.18	MDFIC	NP_951038.1	552	11	18.93
CLEC18A	XP_016878695.1	251	6	22.71	TFEC	XP_016867363.1	748	9	11.43
PDPR	XP_016878877.1	625	3	4.56	TES	NP_056456.1	789	4	4.82
CLEC18C	XP_024306007.1	232	3	12.28	CAV2	NP_937855.1	622	10	15.27
EXOSC6	NP_478126.1	777	4	4.89	CAV1	NP_001744.2	1908	8	3.98
AARS1	NP_001596.2	1169	6	4.88	MET	XP_011514525.1	2393	10	3.97
DDX19B	NP_001350867.1	657	1	1.45	CAPZA2	NP_006127.1	915	17	17.65
DDX19A	NP_060802.1	703	1	1.35	ST7	NP_001356527.1	149	4	25.50
ST3GAL2	NP_008858.1	354	4	10.73	WNT2	NP_003382.1	1399	15	10.19
FCSK	XP_016878501.1	291	4	13.06	ASZ1	NP_001288750.1	1141	14	11.66
COG4	NP_056201.2	467	6	12.20	CFTR	NP_000483.3	2024	9	4.22
SF3B3	NP_036558.3	1234	4	3.08	CTTNBP2	NP_219499.1	1215	13	10.16
IL34	NP_689669.2	335	0	0.00	LSM8	NP_057284.1	1057	0	0.00
MTSS2	XP_005256293.3	601	1	1.58	ANKRD7	NP_062618.2	892	1	1.06
VAC14	NP_060522.3	446	3	6.39	KCND2	NP_036413.1	1414	12	8.06
HYDIN	XP_006721269.1	518	1	1.83	TSPAN12	XP_016867402.1	226	12	50.44
CMTR2	NP_001311308.1	159	0	0.00	ING3	NP_061944.2	668	10	14.22

CALB2	NP_001731.2	1892	1	0.50	CPED1	NP_079189.4	157	9	54.46
ZNF23	NP_001368913.1	253	0	0.00	WNT16	NP_057171.2	846	5	5.61
ZNF19	NP_008892.2	182	0	0.00	FAM3C	NP_055703.1	374	8	20.32
CHST4	NP_001159867.1	196	1	4.85	PTPRZ1	NP_002842.2	1359	6	4.19
TAT	NP_000344.1	695	3	4.10	AASS	XP_011514027.1	558	1	1.70
MARVELD3	NP_001017967.2	186	8	40.86	FEZF1	NP_001153736.1	614	7	10.83
PHLPP2	NP_001275932.1	1550	8	4.90	CADPS2	NP_001350327.1	800	11	13.06
AP1G1	NP_001119.3	797	11	13.11	RNF133	NP_631914.1	137	4	27.74
ATXN1L	NP_001131147.1	226	11	46.24	RNF148	NP_932351.1	176	4	21.59
ZNF821	XP_016878902.1	213	11	49.06	TAS2R16	NP_058641.1	396	5	11.99
IST1	NP_001257905.1	523	11	19.98	SLC13A1	XP_016868043.1	432	5	10.99
PKD1L3	XP_024306022.1	292	10	32.53	IQUB	XP_016867261.1	214	5	22.20
DHODH	NP_001352.2	1070	11	9.77	NDUFA5	NP_001278233.1	671	5	7.08
TXNL4B	XP_016878866.1	746	11	14.01	ASB15	XP_011514118.1	1215	6	4.69
HP	NP_005134.1	1094	1	0.87	LMOD2	NP_997046.1	324	2	5.86
HPR	NP_066275.3	1094	0	0.00	WASL	NP_003932.3	1322	2	1.44
DHX38	NP_054722.2	1156	11	9.04	HYAL4	XP_016867400.1	285	2	6.67
PMFBP1	XP_011521659.1	121	9	70.66	SPAM1	NP_003108.2	433	2	4.39
ZFHX3	XP_024306059.1	655	7	10.15	TMEM229A	NP_001129474.1	163	0	0.00
PSMD7	NP_002802.2	1462	3	1.95	GPR37	NP_005293.1	952	2	2.00
NPIPB15	XP_011521798.1	17	0	0.00	POT1	NP_056265.2	302	0	0.00
CLEC18B	NP_001372124.1	205	0	0.00	GRM8	NP_001358015.1	1058	7	6.29
GLG1	NP_001139138.1	295	3	9.66	ZNF800	XP_006715932.1	270	2	7.04
RFWD3	NP_001357465.1	313	5	15.17	GCC1	NP_078799.2	316	6	18.04
MLKL	NP_001135969.1	1041	3	2.74	ARF5	NP_001653.1	1662	7	4.00
FA2H	NP_077282.3	1769	7	3.76	FSCN3	NP_065102.1	128	6	44.53
WDR59	NP_085058.3	2242	6	2.54	PAX4	NP_001353040.1	813	6	7.01
ZNRF1	XP_016879282.1	445	3	6.40	SND1	NP_055205.2	1255	7	5.30
LDHD	NP_919417.1	458	1	2.07	LRRC4	NP_071426.1	824	7	8.07
ZFP1	XP_011521223.1	257	1	3.70	LEP	NP_000221.1	2318	5	2.05
CTRB2	NP_001020371.3	274	1	3.47	RBM28	NP_001159607.1	909	10	10.45
CTRB1	NP_001316119.1	400	1	2.37	PRRT4	XP_016867694.1	133	9	64.28
BCAR1	NP_001164186.1	879	5	5.40	IMPDH1	XP_024302525.1	1224	5	3.88
CFDP1	NP_006315.1	538	2	3.53	HILPDA	NP_001092256.1	204	3	13.97
TMEM170A	NP_001291925.1	82	2	23.17	METTL2B	NP_060866.2	428	1	2.22
CHST6	NP_067628.1	151	2	12.58	FAM71F2	NP_001012457.3	26	0	0.00
CHST5	NP_078809.2	154	5	30.84	FAM71F1	NP_001269718.1	122	6	46.72
TMEM231	NP_001070884.2	188	2	10.11	CALU	NP_001186600.1	994	9	8.60
GABARAPL2	NP_009216.1	1163	2	1.63	OPN1SW	NP_001372054.1	359	3	7.94
ADAT1	NP_001311377.1	216	4	17.59	CCDC136	XP_011514786.1	207	2	9.18
KARS1	NP_005539.1	1556	2	1.22	FLNC	NP_001449.3	1280	6	4.45
TERF2IP	NP_061848.2	374	5	12.70	ATP6V1F	NP_001185838.1	636	8	11.95
CNTNAP4	NP_207837.2	1016	6	5.61	KCP	XP_016867677.1	287	1	3.31
MON1B	NP_001273569.1	532	4	7.14	IRF5	NP_001334857.1	919	6	6.20
SYCE1L	NP_001123451.1	98	1	9.69	TNPO3	NP_001369152.1	770	11	13.57
ADAMTS18	NP_955387.1	420	7	15.83	TSPAN33	NP_848657.1	225	10	42.22
NUDT7	NP_001230589.1	498	4	7.63	SMO	NP_005622.1	708	2	2.68
VAT1L	NP_065978.1	620	5	7.66	AHCYL2	NP_056143.1	772	9	11.07
CLEC3A	NP_005743.5	600	5	7.92	STRIP2	NP_065755.1	317	2	5.99
WWOX	NP_057457.1	882	5	5.39	SMKR1	XP_024302388.1	732	0	0.00
MAF	XP_016878722.1	1445	3	1.97	NRF1	NP_001280092.1	511	0	0.00
DYNLRB2	NP_001291946.1	424	4	8.96	UBE2H	NP_874356.1	929	10	10.23
CDYL2	XP_011521168.1	1382	4	2.75	ZC3HC1	NP_001350630.1	306	12	37.25
CMC2	XP_016878950.1	322	5	14.75	KLHDC10	NP_055812.1	555	14	23.96
CENPN	NP_001094095.2	679	3	4.20	TMEM209	NP_001288092.1	312	14	42.63
ATMIN	NP_056066.2	338	3	8.43	SSMEM1	NP_660311.1	68	13	181.61
C16orf46	NP_001094343.1	90	4	42.22	CPA2	NP_001860.2	448	12	25.45
GCSH	NP_004474.2	737	8	10.31	CPA4	NP_057436.2	547	11	19.10
PKD1L2	NP_001265354.1	277	3	10.29	CPA5	XP_024302767.1	334	13	36.97
BCO1	NP_059125.2	295	5	16.10	CPA1	NP_001859.1	518	11	20.17
GAN	NP_071324.1	696	0	0.00	CEP41	NP_001244088.1	414	12	27.53
CMIP	NP_938204.2	336	2	5.65	MEST	NP_002393.2	430	8	17.67
PLCG2	NP_002652.2	952	2	2.00	COPG2	NP_036265.3	898	14	14.81
SDR42E1	NP_660151.2	441	4	8.62	TSGA13	NP_443165.1	65	12	175.38
HSD17B2	NP_002144.1	436	2	4.36	KLF14	NP_619638.2	507	6	11.24
MPHOSPH6	NP_005783.2	402	1	2.36	MKLN1	NP_001138826.1	322	8	23.60

CDH13	NP_001207417.1	1079	5	4.40	PODXL	NP_005388.2	545	0	0.00
HSBP1	NP_001528.1	232	5	20.47	PLXNA4	XP_016868268.1	641	0	0.00
MLYCD	NP_036345.2	384	5	12.37	CHCHD3	NP_060282.1	468	2	4.06
OSGIN1	NP_892026.1	296	10	32.09	EXOC4	NP_068579.3	775	1	1.23
NECAB2	NP_061938.2	379	10	25.06	LRGUK	XP_024302427.1	1692	5	2.81
SLC38A8	XP_016878435.1	179	10	53.07	SLC35B4	XP_011514947.1	266	0	0.00
MBTPS1	NP_003782.1	842	8	9.03	AKR1B1	NP_001619.1	1017	1	0.93
HSDL1	NP_001139523.1	424	9	20.16	AKR1B10	NP_064695.3	680	1	1.40
DNAAF1	XP_011521156.1	631	7	10.54	AKR1B15	XP_016867713.1	362	1	2.62
TAF1C	XP_006721389.1	395	7	16.83	BPGM	NP_954655.1	539	0	0.00
ADAD2	NP_631913.3	140	7	47.50	CALD1	NP_149129.2	775	0	0.00
KCNG4	NP_758857.1	747	1	1.27	AGBL3	XP_016867629.1	352	2	5.40
WFDC1	XP_024306132.1	319	1	2.98	CYREN	NP_001292559.1	96	0	0.00
ATP2C2	NP_001273456.2	989	1	0.96	TMEM140	NP_060765.4	142	0	0.00
MEAK7	XP_016878999.1	116	0	0.00	WDR91	NP_054868.3	161	3	17.70
COTL1	NP_066972.1	663	2	2.87	STRA8	XP_011514439.1	395	2	4.81
KLHL36	NP_079007.2	148	1	6.42	CNOT4	XP_024302540.1	793	4	4.79
USP10	NP_005144.2	1168	0	0.00	NUP205	NP_055950.2	704	4	5.40
CRISPLD2	NP_113664.1	313	2	6.07	STMP1	NP_001124401.1	46	4	82.60
ZDHHC7	XP_024306111.1	239	2	7.95	SLC13A4	NP_036582.2	388	6	14.69
KIAA0513	XP_016879401.1	150	0	0.00	FAM180A	NP_001356626.1	98	7	67.85
CIBAR2	NP_001353849.1	159	1	5.97	LUZP6	NP_001122091.2	19	0	0.00
GSE1	XP_016878573.1	250	8	30.40	CHRM2	NP_001365902.1	779	3	3.66
GINS2	NP_057179.1	739	9	11.57	PTN	NP_001308316.1	1105	5	4.30
C16orf74	NP_996850.1	80	8	95.00	DGKI	NP_001308637.1	661	6	8.62
EMC8	NP_006058.1	342	11	30.55	CREB3L2	NP_919047.2	465	3	6.13
COX4I1	XP_024305924.1	1122	11	9.31	AKR1D1	NP_005980.1	447	4	8.50
IRF8	NP_001350836.1	1301	9	6.57	TRIM24	NP_056989.2	978	8	7.77
FOXF1	NP_001442.2	856	12	13.32	SVOPL	NP_001132928.1	335	13	36.86
MTHFSD	XP_016879061.2	272	7	24.45	ATP6V0A4	XP_005250450.1	723	8	10.51
FOXC2	NP_005242.1	1016	12	11.22	TMEM213	XP_005250224.1	167	12	68.26
FOXL1	NP_005241.1	793	13	15.57	KIAA1549	XP_011514744.1	283	12	40.28
C16orf95	NP_001182053.1	45	9	189.99	ZC3HAV1L	XP_011514990.1	100	12	113.99
FBXO31	NP_079011.3	392	6	14.54	ZC3HAV1	NP_064504.2	665	10	14.28
MAP1LC3B	NP_073729.1	1324	4	2.87	TTC26	NP_001308669.1	290	12	39.31
ZCCHC14	NP_055959.2	541	11	19.32	UBN2	XP_011514305.1	369	13	33.47
JPH3	NP_065706.2	1114	6	5.12	FMC1-LUC7L2	NP_001231513.1	671	17	24.07
KLHDC4	NP_001171783.1	325	4	11.69	FMC1	NP_932068.2	383	12	29.76
SLC7A5	NP_003477.4	630	5	7.54	KLRG2	XP_011514443.1	250	13	49.40
CA5A	NP_001354154.1	306	2	6.21	CLEC2L	NP_001073980.2	760	6	7.50
BANP	XP_016878867.1	287	6	19.86	HIPK2	NP_001106710.1	1300	13	9.50
ZNF469	XP_016879273.1	334	1	2.84	TBXAS1	XP_011514846.1	649	9	13.17
ZFPM1	XP_011521216.1	586	2	3.24	PARP12	NP_073587.1	285	5	16.67
ZC3H18	NP_001281269.1	530	0	0.00	KDM7A	NP_085150.1	814	6	7.00
IL17C	NP_037410.1	300	0	0.00	SLC37A3	NP_001350306.1	300	3	9.50
CYBA	NP_000092.2	605	3	4.71	RAB19	XP_016867696.1	618	5	7.69
MVD	NP_002452.1	475	4	8.00	MKRN1	XP_011514298.1	541	7	12.29
SNAI3	NP_840101.1	250	2	7.60	DENND2A	XP_011514356.1	140	1	6.79
RNF166	NP_001165286.1	147	5	32.31	ADCK2	NP_443085.2	349	11	29.94
CTU2	NP_001305436.1	446	1	2.13	NDUFB2	NP_004537.1	620	4	6.13
PIEZO1	NP_001136336.2	348	0	0.00	BRAF	NP_001365403.1	1456	6	3.91
CDT1	NP_112190.2	1010	0	0.00	MRPS33	NP_444263.1	598	5	7.94
APRT	NP_000476.1	1235	7	5.38	TMEM178B	XP_016867125.1	220	3	12.95
GALNS	NP_000503.1	432	5	10.99	AGK	XP_024302603.1	800	8	9.50
TRAPPC2L	NP_001305453.1	341	2	5.57	DENND11	XP_024302611.1	164	0	0.00
PABPN1L	NP_001372638.1	344	0	0.00	WEE2	NP_001099028.1	917	2	2.07
CBFA2T3	XP_005256380.1	582	1	1.63	SSBP1	NP_001243439.1	1100	4	3.45
ACSF3	NP_777577.2	637	1	1.49	TAS2R3	NP_058639.1	327	5	14.53
CDH15	NP_004924.1	697	6	8.18	TAS2R4	NP_058640.1	346	4	10.98
SLC22A31	NP_001371700.1	198	1	4.80	TAS2R5	NP_061853.1	329	4	11.55
ZNF778	NP_001365810.1	222	3	12.84	PRSS37	NP_001165422.1	206	5	23.06
ANKRD11	XP_016878671.1	336	4	11.31	OR9A4	NP_001001656.1	57	2	33.33
SPG7	NP_003110.1	1462	11	7.15	CLEC5A	XP_011514297.1	671	4	5.66
RPL13	NP_150254.1	1119	1	0.85	TAS2R38	NP_789787.5	390	1	2.44
CPNE7	XP_016878629.1	185	10	51.35	MGAM	XP_016868261.1	2519	5	1.89
DPEP1	XP_016878498.1	435	10	21.84	MGAM2	NP_001280555.1	230	1	4.13

CHMP1A	NP_001076783.1	722	12	15.79	PRSS58	NP_001001317.1	350	4	10.86
SPATA33	NP_694570.1	91	14	146.15	MTRNR2L6	NP_001177416.1	72	0	0.00
CDK10	XP_011521709.1	509	14	26.13	PRSS1	XP_011514713.1	591	1	1.61
SPATA2L	NP_689552.2	134	16	113.43	PRSS2	NP_001290343.1	522	1	1.82
VPS9D1	XP_011521778.1	61	17	264.74	EPHB6	XP_024302442.1	1173	4	3.24
ZNF276	NP_001106997.1	241	18	70.95	TRPV6	NP_061116.5	559	6	10.20
FANCA	NP_000126.2	729	13	17.83	TRPV5	NP_062815.3	504	4	7.54
SPIRE2	NP_115827.1	296	17	60.62	LLCFC1	NP_001369425.1	57	4	66.66
TCF25	XP_011521262.1	404	17	47.03	KEL	NP_000411.1	325	7	20.46
MC1R	NP_002377.4	856	14	19.42	OR9A2	NP_001001658.1	59	0	0.00
TUBB3	NP_006077.2	1825	11	7.63	OR6V1	NP_001001667.1	38	0	0.00
DEF8	XP_016878850.1	218	14	87.15	PIP	NP_002643.1	229	2	8.30
CENPBD1	NP_659476.2	129	13	147.28	TAS2R39	NP_795362.2	287	1	3.31
DBNDD1	NP_001036075.1	178	12	106.74	TAS2R40	NP_795363.1	300	0	0.00
GAS8	NP_001472.1	364	9	42.71	GSTK1	NP_001137151.1	392	0	0.00
PRDM7	XP_016878372.1	172	7	77.32	TMEM139	NP_699176.1	54	1	17.59
SCGB1C2	XP_011533664.1	130	0	0.00	CASP2	NP_116764.2	962	2	1.97
DOC2B	XP_011533665.1	352	5	24.53	CLCN1	NP_000074.3	497	3	5.73
RPH3AL	NP_001177341.1	240	5	32.98	FAM131B	NP_001358177.1	719	2	2.64
C17orf97	NP_001013694.4	59	0	0.00	ZYX	NP_003452.1	1024	1	0.93
RFLNB	NP_874364.1	137	0	0.00	EPHA1	XP_006715943.1	1522	1	0.62
VPS53	NP_001121631.1	599	3	6.34	TAS2R60	NP_803186.1	313	1	3.03
TLCD3A	NP_079068.1	140	8	67.85	TAS2R41	NP_795364.2	307	0	0.00
GEMIN4	NP_056536.2	462	5	12.10	CTAGE15	NP_001008747.1	54	0	0.00
GLOD4	NP_057164.3	677	5	7.80	TCAF2	NP_001352358.1	212	1	4.48
MRM3	NP_060616.1	612	10	16.34	CTAGE6	NP_848656.2	67	2	28.36
NXN	XP_005256813.1	374	6	15.24	TCAF1	NP_055534.2	126	1	7.54
TIMM22	NP_037469.2	674	8	11.28	OR2F2	NP_001004685.1	35	0	0.00
ABR	XP_016880029.1	467	2	4.07	OR2F1	XP_011514321.1	113	0	0.00
BHLHA9	NP_001157877.1	120	9	71.25	OR6B1	NP_001005281.1	97	1	9.79
TRARG1	NP_758955.2	324	11	32.25	OR2A5	NP_036497.1	37	0	0.00
YWHAE	NP_006752.1	2871	9	2.98	OR2A25	NP_001373025.1	31	0	0.00
CRK	NP_058431.2	1185	8	6.41	OR2A12	NP_001004135.1	46	0	0.00
MYO1C	NP_001074248.1	1380	11	7.57	OR2A2	NP_001005480.2	62	0	0.00
INPP5K	NP_057616.2	466	5	10.19	OR2A14	NP_001001659.1	67	0	0.00
PITPNA	XP_011522241.1	610	10	15.57	CTAGE4	NP_940897.2	33	3	86.36
SLC43A2	XP_016879668.1	247	1	3.85	ARHGEF35	NP_001355247.1	428	3	6.66
SCARF1	NP_003684.2	357	8	21.29	OR2A42	NP_001001802.2	72	0	0.00
RILP	XP_005256868.1	226	1	4.20	OR2A7	NP_001005328.1	71	3	40.14
PRPF8	XP_024306305.1	1572	4	2.42	CTAGE8	NP_001265436.1	33	3	86.36
TLCD2	NP_001157879.1	49	1	19.39	OR2A1	NP_001005287.1	82	0	0.00
WDR81	NP_001157145.1	476	8	15.97	ARHGEF5	NP_005426.2	681	5	6.97
SERPINF2	XP_016880254.1	583	3	4.89	NOBOX	XP_016867231.1	657	3	4.34
SERPINF1	NP_001316832.1	689	8	11.03	TPK1	NP_001035947.1	424	4	8.96
SMYD4	XP_024306328.1	1505	5	3.16	CNTNAP2	NP_054860.1	867	3	3.29
RPA1	NP_002936.1	1143	2	1.66	C7orf33	NP_660347.1	26	3	109.61
RTN4RL1	NP_848663.1	756	4	5.03	CUL1	NP_001357591.1	2288	3	1.25
DPH1	NP_001333503.1	541	5	8.78	EZH2	XP_024302448.1	3126	9	2.73
OVCA2	NP_543012.1	288	4	13.19	PDIA4	NP_004902.1	1291	0	0.00
HIC1	NP_001091672.1	702	7	9.47	ZNF786	NP_689624.2	146	1	6.51
SMG6	NP_060045.4	820	9	10.43	ZNF425	NP_001001661.1	173	1	5.49
SRR	NP_068766.1	673	7	9.88	ZNF398	NP_733787.1	271	1	3.51
TSR1	NP_060598.3	1087	6	5.24	ZNF282	XP_006716214.1	230	1	4.13
SGSM2	NP_001333629.1	528	7	12.59	ZNF212	NP_036388.2	250	1	3.80
MNT	NP_064706.1	497	10	19.11	ZNF783	NP_001182149.1	164	1	5.79
METTL16	NP_076991.3	191	7	34.81	ZNF777	XP_005250037.1	346	2	5.49
PAFAH1B1	XP_016880192.1	1301	8	5.84	ZNF746	NP_689770.3	275	1	3.45
CLUH	NP_056044.4	654	6	8.72	KRBA1	XP_011514938.1	71	5	66.90
RAP1GAP2	XP_011522040.1	465	1	2.04	ZNF467	NP_001316785.1	292	1	3.25
OR1D5	NP_055381.1	58	0	0.00	ZNF862	NP_001092690.1	306	1	3.10
OR1D2	NP_001373017.1	73	1	13.01	ATP6V0E2	NP_001276919.1	281	5	16.90
OR1G1	NP_003546.1	82	0	0.00	ACTR3C	NP_001337957.1	1059	3	2.69
OR1A2	NP_036484.1	133	0	0.00	ZBED6CL	NP_612443.1	55	6	103.63
OR1A1	NP_001373033.1	91	0	0.00	LRRC61	NP_076431.1	565	6	10.09
OR3A2	XP_016880186.1	81	0	0.00	RARRES2	NP_002880.1	364	4	10.44
OR3A1	XP_016880184.1	54	0	0.00	REPIN1	NP_001349674.1	451	8	16.85

OR1E1	NP_003544.2	79	3	36.07	ZNF775	NP_775951.2	240	6	23.75
OR3A3	NP_001373027.1	45	0	0.00	GIMAP8	NP_783161.1	528	3	5.40
OR1E2	NP_003545.1	69	3	41.30	GIMAP7	NP_694968.1	487	3	5.85
SPATA22	NP_001164167.1	207	6	27.53	GIMAP4	NP_001350461.1	446	2	4.26
ASPA	XP_016880150.1	385	7	17.27	GIMAP6	NP_078987.3	429	4	8.86
TRPV3	NP_659505.1	337	4	11.28	GIMAP2	NP_056475.1	177	2	10.73
TRPV1	NP_542435.2	922	8	8.24	GIMAP1-GIM/	NP_001186506.1	501	2	3.79
SHPK	NP_037408.2	782	9	10.93	GIMAP1	NP_570115.1	187	2	10.16
CTNS	NP_001026851.2	300	4	12.67	GIMAP5	NP_060854.2	501	2	3.79
TAX1BP3	NP_001191627.1	294	4	12.92	TMEM176B	NP_001349621.1	358	11	29.19
EMC6	NP_112588.1	398	1	2.39	TMEM176A	XP_011514678.1	252	9	33.93
P2RX5	NP_778255.1	221	5	21.49	AOC1	XP_016867433.1	433	2	4.39
ITGAE	XP_011522130.1	872	1	1.09	KCNH2	NP_000229.1	900	2	2.11
HASPIN	NP_114171.2	620	1	1.53	NOS3	NP_000594.2	2086	2	0.91
NCBP3	XP_005256774.1	358	2	5.31	ABCB8	NP_001269222.1	392	2	4.85
CAMKK1	NP_757343.2	731	1	1.30	ASIC3	NP_064718.1	350	2	5.43
P2RX1	XP_006721592.1	742	4	5.12	CDK5	NP_001157882.1	1496	1	0.63
ATP2A3	XP_011522187.1	1256	2	1.51	SLC4A2	NP_003031.3	463	5	10.26
ZZEF1	NP_055928.3	545	3	5.23	FASTK	XP_011514065.1	276	0	0.00
CYB5D2	NP_653212.1	263	0	0.00	TMUB1	NP_113622.1	202	0	0.00
ANKFY1	NP_001316992.1	1290	5	3.68	AGAP3	NP_114152.3	522	2	3.64
UBE2G1	NP_003333.1	1727	2	1.10	GBX1	NP_001092304.1	262	0	0.00
SPNS3	NP_872344.3	374	2	5.08	ASB10	NP_543147.2	1263	3	2.26
SPNS2	NP_001118230.1	346	4	10.98	IQCA1L	NP_001291348.1	581	1	1.64
MYBBP1A	NP_001099008.1	1022	4	3.72	ABCF2	NP_009120.1	975	3	2.92
GGT6	XP_024306340.1	257	3	11.09	CHPF2	NP_001271224.1	236	2	8.05
SMTNL2	NP_001108446.1	451	5	10.53	SMARCD3	NP_003069.2	920	3	3.10
ALOX15	NP_001131.3	453	3	6.29	NUB1	XP_016867793.1	395	1	2.40
PELP1	NP_055204.4	480	2	3.96	WDR86	XP_011514447.1	291	0	0.00
ARRB2	NP_001244257.1	1520	3	1.87	CRYGN	NP_653328.1	124	0	0.00
MED11	NP_001001683.1	254	0	0.00	RHEB	NP_005605.1	1955	4	1.94
CXCL16	NP_071342.2	764	2	2.49	PRKAG2	XP_016867758.1	1334	4	2.85
ZMYND15	NP_001254751.1	1500	4	2.53	GALNTL5	XP_016867282.1	184	3	15.49
TM4SF5	NP_003954.2	269	0	0.00	GALNT11	NP_001291443.1	338	4	11.24
VMO1	NP_872372.1	138	2	13.77	KMT2C	NP_733751.2	2049	7	3.25
GLTPD2	NP_001362730.1	88	1	10.79	XRCC2	NP_005422.1	346	0	0.00
PSMB6	NP_002789.1	1167	3	2.44	ACTR3B	XP_016867949.1	1081	6	5.27
PLD2	NP_002654.3	1334	4	2.85	DPP6	NP_001351426.1	1152	10	8.25
MINK1	XP_006721594.1	741	3	3.85	PAXIP1	XP_011514284.1	852	5	5.57
CHRNE	XP_016879604.1	600	9	14.25	HTR5A	NP_076917.1	1383	8	5.50
C17orf107	NP_001139008.1	0	0	#DIV/0!	INSIG1	NP_001333519.1	775	7	8.58
GP1BA	NP_000164.5	826	3	3.45	EN2	NP_001418.2	728	9	11.74
SLC25A11	NP_001158889.1	692	8	10.98	CNPY1	NP_001356742.1	173	12	65.89
RNF167	NP_001357233.1	250	8	30.40	RBM33	XP_005249588.1	151	16	100.66
PFN1	NP_005013.1	1012	1	0.94	SHH	NP_001297391.1	2340	12	4.87
ENO3	NP_001361453.1	1385	6	4.12	RNF32	XP_016867242.1	309	12	36.89
SPAG7	NP_004881.2	119	7	55.88	LMBR1	NP_001350341.1	344	13	37.79
CAMTA2	NP_001164638.1	297	9	28.79	NOM1	NP_001340295.1	532	12	23.81
INCA1	XP_024306514.1	77	1	12.34	MNX1	NP_005506.3	492	9	20.44
KIF1C	NP_006603.2	1038	10	9.15	UBE3C	NP_055486.2	1070	13	14.43
SLC52A1	NP_001098047.1	222	7	29.95	DNAJB6	NP_490647.1	692	9	16.47
ZFP3	NP_694563.1	222	0	0.00	PTPRN2	NP_570857.2	996	8	10.90
ZNF232	NP_001307884.1	255	8	29.80	NCAPG2	XP_016867865.1	787	5	9.29
USP6	XP_016880779.1	1065	5	4.46	ESYT2	XP_024302614.1	344	7	32.22
ZNF594	NP_115919.1	254	0	0.00	DYNC2I1	XP_006716104.1	325	10	53.14
SCIMP	NP_001258771.1	157	0	0.00	VIPR2	NP_001291451.1	427	6	26.70
RABEP1	XP_011522362.1	737	0	0.00	OR4F21	NP_001005504.1	64	0	0.00
NUP88	NP_002523.2	481	2	3.95	ZNF596	XP_016868655.1	218	9	71.31
RPAIN	NP_001028174.2	151	2	12.58	FBXO25	XP_016868802.1	322	10	49.17
C1QBP	NP_001203.1	1693	3	1.68	TDRP	NP_001243042.1	131	9	100.41
DHX33	NP_064547.2	999	4	3.80	ERICH1	XP_016868613.1	150	9	81.42
DERL2	NP_001291706.1	848	0	0.00	DLGAP2	NP_001333739.1	1127	10	11.24
MIS12	XP_016880522.1	285	1	3.33	CLN8	NP_061764.2	373	9	28.65
NLRP1	NP_001028225.1	423	1	2.25	ARHGEF10	XP_016869492.1	400	10	27.94
WSCD1	XP_024306450.1	336	0	0.00	KBTBD11	XP_016869603.1	167	8	50.56
AIPL1	NP_001028227.1	340	1	2.79	MYOM2	NP_003961.3	800	8	10.00

PIMREG	NP_001182157.1	410	2	4.63	CSMD1	XP_011533054.1	721	9	11.86
PITPNM3	NP_001159438.1	521	4	7.29	MCPH1	XP_016869322.1	658	7	10.11
KIAA0753	XP_011522398.1	212	4	17.92	ANGPT2	XP_016868807.1	969	3	2.94
TXNDC17	NP_116120.1	282	0	0.00	AGPAT5	NP_060831.2	506	2	3.75
MED31	NP_057144.1	484	4	7.85	XKR5	NP_997294.3	41	0	0.00
C17orf100	NP_001098990.1	0	0	#DIV/0!	DEFB1	NP_005209.1	426	7	15.61
SLC13A5	NP_001271439.1	475	2	4.00	DEFA6	NP_001917.1	309	4	12.30
XAF1	NP_001340064.1	441	3	6.46	DEFA4	NP_001916.1	258	4	14.73
FBXO39	NP_694962.1	512	1	1.86	DEFA1	XP_011533042.1	378	4	10.05
TEKT1	NP_444515.1	441	0	0.00	DEFA1B	NP_001289194.1	351	6	16.24
ALOX12	XP_011522082.1	425	2	4.47	DEFA3	XP_011533043.1	209	7	31.82
RNASEK	NP_001004333.3	80	1	11.87	DEFA5	NP_066290.1	373	8	20.37
C17orf49	NP_001136270.1	102	0	0.00	USP17L1	NP_001243802.1	89	12	128.08
BCL6B	NP_862827.2	381	0	0.00	USP17L4	NP_001243803.1	60	7	110.83
SLC16A13	NP_963860.1	188	2	10.11	ZNF705G	NP_001157929.1	110	0	0.00
SLC16A11	XP_016879771.1	173	3	16.47	DEFB4B	NP_001192195.1	280	15	50.89
CLEC10A	NP_001316999.1	1133	1	0.84	DEFB103B	NP_061131.1	369	11	28.32
ASGR2	XP_024306523.1	427	2	4.45	SPAG11B	NP_478109.1	106	11	98.58
ASGR1	NP_001184145.1	639	2	2.97	DEFB104B	NP_001035792.1	75	10	126.66
DLG4	NP_001308003.1	2563	3	1.11	DEFB106B	NP_001035794.1	129	9	66.28
ACADVL	NP_001257376.1	733	3	3.89	DEFB105B	NP_001035793.1	84	9	101.78
DVL2	XP_005256559.1	1335	4	2.85	DEFB107B	NP_001035795.1	91	9	93.95
PHF23	XP_024306706.1	585	1	1.62	PRR23D1	NP_001269408.1	27	14	492.57
GABARAP	NP_009209.1	1010	7	6.58	PRR23D2	NP_001269407.1	1	0	0.00
CTDNEP1	NP_001137247.1	508	8	14.96	DEFB107A	NP_001032757.2	87	8	87.35
ELP5	NP_981959.1	169	2	11.24	DEFB105A	NP_689463.1	72	7	92.36
CLDN7	NP_001298.3	499	2	3.81	DEFB106A	NP_689464.1	135	8	56.29
SLC2A4	NP_001033.1	1582	5	3.00	DEFB104A	NP_525128.2	84	8	90.47
YBX2	NP_057066.2	627	5	7.58	SPAG11A	NP_001075021.2	64	10	148.43
EIF5A	XP_024306398.1	1402	1	0.68	DEFB103A	NP_001075020.1	373	7	17.83
GPS2	NP_004480.1	396	6	14.39	DEFB4A	NP_004933.1	473	9	18.08
NEURL4	NP_001005408.1	611	3	4.66	ZNF705B	NP_001180559.1	137	7	48.54
ACAP1	NP_055531.1	779	7	8.54	USP17L8	NP_001243801.1	59	6	96.61
KCTD11	NP_001350571.1	302	5	15.73	USP17L3	NP_001243800.1	54	4	70.37
TMEM95	XP_016880054.1	47	0	0.00	PRAG1	NP_001356688.1	214	1	4.44
TNK1	NP_001238831.1	405	3	7.04	CLDN23	NP_919260.2	205	2	9.27
PLSCR3	NP_001356349.1	483	2	3.93	MFHAS1	NP_004216.2	504	7	13.19
TMEM256	NP_689979.1	404	3	7.05	ERI1	NP_001341567.1	441	3	6.46
NLGN2	NP_065846.1	915	2	2.08	PPP1R3B	XP_016869324.1	586	8	12.97
SPEM1	NP_955371.2	176	2	10.79	TNKS	NP_003738.2	1533	9	5.58
SPEM2	NP_783861.3	1	0	0.00	MSRA	NP_036463.1	783	11	13.35
TMEM102	NP_001307373.1	169	0	0.00	PRSS55	NP_001183949.1	258	6	22.09
FGF11	NP_004103.1	571	4	6.65	RP1L1	NP_849188.4	344	6	16.57
CHRNB1	NP_000738.2	566	3	5.04	C8orf74	NP_001035121.2	48	10	197.91
ZBTB4	NP_065950.2	385	1	2.47	SOX7	NP_113627.1	527	10	18.03
SLC35G6	NP_001096084.1	69	0	0.00	PINX1	NP_060354.4	620	8	12.26
POLR2A	NP_000928.1	2790	5	1.70	XKR6	NP_775954.2	224	12	50.89
TNFSF12-TNFS	NP_742086.1	78	1	12.18	MTMR9	NP_056273.2	497	14	26.76
TNFSF12	NP_003800.1	310	2	6.13	SLC35G5	NP_473369.1	140	2	13.57
TNFSF13	NP_742085.1	435	4	8.74	FAM167A	XP_024303060.1	276	8	27.53
SENP3	NP_056485.2	927	5	5.12	BLK	XP_011542126.1	1398	7	4.76
EIF4A1	NP_001407.1	2150	6	2.65	GATA4	XP_005272442.1	1924	9	4.44
CD68	NP_001242.2	1595	5	2.98	NEIL2	NP_001129220.1	274	10	34.67
MPDU1	XP_006721660.1	215	4	17.67	FDFT1	NP_001274671.1	938	7	7.09
SOX15	NP_008873.1	548	7	12.13	CTSB	NP_001371654.1	1552	5	3.06
FXR2	NP_004851.2	1014	7	6.56	DEFB136	NP_001028190.2	28	0	0.00
SAT2	NP_001307775.1	183	3	15.57	DEFB135	NP_001028189.2	66	8	115.15
SHBG	NP_001031.2	686	4	5.54	DEFB134	XP_016869213.1	45	9	189.99
ATP1B2	NP_001669.3	923	6	6.18	DEFB130B	NP_001182186.1	100	0	0.00
TP53	NP_000537.3	6196	12	1.84	ZNF705D	NP_001034704.2	137	0	0.00
WRAP53	NP_060551.2	481	4	7.90	USP17L7	NP_001243798.1	43	0	0.00
EFNB3	NP_001397.1	934	3	3.05	USP17L2	NP_958804.2	233	4	16.31
DNAH2	XP_011521965.1	702	6	8.12	FAM86B1	XP_006716323.1	139	3	20.50
KDM6B	NP_001073893.1	3611	3	0.79	DEFB130A	NP_001032893.1	137	0	0.00
TMEM88	NP_001306870.1	211	4	18.01	FAM86B2	NP_001131082.1	149	3	19.13
NAA38	NP_001317040.1	919	1	1.03	LONRF1	NP_001316905.1	560	6	10.18

CYB5D1	NP_653208.2	725	2	2.62	TRMT9B	XP_011542898.1	802	6	7.11
CHD3	NP_001005273.1	1457	3	1.96	DLC1	NP_001335010.1	391	6	14.58
KCNAB3	XP_016880794.1	508	2	3.74	C8orf48	NP_001007091.2	21	4	180.94
TRAPPC1	NP_067033.1	485	2	3.92	SGCZ	NP_001309808.1	266	9	32.14
CNTROB	XP_016879617.1	208	1	4.57	TUSC3	NP_839952.1	616	7	10.79
GUCY2D	NP_000171.1	846	8	8.98	MSR1	NP_001350673.1	606	1	1.57
ALOX15B	NP_001132.2	237	5	20.04	FGF20	NP_062825.1	608	5	7.81
ALOX12B	NP_001130.1	292	6	19.52	MICU3	NP_859074.1	214	6	26.63
ALOXE3	NP_001159432.1	353	7	18.84	ZDHHC2	XP_011542846.1	395	7	16.83
HES7	XP_016880721.1	384	8	19.79	CNOT7	NP_001309023.1	662	4	5.74
PER1	XP_005256747.1	404	4	9.41	VPS37A	XP_016868510.1	329	3	8.66
VAMP2	NP_055047.2	1332	1	0.71	MTMR7	NP_004677.3	381	3	7.48
TMEM107	NP_001338209.1	151	6	37.75	SLC7A2	NP_001158243.1	484	5	9.81
BORCS6	NP_060092.2	135	2	14.07	PDGFRL	NP_001359002.1	471	10	20.17
AURKB	NP_001300882.1	1944	3	1.47	MTUS1	NP_001349989.1	262	5	18.13
CTC1	XP_011522312.1	228	10	41.66	FGL1	XP_016868721.1	547	3	5.21
PFAS	XP_024306572.1	893	6	6.38	PCM1	XP_024302935.1	619	5	7.67
RANGRF	NP_057576.2	244	3	11.68	ASAH1	NP_004306.3	506	0	0.00
SLC25A35	NP_001307800.1	168	0	0.00	NAT1	XP_016869436.1	236	0	0.00
ARHGEF15	XP_011522036.1	548	0	0.00	NAT2	XP_016868427.1	303	0	0.00
ODF4	NP_694552.2	84	1	11.31	PSD3	NP_056125.3	639	4	5.95
KRBA2	NP_998762.1	140	0	0.00	SH2D4A	NP_001167631.1	240	1	3.96
RPL26	NP_000978.1	1349	5	3.52	CSGALNACT1	XP_016869112.1	394	4	9.64
RNF222	NP_001140156.1	74	0	0.00	INTS10	NP_001340434.1	398	5	11.93
NDEL1	XP_016880672.1	769	5	6.18	LPL	NP_000228.1	1127	0	0.00
MYH10	XP_011522182.1	2009	6	2.84	SLC18A1	XP_011542927.1	387	5	12.27
CCDC42	NP_001151733.1	151	1	6.29	ATP6V1B2	NP_001684.2	919	3	3.10
SPDYE4	NP_001121548.1	25	1	38.00	LZTS1	NP_066300.1	292	4	13.01
MFSD6L	NP_689812.3	112	1	8.48	GFRA2	NP_001158511.1	589	8	12.90
PIK3R6	XP_011521980.1	507	1	1.87	DOK2	XP_005273737.1	823	5	5.77
PIK3R5	XP_005256637.1	742	2	2.56	XPO7	NP_001093631.1	353	4	10.76
NTN1	NP_004813.2	1017	2	1.87	NPM2	XP_011542662.1	450	7	14.78
STX8	NP_004844.1	632	2	3.01	FGF17	XP_011542985.1	860	7	7.73
CFAP52	NP_659491.4	540	4	7.04	DMTN	NP_001310307.1	475	7	14.00
USP43	NP_694942.3	792	9	10.79	FAM160B2	XP_006716444.1	46	0	0.00
DHRS7C	NP_001099041.1	260	3	10.96	NUDT18	XP_011542952.1	173	0	0.00
GSG1L2	NP_001297148.1	0	0	#DIV/0!	HR	NP_060881.2	214	2	8.88
GLP2R	XP_011522379.1	381	2	4.99	REEP4	NP_001303893.1	802	0	0.00
RCVRN	NP_002894.1	1145	8	6.64	LGI3	NP_644807.1	812	7	8.19
GAS7	NP_958839.1	1759	10	5.40	SFTPC	NP_003009.2	465	2	4.09
MYH13	NP_003793.2	1039	2	1.83	BMP1	NP_006120.1	928	2	2.05
MYH8	NP_002463.2	1329	4	2.86	PHYHIP	NP_001350240.1	982	10	9.67
MYH4	XP_016880165.1	1334	4	2.85	POLR3D	NP_001713.2	594	1	1.60
MYH1	XP_016880164.1	1551	6	3.67	PIWIL2	NP_001129193.1	703	2	2.70
MYH2	NP_001093582.1	1209	6	4.71	SLC39A14	XP_016868782.1	378	1	2.51
MYH3	XP_011522172.1	1273	4	2.98	PPP3CC	NP_001230903.1	3016	6	1.89
SCO1	NP_004580.1	617	0	0.00	SORBS3	XP_006716329.1	634	1	1.50
ADPRM	NP_064618.3	127	0	0.00	PDLIM2	NP_067643.3	629	2	3.02
TMEM220	NP_001346576.1	70	0	0.00	C8orf58	NP_001013864.1	10	0	0.00
PIRT	NP_001094857.1	676	1	1.41	CCAR2	NP_066997.3	775	6	7.35
SHISA6	NP_997269.2	692	9	12.35	BIN3	NP_061158.1	827	5	5.74
DNAH9	XP_011522005.1	889	13	13.89	EGR3	NP_004421.2	709	4	5.36
ZNF18	XP_016880497.1	220	10	43.18	PEBP4	NP_659399.2	465	4	8.17
MAP2K4	NP_001268364.1	945	3	3.02	RHOBTB2	XP_016868740.1	1783	9	4.80
MYOCD	NP_001139784.1	729	7	9.12	TNFRSF10B	NP_671716.2	1024	3	2.78
ARHGAP44	NP_055674.4	373	9	22.92	TNFRSF10C	NP_003832.3	366	3	7.79
ELAC2	NP_001159434.1	911	8	8.34	TNFRSF10D	NP_003831.2	366	4	10.38
HS3ST3A1	NP_006033.1	224	8	33.93	TNFRSF10A	NP_003835.3	1041	3	2.74
COX10	NP_001294.2	859	13	14.38	CHMP7	NP_689485.1	150	9	57.00
CDRT15	NP_001007531.1	37	11	282.42	R3HCC1	NP_001129580.2	226	2	8.41
HS3ST3B1	NP_006032.1	243	9	35.18	LOXL2	NP_002309.1	489	4	7.77
PMP22	NP_696996.1	407	2	4.67	ENTPD4	NP_001122402.1	299	2	6.35
TEKT3	XP_016880443.1	410	7	16.22	SLC25A37	NP_057696.2	452	2	4.20
CDRT4	NP_001191406.1	33	4	115.15	NKX3-1	NP_001243268.1	667	6	8.55
TVP23C-CDRT4	NP_001191407.1	93	3	30.64	NKX2-6	NP_001129743.2	310	2	6.13
TVP23C	NP_660344.2	123	4	30.89	STC1	NP_003146.1	693	7	9.60

CDRT1	NP_001269469.1	269	7	24.72	ADAM28	XP_016868463.1	515	4	7.38
TRIM16	NP_001335050.1	202	6	28.22	ADAMDEC1	NP_055294.1	404	5	11.76
ZNF286A	NP_001275571.1	124	8	61.29	ADAM7	NP_003808.2	341	3	8.36
TBC1D26	NP_848666.2	204	0	0.00	NEFM	NP_005373.2	1240	5	3.83
ADORA2B	XP_011521961.1	825	1	1.15	NEFL	NP_006149.2	1492	5	3.18
ZSWIM7	NP_001036163.1	110	0	0.00	DOCK5	NP_079216.4	578	4	6.57
TTC19	XP_016880290.2	707	3	4.03	GNRH1	NP_000816.4	1213	6	4.70
NCOR1	NP_006302.2	2538	8	2.99	KCTD9	NP_060104.2	210	2	9.05
PIGL	XP_016880843.1	335	1	2.84	CDCA2	XP_011542717.1	585	1	1.62
CENPV	NP_859067.2	369	0	0.00	EBF2	NP_073150.2	611	5	7.77
UBB	NP_061828.1	3233	6	1.76	PPP2R2A	NP_002708.1	1226	6	4.65
TRPV2	NP_057197.2	586	3	4.86	BNIP3L	NP_004322.1	528	1	1.80
LRRC75A	NP_001107039.1	80	0	0.00	PNMA2	NP_009188.1	495	6	11.51
ZNF287	NP_001333099.1	179	1	5.31	DPYSL2	NP_001184222.1	942	9	9.08
ZNF624	XP_011522272.1	172	2	11.05	ADRA1A	NP_001309432.1	781	4	4.87
CCDC144A	XP_024306813.1	924	4	4.11	STMN4	NP_001269983.1	1340	5	3.54
TNFRSF13B	NP_036584.1	508	4	7.48	TRIM35	NP_741983.2	442	3	6.45
MPRIP	XP_011522066.1	434	6	13.13	PTK2B	XP_016868703.1	1233	5	3.85
PLD6	XP_016879799.2	497	6	11.47	CHRNA2	NP_001269384.1	636	4	5.97
FLCN	XP_011522021.1	460	6	12.39	EPHX2	NP_001970.2	1458	2	1.30
COPS3	NP_001303284.1	747	14	17.80	CLU	NP_001822.3	1416	4	2.68
NT5M	XP_011522269.1	332	9	25.75	SCARA3	XP_016869025.1	432	3	6.60
MED9	NP_060489.1	203	11	51.48	CCDC25	NP_001291459.1	185	2	10.27
RASD1	NP_057168.1	1817	14	7.32	ESCO2	XP_011542724.1	811	3	3.51
PEMT	NP_001254480.1	445	3	6.40	PBK	NP_001349969.1	1813	3	1.57
RAI1	NP_109590.3	1499	8	5.07	SCARA5	NP_776194.2	283	3	10.07
SREBF1	XP_024306663.1	1391	8	5.46	NUGGC	NP_001010906.1	69	2	27.53
TOM1L2	NP_001275718.1	561	14	23.71	ELP3	NP_060561.3	953	3	2.99
DRC3	XP_024306729.1	648	15	21.99	PNOC	XP_005273589.1	588	2	3.23
ATPAF2	XP_011522367.1	544	16	27.94	ZNF395	NP_061130.1	308	4	12.34
GID4	NP_076957.3	210	13	58.81	FBXO16	NP_001245140.1	317	2	5.99
DRG2	XP_005256556.1	790	15	18.04	FZD3	NP_665873.1	713	1	1.33
MYO15A	XP_016880204.1	1187	12	9.60	EXTL3	XP_024302862.1	299	0	0.00
ALKBH5	NP_060228.3	301	0	0.00	INTS9	NP_001138631.1	523	2	3.63
LLGL1	XP_011522151.1	754	11	13.86	HMBOX1	NP_001311312.1	268	1	3.54
FLII	NP_001243194.1	1034	8	7.35	KIF13B	XP_005273515.1	834	2	2.28
MIEF2	NP_683684.2	233	5	20.39	DUSP4	NP_001385.1	1062	1	0.89
TOP3A	NP_004609.1	1164	8	6.53	SARAF	NP_057211.4	393	2	4.83
SMCR8	NP_658988.2	273	8	27.84	LEPROTL1	NP_001121680.1	230	2	8.26
SHMT1	XP_024306655.1	1328	7	5.01	MBOAT4	XP_016869215.1	296	1	3.21
EVPLL	NP_001138599.1	439	1	2.16	DCTN6	NP_006562.1	476	1	2.00
LGALS9C	NP_001035167.2	187	0	0.00	RBPMS	XP_016868476.1	533	4	7.13
FAM106A	NP_079250.1	0	0	#DIV/0!	GTF2E2	XP_016868852.1	976	4	3.89
TBC1D28	XP_016879911.1	226	3	12.61	SMIM18	XP_016868432.1	4	0	0.00
TRIM16L	NP_001340150.1	320	3	8.91	GSR	NP_001182033.1	1585	1	0.60
FBXW10	NP_001254514.1	568	3	5.02	UBXN8	XP_011542957.1	368	1	2.58
TVP23B	NP_057162.4	159	3	17.92	PPP2CB	NP_001009552.1	2203	5	2.16
PRPSAP2	NP_001340027.1	693	4	5.48	TEX15	NP_001337091.1	313	0	0.00
SLC5A10	XP_016879680.2	219	3	13.01	PURG	NP_001310241.1	200	1	4.75
FAM83G	XP_016880442.1	89	3	32.02	WRN	NP_000544.2	1339	4	2.84
GRAP	NP_001317077.1	1379	7	4.82	NRG1	NP_001153471.1	1114	1	0.85
GRAPL	XP_016880126.1	730	1	1.30	FUT10	XP_011542979.1	154	3	18.51
EPN2	NP_055779.2	677	6	8.42	MAK16	NP_115898.2	992	7	6.70
B9D1	NP_001308148.1	363	2	5.23	TTI2	NP_001317434.1	315	10	30.16
MAPK7	XP_006721620.1	2329	6	2.45	RNF122	NP_079063.2	402	7	16.54
MFAP4	NP_002395.1	567	3	5.03	DUSP26	NP_076930.1	1331	10	7.14
RNF112	XP_006721634.1	1336	0	0.00	UNC5D	XP_016868533.1	508	8	14.96
SLC47A1	NP_060712.2	608	7	10.94	KCNU1	NP_001027006.2	477	7	13.94
ALDH3A2	NP_001356075.1	1036	4	3.67	ZNF703	NP_079345.1	345	10	27.53
SLC47A2	NP_001093116.1	519	7	12.81	ERLIN2	XP_024302826.1	482	14	27.59
ALDH3A1	NP_001128639.1	949	6	6.01	PLPBP	NP_001336278.1	469	15	30.38
ULK2	NP_055498.3	668	6	8.53	ADGRA2	XP_011542783.1	785	16	19.36
AKAP10	NP_001317081.1	267	5	17.79	BRF2	NP_060780.2	418	8	18.18
SPECC1	NP_001373012.1	499	0	0.00	RAB11FIP1	NP_079427.4	355	15	40.14
LGALS9B	NP_001036150.1	122	0	0.00	GOT1L1	XP_006716348.1	392	17	41.20
CDRT15L2	NP_001177719.1	6	0	0.00	ADRB3	NP_000016.1	748	8	10.16

USP22	NP_056091.1	694	4	5.48	EIF4EBP1	NP_004086.1	1078	10	8.81
DHRS7B	XP_005256652.2	221	9	38.69	ASH2L	XP_005273739.1	875	15	16.28
TMEM11	NP_003867.1	457	5	10.39	STAR	NP_000340.2	493	1	1.93
NATD1	NP_690878.2	66	7	100.75	LSM1	NP_055277.1	803	19	22.48
MAP2K3	NP_659731.1	798	6	7.14	BAG4	NP_004865.1	533	16	28.52
KCNJ12	NP_066292.2	494	5	9.61	DDHD2	NP_001349840.1	383	18	44.65
KCNJ18	XP_005276976.1	171	0	0.00	PLPP5	NP_001341385.1	371	17	43.53
MTRNR2L1	NP_001177381.1	47	1	20.21	NSD3	NP_075447.1	1817	15	7.84
WSB1	NP_056441.6	957	0	0.00	LETM2	XP_016868540.1	332	16	45.78
KSR1	XP_011523731.1	1381	1	0.69	FGFR1	XP_011542751.1	1864	11	5.61
LGALS9	NP_033665.1	734	1	1.29	TACC1	XP_005273686.1	329	13	37.54
NOS2	NP_000616.3	1590	2	1.19	PLEKHA2	NP_067636.1	230	13	53.69
LYRM9	XP_024306404.1	96	2	19.79	HTRA4	NP_710159.1	424	12	26.89
NLK	NP_057315.3	633	0	0.00	TM2D2	NP_510882.1	169	13	73.07
TMEM97	XP_005258022.1	305	3	9.34	ADAM9	NP_003807.1	663	10	14.33
IFT20	NP_001254706.1	415	2	4.58	ADAM32	NP_001300923.1	337	10	28.19
TNFAIP1	XP_016880482.1	341	3	8.36	ADAM18	NP_055052.1	155	3	18.39
POLDIP2	NP_001277074.1	286	4	13.29	ADAM2	XP_011542782.1	487	2	3.90
TMEM199	NP_689677.1	283	4	13.43	IDO1	NP_002155.1	828	0	0.00
SEBOX	NP_001074306.3	503	1	1.89	IDO2	NP_919270.2	273	0	0.00
VTN	NP_000629.3	1152	1	0.82	TCIM	NP_064515.2	275	3	10.36
SARM1	NP_055892.2	436	4	8.72	ZMAT4	NP_001129203.1	301	2	6.31
SLC46A1	XP_005277843.1	405	1	2.35	SFRP1	NP_003003.3	1118	2	1.70
SLC13A2	XP_011523752.1	519	2	3.66	GOLGA7	NP_001349909.1	251	6	22.71
FOXN1	XP_011523660.1	668	3	4.27	GINS4	NP_115712.1	493	2	3.85
UNC119	NP_005139.1	707	1	1.34	GPAT4	NP_001350126.1	446	3	6.39
PIGS	NP_149975.1	129	0	0.00	NKX6-3	NP_001351770.1	257	4	14.79
ALDOC	NP_005156.1	1044	0	0.00	ANK1	XP_011542806.1	1809	5	2.63
SPAG5	NP_006452.3	605	5	7.85	KAT6A	XP_011542961.1	1105	3	2.58
RSKR	NP_001167574.1	2417	2	0.79	AP3M2	XP_016868466.1	412	10	23.06
KIAA0100	NP_001308489.1	234	1	4.06	PLAT	NP_127509.1	975	3	2.92
SDF2	NP_008854.2	393	8	19.34	IKBKB	XP_011542822.1	1956	8	3.89
SUPT6H	NP_001307684.1	1417	9	6.03	POLB	XP_005273592.1	698	4	5.44
PROCA1	NP_689678.1	197	6	28.93	DKK4	XP_016868805.1	314	6	18.15
RAB34	NP_001136097.2	681	1	1.39	VDAC3	NP_001129166.1	1007	10	9.43
RPL23A	NP_000975.2	1439	1	0.66	SLC20A2	XP_016869238.1	685	11	15.25
TLCD1	NP_001153879.1	87	2	21.84	SMIM19	NP_001350115.1	13	7	511.51
NEK8	NP_835464.1	437	5	10.87	CHRNB3	NP_000740.1	515	13	23.98
TRAF4	XP_011523806.1	878	9	9.74	CHRNA6	NP_001186208.1	688	13	17.95
FAM222B	NP_001070966.1	33	5	143.93	THAP1	NP_060575.1	542	13	22.78
ERAL1	NP_005693.1	572	1	1.66	RNF170	NP_001153696.1	369	13	33.47
FLOT2	NP_004466.2	548	6	10.40	HOOK3	NP_115786.1	312	15	45.67
DHRS13	NP_653284.2	260	3	10.96	FNTA	NP_002018.1	481	15	29.62
PHF12	NP_001028733.1	754	4	5.04	POMK	NP_115613.1	213	12	53.52
SEZ6	XP_011522617.1	544	7	12.22	HGSNAT	XP_005273466.1	357	12	31.93
PIPOX	NP_057602.2	551	2	3.45	SPIDR	XP_016868757.1	136	8	55.88
TIAF1	NP_004731.2	206	2	9.22	CEBPD	NP_005186.2	998	9	8.57
MYO18A	NP_001333696.1	1153	5	4.12	PRKDC	NP_001075109.1	2455	10	3.87
CRYBA1	XP_016879687.1	316	4	12.02	MCM4	NP_877423.1	1653	8	4.60
NUFIP2	NP_065823.1	374	7	17.78	UBE2V2	XP_016869297.1	1543	4	2.46
TAOK1	NP_065842.1	713	7	9.33	EFCAB1	NP_001350903.1	850	1	1.12
ABHD15	NP_937790.2	173	2	10.98	SNAI2	NP_003059.1	1602	3	1.78
TP53I13	NP_612358.3	129	1	7.36	PPDPFL	NP_001007177.1	58	0	0.00
GIT1	XP_024306502.1	945	4	4.02	SNTG1	NP_001308702.1	554	1	1.71
ANKRD13B	XP_024306338.1	86	0	0.00	PXDNL	NP_653252.4	302	2	6.29
CORO6	XP_024306777.1	842	2	2.26	PCMTD1	NP_001273711.1	352	4	10.79
SSH2	NP_203747.2	833	9	10.26	ST18	NP_001339805.1	301	3	9.47
EFCAB5	XP_011523061.1	115	9	74.34	ALKAL1	NP_997296.1	127	0	0.00
NSRP1	XP_011523647.1	251	5	18.92	RB1CC1	XP_011515947.1	830	3	3.43
SLC6A4	NP_001036.1	1021	7	6.51	NPBWR1	NP_005276.2	519	2	3.66
BLMH	NP_000377.1	468	11	22.33	OPRK1	NP_001305426.1	988	3	2.88
TMIGD1	NP_996663.1	248	0	0.00	ATP6V1H	NP_998784.1	559	3	5.10
CPD	NP_001295.2	287	4	13.24	RGS20	NP_733466.1	580	3	4.91
GOSR1	NP_004862.1	678	4	5.60	TCEA1	NP_958845.1	1107	2	1.72
TBC1D29P	XP_011522898.1	219	1	4.34	LYPLA1	XP_016868445.1	663	7	10.03
CRLF3	NP_057070.3	228	14	58.33	MRPL15	NP_054894.1	1127	2	1.69

ATAD5	XP_011523571.1	751	13	16.44	SOX17	NP_071899.1	1006	1	0.94
TEFM	NP_078959.3	309	13	39.97	RP1	XP_016869210.1	533	0	0.00
ADAP2	NP_001333641.1	867	15	16.44	XKR4	NP_443130.1	237	8	32.07
RNF135	NP_115698.3	411	15	34.67	TMEM68	NP_689630.1	193	8	39.38
NF1	NP_001035957.1	2356	15	6.05	TGS1	NP_079107.6	407	8	18.67
OMG	NP_002535.3	1026	13	12.04	LYN	XP_011515831.1	1905	6	2.99
EVI2B	XP_005258003.1	531	13	23.26	RPS20	NP_001139699.1	1699	14	7.83
EVI2A	NP_001003927.1	356	14	37.36	MOS	NP_005363.1	1118	7	5.95
RAB11FIP4	NP_116321.2	525	15	27.14	PLAG1	XP_016869065.1	721	15	19.76
COPRS	NP_060875.2	201	13	61.44	CHCHD7	NP_001011668.1	365	17	44.24
UTP6	NP_060898.2	982	15	14.51	SDR16C5	NP_001304979.1	389	14	34.19
SUZ12	NP_056170.2	1275	15	11.18	PENK	NP_001129162.1	1030	14	12.91
LRRC37B	NP_443120.2	132	13	93.56	BPNT2	NP_060283.3	434	4	8.76
RHOT1	XP_011523273.2	1677	7	3.97	FAM110B	XP_016869437.1	195	10	48.72
RHBDL3	NP_001350764.1	2174	5	2.18	UBXN2B	NP_001071087.1	297	6	19.19
C17orf75	NP_071739.2	124	1	7.66	CYP7A1	NP_000771.2	809	5	5.87
ZNF207	NP_001091977.1	661	8	11.50	SDCBP	NP_001335270.1	988	11	10.58
PSMD11	NP_002806.2	1234	10	7.70	NSMAF	NP_003571.2	406	10	23.40
CDK5R1	XP_016880770.1	997	7	6.67	TOX	XP_016869574.1	864	13	14.29
MYO1D	NP_056009.1	1145	10	8.30	CA8	NP_001308768.1	446	10	21.30
TMEM98	NP_001288675.1	120	6	47.50	RAB2A	NP_002856.1	1720	7	3.87
SPACA3	NP_776246.1	254	3	11.22	CHD7	XP_016869101.1	2235	8	3.40
ASIC2	NP_001085.2	1075	5	4.42	CLVS1	XP_016868630.1	316	4	12.02
CCL2	NP_002973.1	2221	5	2.14	ASPH	XP_005251303.1	512	8	14.84
CCL7	NP_006264.2	735	4	5.17	NKAIN3	XP_016868848.1	70	1	13.57
CCL11	NP_002977.1	755	4	5.03	GGH	NP_003869.1	815	3	3.50
CCL8	NP_005614.2	530	5	8.96	TTPA	XP_006716531.1	468	4	8.12
CCL13	NP_005399.1	472	5	10.06	YTHDF3	NP_689971.4	458	0	0.00
CCL1	NP_002972.1	583	5	8.15	BHLHE22	NP_689627.1	841	0	0.00
TMEM132E	NP_001291367.1	521	2	3.65	CYP7B1	NP_001311041.1	658	2	2.89
CCT6B	NP_001180459.1	1081	4	3.52	ARMC1	XP_005251321.1	379	4	10.03
ZNF830	NP_443089.3	278	1	3.42	MTFR1	XP_011515928.1	176	5	26.99
LIG3	XP_005258027.1	1354	3	2.10	PDE7A	NP_001229247.1	506	9	16.90
RFFL	NP_001017368.1	240	2	7.92	DNAJC5B	NP_001336361.1	454	5	10.46
RAD51D	NP_598332.1	1299	2	1.46	TRIM55	NP_908973.1	489	7	13.60
FNDC8	NP_060029.1	3	0	0.00	CRH	NP_000747.1	1062	0	0.00
NLE1	NP_060566.2	955	3	2.98	RRS1	NP_055984.1	1180	4	3.22
UNC45B	NP_775259.1	661	1	1.44	ADHFE1	NP_653251.2	832	7	7.99
SLC35G3	NP_689675.1	47	0	0.00	VXN	NP_689978.2	156	6	36.54
SLFN5	NP_659412.3	179	1	5.31	MYBL1	NP_001281211.1	1387	9	6.16
SLFN11	NP_001098057.1	183	3	15.57	VCPIP1	NP_079330.2	435	9	19.65
SLFN12	XP_016880298.1	112	3	25.45	SGK3	NP_037389.4	1416	7	4.70
SLFN13	XP_016879721.1	129	3	22.09	MCMDC2	XP_011515771.1	668	0	0.00
SLFN12L	NP_001350759.1	166	3	17.17	TCF24	XP_016868429.1	72	0	0.00
SLFN14	XP_016880066.1	166	2	11.45	PPP1R42	NP_001351841.1	580	5	8.19
PEX12	NP_000277.1	383	2	4.96	COPS5	NP_006828.2	1964	3	1.45
AP2B1	XP_011522750.1	1167	4	3.26	CSPP1	XP_011515900.1	355	2	5.35
RASL10B	XP_016880789.1	1344	1	0.71	ARFGEF1	XP_005251193.1	935	8	8.13
GAS2L2	NP_644814.1	359	1	2.65	CPA6	NP_065094.3	260	2	7.31
MMP28	XP_011523533.1	309	2	6.15	PREX2	NP_079146.2	740	2	2.57
C17orf50	NP_660315.2	1	0	0.00	C8orf34	NP_443190.2	81	1	11.73
TAF15	NP_631961.1	695	0	0.00	SULF1	XP_006716501.1	663	3	4.30
HEATR9	NP_001308324.1	87	2	21.84	SLCO5A1	NP_001139481.1	233	6	24.46
CCL5	NP_001265665.1	1727	8	4.40	PRDM14	NP_078780.1	586	5	8.11
RDM1	NP_001156592.1	257	1	3.70	NCOA2	NP_001308640.1	931	5	5.10
LYZL6	NP_001186880.1	119	0	0.00	TRAM1	NP_055109.1	420	2	4.52
CCL16	NP_004581.1	396	6	14.39	LACTB2	NP_057111.1	350	6	16.28
CCL14	NP_116739.1	265	7	25.09	XKR9	XP_011515829.1	74	6	77.02
CCL15	NP_116741.2	233	4	16.31	EYA1	NP_001357265.1	925	6	6.16
CCL23	NP_665905.2	369	7	18.02	MSC	NP_005089.2	322	2	5.90
CCL18	NP_002979.1	544	6	10.48	TRPA1	XP_011515926.1	1273	2	1.49
CCL3	NP_002974.1	1168	6	4.88	KCNB2	NP_004761.2	846	2	2.25
CCL4	NP_002975.1	1335	7	4.98	TERF1	XP_005251348.1	709	1	1.34
TBC1D3B	NP_001001417.6	337	4	11.28	SBSPON	NP_694957.3	137	0	0.00
CCL3L3	NP_001001437.2	442	6	12.90	C8orf89	NP_001230166.1	0	0	#DIV/0!
CCL4L2	NP_001278397.1	498	6	11.45	RPL7	NP_000962.2	1491	3	1.91

TBC1D3I	XP_006722289.1	260	5	18.27	RDH10	NP_742034.1	429	0	0.00
TBC1D3G	XP_005276971.1	227	10	41.85	STAU2	NP_001157853.1	366	3	7.79
TBC1D3H	XP_006722119.1	224	10	42.41	UBE2W	NP_001257944.2	1348	3	2.11
TBC1D3F	NP_115634.3	289	7	23.01	ELOC	NP_001191786.1	1574	3	1.81
ZNHIT3	NP_001268361.1	352	15	40.48	TMEM70	NP_060336.3	340	1	2.79
MYO19	NP_079385.2	1095	16	13.88	LY96	XP_016868788.1	692	1	1.37
PIGW	NP_001333683.1	292	17	55.31	JPH1	NP_001349979.1	397	4	9.57
GGNBP2	XP_005257746.1	223	18	76.68	GDAP1	NP_001349860.1	440	4	8.64
DHRS11	NP_077284.2	443	16	34.31	PI15	NP_001311332.1	257	2	7.39
MRM1	XP_011523577.1	638	18	26.80	CRISPLD1	NP_113649.1	218	2	8.72
LHX1	NP_005559.2	1032	16	14.73	HNF4G	NP_004124.5	507	2	3.75
AATF	NP_036270.1	890	15	16.01	ZFHX4	NP_078997.4	432	1	2.20
ACACA	XP_011523003.1	1328	15	10.73	PEX2	NP_000309.2	371	2	5.12
C17orf78	NP_775896.3	85	17	189.99	PKIA	XP_016869109.1	282	1	3.37
TADA2A	NP_001479.4	837	17	19.29	ZC2HC1A	NP_001349898.1	153	2	12.42
DUSP14	XP_005257034.1	651	16	23.35	IL7	XP_011515825.1	1137	2	1.67
SYNRG	XP_016879591.1	1100	16	13.82	STMN2	NP_008960.2	1487	5	3.19
DDX52	NP_008941.3	870	12	13.10	HEY1	NP_036390.3	858	1	1.11
HNF1B	NP_001291215.1	611	7	10.88	MRPS28	NP_054737.1	386	0	0.00
TBC1D3K	XP_006722298.1	203	0	0.00	TPD52	NP_005070.1	437	2	4.35
TBC1D3L	NP_001356430.1	203	0	0.00	ZBTB10	NP_001264074.1	465	0	0.00
TBC1D3D	XP_006722311.1	362	6	15.75	ZNF704	XP_016869214.1	143	0	0.00
TBC1D3C	XP_006721970.1	365	6	15.62	PAG1	XP_016869129.1	389	1	2.44
TBC1D3E	XP_006722317.1	239	0	0.00	FABP5	NP_001435.1	717	4	5.30
TBC1D3	NP_001116863.3	353	5	13.46	PMP2	NP_002668.1	801	5	5.93
MRPL45	NP_115727.5	640	14	20.78	FABP9	NP_001073995.1	159	1	5.97
GPR179	NP_001004334.3	229	3	12.44	FABP4	NP_001433.1	1051	3	2.71
SOCS7	NP_055413.2	403	7	16.50	FABP12	NP_001098751.1	123	6	46.34
ARHGAP23	XP_006722054.1	366	12	31.15	IMPA1	NP_001138350.1	678	6	8.41
SRCIN1	NP_079524.2	522	9	16.38	SLC10A5	NP_001010893.1	250	6	22.80
EPOP	NP_001124149.1	105	1	9.05	ZFAND1	NP_001164267.1	277	6	20.58
MLLT6	NP_005928.2	635	10	14.96	CHMP4C	NP_689497.1	438	6	13.01
CISD3	NP_001129970.1	190	10	50.00	SNX16	NP_001335118.1	301	9	28.40
PCGF2	XP_016880505.1	662	10	14.35	RALYL	NP_001341252.1	1503	2	1.26
PSMB3	NP_002786.2	1498	11	6.98	LRRCC1	XP_016869410.1	573	1	1.66
PIP4K2B	NP_003550.1	758	13	16.29	E2F5	NP_001942.2	962	0	0.00
CWC25	NP_060218.1	663	12	17.19	RBIS	NP_001280249.1	120	1	7.92
C17orf98	NP_001073934.1	46	0	0.00	CA13	NP_940986.1	350	0	0.00
RPL23	NP_000969.1	1390	2	1.37	CA1	NP_001278896.1	590	1	1.61
LASP1	NP_006139.1	712	11	14.68	CA3	NP_005172.1	629	0	0.00
FBXO47	XP_011523168.1	169	2	11.24	CA2	NP_000058.1	1351	1	0.70
PLXDC1	NP_065138.2	249	8	30.52	PSKH2	XP_016869418.1	507	0	0.00
ARL5C	NP_001137440.1	1202	2	1.58	ATP6V0D2	NP_689778.1	722	5	6.58
CACNB1	NP_000714.3	554	8	13.72	SLC7A13	XP_011515169.1	273	5	17.40
RPL19	NP_000972.1	1305	4	2.91	WWP1	NP_008944.1	1288	6	4.43
STAC2	XP_016880070.1	190	3	15.00	RMDN1	XP_016869007.1	195	0	0.00
FBXL20	NP_001357138.1	661	10	14.37	CPNE3	XP_016869434.1	429	4	8.86
MED1	XP_006722020.1	775	5	6.13	CNGB3	NP_061971.3	389	4	9.77
CDK12	XP_016880239.1	1129	13	10.94	CNBD1	XP_016868638.1	107	5	44.39
NEUROD2	NP_006151.3	580	11	18.02	DCAF4L2	NP_689631.1	140	1	6.79
PPP1R1B	XP_016880705.1	974	12	11.70	MMP16	NP_005932.2	547	3	5.21
STARD3	XP_016879530.1	354	16	42.94	RIPK2	NP_003812.1	1341	4	2.83
TCAP	NP_003664.1	533	13	23.17	OSGIN2	NP_001119583.1	267	4	14.23
PNMT	NP_002677.1	353	7	18.84	NBN	NP_002476.2	639	4	5.95
PGAP3	NP_001278655.1	323	19	55.88	DECR1	NP_001350.1	3031	5	1.57
ERBB2	XP_024306410.1	3795	17	4.26	CALB1	NP_001353724.1	1966	4	1.93
MIEN1	NP_115715.3	363	18	47.10	TMEM64	NP_001139745.1	180	0	0.00
GRB7	NP_001229371.2	628	19	28.74	NECAB1	XP_011515515.1	268	2	7.09
IKZF3	NP_001244338.1	855	15	16.67	C8orf88	NP_001350204.1	24	0	0.00
ZPBP2	NP_955353.1	256	10	37.11	PIP4P2	NP_061180.1	197	0	0.00
GSDMB	NP_061000.2	391	16	38.87	OTUD6B	NP_057107.4	410	1	2.32
ORMDL3	NP_001307731.1	590	16	25.76	LRRC69	NP_001123362.1	484	0	0.00
LRRC3C	XP_016879492.1	417	10	22.78	SLC26A7	NP_439897.1	392	0	0.00
GSDMA	NP_835465.2	345	10	27.53	RUNX1T1	NP_001185562.1	755	3	3.77
PSMD3	NP_002800.2	1140	17	14.17	TRIQK	XP_016868836.1	41	0	0.00
CSF3	NP_000750.1	1331	6	4.28	CIBAR1	NP_660312.2	88	1	10.79

MED24	NP_001072986.1	446	7	14.91	RBM12B	XP_016868897.1	522	0	0.00
THRA	NP_003241.2	922	8	8.24	TMEM67	NP_714915.3	544	0	0.00
NR1D1	NP_068370.1	675	3	4.22	PDP1	XP_016869077.1	909	1	1.05
MSL1	NP_001352848.1	180	2	10.56	CDH17	XP_011515092.1	1887	1	0.50
CASC3	NP_031385.2	706	8	10.76	GEM	NP_859053.1	1274	1	0.75
RAPGEFL1	NP_001290462.1	290	1	3.28	RAD54B	NP_036547.1	1412	4	2.69
WIPF2	NP_573571.1	475	4	8.00	FSBP	NP_001243070.1	94	0	0.00
CDC6	XP_011523843.1	2587	7	2.57	VIRMA	NP_056311.2	246	2	7.72
RARA	NP_001138773.1	1439	12	7.92	ESRP1	XP_005251048.1	562	2	3.38
GJD3	NP_689343.3	235	1	4.04	DPY19L4	XP_005250951.2	203	2	9.36
TOP2A	NP_001058.2	2618	8	2.90	INTS8	XP_016869105.1	460	5	10.33
IGFBP4	NP_001543.2	680	7	9.78	CCNE2	XP_016869447.1	1813	3	1.57
TNS4	XP_005257801.1	691	7	9.62	TP53INP1	NP_150601.1	629	4	6.04
CCR7	NP_001829.1	1408	2	1.35	NDUFAF6	NP_001341445.1	549	1	1.73
SMARCE1	NP_003070.3	812	4	4.68	PLEKHF2	NP_078889.1	236	2	8.05
KRT222	NP_689562.1	615	4	6.18	C8orf37	NP_001350189.1	188	1	5.05
KRT24	XP_016879788.1	363	1	2.62	GDF6	NP_001001557.1	567	1	1.68
KRT25	XP_011522716.1	259	4	14.67	UQCRB	NP_006285.1	614	1	1.55
KRT26	NP_853517.2	212	2	8.96	MTERF3	NP_057026.3	531	5	8.94
KRT27	NP_853515.2	297	2	6.40	PTDSS1	NP_055569.1	438	7	15.18
KRT28	NP_853513.2	126	0	0.00	SDC2	NP_002989.2	776	2	2.45
KRT10	NP_001366295.1	517	1	1.84	CPQ	NP_057218.1	308	7	21.59
KRT12	NP_000214.1	449	0	0.00	TSPYL5	NP_277047.2	663	6	8.60
KRT20	NP_061883.1	457	0	0.00	MTDH	NP_001350066.1	629	4	6.04
KRT23	NP_056330.3	308	0	0.00	LAPTM4B	NP_060877.4	457	7	14.55
KRT39	NP_998821.3	216	0	0.00	MATN2	NP_002371.3	686	5	6.92
KRT40	XP_016879678.1	230	0	0.00	RPL30	NP_000980.1	1245	6	4.58
KRTAP3-3	NP_149441.1	187	0	0.00	ERICH5	NP_775820.2	68	0	0.00
KRTAP3-2	NP_114165.1	234	0	0.00	RIDA	NP_005827.1	557	0	0.00
KRTAP3-1	NP_114164.1	264	0	0.00	POP1	NP_055844.2	352	0	0.00
KRTAP1-5	NP_114163.1	190	0	0.00	NIPAL2	NP_001308564.1	222	0	0.00
KRTAP1-4	NP_001244234.1	193	0	0.00	KCNS2	NP_065748.1	787	5	6.04
KRTAP1-3	NP_112228.1	199	0	0.00	STK3	XP_011515553.1	576	0	0.00
KRTAP1-1	NP_112229.1	266	0	0.00	OSR2	NP_001273770.1	632	2	3.01
KRTAP2-1	NP_001116859.1	208	0	0.00	VPS13B	NP_689777.3	643	3	4.43
KRTAP2-2	NP_149021.2	192	0	0.00	COX6C	XP_016868509.1	588	2	3.23
KRTAP2-3	NP_001158724.1	252	0	0.00	RGS22	NP_001273621.1	292	1	3.25
KRTAP2-4	NP_149440.1	225	0	0.00	FBXO43	NP_001025031.2	271	1	3.51
KRTAP4-7	NP_149050.3	141	0	0.00	POLR2K	NP_005025.1	936	7	7.10
KRTAP4-8	NP_114166.1	202	0	0.00	SPAG1	XP_016869243.1	986	3	2.89
KRTAP4-9	NP_001139513.1	204	0	0.00	RNF19A	XP_016868793.1	675	7	9.85
KRTAP4-11	NP_149048.2	226	0	0.00	ANKRD46	NP_001257308.1	838	7	7.94
KRTAP4-12	NP_114060.1	208	0	0.00	SNX31	XP_024302853.1	138	0	0.00
KRTAP4-6	NP_112238.1	183	0	0.00	PABPC1	NP_002559.2	1831	7	3.63
KRTAP4-5	NP_149445.3	228	0	0.00	YWHAZ	XP_016869299.1	2954	11	3.54
KRTAP4-4	NP_115913.1	194	0	0.00	ZNF706	NP_001035975.1	291	7	22.85
KRTAP4-3	NP_149443.1	214	0	0.00	GRHL2	XP_011515609.1	490	7	13.57
KRTAP4-2	NP_149051.2	208	0	0.00	NCALD	NP_114430.2	1253	9	6.82
KRTAP4-1	NP_149049.2	191	5	24.87	RRM2B	NP_001165949.1	1080	4	3.52
KRTAP9-1	NP_001177389.1	197	0	0.00	UBR5	XP_011515408.2	1511	9	5.66
KRTAP9-2	NP_114167.2	152	1	6.25	ODF1	NP_077721.2	381	0	0.00
KRTAP9-3	NP_114168.1	206	1	4.61	KLF10	NP_005646.1	689	1	1.38
KRTAP9-8	NP_114169.2	200	1	4.75	AZIN1	NP_001349941.1	852	9	10.03
KRTAP9-4	NP_149461.2	200	1	4.75	ATP6V1C1	NP_001686.1	565	7	11.77
KRTAP9-9	NP_112237.2	213	0	0.00	BAALC	NP_001351803.1	711	4	5.34
KRTAP9-6	NP_001264260.1	173	0	0.00	FZD6	NP_001158087.1	611	4	6.22
KRTAP9-7	NP_001264261.1	177	0	0.00	CTHRC1	NP_612464.1	428	4	8.88
KRTAP29-1	NP_001244238.1	141	1	6.74	SLC25A32	NP_110407.2	384	3	7.42
KRTAP16-1	NP_001139654.1	165	0	0.00	DCAF13	NP_056235.5	998	5	4.76
KRTAP17-1	NP_114170.1	204	0	0.00	RIMS2	XP_016869504.1	788	7	8.44
KRT33A	NP_004129.2	240	0	0.00	DCSTAMP	NP_110415.1	235	3	12.13
KRT33B	NP_002270.1	145	0	0.00	DPYS	XP_006716581.1	607	4	6.26
KRT34	XP_011523095.1	269	0	0.00	LRP12	NP_001129175.1	284	7	23.41
KRT31	NP_002268.2	472	0	0.00	ZFPM2	NP_036214.2	686	5	6.92
KRT37	NP_003761.3	228	0	0.00	OXR1	NP_001185462.1	523	7	12.71
KRT38	NP_006762.3	187	0	0.00	ABRA	XP_024302831.1	392	6	14.54

KRT32	NP_002269.3	144	0	0.00	ANGPT1	NP_001186788.1	1021	3	2.79
KRT35	XP_011523096.1	199	0	0.00	RSPO2	NP_001269792.1	1012	7	6.57
KRT36	NP_003762.1	200	0	0.00	EIF3E	NP_001559.1	1225	12	9.31
KRT13	NP_705694.3	316	0	0.00	EMC2	NP_001316422.1	489	13	25.25
KRT15	NP_002266.3	584	1	1.63	TMEM74	NP_694560.1	169	15	84.32
KRT19	NP_002267.2	1017	2	1.87	TRHR	NP_003292.1	533	11	19.60
KRT9	NP_000217.2	391	2	4.86	NUDCD1	NP_116258.2	230	15	61.95
KRT14	NP_000517.3	908	3	3.14	ENY2	NP_064574.1	721	12	15.81
KRT16	NP_005548.2	601	1	1.58	PKHD1L1	XP_011515673.1	423	15	33.69
KRT17	NP_000413.1	690	1	1.38	EBAG9	XP_016869449.1	304	14	43.75
EIF1	NP_005792.1	728	7	9.13	SYBU	NP_060256.3	345	14	38.55
GAST	NP_000796.1	763	5	6.23	KCNV1	NP_055194.1	680	15	20.95
HAP1	NP_001354391.1	454	3	6.28	CSMD3	NP_001350114.1	678	14	19.62
JUP	XP_016880077.1	1603	10	5.93	TRPS1	NP_001269831.1	1011	12	11.28
P3H4	XP_006721703.1	228	6	25.00	EIF3H	NP_003747.1	985	17	16.40
FKBP10	XP_011523401.1	1554	4	2.45	UTP23	NP_115710.2	619	18	27.62
NT5C3B	XP_011522578.1	306	6	18.63	RAD21	NP_006256.1	1543	9	5.54
KLHL10	NP_001316524.1	438	3	6.51	AARD	NP_001020528.1	148	17	109.12
KLHL11	NP_060613.1	468	6	12.18	SLC30A8	NP_776250.2	632	7	10.52
ACLY	NP_001290204.1	1362	3	2.09	MED30	NP_542382.1	632	17	25.55
TTC25	NP_113609.1	768	3	3.71	EXT1	NP_000118.2	595	12	19.16
CNP	NP_149124.3	358	1	2.65	SAMD12	NP_001336740.1	187	16	81.28
DNAJC7	NP_003306.3	891	7	7.46	TNFRSF11B	NP_002537.3	857	3	3.33
NKIRAS2	NP_060065.2	257	3	11.09	COLEC10	NP_006429.2	314	9	27.23
ZNF385C	NP_001365199.1	57	0	0.00	MAL2	NP_443118.1	541	8	14.05
DHX58	XP_016880548.1	800	10	11.87	CCN3	NP_002505.1	242	1	3.93
KAT2A	NP_066564.2	1936	12	5.89	ENPP2	XP_024302950.1	818	8	9.29
HSPB9	NP_149971.1	284	7	23.41	TAF2	XP_016869268.1	753	12	15.14
RAB5C	NP_001238968.1	1250	6	4.56	DSCC1	XP_005251122.1	746	11	14.01
KCNH4	XP_016879890.1	397	7	16.75	DEPTOR	NP_073620.2	352	8	21.59
HCRT	NP_001515.1	962	9	8.89	COL14A1	XP_016869298.1	547	10	17.37
GHDC	NP_115873.1	353	11	29.60	MRPL13	NP_054797.2	1519	10	6.25
STAT5B	XP_024306665.1	1700	9	5.03	MTBP	NP_071328.2	230	6	24.78
STAT5A	XP_005257681.1	1926	7	3.45	SNTB1	NP_066301.1	518	9	16.50
STAT3	NP_001371914.1	3484	7	1.91	HAS2	NP_005319.1	1000	2	1.90
CAVIN1	NP_036364.2	724	8	10.50	ZHX2	NP_001349726.1	472	4	8.05
ATP6V0A1	NP_001365468.1	727	2	2.61	DERL1	NP_001128143.1	949	5	5.01
NAGLU	XP_024306539.1	412	2	4.61	TBC1D31	XP_011515681.1	732	7	9.08
HSD17B1	NP_001317148.1	460	3	6.20	FAM83A	NP_001275516.1	96	1	9.90
COASY	NP_001035997.2	1173	3	2.43	C8orf76	NP_116236.1	29	3	98.27
MLX	NP_733752.1	465	2	4.09	ZHX1-C8orf76	NP_001191109.1	23	0	0.00
PSMC3IP	NP_037422.2	543	1	1.75	ZHX1	NP_009153.3	264	2	7.20
RETREG3	NP_835227.1	151	0	0.00	ATAD2	XP_011515296.1	1428	9	5.99
TUBG1	XP_024306672.1	1513	3	1.88	NTAQ1	XP_016869090.1	166	1	5.72
TUBG2	XP_024306462.1	1039	3	2.74	FBXO32	NP_001229392.1	960	1	0.99
PLEKHH3	XP_016880607.1	781	0	0.00	KLHL38	NP_001075144.2	223	1	4.26
CCR10	NP_057686.2	595	0	0.00	ANXA13	NP_004297.2	360	1	2.64
CNTNAP1	XP_016880727.1	576	1	1.65	FAM91A1	NP_659400.3	271	5	17.53
EZH1	XP_011522819.1	1728	2	1.10	FER1L6	XP_011515533.1	257	1	3.70
RAMP2	NP_005845.2	327	3	8.72	TMEM65	NP_919267.2	214	0	0.00
VPS25	NP_115729.1	436	4	8.72	TRMT12	NP_060426.2	402	4	9.45
WNK4	XP_016880451.1	330	6	17.27	RNF139	NP_009149.2	647	7	10.28
COA3	NP_001035521.1	448	7	14.84	TATDN1	XP_011515633.1	403	8	18.86
CNTD1	XP_005257100.2	132	5	35.98	NDUFB9	NP_004996.1	662	10	14.35
BECN1	XP_016880752.1	1554	3	1.83	MTSS1	NP_001350230.1	463	10	20.52
PSME3	NP_789839.1	1051	8	7.23	ZNF572	NP_689625.2	179	10	53.07
AOC2	NP_033720.2	226	6	25.22	SQLE	NP_003120.2	982	11	10.64
AOC3	XP_011523721.1	411	5	11.56	WASHC5	XP_016869602.1	336	12	33.93
G6PC	NP_000142.2	858	1	1.11	NSMCE2	XP_016868820.1	675	13	18.30
PTGES3L-AARS	NP_001129514.2	612	0	0.00	TRIB1	NP_079471.1	1550	10	6.13
AARSD1	NP_001248363.1	219	0	0.00	LRATD2	XP_016868597.1	275	10	34.54
PTGES3L	NP_001136126.2	588	2	3.23	POU5F1B	NP_001153014.1	482	8	15.77
RUNDC1	XP_005257135.1	165	4	23.03	MYC	NP_002458.2	5787	10	1.64
RPL27	NP_001336851.1	1360	3	2.10	GSDMC	XP_011515461.1	324	7	20.52
IFI35	NP_005524.2	521	5	9.12	CYRIB	NP_001340245.1	281	5	16.90
VAT1	NP_006364.2	421	0	0.00	ASAP1	NP_001349855.1	811	7	8.20

RND2	XP_011523618.1	1737	4	2.19	ADCY8	XP_016868495.1	1826	5	2.60
BRCA1	NP_009229.2	2824	8	2.69	EFR3A	NP_001310487.1	385	4	9.87
NBR1	XP_016880131.1	1353	7	4.91	OC90	NP_001073868.2	150	1	6.33
TMEM106A	NP_659478.1	85	3	33.53	HHLA1	NP_001138567.1	136	1	6.98
ARL4D	NP_001652.2	1366	2	1.39	KCNQ3	NP_004510.1	1100	3	2.59
DHX8	NP_001309148.1	1461	2	1.30	LRRC6	XP_011515252.1	782	3	3.64
ETV4	NP_001356296.1	871	2	2.18	TMEM71	NP_001369325.1	264	3	10.79
MEOX1	NP_004518.1	377	3	7.56	PHF20L1	XP_011515377.1	767	3	3.72
SOST	NP_079513.1	477	2	3.98	TG	NP_003226.4	823	3	3.46
DUSP3	NP_004081.1	804	3	3.54	SLA	NP_001269893.1	538	3	5.30
CFAP97D1	NP_001340329.1	52	1	18.27	CCN4	NP_003873.1	500	5	9.50
MPP3	NP_001317162.1	836	4	4.55	NDRG1	NP_001361775.1	794	5	5.98
CD300LG	XP_006721784.1	145	0	0.00	ST3GAL1	XP_016869225.1	416	2	4.57
MPP2	NP_001265299.1	1257	1	0.76	ZFAT	NP_001167628.1	406	4	9.36
PPY	XP_011523280.1	670	1	1.42	KHDRBS3	XP_011515100.1	727	3	3.92
PYY	NP_004151.4	596	1	1.59	FAM135B	XP_016868960.1	426	4	8.92
NAGS	NP_694551.1	663	0	0.00	COL22A1	XP_016868639.1	534	7	12.45
TMEM101	NP_115752.1	178	3	16.01	KCNK9	XP_011515403.1	985	6	5.79
LSM12	NP_001356415.1	572	3	4.98	TRAPPC9	XP_011515628.1	950	5	5.00
G6PC3	NP_612396.1	235	0	0.00	CHRAC1	NP_059140.1	744	6	7.66
HDAC5	NP_001369322.1	2413	3	1.18	AGO2	XP_011515270.2	1843	4	2.06
HROB	NP_076937.2	113	2	16.81	PTK2	NP_005598.3	1536	1	0.62
ASB16	NP_543139.4	1097	1	0.87	DENND3	NP_001339819.2	276	1	3.44
TMUB2	XP_011523502.1	302	4	12.58	SLC45A4	NP_001073900.1	223	1	4.26
ATXN7L3	NP_001369242.1	366	2	5.19	GPR20	NP_005284.2	284	6	20.07
UBTF	XP_016880492.1	1160	2	1.64	PTP4A3	XP_024302825.1	434	4	8.76
SLC4A1	XP_011523431.1	687	6	8.30	TSNARE1	NP_001353830.1	695	1	1.37
RUNDC3A	XP_016879524.1	592	5	8.02	ADGRB1	XP_016869184.1	384	9	22.26
SLC25A39	NP_001308169.1	269	4	14.13	ARC	NP_056008.1	401	3	7.11
GRN	XP_024306498.1	861	7	7.72	JRK	XP_011515657.1	386	4	9.84
FAM171A2	NP_940877.2	187	4	20.32	PSCA	NP_005663.2	414	12	27.53
ITGA2B	XP_011523051.1	1035	5	4.59	LY6K	XP_005251046.1	304	10	31.25
GPATCH8	NP_001291868.1	632	5	7.52	THEM6	NP_057731.1	97	2	19.59
FZD2	NP_001457.1	734	1	1.29	SLURP1	NP_065160.1	258	11	40.50
MEIOC	XP_005257293.1	184	1	5.16	LYPD2	NP_991108.1	188	10	50.53
CCDC43	NP_653210.2	221	3	12.90	SLURP2	NP_001343301.1	537	11	19.46
DBF4B	NP_079380.2	369	0	0.00	LYNX1-SLURP	NP_076435.1	536	10	17.72
ADAM11	NP_002381.2	825	3	3.45	LYNX1	NP_001343299.1	536	10	17.72
GJC1	NP_001073852.1	335	3	8.51	LY6D	NP_003686.1	243	8	31.27
HIGD1B	XP_016880231.1	85	0	0.00	GML	NP_002057.1	210	13	58.81
EFTUD2	NP_001245283.1	2125	5	2.24	CYP11B1	NP_001021384.1	410	2	4.63
CCDC103	NP_001245324.1	136	1	6.98	CYP11B2	NP_000489.3	505	2	3.76
FAM187A	NP_001245329.1	136	1	6.98	LY6E	NP_001120685.1	432	7	15.39
GFAP	NP_002046.1	2416	3	1.18	LY6L	NP_001355089.1	28	4	135.71
KIF18B	XP_011522692.1	1124	10	8.45	LY6H	NP_001123950.1	979	9	8.73
C1QL1	NP_006679.1	304	13	40.62	GPIHBP1	NP_001288701.1	568	10	16.72
DCAKD	NP_001308255.1	440	12	25.91	ZFP41	NP_001258085.3	168	0	0.00
NMT1	NP_066565.1	722	7	9.21	GLI4	NP_612474.1	411	3	6.93
PLCD3	NP_588614.1	624	12	18.27	ZNF696	NP_112157.2	153	2	12.42
ACBD4	XP_016880573.1	187	9	45.72	TOP1MT	NP_443195.1	748	6	7.62
HEXIM1	NP_006451.1	499	4	7.61	RHPN1	XP_011515119.1	659	5	7.21
HEXIM2	XP_006721750.1	199	6	28.64	MAFA	NP_963883.2	321	0	0.00
FMNL1	XP_006722129.1	1378	9	6.20	ZC3H3	XP_006716599.2	696	7	9.55
SPATA32	NP_689556.2	137	12	83.21	GSDMD	XP_011515603.1	437	4	8.70
MAP3K14	NP_003945.2	1565	9	5.46	MROH6	XP_011515517.1	135	3	21.11
ARHGAP27	XP_011522773.1	921	16	16.50	NAPRT	XP_016869466.1	550	7	12.09
PLEKHM1	XP_011523827.1	314	15	45.38	EEF1D	XP_016868660.1	1009	8	7.53
CRHR1	NP_001138618.1	1532	10	6.20	TIGD5	NP_116251.4	104	6	54.80
SPPL2C	NP_787078.2	494	6	11.54	PYCR3	NP_001316795.2	485	6	11.75
MAPT	NP_001364196.1	2286	12	4.99	GFUS	NP_001304712.1	668	4	5.69
STH	NP_001007533.1	160	8	47.50	ZNF623	NP_055604.3	129	0	0.00
KANSL1	XP_011522931.1	433	11	24.13	ZNF707	NP_776192.2	218	1	4.36
LRRC37A	NP_055649.4	469	9	18.23	CCDC166	NP_001156386.1	14	0	0.00
ARL17B	NP_001339698.1	1591	10	5.97	MAPK15	NP_620590.2	1858	3	1.53
LRRC37A2	XP_024306541.1	402	5	11.82	FAM83H	NP_940890.4	253	6	22.53
ARL17A	NP_057716.2	1541	8	4.93	SCRIB	NP_056171.3	1432	7	4.64

NSF	NP_006169.2	2008	9	4.26	PUF60	NP_001258026.1	937	11	11.15
WNT3	NP_110380.1	1042	10	9.12	NRBP2	XP_016868871.1	145	3	19.65
WNT9B	XP_011523480.1	877	3	3.25	EPPK1	XP_016869381.1	650	4	5.85
GOSR2	XP_016880872.1	730	6	7.81	PLEC	NP_000436.2	1847	8	4.11
RPRML	NP_981945.1	345	1	2.75	PARP10	XP_011515638.1	396	3	7.20
CDC27	NP_001280018.1	1907	6	2.99	GRINA	NP_000828.1	601	7	11.06
MYL4	XP_011523141.2	1383	2	1.37	SPATC1	XP_011515324.1	96	0	0.00
ITGB3	NP_000203.2	1429	2	1.33	OPLAH	XP_011515262.1	311	1	3.05
EFCAB13	NP_689560.3	1009	2	1.88	EXOSC4	NP_061910.1	1329	9	6.43
NPEPPS	NP_001317186.1	800	5	5.94	GPAA1	NP_003792.1	261	13	47.32
KPNB1	NP_002256.2	1725	0	0.00	CYC1	NP_001907.3	989	7	6.72
TBKBP1	XP_005257916.1	402	5	11.82	SHARPIN	XP_016869376.1	508	12	22.44
TBX21	NP_037483.1	1336	5	3.56	MAF1	NP_115648.2	591	5	8.04
OSBPL7	XP_016879613.1	380	6	15.00	WDR97	NP_001303238.1	209	12	54.54
MRPL10	NP_660298.2	265	0	0.00	HGH1	NP_057542.2	528	10	17.99
LRRC46	NP_219481.1	560	0	0.00	MROH1	XP_011515572.1	167	15	85.32
SCRN2	XP_016880775.1	313	0	0.00	BOP1	NP_056016.1	1142	17	14.14
SP6	NP_001245177.1	634	3	4.50	SCX	NP_001073983.1	542	10	17.53
SP2	XP_011523441.1	429	2	4.43	HSF1	XP_005272372.1	1029	6	5.54
PNPO	NP_060599.1	524	6	10.88	DGAT1	NP_036211.2	1044	17	15.47
PRR15L	NP_077296.1	197	5	24.11	SCRT1	NP_112599.2	1096	13	11.27
CDK5RAP3	NP_001265126.1	263	5	18.06	TMEM249	NP_001239333.1	38	13	324.98
COPZ2	XP_011523176.1	570	2	3.33	FBXL6	NP_078831.4	656	17	24.62
NFE2L1	NP_003195.1	706	13	17.49	SLC52A2	NP_078807.1	266	13	46.43
CBX1	NP_001120700.1	1195	6	4.77	ADCK5	NP_777582.4	332	16	45.78
SNX11	NP_037455.2	293	5	16.21	CPSF1	XP_006716611.1	1116	12	10.21
SKAP1	XP_005257812.1	389	7	17.09	SLC39A4	XP_024302957.1	306	8	24.84
HOXB1	NP_002135.2	509	9	16.80	VPS28	XP_005272381.1	528	6	10.79
HOXB2	NP_002136.1	521	11	20.06	TONSL	NP_038460.4	1279	11	8.17
HOXB3	XP_016880049.1	514	9	16.63	CYHR1	NP_612505.1	351	14	37.89
HOXB4	NP_076920.1	743	10	12.79	KIFC2	NP_665697.1	1330	9	6.43
HOXB5	NP_002138.1	502	12	22.71	FOXH1	NP_003914.1	705	6	8.08
HOXB6	XP_011523029.1	582	16	26.12	PPP1R16A	XP_024303079.1	868	14	15.32
HOXB7	NP_004493.3	625	11	16.72	GPT	NP_001369593.1	1643	4	2.31
HOXB8	XP_005257343.1	445	10	21.35	MFSD3	XP_016868494.1	470	5	10.11
HOXB9	NP_076922.1	528	13	23.39	RECQL4	XP_016869483.1	1057	9	8.09
PRAC1	NP_115767.1	99	4	38.38	LRRC14	XP_024303104.1	210	4	18.09
PRAC2	NP_001269205.1	40	5	118.74	LRRC24	NP_001019849.2	499	1	1.90
HOXB13	NP_006352.2	427	11	24.47	C8orf82	NP_001001795.1	31	0	0.00
TTLL6	NP_001353243.1	246	3	11.58	ARHGAP39	NP_001295136.1	329	6	18.24
CALCOCO2	NP_001248320.1	526	6	10.84	ZNF251	XP_024303092.1	170	3	18.63
ATP5MC1	NP_005166.1	845	8	8.99	ZNF34	NP_001273699.1	199	3	16.85
UBE2Z	NP_075567.2	1425	9	6.00	RPL8	NP_150644.1	2479	8	3.83
SNF8	NP_001304122.1	455	8	16.70	ZNF517	XP_011515319.1	177	2	14.31
GIP	NP_004114.1	481	4	7.90	ZNF7	XP_016869305.1	237	3	17.18
IGF2BP1	NP_006537.3	852	2	2.23	COMMD5	NP_001074473.1	318	7	32.17
B4GALNT2	NP_001152859.1	410	7	16.22	ZNF250	NP_001350034.1	171	2	18.52
GNGT2	NP_113686.1	854	9	10.01	ZNF16	XP_024303035.1	225	3	23.03
ABI3	XP_005257486.1	853	6	6.68	C8orf33	NP_075568.1	151	1	12.58
PHOSPHO1	NP_848595.1	248	9	34.47	WASHC1	XP_011515968.1	147	1	12.92
ZNF652	XP_024306424.1	387	1	2.45	FOXD4	NP_997188.2	430	8	32.13
PHB	NP_001268426.1	1390	3	2.05	CBWD1	XP_005251570.1	205	5	38.62
NGFR	NP_002498.1	1248	5	3.81	DOCK8	NP_982272.2	777	8	15.05
NXPH3	NP_009156.2	593	4	6.41	KANK1	NP_001341263.1	1134	10	11.97
SPOP	XP_024306763.1	948	3	3.01	DMRT1	XP_006716795.1	581	7	15.26
SLC35B1	XP_011522481.1	525	1	1.81	DMRT3	NP_067063.1	438	8	21.69
FAM117A	NP_110429.1	87	7	76.43	DMRT2	XP_016869702.1	440	7	17.78
KAT7	NP_008998.1	888	1	1.07	SMARCA2	NP_003061.3	2305	8	3.66
TAC4	NP_001070973.1	142	0	0.00	VLDLR	NP_003374.3	889	10	11.25
DLX4	NP_612138.1	381	2	4.99	KCNV2	NP_598004.1	762	3	3.74
DLX3	NP_005211.1	637	4	5.97	PUM3	NP_055693.4	1104	3	2.58
ITGA3	NP_002195.1	785	5	6.05	RFX3	XP_006716903.1	787	7	8.45
PDK2	NP_002602.2	844	1	1.13	GLIS3	NP_001035878.1	721	5	6.59
SAMD14	XP_016879812.1	359	2	5.29	SLC1A1	XP_011516309.1	717	5	6.62
PPP1R9B	NP_115984.3	683	1	1.39	SPATA6L	NP_001034484.3	43	1	22.09
SGCA	XP_011523422.2	430	0	0.00	PLPP6	NP_982278.3	160	2	11.87

COL1A1	XP_005257116.2	1696	4	2.24	CDC37L1	NP_060383.2	550	3	5.18
TMEM92	NP_694961.2	136	0	0.00	AK3	NP_001186781.1	791	1	1.20
XYLT2	NP_071450.2	251	0	0.00	RCL1	NP_005763.3	826	4	4.60
MRPL27	NP_057588.1	841	0	0.00	INSL6	XP_011516004.1	166	5	28.61
EME1	NP_001159603.1	586	1	1.62	JAK2	NP_001309124.1	2656	5	1.79
LRRC59	NP_060979.2	684	2	2.78	INSL4	NP_002186.1	135	5	35.18
ACSF2	NP_001275897.1	524	1	1.81	RLN2	XP_024303414.1	202	3	14.11
CHAD	XP_011522516.1	728	2	2.61	RLN1	NP_008842.1	113	3	25.22
RSAD1	NP_060816.1	297	2	6.40	PLGRKT	NP_060935.2	317	13	38.96
MYCBPAP	XP_005257783.1	208	1	4.57	CD274	NP_054862.1	1132	6	5.04
EPN3	NP_060427.2	599	2	3.17	PDCD1LG2	XP_005251657.1	610	8	12.46
SPATA20	NP_073738.2	230	2	8.26	RIC1	NP_065880.2	553	11	18.90
CACNA1G	NP_001243254.1	847	2	2.24	ERMP1	XP_016870628.1	140	9	61.07
ABCC3	NP_003777.2	719	1	1.32	MLANA	NP_005502.1	488	3	5.84
ANKRD40	NP_443087.1	494	4	7.69	KIAA2026	XP_016869824.1	86	8	88.37
LUC7L3	XP_005257506.1	843	3	3.38	RANBP6	NP_001230131.1	916	10	10.37
WFIKKN2	NP_783165.1	243	3	11.73	IL33	NP_254274.1	763	6	7.47
TOB1	NP_001230806.1	356	4	10.67	TPD52L3	NP_001001874.2	85	4	44.70
SPAG9	NP_003962.3	655	6	8.70	UHRF2	NP_690856.1	631	11	16.56
NME1	NP_937818.1	1470	3	1.94	GLDC	NP_000161.2	658	6	8.66
NME1-NME2	NP_001018146.1	1354	3	2.10	KDM4C	NP_001140168.1	761	8	9.99
NME2	NP_002503.1	1360	3	2.10	DMAC1	NP_001304988.1	87	8	87.35
MBTD1	XP_005257525.1	404	5	11.76	PTPRD	XP_006716897.1	1636	6	3.48
UTP18	NP_057085.2	1083	2	1.75	TYRP1	NP_000541.1	714	9	11.97
CA10	NP_001076002.1	1287	3	2.21	LURAP1L	NP_981948.1	143	9	59.79
KIF2B	NP_115948.4	950	7	7.00	MPDZ	NP_001362356.1	1188	7	5.60
TOM1L1	NP_005477.2	496	8	15.32	NFIB	NP_001356391.1	689	11	15.17
COX11	XP_016879685.1	534	8	14.23	ZDHHC21	XP_016870180.1	201	11	51.99
STXBP4	XP_016879899.1	378	8	20.10	CER1	NP_005445.1	258	8	29.46
HLF	XP_005257326.1	424	5	11.20	FREM1	NP_659403.4	396	14	33.58
MMD	NP_036461.2	691	8	11.00	TTC39B	NP_001161813.1	194	13	63.66
TMEM100	XP_016880304.1	226	7	29.42	SNAPC3	NP_001356577.1	321	11	32.55
PCTP	NP_001317307.1	262	12	43.51	PSIP1	NP_001121689.1	1529	6	3.73
ANKFN1	XP_011522730.1	218	14	61.01	CCDC171	XP_016869926.1	115	13	107.39
NOG	NP_005441.1	1115	2	1.70	C9orf92	NP_001351908.1	23	1	41.30
C17orf67	NP_001078899.2	109	9	78.44	BNC2	NP_060107.3	469	9	18.23
DGKE	XP_011523696.1	475	5	10.00	CNTLN	XP_016870329.1	473	6	12.05
TRIM25	NP_005073.2	878	4	4.33	SH3GL2	NP_003017.1	1431	7	4.65
COIL	NP_004636.1	766	7	8.68	ADAMTSL1	XP_011516366.1	514	8	14.79
SCPEP1	NP_067639.1	522	8	14.56	SAXO1	NP_714918.2	32	0	0.00
AKAP1	XP_016880679.1	531	5	8.94	RRAGA	NP_006561.1	477	1	1.99
MSI2	XP_005257072.1	747	3	3.82	HAUS6	NP_001257819.1	596	6	9.56
CCDC182	NP_001269473.1	33	1	28.79	PLIN2	XP_016869748.1	770	0	0.00
MRPS23	NP_057154.2	434	0	0.00	DENND4C	NP_001317569.1	227	5	20.92
CUEDC1	XP_016880129.1	79	6	72.15	RPS6	NP_001001.2	2083	2	0.91
VEZF1	NP_009077.2	630	3	4.52	ACER2	NP_001010887.2	312	0	0.00
SRSF1	NP_008855.1	2038	6	2.80	SLC24A2	NP_001362780.1	815	6	6.99
DYNLL2	NP_542408.1	896	1	1.06	MLLT3	XP_016870215.1	1150	3	2.48
OR4D1	NP_001373024.1	60	0	0.00	FOCAD	XP_016870341.1	181	9	47.24
OR4D2	NP_001004707.1	106	0	0.00	HACD4	NP_001010915.2	341	10	27.86
EPX	NP_000493.1	389	1	2.44	IFNB1	NP_002167.1	1237	12	9.22
MKS1	NP_001308198.1	356	0	0.00	IFNW1	NP_002168.1	210	6	27.14
LPO	NP_006142.1	294	0	0.00	IFNA21	NP_002166.2	793	4	4.79
MPO	NP_000241.1	1618	1	0.59	IFNA4	NP_066546.1	202	4	18.81
TSPOAP1	NP_077729.1	322	0	0.00	IFNA7	NP_066401.2	146	5	32.53
SUPT4H1	NP_003159.1	689	2	2.76	IFNA10	NP_002162.1	131	2	14.50
RNF43	NP_060233.3	559	5	8.50	IFNA16	NP_002164.1	111	2	17.12
HSF5	NP_001073908.2	525	0	0.00	IFNA17	NP_067091.1	830	6	6.87
MTMR4	NP_004678.3	322	4	11.80	IFNA14	NP_002163.2	154	3	18.51
SEPTIN4	NP_001355700.1	1194	5	3.98	IFNA5	NP_002160.1	151	2	12.58
TEX14	NP_112562.3	233	7	28.54	KLHL9	NP_061335.1	583	18	29.33
RAD51C	XP_006722064.1	1566	6	3.64	IFNA6	NP_066282.1	186	3	15.32
PPM1E	NP_055721.3	1061	10	8.95	IFNA13	NP_008831.3	798	5	5.95
TRIM37	XP_016880152.1	829	12	13.75	IFNA2	NP_000596.2	895	5	5.31
SKA2	NP_872426.1	452	10	21.02	IFNA8	NP_002161.2	167	3	17.06
PRR11	XP_024306596.1	405	5	11.73	IFNA1	NP_076918.1	799	5	5.94

SMG8	NP_060619.4	209	2	9.09	IFNE	NP_795372.1	216	9	39.58
GDPD1	NP_001159465.1	386	3	7.38	MTAP	NP_002442.2	726	14	18.32
YPEL2	XP_016880110.1	237	12	48.10	CDKN2A	NP_000068.1	3925	11	2.66
DHX40	NP_078888.4	945	13	13.07	CDKN2B	NP_004927.2	1931	11	5.41
CLTC	NP_001275582.1	1896	9	4.51	DMRTA1	NP_071443.2	329	6	17.32
PTRH2	NP_001015509.1	532	13	23.21	ELAVL2	NP_001372623.1	1278	5	3.72
VMP1	NP_001316324.1	474	10	20.04	IZUMO3	XP_005251390.1	43	2	44.18
TUBD1	NP_001180540.1	1021	13	12.10	TUSC1	NP_001004125.2	187	12	60.96
RPS6KB1	NP_001258972.1	2190	11	4.77	CAAP1	NP_079104.3	150	2	12.67
RNFT1	NP_057209.3	393	0	0.00	PLAA	NP_001308475.1	730	9	11.71
HEATR6	NP_071353.4	607	9	14.08	IFT74	NP_001092693.1	306	9	27.94
CA4	XP_005257696.1	769	0	0.00	LRRC19	NP_075052.1	443	10	21.44
USP32	XP_011523674.1	1646	13	7.50	TEK	NP_000450.3	1173	1	0.81
C17orf64	XP_005257091.1	921	4	4.13	EQTN	NP_001155057.1	557	9	15.35
APPBP2	NP_006371.2	842	12	13.54	MOB3B	NP_079037.3	262	11	39.88
PPM1D	NP_003611.1	1317	12	8.66	IFNK	NP_064509.2	271	10	35.05
BCAS3	XP_011523241.1	576	10	16.49	C9orf72	NP_001242983.1	483	6	11.80
TBX2	NP_005985.3	954	10	9.96	LINGO2	XP_016869792.1	768	11	13.61
TBX4	XP_011523792.1	563	7	11.81	ACO1	NP_001349769.1	993	6	5.74
NACA2	NP_954984.1	613	5	7.75	DDX58	NP_001372842.1	1392	3	2.05
BRIP1	XP_011523639.1	713	3	4.00	TOPORS	NP_001182551.1	533	4	7.13
INTS2	NP_065799.2	348	5	13.65	NDUFB6	NP_001186916.1	699	9	12.23
MED13	XP_011523853.1	676	8	11.24	TAF1L	NP_722516.1	1238	1	0.77
EFCAB3	NP_001138405.1	952	5	4.99	TMEM215	NP_997723.2	115	1	8.26
METTL2A	NP_859076.3	394	4	9.64	APTX	XP_016870320.1	765	4	4.97
TLK2	XP_024306320.1	793	8	9.58	DNAJA1	NP_001530.1	1516	5	3.13
MRC2	NP_006030.2	422	3	6.75	SMU1	XP_005251560.1	804	4	4.73
MARCHF10	XP_005257159.2	317	4	11.99	B4GALT1	NP_001365426.1	527	0	0.00
TANC2	XP_011522899.1	1126	4	3.37	SPINK4	NP_055286.1	240	0	0.00
CYB561	NP_001317350.1	235	0	0.00	BAG1	NP_004314.6	641	2	2.96
ACE	NP_000780.1	1655	0	0.00	CHMP5	NP_057494.3	605	4	6.28
KCNH6	XP_016880664.1	405	1	2.35	NFX1	NP_001305687.1	441	2	4.31
DCAF7	NP_005819.3	486	4	7.82	AQP7	NP_001363120.1	627	1	1.52
TACO1	NP_057444.2	516	0	0.00	AQP3	NP_001305073.1	781	2	2.43
MAP3K3	NP_976226.1	1554	4	2.45	NOL6	NP_631981.2	849	2	2.24
LIMD2	NP_085053.1	725	2	2.62	ANKRD18B	NP_001340361.1	854	2	2.22
STRADA	NP_001003786.1	278	2	6.83	PRSS3	XP_011516267.1	611	1	1.55
CCDC47	NP_064583.2	147	0	0.00	UBE2R2	NP_060281.2	1605	5	2.96
DDX42	XP_016879600.1	1198	2	1.59	UBAP2	NP_001356996.2	291	2	6.53
FTSJ3	NP_060117.3	1088	5	4.37	DCAF12	NP_056212.1	188	3	15.16
PSMC5	XP_024306608.1	1726	2	1.10	UBAP1	NP_001164673.1	265	6	21.51
SMARCD2	NP_001091896.1	804	3	3.54	KIF24	XP_011516163.1	1048	7	6.35
CSH2	NP_072171.1	299	0	0.00	NUDT2	XP_024303296.1	638	8	11.91
GH2	NP_072052.1	297	2	6.40	MYORG	XP_016870419.1	245	12	46.53
CSH1	NP_001308.1	332	2	5.72	C9orf24	XP_005251673.1	94	13	131.38
CSHL1	NP_072102.1	271	1	3.51	FAM219A	NP_001171874.1	75	11	139.33
GH1	NP_072054.1	348	2	5.46	DNAI1	NP_001268357.1	412	8	18.45
CD79B	NP_067613.1	778	7	8.55	ENHO	NP_940975.2	485	9	17.63
SCN4A	NP_000325.4	741	6	7.69	CNTFR	XP_016869749.1	748	12	15.24
PRR29	NP_001177958.1	17	1	55.88	RPP25L	NP_680544.1	259	14	51.35
ICAM2	NP_001093256.1	545	4	6.97	DCTN3	NP_001268354.1	588	14	22.62
ERN1	NP_001424.3	1561	2	1.22	ARID3C	XP_016869772.1	217	12	52.53
TEX2	XP_016880335.1	342	2	5.56	SIGMAR1	NP_001269134.1	575	9	14.87
PECAM1	XP_016880230.1	1827	4	2.08	GALT	NP_000146.2	325	3	8.77
MILR1	XP_024306474.1	83	0	0.00	IL11RA	NP_001136256.1	335	12	34.03
POLG2	NP_009146.2	710	2	2.68	CCL27	NP_006655.1	629	8	12.08
DDX5	NP_001307525.1	2312	6	2.47	LOC730098	XP_016870595.1	0	0	#DIV/0!
CEP95	NP_612372.1	129	2	14.73	CCL19	NP_006265.1	914	4	4.16
SMURF2	XP_005257642.1	1324	6	4.30	CCL21	NP_002980.1	1025	4	3.71
LRRC37A3	NP_001290184.1	479	2	3.97	FAM205A	NP_001135389.1	87	7	76.43
GNA13	NP_006563.2	1209	9	7.07	PHF24	XP_016870042.1	35	0	0.00
RGS9	NP_003826.2	453	3	6.29	DNAJB5	NP_001336652.1	658	1	1.44
AXIN2	XP_016880682.1	1285	9	6.65	C9orf131	NP_001035502.1	21	0	0.00
CEP112	NP_001340057.1	149	7	44.63	VCP	NP_009057.1	2386	5	1.99
APOH	NP_000033.2	792	1	1.20	FANCG	NP_004620.1	601	1	1.58
PRKCA	NP_002728.2	1411	10	6.73	PIGO	XP_016870712.1	307	0	0.00

CACNG5	NP_001358405.1	328	8	23.17	STOML2	NP_001273962.1	802	3	3.55
CACNG4	NP_055220.1	405	5	11.73	FAM214B	XP_024303457.1	115	0	0.00
CACNG1	NP_000718.1	389	10	24.42	UNC13B	NP_001358118.1	730	1	1.30
HELZ	NP_055692.3	685	11	15.25	RUSC2	NP_055621.2	246	2	7.72
PSMD12	NP_777360.1	1262	8	6.02	FAM166B	NP_001274168.1	166	0	0.00
PITPNC1	NP_036549.2	396	6	14.39	TESK1	NP_006276.2	1137	3	2.51
NOL11	NP_056277.2	673	3	4.23	CD72	XP_006716956.1	561	2	3.39
BPTF	XP_005257207.1	3261	6	1.75	SIT1	NP_055265.1	173	0	0.00
C17orf58	NP_001369288.1	63	1	15.08	CCDC107	NP_777583.2	46	0	0.00
KPNA2	NP_001307540.1	1597	6	3.57	ARHGEF39	XP_016870715.1	246	0	0.00
AMZ2	NP_001333414.1	153	8	49.67	CA9	NP_001207.2	747	0	0.00
SLC16A6	XP_005257846.2	327	2	5.81	TPM2	NP_001288155.1	1467	1	0.65
ARSG	NP_001339839.1	375	7	17.73	TLN1	NP_006280.3	920	4	4.13
WIPI1	NP_060453.3	560	1	1.70	CREB3	NP_006359.3	520	0	0.00
PRKAR1A	NP_001263219.1	1400	6	4.07	GBA2	XP_016870429.1	795	1	1.19
FAM20A	NP_060035.2	289	0	0.00	RGP1	NP_001073965.2	173	0	0.00
ABCA8	NP_009099.1	408	4	9.31	MSMP	NP_001037729.1	82	1	11.58
ABCA9	XP_024306297.1	362	3	7.87	NPR2	XP_024303324.1	798	2	2.38
ABCA6	XP_016879893.1	397	6	14.36	SPAG8	NP_758516.1	199	1	4.77
ABCA10	NP_525021.3	322	3	8.85	HINT2	XP_024303470.1	425	1	2.24
ABCA5	NP_061142.2	436	5	10.89	FAM221B	XP_024303310.1	10	0	0.00
MAP2K6	NP_002749.2	718	9	11.91	TMEM8B	NP_001036055.2	166	2	11.45
KCNJ16	XP_016880098.1	425	8	17.88	OR13J1	NP_001004487.1	53	0	0.00
KCNJ2	NP_000882.1	549	5	8.65	HRCT1	NP_001034881.1	39	0	0.00
SOX9	NP_000337.1	1995	4	1.90	OR2S2	NP_063950.2	40	2	47.50
SLC39A11	XP_016879824.1	286	2	6.64	RECK	NP_066934.1	474	1	2.00
SSTR2	NP_001041.1	790	1	1.20	GLIPR2	NP_071738.1	248	1	3.83
COG1	NP_061184.1	480	0	0.00	CCIN	NP_005884.2	269	1	3.53
FAM104A	NP_116226.2	25	0	0.00	CLTA	NP_009027.1	790	3	3.61
C17orf80	NP_001338193.2	198	1	4.80	GNE	XP_005251391.1	572	5	8.30
CPSF4L	XP_011523417.1	348	0	0.00	RNF38	XP_016869783.1	433	3	6.58
CDC42EP4	NP_036253.2	256	1	3.71	MELK	NP_055606.1	1725	2	1.10
SDK2	XP_011523217.1	677	1	1.40	PAX5	NP_001267479.1	1296	2	1.47
RPL38	NP_000990.1	1057	2	1.80	ZCCHC7	NP_115602.2	622	4	6.11
TTYH2	NP_116035.5	291	4	13.06	GRHPR	XP_024303484.1	1030	1	0.92
DNAI2	NP_001340096.1	331	2	5.74	ZBTB5	XP_005251691.1	230	3	12.39
KIF19	XP_016879639.1	947	1	1.00	POLR1E	NP_071935.1	790	3	3.61
BTBD17	XP_016880111.1	183	4	20.76	FBXO10	XP_016870108.1	481	5	9.87
GPR142	NP_861455.1	185	5	25.67	TOMM5	NP_001001790.1	434	4	8.76
GPRC5C	XP_005257571.1	307	1	3.09	FRMPD1	NP_001358152.1	179	4	21.23
CD300A	NP_009192.2	702	6	8.12	TRMT10B	XP_011516037.1	303	2	6.27
CD300LB	XP_005257084.1	550	7	12.09	EXOSC3	NP_057126.2	997	4	3.81
CD300C	XP_016879522.1	685	5	6.93	DCAF10	NP_001273739.1	1930	6	2.95
CD300LD	NP_001108624.1	54	2	35.18	SLC25A51	NP_219480.1	153	4	24.84
C17orf77	NP_689673.2	39	0	0.00	SHB	NP_003019.2	290	1	3.28
CD300E	NP_852114.2	195	3	14.61	ALDH1B1	NP_000683.3	757	2	2.51
RAB37	NP_783865.1	438	5	10.84	IGFBPL1	NP_001007564.1	266	1	3.57
CD300LF	NP_620587.2	564	4	6.74	ANKRD18A	XP_024303251.1	885	1	1.07
SLC9A3R1	NP_004243.1	1269	5	3.74	CNTNAP3	NP_387504.2	317	4	11.99
NAT9	NP_001292011.1	227	5	20.92	SPATA31A1	XP_011516308.1	40	2	47.50
TMEM104	NP_001308193.1	34	4	111.76	LOC10192995	XP_016870865.1	988	2	1.92
GRIN2C	XP_006721909.1	965	2	1.97	FOXD4L6	NP_001078945.1	252	0	0.00
FDXR	NP_001244943.2	733	3	3.89	CBWD6	XP_016870514.1	101	1	9.41
FADS6	XP_016879947.1	53	6	107.54	CNTNAP3B	NP_001188309.2	257	3	11.09
USH1G	NP_775748.2	1100	5	4.32	SPATA31A6	NP_001138668.1	5	0	0.00
OTOP2	NP_835454.1	126	4	30.16	SPATA31A5	XP_011543973.1	19	0	0.00
OTOP3	NP_839947.1	181	5	26.24	SPATA31A7	NP_056482.2	45	2	42.22
HID1	XP_005257283.1	394	4	9.64	FOXD4L5	NP_001119806.1	261	0	0.00
CDR2L	NP_055418.2	273	12	41.76	CBWD5	NP_001317597.1	111	0	0.00
MRPL58	NP_001290194.1	1100	7	6.05	FOXD4L4	NP_954714.2	290	2	6.55
ATP5PD	NP_001003785.1	1062	8	7.16	ZNF658	XP_016870103.1	196	1	4.85
KCTD2	NP_056168.1	272	7	24.45	SPATA31A3	NP_001076593.1	25	1	38.00
SLC16A5	XP_011523764.1	227	8	33.48	ANKRD20A1	NP_115626.2	811	2	2.34
ARMC7	NP_078861.1	123	11	84.95	CBWD3	NP_958861.2	215	3	13.26
NT5C	NP_001239306.1	435	2	4.37	FOXD4L3	NP_954586.4	265	1	3.58
JPT1	NP_001002032.1	114	0	0.00	PGM5	NP_068800.2	674	3	4.23

SUMO2	NP_001005849.1	2539	4	1.50	TMEM252	NP_694969.1	64	1	14.84
NUP85	NP_079120.1	689	9	12.41	FAM122A	NP_612206.5	115	2	16.52
GGA3	XP_011522865.1	762	7	8.73	PIP5K1B	NP_001362969.1	827	7	8.04
MRPS7	NP_057055.2	1492	6	3.82	PRKACG	NP_002723.2	2591	7	2.57
MIF4GD	XP_011523357.1	196	3	14.54	FXN	NP_000135.2	924	3	3.08
SLC25A19	XP_016880417.1	354	2	5.37	TJP2	NP_004808.2	667	6	8.55
GRB2	NP_987102.1	2342	4	1.62	FAM189A2	NP_001334924.1	108	4	35.18
TMEM94	XP_011523816.1	215	3	13.26	APBA1	XP_005252025.1	907	5	5.24
CASKIN2	NP_065804.2	1261	3	2.26	PTAR1	NP_001353867.1	119	0	0.00
TSEN54	NP_997229.2	289	2	6.57	C9orf135	XP_011516531.1	72	1	13.19
LLGL2	XP_016880117.1	729	3	3.91	MAMDC2	NP_694999.3	261	3	10.92
RECQL5	NP_004250.4	691	1	1.37	SMC5	NP_055925.2	1207	1	0.79
SMIM5	XP_016880433.1	33	0	0.00	KLF9	NP_001197.1	742	6	7.68
SMIM6	NP_001156469.1	0	0	#DIV/0!	TRPM3	NP_001353072.1	570	9	15.00
SAP30BP	NP_001288784.1	594	0	0.00	CEMIP2	NP_001129292.1	284	5	16.72
ITGB4	XP_006721929.1	939	4	4.05	ABHD17B	XP_016870276.1	170	6	33.53
GALK1	NP_001368914.1	964	2	1.97	C9orf85	NP_001351982.1	71	7	93.66
H3-3B	NP_005315.1	1576	5	3.01	C9orf57	NP_001358539.1	17	7	391.16
UNK	NP_001073888.2	1282	2	1.48	GDA	XP_016870824.1	467	0	0.00
UNC13D	NP_954712.1	466	6	12.23	ZFAND5	XP_024303433.1	400	5	11.87
WBP2	NP_001317428.1	303	6	18.81	TMC1	XP_016869745.1	335	2	5.67
TRIM47	NP_258411.2	359	9	23.81	ALDH1A1	NP_000680.2	1292	6	4.41
TRIM65	XP_011522803.1	322	7	20.65	ANXA1	XP_016870146.1	1543	6	3.69
MRPL38	NP_115867.2	498	6	11.45	RORB	NP_008845.2	630	6	9.05
FBF1	NP_001306122.1	347	6	16.43	TRPM6	NP_060132.3	414	6	13.77
ACOX1	NP_004026.2	905	8	8.40	C9orf40	NP_060468.2	65	5	73.07
TEN1	NP_001106795.2	268	4	14.18	CARNMT1	NP_689633.1	211	5	22.51
CDK3	NP_001249.1	1761	3	1.62	NMRK1	XP_016870363.1	707	7	9.41
EVPL	NP_001979.2	943	5	5.04	OSTF1	XP_006717116.1	294	5	16.16
SRP68	NP_001247431.1	845	7	7.87	PCSK5	NP_001358972.1	925	1	1.03
GALR2	XP_011523729.1	483	3	5.90	RFK	NP_060809.3	715	3	3.99
ZACN	NP_851321.2	368	2	5.16	GCNT1	NP_001091102.1	450	4	8.44
EXOC7	NP_056034.2	1056	2	1.80	PRUNE2	XP_016869838.1	323	0	0.00
FOXJ1	NP_001445.2	943	3	3.02	FOXB2	NP_001013757.1	320	3	8.91
RNF157	NP_001317430.1	343	10	27.70	VPS13A	NP_150648.2	635	3	4.49
UBALD2	NP_872371.1	68	0	0.00	GNA14	NP_004288.1	969	7	6.86
QRICH2	XP_016880696.1	86	2	22.09	GNAQ	NP_002063.2	1335	8	5.69
PRPSAP1	NP_002757.2	587	9	14.56	CEP78	NP_001336768.1	313	3	9.10
SPHK1	NP_892010.2	942	12	12.10	PSAT1	NP_478059.1	1049	9	8.15
UBE2O	XP_016880424.1	1493	8	5.09	TLE4	XP_011517254.1	891	8	8.53
AANAT	NP_001160051.1	530	10	17.92	TLE1	XP_016870555.1	911	9	9.38
RHBDF2	NP_001363159.1	491	13	25.15	SPATA31D4	NP_001138669.1	27	0	0.00
CYGB	NP_599030.1	612	12	18.63	SPATA31D3	NP_997299.2	27	0	0.00
PRCD	XP_016880502.1	174	3	16.38	SPATA31D1	XP_016870199.1	27	0	0.00
ST6GALNAC2	XP_005257011.1	340	10	27.94	RASEF	XP_005251787.1	493	12	23.12
ST6GALNAC1	XP_011523298.1	364	3	7.83	FRMD3	NP_001231888.1	347	10	27.38
MXRA7	NP_001008528.1	211	11	49.52	IDNK	XP_016870211.1	227	9	37.66
JMJD6	NP_001074930.1	763	13	16.19	UBQLN1	NP_444295.1	2414	9	3.54
METTL23	XP_006721742.1	194	10	48.97	GKAP1	NP_001129425.1	176	11	59.37
SRSF2	XP_016880431.1	1326	5	3.58	KIF27	XP_016870396.1	903	16	16.83
MFSD11	XP_011523544.1	168	2	11.31	C9orf64	NP_115683.3	170	10	55.88
MGAT5B	NP_001186101.1	492	4	7.72	HNRNPK	XP_016870158.1	1557	6	3.66
SEC14L1	NP_001034662.3	432	3	6.60	RMI1	XP_016870629.1	535	8	14.20
SEPTIN9	NP_001106963.1	863	5	5.50	SLC28A3	NP_071410.1	289	6	19.72
TNRC6C	XP_006722059.3	399	3	7.14	NTRK2	XP_016870240.1	2822	6	2.02
TMC6	XP_011522558.1	338	3	8.43	AGTPBP1	NP_001273646.1	335	7	19.85
TMC8	XP_024306391.1	352	4	10.79	NAA35	NP_001308811.1	296	7	22.47
C17orf99	XP_011522454.1	27	0	0.00	GOLM1	NP_057632.2	364	5	13.05
SYNGR2	NP_001350707.1	259	6	22.01	C9orf153	NP_001263295.1	25	7	265.99
TK1	NP_001333592.1	1400	7	4.75	ISCA1	NP_112202.2	428	5	11.10
AFMID	NP_001138998.1	312	2	6.09	TUT7	NP_001172003.1	444	4	8.56
BIRC5	NP_001012270.1	1685	4	2.26	GAS1	NP_002039.2	487	0	0.00
TMEM235	XP_011522922.1	715	0	0.00	DAPK1	NP_001275658.1	1220	1	0.78
SOCS3	NP_001365861.1	1553	5	3.06	CTSL	NP_001369686.1	1468	1	0.65
PGS1	XP_016880855.1	471	0	0.00	SPATA31E1	NP_849150.3	21	0	0.00
DNAH17	XP_024306781.1	775	2	2.45	CDK20	XP_016870052.1	652	2	2.91

CYTH1	NP_059430.2	735	2	2.58	SPIN1	NP_006708.2	325	0	0.00
USP36	XP_011523373.1	552	1	1.72	NXNL2	XP_011516578.1	141	0	0.00
TIMP2	NP_003246.1	868	5	5.47	C9orf47	NP_001001938.1	0	0	#DIV/0!
CEP295NL	NP_001230469.1	88	1	10.79	S1PR3	NP_005217.2	487	1	1.95
LGALS3BP	NP_005558.1	654	6	8.72	SHC3	NP_058544.3	807	3	3.53
CANT1	XP_024306332.1	462	3	6.17	CKS2	NP_001818.1	1139	3	2.50
C1QTNF1	XP_006721727.1	186	1	5.11	SECISBP2	NP_076982.3	264	0	0.00
ENGASE	NP_001036038.1	322	1	2.95	SEMA4D	NP_001358131.1	550	1	1.73
RBFOX3	NP_001372772.1	1788	2	1.06	GADD45G	NP_006696.1	554	1	1.71
ENPP7	XP_011523039.1	233	1	4.08	DIRAS2	NP_060064.2	2021	3	1.41
CBX2	XP_011523684.1	790	3	3.61	SYK	NP_003168.2	2283	3	1.25
CBX8	NP_065700.1	648	3	4.40	AUH	NP_001293119.1	658	0	0.00
CBX4	NP_003646.2	682	3	4.18	NFIL3	XP_016870232.1	767	2	2.48
TBC1D16	XP_005257107.1	412	0	0.00	ROR2	NP_004551.2	1067	3	2.67
CCDC40	NP_060420.2	282	1	3.37	SPTLC1	NP_001268232.1	766	1	1.24
GAA	NP_000143.2	649	3	4.39	IARS1	NP_001365512.1	1411	2	1.35
EIF4A3	XP_011523824.1	1979	2	0.96	NOL8	XP_016870365.1	461	1	2.06
CARD14	NP_001353314.1	740	3	3.85	CENPP	NP_001012267.1	330	3	8.64
SGSH	XP_005257640.1	291	3	9.79	OGN	NP_077727.3	926	3	3.08
SLC26A11	XP_016879994.1	211	4	18.01	OMD	NP_005005.1	653	3	4.36
RNF213	XP_005257602.2	741	5	6.41	ASPN	NP_060150.4	1111	5	4.28
ENDOV	XP_011522972.1	313	4	12.14	ECM2	NP_001184225.1	626	4	6.07
NPTX1	NP_002513.2	739	6	7.71	IPPK	NP_073592.1	157	0	0.00
RPTOR	NP_065812.1	1509	7	4.41	BICD2	NP_056065.1	458	1	2.07
CHMP6	NP_078867.2	487	2	3.90	ZNF484	NP_001248387.1	142	2	13.38
BAIAP2	NP_001372058.1	721	6	7.91	FGD3	XP_016870763.1	745	3	3.83
AATK	NP_001073864.2	428	6	13.32	SUSD3	NP_659443.1	174	1	5.46
CEP131	NP_001009811.2	321	4	11.84	CARD19	NP_115686.3	62	0	0.00
TEPSIN	NP_653280.1	206	6	27.67	NINJ1	NP_004139.2	201	1	4.73
NDUFAF8	NP_001340331.1	201	5	23.63	WNK2	NP_006639.3	385	1	2.47
SLC38A10	NP_001033073.1	238	4	15.97	FAM120AOS	NP_942138.2	63	1	15.08
BAHCC1	NP_001278253.1	228	0	0.00	FAM120A	XP_011516714.1	205	2	9.27
ACTG1	NP_001186883.1	3580	5	1.33	PHF2	XP_005252108.1	513	0	0.00
FSCN2	NP_001070650.1	345	3	8.26	BARX1	NP_067545.3	435	4	8.74
FAAP100	XP_006722174.1	168	1	5.65	PTPDC1	XP_016869769.1	781	8	9.73
NPLOC4	NP_060391.2	780	3	3.65	ZNF169	NP_003439.2	209	4	18.18
TSPAN10	NP_114151.3	225	0	0.00	NUTM2F	NP_060031.1	41	2	46.34
PDE6G	XP_016880225.1	390	1	2.44	MFSD14B	NP_115947.2	227	5	20.92
OXLD1	NP_001034931.1	68	3	41.91	FBP2	NP_003828.2	664	0	0.00
CCDC137	XP_016880062.1	71	0	0.00	FBP1	NP_000498.2	924	0	0.00
ARL16	NP_001035114.2	1192	3	2.39	AOPEP	XP_011517423.1	364	9	23.49
HGS	NP_004703.1	1199	3	2.38	FANCC	XP_006717064.1	601	7	11.06
MRPL12	NP_002940.2	1165	3	2.45	PTCH1	NP_001077072.1	1295	8	5.87
SLC25A10	NP_001257882.1	586	7	11.35	ERCC6L2	XP_011516943.1	839	9	10.19
GCGR	NP_000151.1	597	3	4.77	HSD17B3	XP_016870163.1	530	5	8.96
MCRIP1	XP_011521874.1	54	1	17.59	SLC35D2	XP_011516466.1	337	6	16.91
PPP1R27	NP_001007534.1	774	6	7.36	ZNF367	NP_710162.1	475	5	10.00
P4HB	NP_000909.2	2290	7	2.90	HABP4	NP_055097.2	545	8	13.94
ARHGDIA	NP_001288171.1	1071	9	7.98	CDC14B	XP_011517454.2	935	6	6.10
ALYREF	NP_005773.3	1426	1	0.67	PRXL2C	XP_005251841.1	170	1	5.59
ANAPC11	NP_001276349.1	1022	7	6.51	ZNF510	XP_005251865.1	130	1	7.31
NPB	NP_683694.1	457	0	0.00	ZNF782	XP_016869832.1	157	0	0.00
PCYT2	NP_001243362.1	666	5	7.13	NUTM2G	NP_001038942.1	26	1	36.54
SIRT7	XP_011521882.1	1412	9	6.05	MFSD14C	XP_024303466.1	122	0	0.00
MAFG	NP_002350.1	402	6	14.18	CTSV	NP_001324.2	539	0	0.00
PYCR1	NP_722546.1	1004	6	5.68	CCDC180	NP_065944.3	149	1	6.38
MYADML2	NP_001138585.2	109	3	26.15	TDRD7	NP_055105.2	971	6	5.87
NOTUM	NP_848588.3	499	6	11.42	TMOD1	NP_001159588.1	516	5	9.20
ASPSCR1	XP_016880525.1	598	6	9.53	TSTD2	NP_640339.4	72	5	65.97
CENPX	XP_024306407.1	271	4	14.02	NCBP1	NP_002477.1	1595	6	3.57
LRRC45	NP_659436.1	167	1	5.69	XPA	XP_006717341.1	891	8	8.53
RAC3	NP_005043.1	2201	3	1.29	FOXE1	NP_004464.2	861	4	4.41
DCXR	NP_001182147.1	410	4	9.27	TRMO	XP_011517078.1	188	5	25.26
RFNG	NP_002908.1	324	3	8.80	HEMGN	NP_932095.1	354	2	5.37
GPS1	XP_024306483.1	754	6	7.56	ANP32B	NP_006392.1	764	6	7.46
DUS1L	XP_006722352.1	554	6	10.29	NANS	NP_061819.2	496	4	7.66

FASN	NP_004095.4	1418	4	2.68	TRIM14	XP_016870841.1	344	2	5.52
CCDC57	NP_932348.2	142	2	13.38	CORO2A	NP_438171.1	743	8	10.23
SLC16A3	NP_001035888.1	541	3	5.27	TBC1D2	NP_060891.3	760	0	0.00
CSNK1D	NP_001350678.1	1359	7	4.89	GABBR2	NP_005449.5	1160	2	1.64
CD7	NP_006128.1	516	1	1.84	ANKS6	XP_005251851.1	1161	5	4.09
SECTM1	NP_002995.1	270	5	17.59	GALNT12	NP_078918.3	332	3	8.58
TEX19	XP_016880127.1	125	2	15.20	COL15A1	NP_001846.3	502	1	1.89
UTS2R	NP_001368826.1	508	8	14.96	TGFBR1	NP_001293139.1	1954	5	2.43
OGFOD3	NP_078924.1	68	4	55.88	ALG2	NP_149078.1	566	2	3.36
HEXD	NP_775891.2	119	0	0.00	SEC61B	NP_006799.1	1002	1	0.95
CYBC1	XP_016880562.1	158	2	12.02	NR4A3	NP_775292.1	609	2	3.12
NARF	NP_114174.1	248	1	4.03	STX17	NP_060389.2	904	2	2.10
FOXK2	NP_004505.2	940	7	7.86	ERP44	NP_055866.1	812	3	3.51
WDR45B	XP_005256434.1	362	4	12.35	INVS	NP_055240.2	1245	4	3.05
RAB40B	NP_006813.1	602	9	17.75	TEX10	NP_001155056.1	682	1	1.39
FN3KRP	NP_078895.2	183	9	62.29	MSANTD3-TN	NP_001185741.1	82	0	0.00
FN3K	NP_071441.1	204	7	46.57	MSANTD3	NP_542386.1	164	0	0.00
TBCD	NP_005984.3	736	10	19.86	TMEFF1	NP_003683.2	267	0	0.00
ZNF750	NP_078978.2	227	6	41.85	CAVIN4	NP_001018126.1	274	0	0.00
B3GNTL1	XP_011521834.1	443	6	23.39	PLPPR1	NP_060223.2	278	0	0.00
METRNL	NP_001004431.1	230	7	57.82	BAAT	NP_001361644.1	338	2	5.62
USP14	NP_005142.1	999	6	11.41	MRPL50	NP_061924.1	328	1	2.90
THOC1	NP_005122.2	596	11	31.88	ZNF189	NP_003443.2	257	0	0.00
COLEC12	XP_011524043.1	369	9	38.62	ALDOB	NP_000026.2	1078	1	0.88
CETN1	NP_004057.1	1402	10	10.42	PGAP4	XP_024303469.1	71	0	0.00
CLUL1	NP_001275965.1	196	14	96.93	RNF20	NP_062538.5	1244	1	0.76
TYMS	NP_001062.1	1832	10	6.91	GRIN3A	NP_597702.2	666	1	1.43
ENOSF1	XP_024306976.1	443	15	40.21	PPP3R2	NP_671709.2	1093	1	0.87
YES1	XP_024307015.1	1619	14	9.66	CYLC2	NP_001331.1	77	0	0.00
ADCYAP1	XP_005258138.2	871	10	12.12	SMC2	XP_016869695.1	1472	1	0.65
METTL4	NP_073751.3	236	15	63.56	OR13F1	NP_001004485.1	56	0	0.00
NDC80	NP_006092.1	1042	11	10.03	OR13C4	NP_001001919.1	45	0	0.00
SMCHD1	XP_011523944.1	446	12	25.56	OR13C3	NP_001001961.1	39	0	0.00
EMILIN2	NP_114437.2	144	8	52.78	OR13C8	NP_001004483.1	97	0	0.00
LPIN2	XP_005258234.1	699	14	19.03	OR13C5	NP_001004482.1	53	0	0.00
MYOM1	NP_062830.1	503	10	18.89	OR13C2	NP_001004481.1	25	0	0.00
MYL12A	NP_001289978.1	1386	11	7.54	OR13C9	NP_001001956.1	33	0	0.00
MYL12B	NP_291024.1	1499	13	8.24	OR13D1	NP_001004484.1	46	0	0.00
TGIF1	NP_775299.1	555	5	8.56	NIPSNAP3A	NP_056284.1	127	2	14.96
DLGAP1	NP_004737.2	1260	14	10.56	NIPSNAP3B	NP_060846.2	154	2	12.34
AKAIN1	NP_001317482.1	1	0	0.00	ABCA1	XP_005251830.1	1343	4	2.83
ZBTB14	XP_024307029.1	413	17	39.10	SLC44A1	NP_001317660.1	454	4	8.37
EPB41L3	NP_001371623.1	698	13	17.69	FSD1L	XP_011517380.2	262	5	18.13
TMEM200C	NP_001073678.1	105	4	36.19	FKTN	XP_016869958.1	418	4	9.09
L3MBTL4	XP_016881563.1	360	7	18.47	TAL2	NP_005412.1	185	5	25.67
ARHGAP28	XP_005258203.1	297	9	28.79	TMEM38B	NP_060582.1	272	5	17.46
LAMA1	NP_005550.2	725	9	11.79	ZNF462	XP_016870485.1	411	9	20.80
LRRC30	NP_001099051.1	480	14	27.71	RAD23B	NP_002865.1	2447	5	1.94
PTPRM	XP_011524010.1	870	13	14.19	KLF4	NP_004226.3	1847	5	2.57
RAB12	NP_001020471.2	1913	9	4.47	ACTL7B	NP_006677.1	1039	4	3.66
MTCL1	NP_001365136.1	281	6	20.28	ACTL7A	NP_006678.1	952	4	3.99
NDUFV2	NP_066552.2	889	11	11.75	ELP1	NP_003631.2	1017	7	6.54
ANKRD12	NP_056023.3	383	13	32.24	ABITRAM	XP_011517118.1	188	5	25.26
TWSG1	NP_065699.1	497	8	15.29	CTNNAL1	XP_005252348.1	665	6	8.57
RALBP1	NP_006779.1	405	3	7.04	TMEM245	XP_011516751.1	255	5	18.63
PPP4R1	XP_011524078.1	664	18	25.75	FRRS1L	NP_055149.3	829	4	4.58
RAB31	XP_016881017.1	944	12	12.08	EPB41L4B	XP_016870304.1	456	2	4.17
TXNDC2	NP_001091999.1	857	11	12.19	PTPN3	XP_011517190.1	821	1	1.16
VAPA	NP_003565.4	1376	12	8.28	PALM2AKAP2	NP_001032370.1	299	3	9.53
APCDD1	NP_694545.1	145	5	32.76	C9orf152	NP_001013011.2	92	0	0.00
NAPG	NP_003817.1	365	12	31.23	TXN	NP_001231867.1	2466	4	1.54
PIEZO2	XP_016881407.1	273	8	27.84	TXNDC8	XP_016870071.1	733	7	9.07
SLC35G4	NP_001269229.1	63	0	0.00	SVEP1	NP_699197.3	638	4	5.96
GNAL	NP_892023.1	1487	5	3.19	MUSK	XP_005252051.1	1326	4	2.87
CHMP1B	NP_065145.2	441	10	21.54	LPAR1	XP_016869893.1	910	3	3.13
MPPE1	XP_024307005.1	331	12	34.44	OR2K2	NP_995581.1	92	1	10.33

IMPA2	NP_055029.1	647	14	20.56	ECPAS	NP_001350685.1	574	3	4.96
ANKRD62	NP_001264262.1	810	4	4.69	ZNF483	NP_001007170.1	305	2	6.23
CIDEA	NP_001270.1	494	2	3.85	PTGR1	NP_001139581.1	440	2	4.32
TUBB6	NP_001290454.1	1261	11	8.29	DNAJC25-GN(	NP_004116.2	415	2	4.58
AFG3L2	XP_011523903.1	1275	14	10.43	DNAJC25	NP_001015882.2	430	2	4.42
PRELID3A	NP_001135877.1	148	3	19.26	GNG10	NP_001185593.1	787	1	1.21
SPIRE1	XP_024306993.1	330	8	23.03	SHOC1	XP_016869829.1	56	0	0.00
CEP76	XP_005258206.1	318	13	38.83	UGCG	NP_003349.1	750	1	1.27
PSMG2	NP_064617.2	447	13	27.63	SUSD1	NP_071931.2	193	4	19.69
PTPN2	XP_024306996.1	1323	12	8.62	PTBP3	NP_001231826.1	468	4	8.12
SEH1L	XP_011524044.1	587	11	17.80	HSDL2	NP_115679.2	726	4	5.23
CEP192	XP_005258164.1	371	6	15.36	KIAA1958	NP_001273965.1	187	3	15.24
LDLRAD4	XP_024307017.1	235	5	20.21	INIP	NP_001316516.1	193	3	14.77
FAM210A	NP_689565.2	308	8	24.67	SNX30	XP_011516993.1	359	0	0.00
RNMT	NP_001365063.1	499	8	15.23	SLC46A2	NP_149040.3	303	5	15.68
MC5R	NP_005904.1	429	7	15.50	ZFP37	NP_003399.1	339	4	11.21
MC2R	XP_016881270.1	523	6	10.90	SLC31A2	NP_001851.1	617	4	6.16
ZNF519	NP_660330.2	304	9	28.12	FKBP15	XP_006717081.1	1446	9	5.91
POTEC	NP_001131143.1	926	1	1.03	SLC31A1	NP_001850.1	511	4	7.44
ANKRD30B	NP_001354536.1	849	1	1.12	CDC26	XP_016870062.1	554	1	1.71
ROCK1	NP_005397.1	2034	8	3.74	PRPF4	NP_004688.2	982	3	2.90
GREB1L	XP_006722610.1	205	0	0.00	RNF183	XP_016869757.1	286	2	6.64
ESCO1	XP_011524101.1	648	8	11.73	WDR31	NP_660284.1	1084	2	1.75
SNRPD1	NP_008869.1	1563	2	1.22	BSPRY	NP_001304872.1	311	2	6.11
ABHD3	NP_001295185.1	353	3	8.07	HDHD3	NP_112496.1	269	5	17.66
MIB1	NP_065825.1	2649	5	1.79	ALAD	NP_001304674.1	760	3	3.75
GATA6	NP_005248.2	1548	9	5.52	POLE3	NP_059139.3	800	4	4.75
CTAGE1	NP_758441.2	172	3	16.57	C9orf43	NP_001265559.1	53	0	0.00
RBBP8	NP_976037.1	839	6	6.79	RGS3	NP_652759.3	664	0	0.00
CABLES1	NP_001094089.1	396	9	21.59	ZNF618	XP_005251749.1	669	6	8.52
TMEM241	XP_016881532.1	241	3	11.83	AMBP	NP_001624.1	869	7	7.65
RIOK3	NP_003822.2	538	1	1.77	KIF12	XP_005251740.1	788	11	13.26
RMC1	NP_037458.3	181	2	10.50	COL27A1	XP_011517440.1	408	5	11.64
NPC1	XP_005258336.1	746	3	3.82	ORM1	NP_000598.2	541	5	8.78
ANKRD29	XP_016881050.1	964	5	4.93	ORM2	NP_000599.1	569	4	6.68
LAMA3	XP_011524280.1	676	5	7.03	AKNA	XP_005252305.1	247	4	15.38
TTC39C	NP_001129465.1	177	2	10.73	WHRN	XP_011516789.1	882	4	4.31
CABYR	NP_619585.1	301	2	6.31	ATP6V1G1	NP_004879.1	400	0	0.00
OSBPL1A	XP_016881019.1	497	5	9.56	TMEM268	XP_016869918.1	11	7	604.51
IMPACT	NP_060909.2	224	1	4.24	TNFSF15	NP_005109.2	735	5	6.46
HRH4	NP_067637.2	432	1	2.20	TNFSF8	NP_001239219.1	576	4	6.60
ZNF521	XP_011524211.1	545	5	8.72	TNC	XP_005252032.1	811	2	2.34
SS18	NP_005628.2	361	2	5.26	PAPPA	NP_002572.2	385	1	2.47
PSMA8	NP_653263.2	959	4	3.96	ASTN2	NP_937831.1	514	4	7.39
TAF4B	NP_001280654.1	359	0	0.00	TRIM32	NP_001093149.1	956	4	3.97
KCTD1	NP_001245151.1	289	2	6.57	TLR4	NP_612564.1	3182	7	2.09
AQP4	NP_001304313.1	1781	1	0.53	BRINP1	NP_055433.2	750	3	3.80
CHST9	XP_016881522.1	189	1	5.03	CDK5RAP2	NP_001258968.1	635	4	5.98
CDH2	XP_016881003.1	2178	6	2.62	MEGF9	NP_001073966.2	212	4	17.92
DSC3	NP_001932.2	703	7	9.46	FBXW2	XP_006717114.1	779	4	4.88
DSC2	NP_077740.1	650	7	10.23	PSMD5	NP_001257356.1	774	4	4.91
DSC1	NP_077739.1	334	6	17.06	PHF19	NP_001273769.1	493	7	13.49
DSG1	NP_001933.2	643	9	13.30	TRAF1	NP_001177874.1	1179	4	3.22
DSG4	NP_817123.1	445	7	14.94	C5	NP_001304092.1	621	4	6.12
DSG3	NP_001935.2	515	7	12.91	CNTRL	XP_011516468.1	836	3	3.41
DSG2	NP_001934.2	760	8	10.00	RAB14	NP_057406.2	1601	5	2.97
TTR	NP_000362.1	1852	7	3.59	GSN	XP_024303290.1	1301	6	4.38
B4GALT6	XP_005258444.1	343	6	16.62	STOM	NP_001257456.1	845	5	5.62
SLC25A52	NP_001029344.4	102	1	9.31	DAB2IP	NP_115941.2	987	5	4.81
TRAPPC8	XP_016881105.1	602	4	6.31	TTLL11	NP_001132914.2	235	3	12.13
RNF125	XP_011524347.1	245	3	11.63	NDUFA8	NP_001305124.1	660	2	2.88
RNF138	NP_057355.2	542	3	5.26	MORN5	NP_940871.2	142	2	13.38
MEP1B	NP_005916.2	485	11	21.55	LHX6	XP_011516823.1	736	1	1.29
GAREM1	NP_073588.1	121	9	70.66	RBM18	NP_149108.1	68	1	13.97
KLHL14	NP_065856.1	185	9	46.21	MRRF	NP_001333268.1	1121	0	0.00
CCDC178	NP_945346.2	126	12	90.47	PTGS1	NP_000953.2	942	2	2.02

ASXL3	XP_005258413.1	331	11	31.57	OR1J1	NP_001004451.1	70	0	0.00
NOL4	NP_001185477.1	604	2	3.15	OR1J2	XP_024303284.1	146	0	0.00
DTNA	XP_016881065.1	663	12	17.19	OR1J4	NP_001004452.1	78	0	0.00
MAPRE2	NP_001137299.1	861	12	13.24	OR1N1	NP_036495.1	39	0	0.00
ZNF397	NP_115723.1	246	12	46.34	OR1N2	NP_001004457.2	66	2	28.79
ZSCAN30	XP_016881004.1	286	0	0.00	OR1L8	XP_016869773.1	101	0	0.00
ZNF24	NP_001362744.1	370	4	10.27	OR1Q1	NP_036496.1	135	3	21.11
ZNF396	XP_016881178.1	204	12	55.88	OR1B1	XP_016870185.1	62	0	0.00
INO80C	NP_919257.2	295	5	16.10	OR1L1	NP_001005236.3	78	0	0.00
GALNT1	NP_065207.2	368	8	20.65	OR1L3	NP_001005234.1	42	0	0.00
C18orf21	XP_005258421.1	59	10	161.01	OR1L4	NP_001005235.1	31	0	0.00
RPRD1A	NP_060640.2	469	12	24.31	OR1L6	NP_001004453.2	39	0	0.00
SLC39A6	NP_036451.4	335	8	22.69	OR5C1	NP_001001923.1	49	0	0.00
ELP2	NP_001229804.1	597	8	12.73	OR1K1	NP_543135.1	55	0	0.00
MOCOS	NP_060417.4	399	5	11.90	PDCL	NP_005379.3	418	2	4.55
FHOD3	XP_011524491.1	367	1	2.59	RC3H2	NP_001341408.1	378	5	12.57
TPGS2	XP_016881191.1	234	2	8.12	ZBTB6	NP_006617.1	230	1	4.13
KIAA1328	XP_005258372.2	316	2	6.01	ZBTB26	NP_065975.1	414	3	6.88
CELF4	NP_001340668.1	959	9	8.92	RABGAP1	XP_016870056.1	692	6	8.24
PIK3C3	NP_002638.2	2172	4	1.75	GPR21	NP_005285.1	179	4	21.23
RIT2	NP_002921.1	2139	5	2.22	STRBP	XP_024303373.1	426	6	13.38
SYT4	NP_065834.1	1296	3	2.20	CRB2	NP_775960.4	1006	8	7.55
SETBP1	XP_024306917.1	1802	6	3.16	DENND1A	NP_001339894.1	238	6	23.95
SLC14A2	XP_024307038.1	339	2	5.60	LHX2	NP_004780.3	1110	5	4.28
SLC14A1	XP_005258386.1	438	1	2.17	NEK6	NP_001159639.1	591	4	6.43
EPG5	XP_016881379.1	167	4	22.75	PSMB7	NP_002790.1	1432	7	4.64
SIGLEC15	NP_998767.1	135	1	7.04	ADGRD2	XP_006717411.2	359	3	7.94
PSTPIP2	XP_011524555.1	670	2	2.84	NR5A1	NP_004950.2	1158	4	3.28
ATP5F1A	NP_001244263.1	1826	1	0.52	NR6A1	NP_001480.3	583	5	8.15
HAUS1	NP_612452.1	485	2	3.92	OLFML2A	NP_872293.2	297	2	6.40
C18orf25	NP_659492.1	123	2	15.45	WDR38	NP_001263303.1	2079	3	1.37
RNF165	NP_689683.2	351	3	8.12	RPL35	NP_009140.1	1287	2	1.48
LOXHD1	NP_001138944.1	246	4	15.45	ARPC5L	NP_112240.1	625	1	1.52
ST8SIA5	NP_001294916.1	441	8	17.23	GOLGA1	NP_002068.2	479	2	3.97
PIAS2	XP_005258434.1	864	8	8.80	SCAI	NP_001138349.1	203	1	4.68
KATNAL2	NP_001340828.1	936	6	6.09	PPP6C	NP_001116841.1	2215	6	2.57
ELOA2	NP_057511.2	209	4	18.18	RABEPK	XP_005251697.1	654	4	5.81
HDHD2	XP_016881528.1	263	6	21.67	HSPA5	NP_005338.1	3029	6	1.88
IER3IP1	NP_057181.1	329	6	17.32	GAPVD1	XP_016870089.1	1351	5	3.52
SKOR2	NP_001264992.1	208	7	31.97	MAPKAP1	NP_077022.1	586	3	4.86
SMAD2	XP_016881235.1	2074	4	1.83	PBX3	XP_006717193.1	583	1	1.63
ZBTB7C	XP_011524171.1	279	4	13.62	MVB12B	NP_258257.1	189	3	15.08
CTIF	XP_016881589.1	316	8	24.05	LMX1B	NP_001167617.1	927	4	4.10
SMAD7	NP_001177750.1	1190	8	6.39	ZBTB43	NP_001129248.1	300	3	9.50
DYM	XP_016881284.1	226	7	29.42	ZBTB34	XP_005252046.1	233	1	4.08
C18orf32	NP_001186275.1	850	4	4.47	RALGPS1	XP_011517530.1	614	7	10.83
RPL17-C18orf	NP_001186284.1	840	0	0.00	ANGPTL2	NP_036230.1	668	3	4.27
RPL17	NP_001356490.1	1224	0	0.00	GARNL3	XP_005252324.1	208	4	18.27
LIPG	XP_005258447.1	459	5	10.35	SLC2A8	XP_006717147.1	2604	9	3.28
ACAA2	NP_006102.2	766	2	2.48	ZNF79	NP_009066.2	268	2	7.09
MYO5B	NP_001073936.1	1474	12	7.73	RPL12	NP_000967.1	1382	2	1.37
CFAP53	NP_659457.2	160	12	71.25	LRSAM1	NP_001371071.1	968	1	0.98
MBD1	NP_001191070.1	191	2	9.95	NIBAN2	NP_001030611.1	300	4	12.67
CXXC1	NP_055408.2	748	7	8.89	STXBP1	NP_001361243.1	1503	3	1.90
SKA1	NP_659497.1	643	9	13.30	CFAP157	XP_011516861.3	69	0	0.00
MAPK4	XP_016881328.1	2279	11	4.59	PTRH1	NP_001332906.1	323	3	8.82
MRO	NP_001120647.1	226	7	29.42	TTC16	NP_659402.1	627	1	1.52
ME2	NP_002387.1	598	6	9.53	TOR2A	XP_011516856.1	145	0	0.00
ELAC1	NP_061166.1	589	13	20.97	SH2D3C	NP_733745.1	377	2	5.04
SMAD4	NP_005350.1	2110	12	5.40	CDK9	NP_001252.1	1175	0	0.00
MEX3C	NP_057710.3	697	13	17.72	FPGS	XP_005251921.1	1505	3	1.89
DCC	NP_005206.2	673	5	7.06	ENG	NP_001108225.1	1043	2	1.82
MBD2	NP_003918.1	482	2	3.94	AK1	XP_016869917.1	891	2	2.13
POLI	NP_001338550.1	1325	0	0.00	ST6GALNAC6	XP_016870148.1	224	1	4.24
STARD6	NP_001358030.1	141	11	74.11	ST6GALNAC4	NP_778204.1	352	2	5.40
C18orf54	XP_016881056.1	69	1	13.77	PIP5KL1	NP_001128691.1	460	0	0.00

DYNAP	XP_011524225.1	47	6	121.27	DPM2	NP_001365366.1	308	2	6.17
RAB27B	XP_006722581.1	480	9	17.81	FAM102A	NP_001030331.1	221	1	4.30
CCDC68	XP_016881500.1	199	10	47.74	NAIF1	NP_931045.1	162	2	11.73
TCF4	NP_001230155.2	1137	2	1.67	SLC25A25	NP_001317917.1	346	0	0.00
TXNL1	NP_004777.1	562	5	8.45	PTGES2	NP_079348.1	567	0	0.00
WDR7	NP_001369416.1	805	4	4.72	LCN2	NP_005555.2	1175	1	0.81
BOD1L2	NP_001244893.1	76	0	0.00	C9orf16	XP_011517306.1	84	0	0.00
ST8SIA3	NP_056963.2	1005	1	0.95	CIZ1	XP_016870083.1	307	2	6.19
ONECUT2	NP_004843.2	477	1	1.99	DNM1	NP_004399.2	2166	2	0.88
FECH	NP_001358023.1	956	2	1.99	GOLGA2	XP_005251989.1	821	2	2.31
NARS1	XP_005266757.1	876	7	7.59	SWI5	NP_001366196.1	141	0	0.00
ATP8B1	XP_011524325.1	928	0	0.00	TRUB2	NP_001316790.1	909	1	1.05
NEDD4L	XP_006722489.1	1554	0	0.00	COQ4	NP_057119.3	287	1	3.31
ALPK2	NP_443179.3	214	6	26.63	SLC27A4	XP_016869711.1	516	1	1.84
MALT1	NP_006776.1	505	0	0.00	URM1	NP_112176.1	419	1	2.27
ZNF532	XP_016881300.1	399	6	14.28	CERCAM	XP_016870283.1	268	0	0.00
SEC11C	NP_150596.1	856	9	9.99	ODF2	NP_702914.1	394	1	2.41
GRP	XP_011524235.1	553	2	3.44	GLE1	XP_011516853.1	717	3	3.97
RAX	NP_038463.2	487	3	5.85	SPTAN1	NP_001362247.1	1165	4	3.26
CPLX4	NP_857637.1	216	9	39.58	DYNC2I2	XP_011517481.1	359	2	5.29
LMAN1	NP_005561.1	658	10	14.44	SET	NP_001116293.1	1555	2	1.22
CCBE1	XP_024306859.1	299	2	6.35	PKN3	NP_037487.2	926	2	2.05
PMAIP1	NP_001369546.1	670	1	1.42	ZDHHC12	NP_001304952.2	142	1	6.69
MC4R	NP_005903.2	924	5	5.14	ZER1	XP_016869676.1	213	0	0.00
CDH20	XP_024306933.1	923	8	8.23	TBC1D13	NP_060671.3	276	3	10.33
RNF152	XP_016881101.1	465	14	28.60	ENDOG	XP_011516649.1	603	3	4.73
PIGN	XP_016881175.1	414	14	32.12	SPOUT1	XP_016870293.1	568	4	6.69
RELCH	NP_001333159.1	621	14	21.42	KYAT1	NP_001339927.1	520	4	7.31
TNFRSF11A	XP_011524546.1	553	4	6.87	LRRC8A	NP_001120716.1	658	3	4.33
ZCCHC2	NP_060212.4	592	8	12.84	PHYHD1	NP_777593.2	252	1	3.77
PHLPP1	NP_919431.2	1043	8	7.29	DOLK	NP_055723.1	458	2	4.15
BCL2	NP_000624.2	723	1	1.31	NUP188	NP_056169.1	327	2	5.81
KDSR	XP_005266734.1	416	14	31.97	SH3GLB2	NP_001356843.1	249	0	0.00
VPS4B	NP_004860.2	649	16	23.42	MIGA2	NP_116198.3	138	0	0.00
SERPINB5	NP_002630.2	1128	12	10.11	DOLPP1	NP_065171.2	526	2	3.61
SERPINB12	XP_005266835.2	321	15	44.39	CRAT	NP_001333478.2	427	0	0.00
SERPINB13	NP_001294852.1	261	8	29.12	PTPA	XP_011517140.1	1372	1	0.69
SERPINB4	NP_778206.1	355	7	18.73	IER5L	NP_982258.2	184	0	0.00
SERPINB3	NP_008850.1	749	7	8.88	C9orf50	XP_011516964.1	70	0	0.00
SERPINB11	NP_536723.2	226	5	21.02	NTMT1	NP_001273726.1	313	0	0.00
SERPINB7	XP_024307046.1	592	12	19.26	ASB6	NP_001189332.1	1185	2	1.60
SERPINB2	XP_024306960.1	824	9	10.38	PRRX2	NP_057391.1	726	5	6.54
SERPINB10	NP_005015.1	319	8	23.82	PTGES	NP_004869.1	413	0	0.00
HMSD	XP_016881199.1	309	6	18.45	TOR1B	XP_005251984.1	276	4	13.77
SERPINB8	NP_001335296.1	585	9	14.61	TOR1A	NP_000104.1	535	5	8.88
CDH7	NP_001349367.1	443	7	15.01	C9orf78	XP_011517082.1	170	6	33.53
CDH19	NP_066976.1	529	6	10.77	USP20	NP_001103773.2	650	5	7.31
DSEL	NP_115536.2	236	4	16.10	FNBP1	XP_005251891.1	790	7	8.42
TMX3	NP_001337443.1	700	8	10.86	GPR107	NP_001130029.1	296	1	3.21
CCDC102B	XP_011524476.1	148	9	57.77	NCS1	NP_055101.2	1517	1	0.63
DOK6	NP_689934.2	526	12	21.67	HMCN2	NP_001278744.1	890	5	5.34
CD226	XP_006722437.1	571	4	6.65	ASS1	XP_016870218.1	1046	6	5.45
RTTN	XP_016881182.1	580	10	16.38	FUBP3	NP_003925.1	523	3	5.45
SOCS6	XP_016881575.1	563	4	6.75	PRDM12	NP_067632.2	230	3	12.39
CBLN2	XP_016881048.1	622	2	3.05	EXOSC2	NP_055100.2	1092	3	2.61
NETO1	XP_016881506.1	469	8	16.20	ABL1	NP_009297.2	2555	11	4.09
FBXO15	XP_011524158.1	617	4	6.16	QRFP	NP_937823.1	311	3	9.16
TIMM21	NP_054896.2	281	10	33.81	FIBCD1	NP_001138578.1	346	3	8.24
CYB5A	NP_001177736.1	1184	6	4.81	LAMC3	XP_011516423.1	513	8	14.81
C18orf63	NP_001167594.1	96	3	29.69	AIF1L	NP_001172024.1	1192	3	2.39
DIPK1C	NP_001037834.2	294	10	32.31	NUP214	NP_005076.3	1060	9	8.07
CNDP2	NP_001357177.1	821	7	8.10	FAM78A	XP_011516870.1	208	0	0.00
CNDP1	NP_116038.4	844	6	6.75	PLPP7	NP_116117.3	267	2	7.12
ZNF407	NP_060227.2	254	11	41.14	PRRC2B	NP_037450.2	315	4	12.06
ZADH2	NP_787103.1	325	10	29.23	POMT1	NP_001340122.2	484	2	3.93
TSHZ1	NP_001295139.1	296	8	25.67	UCK1	NP_001248379.1	896	4	4.24

SMIM21	NP_001032408.1	48	6	118.74	RAPGEF1	NP_001364866.1	951	2	2.00
ZNF516	XP_016881586.1	324	8	23.46	MED27	NP_001240810.1	282	1	3.37
ZNF236	NP_001293018.1	326	13	37.88	NTNG2	XP_016870701.1	670	2	2.84
MBP	NP_001020272.1	1543	2	1.23	SETX	NP_055861.3	1286	4	2.95
GALR1	NP_001471.2	532	11	19.64	TTF1	NP_031370.2	1002	2	1.90
SALL3	NP_741996.2	458	6	12.44	CFAP77	NP_997300.1	46	0	0.00
ATP9B	XP_011524266.1	373	9	24.13	BARHL1	NP_064448.1	713	3	4.00
NFATC1	NP_001265598.1	1244	4	3.39	DDX31	NP_001309269.1	1392	9	6.14
CTDP1	NP_004706.3	903	7	8.66	GTF3C4	NP_036336.2	376	3	7.58
KCNG2	XP_016881193.1	592	0	0.00	AK8	NP_001358701.1	745	10	12.75
SLC66A2	NP_001139817.1	167	3	22.75	SPACA9	XP_016869720.1	104	8	73.07
HSBP1L1	NP_001129652.1	24	2	113.09	TSC1	NP_001155899.1	987	8	7.70
TXNL4A	NP_001292486.1	835	4	7.00	GFI1B	XP_016870664.1	693	8	10.97
RBFA	NP_079081.2	131	0	0.00	GTF3C5	XP_016870804.1	518	13	23.84
ADNP2	XP_005266713.1	176	1	9.81	CEL	NP_001798.3	633	9	13.51
PARD6G	NP_115899.1	638	4	11.91	RALGDS	NP_001035827.1	591	9	14.47
OR4F17	NP_001005240.1	42	2	90.47	GBGT1	NP_001269561.1	306	7	21.73
PLPP2	NP_003703.1	488	9	31.85	OBP2B	XP_016870143.1	134	5	35.45
MIER2	NP_060020.1	301	9	47.34	ABO	NP_065202.2	295	5	16.10
THEG	XP_024307300.1	139	7	73.60	SURF6	NP_006744.2	630	15	22.62
C2CD4C	XP_011525996.1	375	13	47.05	MED22	NP_598395.1	319	12	35.73
SHC2	XP_011526197.1	519	12	29.29	RPL7A	NP_000963.1	1570	6	3.63
ODF3L2	NP_001372526.1	144	14	115.45	SURF1	NP_003163.1	520	4	7.31
MADCAM1	NP_570116.2	501	7	15.62	SURF2	NP_059973.4	174	4	21.84
TPGS1	NP_277048.2	161	15	98.34	SURF4	NP_001267718.1	827	9	10.34
CDC34	NP_004350.1	2168	11	5.07	STKLD1	NP_714921.4	92	1	10.33
GZMM	NP_005308.2	722	12	15.79	REXO4	NP_001266278.1	681	3	4.18
BSG	XP_016882662.1	962	9	8.89	ADAMTS13	XP_016869721.1	371	9	23.04
HCN2	NP_001185.3	829	7	8.02	CACFD1	NP_001229298.1	62	2	30.64
POLRMT	NP_005026.3	767	16	19.82	SLC2A6	NP_001138571.1	2454	2	0.77
FGF22	NP_001287741.1	621	11	16.83	MYMK	NP_001073952.1	136	1	6.98
RNF126	NP_001352947.1	867	18	19.72	ADAMTSL2	XP_011517543.2	194	3	14.69
FSTL3	NP_005851.1	528	15	26.99	FAM163B	XP_016870506.1	808	3	3.53
PRSS57	NP_001295138.2	387	15	36.82	DBH	NP_000778.3	617	2	3.08
PALM	NP_002570.2	277	1	3.43	SARDH	XP_016869856.1	484	6	11.78
MISP	NP_775752.1	271	9	31.55	VAV2	NP_003362.2	852	6	6.69
PTBP1	NP_002810.1	1570	8	4.84	BRD3	XP_016870654.1	1578	3	1.81
PLPPR3	NP_001257295.1	279	15	51.07	WDR5	NP_060058.1	1623	8	4.68
AZU1	NP_001691.1	511	7	13.01	RXRA	NP_002948.1	1295	5	3.67
PRTN3	NP_002768.3	901	9	9.49	COL5A1	NP_000084.3	884	4	4.30
ELANE	NP_001963.1	1405	10	6.76	FCN2	XP_011516694.1	420	1	2.26
CFD	NP_001304264.1	595	5	7.98	FCN1	NP_001994.2	636	1	1.49
MED16	XP_016881609.1	359	15	39.69	OLFM1	NP_055094.1	935	7	7.11
R3HDM4	NP_620129.2	258	16	58.91	C9orf62	NP_775791.1	7	0	0.00
KISS1R	NP_115940.2	526	8	14.45	PPP1R26	XP_016870844.1	130	0	0.00
ARID3A	XP_016881934.1	797	11	13.11	C9orf116	NP_001041730.1	63	0	0.00
WDR18	NP_001359014.1	1261	15	11.30	MRPS2	NP_001358330.1	1173	7	5.67
GRIN3B	NP_619635.1	448	9	19.08	LCN1	XP_011516977.1	270	5	17.59
TMEM259	XP_024307541.1	319	14	41.69	OBP2A	XP_016870145.1	171	5	27.78
CNN2	NP_001290430.1	691	9	12.37	PAEP	XP_011517047.1	392	3	7.27
ABCA7	XP_024307083.1	532	6	10.71	GLT6D1	NP_892019.2	161	10	59.00
ARHGAP45	NP_001245257.1	715	16	21.26	LCN9	XP_016870201.1	155	6	36.77
POLR2E	NP_002686.3	1438	11	7.27	SOHLH1	NP_001095147.2	820	6	6.95
GPX4	NP_001034936.1	911	8	8.34	KCNT1	NP_065873.2	758	9	11.28
SBNO2	NP_055778.2	479	17	33.71	CAMSAP1	NP_056262.3	365	8	20.82
STK11	NP_000446.1	2138	7	3.11	UBAC1	NP_057256.2	505	7	13.17
CBARP	XP_016882044.1	140	13	88.21	NACC2	NP_653254.1	435	0	0.00
ATP5F1D	NP_001678.1	1136	15	12.54	TMEM250	XP_024303480.1	300	0	0.00
MIDN	XP_024307521.1	207	19	87.19	LHX3	NP_835258.1	593	1	1.60
CIRBP	XP_016881726.1	1301	11	8.03	QSOX2	NP_859052.3	249	4	15.26
FAM174C	NP_060384.3	260	19	69.42	GPSM1	NP_001139110.2	763	6	7.47
EFNA2	XP_016881938.1	614	7	10.83	DNLZ	NP_001074318.1	244	3	11.68
PWWP3A	XP_016882890.1	95	0	0.00	CARD9	NP_434701.1	669	11	15.62
NDUFS7	NP_077718.3	854	13	14.46	SNAPC4	XP_016870547.1	1100	12	10.36
GAMT	NP_000147.1	760	14	17.50	ENTR1	NP_001034797.1	266	5	17.86
DAZAP1	NP_061832.2	774	14	17.18	PMPCA	NP_055975.1	810	7	8.21

RPS15	NP_001009.1	1922	11	5.44	INPP5E	NP_001305431.1	605	2	3.14
APC2	XP_005259532.1	1028	5	4.62	SEC16A	XP_011517565.1	549	6	10.38
C19orf25	NP_689695.2	59	18	289.82	C9orf163	NP_689784.1	0	0	#DIV/0!
PCSK4	XP_011526393.1	594	14	22.39	NOTCH1	NP_060087.3	4208	6	1.35
REEP6	NP_001316485.1	826	15	17.25	EGFL7	XP_016870284.1	430	3	6.63
ADAMTSL5	NP_001354126.1	166	13	74.39	AGPAT2	NP_001012745.1	833	3	3.42
PLK5	NP_001230008.1	1568	15	9.09	DIPK1B	NP_689634.2	153	8	49.67
MEX3D	NP_001167589.1	268	15	53.17	LCN10	NP_001355018.1	136	2	13.97
MBD3	NP_001268383.1	555	8	13.69	LCN6	NP_945184.1	102	3	27.94
UQCR11	NP_006821.1	521	11	20.06	LCN8	NP_001332863.1	180	3	15.83
TCF3	XP_011526529.1	1347	0	0.00	LCN15	NP_976222.1	128	3	22.26
ONECUT3	NP_001073957.1	281	9	30.43	TMEM141	NP_116317.1	18	0	0.00
ATP8B3	XP_011526010.1	505	9	16.93	CCDC183	NP_001034463.4	82	1	11.58
REXO1	XP_016882517.1	670	15	21.27	RABL6	NP_001167459.1	658	3	4.33
KLF16	NP_114124.1	543	6	10.50	AJM1	NP_001073951.2	93	0	0.00
ABHD17A	NP_001123583.1	334	8	22.75	PHPT1	NP_001274271.1	385	1	2.47
ADAT3	NP_612431.2	573	4	6.63	MAMDC4	NP_996803.2	750	1	1.27
SCAMP4	NP_524558.1	147	5	32.31	EDF1	NP_001268227.1	864	2	2.20
CSNK1G2	XP_016881786.1	806	7	8.25	TRAF2	NP_066961.2	906	0	0.00
BTBD2	NP_060267.2	327	5	14.53	FBXW5	XP_016870301.1	584	0	0.00
MKNK2	NP_060042.2	468	4	8.12	C8G	NP_000597.2	212	2	8.96
MOB3A	XP_024307128.1	260	0	0.00	LCN12	XP_011516862.1	107	3	26.63
IZUMO4	XP_024307110.1	191	0	0.00	PTGDS	NP_000945.3	978	5	4.86
AP3D1	NP_003929.4	808	2	2.35	LCNL1	NP_997393.3	253	2	7.51
DOT1L	NP_115871.1	1061	7	6.27	PAXX	NP_001316607.1	171	3	16.67
PLEKHJ1	NP_001287765.1	156	3	18.27	CLIC3	XP_016870770.1	358	6	15.92
SF3A2	NP_009096.2	965	7	6.89	ABCA2	NP_997698.1	657	7	10.12
AMH	NP_000470.3	861	7	7.72	FUT7	NP_004470.1	525	4	7.24
JSRP1	XP_016881775.1	141	3	20.21	C9orf139	XP_016870206.1	40	3	71.25
OAZ1	NP_004143.1	426	3	6.69	NPDC1	NP_056207.3	437	6	13.04
PEAK3	NP_940934.1	91	0	0.00	ENTPD2	NP_001237.1	430	3	6.63
LINGO3	NP_001094861.1	537	5	8.84	SAPCD2	NP_848543.2	245	0	0.00
LSM7	NP_057283.1	1091	5	4.35	UAP1L1	NP_997192.2	604	2	3.15
SPPL2B	XP_011526440.1	288	1	3.30	MAN1B1	XP_024303171.1	489	3	5.83
TMPRSS9	XP_011526280.1	241	1	3.94	DPP7	XP_011516902.1	412	4	9.22
TIMM13	NP_036590.1	966	3	2.95	GRIN1	NP_001172019.1	1939	4	1.96
LMNB2	NP_116126.3	1212	4	3.14	LRRC26	NP_001013675.1	457	0	0.00
GADD45B	NP_056490.2	678	1	1.40	TMEM210	NP_001269406.1	0	0	#DIV/0!
GNG7	XP_016882095.1	1295	3	2.20	ANAPC2	NP_037498.1	1023	4	3.71
DIRAS1	XP_011526015.1	1882	1	0.50	SSNA1	NP_003722.2	465	1	2.04
SLC39A3	NP_653165.2	269	4	14.13	TPRN	NP_001121700.2	268	7	24.81
SGTA	NP_003012.1	1376	4	2.76	TMEM203	NP_444273.1	313	2	6.07
THOP1	NP_003240.1	678	2	2.80	NDOR1	NP_001137498.1	685	5	6.93
ZNF554	NP_001096121.1	149	0	0.00	RNF208	NP_112587.2	335	2	5.67
ZNF555	NP_690004.4	153	0	0.00	CYSRT1	NP_945352.4	27	1	35.18
ZNF556	NP_079243.1	189	0	0.00	RNF224	NP_001177157.1	3	2	633.30
ZNF57	NP_775751.1	230	0	0.00	SLC34A3	XP_016869781.1	147	3	19.39
ZNF77	NP_067040.1	283	0	0.00	TUBB4B	NP_006079.1	1765	3	1.61
TLE6	NP_001137458.1	527	4	7.21	FAM166A	XP_006717171.2	117	0	0.00
TLE2	NP_003251.2	636	3	4.48	STPG3	NP_001243628.1	14	0	0.00
TLE5	XP_006722727.1	653	0	0.00	NELFB	NP_056271.3	652	4	5.83
GNA11	NP_002058.2	1484	4	2.56	TOR4A	NP_060193.2	166	0	0.00
GNA15	NP_002059.3	1407	4	2.70	NRARP	NP_001004354.1	1032	1	0.92
S1PR4	NP_003766.1	797	2	2.38	EXD3	NP_060290.3	236	0	0.00
NCLN	NP_064555.2	361	0	0.00	NOXA1	NP_006638.1	479	3	6.26
CELF5	NP_068757.2	683	6	8.35	ENTPD8	XP_011516968.1	430	2	4.91
NFIC	NP_995315.1	529	0	0.00	NSMF	XP_005266119.1	201	3	16.68
SMIM24	NP_001129975.1	79	1	12.02	PNPLA7	NP_689499.4	842	4	5.64
DOHH	NP_112594.1	850	2	2.24	MRPL41	NP_115866.1	514	1	2.46
FZR1	XP_016882352.1	1861	6	3.06	DPH7	NP_001333323.1	153	1	8.87
MFSD12	XP_011525986.1	201	2	9.45	ZMYND19	NP_612471.1	135	3	32.48
C19orf71	NP_001129052.1	12	0	0.00	ARRDC1	NP_689498.1	505	0	0.00
HMG20B	NP_006330.2	674	3	4.23	EHMT1	NP_079033.4	1966	2	1.76
GIPC3	XP_005259549.1	372	7	17.88	CACNA1B	NP_000709.1	1399	1	1.36
TBXA2R	NP_963998.2	506	4	7.51	PLCXD1	XP_024308163.1	193	9	88.60
CACTIN	NP_001074012.1	486	2	3.91	GTPBP6	NP_036359.3	393	11	48.34

PIP5K1C	XP_016882029.1	892	7	7.45	PPP2R3B	NP_037371.2	491	11	35.47
TJP3	NP_001254490.1	376	3	7.58	SHOX	NP_006874.1	737	13	25.78
APBA3	XP_006723013.1	319	4	11.91	CRLF2	XP_011544483.1	319	8	34.03
MRPL54	NP_758455.1	563	5	8.44	CSF2RA	NP_001366092.1	628	14	28.24
RAX2	NP_116142.1	539	7	12.34	IL3RA	NP_002174.1	704	15	25.30
MATK	NP_002369.2	1350	4	2.81	SLC25A6	NP_001627.2	1121	16	15.95
ZFR2	NP_055989.1	108	11	96.75	ASMTL	NP_001166945.1	506	16	33.38
ATCAY	NP_149053.1	1217	11	8.59	P2RY8	XP_006724506.2	542	13	23.98
NMRK2	NP_001276046.1	927	7	7.17	AKAP17A	NP_005079.2	513	13	24.07
DAPK3	NP_001362587.1	880	9	9.72	ASMT	NP_004034.2	382	16	39.79
EEF2	NP_001952.1	2647	5	1.79	DHRSX	NP_660160.2	263	14	50.57
PIAS4	NP_056981.2	905	7	7.35	ZBED1	NP_001164606.1	410	9	20.85
ZBTB7A	XP_005259627.3	612	8	12.42	CD99	NP_002405.1	324	13	38.12
MAP2K2	XP_006722862.1	1645	8	4.62	XG	XP_005274644.1	116	14	114.65
CREB3L3	NP_115996.1	557	1	1.71	GYG2	XP_016885416.1	414	12	27.53
SIRT6	NP_001307988.1	1439	4	2.64	ARSD	XP_005274571.1	324	10	29.32
ANKRD24	XP_011526058.1	959	3	2.97	ARSL	NP_001356008.1	412	15	34.59
EBI3	NP_005746.2	127	1	7.48	ARSH	NP_001011719.1	432	12	26.39
YJU2	NP_060544.2	511	1	1.86	ARSF	XP_011543824.1	304	7	21.87
SHD	NP_001358940.1	166	0	0.00	MXRA5	NP_056234.2	756	7	8.80
TMIGD2	NP_001295161.1	56	0	0.00	PRKX	NP_005035.1	2032	14	6.54
FSD1	NP_077309.1	369	0	0.00	NLGN4X	XP_016885180.1	990	11	10.56
STAP2	NP_001013863.1	205	0	0.00	VCX3A	NP_057463.2	242	9	35.33
MPND	NP_001287791.1	867	2	2.19	PUDP	NP_001171607.1	638	15	22.33
SH3GL1	NP_001186872.1	637	0	0.00	STS	NP_001307679.1	848	15	16.80
CHAF1A	XP_011525907.1	920	1	1.03	VCX	NP_038480.2	210	7	31.67
UBXN6	XP_016882814.1	310	0	0.00	PNPLA4	NP_001135861.1	298	10	31.88
HDGFL2	NP_001001520.1	1164	0	0.00	VCX2	NP_057462.2	171	7	38.89
PLIN4	NP_001354797.1	393	3	7.25	VCX3B	NP_001001888.3	148	6	38.51
PLIN5	NP_001013728.2	268	2	7.09	ANOS1	NP_000207.2	398	16	38.19
LRG1	NP_443204.1	702	0	0.00	FAM9A	NP_777611.1	33	2	57.57
SEMA6B	NP_115484.2	430	1	2.21	FAM9B	XP_024308113.1	50	2	38.00
TNFAIP8L1	NP_001161414.1	239	0	0.00	TBL1X	NP_001132938.1	1338	10	7.10
MYDGF	NP_061980.1	255	0	0.00	GPR143	NP_000264.2	437	6	13.04
DPP9	NP_001371548.1	255	0	0.00	SHROOM2	NP_001640.1	396	10	23.99
FEM1A	NP_061178.1	1010	0	0.00	CLDN34	NP_001182010.1	21	0	0.00
TICAM1	NP_001372607.1	467	0	0.00	WWC3	NP_056506.2	621	8	12.24
PLIN3	NP_001157666.1	427	2	4.45	CLCN4	NP_001821.2	637	10	14.91
ARRDC5	XP_016882633.1	452	1	2.10	MID1	NP_150631.1	622	11	16.80
UHRF1	NP_037414.3	1004	2	1.89	HCCS	NP_005324.3	1186	10	8.01
KDM4B	XP_016881992.1	797	5	5.96	ARHGAP6	NP_038286.2	741	9	11.54
PTPRS	XP_005259667.1	980	6	5.82	AMELX	NP_872621.1	481	8	15.80
ZNRF4	NP_859061.3	203	4	18.72	MSL3	NP_523353.2	747	9	11.45
SAFB2	NP_055464.1	540	8	14.07	FRMPD4	NP_001355324.1	1122	10	8.47
SAFB	NP_001188267.1	859	6	6.64	PRPS2	NP_001034180.1	1109	8	6.85
MICOS13	NP_991330.1	257	1	3.70	TLR7	NP_057646.1	2153	5	2.21
HSD11B1L	NP_001254797.1	346	4	10.98	TLR8	NP_619542.1	2003	2	0.95
RPL36	NP_056229.2	1120	5	4.24	TMSB4X	NP_066932.1	540	4	7.04
LONP1	NP_001263408.1	947	5	5.02	FAM9C	XP_024308115.1	21	1	45.24
CATSPERD	XP_011526187.1	86	1	11.05	ATXN3L	NP_001129467.1	332	0	0.00
PRR22	NP_001127788.1	67	3	42.54	EGFL6	NP_001161362.1	350	5	13.57
DUS3L	NP_001155091.1	596	3	4.78	TCEANC	XP_016884805.1	404	0	0.00
NRTN	NP_004549.1	257	2	7.39	RAB9A	NP_001182257.1	846	11	12.35
FUT6	NP_001368887.1	252	2	7.54	TRAPPC2	NP_001122307.2	389	9	21.98
FUT3	NP_001091108.3	389	2	4.88	OFD1	NP_003602.1	597	6	9.55
FUT5	NP_002025.2	152	4	25.00	GPM6B	NP_001305658.1	837	5	5.67
NDUFA11	NP_001180304.1	600	4	6.33	GEMIN8	XP_005274612.1	332	9	25.75
VMAC	NP_001017921.1	80	0	0.00	GLRA2	NP_001112357.1	865	7	7.69
CAPS	NP_004049.3	496	1	1.92	FANCB	XP_011543772.1	552	9	15.49
RANBP3	NP_001287794.1	357	2	5.32	MOSPD2	NP_001317170.1	527	10	18.03
RFX2	NP_602309.1	777	5	6.11	ASB9	XP_016884773.1	1192	4	3.19
ACSBG2	XP_016882822.1	681	4	5.58	ASB11	NP_001188512.1	1403	13	8.80
MLLT1	NP_005925.2	1116	6	5.11	PIGA	NP_065206.3	858	12	13.29
ACER1	NP_597999.1	304	1	3.12	VEGFD	NP_004460.1	500	1	1.90
CLPP	NP_006003.1	1256	4	3.03	PIR	NP_003653.1	377	3	7.56
ALKBH7	NP_115682.1	204	10	46.57	BMX	NP_975010.1	957	5	4.96

PSPN	NP_004149.1	159	4	23.90	ACE2	NP_001373188.1	689	4	5.51
GTF2F1	NP_002087.2	1046	7	6.36	CLTRN	XP_016885170.1	253	1	3.75
KHSRP	NP_001353229.1	1087	6	5.24	CA5B	NP_009151.1	403	11	25.93
SLC25A41	XP_011526228.1	147	10	64.62	ZRSR2	XP_016885370.1	521	11	20.06
SLC25A23	XP_016882776.1	347	5	13.69	AP1S2	NP_003907.3	693	6	8.22
CRB3	XP_016882960.1	194	0	0.00	GRPR	NP_005305.1	608	11	17.19
DENND1C	NP_079174.2	325	8	23.38	MAGEB17	NP_001264236.1	47	1	20.21
TUBB4A	NP_001276056.1	2125	5	2.24	CTPS2	XP_011543847.1	1387	11	7.53
TNFSF9	NP_003802.1	407	3	7.00	S100G	XP_016885330.1	250	5	19.00
CD70	NP_001317261.1	498	2	3.82	SYAP1	NP_116185.2	237	10	40.08
TNFSF14	XP_016882907.1	446	4	8.52	TXLNG	NP_060830.2	394	9	21.70
C3	NP_000055.2	1404	2	1.35	RBBP7	NP_002884.1	2354	8	3.23
GPR108	XP_016882502.1	282	1	3.37	REPS2	XP_024308246.1	371	10	25.61
TRIP10	XP_006723003.1	784	3	3.64	NHS	NP_001278796.1	335	17	48.21
SH2D3A	XP_006722665.1	273	0	0.00	SCML1	XP_005274635.1	224	3	12.72
VAV1	XP_005259699.1	1447	7	4.60	RAI2	NP_001166203.2	152	12	75.00
ADGRE1	XP_011526096.1	700	6	8.14	BEND2	XP_016884755.1	77	4	49.35
MBD3L5	NP_001129979.1	183	3	15.57	SCML2	XP_016884708.1	542	12	21.03
MBD3L4	NP_001157891.1	136	3	20.95	CDKL5	NP_003150.1	624	11	16.75
MBD3L2	NP_653215.2	247	2	7.69	RS1	NP_000321.1	355	6	16.06
MBD3L3	NP_001157897.1	171	3	16.67	PPEF1	NP_001364915.1	1789	9	4.78
ZNF557	NP_077317.2	195	6	29.23	PHKA2	XP_011543839.1	555	8	13.69
INSR	XP_011526291.2	2254	5	2.11	ADGRG2	NP_001171764.1	483	2	3.93
ARHGEF18	XP_005272521.1	611	4	6.22	PDHA1	NP_001166925.1	1323	8	5.74
PEX11G	XP_011526734.1	318	1	2.99	MAP3K15	NP_001001671.3	544	1	1.75
TEX45	NP_940936.2	43	0	0.00	SH3KBP1	NP_001340819.1	804	4	4.73
ZNF358	NP_060553.4	268	1	3.54	BCLAF3	XP_016884879.1	215	13	57.44
MCOLN1	NP_065394.1	495	1	1.92	MAP7D2	NP_001161938.1	360	7	18.47
PNPLA6	NP_001159583.1	1005	4	3.78	EIF1AX	NP_001403.1	1404	7	4.74
CAMSAP3	NP_001073898.1	346	4	10.98	RPS6KA3	XP_011543857.1	1115	10	8.52
XAB2	NP_064581.2	1291	11	8.09	CNKSR2	NP_055742.2	767	6	7.43
PET100	NP_001164626.1	221	8	34.39	KLHL34	NP_695002.1	285	1	3.33
PCP2	XP_016881738.1	487	8	15.60	SMPX	NP_055147.1	514	4	7.39
STXBP2	NP_001258963.1	846	12	13.47	MBTPS2	NP_056968.1	628	14	21.18
RETN	NP_001372655.1	934	10	10.17	YY2	NP_996806.2	451	1	2.11
MCEMP1	NP_777578.2	470	8	16.17	SMS	NP_004586.2	914	9	9.35
TRAPPC5	NP_777554.1	378	10	25.13	PHEX	NP_000435.3	715	7	9.30
FCER2	NP_001993.2	649	8	11.71	CBLL2	NP_689790.1	384	3	7.42
CLEC4G	NP_001231785.1	359	10	26.46	DDX53	NP_874358.2	1407	1	0.68
CD209	NP_001138365.1	860	4	4.42	PTCHD1	NP_775766.2	371	7	17.92
CLEC4M	NP_055072.3	370	4	10.27	PRDX4	NP_006397.1	1271	13	9.72
EVI5L	XP_016881728.1	234	1	4.06	ACOT9	NP_001028755.2	457	14	29.10
PRR36	NP_001177396.1	118	1	8.05	SAT1	NP_002961.1	349	8	21.78
LRRC8E	NP_001255213.1	427	1	2.22	APOO	XP_024308215.1	519	11	20.13
MAP2K7	XP_006722863.1	1972	2	0.96	CXorf58	NP_689974.2	200	9	42.75
TGFBR3L	XP_011525912.1	40	1	23.75	KLHL15	NP_085127.2	332	12	34.34
SNAPC2	NP_003074.1	128	1	7.42	EIF2S3	NP_001406.1	1305	9	6.55
CTXN1	NP_996664.1	360	1	2.64	ZFX	XP_016885277.1	793	8	9.58
TIMM44	NP_006342.2	759	4	5.01	PDK3	NP_001135858.1	940	13	13.14
ELAVL1	NP_001410.2	1800	2	1.06	PCYT1B	NP_001156737.1	732	11	14.28
CCL25	XP_016882608.1	551	1	1.72	POLA1	NP_001317289.1	1548	14	8.59
FBN3	XP_016882863.1	578	7	11.50	ARX	NP_620689.1	697	3	4.09
CERS4	XP_016882792.1	434	6	13.13	MAGEB18	NP_775970.2	110	0	0.00
CD320	NP_001159367.1	231	6	24.67	MAGEB6	NP_775794.2	216	5	21.99
NDUFA7	NP_004992.2	516	7	12.89	MAGEB5	NP_001258681.1	124	0	0.00
RPS28	NP_001022.1	1268	8	5.99	DCAF8L2	NP_001340379.1	102	1	9.31
KANK3	XP_016882052.1	853	7	7.80	MAGEB10	NP_872312.2	95	1	10.00
ANGPTL4	XP_005272541.1	1077	4	3.53	DCAF8L1	NP_001017930.1	99	1	9.60
RAB11B	NP_004209.2	1881	8	4.04	IL1RAPL1	XP_016884729.1	697	15	20.44
MARCHF2	NP_001356706.1	423	4	8.98	MAGEB2	NP_002355.2	209	5	22.73
HNRNPM	NP_005959.2	1309	2	1.45	MAGEB3	NP_002356.2	91	6	62.63
PRAM1	NP_115528.4	441	1	2.15	MAGEB4	NP_002358.1	155	6	36.77
ZNF414	NP_001139647.1	232	4	16.38	MAGEB1	NP_002354.2	195	6	29.23
MYO1F	NP_001335284.1	1374	5	3.46	NR0B1	NP_000466.2	789	8	9.63
ADAMTS10	XP_016882827.1	282	4	13.47	TASL	NP_079435.1	71	9	120.42
ACTL9	NP_848620.3	1030	7	6.46	GK	XP_011543793.1	1271	6	4.48

OR2Z1	NP_001004699.1	73	4	52.05	TAB3	XP_005274540.2	554	9	15.43
ZNF558	XP_024307154.1	231	4	16.45	FTHL17	NP_114100.1	285	7	23.33
MBD3L1	NP_660209.2	221	4	17.19	DMD	XP_006724532.1	1819	12	6.27
MUC16	XP_016882989.1	450	4	8.44	FAM47A	NP_981953.2	62	6	91.93
OR1M1	NP_001004456.1	99	0	0.00	TMEM47	NP_113630.1	495	9	17.27
OR7G2	NP_001005193.1	59	0	0.00	FAM47B	NP_689844.2	79	7	84.17
OR7G1	NP_001005192.2	102	1	9.31	MAGEB16	NP_001093391.1	242	9	35.33
OR7G3	NP_001001958.1	89	0	0.00	CFAP47	NP_001291477.1	276	12	41.30
ZNF317	XP_024307394.1	143	0	0.00	FAM47C	NP_001013758.1	48	1	19.79
OR7D2	NP_001373041.1	54	0	0.00	PRRG1	NP_001135867.1	106	7	62.73
OR7D4	NP_001005191.1	113	0	0.00	LANCL3	NP_001163802.1	113	9	75.66
OR7E24	NP_001373037.1	138	0	0.00	XK	NP_066569.1	365	8	20.82
ZNF699	NP_940937.1	177	1	5.37	CYBB	NP_000388.2	1505	9	5.68
ZNF559	NP_001189335.1	175	0	0.00	DYNLT3	NP_006511.1	417	13	29.61
ZNF177	NP_001371588.1	172	0	0.00	H2AP	NP_036406.1	40	0	0.00
ZNF266	NP_001357303.1	199	0	0.00	SYTL5	XP_016885461.1	446	9	19.17
ZNF560	XP_016881816.1	271	0	0.00	SRPX	NP_001164221.1	391	4	9.72
ZNF426	NP_077011.1	221	1	4.30	RPGR	NP_001354178.1	1466	14	9.07
ZNF121	XP_016882728.1	189	0	0.00	OTC	NP_000522.3	991	8	7.67
ZNF561	XP_006723001.1	135	1	7.04	TSPAN7	NP_004606.2	620	3	4.60
ZNF562	NP_060126.2	159	1	5.97	MID1IP1	NP_067065.1	204	4	18.63
ZNF846	NP_001340729.1	175	1	5.43	BCOR	NP_001116856.1	1251	6	4.56
FBXL12	XP_016882401.1	868	7	7.66	ATP6AP2	NP_005756.2	593	6	9.61
UBL5	NP_001041706.1	729	5	6.52	CXorf38	XP_016884791.1	177	8	42.94
PIN1	NP_006212.1	1130	2	1.68	MED14	NP_004220.2	1019	9	8.39
OLFM2	NP_477512.1	564	4	6.74	USP9X	XP_005272732.1	1367	14	9.73
COL5A3	XP_011526344.1	492	3	5.79	DDX3X	NP_001347.3	2288	10	4.15
RDH8	NP_056540.3	289	1	3.29	NYX	NP_072089.1	742	12	15.36
SHFL	NP_060851.2	95	1	10.00	CASK	XP_006724629.1	1500	15	9.50
ANGPTL6	XP_011526650.1	399	2	4.76	GPR34	NP_001091048.1	318	5	14.94
PPAN-P2RY11	NP_001035754.1	817	3	3.49	GPR82	NP_543007.1	177	5	26.83
PPAN	NP_064615.3	953	3	2.99	MAOA	NP_000231.1	1122	8	6.77
P2RY11	NP_002557.2	347	3	8.21	MAOB	NP_000889.3	1106	6	5.15
EIF3G	NP_003746.2	1112	7	5.98	NDP	NP_000257.1	401	4	9.48
DNMT1	NP_001370.1	2317	4	1.64	EFHC2	NP_079460.2	421	17	38.36
S1PR2	NP_004221.3	616	4	6.17	FUNDC1	NP_776155.1	375	11	27.87
MRPL4	NP_057040.2	1258	9	6.80	DUSP21	NP_071359.3	496	3	5.75
ICAM1	NP_000192.2	2005	8	3.79	KDM6A	XP_011542260.1	3882	13	3.18
ICAM4	NP_001034221.1	120	5	39.58	DIPK2B	XP_006724622.1	180	4	21.11
ICAM5	NP_003250.3	388	4	9.79	KRBOX4	NP_001123370.1	306	8	24.84
ZGLP1	NP_001096637.1	581	3	4.91	ZNF674	NP_001139763.1	236	4	16.10
FDX2	NP_001026904.2	394	2	4.82	CHST7	NP_063939.2	211	8	36.02
RAVER1	NP_001353103.1	954	8	7.97	SLC9A7	NP_115980.1	865	16	17.57
ICAM3	NP_001307534.1	540	6	10.56	RP2	NP_008846.2	323	8	23.53
TYK2	NP_001372134.1	1496	5	3.17	JADE3	NP_001070913.1	559	16	27.19
CDC37	NP_008996.1	862	2	2.20	RGN	XP_024308245.1	704	6	8.10
PDE4A	NP_001230050.1	803	4	4.73	NDUFB11	NP_001129470.1	715	5	6.64
KEAP1	NP_036421.2	1244	3	2.29	RBM10	XP_024308225.1	1064	13	11.61
S1PR5	NP_110387.1	425	0	0.00	UBA1	XP_016885267.1	1729	13	7.14
ATG4D	NP_116274.3	745	3	3.83	CDK16	XP_011542223.1	1978	12	5.76
KRI1	NP_075384.4	607	1	1.56	USP11	NP_001358001.1	825	7	8.06
CDKN2D	NP_524145.1	1169	2	1.63	ZNF157	NP_003437.2	325	12	35.08
AP1M2	NP_005489.2	669	2	2.84	ZNF41	NP_001311086.1	434	11	24.08
SLC44A2	NP_001138528.1	481	7	13.82	ARAF	NP_001243125.1	1520	14	8.75
ILF3	XP_016882252.1	1155	6	4.93	SYN1	NP_008881.2	1714	8	4.43
QTRT1	NP_112486.1	698	4	5.44	TIMP1	NP_003245.1	1489	8	5.10
DNM2	NP_001005360.1	1951	8	3.90	CFP	NP_001138724.1	1002	3	2.84
TMED1	NP_006849.1	164	1	5.79	ELK1	NP_005220.2	318	5	14.94
C19orf38	NP_001129954.1	16	1	59.37	UXT	NP_705582.1	711	5	6.68
CARM1	NP_954592.1	1416	8	5.37	ZNF81	NP_001365083.1	246	4	15.45
YIPF2	XP_024307468.1	202	0	0.00	ZNF182	NP_001007089.1	228	5	20.83
TIMM29	NP_612367.1	169	1	5.62	SPACA5	XP_016885001.1	149	6	38.25
SMARCA4	XP_024307426.1	2873	4	1.32	ZNF630	NP_001177184.1	190	4	20.00
LDLR	NP_000518.1	1835	12	6.21	SPACA5B	NP_001073369.1	146	5	32.53
SPC24	NP_001303960.1	488	1	1.95	SSX5	XP_016885247.1	112	1	8.48
KANK2	NP_001316380.1	1061	10	8.95	SSX1	NP_005626.1	206	7	32.28

DOCK6	NP_065863.2	424	9	20.16	SSX3	NP_066294.1	137	2	13.87
ANGPTL8	NP_061157.3	111	8	68.46	SSX4	NP_783856.1	171	1	5.56
TSPAN16	NP_036598.1	281	1	3.38	SSX4B	NP_001035702.1	166	1	5.72
RAB3D	NP_004274.1	536	9	15.95	SLC38A5	XP_005272751.2	517	2	3.67
TMEM205	XP_016882258.1	178	8	42.69	FTSJ1	XP_024308127.1	866	5	5.48
CCDC159	NP_001073972.2	28	8	271.41	PORCN	XP_024308193.1	305	2	6.23
PLPPR2	XP_016882636.1	290	9	29.48	EBP	NP_006570.1	276	1	3.44
SWSAP1	NP_787067.3	140	9	61.07	TBC1D25	NP_001335192.1	545	12	20.92
EPOR	NP_000112.1	674	1	1.41	RBM3	NP_006734.1	1493	2	1.27
RGL3	NP_001030300.3	286	0	0.00	WDR13	NP_001334146.1	540	7	12.31
CCDC151	NP_001289382.1	108	0	0.00	WAS	NP_000368.1	1617	4	2.35
PRKCSH	NP_001276032.1	533	1	1.78	SUV39H1	NP_001269095.1	2166	4	1.75
ELAVL3	XP_024307181.1	1573	0	0.00	GLOD5	XP_011542214.1	14	2	135.71
ZNF653	NP_620138.2	388	1	2.45	GATA1	XP_011542199.1	1999	8	3.80
ECSIT	NP_001135937.1	404	1	2.35	HDAC6	NP_001308154.1	2648	6	2.15
CNN1	NP_001290.2	674	0	0.00	ERAS	NP_853510.1	1395	2	1.36
ELOF1	XP_016882846.1	199	0	0.00	PCSK1N	NP_037403.1	464	0	0.00
ACP5	NP_001308952.1	1045	0	0.00	TIMM17B	NP_005825.1	544	3	5.24
ZNF627	NP_660338.1	171	0	0.00	PQBP1	NP_005701.1	599	2	3.17
ZNF823	NP_001073962.1	175	0	0.00	SLC35A2	NP_001269576.1	618	1	1.54
ZNF441	NP_689568.2	129	0	0.00	PIM2	NP_006866.2	1333	4	2.85
ZNF491	NP_689569.2	133	0	0.00	OTUD5	NP_001129629.1	402	1	2.36
ZNF440	XP_005259788.1	160	0	0.00	KCND1	NP_004970.3	686	0	0.00
ZNF439	NP_001335647.1	138	0	0.00	GRIPAP1	NP_064522.4	336	4	11.31
ZNF69	XP_016882720.1	202	0	0.00	TFE3	NP_006512.2	666	3	4.28
ZNF700	NP_001258777.1	133	0	0.00	CCDC120	NP_001156793.2	201	4	18.90
ZNF763	NP_001012771.1	627	0	0.00	PRAF2	NP_009144.1	227	7	29.29
ZNF433	NP_001295277.1	154	0	0.00	WDR45	NP_001025067.1	342	3	8.33
ZNF878	NP_001073873.2	128	0	0.00	GPKOW	XP_016884904.1	453	1	2.10
ZNF844	NP_001129973.1	136	0	0.00	MAGIX	NP_079135.3	464	5	10.24
ZNF20	NP_001190179.1	162	0	0.00	PLP2	NP_002659.1	276	1	3.44
ZNF625	NP_660276.2	187	0	0.00	PRICKLE3	NP_006141.2	600	2	3.17
ZNF136	NP_003428.1	141	0	0.00	SYP	NP_003170.1	1899	3	1.50
ZNF44	NP_057348.3	638	0	0.00	CACNA1F	NP_001243718.1	798	7	8.33
ZNF563	XP_005259807.1	133	0	0.00	CCDC22	NP_054727.1	420	2	4.52
ZNF442	XP_016882805.1	150	0	0.00	FOXP3	XP_016885056.1	1988	5	2.39
ZNF799	NP_001074290.1	144	0	0.00	PPP1R3F	XP_016885423.1	261	4	14.56
ZNF443	NP_005806.3	208	1	4.57	GAGE10	NP_001091883.3	36	1	26.39
ZNF709	NP_689814.1	141	0	0.00	GAGE12J	NP_001091876.2	21	0	0.00
ZNF564	NP_659413.1	115	0	0.00	GAGE13	NP_001091882.2	97	1	9.79
ZNF490	NP_065765.1	276	0	0.00	GAGE12B	NP_001120817.2	55	1	17.27
ZNF791	NP_699189.2	233	0	0.00	GAGE12C	NP_001091878.1	55	1	17.27
MAN2B1	NP_001166969.1	505	2	3.76	GAGE12D	NP_001120671.1	116	1	8.19
WDR83OS	XP_016882353.1	245	0	0.00	GAGE12E	NP_001091888.1	115	1	8.26
WDR83	NP_115708.1	418	1	2.27	GAGE12F	NP_001091875.1	199	3	14.32
DHPS	NP_001356620.1	570	4	6.67	GAGE12G	NP_001091879.1	149	2	12.75
FBXW9	NP_115677.2	559	1	1.70	GAGE12H	NP_001091880.1	43	2	44.18
TNPO2	NP_001369172.1	765	4	4.97	GAGE2A	NP_001120684.1	134	2	14.18
TRIR	NP_001316667.1	329	0	0.00	GAGE1	NP_001035753.1	177	3	16.10
GET3	NP_001358417.1	732	1	1.30	PAGE1	NP_003776.2	81	8	93.82
BEST2	NP_060152.2	329	0	0.00	PAGE4	NP_008934.1	141	6	40.42
HOOK2	NP_001093646.1	323	3	8.82	USP27X	NP_001138545.1	530	0	0.00
JUNB	NP_002220.1	1025	1	0.93	CLCN5	XP_016884746.1	516	1	1.84
PRDX2	NP_005800.3	1526	6	3.74	AKAP4	NP_003877.2	416	3	6.85
RNASEH2A	NP_006388.2	1085	6	5.25	CCNB3	XP_016885402.1	1499	3	1.90
RTBDN	NP_113617.1	95	1	10.00	DGKK	XP_016884757.1	238	2	7.98
MAST1	NP_055790.1	2374	2	0.80	SHROOM4	XP_016885172.1	172	6	33.14
DNASE2	NP_001366.1	261	7	25.48	BMP15	NP_005439.2	739	0	0.00
KLF1	NP_006554.1	753	7	8.83	NUDT10	NP_694853.1	534	1	1.78
GCDH	NP_039663.1	438	5	10.84	EZHIP	NP_981952.1	41	0	0.00
SYCE2	XP_011526184.1	211	7	31.51	NUDT11	NP_060629.2	652	4	5.83
FARSA	NP_004452.1	849	9	10.07	GSPT2	NP_060564.2	1249	5	3.80
CALR	NP_004334.1	1866	3	1.53	MAGED1	NP_001005333.1	369	4	10.30
RAD23A	NP_005044.1	2250	6	2.53	MAGED4B	NP_110428.2	261	6	21.84
GADD45GIP1	NP_443082.2	452	7	14.71	MAGED4	NP_001258990.1	242	7	27.48
DAND5	NP_689867.1	264	3	10.79	XAGE2	NP_570133.1	117	0	0.00

NFIX	NP_001352831.1	388	6	14.69	XAGE1A	XP_016885237.1	176	4	21.59
LYL1	NP_005574.2	398	3	7.16	XAGE1B	NP_001091073.2	184	4	20.65
TRMT1	NP_001338689.1	772	10	12.31	SSX7	NP_775494.1	76	4	50.00
NACC1	NP_443108.1	766	5	6.20	SSX2	NP_783629.1	327	6	17.43
STX10	NP_001258540.1	600	1	1.58	SSX2B	NP_001265631.1	15	0	0.00
IER2	NP_004898.2	267	4	14.23	SPANXN5	NP_001009616.1	35	0	0.00
CACNA1A	NP_001120694.1	1563	1	0.61	XAGE5	XP_016884809.1	94	2	20.21
CCDC130	NP_001307490.1	513	9	16.67	XAGE3	NP_570132.1	109	1	8.72
MRI1	NP_001026897.1	482	8	15.77	FAM156B	NP_001308112.1	68	5	69.85
C19orf53	NP_054766.1	370	14	35.94	FAM156A	XP_016884946.1	68	4	55.88
ZSWIM4	XP_016882642.1	166	12	68.67	GPR173	XP_011529100.1	304	5	15.62
NANOS3	NP_001092092.1	533	8	14.26	TSPYL2	XP_016885216.1	728	10	13.05
C19orf57	NP_001332777.1	73	12	156.16	KDM5C	NP_001340911.1	2313	8	3.29
CC2D1A	XP_024307330.1	489	12	23.31	IQSEC2	XP_006724645.1	671	12	16.99
PODNL1	XP_011526610.1	480	15	29.69	SMC1A	NP_006297.2	1687	7	3.94
DCAF15	NP_612362.2	251	17	64.34	RIBC1	XP_005262045.1	157	4	24.20
RFX1	NP_002909.4	1026	10	9.26	HSD17B10	NP_001032900.1	707	6	8.06
RLN3	NP_543140.1	459	14	28.97	HUWE1	XP_005262022.1	1854	16	8.20
IL27RA	NP_004834.1	331	10	28.70	PHF8	XP_005262057.1	730	8	10.41
PALM3	XP_011526267.1	169	16	89.94	FAM120C	NP_060318.4	173	6	32.95
MISP3	NP_001278220.1	70	2	27.14	WNK3	NP_001002838.1	251	4	15.14
C19orf67	XP_011526486.1	23	16	660.83	TSR2	NP_477511.1	695	4	5.47
SAMD1	NP_612361.1	644	16	23.60	FGD1	NP_004454.2	683	7	9.74
PRKACA	XP_016882437.1	2859	5	1.66	GNL3L	NP_001171748.1	1005	4	3.78
ASF1B	NP_060624.1	1284	10	7.40	ITIH6	NP_940912.1	199	2	9.55
ADGRL1	NP_055736.2	643	7	10.34	MAGED2	NP_055414.2	416	3	6.85
ADGRE5	XP_011526753.1	608	8	12.50	TRO	XP_016885258.1	260	3	10.96
DDX39A	NP_005795.2	2031	10	4.68	PFKFB1	NP_001258734.1	684	3	4.17
PKN1	NP_002732.3	1239	5	3.83	APEX2	NP_055296.2	739	4	5.14
PTGER1	NP_000946.2	508	2	3.74	ALAS2	NP_001033057.1	930	2	2.04
GIPC1	XP_016881636.1	664	7	10.01	PAGE2B	XP_011529087.1	75	1	12.67
DNAJB1	NP_006136.1	1617	6	3.52	PAGE2	XP_016884842.1	54	0	0.00
TECR	XP_024307560.1	538	4	7.06	FAM104B	NP_612371.2	83	0	0.00
NDUFB7	NP_004137.2	758	4	5.01	MTRNR2L10	NP_001177637.1	12	1	79.16
CLEC17A	NP_001191047.1	218	0	0.00	PAGE5	NP_569734.2	151	1	6.29
ADGRE3	XP_011526676.1	475	5	10.00	PAGE3	XP_016884771.1	34	0	0.00
ZNF333	NP_001287841.1	212	0	0.00	MAGEH1	NP_054780.2	227	3	12.55
ADGRE2	NP_001257981.1	480	1	1.98	USP51	XP_016884788.1	375	2	5.07
OR7C1	NP_001357414.1	100	0	0.00	FOXR2	NP_940853.1	518	4	7.34
OR7A5	NP_001357409.1	112	1	8.48	RRAGB	NP_057740.2	535	4	7.10
OR7A10	NP_001005190.1	92	0	0.00	KLF8	NP_001311033.1	512	4	7.42
OR7A17	NP_112163.1	48	0	0.00	UBQLN2	NP_038472.2	2015	6	2.83
OR7C2	NP_036509.1	114	0	0.00	SPIN3	XP_016884804.1	427	9	20.02
SLC1A6	XP_016882641.1	497	3	5.73	SPIN2B	XP_005262065.3	194	1	4.90
CCDC105	NP_775753.2	68	7	97.79	SPIN2A	XP_016885088.1	192	1	4.95
CASP14	NP_036246.1	582	6	9.79	FAAH2	NP_777572.2	447	0	0.00
OR1I1	NP_001004713.1	165	1	5.76	ZXDB	NP_009088.1	231	0	0.00
SYDE1	NP_149014.3	445	11	23.48	ZXDA	NP_009087.1	248	0	0.00
ILVBL	NP_006835.2	547	8	13.89	SPIN4	NP_001012986.2	174	4	21.84
NOTCH3	XP_005259981.1	1472	5	3.23	ARHGEF9	NP_001166950.1	695	6	8.20
EPHX3	NP_079070.1	374	9	22.86	AMER1	NP_689637.3	434	7	15.32
BRD4	NP_001366220.1	1989	7	3.34	ASB12	NP_569059.3	1266	7	5.25
AKAP8	NP_005849.1	298	8	25.50	MTMR8	NP_060147.2	475	11	22.00
AKAP8L	NP_001278407.1	220	3	12.95	ZC4H2	NP_001171504.1	304	7	21.87
WIZ	NP_067064.2	460	10	20.65	ZC3H12B	XP_016884967.1	229	2	8.30
RASAL3	XP_011526488.1	763	2	2.49	LAS1L	NP_001164121.1	471	5	10.08
PGLYRP2	NP_443122.3	348	3	8.19	MSN	XP_005262326.1	1276	3	2.23
CYP4F22	NP_775754.2	335	3	8.51	VSIG4	XP_016884740.1	593	1	1.60
CYP4F8	XP_024307108.1	376	3	7.58	HEPH	NP_620074.3	564	4	6.74
CYP4F3	NP_001356625.1	406	2	4.68	EDA2R	XP_011529308.1	634	9	13.49
CYP4F12	NP_076433.3	384	2	4.95	AR	NP_000035.2	2994	7	2.22
OR10H2	NP_039227.1	67	0	0.00	OPHN1	XP_006724716.1	683	11	15.30
OR10H3	NP_039226.1	108	0	0.00	YIPF6	XP_011529225.1	514	11	20.33
OR10H5	NP_001004466.1	70	0	0.00	STARD8	XP_005262371.1	334	9	25.60
OR10H1	NP_039228.1	45	0	0.00	EFNB1	NP_004420.1	957	6	5.96
CYP4F2	NP_001073.3	444	2	4.28	PJA1	NP_071763.2	699	6	8.15

CYP4F11	NP_067010.3	366	0	0.00	FAM155B	NP_056501.2	255	6	22.35
OR10H4	NP_001004465.1	136	0	0.00	EDA	NP_001390.1	765	7	8.69
TPM4	NP_001138632.1	1225	2	1.55	AWAT2	NP_001002254.1	243	4	15.64
RAB8A	NP_005361.2	1265	2	1.50	OTUD6A	NP_997203.1	305	10	31.15
HSH2D	NP_116244.1	297	0	0.00	IGBP1	XP_016884978.1	1338	11	7.81
CIB3	NP_001287851.1	922	3	3.09	DGAT2L6	NP_940914.1	169	8	44.97
FAM32A	XP_011526200.1	82	0	0.00	AWAT1	XP_016884784.1	265	3	10.75
AP1M1	NP_001123996.1	721	1	1.32	P2RY4	NP_002556.1	485	5	9.79
KLF2	NP_057354.1	1154	1	0.82	ARR3	XP_016885007.1	794	7	8.37
EPS15L1	XP_016882579.1	955	3	2.98	RAB41	NP_001350736.1	594	1	1.60
CALR3	NP_659483.2	432	1	2.20	PDZD11	XP_016885057.1	635	13	19.45
C19orf44	NP_115583.1	17	0	0.00	KIF4A	NP_036442.3	1367	6	4.17
CHERP	NP_006378.3	599	3	4.76	GDPD2	NP_001164663.1	350	10	27.14
SLC35E1	NP_079157.3	209	3	13.64	DLG3	XP_006724688.1	1366	12	8.35
MED26	NP_004822.2	705	0	0.00	TEX11	XP_016885138.1	365	3	7.81
SMIM7	NP_077009.2	26	1	36.54	SLC7A3	NP_116192.4	544	4	6.98
TMEM38A	NP_076979.1	367	1	2.59	SNX12	NP_001243117.1	675	9	12.67
NWD1	XP_011526231.1	1019	1	0.93	FOXO4	NP_005929.2	1057	5	4.49
SIN3B	NP_056075.1	1834	6	3.11	CXorf65	XP_005262301.1	13	0	0.00
F2RL3	NP_003941.2	472	1	2.01	IL2RG	NP_000197.1	1134	4	3.35
CPAMD8	XP_011526219.1	235	1	4.04	MED12	NP_005111.2	1212	12	9.41
HAUS8	NP_001011699.1	297	1	3.20	NLGN3	XP_006724725.2	1017	4	3.74
MYO9B	NP_004136.2	1578	7	4.21	GJB1	NP_001091111.1	566	1	1.68
USE1	NP_060937.2	398	1	2.39	ZMYM3	NP_001164633.1	609	8	12.48
OCEL1	XP_006722962.1	73	2	26.03	NONO	NP_001138880.1	1188	5	4.00
NR2F6	NP_005225.2	380	8	20.00	ITGB1BP2	NP_036410.1	351	2	5.41
USHBP1	NP_001308346.1	203	5	23.40	TAF1	XP_005262352.1	1289	10	7.37
BABAM1	NP_001275685.1	416	9	20.55	OGT	NP_858058.1	2374	10	4.00
ANKLE1	NP_001265373.2	1068	9	8.01	GCNA	XP_016885454.1	245	2	7.75
ABHD8	NP_078803.4	407	8	18.67	CXCR3	XP_016884925.1	1213	2	1.57
MRPL34	NP_076426.1	441	5	10.77	CXorf49	NP_001138612.1	54	0	0.00
DDA1	NP_076955.1	221	2	8.60	CXorf49B	NP_001138611.1	55	0	0.00
ANO8	NP_066010.1	382	3	7.46	NHSL2	NP_001013649.2	138	0	0.00
GTPBP3	NP_001182351.1	675	3	4.22	RTL5	NP_001019626.1	179	0	0.00
PLVAP	NP_112600.1	283	1	3.36	PIN4	NP_001164218.1	1141	3	2.50
BST2	NP_004326.1	679	3	4.20	ERCC6L	NP_060139.2	1483	3	1.92
MVB12A	NP_612410.1	182	2	10.44	RPS4X	NP_000998.1	1451	8	5.24
TMEM221	XP_011525905.1	45	0	0.00	CITED1	NP_001138357.1	258	0	0.00
NXNL1	NP_612463.1	272	4	13.97	HDAC8	XP_016885133.1	2220	4	1.71
SLC27A1	XP_016882270.1	545	3	5.23	PHKA1	NP_001165907.1	430	4	8.84
PGLS	NP_036220.1	755	0	0.00	DMRTC1B	XP_016885265.1	339	2	5.60
NIBAN3	NP_775815.3	156	1	6.09	DMRTC1	XP_016885214.1	114	3	25.00
COLGALT1	NP_078932.2	284	3	10.03	PABPC1L2B	NP_001035971.1	593	2	3.20
UNC13A	XP_011526112.1	1041	1	0.91	PABPC1L2A	NP_001012995.1	1311	4	2.90
MAP1S	XP_016882419.1	482	0	0.00	NAP1L2	NP_068798.1	1070	11	9.77
FCHO1	NP_001371320.1	675	0	0.00	CDX4	NP_005184.1	648	8	11.73
B3GNT3	XP_011525928.1	350	4	10.86	CHIC1	NP_001034929.2	323	11	32.35
INSL3	NP_005534.2	300	1	3.17	ZCCHC13	NP_976048.1	298	9	28.69
JAK3	NP_000206.2	1631	9	5.24	SLC16A2	NP_006508.2	489	10	19.43
RPL18A	NP_000971.1	1161	1	0.82	RLIM	NP_057204.2	860	12	13.26
SLC5A5	XP_011526494.1	459	7	14.49	NEXMIF	NP_001008537.1	300	12	38.00
CCDC124	NP_612451.1	471	5	10.08	ABCB7	NP_001258627.1	806	9	10.61
KCNN1	XP_005259962.1	436	7	15.25	UPRT	NP_659489.1	615	6	9.27
ARRDC2	NP_001020775.1	642	1	1.48	ZDHHC15	NP_001139728.1	439	10	21.64
IL12RB1	XP_011526277.1	671	6	8.49	MAGEE2	NP_619648.1	761	3	3.74
MAST3	XP_011526125.1	458	4	8.30	PBDC1	NP_001287817.1	213	2	8.92
PIK3R2	NP_005018.2	972	4	3.91	MAGEE1	NP_065983.1	375	3	7.60
IFI30	NP_006323.2	621	7	10.71	FGF16	NP_003859.1	593	1	1.60
MPV17L2	NP_116072.2	274	3	10.40	ATRX	XP_005262214.2	2074	8	3.66
RAB3A	NP_002857.1	1217	4	3.12	MAGT1	NP_115497.4	572	4	6.64
PDE4C	NP_001317101.1	511	3	5.58	COX7B	NP_001857.1	349	1	2.72
IQCN	NP_079525.1	101	8	75.24	PGAM4	NP_001025062.1	605	2	3.14
JUND	NP_005345.3	1155	5	4.11	ATP7A	NP_000043.4	1329	4	2.86
LSM4	NP_001239058.1	1312	5	3.62	PGK1	NP_000282.1	2447	7	2.72
PGPEP1	NP_001316400.1	224	7	29.69	TAF9B	NP_057059.2	644	1	1.48
GDF15	XP_024307557.1	1011	3	2.82	CYSLTR1	NP_001269115.1	572	7	11.63

LRRC25	NP_660299.2	344	8	22.09	RTL3	NP_689907.1	113	10	84.07
SSBP4	XP_006722728.1	143	7	46.50	LPAR4	XP_016884926.1	408	11	25.61
ISYNA1	XP_024307304.1	803	7	8.28	P2RY10	NP_001311154.1	623	11	16.77
ELL	NP_006523.1	516	6	11.05	GPR174	NP_115942.1	439	13	28.13
FKBP8	NP_001295302.1	1373	1	0.69	ITM2A	NP_001165052.1	310	9	27.58
KXD1	XP_016882770.1	581	1	1.64	TBX22	NP_001103348.1	518	11	20.17
UBA52	XP_016882687.1	3138	4	1.21	TENT5D	XP_024308112.1	419	12	27.21
REX1BD	NP_001093888.1	123	1	7.72	BRWD3	XP_005262170.1	1049	20	18.11
CRLF1	NP_004741.1	377	3	7.56	HMGN5	NP_110390.1	323	14	41.17
TMEM59L	NP_036241.1	1059	2	1.79	SH3BGRL	NP_003013.1	416	16	36.54
KLHL26	NP_001332913.1	156	1	6.09	POU3F4	NP_000298.3	717	16	21.20
CRTC1	NP_001091952.1	590	1	1.61	CYLC1	NP_066941.1	360	19	50.14
COMP	NP_000086.2	481	0	0.00	RPS6KA6	NP_055311.1	602	15	23.67
UPF1	XP_016882594.1	1661	5	2.86	HDX	XP_016884764.1	495	17	32.62
GDF1	NP_001483.3	803	2	2.37	APOOL	XP_016884761.1	530	16	28.68
COPE	NP_955474.1	840	2	2.26	SATL1	NP_001354787.1	166	13	74.39
DDX49	NP_061943.2	1629	5	2.92	ZNF711	XP_016885292.1	590	18	28.98
HOMER3	NP_001139196.1	322	1	2.95	POF1B	XP_005262260.1	289	14	46.02
SUGP2	NP_001308627.1	215	2	8.84	CHM	NP_000381.1	787	11	13.28
ARMC6	NP_001186125.1	127	1	7.48	DACH2	XP_011529148.1	450	17	35.89
SLC25A42	XP_016882159.1	294	6	19.39	KLHL4	NP_476503.1	455	18	37.58
TMEM161A	NP_001243695.1	145	3	19.65	CPXCR1	NP_149037.5	208	16	73.07
MEF2B	NP_001354211.1	783	8	9.71	TGIF2LX	NP_620410.3	295	8	25.76
BORCS8	NP_001139256.1	125	3	22.80	PABPC5	NP_543022.1	736	7	9.03
RFXANK	NP_001357167.1	1101	13	11.22	PCDH11X	XP_016884906.1	862	10	11.02
NR2C2AP	NP_001287874.1	172	14	77.32	NAP1L3	NP_004529.2	1039	3	2.74
NCAN	NP_004377.2	1194	12	9.55	FAM133A	XP_016884943.1	145	8	52.41
HAPLN4	NP_075378.1	700	13	17.64	DIAPH2	NP_006720.1	841	13	14.68
TM6SF2	NP_001001524.2	219	15	65.07	RPA4	NP_037479.1	632	3	4.51
SUGP1	NP_757386.2	749	14	17.76	PCDH19	NP_065817.2	609	12	18.72
MAU2	XP_005259895.3	571	12	19.96	TNMD	NP_071427.2	275	6	20.73
GATAD2A	XP_016882391.1	681	18	25.11	TSPAN6	NP_001265671.1	417	7	15.95
TSSK6	NP_114426.1	1323	18	12.92	SRPX2	NP_055282.1	541	9	15.80
NDUFA13	NP_057049.5	715	12	15.94	SYTL4	NP_001357098.1	412	5	11.53
YJEFN3	NP_940939.2	326	13	37.88	CSTF2	NP_001293135.1	1315	7	5.06
CILP2	NP_694953.2	299	15	47.66	NOX1	NP_001258744.1	760	5	6.25
PBX4	NP_079521.1	313	11	33.38	XKRX	XP_011529256.1	120	0	0.00
LPAR2	XP_016882957.1	694	5	6.84	ARL13A	NP_001155963.1	365	3	7.81
GMIP	NP_001275927.1	487	13	25.36	TRMT2B	XP_016885351.1	638	2	2.98
ATP13A1	NP_065143.2	889	12	12.82	TMEM35A	NP_067650.1	191	3	14.92
ZNF101	NP_149981.2	207	9	41.30	CENPI	XP_016884872.1	769	6	7.41
ZNF14	NP_066358.2	171	8	44.44	DRP2	NP_001930.2	784	5	6.06
ZNF506	NP_001138876.1	165	0	0.00	TAF7L	NP_079161.3	702	4	5.41
ZNF253	NP_066385.2	159	0	0.00	TIMM8A	NP_004076.1	809	5	5.87
ZNF93	NP_112495.2	198	0	0.00	BTK	NP_001274273.1	2084	5	2.28
ZNF682	NP_149973.1	159	0	0.00	RPL36A-HNRN	NP_001186902.2	495	2	3.84
ZNF90	NP_009069.1	217	0	0.00	RPL36A	NP_066357.3	723	3	3.94
ZNF486	NP_443084.2	156	0	0.00	GLA	NP_000160.1	590	2	3.22
ZNF737	XP_005259754.1	205	0	0.00	HNRNPH2	NP_001027565.1	705	5	6.74
ZNF626	NP_001070143.1	205	0	0.00	ARMCX4	NP_001243084.2	140	4	27.14
ZNF66	NP_001342126.2	168	0	0.00	ARMCX1	NP_057692.1	139	0	0.00
ZNF85	XP_011526565.1	201	0	0.00	ARMCX6	XP_011529278.1	95	4	40.00
ZNF430	NP_079465.3	202	0	0.00	ARMCX3	NP_808816.1	231	0	0.00
ZNF714	NP_872321.2	227	0	0.00	ARMCX2	XP_016885476.1	427	3	6.67
ZNF431	XP_011526052.1	191	0	0.00	NXF5	NP_116564.2	679	3	4.20
ZNF708	XP_016882693.1	198	0	0.00	ZMAT1	XP_005262271.2	202	1	4.70
ZNF738	NP_001342166.1	183	0	0.00	TCEAL2	NP_525129.1	544	13	22.70
ZNF493	NP_001070146.1	191	0	0.00	TCEAL6	NP_001354719.1	898	11	11.64
ZNF429	NP_001001415.2	189	0	0.00	BEX5	NP_001153032.1	276	14	48.19
ZNF100	NP_001338599.1	196	0	0.00	TCP11X1	NP_001355087.1	575	7	11.56
ZNF43	NP_001243582.1	193	0	0.00	NXF2	NP_071336.1	585	7	11.37
ZNF208	NP_001316900.1	276	0	0.00	NXF2B	NP_001093156.1	573	6	9.95
ZNF257	NP_258429.2	175	0	0.00	TCP11X2	NP_001264352.1	192	2	9.90
ZNF676	NP_001001411.2	225	0	0.00	TMSB15A	NP_068832.1	180	6	31.67
ZNF729	NP_001229609.1	222	0	0.00	ARMCX5	NP_001161950.1	157	15	90.76
ZNF98	NP_001092096.1	194	0	0.00	GPRASP1	NP_055525.3	414	13	29.83

ZNF492	NP_065906.1	193	0	0.00	GPRASP2	NP_612446.1	458	12	24.89
ZNF99	NP_001073878.2	221	0	0.00	BHLHB9	NP_001337198.1	181	4	20.99
ZNF728	NP_001254645.1	137	0	0.00	RAB40AL	NP_001027004.1	632	7	10.52
ZNF730	XP_016881604.1	158	0	0.00	BEX1	NP_060946.3	569	12	20.03
ZNF724	NP_001342333.1	142	0	0.00	NXF3	NP_071335.1	481	8	15.80
ZNF91	XP_024307461.1	261	0	0.00	BEX4	NP_001073894.1	114	4	33.33
ZNF675	NP_612203.2	223	0	0.00	TCEAL8	NP_699164.1	134	6	42.54
ZNF681	NP_612143.2	164	0	0.00	TCEAL5	NP_001012997.1	537	9	15.92
RPSAP58	NP_001342212.1	1038	0	0.00	BEX2	NP_001161872.1	405	14	32.84
ZNF726	NP_001335616.1	140	0	0.00	TCEAL7	NP_001335187.1	444	6	12.84
ZNF254	NP_975011.3	246	0	0.00	TCEAL9	NP_057387.1	140	4	27.14
UQCRFS1	NP_005994.2	1280	7	5.20	BEX3	NP_001269603.1	268	15	53.17
VSTM2B	NP_001139811.1	897	8	8.47	RAB40A	NP_543155.2	511	8	14.87
POP4	NP_006618.1	612	8	12.42	TCEAL4	NP_001287830.1	101	2	18.81
PLEKHF1	XP_005259313.1	358	8	21.23	TCEAL3	NP_116315.1	102	7	65.19
C19orf12	NP_001026896.2	249	8	30.52	TCEAL1	NP_004771.2	188	5	25.26
CCNE1	NP_001229.1	2145	9	3.99	MORF4L2	NP_001135890.1	1032	13	11.97
URI1	NP_001239570.1	387	7	17.18	TMEM31	NP_872347.2	59	0	0.00
ZNF536	XP_016883016.1	738	8	10.30	PLP1	NP_001122306.1	1433	7	4.64
TSHZ3	NP_065907.2	480	6	11.87	RAB9B	NP_057454.1	867	7	7.67
ZNF507	NP_001129628.1	480	1	1.98	TMSB15B	NP_001337142.1	167	5	28.44
DPY19L3	NP_997208.2	253	0	0.00	H2BW1	NP_001002916.3	712	6	8.01
PDCD5	NP_004699.1	837	3	3.40	H2BW2	NP_001364002.1	709	2	2.68
ANKRD27	NP_115515.2	1181	2	1.61	SLC25A53	XP_016885003.1	66	0	0.00
RGS9BP	NP_997274.2	224	2	8.48	ZCCHC18	NP_001137450.1	117	1	8.12
NUDT19	NP_001099040.1	500	1	1.90	FAM199X	NP_997201.1	42	0	0.00
TDRD12	XP_011525773.1	1305	1	0.73	ESX1	NP_703149.1	774	2	2.45
SLC7A9	NP_001119807.1	417	2	4.56	IL1RAPL2	NP_059112.1	519	3	5.49
CEP89	XP_005259401.1	304	4	12.50	TEX13A	NP_112564.1	454	0	0.00
FAAP24	NP_689479.1	358	1	2.65	NRK	NP_940867.2	182	2	10.44
RHPN2	NP_149094.3	599	1	1.59	SERPINA7	NP_000345.2	632	1	1.50
GPATCH1	NP_060495.2	262	0	0.00	PWWP3B	XP_016884758.1	141	3	20.21
WDR88	NP_775750.3	428	0	0.00	RADX	NP_060485.4	171	6	33.33
LRP3	XP_005259002.1	311	0	0.00	RNF128	NP_078815.3	366	3	7.79
SLC7A10	XP_024307377.1	332	3	8.58	TBC1D8B	NP_060222.2	636	7	10.46
CEBPA	NP_001274353.1	1699	2	1.12	RIPPLY1	NP_001165177.1	97	3	29.38
CEBPG	XP_024307095.1	488	3	5.84	CLDN2	NP_001164563.1	442	5	10.75
PEPD	NP_001159529.1	1072	2	1.77	MORC4	XP_006724754.1	277	6	20.58
CHST8	XP_016882632.1	348	1	2.73	RBM41	NP_001164551.1	263	5	18.06
KCTD15	XP_016882773.1	404	2	4.70	NUP62CL	XP_024308161.1	286	7	23.25
LSM14A	NP_001371349.1	768	10	12.37	DNAAF6	NP_001162625.1	122	2	15.57
GARRE1	NP_055501.2	119	5	39.91	FRMPD3	NP_115804.1	283	0	0.00
GPI	NP_001276718.1	1477	5	3.22	PRPS1	NP_002755.1	1278	6	4.46
PDCD2L	NP_115722.1	396	3	7.20	TSC22D3	XP_016884824.1	531	1	1.79
UBA2	XP_005258461.2	1577	11	6.63	NCBP2L	NP_001335301.1	851	1	1.12
WTIP	XP_011524754.1	643	10	14.77	MID2	NP_036348.2	570	9	15.00
SCGB2B2	NP_001020762.1	172	9	49.71	TEX13B	NP_112563.1	58	0	0.00
ZNF302	XP_016882467.1	263	5	18.06	VSIG1	XP_011529238.1	235	5	20.21
ZNF181	NP_001025168.2	174	5	27.30	PSMD10	NP_002805.1	1482	9	5.77
ZNF599	NP_001007249.1	139	5	34.17	ATG4A	NP_443168.2	964	8	7.88
ZNF30	XP_016882912.1	261	5	18.20	COL4A6	NP_001838.2	612	8	12.42
ZNF792	NP_787068.3	203	0	0.00	COL4A5	NP_203699.1	796	10	11.93
GRAMD1A	XP_011525455.1	127	1	7.48	IRS4	XP_011529363.1	583	5	8.15
SCN1B	NP_001028.1	353	8	21.53	GUCY2F	NP_001513.2	1006	16	15.11
HPN	XP_016882220.1	536	2	3.54	NXT2	NP_061168.2	414	13	29.83
FXYD3	XP_011525334.1	295	4	12.88	KCNE5	NP_036414.1	179	7	37.15
LGI4	NP_644813.1	501	5	9.48	ACSL4	XP_024308119.1	1118	14	11.90
FXYD1	XP_016882364.1	601	6	9.48	TMEM164	NP_001340778.1	204	13	60.54
FXYD7	NP_071289.1	424	6	13.44	AMMECR1	NP_001020751.1	451	17	35.81
FXYD5	NP_054883.3	200	3	14.25	RTL9	XP_016885184.1	210	15	67.85
FAM187B	NP_689694.1	55	0	0.00	CHRDL1	NP_001137455.2	354	9	24.15
LSR	XP_005259037.1	295	0	0.00	PAK3	XP_016885052.1	1392	9	6.14
USF2	XP_024307452.1	702	1	1.35	CAPN6	NP_055104.2	676	3	4.22
HAMP	NP_066998.1	596	2	3.19	DCX	NP_000546.2	904	7	7.36
MAG	NP_002352.1	1482	4	2.56	ALG13	XP_011529330.1	900	11	11.61
CD22	NP_001762.2	894	5	5.31	TRPC5	XP_016885263.1	601	10	15.81

FFAR1	NP_005294.1	413	4	9.20	RTL4	NP_001004308.2	122	4	31.15
FFAR3	XP_011525160.1	397	3	7.18	LHFPL1	XP_016884974.1	306	11	34.15
GPR42	NP_001335124.1	116	2	16.38	AMOT	NP_001106962.1	556	9	15.38
FFAR2	XP_016882200.1	441	3	6.46	HTR2C	NP_001243689.1	922	4	4.12
KRTDAP	NP_001231776.1	348	3	8.19	IL13RA2	NP_000631.1	382	1	2.49
DMKN	NP_001177276.2	147	2	12.92	LRCH2	XP_006724787.2	630	0	0.00
SBSN	NP_001159507.1	255	2	7.45	RBMXL3	NP_001138818.1	74	0	0.00
GAPDHS	NP_055179.1	2198	0	0.00	LUZP4	NP_057467.1	191	0	0.00
TMEM147	NP_116024.1	361	3	7.89	PLS3	NP_001129497.1	1294	3	2.20
ATP4A	NP_000695.2	1404	1	0.68	AGTR2	NP_001372553.1	830	7	8.01
HAUS5	NP_056117.1	296	1	3.21	SLC6A14	NP_009162.1	505	4	7.52
RBM42	NP_077297.2	356	0	0.00	CT83	NP_001017978.1	141	1	6.74
ETV2	XP_005258709.1	354	0	0.00	KLHL13	XP_016885439.1	616	6	9.25
COX6B1	NP_001854.1	783	3	3.64	WDR44	NP_061918.3	303	7	21.95
UPK1A	NP_008931.1	256	3	11.13	DOCK11	XP_005262425.1	484	6	11.78
ZBTB32	XP_016882080.1	418	3	6.82	IL13RA1	XP_011529638.1	546	6	10.44
KMT2B	NP_055542.1	2068	2	0.92	ZCCHC12	NP_776159.1	775	2	2.45
IGFLR1	NP_001332933.1	120	7	55.41	LONRF3	XP_005262533.1	1136	4	3.34
U2AF1L4	NP_659424.2	655	1	1.45	KIAA1210	XP_016885178.1	189	4	20.10
PSENEN	NP_758844.1	434	5	10.94	PGRMC1	NP_006658.1	884	5	5.37
LIN37	NP_061977.1	217	7	30.64	SLC25A43	NP_660348.2	393	15	36.26
HSPB6	NP_653218.1	567	2	3.35	SLC25A5	NP_001143.2	1193	11	8.76
PROSER3	XP_011524830.1	101	3	28.22	CXorf56	NP_001164041.1	434	12	26.27
ARHGAP33	XP_006723062.1	353	3	8.07	UBE2A	NP_003327.2	2233	15	6.38
PRODH2	NP_001365221.1	541	6	10.54	NKRF	NP_060014.3	908	8	8.37
NPHS1	NP_004637.1	902	2	2.11	SEPTIN6	XP_011529619.1	640	7	10.39
KIRREL2	NP_115499.5	315	10	30.16	SOWAHD	NP_001099046.1	60	0	0.00
APLP1	NP_001019978.1	1375	11	7.60	RPL39	NP_000991.1	1017	4	3.74
NFKBID	NP_116110.2	1021	10	9.30	UPF3B	XP_016885227.1	876	12	13.01
HCST	XP_016881682.1	604	1	1.57	RNF113A	NP_008909.1	501	13	24.65
TYROBP	NP_001166986.1	1055	5	4.50	NDUFA1	NP_004532.1	615	6	9.27
LRFN3	NP_078785.1	561	11	18.63	AKAP14	NP_848928.1	276	4	13.77
SDHAF1	NP_001036096.2	258	10	36.82	NKAP	NP_078804.2	415	12	27.47
SYNE4	NP_001284664.1	120	0	0.00	RHOXF2B	NP_001093155.1	391	3	7.29
ALKBH6	NP_116267.3	72	2	26.39	RHOXF1	XP_011529583.1	426	2	4.46
CLIP3	NP_056341.1	933	9	9.16	RHOXF2	NP_115887.1	501	3	5.69
THAP8	NP_001318031.1	46	0	0.00	TMEM255A	XP_016885108.1	207	0	0.00
WDR62	XP_016882154.1	478	1	1.99	ZBTB33	NP_001171671.1	615	2	3.09
OVOL3	XP_016882679.1	122	7	54.51	ATP1B4	NP_001135919.1	392	2	4.85
POLR2I	NP_006224.1	1334	7	4.98	LAMP2	NP_001116078.1	1274	4	2.98
TBCB	NP_001272.2	595	2	3.19	CUL4B	NP_003579.3	1441	7	4.61
CAPNS1	NP_001740.1	623	7	10.67	MCTS1	NP_054779.1	1004	4	3.78
COX7A1	NP_001855.1	526	5	9.03	C1GALT1C1	NP_689905.1	520	4	7.31
ZNF565	NP_689690.3	169	0	0.00	CT47B1	XP_016885223.1	26	0	0.00
ZNF146	XP_016882735.1	308	0	0.00	CT47A12	XP_024308089.1	52	0	0.00
ZFP14	XP_016882527.1	292	0	0.00	CT47A11	NP_775842.2	53	0	0.00
ZFP82	NP_001308848.1	172	0	0.00	CT47A10	NP_001073606.1	137	0	0.00
ZNF566	XP_005259411.1	192	1	4.95	CT47A9	NP_001073607.1	52	0	0.00
ZNF260	XP_016882230.1	258	0	0.00	CT47A8	NP_001073608.1	52	0	0.00
ZNF529	XP_011525471.1	170	0	0.00	CT47A7	NP_001073609.1	52	0	0.00
ZNF382	NP_001243767.1	239	0	0.00	CT47A6	NP_001073610.1	52	0	0.00
ZNF461	NP_001284552.1	217	0	0.00	CT47A5	NP_001073611.1	53	0	0.00
ZNF567	XP_016881908.1	188	0	0.00	CT47A4	NP_001073612.1	53	0	0.00
ZNF850	NP_001254708.1	188	0	0.00	CT47A3	NP_001073613.1	52	0	0.00
ZNF790	NP_996777.2	178	0	0.00	CT47A2	NP_001073614.1	54	0	0.00
ZNF345	XP_016882057.1	198	0	0.00	CT47A1	NP_001073615.1	53	0	0.00
ZNF829	NP_001165450.1	202	0	0.00	GLUD2	NP_036216.2	908	2	2.09
ZNF568	XP_016882261.1	180	0	0.00	GRIA3	NP_000819.4	1134	6	5.03
ZNF420	NP_001316444.1	287	0	0.00	THOC2	NP_001075019.1	1179	7	5.64
ZNF585A	NP_001275729.1	151	0	0.00	XIAP	NP_001365519.1	1442	7	4.61
ZNF585B	NP_689492.3	175	0	0.00	STAG2	NP_001036214.1	1276	5	3.72
ZNF383	XP_016881911.1	279	0	0.00	SH2D1A	NP_002342.1	864	4	4.40
ZNF875	NP_001316694.1	223	0	0.00	TENM1	NP_001156751.1	610	9	14.02
ZNF527	NP_115829.1	136	0	0.00	DCAF12L2	NP_001013650.1	95	0	0.00
ZNF569	XP_006723109.1	725	0	0.00	DCAF12L1	NP_848565.2	328	12	34.75
ZNF570	NP_001287922.1	171	0	0.00	PRR32	NP_001116188.1	74	7	89.86

ZNF793	XP_024307279.1	160	0	0.00	ACTRT1	NP_612146.1	982	4	3.87
ZNF571	XP_016882344.1	219	0	0.00	SMARCA1	NP_001269804.1	1887	9	4.53
ZNF540	NP_689819.1	214	0	0.00	OCRL	NP_001305713.1	1226	15	11.62
ZFP30	NP_001307599.1	230	0	0.00	APLN	NP_059109.3	414	2	4.59
ZNF781	NP_689818.2	173	0	0.00	XPNPEP2	NP_003390.4	814	10	11.67
ZNF607	NP_001362824.1	161	0	0.00	SASH3	XP_006724826.1	829	7	8.02
ZNF573	NP_001166162.1	209	0	0.00	ZDHHC9	NP_057116.2	400	13	30.87
WDR87	NP_114157.4	77	1	12.34	UTP14A	NP_006640.2	989	13	12.49
SIPA1L3	XP_005258728.1	354	2	5.37	BCORL1	XP_016885210.1	1060	11	9.86
DPF1	XP_005259349.1	299	0	0.00	ELF4	NP_001120669.1	459	2	4.14
PPP1R14A	NP_001230876.1	412	4	9.22	RAB33A	XP_016885452.1	767	8	9.91
SPINT2	NP_066925.1	498	4	7.63	AIFM1	NP_665811.1	1152	9	7.42
C19orf33	NP_277055.1	111	2	17.12	ZNF280C	XP_006724828.1	318	9	26.89
YIF1B	NP_001034762.1	342	1	2.78	SLC25A14	XP_005262546.1	428	9	19.98
KCNK6	NP_004814.1	275	3	10.36	GPR119	NP_848566.1	299	3	9.53
CATSPERG	NP_067008.3	147	3	19.39	RBMX2	NP_057108.2	466	6	12.23
PSMD8	NP_002803.2	1314	4	2.89	ENOX2	XP_011529547.1	307	11	34.04
GGN	XP_016881940.1	293	0	0.00	ARHGAP36	NP_001269536.1	462	5	10.28
SPRED3	NP_001035987.1	249	1	3.82	IGSF1	NP_001164433.1	293	10	32.42
FAM98C	NP_777565.3	99	2	19.19	OR13H1	NP_001004486.1	79	4	48.10
RASGRP4	NP_001139674.1	673	2	2.82	STK26	NP_057626.2	776	9	11.02
RYR1	NP_000531.2	1110	5	4.28	FRMD7	NP_001293122.1	169	9	50.59
MAP4K1	NP_001036065.1	400	2	4.75	RAP2C	NP_067006.3	1685	6	3.38
EIF3K	NP_037366.1	807	3	3.53	MBNL3	XP_024308170.1	459	12	24.84
ACTN4	XP_016882820.1	1473	5	3.22	HS6ST2	NP_671704.3	392	11	26.66
CAPN12	XP_016881846.1	561	2	3.39	USP26	XP_016885381.1	521	1	1.82
LGALS7	NP_002298.1	434	0	0.00	TFDP3	NP_057605.3	429	3	6.64
LGALS7B	NP_001035972.1	430	0	0.00	GPC4	NP_001439.2	658	4	5.77
LGALS4	XP_011525275.1	2355	3	1.21	GPC3	NP_001158091.1	1103	6	5.17
ECH1	NP_001389.2	660	5	7.20	CCDC160	NP_001340382.1	23	1	41.30
HNRNPL	XP_024307249.1	1334	1	0.71	PHF6	NP_115834.1	1918	9	4.46
RINL	NP_001182762.1	212	1	4.48	HPRT1	NP_000185.1	2315	9	3.69
SIRT2	XP_011524957.1	1580	3	1.80	PLAC1	XP_016884725.1	281	6	20.28
NFKBIB	XP_006723290.3	1272	2	1.49	FAM122B	NP_001318022.1	173	7	38.44
CCER2	XP_011525521.1	18	2	105.55	FAM122C	XP_011529598.1	222	5	21.40
SARS2	NP_060297.1	835	4	4.55	MOSPD1	XP_016885143.1	762	7	8.73
MRPS12	NP_066930.1	1189	1	0.80	SMIM10	NP_001156910.1	1	0	0.00
FBXO17	NP_680474.1	472	4	8.05	RTL8B	NP_001071641.1	61	0	0.00
FBXO27	XP_016881779.1	409	2	4.65	RTL8C	NP_001071639.1	133	0	0.00
ACP7	XP_016882296.1	300	1	3.17	RTL8A	NP_001127793.1	83	1	11.45
PAK4	NP_005875.1	1281	4	2.97	CT55	XP_005262484.1	479	1	1.98
NCCRP1	NP_001001414.1	471	2	4.03	ZNF75D	XP_011529697.1	251	2	7.57
SYCN	NP_001073937.1	201	1	4.73	ZNF449	NP_689908.3	260	1	3.65
IFNL3	NP_001333866.1	321	1	2.96	INTS6L	XP_006724803.1	164	6	34.75
IFNL2	NP_742150.1	253	1	3.75	CT45A1	XP_005278198.1	196	2	9.69
IFNL1	NP_742152.1	342	2	5.56	CT45A3	NP_001357077.1	184	2	10.33
LRFN1	XP_016882522.1	567	2	3.35	CT45A5	NP_001165759.2	184	2	10.33
GMFG	NP_004868.1	570	3	5.00	CT45A6	XP_005262482.2	153	1	6.21
SAMD4B	XP_016882407.1	378	3	7.54	CT45A2	XP_006724846.1	113	1	8.41
PAF1	NP_001243755.1	737	3	3.87	CT45A7	XP_003960131.1	184	3	15.49
MED29	NP_001304699.2	280	3	10.18	CT45A8	XP_006724862.1	113	1	8.41
ZFP36	NP_003398.3	964	0	0.00	CT45A9	NP_001308200.1	112	2	16.96
PLEKHG2	NP_001338622.1	208	3	13.70	CT45A10	NP_001372148.1	184	3	15.49
RPS16	NP_001308040.1	1609	1	0.59	SAGE1	XP_016885111.1	256	8	29.69
SUPT5H	NP_001104490.1	1413	5	3.36	MMGT1	NP_001316929.1	254	0	0.00
TIMM50	NP_001001563.2	730	2	2.60	SLC9A6	NP_001036002.1	735	4	5.17
DLL3	NP_982353.1	457	0	0.00	FHL1	NP_001153173.1	763	2	2.49
SELENOV	NP_874363.1	111	0	0.00	MAP7D3	NP_001166987.1	333	11	31.38
EID2B	NP_689574.1	95	0	0.00	ADGRG4	NP_722576.3	572	8	13.29
EID2	NP_694964.3	107	0	0.00	BRS3	NP_001718.1	471	2	4.03
LGALS13	XP_016882204.1	134	0	0.00	HTATSF1	NP_055315.2	499	3	5.71
LGALS16	NP_001177370.2	62	0	0.00	VGLL1	NP_057351.1	232	4	16.38
LGALS14	NP_064514.1	95	0	0.00	CD40LG	NP_000065.1	1555	7	4.28
CLC	NP_001819.2	299	2	6.35	ARHGEF6	XP_005262556.1	655	8	11.60
LEUTX	NP_001369274.1	251	0	0.00	RBMX	NP_001158275.1	1081	7	6.15
DYRK1B	NP_006474.1	1021	8	7.44	GPR101	NP_473362.1	237	6	24.05

FBL	XP_011524925.1	2008	3	1.42	ZIC3	NP_001317590.1	876	3	3.25
FCGBP	NP_003881.2	592	7	11.23	FGF13	NP_001132970.1	1856	4	2.05
PSMC4	NP_006494.1	1511	9	5.66	F9	NP_000124.1	677	6	8.42
ZNF546	NP_848639.2	244	8	31.15	MCF2	XP_016885019.1	799	3	3.57
ZNF780B	XP_016881914.1	246	9	34.75	ATP11C	NP_001340739.2	768	9	11.13
ZNF780A	NP_001136051.1	206	12	55.34	CXorf66	NP_001013421.1	30	0	0.00
MAP3K10	XP_011525283.1	1146	10	8.29	SOX3	NP_005625.2	888	8	8.56
TTC9B	NP_689692.2	732	16	20.76	CDR1	NP_004056.2	332	4	11.45
CCNP	XP_006723458.2	1262	16	12.04	SPANXB1	NP_115850.2	121	2	15.70
AKT2	NP_001317440.1	2135	6	2.67	LDOC1	NP_036449.1	223	10	42.60
C19orf47	XP_011524762.1	94	17	171.80	SPANXC	NP_073152.2	151	4	25.16
PLD3	XP_016882035.1	380	11	27.50	SPANXA1	NP_038481.2	196	4	19.39
HIPK4	NP_653286.2	865	8	8.79	SPANXA2	NP_663695.1	196	4	19.39
PRX	NP_870998.2	222	4	17.12	SPANXD	NP_115793.1	121	5	39.25
SERTAD1	NP_037508.2	246	16	61.79	MAGEC3	NP_619647.1	305	7	21.80
SERTAD3	NP_037500.2	187	15	76.20	MAGEC1	NP_005453.2	306	9	27.94
BLVRB	NP_000704.1	425	8	17.88	MAGEC2	NP_057333.1	395	7	16.83
SPTBN4	XP_016882538.1	1279	15	11.14	SPANXN4	XP_016885032.1	65	2	29.23
SHKBP1	XP_006723537.1	349	9	24.50	SPANXN3	NP_001009609.2	83	0	0.00
LTBP4	NP_003564.2	392	11	26.66	SLITRK4	NP_775101.1	233	3	12.23
NUMBL	NP_004747.1	819	9	10.44	SPANXN2	NP_001009615.1	82	0	0.00
COQ8B	NP_001136027.1	447	8	17.00	SPANXN1	NP_001009614.1	63	0	0.00
ITPKC	NP_079470.1	1402	10	6.78	SLITRK2	NP_001137475.1	418	3	6.82
C19orf54	XP_016882111.1	90	15	158.33	FMR1	NP_002015.1	1994	5	2.38
SNRPA	NP_004587.1	1509	2	1.26	FMR1NB	NP_689791.1	184	7	36.14
MIA	NP_001189482.1	146	1	6.51	AFF2	NP_002016.2	439	8	17.31
RAB4B	NP_057238.3	1408	14	9.45	IDS	NP_000193.1	298	2	6.38
EGLN2	NP_542770.2	673	9	12.70	EOLA1	NP_001311208.1	70	2	27.14
CYP2A6	NP_000753.3	597	6	9.55	MAGEA9B	NP_001074259.1	185	5	25.67
CYP2A7	NP_000755.2	426	5	11.15	HSFX2	NP_001157887.1	607	5	7.82
CYP2B6	NP_000758.1	925	7	7.19	TMEM185A	NP_001167563.1	168	8	45.24
CYP2A13	NP_000757.2	432	5	10.99	MAGEA11	NP_001011544.1	293	3	9.73
CYP2F1	XP_016881874.1	388	0	0.00	HSFX1	NP_057237.1	597	5	7.96
CYP2S1	NP_085125.1	404	7	16.46	MAGEA9	XP_024308149.1	172	7	38.66
AXL	NP_068713.2	1451	3	1.96	MAGEA8	XP_016885009.1	168	1	5.65
HNRNPUL1	XP_005258516.1	795	7	8.36	EOLA2	XP_016885079.1	51	1	18.63
CCDC97	XP_016882931.1	140	5	33.93	MAMLD1	XP_024308085.1	314	4	12.10
TGFB1	NP_000651.3	2368	7	2.81	MTM1	XP_016885037.1	498	5	9.54
B9D2	NP_085055.2	416	9	20.55	MTMR1	XP_005274822.1	463	4	8.21
TMEM91	NP_001092292.1	148	13	83.44	CD99L2	NP_604394.1	167	5	28.44
EXOSC5	NP_064543.3	1161	8	6.55	HMGB3	NP_001288160.1	995	3	2.86
BCKDHA	NP_000700.1	660	5	7.20	GPR50	NP_004215.2	296	6	19.26
B3GNT8	NP_001372577.1	78	1	12.18	VMA21	NP_001350739.1	179	5	26.53
DMAC2	NP_001161343.1	250	11	41.80	PASD1	NP_775764.2	277	5	17.15
ERICH4	XP_016881608.1	49	0	0.00	PRRG3	NP_001359092.1	127	4	29.92
CEACAM21	XP_016882919.1	219	6	26.03	FATE1	NP_149076.1	206	6	27.67
CEACAM4	XP_016881689.1	487	6	11.70	CNGA2	NP_005131.1	457	1	2.08
CEACAM7	NP_001278414.1	257	7	25.87	MAGEA4	NP_001373132.1	307	3	9.28
CEACAM5	NP_004354.3	784	2	2.42	GABRE	NP_004952.2	352	3	8.10
CEACAM6	XP_011525292.1	474	3	6.01	MAGEA10	NP_066386.3	314	3	9.08
CEACAM3	NP_001806.2	368	2	5.16	GABRA3	NP_000799.1	669	5	7.10
LYPD4	XP_024307142.1	179	2	10.61	GABRQ	XP_011529486.1	509	6	11.20
DMRTC2	XP_016882612.1	242	3	11.78	MAGEA3	NP_005353.1	448	3	6.36
RPS19	NP_001308414.1	1463	1	0.65	MAGEA2B	NP_001308329.1	213	4	17.84
CD79A	NP_001774.1	782	7	8.50	CSAG1	NP_705611.2	132	10	71.97
ARHGEF1	NP_945353.1	953	4	3.99	MAGEA12	NP_001159858.1	203	2	9.36
RABAC1	NP_006414.2	563	3	5.06	MAGEA2	NP_001373059.1	218	1	4.36
ATP1A3	NP_689509.1	1370	8	5.55	MAGEA6	NP_787064.1	195	1	4.87
GRIK5	NP_002079.3	962	2	1.97	CETN2	NP_004335.1	1706	2	1.11
ZNF574	NP_001317448.1	236	6	24.15	NSDHL	XP_016885053.1	668	2	2.84
POU2F2	XP_005259067.1	557	2	3.41	ZNF185	XP_016885310.1	636	1	1.49
DEDD2	NP_001257544.1	263	0	0.00	PNMA5	XP_016884741.1	183	1	5.19
ZNF526	NP_597701.1	255	8	29.80	PNMA3	NP_001269464.1	453	1	2.10
GSK3A	NP_063937.2	2249	8	3.38	PNMA6A	NP_116271.3	292	1	3.25
ERF	NP_006485.2	508	1	1.87	MAGEA1	NP_004979.3	386	3	7.38
CIC	NP_001291744.1	305	8	24.92	ZNF275	NP_001354686.1	209	2	9.09

PAFAH1B3	XP_016882335.1	343	12	33.23	ZFP92	XP_016884770.1	125	0	0.00
PRR19	XP_005258836.2	46	7	144.56	TREX2	NP_542432.2	292	1	3.25
TMEM145	NP_775904.2	341	15	41.79	HAUS7	NP_001372411.1	178	4	21.35
MEGF8	NP_001258867.1	237	12	48.10	BGN	XP_016885213.1	1369	1	0.69
CNFN	XP_005259389.1	268	10	35.45	ATP2B3	XP_005274746.1	1749	8	4.35
LIPE	XP_005258994.1	842	7	7.90	CCNQ	XP_005277978.1	446	3	6.39
CXCL17	NP_940879.1	192	2	9.90	DUSP9	XP_011529425.1	826	3	3.45
CEACAM1	NP_001171742.1	634	4	5.99	PNCK	XP_011529410.1	1239	8	6.13
CEACAM8	XP_016881684.1	459	1	2.07	SLC6A8	NP_005620.1	561	12	20.32
PSG3	XP_011525429.1	111	2	17.12	BCAP31	NP_001132929.1	768	9	11.13
PSG8	NP_001123640.1	71	2	26.76	ABCD1	NP_000024.2	534	9	16.01
PSG1	NP_001171755.1	138	2	13.77	PLXNB3	NP_005384.2	703	10	13.51
PSG6	NP_001027020.1	94	2	20.21	SRPK3	NP_055185.2	529	11	19.75
PSG7	NP_001193579.1	98	1	9.69	IDH3G	NP_004126.1	938	15	15.19
PSG11	NP_976032.2	109	2	17.43	SSR4	NP_001191456.1	661	10	14.37
PSG2	NP_112536.2	102	0	0.00	PDZD4	NP_001290443.1	933	14	14.25
PSG5	XP_016882492.1	98	0	0.00	L1CAM	NP_001137435.1	884	8	8.60
PSG4	NP_001303268.1	134	1	7.09	AVPR2	NP_000045.1	951	11	10.99
PSG9	NP_001288638.1	122	1	7.79	ARHGAP4	NP_001657.3	656	12	17.38
CD177	XP_016882510.1	416	6	13.70	NAA10	NP_001243049.1	1031	12	11.06
TEX101	NP_113639.4	318	6	17.92	RENBP	NP_002901.2	258	14	51.55
LYPD3	NP_055215.2	321	7	20.72	HCFC1	XP_016884960.1	1022	8	7.44
PHLDB3	XP_005259227.1	82	5	57.92	TMEM187	NP_003483.1	92	11	113.58
ETHE1	NP_001307798.1	397	10	23.93	IRAK1	NP_001020414.1	1752	12	6.51
ZNF575	NP_777605.1	197	5	24.11	MECP2	NP_001104262.1	1398	13	8.83
XRCC1	NP_006288.2	795	2	2.39	OPN1LW	NP_064445.2	307	3	9.28
PINLYP	NP_001180550.2	101	6	56.43	OPN1MW	NP_000504.1	291	3	9.79
IRGQ	XP_005258572.1	125	2	15.20	OPN1MW2	NP_001041646.1	282	2	6.74
ZNF576	NP_077303.1	162	0	0.00	OPN1MW3	NP_001316996.1	213	0	0.00
ZNF428	NP_872304.2	88	0	0.00	TEX28	XP_011529418.1	159	9	53.77
SRRM5	NP_001139113.1	18	0	0.00	TKTL1	NP_036385.3	878	10	10.82
CADM4	XP_016881941.1	561	1	1.69	FLNA	NP_001447.2	1811	11	5.77
PLAUR	NP_001005376.1	1035	2	1.84	EMD	NP_000108.1	601	6	9.48
IRGC	XP_005259115.1	239	1	3.97	RPL10	NP_001243506.2	1394	3	2.04
SMG9	XP_005259114.1	292	0	0.00	DNASE1L1	XP_016884821.1	315	5	15.08
KCNN4	XP_005258939.1	596	0	0.00	TAZ	XP_006724899.1	595	8	12.77
LYPD5	NP_001026919.2	192	2	9.90	ATP6AP1	NP_001174.2	454	4	8.37
ZNF283	XP_016882122.1	183	1	5.19	GDI1	NP_001484.1	1018	7	6.53
ZNF404	NP_001028891.2	197	0	0.00	FAM50A	NP_004690.1	178	8	42.69
ZNF45	XP_016882706.1	250	0	0.00	PLXNA3	NP_059984.3	492	2	3.86
ZNF221	XP_016882721.1	121	0	0.00	LAGE3	NP_006005.2	244	2	7.79
ZNF155	NP_932355.3	151	0	0.00	UBL4A	NP_055050.1	2274	5	2.09
ZNF230	NP_006291.2	114	0	0.00	SLC10A3	XP_006724911.1	267	1	3.56
ZNF222	NP_001123468.1	177	0	0.00	FAM3A	NP_001164604.1	67	4	56.71
ZNF223	XP_016882747.1	175	0	0.00	G6PD	NP_000393.4	1615	9	5.29
ZNF284	XP_024307254.1	114	0	0.00	IKBKG	NP_001093326.2	1682	10	5.65
ZNF224	NP_001308574.1	305	0	0.00	CTAG1A	NP_640343.1	418	1	2.27
ZNF225	NP_001308614.1	194	0	0.00	CTAG1B	NP_001318.1	419	1	2.27
ZNF234	XP_016881638.1	154	0	0.00	CTAG2	NP_758965.2	185	4	20.54
ZNF226	XP_016882751.1	259	0	0.00	GAB3	XP_011529407.1	160	2	11.87
ZNF227	XP_016882755.1	208	0	0.00	DKC1	NP_001354.1	1673	5	2.84
ZNF233	XP_024307256.1	140	0	0.00	MPP1	XP_024308153.1	608	3	4.69
ZNF235	XP_005259469.1	249	0	0.00	SMIM9	NP_001156408.1	0	0	#DIV/0!
ZNF112	NP_001335210.1	176	0	0.00	F8	NP_000123.1	511	9	16.73
ZNF285	NP_689567.4	142	0	0.00	H2AB1	NP_001017990.1	929	2	2.05
ZNF229	NP_001265439.2	156	0	0.00	F8A1	NP_036283.2	192	2	9.90
ZNF180	NP_001265437.2	221	0	0.00	FUNDC2	NP_076423.2	275	10	34.54
CEACAM20	XP_024307113.1	134	2	14.18	CMC4	NP_001018024.1	91	6	62.63
IGSF23	NP_001192209.1	57	3	50.00	MTCP1	NP_001018025.1	77	1	12.34
PVR	NP_006496.4	547	6	10.42	BRCC3	XP_005274808.1	1057	6	5.39
CEACAM19	NP_064604.2	75	7	88.66	VBP1	NP_001290473.1	1039	9	8.66
CEACAM16	NP_001034302.2	265	7	25.09	RAB39B	NP_741995.1	1501	6	4.22
BCL3	XP_011525500.2	1787	10	5.32	CLIC2	NP_001280.3	429	8	20.84
CBLC	NP_036248.3	544	2	3.49	H2AB2	NP_001017991.1	714	4	6.65
BCAM	NP_005572.2	261	8	29.12	F8A2	NP_001007524.1	215	4	23.56
NECTIN2	NP_001036189.1	439	10	21.64	F8A3	NP_001007525.1	160	3	25.45

TOMM40	NP_001122388.1	1224	12	9.31	H2AB3	NP_542451.1	714	3	6.14
APOE	NP_001289617.1	2845	12	4.01	TMLHE	NP_060666.1	527	8	24.03
APOC1	NP_001307994.1	529	10	17.96	SPRY3	NP_005831.1	283	6	36.62
					VAMP7	NP_001172112.1	1253	2	3.03

Mus musculus

Locus	Protein product	Co-expressions and co-interactions		Co-localization likelihood fold-change (FC)	Locus	Protein product	Co-expressions and co-interactions		Co-localization likelihood fold-change (FC)
		Total	witihin ±10 neighbor genes				Total	witihin ±10 neighbor genes	
Xkr4	XP_006495613.1	292	1	7.22	Rmdn3	NP_001028308.1	290	7	25.44
Rp1	XP_030108185.2	860	0	0.00	Gchfr	NP_796131.1	356	5	14.80
Sox17	NP_001276394.1	963	0	0.00	Dnajc17	NP_631878.2	408	11	28.41
Mrpl15	NP_079576.1	1054	0	0.00	Gm14137	NP_001034312.2	11	1	95.81
Lypla1	NP_032892.1	575	2	5.24	Zfyve19	XP_006500267.1	761	8	11.08
Tcea1	NP_035671.1	1118	2	2.51	Ppp1r14d	NP_001277725.1	78	0	0.00
Rgs20	NP_001171266.1	588	1	2.24	Spint1	XP_030104818.1	656	6	9.64
Atp6v1h	NP_598587.2	668	1	1.86	Rhov	XP_011237767.1	1527	0	0.00
Oprk1	NP_001305664.1	757	3	4.64	Vps18	NP_758473.3	693	5	7.60
Npbwr1	NP_034472.1	480	2	4.62	Dll4	NP_062327.2	895	6	7.07
Rb1cc1	XP_036012136.1	672	3	4.70	Chac1	NP_081205.1	405	7	18.22
Alkal1	NP_001182661.1	68	0	0.00	Ino80	XP_036018407.1	1274	3	2.48
St18	XP_030109821.1	197	2	10.70	Exd1	XP_017173654.1	223	0	0.00
Pcmtd1	NP_898849.1	493	3	6.41	Chp1	NP_062743.1	1251	1	0.84
Sntg1	XP_036009553.1	591	1	1.78	Oip5	NP_001036118.1	542	8	15.56
Rrs1	NP_067486.2	1133	1	0.93	Nusap1	NP_598612.1	703	3	4.50
Adhfe1	NP_780445.1	886	4	4.76	Ndufaf1	NP_001343396.1	466	5	11.31
Vxn	NP_848486.1	307	4	13.73	H3f3c	NP_001357860.1	1927	3	1.64
Mybl1	XP_006495524.1	2503	8	3.37	Rtf1	NP_084388.2	889	5	5.93
Vcpip1	NP_775619.2	468	7	15.76	Itpka	NP_666237.1	494	3	6.40
Sgk3	NP_573483.1	1597	6	3.96	Ltk	NP_996825.2	990	1	1.06
Mcmdc2	NP_808390.2	728	1	1.45	Rpap1	NP_796268.3	634	3	4.99
Tcf24	NP_001272354.1	75	0	0.00	Tyro3	NP_001277729.1	1294	4	3.26
Ppp1r42	NP_663730.1	641	3	4.93	Mga	XP_017174451.1	354	1	2.98
Cops5	NP_001264030.1	1794	3	1.76	Mapkbp1	NP_001342222.1	255	3	12.40
Cspp1	XP_036019142.1	461	1	2.29	Jmjd7	NP_001108109.1	165	1	6.39
Arfgef1	NP_001095900.1	1181	6	5.35	Pla2g4b	NP_663353.3	417	3	7.58
Cpa6	XP_011236694.1	314	2	6.71	Sptbn5	NP_001357867.1	752	2	2.80
Prex2	XP_006495504.1	717	2	2.94	Ehd4	NP_598599.2	534	1	1.97
A830018L16R	NP_001153841.1	72	1	14.64	Pla2g4e	NP_808513.2	444	2	4.75
Sulf1	XP_036020591.1	652	4	6.47	Pla2g4d	NP_001019308.1	348	1	3.03
Slco5a1	XP_030109841.1	216	6	29.28	Pla2g4f	NP_001350004.1	403	4	10.46
Prdm14	NP_001074678.2	621	5	8.49	Vps39	NP_849182.1	948	1	1.11
Ncoa2	XP_030107955.1	799	5	6.60	Tmem87a	NP_001103967.1	223	1	4.73
Tram1	NP_082449.1	284	0	0.00	Ganc	NP_766260.2	443	1	2.38
Lactb2	XP_006495553.1	375	6	16.86	Capn3	NP_031627.2	657	3	4.81
Xkr9	XP_006495610.1	450	6	14.05	Zfp106	XP_030104740.1	490	2	4.30
Eya1	XP_017170870.1	833	5	6.33	Snap23	NP_001171263.1	895	7	8.24
Msc	NP_034957.1	249	3	12.70	Lrrc57	NP_001153081.1	566	8	14.90
Trpa1	NP_001335217.1	1303	3	2.43	Haus2	NP_079751.1	280	5	18.82
Kcnb2	NP_001091998.1	874	2	2.41	Cdan1	NP_081167.2	298	7	24.76
Terf1	NP_033378.1	679	2	3.10	Ttbk2	NP_542966.2	1011	8	8.34
Sbspon	NP_001028460.1	99	2	21.29	Ubr1	XP_006499227.1	1342	7	5.50
4930444P10R	XP_006495645.1	0	0	#DIV/0!	Tmem62	NP_780494.1	44	0	0.00
Rpl7	NP_035421.2	1434	1	0.73	Ccndbp1	NP_034891.2	156	7	47.29
Rdh10	NP_598593.1	494	0	0.00	Epb42	NP_038541.1	232	4	18.17
Stau2	NP_001104742.1	525	2	4.01	Tgm5	NP_083075.1	492	0	0.00
Ube2w	NP_001257945.2	1423	2	1.48	Tgm7	NP_001153896.1	97	1	10.86
Eloc	XP_030098437.1	1450	3	2.18	Lcmt2	NP_808514.2	228	0	0.00
Tmem70	NP_080668.1	354	1	2.98	Adal	XP_011238139.1	609	1	1.73
Ly96	NP_058619.1	437	1	2.41	Zscan29	NP_001277748.1	201	0	0.00
Jph1	NP_065629.1	373	4	11.30	Tubgcp4	NP_700436.1	301	5	17.51
Gdap1	XP_036016287.1	794	3	3.98	Trp53bp1	XP_036018161.1	2073	2	1.02
Pi15	NP_444421.2	264	2	7.98	Map1a	NP_115769.1	1100	1	0.96
Crispld1	NP_113579.2	179	2	11.78	Ppip5k1	XP_030107639.1	432	3	7.32

Crisp4	NP_001333977.1	242	3	13.06	Ckmt1	NP_001341998.1	920	6	6.87
Defb18	NP_001034212.1	91	1	11.58	Strc	NP_536707.2	216	5	24.40
Defb41	NP_898947.1	107	3	29.55	Catsper2	XP_006499147.1	419	4	10.06
Tfap2d	NP_694794.1	242	5	21.77	Pdia3	NP_031978.2	2093	2	1.01
Tfap2b	XP_006495557.1	560	1	1.88	Ell3	NP_666085.2	284	1	3.71
Pkhd1	NP_694819.2	185	2	11.39	Serf2	NP_001277766.1	220	10	47.90
Il17a	NP_034682.1	1431	2	1.47	Serinc4	NP_001350026.1	44	5	119.76
Il17f	NP_665855.2	505	4	8.35	Hypk	NP_080594.2	368	5	14.32
Mcm3	NP_032589.1	1701	4	2.48	Mfap1b	NP_001075444.1	595	7	12.40
Paqr8	NP_001342051.1	265	5	19.88	Mfap1a	NP_080496.1	595	7	12.40
Efhc1	NP_082250.1	377	4	11.18	Wdr76	NP_001356114.1	410	4	10.28
Tram2	NP_803128.1	258	6	24.51	Frmd5	XP_017173229.1	401	6	15.77
Tmem14a	XP_006495644.1	214	2	9.85	Golm2	NP_001192298.1	170	2	12.40
Gsta3	NP_001070821.1	621	3	5.09	Mageb3	NP_032571.2	95	0	0.00
Khdc1a	NP_899145.2	69	2	30.55	Ctdspl2	NP_997615.1	613	0	0.00
Khdc1c	NP_001029076.1	43	1	24.51	Eif3j1	NP_653128.2	563	4	7.49
Khdc1b	NP_001106658.1	172	1	6.13	Spg11	NP_663506.2	376	4	11.21
Kcnq5	NP_001297406.1	965	3	3.28	Patl2	XP_017174713.1	515	4	8.19
Rims1	XP_036010351.1	1178	6	5.37	B2m	NP_033865.2	1682	6	3.76
Ppp1r14bl	NP_080022.2	274	1	3.85	Trim69	NP_536771.2	706	4	5.97
Ogfrl1	NP_001366012.1	122	5	43.19	Terb2	NP_083190.1	53	0	0.00
B3gat2	NP_742122.2	453	4	9.31	Rps12-ps10	XP_036018677.1	1285	0	0.00
Smap1	NP_082810.1	546	4	7.72	Sord	NP_666238.1	1156	0	0.00
Sdhaf4	NP_080779.2	189	1	5.58	Duox2	NP_001349684.1	453	6	13.96
Fam135a	XP_017177764.1	259	5	20.35	Duoxa2	NP_080053.1	242	2	8.71
Col9a1	NP_031766.3	452	5	11.66	Duoxa1	XP_017172410.1	246	2	8.57
Col19a1	XP_030101271.1	355	5	14.84	Duox1	NP_001092767.1	446	6	14.18
Lmbrd1	NP_080995.2	148	0	0.00	Shf	XP_036018261.1	248	2	8.50
Adgrb3	XP_036019108.1	627	0	0.00	Slc28a2	NP_001343461.1	328	4	12.85
4931408C20R	NP_001028936.2	0	0	#DIV/0!	Slc28a2b	NP_001078987.1	116	4	36.34
Gm597	NP_001013772.1	45	0	0.00	Gatm	NP_080237.1	499	7	14.78
Phf3	XP_030108618.1	573	8	14.71	AA467197	NP_001004174.1	152	2	13.87
Ptp4a1	XP_036018684.1	518	8	16.28	Slc30a4	NP_001277922.1	420	6	15.06
Lgsn	NP_705829.1	1259	5	4.19	Bloc1s6	NP_062762.1	203	3	15.57
Dnaaf6	NP_083338.1	133	0	0.00	Sqor	NP_067482.4	627	5	8.40
Khdrbs2	NP_573498.1	774	6	8.17	Sema6d	XP_030105194.1	495	0	0.00
Gm9839	NP_001186885.1	226	0	0.00	Slc24a5	NP_778199.2	413	6	15.31
Gm5415	NP_001157758.1	226	0	0.00	Myef2	XP_030104088.1	1336	8	6.31
Prim2	NP_032948.1	973	9	9.75	Ctxn2	NP_001156406.2	320	2	6.59
Rab23	NP_001153201.1	508	4	8.30	Slc12a1	XP_030104745.1	576	4	7.32
Bag2	NP_663367.1	494	9	19.20	Dut	NP_001153118.1	2336	2	0.90
Zfp451	NP_001346203.1	475	5	11.09	Fbn1	NP_032019.2	1382	8	6.10
Gm15455	NP_001155288.1	666	1	1.58	Cep152	XP_036018650.1	531	3	5.95
Bend6	NP_796209.2	73	4	57.75	Shc4	NP_950187.1	429	3	7.37
Dst	XP_017170110.1	1349	3	2.34	Eid1	NP_079889.2	218	3	14.50
Ccdc115	NP_081435.1	174	0	0.00	Secisbp2l	NP_808276.2	107	7	68.95
Imp4	NP_848716.2	918	0	0.00	Cops2	NP_034069.2	1437	3	2.20
Ptpn18	XP_036018686.1	937	0	0.00	Galk2	NP_780363.1	714	1	1.48
Prss39	NP_033381.1	234	2	9.01	Fam227b	XP_006500411.1	7	0	0.00
Cfc1	NP_031711.1	379	2	5.56	Fgf7	NP_032034.1	1043	2	2.02
Prss40	XP_006495920.1	105	2	20.07	Dtwd1	NP_001342512.1	105	8	80.30
Amer3	NP_998892.1	600	2	3.51	Atp8b4	NP_001346904.1	686	6	9.22
Arhgef4	NP_001355702.1	660	2	3.19	Slc27a2	NP_036108.2	1067	4	3.95
Fam168b	NP_778162.1	98	2	21.51	Hdc	XP_006498847.1	720	2	2.93
Plekhb2	NP_001344354.1	148	0	0.00	Gabpb1	XP_036014247.1	1080	1	0.98
Hs6st1	NP_056633.2	263	1	4.01	Usp8	NP_001239509.1	849	1	1.24
Uggt1	XP_006496125.1	515	2	4.09	Usp50	NP_083439.2	347	0	0.00
Neurl3	NP_700457.1	137	2	15.39	Trpm7	NP_001157797.1	781	2	2.70
Arid5a	NP_001165676.1	465	6	13.60	Sppl2a	NP_075709.2	313	4	13.47
Kansl3	NP_766240.1	176	0	0.00	Ap4e1	XP_006498611.1	835	3	3.79
Lman2l	NP_001013392.1	342	7	21.57	Blvra	XP_006498632.1	512	0	0.00
Cnnm4	NP_291048.2	569	5	9.26	Ncaph	NP_659067.2	1118	4	3.77
Cnnm3	NP_444416.2	362	6	17.47	Itpripl1	NP_001156999.1	75	7	98.36
Ankrd23	NP_705722.2	1196	7	6.17	1810024B03R	NP_941032.1	141	0	0.00
Ankrd39	NP_080517.1	959	9	9.89	Snrnp200	NP_796188.2	1321	5	3.99
Sema4c	XP_006495867.1	500	1	2.11	Ciao1	NP_079572.2	392	6	16.13

Fam178b	NP_001119518.1	58	9	163.54	Tmem127	NP_780354.1	145	6	43.61
Cox5b	NP_034072.2	840	2	2.51	Stard7	NP_647469.2	222	3	14.24
Actr1b	NP_666219.1	1512	5	3.49	Dusp2	NP_034220.2	848	2	2.49
Zap70	NP_001276695.1	1822	0	0.00	Astl	NP_001277932.1	315	3	10.04
Tmem131	XP_036008366.1	527	0	0.00	Adra2b	NP_033763.3	557	1	1.89
Vwa3b	NP_001357757.1	83	3	38.09	Gpat2	XP_006499195.3	296	1	3.56
Cnga3	NP_001268939.1	478	3	6.61	Fahd2a	NP_083905.1	305	8	27.64
Inpp4a	XP_006496074.1	283	0	0.00	Kcnip3	NP_001277934.1	1222	2	1.72
Coa5	NP_932123.3	190	2	11.09	Prom2	NP_835148.2	244	6	25.92
Unc50	NP_001343457.1	216	1	4.88	Zfp661	XP_030107968.1	357	6	17.71
Mgat4a	XP_017176450.1	349	1	3.02	Mrps5	NP_084239.1	1326	6	4.77
Cracdl	XP_036009588.1	164	0	0.00	Mal	NP_001164658.1	313	1	3.37
Tsga10	XP_030108520.1	206	3	15.35	Mall	NP_663507.1	168	5	31.37
Lipt1	NP_001033007.2	288	4	14.64	Nphp1	NP_001277942.1	554	7	13.32
Mitd1	NP_081189.1	182	6	34.74	Bub1	XP_036013667.1	1588	7	4.65
Mrpl30	NP_001343411.1	459	5	11.48	Acoxl	XP_006500336.1	469	14	31.46
Lyg2	NP_001028599.1	260	7	28.37	Bcl2l11	XP_006498677.1	1152	1	0.91
Lyg1	NP_081387.1	91	6	69.49	Spdye4c	XP_017173687.1	10	0	0.00
Txndc9	NP_742051.1	1025	11	11.31	Anapc1	NP_032595.2	1000	7	7.38
Eif5b	NP_938045.2	1353	7	5.45	Mertk	NP_032613.1	1243	6	5.09
Rev1	XP_006496219.1	791	1	1.33	Tmem87b	NP_001342557.1	221	7	33.38
Aff3	XP_030107416.1	431	6	14.67	Fbln7	NP_077199.2	397	7	18.58
Lonrf2	NP_001025049.2	823	6	7.68	Zc3h8	XP_006500010.1	595	5	8.86
Chst10	XP_006496427.1	326	3	9.70	Zc3h6	NP_848491.2	451	6	14.02
Nms	NP_001011684.3	429	0	0.00	Vinac1	NP_001357870.1	233	0	0.00
Pdcl3	NP_081126.2	353	4	11.94	Ttl	NP_081468.1	296	0	0.00
Npas2	NP_032745.2	629	9	15.08	Polr1b	NP_033112.2	2071	0	0.00
Rpl31	NP_444487.1	1072	3	2.95	Chchd5	NP_079671.1	76	1	13.87
Tbc1d8	NP_061245.3	708	7	10.42	Slc20a1	XP_036015794.1	751	1	1.40
Cnot11	NP_082319.1	214	1	4.92	Ckap2l	XP_006500221.1	436	3	7.25
Rnf149	NP_001028307.2	242	8	34.84	Il1a	NP_034684.2	773	3	4.09
Creg2	NP_733485.1	232	1	4.54	Il1b	NP_032387.1	1858	3	1.70
Rfx8	NP_001139132.1	406	0	0.00	F830045P16R	NP_001366373.1	18	0	0.00
Gm3646	NP_001170819.1	47	0	0.00	Sirpa	XP_036015585.1	682	2	3.09
Map4k4	XP_006496104.1	562	12	22.50	Pdyn	NP_061351.2	995	3	3.18
Il1r2	XP_017173211.1	610	9	15.55	Stk35	NP_001033724.2	527	1	2.00
Il1r1	XP_006495776.1	763	9	12.43	Tgm3	XP_011237739.1	264	0	0.00
Il1rl2	NP_001343407.1	204	6	31.00	Tgm6	NP_808394.1	202	3	15.65
Il1rl1	XP_036018391.1	667	7	11.06	Snrpb	XP_036015825.1	1564	3	2.02
Il18r1	XP_006495790.1	526	7	14.03	Tmc2	NP_619596.1	234	0	0.00
Il18rap	NP_034683.1	432	7	17.08	Nop56	NP_077155.2	1884	4	2.24
Slc9a4	NP_796058.1	415	5	12.70	Idh3b	NP_001349681.1	968	1	1.09
Slc9a2	NP_001028461.2	566	2	3.72	Ebf4	XP_030106297.1	676	1	1.56
Mfsd9	NP_766087.1	106	0	0.00	Cpxm1	NP_062670.2	335	1	3.15
Tmem182	NP_001074667.1	238	1	4.43	1700020A23R	NP_001156955.1	0	0	#DIV/0!
Pou3f3	NP_032926.2	584	0	0.00	Tmem239	NP_080029.1	4	0	0.00
Mrps9	NP_076003.3	847	2	2.49	Pced1a	XP_036018180.1	89	0	0.00
Gpr45	XP_017168482.1	362	3	8.73	Vps16	NP_085036.3	520	1	2.03
Tgfbrap1	XP_006496363.1	793	3	3.99	Ptpra	XP_036015592.1	718	8	11.74
AI597479	NP_598579.1	184	0	0.00	Mrps26	NP_997090.1	391	7	18.87
Fhl2	NP_001276462.1	1209	3	2.62	Oxt	NP_035155.1	829	6	7.63
Nck2	XP_030107914.1	699	2	3.02	Avp	NP_033862.1	852	6	7.42
Ecrg4	NP_077245.2	189	0	0.00	Ubox5	NP_542129.2	871	11	13.31
Uxs1	NP_080706.1	415	0	0.00	Fastkd5	NP_937819.3	185	2	11.39
Tpp2	NP_033444.1	903	3	3.50	Lzts3	XP_036017864.1	183	9	51.83
Mettl21c	XP_006496189.1	215	0	0.00	Ddrgk1	NP_084108.1	133	3	23.77
Gm8251	NP_001363913.1	35	0	0.00	Itpa	XP_017171220.1	991	11	11.70
Tex30	NP_001343226.1	359	1	2.94	Slc4a11	NP_001074631.1	424	7	17.40
Poglut2	NP_076134.1	141	3	22.42	4930402H24R	XP_011237775.1	55	1	19.16
Bivm	XP_006496061.3	54	1	19.52	Atrn	XP_006498669.1	796	7	9.27
Ercc5	NP_035859.2	1153	2	1.83	Gfra4	NP_001129535.1	213	6	29.69
Mettl21e	XP_036008140.1	138	0	0.00	Adam33	XP_006498639.2	384	3	8.23
Gulp1	XP_006496339.1	392	2	5.38	Siglec1	NP_035556.3	848	2	2.49
Col3a1	NP_034060.2	1141	4	3.69	Hspa12b	NP_082582.1	1693	10	6.23
Col5a2	NP_031763.2	778	4	5.42	1700037H04R	NP_080367.1	79	11	146.75
Wdr75	NP_082875.1	700	1	1.51	Spef1	NP_081917.1	163	9	58.19

Slc40a1	XP_017177198.1	616	2	3.42	Cenpb	NP_031708.2	424	7	17.40
Dnah7b	NP_001355646.1	590	1	1.79	Cdc25b	NP_001104545.1	1182	6	5.35
Dnah7c	XP_011236933.1	586	1	1.80	Ap5s1	NP_001277961.1	98	15	161.31
Slc39a10	XP_006495995.1	259	4	16.28	Mavs	NP_659137.1	815	4	5.17
Tmeff2	XP_030111510.1	464	3	6.81	Pank2	NP_705721.3	394	9	24.07
Cavin2	NP_620080.1	554	5	9.51	Rnf24	NP_848722.1	293	12	43.16
Nabp1	NP_082972.2	321	5	16.42	Smox	NP_001171305.1	644	9	14.73
Myo1b	NP_001277911.1	1324	2	1.59	Adra1d	NP_038488.4	840	7	8.78
Stat4	XP_006495878.1	826	3	3.83	Erv3	NP_001159678.1	46	11	252.02
Stat1	NP_001192242.1	2157	4	1.95	Prnp	NP_035300.1	1030	6	6.14
Gls	NP_001074550.1	736	3	4.30	Prn	NP_001265187.1	138	4	30.55
Nab1	XP_030107908.1	308	4	13.69	Prnd	NP_075530.1	199	4	21.18
Nemp2	NP_001136119.1	103	8	81.86	Rassf2	NP_001349795.1	469	8	17.98
Mfsd6	NP_598590.2	280	10	37.64	Slc23a2	NP_001342359.1	519	4	8.12
Inpp1	NP_032410.2	484	8	17.42	Tmem230	NP_081754.2	137	2	15.39
Hibch	NP_666220.1	955	11	12.14	Pcna	NP_035175.1	1851	1	0.57
1700019D03R	NP_001355203.1	64	8	131.74	Cds2	NP_619592.1	570	2	3.70
Mstn	NP_034964.1	1030	4	4.09	Prokr2	NP_659193.3	372	1	2.83
1700019A02R	XP_006496318.1	20	0	0.00	Gpcpd1	NP_083078.3	512	2	4.12
Pms1	NP_705784.1	663	8	12.72	Shld1	XP_036018512.1	31	2	67.99
Ormdl1	NP_663492.3	243	9	39.03	Chgb	NP_031720.1	1274	1	0.83
Osgepl1	NP_001272768.1	531	9	17.86	Trmt6	NP_780322.2	772	1	1.37
Asnsd1	NP_001277913.1	792	9	11.98	Mcm8	NP_001277983.1	1084	4	3.89
Dnah7a	NP_001238999.1	641	1	1.64	Crls1	NP_001019556.1	608	5	8.67
Stk17b	NP_598571.2	994	1	1.06	Lrrn4	NP_796277.2	526	1	2.00
Hecw2	NP_001001883.1	1046	2	2.02	Fermt1	NP_932146.2	549	3	5.76
Ccdc150	NP_084301.2	6	0	0.00	Bmp2	NP_031579.2	1127	2	1.87
Gtf3c3	NP_001028366.1	410	4	10.28	Hao1	NP_034533.1	1122	5	4.70
Pgap1	NP_001156786.1	368	0	0.00	Tmx4	NP_083424.1	836	3	3.78
Ankrd44	NP_001074902.2	1611	4	2.62	Plcb1	NP_001139302.1	1298	6	4.87
Sf3b1	NP_112456.2	1317	3	2.40	Plcb4	XP_030104392.1	1022	4	4.12
Coq10b	NP_001034799.1	367	0	0.00	Lamp5	NP_001343477.1	768	4	5.49
Hspd1	NP_001343441.1	2491	6	2.54	Pak5	XP_006499573.1	1664	7	4.43
Hspe1	NP_032329.1	1960	2	1.08	Ankef1	NP_783598.1	1063	7	6.94
Mob4	NP_079559.2	767	6	8.24	Snap25	XP_017172249.1	2194	4	1.92
Rftn2	NP_082989.1	94	7	78.48	Mkks	NP_001273910.1	1577	0	0.00
Mars2	NP_780648.1	708	4	5.95	Slx4ip	XP_011238116.1	100	5	52.70
Boll	NP_001355696.1	1420	6	4.45	Jag1	NP_038850.1	1775	4	2.37
Plcl1	NP_001108135.1	596	3	5.30	Btbd3	NP_663509.2	328	3	9.64
Hsfy2	NP_081937.1	874	8	9.65	Sptlc3	NP_001343436.1	601	3	5.26
Satb2	NP_001345509.1	680	10	15.50	Ism1	NP_001263418.1	196	1	5.38
1700066M21F	NP_082822.1	174	9	54.51	Tasp1	XP_017174800.1	269	1	3.92
Tyw5	NP_001289891.1	461	12	27.43	Esf1	NP_001074559.1	1047	2	2.01
Maip1	NP_001074650.1	78	9	121.60	Ndufaf5	NP_081369.2	732	0	0.00
Spats2l	XP_036008969.1	43	0	0.00	Sel1l2	NP_001028468.1	491	0	0.00
Kctd18	XP_036008217.1	200	10	52.70	Macrod2	XP_006500309.1	505	1	2.09
Sgo2a	NP_001171338.1	450	0	0.00	Flrt3	NP_001165631.1	732	4	5.76
Aox1	NP_033806.2	715	11	16.21	Kif16b	XP_006498882.1	1342	4	3.14
Aox3	NP_076106.2	698	3	4.53	Snrpb2	NP_067310.1	985	5	5.35
Aox4	NP_076120.2	242	8	34.84	Otor	NP_065620.1	150	2	14.05
Aox2	NP_001008419.1	380	8	22.19	Pcsk2	NP_032818.1	1420	3	2.23
Bzw1	NP_080100.1	832	12	15.20	Bfsp1	NP_033881.2	206	0	0.00
Clk1	NP_001036099.1	1030	7	7.16	Dstn	NP_062745.1	1562	2	1.35
Ppil3	XP_030098742.1	1250	9	7.59	Rrbp1	XP_006500504.1	473	2	4.46
Nif3l1	NP_075364.2	248	11	46.75	Banf2	NP_001038215.1	261	2	8.08
Orc2	XP_030108010.1	1191	0	0.00	Snx5	NP_077187.1	531	7	13.89
Fam126b	XP_017175146.1	150	10	70.26	Mgme1	NP_001342617.1	209	0	0.00
Ndufb3	NP_079873.1	536	3	5.90	Ovol2	NP_081200.2	281	9	33.75
Flacc1	NP_001333985.1	83	9	114.28	Pet117	NP_001158285.1	57	0	0.00
Cflar	XP_030100848.1	1058	4	3.98	Kat14	NP_852082.2	391	7	18.87
Casp8	NP_001264855.1	1922	5	2.74	Dzank1	XP_030107026.1	270	6	23.42
Trak2	XP_036009532.1	346	7	21.32	Polr3f	NP_084039.2	567	8	14.87
Stradb	NP_766244.4	320	5	16.47	Rbbp9	NP_056569.2	218	10	48.34
C2cd6	NP_080876.1	120	6	52.70	Sec23b	NP_001342309.1	1049	8	8.04
Tmem237	XP_006496159.1	189	4	22.30	Smim26	NP_001028469.1	152	0	0.00
Mpp4	NP_001158154.1	544	1	1.94	Dtd1	NP_079590.1	305	7	24.19

Als2	NP_001153420.2	957	5	5.51	Scp2d1	NP_079766.3	330	2	6.39
Cdk15	XP_006496111.1	1977	0	0.00	Slc24a3	NP_001343426.1	640	6	9.88
Fzd7	NP_032083.3	705	1	1.49	Rin2	XP_006500332.1	328	7	22.49
Gm973	NP_001013793.2	577	0	0.00	Naa20	NP_080701.1	591	5	8.92
Sumo1	NP_033486.1	2610	2	0.81	Crnkl1	NP_080096.1	779	5	6.76
Nop58	NP_061356.2	1696	2	1.24	Cfap61	XP_030108105.1	166	5	31.74
Bmpr2	NP_031587.1	1438	2	1.47	Insm1	NP_058585.2	478	4	8.82
Fam117b	NP_001032814.1	188	2	11.21	Ralgapa2	NP_001028520.2	331	3	9.55
Ica1l	XP_036009500.1	331	3	9.55	Kiz	NP_001028470.1	182	2	11.58
Wdr12	XP_017177350.1	1327	7	5.56	Xrn2	NP_036047.2	1224	5	4.31
Carf	XP_030109944.1	145	4	29.07	Nkx2-4	XP_006499364.1	421	5	12.52
Nbeal1	XP_036021352.1	411	2	5.13	Nkx2-2	XP_036015222.1	862	4	4.89
Cyp20a1	NP_084289.1	328	4	12.85	Pax1	XP_006498974.1	795	3	3.98
Abi2	NP_001185499.1	753	3	4.20	Foxa2	NP_001277994.1	1642	4	2.57
Raph1	XP_017168365.1	400	1	2.63	Sstr4	NP_033245.2	379	2	5.56
Gm11578	XP_036011065.1	320	0	0.00	Thbd	NP_033404.1	779	2	2.71
Cd28	NP_031668.3	1220	2	1.73	Cd93	NP_034870.1	791	2	2.66
Ctla4	NP_033973.2	1097	2	1.92	Nxt1	NP_062735.4	584	7	12.63
Icos	NP_059508.2	372	2	5.67	Gzf1	NP_001342618.1	314	9	30.21
Pard3b	XP_006496361.1	861	2	2.45	Napb	NP_062606.1	896	1	1.18
Nrp2	NP_001070871.1	377	2	5.59	Cstl1	NP_808323.1	89	8	94.73
Ino80d	NP_001108081.1	307	1	3.43	Cst11	NP_084335.1	246	11	47.13
Ndufs1	XP_030109554.1	1109	4	3.80	Cstdc1	NP_084411.1	50	6	126.47
Eef1b2	NP_061266.2	1074	1	0.98	Cst12	NP_081330.2	178	4	23.68
Gpr1	NP_001343974.1	400	2	5.27	Cst8	NP_034108.1	386	9	24.57
Zdbf2	XP_036009742.1	128	2	16.47	Cst13	NP_081300.1	194	6	32.59
Adam23	XP_006496033.1	513	6	12.33	Cst9	NP_034109.1	138	9	68.73
Dytn	NP_001075127.1	463	4	9.10	Cstdc2	NP_084236.1	203	3	15.57
Mdh1b	XP_036010132.1	866	2	2.43	Cst3	NP_034106.2	720	4	5.86
Fastkd2	XP_006496376.2	430	7	17.16	Cst10	NP_067380.1	205	2	10.28
4933402D24R	NP_001243087.1	0	0	#DIV/0!	Syndig1	NP_001350024.1	467	0	0.00
Klf7	XP_030099629.1	613	6	10.32	Zfp120	NP_851783.2	361	0	0.00
Creb1	NP_034082.1	2426	3	1.30	Gm14139	NP_001139335.1	164	0	0.00
Mettl21a	NP_080240.1	181	8	46.58	Zfp937	NP_001135883.1	126	0	0.00
Ccnyl1	XP_006496016.1	244	6	25.92	Gm14124	NP_001135882.1	175	0	0.00
Fzd5	NP_001036124.1	698	6	9.06	3300002I08Ri	XP_036018428.1	188	0	0.00
Plekhm3	XP_006496045.1	187	11	61.99	Gm10130	NP_001361605.1	130	0	0.00
Akr1cl	NP_081858.2	247	0	0.00	Zfp442	NP_001297574.1	126	0	0.00
Cryge	NP_031803.3	82	4	51.41	Zfp345	NP_001030072.2	130	0	0.00
Crygd	NP_031802.2	123	4	34.27	Cst7	NP_034107.2	410	3	7.71
Crygc	NP_031801.1	124	2	17.00	Apmap	NP_082253.1	752	1	1.40
Crygb	NP_658906.1	202	5	26.09	Acss1	NP_542142.1	735	1	1.43
Cryga	NP_031800.1	97	3	32.59	Vsx1	NP_473409.1	370	1	2.85
D630023F18R	NP_001272811.1	38	0	0.00	Entpd6	NP_001341997.1	358	4	11.78
Idh1	NP_034627.3	1363	7	5.41	Pygb	NP_722476.1	966	7	7.64
Pikfyve	XP_006495839.1	1221	4	3.45	Abhd12	NP_077785.2	353	4	11.94
Pth2r	XP_011236775.1	404	3	7.83	Gins1	NP_081290.1	815	5	6.47
Crygf	NP_081286.1	77	4	54.75	Ninl	XP_006500472.1	317	4	13.30
Map2	XP_036018500.1	1641	3	1.93	Nanp	NP_080362.1	362	0	0.00
Unc80	NP_001355753.1	835	4	5.05	Gm14151	NP_001091446.1	97	0	0.00
Rpe	NP_079959.2	981	2	2.15	Gm14147	NP_001365536.1	97	0	0.00
Kansl1l	XP_006496312.1	99	4	42.58	4921509C19R	XP_036018236.1	151	0	0.00
Acadl	NP_031407.2	702	4	6.01	Nsfl1c	NP_938085.1	543	0	0.00
Myl1	XP_036018509.1	1305	4	3.23	Fkbp1a	NP_032045.1	1530	0	0.00
Lancl1	NP_067270.1	220	3	14.37	Sdcbp2	XP_036017333.1	116	0	0.00
Cps1	NP_001074278.1	1566	3	2.02	Snph	XP_030107031.1	871	0	0.00
Erbb4	NP_034284.1	2130	6	2.97	Rad21l	XP_006500107.1	755	2	2.79
Ikzf2	XP_006496024.1	464	4	9.09	Tmem74b	XP_036013317.1	47	0	0.00
Spag16	NP_083436.2	509	3	6.21	Psmf1	XP_006499369.1	407	0	0.00
Vwc2l	XP_006496126.1	307	6	20.60	Rspo4	NP_001035779.1	568	0	0.00
Bard1	XP_006495694.1	3277	8	2.57	Angpt4	NP_033771.1	492	0	0.00
Abca12	NP_780419.2	446	4	9.45	Fam110a	XP_006500331.2	265	0	0.00
Atic	NP_080471.2	1134	2	1.86	Slc52a3	NP_081448.2	262	0	0.00
Fn1	NP_001263342.1	3439	5	1.53	Scrt2	NP_001153882.1	378	0	0.00
Gm5528	XP_036011071.1	899	2	2.34	Srxn1	XP_036018567.1	642	1	1.64
Mreg	NP_001005423.1	238	2	8.86	Tcf15	XP_006499163.1	374	0	0.00

Pecr	NP_076012.3	701	2	3.01	Csnk2a1	XP_030102934.1	1945	3	1.63
Tmem169	NP_780773.1	233	3	13.57	Tbc1d20	NP_077158.1	310	3	10.20
Xrcc5	XP_006495955.1	1305	4	3.23	Rbck1	XP_030106804.1	757	2	2.78
Marchf4	NP_001038998.1	519	0	0.00	Trib3	NP_780302.2	1562	4	2.70
Smarcal1	NP_061287.2	1130	2	1.87	Nrsn2	XP_006499374.1	621	2	3.39
Ankar	XP_006496123.1	741	0	0.00	Sox12	NP_035568.1	245	2	8.60
Rpl37a	NP_033110.1	1208	2	1.74	Zcchc3	NP_780335.1	406	0	0.00
Igfbp2	NP_032368.2	755	3	4.19	6820408C15R	XP_036017356.1	111	0	0.00
Igfbp5	NP_034648.2	888	4	4.75	Defb23	NP_001033022.1	66	8	127.75
Tnp1	NP_033433.1	742	1	1.42	Defb20	NP_795924.2	55	3	57.49
Tns1	NP_001276824.1	1413	4	2.98	Defb22	NP_001002791.1	123	3	25.70
Rufy4	XP_006496192.1	169	9	56.12	Defb26	NP_001034209.1	32	2	65.87
Cxcr2	XP_006495701.1	942	8	8.95	Defb28	NP_001032591.1	31	2	67.99
Cxcr1	XP_030109560.1	762	8	11.06	Defb29	NP_001001444.1	97	8	86.92
Arpc2	NP_083987.1	845	8	9.98	Defb21	NP_001366406.1	100	2	21.08
Gpbar1	NP_778150.1	339	7	21.76	Defb19	NP_660139.1	48	5	109.78
Aamp	XP_036020351.1	439	12	28.81	Defb45	NP_001032841.1	32	3	98.80
Pnkd	XP_006496230.1	534	12	23.68	Defb36	NP_001032324.1	106	3	29.83
Tmbim1	NP_081430.3	440	15	35.93	Defb25	NP_001034211.1	80	3	39.52
Catip	XP_011236823.1	147	4	28.68	Rem1	NP_033073.1	3380	3	0.94
Slc11a1	XP_036018625.1	1274	17	14.06	H13	XP_006498846.1	456	4	9.24
Ctdsp1	XP_036020359.1	601	10	17.54	Mcts2	NP_079819.1	494	1	2.13
Vil1	NP_033535.2	690	11	16.80	Id1	NP_001342042.1	554	1	1.90
Usp37	NP_001297591.1	573	8	14.71	Cox4i2	NP_444321.1	581	7	12.70
Cnot9	NP_067358.1	467	10	22.57	Bcl2l1	NP_001276668.1	1986	6	3.18
Plcd4	XP_006495846.1	586	9	16.19	Tpx2	NP_001135447.1	833	1	1.27
Zfp142	XP_030099409.1	257	7	28.71	Mylk2	NP_001074513.2	805	8	10.47
Bcs1l	NP_080060.1	1008	3	3.14	Foxs1	NP_034356.1	534	6	11.84
Rnf25	NP_001292159.1	483	3	6.55	Dusp15	XP_011237861.1	749	8	11.26
Stk36	NP_778196.2	385	12	32.85	Ttll9	XP_030108012.1	396	4	10.65
Ttll4	XP_006496287.1	286	2	7.37	Pdrg1	XP_030107898.1	262	1	4.02
Cyp27a1	NP_077226.2	805	1	1.31	Xkr7	NP_001011732.1	198	8	42.58
Prkag3	NP_714966.1	1275	8	6.61	Ccm2l	NP_001359292.1	181	7	40.76
Wnt6	NP_033552.2	671	4	6.28	Hck	NP_034537.2	1688	7	4.37
Wnt10a	NP_033544.1	602	5	8.75	Tm9sf4	XP_006500539.1	287	8	29.38
Cdk5r2	NP_034002.2	1126	6	5.62	Tspyl3	NP_941019.2	515	6	12.28
Fev	NP_694751.1	411	3	7.69	Plagl2	NP_061277.2	578	7	12.76
Cryba2	NP_067516.1	268	4	15.73	Pofut1	NP_536711.3	333	10	31.65
Cfap65	XP_006496056.1	135	7	54.65	Kif3b	NP_032470.3	1098	14	13.44
Ihh	NP_034674.2	1049	6	6.03	Asxl1	NP_001035028.1	463	12	27.31
Nhej1	NP_083618.3	287	4	14.69	Nol4l	XP_006499842.2	175	12	72.27
Slc23a3	XP_006496543.1	215	3	14.71	Commd7	NP_598611.2	408	9	23.25
Cnppd1	NP_001343193.1	509	3	6.21	Dnmt3b	XP_006498745.1	1271	4	3.32
Retreg2	NP_739561.2	130	2	16.21	Mapre1	XP_036014029.1	1126	11	10.30
Zfand2b	NP_081122.2	292	0	0.00	Sun5	XP_006500435.2	547	12	23.12
Abcb6	NP_076221.1	625	2	3.37	Bpifb2	XP_017174674.1	218	7	33.84
Atg9a	XP_030110127.1	678	3	4.66	Bpifb6	XP_017173296.1	151	9	62.82
Ankzf1	XP_006496570.1	296	0	0.00	Bpifb3	NP_001366402.1	137	10	76.93
Glb1l	XP_011237015.1	162	3	19.52	Bpifb4	NP_001030047.2	142	11	81.64
Stk16	NP_035624.3	364	0	0.00	Bpifa6	NP_001074280.1	83	11	139.67
Tuba4a	NP_033473.1	1770	2	1.19	Bpifa2	NP_032979.1	181	6	34.94
A630095N17R	NP_001230019.1	0	0	#DIV/0!	Bpifa3	NP_082804.1	301	6	21.01
Dnajb2	XP_006496574.1	655	2	3.22	Bpifa1	NP_035256.2	258	6	24.51
Ptprn	XP_036018688.1	1301	5	4.05	Bpifa5	NP_080266.2	103	10	102.32
Resp18	NP_033075.1	893	1	1.18	Bpifb1	NP_700467.2	241	8	34.98
Dnpep	NP_001104301.1	422	5	12.49	Bpifb5	NP_659139.2	117	13	117.10
Des	NP_034173.1	685	1	1.54	Bpifb9a	NP_780376.2	90	8	93.68
Speg	XP_011236945.1	1394	9	6.80	Bpifb9b	NP_001020745.1	124	7	59.49
Gmppa	NP_001344100.1	453	6	13.96	Cdk5rap1	NP_080152.1	593	8	14.22
Asic4	XP_036020902.1	433	5	12.17	Snta1	NP_033254.2	519	5	10.15
Chpf	NP_001001566.1	257	5	20.50	Cbfa2t2	NP_766448.1	285	1	3.70
Tmem198	XP_036021589.1	150	0	0.00	Necab3	XP_011238015.1	110	1	9.58
Obsl1	NP_849215.4	570	1	1.85	1700003F12R	XP_017174794.1	54	0	0.00
Inha	NP_034694.3	605	8	13.94	Actl10	NP_001165111.1	1200	4	3.51
Stk11ip	NP_082162.3	152	0	0.00	E2f1	NP_031917.1	1225	0	0.00
Slc4a3	NP_001344078.1	491	7	15.03	Pxmp4	NP_067509.1	232	2	9.09

Epha4	NP_031962.2	1703	6	3.71	Zfp341	NP_955008.2	156	2	13.51
Pax3	NP_001152992.1	1258	3	2.51	Chmp4b	NP_083638.1	683	4	6.17
Sgpp2	XP_030111230.1	275	2	7.66	Raly	XP_036015619.1	1581	6	4.00
Farsb	NP_035941.2	1067	6	5.93	Eif2s2	NP_080306.1	1357	7	5.44
Mogat1	XP_017177770.1	520	2	4.05	a	XP_011237991.1	245	5	21.51
Utp14b	NP_001129698.1	663	3	4.77	Ahcy	NP_057870.3	1255	6	5.04
Acsl3	NP_001028778.2	1141	3	2.77	Itch	NP_032421.2	1653	8	5.10
Kcne4	NP_067317.1	177	1	5.95	Dynlrb1	NP_080223.2	599	1	1.76
Scg2	NP_001297609.1	1223	1	0.86	Map1lc3a	NP_080011.1	1095	6	5.77
Ap1s3	NP_898848.1	380	4	11.09	Pigu	NP_001004721.1	156	10	67.56
Wdfy1	NP_001104749.1	836	1	1.26	Trp53inp2	NP_835212.1	173	1	6.09
Mrpl44	NP_001074679.1	2261	2	0.93	Ncoa6	NP_001229487.1	573	5	9.20
Serpine2	XP_006496522.1	1002	3	3.16	Ggt7	XP_006499123.1	600	8	14.05
Fam124b	NP_775601.1	137	2	15.39	Acss2	NP_062785.2	857	4	4.92
Cul3	NP_057925.1	2544	2	0.83	Gss	XP_006498844.1	445	6	14.21
Dock10	XP_030108464.1	544	2	3.87	Myh7b	NP_001078847.1	1389	5	3.79
Nyap2	XP_006496551.1	341	5	15.45	Trpc4ap	NP_062802.2	288	4	14.64
Irs1	NP_034700.2	1421	5	3.71	Edem2	NP_663512.2	321	11	36.11
Rhbdd1	NP_084053.3	296	4	14.24	Procr	NP_035301.2	445	4	9.47
Col4a4	NP_031761.1	538	4	7.84	Mmp24	NP_034938.3	555	2	3.80
Col4a3	NP_031760.2	514	1	2.05	Eif6	NP_034709.1	1154	6	5.48
Mff	XP_036010058.1	408	1	2.58	Fam83c	NP_082064.3	59	9	160.76
Tm4sf20	NP_079729.1	120	0	0.00	Uqcc1	NP_001342372.1	424	7	17.40
Agfg1	XP_006496493.1	1177	5	4.48	Gdf5	NP_032135.2	734	2	2.87
Slc19a3	XP_036010332.1	285	1	3.70	Cep250	NP_001123472.1	344	6	18.38
Krtap28-13	NP_082054.2	0	0	#DIV/0!	6430550D23F	XP_030107611.1	7	0	0.00
Ccl20	NP_058656.1	912	0	0.00	Ergic3	NP_001343342.1	713	5	7.39
Daw1	NP_001359172.1	757	0	0.00	Fer1l4	XP_006500370.1	145	0	0.00
Sphkap	NP_766018.3	947	1	1.11	Spag4	NP_631890.3	430	2	4.90
Pid1	XP_036010586.1	262	1	4.02	Cpne1	NP_733467.1	324	4	13.01
Dner	NP_690879.1	1089	4	3.87	Rbm12	NP_083673.3	307	2	6.87
Trip12	XP_036017235.1	2116	4	1.99	Nfs1	XP_006498960.1	642	1	1.64
Fbxo36	NP_079662.1	187	2	11.27	Romo1	NP_080222.1	584	1	1.80
Slc16a14	NP_082197.1	204	3	15.50	Rbm39	NP_001349693.1	976	1	1.08
A530032D15R	NP_998780.2	225	4	18.74	Phf20	NP_766262.2	809	2	2.61
Gm7609	XP_006496584.1	70	3	45.17	Scand1	NP_064651.2	475	2	4.44
C130026I21Ri	XP_036008471.1	255	6	24.80	Cnbd2	NP_081861.2	804	1	1.31
Gm7592	XP_006530041.1	70	3	45.17	Epb41l1	XP_006498771.1	537	1	1.96
Sp110	XP_011246203.1	567	3	5.58	Aar2	NP_001158290.1	385	0	0.00
Sp140	XP_030111256.1	528	4	7.98	Dlgap4	XP_036017439.1	396	3	7.98
Sp100	NP_001359160.1	854	4	4.94	Myl9	NP_742116.1	1625	3	1.95
A630001G21R	XP_017176680.1	260	0	0.00	Tgif2	NP_775572.1	424	0	0.00
Cab39	NP_001341976.1	775	3	4.08	Rab5if	NP_080400.1	189	2	11.15
Itm2c	NP_071862.2	300	1	3.51	Sla2	NP_084259.1	235	4	17.94
Gpr55	XP_030109565.1	429	3	7.37	Ndrg3	XP_030107594.1	397	3	7.96
Spata3	XP_011246374.3	125	3	25.29	Dsn1	NP_080129.2	588	1	1.79
2810459M11F	NP_001138464.1	185	3	17.09	Soga1	XP_017174512.3	127	0	0.00
Psmd1	NP_081633.1	1471	4	2.87	Tldc2	XP_006499901.1	123	0	0.00
Htr2b	NP_032337.2	471	3	6.71	Samhd1	NP_061339.4	746	2	2.83
Armc9	XP_006530020.1	190	3	16.64	Rbl1	NP_035379.2	1101	2	1.91
B3gnt7	NP_660257.2	266	1	3.96	Mroh8	XP_017174648.1	39	1	27.02
Ncl	NP_035010.3	2050	2	1.03	Rpn2	NP_001342092.1	673	1	1.57
Nmur1	NP_001306156.1	560	2	3.76	Ghrh	XP_006498836.1	454	1	2.32
Tex44	NP_082242.1	129	0	0.00	Manbal	NP_081244.2	84	0	0.00
Ptma	NP_032998.1	623	2	3.38	Src	XP_030104859.1	3901	2	0.54
Pde6d	NP_032827.1	493	2	4.28	Blcap	NP_001342355.1	304	3	10.40
Cops7b	XP_006529688.1	494	2	4.27	Nnat	XP_036015262.1	580	4	7.27
Nppc	NP_035063.1	273	2	7.72	Ctnnbl1	NP_079956.3	708	5	7.44
Dis3l2	XP_030108343.1	901	2	2.34	Vstm2l	NP_941029.2	667	4	6.32
Alppl2	NP_031459.3	500	3	6.32	Tti1	NP_083558.1	371	2	5.68
Alpi	NP_001074551.1	2702	5	1.95	Rprd1b	NP_001278063.1	336	2	6.27
Akp3	NP_031458.2	481	4	8.76	Tgm2	NP_033399.1	1055	2	2.00
Ecel1	XP_017170387.1	363	8	23.23	D630003M21	NP_001124493.1	199	0	0.00
Prss56	XP_006529921.1	224	2	9.41	Bpi	XP_017174544.1	351	1	3.00
Chrnd	NP_067611.2	602	6	10.50	Lbp	NP_032515.2	343	8	24.58
Chrng	NP_033734.3	632	6	10.01	Ralgapb	XP_006499422.1	219	6	28.87

Eif4e2	XP_036021474.1	847	10	12.44	Adig	NP_663610.1	179	7	41.21
Efhd1	NP_083165.1	408	0	0.00	Arhgap40	NP_001138487.1	170	0	0.00
Gigyf2	NP_666224.3	568	3	5.57	Slc32a1	NP_033534.2	1446	4	2.92
Kcnj13	NP_001103697.1	255	2	8.27	Actr5	NP_780628.3	406	7	18.17
Snorc	XP_036009654.1	45	0	0.00	Ppp1r16b	NP_001153134.1	963	7	7.66
Ngef	NP_001104784.1	857	3	3.69	Fam83d	NP_082251.2	381	6	16.60
Neu2	NP_001153635.1	297	6	21.29	Dhx35	NP_001359442.1	1051	8	8.02
Inpp5d	XP_006529212.1	1053	5	5.00	Gm826	XP_006499857.1	41	0	0.00
Atg16l1	NP_001192320.1	622	5	8.47	Mafb	NP_034788.1	604	1	1.74
Sag	XP_030108187.1	1317	6	4.80	Top1	NP_033434.2	1438	3	2.20
Dgkd	XP_006529535.1	258	5	20.42	Plcg1	NP_067255.2	1052	4	4.01
Usp40	NP_001028463.1	771	5	6.83	Zhx3	NP_796237.2	223	3	14.18
Ugt1a10	NP_964003.2	641	2	3.29	Lpin3	NP_075021.1	618	1	1.71
Ugt1a9	NP_964006.2	622	1	1.69	Emilin3	NP_001278074.1	8	0	0.00
Ugt1a8	NP_001365304.1	361	2	5.84	Chd6	XP_011238078.1	1341	3	2.36
Ugt1a7c	NP_964004.1	454	3	6.96	Ptprt	XP_036015600.1	1244	6	5.08
Ugt1a6b	NP_958812.3	768	9	12.35	Srsf6	NP_080775.3	1430	6	4.42
Ugt1a6a	NP_659545.2	452	1	2.33	L3mbtl1	XP_030107034.1	584	5	9.02
Ugt1a5	NP_964005.2	779	4	5.41	Sgk2	XP_006499721.1	1357	5	3.88
Ugt1a2	NP_038729.1	315	3	10.04	Ift52	NP_001343451.1	413	4	10.21
Dnajb3	NP_032325.2	615	5	8.57	Mybl2	XP_006498936.1	2913	4	1.45
Ugt1a1	NP_964007.2	903	4	4.67	Gtsf1l	NP_080906.1	127	0	0.00
Mroh2a	XP_006529103.1	63	3	50.19	Tox2	XP_006499673.1	675	9	14.05
Hjurp	XP_006529784.1	537	4	7.85	Jph2	NP_067541.1	536	3	5.90
Trpm8	NP_599013.1	457	2	4.61	Oser1	NP_079975.2	154	0	0.00
Spp2	XP_006529981.1	766	8	11.01	Gdap1l1	NP_659140.2	578	3	5.47
Glrp1	NP_032158.2	12	0	0.00	Fitm2	NP_775573.1	199	3	15.89
Arl4c	NP_796279.2	1562	8	5.40	R3hdml	NP_001092801.1	148	3	21.36
Sh3bp4	XP_030099747.1	262	2	8.05	Hnf4a	XP_006498851.1	1736	4	2.43
Agap1	NP_835220.1	590	7	12.50	Ttpal	NP_859423.2	250	0	0.00
Gbx2	NP_034392.1	644	1	1.64	Serinc3	NP_036162.3	279	2	7.55
Asb18	NP_001342063.1	1280	4	3.29	Pkig	XP_030104342.1	226	4	18.65
Iqca	NP_083398.2	594	2	3.55	Ada	NP_001258981.1	1182	2	1.78
Ackr3	XP_036012962.1	713	3	4.43	Ccn5	NP_058569.2	393	0	0.00
Cops8	NP_598566.3	803	3	3.94	Kcnk15	NP_001025463.1	195	3	16.21
Col6a3	XP_030101311.1	763	2	2.76	Rims4	NP_001363871.1	508	4	8.30
Mlph	NP_443748.2	351	2	6.01	Ywhab	NP_061223.2	3539	4	1.19
Prlh	NP_001095117.1	185	2	11.39	Pabpc1l	NP_001107551.1	1063	2	1.98
Rab17	NP_001153197.2	1075	6	5.88	Tomm34	NP_001278084.1	902	1	1.17
Lrrfip1	XP_006529272.1	450	4	9.37	Stk4	NP_067395.1	756	5	6.97
Rbm44	XP_006529765.1	68	2	31.00	Kcns1	NP_032461.2	733	5	7.19
Ramp1	NP_058590.1	315	4	13.38	Wfdc5	NP_663344.2	158	5	33.35
Ube2f	XP_017177752.1	612	3	5.17	Wfdc12	NP_619625.1	66	3	47.90
Scly	XP_006529807.1	549	1	1.92	Wfdc15a	NP_899094.1	13	0	0.00
Espnl	NP_001028464.1	1041	5	5.06	Wfdc15b	NP_001039019.1	116	5	45.43
Klhl30	NP_081827.1	363	1	2.90	Svs2	NP_059086.3	198	7	37.26
Erfe	XP_006529547.1	138	0	0.00	Svs3b	NP_775553.1	43	1	24.51
Ilkap	XP_030098247.1	1114	2	1.89	Svs4	NP_033326.1	112	1	9.41
Hes6	NP_001347828.1	537	2	3.93	Svs3a	NP_067338.2	123	2	17.14
Per2	XP_006529312.1	324	1	3.25	Svs6	NP_038707.2	119	2	17.71
Traf3ip1	XP_036009757.1	371	3	8.52	Svs5	NP_033327.1	121	1	8.71
Asb1	NP_001034215.1	1299	5	4.06	Slpi	XP_036015798.1	716	8	11.78
Twist2	NP_031881.1	791	1	1.33	Matn4	NP_038620.2	591	2	3.57
Hdac4	XP_030108410.1	2049	3	1.54	Rbpjl	NP_033062.1	448	2	4.70
Ndufa10	NP_077159.1	657	0	0.00	Sdc4	XP_036016111.1	704	1	1.50
Olfr1416	NP_667249.1	52	0	0.00	Sys1	NP_079851.2	294	9	32.26
Olfr1415	NP_001011525.1	21	0	0.00	Trp53tg5	XP_006500323.3	46	8	183.29
Olfr1414	NP_667250.1	43	0	0.00	Dbndd2	NP_001041692.1	107	2	19.70
Olfr1413	NP_667248.1	41	0	0.00	Pigt	XP_006500484.3	389	2	5.42
Olfr1412	NP_666389.1	75	0	0.00	Wfdc2	NP_080599.1	348	12	36.34
Olfr1411	NP_666701.1	36	0	0.00	Spint3	NP_001170872.1	61	7	120.94
Olfr1410	NP_666702.1	38	0	0.00	Wfdc6a	XP_011237710.1	354	8	23.82
Olfr12	NP_996779.1	16	0	0.00	Eppin	NP_083601.1	550	12	22.99
Cops9	NP_001156897.1	374	0	0.00	Wfdc8	NP_001263360.1	318	12	39.77
Otos	XP_017176304.1	166	0	0.00	Wfdc6b	XP_006499938.1	340	10	31.00
Gpc1	NP_057905.1	567	0	0.00	Wfdc16	NP_001012741.2	43	6	147.06

Ankmy1	XP_006529654.1	77	5	68.44	Wfdc9	XP_006500047.3	57	10	184.89
Dusp28	NP_780327.1	405	3	7.81	Wfdc10	NP_001034590.1	112	8	75.28
Rnpepl1	NP_852070.3	411	7	17.95	Wfdc11	NP_001155278.1	40	5	131.74
Capn10	NP_035926.2	382	3	8.28	Wfdc13	NP_001012722.1	340	11	34.10
Gpr35	XP_006529840.1	249	3	12.70	Spint4	NP_084334.1	430	10	24.51
Aqp12	NP_808255.1	165	1	6.39	Spint5	NP_001035144.1	309	6	20.46
Kif1a	NP_001103785.1	1940	6	3.26	Wfdc3	NP_082237.1	44	3	71.86
Agxt	NP_057911.2	1464	1	0.72	Dnttip1	NP_598524.1	216	4	19.52
Mab21l4	XP_006529940.1	109	7	67.68	Ube2c	NP_081061.1	2185	0	0.00
Crocc2	XP_006530046.1	32	0	0.00	Tnnc2	NP_033420.1	1296	2	1.63
Sned1	NP_766051.4	543	10	19.41	Snx21	XP_030102304.1	131	5	40.23
Mterf4	NP_001297685.1	255	0	0.00	Acot8	NP_573503.2	391	4	10.78
Pask	NP_543126.2	1141	4	3.69	Zswim3	NP_848462.1	194	2	10.86
Ppp1r7	NP_075689.1	920	8	9.16	Zswim1	NP_082304.1	8	0	0.00
Ano7	NP_001258813.1	185	10	56.97	Spata25	NP_083646.1	51	4	82.66
Hdlbp	NP_001344485.1	770	4	5.47	Neurl2	NP_001076443.1	364	2	5.79
Septin2	XP_006529303.1	1087	11	10.67	Ctsa	XP_030104427.1	1109	0	0.00
Farp2	NP_663494.2	362	8	23.29	Pltp	XP_036015508.1	545	4	7.74
Stk25	XP_006529831.1	821	8	10.27	Pcif1	NP_001361055.1	141	2	14.95
Bok	NP_058058.1	458	2	4.60	Zfp335	NP_950192.2	380	4	11.09
Thap4	NP_080196.3	114	7	64.71	Mmp9	NP_038627.1	2197	3	1.44
Atg4b	NP_777363.1	888	0	0.00	Slc12a5	NP_001342409.1	1132	4	3.72
Dtymk	NP_001099137.1	1212	10	8.70	Ncoa5	NP_659141.1	858	7	8.60
Ing5	NP_079730.1	709	2	2.97	Cd40	NP_035741.2	1416	2	1.49
D2hgdh	NP_001297696.1	270	0	0.00	Cdh22	NP_778153.2	763	1	1.38
Gal3st2	NP_955398.3	31	2	67.99	Slc35c2	NP_001349945.1	355	4	11.87
Gal3st2b	NP_001192178.1	23	2	91.64	Elmo2	NP_001289683.1	466	4	9.05
Gal3st2c	XP_006529834.1	19	2	110.94	Zfp663	NP_001366403.1	139	0	0.00
Neu4	NP_001297698.1	211	5	24.97	Zfp334	XP_036017534.1	169	5	31.18
Pdcd1	NP_032824.1	603	0	0.00	Ocstamp	NP_083297.1	129	1	8.17
Fam174a	NP_080597.2	56	7	131.74	Slc13a3	NP_473396.1	521	10	20.23
St8sia4	XP_006529338.1	366	4	11.52	Trp53rka	NP_001007582.1	395	2	5.34
Slco4c1	NP_766246.1	429	4	9.83	Slc2a10	NP_569718.1	2123	7	3.47
Slco6c1	XP_006529976.1	109	1	9.67	Eya2	NP_034295.1	534	3	5.92
Macir	NP_001342000.1	32	3	98.80	Zmynd8	XP_006499453.1	502	3	6.30
Ppip5k2	XP_036020456.1	619	5	8.51	Ncoa3	NP_032705.2	974	1	1.08
Gin1	NP_080526.2	353	4	11.94	Sulf2	NP_001239508.1	539	7	13.69
Pam	XP_030108050.1	446	0	0.00	Prex1	NP_808450.2	694	4	6.07
Slco6d1	XP_011246380.1	179	1	5.89	Trp53rkb	XP_006500434.1	444	2	4.75
Cntnap5b	XP_006529657.1	489	0	0.00	Arfgef2	NP_001078964.1	981	10	10.74
Cdh20	NP_035930.1	596	5	8.84	Cse1l	NP_076054.1	1392	4	3.03
Rnf152	XP_017176696.1	500	6	12.65	Stau1	NP_035620.1	593	3	5.33
Pign	XP_017176565.1	404	9	23.48	Ddx27	NP_694705.2	1614	5	3.26
Relch	XP_006529564.1	889	10	11.85	Znfx1	NP_001028368.2	655	1	1.61
Gm10193	XP_036011919.1	219	0	0.00	Kcnb1	XP_017171221.1	1183	1	0.89
Tnfrsf11a	NP_033425.3	428	4	9.85	Ptgis	NP_032994.1	579	1	1.82
Zcchc2	XP_006529575.1	499	4	8.45	B4galt5	XP_036018302.1	318	1	3.31
Phlpp1	NP_598582.3	1104	6	5.73	Slc9a8	XP_036018592.1	632	2	3.34
Bcl2	NP_033871.2	680	0	0.00	Spata2	NP_001343452.1	102	0	0.00
Kdsr	NP_081810.1	359	9	26.42	Rnf114	NP_001347859.1	464	5	11.36
Vps4b	NP_033216.2	786	11	14.75	Snai1	NP_035557.1	1514	3	2.09
Serpinb5	NP_001347782.1	1405	10	7.50	Ube2v1	NP_001298075.1	809	2	2.61
Serpinb12	NP_082247.1	254	14	58.09	Tmem189	NP_663513.1	204	4	20.66
Serpinb13	NP_766440.2	179	9	52.99	Cebpb	NP_034013.1	1239	5	4.25
Serpinb3a	NP_033152.3	442	8	19.08	Ptpn1	NP_035331.3	1229	3	2.57
Serpinb3d	NP_958764.1	226	3	13.99	Ripor3	NP_001074177.1	57	0	0.00
Serpinb3b	NP_941373.1	275	7	26.83	Pard6b	NP_067384.2	614	2	3.43
Serpinb3c	NP_958751.2	1165	9	8.14	Adnp	NP_001297015.1	384	3	8.23
Serpinb11	NP_080143.1	297	11	39.03	Dpm1	NP_001297013.1	803	0	0.00
Serpinb7	NP_081824.1	1110	10	9.49	Mocs3	NP_001153802.1	865	1	1.22
Serpinb2	NP_001167641.1	1228	8	6.87	Kcng1	NP_001074603.1	828	2	2.55
Serpinb10	XP_006529660.1	374	4	11.27	Nfatc2	XP_036015206.1	760	3	4.16
Serpinb8	NP_035589.1	1073	3	2.95	Atp9a	NP_001276375.1	608	6	10.40
Cdh7	XP_017176182.1	567	0	0.00	Sall4	NP_958798.2	722	1	1.46
Cdh19	NP_001074855.1	443	2	4.76	Zfp64	NP_001355988.1	332	8	25.40
Dsel	XP_017176672.1	212	1	4.97	Tshz2	NP_808327.1	419	8	20.12

Cntnap5a	NP_001357942.1	308	1	3.42	Zfp217	XP_006499497.1	644	9	14.73
B020011L13Ri	NP_001359285.1	126	0	0.00	Bcas1	XP_030108061.1	389	5	13.55
Tsn	NP_035780.1	568	5	9.28	Cyp24a1	NP_034126.1	566	8	14.90
Nifk	NP_080748.3	1194	1	0.88	Pfdn4	NP_001103622.1	791	8	10.66
Clasp1	XP_006529983.1	605	0	0.00	Dok5	NP_084037.3	482	6	13.12
Tfcp2l1	NP_076244.2	458	2	4.60	Cbln4	NP_783439.1	701	8	12.03
Gli2	NP_001074594.1	1331	6	4.75	Mc3r	NP_032587.1	436	9	21.75
Inhbb	NP_032407.1	667	6	9.48	Fam210b	NP_080188.3	173	5	30.46
Ralb	NP_071722.1	1654	6	3.82	Aurka	NP_035627.1	2386	12	5.30
Tmem185b	NP_666215.1	115	4	36.66	Cstf1	NP_077161.1	493	12	25.65
Epb41l5	XP_006529462.1	516	10	20.42	Cass4	NP_001366394.1	182	6	34.74
Ptpn4	XP_006529318.1	652	9	14.55	Rtf2	NP_079818.1	268	10	39.32
Tmem177	NP_780315.1	137	0	0.00	Gcnt7	XP_006500059.1	286	5	18.42
Cfap221	XP_017175371.1	158	5	33.35	Fam209	NP_083884.1	36	3	87.83
Sctr	NP_001012322.2	386	7	19.11	Tfap2c	XP_006499166.1	687	7	10.74
Tmem37	NP_062305.1	169	3	18.71	Bmp7	NP_031583.2	1188	4	3.55
Dbi	NP_031856.1	672	4	6.27	Spo11	XP_006499680.1	1095	10	9.62
3110009E18Ri	XP_017167942.1	17	2	123.99	Rae1	NP_780321.1	1010	7	7.30
Steap3	XP_006529908.1	352	6	17.96	Rbm38	NP_062420.2	368	8	22.91
C1ql2	NP_997116.1	373	1	2.83	Ctcfl	NP_001074856.1	777	5	6.78
Marco	NP_034896.1	441	0	0.00	Pck1	NP_035174.1	1441	10	7.31
En1	NP_034263.2	614	0	0.00	Zbp1	NP_067369.2	379	2	5.56
Insig2	NP_001344180.1	662	0	0.00	Pmepa1	NP_075371.2	352	6	17.96
Ccdc93	NP_001020327.1	118	1	8.93	1700021F07R	NP_082434.1	237	2	8.89
Htr5b	NP_034613.2	403	2	5.23	Ankrd60	NP_081579.2	1132	3	2.79
Ddx18	NP_080136.2	1956	0	0.00	Rab22a	NP_077756.2	953	8	8.85
Dpp10	XP_006529689.1	827	0	0.00	1700010B08R	NP_083584.1	0	0	#DIV/0!
Actr3	NP_076224.1	1632	3	1.94	Vapb	NP_062780.2	1136	5	4.64
Slc35f5	NP_001343223.1	243	6	26.02	Stx16	NP_766263.2	948	8	8.89
Gpr39	NP_081953.2	392	3	8.07	Npepl1	NP_001365722.1	691	2	3.05
Lypd1	XP_006529948.3	386	5	13.65	Gnas	NP_062664.2	1544	9	6.14
Nckap5	XP_036019094.1	225	3	14.05	Nelfcd	NP_065605.2	341	5	15.45
Mgat5	XP_030110888.1	351	1	3.00	Ctsz	NP_071720.1	803	9	11.81
Tmem163	NP_082411.1	401	7	18.40	Tubb1	NP_001074440.1	1376	8	6.13
Acmsd	NP_001028213.1	224	8	37.64	Atp5e	NP_080259.1	733	9	12.94
Ccnt2	NP_082675.1	1080	6	5.86	Prelid3b	NP_079807.1	298	7	24.76
Map3k19	NP_035867.2	1197	9	7.92	Zfp831	XP_006498580.1	165	5	31.94
Rab3gap1	XP_006529468.1	520	11	22.29	Edn3	NP_031929.1	653	7	11.30
Zranb3	NP_001272874.1	1105	7	6.68	Gm14444	NP_001365634.1	130	0	0.00
R3hdm1	NP_001344411.1	461	9	20.58	Gm14393	XP_030107802.1	126	0	0.00
Ubxn4	NP_001343458.1	335	9	28.31	Gm14391	NP_001170951.1	126	0	0.00
Lct	NP_001074547.1	495	5	10.65	Gm14440	NP_001186237.1	126	0	0.00
Mcm6	NP_032593.1	1475	7	5.00	Gm4631	XP_017174895.1	160	0	0.00
Dars	NP_803228.2	1397	6	4.53	Zfp968	NP_001361675.1	128	0	0.00
Cxcr4	NP_001343438.1	2037	1	0.52	Gm4724	XP_030101875.1	160	0	0.00
Thsd7b	XP_036019103.1	249	0	0.00	Zfp965	XP_036012916.1	156	0	0.00
Cd55b	XP_017169885.1	457	3	6.92	Gm2007	XP_030108177.1	160	0	0.00
Cd55	XP_030102097.1	420	4	10.04	Gm14288	NP_001028295.2	126	0	0.00
Zp3r	XP_011246294.1	189	2	11.15	Gm6710	XP_030107788.2	128	0	0.00
C4bp	XP_017167981.1	715	6	8.84	Zfp966	NP_001170876.1	128	0	0.00
Pfkfb2	NP_001155887.1	645	5	8.17	Gm2026	XP_030101730.1	160	0	0.00
Yod1	NP_848806.2	512	0	0.00	Gm2004	XP_030107790.1	127	0	0.00
AA986860	NP_808272.2	118	0	0.00	2210418O10R	NP_001292065.1	127	0	0.00
Fcamr	NP_001164103.1	104	5	50.67	Gm14434	NP_001095274.1	160	0	0.00
Pigr	NP_035212.2	958	4	4.40	Zfp967	NP_001170875.1	129	0	0.00
Fcmr	NP_081252.1	231	5	22.81	Gm14308	NP_001092819.1	128	0	0.00
Il24	NP_444325.2	271	5	19.44	Zfp973	NP_001229872.1	156	0	0.00
Il20	NP_001298020.1	178	2	11.84	Gm14305	NP_001092797.1	155	0	0.00
Il19	XP_006529768.1	354	4	11.91	Gm14295	XP_030101746.1	127	0	0.00
Il10	NP_034678.1	2285	10	4.61	Gm14296	NP_001292061.1	127	0	0.00
Mapkapk2	NP_032577.1	532	2	3.96	Gm14410	NP_001361058.1	126	0	0.00
Dyrk3	NP_663483.1	896	4	4.70	Zfp970	NP_001171039.1	133	0	0.00
Eif2d	XP_006529228.1	437	3	7.24	Gm14322	XP_011238254.1	126	0	0.00
Rassf5	NP_061220.2	447	0	0.00	Gm14325	NP_001020020.2	128	0	0.00
Ikbke	NP_062751.2	1471	2	1.43	Zfp972	NP_898958.1	126	0	0.00
Srgap2	NP_001074480.2	749	1	1.41	Gm14326	NP_001268957.1	126	0	0.00

Fam72a	NP_780591.1	218	1	4.83	Zfp971	NP_001170870.1	130	0	0.00
Avpr1b	NP_036054.1	414	0	0.00	Zfp931	XP_036018232.1	156	4	27.02
Ctse	NP_031825.2	830	6	7.62	Phactr3	NP_001171260.1	759	4	5.55
Rab7b	XP_036019826.1	965	3	3.28	Sycp2	NP_796165.2	364	4	11.58
Slc26a9	NP_796217.2	351	2	6.01	Ppp1r3d	NP_001078970.1	280	4	15.06
Pm20d1	XP_006529402.1	685	5	7.69	Fam217b	NP_001074758.1	68	4	61.99
Slc41a1	XP_036010523.1	308	4	13.69	Cdh26	NP_941058.1	266	0	0.00
Rab29	NP_659124.2	381	5	13.83	Cdh4	NP_033997.1	573	6	11.04
Nucks1	NP_780503.2	371	4	11.36	Taf4	XP_006500651.2	698	7	10.57
Slc45a3	NP_666089.1	435	6	14.54	Lsm14b	XP_006500681.1	512	9	18.53
Elk4	NP_031949.2	545	4	7.74	Psma7	NP_036099.1	1574	7	4.69
Mfsd4a	NP_766098.2	194	1	5.43	Ss18l1	NP_848865.4	322	9	29.46
Cdk18	NP_032821.1	1984	5	2.66	Mtg2	XP_017174613.1	605	9	15.68
Lemd1	XP_006529410.1	122	1	8.64	Hrh3	NP_598610.1	1050	7	7.03
Gm7241	XP_036010734.1	425	0	0.00	Osbpl2	XP_017173482.1	374	12	33.81
Klhdc8a	NP_659059.1	114	0	0.00	Adrm1	NP_062796.2	768	9	12.35
Nuak2	NP_001181954.1	303	2	6.96	Lama5	NP_001074640.1	684	13	20.03
Tmcc2	NP_849205.1	255	6	24.80	Rps21	NP_001342450.1	933	5	5.65
Dstyk	NP_766104.2	952	11	12.18	Cables2	XP_006500692.1	247	5	21.33
Rbbp5	NP_766105.2	791	8	10.66	Rbbp8nl	XP_006500714.1	108	6	58.55
Tmem81	XP_030099079.1	30	11	386.43	Gata5	NP_032119.2	1060	2	1.99
Cntn2	NP_796103.2	1198	10	8.80	Slco4a1	NP_001342147.1	344	2	6.13
Nfasc	XP_006529716.2	1069	4	3.94	Ntsr1	NP_061236.1	739	1	1.43
Lrrn2	NP_034862.1	1159	13	11.82	Mrgbp	NP_082755.1	415	2	5.08
Mdm4	NP_032601.2	759	4	5.55	Ogfr	NP_113550.3	183	1	5.76
Pik3c2b	NP_001092746.2	1959	12	6.46	Col9a3	NP_001365706.1	424	2	4.97
Ppp1r15b	NP_598580.1	400	15	39.52	Tcfl5	NP_839985.2	248	0	0.00
Plekha6	XP_017175990.1	247	11	46.93	Dido1	XP_006500671.1	1010	0	0.00
Golt1a	NP_080956.1	957	13	14.32	Gid8	XP_030108051.1	257	1	4.10
Kiss1	XP_006529744.1	957	12	13.22	Slc17a9	NP_898984.3	271	1	3.89
Ren1	NP_112469.1	1030	9	9.21	Bhlhe23	NP_542372.2	226	3	13.99
Etnk2	NP_780652.2	865	11	13.40	Ythdf1	NP_776122.1	410	1	2.57
Sox13	XP_006529340.1	575	4	7.33	Birc7	NP_001156719.1	586	0	0.00
Snrpe	NP_033253.1	1595	11	7.27	Nkain4	XP_030107776.1	124	0	0.00
Zc3h11a	NP_653113.4	325	3	9.73	Arfgap1	NP_001361599.1	814	5	6.47
Zbed6	NP_001160024.1	325	3	9.73	Col20a1	XP_036018507.1	242	4	17.42
Lax1	NP_001153121.1	288	7	25.62	Chrna4	XP_006500614.1	969	3	3.26
Atp2b4	XP_006529789.1	1147	3	2.76	Kcnq2	NP_001006669.1	1575	4	2.68
Optc	NP_001153892.1	682	3	4.64	Eef1a2	NP_031932.1	2996	6	2.11
Prelp	NP_473418.3	841	4	5.01	Ppdpf	NP_001343356.1	177	3	17.86
Fmod	NP_067330.1	1044	3	3.03	Ptk6	NP_033210.1	1012	2	2.08
Btg2	NP_031596.1	655	0	0.00	Srms	NP_035611.3	917	9	10.34
Gm8618	XP_036010771.1	1516	1	0.70	Fndc11	NP_955016.1	30	7	245.91
Chit1	NP_001271453.1	345	1	3.05	Helz2	XP_006500664.1	2033	3	1.56
Chil1	NP_001361555.1	486	1	2.17	Gmeb2	XP_036017633.1	298	11	38.90
Mybph	NP_001344444.1	401	2	5.26	Stmn3	NP_033159.1	1321	10	7.98
Adora1	NP_001278857.1	1018	0	0.00	Rtel1	XP_006500696.1	816	9	11.62
Myog	NP_112466.1	1109	2	1.90	Arfrp1	NP_083978.3	466	10	22.62
Ppfia4	XP_006529911.1	481	5	10.96	Zgpat	NP_001041613.1	231	10	45.62
Tmem183a	NP_001035950.1	109	0	0.00	Zbtb46	XP_006500777.1	390	8	21.62
Mgat4f	NP_776132.2	72	0	0.00	Lime1	NP_076173.1	114	6	55.47
Cyb5r1	NP_082333.1	491	2	4.29	Abhd16b	NP_899004.1	149	3	21.22
Adipor1	NP_082596.2	441	1	2.39	Tpd52l2	XP_036018341.1	319	1	3.30
Klhl12	XP_006529624.1	383	4	11.01	Dnajc5	XP_036013878.1	861	5	6.12
Rabif	NP_663485.1	217	1	4.86	Uckl1	XP_030107893.1	1052	3	3.01
Mgat4e	XP_011246388.2	72	0	0.00	Zfp512b	XP_030107531.1	294	1	3.58
Kdm5b	NP_690855.2	2104	2	1.00	Samd10	NP_766264.2	144	0	0.00
Syt2	NP_001342655.1	937	1	1.12	Prpf6	NP_598462.1	1087	2	2.04
Ppp1r12b	NP_001355756.1	1192	2	1.77	Sox18	NP_033262.2	512	0	0.00
Ube2t	NP_080300.1	1770	4	2.38	Tcea2	NP_033352.1	685	3	5.43
Lgr6	XP_006529777.1	898	2	2.35	Rgs19	XP_030107756.1	483	1	2.73
Ptpn7	NP_001343311.1	702	0	0.00	Lkaaear1	XP_006500718.1	14	0	0.00
Arl8a	NP_081099.1	1436	7	5.14	Oprl1	NP_001305848.1	1129	3	4.00
Gpr37l1	NP_602320.2	826	1	1.28	Myt1	NP_001165086.1	410	2	7.91
Elf3	NP_031947.1	461	4	9.14	Pcmtd2	XP_036017949.1	415	1	4.23
Rnpep	NP_001153096.1	559	2	3.77	Polr3k	NP_080177.1	779	1	2.46

Timm17a	NP_035720.1	1026	1	1.03	Gm14496	XP_030107841.1	18	0	0.00
Lmod1	NP_444336.2	381	2	5.53	Hnf4g	NP_038948.1	633	1	3.33
Shisa4	NP_780468.1	93	0	0.00	Zfhx4	XP_017175305.1	457	1	4.19
Ipo9	NP_722469.1	608	2	3.47	Pex2	XP_017174994.1	323	1	5.44
Nav1	XP_006529441.1	244	3	12.96	1700008P02R	NP_081324.1	136	0	0.00
Csrp1	NP_001347711.1	946	6	6.68	Pkia	XP_006530119.1	418	1	3.60
Phlda3	NP_038778.1	279	0	0.00	Zc2hc1a	XP_006530149.1	103	1	13.64
Tnni1	NP_067442.1	701	3	4.51	Il7	NP_032397.1	955	0	0.00
Lad1	NP_598425.2	208	3	15.20	Stmn2	NP_079561.1	1365	2	1.82
Tnnt2	XP_006529446.2	1100	4	3.83	Hey1	NP_034553.2	824	2	2.84
Pkp1	NP_001300630.1	567	2	3.72	Mrps28	NP_079710.3	470	0	0.00
Igfn1	NP_808310.2	520	1	2.03	Tpd52	NP_033438.1	531	3	5.95
Tmem9	NP_079715.1	170	1	6.20	Zbtb10	NP_808328.2	482	0	0.00
Ascl5	NP_001257538.1	110	0	0.00	Zfp704	NP_573481.1	146	3	21.66
Cacna1s	NP_001074492.1	982	5	5.37	Pag1	XP_030108786.1	287	0	0.00
Kif21b	NP_001034561.1	1055	4	4.00	Fabp5	NP_034764.1	908	1	1.16
Mroh3	NP_001316539.1	34	0	0.00	Pmp2	NP_001025476.1	372	0	0.00
Inava	NP_083148.3	259	2	8.14	Fabp9	NP_035728.2	342	0	0.00
Gpr25	NP_001094986.1	282	2	7.47	Fabp4	NP_077717.1	1075	0	0.00
Camsap2	NP_001334039.1	348	1	3.03	Fabp12	XP_017175272.1	174	1	6.06
Ddx59	XP_006529907.1	1467	3	2.16	Impa1	XP_006530140.1	672	3	4.70
Kif14	XP_011246341.1	1136	6	5.57	Slc10a5	NP_001010834.1	507	1	2.08
Zfp281	NP_001153723.1	516	1	2.04	Zfand1	NP_079788.2	239	4	17.64
Nr5a2	XP_006529684.1	1223	7	6.03	Chmp4c	NP_079795.1	501	0	0.00
Ptprc	NP_001255215.1	2594	2	0.81	Snx16	NP_001120663.1	330	1	3.19
Atp6v1g3	NP_796371.1	299	2	7.05	Ralyl	NP_001343963.1	1931	1	0.55
Nek7	NP_067618.1	539	6	11.73	Slc7a12	NP_543128.1	230	0	0.00
Lhx9	XP_030107588.1	790	7	9.34	Lrrcc1	NP_083191.2	654	1	1.61
2310009B15R	NP_001074695.1	18	1	58.55	E2f5	NP_031918.2	932	0	0.00
Dennd1b	XP_006529779.1	363	3	8.71	Rbis	NP_001334135.1	96	0	0.00
Crb1	XP_006529280.1	1100	10	9.58	Car13	NP_078771.1	360	0	0.00
Zbtb41	NP_766231.2	364	6	17.37	Car1	NP_033929.2	406	0	0.00
Aspm	NP_033921.3	2040	2	1.03	Car3	NP_031632.2	593	0	0.00
F13b	NP_112441.2	768	9	12.35	Car2	NP_001344263.1	760	0	0.00
Cfhr1	NP_056595.1	361	6	17.52	Gm9733	NP_001070147.1	237	4	17.79
Cfhr3	NP_001361031.1	509	5	10.35	Sirpb1a	NP_001002898.1	374	4	11.27
Gm4788	NP_001153775.1	594	7	12.42	Sirpb1b	XP_017175178.1	327	4	12.89
Cfhr2	NP_001020746.1	834	6	7.58	Sirpb1c	XP_006535434.1	297	4	14.19
Cfh	NP_034018.2	673	9	14.09	Gm5150	XP_036019040.1	200	4	21.08
Kcnt2	XP_017175995.1	327	4	12.89	Ythdf3	NP_766265.3	564	0	0.00
Cdc73	NP_666103.1	877	6	7.21	Cypt12	NP_083565.1	300	1	3.51
B3galt2	NP_064409.3	265	6	23.86	Bhlhe22	NP_067535.3	704	0	0.00
Glrx2	NP_001033681.1	651	6	9.71	Cyp7b1	NP_031851.3	782	1	1.35
Ro60	XP_017174940.1	378	9	25.09	Armc1	NP_001344618.1	230	2	9.16
Uchl5	NP_062508.2	1225	7	6.02	Mtfr1	NP_001343224.1	152	3	20.80
Rgs2	NP_033087.2	852	5	6.18	Pde7a	XP_006535469.1	618	4	6.82
Rgs13	NP_694811.1	435	2	4.85	Dnajc5b	NP_079765.3	576	4	7.32
Rgs1	XP_036008186.1	944	3	3.35	Trim55	NP_001074750.1	605	2	3.48
Rgs21	XP_036008542.1	307	0	0.00	Crh	NP_991338.1	835	1	1.26
Rgs18	NP_075019.1	695	6	9.10	Cp	XP_006535444.1	1166	1	0.90
Brinp3	NP_001344500.1	413	7	17.86	Hps3	NP_001139795.1	1919	4	2.20
Pla2g4a	NP_032895.1	1027	3	3.08	Hltf	NP_001342026.1	1548	0	0.00
Ptgs2	NP_035328.2	1991	3	1.59	Gyg	NP_038783.1	584	1	1.80
Pdc	NP_077778.1	373	0	0.00	Cpa3	NP_031779.1	514	0	0.00
Odr4	XP_036019881.1	136	0	0.00	Cpb1	XP_036019285.1	411	0	0.00
Tpr	NP_598541.3	1261	2	1.67	Agtr1b	NP_780295.2	555	2	3.80
Prg4	XP_036010444.1	349	0	0.00	Tbl1xr1	XP_017175306.1	1505	4	2.80
Hmcn1	NP_001019891.2	736	1	1.43	Nlgn1	XP_036018847.1	848	1	1.24
Ivns1abp	NP_473443.2	565	5	9.33	Spata16	NP_083426.1	296	0	0.00
Swt1	XP_011246360.1	342	4	12.33	Ect2	XP_036018777.1	890	1	1.18
Trmt1l	NP_081152.2	707	3	4.47	Nceh1	NP_848887.1	409	1	2.58
Rnf2	NP_001347773.1	1118	3	2.83	Tnfsf10	NP_033451.1	941	2	2.24
Niban1	NP_071301.2	241	1	4.37	Ghsr	XP_017175002.1	533	4	7.91
Edem3	XP_006529858.1	575	1	1.83	Fndc3b	XP_036019240.1	627	2	3.36
1700025G04R	XP_030098665.1	32	0	0.00	Tmem212	XP_006535494.1	91	1	11.58
Tsen15	NP_079953.2	137	1	7.69	Pld1	XP_017174978.1	1535	4	2.75

Colgalt2	NP_808424.3	317	1	3.32	Tnik	XP_006535582.1	825	1	1.28
Rgl1	NP_001333048.1	288	0	0.00	Slc2a2	NP_112474.2	1236	1	0.85
Apobec4	NP_001074666.1	114	2	18.49	Eif5a2	NP_808254.1	1309	3	2.42
Arpc5	NP_080645.2	595	0	0.00	Rpl22l1	NP_080793.1	996	1	1.06
Ncf2	XP_006529299.1	1160	7	6.36	Gm1527	XP_030108523.1	119	0	0.00
Smg7	XP_030109154.1	551	6	11.48	Egfem1	XP_036019265.1	98	1	10.75
Nmnat2	NP_780669.1	846	5	6.23	Mecom	NP_001357698.1	810	5	6.51
Lamc2	XP_011246229.1	503	5	10.48	Actrt3	NP_083966.1	1296	6	4.88
Lamc1	NP_034813.2	727	2	2.90	Mynn	NP_085034.2	427	10	24.68
Shcbp1l	XP_036009583.1	43	0	0.00	Lrrc34	NP_082217.1	191	2	11.04
Dhx9	NP_031868.2	1932	3	1.64	Lrriq4	XP_011248011.1	455	2	4.63
Npl	NP_083025.1	836	4	5.04	Lrrc31	XP_017175101.1	103	1	10.23
Rgs8	NP_001334044.1	840	8	10.04	Samd7	XP_017175280.1	70	0	0.00
Rgs16	NP_035397.2	442	3	7.15	Sec62	NP_001344457.1	712	6	8.88
Rnasel	XP_036020561.1	4270	11	2.71	Gpr160	XP_017175223.1	179	6	35.33
Rgsl1	XP_006529639.1	89	3	35.52	Phc3	XP_030108472.1	350	6	18.07
Teddm2	NP_839974.2	67	0	0.00	Prkci	NP_032883.2	1529	9	6.20
Teddm1b	NP_001008426.1	29	1	36.34	Skil	XP_006535490.1	425	6	14.88
Teddm1a	NP_839975.2	91	1	11.58	Cldn11	NP_032796.1	716	1	1.47
Glul	NP_032157.2	1893	4	2.23	Slc7a14	XP_030108473.1	730	0	0.00
Zfp648	NP_001191837.1	207	5	25.46	Kcnmb2	XP_006535611.1	92	1	11.46
Cacna1e	XP_017168201.1	1092	6	5.79	Zmat3	NP_033543.2	233	1	4.52
Ier5	NP_034630.1	306	5	17.22	Pik3ca	NP_032865.2	2772	7	2.66
Mr1	NP_032235.1	153	0	0.00	Kcnmb3	XP_017174903.1	226	1	4.66
Stx6	XP_006529829.1	785	5	6.71	Zfp639	NP_001343554.1	349	2	6.04
BC034090	XP_006530094.1	419	2	5.03	Mfn1	NP_077162.2	1125	3	2.81
Xpr1	NP_035403.1	518	2	4.07	Gnb4	NP_001334991.1	1397	6	4.53
Acbd6	NP_082526.2	279	6	22.66	Actl6a	NP_062647.2	2797	4	1.51
Lhx4	NP_034842.2	503	4	8.38	Mrpl47	NP_083293.1	630	2	3.35
Qsox1	NP_075757.1	783	8	10.77	Ndufb5	NP_079592.2	723	4	5.83
Cep350	NP_001034273.1	729	8	11.57	Usp13	NP_001013042.1	701	2	3.01
Tor1aip1	NP_659040.2	180	10	58.55	Pex5l	NP_001156988.1	914	2	2.31
Tor1aip2	NP_071724.1	130	9	72.96	Ttc14	XP_006535587.1	308	6	20.53
Fam163a	XP_017176771.1	45	4	93.68	Ccdc39	XP_006535561.1	343	3	9.22
Tdrd5	NP_001264659.1	904	9	10.49	Fxr1	NP_001106659.1	1043	7	7.07
Nphs2	NP_569723.1	746	9	12.71	Dnajc19	NP_001021382.1	572	9	16.58
Axdnd1	NP_001333896.1	45	9	210.78	Sox2	NP_035573.3	2312	6	2.74
Soat1	NP_033256.2	754	9	12.58	Atp11b	NP_083846.2	622	6	10.17
Abl2	NP_033725.2	1570	8	5.37	Dcun1d1	NP_001192290.1	453	6	13.96
Tor3a	NP_075630.2	338	14	43.65	Mccc1	NP_076133.3	511	8	16.50
Fam20b	NP_663388.1	230	7	32.08	Ccdc144b	NP_848505.2	1131	0	0.00
Ralgps2	NP_001153440.1	624	5	8.44	Acad9	NP_766266.3	601	2	3.51
Angptl1	NP_082609.2	476	6	13.28	Zfp267	NP_001094948.1	215	0	0.00
Tex35	NP_001361566.1	82	1	12.85	Qrfpr	NP_937835.1	272	0	0.00
Rasal2	XP_006496815.1	584	10	18.05	Anxa5	NP_033803.1	2142	6	2.95
Cryzl2	XP_017175484.1	242	0	0.00	1810062G17F	NP_082459.1	0	0	#DIV/0!
Sec16b	XP_017168472.1	583	4	7.23	Exosc9	NP_062266.1	808	1	1.30
Brinp2	NP_997466.2	535	6	11.82	Ccna2	NP_033958.2	2367	4	1.78
Astn1	XP_011237041.1	974	8	8.66	Bbs7	XP_006535598.1	396	6	15.97
Pappa2	NP_001078845.1	240	2	8.78	Trpc3	XP_006535502.1	626	1	1.68
Cop1	NP_036061.1	1062	4	3.97	4932438A13F	XP_006535509.1	547	4	7.71
Tnr	XP_030108834.1	846	2	2.49	Adad1	NP_033376.2	377	8	22.36
4930523C07R	NP_001156368.1	62	2	34.00	Il2	NP_032392.1	2018	6	3.13
Tnn	XP_006496967.1	760	2	2.77	Il21	NP_068554.1	645	5	8.17
Mrps14	NP_079750.1	1019	0	0.00	Cetn4	NP_665824.1	1198	3	2.64
Cacybp	NP_033916.1	986	5	5.34	Bbs12	NP_001008502.2	185	3	17.09
Rabgap1l	XP_006496939.1	721	3	4.39	Fgf2	XP_006535462.1	2368	8	3.56
Rc3h1	NP_001020123.1	493	3	6.41	Nudt6	XP_036018924.1	311	3	10.17
Serpinc1	XP_030099556.1	1011	2	2.08	Spata5	NP_001156983.1	1549	8	5.44
Zbtb37	NP_001343418.1	182	4	23.16	Spry1	NP_036026.1	571	1	1.85
Dars2	XP_006496820.1	623	5	8.46	Gm5148	NP_941059.1	9	0	0.00
Cenpl	NP_001153402.1	376	5	14.01	Ankrd50	NP_001161355.1	1197	6	5.28
Klhl20	NP_001034571.1	589	0	0.00	Fat4	NP_899044.3	1009	3	3.13
Ankrd45	XP_030099044.2	1997	7	3.69	Intu	XP_017175116.1	319	6	19.82
Prdx6	NP_031479.1	1124	3	2.81	Slc25a31	NP_848473.2	929	4	4.54
Tnfsf4	NP_033478.1	486	3	6.51	Hspa4l	XP_006500829.2	2137	7	3.45

Tnfsf18	NP_899247.3	247	2	8.53	Plk4	NP_035625.2	1872	7	3.94
Fasl	NP_034307.1	647	2	3.26	Mfsd8	NP_082416.2	212	4	19.88
Suco	XP_006496826.1	394	8	21.40	1700034I23Ri	NP_082770.1	54	0	0.00
4930558K02R	XP_006497078.1	11	3	287.43	Abhd18	XP_017175080.1	38	1	27.73
Pigc	XP_030098184.1	386	7	19.11	Larp1b	XP_036018902.1	419	1	2.52
Dnm3	XP_036021487.1	1949	9	4.87	Pgrmc2	NP_081834.1	645	1	1.63
Eef1aknmt	NP_659126.1	236	6	26.79	Jade1	XP_006500847.1	663	0	0.00
Vamp4	NP_001343455.1	1036	6	6.10	Sclt1	NP_001074880.1	357	1	2.95
Myoc	NP_034995.3	631	6	10.02	D3Ertd751e	XP_011238275.1	115	1	9.16
Myocos	NP_001171052.1	130	0	0.00	Pcdh10	NP_001091640.1	756	1	1.39
Prrc2c	XP_006496832.1	496	6	12.75	Pabpc4l	NP_001094949.1	803	2	2.62
Fmo4	XP_006496836.1	575	0	0.00	Pcdh18	NP_569715.3	408	0	0.00
Fmo1	NP_001317220.1	432	0	0.00	Slc7a11	XP_006500840.1	626	0	0.00
Fmo2	NP_001347843.1	522	0	0.00	Noct	NP_033964.1	637	5	8.27
Fmo6	NP_001171509.1	304	0	0.00	Elf2	XP_036019181.1	279	1	3.78
Fmo3	NP_032056.1	675	0	0.00	Mgarp	NP_080634.2	199	5	26.48
Mroh9	NP_084347.1	157	0	0.00	Ndufc1	NP_079799.1	578	6	10.94
Prrx1	NP_035257.1	907	5	5.81	Naa15	NP_444319.3	906	0	0.00
Gorab	NP_849214.2	229	4	18.41	Rab33b	NP_058554.1	799	4	5.28
Mettl11b	NP_001137428.1	206	4	20.46	Setd7	NP_542983.3	1647	6	3.84
Kifap3	XP_006496742.1	760	9	12.48	Mgst2	NP_778160.2	188	1	5.61
Scyl3	NP_083052.1	200	6	31.62	Maml3	XP_006501708.1	207	1	5.09
BC055324	NP_001343978.1	302	12	41.88	Foxo1	NP_062713.2	1908	2	1.10
Mettl18	NP_001342061.1	165	8	51.10	Cog6	NP_080501.2	372	8	22.66
Sele	XP_006496778.1	1031	4	4.09	Lhfp	NP_780595.1	268	2	7.86
Sell	NP_035476.1	1301	6	4.86	Nhlrc3	NP_766089.1	335	1	3.15
Selp	NP_035477.1	977	6	6.47	Proser1	NP_775558.2	51	1	20.66
F5	NP_032002.1	861	5	6.12	Stoml3	NP_694796.1	609	1	1.73
Slc19a2	NP_473428.1	516	7	14.30	Frem2	NP_766450.2	877	6	7.21
Ccdc181	NP_083391.2	118	7	62.52	Ufm1	NP_080711.1	537	5	9.81
Blzf1	NP_001153680.1	322	7	22.91	Trpc4	XP_006501359.1	626	3	5.05
Nme7	NP_612187.2	811	8	10.40	Postn	NP_001355607.1	1108	0	0.00
Atp1b1	NP_033851.1	1069	8	7.89	Supt20	NP_001344944.1	390	1	2.70
Dpt	NP_062733.1	589	1	1.79	Exosc8	NP_081424.3	675	2	3.12
Xcl1	NP_032536.1	586	2	3.60	Alg5	NP_079718.1	648	4	6.51
Tbx19	NP_114394.1	504	2	4.18	Smad9	XP_036019060.1	715	5	7.37
Sft2d2	NP_663487.1	255	3	12.40	Rfxap	NP_573494.1	113	3	27.98
Tiprl	NP_663488.1	448	4	9.41	Sertm1	NP_808522.1	285	1	3.70
Gpr161	NP_001074595.1	405	6	15.61	Ccna1	XP_036018767.1	2021	3	1.56
Dcaf6	NP_083035.1	930	5	5.67	Spg20	XP_036018927.1	437	2	4.82
Mpc2	NP_081706.1	441	5	11.95	Ccdc169	XP_006501635.1	25	0	0.00
Adcy10	XP_017176545.1	1227	4	3.44	Sohlh2	NP_083213.2	269	2	7.84
Mpzl1	NP_001334057.1	354	4	11.91	Dclk1	NP_064362.1	1956	5	2.69
Rcsd1	XP_006496847.1	291	3	10.86	Nbea	XP_036019011.1	692	5	7.61
Creg1	NP_035934.1	455	3	6.95	Mab21l1	NP_034880.1	449	3	7.04
Cd247	NP_001106863.1	666	2	3.16	Tm4sf1	NP_001342059.1	342	1	3.08
Pou2f1	NP_945150.2	604	1	1.74	Tm4sf4	NP_663514.2	730	3	4.33
Dusp27	NP_001028516.2	759	1	1.39	Wwtr1	NP_001161753.1	1219	7	6.05
Gpa33	NP_067623.1	277	0	0.00	Commd2	NP_780304.2	246	3	12.85
Mael	NP_780505.1	600	0	0.00	Ankub1	NP_001028521.1	40	3	79.04
Ildr2	NP_001158000.1	221	5	23.84	Rnf13	NP_001344009.1	258	0	0.00
Tada1	NP_084521.1	287	11	40.39	Pfn2	NP_062283.1	1164	3	2.72
Pogk	XP_017167846.1	225	12	56.21	Tsc22d2	NP_001074698.1	343	0	0.00
Gm4846	NP_001157778.1	206	8	40.93	Serp1	NP_109610.1	487	1	2.16
Gm4847	NP_001157784.1	253	9	37.49	Eif2a	NP_001005509.1	883	1	1.19
Fmo9	XP_006496916.1	265	9	35.79	Selenot	NP_001035486.2	260	1	4.05
Fam78b	NP_001153733.1	73	13	187.68	Erich6	NP_001074731.1	31	1	34.00
Uck2	NP_109649.1	1246	10	8.46	Siah2	NP_033200.2	883	2	2.39
Tmco1	NP_001034572.1	511	12	24.75	Clrn1	NP_700435.1	219	1	4.81
Aldh9a1	NP_064377.2	857	12	14.76	Med12l	XP_030108505.1	521	4	8.09
Mgst3	NP_079845.1	373	11	31.08	Gpr171	NP_775574.1	289	4	14.59
Lrrc52	NP_001013400.1	576	12	21.96	P2ry14	NP_001274053.1	470	3	6.73
Rxrg	NP_033133.1	763	6	8.29	Gpr87	XP_006502457.1	381	3	8.30
Lmx1a	XP_017177173.1	666	5	7.91	P2ry13	NP_083084.2	568	5	9.28
Pbx1	XP_006496763.1	998	4	4.22	P2ry12	NP_001343936.1	631	6	10.02
Nuf2	NP_075773.2	844	0	0.00	Igsf10	NP_001156356.1	738	3	4.28

Rgs5	NP_033089.2	584	0	0.00	Gm5709	XP_893237.4	39	1	27.02
Rgs4	NP_033088.2	1109	3	2.85	Aadacl2fm2	NP_001095001.1	39	1	27.02
Ccdc190	NP_001334067.1	22	0	0.00	Aadacl2fm3	NP_001229932.1	39	1	27.02
Hsd17b7	XP_006496730.1	661	4	6.38	Aadacl2fm1	NP_808329.1	40	1	26.35
Ddr2	XP_036018652.1	493	2	4.28	Aadacl2	NP_001121563.1	155	1	6.80
Uap1	XP_006496671.3	762	3	4.15	Aadac	NP_075872.1	798	5	6.60
Uhmk1	NP_034763.3	747	5	7.05	Sucnr1	NP_115776.2	830	12	15.24
Sh2d1b2	NP_001028671.1	572	2	3.68	Mbnl1	XP_030108561.1	786	6	8.05
Sh2d1b1	NP_036139.3	572	3	5.53	P2ry1	NP_001268945.1	552	6	11.46
Gm7694	NP_001185884.1	666	6	9.49	Rap2b	NP_082988.1	1605	4	2.63
Spata46	NP_001034682.1	60	4	70.26	Arhgef26	NP_001074764.1	447	5	11.79
Nos1ap	NP_001103455.1	666	6	9.49	Dhx36	NP_082412.2	1428	3	2.21
Olfml2b	XP_006496964.1	374	6	16.91	Gpr149	NP_796320.2	1696	3	1.86
Atf6	XP_011237098.1	950	6	6.66	Mme	NP_001344264.1	951	1	1.11
Dusp12	NP_075662.2	1061	3	2.98	Plch1	XP_017175084.1	733	1	1.44
Fcrlb	XP_006496994.1	134	2	15.73	E130311K13R	XP_030108510.1	1	0	0.00
Fcrla	NP_660123.1	340	4	12.40	Slc33a1	NP_056543.2	775	3	4.08
Fcgr2b	XP_036015707.1	1021	8	8.26	Gmps	XP_011238380.1	2058	5	2.56
Fcgr4	NP_653142.2	830	5	6.35	Vmn2r1	XP_036019070.1	61	1	17.28
Fcgr3	NP_001343440.1	741	5	7.11	Vmn2r2	XP_030108190.1	54	0	0.00
Cfap126	XP_036009999.1	115	2	18.33	Vmn2r3	NP_001371878.1	61	0	0.00
Sdhc	XP_006497018.2	1093	7	6.75	Vmn2r4	XP_036019104.1	47	0	0.00
Mpz	NP_001302429.1	425	2	4.96	Vmn2r5	NP_001372055.1	41	0	0.00
Pcp4l1	NP_079833.1	163	8	51.73	Vmn2r6	NP_001372142.1	47	0	0.00
Nr1i3	NP_033933.2	872	2	2.42	Vmn2r7	NP_783605.2	63	0	0.00
Tomm40l	XP_006497016.1	946	8	8.91	Kcnab1	NP_001344287.1	671	2	3.14
Apoa2	NP_038502.2	850	4	4.96	Ssr3	NP_080431.1	499	3	6.34
Fcer1g	NP_034315.1	976	6	6.48	Tiparp	NP_849223.2	423	2	4.98
Ndufs2	XP_030109284.1	994	8	8.48	Lekr1	NP_001160131.1	960	4	4.39
Adamts4	NP_766433.2	533	3	5.93	Ccnl1	NP_064321.2	690	2	3.05
B4galt3	NP_065604.2	329	10	32.03	Veph1	XP_006502206.1	145	1	7.27
Ppox	XP_030108150.1	450	3	7.03	Ptx3	NP_033013.3	506	0	0.00
Usp21	NP_038947.2	581	2	3.63	Shox2	NP_001289286.1	681	1	1.55
Ufc1	NP_079664.1	565	13	24.25	Rsrc1	NP_001343202.1	1207	5	4.37
Dedd	NP_001344480.1	361	9	26.27	Mlf1	NP_001034632.1	476	1	2.21
Nit1	NP_036179.1	404	8	20.87	Gfm1	NP_613057.2	2088	2	1.01
Pfdn2	NP_035200.2	896	5	5.88	Lxn	NP_058033.2	290	1	3.63
Klhdc9	NP_001028211.1	453	4	9.31	Rarres1	NP_001158235.1	372	2	5.67
Nectin4	NP_082169.2	237	2	8.89	Mfsd1	NP_080089.1	192	0	0.00
Arhgap30	NP_001005508.2	333	6	18.99	Iqcj	NP_808253.1	59	1	17.86
Usf1	XP_030108912.1	795	4	5.30	Iqschfp	NP_001106890.1	261	6	24.23
Tstd1	NP_001157997.1	795	3	3.98	Schip1	NP_001106892.1	262	7	28.16
F11r	NP_766235.1	551	3	5.74	Gm5848	XP_036019390.1	1213	3	2.61
Alyref2	NP_062357.3	614	1	1.72	Il12a	NP_001152896.1	365	2	5.77
Itln1	NP_034714.1	313	6	20.20	1110032F04R	NP_001161468.1	1	0	0.00
Cd244a	NP_061199.2	544	8	15.50	Ift80	XP_006502030.1	969	7	7.61
Ly9	NP_001264897.1	561	10	18.79	Smc4	NP_598547.1	1894	8	4.45
Slamf7	NP_653122.2	514	8	16.40	Trim59	NP_080139.3	666	3	4.75
Cd48	NP_031675.1	803	7	9.19	Kpna4	NP_032493.1	999	10	10.55
Slamf1	NP_038758.2	649	9	14.61	Arl14	NP_082119.1	1301	6	4.86
Cd84	NP_038517.1	490	11	23.66	Ppm1l	NP_848841.2	1342	6	4.71
Slamf6	NP_001334115.1	444	6	14.24	B3galnt1	XP_036019016.1	269	2	7.84
Vangl2	XP_017168497.1	613	8	13.75	Nmd3	NP_598548.1	1303	3	2.43
Nhlh1	NP_035046.1	456	7	16.18	Sptssb	NP_001273888.1	223	1	4.73
Ncstn	NP_067620.3	601	8	14.03	Otol1	NP_001018041.2	92	1	11.46
Copa	XP_036013272.1	680	1	1.55	Sis	NP_001074606.1	484	0	0.00
Pex19	NP_075528.3	531	14	27.79	Slitrk3	NP_001344780.1	702	3	4.50
Dcaf8	NP_001344133.1	245	13	55.92	Bche	XP_011238302.1	1091	2	1.93
Pea15a	NP_001316800.1	771	17	23.24	Zbbx	XP_006501321.1	77	4	54.75
Casq1	NP_033943.2	426	15	37.11	Serpini2	NP_080736.2	181	3	17.47
Atp1a4	NP_038762.1	554	17	32.34	Wdr49	XP_030108392.1	486	2	4.34
Atp1a2	NP_848492.1	1367	15	11.56	Pdcd10	NP_062719.2	623	4	6.77
Igsf8	NP_536344.1	439	16	38.41	Serpini1	NP_033276.1	823	6	7.68
Kcnj9	NP_032455.2	1056	16	15.97	Golim4	NP_780402.1	346	3	9.14
Kcnj10	XP_006496740.3	700	12	18.07	Fstl5	XP_030108393.1	568	6	11.13
Pigm	NP_080510.1	386	13	35.49	Rapgef2	XP_006502313.1	483	0	0.00

Slamf9	NP_083888.3	309	14	47.75	Gm17359	NP_001136425.1	5	1	210.78
Igsf9	XP_030099645.1	267	15	59.21	Fnip2	XP_036019031.1	268	1	3.93
Tagln2	NP_848713.1	806	14	18.31	Ppid	NP_080628.1	1735	1	0.61
Cfap45	NP_082248.1	205	13	66.83	Etfdh	NP_080070.2	630	3	5.02
Vsig8	NP_808391.2	192	10	54.89	4930579G24R	NP_083758.1	187	0	0.00
Slamf8	XP_006497072.1	384	5	13.72	Rxfp1	XP_006501692.1	960	3	3.29
Fcrl6	XP_011237163.1	105	6	60.22	Tmem144	NP_001366471.1	120	0	0.00
Dusp23	NP_081001.1	796	0	0.00	Gask1b	NP_573450.2	148	0	0.00
Crp	NP_031794.3	1353	2	1.56	Gria2	NP_001344853.2	1619	4	2.60
Apcs	NP_035448.2	872	5	6.04	Glrb	NP_001332925.1	1163	4	3.62
Olfr16	NP_032789.1	253	2	8.33	Pdgfc	NP_064355.1	411	0	0.00
Olfr1408	NP_666975.1	42	0	0.00	Ctso	NP_808330.1	406	10	25.96
Olfr1406	NP_666974.2	10	0	0.00	Tdo2	NP_064295.2	966	10	10.91
Olfr218	NP_001001809.2	206	1	5.12	Asic5	NP_067345.1	503	11	23.05
Olfr1404	NP_667092.1	65	1	16.21	Gucy1b1	NP_059497.1	1575	9	6.02
Fcer1a	XP_006496718.1	373	9	25.43	Gucy1a1	NP_001343916.1	1322	8	6.38
Olfr418	NP_666862.2	14	0	0.00	Map9	NP_001074699.1	357	6	17.71
Mptx2	NP_001191940.1	313	2	6.73	Npy2r	NP_001192028.1	702	8	12.01
Ackr1	NP_034175.2	433	2	4.87	Rbm46	NP_001139800.1	374	8	22.54
Cadm3	XP_006497123.3	955	10	11.04	Lrat	NP_076113.1	419	4	10.06
Aim2	XP_017176975.1	405	11	28.62	Fgg	NP_598623.1	1422	4	2.96
Ifi206	NP_001359366.1	80	4	52.70	Fga	NP_001104518.1	1479	5	3.56
Ifi214	NP_001019892.2	103	5	51.16	Fgb	NP_862897.1	1291	4	3.27
Ifi213	XP_011237154.1	94	3	33.64	Plrg1	NP_058064.2	1183	2	1.78
Ifi209	NP_778191.1	328	7	22.49	Sfrp2	NP_033170.1	871	1	1.21
Ifi208	NP_001156410.1	189	2	11.15	Tlr2	XP_030108471.1	2148	1	0.49
Ifi207	XP_011237100.1	244	4	17.28	Tmem131l	XP_036018932.1	139	2	15.16
Ifi204	XP_006496738.1	474	3	6.67	Mnd1	XP_006502325.1	407	0	0.00
Mndal	NP_001361569.1	163	13	84.05	Trim2	XP_030108778.1	727	3	4.35
Ifi211	NP_001288674.1	275	7	26.83	Fhdc1	NP_001028473.2	860	2	2.45
Ifi203	XP_030106120.1	269	13	50.93	Arfip1	XP_036019376.1	558	4	7.55
Ifi202b	NP_036070.2	246	6	25.70	Tigd4	NP_997161.1	198	2	10.65
Ifi205	XP_036020054.1	315	6	20.07	Tmem154	NP_796234.1	136	2	15.50
Olfr433	NP_666928.2	49	5	107.54	Fbxw7	NP_001171244.1	1586	4	2.66
Olfr432	NP_666927.2	57	5	92.45	Gatb	XP_006501393.1	405	0	0.00
Olfr430	NP_666929.2	76	0	0.00	Fam160a1	NP_766270.2	92	0	0.00
Olfr429	NP_666933.2	101	0	0.00	Glt28d2	NP_796104.2	119	0	0.00
Olfr427	NP_997041.2	53	0	0.00	Sh3d19	XP_006501609.3	632	4	6.67
Olfr231	NP_001005520.2	49	6	129.05	Prss48	NP_001001650.1	141	0	0.00
Olfr424	NP_666932.2	38	0	0.00	Rps3a1	NP_058655.3	1407	1	0.75
Olfr420	NP_666417.2	102	0	0.00	Lrba	XP_006502410.1	378	4	11.15
Spta1	NP_035595.2	955	4	4.41	Mab21l2	NP_035969.2	416	1	2.53
Olfr419	NP_666926.2	24	1	43.91	Dclk2	XP_030108678.1	816	2	2.58
Mptx1	NP_079746.1	182	0	0.00	Cd1d1	NP_001366431.1	740	4	5.70
Olfr417	NP_997020.3	25	1	42.16	Kirrel	NP_001363975.1	546	7	13.51
Olfr248	NP_666925.2	55	2	38.32	Fcrls	NP_109632.2	241	6	26.24
Olfr414	NP_666972.2	36	0	0.00	Cd5l	NP_033820.2	576	5	9.15
Olfr220	NP_997577.1	68	2	31.00	Fcrl1	NP_001297474.1	166	5	31.74
Fmn2	NP_062318.2	1303	8	6.47	Fcrl5	NP_899045.3	292	6	21.66
Grem2	XP_036020540.1	364	4	11.58	Etv3	NP_001076787.1	476	4	8.86
Rgs7	XP_017175954.1	993	10	10.61	Arhgef11	XP_006501327.1	774	6	8.17
Fh1	NP_034339.2	1134	3	2.79	Gm6570	NP_001357792.1	1165	2	1.81
Kmo	NP_598570.1	1184	8	7.12	Lrrc71	XP_006502270.1	82	6	77.11
Opn3	XP_006496705.1	297	11	39.03	Pear1	NP_082736.1	323	5	16.31
Chml	NP_067325.2	589	4	7.16	Ntrk1	XP_006501187.1	1895	4	2.22
Wdr64	NP_083729.2	1044	10	10.09	Insrr	XP_006501514.1	1710	10	6.16
Exo1	XP_030110359.1	1567	7	4.71	Sh2d2a	XP_006501617.1	237	4	17.79
Pld5	XP_006496960.1	172	12	73.53	Prcc	NP_291051.1	470	4	8.97
Cep170	XP_036008340.1	336	6	18.82	Hdgf	NP_032257.3	1286	0	0.00
Sdccag8	XP_036010197.1	410	9	23.13	Mrpl24	NP_080867.2	1129	3	2.80
Akt3	XP_036020522.1	2147	10	4.91	Rrnad1	XP_030108430.1	74	0	0.00
Zbtb18	NP_001342027.1	533	11	21.75	Isg20l2	XP_036018941.1	524	2	4.02
1700016C15R	NP_081353.1	20	6	316.17	Crabp2	NP_031785.1	531	2	3.97
Adss	NP_031448.2	951	4	4.43	Nes	NP_057910.3	1454	3	2.17
Catspere1	XP_006497147.1	99	6	63.87	Bcan	XP_030108256.1	915	4	4.61
Catspere2	XP_036008343.1	99	6	63.87	Hapln2	NP_071314.1	725	5	7.27

Desi2	NP_077244.1	265	7	27.84	Gpatch4	NP_079939.3	551	5	9.56
Cox20	XP_017177451.1	253	14	58.32	Naxe	NP_659146.1	681	3	4.64
Hnrnpu	XP_036008206.1	1299	13	10.55	Ttc24	NP_766114.1	722	0	0.00
Efcab2	NP_080902.1	1074	14	13.74	Iqgap3	NP_001028656.1	909	1	1.16
Kif26b	NP_001155137.1	1013	9	9.36	Mef2d	XP_017174961.1	1186	2	1.78
Smyd3	NP_081464.1	1015	8	8.31	Rhbg	XP_006501889.1	589	1	1.79
Tfb2m	NP_001317983.1	1288	6	4.91	Tsacc	NP_084077.1	67	0	0.00
Cnst	NP_666217.2	131	7	56.32	Cct3	NP_033966.1	1960	5	2.69
Sccpdh	XP_017176959.2	236	7	31.26	Glmp	NP_064387.1	168	1	6.27
Kif28	NP_001357826.1	669	0	0.00	Tmem79	NP_077208.1	161	4	26.18
Ahctf1	NP_080651.2	654	8	12.89	Smg5	NP_839977.2	554	2	3.80
Cdc42bpa	XP_017175608.1	1274	3	2.48	Paqr6	XP_006502072.1	150	2	14.05
Coq8a	NP_001156762.1	498	0	0.00	Bglap3	NP_112736.3	192	2	10.98
Psen2	XP_006496775.1	1147	4	3.68	Bglap2	NP_001027469.2	655	2	3.22
Itpkb	NP_001074644.1	5802	9	1.63	Bglap	NP_031567.1	698	4	6.04
Stum	XP_006496977.1	430	0	0.00	Pmf1	NP_001297534.1	378	3	8.36
Parp1	NP_031441.2	1627	2	1.30	Slc25a44	NP_848811.2	118	4	35.73
Lin9	NP_001096652.2	531	1	1.98	Sema4a	XP_006501250.1	454	2	4.64
Mixl1	NP_038757.1	690	3	4.58	Lmna	NP_001002011.2	1742	3	1.81
Acbd3	NP_573488.2	403	2	5.23	Mex3a	NP_001025061.2	135	1	7.81
H3f3a	NP_032236.1	1927	2	1.09	Rab25	NP_058595.2	1761	3	1.80
Sde2	NP_666055.1	201	4	20.97	Lamtor2	NP_112538.1	603	1	1.75
Lefty2	NP_796073.1	643	4	6.56	Ubqln4	NP_277068.1	2244	4	1.88
Pycr2	NP_598466.1	676	4	6.24	Ssr2	NP_001343246.1	538	1	1.96
Lefty1	NP_034224.1	787	6	8.03	Arhgef2	XP_006501121.1	714	2	2.95
Tmem63a	XP_036019041.1	379	6	16.68	Rxfp4	NP_861538.1	377	0	0.00
Ephx1	NP_001299847.1	818	3	3.87	Khdc4	XP_017175241.1	187	0	0.00
9130409I23Ril	NP_001028991.1	420	1	2.51	Rit1	XP_006501221.1	1507	3	2.10
Nvl	NP_080447.1	1067	1	0.99	Syt11	XP_036018945.1	1208	3	2.62
Cnih4	NP_084407.1	310	3	10.20	Gon4l	XP_036019277.1	234	2	9.01
Wdr26	XP_006496880.1	600	6	10.54	Msto1	NP_659147.2	273	1	3.86
Cnih3	XP_030099002.1	509	3	6.21	Dap3	XP_006501931.1	623	0	0.00
Ccdc121	NP_997163.2	101	1	10.43	Ash1l	XP_030108328.1	1412	1	0.75
Dnah14	NP_001363968.1	484	4	8.71	Rusc1	NP_001077276.1	109	1	9.67
Lbr	XP_017168530.1	1060	4	3.98	Fdps	NP_001240680.1	997	2	2.11
Enah	NP_001076590.1	746	2	2.83	Pklr	XP_006501198.1	1982	4	2.13
Srp9	NP_036188.1	1108	4	3.80	Hcn3	NP_032253.1	619	1	1.70
Vmn1r1	NP_001160200.1	1	0	0.00	Clk2	NP_031738.2	822	0	0.00
Degs1	NP_031879.1	397	3	7.96	Scamp3	NP_001296838.1	340	8	24.80
Fbxo28	NP_780336.1	238	9	39.85	Fam189b	XP_036019158.1	35	4	120.45
Trp53bp2	NP_775554.2	404	3	7.83	Gba	NP_001070879.1	988	7	7.47
Capn2	NP_033924.2	680	1	1.55	Mtx1	NP_001155296.2	402	6	15.73
Capn8	XP_006496749.1	825	2	2.55	Thbs3	NP_038719.2	435	9	21.80
Ccdc185	NP_001028719.1	277	1	3.80	Muc1	NP_038633.1	240	0	0.00
Susd4	NP_001343392.1	258	1	4.08	Trim46	NP_898858.1	438	5	12.03
Tlr5	XP_017177186.1	1206	0	0.00	Krtcap2	XP_006501933.3	550	6	11.50
Disp1	XP_006535659.1	248	0	0.00	Dpm3	XP_006502046.1	316	1	3.34
Brox	NP_001344171.1	128	1	8.23	Slc50a1	NP_033083.2	278	1	3.79
Aida	NP_859421.1	98	1	10.75	Efna1	NP_034237.3	561	1	1.88
Mia3	XP_036021861.1	382	1	2.76	Efna3	NP_001364045.1	528	0	0.00
Taf1a	NP_001264887.1	327	0	0.00	Efna4	NP_031936.2	451	5	11.68
Hhipl2	XP_011248035.1	128	0	0.00	Adam15	NP_033744.1	417	7	17.69
1700056E22Ril	NP_082792.1	0	0	#DIV/0!	Dcst1	NP_084250.1	178	7	41.45
Dusp10	NP_071302.2	881	3	3.59	Zbtb7b	NP_001342135.1	526	4	8.01
Hlx	NP_032276.1	432	3	7.32	Lenep	NP_065263.1	103	0	0.00
Mtarc1	NP_001277202.1	820	1	1.29	Flad1	NP_001349305.1	548	0	0.00
Mtarc2	NP_001355214.1	235	1	4.48	Cks1b	NP_058600.1	981	0	0.00
C130074G19R	NP_848807.1	66	0	0.00	Shc1	NP_001106802.1	991	5	5.32
Mark1	XP_006497204.1	406	1	2.60	Pygo2	NP_001280697.1	294	2	7.17
Rab3gap2	NP_001157226.1	390	0	0.00	Pbxip1	XP_006501414.1	202	9	46.96
Iars2	NP_941055.1	928	1	1.14	Pmvk	NP_081060.2	265	1	3.98
Bpnt1	NP_001334139.1	258	3	12.25	Kcnn3	NP_536714.2	601	6	10.52
Eprs	NP_001344403.1	2769	7	2.66	Adar	XP_006501816.3	770	1	1.37
Slc30a10	NP_001028458.1	631	1	1.67	Chrnb2	NP_033732.2	926	5	5.69
Lyplal1	NP_666218.2	473	2	4.46	Ube2q1	NP_081591.3	509	8	16.56
Tgfb2	NP_033393.2	1126	1	0.94	She	NP_766118.3	125	2	16.86

Rrp15	NP_080317.3	835	3	3.79	Il6ra	NP_001297605.1	779	5	6.76
D1Pas1	NP_149068.1	1720	6	3.68	Atp8b2	NP_001074651.2	516	0	0.00
Spata17	XP_036009920.1	104	3	30.40	Hax1	NP_001268961.1	515	2	4.09
Gpatch2	XP_006497262.1	323	5	16.31	Ubap2l	NP_705693.2	411	6	15.39
Esrrg	XP_030110282.1	634	6	9.97	4933434E20R	NP_080038.1	106	2	19.88
Ush2a	NP_067383.3	627	4	6.72	1700094D03F	NP_082843.2	106	3	29.83
Kctd3	NP_001346506.1	276	6	22.91	Tpm3	NP_071709.3	1247	5	4.23
Kcnk2	XP_006497184.1	374	2	5.64	Nup210l	NP_084213.1	260	5	20.27
Cenpf	NP_001074832.2	776	2	2.72	Rps27	NP_081291.1	1006	2	2.10
Ptpn14	NP_033002.2	625	1	1.69	Rab13	NP_080953.1	761	3	4.15
Smyd2	XP_006497209.1	924	0	0.00	Jtb	NP_996807.1	176	4	23.95
Prox1	NP_001347756.1	559	2	3.77	Creb3l4	XP_006502373.5	390	2	5.40
Rps6kc1	XP_006497235.1	541	1	1.95	Slc39a1	NP_038929.2	346	4	12.18
Angel2	NP_067396.3	480	2	4.39	Crtc2	NP_083157.1	344	4	12.25
Vash2	NP_659128.2	117	1	9.01	Dennd4b	NP_958809.3	176	3	17.96
Flvcr1	NP_001074728.1	337	1	3.13	Gatad2b	XP_006501422.3	365	1	2.89
Spata45	XP_030099146.1	21	0	0.00	Slc27a3	NP_036118.2	247	0	0.00
Tatdn3	NP_001344095.1	276	0	0.00	Ints3	NP_663515.2	415	1	2.54
Nsl1	NP_941056.3	315	3	10.04	Npr1	NP_032753.5	915	2	2.30
Batf3	NP_084336.1	688	1	1.53	Ilf2	NP_080650.1	1157	6	5.47
Fam71a	NP_001103229.1	91	1	11.58	Snapin	NP_598615.1	408	0	0.00
Atf3	NP_031524.2	1399	3	2.26	Chtop	NP_001280708.1	551	3	5.74
Nenf	NP_079700.1	543	3	5.82	S100a1	NP_035439.1	531	13	25.80
Pacc1	NP_080140.1	44	0	0.00	S100a13	XP_030108341.1	237	12	53.36
Ppp2r5a	NP_659129.2	1029	4	4.10	S100a14	NP_079669.2	163	7	45.26
Dtl	NP_084042.1	782	3	4.04	S100a16	NP_001343537.1	158	7	46.69
Ints7	NP_848747.4	285	2	7.40	S100a3	XP_036018860.1	320	10	32.93
Lpgat1	NP_001128301.1	446	5	11.82	S100a2	NP_001182689.1	48	6	131.74
Nek2	NP_035022.2	1382	7	5.34	S100a4	NP_035441.1	862	6	7.34
Slc30a1	NP_033605.1	472	8	17.86	S100a5	XP_036018861.1	270	7	27.32
Rd3	XP_036009770.1	315	8	26.77	S100a6	NP_035443.1	602	10	17.51
Traf5	XP_036019524.1	645	8	13.07	S100a7a	XP_006501694.1	115	8	73.31
Rcor3	NP_659063.3	525	8	16.06	S100a8	NP_038678.1	678	4	6.22
Kcnh1	NP_034730.1	830	11	13.97	S100a9	NP_001268781.1	617	4	6.83
Hhat	NP_659130.2	462	0	0.00	Pglyrp4	NP_001159440.1	112	1	9.41
Sertad4	XP_017175228.1	175	6	36.13	S100a7l2	XP_011238390.1	18	0	0.00
Syt14	XP_030110891.1	413	11	28.07	Pglyrp3	XP_006501534.1	159	3	19.88
Utp25	NP_663390.2	619	4	6.81	Lor	NP_032534.2	392	11	29.57
Irf6	NP_058547.2	553	4	7.62	Prr9	NP_780633.1	128	0	0.00
A130010J15Ri	NP_001153831.1	13	3	243.21	Lelp1	NP_081318.1	271	1	3.89
Traf3ip3	NP_694777.3	431	6	15.44	Sprr2a1	NP_035598.2	0	0	#DIV/0!
Hsd11b1	XP_006497291.1	1134	3	3.10	Sprr2a2	NP_001158259.1	17	0	0.00
G0s2	NP_032085.1	427	6	17.42	Sprr2a3	NP_001296311.1	17	0	0.00
Lamb3	NP_032510.2	466	7	19.79	Sprr2b	NP_035599.2	58	2	36.34
Camk1g	NP_659066.1	1352	4	4.16	Sprr2d	NP_035600.1	66	4	63.87
Plxna2	XP_006497303.1	717	4	8.40	Sprr2e	NP_035601.1	38	3	83.20
Cd34	NP_598415.1	1792	2	1.81	Sprr2f	NP_035602.1	79	2	26.68
Cd46	NP_034908.1	622	4	11.30	Sprr2h	NP_035604.1	152	10	69.34
Cr1l	NP_001341989.1	327	3	17.58	Sprr2i	NP_035605.1	71	4	59.37
Cr2	NP_031784.1	696	3	9.09	Sprr2k	NP_035607.2	39	4	108.09
9230019H11R	NP_001029149.1	20	2	210.78	Sprr1b	XP_017175001.1	265	6	23.86
H60c	XP_030101086.1	54	1	35.48	Sprr3	NP_001191356.1	233	6	27.14
Ppp1r14c	XP_006512552.1	261	3	20.19	Sprr1a	NP_033290.1	332	6	19.05
Iyd	NP_081667.1	1146	3	4.24	Sprr4	NP_775093.1	166	9	57.14
Plekhg1	XP_006512502.1	272	6	33.21	Ivl	XP_006501109.1	267	9	35.52
Mthfd1l	NP_758512.3	913	6	9.23	Smcp	NP_032600.3	363	0	0.00
Akap12	NP_112462.1	461	5	14.29	Lce6a	NP_001159644.1	56	9	169.38
Zbtb2	NP_001346740.1	253	9	44.11	Lce1a1	NP_080260.1	72	7	102.46
Rmnd1	XP_017169542.1	248	10	47.22	Lce1b	NP_081098.1	56	8	150.56
Armt1	NP_077223.2	331	8	26.81	Lce1a2	NP_082901.1	89	10	118.42
Ccdc170	XP_036011338.1	151	7	48.86	Lce1c	NP_082898.1	70	10	150.56
Esr1	XP_006512496.1	2915	6	2.17	Lce1d	NP_081413.1	90	12	140.52
Syne1	XP_036011812.1	1287	8	6.55	Lce1e	NP_081087.1	51	11	227.31
Myct1	NP_081069.1	265	8	31.82	Lce1f	NP_080670.2	35	10	301.11
Vip	NP_035832.1	622	2	3.39	Lce1g	NP_079689.2	54	5	97.58
Fbxo5	NP_080271.2	851	2	2.48	Lce1h	NP_080611.1	70	9	135.50

Mtrf1l	NP_780583.1	689	5	7.65	Lce1i	NP_083943.1	30	10	351.30
Rgs17	XP_030101045.1	595	2	3.54	Lce1j	NP_001268428.1	53	9	178.96
Oprm1	NP_001289724.1	828	8	10.18	Lce1k	NP_001241689.1	53	10	198.85
Ipcef1	XP_011241383.1	278	2	7.58	Kprp	NP_082905.1	346	18	54.83
Cnksr3	NP_766134.1	305	1	3.46	Lce1l	NP_082904.1	37	8	227.87
Ulbp1	NP_084251.1	293	0	0.00	2310050C09R	NP_079897.2	131	15	120.68
Lrp11	NP_001366080.1	276	5	19.09	Lce3a	NP_001034683.1	91	5	57.91
Pcmt1	XP_017169321.1	617	5	8.54	Lce3b	NP_079777.1	92	9	103.10
Nup43	NP_663752.2	856	4	4.92	Lce3c	NP_149410.1	148	14	99.69
Lats1	NP_034820.1	736	3	4.30	Lce3d	NP_001257355.1	114	7	64.71
Katna1	NP_035965.2	1010	5	5.22	Lce3e	NP_001241654.1	94	12	134.54
Ginm1	NP_663393.1	17	0	0.00	Lce3f	NP_001018089.1	44	11	263.48
Ppil4	NP_080417.2	1298	3	2.44	Crct1	NP_083074.2	140	16	120.45
Zc3h12d	NP_766373.2	273	0	0.00	Lce1m	NP_001342654.1	83	7	88.88
Tab2	NP_001346463.1	747	3	4.23	Tdpoz8	NP_001171165.1	381	0	0.00
Ust	NP_796361.2	229	4	18.41	Crnn	NP_001074669.1	274	14	53.85
Sash1	XP_006512900.1	515	7	14.32	Flg2	NP_001013826.2	248	12	51.00
Samd5	XP_017169459.1	293	8	28.78	Flg	XP_017175331.1	1579	1	0.67
Stxbp5	XP_006512991.1	746	7	9.89	Hrnr	XP_006502049.1	417	12	30.33
Adgb	XP_011241465.1	171	2	12.33	Rptn	NP_033126.2	237	12	53.36
Rab32	NP_080681.1	624	7	11.82	Tchh	XP_036019373.1	323	10	32.63
Grm1	XP_036011515.1	1217	6	5.20	Tchhl1	NP_082038.2	183	9	51.83
Shprh	NP_001071175.1	1368	3	2.31	S100a11	NP_058020.1	614	8	13.73
Fbxo30	NP_082244.2	393	2	5.36	S100a10	NP_033138.1	930	7	7.93
Epm2a	NP_034276.2	808	8	10.43	Tdpoz2	NP_001007223.2	253	0	0.00
Utrn	NP_035812.3	1166	6	5.42	Tdpoz1	NP_683751.2	419	1	2.52
Stx11	NP_083351.1	739	12	17.11	Tdpoz4	NP_997155.2	376	1	2.80
Sf3b5	NP_780311.2	1000	7	7.38	Tdpoz3	NP_997154.2	424	6	14.91
Plagl1	NP_001351573.1	640	2	3.29	Tdpoz5	NP_997156.2	395	1	2.67
Zc2hc1b	NP_083448.1	66	9	143.71	Tdpoz9	NP_001157202.1	436	1	2.42
Ltv1	NP_852135.1	913	6	6.93	Tdpoz7	NP_001157203.1	347	1	3.04
Phactr2	XP_006512739.1	421	8	20.03	Spopfm2	NP_001139579.1	338	6	18.71
Fuca2	NP_001317127.1	480	9	19.76	Them4	NP_083707.1	246	1	4.28
Pex3	NP_001157667.1	487	2	4.33	Them5	NP_079692.1	255	4	16.53
Adat2	NP_080024.3	625	7	11.80	C2cd4d	NP_001129589.1	116	1	9.09
Aig1	XP_030101074.1	125	0	0.00	Rorc	NP_035411.2	742	2	2.84
Hivep2	NP_001345712.1	572	5	9.21	Lingo4	NP_796224.1	433	5	12.17
Adgrg6	XP_006512744.1	710	3	4.45	Tdrkh	NP_082583.1	323	1	3.26
Vta1	XP_006512872.1	578	4	7.29	Oaz3	NP_058597.2	441	2	4.78
Gje1	NP_083998.1	78	3	40.53	Mrpl9	XP_006502385.1	710	0	0.00
Nmbr	NP_032729.1	449	3	7.04	Riiad1	XP_036019117.1	93	0	0.00
Cited2	XP_036011557.1	555	0	0.00	Celf3	NP_001366178.1	1267	1	0.83
Txlnb	NP_619534.2	353	2	5.97	Snx27	NP_001075953.1	348	0	0.00
Heca	NP_001028604.1	230	1	4.58	Tuft1	NP_001280657.1	235	6	26.91
Abracl	NP_001346489.1	277	1	3.80	Selenbp2	NP_062287.1	611	2	3.45
Reps1	NP_033074.2	311	1	3.39	Cgn	XP_030108677.1	278	2	7.58
Ect2l	NP_001181965.1	841	1	1.25	Pogz	NP_766271.2	761	3	4.15
Ccdc28a	NP_659069.2	110	3	28.74	Psmb4	NP_032971.2	1275	4	3.31
Nhsl1	XP_006512749.1	106	3	29.83	Selenbp1	NP_033176.2	566	3	5.59
Hebp2	NP_062360.1	292	2	7.22	Rfx5	XP_017175140.1	643	3	4.92
Arfgef3	NP_001028430.1	144	1	7.32	Pi4kb	NP_001280644.1	2219	6	2.85
Gm4922	XP_017169423.1	124	0	0.00	Zfp687	NP_001344792.1	294	1	3.58
Perp	NP_071315.1	565	3	5.60	Psmd4	NP_032977.1	1781	3	1.78
Tnfaip3	NP_001159874.1	1056	8	7.98	Pip5k1a	XP_036018832.1	911	2	2.31
Olig3	NP_443734.2	320	4	13.17	Vps72	NP_033362.2	630	1	1.67
Ifngr1	NP_034641.1	632	4	6.67	Tmod4	XP_017175125.1	497	2	4.24
Il22ra2	NP_839989.2	279	7	26.44	Scnm1	NP_001157045.1	136	4	31.00
Il20ra	NP_766374.1	341	9	27.82	Lysmd1	NP_694761.1	20	4	210.78
Slc35d3	NP_083805.1	338	3	9.35	Tnfaip8l2	NP_081482.1	346	2	6.09
Pex7	NP_001155297.1	630	4	6.69	Sema6c	NP_001258953.1	376	0	0.00
Map3k5	NP_032606.4	889	6	7.11	Gabpb2	XP_017175004.1	1013	2	2.08
Map7	NP_001185564.1	286	3	11.05	Mllt11	XP_030108588.1	513	4	8.22
Rpl32l	NP_001095031.2	1139	1	0.93	Cdc42se1	NP_765983.1	192	6	32.93
Bclaf1	NP_001020563.1	738	10	14.28	Gm128	XP_030108439.1	101	1	10.43
Mtfr2	NP_082206.2	298	5	17.68	Bnipl	NP_001161828.1	151	6	41.88
Pde7b	NP_038903.3	616	13	22.24	Prune1	XP_036018950.1	296	6	21.36

Ahi1	NP_080479.2	1154	15	13.70	Mindy1	XP_036019260.1	1334	9	7.11
Myb	NP_034978.3	2919	9	3.25	Anxa9	NP_001366475.1	358	5	14.72
Hbs1l	NP_062676.2	1344	16	12.55	Cers2	NP_001307421.1	535	4	7.88
Aldh8a1	NP_848828.1	967	12	13.08	Setdb1	XP_036019318.1	1427	1	0.74
Sgk1	NP_001155317.2	1739	12	7.27	Arnt	NP_001032826.1	570	0	0.00
4930444G20R	NP_444494.1	246	6	25.70	Ctsk	NP_031828.2	839	2	2.51
E030030I06Ril	NP_001241673.1	1648	8	5.12	Ctss	NP_001254624.2	1273	3	2.48
Raet1e	NP_001346737.1	35	2	60.22	Hormad1	NP_001360815.1	536	2	3.93
H60b	NP_001171246.1	37	2	56.97	Golph3l	NP_001171141.1	521	2	4.05
Raet1d	XP_036011793.1	81	7	91.08	Ensa	NP_062507.1	297	0	0.00
Slc2a12	XP_036011763.1	2183	5	2.41	Mcl1	XP_036018808.1	1335	2	1.58
Tbpl1	XP_006512793.1	1151	7	6.41	Adamtsl4	XP_036018952.1	389	2	5.42
Tcf21	NP_035675.1	641	8	13.15	Ecm1	XP_030108273.1	427	4	9.87
Eya4	XP_030100740.1	743	5	7.09	Tars2	XP_036019229.1	1021	1	1.03
Rps12	NP_035425.2	1285	4	3.28	Rprd2	XP_030108723.1	409	1	2.58
Slc18b1	NP_898939.2	145	0	0.00	Prpf3	NP_081817.2	961	0	0.00
Vnn3	NP_036109.3	884	10	11.92	Mrps21	NP_001342688.1	370	1	2.85
Vnn1	NP_035834.2	893	12	14.16	Ciart	XP_036018956.1	176	0	0.00
Taar1	NP_444435.1	381	2	5.53	BC028528	XP_036018959.1	76	0	0.00
Taar2	NP_001007267.1	314	2	6.71	Aph1a	NP_666246.1	326	2	6.47
Taar3	NP_001008429.1	172	2	12.25	Car14	XP_006501505.1	290	1	3.63
Taar4	NP_001008499.1	297	2	7.10	Anp32e	NP_075699.3	922	4	4.57
Taar5	NP_001009574.1	384	4	10.98	Plekho1	XP_006501993.1	248	3	12.75
Taar6	NP_001010828.1	374	5	14.09	Vps45	NP_038869.1	670	2	3.15
Taar7a	NP_001010829.1	93	3	34.00	Otud7b	XP_017175035.1	598	0	0.00
Taar7b	NP_001010827.1	190	3	16.64	Mtmr11	NP_852074.2	117	0	0.00
Taar7d	NP_001010838.1	91	3	34.74	Sf3b4	NP_694693.1	968	0	0.00
Taar7e	NP_001010835.1	136	2	15.50	Sv2a	NP_071313.1	1277	0	0.00
Taar7f	NP_001010839.1	96	1	10.98	Bola1	XP_036019163.1	332	0	0.00
Taar8a	NP_001010830.1	281	3	11.25	H2ac21	NP_835585.3	1107	7	6.66
Taar8b	NP_001010837.1	209	5	25.21	H2ac20	NP_783593.1	1097	7	6.72
Taar8c	NP_001010840.1	210	5	25.09	H2bc21	NP_835586.2	1133	8	7.44
Taar9	NP_001010831.1	278	3	11.37	H3c15	NP_473386.1	1730	7	4.26
Stx7	NP_001345492.1	1000	14	14.75	H2ac19	NP_835584.1	1084	6	5.83
Moxd1	NP_067484.2	436	3	7.25	H2ac18	NP_038577.1	940	6	6.73
Ccn2	NP_034347.2	1397	3	2.26	H3c14	NP_835734.2	1387	7	5.32
Enpp1	NP_032839.3	732	2	2.88	H4c14	NP_291074.1	1933	9	4.91
Enpp3	NP_598766.2	595	2	3.54	H3c13	NP_835587.1	1369	7	5.39
Med23	NP_001159888.1	535	1	1.97	H2bc18	NP_783597.2	1030	8	8.19
Arg1	NP_031508.1	1547	2	1.36	Fcgr1	NP_034316.1	866	0	0.00
Akap7	XP_030101016.1	356	4	11.84	BC107364	NP_001243109.1	0	0	#DIV/0!
Epb41l2	XP_036011470.1	540	3	5.86	Hjv	NP_081402.3	607	4	6.94
Smlr1	NP_001182525.1	166	0	0.00	Txnip	NP_001009935.1	1072	8	7.86
Tmem200a	XP_017169636.1	120	3	26.35	Polr3gl	NP_081517.2	255	11	45.46
Samd3	XP_006512806.1	121	3	26.13	Ankrd34a	NP_001020022.2	267	9	35.52
L3mbtl3	NP_001334396.1	392	2	5.38	Lix1l	NP_001156642.1	247	14	59.74
Arhgap18	NP_789807.1	280	2	7.53	Rbm8a	NP_001095877.1	1007	4	4.19
Gm30534	XP_006513019.1	966	2	2.18	Pex11b	NP_035199.3	340	10	31.00
Lama2	XP_017169304.1	773	4	5.45	Itga10	NP_001289400.1	382	11	30.35
Gm10145	XP_036012045.1	1647	1	0.64	Ankrd35	NP_001074608.1	713	13	19.22
Ptprk	XP_006512669.1	872	6	7.25	Pias3	NP_001159421.1	839	9	11.31
Themis	NP_848781.1	226	6	27.98	Nudt17	XP_006502380.1	113	17	158.55
2310057J18Ri	NP_080612.1	120	2	17.57	Polr3c	NP_083201.1	589	16	28.63
Soga3	NP_080414.2	503	4	8.38	Rnf115	NP_080682.3	750	17	23.89
9330159F19Ri	XP_017169358.1	57	1	18.49	Cd160	NP_001156969.1	225	6	28.10
Echdc1	XP_006512860.1	752	5	7.01	Pdzk1	XP_017175164.1	1140	8	7.40
Rnf146	NP_001103666.1	378	6	16.73	Gpr89	NP_080505.1	298	14	49.51
Rspo3	NP_082627.3	891	5	5.91	Gja8	NP_032149.1	292	11	39.70
Cenpw	NP_001103217.1	482	1	2.19	Gja5	NP_001258557.1	462	10	22.81
Trmt11	XP_006512940.1	505	2	4.17	Acp6	NP_062774.2	350	12	36.13
Hint3	NP_080074.1	241	0	0.00	Bcl9	XP_030108752.1	496	13	27.62
Ncoa7	NP_001345771.1	322	2	6.55	Olfr1402	NP_666387.1	112	2	18.82
Hey2	NP_038932.1	659	1	1.60	Chd1l	XP_036019151.1	1509	10	6.98
Hddc2	XP_030101111.1	241	6	26.24	Fmo5	NP_001155235.1	598	10	17.62
Tpd52l1	XP_030100922.1	361	1	2.92	Prkab2	XP_006500903.1	797	8	10.58
Supt4b	NP_035639.1	571	0	0.00	Pde4dip	NP_001276631.1	686	5	7.68

Rnf217	NP_001139821.1	500	2	4.22	Sec22b	NP_035472.1	1006	0	0.00
Nkain2	XP_036011781.1	539	2	3.91	Notch2	NP_035058.2	1810	5	2.91
Trdn	NP_084002.2	561	7	13.15	Adam30	NP_081941.1	233	5	22.62
Clvs2	NP_780657.1	617	9	15.37	Reg4	NP_080604.2	371	3	8.52
Sult3a2	NP_001094922.1	187	7	39.45	Hmgcs2	NP_032282.2	1287	12	9.83
Sult3a1	NP_065590.2	507	7	14.55	Phgdh	NP_058662.2	1448	4	2.91
Rsph4a	NP_001156429.1	291	8	28.97	Zfp697	NP_766451.3	220	4	19.16
Zup1	NP_001346385.1	264	8	31.94	Gm4450	XP_001480548.2	244	3	12.96
Rwdd1	NP_079890.1	329	7	22.42	Hsd3b4	XP_017174957.1	344	2	6.13
Calhm4	NP_001074634.1	108	0	0.00	Gm10681	XP_006500871.1	344	2	6.13
Trappc3l	NP_001156409.1	328	0	0.00	Hsd3b5	NP_032321.2	781	4	5.40
Calhm5	NP_849239.2	21	0	0.00	Hsd3b2	NP_001346670.1	499	6	12.67
Calhm6	NP_780658.2	405	0	0.00	Hsd3b3	XP_006501105.2	803	7	9.19
Dse	XP_036011615.1	376	7	19.62	Hsd3b6	NP_038849.2	400	5	13.17
Tspyl1	NP_033459.1	652	4	6.47	Hsd3b1	XP_006501099.1	549	2	3.84
Tspyl4	NP_084479.1	1000	3	3.16	Hao2	NP_062418.3	947	9	10.02
Nt5dc1	NP_795942.2	279	3	11.33	Wars2	XP_006502115.1	710	7	10.39
Col10a1	NP_034055.1	474	3	6.67	Tbx15	NP_033349.2	607	7	12.15
Frk	NP_001345698.1	1681	5	3.13	Spag17	NP_083168.3	341	8	24.72
Amd2	NP_031470.3	470	2	4.48	Wdr3	NP_780761.1	1185	3	2.67
Hs3st5	NP_001074677.1	299	7	24.67	Gdap2	NP_034399.1	257	3	12.30
Hdac2	NP_032255.2	3198	6	1.98	Tent5c	NP_001136424.1	353	3	8.96
Marcks	NP_032564.1	587	5	8.98	Man1a2	NP_034893.1	642	1	1.64
Rfpl4b	NP_001171254.1	481	1	2.19	Vtcn1	NP_848709.2	224	3	14.11
Lama4	NP_034811.2	680	5	7.75	Trim45	NP_001159425.1	323	0	0.00
Fam229b	NP_001346370.1	37	0	0.00	Ttf2	XP_036019253.1	1075	5	4.90
Tube1	XP_036011922.1	1417	5	3.72	Cd101	NP_001161378.1	233	4	18.09
Ccn6	XP_030100987.1	158	4	26.68	Ptgfrn	XP_017174992.1	288	7	25.62
Fyn	XP_030100741.1	2316	4	1.82	Cd2	NP_038514.1	453	5	11.63
Traf3ip2	XP_011241407.1	236	1	4.47	Igsf3	XP_006502405.1	272	2	7.75
Rev3l	XP_006512685.1	1205	8	7.00	Atp1a1	NP_659149.1	1167	6	5.42
Mfsd4b4	NP_001371154.1	107	5	49.25	Mab21l3	NP_758499.3	168	1	6.27
Mfsd4b5	NP_001106859.1	96	5	54.89	Slc22a15	NP_001034460.2	285	0	0.00
Mfsd4b1	NP_659070.2	98	5	53.77	Nhlh2	NP_848892.1	354	0	0.00
Slc16a10	NP_001107804.1	386	6	16.38	Casq2	NP_001342592.1	559	2	3.77
Rpf2	NP_075812.3	1200	8	7.03	Vangl1	NP_808213.2	453	0	0.00
Gtf3c6	NP_080389.2	191	8	44.14	Ngf	XP_006501171.1	1997	3	1.58
Amd1	NP_033795.1	544	8	15.50	Tspan2	NP_081809.2	409	3	7.73
Cdk19	NP_001161776.1	806	1	1.31	Tshb	XP_017175024.1	422	4	9.99
Slc22a16	NP_081848.1	344	3	9.19	Sycp1	NP_035646.2	465	1	2.27
Ddo	XP_036011896.1	611	5	8.62	Nr1h5	XP_006501689.1	231	1	4.56
Mettl24	XP_006512828.1	22	3	143.71	Sike1	NP_079955.1	197	0	0.00
Cdc40	NP_082155.1	801	8	10.53	Csde1	XP_006501460.1	637	6	9.93
Wasf1	NP_114083.1	969	2	2.18	Nras	XP_006501181.1	2627	8	3.21
Gpr6	NP_951013.1	688	6	9.19	Ampd1	NP_001028475.2	616	6	10.27
Fig4	NP_598760.1	655	4	6.44	Dennd2c	XP_030108515.1	226	6	27.98
Ak9	NP_001357742.1	572	0	0.00	Bcas2	NP_080878.2	891	1	1.18
Zbtb24	NP_001264158.1	509	3	6.21	Trim33	NP_444400.2	1171	2	1.80
Mical1	XP_006512633.1	717	7	10.29	Syt6	XP_006501767.1	751	6	8.42
Smpd2	NP_033239.1	469	2	4.49	Olfml3	NP_598620.2	442	11	26.23
Ppil6	NP_082706.1	1100	11	10.54	Hipk1	XP_011238326.1	887	7	8.32
Cd164	NP_058594.1	396	5	13.31	Dclre1b	NP_598626.2	621	5	8.49
Ccdc162	XP_011241532.1	38	0	0.00	Ap4b1	XP_036019138.1	816	10	12.92
Cep57l1	XP_036011364.1	85	11	136.39	Bcl2l15	NP_001136432.1	248	8	34.00
Sesn1	NP_001156380.1	543	6	11.65	Ptpn22	XP_036018851.1	1160	8	7.27
Armc2	NP_001030030.2	309	8	27.29	Rsbn1	NP_001343487.1	250	7	29.51
Foxo3	NP_001363896.1	1962	4	2.15	Phtf1	NP_038657.2	188	8	44.85
Afg1l	NP_665686.2	178	12	71.05	Magi3	NP_001152826.1	1927	9	4.92
Snx3	NP_059500.2	696	11	16.66	Lrig2	XP_036019020.1	664	4	6.35
Nr2e1	NP_689415.1	781	6	8.10	Slc16a1	NP_033222.1	554	1	1.90
Ostm1	NP_766004.1	289	11	40.11	Tafa3	XP_036019032.1	59	0	0.00
Sec63	NP_694695.3	991	12	12.76	Ppm1j	NP_082258.2	130	0	0.00
Scml4	XP_006512814.1	242	0	0.00	Rhoc	NP_031510.2	2249	4	1.87
Sobp	XP_017169252.1	342	11	33.90	Mov10	NP_001156912.1	961	4	4.39
Pdss2	NP_001161761.1	698	2	3.02	Capza1	NP_001341973.1	762	3	4.15
Bend3	NP_001346529.1	242	6	26.13	St7l	NP_694731.1	163	4	25.86

Mtres1	XP_017169558.1	123	7	59.98	Wnt2b	NP_033546.2	932	2	2.26
Cd24a	XP_036011462.1	909	2	2.32	Cttnbp2nl	NP_084525.1	243	1	4.34
Qrsl1	NP_001074523.1	852	7	8.66	4930564D02F	NP_083504.1	0	0	#DIV/0!
Rtn4ip1	NP_570962.2	432	7	17.08	Kcnd3	XP_030108559.1	1226	0	0.00
Crybg1	NP_001355234.1	146	1	7.22	Ddx20	NP_059093.3	1547	3	2.04
Atg5	NP_444299.1	1210	3	2.61	Inka2	NP_780607.2	92	0	0.00
Prdm1	XP_036011460.1	986	3	3.21	Rap1a	XP_030108244.1	2140	3	1.48
Prep	NP_035286.1	350	1	3.01	Adora3	NP_033761.2	487	1	2.16
Popdc3	XP_036012007.1	206	3	15.35	Tmigd3	NP_081301.2	453	1	2.33
Bves	NP_077247.1	331	1	3.18	I830077J02Ri	XP_006501711.1	68	0	0.00
Lin28b	XP_011241484.1	864	4	4.88	Atp5pb	NP_033855.2	866	0	0.00
Hace1	XP_006512697.1	3553	6	1.78	Wdr77	NP_081708.1	1455	1	0.72
Grik2	NP_001345795.2	1118	4	3.77	Ovgp1	NP_031722.1	222	2	9.49
Ascc3	NP_932124.2	1165	5	4.52	Pifo	NP_001186957.1	362	3	8.73
Sim1	XP_006512689.1	536	5	9.83	Chil5	XP_006502585.1	98	0	0.00
Lilr4b	NP_032173.1	271	1	3.89	Chia1	NP_075675.2	311	2	6.78
Lilrb4a	NP_038560.1	445	1	2.37	Chil3	NP_034022.2	471	1	2.24
Fam162b	NP_084170.1	119	4	35.43	Chil4	NP_660108.2	214	0	0.00
Gprc6a	NP_694711.1	400	3	7.90	Chil6	NP_848499.1	104	0	0.00
Rfx6	NP_001152861.1	663	5	7.95	Dennd2d	NP_001087223.1	188	6	33.64
Vgll2	NP_722481.1	363	2	5.81	Cept1	NP_001344748.1	446	2	4.73
Ros1	NP_035412.2	1363	6	4.64	Dram2	XP_036019134.1	220	3	14.37
Dcbld1	NP_079981.2	96	3	32.93	Lrif1	NP_001034567.1	224	2	9.41
Gopc	NP_444417.2	927	4	4.55	Cd53	NP_031677.1	725	4	5.81
Nepn	XP_006512882.1	642	0	0.00	Olfr266	NP_666700.1	32	0	0.00
Nus1	NP_084526.1	549	1	1.92	Kcna3	NP_032444.2	975	3	3.24
Slc35f1	NP_848790.2	643	4	6.56	Kcna2	NP_032443.3	1375	5	3.83
Cep85l	XP_036011337.1	127	3	24.90	Kcna10	NP_001074609.1	700	4	6.02
Pln	NP_075618.1	395	0	0.00	Cym	NP_001104613.1	382	0	0.00
Mcm9	NP_082106.3	938	1	1.12	A630076J17R	NP_001243103.1	0	0	#DIV/0!
Asf1a	NP_079817.1	1208	3	2.62	Prok1	NP_001037847.1	453	3	6.98
Fam184a	XP_036011966.1	77	1	13.69	Lamtor5	NP_081050.2	523	0	0.00
Man1a	XP_006512631.1	399	0	0.00	Slc16a4	NP_666248.1	317	2	6.65
Tbc1d32	NP_001028557.2	198	0	0.00	Rbm15	NP_001039272.1	644	2	3.27
Msl3l2	NP_001157305.1	573	1	1.84	Kcnc4	NP_001343376.1	1232	5	4.28
Gja1	XP_036011510.1	1215	2	1.73	Slc6a17	NP_758475.1	964	8	8.75
Hsf2	XP_006512627.1	827	1	1.27	Ubl4b	NP_080537.1	2771	6	2.28
Serinc1	NP_062734.1	602	2	3.50	Alx3	NP_031467.1	631	7	11.69
Pkib	XP_006512667.1	317	1	3.32	Strip1	NP_705791.2	325	4	12.97
Fabp7	NP_067247.1	699	3	4.52	Ahcyl1	NP_663517.2	1138	2	1.85
Smpdl3a	NP_065586.3	439	0	0.00	Csf1	NP_001107001.1	1293	2	1.63
Gm4981	NP_001030041.1	385	0	0.00	Eps8l3	NP_598628.1	261	3	12.11
Gcc2	NP_081651.2	937	1	1.12	Gstm5	NP_034490.1	840	1	1.25
Lims1	NP_080424.2	752	0	0.00	Gstm7	XP_006502034.1	484	1	2.18
Ranbp2	NP_035370.2	3180	3	0.99	Gstm6	NP_001366434.1	689	6	9.18
Ccdc138	NP_001156428.1	110	1	9.58	Gstm3	NP_034489.1	773	1	1.36
Edar	XP_006513269.1	441	1	2.39	Gstm2	NP_032209.1	558	1	1.89
Sh3rf3	NP_766376.2	370	3	8.55	Gstm1	NP_034488.1	546	1	1.93
Septin10	NP_001020081.2	380	1	2.77	Gstm4	XP_017174951.1	509	4	8.28
Sowahc	NP_766527.3	50	0	0.00	Ampd2	NP_083055.1	528	4	7.98
P4ha1	NP_035160.1	507	2	4.16	Gnat2	XP_036018795.1	1027	3	3.08
Pla2g12b	XP_006514124.1	751	2	2.81	Gnai3	NP_034436.1	1440	6	4.39
Oit3	NP_035089.1	340	2	6.20	Gpr61	XP_017175048.1	394	7	18.72
Mcu	NP_001028431.2	464	1	2.27	Amigo1	XP_036018969.1	236	4	17.86
Micu1	NP_001278371.1	337	1	3.13	Cyb561d1	XP_006502189.1	102	1	10.33
Dnajb12	NP_001361685.1	524	0	0.00	Atxn7l2	XP_006502200.1	52	3	60.80
Ddit4	NP_083359.1	710	1	1.48	Sypl2	NP_032622.1	189	9	50.19
Anapc16	NP_001334380.1	382	0	0.00	Psma5	NP_036097.1	1344	5	3.92
Ascc1	XP_006514102.1	218	2	9.67	Sort1	XP_006501286.3	876	7	8.42
Spock2	NP_443720.1	823	2	2.56	Mybphl	NP_081107.1	136	5	38.75
Chst3	XP_017169519.1	236	2	8.93	Psrc1	NP_001177090.1	447	8	18.86
Psap	NP_035309.3	701	2	3.01	Celsr2	XP_017175138.1	909	12	13.91
Cdh23	XP_017169419.1	763	0	0.00	Sars	NP_001191908.1	1065	0	0.00
Vsir	NP_083008.1	274	0	0.00	Elapor1	NP_001333447.1	442	8	19.08
Gm17455	XP_006513024.2	6	0	0.00	1700013F07R	NP_083590.1	143	11	81.07
Slc29a3	XP_006514182.1	393	3	8.05	Tmem167b	NP_080474.2	96	15	164.67

Unc5b	NP_001346202.1	522	6	12.11	Taf13	NP_079720.1	523	13	26.20
Pcbd1	NP_079549.1	554	5	9.51	Wdr47	XP_030108792.1	200	14	73.77
Sgpl1	XP_017169349.1	753	8	11.20	Clcc1	XP_036018970.1	204	16	82.66
Tbata	NP_001017433.1	131	1	8.05	Gpsm2	NP_083798.2	741	17	24.18
Adamts14	NP_001074596.1	319	4	13.22	Aknad1	NP_808527.2	87	17	205.93
Prf1	NP_035203.3	873	1	1.21	Stxbp3	NP_035634.1	807	4	5.22
Pald1	NP_038781.2	157	3	20.14	Fndc7	XP_036019025.1	58	14	254.39
Nodal	NP_038639.2	688	4	6.13	Prpf38b	NP_080121.1	520	13	26.35
Eif4ebp2	NP_034254.1	666	4	6.33	Henmt1	NP_001072114.1	253	14	58.32
Lrrc20	NP_705770.1	444	6	14.24	Fam102b	XP_036019034.1	141	14	104.64
Npffr1	NP_001170982.1	442	5	11.92	Slc25a54	NP_083330.1	130	11	89.18
Ppa1	NP_080714.2	1377	2	1.53	Slc25a24	NP_766273.1	442	10	23.84
Sar1a	NP_001345417.1	757	0	0.00	Vav3	NP_001365916.1	952	9	9.96
Tysnd1	NP_082188.1	629	2	3.35	Ntng1	NP_001156821.1	1024	9	9.26
Aifm2	NP_722474.2	759	2	2.78	Prmt6	NP_849222.3	1205	1	0.87
Macroh2a2	NP_996883.1	1215	1	0.87	Amy2b	NP_001177333.1	327	5	16.11
Col13a1	XP_036011464.1	380	7	19.41	Amy2a5	NP_001036176.1	388	1	2.72
Fam241b	NP_001345181.1	23	6	274.93	Amy2a4	NP_001153622.1	388	1	2.72
Neurog3	NP_033849.3	509	3	6.21	Amy2a3	NP_001153623.1	388	1	2.72
Tspan15	NP_932113.2	148	10	71.21	Amy2a2	NP_001153624.1	387	0	0.00
Tacr2	NP_033340.3	356	11	32.56	Amy2a1	XP_036019386.1	388	1	2.72
Hk1	XP_006513305.1	1090	1	0.97	Amy1	NP_031472.2	453	0	0.00
Hkdc1	NP_663394.1	824	9	11.51	Rnpc3	NP_001273944.1	939	2	2.24
Supv3l1	NP_852088.1	342	11	33.90	Col11a1	NP_031755.2	694	2	3.04
Vps26a	NP_598433.1	813	12	15.56	Olfm3	NP_703188.1	764	2	2.76
Srgn	XP_006513437.1	688	3	4.60	S1pr1	NP_031927.2	823	8	10.24
Kifbp	NP_082473.2	247	0	0.00	Dph5	NP_081469.2	711	9	13.34
Ddx21	NP_062426.2	2025	11	5.72	Slc30a7	NP_075703.1	396	9	23.95
Ddx50	XP_006514408.1	1682	14	8.77	Extl2	NP_067363.3	237	0	0.00
Stox1	NP_001028432.1	276	12	45.82	Vcam1	NP_035823.3	1559	9	6.08
Ccar1	XP_036011856.1	1041	9	9.11	Gpr88	NP_071872.1	570	10	18.49
Tet1	NP_001240786.1	634	7	11.64	Cdc14a	NP_001167024.1	1048	8	8.05
Slc25a16	NP_780403.1	598	8	14.10	Rtca	NP_079793.2	857	9	11.07
Dna2	NP_796346.2	1347	11	8.61	Dbt	NP_034152.2	691	9	13.73
Rufy2	NP_081701.2	297	9	31.94	Lrrc39	NP_780622.1	581	10	18.14
Hnrnph3	NP_001334386.1	580	5	9.09	Trmt13	XP_030108459.1	182	5	28.95
Pbld2	XP_006514044.1	308	11	37.64	Sass6	XP_006502203.1	556	0	0.00
Pbld1	XP_006514092.1	308	11	37.64	Mfsd14a	NP_032272.2	414	3	7.64
Atoh7	NP_001351577.1	561	8	15.03	Slc35a3	XP_006501488.1	640	4	6.59
Mypn	NP_892037.2	480	6	13.17	Agl	NP_001074795.1	787	0	0.00
Herc4	NP_084390.1	1125	4	3.75	Frrs1	NP_033172.2	391	6	16.17
Sirt1	NP_001153061.1	2728	6	2.32	Palmd	NP_075734.3	215	7	34.31
Dnajc12	NP_038916.1	415	3	7.62	Plppr4	NP_808332.3	343	7	21.51
Ctnna3	NP_808280.2	643	1	1.64	Plppr5	NP_001292380.1	247	6	25.60
Lrrtm3	NP_848793.1	876	2	2.41	Snx7	NP_001177085.1	557	6	11.35
Reep3	NP_848721.1	950	2	2.22	Dpyd	NP_740748.1	1569	1	0.67
Jmjd1c	XP_006513098.1	823	5	6.40	Ptbp2	NP_001297640.1	956	5	5.51
Nrbf2	XP_006514001.1	68	0	0.00	Rwdd3	NP_082732.2	48	0	0.00
Egr2	XP_030100730.1	1322	5	3.99	Tlcd4	XP_030108816.1	203	4	20.77
Ado	NP_001005419.2	245	3	12.90	Alg14	XP_006501962.1	211	0	0.00
Zfp365	NP_848794.1	402	2	5.24	Cnn3	NP_082320.1	738	3	4.28
Rtkn2	NP_001346247.1	206	5	25.58	Slc44a3	NP_663369.2	270	1	3.90
Arid5b	NP_076087.2	835	4	5.05	4930432M17I	NP_001028986.1	0	0	#DIV/0!
Cabcoco1	NP_082767.1	124	3	25.50	F3	NP_034301.3	761	2	2.77
Tmem26	NP_808462.2	167	3	18.93	Abcd3	NP_033017.2	551	4	7.65
A930033H14R	NP_001318122.1	0	0	#DIV/0!	Arhgap29	XP_006501353.1	668	2	3.16
Rhobtb1	NP_001074816.1	1677	5	3.14	Abca4	NP_031404.1	564	3	5.61
Cdk1	NP_031685.2	3527	7	2.09	Gclm	NP_032155.1	548	0	0.00
Ank3	XP_036011445.1	2476	5	2.13	Dnttip2	NP_722501.1	714	0	0.00
Ccdc6	NP_001104591.1	401	2	5.26	Bcar3	NP_038895.1	315	3	10.04
Slc16a9	NP_080083.2	416	1	2.53	Fnbp1l	NP_694758.2	670	1	1.57
Fam13c	NP_077206.3	96	0	0.00	Pde5a	NP_700471.2	672	1	1.57
Phyhipl	NP_848736.2	579	2	3.64	Fabp2	NP_032006.1	1075	0	0.00
Bicc1	XP_036012017.1	859	1	1.23	1810037I17Ri	NP_077781.1	76	0	0.00
Tfam	XP_017169407.1	1439	4	2.93	Usp53	XP_017175309.1	250	1	4.22
Ube2d1	XP_030100873.1	2025	3	1.56	Myoz2	NP_067478.1	417	1	2.53

Cisd1	NP_598768.1	430	1	2.45	Synpo2	NP_536699.2	875	1	1.20
Ipmk	NP_001334120.1	866	1	1.22	Sec24d	XP_030108669.1	688	2	3.06
Zwint	NP_079911.1	843	2	2.50	Mettl14	NP_964000.2	310	1	3.40
Pcdh15	NP_001136218.1	680	1	1.55	Prss12	NP_032965.1	388	3	8.15
Rsph14	NP_082006.1	185	2	11.39	Ndst3	XP_006502433.1	209	2	10.09
Gnaz	NP_001345777.1	1070	3	2.95	Tram1l1	NP_666252.1	270	2	7.81
Rab36	NP_001346199.1	696	3	4.54	1700006A11F	NP_082215.1	128	1	8.23
Bcr	NP_001074881.1	615	1	1.71	1700003H04F	NP_001355164.1	188	1	5.61
Specc1l	NP_001139298.1	642	4	6.57	Ndst4	NP_072087.2	314	2	6.71
Adora2a	NP_001318025.1	916	2	2.30	Ugt8a	NP_035804.2	369	1	2.86
Upb1	NP_598756.1	715	2	2.95	Arsj	NP_775627.1	266	4	15.85
Gucd1	NP_780342.1	25	6	252.94	Camk2d	XP_006500892.1	1013	2	2.08
Snrpd3	NP_080371.1	1575	3	2.01	Ank2	XP_036018748.1	2331	3	1.36
Lrrc75b	NP_942560.1	24	8	351.30	Larp7	NP_613059.2	1487	1	0.71
Ggt1	NP_001366467.1	1339	7	5.51	Zgrf1	XP_017175214.1	1073	8	7.86
Ggt5	NP_035950.2	336	6	18.82	Neurog2	XP_030108254.1	611	6	10.35
Susd2	NP_001156385.1	200	3	15.81	Alpk1	XP_006502164.1	227	7	32.50
Cabin1	XP_006513083.1	406	2	5.19	Tifa	XP_036018886.1	226	5	23.32
Ddt	NP_034157.1	422	4	9.99	Ap1ar	XP_006501305.1	189	6	33.46
Gstt3	NP_598755.1	889	3	3.56	Fam241a	NP_081758.1	60	8	140.52
Gstt1	NP_032211.3	1094	4	3.85	Pitx2	NP_035228.2	1070	5	4.92
Gstt4	NP_083748.3	692	2	3.05	Enpep	NP_031960.1	1051	5	5.01
Gstt2	XP_006513304.1	913	4	4.62	Elovl6	NP_569717.1	706	4	5.97
Mif	NP_034928.1	833	3	3.80	Egf	XP_006501053.1	3750	4	1.12
Derl3	NP_077760.1	792	1	1.33	Lrit3	NP_001274153.1	1081	2	1.95
Smarcb1	NP_001155325.1	1340	2	1.57	Rrh	NP_033128.1	236	7	31.26
Mmp11	NP_032632.1	517	0	0.00	Gar1	NP_080854.1	1066	7	6.92
Chchd10	XP_036011348.1	368	0	0.00	Cfi	NP_031712.2	1085	7	6.80
Vpreb3	XP_030100932.1	141	0	0.00	Pla2g12a	NP_075685.2	339	8	24.87
Gm5134	XP_006513854.1	59	1	17.86	Casp6	NP_033941.3	738	1	1.43
Zfp280b	NP_803426.2	274	3	11.54	Mcub	XP_006501963.1	233	5	22.62
Slc5a4b	NP_075708.2	185	2	11.39	Sec24b	XP_006502532.1	783	6	8.08
Slc5a4a	NP_573447.2	193	2	10.92	Col25a1	XP_006502329.1	260	6	24.32
Prmt2	NP_573445.2	1183	7	6.24	Etnppl	NP_082183.2	678	3	4.66
S100b	NP_033141.1	1020	6	6.20	Ostc	NP_079785.1	328	1	3.21
Dip2a	XP_006514003.1	472	9	20.10	Rpl34	XP_036019156.1	918	3	3.44
Pcnt	XP_030100810.1	950	5	5.55	Lef1	NP_001365988.1	1640	2	1.29
2610028H24R	NP_084092.2	74	4	56.97	Hadh	NP_032238.2	784	2	2.69
Ybey	NP_766138.3	396	8	21.29	Cyp2u1	NP_082092.2	456	4	9.24
Mcm3ap	NP_062307.2	709	12	17.84	Sgms2	NP_001366515.1	324	2	6.51
Lss	NP_666118.1	761	4	5.54	Papss1	NP_035993.1	470	2	4.48
Spatc1l	XP_006514342.1	269	3	11.75	Dkk2	XP_006501881.1	459	1	2.30
Ftcd	NP_543121.1	834	14	17.69	Gimd1	NP_001359070.1	15	0	0.00
Col6a2	NP_666119.1	694	6	9.11	Aimp1	NP_031952.2	1284	3	2.46
Col6a1	NP_034063.1	877	6	7.21	Tbck	NP_001156927.1	281	6	22.50
Pcbp3	XP_006513989.1	803	10	13.12	Npnt	XP_006500978.1	587	4	7.18
Slc19a1	NP_112473.1	448	7	16.47	Gstcd	NP_001343238.1	673	3	4.70
Col18a1	XP_036011465.1	821	9	11.55	Ints12	NP_082203.1	327	3	9.67
Pofut2	NP_084538.2	289	9	32.82	Arhgef38	NP_001355681.1	392	4	10.75
Adarb1	XP_006513126.1	575	5	9.16	Ppa2	NP_666253.1	1246	3	2.54
Fam207a	XP_030100663.1	62	0	0.00	Tet2	XP_006501350.1	768	4	5.49
Itgb2	NP_032430.2	1660	3	1.90	Cxxc4	XP_030108502.1	197	9	48.15
Pttg1ip	NP_666037.1	184	2	11.46	Tacr3	NP_067357.1	592	8	14.24
Sumo3	NP_001288602.1	2189	4	1.93	Cenpe	NP_776123.3	1590	8	5.30
Ube2g2	NP_062777.2	720	1	1.46	Bdh2	NP_001165526.1	523	9	18.14
Tspear	NP_001274003.1	205	0	0.00	Slc9b2	NP_849208.4	418	10	25.21
Gm19402	NP_001191961.1	138	10	76.37	Slc9b1	XP_017175264.1	320	8	26.35
Gm9508	NP_001357746.1	114	9	83.20	Cisd2	NP_080178.1	363	10	29.03
Gm10272	NP_001371137.1	127	12	99.58	Ube2d3	NP_001343525.1	2328	11	4.98
Gm10024	NP_001074921.1	93	12	135.99	Manba	NP_081564.3	500	12	25.29
Gm10142	NP_001191964.1	83	14	177.77	Nfkb1	NP_032715.2	2281	10	4.62
Krtap12-1	NP_034800.1	180	14	81.97	Slc39a8	NP_080504.3	453	11	25.59
Gm10100	NP_001191962.1	88	16	191.62	Bank1	NP_001297678.1	383	14	38.52
Gm9736	NP_001357737.1	114	15	138.67	Ppp3ca	NP_032939.1	3454	11	3.36
Gm3233	NP_001357760.1	114	16	147.92	Emcn	NP_001156994.1	436	1	2.42
Gm3238	NP_001095100.1	118	19	169.70	Gm4861	XP_030108461.1	2	0	0.00

Gm7138	XP_006514415.1	117	18	162.14	Ddit4l	NP_084419.2	433	11	26.77
Gm3250	NP_001357762.1	108	19	185.41	H2az1	NP_058030.1	2361	9	4.02
Gm7137	XP_001477836.1	116	17	154.45	Dnajb14	NP_001028327.1	620	8	13.60
Gm9639	NP_001357753.1	117	15	135.12	Lamtor3	NP_064304.1	224	2	9.41
Gm19668	NP_001191963.1	133	16	126.78	Dapp1	NP_001297682.1	471	9	20.14
Gm9507	NP_001357745.1	114	15	138.67	1110002E22R	NP_001357791.1	61	0	0.00
Gm2696	NP_001191938.1	116	13	118.11	Mttp	NP_001156929.1	567	12	22.30
Krtap10-4	NP_001129463.1	179	11	64.76	Trmt10a	NP_001335125.1	510	2	4.13
Krtap10-10	NP_001019880.2	179	10	58.88	4930579F01R	NP_001156857.1	27	2	78.07
Gm10318	NP_001156416.1	112	9	84.69	Adh7	NP_033756.2	724	5	7.28
Gm3285	NP_001095101.1	117	8	72.06	Adh1	NP_031435.1	1005	5	5.24
Lrrc3	NP_660134.1	525	0	0.00	Adh6a	NP_081221.1	268	2	7.86
Trpm2	XP_036011693.1	440	0	0.00	Adh6b	NP_001357786.1	245	1	4.30
Cfap410	XP_006514071.1	111	2	18.99	Adh4	XP_030108487.1	1015	3	3.11
Pfkl	NP_032852.2	1127	8	7.48	Adh5	NP_031436.2	660	1	1.60
Aire	NP_001258484.1	1418	8	5.95	Metap1	NP_780433.1	1189	11	9.75
Dnmt3l	NP_001075164.1	578	2	3.65	Eif4e	XP_006501056.1	1866	4	2.26
Icosl	XP_036011782.1	504	2	4.18	Tspan5	NP_062517.1	296	3	10.68
Gatd3a	NP_001351575.1	405	5	13.01	Rap1gds1	NP_001343317.1	449	2	4.69
Pwp2	NP_083822.1	1067	4	3.95	Gm4862	XP_036019407.1	769	0	0.00
Trappc10	NP_001074524.1	511	5	10.31	Stpg2	NP_941061.1	204	1	5.17
Agpat3	XP_030100970.1	592	1	1.78	Pdha2	NP_032837.1	949	0	0.00
Rrp1	NP_035055.2	828	3	3.82	Unc5c	NP_001280490.1	548	3	5.77
Cstb	NP_031819.1	435	5	12.11	Bmpr1b	NP_001341972.1	1595	1	0.66
Pdxk	NP_742146.1	688	5	7.66	Pdlim5	XP_017175148.1	1035	3	3.05
Ilvbl	NP_001346230.1	563	6	11.23	Gbp5	NP_705792.2	543	6	11.65
Syde1	NP_082151.1	623	4	6.77	Gbp7	NP_663520.2	387	5	13.62
Olfr1357	NP_001011737.1	52	0	0.00	Gbp3	XP_006501782.1	472	5	11.16
Casp14	NP_033939.1	638	3	4.96	Gbp2b	NP_034389.2	541	4	7.79
Ccdc105	NP_081906.1	70	4	60.22	Gbp2	NP_034390.1	513	5	10.27
Slc1a6	XP_006513485.3	529	3	5.98	Kyat3	NP_001280489.1	519	7	14.21
Olfr1356	NP_666420.2	61	0	0.00	Gtf2b	NP_663521.1	1626	4	2.59
Olfr1355	NP_997454.1	42	0	0.00	Pkn2	NP_848769.2	1633	7	4.52
Olfr1354	NP_001186769.1	36	0	0.00	Lmo4	NP_001155241.1	514	0	0.00
Olfr8	NP_997084.1	70	0	0.00	Hs2st1	NP_035958.3	233	0	0.00
Olfr1353	NP_667253.1	36	0	0.00	Selenof	NP_444332.1	302	2	6.98
Olfr1352	NP_667282.1	42	0	0.00	Sh3glb1	NP_001366102.1	417	4	10.11
Olfr1351	NP_667251.1	39	0	0.00	Clca3a1	NP_034029.2	341	2	6.18
Olfr57	NP_667252.1	44	0	0.00	Clca3a2	XP_017175301.1	273	2	7.72
2610008E11R	NP_001346644.1	209	0	0.00	Clca3b	NP_631887.2	224	2	9.41
Vmn2r80	NP_001096838.1	11	0	0.00	Clca4b	NP_001028371.1	160	4	26.35
Vmn2r81	NP_787950.1	40	0	0.00	Clca4a	NP_997091.3	247	4	17.07
Vmn2r82	NP_001095042.1	27	0	0.00	Clca1	NP_059502.1	411	3	7.69
Vmn2r83	NP_001098007.1	24	0	0.00	Clca2	NP_848812.1	84	3	37.64
Plpp2	NP_056632.2	499	7	14.78	Odf2l	XP_006501738.1	120	7	61.48
Mier2	NP_081698.2	340	4	12.40	Col24a1	NP_082046.2	353	0	0.00
Theg	NP_954696.1	121	9	78.39	Znhit6	NP_001074563.1	537	4	7.85
C2cd4c	NP_001162095.1	165	12	76.65	Ccn1	NP_034646.1	895	3	3.53
Shc2	NP_001019710.1	418	11	27.73	Ddah1	NP_081269.1	354	3	8.93
Odf3l2	NP_001028645.1	133	14	110.94	Bcl10	NP_033870.1	647	2	3.26
Madcam1	NP_038619.2	350	7	21.08	2410004B18R	NP_079831.1	78	5	67.56
Tpgs1	NP_683736.2	168	7	43.91	Syde2	XP_006501355.3	245	2	8.60
Cdc34	XP_017169376.1	2040	8	4.13	Wdr63	NP_766452.2	264	0	0.00
Gzmm	NP_032530.1	374	10	28.18	Mcoln3	NP_598921.1	364	1	2.90
Bsg	NP_033898.1	969	10	10.88	Mcoln2	NP_080932.2	343	1	3.07
Hcn2	NP_032252.1	689	5	7.65	Lpar3	NP_075359.1	654	2	3.22
Polrmt	NP_766139.2	726	14	20.32	Ssx2ip	NP_001342590.1	366	0	0.00
Fgf22	XP_030101088.1	647	11	17.92	Ctbs	NP_001280601.1	288	3	10.98
Rnf126	NP_653111.1	766	17	23.39	Spata1	XP_036019210.1	74	2	28.48
Fstl3	NP_113557.1	439	11	26.41	Gng5	NP_034448.2	782	5	6.74
Prss57	XP_006514263.1	345	10	30.55	Rpf1	NP_081647.2	980	0	0.00
Palm	NP_075617.3	249	1	4.23	Dnase2b	NP_064341.3	761	1	1.38
Misp	NP_084494.1	139	1	7.58	Uox	NP_033500.1	868	1	1.21
Ptbp1	NP_001070831.1	1229	8	6.86	4930503B20R	NP_001355654.1	388	1	2.72
Plppr3	NP_001164406.1	259	16	65.11	Prkacb	NP_035230.1	3800	2	0.55
Prtn3	NP_035308.2	655	8	12.87	Ttll7	XP_036019207.1	542	5	9.72

Elane	NP_056594.2	1068	10	9.87	Adgrl2	XP_036019339.1	848	6	7.46
Cfd	NP_038487.1	698	4	6.04	Adgrl4	NP_573485.2	585	5	9.01
Med16	NP_001156748.1	532	13	25.75	Ifi44	NP_598632.2	436	0	0.00
R3hdm4	NP_818775.2	306	8	27.55	Ifi44l	NP_112735.1	349	0	0.00
Kiss1r	NP_444474.1	515	8	16.37	Ptgfr	NP_032992.1	455	4	9.27
Arid3a	NP_001275554.1	693	11	16.73	Gipc2	NP_058563.1	400	6	15.81
Wdr18	NP_780659.2	1183	16	14.25	Dnajb4	NP_001343292.1	846	2	2.49
Grin3b	XP_006513350.1	429	10	24.57	Fubp1	XP_006501723.1	847	0	0.00
Tmem259	XP_006513554.1	267	11	43.42	Nexn	XP_011238518.1	495	0	0.00
Cnn2	NP_031751.1	838	10	12.58	Miga1	NP_001155847.1	150	6	42.16
Abca7	NP_001334010.1	374	7	19.73	Usp33	NP_573510.2	906	1	1.16
Arhgap45	NP_001136173.1	941	16	17.92	Zzz3	XP_030108212.1	293	1	3.60
Polr2e	NP_079830.2	1437	14	10.27	Ak5	XP_036018983.1	894	0	0.00
Gpx4	NP_032188.3	1124	10	9.38	Pigk	XP_036019035.1	449	0	0.00
Sbno2	XP_036011630.1	376	18	50.45	St6galnac5	NP_036158.3	477	2	4.42
Stk11	NP_035622.1	1856	6	3.41	St6galnac3	XP_030108371.1	250	7	29.51
Cbarp	XP_006513030.1	113	4	37.31	Asb17	NP_080034.2	339	2	6.22
Atp5d	XP_017169541.1	1228	15	12.87	Msh4	NP_114076.1	735	4	5.74
Midn	NP_067540.1	220	18	86.23	Rabggtb	NP_001156950.1	600	3	5.27
Cirbp	NP_001366352.1	1675	14	8.81	Acadm	NP_031408.1	894	5	5.89
Fam174c	NP_081483.1	231	9	41.06	Slc44a5	XP_006501552.1	290	6	21.80
Efna2	NP_031935.3	464	9	20.44	Lhx8	NP_034843.2	781	1	1.35
Pwwp3a	XP_030101106.1	96	0	0.00	Tyw3	NP_766062.1	247	3	12.80
Ndufs7	XP_030101204.1	963	14	15.32	Cryz	NP_001344601.1	751	5	7.02
Gamt	NP_034385.1	659	12	19.19	Erich3	NP_780385.1	62	0	0.00
Dazap1	XP_006514133.1	742	14	19.88	Tnni3k	NP_796040.3	989	3	3.20
Rps15	NP_033117.1	1436	11	8.07	Fpgt	NP_083606.2	321	3	9.85
Apc2	NP_001359167.1	761	1	1.38	Lrriq3	NP_083214.2	588	3	5.38
2310011J03Ri	NP_079797.1	55	13	249.10	Negr1	XP_036019026.1	1180	5	4.47
Pcsk4	XP_006513428.1	680	16	24.80	Zranb2	NP_059077.1	794	1	1.40
Reep6	XP_006514140.1	1118	16	15.08	Ptger3	NP_035326.2	673	0	0.00
Adamtsl5	XP_011241828.1	140	14	105.39	Cth	XP_006500878.1	1154	1	1.07
Plk5	XP_006513561.1	1424	14	10.36	Ankrd13c	NP_001013828.1	121	0	0.00
Mex3d	XP_006513682.1	172	14	85.78	Srsf11	NP_001366461.1	731	1	1.92
Mbd3	NP_001293072.1	654	8	12.89	Lrrc40	NP_077156.2	585	1	2.57
Uqcr11	NP_079926.1	661	11	17.54	Lrrc7	XP_006501575.1	1237	1	1.31
Tcf3	NP_035678.3	1176	0	0.00	Depdc1a	NP_001165563.1	421	0	0.00
Onecut3	NP_631972.2	275	9	34.49	Rpe65	XP_006501228.1	569	0	0.00
Atp8b3	XP_006514047.1	513	9	18.49	Wls	NP_001343279.1	476	0	0.00
Rexo1	NP_080128.2	478	12	26.46	Vmn1r2	NP_001161006.1	12	0	0.00
Klf16	NP_510962.2	434	1	2.43	Vmn1r3	NP_001161007.1	2	0	0.00
Abhd17a	XP_030100883.1	325	5	16.21	Tmem68	NP_082373.1	226	7	54.40
Adat3	NP_001094076.1	393	0	0.00	Tgs1	NP_473430.3	367	6	26.51
Scamp4	NP_062521.1	171	4	24.65	Lyn	NP_001104566.1	1884	7	5.59
Csnk1g2	NP_598763.1	971	5	5.43	Rps20	NP_080423.1	1543	10	9.11
Btbd2	NP_663336.3	253	3	12.50	Mos	NP_064405.2	1273	4	4.14
Mknk2	XP_006513379.1	460	4	9.16	Plag1	XP_036020164.1	713	12	20.87
Mob3a	XP_036011609.1	197	0	0.00	Chchd7	XP_017175838.1	289	15	60.78
Izumo4	XP_017169585.1	172	0	0.00	Sdr16c5	NP_871789.1	426	12	31.25
Ap3d1	XP_030100710.1	812	1	1.30	Sdr16c6	NP_001074179.1	344	14	42.89
Dot1l	NP_955354.1	920	5	5.73	Penk	NP_001335138.1	922	14	16.00
Plekhj1	XP_030101255.1	230	2	9.16	Bpnt2	NP_808398.1	572	5	9.21
Sf3a2	NP_038679.3	1227	4	3.44	Fam110b	NP_001342645.1	194	11	59.76
Amh	NP_031471.2	785	5	6.71	Ubxn2b	NP_080810.2	357	7	20.66
Jsrp1	XP_036011921.1	181	2	11.65	Cyp7a1	NP_031850.2	1069	3	2.96
Oaz1	NP_032779.2	467	3	6.77	Sdcbp	NP_001091697.1	1008	10	10.46
Lingo3	NP_001346675.1	641	6	9.86	Nsmaf	NP_035075.2	435	8	19.38
Lsm7	NP_079625.1	870	5	6.06	Tox	NP_001364008.1	790	12	16.01
Sppl2b	XP_006514272.1	255	1	4.13	Car8	NP_031618.2	413	6	15.31
Tmprss9	XP_006513906.2	252	1	4.18	Gm11810	XP_036020500.1	1002	0	0.00
Timm13	NP_038923.1	1087	3	2.91	Rab2a	NP_067493.1	1711	7	4.31
Lmnb2	NP_034852.3	1000	4	4.22	Chd7	NP_001264078.1	2007	7	3.68
Gadd45b	NP_032681.1	626	1	1.68	Clvs1	XP_011248428.1	593	4	7.11
Gng7	XP_036011511.1	1995	4	2.11	Asph	NP_001171320.1	462	7	15.97
Diras1	NP_660252.1	2177	5	2.42	Gdf6	NP_038554.1	541	1	1.95
Slc39a3	XP_030100662.1	294	1	3.58	2610301B20R	NP_080281.3	92	1	11.46

Sgta	NP_001345479.1	1191	4	3.54	Plekhf2	NP_780384.1	242	1	4.35
Thop1	NP_073144.3	639	1	1.65	Ndufaf6	XP_030109763.1	685	0	0.00
Creb3l3	NP_001369747.1	911	2	2.31	Trp53inp1	NP_068697.1	424	2	4.97
Map2k2	NP_075627.2	1474	10	7.15	Ccne2	NP_001269872.1	1992	4	2.12
Zbtb7a	XP_006513342.3	578	6	10.94	Ints8	NP_835213.3	307	3	10.30
Pias4	NP_067476.2	838	7	8.80	Dpy19l4	NP_001074670.1	336	2	6.27
Eef2	NP_031933.1	2668	6	2.37	Esrp1	XP_006537779.1	539	3	5.87
Dapk3	NP_001177403.2	958	9	9.90	Virma	NP_001074652.1	258	1	4.08
Nmrk2	NP_081396.1	1060	7	6.96	Rad54b	NP_001034645.1	1510	4	2.79
Atcay	NP_848777.1	1553	10	6.79	Fsbp	NP_001243071.1	88	1	11.98
Zfr2	NP_001030067.2	390	11	29.73	Gem	NP_034406.2	1440	1	0.73
Matk	XP_011241678.1	1488	8	5.67	Cdh17	XP_006537646.1	756	1	1.39
Mrpl54	NP_079593.1	569	4	7.41	Pdp1	NP_001277316.1	1055	0	0.00
Apba3	XP_036011794.1	315	4	13.38	Tmem67	XP_006538070.3	527	0	0.00
Tjp3	NP_001269025.1	446	3	7.09	Rbm12b2	XP_017175942.1	251	1	4.20
Pip5k1c	NP_001280575.1	770	4	5.47	Rbm12b1	XP_030109670.1	295	0	0.00
Cactin	NP_081657.1	617	2	3.42	Cibar1	NP_001297672.1	74	0	0.00
Tbxa2r	NP_001345441.1	513	3	6.16	Triqk	NP_776107.2	18	0	0.00
Gipc3	XP_030100839.1	397	3	7.96	Runx1t1	NP_001104496.1	532	1	1.98
Hmg20b	XP_006513311.1	742	1	1.42	Slc26a7	NP_666059.2	406	0	0.00
Mfsd12	XP_006514282.1	176	2	11.98	Lrrc69	XP_030109691.1	439	0	0.00
4930404N11R	NP_001014836.2	21	1	50.19	Otud6b	XP_036020347.1	531	1	1.98
Fzr1	NP_062731.1	2412	2	0.87	Pip4p2	NP_082540.1	213	0	0.00
Dohh	NP_598725.2	768	1	1.37	Necab1	XP_006538297.1	178	3	17.76
Smim24	NP_001093387.1	73	1	14.44	Tmem64	NP_852066.2	217	0	0.00
Nfic	XP_030100800.1	444	1	2.37	Calb1	NP_033918.1	2198	4	1.92
Celf5	XP_006513806.1	882	0	0.00	Decr1	NP_080448.1	2579	4	1.63
Ncln	XP_017169251.1	313	0	0.00	Nbn	NP_038780.3	736	1	1.43
S1pr4	NP_034232.1	512	2	4.12	Osgin2	NP_666062.3	193	4	21.84
Gna15	XP_006513297.1	941	7	7.84	Ripk2	XP_006537740.1	1279	3	2.47
Gna11	NP_034431.1	1180	7	6.25	Mmp16	NP_001366447.1	535	2	3.94
Tle5	NP_034477.1	624	2	3.38	Cnbd1	NP_001358198.1	119	5	44.28
Tle2	XP_017169412.1	564	3	5.61	Cngb3	NP_038955.1	374	3	8.45
Tle6	NP_001333624.1	508	3	6.22	Cpne3	NP_082045.1	389	6	16.26
Sirt6	NP_001365873.1	1104	3	2.86	Rmdn1	NP_079752.3	269	3	11.75
Ankrd24	XP_006514156.1	717	5	7.35	Wwp1	NP_796301.2	1467	7	5.03
Gm10778	NP_001136435.1	126	0	0.00	Slc7a13	NP_083022.1	307	5	17.16
Zfp433	NP_001229996.1	165	0	0.00	Atp6v0d2	NP_780615.2	1164	3	2.72
Gm3055	NP_001363914.1	260	0	0.00	Ttpa	XP_036020118.1	772	0	0.00
Gm4767	XP_011241947.1	127	0	0.00	Ggh	NP_034411.2	674	1	1.56
Zfp781	NP_001371116.1	260	0	0.00	Nkain3	XP_006538014.1	84	0	0.00
Gm8290	XP_036012032.1	1167	0	0.00	Prdm13	NP_001074240.1	335	7	22.02
Zfp873	XP_011241806.1	160	0	0.00	Ccnc	NP_001277349.1	971	7	7.60
BC024063	NP_001360962.1	260	0	0.00	Tstd3	NP_084116.1	307	0	0.00
AU041133	NP_001156536.1	126	0	0.00	Usp45	NP_690038.1	582	6	10.86
Zfp938	NP_001099027.2	126	0	0.00	Pnisr	NP_079945.1	593	8	14.22
LOC10263599	XP_006514425.1	52	0	0.00	Coq3	NP_766275.1	712	7	10.36
Gm4924	XP_017169670.1	133	0	0.00	Faxc	NP_780443.2	112	8	75.28
Gm1553	NP_001242919.1	70	0	0.00	Fbxl4	NP_766576.1	879	9	10.79
1190007I07Ril	NP_001129039.1	27	0	0.00	Pou3f2	NP_032925.1	734	9	12.92
Tdg	NP_766140.2	567	0	0.00	Mms22l	XP_036019830.1	434	4	9.71
Glt8d2	XP_030101195.1	174	0	0.00	Klhl32	XP_036019834.1	122	4	34.55
Hcfc2	XP_030101101.1	649	0	0.00	Ndufaf4	NP_081018.1	435	1	2.42
Nfyb	NP_035044.1	1756	0	0.00	Gpr63	NP_001366555.1	335	5	15.73
Txnrd1	NP_001035988.1	1212	0	0.00	Fhl5	NP_067293.1	562	1	1.88
Chst11	NP_067414.2	356	1	2.96	Ufl1	NP_001342441.1	573	6	11.04
Slc41a2	NP_796362.1	276	0	0.00	Fut9	NP_034373.1	654	5	8.06
D10Wsu102e	NP_080855.1	43	0	0.00	Manea	NP_766453.2	297	5	17.74
Aldh1l2	NP_705771.2	597	1	1.77	Epha7	XP_006537670.1	1578	5	3.34
Washc4	XP_036011700.1	470	1	2.24	Map3k7	NP_033342.1	1512	5	3.49
Appl2	XP_006513572.1	692	2	3.05	Bach2	XP_030108969.1	606	4	6.96
1500009L16Ri	XP_011241859.1	32	0	0.00	Gja10	NP_034419.2	161	1	6.55
Nuak1	NP_001004363.1	371	3	8.52	Casp8ap2	XP_006538012.1	538	3	5.88
Ckap4	NP_780660.1	796	0	0.00	Mdn1	XP_006537539.1	799	12	15.83
Tcp11l2	NP_666120.1	226	0	0.00	Lyrm2	NP_780573.1	171	0	0.00
Polr3b	NP_081699.1	1885	3	1.68	Ankrd6	XP_036019567.1	1217	11	9.53

Rfx4	NP_001020089.1	727	0	0.00	Rragd	NP_001355984.1	565	12	22.38
Ric8b	NP_898995.1	544	4	7.75	Ube2j1	NP_062532.2	1690	10	6.24
Fhl4	NP_034344.2	711	0	0.00	Gabrr2	XP_006537679.1	308	8	27.37
Tmem263	NP_001013046.1	60	0	0.00	Gabrr1	NP_032101.3	438	12	28.87
Mterf2	XP_030101190.1	283	0	0.00	Pm20d2	NP_001030039.2	150	10	70.26
Cry1	NP_031797.1	805	3	3.93	Srsf12	NP_808442.2	1332	8	6.33
Btbd11	NP_082985.2	249	3	12.70	Pnrc1	NP_001028397.2	214	10	49.25
Pwp1	NP_598754.2	960	1	1.10	Rngtt	NP_001292202.1	564	11	20.55
Prdm4	XP_030101164.1	399	0	0.00	Cnr1	NP_001341950.1	1371	4	3.07
Ascl4	NP_001157086.1	129	0	0.00	Spaca1	NP_001277372.1	176	0	0.00
Rtcb	NP_663397.1	503	0	0.00	Akirin2	XP_011248362.1	294	11	39.43
Bpifc	NP_808440.2	110	0	0.00	Orc3	NP_001153035.1	760	11	15.25
Fbxo7	NP_694875.2	603	0	0.00	Rars2	NP_852071.2	918	9	10.33
Syn3	NP_038750.2	482	3	6.56	Slc35a1	NP_036025.2	481	4	8.76
Timp3	NP_035725.1	951	2	2.22	Cfap206	NP_001333991.1	183	6	34.55
Gm6729	XP_017169674.1	173	1	6.09	Gm136	NP_001028427.1	103	7	71.62
Gm5174	XP_036011773.1	115	1	9.16	Smim8	NP_001317684.1	86	3	36.76
Hsp90b1	NP_035761.1	2773	3	1.14	Zfp292	NP_038917.2	601	9	15.78
Ttc41	XP_006513068.1	1	0	0.00	Cga	XP_006537649.1	420	1	2.51
Nt5dc3	NP_780540.2	151	3	20.94	Mob3b	XP_030109254.1	219	5	24.06
Stab2	NP_619614.1	815	5	6.47	Ifnk	NP_954608.1	235	5	22.42
1700113H08R	XP_036011975.1	5	0	0.00	C9orf72	NP_001074812.1	447	4	9.43
Ascl1	NP_032579.2	811	1	1.30	Lingo2	XP_017175687.1	904	6	6.99
Pah	NP_032803.2	1064	4	3.96	1700009N14R	NP_001074564.1	1072	1	0.98
Igf1	NP_001104745.1	2707	9	3.50	Aco1	NP_031412.2	1085	2	1.94
Pmch	NP_084247.1	821	4	5.13	Ddx58	NP_766277.3	1373	1	0.77
Parpbp	XP_030101203.1	412	7	17.91	Topors	XP_036019455.1	692	2	3.05
Nup37	NP_081467.2	1386	5	3.80	Ndufb6	NP_001028477.1	672	6	9.41
Washc3	NP_080346.1	273	7	27.02	Tmem215	NP_796149.2	123	0	0.00
Dram1	NP_082154.2	357	2	5.90	Aptx	NP_079821.3	831	2	2.54
Gnptab	NP_001004164.2	269	6	23.51	Dnaja1	NP_001158143.1	1230	6	5.14
Sycp3	NP_035647.2	496	1	2.12	Smu1	NP_067510.3	837	3	3.78
Chpt1	XP_006513517.1	604	6	10.47	B4galt1	NP_071641.1	685	0	0.00
Mybpc1	XP_006513120.1	394	2	5.35	Spink4	NP_035593.2	191	0	0.00
Spic	NP_035591.3	307	0	0.00	Bag1	NP_033866.4	626	3	5.05
Arl1	NP_080135.2	1570	1	0.67	Chmp5	NP_084090.1	620	5	8.50
Utp20	NP_780367.2	506	2	4.17	Nfx1	NP_076228.2	401	2	5.26
Slc5a8	NP_663398.2	456	1	2.31	Aqp7	XP_036019499.1	681	2	3.10
Gas2l3	NP_001271273.1	751	1	1.40	Aqp3	NP_057898.2	913	3	3.46
Nr1h4	NP_001372640.1	1267	2	1.66	Nol6	NP_631982.2	867	2	2.43
Slc17a8	NP_892004.1	534	2	3.95	Ube2r2	NP_080551.1	1931	6	3.27
Scyl2	NP_001297633.1	181	1	5.82	Ubap2	XP_006538289.1	317	1	3.32
Actr6	NP_080190.1	581	1	1.81	Dcaf12	XP_006538292.1	159	3	19.88
Uhrf1bp1l	NP_083442.2	174	1	6.06	Ubap1	NP_001342437.1	272	5	19.37
Anks1b	XP_006514364.1	1909	5	2.76	Kif24	XP_017175402.1	954	5	5.52
Apaf1	XP_036011453.1	1779	2	1.18	Nudt2	XP_030109557.1	759	7	9.72
Ikbip	NP_081354.1	173	5	30.46	Myorg	NP_001078984.1	331	8	25.47
Slc25a3	NP_598429.1	1251	2	1.68	1110017D15R	NP_001240706.1	93	5	56.66
Tmpo	NP_001073601.1	637	1	1.65	Fam219a	NP_001364034.1	82	7	89.97
Nedd1	NP_032708.2	537	1	1.96	Dnaic1	NP_780347.2	386	4	10.92
Cfap54	XP_011241941.1	126	0	0.00	Enho	NP_081423.1	170	5	31.00
Cdk17	XP_030100944.1	2038	3	1.55	Cntfr	XP_006537659.1	424	5	12.43
Elk3	NP_001269896.1	314	1	3.36	Rpp25l	NP_081554.2	271	9	35.00
Lta4h	NP_032543.2	757	3	4.18	Dctn3	NP_001153037.1	604	8	13.96
Hal	NP_034531.1	744	2	2.83	Arid3c	NP_001239551.1	340	0	0.00
Amdhd1	NP_082184.1	746	2	2.83	Sigmar1	NP_001273467.1	586	2	3.60
Ccdc38	XP_006513691.1	112	2	18.82	Galt	NP_001356064.1	359	5	14.68
Snrpf	NP_081522.1	1469	4	2.87	Il11ra1	NP_001156873.1	200	3	15.81
Ntn4	NP_067295.2	276	3	11.46	Ccl27a	XP_030109229.1	491	3	6.44
Usp44	XP_030100994.1	559	0	0.00	Gm12407	XP_006538475.4	331	5	15.92
Metap2	XP_017169521.1	1418	2	1.49	Ccl21d	XP_036019450.1	502	2	4.20
Vezt	NP_766126.2	358	0	0.00	Gm20878	NP_001257360.1	21	2	100.37
Fgd6	XP_036011484.1	340	2	6.20	Fam205a4	XP_001481289.2	59	1	17.86
Nr2c1	NP_035759.3	552	3	5.73	Gm13304	NP_001180595.1	80	4	52.70
Ndufa12	NP_079827.3	538	3	5.88	Gm13306	NP_001371186.1	493	6	12.83
Tmcc3	NP_001346691.1	306	0	0.00	Gm13305	XP_006537576.2	127	2	16.60

Cep83	NP_084128.2	379	3	8.34	LOC10105567	XP_030107501.2	6	0	0.00
Plxnc1	NP_061267.1	555	5	9.49	Ccl21b	XP_036019422.1	502	4	8.40
Cradd	NP_001317067.1	482	7	15.31	Gm21953	NP_001171051.1	1	1	1053.90
Socs2	XP_017169385.1	1063	5	4.96	Fam205a3	XP_003688841.1	59	0	0.00
Mrpl42	NP_001346405.1	536	5	9.83	Gm21586	XP_006537603.3	1	1	1053.90
Ube2n	NP_542127.1	2214	5	2.38	Fam205a2	XP_030109537.1	59	0	0.00
Nudt4	NP_081998.3	663	7	11.13	Gm10591	NP_001180597.1	81	3	39.03
Eea1	XP_006513585.1	1591	1	0.66	Ccl27b	NP_001371189.1	493	5	10.69
Btg1	NP_031595.1	232	0	0.00	Il11ra2	XP_006537688.2	127	0	0.00
Dcn	NP_001177380.1	1355	5	3.89	Ccl19	NP_036018.1	562	4	7.50
Lum	NP_032550.2	1297	4	3.25	Ccl21a	NP_035254.1	376	2	5.61
Kera	XP_006513336.1	755	4	5.58	Fam205a1	NP_001264096.1	7	0	0.00
Epyc	NP_001366385.1	662	5	7.96	Fam205c	XP_011248353.1	7	0	0.00
Ccer1	NP_080000.1	288	0	0.00	Phf24	XP_006537872.1	56	0	0.00
Atp2b1	NP_001346435.1	1256	6	5.03	Dnajb5	NP_001342367.1	745	1	1.41
Poc1b	NP_082016.1	330	3	9.58	1700022I11Ri	NP_080364.1	12	0	0.00
Galnt4	NP_056552.1	201	2	10.49	Vcp	NP_033529.3	2438	5	2.16
Dusp6	NP_080544.1	1069	3	2.96	Fancg	XP_017175825.1	417	1	2.53
Csl	NP_082221.2	1684	0	0.00	Pigo	NP_064419.2	340	0	0.00
Kitl	XP_006513377.1	1298	2	1.62	Stoml2	NP_075720.1	862	3	3.67
Gm4301	NP_001160109.1	551	2	3.83	Fam214b	XP_030109273.1	108	0	0.00
Gm4302	NP_001160106.1	550	2	3.83	Unc13b	XP_036019864.1	701	1	1.50
Gm4303	NP_001160110.1	485	0	0.00	Atp8b5	XP_006538058.1	480	0	0.00
Gm4305	NP_001160111.1	485	0	0.00	Rusc2	XP_030108916.1	168	1	6.27
Gm4307	NP_001160113.1	484	0	0.00	Fam166b	XP_011248356.1	50	0	0.00
Gm4312	NP_001160108.1	507	2	4.16	Tesk1	NP_035701.3	1040	3	3.04
Tmtc3	NP_001103483.1	869	0	0.00	Cd72	NP_001103792.1	592	2	3.56
Cep290	NP_666121.2	691	1	1.53	Sit1	NP_062309.2	91	0	0.00
4930430F08Ri	NP_780337.2	72	2	29.28	Ccdc107	NP_001033002.1	54	1	19.52
1700017N19R	NP_001074715.1	250	1	4.22	Arhgef39	NP_001013395.2	368	0	0.00
Mgat4c	XP_030101095.1	263	2	8.01	Car9	NP_647466.2	579	1	1.82
Nts	NP_077755.1	902	1	1.17	Tpm2	NP_001264804.1	1206	1	0.87
Rassf9	NP_666352.1	139	0	0.00	Tln1	XP_006537832.3	861	3	3.67
Alx1	XP_036011647.1	538	2	3.92	Creb3	NP_038525.2	439	0	0.00
Lrriq1	XP_030101198.1	664	3	4.76	Gba2	NP_766280.2	1338	1	0.79
Slc6a15	NP_001345750.1	813	5	6.48	Rgp1	NP_766454.1	212	0	0.00
Gm21293	NP_001257827.1	484	0	0.00	Msmp	NP_001092784.1	68	1	15.50
Gm6763	NP_001257828.1	584	1	1.80	Npr2	NP_776149.1	926	1	1.14
Gm8764	NP_001257829.1	584	1	1.80	Spag8	NP_001277391.1	166	1	6.35
Gm21304	NP_001257830.1	584	1	1.80	Hint2	NP_081147.1	670	0	0.00
Gm21312	NP_001257571.1	578	4	7.29	Fam221b	NP_780726.1	4	0	0.00
Gm20765	NP_001257573.1	584	1	1.80	Tmem8b	NP_001355769.1	158	1	6.67
Gm4340	NP_001171006.1	523	0	0.00	Olfr70	NP_062358.2	33	0	0.00
Tmtc2	NP_796342.2	887	4	4.75	Olfr71	NP_062359.1	57	0	0.00
Mettl25	XP_017169392.1	100	2	21.08	Hrct1	NP_081787.1	33	0	0.00
Ccdc59	NP_079878.1	314	0	0.00	Olfr159	NP_062349.1	49	0	0.00
Ppfia2	XP_006513843.1	973	7	7.58	Olfr156	NP_062347.2	36	0	0.00
Acss3	NP_001136276.1	557	1	1.89	Olfr157	NP_062348.1	46	0	0.00
Lin7a	NP_001034443.1	591	4	7.13	Olfr155	NP_062346.1	24	0	0.00
Myf5	XP_006513382.1	800	4	5.27	Reck	NP_057887.2	481	0	0.00
Myf6	NP_032683.1	639	4	6.60	Glipr2	NP_081726.1	225	0	0.00
Ptprq	NP_001074901.1	825	8	10.22	Ccin	NP_001002787.1	319	1	3.30
Otogl	XP_017169530.1	280	2	7.53	Clta	NP_001073854.1	818	2	2.58
Ppp1r12a	NP_001355665.1	1626	2	1.30	Gne	NP_056643.3	590	4	7.15
Pawr	XP_036011407.1	382	9	24.83	Rnf38	NP_001366509.1	441	4	9.56
Syt1	NP_001345435.1	1806	7	4.08	Melk	NP_034920.2	1817	2	1.16
Nav3	XP_036011681.1	506	10	20.83	Pax5	NP_032808.1	1114	2	1.89
E2f7	NP_001345489.1	802	7	9.20	Zcchc7	NP_613056.2	795	3	3.98
Csrp2	NP_031818.3	914	3	3.46	Grhpr	NP_525028.1	1040	1	1.01
Zdhhc17	NP_766142.2	708	8	11.91	Zbtb5	NP_001156755.1	238	0	0.00
Osbpl8	NP_780698.2	431	2	4.89	Polr1e	NP_001272729.1	934	3	3.39
Bbs10	NP_082190.1	2099	9	4.52	Fbxo10	NP_001019313.1	508	4	8.30
Nap1l1	XP_030101038.1	1487	3	2.13	Tomm5	NP_001093145.1	536	3	5.90
Phlda1	NP_033370.2	391	2	5.39	Frmpd1	NP_001074641.1	153	4	27.55
Krr1	NP_848725.2	1168	8	7.22	Trmt10b	NP_081542.2	327	2	6.45
Glipr1	NP_082884.1	642	7	11.49	Exosc3	NP_001349717.1	856	5	6.16

Glipr1l1	NP_081294.1	90	4	46.84	Dcaf10	NP_694807.2	1359	5	3.88
Glipr1l2	NP_080499.1	63	2	33.46	Slc25a51	NP_001009949.3	137	2	15.39
Caps2	NP_840062.2	464	0	0.00	Shb	NP_001028478.1	276	1	3.82
Kcnc2	NP_001366573.1	1389	4	3.03	Aldh1b1	NP_082546.1	865	1	1.22
Atxn7l3b	NP_001028646.2	60	4	70.26	Igfbpl1	NP_061211.1	244	0	0.00
Trhde	NP_666353.2	714	1	1.48	Stra6l	XP_006538373.1	538	1	1.96
Tph2	NP_775567.2	551	1	1.91	Ccdc180	NP_941062.2	100	1	10.54
Tbc1d15	NP_079982.3	967	3	3.27	Tdrd7	NP_001277404.1	1004	6	6.30
Rab21	NP_077774.1	632	1	1.67	Tmod1	NP_068683.1	617	5	8.54
Tmem19	NP_001346399.1	113	1	9.33	Tstd2	NP_001342305.1	59	4	71.45
Thap2	NP_080056.1	129	1	8.17	Ncbp1	NP_001028373.2	1686	6	3.75
Zfc3h1	XP_006513603.1	639	3	4.95	Xpa	NP_035858.2	812	6	7.79
Lgr5	NP_034325.2	1363	1	0.77	Foxe1	NP_899121.1	669	2	3.15
A930009A15R	NP_084258.1	0	0	#DIV/0!	Trmo	NP_083362.1	124	5	42.50
Tspan8	NP_666122.1	517	1	2.04	Hemgn	NP_444379.1	274	2	7.69
Ptprr	NP_035347.1	893	5	5.90	Anp32b	NP_570959.1	794	5	6.64
Taf7l2	NP_001155327.1	456	1	2.31	Nans	NP_444409.1	485	3	6.52
Ptprb	XP_036011586.1	930	6	6.80	Trim14	NP_083353.1	347	2	6.07
Kcnmb4	XP_036011795.1	209	4	20.17	Coro2a	NP_849224.2	768	8	10.98
Cnot2	XP_030101160.1	830	6	7.62	Tbc1d2	NP_941066.3	783	0	0.00
Myrfl	NP_001028505.1	74	6	85.45	Gabbr2	NP_001074610.1	1282	0	0.00
Rab3ip	XP_030100901.1	639	5	8.25	Anks6	XP_006538397.1	110	1	9.58
Best3	NP_001007584.1	369	13	37.13	Galnt12	NP_766281.1	345	1	3.05
Lrrc10	NP_666354.1	626	8	13.47	Col15a1	NP_034058.2	568	0	0.00
Cct2	NP_031662.2	2260	14	6.53	Tgfbr1	NP_033396.1	1902	3	1.66
Frs2	XP_036011744.1	519	11	22.34	Alg2	NP_064382.3	642	2	3.28
Yeats4	NP_080846.1	946	14	15.60	Sec61b	NP_077133.1	871	1	1.21
9530003J23Ri	NP_084182.2	342	8	24.65	Nr4a3	XP_030109168.1	514	3	6.15
Lyz2	NP_059068.1	1068	10	9.87	Stx17	XP_036020268.1	933	2	2.26
Lyz1	NP_038618.1	823	12	15.37	Erp44	NP_083848.1	1002	2	2.10
Cpsf6	XP_030101033.1	959	15	16.48	Invs	XP_006537689.1	1358	3	2.33
Cpm	NP_081744.1	366	7	20.16	Tex10	XP_036020032.1	627	1	1.68
Mdm2	NP_034916.1	2060	10	5.12	Msantd3	XP_036020237.1	155	0	0.00
Slc35e3	NP_084151.2	184	6	34.37	Tmeff1	NP_001343201.1	262	0	0.00
Nup107	NP_598771.1	1034	17	17.33	Cavin4	NP_080785.2	370	0	0.00
Rap1b	NP_077777.1	2022	5	2.61	Plppr1	XP_017175739.1	315	0	0.00
Mdm1	XP_006513368.1	204	8	41.33	Acnat2	XP_036019828.1	604	2	3.49
Il22	XP_006513928.1	409	4	10.31	Acnat1	XP_006537902.1	98	0	0.00
Iltifb	NP_473420.2	126	2	16.73	Baat	NP_031545.2	749	3	4.22
Gm9040	XP_036012051.1	3263	3	0.97	Mrpl50	NP_848718.1	380	2	5.55
Gm9044	XP_036012052.1	3263	3	0.97	Zfp189	NP_663522.2	213	1	4.95
Gm9045	XP_036012053.1	3263	2	0.65	Aldob	NP_659152.1	1395	2	1.51
Gm9046	XP_036012054.1	3263	2	0.65	Pgap4	NP_001342432.1	56	0	0.00
Gm9048	XP_036012055.1	3263	2	0.65	Rnf20	NP_001156735.1	1013	1	1.04
Gm9049	XP_036012056.1	3263	2	0.65	Grin3a	NP_001028523.1	685	0	0.00
Ifng	NP_032363.1	1736	4	2.43	Ppp3r2	NP_001004025.1	1159	0	0.00
Dyrk2	XP_006514109.3	1018	3	3.11	Cylc2	XP_017175914.1	260	2	8.11
Cand1	NP_082270.1	818	7	9.02	Smc2	XP_036019572.1	1588	1	0.66
Grip1	XP_006514301.1	948	1	1.11	Toporsl	NP_001343222.1	330	1	3.19
Helb	NP_536694.2	520	8	16.21	Olfr275	NP_667069.2	117	0	0.00
Irak3	NP_082955.2	1217	8	6.93	Olfr273	NP_667035.1	26	0	0.00
Tmbim4	NP_080893.1	362	5	14.56	Olfr272	NP_667050.1	64	0	0.00
Llph	NP_079707.1	280	2	7.53	Olfr270	NP_666818.1	43	0	0.00
Hmga2	NP_034571.1	831	4	5.07	Nipsnap3a	NP_082805.1	229	2	9.20
Msrb3	XP_006513829.1	442	10	23.84	Nipsnap3b	NP_079899.1	159	2	13.26
Lemd3	NP_001074662.2	696	11	16.66	Abca1	XP_006537619.1	1177	3	2.69
Wif1	NP_036045.1	682	4	6.18	Slc44a1	NP_598652.3	507	3	6.24
Tbc1d30	NP_001347944.1	871	10	12.10	Fsd1l	XP_006538043.1	295	5	17.86
Gns	NP_083640.1	527	10	20.00	Fktn	XP_006538004.1	354	3	8.93
Rassf3	NP_620406.1	192	12	65.87	Tal2	NP_033343.1	174	4	24.23
Tbk1	NP_062760.3	1946	6	3.25	Tmem38b	NP_082329.1	255	5	20.66
Xpot	XP_030101166.1	799	10	13.19	Zfp462	XP_030109394.1	398	5	13.24
D930020B18R	XP_006513614.1	8	2	263.48	Gm12508	XP_036020498.1	492	2	4.28
BC048403	NP_766610.2	102	1	10.33	Rad23b	NP_033037.2	2502	5	2.11
Srgap1	XP_036011451.1	558	8	15.11	Klf4	NP_034767.2	1728	4	2.44
Rxylt1	NP_694699.1	148	8	56.97	Actl7b	NP_079547.2	1430	3	2.21

Avpr1a	NP_058543.2	763	3	4.14	Actl7a	NP_033741.1	1358	4	3.10
Ppm1h	NP_001103688.1	160	2	13.17	Elp1	NP_080355.2	849	5	6.21
Mon2	NP_700444.2	1337	3	2.36	Abitram	NP_001074889.1	124	2	17.00
Usp15	NP_001288557.1	987	7	7.47	Ctnnal1	NP_061231.3	546	7	13.51
Tafa2	XP_017169441.1	243	0	0.00	Tmem245	NP_001356190.1	208	3	15.20
Slc16a7	XP_017169350.1	364	0	0.00	Frrs1l	NP_001136437.1	926	6	6.83
Lrig3	NP_796126.4	940	0	0.00	Epb41l4b	NP_001342421.1	526	2	4.01
Atp23	NP_081134.2	365	7	20.21	Ptpn3	XP_006538160.1	720	1	1.46
Ctdsp2	NP_001106941.1	534	1	1.97	Gm12538	XP_036020554.1	625	2	3.37
Avil	XP_006513161.1	527	9	18.00	Pakap	NP_001371086.1	273	2	7.72
Tsfm	NP_079813.1	970	10	10.86	D630039A03F	NP_848842.1	190	0	0.00
Eef1akmt3	XP_017169241.1	145	11	79.95	Txn1	NP_035790.1	2315	2	0.91
Mettl1	XP_006513375.1	883	11	13.13	Txndc8	XP_006538264.1	1351	5	3.90
Cyp27b1	NP_034139.2	506	8	16.66	Svep1	NP_073725.2	700	5	7.53
Marchf9	NP_001028434.1	458	10	23.01	Musk	XP_006537722.1	1273	3	2.48
Cdk4	NP_034000.1	3254	11	3.56	Lpar1	NP_034466.2	806	3	3.92
Tspan31	NP_080258.1	280	11	41.40	Olfr267	NP_667131.2	43	1	24.51
Agap2	XP_006513624.1	892	13	15.36	Ecpas	NP_759013.2	685	4	6.15
Os9	NP_808282.2	898	10	11.74	Zkscan16	NP_001342337.1	589	2	3.58
B4galnt1	NP_082015.2	430	11	26.96	Ptgr1	NP_080244.1	487	3	6.49
Slc26a10	XP_011241723.1	254	2	8.30	Dnajc25	NP_001028337.2	465	2	4.53
Arhgef25	NP_082303.2	673	2	3.13	Gng10	NP_079553.1	789	1	1.34
Dtx3	NP_001355261.1	181	1	5.82	Shoc1	NP_001357772.1	22	0	0.00
Pip4k2c	XP_036011413.1	568	2	3.71	Ugcg	NP_035803.1	720	1	1.46
Kif5a	XP_030100779.1	2328	8	3.62	Susd1	XP_036020180.1	185	3	17.09
Dctn2	NP_001177383.1	911	1	1.16	Ptbp3	NP_659153.2	585	3	5.40
Mbd6	NP_001334100.1	101	0	0.00	Hsdl2	NP_077217.2	859	2	2.45
Ddit3	XP_030100727.1	1073	4	3.93	E130308A19F	XP_006537918.1	196	3	16.13
Mars1	NP_001003913.1	2037	1	0.52	Inip	NP_001013595.1	133	1	7.92
Arhgap9	NP_001272714.1	724	2	2.91	Snx30	NP_766056.1	437	0	0.00
Gli1	NP_034426.2	1497	3	2.11	Slc46a2	NP_066395.3	259	5	20.35
Inhbe	NP_032408.2	916	4	4.60	Mup4	NP_032674.1	433	0	0.00
Inhbc	NP_034695.1	860	6	7.35	Mup6	NP_001074754.1	54	0	0.00
R3hdm2	NP_001346172.1	379	1	2.78	Mup7	XP_030108860.1	464	0	0.00
Stac3	NP_001346680.1	250	0	0.00	Mup2	XP_006537714.1	353	1	2.99
Ndufa4l2	NP_001092259.1	285	0	0.00	Mup8	XP_030108871.1	401	1	2.63
Shmt2	NP_001239245.1	1484	4	2.84	Mup9	XP_006537543.1	464	0	0.00
Nxph4	NP_899120.2	240	0	0.00	Mup1	NP_001156483.1	487	0	0.00
Lrp1	NP_032538.2	893	1	1.18	Mup10	XP_006537544.1	462	0	0.00
Stat6	NP_033310.2	1268	3	2.49	Mup11	XP_006537546.1	352	1	2.99
Nab2	NP_001116367.1	366	4	11.52	Mup12	XP_006537547.1	397	1	2.65
Nemp1	NP_001106682.1	209	9	45.38	Mup22	XP_003688833.1	459	0	0.00
Myo1a	NP_001074688.1	1110	6	5.70	Mup13	XP_030108849.1	350	0	0.00
Tac2	NP_033338.2	409	6	15.46	Mup14	XP_006537549.1	395	0	0.00
Zbtb39	NP_932152.1	281	11	41.26	Mup15	XP_006537550.1	458	0	0.00
Gpr182	NP_031438.2	267	6	23.68	Mup16	XP_006537552.1	350	1	3.01
Rdh1	NP_536684.2	173	1	6.09	Mup-ps12	XP_017174537.1	1	0	0.00
Rdh9	NP_694773.1	165	1	6.39	Mup17	XP_006537554.1	352	1	2.99
Rdh16	XP_006513452.1	238	5	22.14	Mup18	XP_006537578.1	462	0	0.00
Rdh19	NP_671755.2	118	1	8.93	Mup19	XP_006537589.1	354	1	2.98
Rdh16f2	NP_663399.2	490	7	15.06	Mup5	NP_032675.2	523	0	0.00
Rdh7	NP_001144221.1	680	5	7.75	Mup20	XP_006538108.1	499	1	2.11
Sdr9c7	NP_081577.1	501	8	16.83	Mup3	NP_001034633.1	594	1	1.77
Hsd17b6	XP_030100969.1	814	6	7.77	Mup21	NP_001009550.1	390	1	2.70
Prim1	NP_032947.1	1092	4	3.86	Zfp37	NP_033580.3	362	8	23.29
Naca	NP_001106670.1	1120	3	2.82	Slc31a2	NP_079562.1	599	3	5.28
Ptges3	NP_062740.1	1285	4	3.28	Fkbp15	NP_001342314.1	1328	5	3.97
Atp5b	NP_058054.2	2690	1	0.39	Slc31a1	NP_780299.2	531	2	3.97
Baz2a	XP_030100690.1	1307	3	2.42	Cdc26	NP_001355170.1	677	1	1.56
Rbms2	XP_006513962.1	566	3	5.59	Prpf4	NP_081573.1	907	2	2.32
Gls2	NP_001028436.2	898	8	9.39	Rnf183	NP_001366554.1	330	0	0.00
Spryd4	NP_079992.1	316	2	6.67	Wdr31	XP_006538320.1	1398	2	1.51
Mip	NP_032626.2	543	3	5.82	Bspry	XP_006537739.2	398	0	0.00
Timeless	XP_006513643.1	1085	4	3.89	Hdhd3	NP_077219.1	250	4	16.86
Apon	NP_598757.1	509	2	4.14	Alad	NP_032551.3	733	2	2.88
Apof	NP_598758.2	503	1	2.10	Pole3	NP_067473.2	618	3	5.12

Stat2	NP_064347.1	1049	2	2.01	4933430I17Ri	XP_011248273.1	72	0	0.00
Il23a	NP_112542.1	338	1	3.12	Rgs3	XP_006538120.1	608	1	1.73
Pan2	XP_036011343.1	637	0	0.00	Zfp618	XP_030109674.1	348	4	12.11
Cnpy2	XP_030101044.1	270	1	3.90	Ambp	NP_031469.1	1183	6	5.35
Cs	NP_080720.1	1740	2	1.21	Kif12	XP_017175487.1	843	9	11.25
Coq10a	NP_001361629.1	278	1	3.79	Col27a1	XP_030109489.1	425	6	14.88
Ankrd52	NP_766378.1	1279	6	4.94	Orm1	NP_032794.1	943	5	5.59
Slc39a5	NP_082327.2	267	4	15.79	Orm3	NP_001366450.1	637	4	6.62
Nabp2	NP_001345901.1	293	4	14.39	Orm2	NP_035146.1	782	6	8.09
Rnf41	NP_001157709.1	845	6	7.48	Akna	XP_036019435.1	206	4	20.46
Smarcc2	NP_001359324.1	1249	2	1.69	Whrn	NP_001008793.1	834	3	3.79
Myl6	NP_034990.1	1337	2	1.58	Atp6v1g1	NP_077135.1	315	0	0.00
Myl6b	NP_758463.1	1126	1	0.94	Tmem268	NP_001343283.1	9	8	936.80
Esyt1	NP_035973.1	375	2	5.62	Tex48	NP_001343254.1	1040	0	0.00
Zc3h10	NP_598764.1	224	1	4.70	Tnfsf15	NP_796345.4	685	4	6.15
Rpl41	NP_061348.1	182	1	5.79	Tnfsf8	XP_036019860.1	377	3	8.39
Pa2g4	NP_035249.1	1916	3	1.65	Tnc	NP_001356143.1	819	2	2.57
Erbb3	NP_034283.1	2017	8	4.18	Pappa	NP_067337.1	330	1	3.19
Gm9182	XP_036012024.1	70	2	30.11	Astn2	XP_030109540.1	418	4	10.09
Rps26	NP_038793.2	965	8	8.74	Trim32	XP_030109625.1	989	3	3.20
Ikzf4	XP_036011661.1	360	9	26.35	Tlr4	NP_067272.1	2550	4	1.65
Suox	NP_776094.2	817	8	10.32	Brinp1	NP_064351.2	823	2	2.56
Rab5b	NP_803130.1	1340	7	5.51	Cdk5rap2	NP_666102.2	593	2	3.55
Cdk2	NP_058036.1	3195	5	1.65	Megf9	NP_766282.1	178	2	11.84
Pmel	XP_036011605.1	623	7	11.84	Tle1	XP_036019853.1	880	2	2.40
Dgka	XP_011241653.2	490	5	10.75	Aldoart1	NP_001186199.1	774	0	0.00
Pym1	XP_036011999.1	320	5	16.47	Gm11487	XP_011248365.1	208	9	45.60
Mmp19	NP_067387.1	270	1	3.90	Gm38423	NP_001243410.2	202	9	46.96
Tmem198b	XP_030101184.1	44	0	0.00	Gm11237	NP_001264504.1	202	9	46.96
Dnajc14	NP_001346754.1	359	2	5.87	Gm11236	NP_001264460.1	174	10	60.57
Ormdl2	NP_001346099.1	116	0	0.00	Gm11238	NP_001264441.1	174	10	60.57
Sarnp	NP_079640.1	837	1	1.26	Gm11239	NP_001264416.1	169	10	62.36
Gdf11	NP_034402.1	679	2	3.10	Gm428	XP_011248342.1	187	10	56.36
Cd63	NP_001036045.1	1041	3	3.04	Rasef	XP_006537989.1	587	3	5.39
Rdh5	NP_001345456.1	519	2	4.06	Gm11758	NP_001091447.2	119	8	70.85
Bloc1s1	NP_056555.2	383	3	8.26	Gm11757	NP_001079007.1	181	10	58.23
Itga7	XP_030100775.1	783	2	2.69	Gm13871	NP_001171049.1	119	8	70.85
Mettl7b	NP_082129.2	1150	0	0.00	Gm11756	NP_001119798.1	74	7	99.69
Olfr9	NP_667072.1	17	0	0.00	2310002L09R	NP_081380.2	394	1	2.67
Olfr763	NP_667073.1	17	0	0.00	Frmd3	NP_001157204.1	438	1	2.41
Olfr765	NP_001078946.1	138	0	0.00	Kdm4c	NP_001165566.1	1499	3	2.11
Olfr767	NP_666430.2	80	0	0.00	Dmac1	NP_080125.1	239	1	4.41
Olfr768	NP_667075.1	30	0	0.00	Ptprd	XP_036019771.1	1427	6	4.43
Olfr769	NP_666379.1	62	0	0.00	Tyrp1	XP_006537844.1	571	8	14.77
Olfr770	NP_667074.1	32	0	0.00	Lurap1l	NP_081097.2	82	0	0.00
Olfr771	NP_666758.1	13	0	0.00	Mpdz	XP_006537707.1	1010	6	6.26
Olfr772	NP_666378.1	21	0	0.00	Nfib	NP_001273060.1	715	9	13.27
Olfr773	NP_996891.2	11	0	0.00	Zdhhc21	XP_036020274.1	272	10	38.75
Olfr774	NP_997503.1	10	0	0.00	Cer1	NP_034017.1	174	5	30.28
Olfr775	NP_666756.2	64	0	0.00	Frem1	NP_001185740.1	361	13	37.95
Olfr776	NP_997442.1	20	0	0.00	Ttc39b	NP_081514.1	171	14	86.28
Olfr777	NP_666755.1	63	0	0.00	Snapc3	XP_017175944.1	344	9	27.57
Olfr780	NP_666396.1	21	0	0.00	Psip1	NP_598709.1	1667	6	3.79
Olfr781	NP_666939.1	51	0	0.00	Ccdc171	NP_001342307.1	131	14	112.63
Olfr782	NP_001011797.1	197	0	0.00	Bnc2	NP_766458.3	428	10	24.62
Olfr784	XP_017169433.1	46	0	0.00	Cntln	XP_006538089.1	454	6	13.93
Olfr786	NP_666760.1	41	0	0.00	Sh3gl2	NP_062408.2	1114	6	5.68
Olfr787	XP_006513721.1	12	0	0.00	Adamtsl1	XP_006538438.1	287	6	22.03
Olfr788	NP_666762.1	54	0	0.00	Saxo1	NP_001074565.1	48	0	0.00
Olfr790	NP_667144.1	71	0	0.00	Rraga	NP_848463.1	454	1	2.32
Olfr791	NP_667141.1	173	1	6.09	Haus6	XP_006537938.1	575	7	12.83
Olfr792	NP_001011849.1	11	0	0.00	Plin2	XP_011248209.1	714	0	0.00
Olfr794	NP_666490.1	47	0	0.00	Dennd4c	XP_006538085.1	240	6	26.35
Olfr796	NP_667142.1	59	0	0.00	Rps6	NP_033122.1	1890	2	1.12
Olfr798	NP_666767.2	21	0	0.00	Acer2	NP_001277472.1	383	0	0.00
Olfr799	NP_667138.1	80	0	0.00	Slc24a2	XP_036020420.1	1172	1	0.90

Olfr800	NP_666759.1	311	1	3.39	Mllt3	NP_081602.3	1126	0	0.00
Olfr801	NP_666397.1	10	0	0.00	Focad	XP_036019889.1	114	4	36.98
Olfr802	NP_667143.1	38	0	0.00	Hacd4	XP_006538256.1	283	7	26.07
Olfr803	NP_666765.1	50	0	0.00	Ifnb1	NP_034640.1	754	11	15.38
Olfr804	NP_001011821.1	98	0	0.00	Ifna15	NP_996753.1	181	5	29.11
Olfr805	NP_666766.1	27	0	0.00	Ifna14	NP_996858.1	119	6	53.14
Olfr806	NP_666764.1	10	0	0.00	Ifna9	NP_034637.1	193	9	49.15
Olfr807	NP_667140.1	55	0	0.00	Ifna12	NP_796335.1	111	8	75.96
Olfr808	NP_667139.1	33	0	0.00	Pramel32	NP_941065.2	192	0	0.00
Olfr809	NP_666436.1	50	0	0.00	Ifna13	NP_796321.1	132	5	39.92
Olfr810	NP_666761.1	10	0	0.00	Ifna16	NP_996750.1	85	5	61.99
Olfr811	NP_666763.1	48	0	0.00	Ifna2	NP_034633.2	217	7	34.00
Olfr812	NP_667006.1	10	0	0.00	Ifnab	NP_032362.2	138	6	45.82
Olfr813	NP_997030.1	45	0	0.00	Klhl9	NP_766459.2	624	4	6.76
Olfr814	NP_997042.1	22	0	0.00	Gm13271	NP_001078997.1	49	3	64.52
Olfr815	NP_666881.1	82	0	0.00	Gm13286	NP_001230079.1	1	0	0.00
Olfr816	NP_666883.1	17	0	0.00	Gm13283	NP_001079000.1	37	0	0.00
Olfr818	NP_666988.1	16	0	0.00	Gm13290	NP_001230084.1	40	0	0.00
Olfr819	NP_001159416.1	43	1	24.51	Gm13289	NP_001230094.1	40	0	0.00
Olfr247	NP_666381.2	35	0	0.00	Gm13272	NP_001155080.1	1	0	0.00
Gm10310	NP_001357809.1	36	1	29.28	Ifnz	NP_922871.1	62	2	34.00
Olfr820	NP_666886.1	155	1	6.80	Gm13276	NP_001079001.1	1	0	0.00
Olfr821	NP_666987.1	41	0	0.00	Gm13277	NP_001092310.1	40	0	0.00
Olfr822	NP_666882.1	38	0	0.00	Gm13278	NP_001092311.1	40	0	0.00
Olfr823	NP_666884.2	176	0	0.00	Gm13275	NP_001079002.1	2	0	0.00
Olfr824	NP_666885.1	99	0	0.00	Gm13279	NP_001230095.1	40	0	0.00
Olfr825	NP_666888.1	122	1	9.60	Gm13285	NP_001155081.1	1	0	0.00
Olfr826	NP_666887.1	42	0	0.00	Gm13287	NP_001155082.1	1	0	0.00
Olfr827	NP_666412.1	86	0	0.00	Gm13288	NP_001230096.1	37	0	0.00
Neurod4	NP_001316419.1	240	0	0.00	Ifna7	NP_032360.2	314	4	13.43
Tespa1	NP_899087.2	210	0	0.00	Ifna11	NP_032359.2	184	5	28.64
Vmn2r84	NP_001074917.1	30	0	0.00	Ifna6	NP_996754.1	137	2	15.39
Vmn2r85	XP_036011800.1	7	0	0.00	Ifna5	NP_034635.2	149	2	14.15
Vmn2r86	NP_001096835.1	48	0	0.00	Ifna4	NP_034634.1	175	2	12.04
Vmn2r87	NP_001096836.1	8	0	0.00	Ifna1	NP_034632.2	194	2	10.86
Sfi1	XP_006514961.1	490	3	12.90	Ifne	NP_796322.1	200	5	26.35
Eif4enif1	NP_076232.2	455	1	4.21	Mtap	NP_077753.1	744	10	14.17
Drg1	NP_031905.1	780	1	2.25	Cdkn2a	NP_001035744.1	734	3	4.31
Patz1	NP_062520.1	457	0	0.00	Cdkn2b	NP_031696.1	2146	7	3.44
Pik3ip1	NP_835362.2	215	0	0.00	Dmrta1	NP_783578.1	254	4	16.60
Limk2	NP_034848.1	1208	4	4.65	Zfp352	NP_694742.2	371	2	5.68
Rnf185	NP_663330.2	394	2	6.69	Elavl2	XP_006502851.1	1235	3	2.56
Pla2g3	XP_006514752.1	279	0	0.00	Izumo3	NP_081310.1	377	1	2.80
Inpp5j	XP_030101477.1	685	3	5.13	Tusc1	NP_081230.1	165	6	38.32
Selenom	NP_444497.1	96	0	0.00	Caap1	NP_080644.2	269	1	3.92
Smtn	XP_017170075.1	762	0	0.00	Plaa	NP_766283.2	781	8	10.80
Morc2a	XP_006514934.1	175	0	0.00	Gm12657	NP_001074488.1	1927	5	2.73
Osbp2	NP_690031.2	530	4	7.95	Ift74	XP_036020264.1	446	4	9.45
4921536K21R	NP_080426.1	18	0	0.00	Lrrc19	NP_001343210.1	473	4	8.91
Dusp18	NP_776106.1	480	1	2.20	Tek	NP_038718.2	1230	1	0.86
Slc35e4	NP_694782.1	174	2	12.11	Eqtn	XP_006503396.1	1021	1	1.03
Tcn2	NP_001123931.1	321	2	6.57	Gm12693	XP_036020506.1	329	0	0.00
Pes1	NP_075027.1	1233	2	1.71	Mysm1	XP_036020079.1	2454	4	1.72
Gal3st1	XP_036012743.1	406	2	5.19	Jun	NP_034721.1	3451	4	1.22
Sec14l4	NP_666125.1	611	0	0.00	Fggy	XP_011238935.1	1011	11	11.47
Sec14l3	NP_001025108.1	423	0	0.00	Hook1	NP_084290.1	306	4	13.78
Mtfp1	NP_080719.2	244	1	4.32	Cyp2j13	XP_006502998.1	221	2	9.54
Sec14l2	NP_653103.1	640	0	0.00	Cyp2j12	NP_001093652.1	165	2	12.77
Rnf215	NP_082135.2	274	0	0.00	Cyp2j11	NP_001004141.2	173	2	12.18
Ccdc157	XP_011241975.1	58	0	0.00	Cyp2j8	NP_001098397.1	167	2	12.62
Sf3a1	NP_080451.4	1151	5	4.58	Cyp2j6	XP_011238726.1	284	7	25.98
Tbc1d10a	NP_598784.1	475	4	8.87	Cyp2j9	XP_030109713.1	224	2	9.41
Castor1	NP_082298.1	171	3	18.49	Cyp2j5	XP_006502783.1	553	3	5.72
Osm	NP_001013383.1	633	1	1.66	Gm12695	NP_001074753.1	98	0	0.00
Lif	XP_006514605.1	882	3	3.58	Nfia	XP_006502904.1	620	5	8.50
Hormad2	NP_083734.1	343	4	12.29	Tm2d1	NP_444387.1	208	11	55.74

Mtmr3	NP_083136.2	248	7	29.75	Patj	NP_766284.2	1722	7	4.28
Ascc2	NP_083567.1	228	7	32.36	L1td1	NP_001074671.1	237	11	48.92
Uqcr10	NP_932096.1	722	3	4.38	Kank4	NP_766460.2	946	11	12.25
Zmat5	NP_001343319.1	753	11	15.40	Usp1	NP_001343353.1	1382	11	8.39
Cabp7	NP_620398.1	1058	2	1.99	Dock7	NP_080358.3	601	13	22.80
Nf2	XP_036012294.1	1037	9	9.15	Angptl3	NP_038941.1	1008	14	14.64
Nipsnap1	NP_032724.1	336	5	15.68	Atg4c	NP_778194.3	841	15	18.80
Thoc5	XP_006514511.2	598	4	7.05	Foxd3	NP_034555.3	1049	14	14.07
Nefh	NP_035034.2	848	10	12.43	Alg6	NP_001074733.1	315	20	66.91
Ap1b1	NP_001342588.1	903	6	7.00	Itgb3bp	NP_001349904.1	341	16	49.45
Gas2l1	NP_084504.2	597	10	17.65	Efcab7	XP_036019895.1	1133	19	17.67
Rasl10a	NP_660251.1	1373	8	6.14	Pgm1	NP_082408.3	792	3	3.99
Ewsr1	NP_001269991.1	1159	10	9.09	Ror1	NP_038873.2	1049	5	5.02
Rhbdd3	XP_017170073.1	246	0	0.00	Ube2u	NP_001028945.2	1648	13	8.31
Emid1	NP_001357930.1	172	0	0.00	Cachd1	NP_932154.1	185	13	74.06
Kremen1	XP_006514975.1	431	3	7.34	Raver2	NP_898845.1	175	9	54.20
Znrf3	NP_001074393.1	511	3	6.19	Jak1	NP_038595.1	1943	10	5.42
Xbp1	NP_001258659.1	1145	2	1.84	Ak4	NP_001171073.1	872	12	14.50
Ccdc117	NP_598794.2	53	4	79.54	Dnajc6	NP_001342108.1	2400	12	5.27
Ankrd36	NP_076305.2	1335	5	3.95	Leprot	NP_778201.1	175	1	6.02
Gm11964	XP_036013015.1	953	1	1.11	Lepr	XP_036019643.1	813	5	6.48
Mrps24	NP_001349800.1	492	1	2.14	Pramel17	NP_001028962.1	90	0	0.00
Urgcp	XP_011242056.1	70	0	0.00	C130073F10R	NP_001318108.1	14	1	75.28
Dbnl	XP_036012173.1	769	1	1.37	Pramel19	NP_001078985.1	138	0	0.00
Pgam2	NP_061358.1	874	1	1.21	Gm12790	NP_001364279.1	14	1	75.28
Polm	NP_059097.2	381	1	2.77	Gm12789	NP_001078989.1	5	0	0.00
Aebp1	NP_001278786.1	521	4	8.09	Pde4b	XP_006502918.1	1375	6	4.60
Pold2	NP_001348871.1	1000	6	6.32	Sgip1	XP_036020367.1	866	9	10.95
Myl7	NP_075017.2	1292	4	3.26	Tctex1d1	NP_001361635.1	230	11	50.40
Gck	NP_034422.2	1580	4	2.67	Insl5	NP_001277577.1	391	6	16.17
Ykt6	NP_062635.2	927	1	1.14	Wdr78	NP_666366.4	93	0	0.00
Camk2b	XP_036012160.1	1586	3	1.99	Mier1	XP_006503462.1	575	8	14.66
Nudcd3	NP_001350375.1	334	2	6.31	Slc35d1	NP_808400.1	485	11	23.90
Npc1l1	NP_997125.2	608	1	1.73	4921539E11R	XP_006503458.1	91	3	34.74
Ddx56	XP_017170139.1	1575	3	2.01	Oma1	XP_006503366.1	632	1	1.67
Tmed4	NP_598781.1	403	1	2.62	Dab1	XP_030109029.1	548	3	5.77
Ogdh	NP_001348831.1	966	4	4.36	C8b	NP_598643.1	846	11	13.70
Zmiz2	NP_082877.2	566	1	1.86	C8a	NP_001303596.1	776	11	14.94
Ppia	NP_032933.1	1921	6	3.29	Fyb2	XP_017175708.1	135	12	93.68
H2az2	NP_001333993.1	2113	3	1.50	Prkaa2	NP_835279.2	1742	7	4.23
Purb	NP_035351.1	330	3	9.58	Plpp3	NP_542122.1	689	7	10.71
Myo1g	NP_848534.2	2032	2	1.04	Usp24	XP_006503240.1	919	1	1.15
Ccm2	NP_666126.1	288	2	7.32	Pcsk9	NP_705793.1	1618	6	3.91
Nacad	XP_036012341.1	854	0	0.00	Bsnd	NP_536706.2	262	3	12.07
Tbrg4	NP_001349643.1	473	5	11.14	Dhcr24	NP_444502.2	731	2	2.88
Wap	NP_035839.3	242	2	8.71	Lexm	XP_011238830.1	144	0	0.00
Ramp3	NP_062384.1	290	3	10.90	Ttc22	NP_808335.1	58	0	0.00
Adcy1	NP_033752.1	1623	2	1.30	Pars2	NP_001077356.1	777	0	0.00
Igfbp1	NP_032367.3	1191	2	1.77	Ttc4	XP_006503471.1	1177	0	0.00
Igfbp3	XP_011241967.1	1117	2	1.89	Mroh7	XP_011238866.1	55	0	0.00
Tns3	XP_011242029.1	1192	1	0.88	Fam151a	NP_666261.1	260	0	0.00
Pkd1l1	XP_036013024.1	218	0	0.00	Acot11	NP_001334088.1	420	1	2.51
Hus1	NP_001290461.1	517	0	0.00	Ssbp3	XP_006503474.1	290	0	0.00
Sun3	XP_006514660.1	409	2	5.15	Mrpl37	NP_079776.1	389	0	0.00
Gm11992	NP_001033017.1	34	1	31.00	Cyb5rl	XP_011238784.1	271	0	0.00
Upp1	NP_033503.2	636	1	1.66	Cdcp2	XP_030109426.1	28	0	0.00
Abca13	NP_839990.2	656	4	6.43	Tceanc2	NP_079893.1	507	3	6.24
Vwc2	XP_036012653.1	245	8	34.41	Tmem59	NP_083841.4	186	2	11.33
Zpbp	NP_001172082.1	279	7	26.44	Ldlrad1	XP_006503304.1	55	2	38.32
Spata48	NP_001333997.1	127	7	58.09	Lrrc42	NP_084261.1	7	0	0.00
4930512M02F	NP_001292072.1	0	0	#DIV/0!	Hspb11	NP_082670.1	670	0	0.00
Ikzf1	XP_011242004.1	1355	7	5.44	Dio1	NP_031886.3	606	0	0.00
Fignl1	XP_030102074.1	1190	8	7.09	Yipf1	XP_006503031.1	206	0	0.00
Ddc	XP_036012174.1	731	3	4.33	Ndc1	NP_082631.1	720	1	1.46
Grb10	NP_001171100.1	730	8	11.55	Glis1	XP_006503035.1	435	0	0.00
Cobl	XP_006514548.1	329	7	22.42	Dmrtb1	XP_030109541.1	206	0	0.00

Vstm2a	NP_001277468.1	787	2	2.68	Lrp8	XP_011238756.1	692	0	0.00
Sec61g	NP_035473.2	966	4	4.36	Magoh	NP_001269666.1	1295	1	0.81
Egfr	NP_997538.1	4317	7	1.71	Czib	NP_001334089.1	186	0	0.00
Fbxo48	XP_017170091.1	64	6	98.80	Cpt2	NP_034079.2	718	3	4.40
Plek	NP_062422.1	1645	9	5.77	Slc1a7	NP_666367.3	410	1	2.57
Cnrip1	NP_084137.1	161	6	39.28	Podn	NP_766462.2	665	2	3.17
Ppp3r1	NP_077779.2	1633	8	5.16	Scp2	XP_011238779.1	838	2	2.52
Pno1	NP_079719.1	1429	7	5.16	Echdc2	NP_081004.2	754	2	2.80
Wdr92	NP_849240.1	165	6	38.32	Zyg11a	XP_006503039.1	144	0	0.00
C1d	NP_001317578.1	608	1	1.73	Zyg11b	NP_001028806.2	115	0	0.00
Etaa1	NP_080852.2	183	8	46.07	Coa7	NP_081526.1	302	1	3.49
Meis1	XP_006514622.1	1043	4	4.04	Shisal2a	NP_001092773.1	24	0	0.00
Spred2	NP_277058.1	567	6	11.15	Gpx7	NP_077160.1	843	3	3.75
Actr2	NP_666355.1	1911	6	3.31	Tut4	XP_036019916.1	590	0	0.00
Rab1a	NP_033022.1	1357	7	5.44	Prpf38a	NP_766285.2	765	2	2.76
Cep68	NP_758464.2	103	11	112.55	Orc1	NP_035145.2	1503	0	0.00
Slc1a4	NP_061349.3	459	1	2.30	Cc2d1b	NP_796019.1	290	3	10.90
Sertad2	NP_001349796.1	215	7	34.31	Zfyve9	XP_006503051.1	406	2	5.19
Aftph	NP_852076.1	173	10	60.92	Btf3l4	XP_011238912.1	712	1	1.48
Lgalsl	XP_030101642.1	167	9	56.80	Txndc12	NP_079610.1	423	2	4.98
Peli1	NP_075813.2	425	9	22.32	Kti12	NP_083847.1	233	1	4.52
Vps54	NP_620692.3	613	7	12.03	Rab3b	NP_076026.1	745	1	1.41
Ugp2	NP_647458.1	1068	9	8.88	Nrd1	NP_001334098.1	426	0	0.00
Mdh1	NP_001303604.1	1172	7	6.29	Osbpl9	NP_598646.4	257	3	12.30
Wdpcp	XP_006514694.1	216	10	48.79	Calr4	NP_001272824.1	400	0	0.00
Otx1	XP_036012308.1	543	4	7.76	Eps15	XP_006502801.2	1391	7	5.30
Ehbp1	XP_036012428.1	1114	8	7.57	Ttc39a	XP_006503057.1	121	4	34.84
Tmem17	NP_705824.1	188	4	22.42	Rnf11	NP_038904.1	660	7	11.18
B3gnt2	NP_001162585.1	291	5	18.11	4930522H14R	NP_080567.1	192	2	10.98
Commd1	NP_653097.2	639	9	14.84	Cdkn2c	NP_031697.1	1462	3	2.16
Zrsr1	NP_035793.1	466	3	6.78	Faf1	NP_032009.2	831	3	3.80
Cct4	NP_033967.1	1867	9	5.08	Dmrta2	NP_758500.2	259	1	4.07
Fam161a	XP_011242062.1	332	8	25.40	Elavl4	NP_001156869.1	1660	2	1.27
Xpo1	NP_598775.2	2797	11	4.14	Agbl4	XP_011238955.1	231	0	0.00
Usp34	XP_006514628.1	1155	8	7.30	Bend5	NP_080555.1	95	0	0.00
Ahsa2	NP_765979.3	434	16	38.85	Spata6	NP_080746.3	216	0	0.00
1700093K21R	XP_006514852.1	64	6	98.80	Slc5a9	NP_663526.3	273	0	0.00
0610010F05Ri	XP_036012887.1	272	11	42.62	Skint8	XP_017175827.1	2	0	0.00
Pex13	NP_076140.2	468	9	20.27	Skint7	NP_001136247.1	10	0	0.00
Pus10	XP_006514928.2	415	12	30.47	Skint1	NP_001096132.1	72	0	0.00
Rel	NP_033070.2	1501	6	4.21	Skint4	NP_848901.2	53	0	0.00
Papolg	NP_766143.2	817	12	15.48	Skint3	NP_001095944.1	39	0	0.00
Bcl11a	XP_011241961.1	947	9	10.02	Skint9	NP_808532.1	46	0	0.00
Fancl	NP_080199.1	486	7	15.18	Skint2	XP_017175754.1	17	0	0.00
Vrk2	NP_001239376.1	875	6	7.23	Skint10	NP_808336.1	55	0	0.00
Ccdc85a	NP_001160133.1	312	1	3.38	Skint6	NP_001096669.1	16	0	0.00
Efemp1	NP_666127.2	824	3	3.84	Gm12816	XP_036020517.1	1594	2	1.32
Pnpt1	NP_082145.1	1063	3	2.97	Skint5	NP_001161350.1	4	0	0.00
Ppp4r3b	XP_036012096.1	954	7	7.73	Skint11	NP_001159499.1	9	0	0.00
Cfap36	NP_080016.1	156	4	27.02	Trabd2b	NP_001079018.1	84	0	0.00
Ccdc88a	XP_006514518.1	426	7	17.32	Foxd2	NP_032619.1	583	1	1.81
Prorsd1	NP_080741.1	49	5	107.54	Foxe3	NP_056573.1	674	0	0.00
Mtif2	NP_001269048.1	1072	5	4.92	Cmpk1	NP_079923.3	805	4	5.24
Rps27a	NP_077239.1	2977	6	2.12	Stil	NP_033211.2	548	4	7.69
Clhc1	NP_082780.1	1255	1	0.84	Tal1	XP_006502972.1	811	3	3.90
Rtn4	NP_918943.1	1098	3	2.88	Pdzk1ip1	NP_001158029.1	180	4	23.42
Eml6	XP_036012547.1	731	2	2.88	Cyp4x1	NP_001003947.1	267	2	7.89
Fem1al	NP_789799.1	925	3	3.42	Cyp4a29	NP_001093653.1	311	2	6.78
Sptbn1	NP_787030.2	1261	3	2.51	Cyp4a12a	NP_803125.2	680	7	10.85
4930505A04R	NP_001093864.1	59	0	0.00	Cyp4a12b	NP_758510.2	574	2	3.67
Acyp2	NP_083620.1	264	1	3.99	Cyp4a30b	XP_006503285.1	214	2	9.85
Psme4	NP_598774.2	873	3	3.62	Cyp4a14	XP_006502787.2	861	3	3.67
Erlec1	XP_011242046.1	794	3	3.98	Cyp4a10	NP_034141.3	967	7	7.63
Gpr75	NP_780699.2	136	1	7.75	Cyp4a31	NP_964002.2	347	2	6.07
Chac2	NP_001277596.1	244	1	4.32	Cyp4a32	NP_001093651.1	710	3	4.45
Asb3	NP_076395.2	1406	5	3.75	Cyp4b1	NP_031849.1	738	4	5.71

Stc2	NP_035621.1	603	2	3.50	Efcab14	NP_766286.2	75	0	0.00
Bod1	NP_001020090.1	207	0	0.00	Tex38	NP_083472.1	57	0	0.00
Cpeb4	NP_080528.2	445	2	4.74	Atpaf1	NP_851383.2	408	1	2.58
4930524B15R	NP_080538.1	45	0	0.00	Mob3c	XP_036019447.1	195	0	0.00
Nsg2	XP_030101507.1	850	0	0.00	Mknk1	NP_001272416.1	367	3	8.61
Il9r	XP_017169787.1	412	3	7.67	Kncn	NP_001307474.1	176	2	11.98
Snrnp25	NP_084369.2	260	7	28.37	Dmbx1	XP_017175446.1	457	4	9.22
Rhbdf1	XP_036012182.1	460	8	18.33	Faah	NP_034303.3	670	0	0.00
Mpg	NP_034952.2	306	4	13.78	Nsun4	NP_082418.1	37	0	0.00
Nprl3	NP_853547.1	482	8	17.49	Uqcrh	NP_079917.1	861	1	1.22
Hba-x	NP_034535.1	542	7	13.61	Lrrc41	NP_705741.3	351	0	0.00
Hba-a1	NP_032244.2	688	7	10.72	Rad54l	XP_030109224.1	1557	3	2.03
Hbq1b	NP_001029153.1	260	4	16.21	Lurap1	NP_080823.1	83	5	63.49
Hba-a2	NP_001077424.1	447	7	16.50	Pomgnt1	NP_080927.1	258	0	0.00
Hbq1a	NP_778165.1	325	9	29.18	Tspan1	NP_598442.1	362	0	0.00
Sh3pxd2b	XP_006514775.1	1685	2	1.25	1700042G07F	NP_001092765.1	479	0	0.00
Ubtd2	NP_776145.2	107	2	19.70	Llph-ps1	XP_036020520.1	280	0	0.00
Efcab9	NP_081307.2	893	2	2.36	Pik3r3	XP_006502922.1	946	2	2.23
Stk10	NP_033314.2	496	5	10.62	Mast2	NP_001355991.1	560	6	11.29
Fbxw11	NP_598776.1	1068	11	10.85	Ipp	XP_006502873.1	459	0	0.00
Smim23	NP_081326.1	270	0	0.00	Tmem69	NP_808338.2	76	3	41.60
Fgf18	NP_032031.1	758	9	12.51	Gpbp1l1	XP_017175941.1	78	4	54.05
Npm1	NP_001239189.1	1575	5	3.35	Ccdc17	NP_001033005.1	61	3	51.83
Tlx3	XP_036012630.1	514	8	16.40	Nasp	NP_001271158.1	1296	2	1.63
Ranbp17	NP_075635.1	252	9	37.64	Akr1a1	NP_067448.1	698	1	1.51
Gabrp	NP_666129.1	389	10	27.09	Prdx1	NP_035164.1	1614	12	7.84
Kcnip1	NP_001177815.1	1173	9	8.09	Mmachc	NP_080238.2	341	5	15.45
Kcnmb1	NP_112446.2	278	5	18.96	Ccdc163	XP_006503421.1	13	2	162.14
Lcp2	NP_034826.2	1019	3	3.10	Tesk2	XP_030109303.1	963	7	7.66
Foxi1	NP_076396.3	739	7	9.98	Toe1	NP_080930.1	344	5	15.32
Dock2	NP_203538.2	1013	10	10.40	Mutyh	XP_036020311.1	769	8	10.96
Insyn2b	XP_006514826.1	89	1	11.84	Hpdl	NP_666368.1	281	4	15.00
Spdl1	NP_081687.2	507	2	4.16	Zswim5	XP_036020394.1	207	0	0.00
Slit3	NP_035542.2	1328	4	3.17	Urod	XP_036019871.1	1522	5	3.46
Pank3	NP_666074.1	339	0	0.00	Hectd3	NP_780453.1	941	1	1.12
Fbll1	NP_001004147.1	1709	4	2.47	Eif2b3	NP_001104747.1	806	3	3.92
Rars	NP_080212.2	1566	2	1.35	Ptch2	NP_032984.1	527	3	6.00
Wwc1	NP_740749.1	871	1	1.21	Btbd19	XP_036020450.1	83	0	0.00
Tenm2	XP_036012582.1	1200	6	5.27	Tctex1d4	NP_778195.1	70	0	0.00
Mat2b	NP_001186203.1	521	1	2.02	Plk3	NP_001300845.1	1733	2	1.22
Hmmr	XP_036012237.1	760	3	4.16	Rps8	NP_033124.1	1450	1	0.73
Nudcd2	NP_080299.4	676	3	4.68	Kif2c	NP_608301.3	1443	5	3.65
Ccng1	NP_033961.1	1846	5	2.85	Armh1	NP_001139109.1	12	2	175.65
Gabrg2	XP_036012226.1	1500	9	6.32	Tmem53	NP_001272741.1	115	4	36.66
Gabra1	XP_006532258.1	1313	8	6.42	Rnf220	XP_036020239.1	496	8	17.00
Gabra6	XP_030101419.1	603	2	3.50	Eri3	XP_030109058.2	255	6	24.80
Gabrb2	NP_001334243.1	1239	9	7.66	Dmap1	NP_075667.1	849	7	8.69
Atp10b	XP_017170095.1	622	8	13.55	Klf17	NP_083692.2	484	1	2.18
Pttg1	NP_001349728.1	956	10	11.02	Slc6a9	NP_001355945.1	655	5	8.05
Slu7	NP_683514.2	768	12	16.47	Ccdc24	XP_030109496.1	181	11	64.05
C1qtnf2	XP_036012851.1	169	6	37.42	B4galt2	XP_011238877.1	369	10	28.56
Ccnjl	NP_001038995.1	1215	3	2.60	Atp6v0b	NP_291095.1	609	3	5.19
Fabp6	NP_032401.1	466	5	11.31	Dph2	NP_080620.1	451	7	16.36
Pwwp2a	XP_006534263.1	176	0	0.00	Ipo13	NP_666264.1	407	4	10.36
Ttc1	NP_598556.1	510	13	26.86	Artn	XP_030108965.1	280	1	3.76
Adra1b	NP_001271309.1	725	7	10.18	St3gal3	XP_036019824.1	366	6	17.28
Il12b	NP_001290173.1	548	0	0.00	Kdm4a	NP_001155295.1	1569	7	4.70
Ublcp1	NP_077795.2	1191	13	11.50	Ptprf	XP_030109223.1	1032	5	5.11
Rnf145	NP_001350095.1	548	7	13.46	Szt2	NP_937813.3	188	2	11.21
Ebf1	NP_001277638.1	1014	7	7.28	Med8	XP_006503554.1	322	1	3.27
Clint1	NP_001333689.1	585	1	1.80	Elovl1	XP_006503296.1	428	1	2.46
Lsm11	NP_082461.1	234	0	0.00	Cdc20	NP_075712.2	3089	3	1.02
Thg1l	NP_001074438.1	167	8	50.49	Mpl	NP_001272425.1	402	1	2.62
Sox30	NP_775560.1	393	3	8.05	Tie1	NP_035717.2	854	3	3.70
Adam19	NP_001278819.1	481	6	13.15	2610528J11R	NP_079848.1	196	1	5.38
Nipal4	NP_766112.1	271	0	0.00	Tmem125	XP_017175638.1	102	2	20.66

Cyfip2	NP_001239388.1	836	4	5.04	Cfap57	XP_006503434.1	151	1	6.98
Fndc9	NP_796049.1	44	0	0.00	Ebna1bp2	NP_081208.1	1237	1	0.85
Itk	NP_001268897.1	1498	8	5.63	Olfr62	NP_666427.2	134	0	0.00
Fam71b	NP_001013805.1	44	1	23.95	Olfr1342	NP_666924.1	12	0	0.00
Med7	NP_079702.3	1012	1	1.04	Olfr1341	NP_667064.2	23	0	0.00
Havcr2	NP_599011.2	592	3	5.34	Olfr1340	XP_017175720.1	12	0	0.00
Gm12169	NP_001156829.1	56	1	18.82	Olfr1339	NP_667063.1	143	0	0.00
BC053393	NP_001020606.2	53	0	0.00	Olfr1338	XP_006503177.1	52	0	0.00
Dppa1	XP_017170109.1	100	1	10.54	Olfr1337	NP_666421.2	91	0	0.00
Timd2	XP_030101484.1	357	3	8.86	Olfr1335	NP_997586.1	32	0	0.00
Havcr1	XP_006532455.1	307	2	6.87	Olfr1333	NP_997040.1	71	0	0.00
Timd4	NP_848874.3	503	5	10.48	Olfr1331	NP_001011856.2	0	0	#DIV/0!
Sgcd	XP_036012592.1	448	1	2.35	Olfr1330	NP_666446.1	32	0	0.00
Rack1	NP_032169.1	2214	2	0.95	Olfr1329	NP_001011870.2	34	0	0.00
Trim41	NP_663352.2	644	1	1.64	Olfr1328	NP_666511.2	29	0	0.00
Trim7	XP_006534631.1	500	2	4.22	Lao1	NP_598653.3	575	0	0.00
Irgm1	NP_032352.1	416	6	15.20	Slc2a1	NP_035530.2	1096	0	0.00
Gm5431	NP_001019401.2	17	1	61.99	Zfp691	XP_036019816.1	193	0	0.00
Gm12185	NP_001039005.1	27	1	39.03	Ermap	XP_006503203.1	393	1	2.68
Psme2b	NP_001268401.1	262	0	0.00	Svbp	NP_001034087.1	44	1	23.95
9930111J21Ri	NP_001108151.1	56	1	18.82	Tmem269	XP_006503519.1	32	0	0.00
Tgtp1	NP_035709.3	216	2	9.76	AU022252	XP_006503079.1	41	1	25.70
9930111J21Ri	NP_775610.1	44	1	23.95	P3h1	NP_062756.2	309	1	3.41
Tgtp2	NP_001138636.1	159	2	13.26	Cldn19	NP_001033679.1	229	0	0.00
Ifi47	NP_001258606.1	461	4	9.14	Ybx1	XP_030109267.1	1430	2	1.47
Olfr1396	NP_666449.2	31	0	0.00	Ppih	NP_001103599.1	1472	1	0.72
Olfr56	NP_035129.2	47	0	0.00	Ccdc30	NP_001257364.1	31	1	34.00
Olfr1395	NP_667088.1	98	0	0.00	Ppcs	NP_080770.2	524	0	0.00
Olfr1394	NP_666388.1	49	0	0.00	Zmynd12	NP_001014900.2	60	1	17.57
Btnl9	NP_766381.1	294	0	0.00	Rimkla	NP_808240.2	246	3	12.85
Zfp62	NP_001349655.1	565	0	0.00	Frg2f1	NP_001004178.1	51	0	0.00
Mgat1	XP_017169803.1	276	0	0.00	Foxj3	NP_001344106.1	607	1	1.74
Olfr1393	XP_017170047.1	25	0	0.00	Guca2a	NP_032216.1	276	0	0.00
Olfr1392	NP_666681.1	25	0	0.00	Guca2b	NP_032217.2	263	0	0.00
Olfr10	NP_996558.1	43	0	0.00	Hivep3	NP_034787.2	590	1	1.79
Olfr1391	NP_666679.1	11	0	0.00	Edn2	NP_031928.2	595	0	0.00
Olfr1390	NP_667276.1	66	0	0.00	Foxo6	NP_918949.1	534	0	0.00
Olfr1389	NP_667277.1	90	0	0.00	Scmh1	NP_001366113.1	542	1	1.94
Olfr1388	NP_666678.1	21	0	0.00	Slfnl1	NP_808238.1	90	1	11.71
Olfr1387	NP_666684.1	146	0	0.00	Ctps	NP_001342420.1	1505	1	0.70
Olfr1386	NP_001011741.1	27	0	0.00	Gm8439	NP_001095073.1	0	0	#DIV/0!
Olfr1385	NP_001011805.1	13	0	0.00	Cited4	NP_062509.1	246	1	4.28
Olfr1384	NP_666683.1	11	1	95.81	Kcnq4	XP_011238885.1	881	1	1.20
Olfr1383	NP_997457.1	22	1	47.90	Nfyc	XP_030109167.1	937	3	3.37
Olfr1382	NP_001011790.1	31	0	0.00	Rims3	NP_891559.1	654	2	3.22
Olfr1381	NP_666680.2	10	0	0.00	Exo5	NP_082733.1	101	2	20.87
Olfr1380	NP_997456.1	52	0	0.00	Zfp69	XP_036020102.1	292	5	18.05
Flt4	NP_032055.1	1127	4	3.74	Smap2	NP_598477.2	669	6	9.45
Scgb3a1	NP_473378.1	184	0	0.00	Col9a2	NP_031767.2	446	4	9.45
Cnot6	XP_036012102.1	939	5	5.61	Zmpste24	NP_766288.1	826	4	5.10
Gfpt2	NP_038557.1	919	0	0.00	Tmco2	NP_001074781.1	145	8	58.15
Mapk9	NP_001157143.1	1365	6	4.63	Rlf	NP_001333589.1	407	2	5.18
Rasgef1c	NP_001334391.1	316	5	16.68	Gm12888	NP_001028963.1	0	0	#DIV/0!
Rnf130	NP_001277679.1	644	8	13.09	Gm12886	NP_001138420.1	0	0	#DIV/0!
Tbc1d9b	NP_001277689.1	765	10	13.78	Gm12887	NP_001092779.1	92	0	0.00
Mrnip	NP_080819.2	43	0	0.00	9530002B09R	NP_076354.1	62	0	0.00
Sqstm1	XP_036012307.1	1868	4	2.26	Ppt1	NP_032943.2	724	11	16.01
Mgat4b	NP_666038.3	258	4	16.34	Cap1	NP_001344964.1	1168	0	0.00
Ltc4s	NP_001300897.1	292	4	14.44	Mfsd2a	XP_006503540.1	392	2	5.38
Maml1	NP_780543.2	253	0	0.00	Mycl	NP_001290050.1	418	3	7.56
Canx	NP_001103969.1	1966	6	3.22	Trit1	NP_080149.2	732	11	15.84
Cby3	NP_083967.1	321	0	0.00	Bmp8b	NP_031585.2	449	7	16.43
Hnrnph1	NP_001349451.1	988	5	5.33	Oxct2b	NP_862907.2	214	9	44.32
Rufy1	NP_766145.1	473	6	13.37	Ppie	NP_062362.1	1377	12	9.18
Adamts2	NP_783574.1	533	1	1.98	Hpcal4	NP_778163.1	1752	14	8.42
Zfp354c	NP_038950.3	334	3	9.47	Nt5c1a	NP_001078971.1	317	13	43.22

Zfp879	XP_030101639.1	189	1	5.58	Heyl	NP_038933.2	392	1	2.69
Grm6	XP_036012112.1	805	1	1.31	Pabpc4	XP_006503083.1	1276	9	7.43
Zfp454	NP_001346460.1	176	0	0.00	Bmp8a	NP_031584.1	392	5	13.44
Zfp2	XP_030101795.1	280	0	0.00	Oxct2a	NP_071316.1	282	10	37.37
Zfp354b	NP_038772.1	265	3	11.93	Macf1	XP_011238719.2	1035	3	3.05
Prop1	NP_032962.1	486	8	17.35	Ndufs5	NP_001025445.1	769	7	9.59
Olfr1378	NP_667121.1	24	3	131.74	Akirin1	NP_075912.2	205	2	10.28
Olfr1377	NP_667122.1	31	0	0.00	Rhbdl2	NP_898986.2	424	8	19.88
Olfr51	NP_667120.1	63	0	0.00	Mycbp	NP_062634.2	239	5	22.05
Olfr54	NP_035127.1	43	4	98.04	Rragc	NP_059503.2	526	5	10.02
Zfp354a	XP_011247187.1	226	1	4.66	Pou3f1	NP_035271.1	590	1	1.79
BC049762	NP_001345210.1	0	0	#DIV/0!	Utp11	NP_080307.1	850	1	1.24
Clk4	NP_031740.1	1019	1	1.03	Fhl3	XP_006502829.1	688	1	1.53
Col23a1	NP_700442.2	265	1	3.98	Sf3a3	XP_036020403.1	1466	2	1.44
Phykpl	XP_006534388.1	317	3	9.97	Inpp5b	XP_006502867.1	1183	2	1.78
Hnrnpab	NP_034578.1	1786	3	1.77	Mtf1	NP_032662.3	328	0	0.00
Nhp2	NP_080907.1	1912	2	1.10	1110065P20R	NP_001334125.1	29	0	0.00
Rmnd5b	NP_079622.1	368	0	0.00	Yrdc	NP_705794.2	677	1	1.56
N4bp3	XP_030101617.1	47	1	22.42	Maneal	NP_001007574.1	347	2	6.07
0610009B22R	NP_079595.1	403	3	7.85	Epha10	NP_001243361.1	1156	3	2.74
Sec24a	NP_001277714.1	775	3	4.08	Cdca8	NP_080836.3	718	0	0.00
Sar1b	NP_079811.1	951	5	5.54	Rspo1	NP_619624.2	910	0	0.00
Jade2	NP_001344517.1	455	0	0.00	Gnl2	XP_036019938.1	958	4	4.40
Cdkn2aipnl	NP_084252.1	158	1	6.67	Dnali1	NP_780432.1	322	1	3.27
Ube2b	NP_001349615.1	2066	6	3.06	Snip1	NP_780455.2	545	2	3.87
Cdkl3	XP_036012392.1	197	2	10.70	Meaf6	NP_081586.1	398	0	0.00
Ppp2ca	NP_062284.1	2818	4	1.50	Zc3h12a	NP_694799.1	500	1	2.11
Olfr1373	NP_997110.1	10	0	0.00	Grik3	NP_001074566.1	664	1	1.59
Olfr1371	NP_997136.1	12	0	0.00	Csf3r	NP_031808.2	650	0	0.00
Skp1	NP_035673.3	2866	8	2.94	Mrps15	NP_079820.2	1225	1	0.86
Tcf7	XP_030101627.1	1103	2	1.91	Oscp1	NP_001343375.1	169	0	0.00
Vdac1	NP_001349622.1	1102	4	3.83	Lsm10	NP_620046.1	968	3	3.27
9530068E07R	NP_694757.1	157	1	6.71	Stk40	NP_001139299.1	1192	3	2.65
Fstl4	NP_796033.2	304	2	6.93	Eva1b	XP_036019939.1	63	1	16.73
Hspa4	NP_032326.3	2704	5	1.95	Sh3d21	XP_030109569.1	270	2	7.81
Zcchc10	NP_080755.2	147	0	0.00	Thrap3	NP_001343384.1	567	0	0.00
Aff4	NP_291043.1	526	0	0.00	Map7d1	NP_001139442.1	317	1	3.32
Leap2	NP_694709.1	589	1	1.79	Trappc3	NP_038746.1	477	1	2.21
Uqcrq	NP_001313543.1	634	1	1.66	Col8a2	XP_017175755.1	388	1	2.72
Gdf9	NP_032136.2	762	1	1.38	Adprhl2	NP_598644.1	217	0	0.00
Shroom1	NP_082193.2	209	2	10.09	Tekt2	NP_001344191.1	346	0	0.00
Sowaha	NP_898996.2	171	2	12.33	Ago3	XP_006502980.1	800	2	2.63
Septin8	NP_001334425.1	514	3	6.15	Ago1	NP_001304102.1	1161	2	1.82
Kif3a	XP_006532388.1	1434	7	5.14	Ago4	NP_694817.2	949	1	1.11
Il4	NP_067258.1	2008	9	4.72	Clspn	XP_006503185.1	724	1	1.46
Il13	NP_032381.1	1358	7	5.43	5730409E04R	NP_001013777.2	4	0	0.00
Rad50	NP_033038.2	841	0	0.00	Psmb2	NP_036100.3	1301	2	1.62
Il5	NP_034688.1	948	6	6.67	Tfap2e	NP_945198.2	424	2	4.97
Irf1	NP_001152865.1	997	7	7.40	Ncdn	NP_036116.3	491	4	8.59
Slc22a5	XP_006532713.1	515	8	16.37	AU040320	NP_001030602.1	239	0	0.00
Slc22a21	XP_006533868.1	416	4	10.13	Zmym4	NP_001344130.1	729	2	2.89
Slc22a4	NP_062661.1	460	8	18.33	Sfpq	XP_036020326.1	1021	4	4.13
Pdlim4	NP_062290.2	735	7	10.04	Zmym1	NP_080946.3	231	0	0.00
P4ha2	XP_006532515.1	527	4	8.00	Zmym6	NP_803413.1	167	1	6.31
Csf2	NP_034099.2	1545	7	4.77	Tmem35b	NP_001092789.1	14	0	0.00
Il3	NP_034686.2	999	4	4.22	Dlgap3	XP_030109431.1	904	2	2.33
Acsl6	XP_030101662.1	1172	5	4.50	Smim12	NP_084528.1	101	2	20.87
Meikin	NP_083381.1	63	0	0.00	Gja4	NP_032146.1	313	3	10.10
Fnip1	NP_776114.2	373	7	19.78	Gjb3	NP_001153484.1	387	3	8.17
Rapgef6	XP_006532623.1	457	5	11.53	Gjb4	XP_017175464.1	170	3	18.60
Cdc42se2	XP_030102229.1	262	5	20.11	Gjb5	NP_034421.1	216	3	14.64
Lyrm7	NP_001350120.2	284	4	14.84	CK137956	NP_001128205.1	4	0	0.00
Hint1	NP_032274.1	1022	6	6.19	Csmd2	XP_011238862.1	474	1	2.22
Gpx3	NP_001316789.1	920	3	3.44	Hmgb4	NP_081312.2	829	1	1.27
Tnip1	XP_030102056.1	476	3	6.64	Zscan20	XP_011238847.1	250	5	21.08
Anxa6	NP_001103681.1	665	4	6.34	Tlr12	NP_991392.1	644	1	1.64

Ccdc69	NP_803422.2	114	1	9.24	Phc2	XP_017175809.1	418	4	10.09
Gm2a	NP_034429.1	413	1	2.55	A3galt2	NP_001009819.1	179	4	23.55
Slc36a3	XP_011247200.1	229	1	4.60	Zfp362	NP_001074567.1	374	4	11.27
Slc36a2	XP_006533387.1	469	1	2.25	Trim62	NP_001357685.1	431	4	9.78
Slc36a1	NP_694779.3	504	2	4.18	Azin2	NP_001288770.1	482	1	2.19
Fat2	XP_030101863.1	375	2	5.62	Ak2	NP_058591.2	1101	2	1.91
Sparc	NP_001277746.1	1151	7	6.41	Rnf19b	NP_001355692.1	660	1	1.60
Atox1	NP_033850.1	582	0	0.00	Tmem54	NP_079728.2	117	2	18.02
G3bp1	NP_038744.1	1144	2	1.84	Hpca	XP_030109084.1	1770	0	0.00
Glra1	NP_065238.2	513	3	6.16	Fndc5	XP_006503275.1	371	1	2.84
Nmur2	NP_694719.2	597	2	3.53	S100pbp	NP_001334132.1	110	3	28.74
Gria1	NP_032191.2	1952	2	1.08	Yars	NP_598912.5	2025	2	1.04
Fam114a2	XP_030102110.1	129	2	16.34	C77080	NP_001028361.1	42	0	0.00
Mfap3	XP_006532913.1	139	3	22.75	Sync	XP_006503437.1	179	1	5.89
Galnt10	NP_598950.2	319	2	6.61	Rbbp4	NP_033056.2	2037	5	2.59
Sap30l	NP_001074637.1	243	4	17.35	Zbtb8os	XP_017175850.1	122	0	0.00
Hand1	NP_032239.1	405	0	0.00	Zbtb8a	NP_082879.1	228	5	23.11
Larp1	NP_082727.1	669	3	4.73	Zbtb8b	XP_017175590.1	237	4	17.79
Cnot8	NP_001343406.1	525	2	4.01	Bsdc1	NP_598650.2	92	0	0.00
Gemin5	XP_036012438.1	380	1	2.77	Tssk3	XP_030109549.1	1138	7	6.48
Mrpl22	XP_017169936.1	1130	0	0.00	Fam229a	NP_001078960.1	1	1	1053.90
Igtp	NP_061208.3	346	1	3.05	Marcksl1	NP_034937.1	404	2	5.22
Irgm2	NP_062313.3	287	1	3.67	Hdac1	NP_032254.1	3392	6	1.86
4930438A08R	NP_001361634.1	533	1	1.98	Lck	NP_001155904.1	2071	4	2.04
Zfp692	NP_001035776.1	288	1	3.66	Fam167b	NP_877584.2	139	0	0.00
Zfp672	XP_017170079.1	220	0	0.00	Eif3i	NP_061269.1	1346	3	2.35
Sh3bp5l	NP_077800.3	209	0	0.00	Tmem234	NP_084024.1	41	1	25.70
Lypd8	NP_001317126.1	255	1	4.13	Dcdc2b	NP_001182659.1	117	0	0.00
1810065E05R	NP_081515.2	11	0	0.00	Iqcc	XP_036019947.1	13	0	0.00
Gm12253	NP_001039007.1	5	0	0.00	Ccdc28b	XP_036020200.1	153	1	6.89
Lypd9	NP_997410.1	205	1	5.14	Txlna	NP_001186624.1	276	0	0.00
Olfr30	NP_667089.1	38	0	0.00	Kpna6	NP_032494.3	978	2	2.16
Olfr332	NP_001011770.2	38	0	0.00	Tmem39b	NP_955009.1	61	0	0.00
Olfr331	NP_001011861.3	76	0	0.00	Khdrbs1	NP_035447.3	1095	2	1.92
Olfr330	NP_667090.1	27	0	0.00	Ptp4a2	XP_017175534.1	489	3	6.47
Olfr329	NP_001011531.3	54	0	0.00	Spocd1	XP_017175994.1	328	0	0.00
Olfr328	NP_666713.2	27	0	0.00	Adgrb2	XP_011238809.1	223	0	0.00
Olfr224	NP_997578.1	27	0	0.00	Col16a1	XP_006502703.1	509	2	4.14
Olfr325	NP_997036.2	54	0	0.00	Pef1	NP_080717.2	380	0	0.00
Olfr324	NP_001011743.2	10	0	0.00	Hcrtr1	XP_017175645.1	385	0	0.00
Lypd8l	NP_653127.2	32	0	0.00	Tinagl1	NP_075965.2	450	2	4.68
Olfr323	NP_666488.2	81	0	0.00	Ldc1	XP_006503256.1	479	0	0.00
Trim58	NP_001034136.1	519	7	14.21	Serinc2	XP_006503126.1	297	8	28.39
Olfr322	NP_997576.1	90	1	11.71	Fabp3	NP_034304.1	640	7	11.53
Olfr320	NP_997113.1	84	1	12.55	Zcchc17	NP_694800.1	995	7	7.41
Olfr319	NP_666711.2	102	1	10.33	Snrnp40	NP_079921.2	885	5	5.95
Olfr318	NP_666712.2	143	1	7.37	Nkain1	NP_080274.2	241	8	34.98
Olfr317	NP_001011769.2	63	1	16.73	Pum1	XP_006539354.1	1185	8	7.11
Olfr316	NP_001011818.2	60	1	17.57	Sdc3	NP_035650.2	493	7	14.96
Olfr315	NP_666749.1	14	0	0.00	Laptm5	NP_034816.1	624	5	8.44
Olfr314	NP_001011760.1	117	1	9.01	Matn1	NP_034899.2	428	1	2.46
Fam183b	NP_001156350.2	41	0	0.00	Ptpru	XP_006538737.1	715	10	14.74
Olfr313	NP_666747.2	28	0	0.00	Mecr	XP_006538948.1	660	4	6.39
Olfr312	NP_001011819.1	65	0	0.00	Srsf4	NP_065612.2	1226	7	6.02
Olfr311	NP_666748.2	94	0	0.00	Tmem200b	NP_001188296.1	77	4	54.75
Gm12258	NP_001359510.1	132	0	0.00	Epb41	XP_017175732.1	681	3	4.64
2810021J22Ri	XP_030102152.1	154	0	0.00	Oprd1	NP_038650.3	644	4	6.55
Zfp39	NP_035888.2	258	1	4.08	Ythdf2	NP_663368.3	490	3	6.45
Btnl10	NP_619619.2	227	3	13.93	Gmeb1	XP_017175821.1	290	2	7.27
Rnf187	NP_071868.2	323	3	9.79	Taf12	XP_036020207.1	698	1	1.51
H2bu2	NP_084358.1	1078	5	4.89	Rab42	NP_001075120.1	1150	0	0.00
H2aw	NP_835736.1	1079	6	5.86	Trnau1ap	XP_006539243.1	1372	1	0.77
Trim17	NP_112449.1	426	3	7.42	Rcc1	NP_598639.1	1565	3	2.02
Trim11	NP_001277917.1	605	4	6.97	Phactr4	XP_036019431.1	405	1	2.60
Obscn	NP_001164983.2	2172	10	4.85	Med18	NP_080315.1	234	1	4.50
Iba57	NP_776146.1	306	2	6.89	Sesn2	NP_659156.1	514	0	0.00

Gjc2	XP_036012142.1	316	0	0.00	Atpif1	XP_030108966.1	519	0	0.00
Guk1	NP_032219.2	1391	3	2.27	Dnajc8	NP_765988.2	430	1	2.45
Mrpl55	NP_001349837.1	498	2	4.23	Ptafr	NP_001074680.1	749	2	2.81
2310033P09R	NP_077172.1	93	2	22.66	Eya3	XP_006538600.1	360	1	2.93
Arf1	NP_001123880.1	2214	2	0.95	Xkr8	NP_958756.1	149	0	0.00
Wnt3a	NP_033548.1	1315	2	1.60	Smpdl3b	NP_598649.1	324	0	0.00
Wnt9a	NP_647459.1	766	2	2.75	Rpa2	NP_035414.3	1235	2	1.71
Prss38	NP_001038986.1	126	0	0.00	Themis2	NP_001028480.1	498	4	8.47
Snap47	NP_001343381.1	462	4	9.12	Ppp1r8	NP_001277654.1	527	2	4.00
Jmjd4	NP_848774.1	188	3	16.82	Stx12	NP_598648.1	912	1	1.16
Zfp867	XP_011247286.1	128	0	0.00	Fam76a	NP_001157264.1	171	0	0.00
Zkscan17	XP_006533448.1	282	1	3.74	Fgr	NP_034338.3	1324	4	3.18
Nlrp3	NP_001346567.1	1108	0	0.00	Ahdc1	NP_666267.3	66	0	0.00
Olfr222	NP_001011789.1	76	1	13.87	Wasf2	NP_700472.1	709	2	2.97
Olfr223	NP_666640.1	43	0	0.00	Gpr3	NP_032180.1	447	3	7.07
Olfr225	NP_001011740.2	11	0	0.00	Cd164l2	NP_081428.1	46	0	0.00
Mprip	NP_001355860.1	359	4	11.74	Map3k6	NP_057902.5	357	0	0.00
Pld6	NP_001277212.1	537	2	3.93	Sytl1	NP_113570.2	371	3	8.52
Flcn	XP_036012443.1	372	1	2.83	Tmem222	NP_079943.2	111	0	0.00
Cops3	NP_036121.1	831	11	13.95	Wdtc1	XP_006538808.1	1540	3	2.05
Nt5m	NP_598790.1	330	11	35.13	Slc9a1	NP_058677.1	741	0	0.00
Med9	NP_619616.2	242	11	47.90	Tent5b	NP_780516.1	168	0	0.00
Rasd1	NP_033052.1	1752	11	6.62	Trnp1	NP_001074625.1	347	0	0.00
Pemt	XP_006532541.1	631	13	21.71	Kdf1	NP_001077385.1	126	1	8.36
Rai1	XP_036012349.1	766	9	12.38	Nudc	NP_035078.1	887	0	0.00
Srebf1	NP_001300908.1	1258	7	5.86	Nr0b2	NP_035980.1	1219	1	0.86
Tom1l2	XP_036012447.1	560	18	33.88	Gpatch3	NP_766464.1	117	0	0.00
Drc3	XP_036012946.1	701	17	25.56	Gpn2	NP_598645.2	1305	1	0.81
Atpaf2	NP_663402.2	582	19	34.41	Sfn	NP_061224.2	2762	2	0.76
Gid4	NP_080033.3	207	18	91.64	Zdhhc18	XP_017175794.1	229	1	4.60
Drg2	NP_067329.1	836	18	22.69	Pigv	NP_001139428.1	335	0	0.00
Myo15	NP_034992.2	1257	15	12.58	Arid1a	XP_006539389.1	863	2	2.44
Alkbh5	NP_766531.2	318	12	39.77	Rps6ka1	NP_001272434.1	856	0	0.00
Llgl1	NP_001152877.1	700	18	27.10	Hmgn2	NP_058653.1	195	0	0.00
Flii	NP_001289136.1	925	16	18.23	Dhdds	NP_001349888.1	629	0	0.00
Mief2	NP_001009927.1	230	11	50.40	Lin28a	NP_665832.1	1220	0	0.00
Top3a	NP_033436.1	1166	12	10.85	Crybg2	XP_006538811.1	164	1	6.43
Smcr8	NP_001078909.1	261	16	64.61	Cd52	NP_038734.1	384	1	2.74
Shmt1	XP_006532707.1	1404	16	12.01	Ubxn11	XP_006539187.1	335	0	0.00
Dhrs7b	NP_663403.1	317	20	66.49	Sh3bgrl3	NP_542126.1	225	1	4.68
Tmem11	NP_775655.1	403	18	47.07	Cep85	XP_036020301.1	123	1	8.57
Natd1	NP_079570.1	80	19	250.30	Zpld2	NP_001074181.1	6	0	0.00
Map2k3	NP_032954.1	742	7	9.94	Catsper4	NP_001123502.1	246	1	4.28
Kcnj12	XP_030101466.1	441	8	19.12	Cnksr1	NP_001074516.1	324	0	0.00
Tnfrsf13b	NP_067324.1	468	5	11.26	Zfp593	NP_077177.2	736	2	2.86
Usp22	NP_001004143.2	706	6	8.96	Grrp1	XP_006539260.1	84	0	0.00
Aldh3a1	NP_001318041.1	1193	4	3.53	Pdik1l	NP_666268.1	491	4	8.59
Aldh3a2	XP_006532094.1	1045	8	8.07	Trim63	NP_001034137.2	995	5	5.30
Slc47a2	NP_001028714.1	534	8	15.79	Slc30a2	NP_001034766.1	344	2	6.13
Slc47a1	XP_006534056.1	830	11	13.97	Extl1	NP_062524.2	245	4	17.21
Rnf112	XP_036012528.1	1537	6	4.11	Pafah2	NP_001272801.1	347	3	9.11
Mfap4	NP_001334474.1	644	2	3.27	Stmn1	NP_062615.1	1335	3	2.37
Mapk7	XP_006533340.1	2453	8	3.44	Paqr7	XP_036020344.1	251	1	4.20
B9d1	NP_038745.1	423	2	4.98	Aunip	NP_001074568.1	268	1	3.93
Epn2	XP_030101396.1	711	6	8.89	Mtfr1l	NP_001342116.1	36	0	0.00
Grap	NP_082093.1	1141	8	7.39	Selenon	NP_083376.2	239	0	0.00
Slc5a10	XP_006532041.1	229	6	27.61	Man1c1	NP_997120.1	262	0	0.00
Fam83g	NP_848733.2	96	6	65.87	Ldlrap1	NP_663529.2	557	3	5.68
Prpsap2	NP_001351016.1	728	1	1.45	Maco1	NP_079658.2	243	5	21.69
Ulk2	XP_030101926.1	703	6	8.99	Rhd	NP_035400.3	271	4	15.56
Akap10	NP_064305.2	233	7	31.66	Tmem50a	NP_082211.1	111	4	37.98
Specc1	NP_001268747.1	545	2	3.87	Rsrp1	NP_076154.3	85	4	49.60
Adora2b	NP_031439.2	771	2	2.73	Syf2	NP_081056.1	653	6	9.68
Zswim7	NP_081474.1	107	0	0.00	Runx3	NP_062706.2	914	2	2.31
Ttc19	XP_006534384.1	799	3	3.96	Clic4	NP_038913.1	651	3	4.86
Ncor1	NP_001239242.1	2290	4	1.84	Srrm1	XP_036020123.1	873	5	6.04

Pigl	NP_001034625.1	277	1	3.80	Ncmap	NP_001230235.1	78	0	0.00
Cenpv	NP_082724.1	212	0	0.00	Rcan3	XP_017175806.1	323	3	9.79
Ubb	NP_035794.1	3266	6	1.94	Nipal3	NP_001334149.1	228	4	18.49
Trpv2	NP_035836.2	531	5	9.92	Stpg1	XP_006539352.1	34	0	0.00
Lrrc75a	NP_942561.1	55	0	0.00	Grhl3	XP_006538823.1	395	1	2.67
Mmgt2	XP_011247211.2	158	1	6.67	Ifnlr1	NP_777276.3	279	1	3.78
Zfp287	NP_001369752.1	195	7	37.83	Il22ra1	NP_839988.1	502	2	4.20
Zfp286	XP_030101554.1	150	9	63.23	Myom3	NP_001078978.1	206	0	0.00
Trim16	NP_444399.2	205	7	35.99	Srsf10	XP_011248490.1	1777	4	2.37
Fbxw10	NP_001278370.1	655	7	11.26	Pnrc2	NP_080659.1	383	4	11.01
Tvp23b	NP_080486.1	180	6	35.13	Cnr2	NP_034054.3	553	2	3.81
Cdrt4	NP_079772.1	142	6	44.53	Fuca1	NP_077205.3	523	2	4.03
Tekt3	NP_081936.1	490	9	19.36	Hmgcl	NP_032280.2	813	0	0.00
Pmp22	XP_030101521.1	386	1	2.73	Gale	NP_001343422.1	778	0	0.00
Hs3st3b1	NP_061275.2	243	7	30.36	Lypla2	NP_036072.1	443	1	2.38
Cox10	NP_848466.1	864	11	13.42	Pithd1	NP_079687.3	121	2	17.42
Hs3st3a1	NP_849201.1	149	6	42.44	Eloa	NP_038764.2	512	3	6.18
Elac2	XP_006534128.1	831	7	8.88	Rpl11	NP_080195.1	1333	3	2.37
Arhgap44	XP_006532936.1	535	8	15.76	Id3	NP_032347.1	396	3	7.98
Myocd	NP_666498.2	568	4	7.42	E2f2	NP_808401.1	998	3	3.17
Map2k4	NP_033183.1	644	2	3.27	Asap3	NP_001008233.1	494	4	8.53
Zkscan6	NP_080383.2	208	9	45.60	Tcea3	XP_006538773.1	677	4	6.23
Dnah9	XP_006533276.1	842	13	16.27	Zfp46	NP_001357580.1	274	4	15.39
Shisa6	XP_036012708.1	305	7	24.19	Hnrnpr	XP_006539285.1	1055	3	3.00
Pirt	NP_848771.1	308	0	0.00	Htr1d	XP_017175475.1	506	1	2.08
Tmem220	NP_796366.1	51	0	0.00	Luzp1	XP_030109448.1	219	2	9.62
Adprm	NP_079786.2	138	0	0.00	Kdm1a	NP_598633.2	1612	1	0.65
Sco1	NP_001035115.1	382	0	0.00	Tex46	XP_011248629.1	82	0	0.00
Myh3	NP_001093105.1	1297	3	2.44	Lactbl1	NP_001230191.1	88	0	0.00
Myh2	NP_001034634.2	1246	6	5.07	Ephb2	XP_030109051.1	1684	3	1.88
Myh1	XP_017169807.1	1683	6	3.76	C1qb	NP_033907.1	855	1	1.23
Myh4	NP_034985.2	1356	5	3.89	C1qc	XP_017175416.1	756	1	1.39
Myh8	XP_036012291.1	1305	4	3.23	C1qa	NP_031598.2	1200	3	2.63
Myh13	NP_001074719.1	1082	3	2.92	Epha8	XP_036019550.1	1266	3	2.50
Gas7	XP_006532265.1	1851	10	5.69	Zbtb40	XP_006538826.1	328	2	6.43
Glp2r	XP_006534625.1	367	2	5.74	Wnt4	NP_033549.1	1054	4	4.00
Rcvrn	NP_033064.1	1087	8	7.76	Cdc42	XP_030108999.1	3361	7	2.19
Gsg1l2	NP_001333986.1	54	0	0.00	Cela3a	NP_001119790.1	211	0	0.00
Dhrs7c	NP_001013031.2	442	4	9.54	Cela3b	NP_080695.1	307	0	0.00
Usp43	NP_001277978.1	966	11	12.00	1700013G24F	NP_081339.1	225	0	0.00
Cfap52	XP_006534328.1	631	3	5.01	Hspg2	XP_030109089.1	1001	4	4.21
Stx8	XP_030102021.1	611	1	1.72	Ldlrad2	XP_006539076.1	17	0	0.00
Ntn1	NP_032770.2	930	2	2.27	Usp48	XP_017175494.1	781	1	1.35
Pik3r5	XP_006533611.1	517	3	6.12	Rap1gap	XP_006538538.1	525	1	2.01
Pik3r6	NP_001075035.1	265	0	0.00	Alpl	XP_017175413.1	1184	5	4.45
Mfsd6l	NP_666116.1	126	0	0.00	Ece1	NP_001356106.1	488	3	6.48
Ccdc42	NP_808447.2	150	0	0.00	Eif4g3	XP_006538839.1	1233	2	1.71
Myh10	XP_036012982.1	2132	9	4.45	Hp1bp3	XP_006538629.1	748	2	2.82
Ndel1	XP_030102302.1	734	5	7.18	Sh2d5	NP_001093101.1	149	0	0.00
Rnf222	NP_796034.2	69	0	0.00	Kif17	NP_034753.1	1144	1	0.92
Rpl26	NP_033106.1	1517	6	4.17	Ddost	NP_031864.2	787	6	8.03
Odf4	NP_665689.1	94	0	0.00	Pink1	NP_081156.2	1145	5	4.60
Arhgef15	XP_011247414.1	559	0	0.00	Cda	NP_082452.1	936	0	0.00
Slc25a35	NP_082324.1	154	0	0.00	Fam43b	NP_001075141.1	317	4	13.30
Rangrf	NP_001272370.1	233	3	13.57	Mul1	NP_080965.2	344	7	21.45
Pfas	NP_001152991.1	916	6	6.90	Camk2n1	NP_079727.1	523	4	8.06
Ctc1	XP_006534151.1	198	9	47.90	Vwa5b1	XP_036020417.1	56	0	0.00
Aurkb	NP_035626.1	2187	3	1.45	Ubxn10	NP_848786.1	133	1	7.92
Borcs6	NP_082281.2	72	1	14.64	Pla2g2c	NP_032894.2	196	1	5.38
Tmem107	NP_080114.1	157	7	46.99	Pla2g2f	NP_036175.2	214	1	4.92
Vamp2	NP_033523.1	1491	7	4.95	Pla2g2d	NP_035239.1	345	1	3.05
Per1	XP_036012313.1	401	3	7.88	Pla2g5	XP_017175532.1	453	1	2.33
Hes7	NP_149030.2	387	7	19.06	Gm13030	NP_001295416.1	11	0	0.00
Aloxe3	NP_035916.2	381	7	19.36	Pla2g2e	NP_036174.1	188	3	16.82
Alox12b	NP_033789.1	334	7	22.09	Otud3	NP_082729.1	175	7	42.16
Alox8	NP_033791.1	255	5	20.66	Rnf186	NP_080062.5	661	2	3.19

Gucy2e	NP_032218.2	798	5	6.60	Tmco4	NP_084133.1	199	0	0.00
Cntrob	NP_766148.2	168	1	6.27	Htr6	NP_067333.1	421	0	0.00
Trappc1	NP_001019377.1	584	3	5.41	Nbl1	XP_036019702.1	192	0	0.00
Kcnab3	NP_034729.3	597	1	1.77	Micos10	NP_001156478.1	317	0	0.00
Chd3	XP_036012461.1	1741	3	1.82	Capzb	NP_033928.1	1202	2	1.75
Cyb5d1	NP_001038990.1	747	1	1.41	Slc66a1	NP_663359.2	220	0	0.00
Naa38	NP_084359.1	1335	2	1.58	Akr7a5	NP_079613.3	536	6	11.80
Tmem88	NP_080191.3	211	4	19.98	Mrto4	NP_076025.1	1615	3	1.96
Kdm6b	NP_001017426.1	3620	2	0.58	Emc1	NP_666269.2	335	4	12.58
Dnah2	XP_036012664.1	735	3	4.30	Ubr4	NP_001153791.1	1125	0	0.00
Efnb3	XP_006532223.1	728	3	4.34	Iffo2	NP_001192102.1	89	2	23.68
Wrap53	NP_001351698.1	493	3	6.41	Aldh4a1	NP_780647.3	915	3	3.46
Trp53	NP_035770.2	5490	10	1.92	Tas1r2	NP_114079.1	463	4	9.10
Atp1b2	NP_038201.1	758	3	4.17	Pax7	XP_006538694.1	1098	1	0.96
Shbg	XP_011247140.1	546	2	3.86	Klhdc7a	NP_775603.2	135	1	7.81
Sat2	XP_006534173.1	303	1	3.48	Igsf21	XP_006538862.1	429	5	12.28
Fxr2	NP_035944.2	608	5	8.67	Arhgef10l	NP_001106194.1	249	0	0.00
Sox15	NP_033261.1	447	8	18.86	Rcc2	XP_036019464.1	903	2	2.33
Mpdu1	NP_001288639.1	198	6	31.94	Padi6	NP_694746.2	229	1	4.60
Cd68	NP_001277987.1	1433	3	2.21	Padi4	NP_035191.2	300	0	0.00
Eif4a1	NP_659207.1	2331	7	3.16	Padi3	NP_035190.3	192	0	0.00
Senp3	NP_109627.3	519	6	12.18	Padi1	NP_035189.1	187	0	0.00
Tnfsfm13	NP_001152975.1	52	0	0.00	Padi2	XP_006538695.1	312	0	0.00
Tnfsf13	NP_001152977.1	266	3	11.89	Sdhb	NP_075863.2	1390	0	0.00
Tnfsf12	NP_035744.1	178	3	17.76	Atp13a2	XP_006539299.1	777	3	4.07
Polr2a	NP_001277997.1	2818	4	1.50	Mfap2	XP_006538675.1	303	2	6.96
Slc35g3	NP_063924.2	122	0	0.00	Crocc	XP_006538865.1	380	1	2.77
Zbtb4	NP_001350107.1	440	1	2.40	Necap2	NP_079659.1	387	1	2.72
Chrnb1	XP_006532066.1	592	6	10.68	Spata21	XP_006539018.1	949	1	1.11
Fgf11	XP_006532251.1	650	5	8.11	Szrd1	NP_001264124.1	83	0	0.00
Tmem102	NP_001028605.2	126	1	8.36	Fbxo42	NP_766106.2	555	5	9.49
Spem2	XP_006532033.1	142	0	0.00	Cplane2	NP_001074643.1	1302	4	3.24
Spem1	NP_083131.1	188	1	5.61	Arhgef19	XP_017175587.1	473	4	8.91
Nlgn2	XP_006532965.1	798	2	2.64	Epha2	NP_034269.2	1612	6	3.92
Tmem256	XP_036012853.1	345	5	15.27	Fam131c	NP_001078982.1	59	1	17.86
Plscr3	NP_076053.1	405	1	2.60	Clcnka	NP_077723.3	303	4	13.91
Tnk1	NP_114086.3	448	3	7.06	Clcnkb	XP_006539130.1	328	2	6.43
Tmem95	NP_001182639.1	64	0	0.00	Hspb7	NP_038896.2	729	6	8.67
Kctd11	NP_694783.1	308	2	6.84	Srarp	NP_001028546.1	40	1	26.35
Acap1	NP_722483.2	668	8	12.62	Zbtb17	NP_001365755.1	416	6	15.20
2810408A11R	NP_001303626.1	279	0	0.00	Spen	XP_036020160.1	695	2	3.03
Neurl4	NP_001013432.1	292	3	10.83	Fblim1	XP_006539276.1	746	5	7.06
Gps2	XP_030102047.1	360	6	17.57	Tmem82	NP_666099.2	80	1	13.17
Eif5a	XP_030101910.1	1612	5	3.27	Slc25a34	NP_001013802.1	144	0	0.00
Ybx2	XP_036012737.1	893	7	8.26	Plekhm2	NP_001334166.1	99	0	0.00
Slc2a4	NP_033230.2	1496	5	3.52	Rsc1a1	NP_076033.4	465	0	0.00
Cldn7	NP_001180548.1	484	0	0.00	Ddi2	NP_001017966.1	465	0	0.00
Elp5	NP_061210.2	201	0	0.00	Agmat	NP_001365791.1	995	1	1.06
Ctdnep1	NP_080293.1	599	7	12.32	Dnajc16	XP_036019839.1	593	2	3.55
Gabarap	NP_062723.1	1168	6	5.41	Casp9	NP_056548.2	1501	0	0.00
Phf23	NP_084340.2	538	1	1.96	Cela2a	NP_031945.1	430	1	2.45
Dvl2	NP_031914.3	1255	3	2.52	Efhd2	NP_080270.2	400	2	5.27
Acadvl	NP_059062.1	766	2	2.75	Fhad1	NP_808536.2	153	1	6.89
Dlg4	NP_001357604.1	2342	1	0.45	Tmem51	NP_663377.1	160	1	6.59
Asgr1	XP_017169720.1	1016	1	1.04	Kazn	XP_036020330.1	322	1	3.27
Asgr2	NP_031519.1	682	3	4.64	Prdm2	XP_011248477.1	433	2	4.87
Mgl2	XP_006532975.1	485	3	6.52	Pdpn	NP_001277751.1	696	1	1.51
Clec10a	NP_001191181.1	600	3	5.27	Lrrc38	XP_006538918.1	557	1	1.89
Slc16a11	NP_694721.2	183	4	23.04	Pramel1	XP_036020468.1	74	1	14.24
Slc16a13	XP_006534176.1	187	3	16.91	Pramel12	NP_001342653.1	14	0	0.00
Bcl6b	NP_031554.1	426	0	0.00	Oog4	NP_001334167.1	229	6	27.61
0610010K14R	NP_001171077.1	173	1	6.09	Gm13043	XP_017175815.1	91	3	34.74
Rnasek	NP_776103.1	52	0	0.00	Gm13040	NP_001107208.1	36	1	29.28
Alox12	NP_031466.2	384	4	10.98	Gm13057	NP_001107207.1	15	0	0.00
Alox12e	XP_006532098.1	221	3	14.31	Pramel20	NP_001007580.1	118	4	35.73
Alox15	XP_006532099.1	502	7	14.70	Pramel21	NP_001119796.1	5	0	0.00

Pelp1	NP_083507.3	403	1	2.62	Pramel22	XP_011248580.1	121	3	26.13
Arrb2	NP_001258287.1	2129	2	0.99	Pramel23	NP_001343501.1	143	4	29.48
Med11	NP_079673.2	138	0	0.00	Pramel24	NP_001078881.1	131	7	56.32
Cxcl16	NP_075647.3	1125	2	1.87	Pramel25	NP_001007078.2	202	11	57.39
Zmynd15	NP_001025100.1	670	1	1.57	Pramel26	NP_001334186.1	179	7	41.21
Tm4sf5	NP_083636.2	87	0	0.00	Pramel27	NP_808239.2	141	4	29.90
Vmo1	NP_001013625.1	456	1	2.31	Pramel11	NP_001078883.2	34	3	92.99
Gltpd2	NP_666132.1	563	1	1.87	Pramel16	NP_001119787.1	121	3	26.13
Psmb6	NP_032972.3	1215	4	3.47	Pramel14	NP_001079009.1	26	2	81.07
Pld2	NP_001289404.1	1285	5	4.10	Pramel4	XP_006539048.2	52	0	0.00
Mink1	XP_006533740.1	564	2	3.74	Gm13102	XP_017175368.1	94	0	0.00
Chrne	XP_036012125.1	647	7	11.40	Oog3	NP_957710.2	268	10	39.32
4930544D05R	XP_006534029.1	2	1	526.95	Oog2	XP_006539054.1	179	5	29.44
Gp1ba	NP_034456.2	793	2	2.66	Pramel29	NP_001171013.1	168	5	31.37
Slc25a11	NP_077173.1	714	4	5.90	Pramel5	NP_001078887.1	70	0	0.00
Rnf167	NP_081721.1	233	1	4.52	Pramel30	NP_001079010.1	15	1	70.26
Pfn1	XP_030101518.1	891	2	2.37	Pramel31	XP_017175786.1	99	1	10.65
Eno3	XP_006532224.1	1551	4	2.72	Pramel15	NP_001108549.1	203	4	20.77
Spag7	NP_001161141.1	87	0	0.00	Pramel13	NP_084224.1	284	1	3.71
Camta2	XP_006532986.1	269	0	0.00	1700012P22R	XP_006539217.1	158	0	0.00
Inca1	XP_036012091.1	70	1	15.06	Aadacl3	NP_001078972.1	83	0	0.00
Kif1c	XP_036012244.1	1063	4	3.97	Aadacl4fm1	NP_941064.2	125	0	0.00
Zfp3	NP_808233.1	256	0	0.00	Aadacl4fm2	NP_001079011.1	49	0	0.00
Scimp	NP_001038991.1	138	0	0.00	Aadacl4	NP_001074717.1	145	0	0.00
Rabep1	NP_062273.2	649	0	0.00	Aadacl4fm4	NP_001078973.1	47	0	0.00
Nup88	NP_765982.2	460	0	0.00	AAdacl4fm3	NP_001079005.1	46	0	0.00
Rpain	NP_081462.1	150	2	14.05	Aadacl4fm5	NP_001119788.1	46	0	0.00
C1qbp	NP_031599.2	962	2	2.19	Dhrs3	NP_035433.1	642	2	3.28
Dhx33	NP_848144.3	1109	4	3.80	Vps13d	XP_006538868.1	736	2	2.86
Derl2	NP_001278077.1	853	0	0.00	Tnfrsf1b	NP_035740.2	905	3	3.49
Mis12	NP_080269.1	517	0	0.00	Tnfrsf8	NP_033427.1	495	1	2.13
Nlrp1a	XP_017169843.1	308	3	10.27	Zfp990	NP_001311482.1	137	0	0.00
Nlrp1b	XP_017170187.1	194	2	10.86	Gm13212	NP_001311345.1	210	1	5.02
Wscd1	NP_001344889.1	375	0	0.00	Zfp980	NP_001096628.2	251	1	4.20
Aipl1	XP_006532062.1	436	1	2.42	Zfp987	NP_001033015.1	197	1	5.35
Pimreg	NP_653109.2	407	2	5.18	Zfp600	NP_001171016.1	282	1	3.74
Pitpnm3	NP_001075110.1	559	4	7.54	Zfp992	NP_001078991.1	170	0	0.00
4933427D14R	XP_017170302.1	215	4	19.61	Zfp981	NP_001230067.1	269	1	3.92
Txndc17	NP_080835.1	284	0	0.00	Rex2	XP_030108872.1	277	9	34.24
Med31	NP_080344.2	490	4	8.60	Zfp991	NP_001077387.1	207	0	0.00
Slc13a5	XP_017170016.1	439	2	4.80	Zfp988	NP_001371161.1	203	1	5.19
Xaf1	XP_017170102.1	399	3	7.92	Zfp978	NP_001365671.1	207	2	10.18
Fbxo39	XP_006533964.2	551	1	1.91	Zfp982	NP_001352351.1	226	0	0.00
Tekt1	XP_006532996.1	443	0	0.00	Zfp985	NP_001014419.2	200	1	5.27
Smtnl2	NP_808444.1	520	2	4.05	Zfp979	NP_659544.2	239	1	4.41
Ggt6	NP_082095.2	573	1	1.84	Zfp534	XP_036019448.1	274	1	3.85
Mybbp1a	NP_058056.2	1096	0	0.00	Zfp984	XP_036019420.1	152	0	0.00
Spns2	XP_006532998.1	390	0	0.00	Zfp933	NP_941021.1	127	0	0.00
Spns3	XP_006534549.1	282	0	0.00	Miip	NP_001361633.1	61	4	69.11
Ube2g1	NP_080261.2	2298	2	0.92	Fv1	NP_034374.2	138	1	7.64
Ankfy1	NP_033801.4	1372	2	1.54	Mfn2	NP_573464.2	1478	4	2.85
Cyb5d2	NP_001020097.2	451	0	0.00	Plod1	NP_035252.1	498	7	14.81
Zzef1	XP_006532664.1	560	2	3.76	2510039O18F	XP_006539342.1	97	6	65.19
Atp2a3	NP_058025.2	1228	2	1.72	Nppb	NP_032752.1	522	3	6.06
P2rx1	NP_032797.3	742	3	4.26	Nppa	NP_032751.1	817	7	9.03
Camkk1	NP_061371.2	866	1	1.22	Clcn6	NP_036059.1	362	6	17.47
Ncbp3	NP_080094.3	240	1	4.39	Mthfr	NP_001155270.1	1312	5	4.02
Itgae	NP_032425.2	739	1	1.43	Agtrap	NP_033772.2	233	5	22.62
Haspin	NP_034483.1	687	1	1.53	Draxin	XP_006539233.1	179	0	0.00
P2rx5	NP_001363911.1	218	2	9.67	Mad2l2	NP_082261.2	608	4	6.93
Emc6	NP_079594.1	399	1	2.64	Fbxo6	NP_001157176.1	578	4	7.29
Tax1bp3	NP_083840.1	297	1	3.55	Fbxo44	XP_030109360.1	496	4	8.50
Ctns	XP_011247627.1	255	2	8.27	Fbxo2	NP_789818.1	662	7	11.14
Shpk	NP_083307.1	1089	10	9.68	Disp3	NP_001076811.1	477	7	15.47
Trpv1	NP_001001445.1	942	11	12.31	Ubiad1	NP_082149.1	361	8	23.36
Trpv3	NP_659567.2	343	7	21.51	Mtor	NP_064393.2	3756	2	0.56

Aspa	XP_036012130.1	388	8	21.73	Angptl7	NP_001034643.1	467	7	15.80
Spata22	NP_001038996.1	191	8	44.14	Exosc10	NP_057908.2	1540	7	4.79
Olfr20	NP_667134.2	49	5	107.54	Srm	NP_033298.1	903	6	7.00
Olfr376	NP_001166157.1	114	5	46.22	Masp2	XP_011248504.1	650	8	12.97
Olfr1	XP_030101874.1	41	3	77.11	Tardbp	NP_001008546.1	1213	7	6.08
Olfr378	NP_667235.1	55	0	0.00	Gm572	NP_001355764.1	58	9	163.54
Olfr380	NP_667236.1	114	5	46.22	Casz1	XP_006539220.1	359	4	11.74
Olfr381	NP_667233.2	31	1	34.00	Pex14	NP_062755.1	346	2	6.09
Olfr382	XP_036012600.1	104	0	0.00	Dffa	NP_001020467.1	408	3	7.75
Olfr385	NP_667234.1	26	0	0.00	Cort	XP_030109015.1	574	1	1.84
Olfr384	NP_997107.1	74	0	0.00	Cenps	NP_081539.1	548	4	7.69
Zfp735	NP_001119961.1	193	0	0.00	Pgd	NP_001074743.1	929	2	2.27
Olfr389	NP_667220.2	79	0	0.00	Kif1b	XP_030109130.1	1529	11	7.58
Olfr390	NP_666459.1	23	0	0.00	Ube4b	NP_071305.2	888	14	16.62
Olfr392	NP_667217.2	58	0	0.00	Rbp7	XP_006539148.1	264	10	39.92
Olfr393	NP_667219.1	39	0	0.00	Nmnat1	NP_001343286.1	543	13	25.23
Olfr394	NP_667218.1	179	0	0.00	Lzic	XP_030109620.1	103	12	122.78
Olfr395	NP_667216.1	90	0	0.00	Ctnnbip1	NP_075954.1	246	14	59.98
Olfr23	XP_006532510.1	37	0	0.00	Clstn1	NP_075538.1	750	8	11.24
Olfr397	NP_666458.1	37	0	0.00	Pik3cd	NP_001157524.1	2054	2	1.03
Olfr398	NP_666921.1	19	0	0.00	Tmem201	XP_006538883.1	293	14	50.36
Olfr139	NP_667214.1	72	0	0.00	Slc25a33	XP_036020307.1	549	17	32.63
Olfr399	NP_667215.2	82	0	0.00	Spsb1	NP_083311.1	652	16	25.86
Zfp616	NP_001171041.1	332	0	0.00	H6pd	NP_775547.2	1920	18	9.88
Olfr401	NP_666917.1	49	0	0.00	Gpr157	NP_796340.2	170	18	111.59
Olfr402	NP_666919.1	32	0	0.00	Slc2a5	NP_062715.2	781	17	22.94
Olfr403	NP_997505.1	153	0	0.00	Slc2a7	XP_017175793.1	212	12	59.65
Olfr43	NP_666922.2	63	0	0.00	Car6	NP_033932.2	312	18	60.80
Olfr406	NP_001011863.1	22	0	0.00	Eno1	NP_001366056.1	1766	9	5.37
Olfr59	NP_001349594.1	38	0	0.00	Rere	XP_030109601.1	792	16	21.29
Olfr410	NP_666918.1	272	0	0.00	Slc45a1	NP_776135.2	587	17	30.52
Olfr411	NP_666920.2	104	0	0.00	Errfi1	NP_001343252.1	456	16	36.98
Olfr412	NP_001011851.1	41	0	0.00	Park7	NP_065594.2	2209	14	6.68
Rap1gap2	XP_006533685.2	495	2	4.26	Tnfrsf9	NP_001070977.1	635	10	16.60
Ccdc92b	NP_001013806.1	47	0	0.00	Uts2	NP_036040.1	380	14	38.83
Cluh	NP_001074627.1	570	7	12.94	Per3	XP_036019720.1	319	10	33.04
Pafah1b1	NP_038653.1	1268	8	6.65	Vamp3	NP_033524.1	1004	11	11.55
Mettl16	NP_080473.1	165	7	44.71	Camta1	XP_030108881.1	582	12	21.73
Mnt	NP_034943.3	726	8	11.61	Gm13090	NP_001295375.1	0	0	#DIV/0!
Sgsm2	XP_006534646.1	648	7	11.38	Dnajc11	NP_766292.2	489	3	6.47
Tsr1	NP_796299.2	1266	6	4.99	Thap3	NP_001139401.1	108	8	78.07
Srr	NP_001349672.1	560	7	13.17	Phf13	NP_766293.2	565	4	7.46
Smg6	NP_001002764.1	626	9	15.15	Klhl21	NP_001028524.1	591	4	7.13
Hic1	NP_034560.2	698	6	9.06	Zbtb48	XP_017175373.1	302	3	10.47
Ovca2	NP_081412.1	258	3	12.25	Tas1r1	NP_114073.1	477	2	4.42
Dph1	NP_652762.2	430	5	12.25	Nol9	XP_017175901.1	410	4	10.28
Rtn4rl1	NP_808376.1	777	4	5.43	Plekhg5	XP_006538986.1	504	0	0.00
Rpa1	NP_080929.1	1076	1	0.98	Tnfrsf25	NP_001277939.1	546	1	1.93
Smyd4	NP_001096081.1	783	4	5.38	Espn	XP_006539126.1	1365	3	2.32
Serpinf1	NP_035470.3	769	8	10.96	Hes2	XP_017175471.1	186	1	5.67
Serpinf2	NP_032904.1	929	4	4.54	Acot7	NP_579926.2	452	7	16.32
Wdr81	XP_006532611.1	415	8	20.32	Gpr153	NP_848493.1	287	5	18.36
Tlcd2	NP_081525.1	57	1	18.49	Hes3	XP_006538613.1	343	5	15.36
Prpf8	NP_619600.2	1694	4	2.49	Icmt	NP_598549.1	486	9	19.52
Rilp	NP_001025109.1	199	1	5.30	Rnf207	XP_011248597.1	208	7	35.47
Scarf1	NP_001004157.2	333	5	15.82	Rpl22	NP_001264042.1	1138	8	7.41
Slc43a2	XP_006532882.1	233	1	4.52	Chd5	XP_006538994.1	1987	4	2.12
Pitpna	NP_032876.1	483	5	10.91	Kcnab2	NP_001239583.1	844	9	11.24
Inpp5k	XP_006532570.1	488	2	4.32	Nphp4	XP_006538930.1	515	6	12.28
Myo1c	NP_001357540.1	1376	6	4.60	Ajap1	XP_006538887.1	674	14	21.89
Crk	NP_598417.2	1168	5	4.51	A430005L14R	NP_780496.2	54	3	58.55
Ywhae	NP_033562.3	3483	5	1.51	Dffb	NP_031885.3	281	8	30.00
Doc2b	NP_031899.2	640	3	4.94	Cep104	NP_808341.1	237	12	53.36
Rph3al	XP_030101963.1	205	1	5.14	Lrrc47	NP_957678.1	866	13	15.82
1700016K19R	NP_941039.1	38	0	0.00	Smim1	XP_017175868.1	55	0	0.00
Rflnb	NP_083934.1	158	0	0.00	Ccdc27	XP_030109499.1	37	12	341.81

Vps53	NP_080940.2	470	0	0.00	Trp73	XP_006538783.2	981	9	9.67
Tlcd3a	NP_082049.1	89	1	11.84	Wrap73	NP_067474.2	244	13	56.15
Gemin4	NP_796341.2	345	3	9.16	Tprgl	NP_080664.1	102	9	92.99
Dbil5	NP_067269.1	242	0	0.00	Megf6	NP_001156449.1	296	14	49.85
Glod4	NP_080305.2	714	2	2.95	Arhgef16	XP_011248543.1	642	8	13.13
Mrm3	NP_899086.2	573	2	3.68	Prdm16	NP_001277955.1	904	6	6.99
Nxn	NP_032776.1	364	1	2.90	Actrt2	NP_082789.1	1566	10	6.73
Timm22	NP_001278090.1	522	1	2.02	Ttc34	NP_766466.4	39	0	0.00
Abr	XP_030101329.1	425	2	4.96	Mmel1	NP_038811.2	256	6	24.70
Bhlha9	NP_796156.3	39	2	54.05	Prxl2b	NP_079858.2	227	1	4.64
Trarg1	NP_808377.1	235	3	13.45	Tnfrsf14	XP_006538895.1	445	1	2.37
Gosr1	NP_001343250.1	505	0	0.00	Hes5	XP_006538625.3	615	1	1.71
Cpd	NP_031780.2	271	2	7.78	Pank4	NP_001292733.1	377	8	22.36
Tmigd1	NP_001369175.1	186	1	5.67	Plch2	XP_011248572.1	958	7	7.70
Blmh	NP_848760.1	645	4	6.54	Pex10	NP_001035866.1	471	2	4.48
Slc6a4	NP_034614.2	777	4	5.43	Rer1	NP_080671.1	546	1	1.93
Nsrp1	NP_001012309.1	298	3	10.61	Morn1	XP_030109754.1	156	5	33.78
Efcab5	XP_017170081.1	102	4	41.33	Ski	NP_001344120.1	258	1	4.08
Ssh2	XP_006533290.1	797	4	5.29	Faap20	NP_001177374.1	110	0	0.00
Coro6	XP_017169951.2	816	2	2.58	Prkcz	NP_032886.2	1670	7	4.42
Ankrd13b	NP_001360812.1	135	0	0.00	Gabrd	XP_036019574.1	775	4	5.44
Git1	NP_001004144.1	881	2	2.39	Cfap74	NP_001355667.1	0	0	#DIV/0!
Trp53i13	XP_006533015.1	54	1	19.52	Tmem52	NP_081437.1	105	3	30.11
Abhd15	NP_080461.3	244	2	8.64	Gnb1	XP_017175466.1	2784	6	2.27
Taok1	NP_659074.2	920	2	2.29	Nadk	NP_001153109.1	490	0	0.00
Nufip2	XP_036012823.1	375	4	11.24	Slc35e2	NP_796160.1	251	0	0.00
Cryba1	NP_034095.1	239	2	8.82	Cdk11b	XP_030108992.1	697	1	1.51
Myo18a	XP_006533659.1	1176	3	2.69	Mmp23	NP_001307164.1	292	2	7.22
Gm10277	XP_030102324.1	14	0	0.00	Mib2	NP_660106.2	2925	2	0.72
Pipox	XP_011247109.1	841	2	2.51	Fndc10	XP_030109373.1	33	0	0.00
Sez6	XP_006532699.1	588	2	3.58	Ssu72	NP_081175.2	530	0	0.00
Phf12	NP_777277.2	724	1	1.46	Tmem240	XP_036020107.1	124	2	17.00
Dhrs13	NP_899109.2	251	3	12.60	Atad3a	NP_849534.2	913	2	2.31
Flot2	NP_001271156.1	627	6	10.09	Vwa1	NP_680085.3	910	4	4.63
Eral1	NP_071708.2	489	0	0.00	Tmem88b	NP_001028566.2	175	1	6.02
Fam222b	NP_001351039.1	49	4	86.03	Ankrd65	XP_017176057.1	976	4	4.32
Traf4	NP_033449.2	888	8	9.49	Mrpl20	XP_036020206.1	914	2	2.31
Nek8	XP_006532247.1	428	5	12.31	Ccnl2	XP_036020147.1	688	5	7.66
Tlcd1	NP_080984.1	80	2	26.35	Aurkaip1	NP_079614.1	637	2	3.31
Rpl23a	NP_997406.1	1167	1	0.90	Mxra8	NP_077225.4	495	7	14.90
Rab34	NP_001152954.1	694	1	1.52	Dvl1	XP_006538581.1	1257	4	3.35
Proca1	NP_001355805.1	172	6	36.76	Tas1r3	NP_114078.1	597	6	10.59
Supt6	NP_033323.2	1425	9	6.66	Cptp	NP_077792.2	139	6	45.49
Sdf2	NP_033169.3	418	8	20.17	Ints11	NP_082296.1	705	1	1.49
2610507B11R	NP_001002004.2	211	0	0.00	Pusl1	NP_001028662.1	432	2	4.88
Rskr	XP_006533028.1	920	1	1.15	Acap3	NP_997106.1	592	2	3.56
Spag5	NP_059103.1	597	5	8.83	Ube2j2	NP_001271241.1	628	6	10.07
Aldoc	NP_033787.2	1144	0	0.00	C1qtnf12	NP_080401.1	179	1	5.89
Pigs	NP_958808.1	123	0	0.00	B3galt6	NP_536693.1	208	4	20.27
Unc119	NP_001300914.1	767	1	1.37	Sdf4	NP_035471.1	280	5	18.82
Foxn1	XP_006532328.1	780	2	2.70	Tnfrsf4	XP_006538787.3	514	2	4.10
Slc13a2	NP_071856.1	500	2	4.22	Tnfrsf18	NP_033426.1	532	3	5.94
Slc46a1	NP_081016.2	423	1	2.49	Ttll10	NP_083540.1	316	2	6.67
Sarm1	NP_766383.2	444	3	7.12	9430015G10F	NP_663532.2	17	0	0.00
Vtn	NP_035837.1	1383	2	1.52	Agrn	NP_067617.3	1051	3	3.34
Sebox	NP_032785.1	490	2	4.30	Isg15	NP_056598.2	3402	1	0.36
Tmem199	NP_954669.2	117	1	9.01	Perm1	NP_766005.2	148	3	26.70
Poldip2	NP_080665.1	266	2	7.92	Plekhn1	NP_001355568.1	72	4	78.07
Tnfaip1	NP_033421.3	414	2	5.09	Klhl17	NP_938047.1	236	4	25.52
Ift20	NP_061342.1	405	1	2.60	Noc2l	XP_030109548.1	1098	3	4.43
Tmem97	NP_598467.1	204	1	5.17	Samd11	XP_030109375.1	212	4	33.14
Nlk	NP_032728.3	735	0	0.00	Dhrsx	NP_001028498.2	299	0	0.00
Ccnq	NP_932106.1	543	1	1.94	Vmn2r122	NP_033518.3	60	0	0.00
Lyrm9	NP_001070149.1	72	1	14.64	Cdk6	NP_034003.1	2532	1	0.83
Nos2	NP_035057.1	1491	2	1.41	Fam133b	NP_001035966.1	193	8	79.43
Lgals9	NP_001152773.1	703	1	1.50	1700109H08F	NP_084119.1	718	1	2.45

Ksr1	NP_038599.1	1349	2	1.56	Rbm48	NP_766579.3	32	7	354.68
Wsb1	NP_062627.3	1008	1	1.05	Pex1	NP_001280735.1	668	3	6.76
Nf1	XP_006532505.1	1961	12	6.45	Gatad1	NP_080309.2	233	9	54.28
Omg	NP_062282.2	1296	11	8.95	Tmbim7	XP_006503675.1	329	0	0.00
Evi2b	NP_001070964.1	210	9	45.17	Ankib1	NP_001003909.2	339	9	32.92
Evi2a	NP_034291.1	388	11	29.88	Krit1	XP_030110771.1	591	8	15.85
Rab11fip4	NP_780752.1	548	12	23.08	Lrrd1	NP_766467.2	405	0	0.00
Utp6	NP_659075.1	909	12	13.91	Mterf1a	XP_017176488.1	331	7	22.29
Suz12	NP_954666.1	1199	13	11.43	Akap9	XP_006503583.1	861	8	9.79
Crlf3	NP_061246.1	192	14	76.85	Cyp51	NP_064394.2	699	6	9.05
Atad5	NP_001025027.1	763	13	17.96	Mterf1b	NP_001036135.2	330	7	22.36
Tefm	NP_899098.2	193	10	54.61	Fzd1	NP_067432.2	704	1	1.50
Adap2	NP_742145.1	829	16	20.34	Cdk14	NP_035204.2	2114	1	0.50
Rnf135	NP_082295.1	367	11	31.59	Pttg1ip2	XP_006503657.1	0	0	#DIV/0!
Rhot1	XP_006533949.1	1619	4	2.60	Cldn12	XP_030110610.1	220	2	9.58
Rhbdl3	XP_006533389.1	1882	3	1.68	Gtpbp10	NP_001343961.1	710	3	4.45
5730455P16R	NP_081748.1	103	1	10.23	Gm8773	NP_001371160.1	30	0	0.00
Zfp207	NP_001123641.1	650	10	16.21	Cfap69	NP_766035.1	68	1	15.50
Psmd11	NP_848731.2	1083	7	6.81	Steap2	NP_001272400.1	220	2	9.58
Cdk5r1	NP_034001.1	980	6	6.45	Steap1	NP_081675.2	270	4	15.61
Myo1d	NP_796364.2	1201	10	8.78	Zfp804b	NP_001156695.1	130	0	0.00
Tmem98	NP_083813.1	103	3	30.70	Tex47	NP_081879.1	37	0	0.00
Spaca3	NP_083643.1	374	2	5.64	Steap4	NP_473439.2	665	1	1.58
Asic2	NP_001029185.1	1023	2	2.06	Sri	NP_079894.2	398	4	10.59
1700071K01R	NP_001028937.1	1408	2	1.50	Adam22	XP_006503605.1	710	6	8.91
Ccl2	NP_035463.1	1835	4	2.30	Dbf4	NP_001177646.1	806	8	10.46
Ccl7	NP_038682.1	752	5	7.01	Slc25a40	XP_006503655.3	194	10	54.32
Ccl11	NP_035460.1	818	5	6.44	Rundc3b	NP_941022.1	276	10	38.18
Ccl12	NP_035461.2	505	4	8.35	Abcb1a	NP_035206.2	1453	12	8.70
Ccl8	NP_067418.1	290	5	18.17	Abcb1b	NP_035205.1	924	5	5.70
Ccl1	NP_035459.1	544	5	9.69	Abcb4	NP_032856.2	883	9	10.74
Tmem132e	NP_001291368.1	189	1	5.58	Crot	NP_076222.1	462	9	20.53
Cct6b	XP_006532171.1	1425	5	3.70	Tmem243	NP_001074498.1	40	8	210.78
Zfp830	NP_080160.2	306	1	3.44	Dmtf1	XP_030110323.1	2188	11	5.30
Lig3	XP_006532410.1	895	4	4.71	Elapor2	NP_766294.2	409	11	28.34
Rffl	XP_006534053.2	229	4	18.41	Grm3	XP_036020611.1	1120	6	5.65
Rad51d	NP_001264868.1	1642	4	2.57	Gm5861	NP_001296950.1	4	1	263.48
Fndc8	NP_084500.1	1	0	0.00	Speer1	NP_001296449.1	4	1	263.48
Nle1	NP_663406.2	924	3	3.42	Gm6460	NP_001033008.1	2	0	0.00
Unc45b	NP_848795.3	881	1	1.20	4933402N22F	NP_001170981.1	2	0	0.00
Slfn5	XP_017170104.1	227	5	23.21	Sema3d	XP_030109906.1	316	3	10.01
Slfn9	NP_766384.2	96	5	54.89	Sema3a	XP_006503620.2	725	3	4.36
Slfn8	XP_036012637.1	166	6	38.09	Speer3	NP_081926.2	0	0	#DIV/0!
Slfn2	NP_035538.1	389	5	13.55	Sema3e	NP_035478.2	343	3	9.22
Slfn1	NP_035537.1	232	4	18.17	Pclo	XP_006503645.2	961	6	6.58
Slfn4	XP_006532722.1	198	5	26.61	Gm9758	NP_941068.1	35	0	0.00
Slfn3	NP_035539.1	114	7	64.71	Speer4e	NP_001116133.1	65	0	0.00
Slfn14	NP_001159500.1	85	2	24.80	Gm10354	XP_006503580.1	0	0	#DIV/0!
Pex12	NP_001351691.1	368	2	5.73	Gm17019	XP_006503662.1	41	0	0.00
Ap2b1	XP_017170261.1	1099	3	2.88	Speer4d	NP_080035.2	75	0	0.00
Rasl10b	NP_001348497.1	1930	2	1.09	Speer4c	XP_006535672.1	42	0	0.00
Gas2l2	NP_001013781.1	532	1	1.98	Cacna2d1	XP_011248046.1	695	6	9.10
1700020L24Ri	NP_079768.2	1	1	1053.90	Hgf	NP_034557.3	1433	2	1.47
Mmp28	NP_766385.1	287	2	7.34	Speer4f1	NP_081885.1	0	0	#DIV/0!
Taf15	NP_081703.1	740	1	1.42	Sema3c	NP_038685.3	451	2	4.67
Heatr9	XP_011247471.1	59	0	0.00	Cd36	XP_030109948.1	755	1	1.40
Ccl5	NP_038681.2	1627	5	3.24	Gnat3	NP_001074612.1	1123	4	3.75
Ccl9	NP_035468.1	761	4	5.54	Gnai1	NP_034435.1	1470	3	2.15
Ccl6	NP_033165.1	772	4	5.46	Magi2	XP_006535806.1	1114	1	0.95
Ccl3	NP_035467.1	976	5	5.40	Phtf2	XP_006535864.1	241	2	8.75
Ccl4	NP_038680.1	1237	5	4.26	Tmem60	NP_808269.1	132	0	0.00
Wfdc17	NP_001075426.1	235	5	22.42	Rsbn1l	NP_001074446.1	116	2	18.17
Wfdc18	NP_031995.3	222	0	0.00	Ptpn12	NP_035333.2	950	3	3.33
Wfdc21	NP_899072.1	51	0	0.00	Gsap	XP_036020867.1	93	0	0.00
Heatr6	NP_663407.2	618	0	0.00	Ccdc146	NP_083471.1	117	0	0.00
Hnf1b	NP_033356.2	750	7	9.84	Fgl2	NP_032039.2	797	2	2.64

Ddx52	NP_084372.2	1000	11	11.59	Fam185a	XP_006535794.1	46	1	22.91
Synrg	XP_006533042.1	912	12	13.87	Fbxl13	NP_796050.2	823	10	12.81
Dusp14	XP_036012759.1	618	12	20.46	Lrrc17	NP_083253.1	726	12	17.42
Tada2a	XP_006533052.1	1277	14	11.55	Armc10	NP_080310.1	126	10	83.64
Acaca	XP_006532016.1	1269	13	10.80	Napepld	NP_001346893.1	280	9	33.88
Gm11437	NP_001033021.2	382	13	35.87	Pmpcb	NP_082707.1	1136	10	9.28
Aatf	XP_006533852.1	819	14	18.02	Dnajc2	NP_033610.1	1220	10	8.64
Lhx1	NP_032524.1	929	14	15.88	Psmc2	NP_001355590.1	1613	11	7.19
Mrm1	NP_663408.2	569	14	25.93	Slc26a5	NP_001276716.1	512	9	18.53
Dhrs11	NP_808232.2	459	14	32.15	Reln	NP_001297393.1	1073	2	1.96
Ggnbp2	XP_017169961.1	273	13	50.19	Orc5	NP_036089.1	966	10	10.91
Pigw	NP_081664.2	260	12	48.64	Kmt2e	NP_081260.1	989	10	10.66
Myo19	NP_079690.2	969	13	14.14	Srpk2	XP_017176257.1	904	9	10.49
Znhit3	NP_001005223.1	270	10	39.03	Pus7	NP_001276710.1	543	1	1.94
Car4	NP_031633.1	572	0	0.00	Rint1	NP_796297.2	325	2	6.49
Usp32	NP_001025105.1	2023	5	2.60	Tomm7	NP_079670.1	597	0	0.00
1700125H20R	XP_011247583.1	1027	4	4.10	Fam126a	XP_017176677.1	124	1	8.50
Appbp2	NP_080101.1	941	7	7.84	Klhl7	NP_080724.2	614	1	1.72
Ppm1d	NP_058606.3	1348	5	3.91	Nupl2	NP_694732.3	316	1	3.34
Bcas3	XP_006532599.1	625	7	11.80	Kcnh2	XP_006535696.1	709	2	2.97
Tbx2	NP_033350.2	867	6	7.29	Nos3	NP_032739.3	1580	1	0.67
Tbx4	NP_035666.2	538	5	9.79	Atg9b	NP_001002897.2	402	0	0.00
Brip1	XP_036012556.1	764	2	2.76	Abcb8	NP_083296.2	429	3	7.37
Ints2	XP_006534241.1	361	4	11.68	Cdk5	XP_006535689.1	1264	2	1.67
Med13	NP_001074400.1	728	6	8.69	Asic3	XP_006535701.1	327	2	6.45
Rnft1	NP_084064.1	676	2	3.12	Slc4a2	NP_033233.2	385	4	10.95
Rps6kb1	XP_006534378.1	2075	8	4.06	Fastk	NP_075718.2	253	0	0.00
Tubd1	NP_001185974.1	1193	5	4.42	Tmub1	NP_071863.1	198	0	0.00
Vmp1	NP_001343460.1	690	6	9.16	Agap3	NP_631892.2	573	1	1.84
Ptrh2	NP_778169.1	402	4	10.49	Gbx1	NP_056554.1	279	0	0.00
Cltc	NP_001003908.1	2024	8	4.17	Asb10	NP_001297405.1	1410	2	1.49
Dhx40	NP_080467.3	1049	6	6.03	Iqca1l	NP_808344.3	664	2	3.17
Ypel2	NP_001005341.1	214	7	34.47	Abcf2	NP_001177372.1	1084	3	2.92
Gdpd1	NP_079914.1	341	2	6.18	Chpf2	NP_598674.2	205	1	5.14
Smg8	NP_077224.1	244	0	0.00	Smarcd3	XP_006535861.2	895	2	2.36
Prr11	NP_780772.1	354	5	14.89	Nub1	NP_001292193.1	374	0	0.00
Ska2	NP_079653.1	445	4	9.47	Wdr86	XP_006535783.1	323	0	0.00
Trim37	NP_001349954.1	847	4	4.98	Crygn	NP_694716.2	148	0	0.00
Ppm1e	XP_036012659.1	1438	9	6.60	Rheb	NP_444305.2	2044	3	1.55
Rad51c	NP_001278369.1	1476	3	2.14	Prkag2	XP_036020617.1	1392	3	2.27
Tex14	NP_001186222.1	218	4	19.34	Galntl5	NP_080725.2	248	2	8.50
Septin4	XP_006532556.1	126	0	0.00	Galnt11	NP_001346819.1	334	2	6.31
Mtmr4	XP_030101475.1	268	5	19.66	Kmt2c	XP_017176309.1	1473	4	2.86
Hsf5	NP_001038992.1	586	0	0.00	Cct8l1	NP_941023.1	890	2	2.37
Rnf43	NP_766036.2	601	5	8.77	Xrcc2	NP_065595.2	363	0	0.00
Supt4a	NP_033322.1	571	1	1.85	Actr3b	NP_001004365.1	1412	1	0.75
Tspoap1	XP_006532765.1	510	2	4.13	Gm21663	NP_001365566.1	1	1	1053.90
Mpo	NP_034954.2	1314	0	0.00	Gm21671	NP_001268445.1	0	0	#DIV/0!
Lpo	NP_536345.2	252	0	0.00	Gm21680	NP_001365573.1	0	0	#DIV/0!
Mks1	XP_036012711.1	321	0	0.00	Gm21698	NP_001365574.1	2	2	1053.90
Epx	NP_031972.2	370	0	0.00	Gm1979	XP_030109862.1	0	0	#DIV/0!
Olfr462	NP_666523.2	72	0	0.00	Gm5862	NP_001268454.1	35	0	0.00
Olfr463	NP_666525.2	58	0	0.00	Speer4a	NP_083652.2	25	0	0.00
Olfr464	NP_666524.2	52	0	0.00	Gm7347	NP_001365564.1	0	0	#DIV/0!
Dynll2	XP_030102117.1	889	2	2.37	Gm10471	NP_001171050.1	0	0	#DIV/0!
Srsf1	NP_775550.2	2062	4	2.04	5031410I06Ri	NP_997540.2	1	1	1053.90
Vezf1	XP_011247270.1	467	1	2.26	Gm10220	NP_001127771.1	0	0	#DIV/0!
Cuedc1	NP_001165570.1	69	6	91.64	Gm7361	XP_006535855.1	13	0	0.00
Mrps23	NP_077136.2	399	0	0.00	Dpp6	NP_034205.1	1247	6	5.07
Ccdc182	NP_083135.1	51	1	20.66	Speer4b	NP_082837.1	51	0	0.00
Msi2	XP_036012961.1	710	5	7.42	Paxip1	NP_061366.2	1111	3	2.85
Akap1	NP_001036006.1	484	4	8.71	Htr5a	XP_006535694.1	939	6	6.73
Scpep1	NP_083299.3	557	9	17.03	Insig1	NP_705746.1	786	4	5.36
Elobl	NP_001347709.1	365	0	0.00	En2	NP_034264.1	622	10	16.94
Coil	NP_057915.2	498	5	10.58	Cnpy1	NP_001297441.1	193	6	32.76
Trim25	NP_033572.2	876	3	3.61	Rbm33	NP_082510.1	142	12	89.06

Dgke	XP_036012756.1	442	6	14.31	Shh	NP_033196.1	2020	11	5.74
Gm525	XP_036012480.1	90	1	11.71	Rnf32	NP_067445.3	297	10	35.48
Nog	NP_032737.1	943	2	2.24	Lmbr1	XP_006535830.1	278	8	30.33
Ankfn1	NP_001314739.1	167	10	63.11	Nom1	NP_001028629.2	467	8	18.05
Pctp	NP_001303301.1	284	9	33.40	Mnx1	NP_064328.2	501	8	16.83
Tmem100	NP_080709.1	207	5	25.46	Ube3c	NP_598668.1	1142	8	7.38
Mmd	XP_011247489.1	519	5	10.15	Dnajb6	NP_001033029.1	780	2	2.70
Hlf	NP_766151.1	373	5	14.13	Il6	NP_112445.1	3461	4	1.22
Stxbp4	NP_035635.1	391	9	24.26	Tyms	NP_067263.1	1766	2	1.19
Cox11	NP_950173.1	455	8	18.53	3110082J24R	NP_001243192.1	0	0	#DIV/0!
Tom1l1	NP_001344473.1	500	9	18.97	Garem2	NP_001161351.1	56	0	0.00
Kif2b	NP_082823.1	1298	1	0.81	Hadha	NP_849209.1	1104	2	1.91
Car10	NP_001348636.1	852	5	6.18	Hadhb	XP_017176318.1	928	4	4.54
Utp18	NP_001013393.1	954	0	0.00	Adgrf3	NP_001014416.2	300	0	0.00
Mbtd1	XP_006531947.1	726	4	5.81	Selenoi	NP_081928.2	366	2	5.76
Nme2	NP_001070997.1	1335	2	1.58	Drc1	NP_001028632.1	138	0	0.00
Nme1	NP_001365783.1	1330	2	1.58	Otof	NP_001300696.1	866	0	0.00
Spag9	XP_006534273.1	615	4	6.85	Fam166c	NP_083561.1	67	0	0.00
Tob1	XP_030101781.1	389	1	2.71	Cib4	NP_082759.1	1042	1	1.01
Wfikkn2	XP_006533560.1	227	0	0.00	Kcnk3	NP_034738.1	469	0	0.00
Gm21885	NP_001365187.1	393	1	2.68	Slc35f6	NP_783606.2	211	0	0.00
Luc7l3	NP_001348502.1	890	4	4.74	Cenpa	NP_001289061.1	1766	1	0.60
Ankrd40	NP_082075.2	495	4	8.52	Dpysl5	NP_001343877.1	533	2	3.95
Abcc3	NP_001350118.1	1119	3	2.83	Mapre3	XP_030109882.1	1086	2	1.94
Cacna1g	NP_001171361.1	841	2	2.51	Tmem214	NP_653108.2	148	1	7.12
Spata20	NP_659076.2	156	1	6.76	Agbl5	XP_036020907.1	167	2	12.62
Epn3	NP_001348487.1	580	3	5.45	Ost4	NP_077780.3	223	0	0.00
Mycbpap	XP_006532001.1	278	2	7.58	Emilin1	NP_598679.1	198	1	5.32
Rsad1	NP_001013399.1	259	2	8.14	Khk	NP_001297453.1	433	7	17.04
Acsf2	NP_722502.1	688	1	1.53	Cgref1	NP_081046.1	204	2	10.33
Chad	NP_031715.1	771	2	2.73	Preb	XP_006504078.1	612	2	3.44
Lrrc59	NP_598568.1	855	1	1.23	Tcf23	XP_006504158.1	200	4	21.08
Eme1	NP_808420.1	586	0	0.00	Slc5a6	NP_001346951.1	288	3	10.98
Mrpl27	NP_444391.1	873	0	0.00	Atraid	NP_082131.2	165	5	31.94
Xylt2	NP_665827.2	222	0	0.00	Cad	XP_006504154.1	1127	6	5.61
Gm11541	NP_001007585.1	0	0	#DIV/0!	Slc30a3	NP_035903.2	557	4	7.57
Tmem92	NP_001030068.1	112	0	0.00	Dnajc5g	NP_001334253.1	423	0	0.00
Col1a1	NP_031768.2	1418	4	2.97	Trim54	NP_067422.1	734	2	2.87
Sgca	NP_033187.1	450	2	4.68	Ucn	NP_001332939.1	398	3	7.94
H1f9	NP_061262.1	321	3	9.85	Mpv17	NP_001281251.1	308	2	6.84
Ppp1r9b	NP_758465.2	506	1	2.08	Gtf3c2	NP_082177.2	238	12	53.14
Samd14	NP_001351064.1	158	1	6.67	Eif2b4	NP_034252.2	550	12	22.99
Pdk2	NP_598428.2	1060	1	0.99	Snx17	NP_710147.1	440	14	33.53
Itga3	XP_006532373.1	798	4	5.28	Zfp513	XP_006503720.3	271	12	46.67
Dlx3	NP_034185.1	597	3	5.30	Ppm1g	NP_032040.1	1372	14	10.75
Dlx4	NP_031893.3	331	2	6.37	Nrbp1	NP_001334255.1	255	13	53.73
Tac4	NP_444323.1	128	0	0.00	Krtcap3	NP_001350266.1	242	13	56.61
Kat7	NP_808287.1	969	1	1.09	Ift172	NP_080574.5	470	15	33.64
Fam117a	NP_766131.2	87	7	84.80	Fndc4	NP_001365819.1	152	13	90.14
Slc35b1	NP_058032.3	593	0	0.00	Gckr	NP_001361670.1	715	13	19.16
Spop	NP_079563.2	561	0	0.00	Zfp512	NP_766581.2	410	14	35.99
Nxph3	NP_570928.1	228	2	9.24	4930548H24F	NP_080572.1	30	0	0.00
Ngfr	NP_150086.2	1031	2	2.04	Gpn1	NP_598517.1	586	11	19.78
Phb	NP_032857.1	1408	2	1.50	Supt7l	NP_082426.1	165	11	70.26
Zfp652	NP_963903.2	405	0	0.00	Slc4a1ap	NP_001346085.1	221	10	47.69
Phospho1	NP_694744.1	200	9	47.43	Mrpl33	NP_080072.2	570	5	9.24
Abi3	XP_030102088.1	593	8	14.22	Rbks	XP_006504177.1	837	5	6.30
Gngt2	NP_001033753.2	942	9	10.07	Babam2	NP_653124.1	243	0	0.00
B4galnt2	XP_006532264.1	346	8	24.37	Fosl2	NP_032063.2	850	1	1.24
Igf2bp1	NP_034081.1	599	1	1.76	Plb1	NP_001074876.1	377	2	5.59
Gip	NP_032145.2	373	2	5.65	Ppp1cb	NP_766295.2	2921	5	1.80
Snf8	NP_291046.1	507	8	16.63	Yes1	NP_001192061.1	1934	3	1.63
Ube2z	NP_758504.3	1603	8	5.26	Pisd	NP_796272.2	428	2	4.92
Atp5g1	XP_030101350.1	816	8	10.33	Prr14l	XP_006503906.1	95	3	33.28
Calcoco2	XP_006534523.1	586	8	14.39	Depdc5	XP_006504041.1	414	0	0.00
Ttll6	NP_766387.2	199	3	15.89	Ywhah	NP_035868.1	2956	4	1.43

Hoxb13	NP_032293.1	381	9	24.90	Slc5a1	NP_062784.3	669	1	1.58
Hoxb9	NP_032296.2	437	11	26.53	Spon2	NP_598664.3	517	2	4.08
Hoxb8	XP_017169784.1	449	11	25.82	Ctbp1	NP_001185788.1	1185	8	7.11
Hoxb7	XP_030101452.1	636	11	18.23	Maea	NP_067475.2	661	2	3.19
Hoxb6	XP_006532355.1	502	14	29.39	Uvssa	NP_001074570.1	226	1	4.66
Hoxb5	NP_032294.2	458	12	27.61	Nkx1-1	NP_035450.1	207	1	5.09
Hoxb4	NP_034589.3	480	10	21.96	Fam53a	NP_001346391.1	94	6	67.27
Hoxb3	NP_001073338.1	460	9	20.62	Slbp	NP_001276654.1	1071	9	8.86
Hoxb2	NP_598793.2	460	10	22.91	Tmem129	NP_080974.2	154	9	61.59
Hoxb1	XP_006532343.1	520	9	18.24	Tacc3	XP_011239017.1	818	10	12.88
Skap1	XP_006534571.1	330	6	19.16	Fgfr3	NP_001156689.1	1358	7	5.43
Snx11	NP_083241.1	340	6	18.60	Letm1	NP_062668.1	455	8	18.53
Cbx1	NP_001349493.1	1182	7	6.24	Nsd2	NP_001074571.2	1451	10	7.26
Nfe2l1	XP_036012296.1	613	11	18.91	Nelfa	NP_036044.1	381	6	16.60
Copz2	NP_063930.1	549	2	3.84	Gm1673	NP_001028630.2	27	0	0.00
Cdk5rap3	NP_084524.1	291	6	21.73	Nat8l	NP_001001985.3	706	1	1.49
Prr15l	XP_030101723.1	168	5	31.37	Poln	NP_001276732.1	528	1	2.00
Pnpo	NP_598782.1	488	6	12.96	Haus3	NP_666271.1	533	5	9.89
Sp2	NP_001074433.1	315	1	3.35	Mxd4	NP_034883.2	359	2	5.87
Sp6	NP_001350159.1	551	1	1.91	Zfyve28	XP_006503949.1	235	1	4.48
Scrn2	XP_017169969.1	242	0	0.00	Gm5553	XP_036021569.1	116	0	0.00
Lrrc46	NP_081302.2	631	0	0.00	Rnf4	NP_035408.1	1539	0	0.00
Mrpl10	NP_080430.1	275	1	3.83	Fam193a	NP_001230052.1	38	6	166.41
Osbpl7	XP_011247566.1	369	5	14.28	Tnip2	NP_620703.1	252	0	0.00
Tbx21	NP_062380.2	967	4	4.36	Sh3bp2	NP_001139330.1	448	2	4.70
Tbkbp1	NP_932768.2	394	5	13.37	Add1	NP_001318011.1	560	5	9.41
Kpnb1	NP_032405.3	1767	0	0.00	Mfsd10	XP_017176573.1	242	2	8.71
Npepps	NP_032968.2	759	2	2.78	Nop14	NP_083554.2	835	3	3.79
Mrpl45	XP_017170202.1	643	9	14.75	Grk4	NP_062370.2	738	5	7.14
Gpr179	NP_001074689.1	146	3	21.66	Htt	NP_034544.1	1496	1	0.70
Socs7	NP_619598.2	317	8	26.60	Msantd1	XP_011239055.1	157	1	6.71
Arhgap23	XP_006533930.1	297	10	35.48	Rgs12	XP_006504183.1	521	5	10.11
4933428G20R	XP_030101293.1	21	5	250.93	Hgfac	XP_006504094.1	1293	3	2.45
Srcin1	XP_030102032.1	671	10	15.71	Dok7	NP_001335407.1	252	0	0.00
Epop	NP_780541.2	210	0	0.00	Lrpap1	NP_038615.2	540	5	9.76
Mllt6	XP_006533395.1	631	12	20.04	Adra2c	NP_031444.2	901	1	1.17
Cisd3	NP_001351082.1	70	10	150.56	Hmx1	NP_001350073.1	453	3	6.98
Pcgf2	XP_036012525.1	671	10	15.71	Cpz	NP_694747.2	322	5	16.36
Psmb3	NP_036101.1	1327	11	8.74	Trmt44	NP_084484.2	97	0	0.00
Pip4k2b	NP_473392.1	725	13	18.90	Acox3	XP_006504262.1	554	2	3.80
Cwc25	NP_080462.2	660	13	20.76	Htra3	NP_084403.2	450	1	2.34
1700001P01R	NP_082432.2	34	0	0.00	Sh3tc1	XP_036020913.1	111	4	37.98
Rpl23	NP_075029.1	1261	2	1.67	Ablim2	XP_006503969.1	475	6	13.31
Lasp1	XP_006532404.1	695	11	16.68	Afap1	XP_006504161.1	398	4	10.59
Fbxo47	XP_006534395.1	184	2	11.46	Sorcs2	NP_112151.2	571	7	12.92
Plxdc1	NP_082475.3	213	10	49.48	Psapl1	NP_780458.2	192	4	21.96
Arl5c	XP_011247243.1	1331	2	1.58	Grpel1	NP_077798.1	1217	1	0.87
Cacnb1	NP_660099.2	525	9	18.07	Tada2b	NP_001163925.1	1236	0	0.00
Rpl19	XP_036012353.1	1265	3	2.50	Ccdc96	NP_080001.1	62	0	0.00
Stac2	NP_666140.1	106	0	0.00	Tbc1d14	NP_001106833.1	508	1	2.07
Fbxl20	XP_006534361.1	579	8	14.56	D5Ertd579e	XP_036021103.1	124	0	0.00
Med1	NP_001348879.1	582	3	5.43	Bloc1s4	NP_598485.1	75	0	0.00
Cdk12	XP_036012849.1	1068	14	13.82	Mrfap1	NP_080518.1	155	1	6.80
Neurod2	NP_035025.3	570	9	16.64	Man2b2	NP_032576.2	321	3	9.85
Ppp1r1b	NP_659077.1	730	11	15.88	Ppp2r2c	XP_006504034.1	1756	6	3.60
Stard3	XP_006533957.1	316	16	53.36	Wfs1	NP_035846.1	717	1	1.47
Tcap	NP_035670.2	560	12	22.58	Jakmip1	XP_011239092.1	228	0	0.00
Pnmt	NP_032916.1	257	6	24.60	Gm1043	XP_011239113.1	0	0	#DIV/0!
Pgap3	XP_006533612.1	256	16	65.87	Crmp1	NP_001129530.1	884	7	8.35
Erbb2	NP_001003817.1	3152	11	3.68	Evc	XP_011239063.1	252	6	25.09
Mien1	NP_079835.1	323	17	55.47	Evc2	NP_666032.1	268	7	27.53
Grb7	XP_030101435.1	662	17	27.06	Stk32b	NP_071861.1	1076	6	5.88
Ikzf3	XP_006533267.1	764	16	22.07	Cytl1	NP_001074575.1	279	0	0.00
Zpbp2	NP_081337.2	216	9	43.91	Msx1	NP_034965.2	931	6	6.79
Ormdl3	NP_079937.1	625	12	20.23	Stx18	NP_001276464.1	483	11	24.00
Gsdma3	NP_001007462.1	124	3	25.50	Nsg1	NP_001346019.1	650	10	16.21

Gsdma2	XP_036012972.1	88	7	83.83	Zbtb49	XP_006504226.1	319	7	23.13
Gsdma	XP_006533913.1	221	7	33.38	Lyar	NP_079557.2	1051	3	3.01
Psmd3	NP_033465.1	1092	13	12.55	Tmem128	NP_001343889.1	92	7	80.19
Csf3	XP_006532191.3	1052	4	4.01	Otop1	NP_766297.2	200	6	31.62
Med24	NP_035999.2	232	5	22.71	Drd5	NP_038531.1	824	6	7.67
Thra	NP_001300912.1	909	7	8.12	Slc2a9	NP_663534.1	679	6	9.31
Nr1d1	NP_663409.2	558	3	5.67	Wdr1	NP_035845.1	742	8	11.36
Msl1	NP_082998.2	183	1	5.76	Zfp518b	XP_030109865.1	278	3	11.37
Casc3	NP_619601.2	696	0	0.00	Clnk	XP_011239036.1	283	3	11.17
Rapgefl1	NP_001074394.1	294	1	3.58	Hs3st1	XP_017176175.1	233	1	4.52
Wipf2	NP_922922.1	452	2	4.66	Rab28	NP_001297519.1	751	1	1.40
Cdc6	NP_035929.1	2584	4	1.63	Nkx3-2	NP_031550.2	475	1	2.22
Rara	NP_033050.2	1059	7	6.97	Bod1l	XP_017176554.1	237	0	0.00
Gjd3	NP_848711.2	196	0	0.00	Cpeb2	NP_001355734.1	313	1	3.37
Top2a	XP_006533217.1	2457	6	2.57	C1qtnf7	NP_001128644.1	105	0	0.00
Igfbp4	XP_030101458.1	635	4	6.64	Cc2d2a	XP_036020946.1	410	3	7.71
Tns4	NP_766152.2	640	6	9.88	Fbxl5	NP_001153435.1	538	1	1.96
Ccr7	NP_031745.2	1191	0	0.00	Bst1	NP_033893.2	271	1	3.89
Smarce1	NP_065643.1	763	3	4.14	Cd38	NP_031672.2	815	2	2.59
Krt222	NP_766534.1	315	2	6.69	Fgfbp1	XP_030109989.1	347	4	12.15
Krt24	NP_083669.1	218	1	4.83	Prom1	NP_001157056.1	1180	2	1.79
Krt25	NP_598491.1	161	4	26.18	Tapt1	XP_006504003.1	312	8	27.02
Krt26	NP_001028569.2	68	2	31.00	Ldb2	XP_036020723.1	624	6	10.13
Krt27	NP_034796.1	207	2	10.18	Qdpr	NP_077198.1	726	8	11.61
Krt28	XP_036012881.1	106	0	0.00	Clrn2	NP_001156789.1	16	0	0.00
Krt10	XP_011247070.1	515	0	0.00	Lap3	NP_077754.3	919	8	9.17
Krt12	NP_034791.2	409	0	0.00	Med28	NP_080171.1	365	6	17.32
Krt20	NP_075745.1	352	0	0.00	Fam184b	NP_067391.3	157	9	60.41
Krt23	NP_203537.1	194	0	0.00	Ncapg	NP_062311.1	996	6	6.35
Krt39	NP_998895.2	202	1	5.22	Lcorl	NP_835278.2	389	12	32.51
Krt40	NP_001304142.1	189	1	5.58	Slit2	NP_001278156.1	1233	9	7.69
Krtap3-3	NP_079800.1	152	1	6.93	Pacrgl	NP_080031.1	197	7	37.45
Krtap3-2	NP_079996.2	153	2	13.78	Kcnip4	NP_001186172.1	1301	12	9.72
Krtap3-1	NP_076000.1	239	2	8.82	Adgra3	NP_598672.1	955	9	9.93
Krtap1-5	NP_081433.2	58	2	36.34	Ppargc1a	XP_036020787.1	1128	5	4.67
Krtap1-4	NP_001034591.1	143	3	22.11	Dhx15	NP_031865.2	1973	11	5.88
Krtap1-3	NP_001078995.1	144	3	21.96	Sod3	NP_035565.1	1499	5	3.52
Krtap9-3	NP_083627.2	185	3	17.09	Ccdc149	XP_036020579.1	50	2	42.16
Gm11938	NP_001120826.1	120	19	166.87	Lgi2	NP_659194.1	342	3	9.24
Gm11937	NP_001092816.1	139	4	30.33	Sepsecs	NP_766078.1	477	6	13.26
Krtap2-4	NP_082076.1	139	4	30.33	Pi4k2b	NP_083020.2	590	11	19.65
Gm11562	NP_001171008.2	140	5	37.64	Zcchc4	NP_001295073.1	109	5	48.34
Krtap4-1	NP_001041661.1	181	20	116.45	Anapc4	NP_077175.1	998	2	2.11
Krtap4-2	NP_081083.2	171	6	36.98	Slc34a2	NP_035532.2	398	4	10.59
Krtap4-7	XP_036012954.1	181	6	34.94	Sel1l3	NP_766298.2	587	6	10.77
Gm11555	XP_030102334.1	112	19	178.79	Smim20	NP_001138905.1	39	0	0.00
Gm11563	NP_001119792.1	99	7	74.52	Rbpj	NP_001074397.1	1204	2	1.75
Krtap4-6	NP_081110.1	152	7	48.53	Cckar	NP_033957.1	565	5	9.33
Gm11595	NP_001119794.1	141	16	119.59	Tbc1d19	XP_036021325.1	146	11	79.40
Krtap4-8	NP_001079016.1	171	7	43.14	Stim2	NP_001350277.1	399	3	7.92
Krtap4-9	NP_001079017.1	168	7	43.91	Pcdh7	XP_006504082.1	592	5	8.90
Gm11596	NP_001092781.1	104	15	152.00	G6pd2	NP_062341.2	927	3	3.41
Gm11569	NP_001092782.1	70	18	271.00	Arap2	XP_036020872.1	513	2	4.11
Gm11554	NP_001092783.1	83	9	114.28	Dthd1	NP_001164176.2	57	1	18.49
Krtap4-13	NP_081363.1	67	1	15.73	Nwd2	NP_795980.2	978	0	0.00
Gm11564	NP_001094084.1	72	14	204.93	0610040J01R	NP_083830.3	83	0	0.00
2300003K06R	NP_001182312.1	2	0	0.00	Rell1	NP_666035.2	140	0	0.00
Krtap4-16	NP_001013845.1	131	9	72.41	Pgm2	NP_079976.1	720	1	1.46
Gm11568	NP_001191959.1	102	16	165.32	Tbc1d1	XP_011239059.1	525	9	18.07
Gm11559	NP_001170955.1	4	0	0.00	Klf3	XP_006503814.1	533	10	19.77
Krtap9-1	NP_056556.1	184	8	45.82	Tlr1	NP_109607.1	1319	5	4.00
Gm11567	NP_001095083.1	98	16	172.07	Tlr6	XP_030110224.1	1167	5	4.52
Krtap31-1	NP_081844.1	96	15	164.67	Fam114a1	NP_080943.3	182	14	81.07
Gm11565	NP_001119795.1	99	15	159.68	Tmem156	XP_017176703.1	110	15	143.71
Krtap31-2	NP_001020415.1	92	13	148.92	Klhl5	NP_780383.2	609	13	22.50
Krtap9-5	NP_001078996.1	142	6	44.53	Wdr19	NP_001346808.1	344	15	45.95

Krtap29-1	NP_001365439.1	176	5	29.94	Rfc1	NP_001334287.1	719	13	19.06
Krtap16-1	NP_001242986.1	97	5	54.32	Klb	NP_112457.1	580	13	23.62
Krtap17-1	NP_001093244.1	147	0	0.00	Rpl9	NP_035422.1	1183	14	12.47
Krt33a	NP_082259.2	226	4	18.65	Lias	NP_077791.1	630	13	21.75
Krt33b	NP_038598.1	232	4	18.17	Ugdh	XP_006503925.1	843	12	15.00
Krt34	NP_081839.1	473	4	8.91	Smim14	NP_001363903.1	112	11	103.51
Krt31	NP_034789.2	253	3	12.50	Ube2k	NP_058066.2	2018	13	6.79
Krt32	NP_001152846.2	247	1	4.27	Pds5a	NP_001074790.1	958	11	12.10
Krt35	NP_058576.2	225	0	0.00	N4bp2	XP_006504066.1	241	1	4.37
Krt36	XP_017169791.1	348	0	0.00	Rhoh	NP_001074574.1	1696	3	1.86
Krt13	NP_034792.1	316	0	0.00	Chrna9	XP_006504008.1	578	2	3.65
Krt15	NP_032495.2	530	1	1.99	Rbm47	XP_006504025.1	520	1	2.03
Krt19	NP_032497.1	1073	3	2.95	Nsun7	NP_001289762.1	823	6	7.68
Krt9	NP_957707.2	578	2	3.65	Apbb2	NP_001297555.1	315	0	0.00
Krt14	NP_001300885.1	821	3	3.85	Uchl1	NP_035800.2	1478	3	2.14
Krt16	NP_032496.1	475	1	2.22	Limch1	XP_036021493.1	963	4	4.38
Krt17	NP_034793.1	553	2	3.81	Phox2b	NP_032914.1	518	4	8.14
Krt42	NP_997648.2	269	2	7.84	Tmem33	NP_084384.1	326	5	16.16
Eif1	NP_035638.1	875	5	6.02	Slc30a9	NP_848766.2	285	6	22.19
Gast	NP_034387.3	625	5	8.43	Bend4	XP_030110626.1	95	6	66.56
Hap1	NP_001345981.1	381	2	5.53	Shisa3	NP_001028587.1	126	0	0.00
Jup	XP_030101465.1	1632	9	5.81	Atp8a1	XP_036020643.1	1061	3	2.98
P3h4	NP_789800.1	244	5	21.60	Gm5108	NP_001243113.1	0	0	#DIV/0!
Fkbp10	NP_034351.2	1349	4	3.12	Grxcr1	NP_001018019.2	234	3	13.51
Nt5c3b	XP_036012819.1	258	1	4.08	Kctd8	NP_780728.3	451	4	9.35
Klhl10	NP_080003.1	472	3	6.70	Yipf7	NP_076273.1	381	1	2.77
Klhl11	NP_766153.1	448	4	9.41	Guf1	NP_766299.1	1000	0	0.00
Acly	NP_598798.1	1436	3	2.20	Gnpda2	NP_001334292.1	561	5	9.39
Ttc25	NP_083194.2	840	3	3.76	Gabrg1	NP_034382.2	874	11	13.26
Cnp	NP_034053.2	449	1	2.35	Gabra2	XP_006503796.1	815	9	11.64
Dnajc7	XP_030102048.1	646	7	11.42	Cox7b2	NP_084328.1	170	11	68.19
Nkiras2	XP_030102201.1	222	1	4.75	Gabra4	NP_001345973.1	1079	11	10.74
Zfp385c	XP_030101919.1	59	1	17.86	Gabrb1	NP_032095.1	1052	14	14.03
Dhx58	NP_084426.2	876	8	9.62	Commd8	NP_001343541.1	399	14	36.98
Kat2a	XP_017169760.2	2595	10	4.06	Corin	NP_001116228.1	675	2	3.12
Hspb9	NP_083583.2	313	5	16.84	Nfxl1	NP_598682.2	291	12	43.46
Rab5c	XP_036012346.1	1434	2	1.47	Zar1	XP_006504053.1	318	7	23.20
Kcnh4	NP_001074663.1	412	6	15.35	Cnga1	XP_006503772.1	508	9	18.67
Hcrt	NP_034540.1	647	9	14.66	Nipal1	NP_001074674.1	306	13	44.77
Ghdc	NP_114077.1	224	9	42.34	Txk	NP_001276423.1	1176	12	10.75
Stat5b	NP_001349611.1	1063	9	8.92	Tec	NP_001106932.1	1031	2	2.04
Stat5a	NP_001157534.1	1655	7	4.46	Slain2	NP_705795.2	138	10	76.37
Stat3	NP_998825.1	3082	7	2.39	Slc10a4	NP_775579.2	283	9	33.52
Cavin1	NP_033012.1	621	7	11.88	Fryl	XP_006504215.1	499	8	16.90
Atp6v0a1	XP_006532112.1	845	1	1.25	Ociad1	NP_075918.1	147	1	7.17
Naglu	NP_038820.2	448	1	2.35	Ociad2	NP_081226.1	178	1	5.92
Hsd17b1	NP_034605.1	515	3	6.14	Cwh43	NP_851840.1	223	2	9.45
Coasy	NP_001292911.1	1110	2	1.90	Dcun1d4	NP_001177663.1	451	5	11.68
Mlx	NP_035680.3	448	2	4.70	Lrrc66	NP_705796.1	451	3	7.01
Psmc3ip	NP_032975.1	524	1	2.01	Sgcb	NP_036020.1	291	4	14.49
Retreg3	NP_080777.1	183	0	0.00	Spata18	XP_006504218.1	211	5	24.97
Tubg1	NP_598785.1	1561	3	2.03	Usp46	NP_808229.1	564	2	3.74
Tubg2	NP_598789.1	1303	2	1.62	Rasl11b	NP_081154.1	1566	3	2.02
Plekhh3	NP_001333698.1	933	0	0.00	Scfd2	NP_001273366.1	180	8	46.84
Ccr10	NP_031747.2	441	0	0.00	Fip1l1	XP_036021322.1	549	7	13.44
Cntnap1	NP_058062.2	965	0	0.00	Lnx1	XP_036020727.1	1660	13	8.25
Ezh1	NP_031996.1	1618	2	1.30	Chic2	NP_083126.1	205	13	66.83
Ramp2	NP_062317.1	404	5	13.04	Gsx2	NP_573555.1	432	11	26.84
Vps25	NP_001271340.1	464	4	9.09	Pdgfra	NP_001076785.1	1413	9	6.71
Wnk4	XP_006534199.1	331	6	19.10	Kit	NP_001116205.1	2183	7	3.38
Coa3	NP_080894.1	470	4	8.97	Kdr	NP_034742.2	2007	7	3.68
Cntd1	NP_080838.1	132	1	7.98	Srd5a3	NP_065636.2	268	11	43.26
Becn1	NP_062530.2	1570	2	1.34	Tmem165	NP_035756.2	320	13	42.81
Psme3	NP_035322.1	854	7	8.64	Clock	XP_017176140.1	753	11	15.40
Aoc2	NP_849263.1	211	4	19.98	Pdcl2	NP_075997.1	419	12	30.18
Aoc3	NP_033805.1	373	5	14.13	Nmu	NP_062388.1	429	13	31.94

G6pc	NP_032087.2	1162	0	0.00	Exoc1	XP_036021371.1	547	13	25.05
Aarsd1	NP_659078.1	252	0	0.00	Cep135	NP_950197.1	447	10	23.58
Ptges3l	NP_001348052.1	697	3	4.54	Cracd	XP_006535065.1	588	1	1.79
Rundc1	XP_006533098.1	108	0	0.00	Aasdh	XP_030110250.1	1415	13	9.68
Rpl27	NP_035419.1	1160	2	1.82	Ppat	NP_742158.1	874	4	4.82
Ifi35	NP_081596.1	479	5	11.00	Paics	NP_001343900.1	1302	14	11.33
Vat1	NP_036167.1	473	0	0.00	Srp72	NP_079967.1	662	12	19.10
Rnd2	NP_033838.1	1611	2	1.31	Arl9	XP_006535113.4	1343	13	10.20
Brca1	XP_006532131.1	2702	6	2.34	Thegl	NP_082246.1	2	0	0.00
Nbr1	XP_006532493.1	1393	5	3.78	Hopx	XP_036021457.1	741	2	2.84
Tmem106a	NP_001346254.1	212	0	0.00	Spink2	XP_011247866.1	239	10	44.10
Rdm1	XP_006534008.1	133	1	7.92	Rest	NP_035393.2	783	2	2.69
Arl4d	NP_079680.1	1446	0	0.00	Noa1	XP_006535216.1	918	9	10.33
Dhx8	NP_659080.2	1505	1	0.70	Polr2b	NP_722493.2	2663	11	4.35
Etv4	XP_006532524.1	783	2	2.69	Igfbp7	NP_032074.3	844	6	7.49
Meox1	XP_036012267.1	422	2	4.99	Adgrl3	NP_001346759.1	884	4	4.77
Sost	NP_077769.4	437	1	2.41	Tecrl	NP_722496.2	466	0	0.00
Dusp3	XP_006534368.1	765	1	1.38	Epha5	XP_006534838.1	1731	1	0.61
Cfap97d1	NP_083563.1	66	1	15.97	Cenpc1	NP_031709.2	532	2	3.96
Mpp3	NP_031889.2	820	4	5.14	Stap1	NP_064376.1	265	0	0.00
Cd300lg	NP_001154183.1	146	0	0.00	Uba6	NP_766300.1	1382	2	1.53
Mpp2	XP_006533747.1	1226	1	0.86	Gnrhr	NP_034453.1	684	1	1.54
Ppy	NP_032944.1	602	1	1.75	Tmprss11c	NP_001025468.1	192	1	5.49
Pyy	NP_663410.1	592	1	1.78	Tmprss11d	NP_663536.1	290	4	14.54
Nags	NP_665828.1	1054	0	0.00	Tmprss11a	XP_006534903.1	226	3	13.99
Tmem101	NP_083925.2	126	2	16.73	Tmprss11g	NP_796136.2	174	0	0.00
Lsm12	NP_766535.1	375	2	5.62	Tmprss11f	XP_036021019.1	172	1	6.13
G6pc3	NP_787949.2	222	0	0.00	Tmprss11b	NP_795998.2	121	3	26.13
Hdac5	XP_036012236.1	2184	3	1.45	Tmprss11e	NP_766468.1	250	3	12.65
Hrob	XP_006533103.1	223	1	4.73	Ythdc1	XP_006534926.1	642	0	0.00
Asb16	NP_683755.1	1189	1	0.89	Ugt2b34	NP_705826.1	850	4	4.96
Tmub2	NP_082352.1	315	3	10.04	Ugt2b1	NP_690024.1	971	5	5.43
Atxn7l3	XP_030101742.1	333	2	6.33	Ugt2b35	NP_766469.1	761	6	8.31
Ubtf	NP_001289884.1	989	2	2.13	Ugt2b36	NP_001025038.1	824	8	10.23
Slc4a1	NP_035533.1	640	5	8.23	Ugt2b5	NP_033493.3	823	8	10.24
Rundc3a	XP_006533751.1	524	3	6.03	Ugt2b37	NP_444445.2	684	6	9.24
Slc25a39	XP_006534099.1	285	3	11.09	Ugt2a3	NP_082370.2	832	6	7.60
Grn	NP_032201.3	773	8	10.91	Ugt2b38	NP_598655.1	549	8	15.36
Fam171a2	XP_006533112.1	121	3	26.13	Ugt2a1	NP_444414.2	366	2	5.76
Itga2b	NP_034705.2	833	2	2.53	Ugt2a2	NP_001019319.1	366	2	5.76
Gpatch8	NP_001152964.1	657	5	8.02	Sult1b1	NP_001343872.1	692	12	18.28
Fzd2	NP_065256.1	877	1	1.20	Sult1d1	NP_058051.3	811	14	18.19
Meioc	NP_001121048.1	142	0	0.00	Sult1e1	NP_075624.2	476	17	37.64
Ccdc43	NP_080194.1	90	3	35.13	Csn1s1	NP_031810.1	244	10	43.19
Adam11	XP_006532079.1	445	0	0.00	Csn2	NP_001272953.1	269	5	19.59
Gjc1	NP_001345969.1	318	4	13.26	Csn1s2a	NP_031811.1	54	11	214.68
Higd1b	NP_001344509.1	105	0	0.00	Csn1s2b	NP_034103.2	77	4	54.75
Eftud2	XP_006532728.1	2283	5	2.31	Prr27	NP_001157023.1	141	11	82.22
Ccdc103	NP_082768.1	122	0	0.00	Odam	XP_006535272.1	166	14	88.88
Fam187a	NP_080042.1	16	1	65.87	Csn3	NP_001343499.1	284	4	14.84
Gfap	NP_034407.2	2030	3	1.56	Cabs1	NP_081907.1	443	6	14.27
Kif18b	NP_932063.2	1260	3	2.51	Smr3a	XP_017176242.1	45	11	257.62
C1ql1	NP_035925.2	196	3	16.13	Smr2	NP_067264.2	58	11	199.88
Dcakd	NP_080827.2	484	4	8.71	Gm7714	NP_001104249.1	0	0	#DIV/0!
Nmt1	XP_006532508.1	644	2	3.27	Prol1	NP_032670.2	91	5	57.91
Plcd3	XP_006534371.1	635	4	6.64	Amtn	NP_082069.1	253	7	29.16
Hexim1	NP_620092.1	484	1	2.18	Ambn	NP_001290360.1	351	9	27.02
Hexim2	NP_081934.1	280	1	3.76	Enam	NP_059496.1	417	9	22.75
Fmnl1	XP_006533900.1	442	0	0.00	Jchain	NP_690052.2	496	6	12.75
Efcab15	NP_001241653.1	915	1	1.15	Utp3	NP_075541.1	677	1	1.56
Spata32	NP_808469.1	114	3	27.73	Rufy3	XP_006535172.1	308	0	0.00
Map3k14	NP_058592.1	1538	3	2.06	Grsf1	NP_848815.2	573	0	0.00
Arhgap27	XP_036012751.1	980	4	4.30	Mob1b	NP_081011.1	401	0	0.00
Plekhm1	NP_898855.1	283	5	18.62	Dck	NP_031858.1	718	3	4.40
Lrrc37a	XP_011247678.1	72	0	0.00	Slc4a4	XP_017176484.1	653	6	9.68
Gm884	XP_030101964.1	62	0	0.00	Gc	NP_032122.1	876	7	8.42

Lyzl6	NP_001343401.1	104	0	0.00	Npffr2	XP_017176078.1	473	9	20.05
Rprml	NP_001028384.1	296	1	3.56	Adamts3	NP_001074870.1	415	6	15.24
Gosr2	XP_030102050.1	622	7	11.86	Cox18	NP_001028482.2	1027	6	6.16
Wnt9b	NP_035849.3	769	5	6.85	Ankrd17	XP_006535352.3	1560	7	4.73
Wnt3	NP_033547.1	876	9	10.83	Alb	NP_033784.2	5085	14	2.90
Nsf	NP_032766.2	2128	11	5.45	Afp	NP_031449.3	1251	11	9.27
Arf2	NP_031503.1	1672	13	8.19	Afm	NP_660128.2	730	9	12.99
Crhr1	NP_031788.1	1135	10	9.29	Albfm1	NP_001239590.1	459	3	6.89
Sppl2c	NP_001076004.1	321	5	16.42	Rassf6	XP_006535307.1	309	7	23.87
Mapt	NP_001272383.1	1751	9	5.42	Cxcl5	NP_033167.2	1111	10	9.49
Kansl1	NP_001359389.1	465	7	15.87	Ppbp	NP_076274.1	673	11	17.23
Cdc27	NP_001272918.1	1841	5	2.86	Pf4	NP_064316.1	838	10	12.58
Myl4	XP_030101494.1	1367	9	6.94	Cxcl3	NP_976065.1	656	7	11.25
Itgb3	NP_058060.2	1282	6	4.93	Cxcl15	NP_035469.1	1525	10	6.91
Gm11639	XP_017170366.1	983	3	3.22	Cxcl1	NP_032202.1	1292	9	7.34
Efcab3	XP_017170251.1	1014	6	6.24	Cxcl2	NP_033166.1	1091	7	6.76
Mettl2	NP_766155.3	481	2	4.38	Mthfd2l	XP_006535239.1	721	0	0.00
Tlk2	NP_001106176.1	483	3	6.55	Epgn	NP_444317.1	226	5	23.32
Mrc2	XP_006532469.1	337	3	9.38	Ereg	NP_031976.1	478	11	24.25
Marchf10	NP_766156.2	406	2	5.19	Areg	NP_033834.1	827	11	14.02
Tanc2	XP_006534535.1	1275	2	1.65	Btc	NP_031594.1	251	6	25.19
Cyb561	XP_036012169.1	265	0	0.00	Parm1	NP_663537.1	241	0	0.00
Ace	NP_997507.1	1313	2	1.61	Rchy1	NP_001258726.1	1235	0	0.00
Ace3	XP_006533116.1	334	1	3.16	Odaph	NP_001171048.1	9	0	0.00
Kcnh6	NP_001032801.1	410	1	2.57	Cdkl2	XP_036021194.1	192	0	0.00
Dcaf7	NP_082222.1	461	3	6.86	G3bp2	NP_001074264.1	944	1	1.12
Taco1	NP_081622.1	478	0	0.00	Uso1	XP_006535203.1	1139	3	2.78
Map3k3	NP_036077.1	1524	3	2.07	Ppef2	XP_030110066.1	1741	1	0.61
Limd2	NP_001343387.1	499	1	2.11	Naaa	NP_001157159.1	303	0	0.00
Gm10840	XP_030102329.1	0	0	#DIV/0!	Sdad1	NP_766301.1	1038	4	4.06
Strada	NP_001239378.1	261	1	4.04	Cxcl9	NP_032625.2	1086	1	0.97
Ccdc47	NP_080285.2	238	0	0.00	Cxcl10	NP_067249.1	1506	1	0.70
Ddx42	NP_082350.3	1336	1	0.79	Art3	XP_006534769.1	541	2	3.90
Ftsj3	NP_079586.1	1175	5	4.48	Nup54	NP_899248.1	723	3	4.37
Psmc5	NP_032976.1	1782	2	1.18	Scarb2	NP_031670.1	606	2	3.48
Smarcd2	NP_001123659.1	642	3	4.92	Fam47e	NP_001361643.1	52	2	40.53
Tcam1	XP_006534462.1	285	3	11.09	Stbd1	NP_780305.1	287	1	3.67
Gh	XP_006532286.1	449	2	4.69	Ccdc158	XP_006535059.1	0	0	#DIV/0!
Cd79b	NP_001300868.1	654	6	9.67	Shroom3	NP_056571.2	377	2	5.59
Scn4a	NP_573462.2	778	4	5.42	Sowahb	NP_780479.1	63	1	16.73
Prr29	NP_083621.1	4	0	0.00	Septin11	XP_006535166.1	571	1	1.85
Icam2	XP_030101455.1	661	3	4.78	Ccni	XP_030109946.1	1553	1	0.68
Ern1	NP_076402.1	1242	3	2.55	2010109A12F	NP_083639.1	0	0	#DIV/0!
Tex2	XP_036012515.1	293	2	7.19	Ccng2	NP_031661.3	1793	1	0.59
Pecam1	NP_001292087.1	1717	2	1.23	Pramel33	XP_006535359.1	6	0	0.00
Milr1	NP_001258302.1	90	0	0.00	Pramel34	NP_001157756.1	70	0	0.00
Polg2	NP_056625.2	792	1	1.33	Pramel35	XP_006535360.1	6	0	0.00
Ddx5	NP_001342606.1	2248	6	2.81	Pramel36	XP_001476078.1	5	0	0.00
Cep95	XP_006533607.1	117	0	0.00	Gm6346	XP_011247943.2	5	0	0.00
Smurf2	XP_006533999.1	1507	8	5.59	Gm6351	XP_036021534.1	54	3	58.55
Kpna2	NP_034785.1	1610	6	3.93	Pramel37	NP_941069.1	72	3	43.91
Bptf	XP_036012378.1	3461	8	2.44	Pramel38	XP_006535368.1	6	0	0.00
Nol11	NP_001350007.1	402	2	5.24	Pramel39	NP_808581.1	5	0	0.00
Pitpnc1	NP_001346545.1	333	5	15.82	Pramel40	XP_011247944.2	42	2	50.19
Psmd12	NP_080170.1	1508	8	5.59	AA792892	NP_849225.2	89	1	11.84
Helz	NP_938040.1	734	12	17.23	Pramel42	NP_001230866.1	14	0	0.00
Cacng1	NP_031608.1	424	9	22.37	Pramel43	XP_001476360.1	5	0	0.00
Cacng4	NP_062304.1	389	6	16.26	Gm3183	XP_017176765.1	5	0	0.00
Cacng5	NP_542375.1	534	5	9.87	Gm16513	NP_001186984.1	6	0	0.00
Prkca	NP_035231.2	1212	11	9.57	Gm6468	NP_001365656.1	42	3	75.28
Apoh	NP_038503.4	1048	1	1.01	Pramel46	XP_001476598.1	5	0	0.00
Cep112	XP_006534486.1	106	5	49.71	Pramel47	NP_001257385.1	117	1	9.01
Axin2	XP_030101353.1	1224	7	6.03	Pramel48	NP_001346946.1	43	2	49.02
Rgs9	NP_035398.2	553	3	5.72	Gm3286	XP_017176059.1	28	1	37.64
Gna13	NP_034433.3	1225	10	8.60	Pramel49	XP_987278.1	5	0	0.00
Amz2	NP_001239122.1	102	10	103.32	Gm6348	XP_011247946.2	5	0	0.00

Slc16a6	XP_036012105.1	316	3	10.01	Gm7982	XP_036021536.1	43	1	24.51
Arsg	XP_017170289.1	449	9	21.12	Pramel50	NP_001257386.1	72	2	29.28
Wipi1	NP_666052.1	581	1	1.81	E330014E10R	NP_001116140.1	28	1	37.64
Prkar1a	NP_001349606.1	1403	10	7.51	Cxcl13	NP_061354.1	878	0	0.00
Fam20a	NP_722477.1	313	0	0.00	Cnot6l	NP_849185.2	745	0	0.00
1700012B07R	XP_006534177.1	25	0	0.00	Mrpl1	XP_006535355.1	1341	0	0.00
Abca8b	NP_001349678.1	342	4	12.33	Fras1	NP_780682.3	3741	2	0.56
Abca8a	XP_006533124.3	551	7	13.39	Anxa3	NP_038498.2	658	3	4.81
Abca9	XP_017169975.1	329	4	12.81	Bmp2k	NP_542439.1	937	3	3.37
Abca6	NP_671751.2	734	7	10.05	Paqr3	XP_036020961.1	244	6	25.92
Abca5	NP_671752.2	429	4	9.83	Naa11	NP_001028363.1	556	1	1.90
Map2k6	NP_001343274.1	713	10	14.78	Gk2	NP_034424.1	1055	1	1.00
Kcnj16	NP_034734.3	401	9	23.65	Antxr2	NP_598499.1	406	6	15.57
Kcnj2	NP_032451.1	506	3	6.25	Prdm8	NP_084223.2	500	3	6.32
Sox9	NP_035578.3	1688	4	2.50	Fgf5	NP_034333.1	809	6	7.82
Slc39a11	NP_001349866.1	241	2	8.75	Cfap299	XP_006535343.1	242	4	17.42
Sstr2	NP_033243.2	683	0	0.00	Bmp3	NP_775580.1	766	9	12.38
Cog1	XP_006532406.1	430	0	0.00	Prkg2	NP_032952.3	1144	10	9.21
Fam104a	NP_613064.2	35	0	0.00	Gm3470	XP_036021560.1	394	2	5.35
D11Wsu47e	XP_017170063.1	190	0	0.00	Rasgef1b	NP_851835.1	544	6	11.62
Cpsf4l	XP_006533768.1	373	0	0.00	Hnrnpd	NP_001070735.1	1899	5	2.77
Cdc42ep4	XP_006533878.1	227	1	4.64	4930524J08R	XP_030110841.1	0	0	#DIV/0!
Sdk2	XP_006533316.1	341	0	0.00	Hnrnpdl	XP_030110521.1	1726	7	4.27
Rpl38	XP_017170209.1	1088	3	2.91	Enoph1	NP_080697.2	335	9	28.31
Ttyh2	NP_444503.2	243	4	17.35	Tmem150c	NP_001369779.1	191	1	5.52
Dnaic2	NP_001030050.2	334	3	9.47	Sec31a	XP_011247865.1	832	2	2.53
Kif19a	NP_001096085.1	973	1	1.08	Lin54	XP_017176337.1	647	0	0.00
Btbd17	XP_017170266.1	265	4	15.91	Cops4	NP_036131.1	898	3	3.52
Gpr142	NP_001333701.1	174	3	18.17	Plac8	XP_017176338.1	397	1	2.65
Gprc5c	XP_030102156.2	374	0	0.00	Coq2	XP_036021392.1	662	11	17.51
Cd300a	NP_001346741.1	490	4	8.60	Hpse	NP_690016.1	521	5	10.11
Cd300lb	NP_954691.3	289	6	21.88	Helq	XP_006534886.1	590	5	8.93
Cd300c	NP_001355168.1	222	3	14.24	Mrps18c	NP_081102.1	735	8	11.47
Cd300ld	NP_663412.1	185	3	17.09	Abraxas1	XP_006535280.1	319	4	13.22
Cd300c2	NP_598919.1	451	6	14.02	Gpat3	NP_766303.1	587	7	12.57
Cd300ld4	XP_036012060.1	11	0	0.00	Nkx6-1	NP_659204.1	563	0	0.00
Cd300ld2	XP_030102331.1	11	0	0.00	Cds1	NP_775546.2	520	2	4.05
Cd300ld5	NP_001095127.1	11	0	0.00	Wdfy3	XP_036021402.1	1306	2	1.61
Cd300ld3	NP_954671.1	16	0	0.00	Arhgap24	XP_036020963.1	520	6	12.16
Cd300e	NP_742047.1	92	3	34.37	Mapk10	NP_001305031.1	2135	5	2.47
Rab37	NP_001157225.1	416	5	12.67	Ptpn13	NP_035334.2	847	4	4.98
Cd300lf	XP_017170043.1	290	4	14.54	Slc10a6	NP_083691.1	249	0	0.00
Slc9a3r1	NP_036160.1	1140	2	1.85	1700016H13F	XP_017176610.1	17	1	61.99
Nat9	NP_079676.1	158	1	6.67	Aff1	XP_006534864.1	430	1	2.45
Tmem104	NP_001028565.1	41	4	102.82	Klhl8	NP_001346916.1	199	1	5.30
Grin2c	XP_011247040.1	820	3	3.86	Hsd17b13	NP_932147.2	641	1	1.64
Fdxr	XP_006532249.1	675	6	9.37	Hsd17b11	NP_444492.1	409	0	0.00
Fads6	NP_828874.3	88	9	107.79	Nudt9	NP_083070.2	278	3	11.37
Ush1g	NP_789817.1	1214	5	4.34	Gm17660	NP_001157244.1	40	5	131.74
Otop2	NP_766389.2	107	4	39.40	Sparcl1	NP_001345943.1	1050	7	7.03
Otop3	NP_081408.2	158	5	33.35	Dspp	NP_034210.2	406	6	15.57
Hid1	NP_001333703.1	347	4	12.15	Dmp1	NP_001345942.1	718	6	8.81
Cdr2l	NP_001074398.1	239	6	26.46	Ibsp	NP_032344.2	926	6	6.83
Mrpl58	NP_001334574.1	1019	2	2.07	Mepe	NP_444402.2	314	6	20.14
Atp5h	NP_082138.1	1003	7	7.36	Spp1	NP_001191130.1	1463	5	3.60
Kctd2	NP_899108.3	336	2	6.27	Pkd2	NP_032887.3	568	0	0.00
Trim80	NP_001028947.1	95	0	0.00	Thoc2l	XP_036020565.1	865	1	1.22
Slc16a5	NP_001074403.1	317	2	6.65	Zfp951	XP_036021295.1	139	3	22.75
Armc7	NP_808446.1	108	8	78.07	Abcg3	NP_084515.2	562	6	11.25
Nt5c	NP_056622.1	387	4	10.89	Gbp8	XP_006535346.1	245	5	21.51
Jpt1	NP_032284.1	223	1	4.73	Gbp9	NP_766365.1	230	4	18.33
Sumo2	NP_579932.1	2608	5	2.02	Gbp4	NP_001346008.1	386	3	8.19
Nup85	NP_001002929.3	781	3	4.05	Gbp10	NP_001034735.2	342	3	9.24
Gga3	NP_001238996.1	814	4	5.18	Gbp6	XP_006534751.1	342	3	9.24
Mrps7	NP_079581.1	1559	1	0.68	Lrrc8b	XP_030110511.1	604	1	1.74
Mif4gd	XP_036012856.1	170	2	12.40	Lrrc8c	XP_017176062.1	592	1	1.78

Slc25a19	XP_017170203.1	286	3	11.05	Rps15a-ps5	XP_036021575.1	1355	1	0.78
Grb2	NP_032189.1	1972	5	2.67	Lrrc8d	NP_848816.3	577	1	1.83
Tmem94	XP_030102196.1	221	3	14.31	Zfp326	NP_061229.2	321	1	3.28
Caskin2	NP_542374.2	1289	3	2.45	Barhl2	NP_001005477.1	470	2	4.48
Tsen54	XP_006534478.1	267	1	3.95	Zfp644	NP_001350251.1	487	1	2.16
Llgl2	NP_001239461.1	838	4	5.03	Hfm1	XP_011247789.1	1023	5	5.15
Myo15b	NP_001371162.1	958	2	2.20	Cdc7	NP_033993.2	1224	2	1.72
Recql5	XP_006532422.1	671	0	0.00	Tgfbr3	NP_035708.2	568	1	1.86
Smim5	XP_006534005.1	12	0	0.00	Brdt	XP_030109918.1	1469	0	0.00
Smim6	NP_001156470.1	0	0	#DIV/0!	Ephx4	NP_001001804.2	468	0	0.00
Sap30bp	NP_065229.2	259	0	0.00	Lpcat2b	NP_081875.1	271	0	0.00
Itgb4	XP_017169838.1	904	4	4.66	Btbd8	NP_001365195.1	39	0	0.00
Galk1	NP_058601.2	884	2	2.38	1700028K03R	NP_780450.2	29	1	36.34
H3f3b	XP_006532311.1	1735	4	2.43	Glmn	NP_001155211.1	568	3	5.57
Unk	NP_766157.1	1338	2	1.58	Rpap2	NP_659160.2	428	1	2.46
Unc13d	NP_001009573.2	497	6	12.72	Gfi1	NP_001254550.1	1113	4	3.79
Wbp2	NP_001348375.1	291	6	21.73	Evi5	NP_031990.2	437	5	12.06
Trim47	NP_766158.3	377	7	19.57	Ube2d2b	NP_001263326.1	1520	0	0.00
Trim65	XP_036012674.1	357	7	20.66	Rpl5	NP_058676.1	1463	3	2.16
Mrpl38	NP_077139.2	529	6	11.95	Dipk1a	NP_080338.1	151	3	20.94
Fbf1	XP_030101760.1	285	6	22.19	Mtf2	NP_038855.2	668	4	6.31
Acox1	NP_056544.2	1035	8	8.15	Tmed5	NP_001348395.1	457	4	9.22
Ten1	NP_081383.1	187	5	28.18	Ccdc18	NP_082757.1	268	1	3.93
Evpl	NP_079552.3	987	6	6.41	Dr1	NP_080382.2	663	1	1.59
Srp68	NP_666144.3	949	8	8.88	Pigg	NP_001297618.1	315	7	23.42
Galr2	XP_011247029.1	495	2	4.26	Pde6b	NP_032832.2	938	6	6.74
Exoc7	NP_001156344.1	1028	1	1.03	Atp5k	NP_031533.2	624	1	1.69
Foxj1	NP_032266.3	1056	2	2.00	Mfsd7a	XP_006534995.1	283	9	33.52
Rnf157	NP_081534.1	303	10	34.78	Pcgf3	XP_030110654.1	408	9	23.25
Ubald2	NP_795876.1	65	0	0.00	Cplx1	NP_031782.3	1439	12	8.79
Qrich2	XP_006533168.2	84	0	0.00	Gak	XP_006534968.1	2918	12	4.33
Prpsap1	XP_006534081.1	667	8	12.64	Tmem175	NP_001346352.1	86	6	73.53
Sphk1	XP_017169887.1	884	13	15.50	Dgkq	NP_950176.1	507	11	22.87
Ube2o	XP_006533169.1	1725	8	4.89	Idua	NP_032351.2	229	10	46.02
Aanat	XP_030101334.1	625	11	18.55	Slc26a1	NP_001297620.1	694	7	10.63
Rhbdf2	NP_766160.2	757	11	15.31	Fgfrl1	XP_017176107.1	648	6	9.76
Cygb	NP_084482.1	691	11	16.78	Rnf212	XP_006535394.1	265	9	35.79
Prcd	XP_017169685.1	28	0	0.00	Tmed11	NP_080385.2	207	4	20.37
St6galnac2	NP_033206.2	250	9	37.94	Vmn2r8	XP_036021297.1	31	0	0.00
St6galnac1	NP_035501.2	302	0	0.00	Vmn2r9	NP_001372120.1	34	0	0.00
Mxra7	NP_080556.1	232	10	45.43	Vmn2r10	NP_001371964.1	16	0	0.00
Jmjd6	NP_001350292.1	710	14	20.78	Vmn2r11	XP_036021150.1	27	0	0.00
Mettl23	NP_083141.3	230	9	41.24	Vmn2r12	NP_001372158.1	7	0	0.00
Srsf2	XP_030101584.1	1102	5	4.78	Vmn2r13	XP_036020976.1	7	0	0.00
Mfsd11	XP_017170234.1	143	3	22.11	Vmn2r14	XP_017176349.1	7	0	0.00
Mgat5b	NP_766536.2	520	3	6.08	Vmn2r15	XP_036020866.1	24	0	0.00
Sec14l1	NP_083053.2	438	2	4.81	Vmn2r16	XP_030110503.1	27	0	0.00
Septin9	NP_001106958.1	763	4	5.53	Vmn2r17	XP_030110510.1	30	0	0.00
Tnrc6c	XP_030101765.1	309	4	13.64	Crlf2	NP_057924.3	292	0	0.00
Tmc6	XP_030101767.1	311	3	10.17	5430403G16R	NP_001365517.1	160	0	0.00
Tmc8	NP_001182017.1	147	3	21.51	4930522L14R	XP_036021577.1	160	0	0.00
6030468B19R	XP_006534561.1	25	0	0.00	Zfp932	NP_663538.2	197	0	0.00
Syngr2	NP_033330.1	272	7	27.12	Plcxd1	NP_997162.2	159	1	6.63
Tk1	NP_033413.2	1131	9	8.39	Gtpbp6	NP_660129.2	321	1	3.28
Afmid	XP_006534311.1	357	1	2.95	Zfp605	NP_001157468.1	177	2	11.91
Birc5	NP_033819.1	1335	4	3.16	Chfr	NP_001276506.1	808	2	2.61
Tmem235	NP_001079004.1	70	0	0.00	Golga3	NP_001334318.1	323	1	3.26
Tha1	NP_082195.2	276	5	19.09	Ankle2	NP_001240743.1	195	0	0.00
Socs3	NP_031733.1	1434	6	4.41	Pgam5	NP_082549.2	485	1	2.17
Pgs1	NP_598518.1	511	0	0.00	Pxmp2	NP_033019.2	350	0	0.00
Dnah17	XP_006534213.1	717	2	2.94	Pole	NP_035262.2	1404	4	3.00
Cyth1	NP_001106169.1	615	3	5.14	P2rx2	NP_001158306.1	360	0	0.00
Usp36	XP_036012906.1	433	2	4.87	Lrcol1	XP_006535095.1	12	0	0.00
Timp2	NP_035724.2	842	5	6.26	Fbrsl1	NP_001136114.1	116	1	9.09
Cep295nl	XP_006533922.1	106	1	9.94	Galnt9	NP_938048.2	555	0	0.00
Lgals3bp	XP_036012322.1	715	5	7.37	Noc4l	NP_705798.2	927	5	5.68

Cant1	XP_030102267.1	432	3	7.32	Ddx51	NP_081432.2	1344	5	3.92
C1qtnf1	XP_036012760.1	152	1	6.93	Ep400	NP_775089.1	1519	3	2.08
Engase	XP_030101772.1	241	1	4.37	Pus1	XP_006535211.1	956	3	3.31
Rbfox3	XP_030102000.1	1945	1	0.54	Ulk1	NP_033495.2	692	0	0.00
Enpp7	NP_001346503.1	198	2	10.65	Hscb	NP_705799.2	346	1	3.05
Cbx2	XP_006532167.1	671	3	4.71	Chek2	XP_030110519.1	2255	2	0.93
Cbx8	NP_038954.1	622	3	5.08	Ttc28	NP_001254551.1	982	3	3.22
Cbx4	NP_031651.2	664	3	4.76	Pitpnb	NP_001288572.1	531	2	3.97
Tbc1d16	NP_001334554.1	526	0	0.00	Mn1	NP_001074704.1	200	2	10.54
Ccdc40	XP_017169889.1	307	0	0.00	Cryba4	XP_006534812.1	231	0	0.00
Gaa	NP_032090.3	645	0	0.00	Crybb1	XP_006534817.1	200	1	5.27
Eif4a3	NP_619610.1	1988	2	1.06	Tpst2	XP_006534912.1	850	1	1.24
Card14	XP_006532429.1	604	2	3.49	Tfip11	NP_061253.2	615	0	0.00
Sgsh	NP_061292.2	258	1	4.08	Srrd	NP_001346317.1	72	0	0.00
Slc26a11	XP_006533481.1	217	4	19.43	Hps4	NP_001346782.1	217	0	0.00
Rnf213	XP_030102102.1	745	2	2.83	Gm6583	NP_001034317.2	30	1	35.13
Endov	NP_001158108.1	245	2	8.60	Asphd2	XP_017176607.1	373	1	2.83
Nptx1	NP_032756.1	779	4	5.41	Sez6l	XP_030110581.1	962	4	4.38
Rptor	NP_083174.2	1610	4	2.62	Gm6588	NP_001170975.1	30	1	35.13
Chmp6	NP_001078967.1	472	2	4.47	Myo18b	NP_083177.2	1182	3	2.67
Baiap2	NP_001032844.2	624	5	8.44	Grk3	XP_036021101.1	1802	3	1.75
Aatk	NP_001185714.1	406	5	12.98	Crybb2	XP_006534823.2	183	0	0.00
Cep131	NP_033864.3	297	3	10.65	Crybb3	NP_067327.1	189	0	0.00
Tepsin	NP_898960.2	186	6	34.00	2900026A02F	XP_017176370.1	62	0	0.00
Ndufaf8	NP_001103712.1	30	4	140.52	Tmem211	XP_006535084.1	183	0	0.00
Slc38a10	XP_030102210.1	201	3	15.73	Sgsm1	NP_766306.2	626	3	5.05
Bahcc1	XP_030101898.1	232	0	0.00	Aym1	NP_001012744.2	4	0	0.00
Actg1	NP_033739.1	2530	5	2.08	Wscd2	XP_017176417.1	613	4	6.88
Fscn2	XP_006533322.1	418	3	7.56	Cmklr1	NP_001345989.1	330	2	6.39
Faap100	NP_082256.2	145	0	0.00	Ficd	NP_001010825.2	189	0	0.00
Nploc4	NP_955763.1	811	2	2.60	Sart3	NP_058622.1	564	1	1.87
Tspan10	NP_663338.2	199	0	0.00	Iscu	XP_030110614.1	707	1	1.49
Pde6g	XP_006532518.1	404	1	2.61	Tmem119	XP_036020982.1	237	0	0.00
Oxld1	NP_001343314.1	99	4	42.58	Selplg	NP_033177.3	824	2	2.56
Ccdc137	NP_690020.1	58	0	0.00	Coro1c	NP_035909.2	857	2	2.46
Arl16	NP_932112.2	1309	5	4.03	Ssh1	NP_001350398.1	765	2	2.76
Hgs	XP_006532333.2	1263	3	2.50	Dao	NP_034148.2	574	0	0.00
Mrpl12	NP_081480.2	1350	5	3.90	Svop	NP_081081.1	772	1	1.37
Slc25a10	NP_038798.2	585	6	10.81	Usp30	NP_001028374.1	539	2	3.91
Gcgr	XP_017169752.1	1089	3	2.90	Alkbh2	NP_001346869.1	202	1	5.22
Mcrip1	NP_001360863.1	49	1	21.51	Ung	NP_001035781.1	1331	1	0.79
Ppp1r27	NP_081090.1	921	6	6.87	Acacb	XP_006530174.1	1209	4	3.49
P4hb	NP_035162.1	2324	5	2.27	Foxn4	XP_006530184.1	788	4	5.35
Arhgdia	NP_001350354.1	955	8	8.83	Myo1h	NP_001158045.1	1010	6	6.26
Alyref	NP_035698.1	864	1	1.22	Kctd10	NP_080421.2	370	5	14.24
Anapc11	NP_079665.1	986	9	9.62	Ube3b	NP_473434.2	1014	7	7.28
Npb	NP_695020.1	434	0	0.00	Mmab	NP_001334327.1	220	3	14.37
Pcyt2	XP_036012826.1	600	6	10.54	Mvk	XP_006530246.1	644	3	4.91
Sirt7	NP_694696.2	1054	7	7.00	Fam222a	NP_001004180.1	75	0	0.00
Mafg	XP_017169796.1	400	8	21.08	Trpv4	NP_071300.2	643	1	1.64
Pycr1	NP_001366016.1	667	2	3.16	Gltp	NP_062795.2	301	1	3.50
Myadml2	NP_001191749.1	177	3	17.86	Tchp	NP_084268.2	195	1	5.40
Notum	NP_001369198.1	540	4	7.81	Git2	XP_030110387.1	648	1	1.63
Aspscr1	NP_081153.1	492	7	14.99	Ankrd13a	NP_001365826.1	177	0	0.00
Cenpx	XP_011247152.2	244	2	8.64	2610524H06F	NP_851420.1	22	0	0.00
Lrrc45	NP_705773.2	147	0	0.00	4930519G04F	NP_080539.1	3	0	0.00
Rac3	NP_573486.1	1963	0	0.00	Oasl2	NP_035984.2	3023	2	0.70
Dcxr	NP_001334537.1	369	2	5.71	Oasl1	NP_001346874.1	2937	3	1.08
Cbr2	XP_006532159.1	290	1	3.63	2210016L21R	XP_036021414.1	29	0	0.00
Hmga1b	NP_001159948.1	386	0	0.00	Hnf1a	NP_033353.2	1055	0	0.00
Rfng	XP_006532669.1	291	2	7.24	Sppl3	NP_083288.2	275	0	0.00
Gps1	NP_001171345.1	820	3	3.86	Rpl37rt	NP_001258519.1	930	3	3.40
Dus1l	XP_011247517.1	648	3	4.88	Acads	NP_031409.2	818	0	0.00
Fasn	XP_030101416.1	1399	2	1.51	Unc119b	NP_780561.1	484	1	2.18
Ccdc57	XP_017170256.1	103	2	20.46	Mlec	NP_780612.2	357	0	0.00
Slc16a3	NP_109621.1	543	3	5.82	Cabp1	NP_001297641.1	1283	1	0.82

Csnk1d	XP_011246964.1	1244	3	2.54	Pop5	XP_017176108.1	320	1	3.29
Cd7	NP_033984.1	430	2	4.90	Rnf10	NP_001289378.1	447	4	9.43
Sectm1b	NP_081183.3	115	5	45.82	Coq5	NP_080780.1	531	4	7.94
Sectm1a	XP_017169903.1	104	5	50.67	Dynll1	NP_062656.3	752	1	1.40
Tex19.2	NP_081898.1	79	4	53.36	Srsf9	NP_079849.1	1339	5	3.94
Tex19.1	NP_082878.2	270	4	15.61	Gatc	NP_083921.1	86	1	12.25
Uts2r	NP_663415.1	382	6	16.55	Triap1	NP_081209.1	378	0	0.00
Ogfod3	NP_079678.1	53	4	79.54	Cox6a1	NP_031774.2	745	2	2.83
Hexdc	NP_001139545.1	113	0	0.00	Msi1	NP_032655.1	733	3	4.31
Cybc1	NP_001241664.1	92	1	11.46	Pla2g1b	NP_035237.1	602	1	1.75
Narf	NP_080548.3	262	0	0.00	Sirt4	XP_036021468.1	828	5	6.36
Foxk2	XP_011247520.1	862	4	5.15	Pxn	XP_006530265.1	1558	3	2.03
Wdr45b	NP_080069.2	333	3	10.55	Rplp0	NP_031501.1	2141	3	1.48
Rab40b	NP_631886.2	762	4	6.51	Gcn1	NP_766307.2	1040	7	7.09
Fn3krp	XP_006533337.1	135	5	48.79	Rab35	NP_937806.1	590	5	8.93
Fn3k	NP_071297.1	179	4	31.40	Bicdl1	NP_001074277.1	251	3	12.60
Tbcd	NP_084154.1	644	8	18.70	Cit	XP_006530193.1	1394	2	1.51
Zfp750	NP_848878.1	247	5	32.82	Prkab1	NP_114075.1	751	1	1.40
B3gntl1	NP_848779.2	416	6	25.33	Tmem233	NP_001095016.2	32	0	0.00
Metrnl	NP_659046.1	186	7	72.11	Ccdc60	NP_001346933.1	87	0	0.00
Ptchd3	NP_083325.1	147	0	0.00	Hspb8	NP_109629.1	690	0	0.00
Rab10	NP_057885.1	1106	3	5.72	Srrm4	NP_081162.1	377	1	2.80
Kif3c	NP_032471.2	1173	5	8.17	Suds3	NP_001116138.1	380	1	2.77
Asxl2	NP_001257917.1	397	4	17.70	Taok3	NP_001074777.1	394	6	16.05
Dtnb	NP_001155937.1	314	1	5.16	Pebp1	NP_061346.2	989	1	1.07
Dnmt3a	XP_030102398.1	1189	4	5.07	Vsig10	NP_001028483.2	58	1	18.17
Pomc	NP_001265511.1	1316	4	4.27	Wsb2	NP_067514.2	795	6	7.95
Efr3b	NP_001075952.1	474	5	13.90	Rfc5	NP_082404.1	1218	3	2.60
Dnajc27	XP_006515115.1	1500	10	8.27	Ksr2	NP_001299843.1	1285	2	1.64
Adcy3	NP_612178.2	1305	7	6.28	Nos1	NP_032738.1	1273	2	1.66
Cenpo	XP_030102667.1	315	5	17.61	Fbxo21	NP_663539.1	492	5	10.71
Ptrhd1	NP_001191841.1	156	3	20.27	Tesc	NP_067319.2	1241	12	10.19
Ncoa1	XP_030102457.1	800	8	10.54	Fbxw8	NP_766309.2	865	6	7.31
Itsn2	XP_030102487.1	1438	4	2.93	Hrk	NP_031571.1	249	4	16.93
Fam228a	XP_036013527.1	0	0	#DIV/0!	Rnft2	XP_017176390.1	221	2	9.54
Fam228b	NP_001369761.1	19	0	0.00	Spring1	NP_001074705.1	36	8	234.20
Pfn4	NP_001348076.1	554	3	5.71	Med13l	NP_001334374.1	705	5	7.47
Sf3b6	NP_079599.1	586	1	1.80	Tbx3	NP_035665.2	1013	5	5.20
Fkbp1b	NP_058559.3	1116	3	2.83	Tbx5	XP_006530342.1	895	4	4.71
Wdcp	XP_017170537.1	78	0	0.00	Rbm19	NP_083038.1	886	7	8.33
Mfsd2b	NP_001028660.2	154	1	6.84	Lhx5	NP_032525.1	605	5	8.71
Ubxn2a	NP_663416.1	325	0	0.00	Sdsl	XP_011246504.2	605	4	6.97
Atad2b	NP_001349278.1	1210	2	1.74	Sds	XP_006530361.1	991	2	2.13
Klhl29	NP_001157965.1	356	1	2.96	Plbd2	NP_076114.2	129	0	0.00
Apob	NP_033823.2	2067	4	2.04	Slc8b1	NP_001171066.1	294	2	7.17
Ldah	XP_006515265.2	311	5	16.94	Tpcn1	XP_030110373.1	455	2	4.63
Gdf7	NP_038555.1	427	1	2.47	Iqcd	NP_083684.1	89	0	0.00
Hs1bp3	NP_067404.2	101	5	52.17	Rita1	NP_083372.1	29	1	36.34
Rhob	NP_031509.1	2119	6	2.98	Ddx54	NP_082317.1	1579	4	2.67
Slc7a15	XP_011242178.1	236	0	0.00	Cfap73	NP_001182023.1	63	0	0.00
Pum2	XP_030102853.1	1171	6	5.40	Rasal1	NP_001346073.1	500	2	4.22
Sdc1	NP_035649.1	1020	2	2.07	Dtx1	XP_030109991.1	482	3	6.56
Laptm4a	NP_032666.2	283	6	22.34	Oas2	NP_660262.2	462	8	18.25
Matn3	NP_034900.4	521	7	14.16	Oas3	NP_660261.1	329	8	25.63
Wdr35	NP_766058.3	379	6	16.68	Oas1e	NP_001334379.1	2773	4	1.52
Ttc32	NP_083597.1	758	3	4.17	Oas1c	XP_006530183.1	2834	4	1.49
Osr1	XP_036013300.1	281	0	0.00	Oas1f	NP_660135.2	2716	5	1.94
Nt5c1b	NP_001360900.1	377	1	2.80	Oas1h	NP_660263.1	2772	5	1.90
Rdh14	NP_076186.1	496	0	0.00	Oas1g	NP_035982.2	2876	6	2.20
Kcns3	XP_006515145.1	879	5	5.99	Oas1a	NP_660212.2	2837	6	2.23
Msgn1	NP_062417.1	145	2	14.54	Oas1d	NP_598654.1	2826	8	2.98
Gen1	XP_011242154.1	1226	2	1.72	Rph3a	XP_036020824.1	909	4	4.64
Smc6	NP_079971.2	1311	5	4.02	Ptpn11	NP_001103462.1	1699	4	2.48
Vsnl1	NP_001365858.1	1852	11	6.26	Rpl6	XP_006530290.1	1309	12	9.66
Rad51ap2	NP_001104588.1	31	1	34.00	Hectd4	XP_006530414.1	503	8	16.76
Cyria	NP_001351361.1	228	5	23.11	Trafd1	XP_017176356.2	350	15	45.17

Mycn	NP_032735.3	1280	5	4.12	Naa25	NP_766310.2	649	8	12.99
Ddx1	NP_598801.1	1844	7	4.00	Erp29	NP_080405.1	428	4	9.85
Nbas	XP_011242197.1	275	5	19.16	Tmem116	XP_017176661.1	813	15	19.44
Lratd1	NP_083283.2	136	5	38.75	Adam1b	NP_742123.2	174	0	0.00
Trib2	XP_006515118.1	1317	5	4.00	Adam1a	NP_742124.2	180	5	29.28
Lpin1	XP_006515045.1	994	2	2.12	Mapkapk5	NP_034895.1	328	12	38.56
Ntsr2	NP_032773.2	934	2	2.26	Aldh2	NP_033786.1	1168	12	10.83
Greb1	NP_001239000.1	386	0	0.00	Acad12	NP_848914.2	203	2	10.38
E2f6	NP_150373.2	904	8	9.33	Acad10	XP_006530526.1	203	2	10.38
Rock2	XP_036013164.1	2076	2	1.02	Brap	NP_082503.2	393	6	16.09
Pqlc3	NP_766162.2	149	6	42.44	Atxn2	NP_033151.2	1216	9	7.80
2410004P03R	NP_001188262.1	101	0	0.00	Sh2b3	NP_001293057.1	522	8	16.15
Kcnf1	NP_963289.2	954	7	7.73	Pheta1	NP_001346878.1	143	8	58.96
Pdia6	NP_082235.2	1251	6	5.05	Cux2	XP_006530220.1	1081	6	5.85
Atp6v1c2	XP_006515262.1	518	6	12.21	Myl2	NP_034991.3	1419	5	3.71
Nol10	NP_001008421.1	620	7	11.90	Ccdc63	NP_001276738.1	256	3	12.35
Odc1	XP_017170479.1	1081	1	0.97	Ppp1cc	XP_006530263.1	3082	4	1.37
Hpcal1	NP_001348574.1	1091	7	6.76	Hvcn1	NP_001035954.1	546	0	0.00
5730507C01R	XP_011242163.1	129	0	0.00	Tctn1	NP_001034242.2	265	1	3.98
Gm5784	NP_001371075.1	4	0	0.00	Pptc7	NP_796216.2	923	3	3.43
Gm9257	XP_006515363.1	950	2	2.22	Rad9b	XP_006530371.1	336	0	0.00
Gm38702	XP_036013623.1	950	2	2.22	Vps29	NP_001346155.1	838	3	3.77
Gm3993	XP_036013633.1	261	1	4.04	Fam216a	NP_081159.1	81	0	0.00
Gm10479	XP_036013636.1	950	2	2.22	Gpn3	NP_077178.1	468	1	2.25
Asap2	XP_006515110.1	716	2	2.94	Arpc3	NP_062798.1	781	1	1.35
Itgb1bp1	XP_006515052.1	229	1	4.60	Anapc7	XP_036021221.1	959	2	2.20
Cpsf3	NP_061283.2	1073	9	8.84	Atp2a2	NP_033852.1	1623	3	1.95
Iah1	XP_006515238.1	226	2	9.33	Ift81	XP_011246464.1	383	1	2.75
Adam17	NP_033745.4	1074	4	3.93	P2rx7	NP_001033934.1	748	4	5.64
Ywhaq	NP_035869.1	2829	9	3.35	P2rx4	NP_035156.2	374	4	11.27
Gm17330	XP_036013032.1	261	1	4.04	Camkk2	XP_030110166.1	735	3	4.30
9030624G23R	NP_001243418.1	950	3	3.33	Anapc5	NP_001276446.1	935	3	3.38
Taf1b	NP_065639.2	280	5	18.82	Rnf34	NP_085041.1	435	2	4.85
Grhl1	NP_001154878.1	363	4	11.61	Kdm2b	XP_006530439.1	1794	2	1.17
Klf11	NP_848134.1	602	6	10.50	Orai1	NP_780632.1	297	6	21.29
Cys1	NP_619627.3	157	1	6.71	Morn3	NP_083388.1	176	4	23.95
Rrm2	NP_033130.1	1505	5	3.50	Tmem120b	NP_001034812.1	141	5	37.37
Mboat2	NP_080313.2	499	2	4.22	Rhof	XP_030110328.1	1638	7	4.50
Kidins220	XP_006515322.1	1191	4	3.54	Setd1b	XP_006530300.1	1534	7	4.81
Id2	NP_034626.1	958	1	1.10	Hpd	NP_032303.1	1590	1	0.66
Rnf144a	XP_006515007.1	325	4	12.97	Psmd9	NP_080276.2	795	1	1.33
Rsad2	NP_067359.2	627	3	5.04	Wdr66	NP_001357769.1	164	1	6.43
Cmpk2	NP_065582.3	707	7	10.43	Bcl7a	NP_084126.1	314	5	16.78
Sox11	NP_033260.4	629	5	8.38	Mlxip	NP_598678.2	306	1	3.44
Dcdc2c	NP_001171435.2	146	10	72.18	Il31	NP_083870.1	218	0	0.00
Allc	NP_001351288.1	484	8	17.42	Lrrc43	NP_001028633.1	562	1	1.88
Colec11	NP_082142.1	591	12	21.40	B3gnt4	XP_011246501.1	106	0	0.00
Rps7	NP_035430.1	1041	8	8.10	Diablo	XP_036021304.1	608	3	5.20
Rnaseh1	NP_035405.2	742	10	14.20	Vps33a	NP_001346442.1	543	3	5.82
Adi1	NP_598813.1	410	15	38.56	Clip1	XP_030110573.1	949	4	4.44
Trappc12	NP_001363936.1	181	15	87.34	Zcchc8	NP_081770.3	361	6	17.52
Eipr1	XP_006515183.1	175	16	96.36	Rsrc2	NP_001346793.1	317	3	9.97
Myt1l	XP_011242135.1	1247	12	10.14	Kntc1	NP_001035886.1	682	3	4.64
Pxdn	XP_006515268.1	533	14	27.68	Hcar2	NP_109626.1	545	3	5.80
Tpo	NP_033443.1	334	10	31.55	Hcar1	NP_780729.3	465	3	6.80
Sntg2	NP_766539.2	652	15	24.25	Denr	NP_080879.1	798	3	3.96
Tmem18	NP_742046.2	226	13	60.62	Ccdc62	XP_036020857.1	161	2	13.09
Alkal2	NP_001153215.1	106	4	39.77	Hip1r	NP_659507.3	885	2	2.38
Acp1	NP_001103709.1	586	10	17.98	Vps37b	NP_808544.1	252	1	4.18
Sh3yl1	NP_038737.1	559	12	22.62	Abcb9	NP_063928.2	476	2	4.43
Fam110c	NP_082104.2	109	10	96.69	Ogfod2	NP_079947.1	195	4	21.62
Lamb1	NP_032508.3	833	4	5.06	Arl6ip4	NP_653092.1	235	1	4.48
Dld	NP_031887.2	1478	3	2.14	Pitpnm2	XP_030110086.1	477	4	8.84
Slc26a3	XP_017170439.1	528	8	15.97	Mphosph9	NP_001074792.1	325	3	9.73
Cbll1	XP_006515002.1	390	3	8.11	2810006K23R	NP_001128189.1	554	2	3.80
Slc26a4	XP_017170557.1	531	0	0.00	Cdk2ap1	NP_038840.2	234	2	9.01

Bcap29	NP_001157562.1	782	10	13.48	Sbno1	XP_006530385.1	445	5	11.84
Dus4l	XP_006515286.1	482	5	10.93	Kmt5a	NP_084517.2	1855	1	0.57
Cog5	NP_001156598.1	448	8	18.82	Rilpl2	NP_084535.1	160	0	0.00
Gpr22	NP_780400.3	589	5	8.95	Snrnp35	NP_083808.1	850	2	2.48
Hbp1	XP_036013520.1	164	6	38.56	Rilpl1	NP_067405.1	186	0	0.00
Prkar2b	NP_001351336.1	1308	8	6.45	Tmed2	XP_036021223.1	956	2	2.20
Pik3cg	NP_001139672.1	2294	10	4.59	Ddx55	NP_080685.2	2252	8	3.74
Ccdc71l	NP_001156375.1	103	1	10.23	Eif2b1	NP_663346.1	893	3	3.54
Nampt	NP_067499.2	851	3	3.72	Gtf2h3	NP_852075.1	452	1	2.33
Sypl	NP_038663.2	300	5	17.57	Atp6v0a2	NP_035726.2	840	6	7.53
Cdhr3	XP_006515253.1	353	6	17.91	Dnah10	NP_062409.1	676	4	6.24
Atxn7l1	NP_001360952.1	389	1	2.71	Ccdc92	NP_659068.1	226	5	23.32
Efcab10	NP_083428.1	107	2	19.70	Zfp664	NP_001075219.1	273	6	23.16
Twistnb	XP_036013358.1	858	6	7.37	Rflna	NP_082719.1	194	0	0.00
Ferd3l	NP_277057.1	199	5	26.48	Ncor2	NP_001240834.1	2346	4	1.80
Twist1	NP_035788.1	1447	5	3.64	Scarb1	NP_001192011.1	757	4	5.57
Hdac9	XP_030102844.1	1432	9	6.62	Ubc	NP_062613.3	3944	4	1.07
Prps1l1	NP_083570.1	922	7	8.00	Gm10382	XP_036021530.1	0	0	#DIV/0!
Snx13	XP_006515125.3	498	12	25.40	Dhx37	NP_976064.1	1059	5	4.98
Ahr	NP_038492.1	1091	8	7.73	Bri3bp	NP_084028.1	106	7	69.60
Agr3	NP_997414.2	267	10	39.47	Aacs	NP_084486.1	794	2	2.65
Agr2	NP_035913.1	462	6	13.69	Tmem132b	NP_001177281.1	648	5	8.13
Tspan13	NP_079635.1	403	13	34.00	Gm4868	NP_001357804.1	154	0	0.00
Bzw2	NP_080116.2	340	9	27.90	Tmem132c	NP_780641.2	202	4	20.87
Ankmy2	NP_666145.3	815	12	15.52	Slc15a4	XP_006504335.1	501	4	8.41
Lrrc72	NP_001348027.1	593	1	1.78	Glt1d1	NP_795979.2	123	4	34.27
Sostdc1	NP_079588.1	366	6	17.28	Tmem132d	NP_766473.1	285	4	14.79
Crppa	XP_036013543.1	387	9	24.51	Fzd10	NP_780493.1	616	2	3.42
Meox2	NP_032610.1	608	13	22.53	Piwil1	NP_067286.1	788	1	1.34
Agmo	NP_848882.2	632	11	18.34	Rimbp2	NP_001365829.1	383	1	2.75
Dgkb	XP_006515130.1	1077	5	4.89	Stx2	NP_001272962.1	757	2	2.78
Etv1	XP_006515028.1	899	3	3.52	Ran	XP_006504343.1	1464	2	1.44
Arl4a	NP_031513.1	1519	7	4.86	Adgrd1	NP_001074811.1	403	0	0.00
Scin	NP_033158.2	591	1	1.78	Sfswap	NP_001365844.1	403	0	0.00
Lsmem1	NP_001028609.1	181	1	5.82	Mmp17	NP_035976.3	496	0	0.00
Ifrd1	NP_038590.2	489	9	19.40	Gm6139	XP_036021546.1	1326	3	2.38
Zfp277	NP_849173.2	181	7	40.76	Zfp11	NP_766050.1	225	0	0.00
Dock4	XP_006515148.1	1821	5	2.89	Septin14	NP_083102.1	289	8	29.17
Immp2l	NP_444352.2	672	6	9.41	Mrps17	NP_001343884.1	439	8	19.21
Lrrn3	NP_034863.1	1003	7	7.36	Nipsnap2	NP_032121.3	404	7	18.26
Dnajb9	NP_038788.2	739	4	5.70	Psph	XP_017176063.1	850	8	9.92
Pnpla8	NP_080440.2	598	2	3.52	Cct6a	NP_033968.2	1860	13	7.37
Nrcam	XP_006516024.1	1199	5	4.39	Sumf2	XP_036021338.1	160	12	79.04
Stxbp6	XP_036013201.1	737	7	10.01	Phkg1	NP_035209.1	396	10	26.61
Nova1	NP_001351564.1	795	5	6.63	Chchd2	NP_077128.2	431	10	24.45
Foxg1	NP_001153584.1	1340	3	2.36	Zbed5	NP_898911.2	144	2	14.64
Prkd1	NP_001369743.1	783	2	2.69	Nupr1l	NP_001346237.1	112	6	56.46
G2e3	NP_001161435.1	1221	1	0.86	Vkorc1l1	NP_081397.1	56	6	112.92
Scfd1	NP_084101.1	733	2	2.88	Gusb	NP_034498.1	1079	6	5.86
Coch	XP_017170432.1	427	1	2.47	Asl	NP_598529.1	666	1	1.58
Strn3	XP_036013599.1	922	4	4.57	Crcp	NP_031787.1	269	1	3.92
Ap4s1	NP_001316628.1	430	1	2.45	Tpst1	XP_036020889.1	868	3	3.64
Hectd1	XP_036013175.1	1038	0	0.00	Kctd7	XP_036020874.1	514	2	4.10
Heatr5a	NP_796145.2	260	4	16.21	Rabgef1	XP_006504509.1	1940	2	1.09
Dtd2	NP_001334333.1	193	3	16.38	Tmem248	NP_001074863.1	123	0	0.00
Gpr33	NP_032185.1	306	1	3.44	Sbds	NP_075737.1	677	1	1.56
Nubpl	NP_084036.2	622	1	1.69	Tyw1	XP_006504394.1	424	0	0.00
Arhgap5	XP_006515501.1	626	1	1.68	A330070K13F	NP_941067.1	1	0	0.00
Akap6	XP_017170544.1	777	4	5.43	Caln1	NP_851388.1	1469	7	5.02
Npas3	XP_006516004.1	603	4	6.99	Galnt17	NP_660253.2	286	4	14.74
Egln3	NP_082409.2	603	2	3.50	Auts2	NP_001350409.1	641	6	9.86
Sptssa	NP_598815.2	259	0	0.00	Castor2	NP_109644.2	217	4	19.43
Eapp	NP_001318091.1	295	1	3.57	Rcc1l	XP_006504600.1	722	9	13.14
Snx6	XP_006516316.1	456	5	11.56	Gtf2ird2	NP_444496.1	141	5	37.37
Cfl2	NP_031714.1	1460	0	0.00	Ncf1	NP_035006.3	1254	9	7.56
Baz1a	XP_006515734.1	1259	1	0.84	Gtf2i	XP_030110023.1	601	11	19.29

2700097O09R	NP_082590.2	51	0	0.00	Gtf2ird1	XP_017176536.1	352	11	32.93
Srp54a	NP_036029.2	1417	5	3.72	Clip2	NP_001034251.1	775	12	16.32
Fam177a	NP_082803.1	101	1	10.43	Syna	XP_030110216.1	185	0	0.00
Srp54b	NP_001093579.1	1417	4	2.98	Rfc2	NP_064406.1	1300	12	9.73
Fam177a2	NP_001093586.1	101	1	10.43	Lat2	NP_075253.2	267	4	15.79
Srp54c	NP_001363967.1	1462	5	3.60	Eif4h	NP_001299796.1	1646	11	7.04
Ppp2r3c	XP_030102688.1	651	5	8.09	Limk1	NP_034847.1	1495	13	9.16
Prorp	NP_079649.1	247	4	17.07	Eln	XP_011239157.1	1016	9	9.34
Psma6	NP_001297512.1	1634	2	1.29	Tmem270	NP_919041.1	175	3	18.07
Nfkbia	NP_035037.2	1983	4	2.13	Cldn13	NP_065250.1	251	4	16.80
Aldoart2	NP_001264269.1	796	0	0.00	Mettl27	XP_006504585.1	735	8	11.47
Insm2	NP_064683.2	225	1	4.68	Cldn4	NP_034033.1	613	12	20.63
Ralgapa1	XP_006516170.1	341	2	6.18	Cldn3	NP_034032.1	685	11	16.92
Brms1l	NP_001032845.1	407	1	2.59	Abhd11	NP_660250.1	382	9	24.83
Mbip	XP_006515736.1	197	3	16.05	Stx1a	NP_058081.2	1199	14	12.31
Nkx2-1	XP_006515854.1	842	6	7.51	Bud23	NP_001350254.1	1114	13	12.30
Nkx2-9	NP_032727.2	385	6	16.42	Dnajc30	NP_079638.2	225	6	28.10
Pax9	XP_006515642.1	553	6	11.43	Vps37d	NP_808242.1	195	2	10.81
Slc25a21	NP_766165.2	342	6	18.49	Mlxipl	XP_006504540.1	625	11	18.55
Prps1l3	NP_001032835.2	871	0	0.00	Tbl2	NP_038791.2	791	13	17.32
Mipol1	NP_001157842.1	82	5	64.26	Bcl7b	NP_033875.2	279	14	52.88
Foxa1	XP_017170451.1	1490	6	4.24	Baz1b	NP_035844.2	1343	11	8.63
Ttc6	XP_017170673.1	72	3	43.91	Fzd9	NP_034376.1	702	9	13.51
Sstr1	XP_006515697.1	887	3	3.56	Fkbp6	XP_030110810.1	1197	12	10.57
Clec14a	NP_080085.3	235	1	4.48	Trim50	NP_001333674.1	699	9	13.57
Sec23a	NP_001348885.1	1230	4	3.43	Nsun5	XP_036020581.1	930	9	10.20
Gemin2	NP_079932.2	385	1	2.74	Pom121	NP_683734.2	184	0	0.00
Trappc6b	NP_084333.1	409	5	12.88	Hip1	NP_666113.2	823	2	2.56
Pnn	NP_032917.2	714	3	4.43	Ccl26	NP_001013430.2	303	1	3.48
Mia2	NP_001315976.1	531	2	3.97	Ccl24	NP_001343559.1	445	1	2.37
Fbxo33	NP_001028328.2	243	3	13.01	Rhbdd2	NP_666114.1	210	1	5.02
Lrfn5	XP_017170545.1	1044	3	3.03	Por	XP_006504461.1	820	1	1.29
Fscb	NP_001156743.1	346	2	6.09	Tmem120a	NP_766129.1	174	0	0.00
Gm527	NP_001020776.1	28	3	112.92	Styxl1	XP_006504582.1	644	2	3.27
Klhl28	NP_079983.1	204	3	15.50	Mdh2	NP_032643.2	1516	3	2.09
Togaram1	XP_006516083.1	542	6	11.67	Srrm3	NP_067378.2	783	1	1.35
Prpf39	NP_808474.2	818	8	10.31	Hspb1	NP_038588.2	1450	3	2.18
Fkbp3	NP_038930.1	1445	7	5.11	Ywhag	NP_061359.2	3169	6	2.00
Fancm	NP_849243.2	986	3	3.21	Ssc4d	NP_001153838.1	30	0	0.00
Mis18bp1	NP_766166.2	428	6	14.77	Zp3	NP_035906.1	533	0	0.00
Wdr20rt	NP_081890.1	807	0	0.00	Dtx2	XP_030110708.1	357	1	2.95
Rpl10l	NP_001156405.1	1205	2	1.75	Upk3b	NP_780518.1	280	2	7.53
Mdga2	NP_996893.3	374	0	0.00	Upk3bl	XP_011239208.1	13	0	0.00
Rps29	NP_033119.1	1144	6	5.53	Rasa4	NP_598675.2	417	0	0.00
Lrr1	NP_001074875.1	1244	8	6.78	Polr2j	NP_035423.2	1394	2	1.51
Rpl36al	NP_079865.1	962	5	5.48	Lrwd1	NP_082167.2	595	3	5.31
Mgat2	NP_666147.1	193	0	0.00	Alkbh4	NP_001346343.1	155	1	6.80
Dnaaf2	XP_017170423.1	473	1	2.23	Orai2	XP_006504492.1	270	1	3.90
Pole2	NP_035263.1	905	5	5.82	Prkrip1	NP_080050.1	409	3	7.73
Klhdc1	NP_839984.1	519	7	14.21	Sh2b2	XP_030110354.1	250	2	8.43
Klhdc2	NP_081393.2	649	5	8.12	Cux1	NP_941004.2	1168	3	2.71
Nemf	NP_079717.2	541	5	9.74	Myl10	XP_017176539.1	1120	1	0.94
Arf6	NP_031507.1	1970	3	1.60	Col26a1	XP_006504425.1	207	2	10.18
Vcpkmt	NP_001028408.2	178	0	0.00	Ift22	NP_080349.1	366	2	5.76
Sos2	NP_001129031.1	1056	7	6.99	Fis1	NP_001334433.1	773	4	5.45
L2hgdh	NP_663418.1	427	2	4.94	Cldn15	NP_068365.1	207	1	5.09
Dmac2l	NP_080812.1	309	6	20.46	Znhit1	NP_081594.1	619	5	8.51
Cdkl1	XP_011242478.1	339	3	9.33	Plod3	NP_036092.1	420	2	5.02
Map4k5	XP_006516117.1	266	4	15.85	Vgf	XP_017176427.1	975	1	1.08
Atl1	NP_848743.1	610	3	5.18	Ap1s1	NP_031483.1	609	1	1.73
Sav1	NP_071311.1	368	2	5.73	Serpine1	NP_032897.2	1422	1	0.74
Nin	XP_036013121.1	445	3	7.10	Trim56	NP_958761.1	484	0	0.00
Abhd12b	XP_030102346.1	284	6	22.27	Muc3	XP_036021320.1	117	1	9.01
Pygl	NP_573461.2	1047	7	7.05	Muc3a	XP_036021549.1	88	1	11.98
Trim9	XP_030102869.1	1265	7	5.83	Ache	NP_033729.1	1157	2	1.82
Tmx1	NP_082615.1	921	1	1.14	Ufsp1	NP_081632.1	153	3	20.66

Frmd6	XP_036013387.1	469	4	8.99	Srrt	NP_001103380.1	971	4	4.34
Actr10	NP_062759.2	1802	4	2.34	Trip6	NP_035769.1	1103	8	7.64
Psma3	XP_017170485.1	1521	6	4.16	Slc12a9	NP_001346540.1	283	4	14.90
Arid4a	XP_036013289.1	1132	1	0.93	Ephb4	NP_001153043.1	1807	7	4.08
Tomm20l	NP_083503.1	493	2	4.28	Zan	XP_036020895.1	375	0	0.00
Timm9	XP_017170577.1	675	6	9.37	Epo	NP_001299804.1	1036	3	3.05
2700049A03R	XP_006516428.1	236	8	35.73	Pop7	NP_083029.1	543	1	1.94
Dact1	NP_001177395.1	532	3	5.94	Gigyf1	XP_006504672.1	444	7	16.62
Daam1	XP_030102496.1	1127	9	8.42	Gnb2	NP_034442.1	1646	6	3.84
Gpr135	NP_861417.1	244	5	21.60	Actl6b	XP_006504698.1	2737	5	1.93
L3hypdh	XP_011242463.1	71	2	29.69	Tfr2	XP_030110515.1	548	2	3.85
Jkamp	XP_030102354.1	182	5	28.95	Mospd3	NP_084313.1	654	2	3.22
Ccdc175	NP_082963.1	18	1	58.55	Pcolce	NP_032814.2	458	1	2.30
Rtn1	NP_001273377.1	1526	6	4.14	Fbxo24	NP_081984.1	420	6	15.06
Lrrc9	XP_006516440.1	576	2	3.66	Lrch4	NP_001162123.1	688	5	7.66
Pcnx4	NP_080603.2	40	0	0.00	Sap25	NP_001075431.2	18	0	0.00
Dhrs7	NP_079798.2	366	3	8.64	Irs3	NP_034701.1	375	4	11.24
Ppm1a	XP_036013148.1	1199	5	4.39	Agfg2	XP_006504633.1	499	10	21.12
4930447C04R	NP_083720.1	64	1	16.47	Nyap1	NP_001334434.1	337	13	40.65
Six6	NP_035514.1	664	3	4.76	Tsc22d4	NP_084081.1	186	7	39.66
Six1	NP_033215.2	899	2	2.34	Ppp1r35	XP_030110658.1	157	7	46.99
Six4	XP_006515695.1	445	1	2.37	Mepce	XP_017176362.1	371	8	22.73
Mnat1	NP_032638.2	523	0	0.00	Zcwpw1	XP_036021136.1	150	6	42.16
Trmt5	XP_036013547.1	399	3	7.92	Pilra	NP_705730.1	285	6	22.19
Slc38a6	XP_011242456.1	265	1	3.98	Pilrb1	NP_573472.2	274	7	26.92
Tmem30b	NP_848830.1	631	1	1.67	Pilrb2	NP_001371131.1	163	6	38.79
Prkch	NP_032882.2	1455	4	2.90	Cyp3a13	XP_030109981.1	635	1	1.66
Hif1a	NP_001300848.1	1681	2	1.25	Gjc3	NP_536698.2	216	1	4.88
Snapc1	NP_848479.1	270	5	19.52	Azgp1	NP_038506.2	790	3	4.00
Syt16	NP_766392.2	690	2	3.05	Gm4963	XP_036021553.1	1067	1	0.99
Dbpht2	NP_942566.1	139	0	0.00	Smok3a	NP_001119517.1	113	0	0.00
Kcnh5	NP_766393.2	603	3	5.24	Smok3b	NP_001034978.1	113	0	0.00
Rhoj	NP_075764.1	1810	5	2.91	Smok3c	XP_017176542.1	113	0	0.00
Gphb5	XP_017170513.1	176	0	0.00	Zkscan1	NP_598667.2	341	2	6.18
Ppp2r5e	XP_006515989.1	1017	5	5.18	Zscan21	NP_001038168.1	351	2	6.01
Wdr89	NP_082479.1	193	0	0.00	Zfp113	NP_062721.2	247	2	8.53
Sgpp1	NP_109675.1	310	4	13.60	Cops6	NP_036132.1	1252	2	1.68
Syne2	NP_001005510.2	1076	3	2.94	Mcm7	NP_032594.1	1850	2	1.14
Esr2	NP_034287.3	906	2	2.33	Ap4m1	XP_006504614.1	562	0	0.00
Tex21	XP_006516457.1	46	0	0.00	Taf6	NP_001343531.1	607	2	3.47
Mthfd1	NP_620084.2	942	1	1.12	Cnpy4	NP_848727.1	148	0	0.00
Akap5	XP_006515905.1	418	2	5.04	Mblac1	NP_808546.1	55	0	0.00
Zbtb25	XP_006515455.1	340	4	12.40	Nxpe5	XP_011239245.1	57	2	36.98
Zbtb1	NP_001351252.1	529	3	5.98	Lamtor4	NP_001074577.1	331	0	0.00
Hspa2	NP_032327.2	2123	3	1.49	Map11	NP_694801.2	127	1	8.30
Ppp1r36	NP_001156575.1	34	0	0.00	Gal3st4	NP_001334436.1	84	1	12.55
Plekhg3	XP_006515943.1	212	3	14.91	Gpc2	NP_766000.1	425	1	2.48
Sptb	NP_038703.3	941	4	4.48	Stag3	NP_058660.2	682	4	6.18
Churc1	NP_996257.1	279	1	3.78	Zfp157	NP_082406.2	208	0	0.00
Gpx2	NP_109602.2	710	2	2.97	1700123K08R	XP_011239262.1	109	0	0.00
Rab15	NP_598811.3	725	2	2.91	Zfp68	NP_001157269.1	303	0	0.00
Fntb	NP_666039.1	399	6	15.85	A430033K04R	NP_898846.2	134	0	0.00
Max	NP_001139648.1	488	2	4.32	Fam20c	NP_085042.2	413	0	0.00
Fut8	NP_058589.2	398	3	7.94	Foxl3	XP_006504780.1	353	0	0.00
Gphn	XP_006515964.1	848	3	3.73	Pdgfa	XP_030110058.1	743	0	0.00
Fam71d	XP_017170684.1	114	0	0.00	Prkar1b	NP_001346027.1	2010	4	2.10
Mpp5	XP_006516160.1	939	2	2.24	Dnaaf5	NP_001074734.1	214	0	0.00
Atp6v1d	NP_076210.1	718	2	2.94	Sun1	XP_017176645.1	674	1	1.56
Eif2s1	NP_080390.1	1557	3	2.03	Get4	NP_080545.2	438	1	2.41
Plek2	XP_006515997.1	221	5	23.84	Adap1	NP_766311.2	760	5	6.93
Tmem229b	NP_001351820.1	51	0	0.00	Cox19	NP_932097.1	412	4	10.23
Plekhh1	XP_036013196.1	191	2	11.04	Cyp2w1	NP_001153737.1	201	4	20.97
Pigh	NP_084264.1	161	0	0.00	3110082I17Ri	NP_082745.2	213	2	9.90
Arg2	NP_033835.1	865	2	2.44	Gpr146	NP_001349135.1	234	5	22.52
Vti1b	NP_058080.2	616	6	10.27	C130050O18R	XP_006504772.1	28	0	0.00
Rdh11	NP_067532.2	623	4	6.77	Gper1	XP_036021486.1	543	2	3.88

Rdh12	NP_084293.1	642	4	6.57	Zfand2a	NP_579927.1	266	1	3.96
Zfyve26	NP_001008550.1	310	7	23.80	Uncx	NP_038730.1	514	0	0.00
Rad51b	XP_017170492.1	1846	5	2.85	Micall2	NP_777275.2	498	0	0.00
Zfp36l1	NP_031590.1	875	1	1.20	Ints1	XP_006504792.1	467	0	0.00
Actn1	NP_001333598.1	1500	2	1.41	Mafk	XP_006504716.1	434	0	0.00
Dcaf5	NP_796241.3	170	8	49.60	Tmem184a	NP_001155020.1	220	0	0.00
Exd2	XP_011242527.1	352	6	17.96	Psmg3	NP_001343892.1	231	2	9.12
Galnt16	NP_001074890.1	566	3	5.59	Elfn1	XP_017176371.1	252	6	25.09
Erh	NP_031977.1	748	2	2.82	Mad1l1	XP_006504715.1	904	9	10.49
Slc39a9	XP_006516097.1	192	2	10.98	Mrm2	NP_080786.1	865	4	4.87
Plekhd1	NP_001170974.1	42	11	276.02	Nudt1	NP_001343514.1	834	2	2.53
Ccdc177	NP_001008423.2	153	7	48.22	Snx8	XP_030110296.1	516	2	4.08
Susd6	XP_036013204.1	93	5	56.66	Eif3b	NP_598677.1	1445	1	0.73
Srsf5	NP_001334345.1	1338	0	0.00	Chst12	NP_067503.3	256	8	32.93
Slc10a1	NP_001348901.1	798	0	0.00	Grifin	NP_084298.1	182	7	40.53
Smoc1	NP_001139689.1	392	4	10.75	Lfng	NP_032520.1	487	7	15.15
Slc8a3	XP_036013059.1	542	1	1.94	Ttyh3	NP_780483.2	205	10	51.41
Gm20498	NP_001296777.1	753	4	5.60	Iqce	XP_036021452.1	168	8	50.19
Cox16	NP_001296738.1	120	5	43.91	Brat1	XP_006504741.1	133	7	55.47
Gm4787	NP_001034084.2	52	0	0.00	Amz1	NP_775581.1	157	4	26.85
Adam4	NP_033750.1	72	0	0.00	Gna12	NP_034432.1	1259	0	0.00
Synj2bp	NP_079568.1	753	3	4.20	Card11	NP_780571.2	1172	1	0.90
Adam21	NP_065063.1	173	2	12.18	Sdk1	NP_808547.3	425	1	2.48
Med6	NP_001334313.1	440	0	0.00	Foxk1	NP_951031.2	846	2	2.49
Ttc9	NP_001028321.1	411	4	10.26	Ap5z1	NP_766313.2	205	3	15.42
Map3k9	NP_001167578.1	1242	6	5.09	Radil	NP_001297681.1	971	4	4.34
Pcnx	NP_061284.2	210	3	15.06	Papolb	NP_064327.1	877	4	4.81
Sipa1l1	XP_017170515.1	508	3	6.22	Mmd2	NP_780426.1	503	0	0.00
Rgs6	XP_011242435.1	581	3	5.44	Wipi2	NP_848485.1	563	0	0.00
Dpf3	NP_001254554.1	467	2	4.51	Slc29a4	NP_666369.1	300	0	0.00
Dcaf4	NP_001158728.1	123	0	0.00	Tnrc18	NP_001116202.2	288	1	3.66
Zfyve1	NP_898977.2	830	2	2.54	Fbxl18	NP_001028484.2	390	1	2.70
Rbm25	NP_001351344.1	1007	1	1.05	Actb	NP_031419.1	4533	5	1.16
Psen1	NP_001349200.1	1706	2	1.24	Fscn1	NP_032010.2	661	2	3.19
Papln	NP_001192272.1	631	1	1.67	Rnf216	XP_030109902.1	384	4	10.98
Numb	XP_006515638.2	1044	2	2.02	Rbak	NP_001038947.1	203	1	5.19
Heatr4	NP_001363868.1	63	0	0.00	Spdye4b	NP_001371122.1	19	0	0.00
Riox1	NP_076122.2	321	0	0.00	Spdye4a	XP_006504812.1	19	0	0.00
Acot2	NP_598949.3	377	3	8.39	Zfp12	XP_017176364.1	254	3	12.45
Acot1	NP_036136.1	378	3	8.36	Zfp316	NP_059495.3	291	0	0.00
Acot4	NP_599008.3	602	3	5.25	Zfp853	NP_001357755.1	246	1	4.28
Acot3	NP_599007.1	500	3	6.32	E130309D02F	NP_766314.3	34	0	0.00
Acot5	XP_006515756.4	229	4	18.41	Zdhhc4	XP_030110694.1	198	1	5.32
Acot6	NP_766168.1	182	0	0.00	Grid2ip	NP_001152793.1	1970	10	5.35
Dnal1	NP_083097.2	679	0	0.00	Kdelr2	NP_080117.1	708	5	7.44
Pnma1	NP_081714.2	133	0	0.00	Daglb	NP_659164.2	345	5	15.27
Mideas	XP_036013297.1	346	1	3.05	Rac1	NP_033033.1	2562	4	1.65
Ptgr2	NP_001239554.1	298	2	7.07	Fam220a	NP_080326.2	17	0	0.00
Zfp410	NP_659082.1	355	1	2.97	Cyth3	NP_035312.3	509	7	14.49
Fam161b	NP_001345212.1	53	4	79.54	Usp42	NP_084025.2	305	6	20.73
Coq6	NP_766170.2	815	2	2.59	Eif2ak1	NP_038585.2	977	2	2.16
Entpd5	XP_036013066.1	421	1	2.50	Ankrd61	NP_080008.1	969	6	6.53
Bbof1	XP_030102806.1	48	1	21.96	Aimp2	NP_666277.1	667	5	7.90
Rnf113a2	NP_079801.2	364	0	0.00	Pms2	NP_032912.2	966	8	8.73
Aldh6a1	XP_036013042.1	658	2	3.20	Ccz1	NP_808350.1	398	0	0.00
Lin52	XP_036013218.1	248	6	25.50	Ocm	NP_149028.2	1101	1	0.96
Vsx2	NP_001288356.1	567	1	1.86	Lmtk2	NP_001074578.1	262	1	4.02
Abcd4	XP_006515677.1	488	4	8.64	Bhlha15	NP_034930.1	367	0	0.00
Vrtn	NP_001162060.1	170	7	43.40	Tecpr1	NP_081686.1	215	1	4.90
Syndig1l	XP_036013448.1	156	4	27.02	Bri3	NP_001157181.1	143	0	0.00
Npc2	NP_075898.1	713	1	1.48	Baiap2l1	NP_080109.1	342	0	0.00
Isca2	XP_006516360.1	466	2	4.52	Nptx2	NP_058069.1	562	1	1.88
Ltbp2	XP_006515565.1	464	0	0.00	Tmem130	NP_808403.2	744	1	1.42
Arel1	XP_030102752.1	1005	9	9.44	Trrap	XP_006504844.1	2639	2	0.80
Fcf1	NP_082908.2	877	5	6.01	Smurf1	XP_036021480.1	1552	4	2.72
Ylpm1	NP_848140.3	251	6	25.19	Kpna7	NP_001013796.2	1043	1	1.01

Prox2	NP_001348168.1	273	8	30.88	Gm4871	NP_001094933.1	127	0	0.00
Dlst	NP_084501.1	799	3	3.96	1700018F24R	NP_081345.2	278	0	0.00
Rps6kl1	XP_030102568.1	216	4	19.52	Arpc1a	NP_062741.1	717	1	1.47
Pgf	NP_032853.1	572	2	3.68	Arpc1b	NP_075631.2	736	0	0.00
Eif2b2	NP_663420.1	568	3	5.57	Pdap1	NP_001028485.1	259	0	0.00
Mlh3	NP_780546.1	603	2	3.50	Bud31	NP_001297700.1	958	1	1.10
Acyp1	NP_001351289.1	275	0	0.00	Ptcd1	NP_598496.3	564	0	0.00
Zc2hc1c	XP_036013516.1	47	0	0.00	Cpsf4	NP_001361645.1	815	2	2.59
Nek9	NP_660120.2	1052	3	3.01	Atp5j2	NP_065607.1	609	1	1.73
Tmed10	NP_081051.1	784	2	2.69	Zkscan14	NP_075811.2	266	1	3.96
Fos	NP_034364.1	2751	7	2.68	Zkscan5	NP_057892.2	241	1	4.37
Jdp2	XP_011242520.1	618	4	6.82	Zfp655	XP_017176603.1	312	0	0.00
Batf	NP_058047.1	925	2	2.28	Zscan25	NP_001371133.1	293	0	0.00
Flvcr2	NP_663422.1	353	0	0.00	Cyp3a57	NP_001093650.1	348	1	3.03
Erg28	NP_067421.1	323	0	0.00	Cyp3a16	NP_031846.2	571	1	1.85
Ttll5	XP_006516058.1	445	3	7.10	Cyp3a41b	XP_017176060.1	563	1	1.87
Tgfb3	NP_033394.2	977	6	6.47	Cyp3a41a	NP_059092.2	563	1	1.87
Ift43	NP_001186772.1	271	3	11.67	Cyp3a44	NP_796354.1	570	1	1.85
Gpatch2l	NP_081681.2	300	3	10.54	Cyp3a11	NP_031844.1	893	7	8.26
Esrrb	NP_001152972.1	968	2	2.18	Cyp3a25	NP_062766.2	813	1	1.30
Vash1	NP_796328.2	229	3	13.81	Cyp3a59	NP_001098630.1	684	1	1.54
Angel1	XP_011242471.1	287	4	14.69	Gm5565	NP_001361655.1	119	0	0.00
Lrrc74a	XP_017170639.1	86	0	0.00	Gm6309	XP_003688866.1	152	3	20.80
Irf2bpl	NP_665835.1	361	1	2.92	1700001J03R	XP_030110651.1	109	0	0.00
Cipc	XP_036013225.1	115	0	0.00	Rnf6	NP_001243015.1	703	0	0.00
Zdhhc22	NP_001074412.1	498	1	2.12	Cdk8	NP_705827.2	1046	0	0.00
Tmem63c	NP_001348633.1	535	5	9.85	Wasf3	NP_660137.1	571	2	3.69
Ngb	NP_071859.1	500	4	8.43	Gm6408	NP_001230033.1	119	1	8.86
Pomt2	NP_700464.2	449	6	14.08	Gm6370	NP_001361660.1	262	3	12.07
Gstz1	NP_034493.1	897	5	5.87	4930449I24Ri	NP_080412.1	318	5	16.57
Tmed8	XP_036013407.1	172	8	49.02	Gm3402	XP_011239298.1	119	4	35.43
Samd15	NP_001277217.1	61	5	86.39	Gm3404	XP_011239299.1	264	4	15.97
Noxred1	NP_082020.1	369	10	28.56	Gm3409	XP_006504843.1	128	4	32.93
Vipas39	NP_001136053.1	169	4	24.94	Gm3415	XP_011239300.1	121	3	26.13
Ahsa1	NP_666148.1	663	2	3.18	Gpr12	NP_001345986.1	461	1	2.29
Ism2	NP_001277231.1	88	3	35.93	Usp12	NP_035799.1	597	1	1.77
Sptlc2	NP_035609.1	663	2	3.18	Rpl21	NP_062621.2	899	0	0.00
Alkbh1	NP_001096035.1	446	1	2.36	Rasl11a	NP_081140.1	1371	7	5.38
Nrp	NP_001013390.1	446	1	2.36	Gtf3a	NP_079928.2	607	2	3.47
Slirp	NP_081234.2	1694	4	2.49	Mtif3	NP_001346396.1	623	3	5.07
Snw1	NP_079783.2	1126	2	1.87	Lnx2	NP_542985.4	765	3	4.13
Gm8300	NP_001171036.1	523	8	16.12	Polr1d	XP_030110088.1	1277	4	3.30
Oog1	NP_848772.3	258	7	28.59	Gsx1	NP_032204.1	351	4	12.01
Gm4027	NP_001171035.1	523	11	22.17	Pdx1	NP_032840.1	479	4	8.80
Gm16381	NP_001159534.1	569	10	18.52	Cdx2	NP_031699.2	1074	7	6.87
Gm21319	NP_001257651.1	436	4	9.67	Urad	NP_001034767.1	690	5	7.64
BB287469	NP_001171044.1	509	11	22.78	Flt3	NP_034359.2	1263	5	4.17
Gm2001	XP_003945542.1	642	1	1.64	Pan3	XP_006504931.1	431	1	2.45
Gm2016	NP_001116134.1	506	10	20.83	Flt1	NP_034358.2	1530	8	5.51
Gm2022	NP_001171045.1	509	11	22.78	Pomp	NP_079900.1	759	3	4.17
Gm2042	NP_001257721.1	208	8	40.53	Slc46a3	XP_006504930.1	264	0	0.00
Gm2046	XP_036013609.1	524	11	22.12	Mtus2	NP_001346432.1	163	1	6.47
Gm6803	NP_001341505.1	529	9	17.93	Slc7a1	NP_031539.3	657	2	3.21
Gm5955	XP_006516498.1	569	9	16.67	Ubl3	NP_001346128.1	356	0	0.00
Pramel51	NP_001098724.1	210	6	30.11	Katnal1	NP_705800.1	929	7	7.94
Gm5662	NP_001013846.1	518	7	14.24	Hmgb1	NP_034569.1	1694	3	1.87
Adck1	NP_082381.1	252	0	0.00	Uspl1	NP_001108625.1	247	4	17.07
Nrxn3	XP_036013122.1	1405	1	0.75	Alox5ap	NP_033793.1	621	5	8.49
Dio2	NP_034180.2	483	3	6.55	Medag	NP_081795.1	163	4	25.86
Cep128	XP_030102825.1	212	6	29.83	Tex26	NP_083740.1	24	0	0.00
Tshr	NP_035778.3	593	4	7.11	Wdr95	XP_017176434.1	313	0	0.00
Gtf2a1	NP_113568.2	458	3	6.90	Hsph1	XP_006504979.1	2061	4	2.05
Ston2	NP_780576.1	491	6	12.88	B3glct	XP_030110487.1	448	6	14.11
Sel1l	NP_035474.1	790	3	4.00	Rxfp2	NP_001276495.1	962	6	6.57
Flrt2	XP_036013410.1	340	2	6.20	Fry	XP_011239326.1	421	3	7.91
Galc	NP_032105.2	411	12	30.77	Zar1l	NP_001153165.1	183	3	19.20

Gpr65	NP_032178.1	701	11	16.54	Brca2	NP_001074470.1	1394	5	4.45
Kcnk10	NP_084187.2	373	11	31.08	N4bp2l1	NP_598659.3	212	0	0.00
Spata7	NP_849245.2	228	12	55.47	N4bp2l2	NP_001349345.1	321	1	4.38
Ptpn21	NP_036007.2	625	13	21.92	Pds5b	XP_006504964.1	1022	3	4.42
Zc3h14	XP_006516390.3	797	14	18.51	Kl	NP_038851.2	594	0	0.00
Eml5	XP_006516035.1	845	14	17.46	Stard13	XP_006504988.1	427	2	8.23
Ttc8	NP_938053.1	849	14	17.38	Vmn2r18	NP_001372143.1	25	0	0.00
Foxn3	XP_030102789.1	869	18	21.83	Rfc3	NP_081285.1	1337	2	3.15
Efcab11	NP_084448.1	1108	10	9.51	Samd9l	NP_034286.2	543	5	19.41
Tdp1	XP_036013044.1	562	15	28.13	Hepacam2	XP_006505021.1	183	5	52.35
Kcnk13	XP_030102525.1	193	17	92.83	Vps50	NP_077222.4	391	4	17.97
Psmc1	NP_032973.1	1648	18	11.51	Calcr	NP_001036190.1	604	11	29.53
Nrde2	NP_898978.2	280	4	15.06	Tfpi2	XP_011239358.1	1091	13	17.94
Calm1	NP_033920.1	2215	10	4.76	Gngt1	XP_017176877.1	1418	3	2.97
Ttc7b	NP_001028385.1	327	19	61.24	Gng11	NP_079607.1	932	5	7.07
Rps6ka5	XP_006516338.1	548	15	28.85	Bet1	NP_033878.1	479	5	12.94
Dglucy	NP_001347946.1	67	2	31.46	Col1a2	NP_031769.2	1073	4	4.37
Gpr68	XP_017170551.1	394	14	37.45	Casd1	NP_663373.2	171	3	19.46
Ccdc88c	NP_080957.2	302	15	52.35	Sgce	XP_036021868.1	389	9	24.38
Ppp4r3a	NP_997594.1	621	18	30.55	Peg10	NP_570947.2	402	9	23.59
Catsperb	NP_766611.2	201	17	89.14	Ppp1r9a	XP_006505133.1	561	11	20.66
Tc2n	XP_017170706.1	264	18	71.86	Pon1	XP_006505074.1	874	5	6.03
Fbln5	XP_006515929.1	734	8	11.49	Pon3	NP_766594.1	350	9	27.10
Trip11	XP_006515445.1	508	14	29.04	Pon2	NP_899131.1	377	8	22.36
Atxn3	XP_030102382.1	863	6	7.33	Asb4	NP_001342591.1	1519	12	8.33
Cpsf2	XP_006516134.1	1003	13	13.66	Pdk4	NP_038771.1	1311	3	2.41
Slc24a4	NP_742164.2	545	12	23.21	Dync1i1	XP_030110998.1	1362	10	7.74
Rin3	NP_808288.2	549	12	23.04	Slc25a13	NP_001171043.1	548	10	19.23
Lgmn	NP_001365804.1	703	10	14.99	Sem1	NP_033195.1	826	6	7.66
Golga5	NP_001185933.1	357	12	35.43	Dlx6	NP_034187.1	428	6	14.77
Chga	NP_031719.1	1142	4	3.69	Dlx5	NP_034186.2	797	8	10.58
Itpk1	NP_766172.1	304	14	48.53	Sdhaf3	NP_001071181.1	248	0	0.00
Moap1	NP_071718.1	166	7	44.44	Tac1	XP_006505090.1	1255	2	1.68
Gm20604	NP_001136411.1	0	0	#DIV/0!	Asns	NP_036185.1	1062	3	2.98
Tmem251	NP_796114.1	24	2	87.83	C1galt1	NP_443719.3	518	3	6.10
Gon7	NP_001136410.1	31	0	0.00	Col28a1	XP_036021904.1	702	5	7.51
Ubr7	NP_079942.1	292	1	3.61	Mios	XP_036022022.1	234	1	4.50
Btbd7	NP_001363955.1	172	6	36.76	Rpa3	NP_080908.1	855	2	2.47
Cox8c	NP_001034138.1	361	6	17.52	Umad1	NP_001289282.1	2	0	0.00
Unc79	XP_036013240.1	627	3	5.04	Glcci1	NP_573499.2	211	1	4.99
Prima1	XP_006515569.1	203	6	31.15	Ica1	XP_030111053.1	495	3	6.39
Fam181a	NP_001182655.1	105	7	70.26	Nxph1	NP_032777.3	751	2	2.81
Asb2	NP_075536.1	1602	6	3.95	Ndufa4	NP_035016.1	724	3	4.37
Otub2	NP_080856.1	370	6	17.09	Phf14	XP_030111516.1	543	1	1.94
Ddx24	NP_001152974.1	1519	7	4.86	Thsd7a	NP_001158277.1	241	1	4.37
Ifi27l2a	NP_001268759.1	238	2	8.86	Tmem106b	NP_082268.2	328	3	9.64
Ifi27	NP_001351102.1	212	6	29.83	Vwde	NP_001355800.1	316	0	0.00
Ifi27l2b	NP_663424.1	254	6	24.90	Tmem168	NP_083266.1	83	7	88.88
Ppp4r4	XP_006516371.1	783	5	6.73	Bmt2	NP_780521.2	336	7	21.96
Serpina10	NP_001288333.1	651	8	12.95	Gpr85	XP_017177194.1	459	8	18.37
Serpina6	NP_031644.1	819	7	9.01	2610001J05R	NP_001339816.1	2	0	0.00
Serpina16	XP_112098.3	85	0	0.00	Ppp1r3a	NP_536712.2	503	7	14.67
Serpina1f	NP_001158214.1	219	5	24.06	Foxp2	XP_036021645.1	1244	8	6.78
Serpina1b	NP_033270.3	1058	7	6.97	Mdfic	NP_780297.3	328	12	38.56
Serpina1d	NP_033272.1	1302	7	5.67	Tfec	XP_011239355.1	576	7	12.81
Serpina1a	NP_001239498.1	1031	14	14.31	Tes	NP_997059.1	769	8	10.96
Serpina1c	NP_033271.1	1330	7	5.55	Cav2	NP_058596.1	637	11	18.20
Serpina1e	NP_033273.1	1012	7	7.29	Cav1	XP_006505037.1	1835	8	4.59
Serpina11	XP_006516101.1	660	6	9.58	Met	NP_032617.2	2477	10	4.25
Serpina9	NP_001348841.1	186	1	5.67	Capza2	NP_031630.1	845	17	21.20
Serpina12	NP_080811.1	605	10	17.42	St7	NP_001276553.1	134	9	70.78
Serpina5	NP_766541.2	1005	11	11.54	Wnt2	XP_006505108.1	1227	15	12.88
Serpina3a	NP_083016.1	670	4	6.29	Asz1	NP_076218.3	1253	17	14.30
Serpina3b	XP_006515994.1	681	3	4.64	Cftr	NP_066388.1	1683	9	5.64
Serpina3c	XP_011242304.1	715	14	20.64	Cttnbp2	XP_006505167.1	1613	13	8.49
Serpina3f	NP_001161767.1	723	2	2.92	Lsm8	NP_598700.1	987	1	1.07

Serpina3g	NP_001340541.1	647	1	1.63	Ankrd7	XP_006505254.1	1017	1	1.04
Serpina3i	XP_017170641.1	676	1	1.56	Kcnd2	NP_062671.1	1618	12	7.82
Serpina3j	XP_006515922.1	294	1	3.58	Tspan12	XP_036022088.1	264	11	43.91
Serpina3k	NP_035588.2	846	3	3.74	Ing3	NP_076115.3	675	9	14.05
Serpina3m	XP_006515700.1	1153	2	1.83	Cped1	NP_001074820.1	134	9	70.78
Serpina3n	NP_033278.2	951	1	1.11	Wnt16	NP_444346.3	663	6	9.54
Gsc	NP_034481.1	346	0	0.00	Fam3c	NP_613053.3	352	8	23.95
Dicer1	XP_036013153.1	3749	1	0.28	Ptprz1	XP_006505075.1	1282	7	5.75
Clmn	NP_001035772.1	602	0	0.00	Aass	NP_038958.2	886	1	1.19
Syne3	NP_001036164.1	117	0	0.00	Fezf1	NP_082738.1	656	8	12.85
Glrx5	NP_082695.1	854	1	1.23	Cadps2	NP_001239035.1	721	12	17.54
Tcl1b2	NP_038803.1	222	1	4.75	Rnf133	NP_937894.1	229	4	18.41
Tcl1b1	NP_038801.1	296	1	3.56	Rnf148	NP_082030.1	248	4	17.00
Tcl1b5	NP_001348836.1	226	1	4.66	Tas2r118	NP_996905.1	444	5	11.87
Tcl1b3	NP_038800.1	277	1	3.80	Slc13a1	NP_062354.2	481	5	10.96
Tcl1b4	NP_001348835.1	262	1	4.02	Iqub	NP_766123.2	206	5	25.58
Tcl1	NP_001276397.1	490	6	12.90	Ndufa5	NP_001297997.1	718	4	5.87
D430019H16R	NP_001239437.1	77	0	0.00	Asb15	XP_006505269.1	1389	6	4.55
Bdkrb2	XP_036013065.1	642	1	1.64	Lmod2	NP_444328.1	432	2	4.88
Bdkrb1	NP_031565.1	559	3	5.66	Wasl	NP_082735.2	1230	2	1.71
Atg2b	NP_083930.5	412	2	5.12	Hyal6	NP_083196.2	686	3	4.61
Gskip	NP_848728.2	230	1	4.58	Hyal4	NP_084124.1	262	1	4.02
Ak7	NP_084463.1	839	2	2.51	Spam1	NP_001073344.1	446	2	4.73
Papola	NP_035242.1	1083	2	1.95	Hyal5	XP_006505250.1	227	1	4.64
Vrk1	NP_001351296.1	1263	4	3.34	Tmem229a	NP_795987.2	48	0	0.00
Bcl11b	NP_001273272.1	1054	3	3.00	Gpr37	NP_034468.2	782	1	1.35
Setd3	XP_017170622.1	423	1	2.49	Pot1a	NP_598692.1	301	0	0.00
Ccnk	NP_033962.2	760	1	1.39	Grm8	XP_030111034.1	873	6	7.24
Ccdc85c	XP_006516224.1	156	3	20.27	Zfp800	XP_006505206.1	450	2	4.68
Hhipl1	NP_001037845.1	162	3	19.52	Gcc1	NP_083176.3	288	6	21.96
Cyp46a1	NP_034140.1	709	3	4.46	Arf5	NP_031506.1	1719	6	3.68
Eml1	XP_017170657.1	926	7	7.97	Fscn3	NP_062515.2	162	7	45.54
Evl	NP_001156866.1	513	1	2.05	Pax4	NP_001153398.1	740	5	7.12
Degs2	NP_001164473.1	308	5	17.11	Snd1	XP_006505205.1	1099	6	5.75
Yy1	NP_033563.2	1465	10	7.19	Lrrc4	NP_619623.2	963	5	5.47
Slc25a29	NP_851845.1	303	10	34.78	Lep	NP_032519.1	1780	4	2.37
Slc25a47	XP_011242283.1	527	5	10.00	Rbm28	NP_598686.2	821	5	6.42
Wars	NP_035840.3	1049	6	6.03	Prrt4	NP_001094913.1	145	3	21.80
Wdr25	NP_808270.2	653	10	16.14	Impdh1	XP_006505129.2	1263	4	3.34
Begain	NP_001156647.1	552	13	24.82	Hilpda	NP_001177390.1	165	0	0.00
Dlk1	NP_034182.2	932	7	7.92	Fam71f2	XP_011239362.1	18	0	0.00
Rtl1	XP_030102657.1	995	8	8.47	Fam71f1	XP_017177140.1	103	2	20.46
Dio3	NP_742117.2	402	9	23.59	Calu	NP_031620.1	967	7	7.63
Gm6988	XP_036013670.1	1067	1	0.99	Opn1sw	NP_031564.1	427	2	4.94
Ppp2r5c	XP_006515987.1	1051	11	11.03	Ccdc136	XP_006505111.1	225	3	14.05
Dync1h1	NP_084514.2	1466	7	5.03	Flnc	XP_036008175.1	1458	3	2.17
1700001K19R	XP_030102712.1	80	0	0.00	Atp6v1f	NP_079657.1	851	6	7.43
Hsp90aa1	NP_034610.1	4223	6	1.50	Atp6v1fnb	NP_001138832.1	0	0	#DIV/0!
Wdr20	XP_006516267.1	803	1	1.31	Kcp	XP_011239372.1	286	1	3.68
Mok	XP_036013309.1	325	2	6.49	Irf5	XP_006505158.1	731	4	5.77
Zfp839	NP_082641.1	5	0	0.00	Tnpo3	NP_796270.2	629	6	10.05
Cinp	NP_080324.1	157	1	6.71	Tspan33	NP_666285.1	236	5	22.33
Tecpr2	NP_001074526.1	251	0	0.00	Smo	NP_795970.3	993	4	4.25
Ankrd9	NP_001348845.1	1187	5	4.44	Ahcyl2	XP_011239384.1	905	1	1.16
Rcor1	NP_932140.2	614	1	1.72	Strip2	NP_796178.2	364	2	5.79
Traf3	NP_035762.2	978	4	4.31	Nrf1	NP_001348622.1	711	0	0.00
Amn	NP_291081.2	182	2	11.58	Ube2h	NP_001163047.1	901	2	2.34
Cdc42bpb	NP_898837.2	1292	6	4.89	Zc3hc1	NP_001298015.1	270	3	11.71
Lbhd2	NP_001345885.1	38	0	0.00	Klhdc10	NP_084018.1	544	9	17.44
Exoc3l4	NP_083083.3	534	2	3.95	Tmem209	XP_006505235.1	307	7	24.03
Tnfaip2	NP_033422.2	919	4	4.59	Ssmem1	NP_081349.1	63	7	117.10
Gm266	NP_001028420.2	1238	2	1.70	Cpa2	NP_001019869.1	468	6	13.51
Eif5	NP_829887.1	1068	3	2.96	Cpa4	XP_017177239.1	485	8	17.38
Mark3	XP_006515574.1	343	0	0.00	Cpa5	XP_017177284.1	353	9	26.87
Ckb	NP_067248.1	808	2	2.61	Cpa1	NP_079626.2	555	6	11.39
Trmt61a	NP_796348.2	676	1	1.56	Cep41	XP_006505273.1	395	5	13.34

Bag5	NP_001311411.1	322	2	6.55	Mest	XP_036021793.1	356	6	17.76
Coa8	NP_001156860.1	135	1	7.81	Copg2	NP_059506.1	953	11	12.16
Klc1	NP_001020531.2	1218	5	4.33	Tsga13	XP_036021648.1	79	12	160.09
Xrcc3	XP_006516363.1	1449	0	0.00	Klf14	NP_001128565.1	499	6	12.67
Zfyve21	NP_001361650.1	115	2	18.33	Mkln1	XP_036022093.1	256	8	32.93
Ppp1r13b	NP_035755.1	404	0	0.00	Podxl	NP_038751.2	541	0	0.00
Atp5mpl	NP_001348843.1	314	0	0.00	1700012A03R	NP_083863.1	264	0	0.00
Tdrd9	NP_083332.1	1134	0	0.00	Plxna4	NP_786926.2	701	2	3.01
Rd3l	NP_001121157.1	31	3	101.99	Chchd3	NP_079612.1	424	3	7.46
Aspg	NP_001074638.1	958	3	3.30	Exoc4	NP_033174.2	852	1	1.24
Kif26a	XP_006516225.1	932	8	9.05	Lrguk	NP_083162.1	1798	6	3.52
A530016L24R	NP_796013.1	52	0	0.00	Slc35b4	NP_067410.1	262	0	0.00
Tmem179	NP_849246.2	732	1	1.44	Akr1b3	NP_033788.3	864	2	2.44
Inf2	NP_001361128.1	1053	9	9.01	Akr1b8	NP_032038.1	617	3	5.12
Adssl1	NP_031447.1	893	7	8.26	Akr1b10	NP_765986.3	591	2	3.57
Siva1	NP_038957.2	454	11	25.54	Akr1b7	NP_033861.2	661	3	4.78
Akt1	NP_001159366.1	5891	9	1.61	Bpgm	NP_031589.1	621	4	6.79
Zbtb42	NP_001093930.1	170	3	18.60	Cald1	XP_006505411.1	663	0	0.00
Cep170b	NP_001019773.2	150	2	14.05	Agbl3	NP_001276585.1	353	3	8.96
Pld4	NP_849242.1	591	10	17.83	Tmem140	NP_001355294.1	121	0	0.00
Ahnak2	NP_001365427.1	98	9	96.79	Cyren	XP_030111536.1	79	0	0.00
Clba1	NP_663425.2	20	0	0.00	Wdr91	NP_001013384.1	155	4	27.20
Cdca4	NP_001346163.1	333	9	28.48	Stra8	NP_033318.1	484	2	4.35
Gpr132	XP_006516165.1	613	8	13.75	Slc23a4	XP_006506197.1	115	0	0.00
Jag2	XP_006515549.1	1309	7	5.64	Cnot4	NP_001157884.1	922	2	2.29
Nudt14	NP_001303653.1	592	15	26.70	Nup205	NP_081789.1	571	2	3.69
Brf1	XP_006516324.1	514	1	2.05	Slc13a4	NP_766480.1	364	5	14.48
Btbd6	NP_964008.2	530	5	9.94	Fam180a	NP_775551.1	70	4	60.22
Pacs2	XP_006515832.1	464	10	22.71	Mtpn	NP_032124.1	1148	3	2.75
Tex22	NP_001348849.1	76	5	69.34	Chrm2	XP_030111253.1	838	1	1.26
Mta1	XP_006515481.1	456	4	9.24	Ptn	XP_006505820.3	1000	2	2.11
Crip2	NP_077185.1	646	7	11.42	Dgki	XP_006506322.1	654	3	4.83
Crip1	NP_031789.1	704	6	8.98	Creb3l2	NP_848776.2	450	2	4.68
Tedc1	NP_598802.2	143	0	0.00	Akr1d1	XP_017176968.1	687	2	3.07
Tmem121	NP_722471.2	83	6	76.19	Trim24	NP_659542.3	869	3	3.64
Adam6b	NP_001009545.1	96	1	10.98	Svopl	NP_796174.2	323	4	13.05
Adam6a	NP_777479.2	336	4	12.55	Atp6v0a4	NP_536715.3	811	2	2.60
Zfp386	NP_001004066.1	343	2	6.15	Tmem213	NP_084197.1	184	7	40.09
Vipr2	XP_011242404.1	436	4	9.67	D630045J12R	NP_918950.2	205	3	15.42
Wdr60	NP_666151.3	284	5	18.55	Zc3hav1l	NP_766055.1	74	6	85.45
Esyt2	XP_017170618.1	314	4	13.43	Zc3hav1	XP_006506843.1	425	4	9.92
Ncapg2	XP_036013545.1	753	3	4.20	Ttc26	NP_705828.2	307	9	30.90
Ptprn2	NP_035345.2	1456	7	5.07	Ubn2	NP_796159.3	434	8	19.43
Gm6750	XP_036013671.1	195	0	0.00	Fmc1	NP_079639.1	275	9	34.49
Rapgef5	NP_787126.3	447	8	20.96	Luc7l2	XP_036021821.1	953	13	14.38
Cdca7l	XP_036013267.1	388	11	35.15	Klrg2	XP_006506774.1	228	8	36.98
Dnah11	NP_034190.3	802	9	14.78	Clec2l	NP_001094977.1	754	6	8.39
Sp4	XP_036013171.1	463	7	21.24	Hipk2	NP_001281073.1	1326	5	3.97
Sp8	NP_001366308.1	749	7	14.07	Tbxas1	XP_017176988.1	588	4	7.17
Abcb5	NP_084237.1	680	8	19.08	Parp12	NP_766481.2	455	6	13.90
Itgb8	NP_796264.2	844	9	18.73	Kdm7a	NP_001028602.2	774	3	4.08
Macc1	XP_006515924.1	162	8	94.63	Slc37a3	NP_001348548.1	321	2	6.57
Tmem196	NP_001281075.1	264	9	71.86	Rab19	NP_035356.1	781	3	4.05
Gdi2	NP_032138.3	1309	3	4.83	Mkrn1	XP_017177172.1	549	5	9.60
Tasor2	NP_001347767.1	209	0	0.00	Dennd2a	XP_017176974.1	147	0	0.00
Asb13	NP_840068.1	1261	2	2.79	Adck2	NP_849204.1	234	8	36.03
Calml3	NP_081692.1	1168	2	2.78	Ndufb2	NP_001345726.1	567	3	5.58
Calm4	NP_064420.2	1156	2	2.60	Braf	XP_036021643.1	1696	3	1.86
Calm5	NP_001008706.1	1025	3	4.11	Mrps33	NP_034400.1	586	5	8.99
Net1	NP_062645.2	592	3	6.68	Tmem178b	NP_001347907.1	458	3	6.90
Tubal3	NP_001029051.2	1372	6	5.42	Agk	NP_076027.1	598	6	10.57
Ucn3	NP_112540.2	267	4	17.54	Dennd11	NP_001343304.1	116	0	0.00
Akr1c14	XP_030102973.1	700	1	1.58	Wee2	XP_006506390.1	1209	0	0.00
Akr1c18	NP_001333464.1	370	0	0.00	Ssbp1	NP_001351507.1	1085	4	3.89
Akr1c13	NP_038806.2	420	1	2.51	Tas2r137	NP_001020556.1	144	3	21.96
Akr1c19	NP_001013807.2	619	1	1.70	Tas2r108	NP_065248.1	395	5	13.34

Akr1c12	NP_038805.2	580	1	1.82	Prss37	NP_080593.1	363	3	8.71
Akr1c6	NP_085114.1	1013	5	5.20	Olfr461	NP_666494.1	66	3	47.90
Akr1c20	XP_030102990.1	803	1	1.31	Olfr460	NP_666495.1	30	1	35.13
Akr1c21	NP_084177.2	212	0	0.00	Clec5a	NP_067339.1	617	1	1.71
Akr1e1	NP_061347.2	225	2	9.37	Tas2r138	NP_001001451.1	390	1	2.70
Klf6	NP_035933.2	890	0	0.00	Mgam	NP_001355804.1	1343	3	2.35
Pitrm1	NP_660113.1	764	1	1.38	Moxd2	NP_647457.2	187	4	22.54
Pfkp	XP_006516550.1	1208	5	4.36	Prss58	NP_778185.1	378	4	11.15
Gm10029	XP_036014159.1	1002	2	2.10	1700074P13R	XP_030111490.1	438	2	4.81
Adarb2	XP_017171136.1	461	4	9.14	1810009J06R	NP_076196.1	354	0	0.00
Wdr37	XP_036013848.1	886	7	8.33	Gm2663	NP_001096130.1	337	0	0.00
Idi1	NP_663335.2	512	1	2.06	2210010C04R	NP_075822.3	373	6	16.95
Idi2	NP_796171.1	265	5	19.88	Try4	NP_035776.1	522	6	12.11
Gtpbp4	XP_017171081.1	1363	8	6.19	Try5	NP_001003405.1	499	8	16.90
Larp4b	XP_036013869.1	390	9	24.32	Try10	NP_001034085.1	497	3	6.36
Dip2c	XP_006516536.1	482	4	8.75	Prss3	NP_035775.1	605	4	6.97
Zmynd11	NP_001334402.1	466	3	6.78	Gm5771	NP_001034086.1	478	7	15.43
Chrm3	NP_150372.1	851	4	4.95	Gm10334	NP_001096623.1	489	7	15.09
Ryr2	XP_017170950.1	1138	2	1.85	Prss1	NP_444473.1	517	3	6.12
Mtr	NP_001074597.1	478	0	0.00	Prss2	NP_033456.1	559	4	7.54
Actn2	NP_150371.4	1491	3	2.12	Ephb6	XP_030111004.1	1162	5	4.53
Gm5445	XP_036014184.1	899	1	1.17	Trpv6	NP_071858.3	525	2	4.01
Heatr1	NP_659084.3	821	3	3.85	Trpv5	XP_017176953.1	484	3	6.53
Lgals8	XP_017171036.1	379	2	5.56	Llcfc1	NP_001368881.1	111	1	9.49
Edaradd	XP_006516627.1	487	4	8.66	Kel	XP_006506122.1	271	4	15.56
Ero1b	XP_006516794.1	424	2	4.97	Olfr459	NP_666787.1	33	0	0.00
Prl2c3	XP_006516642.1	82	1	12.85	Pip	NP_032869.2	173	1	6.09
Prl2c2	NP_112468.1	130	1	8.11	Sval2	NP_115931.1	96	0	0.00
Prl2c5	XP_011242563.1	97	1	10.86	Sval1	NP_082108.1	49	0	0.00
Gm2423	XP_036014168.1	2829	1	0.37	Sval3	NP_001003952.1	98	0	0.00
Gm7040	XP_006516887.1	234	3	13.51	Sva	NP_033325.2	243	0	0.00
Gpr137b	NP_114388.2	294	6	21.51	Tas2r139	NP_851792.1	270	1	3.90
Nid1	NP_035047.2	841	3	3.76	Tas2r144	NP_001001453.1	432	1	2.44
Lyst	XP_006516624.1	644	4	6.55	Gstk1	NP_083831.1	524	0	0.00
Gng4	NP_034447.1	948	4	4.45	Tmem139	NP_780617.2	46	1	22.91
B3galnt2	NP_001349333.1	165	0	0.00	Casp2	NP_031636.1	1011	1	1.04
Tbce	XP_006516836.1	383	1	2.75	Clcn1	NP_038519.1	506	1	2.08
Ggps1	XP_017170876.1	644	5	8.18	Fam131b	NP_001106798.1	540	1	1.95
Arid4b	NP_919238.1	1256	2	1.68	Zyx	XP_006505999.1	967	1	1.09
Hecw1	XP_006516879.1	1431	0	0.00	Epha1	NP_076069.2	1592	2	1.32
Mrpl32	NP_083547.1	601	8	14.03	Tas2r143	NP_001001452.1	97	4	43.46
Psma2	NP_032970.2	1164	7	6.34	Tas2r135	NP_954610.1	306	1	3.44
AW209491	NP_598828.3	84	2	25.09	Tas2r126	NP_996911.1	328	1	3.21
Gli3	NP_032156.2	1348	4	3.13	Olfr458	NP_666655.1	18	0	0.00
Inhba	XP_006516622.1	864	3	3.66	Olfr457	NP_667198.1	18	0	0.00
Sugct	XP_036013805.1	434	1	2.43	Olfr456	NP_001011528.2	20	0	0.00
Mplkip	NP_079755.1	136	3	23.25	Olfr455	NP_001074770.2	29	1	36.34
Cdk13	NP_081394.1	902	4	4.67	Tcaf3	NP_981933.1	82	0	0.00
Rala	NP_062364.3	1706	3	1.85	Tcaf2	XP_036021992.1	99	0	0.00
Yae1d1	NP_080180.1	98	0	0.00	Tcaf1	NP_001342697.1	62	0	0.00
Pou6f2	XP_036013874.1	350	2	6.02	Olfr453	NP_001011799.1	65	0	0.00
Vps41	NP_742118.3	519	0	0.00	Olfr38	NP_667197.1	30	0	0.00
Amph	NP_001276475.1	1684	4	2.50	Olfr452	NP_001011869.1	31	0	0.00
Stard3nl	XP_036014109.1	113	7	65.29	Olfr450	NP_666656.1	83	0	0.00
Epdr1	NP_598826.3	238	6	26.57	Olfr449	NP_001343339.1	41	0	0.00
Sfrp4	NP_057896.1	596	5	8.84	Olfr448	NP_666385.1	68	0	0.00
Nme8	NP_001161381.1	3032	10	3.48	Olfr447	NP_667199.1	157	0	0.00
Gpr141b	NP_783619.2	57	4	73.96	Olfr446	NP_666407.1	287	0	0.00
Gpr141	XP_036013972.1	175	5	30.11	Olfr444	NP_666867.1	111	0	0.00
Elmo1	XP_006516607.1	612	6	10.33	Olfr441	NP_666866.1	37	0	0.00
Aoah	XP_011242611.1	299	1	3.52	Olfr237	NP_666865.1	63	0	0.00
Olfr1370	NP_666746.1	47	0	0.00	Olfr437	NP_666408.1	107	0	0.00
Olfr263	NP_035114.1	83	1	12.70	Olfr13	NP_666863.1	45	0	0.00
Olfr1368	NP_666745.1	11	0	0.00	Olfr435	NP_666864.1	79	0	0.00
Trim27	NP_033080.2	665	1	1.58	Olfr434	NP_666481.1	31	0	0.00
Gpx5	NP_034473.2	720	1	1.46	Olfr47	NP_666482.1	68	0	0.00

Gpx6	XP_011242657.1	1102	1	0.96	Arhgef5	NP_598435.1	551	1	1.91
Olfr1367	NP_666744.1	87	0	0.00	Nobox	XP_030111080.1	633	1	1.66
Zscan12	NP_057893.2	219	2	9.62	Tpk1	XP_006506281.1	363	2	5.81
Zkscan3	NP_076174.3	359	1	2.94	Cntnap2	NP_001004357.2	1015	1	1.04
Pgbd1	XP_036013960.1	343	6	18.44	Cul1	NP_001342479.1	2298	2	0.92
Zscan26	NP_001013808.2	273	2	7.72	Ezh2	XP_036021720.1	2885	9	3.29
Nkapl	NP_079995.2	295	3	10.72	Pdia4	NP_033917.2	1504	0	0.00
Zkscan4	NP_001034204.1	296	3	10.68	Zfp786	XP_030111337.1	162	1	6.51
Zkscan8	XP_030103336.1	172	1	6.13	Zfp398	NP_081753.1	240	1	4.39
Olfr1366	NP_666395.2	13	0	0.00	Zfp282	XP_011239401.1	235	2	8.97
Olfr1535	NP_997455.1	33	0	0.00	Zfp212	NP_663551.2	248	2	8.50
Olfr1364	NP_666751.2	24	0	0.00	Zfp956	NP_849229.2	170	5	31.00
Olfr1362	NP_666955.2	74	0	0.00	Zfp777	XP_006506738.2	261	3	12.11
Olfr11	NP_666753.2	91	0	0.00	Zfp746	XP_036008183.1	253	2	8.33
Olfr1361	XP_006516742.1	44	0	0.00	Krba1	XP_006506820.1	60	5	87.83
Olfr1360	NP_666754.2	124	1	8.50	Zfp467	XP_006506611.1	296	3	10.68
Olfr1359	NP_001011820.1	47	1	22.42	Sspo	XP_017177069.1	1342	4	3.14
H2bc13	NP_835506.1	1265	8	6.66	Atp6v0e2	NP_001334093.1	325	4	12.97
H2ac13	NP_835489.1	754	9	12.58	Lrrc61	NP_808404.2	621	4	6.79
H3c10	NP_835513.1	1551	11	7.47	Rarres2	NP_001334097.1	490	1	2.15
H2bc14	NP_835507.1	1191	9	7.96	Gm5111	NP_899132.1	0	0	#DIV/0!
H4c11	NP_835582.1	1933	12	6.54	Repin1	NP_001073372.2	334	8	25.24
H4c12	NP_835583.1	1933	12	6.54	Zfp775	XP_036022003.1	208	7	35.47
H2ac15	NP_835490.1	858	13	15.97	Gimap8	NP_001070878.1	216	3	14.64
H2bc15	NP_835508.1	951	13	14.41	Gimap9	NP_777620.1	60	8	140.52
H1f5	NP_064418.1	901	17	19.88	Gimap4	XP_036021614.1	231	4	18.25
H3c11	NP_835514.1	1551	16	10.87	Gimap6	NP_694815.1	185	4	22.79
H2ac22	NP_835491.1	916	14	16.11	Gimap7	NP_666279.1	323	3	9.79
H2bc22	NP_835509.2	891	14	16.56	Gimap1	NP_787056.1	108	3	29.28
H2bc23	NP_001091448.2	1263	13	10.85	Gimap5	NP_778200.1	267	3	11.84
H2ac23	NP_001171015.1	916	14	16.11	Gimap3	NP_112537.2	309	2	6.82
H4c17	NP_001182350.1	1933	13	7.09	Tmem176b	XP_017177199.1	292	11	39.70
H4c18	NP_783588.1	1933	14	7.63	Tmem176a	NP_079602.4	217	3	14.57
H2ac24	NP_835492.1	770	15	20.53	Gm7932	NP_001344932.1	0	0	#DIV/0!
H2bc24	NP_001104025.1	1266	13	10.82	Aoc1	XP_030111522.1	270	5	19.52
Zfp184	NP_898835.1	283	2	7.45	Doxl1	NP_001074742.2	187	2	11.27
Pom121l2	NP_001156401.1	92	2	22.91	Doxl2	NP_001025158.1	197	2	10.70
Prss16	NP_062302.1	217	5	24.28	Svs1	NP_766476.2	321	3	9.85
H2ac12	NP_783590.1	965	9	9.83	Gpnmb	XP_036008305.1	596	1	1.77
H2bc12	NP_783596.1	1171	8	7.20	Malsu1	XP_006506784.1	496	2	4.25
H4c9	NP_783587.1	1933	7	3.82	Igf2bp3	NP_076159.3	515	1	2.05
H2ac11	NP_835493.1	917	7	8.05	Tra2a	XP_030110906.1	1624	2	1.30
H2bc11	NP_835505.1	1266	6	4.99	Ccdc126	NP_780307.1	136	3	23.25
Vmn1r188	NP_665849.1	85	0	0.00	Fam221a	XP_006506001.1	86	3	36.76
Vmn1r189	NP_665843.1	26	0	0.00	Stk31	NP_084192.2	269	2	7.84
Vmn1r191	NP_665848.1	220	0	0.00	Npy	NP_075945.1	1235	1	0.85
Vmn1r192	NP_665844.1	77	0	0.00	Mpp6	XP_030111378.1	780	0	0.00
Vmn1r193	NP_598986.1	60	0	0.00	Gsdme	XP_036008131.1	230	4	18.33
Vmn1r194	NP_001074441.1	36	0	0.00	Osbpl3	XP_006506698.1	420	3	7.53
Vmn1r195	NP_598984.2	0	0	#DIV/0!	Cycs	NP_031834.1	1408	0	0.00
Vmn1r196	NP_001161013.1	0	0	#DIV/0!	4921507P07R	XP_036008196.1	71	0	0.00
Vmn1r197	NP_599005.1	0	0	#DIV/0!	Npvf	NP_068692.1	200	2	10.54
Vmn1r198	NP_598981.1	38	0	0.00	Nfe2l3	NP_035033.1	403	6	15.69
Vmn1r199	NP_598974.1	80	0	0.00	Hnrnpa2b1	XP_030111360.1	2038	4	2.07
Vmn1r200	NP_598973.1	1	0	0.00	Cbx3	NP_031650.3	1117	2	1.89
Vmn1r201	NP_598982.1	89	0	0.00	Snx10	NP_001348510.1	515	3	6.14
Vmn1r202	NP_598985.1	0	0	#DIV/0!	Skap2	NP_061243.1	420	2	5.02
Vmn1r203	NP_598997.1	0	0	#DIV/0!	Hoxa1	NP_034579.3	407	11	28.48
Vmn1r204	NP_001039009.1	0	0	#DIV/0!	Hoxa2	NP_034581.1	576	12	21.96
Vmn1r205	NP_598978.1	244	0	0.00	Hoxa3	XP_017176891.1	506	12	24.99
Vmn1r206	NP_598977.1	63	0	0.00	Hoxa4	NP_032291.1	367	12	34.46
Vmn1r207	NP_001160181.1	50	0	0.00	Hoxa5	NP_034583.1	704	11	16.47
Vmn1r208	NP_598979.1	35	0	0.00	Hoxa6	NP_034584.1	308	11	37.64
Vmn1r209	NP_001013809.1	50	0	0.00	Hoxa7	NP_034585.1	525	13	26.10
Vmn1r210	NP_598996.1	0	0	#DIV/0!	Hoxa9	NP_001264167.1	764	12	16.55
Vmn1r211	NP_599004.1	0	0	#DIV/0!	Hoxa10	NP_001116422.1	621	14	23.76

Vmn1r212	XP_017170892.1	0	0	#DIV/0!	Hoxa11	NP_034580.1	511	15	30.94
Vmn1r213	NP_598976.1	0	0	#DIV/0!	Hoxa13	NP_032290.1	424	15	37.28
Vmn1r214	NP_598975.1	26	0	0.00	Evx1	NP_031992.1	491	14	30.05
Vmn1r215	NP_598980.1	118	0	0.00	Hibadh	NP_663542.1	732	9	12.96
Vmn1r216	NP_599006.1	125	0	0.00	Tax1bp1	NP_001342525.1	493	9	19.24
Vmn1r217	NP_599000.1	53	0	0.00	Jazf1	NP_775582.2	709	10	14.86
Vmn1r218	NP_598983.1	199	0	0.00	Creb5	XP_036021932.1	759	6	8.33
Vmn1r219	NP_598999.1	50	0	0.00	Tril	NP_080093.1	684	1	1.54
Vmn1r220	NP_598998.1	57	0	0.00	Cpvl	XP_006506682.1	910	2	2.32
Vmn1r221	NP_001161014.1	26	0	0.00	Chn2	NP_001157112.1	658	3	4.81
Vmn1r222	NP_599001.1	53	0	0.00	Prr15	XP_036008292.1	132	0	0.00
Vmn1r223	NP_001076780.1	0	0	#DIV/0!	Wipf3	XP_030111340.1	530	0	0.00
Zfp322a	NP_001104577.1	445	1	2.37	Scrn1	XP_036008185.1	566	1	1.86
Abt1	NP_038952.1	653	1	1.61	Fkbp14	NP_705801.1	981	6	6.45
Btn1a1	XP_006516598.1	270	0	0.00	Plekha8	NP_001157833.1	319	7	23.13
Btn2a2	NP_001276543.1	236	0	0.00	Mturn	NP_001276670.1	121	6	52.26
H4c8	NP_694813.1	1933	9	4.91	Znrf2	NP_954594.1	464	6	13.63
H2ac10	NP_783592.1	903	9	10.50	Nod1	XP_006505341.1	722	0	0.00
H3c8	NP_659539.1	1548	9	6.13	Ggct	NP_080913.1	397	6	15.93
H2bc9	NP_835504.1	927	11	12.51	Gars	NP_851009.2	1259	10	8.37
H3c7	NP_038576.1	1795	11	6.46	Crhr2	NP_034083.2	555	11	20.89
H4c6	NP_783586.1	1933	13	7.09	Inmt	NP_033375.1	853	3	3.71
H1f3	NP_663759.3	887	16	19.01	Mindy4	NP_001136253.1	231	3	13.69
H3c6	NP_835512.1	1494	14	9.88	Aqp1	NP_031498.1	1266	8	6.66
H2ac8	NP_835494.1	916	15	17.26	Ghrhr	NP_001003685.2	422	4	9.99
H2bc8	NP_835503.1	987	15	16.02	Adcyap1r1	XP_006505451.1	812	5	6.49
H2bc7	NP_835502.1	1267	16	13.31	Neurod6	NP_033847.1	773	7	9.54
H2ac7	NP_835495.1	765	17	23.42	Itprid1	XP_006506009.1	95	0	0.00
H3c4	NP_835511.1	1440	16	11.71	Ppp1r17	NP_035283.1	147	1	7.17
H4c4	NP_783585.1	1933	18	9.81	Pde1c	XP_006505793.1	496	7	14.87
H2bc6	NP_001171124.1	987	16	17.08	Lsm5	NP_079796.1	1717	3	1.84
H1f4	NP_056602.1	823	20	25.61	Avl9	NP_084511.1	196	2	10.75
H2ac6	NP_835496.1	916	17	19.56	Kbtbd2	NP_001349139.1	328	4	12.85
H2bc4	NP_001277309.1	950	16	17.75	Fkbp9	NP_036186.2	1101	2	1.91
H1f6	NP_034507.2	776	19	25.80	Nt5c3	NP_080280.3	417	3	7.58
H4c3	NP_835515.1	1933	17	9.27	Vmn1r4	XP_036021791.1	10	0	0.00
Hfe	NP_001334422.1	1780	20	11.84	Vmn1r5	NP_598935.2	0	0	#DIV/0!
H1f2	NP_056601.1	870	18	21.80	Vmn1r6	NP_598936.1	55	0	0.00
H3c3	NP_783584.1	1451	15	10.89	Vmn1r7	NP_001160182.1	52	0	0.00
H2bc3	NP_783595.1	1113	15	14.20	Vmn1r8	NP_598948.1	111	0	0.00
H2ac4	NP_783591.2	958	15	16.50	Vmn1r9	NP_598946.2	49	0	0.00
H3c2	NP_835510.1	1473	14	10.02	Vmn1r10	NP_444461.2	72	0	0.00
H4c2	NP_835500.1	1933	14	7.63	Vmn1r11	NP_444463.2	42	0	0.00
H4c1	NP_835499.1	1933	13	7.09	Vmn1r12	NP_001095049.1	90	0	0.00
H3c1	NP_038578.2	1549	12	8.16	Vmn1r13	NP_444465.2	7	0	0.00
H1f1	NP_085112.1	903	13	15.17	Vmn1r14	XP_017176828.1	26	0	0.00
Trim38	XP_006516700.1	414	15	38.18	Vmn1r15	NP_444466.2	33	0	0.00
Slc17a2	XP_006516709.1	680	0	0.00	Vmn1r16	XP_017176915.1	0	0	#DIV/0!
Slc17a3	XP_017170838.1	657	1	1.60	Vmn1r17	NP_598932.1	105	0	0.00
Slc17a1	XP_006516679.1	624	3	5.07	Vmn1r18	NP_598942.1	1	0	0.00
Slc17a4	XP_006516757.1	596	2	3.54	Vmn1r19	NP_598943.1	58	0	0.00
H2bc1	NP_783594.1	1136	7	6.49	Vmn1r20	NP_001095003.1	63	0	0.00
H2ac1	NP_783589.1	1426	7	5.17	Ppm1k	NP_780732.1	1205	1	0.87
Scgn	NP_663374.1	1254	4	3.36	Herc6	NP_080268.1	1138	2	1.85
Carmil1	XP_036014041.1	328	6	19.28	Pyurf	NP_079850.1	300	1	3.51
Cmah	XP_011242567.1	323	4	13.05	Lancl2	NP_598498.1	257	1	4.10
Ripor2	XP_006516650.1	330	9	28.74	Vopp1	NP_666280.1	192	1	5.49
Gmnn	NP_065592.1	1127	6	5.61	Vmn1r21	NP_598944.1	0	0	#DIV/0!
BC005537	NP_077793.1	72	8	117.10	Vmn1r22	NP_598939.1	20	0	0.00
Acot13	NP_080066.1	550	10	19.16	Vmn1r23	NP_598940.1	102	0	0.00
Tdp2	NP_062424.1	586	9	16.19	Vmn1r24	NP_598934.1	0	0	#DIV/0!
D130043K22R	NP_001074520.1	452	10	23.32	Vmn1r25	NP_444468.2	85	0	0.00
Aldh5a1	NP_766120.1	816	5	6.46	Vmn1r26	NP_598933.1	29	0	0.00
Gpld1	NP_032182.2	625	8	13.49	Vmn1r27	NP_602318.2	86	0	0.00
Mrs2	XP_006516770.2	389	9	24.38	Vmn1r28	NP_598941.1	1	0	0.00
Dcdc2a	XP_011242593.1	392	11	29.57	Vmn1r29	NP_444462.1	31	0	0.00

Nrsn1	XP_017170992.1	1001	10	10.53	Vmn1r30	XP_017176912.1	1	0	0.00
Hdgfl1	NP_032258.2	958	2	2.20	Vmn1r31	NP_001160201.1	55	0	0.00
Prl	NP_035294.2	745	11	15.56	Abcg2	NP_001342406.1	1091	2	1.93
Prl3d1	NP_001192251.1	132	2	15.97	Herc3	NP_001348895.1	1270	4	3.32
Prl3d2	NP_001371016.1	94	1	11.21	Nap1l5	NP_067407.1	1653	4	2.55
Prl3d3	NP_001348026.1	78	1	13.51	Fam13a	NP_705802.1	373	5	14.13
Prl3c1	NP_038794.1	62	1	17.00	Tigd2	NP_001074614.1	183	4	23.04
Prl3b1	NP_032891.1	175	1	6.02	Gprin3	XP_006506147.2	203	6	31.15
Prl3a1	NP_080172.1	88	1	11.98	Snca	NP_001035916.1	1714	5	3.07
Prl6a1	NP_035296.1	82	1	12.85	Mmrn1	NP_081889.1	354	5	14.89
Prl8a2	NP_034218.1	125	1	8.43	Ccser1	NP_899133.2	230	5	22.91
Prl2b1	NP_079808.1	94	1	11.21	Grid2	NP_001357895.1	788	4	5.35
Prl8a6	NP_035297.1	93	0	0.00	Atoh1	NP_031526.1	829	0	0.00
Prl8a8	NP_076230.1	85	0	0.00	Smarcad1	NP_001342179.1	866	1	1.22
Prl8a9	NP_001303650.1	84	0	0.00	Hpgds	NP_062328.3	1693	1	0.62
Prl8a1	NP_082753.1	61	0	0.00	Qrfprl	NP_780733.2	202	0	0.00
Prl7b1	NP_083631.1	108	0	0.00	Tnip3	NP_001001495.2	292	0	0.00
Prl7a1	NP_032956.1	82	0	0.00	Ndnf	NP_765987.2	167	0	0.00
Prl7a2	XP_006516644.1	77	0	0.00	Prdm5	XP_006506667.1	535	0	0.00
Prl7d1	NP_001347019.1	87	0	0.00	4930544G11F	NP_001155245.1	1985	0	0.00
Prl7c1	XP_006516796.1	87	0	0.00	Mad2l1	NP_001342553.1	1704	1	0.62
Prl2a1	NP_064375.1	103	0	0.00	Vmn1r32	NP_598931.1	0	0	#DIV/0!
Prl2c1	NP_001347974.1	72	0	0.00	Vmn1r33	NP_598930.1	31	0	0.00
Prl4a1	NP_035295.1	137	0	0.00	Vmn1r34	NP_001160191.1	42	0	0.00
Prl5a1	NP_076235.1	121	0	0.00	Vmn1r35	NP_598928.1	0	0	#DIV/0!
Sox4	NP_033264.2	973	4	4.33	Vmn1r36	NP_598927.1	0	0	#DIV/0!
Cdkal1	NP_653119.1	728	3	4.34	Vmn1r37	NP_598926.1	124	0	0.00
E2f3	XP_030102996.1	1080	4	3.90	Vmn1r38	NP_598929.1	0	0	#DIV/0!
Mboat1	NP_705774.1	352	3	8.98	Vmn1r39	NP_001160192.1	34	0	0.00
Agtr1a	NP_796296.1	1052	0	0.00	Gng12	NP_079554.1	859	0	0.00
Uqcrfs1	NP_079986.1	1311	0	0.00	Gadd45a	NP_031862.1	975	2	2.16
Dusp22	NP_598829.1	894	6	7.07	Serbp1	NP_080090.2	1226	1	0.86
Irf4	NP_038702.1	961	9	9.87	Il12rb2	XP_006505680.1	458	1	2.30
Exoc2	XP_036014008.1	836	10	12.61	Il23r	NP_653131.2	539	1	1.96
Hus1b	NP_694712.1	247	6	25.60	Tacstd2	NP_064431.2	391	1	2.70
Foxq1	NP_032265.3	507	7	14.55	Rpia	NP_033101.2	944	5	5.58
Foxf2	NP_034355.2	770	8	10.95	Eif2ak3	NP_034251.2	885	6	7.15
Foxc1	NP_032618.2	1104	13	12.41	Tex37	XP_030111497.1	302	4	13.96
Gmds	XP_006516719.1	559	9	16.97	Foxi3	NP_001094934.1	579	7	12.74
Mylk4	NP_001355809.1	748	9	12.68	Rpl34-ps1	NP_001186279.1	918	1	1.15
Wrnip1	NP_084491.3	561	0	0.00	Thnsl2	NP_848500.3	440	7	16.77
Serpinb1a	NP_079705.2	1120	5	4.70	Fabp1	NP_059095.1	1167	4	3.61
Serpinb1c	NP_001371017.1	1081	3	2.92	Smyd1	NP_033892.2	1219	8	6.92
Serpinb6b	NP_035584.1	1214	4	3.47	Krcc1	NP_001341635.1	102	5	51.66
Serpinb9	XP_006516689.1	1109	2	1.90	Gm1070	NP_001344834.1	444	1	2.37
Serpinb9b	NP_035582.1	1023	1	1.03	Cd8b1	NP_033988.1	412	2	5.12
Serpinb1b	XP_006516749.1	1087	1	0.97	Cd8a	NP_001074579.1	443	1	2.38
Serpinb9c	NP_001157996.1	1021	1	1.03	Rmnd5a	NP_001342674.1	424	0	0.00
Serpinb9d	NP_035590.1	1040	1	1.01	Rnf103	NP_033569.2	405	2	5.20
Serpinb9e	NP_035586.1	1019	1	1.03	Chmp3	NP_001348334.1	446	1	2.36
Serpinb9f	NP_899020.1	1018	0	0.00	Kdm3a	NP_001349129.1	674	1	1.56
Serpinb9h	NP_001357856.1	77	0	0.00	Reep1	XP_030111355.1	1387	2	1.52
Serpinb9g	NP_035585.2	1017	0	0.00	Mrpl35	NP_079706.1	328	2	6.43
Serpinb6d	NP_001070258.1	339	1	3.11	Immt	NP_083949.2	1006	0	0.00
Serpinb6e	NP_001039000.2	1181	1	0.89	Ptcd3	NP_001348998.1	500	1	2.11
Serpinb6c	XP_036014126.1	1203	1	0.88	Polr1a	NP_033114.3	2273	3	1.39
Serpinb6a	NP_001157589.1	1230	3	2.57	St3gal5	XP_006505879.1	348	2	6.06
Nqo2	XP_036013797.1	241	0	0.00	Atoh8	NP_722473.1	325	0	0.00
Ripk1	NP_001346926.1	1365	0	0.00	Sftpb	NP_680088.1	373	3	8.48
Bphl	XP_006516799.1	316	1	3.34	Usp39	XP_006506279.1	1034	10	10.19
Tubb2a	NP_033476.1	1782	3	1.77	0610030E20R	XP_006506597.1	53	6	119.31
Tubb2b	NP_076205.1	1679	3	1.88	Tmem150a	NP_659165.1	97	13	141.24
Psmg4	NP_001094900.1	218	3	14.50	Rnf181	NP_001318100.1	350	13	39.14
Slc22a23	NP_001369778.1	192	8	43.91	Vamp5	NP_001074211.1	834	10	12.64
Pxdc1	NP_080107.3	68	2	31.00	Vamp8	NP_058074.2	893	11	12.98
Fam50b	NP_001369785.1	140	2	15.06	Ggcx	NP_062776.1	248	9	38.25

Prpf4b	NP_038858.2	975	3	3.24	Mat2a	NP_663544.1	1274	9	7.45
Fam217a	XP_006516848.1	30	0	0.00	Sh2d6	XP_011239846.2	76	13	180.27
4933417A18R	NP_080026.2	65	2	32.43	Capg	XP_017176859.1	564	4	7.47
Eci3	XP_011242645.1	937	7	7.87	Elmod3	NP_001351483.1	131	12	96.54
Eci2	NP_001103801.1	1136	4	3.71	Retsat	NP_080435.3	476	12	26.57
Cdyl	NP_034011.1	2215	10	4.76	Tgoln1	NP_033469.1	861	6	7.34
Rpp40	NP_666050.3	233	11	49.75	Tcf7l1	NP_033358.2	770	4	5.47
Ppp1r3g	NP_083904.1	178	11	65.13	Kcmf1	NP_062689.2	456	0	0.00
Lyrm4	NP_958746.1	306	9	31.00	Tmsb10	NP_079560.1	296	0	0.00
Fars2	XP_030103262.1	623	14	23.68	Dnah6	NP_001158141.1	638	0	0.00
Nrn1	NP_705757.1	482	8	17.49	Suclg1	NP_063932.2	1429	1	0.74
F13a1	NP_001159863.1	640	9	14.82	4931417E11R	NP_080013.1	231	0	0.00
Ly86	NP_034875.1	885	12	14.29	Ctnna2	NP_001342122.1	1337	2	1.58
Rreb1	NP_001171340.1	633	12	19.98	Lrrtm1	NP_001349038.1	1380	2	1.53
Ssr1	NP_080241.3	518	17	34.59	Gm5576	XP_036008337.1	1988	2	1.06
Cage1	XP_017171093.1	183	17	97.90	Reg3b	NP_035166.1	352	1	2.99
Riok1	NP_077204.2	698	15	22.65	Reg3d	NP_001155213.1	205	5	25.70
Dsp	NP_076331.2	1153	1	0.91	Reg3a	NP_035389.1	284	1	3.71
Snrnp48	NP_080658.2	137	15	115.39	Reg2	NP_033069.1	221	1	4.77
Bmp6	NP_031582.1	852	4	4.95	Reg1	NP_033068.1	272	1	3.87
Txndc5	NP_663342.3	1185	13	11.56	Reg3g	NP_035390.1	373	1	2.83
Bloc1s5	NP_620702.1	203	12	62.30	Gm20594	NP_001177661.1	54	0	0.00
Eef1e1	NP_079656.1	1227	10	8.59	Lrrtm4	NP_001128215.1	964	2	2.19
Slc35b3	XP_006516590.1	283	17	63.31	Gcfc2	NP_808552.2	389	2	5.42
Ofcc1	XP_036013880.1	137	16	123.08	Mrpl19	NP_080766.1	811	3	3.90
Tfap2a	XP_006516965.1	851	9	11.15	Eva1a	XP_030111196.1	275	2	7.66
Gcnt2	NP_076376.3	364	11	31.85	Tacr1	NP_033339.2	684	2	3.08
Pak1ip1	NP_080826.2	891	11	13.01	Pole4	NP_001357224.1	677	1	1.56
Tmem14c	NP_079663.1	339	11	34.20	Hk2	NP_038848.1	1083	0	0.00
Mak	NP_001139274.1	484	4	8.71	Sema4f	NP_035480.3	330	5	15.97
Gcm2	NP_032130.2	486	12	26.02	M1ap	NP_149070.2	91	4	46.33
Sycp2l	NP_001311454.1	135	14	109.29	Dok1	NP_034200.4	405	8	20.82
Elovl2	NP_062296.1	779	13	17.59	Loxl3	NP_038614.2	359	9	26.42
Smim13	NP_001129049.1	32	14	461.08	Htra2	NP_062726.3	862	6	7.34
Nedd9	NP_001104794.1	749	5	7.04	Aup1	NP_031543.2	661	9	14.35
Tmem170b	NP_001157044.1	58	10	181.71	Dqx1	XP_006506873.1	1053	10	10.01
Adtrp	NP_780626.1	307	11	37.76	Tlx2	NP_033418.1	375	8	22.48
Hivep1	XP_017170847.1	567	8	14.87	Pcgf1	NP_932109.1	480	8	17.57
Edn1	NP_034234.1	1539	1	0.68	Lbx2	NP_034822.1	247	4	17.07
Phactr1	XP_006516968.1	801	1	1.32	Ccdc142	NP_001074735.1	4	0	0.00
Tbc1d7	XP_036014025.1	318	0	0.00	Mrpl53	NP_081020.1	593	2	3.55
Gfod1	NP_001028571.1	344	1	3.06	Mogs	NP_065644.2	547	0	0.00
Sirt5	XP_030103247.1	1232	3	2.57	Wbp1	NP_001077392.1	113	0	0.00
Nol7	NP_076043.2	373	0	0.00	Ino80b	NP_076036.2	314	2	6.71
Ranbp9	NP_064314.2	604	0	0.00	Rtkn	NP_001129699.1	357	0	0.00
Mcur1	NP_001074528.1	113	0	0.00	Wdr54	NP_076279.1	26	0	0.00
Rnf182	NP_899027.1	603	1	1.75	1700003E16R	NP_082224.1	249	0	0.00
Cd83	NP_033986.1	705	0	0.00	Dctn1	NP_031861.2	1375	3	2.30
Jarid2	NP_068678.1	2360	3	1.34	Slc4a5	XP_006506027.1	335	2	6.29
Dtnbp1	NP_080048.2	566	3	5.59	Mthfd2	XP_006505760.1	1014	0	0.00
Mylip	NP_722484.2	624	3	5.07	Mob1a	NP_663546.1	520	0	0.00
Gmpr	XP_030103212.1	1123	4	3.75	Bola3	NP_001350702.1	515	0	0.00
Atxn1	XP_017170952.1	596	5	8.84	Tet3	XP_006505840.1	500	1	2.11
Stmnd1	NP_001005422.1	3	2	702.60	Dguok	NP_038792.2	499	0	0.00
Rbm24	NP_001074894.1	725	4	5.81	Actg2	NP_033740.2	2136	2	0.99
Cap2	XP_006517009.1	1273	1	0.83	Stambp	NP_001349007.1	1107	1	0.95
Fam8a1	NP_001028364.1	138	6	45.82	Clec4f	NP_058031.2	718	1	1.47
Nup153	NP_786925.2	1038	6	6.09	Cd207	XP_006506202.1	632	1	1.67
Kif13a	NP_034747.2	920	7	8.02	Vax2	NP_036042.1	308	2	6.84
Nhlrc1	NP_780549.1	518	5	10.17	Atp6v1b1	NP_598918.1	790	3	4.00
Tpmt	XP_011242698.1	346	7	21.32	Ankrd53	NP_083521.2	981	3	3.22
Kdm1b	XP_006516976.1	693	6	9.12	Tex261	NP_033383.1	96	0	0.00
Dek	XP_036013726.1	1055	7	6.99	Nagk	NP_001334244.1	325	0	0.00
Rnf144b	NP_666154.3	629	0	0.00	Paip2b	NP_666281.2	160	0	0.00
Id4	NP_112443.1	557	3	5.68	Zfp638	XP_006505767.1	486	0	0.00
Zfp169	NP_001158048.1	188	1	5.61	Dysf	XP_006506227.1	668	4	6.31

Ptpdc1	NP_997115.1	767	5	6.87	Cyp26b1	XP_030111198.1	604	4	6.98
Barx1	NP_031552.2	376	0	0.00	Exoc6b	XP_006506791.1	700	4	6.02
Phf2	NP_035208.2	462	0	0.00	Spr	NP_035597.2	476	0	0.00
Fam120a	NP_001028440.2	236	0	0.00	Emx1	NP_034261.1	609	7	12.11
Wnk2	XP_036014104.1	414	1	2.55	Sfxn5	XP_036008307.1	218	2	9.67
Ninj1	NP_038638.1	177	0	0.00	Rab11fip5	NP_803417.3	413	4	10.21
Card19	XP_006517018.1	56	0	0.00	Noto	NP_001007473.1	232	0	0.00
Susd3	XP_036013999.1	179	1	5.89	Smyd5	NP_659167.2	868	3	3.64
Fgd3	XP_036013959.1	500	4	8.43	Pradc1	NP_082781.1	36	0	0.00
Bicd2	NP_084067.1	470	2	4.48	Cct7	NP_031664.3	1901	1	0.55
Ippk	NP_951011.1	168	1	6.27	Fbxo41	NP_001001160.1	962	1	1.10
Cenpp	NP_079771.1	426	1	2.47	Egr4	NP_065621.1	627	1	1.68
Ecm2	NP_001012324.1	545	3	5.80	Alms1	XP_006506116.1	544	5	9.69
Aspn	NP_079987.2	1094	3	2.89	Nat8f7	NP_001240839.2	136	1	7.75
Omd	NP_001347637.1	706	3	4.48	Nat8f3	XP_017177330.1	202	2	10.43
Ogn	NP_032786.1	1042	3	3.03	Nat8f6	NP_001188318.1	202	2	10.43
Nol8	NP_001258326.1	565	0	0.00	Nat8f5	NP_075982.2	172	1	6.13
Iars	NP_742012.2	1383	1	0.76	Nat8	NP_075944.1	386	3	8.19
Gm8674	XP_017171148.1	7	0	0.00	Nat8f2	NP_444326.2	172	0	0.00
Gm8700	XP_030103379.1	29	1	36.34	Nat8f4	NP_001139367.1	173	0	0.00
Gm906	XP_006516997.1	29	1	36.34	Nat8f1	XP_006506533.1	172	0	0.00
Fbxw17	NP_780610.2	0	0	#DIV/0!	Tprkb	XP_017177221.1	231	3	13.69
Nutm2	NP_001028572.1	91	0	0.00	Dusp11	NP_082375.4	453	1	2.33
Gm904	NP_001028942.1	1	1	1053.90	Figla	NP_036143.1	374	1	2.82
Gm8765	NP_001231578.1	3	3	1053.90	Add2	NP_038486.2	1218	4	3.46
Spin1	XP_017170958.1	283	0	0.00	Tgfa	NP_112476.1	207	1	5.09
Nxnl2	XP_006517022.1	159	0	0.00	Fam136a	NP_001355292.1	322	1	3.27
S1pr3	NP_034231.1	466	0	0.00	Snrpg	NP_080782.1	1357	6	4.66
Shc3	NP_033193.3	688	3	4.60	Pcyox1	NP_001342616.1	216	3	14.64
Cks2	NP_079691.1	1075	1	0.98	Tia1	NP_001157550.1	1426	9	6.65
Secisbp2	NP_083555.1	198	0	0.00	C87436	NP_001351484.1	34	0	0.00
Sema4d	NP_001365253.1	399	1	2.64	Pcbp1	NP_035995.1	1224	6	5.17
Gadd45g	NP_035947.2	549	1	1.92	Asprv1	NP_080690.2	252	0	0.00
Diras2	XP_006517013.1	2085	4	2.02	Mxd1	XP_006505733.1	444	6	14.24
Syk	NP_001185906.1	2347	3	1.35	Snrnp27	NP_079941.1	460	6	13.75
Auh	NP_057918.2	680	0	0.00	Gmcl1	NP_035948.3	218	3	14.50
Nfil3	XP_017170907.1	736	2	2.86	Anxa4	NP_001318049.1	652	6	9.70
Ror2	NP_038874.3	1074	4	3.93	Aak1	XP_036022028.1	1281	6	4.94
Sptlc1	NP_033295.2	676	0	0.00	Nfu1	XP_036008137.1	506	4	8.33
Msx2	NP_038629.2	855	1	1.23	Gfpt1	NP_038556.1	1032	5	5.11
Drd1	NP_034206.1	923	3	3.43	D6Ertd527e	XP_006506430.1	24	0	0.00
Sfxn1	NP_081600.1	352	1	2.99	Antxr1	NP_473382.1	460	4	9.16
Hrh2	XP_036013748.1	351	1	3.00	Gkn1	NP_079742.1	273	6	23.16
Cplx2	XP_017170865.1	782	2	2.70	Gkn2	NP_079743.1	178	1	5.92
Thoc3	NP_082873.2	528	0	0.00	Gkn3	NP_081136.1	86	2	24.51
Simc1	XP_011242830.1	86	4	49.02	Bmp10	NP_033886.2	808	2	2.61
4833439L19Ri	NP_598558.1	52	0	0.00	Arhgap25	XP_017177020.1	422	1	2.50
Arl10	NP_064352.2	1188	2	1.77	Prokr1	NP_001342584.1	323	3	9.79
Nop16	NP_848720.1	516	3	6.13	Aplf	NP_001163960.1	297	4	14.19
Higd2a	NP_080209.1	399	2	5.28	Efcc1	XP_006506501.1	36	0	0.00
Cltb	NP_083146.1	677	1	1.56	Gp9	XP_017177171.1	727	4	5.80
Faf2	NP_848484.2	986	1	1.07	Rab43	NP_001034483.1	687	3	4.60
Rnf44	NP_001139498.1	358	1	2.94	Isy1	NP_598695.1	787	1	1.34
Cdhr2	NP_001028536.2	243	2	8.67	Cnbp	NP_001103216.1	905	1	1.16
Gprin1	NP_036144.2	699	5	7.54	Copg1	NP_059505.1	933	6	6.78
Sncb	NP_001349336.1	1346	6	4.70	Hmces	NP_776098.1	147	1	7.17
Eif4e1b	NP_001273108.1	890	3	3.55	H1f10	NP_941024.1	732	0	0.00
Tspan17	XP_006517477.1	211	3	14.98	Rab7	NP_033031.2	1845	3	1.71
Unc5a	XP_006517100.2	672	8	12.55	Rpn1	NP_598694.3	844	4	4.99
Hk3	NP_001193320.1	942	1	1.12	Gata2	NP_001342182.1	1748	2	1.21
Uimc1	XP_036013821.1	454	2	4.64	Dnajb8	NP_064348.1	641	1	1.64
Zfp346	XP_030103143.1	238	3	13.28	Eefsec	XP_006506528.1	892	3	3.54
Fgfr4	NP_032037.2	1228	5	4.29	Ruvbl1	NP_062659.1	2032	4	2.07
Nsd1	NP_032765.3	1326	6	4.77	Sec61a1	NP_058602.1	1373	6	4.61
Rab24	XP_006517228.1	1116	4	3.78	Kbtbd12	NP_001265600.1	195	1	5.40
Prelid1	NP_079872.4	390	2	5.40	Mgll	XP_036021993.1	998	3	3.17

Mxd3	NP_057871.2	525	6	12.04	Abtb1	XP_006506854.1	916	1	1.15
Lman2	NP_080104.2	415	0	0.00	Podxl2	NP_001334256.1	227	3	13.93
Rgs14	NP_001347643.1	605	6	10.45	Mcm2	NP_032590.2	1408	1	0.75
Slc34a1	NP_035522.2	331	3	9.55	Tpra1	NP_036036.2	202	0	0.00
Pfn3	NP_083579.1	117	3	27.02	Plxna1	NP_032907.1	610	1	1.73
F12	NP_067464.2	1328	2	1.59	Chchd6	NP_001161208.1	313	1	3.37
Grk6	NP_001033107.1	658	4	6.41	Txnrd3	NP_001171529.1	1778	11	6.52
Prr7	NP_001025467.1	80	6	79.04	Vmn1r40	NP_444458.1	15	1	70.26
Dbn1	NP_062787.2	857	6	7.38	Vmn1r41	NP_444460.2	61	1	17.28
Pdlim7	XP_006517407.1	973	6	6.50	Vmn1r42	NP_444451.2	45	1	23.42
Dok3	XP_017171013.1	588	3	5.38	Vmn1r43	NP_444450.2	15	1	70.26
Ddx41	NP_598820.2	1514	6	4.18	Vmn1r44	XP_006505443.1	25	1	42.16
Fam193b	XP_006517262.1	133	6	47.54	Vmn1r45	NP_035814.2	74	1	14.24
Tmed9	NP_080487.2	482	7	15.31	Vmn1r46	NP_444459.1	95	1	11.09
B4galt7	NP_666157.1	337	6	18.76	Vmn1r47	NP_444449.2	11	1	95.81
Caml	NP_031622.2	193	3	16.38	Vmn1r48	NP_444448.1	24	2	87.83
Ddx46	NP_001268984.1	1821	4	2.31	Vmn1r49	NP_036041.1	115	2	18.33
B230219D22R	NP_851795.1	221	1	4.77	Vmn1r50	NP_444455.1	122	1	8.64
Txndc15	NP_780359.2	89	5	59.21	Vmn1r51	NP_035813.2	288	1	3.66
Pcbd2	XP_006517461.1	302	2	6.98	Vmn1r52	NP_444452.1	16	0	0.00
Catsper3	NP_001239416.1	287	4	14.69	V1ra8	NP_444453.1	0	0	#DIV/0!
Pitx1	XP_006517220.1	673	4	6.26	Vmn1r53	NP_444456.2	25	1	42.16
Macroh2a1	NP_001152987.1	1332	3	2.37	Vmn1r54	NP_444454.1	36	1	29.28
Tifab	NP_666088.1	145	0	0.00	BC048671	NP_001345227.1	117	1	9.01
Neurog1	NP_035026.1	725	1	1.45	Chst13	NP_082204.1	405	2	5.20
Cxcl14	NP_062514.2	456	5	11.56	Uroc1	NP_001334258.1	524	7	14.08
Slc25a48	NP_808477.2	436	2	4.83	Zxdc	NP_766590.1	253	2	8.33
Il9	NP_032399.1	563	3	5.62	Cfap100	XP_030111233.1	152	2	13.87
Fbxl21	NP_001333661.1	619	3	5.11	Klf15	XP_011239727.1	769	4	5.48
Lect2	XP_036013772.1	724	1	1.46	Aldh1l1	NP_001343341.1	898	3	3.52
Tgfbi	NP_033395.1	978	3	3.23	Slc41a3	XP_036008201.1	178	1	5.92
Smad5	XP_030103031.1	1007	3	3.14	Iqsec1	XP_011239604.1	520	1	2.03
Trpc7	XP_006517327.1	209	0	0.00	Nup210	XP_006506463.1	411	2	5.13
Spock1	NP_001159937.1	1130	5	4.66	Hdac11	NP_659168.1	1400	4	3.01
Klhl3	NP_001355796.1	696	2	3.03	Fbln2	NP_032018.2	759	4	5.55
Hnrnpa0	NP_084148.1	1523	3	2.08	Wnt7a	NP_033553.2	917	3	3.45
Idnk	NP_932121.2	201	3	15.73	Chchd4	NP_598689.1	549	0	0.00
Ubqln1	NP_689420.1	2304	9	4.12	Tmem43	NP_083042.1	339	2	6.22
Gkap1	XP_030103189.1	170	6	37.20	Xpc	NP_033557.2	710	1	1.48
Kif27	XP_017171112.1	996	5	5.29	Lsm3	NP_080585.1	1249	0	0.00
2210016F16Ri	NP_081611.1	129	6	49.02	Slc6a6	XP_030111163.1	543	0	0.00
Hnrnpk	NP_001347424.1	1319	3	2.40	Grip2	XP_006506167.1	761	2	2.77
Rmi1	XP_036014091.1	489	5	10.78	Ccdc174	NP_766318.2	127	0	0.00
Slc28a3	NP_071712.3	263	4	16.03	4930590J08R	NP_941070.1	210	1	5.02
Ntrk2	NP_001269890.1	2576	3	1.23	Fgd5	XP_006506063.1	412	1	2.56
Agtpbp1	XP_006517402.1	291	3	10.86	Nr2c2	NP_001334271.1	536	1	1.97
Naa35	XP_030103327.1	214	3	14.77	Mrps25	NP_079854.2	322	1	3.27
Golm1	NP_001030294.1	352	3	8.98	Rbsn	NP_084357.2	524	1	2.01
4921517D22R	XP_006517438.1	0	0	#DIV/0!	Trh	NP_033452.2	806	0	0.00
Spata31d1a	NP_082433.2	16	1	65.87	Prickle2	NP_001074615.1	894	4	4.72
Spata31d1b	NP_001161065.1	15	1	70.26	Adamts9	XP_036021608.1	484	4	8.71
Spata31d1d	NP_808379.2	20	0	0.00	Magi1	NP_001273713.1	795	5	6.63
1700014D04R	XP_017171161.1	6	0	0.00	Slc25a26	NP_080531.2	323	5	16.31
Isca1	NP_081197.1	402	1	2.62	Lrig1	NP_001342198.1	1055	6	5.99
Tut7	NP_001360891.1	476	1	2.21	Kbtbd8	NP_001008785.2	624	1	1.69
Gas1	NP_032112.1	345	0	0.00	Suclg2	NP_035637.2	1308	1	0.81
Dapk1	XP_030103260.1	1093	2	1.93	Tafa1	XP_017177127.1	221	1	4.77
BC051665	NP_954599.2	252	6	25.09	Tafa4	XP_017177137.1	170	0	0.00
Ctsll3	NP_081620.2	216	2	9.76	Eogt	XP_036021604.1	292	6	21.66
4930486L24Ri	NP_835199.1	218	9	43.51	Tmf1	NP_001074580.1	476	5	11.07
Ctla2b	NP_031823.1	262	1	4.02	Uba3	XP_030111176.1	1632	6	3.87
Tpbpb	NP_080705.1	73	3	43.31	Arl6ip5	NP_075368.1	337	6	18.76
Ctla2a	NP_031822.2	273	1	3.86	Lmod3	NP_001074626.1	443	6	14.27
Tpbpa	NP_033437.2	114	4	36.98	Frmd4b	NP_001333566.1	461	7	16.00
Ctsj	NP_036137.1	222	4	18.99	Mitf	NP_001106669.1	757	3	4.18
Ctsq	NP_083912.2	252	3	12.55	Gm765	NP_001121564.1	27	0	0.00

Ctsr	NP_064680.1	260	4	16.21	Foxp1	NP_001184250.1	1201	9	7.90
Cts6	NP_067420.1	237	2	8.89	Eif4e3	NP_080105.1	569	7	12.97
Cts8	NP_062414.3	236	1	4.47	Gpr27	NP_032184.1	478	7	15.43
Cts7	NP_001346316.1	254	1	4.15	Prok2	NP_001163890.1	496	7	14.87
Ctsm	NP_001347650.1	243	0	0.00	Rybp	NP_062717.2	567	8	14.87
Cts3	XP_011242767.1	305	0	0.00	Shq1	XP_006506732.1	350	8	24.09
Zfp808	NP_001034328.2	221	1	4.77	Gxylt2	NP_941014.1	136	9	69.74
Gm3604	XP_011242740.1	131	3	24.14	Ppp4r2	NP_891984.1	537	10	19.63
Zfp935	NP_849206.2	126	0	0.00	Pdzrn3	NP_061372.2	989	9	9.59
Zfp934	XP_017171126.1	190	0	0.00	Cntn3	XP_006505792.1	639	4	6.60
6720489N17R	XP_017170962.1	172	3	18.38	Chl1	XP_030110991.1	1080	4	3.90
Gm5141	NP_001242994.1	128	2	16.47	Cntn6	XP_017177167.1	939	6	6.73
Fbp2	NP_032020.2	704	0	0.00	Cntn4	NP_001351494.1	1249	9	7.59
Fbp1	NP_062268.1	1198	0	0.00	Il5ra	XP_017176895.1	401	2	5.26
Aopep	NP_001276853.1	371	2	5.68	Trnt1	XP_036008188.1	896	2	2.35
Fancc	XP_011242778.1	497	2	4.24	Crbn	NP_067424.2	457	8	18.45
Ptch1	NP_001315443.1	1092	2	1.93	Lrrn1	NP_032542.1	979	10	10.77
Ercc6l2	NP_001348053.1	1009	8	8.36	Setmar	NP_848478.2	924	12	13.69
Hsd17b3	NP_032317.2	598	1	1.76	Sumf1	NP_666049.2	273	10	38.60
Slc35d2	XP_006517436.1	342	3	9.24	Itpr1	XP_006505686.1	1454	7	5.07
Zfp367	NP_780703.1	387	4	10.89	Bhlhe40	NP_035628.1	694	5	7.59
Habp4	NP_064370.2	642	6	9.85	Arl8b	NP_080287.1	1446	7	5.10
Cdc14b	XP_036013894.1	1026	1	1.03	Edem1	NP_619618.1	473	2	4.46
Prxl2c	NP_079646.1	196	1	5.38	Grm7	NP_796302.2	1427	5	3.69
Ctsl	XP_006517143.1	729	0	0.00	Lmcd1	NP_659048.1	814	5	6.47
Cdk20	NP_444410.1	704	1	1.50	Ssu2	XP_017177075.1	65	7	113.50
Fam240b	XP_006517423.2	0	0	#DIV/0!	Cav3	NP_031643.1	863	4	4.88
Cntnap3	NP_001074598.1	299	1	3.52	Oxtr	NP_001074616.1	694	3	4.56
Spata31	NP_084323.2	291	2	7.24	Rad18	XP_006506500.1	980	3	3.23
Spata31d1c	NP_001077359.2	164	1	6.43	Srgap3	XP_006506208.1	890	0	0.00
Mfsd14b	NP_598441.3	282	0	0.00	Thumpd3	NP_001342195.1	359	5	14.68
Olfr466	NP_667030.2	12	0	0.00	Setd5	NP_766593.1	694	7	10.63
Nlrp4f	NP_780499.3	147	1	7.17	Lhfpl4	NP_808431.2	370	7	19.94
Zfp369	NP_848141.3	236	0	0.00	Mtmr14	NP_081125.2	350	3	9.03
Gm17353	XP_017171151.2	134	2	15.73	Cpne9	NP_733773.2	224	5	23.52
Gm10324	NP_001171303.1	232	5	22.71	Brpf1	XP_006506845.1	773	2	2.73
Gm10772	XP_036014156.1	134	2	15.73	Ogg1	NP_035087.3	648	2	3.25
2410141K09R	NP_898942.1	186	3	17.00	Camk1	NP_598687.1	1319	0	0.00
Zfp640	XP_030103371.2	240	5	21.96	Tada3	NP_001359322.1	747	3	4.23
Gm10323	XP_017171189.2	150	2	14.05	Arpc4	NP_080828.1	596	2	3.54
Uqcrb	NP_080495.1	553	0	0.00	Ttll3	NP_598684.4	282	3	11.21
Mterf3	XP_006517385.1	444	0	0.00	Rpusd3	NP_001028376.1	375	7	19.67
Gm10767	NP_001171221.1	29	0	0.00	Cidec	NP_001359193.1	551	0	0.00
Ptdss1	NP_032985.2	374	0	0.00	Jagn1	NP_080641.1	243	5	21.69
Zfp712	NP_001159690.1	177	0	0.00	Il17re	NP_665825.2	224	1	4.70
Zfp708	NP_001012451.2	126	0	0.00	Il17rc	NP_849273.1	209	1	5.04
Rslcan18	NP_001242981.1	129	0	0.00	Creld1	NP_598691.1	211	5	24.97
Zfp759	XP_030103132.1	198	0	0.00	Prrt3	XP_030111159.1	249	10	42.33
Rsl1	NP_001013791.1	217	2	9.71	Emc3	NP_780310.1	323	3	9.79
Zfp455	NP_001041669.1	219	1	4.81	Fancd2	NP_001028416.2	1020	0	0.00
Zfp458	XP_017170999.1	200	0	0.00	Fancd2os	XP_036008197.1	47	8	179.39
Zfp457	NP_001003666.2	209	0	0.00	Brk1	NP_598698.1	302	2	6.98
Zfp595	NP_808290.1	200	0	0.00	Vhl	NP_033533.1	910	1	1.16
Zfp953	NP_001033740.1	185	0	0.00	Irak2	NP_751893.3	1144	1	0.92
Zfp456	NP_001001186.3	185	0	0.00	Tatdn2	XP_036022154.1	331	6	19.10
Zfp429	NP_001074410.1	238	0	0.00	Ghrl	NP_001273334.1	757	1	1.39
Zfp459	XP_030103165.1	175	0	0.00	Sec13	NP_077168.2	1382	3	2.29
Zfp874a	NP_808380.1	155	1	6.80	Atp2b2	XP_017176842.1	1944	5	2.71
Zfp874b	NP_001070259.1	127	0	0.00	Slc6a11	NP_766478.1	1263	3	2.50
Zfp58	NP_001007576.1	172	1	6.13	Slc6a1	NP_848818.1	1287	3	2.46
Zfp87	NP_573491.2	209	0	0.00	Hrh1	XP_030111052.1	461	1	2.29
Zfp748	NP_001030308.1	191	2	11.04	Atg7	NP_001366059.1	1817	4	2.32
Zfp729b	NP_001156718.1	223	1	4.73	Vgll4	NP_001343300.1	156	2	13.51
Zfp729a	XP_036013855.1	248	4	17.00	Tamm41	XP_006506615.1	257	3	12.30
Zfp738	NP_001001187.2	196	0	0.00	Syn2	NP_001104485.1	1322	6	4.78
Zfp65	NP_663597.1	469	2	4.49	Timp4	NP_542370.3	314	5	16.78

Zfp85	XP_036013928.1	127	0	0.00	Pparg	XP_006505800.1	2088	6	3.03
Zfp493	NP_082678.2	202	2	10.43	Tsen2	NP_950198.1	372	7	19.83
Zfp273	NP_938081.2	134	0	0.00	Mkrn2os	NP_001094901.1	29	0	0.00
Gm10037	XP_017170821.1	127	0	0.00	Mkrn2	NP_075779.2	183	9	51.83
BC048507	NP_001001185.1	854	1	1.23	Raf1	NP_001343263.1	1632	5	3.23
Mtrr	XP_036013852.1	712	2	2.96	Tmem40	XP_011239815.1	115	8	73.31
Fastkd3	XP_006517427.1	210	2	10.04	Cand2	NP_080234.2	471	5	11.19
1700001L19Ri	NP_081311.1	20	0	0.00	Rpl32	NP_742083.1	1139	5	4.63
Adcy2	NP_705762.2	1442	2	1.46	Efcab12	NP_001103976.1	29	0	0.00
Tent4a	XP_006517250.1	785	5	6.71	Mbd4	XP_006505743.1	364	2	5.79
Srd5a1	NP_780492.2	432	1	2.44	Ift122	XP_006506860.1	973	5	5.42
Nsun2	NP_663329.3	1171	3	2.70	Rho	XP_006505924.1	1550	2	1.36
Ube2ql1	NP_001138634.1	820	5	6.43	H1f8	NP_612184.1	902	3	3.51
Med10	NP_001365854.1	350	3	9.03	Plxnd1	NP_080652.2	642	4	6.57
Ice1	NP_659086.2	474	3	6.67	Tmcc1	XP_036022144.1	187	1	5.64
Adamts16	XP_006517331.1	276	2	7.64	Washc2	NP_080861.2	241	2	8.75
Irx1	NP_034703.2	591	4	7.13	Zfand4	XP_006506569.1	3177	8	2.65
Irx2	NP_034704.1	615	4	6.85	Marchf8	XP_006506702.1	467	2	4.51
Irx4	XP_006517355.1	532	4	7.92	Alox5	NP_033792.1	723	4	5.83
Ndufs6	NP_035018.1	1030	7	7.16	Olfr211	XP_030111255.1	59	0	0.00
Mrpl36	NP_444393.1	842	5	6.26	Olfr212	NP_001011800.1	36	1	29.28
Lpcat1	NP_663351.3	547	10	19.27	Olfr213	NP_001011801.1	26	0	0.00
Slc6a3	NP_034150.1	600	5	8.78	Olfr214	NP_666970.1	71	0	0.00
Clptm1l	NP_666159.2	364	13	37.64	Olfr215	NP_666657.1	34	1	31.00
Tert	NP_033380.1	1618	4	2.61	Zfp422	XP_030111415.1	261	5	20.19
Slc6a18	NP_001129559.1	365	12	34.65	Rassf4	NP_835146.3	201	1	5.24
Slc6a19	NP_083154.1	412	8	20.46	Depp1	NP_666092.1	223	0	0.00
Slc12a7	XP_006517233.1	542	7	13.61	Tmem72	NP_848883.2	172	0	0.00
Nkd2	NP_001334464.1	315	1	3.35	Cxcl12	NP_038683.1	1795	2	1.17
Trip13	NP_081458.1	1562	12	8.10	Zfp637	NP_001333577.1	253	1	4.17
Brd9	NP_001365876.1	417	8	20.22	Zfp239	NP_001347867.1	332	0	0.00
Zdhhc11	NP_081980.1	277	7	26.63	4933440N22F	XP_017177366.1	0	0	#DIV/0!
Tppp	NP_878259.1	884	10	11.92	Hnrnpf	NP_598595.1	977	1	1.08
Cep72	XP_006517486.1	266	6	23.77	Fxyd4	XP_017176786.1	198	0	0.00
Slc9a3	XP_006517084.1	751	7	9.82	Rasgef1a	NP_001349034.1	586	1	1.80
Exoc3	XP_006517254.1	739	4	5.70	Csgalnact2	XP_036008295.1	222	0	0.00
Ahrr	NP_033774.1	240	1	4.39	Ret	NP_033076.2	1275	3	2.48
Pdcd6	NP_001346925.1	532	7	13.87	Bms1	NP_919320.1	951	0	0.00
Sdha	NP_075770.1	1679	4	2.51	Zfp248	XP_036008218.1	280	0	0.00
Ccdc127	NP_001162129.1	119	6	53.14	Zfp9	XP_011239595.1	319	0	0.00
Lrrc14b	NP_001028214.1	148	5	35.60	Ankrd26	XP_006506086.1	911	0	0.00
Zfp72	NP_001360967.1	241	1	4.37	Cacna1c	XP_006505506.1	1395	4	3.02
Zfp825	NP_666343.1	201	1	5.24	Dcp1b	XP_006506301.1	590	4	7.15
Erap1	NP_109636.1	953	5	5.53	Cacna2d4	XP_036022112.1	339	6	18.65
Cast	XP_006517122.1	499	3	6.34	Lrtm2	XP_036021898.1	734	7	10.05
Pcsk1	XP_006517216.1	1305	5	4.04	Adipor2	NP_001342621.1	408	5	12.92
Ell2	NP_620403.2	512	2	4.12	Wnt5b	XP_036021918.1	870	2	2.42
Glrx	NP_001347080.1	804	0	0.00	Fbxl14	NP_598701.2	777	6	8.14
Rhobtb3	XP_006517473.1	1749	3	1.81	Erc1	XP_030110969.1	691	6	9.15
Spata9	NP_083619.1	65	2	32.43	Rad52	NP_001159853.1	956	3	3.31
Rfesd	NP_849247.3	39	1	27.02	Wnk1	XP_017177045.1	518	4	8.14
Gpr150	NP_780704.1	271	0	0.00	Ninj2	NP_057927.1	203	2	10.38
Arsk	NP_084123.2	139	0	0.00	B4galnt3	XP_006506379.1	252	2	8.36
Ttc37	NP_001074821.1	575	0	0.00	Ccdc77	XP_006506563.1	172	3	18.38
Mctp1	XP_006517525.3	284	3	11.13	Kdm5a	NP_666109.2	2235	3	1.41
Slf1	XP_011242758.1	2153	3	1.47	Il17ra	NP_032385.1	494	1	2.13
2210408I21Ril	NP_001139148.1	21	0	0.00	Tmem121b	NP_291045.1	284	2	7.42
Fam172a	XP_006517416.1	144	1	7.32	Hdhd5	NP_659064.1	199	0	0.00
Pou5f2	NP_083591.1	149	0	0.00	Cecr2	NP_001355635.1	2005	3	1.58
Nr2f1	XP_006517148.1	890	4	4.74	Slc25a18	NP_001074517.1	452	1	2.33
Arrdc3	NP_001036056.1	625	7	11.80	Atp6v1e1	NP_031536.2	635	2	3.32
Adgrv1	XP_017170853.1	545	4	7.74	Bcl2l13	NP_705736.1	290	1	3.63
Lysmd3	NP_084533.1	256	6	24.70	Bid	XP_036021660.1	265	1	3.98
Polr3g	NP_001074645.1	307	2	6.87	Mical3	XP_030111124.1	749	1	1.41
Mblac2	NP_082648.1	150	6	42.16	Pex26	NP_083006.1	149	1	7.07
Cetn3	NP_031710.1	1917	9	4.95	Tuba8	NP_059075.1	1589	2	1.33

Mef2c	XP_017170894.1	1907	8	4.42	Usp18	NP_036039.2	1200	1	0.88
Tmem161b	NP_780396.2	117	2	18.02	Slc6a13	XP_006505601.1	964	3	3.28
Ccnh	NP_075732.1	1065	2	1.98	Slc6a12	NP_001368835.1	816	4	5.17
Rasa1	NP_663427.2	974	4	4.33	Iqsec3	NP_001028526.1	890	1	1.18
Cox7c	NP_031775.1	559	2	3.77	A2m	NP_783327.2	866	2	2.43
Edil3	NP_001033076.1	573	5	9.20	Mug1	NP_032671.2	799	2	2.64
Hapln1	NP_038528.3	530	5	9.94	Mug2	XP_006505763.1	799	2	2.64
Vcan	NP_001127947.1	1072	4	3.93	Klrg1	NP_058666.1	546	1	1.93
Xrcc4	NP_082288.1	540	8	15.61	M6pr	NP_034879.2	363	0	0.00
Tmem167	NP_079611.2	118	1	8.93	Phc1	XP_030111001.1	452	3	6.99
Atp6ap1l	XP_006517354.1	127	1	8.30	Rimklb	NP_081940.1	248	2	8.50
Rps23	NP_077137.1	1516	4	2.78	Mfap5	NP_056591.1	328	2	6.43
Atg10	XP_030103225.1	322	0	0.00	Aicda	NP_033775.1	846	6	7.47
Ssbp2	NP_001347704.1	339	7	21.76	Apobec1	NP_112436.1	377	7	19.57
Acot12	NP_083066.1	842	0	0.00	Gdf3	NP_032134.2	717	5	7.35
Zcchc9	XP_006517424.1	549	2	3.84	Dppa3	NP_631964.1	265	4	15.91
Ckmt2	NP_940807.1	876	1	1.20	Gm10420	XP_036008359.1	1326	1	0.79
Rasgrf2	NP_033053.2	1000	6	6.32	Gm10224	XP_036008360.1	1164	1	0.91
Msh3	NP_001298049.1	770	8	10.95	Nanog	NP_082292.1	1873	6	3.38
Gm20379	XP_036014172.1	33	0	0.00	Slc2a3	XP_030111144.1	1019	9	9.31
Dhfr	NP_034179.1	1946	9	4.87	Foxj2	NP_068699.1	696	1	1.51
Ankrd34b	NP_780664.2	366	5	14.40	C3ar1	NP_033909.1	970	9	9.78
Fam151b	NP_001157099.1	235	5	22.42	Necap1	NP_080543.2	417	4	10.11
Zfyve16	NP_775568.1	296	12	42.73	Clec4a1	NP_955015.1	255	8	33.06
Spz1	NP_084513.3	550	0	0.00	Clec4a3	NP_001191170.1	504	8	16.73
Serinc5	NP_766176.1	292	5	18.05	Clec4a4	NP_001005860.1	252	2	8.36
Thbs4	NP_035712.1	585	3	5.40	Clec4b1	NP_001177239.1	313	6	20.20
Mtx3	NP_001156417.1	268	6	23.59	Clec4a2	NP_001163804.1	500	8	16.86
Cmya5	XP_036014111.1	746	6	8.48	Clec4b2	NP_001004159.1	166	0	0.00
Tent2	NP_001348465.1	694	4	6.07	Clec4n	NP_064385.1	580	7	12.72
Homer1	NP_671705.2	583	1	1.81	Clec4d	NP_034949.3	693	7	10.65
Jmy	NP_067285.2	256	2	8.23	Clec4e	NP_064332.1	631	9	15.03
Gm6109	XP_889797.1	1165	0	0.00	Vmn2r19	NP_001098102.1	44	0	0.00
Bhmt	NP_057877.1	743	2	2.84	Vmn2r20	NP_001098104.1	26	0	0.00
Bhmt2	NP_075022.2	536	2	3.93	Vmn2r21	NP_001098105.1	7	0	0.00
Dmgdh	NP_083048.1	659	3	4.80	Vmn2r22	NP_001098107.1	26	0	0.00
Arsb	NP_033842.3	303	3	10.43	Vmn2r23	NP_001098108.1	27	1	39.03
Lhfpl2	XP_030103091.1	187	3	16.91	Vmn2r24	NP_001098109.1	50	0	0.00
Scamp1	NP_083429.1	528	2	3.99	Vmn2r25	NP_001098111.1	88	0	0.00
Ap3b1	XP_006517586.1	1176	4	3.58	Vmn2r26	NP_064301.2	67	0	0.00
Tbca	NP_033347.1	470	0	0.00	Vmn2r27	NP_001098112.1	22	0	0.00
Otp	NP_035151.1	603	0	0.00	Cd163	NP_001163866.1	770	6	8.21
Wdr41	XP_036013908.1	191	0	0.00	Pex5	NP_001347499.1	638	1	1.65
Pde8b	NP_001360935.1	523	2	4.03	Clstn3	NP_705728.1	872	2	2.42
Zbed3	XP_030103276.1	181	0	0.00	C1rl	NP_851989.3	348	4	12.11
Aggf1	NP_079906.2	459	1	2.30	C1ra	NP_075632.3	770	6	8.21
Crhbp	NP_940800.1	327	7	22.56	C1s1	NP_659187.2	885	6	7.15
S100z	NP_001074628.1	133	5	39.62	C1rb	NP_001106827.1	580	6	10.90
F2rl1	NP_032000.3	608	7	12.13	C1s2	NP_776289.2	596	5	8.84
F2r	NP_034299.2	835	6	7.57	Lpcat3	NP_660112.1	462	4	9.12
Iqgap2	XP_036013985.1	997	6	6.34	Emg1	NP_038564.1	1395	4	3.02
F2rl2	NP_034300.3	391	4	10.78	Phb2	NP_031557.2	1506	4	2.80
Sv2c	XP_036014095.1	472	5	11.16	Ptpn6	NP_001071173.1	1390	4	3.03
Poc5	NP_080449.1	196	1	5.38	Grcc10	NP_038563.1	196	0	0.00
Ankdd1b	XP_036013957.1	1017	0	0.00	Atn1	NP_031907.2	708	1	1.49
Polk	NP_001334535.1	537	0	0.00	Eno2	XP_030111003.1	2316	3	1.37
Cert1	XP_036014034.1	398	0	0.00	Lrrc23	XP_006505721.1	704	0	0.00
Hmgcr	NP_001347095.1	1251	3	2.53	Spsb2	XP_030111031.1	390	0	0.00
Gcnt4	NP_001363928.1	213	2	9.90	Tpi1	NP_033441.3	2270	3	1.39
1700029F12Ri	NP_001271134.1	3	0	0.00	Usp5	NP_001313523.1	795	6	7.95
Fam169a	XP_036013962.1	97	0	0.00	Cdca3	XP_006505627.1	568	3	5.57
Nsa2	NP_067527.3	1174	3	2.69	Gnb3	NP_038558.1	1468	4	2.87
Gfm2	NP_001139515.1	472	0	0.00	P3h3	NP_038562.2	263	3	12.02
Hexb	NP_034552.1	356	2	5.92	Gpr162	NP_001342186.1	463	2	4.55
Enc1	XP_030102997.1	530	3	5.97	Cd4	NP_038516.1	884	1	1.19
Arhgef28	XP_006517581.1	1137	6	5.56	Lag3	NP_032505.1	445	1	2.37

Utp15	NP_849249.1	987	4	4.27	Ptms	NP_081264.1	128	1	8.23
Ankra2	NP_001258318.1	975	3	3.24	Mlf2	NP_663360.1	383	4	11.01
Gm21976	XP_006517862.1	6	0	0.00	Cops7a	XP_006506224.2	532	2	3.96
Btf3	NP_663430.2	840	1	1.25	Pianp	NP_783627.2	334	1	3.16
Foxd1	NP_032268.2	706	3	4.48	Zfp384	NP_001359349.1	491	1	2.15
Gm10320	XP_036014136.1	609	1	1.73	Ing4	NP_001355624.1	592	0	0.00
Tmem174	NP_080961.1	102	6	61.99	Acrbp	NP_058541.2	314	0	0.00
Tmem171	NP_001020777.1	142	6	44.53	Lpar5	XP_006506403.1	551	1	1.91
Fcho2	XP_011242954.1	878	9	10.80	Chd4	XP_006505344.1	1614	4	2.61
Tnpo1	NP_848831.2	1010	9	9.39	Nop2	NP_620086.2	1416	2	1.49
H2bl1	NP_081340.1	1207	1	0.87	Iffo1	XP_006506337.2	97	0	0.00
Zfp366	NP_001004149.1	359	7	20.55	Gapdh	NP_001276655.1	6342	9	1.50
Ptcd2	NP_081149.1	135	7	54.65	Ncapd2	NP_666283.1	924	2	2.28
Mrps27	XP_006517665.1	268	6	23.59	Mrpl51	NP_079871.1	339	1	3.11
Map1b	NP_032660.2	1543	8	5.46	Vamp1	NP_033522.1	983	2	2.14
Cartpt	NP_001074962.1	714	1	1.48	Tapbpl	NP_663366.2	251	2	8.40
Mccc2	NP_084302.1	542	1	1.94	Cd27	NP_001036029.1	689	5	7.65
Bdp1	XP_017171035.1	568	2	3.71	Tuba3a	NP_033472.1	1610	2	1.31
Serf1	NP_035483.1	139	4	30.33	Ltbr	NP_034866.1	490	5	10.75
Smn1	NP_035550.1	886	6	7.14	Scnn1a	XP_017176960.1	897	6	7.05
Naip2	NP_035002.2	549	3	5.76	Tnfrsf1a	NP_035739.2	1294	8	6.52
Naip5	XP_011242935.1	569	4	7.41	Plekhg6	XP_017176984.1	359	2	5.87
Naip6	NP_035001.2	447	1	2.36	Cd9	NP_031683.1	964	3	3.28
Naip1	NP_032696.2	501	3	6.31	Vwf	NP_035838.3	1617	6	3.91
Gtf2h2	NP_001347635.1	540	10	19.52	Ano2	NP_705817.2	467	2	4.51
Ocln	NP_001347468.1	1108	5	4.76	Ntf3	XP_006505778.1	746	5	7.06
Marveld2	NP_848497.2	320	2	6.59	Kcna5	NP_666095.1	979	5	5.38
Rad17	NP_035363.2	1311	5	4.02	Kcna1	NP_034725.3	1629	5	3.23
Ak6	NP_081868.1	708	2	2.98	Kcna6	XP_006505701.1	993	2	2.12
Taf9	NP_081415.1	708	2	2.98	Ndufa9	NP_079634.2	929	2	2.27
Ccdc125	XP_017171119.2	58	0	0.00	Akap3	NP_033780.2	383	2	5.50
Cdk7	NP_034004.2	1075	5	4.90	Dyrk4	NP_997093.2	831	5	6.34
Mrps36	NP_001177193.1	388	0	0.00	Rad51ap1	NP_001334384.1	644	2	3.27
Cenph	XP_030103138.1	574	1	1.84	D6Wsu163e	NP_613060.1	121	1	8.71
Ccnb1	NP_758505.2	2992	6	2.11	Fgf6	NP_034334.1	706	3	4.48
Slc30a5	XP_036014047.1	575	3	5.50	Fgf23	XP_036008152.1	733	1	1.44
Pik3r1	NP_001070963.1	1422	1	0.74	Tigar	NP_795977.1	700	4	6.02
Cd180	NP_032559.2	850	0	0.00	Ccnd2	XP_036021678.1	1370	7	5.38
Mast4	NP_780380.2	442	1	2.38	Parp11	XP_017176775.1	214	7	34.47
Gm5454	XP_036014182.1	899	0	0.00	Cracr2a	XP_006506406.1	582	5	9.05
Srek1	NP_001348014.1	453	3	6.98	Prmt8	NP_958759.2	1675	1	0.63
Erbin	NP_001276403.1	1435	9	6.61	Tspan11	XP_036008174.1	197	7	37.45
Nln	NP_083723.1	527	3	6.00	Tspan9	XP_030110945.1	381	8	22.13
Sgtb	NP_001347983.1	1223	11	9.48	Tead4	NP_001074448.2	742	2	2.84
Trappc13	NP_001347707.1	104	5	50.67	Tulp3	NP_035787.1	461	0	0.00
Trim23	XP_036014122.1	1988	10	5.30	Rhno1	NP_001313516.1	221	2	9.54
Ppwd1	NP_766395.2	456	14	32.36	Foxm1	NP_032047.4	1527	3	2.07
Cenpk	NP_068562.1	507	0	0.00	Tex52	NP_081973.1	32	0	0.00
Adamts6	NP_001074489.1	433	12	29.21	Nrip2	XP_006506512.1	424	5	12.43
Cwc27	NP_080348.1	1638	13	8.36	Itfg2	NP_598688.1	134	0	0.00
Srek1ip1	NP_080351.2	169	13	81.07	Fkbp4	NP_034349.1	1539	4	2.74
Shisal2b	NP_084260.1	57	13	240.36	Pzp	NP_031402.3	1111	3	2.85
Rgs7bp	NP_084155.2	759	12	16.66	A2ml1	NP_001001179.2	406	1	2.60
Nt5el	NP_080027.1	268	11	43.26	Klrb1a	NP_001153374.1	271	6	23.33
Rnf180	XP_030103272.1	256	11	45.28	Clec2h	NP_444395.2	212	6	29.83
Htr1a	NP_032334.2	1149	9	8.26	Klrb1	XP_017176770.1	266	7	27.73
Ipo11	NP_083941.2	569	9	16.67	Klrb1c	NP_032553.2	731	8	11.53
Dimt1	NP_079723.1	1120	1	0.94	Klrb1b	NP_085102.4	217	6	29.14
Kif2a	NP_001365867.1	1399	2	1.51	BC035044	NP_001241875.1	4	0	0.00
Zswim6	NP_663431.3	260	0	0.00	Clec2i	NP_001276635.1	270	6	23.42
Smim15	NP_001041715.1	245	1	4.30	Clec2g	NP_081838.1	290	6	21.80
Ndufaf2	XP_036014098.1	347	3	9.11	Clec2f	NP_001264131.1	290	6	21.80
Ercc8	XP_017171097.1	472	3	6.70	Klrb1f	NP_694734.1	212	7	34.80
Elovl7	NP_083277.3	430	3	7.35	Clec2e	NP_705726.3	183	6	34.55
Depdc1b	NP_848798.1	391	6	16.17	Clec2d	NP_444339.1	438	11	26.47
Pde4d	NP_035186.1	802	5	6.57	Cd69	NP_001028294.1	1044	7	7.07

Rab3c	NP_001347028.1	1003	2	2.10	Clec2m	NP_950199.1	305	8	27.64
Gapt	XP_036013938.1	195	0	0.00	Clec12a	NP_808354.1	545	10	19.34
Plk2	NP_690017.2	1859	5	2.83	Clec12b	XP_011239768.1	201	8	41.95
Actbl2	NP_780706.1	1298	2	1.62	Clec1b	NP_064369.1	494	19	40.53
Gpbp1	NP_001116435.1	464	6	13.63	Clec9a	NP_001192294.1	412	6	15.35
Mier3	NP_766181.2	498	8	16.93	Clec1a	NP_780735.2	277	12	45.66
Map3k1	XP_017171005.1	1696	5	3.11	Clec7a	NP_001296566.1	782	12	16.17
Ankrd55	NP_084174.2	1097	10	9.61	Olr1	NP_001288025.1	698	10	15.10
Il6st	XP_036013751.1	646	2	3.26	Tmem52b	NP_001074655.1	94	3	33.64
Il31ra	XP_036013921.1	221	6	28.61	Gabarapl1	NP_065615.1	1128	5	4.67
Ddx4	NP_034159.1	1943	8	4.34	Klre1	XP_006506184.1	199	7	37.07
Slc38a9	XP_030103136.1	270	4	15.61	Klrd1	XP_006505718.1	678	15	23.32
Plpp1	NP_032273.1	562	5	9.38	Klrk1	NP_149069.1	763	12	16.58
Mtrex	NP_082427.1	1626	3	1.94	Klrc3	NP_067353.1	175	12	72.27
Dhx29	NP_766182.2	1139	7	6.48	Klrc2	NP_001092139.1	308	15	51.33
Ccno	NP_001074531.1	1531	6	4.13	Klrc1	XP_017176907.1	388	17	46.18
Mcidas	NP_001033003.1	412	5	12.79	Klri1	XP_006506426.1	151	4	27.92
Cdc20b	XP_036013940.1	1773	3	1.78	Klri2	NP_796129.2	157	6	40.28
Gpx8	NP_081403.1	856	0	0.00	Klrh1	NP_001014997.1	140	3	22.58
Gzma	NP_034500.1	817	5	6.45	Klra17	NP_001368891.1	238	4	17.71
Gzmk	NP_032222.1	500	4	8.43	Klra5	XP_036021782.1	201	5	26.22
Esm1	NP_076101.1	464	2	4.54	Klra6	XP_017176906.1	202	5	26.09
Snx18	NP_570614.2	508	1	2.07	Klra4	NP_034779.1	261	5	20.19
Hspb3	NP_064344.1	939	3	3.37	Klra8	NP_034780.1	291	4	14.49
Arl15	NP_766183.1	1613	3	1.96	Klra9	NP_034781.2	312	2	6.76
Ndufs4	NP_035017.2	653	3	4.84	Klra7	NP_055009.5	252	1	4.18
Fst	NP_032072.1	875	2	2.41	Klra10	XP_036021779.1	141	0	0.00
Mocs2	NP_038854.2	344	5	15.32	Klra3	NP_034778.2	301	0	0.00
Itga2	NP_032422.2	974	3	3.25	Klra1	NP_057868.2	414	1	2.55
Itga1	NP_001028400.2	715	6	8.84	Klra2	XP_017176903.1	213	0	0.00
Pelo	NP_598819.2	583	4	7.23	Magohb	NP_079840.2	820	1	1.29
Isl1	NP_067434.3	1244	2	1.69	Styk1	XP_030111251.1	438	2	4.81
Parp8	NP_001074478.2	202	7	36.52	Ybx3	NP_035863.1	735	2	2.87
Emb	NP_034460.3	227	2	9.29	Gm6619	NP_001334413.1	0	0	#DIV/0!
Hcn1	NP_034538.2	570	2	3.70	5430401F13R	NP_001231557.1	0	0	#DIV/0!
Mrps30	NP_067531.1	413	6	15.31	Tas2r130	NP_954607.1	345	4	12.22
Fgf10	NP_032028.1	1019	5	5.17	Tas2r107	NP_954605.1	271	2	7.78
Nnt	NP_001295435.1	417	0	0.00	Tas2r106	NP_996899.1	315	2	6.69
Paip1	NP_663432.1	1046	3	3.02	Tas2r104	NP_996894.1	357	3	8.86
4833420G17R	NP_001347734.1	63	1	16.73	Tas2r105	NP_065247.1	370	4	11.39
Tmem267	NP_001357555.1	27	1	39.03	Tas2r114	NP_996902.1	353	5	14.93
Ccl28	NP_064675.1	387	0	0.00	Gm4736	XP_036021594.1	69	1	15.27
Hmgcs1	XP_036013850.1	835	0	0.00	Prb1	NP_941071.1	21	1	50.19
Nim1k	NP_780747.1	1169	1	0.90	Prpmp5	NP_001019876.2	21	1	50.19
Zfp131	XP_017171099.1	626	1	1.68	Gm8882	NP_001171059.1	14	2	150.56
AF067063	NP_001001449.1	108	7	68.31	Prh1	NP_035304.4	57	1	18.49
Tcstv1	NP_061226.1	256	6	26.00	Prp2	NP_113687.2	17	1	61.99
Gm21761	NP_001297561.1	18	5	325.28	A630073D07F	NP_001136441.1	0	0	#DIV/0!
B020031M17F	NP_001028941.1	118	8	84.06	Tas2r120	NP_996906.1	386	10	27.30
Gm36079	XP_006517870.1	1	0	0.00	Tas2r121	NP_996907.1	539	10	19.55
Gm21188	XP_036013699.1	1	0	0.00	Tas2r122	NP_001034217.1	464	5	11.36
Gm20767	NP_001034737.2	153	7	68.88	Tas2r115	NP_996903.1	287	3	11.02
Gm21818	NP_001358022.1	181	8	71.66	Tas2r124	NP_996909.1	409	3	7.73
AF067061	NP_951015.1	122	8	115.18	Tas2r102	NP_954604.2	382	4	11.04
BC147527	XP_017171045.1	34	4	225.43	Tas2r136	NP_851793.1	148	9	64.09
Tcstv3	NP_705743.1	216	5	48.79	Tas2r117	NP_996904.1	242	5	21.77
Ube2e2	XP_017171446.1	2102	0	0.00	Tas2r123	NP_996908.1	263	4	16.03
Ube2e1	NP_033481.1	2040	1	0.94	Tas2r116	NP_444442.1	388	5	13.58
Nkiras1	NP_076015.2	285	2	12.33	Tas2r110	NP_954606.2	340	5	15.50
Rpl15	NP_001346826.1	1164	3	4.18	Tas2r113	NP_996901.1	291	3	10.86
Nr1d2	NP_035714.3	508	2	5.93	Tas2r125	NP_996910.1	239	2	8.82
Thrb	NP_001106888.1	791	3	5.33	Tas2r129	NP_996912.1	293	3	10.79
Rarb	NP_001276689.1	928	4	5.68	Tas2r131	NP_996913.1	247	11	46.93
Top2b	NP_033435.2	2252	5	2.75	Tas2r109	NP_996900.1	312	3	10.13
Ngly1	NP_001349362.1	403	2	5.81	Tas2r103	NP_444441.1	349	3	9.06
Oxsm	NP_001346812.1	784	3	4.25	Tas2r140	NP_067537.1	315	9	30.11

Lrrc3b	NP_666164.1	732	3	4.32	Smim10l1	NP_001258511.1	16	0	0.00
Nek10	NP_001182158.1	299	2	7.05	5530400C23R	NP_082060.1	45	0	0.00
Slc4a7	NP_001347445.1	607	1	1.74	Gm5885	NP_001171969.1	44	0	0.00
Il3ra	XP_006517998.3	574	0	0.00	Kap	NP_034724.1	80	0	0.00
Olfr31	NP_667238.2	103	0	0.00	Etv6	NP_031987.3	801	6	7.89
Olfr720	NP_666504.1	37	0	0.00	Bcl2l14	NP_001342615.1	452	7	16.32
Psmd6	NP_079826.2	1363	3	2.32	Lrp6	NP_032540.2	768	6	8.23
Atxn7	XP_006518081.1	584	5	9.02	Mansc1	NP_080621.1	93	6	67.99
Thoc7	NP_001346830.1	546	3	5.79	Borcs5	NP_001163950.1	111	9	85.45
Gm281	XP_011243166.1	98	1	10.75	Dusp16	NP_001041519.1	795	13	17.23
Sntn	NP_808292.1	88	0	0.00	Crebl2	XP_011239631.2	208	12	60.80
Synpr	NP_082328.3	963	2	2.19	Gpr19	NP_001161166.1	209	5	25.21
Cadps	XP_036014507.1	1276	5	4.13	Cdkn1b	NP_034005.2	1685	8	5.00
Fezf2	XP_030103750.1	951	2	2.22	Apold1	NP_001103384.1	271	7	27.22
3830406C13R	XP_006518050.1	116	5	45.43	Ddx47	NP_080636.2	1633	7	4.52
Ptprg	XP_006518019.1	988	5	5.33	Gprc5a	NP_852109.2	371	5	14.20
Fhit	XP_006517987.1	652	3	4.85	Gprc5d	NP_001192325.1	118	4	35.73
Cfap20dc	NP_083210.3	159	0	0.00	Hebp1	NP_038574.3	217	0	0.00
Oit1	NP_666162.1	92	1	11.46	Fam234b	NP_001349039.1	69	5	76.37
Fam107a	NP_001347268.1	380	2	5.55	Gsg1	NP_001074021.1	291	0	0.00
Acox2	NP_444345.2	1063	2	1.98	Pbp2	NP_083871.3	724	1	1.46
Kctd6	XP_036014745.1	473	0	0.00	Emp1	NP_001275556.1	488	3	6.48
Pdhb	XP_030103844.1	1064	1	0.99	Grin2b	NP_032197.3	1642	2	1.28
Pxk	NP_001347242.1	286	2	7.37	Eif4a3l1	NP_001136206.1	1614	2	1.31
Htd2	NP_001335656.1	453	1	2.33	E330021D16R	NP_001188319.1	174	0	0.00
Abhd6	NP_001317993.1	367	2	5.74	Atf7ip	XP_006506453.1	382	1	2.76
Dnase1l3	XP_006517984.1	320	2	6.59	Plbd1	NP_080082.1	293	2	7.19
Flnb	XP_006518113.1	1372	0	0.00	Gucy2c	NP_659504.2	858	1	1.23
Gm10338	XP_011243119.1	0	0	#DIV/0!	H4f16	NP_783583.1	1933	2	1.09
Gm3558	NP_001257771.1	0	0	#DIV/0!	H2aj	NP_808356.1	1134	2	1.86
Gm3752	XP_036014295.1	0	0	#DIV/0!	Wbp11	XP_017177182.1	443	4	9.52
Gm5797	NP_001020256.1	40	0	0.00	Smco3	NP_001034647.2	12	0	0.00
Gm3739	XP_036014215.1	0	0	#DIV/0!	BC049715	XP_017177123.1	9	1	117.10
Gm3727	XP_036014864.1	0	0	#DIV/0!	Art4	NP_080915.1	187	0	0.00
Gm16434	XP_011243125.1	1	0	0.00	Mgp	NP_032623.1	460	3	6.87
Gm3512	XP_036014843.1	0	0	#DIV/0!	Erp27	NP_081259.1	799	3	3.96
Gm6676	XP_011243129.1	1	0	0.00	Arhgdib	XP_030110977.1	867	4	4.86
Gm3696	NP_001019883.2	0	0	#DIV/0!	Pde6h	NP_076387.2	278	1	3.79
Gm3685	XP_017171813.1	0	0	#DIV/0!	Rerg	NP_001157686.1	1472	5	3.58
Gm10406	XP_017171193.1	0	0	#DIV/0!	Ptpro	XP_036021846.1	772	2	2.73
Gm3667	XP_036014213.1	0	0	#DIV/0!	Eps8	NP_001258516.1	649	4	6.50
Gm3642	XP_030104091.1	0	0	#DIV/0!	Strap	NP_035629.2	657	1	1.60
Gm3636	XP_017171210.1	0	0	#DIV/0!	Dera	NP_766321.1	476	3	6.64
Gm8050	XP_003085040.1	0	0	#DIV/0!	Slc15a5	NP_808455.2	153	1	6.89
Gm3629	NP_001361160.1	0	0	#DIV/0!	Mgst1	NP_001334418.1	1199	1	0.88
Gm3594	XP_011243136.1	0	0	#DIV/0!	Lmo3	XP_036021636.1	969	3	3.26
Gm16440	XP_030104048.1	0	0	#DIV/0!	Igbp1b	NP_056592.2	594	5	8.87
Gm21103	XP_030104046.1	0	0	#DIV/0!	Rergl	NP_001121562.1	1248	4	3.38
Gm3411	NP_001257741.1	0	0	#DIV/0!	Pik3c2g	NP_997566.2	2083	6	3.04
Gm21560	XP_011243138.1	0	0	#DIV/0!	Plcz1	XP_006506963.1	751	5	7.02
Gm3476	XP_011243139.1	40	0	0.00	Capza3	NP_031631.3	899	2	2.34
Gm6337	XP_011243141.2	0	0	#DIV/0!	Plekha5	XP_006506927.1	850	3	3.72
Gm3248	XP_017171816.1	0	0	#DIV/0!	Aebp2	NP_033767.2	506	1	2.08
Gm3424	XP_011243156.1	0	0	#DIV/0!	Pde3a	NP_061249.1	504	3	6.27
Gm3383	XP_036014203.1	1	0	0.00	Slco1c1	NP_067446.1	579	3	5.46
Gm3373	XP_036014840.1	0	0	#DIV/0!	Slco1b2	XP_017177100.1	836	3	3.78
Gm3488	NP_001366536.1	0	0	#DIV/0!	Gm5724	NP_001365611.1	102	0	0.00
Gm3187	XP_011243163.1	0	0	#DIV/0!	Slco1a4	NP_001342506.1	427	2	4.94
Gm8246	XP_011243106.1	0	0	#DIV/0!	Slco1a1	NP_038825.1	618	2	3.41
Gm3194	XP_030103998.2	1	0	0.00	Gm6614	XP_017177185.1	134	0	0.00
Gm3500	XP_036014205.1	0	0	#DIV/0!	Slco1a6	NP_076207.1	298	1	3.54
Gm8271	XP_011243108.1	0	0	#DIV/0!	Slco1a5	NP_570931.1	230	1	4.58
Gm3317	XP_030103806.1	0	0	#DIV/0!	Iapp	NP_034621.1	528	0	0.00
Gm8279	XP_030104013.1	1	0	0.00	Pyroxd1	NP_898988.2	362	1	2.91
4930555G01R	NP_780602.2	0	0	#DIV/0!	Recql	XP_030111131.1	816	0	0.00
Gm3264	XP_036014200.1	0	0	#DIV/0!	Golt1b	NP_080148.1	679	5	7.76

Gm9602	XP_006518257.1	1	0	0.00	Spx	NP_001229274.1	298	7	24.76
Gm3252	XP_036014849.1	0	0	#DIV/0!	Gys2	XP_006507020.1	1644	10	6.41
Gm7876	XP_017171823.1	0	0	#DIV/0!	Ldhb	NP_001303251.1	1384	7	5.33
Gm3239	XP_036014857.1	0	0	#DIV/0!	Kcnj8	NP_001317292.1	492	5	10.71
Gm3173	NP_001361085.1	0	0	#DIV/0!	Abcc9	XP_006507015.1	815	9	11.64
Gm3182	XP_017171822.1	0	0	#DIV/0!	Cmas	NP_034038.2	280	3	11.29
Gm3159	XP_011243160.1	0	0	#DIV/0!	Sult6b2	NP_001138862.1	76	0	0.00
2610042L04Ri	XP_017171584.1	24	0	0.00	St8sia1	NP_035504.2	369	5	14.28
Gm2974	XP_036014844.1	0	0	#DIV/0!	C2cd5	NP_084173.1	224	0	0.00
Gm3127	XP_030104090.1	0	0	#DIV/0!	Etnk1	NP_083526.2	721	6	8.77
Gm8108	XP_036014860.1	0	0	#DIV/0!	Sox5	XP_006507005.1	1000	3	3.16
Gm3115	XP_017171800.2	0	0	#DIV/0!	Bcat1	NP_031558.3	969	9	9.79
Gm5796	NP_001025101.2	0	0	#DIV/0!	Lrmp	XP_011239869.1	346	10	30.46
Gm3095	XP_036014858.1	0	0	#DIV/0!	Casc1	XP_006507036.1	242	10	43.55
Gm3050	XP_030103993.1	0	0	#DIV/0!	Etfrf1	NP_001348983.1	164	6	38.56
Gm3012	NP_001365552.1	1	1	1053.90	Kras	NP_067259.4	4630	12	2.73
Gm3005	XP_036014197.1	0	0	#DIV/0!	Lmntd1	XP_036008255.1	268	7	27.53
Gm10408	NP_001243430.1	0	0	#DIV/0!	Tuba3b	NP_033475.1	1610	2	1.31
Gm10413	NP_083564.2	1	1	1053.90	Rassf8	XP_036008200.1	267	13	51.31
Gm2956	XP_036014227.1	0	0	#DIV/0!	Bhlhe41	NP_077789.1	515	10	20.46
D830030K20R	XP_036014564.1	0	0	#DIV/0!	Sspn	NP_034786.1	387	9	24.51
Gm5795	NP_001257735.1	0	0	#DIV/0!	Itpr2	NP_034716.1	875	12	14.45
Gm10340	XP_011243105.1	0	0	#DIV/0!	Ints13	NP_001349037.1	318	5	16.57
Gm2897	XP_036014194.1	0	0	#DIV/0!	Fgfr1op2	NP_080494.1	297	11	39.03
Gm2888	XP_001475333.3	0	0	#DIV/0!	Tm7sf3	NP_080557.2	190	11	61.02
Gm2244	XP_001472902.3	0	0	#DIV/0!	Med21	NP_079591.1	536	11	21.63
Gm2237	NP_001361048.1	0	0	#DIV/0!	Stk38l	XP_006507092.1	1735	13	7.90
Gm5458	XP_030103738.1	0	0	#DIV/0!	Arntl2	XP_017177092.1	311	4	13.55
Nid2	NP_032721.2	704	2	2.99	Smco2	NP_081335.1	39	3	81.07
Rtraf	NP_080804.1	527	3	6.00	1700034J05R	XP_017177266.1	94	0	0.00
Gng2	XP_030103512.1	955	2	2.21	Ppfibp1	NP_001342657.1	309	11	37.52
Saysd1	NP_080485.1	21	0	0.00	Rep15	NP_079896.1	89	0	0.00
Kcnk5	NP_067517.1	351	1	3.00	Mrps35	NP_663548.2	530	8	15.91
Kcnk16	NP_083282.1	225	1	4.68	Mansc4	NP_001030075.1	43	0	0.00
Nudt13	XP_006519526.1	305	2	6.91	Klhl42	NP_001074706.1	504	7	14.64
Ecd	NP_081751.1	275	0	0.00	Pthlh	NP_032996.2	706	2	2.99
Fam149b	XP_036014232.1	30	5	175.65	Ccdc91	NP_001342642.1	165	4	25.55
Dnajc9	NP_598842.1	677	4	6.23	Far2	NP_848912.1	299	2	7.05
Mrps16	NP_079716.1	1059	2	1.99	Ergic2	XP_030111416.1	767	1	1.37
Cfap70	NP_001157110.1	109	5	48.34	Tmtc1	NP_001334447.1	1121	5	4.95
Anxa7	NP_033804.2	589	1	1.79	Ipo8	XP_036022133.1	1281	1	0.91
Mss51	NP_083380.1	218	5	24.17	Caprin2	XP_006507104.1	213	0	0.00
Ppp3cb	XP_006518774.1	3163	3	1.00	Sinhcaf	NP_001342574.1	283	0	0.00
Usp54	NP_084456.2	292	4	14.44	Dennd5b	NP_796166.2	224	0	0.00
Myoz1	NP_067483.1	380	5	13.87	Etfbkmt	XP_017177126.1	164	0	0.00
Synpo2l	NP_780341.1	807	3	3.92	Amn1	NP_001106895.1	562	1	2.89
Sec24c	XP_036014443.1	616	1	1.71	Resf1	XP_030111414.1	111	1	15.82
Fut11	NP_082704.1	181	2	11.65	Bicd1	XP_006507079.1	468	1	4.09
Chchd1	NP_079642.1	570	1	1.85	Gm21814	NP_001347880.1	126	0	0.00
Zswim8	XP_006519097.1	128	0	0.00	AU018091	XP_006540047.1	144	0	0.00
Ndst2	NP_034941.2	289	2	7.29	Nlrp12	XP_006540194.1	200	0	0.00
Camk2g	NP_001034228.1	1167	5	4.52	Myadm	NP_001087234.1	284	0	0.00
Plau	XP_017171408.1	1166	4	3.62	Prkcg	NP_035232.1	1266	4	5.12
Vcl	XP_006518985.1	1754	7	4.21	Cacng7	XP_036009491.1	683	3	6.61
Ap3m1	NP_061299.3	418	6	15.13	Cacng8	NP_573453.2	422	2	6.66
Adk	NP_598840.1	1145	7	6.44	Cacng6	NP_573446.1	156	1	8.44
Kat6b	XP_030103747.1	1158	6	5.46	Tarm1	XP_006540043.1	198	2	12.52
Dupd1	XP_036014638.1	683	6	9.26	Oscar	NP_001277306.1	259	6	27.13
Dusp13	XP_036014538.1	766	6	8.26	Ndufa3	NP_079624.1	431	8	20.59
Samd8	NP_080559.1	191	6	33.11	Tfpt	NP_076013.1	203	10	51.92
Vdac2	NP_035825.1	1217	3	2.60	Prpf31	NP_081604.3	1534	6	4.12
Comtd1	NP_081241.1	513	4	8.22	Cnot3	XP_036008812.1	912	5	5.78
Zfp503	NP_663434.2	312	2	6.76	Leng1	NP_081479.1	123	13	111.39
Lrmda	NP_082551.1	655	3	4.83	Tmc4	NP_861541.2	147	1	7.17
Kcnma1	NP_001240303.1	1447	3	2.19	Mboat7	NP_084210.2	280	9	33.88
Gm6158	XP_036014867.1	1329	1	0.79	Tsen34	XP_017167711.2	239	5	22.05

Dlg5	NP_082002.1	1424	3	2.22	Rps9	NP_084043.1	1551	11	7.47
E330034G19R	NP_001028386.1	151	1	6.98	Pirb	XP_006539698.1	612	8	13.78
Polr3a	NP_001074716.1	2019	2	1.04	Pira1	XP_017177526.1	372	8	22.66
Rps24	NP_997517.1	1030	1	1.02	Gm15448	XP_036009508.1	210	8	40.15
Zmiz1	XP_036014570.1	748	2	2.82	Pira2	XP_006539688.1	326	8	25.86
Ppif	XP_030103456.1	1752	5	3.01	Gm14548	XP_006539493.1	371	7	19.88
Zcchc24	NP_001094903.1	162	1	6.51	Lilra6	XP_006539692.1	321	7	22.98
Anxa11	NP_038497.2	530	5	9.94	Lair1	NP_001289605.1	522	10	20.19
Plac9a	NP_997112.1	113	5	46.63	Ttyh1	XP_036009192.1	634	2	3.32
Tmem254a	NP_080955.1	52	3	60.80	Leng8	XP_036008825.1	386	9	24.57
Cphx1	XP_017171258.1	356	7	20.72	Leng9	NP_780738.1	7	0	0.00
Duxbl1	XP_030103412.1	380	7	19.41	Cdc42ep5	XP_006540312.1	143	0	0.00
Slmap	XP_006519800.1	927	0	0.00	Lilra5	XP_006539828.1	233	4	18.09
Dennd6a	NP_001127937.1	159	0	0.00	Ncr1	NP_034876.2	525	2	4.01
Arf4	NP_031505.1	1847	4	2.28	Gp6	XP_006539973.1	261	1	4.04
Pde12	NP_848783.3	537	0	0.00	Rdh13	NP_001277338.1	490	4	8.60
Dnah12	XP_017171732.1	748	3	4.23	Eps8l1	NP_001361551.1	264	2	7.98
Asb14	XP_006518618.1	1451	7	5.08	Ppp1r12c	NP_084110.2	1041	2	2.02
Appl1	XP_006519671.1	1047	7	7.05	Tnnt1	XP_017177603.1	970	5	5.43
Hesx1	NP_034550.2	610	7	12.09	Tnni3	NP_033432.1	751	2	2.81
Il17rd	NP_602319.1	263	2	8.01	Dnaaf3	NP_001028720.1	128	1	8.23
Arhgef3	XP_006519637.1	711	1	1.48	Syt5	NP_001347351.1	767	3	4.12
Tasor	NP_001108351.1	155	2	13.60	Ptprh	NP_997153.2	463	2	4.55
Ccdc66	XP_006519194.1	179	0	0.00	Tmem86b	XP_011248988.1	124	0	0.00
Erc2	XP_006519000.1	967	7	7.63	Ppp6r1	NP_766482.2	541	2	3.90
Wnt5a	NP_033550.2	1469	4	2.87	Hspbp1	NP_077134.1	1496	3	2.11
Cacna2d3	XP_036014314.1	977	4	4.31	Brsk1	XP_017177814.1	1266	6	4.99
Lrtm1	NP_795894.1	541	7	13.64	Tmem150b	NP_808555.1	92	1	11.46
Selenok	NP_064363.2	309	4	13.64	Kmt5c	XP_006539832.1	400	2	5.27
Actr8	XP_030103757.1	938	6	6.74	Cox6b2	NP_001276778.1	470	0	0.00
Il17rb	NP_062529.2	277	4	15.22	Fam71e2	NP_766483.3	111	0	0.00
Chdh	NP_758468.2	720	8	11.71	Il11	NP_032376.1	640	1	1.65
Cacna1d	NP_083257.2	1152	6	5.49	Tmem190	XP_036009458.1	43	1	24.51
Dcp1a	NP_598522.3	782	2	2.70	Tmem238	NP_083660.1	3	0	0.00
Tkt	NP_033414.1	1095	1	0.96	Rpl28	NP_033107.1	757	2	2.78
Prkcd	XP_030103546.1	1706	4	2.47	Ube2s	NP_598538.1	2058	0	0.00
Rft1	NP_808483.2	98	0	0.00	Shisa7	XP_030098277.1	617	1	1.71
Sfmbt1	XP_030103749.1	493	6	12.83	Isoc2b	XP_006540391.1	285	3	11.09
Stimate	NP_083115.3	63	4	66.91	Isoc2a	NP_001095068.1	297	3	10.65
Mustn1	NP_852055.1	211	3	14.98	Zfp628	NP_739565.2	250	0	0.00
Itih4	NP_061216.2	926	12	13.66	Nat14	NP_001345813.1	118	4	35.73
Itih3	XP_006518649.1	811	11	14.29	Ssc5d	NP_766596.1	78	0	0.00
Itih1	NP_032432.2	781	9	12.14	Sbk2	NP_001139801.1	138	0	0.00
Nek4	NP_035979.1	314	11	36.92	Sbk3	XP_006540196.1	67	1	15.73
Spcs1	NP_081187.2	621	8	13.58	Zfp579	XP_006540398.1	171	3	18.49
Glt8d1	XP_036014787.1	166	10	63.49	Fiz1	NP_001347172.1	290	3	10.90
Gnl3	NP_705775.2	1424	8	5.92	Zfp524	NP_079600.1	212	0	0.00
Pbrm1	NP_001346785.1	1863	11	6.22	Zfp865	XP_017177789.1	205	0	0.00
Smim4	NP_001295393.1	25	0	0.00	Zfp784	NP_001034621.1	187	0	0.00
Nt5dc2	NP_001361016.1	231	9	41.06	Zfp580	XP_030098821.1	195	0	0.00
Stab1	XP_036014414.1	585	7	12.61	Ccdc106	XP_036008835.1	41	0	0.00
Nisch	NP_073147.2	481	2	4.38	U2af2	NP_001192160.1	1281	1	0.82
Tnnc1	NP_033419.1	1159	0	0.00	Epn1	XP_036008550.1	853	3	3.71
Sema3g	NP_001020550.1	280	0	0.00	Rfpl4	NP_620404.1	452	1	2.33
Phf7	XP_011243504.1	837	3	3.78	Rasl2-9	NP_033054.1	1081	0	0.00
Bap1	NP_081364.1	840	1	1.25	Vmn1r55	NP_001160178.1	0	0	#DIV/0!
Dnah1	NP_001028840.1	803	3	3.94	Vmn1r56	NP_109665.1	1	0	0.00
Capn7	NP_033926.1	625	2	3.37	Vmn1r57	NP_001160206.1	0	0	#DIV/0!
Sh3bp5	NP_036024.2	335	0	0.00	Nlrp2	NP_808358.2	415	0	0.00
Mettl6	XP_030103828.1	352	2	5.99	Vmn1r58	NP_109664.2	29	0	0.00
Eaf1	NP_083208.1	228	1	4.62	Vmn1r59	NP_997426.1	0	0	#DIV/0!
Colq	NP_034067.2	204	2	10.33	Vmn2r28	NP_001074874.1	31	0	0.00
Hacl1	XP_036014660.1	637	2	3.31	Vmn1r60	NP_001160204.1	47	0	0.00
Btd	NP_079571.1	435	2	4.85	Vmn1r61	NP_001160205.1	12	0	0.00
Ankrd28	NP_001019775.1	1189	3	2.66	Vmn1r62	NP_109666.2	33	0	0.00
Galnt15	XP_006519762.1	201	0	0.00	Vmn1r63	XP_017167894.1	17	0	0.00

Dph3	NP_001040898.1	244	2	8.64	Vmn1r64	NP_997427.1	45	0	0.00
Oxnad1	NP_663435.2	373	2	5.65	Vmn1r65	NP_109663.1	32	0	0.00
Msmb	NP_065622.1	298	2	7.07	Nlrp4c	NP_113566.2	77	0	0.00
Ncoa4	XP_006519130.1	374	3	8.45	Zfp787	NP_001366229.1	246	1	4.28
Timm23	NP_058593.2	791	3	4.00	Zfp444	NP_082592.2	293	1	3.60
Parg	NP_036090.2	346	1	3.05	Galp	NP_821171.1	194	0	0.00
Ogdhl	NP_001074599.2	839	6	7.54	Zscan5b	XP_006539636.1	296	0	0.00
1700024G13R	NP_001029209.1	67	3	47.19	Epp13	XP_011248967.1	69	0	0.00
Chat	NP_034021.1	855	2	2.47	Zfp667	XP_036009103.1	196	0	0.00
Slc18a3	NP_068358.2	814	9	11.65	Zfp583	XP_030098200.1	189	0	0.00
Ercc6	NP_001074690.1	1266	9	7.49	Zfp78	XP_017177795.1	175	0	0.00
Prrxl1	XP_006518472.1	392	8	21.51	Zfp28	NP_780456.2	264	0	0.00
3425401B19R	XP_036014222.1	171	3	18.49	Smim17	NP_001128224.1	38	0	0.00
Tmem273	NP_001157088.1	68	1	15.50	Olfr1344	NP_796035.2	10	0	0.00
Fam170b	NP_001157957.1	34	8	247.98	Olfr1336	NP_667126.1	31	0	0.00
Vstm4	NP_848906.1	321	3	9.85	Olfr1346	NP_667127.1	11	0	0.00
Wdfy4	NP_001139494.1	1428	8	5.90	Olfr5	NP_667125.1	49	0	0.00
Lrrc18	NP_080529.1	498	7	14.81	Olfr1347	NP_666497.1	121	0	0.00
Arhgap22	XP_036014481.1	360	8	23.42	Olfr1348	NP_667124.1	111	0	0.00
Mapk8	NP_001297382.1	3350	1	0.31	Olfr1349	NP_997019.1	119	0	0.00
Ptpn20	XP_006518791.1	580	1	1.82	Olfr1350	NP_666501.1	12	0	0.00
Gdf10	NP_665684.2	781	4	5.40	Zim1	NP_035899.4	232	2	9.09
Gdf2	NP_062379.3	990	2	2.13	Peg3	XP_017177519.1	644	2	3.27
Rbp3	NP_056560.1	414	1	2.55	Usp29	XP_017177905.1	582	2	3.62
Zfp488	XP_036014635.1	415	2	5.08	Aurkc	NP_001074434.1	1322	0	0.00
Gm5460	XP_006519272.1	47	0	0.00	Zfp954	NP_766326.2	158	0	0.00
A630023A22R	NP_001238772.1	0	0	#DIV/0!	Zfp773	NP_083860.1	191	0	0.00
Antxrl	XP_030103650.1	122	0	0.00	Zfp418	NP_666291.2	197	0	0.00
Anxa8	NP_038501.2	405	0	0.00	Zfp772	NP_663552.2	158	0	0.00
Npy4r	NP_032945.3	562	2	3.75	Vmn2r29	NP_001106939.1	67	0	0.00
Gprin2	NP_899032.2	166	3	19.05	Clcn4	XP_030097911.1	425	0	0.00
Syt15	NP_852682.1	263	3	12.02	Vmn2r30	NP_033516.3	28	0	0.00
Shld2	XP_011243520.1	14	0	0.00	Vmn2r31	NP_001371966.1	28	0	0.00
Glud1	NP_032159.1	1413	1	0.75	Vmn2r32	XP_017177619.1	27	0	0.00
Fam25c	XP_030103850.1	67	2	31.46	Vmn2r33	XP_036009207.1	7	0	0.00
Sncg	NP_035560.1	497	4	8.48	Vmn2r34	XP_017177380.1	7	0	0.00
Mmrn2	NP_694767.3	226	2	9.33	Vmn2r35	XP_036009208.1	7	0	0.00
Bmpr1a	XP_036014305.1	1750	4	2.41	Vmn2r36	XP_036008383.1	7	0	0.00
9230112D13R	NP_084338.1	0	0	#DIV/0!	Vmn2r41	XP_017177384.1	7	0	0.00
Ldb3	XP_006519081.1	983	3	3.22	Vmn2r42	XP_017177618.1	71	0	0.00
Opn4	NP_001122071.1	767	7	9.62	Vmn2r43	NP_945199.1	7	0	0.00
Wapl	NP_001004436.2	452	1	2.33	Vmn2r44	NP_001371988.1	7	0	0.00
Grid1	NP_032192.2	716	5	7.36	Vmn2r45	XP_017177383.1	7	0	0.00
4930596D02R	NP_001028938.1	427	0	0.00	Vmn2r40	XP_017177382.1	7	0	0.00
4930474N05R	NP_778173.1	163	0	0.00	Vmn2r39	NP_001372006.1	7	0	0.00
Ccser2	NP_001334431.1	56	0	0.00	Vmn2r38	XP_036009105.1	7	0	0.00
Rgr	NP_067315.1	278	4	15.16	Vmn2r37	NP_033515.2	45	0	0.00
Lrit1	XP_017171501.1	1330	6	4.75	Vmn2r46	NP_001098546.1	7	0	0.00
Lrit2	NP_775594.1	517	4	8.15	Vmn2r47	XP_017177386.1	48	0	0.00
Cdhr1	NP_570948.1	459	6	13.78	Vmn2r48	NP_001098622.1	24	0	0.00
2610528A11R	NP_001193613.1	34	4	123.99	Vmn2r49	XP_017177925.1	7	0	0.00
Ghitm	NP_001346831.1	381	4	11.06	Vmn2r50	XP_036009108.1	7	0	0.00
Nrg3	XP_036014399.1	839	1	1.26	Vmn2r51	XP_017177387.1	139	1	7.58
Sh2d4b	XP_006519199.1	161	5	32.73	Vmn2r52	NP_001098661.1	7	0	0.00
Tspan14	XP_036014646.1	363	3	8.71	Vmn1r66	NP_598991.2	149	0	0.00
Prxl2a	NP_001303669.1	236	2	8.93	Vmn1r67	NP_598990.2	35	0	0.00
Dydc2	NP_081993.1	98	4	43.02	Zik1	NP_033603.2	263	0	0.00
Dydc1	XP_017171668.1	69	3	45.82	Vmn1r68	NP_001165543.1	31	0	0.00
Mat1a	NP_598414.1	1760	4	2.40	Vmn1r69	NP_665841.1	73	0	0.00
Sftpa1	NP_075623.2	359	2	5.87	Vmn1r70	NP_598989.1	165	0	0.00
Mbl1	XP_006518732.1	925	2	2.28	Nlrp4b	NP_001342080.1	314	1	3.36
Sftpd	NP_033186.1	463	1	2.28	Vmn1r71	NP_665847.1	156	0	0.00
Gm2832	NP_001361053.1	0	0	#DIV/0!	Zscan4b	NP_001172102.1	279	0	0.00
Gm5798	XP_017171741.1	0	0	#DIV/0!	Zscan4c	NP_001013787.1	375	0	0.00
Gm7945	NP_001365660.1	0	0	#DIV/0!	Zscan4d	NP_001093656.1	197	0	0.00
Gm6482	NP_001365335.1	0	0	#DIV/0!	Zscan4f	NP_001103786.2	293	0	0.00

Gm7954	NP_001365575.1	0	0	#DIV/0!	Vmn1r72	NP_665842.1	86	0	0.00
Gm7970	XP_036014689.1	1	0	0.00	Vmn1r73	NP_598964.1	232	0	0.00
1700049E17Ri	XP_030103417.1	0	0	#DIV/0!	Vmn1r74	NP_598967.1	130	0	0.00
Gm3072	NP_001365363.1	0	0	#DIV/0!	Vmn1r75	NP_598968.1	95	0	0.00
Gm8068	NP_001371168.1	0	0	#DIV/0!	Vmn1r76	NP_598966.2	34	0	0.00
1700049E17Ri	XP_036014694.1	0	0	#DIV/0!	Vmn1r77	NP_001160203.1	77	0	0.00
Gm7980	XP_036014692.1	0	0	#DIV/0!	Vmn1r78	NP_598969.2	78	0	0.00
Gm17027	XP_036014888.1	0	0	#DIV/0!	Vmn1r79	NP_001160307.1	2	0	0.00
Gm17026	NP_001365643.1	0	0	#DIV/0!	Vmn1r80	NP_598965.2	205	0	0.00
Gm7995	XP_017171743.1	1	0	0.00	Vmn1r81	NP_598971.1	128	0	0.00
Gm8005	XP_036014695.1	0	0	#DIV/0!	Vmn1r82	NP_598995.1	62	0	0.00
Gm8020	NP_001365662.1	15	0	0.00	Vmn1r83	XP_017177495.1	36	0	0.00
Gm8032	XP_011243580.1	0	0	#DIV/0!	Vmn1r84	NP_598994.1	52	0	0.00
Gm17124	NP_001365664.1	0	0	#DIV/0!	Zfp551	NP_001028992.2	203	0	0.00
Gm10377	NP_001231600.1	36	0	0.00	Zfp606	NP_080388.2	272	0	0.00
Gm21977	XP_036014221.1	0	0	#DIV/0!	2900092C05R	NP_082710.2	119	0	0.00
Gm10376	NP_001371114.1	5	0	0.00	Vmn2r53	XP_036009215.1	11	0	0.00
Gm8094	XP_036014890.1	0	0	#DIV/0!	Vmn2r54	NP_001371875.1	35	0	0.00
Gm8104	XP_011243570.1	0	0	#DIV/0!	Vmn2r55	NP_001361579.2	35	0	0.00
Gm3141	XP_036014891.1	0	0	#DIV/0!	Vmn2r56	XP_036009214.1	15	0	0.00
Gm8126	XP_036014285.1	0	0	#DIV/0!	Zscan18	XP_006539854.1	254	4	16.60
Gm8127	NP_001365644.1	0	0	#DIV/0!	Zfp329	XP_036009273.1	225	1	4.68
1700001F09Ri	NP_082216.1	57	0	0.00	Zfp110	NP_001347505.1	461	2	4.57
Gm8138	NP_001365663.1	0	0	#DIV/0!	Zfp128	NP_722497.1	334	1	3.16
Gm21154	XP_017171826.2	0	0	#DIV/0!	Zscan22	NP_001277369.1	253	0	0.00
Gm21162	XP_036014898.1	0	0	#DIV/0!	Rps5	XP_036008703.1	1670	1	0.63
Gm5799	XP_017171755.1	0	0	#DIV/0!	Rnf225	NP_084085.1	251	0	0.00
Gm10375	XP_017171219.2	0	0	#DIV/0!	Zfp324	NP_848847.3	231	4	18.25
Gm8165	NP_001365645.1	0	0	#DIV/0!	Zfp446	XP_006540088.1	226	3	13.99
Gm16506	NP_001365658.1	0	0	#DIV/0!	Slc27a5	NP_033538.2	929	0	0.00
Gm6526	XP_036014899.1	0	0	#DIV/0!	Zbtb45	NP_001347762.1	223	0	0.00
Ear1	NP_031920.1	181	0	0.00	Trim28	NP_035718.2	1437	5	3.67
Ear10	NP_444342.1	197	0	0.00	Chmp2a	NP_001347662.1	775	1	1.36
Ang5	NP_031474.2	234	0	0.00	Ube2m	NP_663553.1	889	2	2.37
Ang6	NP_001011876.1	174	0	0.00	Mzf1	XP_006539529.1	525	0	0.00
Ear2	NP_031921.1	333	0	0.00	Vmn1r85	NP_665846.1	94	0	0.00
Gm3327	NP_001371031.1	3	0	0.00	Vmn1r86	NP_001161008.1	55	0	0.00
4930503E14Ri	NP_083407.1	8	0	0.00	Vmn1r87	NP_598988.1	66	0	0.00
Gm8220	XP_036014704.1	0	0	#DIV/0!	Vmn1r88	NP_001161009.1	23	0	0.00
Gm5930	NP_001361020.1	0	0	#DIV/0!	Vmn1r89	NP_598987.1	260	0	0.00
Gm8229	NP_001365648.1	0	0	#DIV/0!	Zswim9	NP_796286.2	45	10	234.20
Gm3371	XP_030104076.1	0	0	#DIV/0!	Lig1	NP_001186239.2	1584	6	3.99
Gm8232	NP_001365651.1	0	0	#DIV/0!	Pla2g4c	XP_036008837.1	327	9	29.01
BC061237	NP_941079.1	123	0	0.00	Cabp5	XP_017177786.1	1059	13	12.94
Gm8256	XP_036014901.1	0	0	#DIV/0!	Bsph1	NP_001028590.1	154	13	88.97
Gm8267	NP_001156426.1	0	0	#DIV/0!	Bsph2	NP_001074411.1	75	13	182.68
Ptgdr	NP_032988.3	439	6	14.40	Sult2a5	NP_001171909.1	176	7	41.92
Ptger2	NP_032990.1	554	8	15.22	Sult2a2	NP_033312.2	269	9	35.26
Txndc16	XP_030103874.1	809	8	10.42	Sult2a1	NP_001104766.1	462	11	25.09
Gpr137c	NP_081794.2	134	8	62.92	Sult2a4	XP_006540226.1	265	5	19.88
Ero1a	NP_056589.1	589	7	12.53	Sult2a3	NP_001095056.2	188	7	39.24
Psmc6	NP_080235.2	2173	10	4.85	Sult2a6	XP_006540331.1	151	7	48.86
Styx	NP_062611.2	558	9	17.00	Sult2a8	NP_780459.1	254	5	20.75
Gnpnat1	XP_036014653.1	622	4	6.78	Sult2a7	NP_001171910.2	249	6	25.40
Fermt2	NP_666166.2	660	8	12.77	Vmn1r90	NP_001230960.1	45	0	0.00
Ddhd1	NP_001034195.2	437	9	21.71	Obox7	NP_001033765.1	74	2	28.48
Bmp4	NP_031580.2	1914	4	2.20	Obox2	NP_663754.2	190	2	11.09
Cdkn3	NP_001346970.1	1127	5	4.68	Obox1	NP_082078.1	210	1	5.02
Cnih1	NP_034049.2	462	8	18.25	Obox3	NP_663753.3	183	1	5.76
Gmfb	NP_071306.2	416	5	12.67	Obox5	NP_663755.2	259	1	4.07
Cgrrf1	NP_081108.2	205	3	15.42	Obox6	NP_663756.2	173	1	6.09
Samd4	XP_036014776.1	560	5	9.41	Crx	NP_001106801.1	685	5	7.69
Gch1	NP_032128.1	867	0	0.00	Crxos	NP_001028810.1	142	8	59.37
Wdhd1	NP_766186.2	787	4	5.36	Selenow	NP_033182.1	245	4	17.21
Socs4	NP_543119.2	384	2	5.49	Nop53	NP_598592.2	854	3	3.70
Mapk1ip1l	NP_848799.1	106	4	39.77	Ehd2	NP_694708.2	584	0	0.00

Lgals3	NP_001139425.1	1370	2	1.54	Bicra	XP_030098415.1	181	1	5.82
Dlgap5	XP_006518896.1	821	3	3.85	Zfp541	XP_006540358.1	727	4	5.80
Fbxo34	XP_006519786.2	155	1	6.80	Napa	NP_080174.1	525	1	2.01
Atg14	NP_766187.1	432	1	2.44	Kptn	NP_598488.2	227	0	0.00
Ktn1	XP_006518695.1	707	4	5.96	Slc8a2	NP_683748.1	1093	1	0.96
Peli2	XP_006519805.1	316	1	3.34	Meis3	XP_036008636.1	251	0	0.00
Tmem260	NP_766188.3	23	1	45.82	Dhx34	XP_017167800.1	1037	2	2.03
Otx2	XP_017171406.1	1116	2	1.89	C5ar2	XP_011248896.1	250	1	4.22
Exoc5	XP_017171255.1	843	4	5.00	C5ar1	NP_001167021.1	872	2	2.42
Ap5m1	NP_001346999.1	550	5	9.58	Inafm1	NP_001129742.1	12	0	0.00
Naa30	XP_006519605.1	298	6	21.22	Ccdc9	XP_030098416.1	76	0	0.00
ccdc198	XP_006519502.1	87	4	48.46	Bbc3	XP_006539638.1	125	0	0.00
Slc35f4	NP_083514.1	168	7	43.91	Sae1	NP_001272821.1	1274	5	4.14
Armh4	NP_001345173.1	52	4	81.07	Zc3h4	XP_036009070.1	562	2	3.75
Olfr722	NP_666705.1	81	0	0.00	Tmem160	NP_081214.1	366	3	8.64
Olfr723	NP_001011530.2	76	1	13.87	Npas1	XP_006539677.1	452	1	2.33
Olfr724	NP_666703.2	336	2	6.27	Arhgap35	XP_036008839.1	688	7	10.72
Olfr725	XP_017171512.1	68	0	0.00	Ceacam15	NP_780524.1	20	0	0.00
Olfr726	NP_666428.2	67	0	0.00	Ceacam9	NP_036057.1	20	0	0.00
Olfr727	NP_666431.2	113	0	0.00	Ap2s1	NP_941015.2	781	4	5.40
Olfr728	NP_001011809.1	346	2	6.09	Slc1a5	NP_033227.2	584	0	0.00
Olfr729	NP_666390.1	48	0	0.00	Fkrp	NP_001345775.1	311	5	16.94
Olfr730	NP_666704.2	65	0	0.00	Strn4	NP_598550.2	551	7	13.39
Olfr731	NP_666475.2	46	0	0.00	Prkd2	NP_849231.1	365	4	11.55
Olfr732	NP_666876.1	53	0	0.00	Dact3	NP_001075124.1	471	6	13.43
Olfr733	NP_666874.2	69	0	0.00	Gng8	XP_036008576.1	806	3	3.92
Olfr734	NP_666875.1	69	0	0.00	Ptgir	XP_006539733.1	503	2	4.19
Olfr735	NP_001011754.1	44	0	0.00	Calm3	NP_031616.1	2215	3	1.43
Tlr11	NP_991388.2	669	0	0.00	Pnmal2	NP_001093106.1	1816	2	1.16
Olfr736	NP_666877.1	22	0	0.00	Pnmal1	NP_001007570.1	748	2	2.82
Olfr738	NP_666532.2	52	1	20.27	Ccdc8	NP_001095005.1	394	5	13.37
Olfr739	NP_666879.2	93	0	0.00	Ppp5c	NP_035285.2	2471	6	2.56
Olfr740	NP_666878.2	94	0	0.00	Hif3a	NP_001156422.1	629	1	1.68
Olfr741	NP_997016.2	51	0	0.00	Psg16	XP_030098466.1	298	7	24.76
Olfr742	NP_666641.2	55	0	0.00	Ceacam3	NP_473400.1	320	8	26.35
Olfr743	NP_001170979.1	231	0	0.00	Psg29	NP_473405.1	296	7	24.92
Olfr744	NP_001011738.1	100	0	0.00	Ceacam5	NP_082756.1	400	10	26.35
Olfr745	NP_666411.2	26	0	0.00	Ceacam14	NP_080233.1	279	9	34.00
Olfr746	NP_666410.2	111	0	0.00	Gm5155	NP_001365613.1	80	7	92.22
Olfr747	NP_997039.2	28	0	0.00	Ceacam11	NP_075778.2	283	11	40.96
Olfr748	NP_001011837.2	32	0	0.00	Ceacam13	NP_081486.1	308	12	41.06
Olfr749	NP_064684.2	41	0	0.00	Ceacam12	NP_001155995.1	304	12	41.60
Ttc5	NP_001074418.1	147	0	0.00	Igfl3	NP_001003393.1	30	0	0.00
Ccnb1ip1	NP_001104589.1	317	2	6.65	Mill1	NP_715630.3	115	0	0.00
Parp2	NP_033762.1	967	4	4.36	Psg18	XP_006540069.1	300	7	24.59
Tep1	NP_033377.1	1525	1	0.69	Psg28	NP_473404.3	250	5	21.08
Klhl33	NP_001357693.1	242	0	0.00	Psg26	XP_006540301.1	257	6	24.60
Osgep	NP_598437.2	656	3	4.82	Psg25	NP_473401.1	272	4	15.50
Apex1	NP_033817.1	1450	4	2.91	Psg27	NP_001032245.1	269	4	15.67
Pip4p1	NP_001297434.1	192	2	10.98	Psg23	NP_064657.2	306	3	10.33
Pnp	NP_038660.1	586	1	1.80	Psg21	NP_081679.2	310	2	6.80
Pnp2	NP_001116843.1	560	3	5.65	Psg20	XP_030098617.1	423	1	2.49
Rnase10	NP_083421.2	179	2	11.78	Psg22	XP_017177722.1	257	0	0.00
Rnase9	NP_898853.2	66	2	31.94	Psg19	NP_036094.2	286	0	0.00
Rnase11	XP_006519285.2	62	9	152.99	Psg17	NP_031703.1	351	0	0.00
Rnase12	NP_001011875.1	12	5	439.13	Mill2	XP_017177723.1	345	1	3.05
Olfr750	NP_997441.2	35	0	0.00	Pglyrp1	NP_033428.1	490	0	0.00
Ang	NP_001155203.1	703	5	7.50	Ccdc61	XP_036008840.1	138	0	0.00
Rnase4	NP_067447.2	280	8	30.11	Nova2	NP_001025048.2	510	1	2.07
Eddm3b	NP_987104.1	56	0	0.00	Nanos2	NP_918953.2	624	0	0.00
Rnase6	NP_001347046.1	356	5	14.80	Mypop	NP_663554.1	39	0	0.00
Rnase1	NP_035401.2	1498	6	4.22	Irf2bp1	NP_848872.2	286	2	7.37
Rnase2b	NP_062271.2	214	5	24.62	Foxa3	NP_032286.1	995	1	1.06
Ang2	NP_031475.2	202	3	15.65	Sympk	NP_080881.2	868	6	7.29
Ear14	NP_059085.2	329	6	19.22	Rsph6a	NP_112545.2	258	5	20.42
Rnase2a	NP_444343.2	270	4	15.61	Dmwd	NP_034188.2	836	7	8.82

Gm7247	XP_036014908.1	0	0	#DIV/0!	Dmpk	NP_001366186.1	1292	6	4.89
Vmn2r88	XP_006519463.1	52	0	0.00	Six5	NP_035513.1	386	4	10.92
Vmn2r89	XP_036014470.1	118	0	0.00	Meiosin	NP_001357741.1	91	1	11.58
Gm5622	NP_001013838.1	0	0	#DIV/0!	Fbxo46	NP_780739.1	123	3	25.70
Gm17175	XP_017171763.1	0	0	#DIV/0!	Qpctl	NP_080387.2	210	5	25.09
Gm17078	XP_036014907.1	0	0	#DIV/0!	Snrpd2	XP_030097829.1	1664	6	3.80
Gm17079	XP_036014905.1	0	0	#DIV/0!	Gipr	NP_001074284.1	475	2	4.44
Gm5800	NP_001029274.1	131	0	0.00	Eml2	NP_001334511.1	886	7	8.33
Ang4	NP_808212.2	229	0	0.00	Gpr4	NP_783599.1	341	0	0.00
AY358078	NP_919328.1	28	0	0.00	Opa3	NP_997408.2	266	8	31.70
Ear6	NP_444341.1	203	0	0.00	Vasp	NP_001268951.1	939	5	5.61
Mettl17	NP_001025161.1	327	0	0.00	Ppm1n	XP_017177640.1	722	9	13.14
Slc39a2	NP_001034765.2	348	0	0.00	Rtn2	NP_038676.1	674	9	14.07
Ndrg2	NP_001347199.1	478	0	0.00	Fosb	NP_032062.1	905	5	5.82
Tppp2	NP_001122106.1	361	2	5.84	Ercc1	NP_031974.2	775	7	9.52
Rnase13	XP_017171565.1	55	2	38.32	Cd3eap	NP_665821.1	223	6	28.36
Arhgef40	XP_006519104.1	390	4	10.81	Ppp1r13l	XP_030098560.1	233	6	27.14
Zfp219	XP_017171669.1	505	2	4.17	Ercc2	NP_031975.2	935	9	10.14
Tmem253	XP_017171594.1	52	0	0.00	Klc3	XP_030098293.1	962	3	3.29
Olfr221	NP_001001808.2	21	0	0.00	Ckm	NP_031736.1	773	4	5.45
Hnrnpc	NP_001347112.1	1978	4	2.13	Mark4	NP_758483.1	426	6	14.84
Rpgrip1	XP_006519752.1	319	0	0.00	Exoc3l2	NP_001357729.1	442	4	9.54
Supt16	NP_291096.2	1332	9	7.12	Bloc1s3	NP_808360.2	152	5	34.67
Chd8	NP_963999.2	1690	11	6.86	Trappc6a	NP_001318110.1	343	8	24.58
Rab2b	NP_766189.1	1519	10	6.94	Nkpd1	XP_006540413.2	829	8	10.17
Tox4	NP_075923.2	676	11	17.15	Ppp1r37	NP_954600.1	122	8	69.11
Mettl3	NP_062695.2	435	12	29.07	Gemin7	NP_081465.1	272	6	23.25
Sall2	NP_056587.2	324	12	39.03	Zfp296	NP_071854.2	328	5	16.07
Olfr1513	NP_001012269.2	34	6	185.98	Clasrp	XP_030098632.1	367	6	17.23
Olfr1512	NP_666643.1	125	6	50.59	Relb	NP_001277386.1	1075	5	4.90
Olfr1511	NP_666383.2	123	6	51.41	Clptm1	NP_062623.2	277	9	34.24
Olfr1510	NP_666642.2	103	6	61.39	Apoc2	NP_001264873.1	648	8	13.01
Olfr1509	NP_065260.2	108	0	0.00	Apoc4	NP_031411.1	323	4	13.05
Olfr1508	NP_065259.1	49	0	0.00	Apoc1	NP_001103479.1	722	11	16.06
Olfr1507	NP_065258.2	130	0	0.00	Apoe	NP_033826.2	2063	10	5.11
Dad1	NP_001106829.1	700	4	6.02	Tomm40	NP_058567.2	1219	10	8.65
Abhd4	NP_598837.3	404	3	7.83	Nectin2	NP_033016.3	461	10	22.86
Olfr49	NP_035121.1	82	0	0.00	Bcam	NP_065232.1	184	6	34.37
Oxa1l	NP_081212.1	1074	1	0.98	Cblc	NP_001155316.1	588	2	3.58
Slc7a7	XP_030103562.1	465	0	0.00	Bcl3	XP_006539550.1	1677	7	4.40
Mrpl52	NP_081127.1	412	3	7.67	Gm19345	NP_001257418.1	0	0	#DIV/0!
Mmp14	NP_032634.3	915	0	0.00	Ceacam16	NP_001028591.2	269	5	19.59
Lrp10	NP_075369.2	341	0	0.00	Ceacam19	XP_006540126.1	82	6	77.11
Rem2	NP_542764.2	1422	1	0.74	Pvr	NP_081790.1	452	4	9.33
Prmt5	NP_038796.2	1657	4	2.54	Igsf23	NP_001350953.1	64	3	49.40
Haus4	NP_663437.2	274	7	26.92	Ceacam20	XP_006540423.1	120	2	17.57
Ajuba	NP_034720.2	863	5	6.11	Nlrp9b	NP_918947.2	218	0	0.00
4931414P19R	XP_036014773.1	39	3	81.07	Vmn1r91	NP_001160208.1	28	0	0.00
Psmb5	NP_035316.1	1331	8	6.33	Vmn1r240	NP_001160620.1	146	0	0.00
Psmb11	NP_780413.1	766	3	4.13	Vmn1r93	NP_997430.2	100	0	0.00
Cdh24	XP_030103653.1	210	1	5.02	Vmn1r94	NP_001160195.1	309	0	0.00
Acin1	XP_030103753.1	669	5	7.88	Vmn1r95	NP_001161010.1	43	0	0.00
1700123O20R	NP_067412.1	94	2	22.42	Vmn1r241	NP_001160625.1	176	0	0.00
Cebpe	NP_997014.1	416	0	0.00	Vmn1r242	NP_001160623.1	76	0	0.00
Slc7a8	NP_058668.1	448	1	2.35	Vmn1r243	NP_001160630.1	49	0	0.00
Homez	XP_017171503.1	156	0	0.00	Vmn1r244	NP_001160185.1	133	0	0.00
Ppp1r3e	NP_001161380.1	253	0	0.00	Vmn1r100	NP_001160316.1	1	0	0.00
Bcl2l2	XP_006518520.1	567	1	1.86	Vmn1r101	NP_001160308.1	47	0	0.00
Pabpn1	NP_062275.1	1048	2	2.01	Gm10670	NP_001160633.1	25	0	0.00
Slc22a17	NP_001347335.1	745	3	4.24	Vmn1r103	NP_001160209.1	112	0	0.00
Efs	XP_036014332.1	202	0	0.00	Vmn1r104	NP_001160210.1	1	0	0.00
Il25	NP_542767.1	169	0	0.00	Vmn1r245	NP_001160618.1	83	0	0.00
Cmtm5	NP_080342.1	353	1	2.99	Vmn1r246	NP_001160635.1	42	0	0.00
Myh6	XP_036014396.1	1617	0	0.00	Gm4513	NP_001311480.1	105	0	0.00
Myh7	XP_017171330.1	1518	0	0.00	Vmn1r107	NP_001160231.1	42	0	0.00
Ngdn	NP_081166.1	895	0	0.00	Vmn1r111	NP_001160218.1	32	0	0.00

Zfhx2	XP_017171504.1	130	0	0.00	Gm10668	NP_001357829.1	166	0	0.00
Thtpa	NP_694723.1	144	0	0.00	Vmn1r112	NP_001160319.1	39	0	0.00
Ap1g2	NP_031481.2	586	0	0.00	Vmn1r113	NP_001160188.1	90	0	0.00
Jph4	NP_796023.2	750	2	2.81	Vmn1r114	NP_001160309.1	141	0	0.00
Dhrs2	NP_082066.2	420	1	2.51	Vmn1r115	NP_001160217.1	34	0	0.00
Dhrs4	NP_001033027.2	677	2	3.11	Vmn1r116	NP_001160216.1	31	0	0.00
Carmil3	XP_006519110.1	250	0	0.00	Vmn1r117	NP_001160215.1	192	0	0.00
Cpne6	NP_001347122.1	918	1	1.15	Vmn1r118	NP_001160214.1	59	0	0.00
Nrl	NP_001129546.1	558	1	1.89	Vmn1r119	NP_001160180.1	48	0	0.00
Pck2	NP_083270.2	740	2	2.85	Vmn1r120	NP_001160187.1	79	0	0.00
Dcaf11	XP_030103706.1	706	4	5.97	Vmn1r121	NP_001160213.1	2	0	0.00
Fitm1	NP_081084.1	336	3	9.41	Vmn1r122	NP_001160186.1	90	0	0.00
Psme1	NP_035319.1	699	5	7.54	Vmn1r123	NP_001160179.1	79	0	0.00
Emc9	XP_006519802.1	247	5	21.33	Gm5157	XP_011249060.1	106	0	0.00
Psme2	NP_001025026.1	648	4	6.51	Vmn1r124	NP_001160229.1	50	0	0.00
Rnf31	NP_919327.2	460	5	11.46	Vmn1r125	NP_001160212.1	42	0	0.00
Irf9	NP_001152889.1	557	4	7.57	Vmn1r126	NP_001160310.1	253	0	0.00
Rec8	NP_064386.2	965	2	2.18	Vmn1r127	NP_001160198.1	107	0	0.00
Ipo4	NP_001347007.1	1146	3	2.76	Vmn1r128	NP_001160211.1	34	0	0.00
Tm9sf1	NP_001346992.1	286	1	3.68	Vmn1r129	NP_001160197.1	326	0	0.00
Tssk4	XP_036014743.1	1142	1	0.92	Gm6882	XP_017167971.1	105	0	0.00
Mdp1	NP_075886.1	52	0	0.00	Vmn1r130	NP_001160320.1	40	0	0.00
Nedd8	NP_032709.1	2769	5	1.90	Vmn1r131	NP_001160311.1	32	0	0.00
Gmpr2	XP_011243184.1	957	3	3.30	Vmn1r132	NP_001116154.1	94	0	0.00
Tinf2	XP_006519143.1	313	1	3.37	Gm8600	XP_011249061.1	106	0	0.00
Tgm1	NP_064368.3	445	3	7.10	Vmn1r247	NP_001160637.1	26	0	0.00
Rabggta	NP_001347317.1	421	2	5.01	Vmn1r248	NP_001160639.1	168	1	6.27
Dhrs1	XP_030103746.1	438	2	4.81	Vmn1r135	NP_001160219.1	50	0	0.00
Nop9	NP_080679.3	529	4	7.97	Vmn1r249	NP_001160183.1	376	1	2.80
Cideb	NP_034024.2	715	6	8.84	Gm5891	NP_001030076.1	109	0	0.00
Ltb4r2	XP_036014662.1	426	6	14.84	Vmn1r137	NP_001160321.1	40	0	0.00
Ltb4r1	NP_032545.1	566	5	9.31	Vmn1r138	NP_001160641.1	32	0	0.00
Adcy4	NP_001348533.1	1110	4	3.80	Gm6176	XP_017167972.1	144	0	0.00
Ripk3	NP_064339.2	1279	3	2.47	Vmn1r139	NP_001160220.1	112	0	0.00
Nfatc4	NP_076188.3	481	3	6.57	Gm16451	NP_001160621.1	149	4	28.29
Nynrin	NP_001035161.1	472	6	13.40	Vmn1r250	NP_001161045.1	98	1	10.75
Cbln3	NP_062794.1	198	0	0.00	Vmn1r142	NP_001160221.1	79	0	0.00
Khnyn	NP_081419.1	199	5	26.48	Vmn1r143	NP_001160222.1	43	0	0.00
Sdr39u1	NP_001076444.1	148	15	106.81	Vmn1r251	NP_001160624.1	175	0	0.00
Cma1	NP_034910.2	552	6	11.46	Vmn1r252	NP_001160622.1	76	1	13.87
Cma2	NP_001348566.1	135	3	23.42	Vmn1r253	NP_001160631.1	49	0	0.00
Mcpt1	NP_032596.1	162	0	0.00	Vmn1r254	NP_001160223.1	135	0	0.00
Mcpt2	XP_011243282.1	138	1	7.64	Vmn1r148	NP_109661.1	2	0	0.00
Mcpt9	NP_034912.3	123	0	0.00	Vmn1r149	NP_001160312.1	69	0	0.00
Mcpt4	NP_034909.2	228	2	9.24	Gm10665	NP_001160632.1	25	0	0.00
Mcpt8	NP_032598.1	301	10	35.01	Vmn1r151	NP_001160184.1	170	0	0.00
Ctsg	NP_031826.1	841	5	6.27	Vmn1r152	NP_001160224.1	0	0	#DIV/0!
Gzme	NP_034503.2	253	6	24.99	Vmn1r255	NP_001160619.1	83	0	0.00
Gzmd	XP_006518632.2	228	8	36.98	Vmn1r256	NP_001160634.1	42	0	0.00
Gzmg	NP_034505.1	233	2	9.05	Gm4565	NP_001311481.1	105	0	0.00
Gzmn	NP_694692.1	206	3	15.35	Vmn1r155	NP_001160225.1	42	0	0.00
Gzmf	NP_034504.1	261	4	16.15	Vmn1r257	NP_001160626.1	21	0	0.00
Gzmc	XP_006518631.1	276	3	11.46	Gm4567	NP_001032325.1	150	0	0.00
Gzmb	NP_038570.1	917	7	8.05	Vmn1r157	NP_001160226.1	22	0	0.00
Atp12a	NP_619593.2	1189	2	1.77	Vmn1r158	NP_001160313.1	2	0	0.00
Rnf17	NP_001028215.1	1171	13	11.70	Vmn1r159	NP_001160230.1	47	0	0.00
Cenpj	XP_006518910.1	786	1	1.34	Vmn1r160	NP_001160196.1	89	0	0.00
Parp4	NP_001139450.2	712	6	8.88	Gm8708	XP_011249063.1	106	0	0.00
Mphosph8	NP_001347001.1	2850	6	2.22	Vmn1r258	NP_001160636.1	26	0	0.00
Pspc1	NP_079958.3	642	3	4.92	Vmn1r259	NP_001160638.1	173	0	0.00
Zmym5	XP_017171456.1	210	5	25.09	Vmn1r163	NP_001160227.1	51	0	0.00
Zmym2	XP_011243521.1	817	6	7.74	Vmn1r260	NP_001160228.1	329	0	0.00
B020004C17R	NP_001242989.1	4	0	0.00	Gm10662	NP_001188293.1	109	0	0.00
Gja3	NP_001258552.1	428	4	9.85	Vmn1r165	NP_001160322.1	42	0	0.00
Gjb2	NP_032151.1	803	3	3.94	Vmn1r166	NP_001160640.1	32	0	0.00
Gjb6	XP_030103511.1	616	3	5.13	Gm6902	NP_001257423.1	111	0	0.00

Cryl1	NP_084280.2	545	4	7.74	Nlrp4e	NP_001004194.2	190	1	5.55
Ift88	NP_033402.2	1359	2	1.55	Nlrp5	NP_035990.1	240	1	4.39
Il17d	NP_665836.2	281	1	3.75	Vmn1r167	NP_001095032.1	204	0	0.00
Eef1akmt1	NP_080802.2	186	5	28.33	Vmn1r168	NP_001160314.1	37	0	0.00
Xpo4	NP_065252.1	338	0	0.00	Vmn1r169	NP_001160315.1	56	0	0.00
Lats2	NP_056586.2	680	0	0.00	Vmn1r170	NP_001160194.1	164	0	0.00
Sap18	NP_033145.2	656	0	0.00	Vmn1r171	NP_109662.1	38	0	0.00
Ska3	XP_006518922.3	496	1	2.12	Vmn1r172	NP_109660.1	38	0	0.00
Mrpl57	NP_080677.1	364	3	8.69	Vmn1r173	NP_001160190.1	211	0	0.00
Zdhhc20	XP_030103950.1	306	3	10.33	Vmn1r174	NP_997431.2	78	0	0.00
Micu2	XP_006519547.1	183	3	17.28	Vmn1r175	NP_001160199.1	89	0	0.00
Fgf9	XP_030103508.1	885	2	2.38	Vmn1r176	NP_001160193.1	200	0	0.00
1700129C05R	NP_001361688.1	3	0	0.00	Vmn1r177	NP_996755.2	147	0	0.00
Gm5142	NP_001004158.1	257	0	0.00	Vmn1r178	NP_996751.1	39	0	0.00
Rcbtb1	XP_017171693.1	656	8	12.85	Vmn1r179	NP_997428.1	30	0	0.00
Phf11a	XP_017171459.1	819	6	7.72	Vmn1r180	NP_996752.2	86	0	0.00
Phf11b	NP_001157799.1	650	4	6.49	Vmn1r181	NP_997429.2	47	0	0.00
Phf11d	NP_950180.3	847	6	7.47	V1rd19	NP_997502.2	0	0	#DIV/0!
Phf11c	NP_001157761.1	765	5	6.89	Vmn1r183	NP_987074.1	0	0	#DIV/0!
Setdb2	NP_001307650.1	912	8	9.24	Zfp180	NP_001038951.1	227	0	0.00
Cab39l	NP_001347520.1	562	7	13.13	Zfp112	XP_036009176.1	182	0	0.00
Cdadc1	NP_001162009.1	227	6	27.86	Zfp235	XP_036009165.1	283	0	0.00
Shisa2	NP_663438.1	217	0	0.00	Zfp114	NP_001025104.2	319	0	0.00
Atp8a2	NP_056618.1	844	5	6.24	Zfp111	XP_030098675.1	162	0	0.00
Nupl1	NP_001349369.1	359	1	2.94	Zfp109	XP_030098682.1	188	0	0.00
Mtmr6	XP_017171460.1	418	3	7.56	Zfp108	XP_017177861.1	139	0	0.00
Amer2	NP_082389.2	229	2	9.20	Zfp93	NP_001347156.1	197	0	0.00
Spata13	XP_036014454.1	456	3	6.93	Zfp61	NP_033587.2	237	0	0.00
C1qtnf9	NP_898998.2	172	2	12.25	Zfp94	NP_001186250.1	244	0	0.00
Mipep	NP_081712.2	540	6	11.71	Tescl	NP_001157282.1	1065	1	0.99
Tnfrsf19	NP_038897.4	335	3	9.44	Lypd5	NP_084082.1	180	2	11.71
Sacs	NP_766397.2	426	2	4.95	Kcnn4	NP_032459.3	567	1	1.86
Sgcg	XP_036014490.1	418	4	10.09	Smg9	NP_082323.1	250	0	0.00
Arl11	NP_796311.2	1491	7	4.95	Irgc1	NP_001344962.1	128	1	8.23
Ebpl	NP_080874.2	261	7	28.27	Plaur	NP_035243.1	915	4	4.61
Kpna3	NP_032492.1	1187	8	7.10	Cadm4	NP_694752.1	379	0	0.00
Spryd7	NP_001297532.1	127	6	49.79	Zfp428	XP_006539902.1	85	0	0.00
Trim13	NP_075722.1	573	6	11.04	Irgq	NP_694774.3	94	1	11.21
Kcnrg	NP_001034194.1	341	7	21.63	Pinlyp	XP_006540337.1	109	7	67.68
Dleu7	NP_775595.1	321	9	29.55	Xrcc1	NP_033558.3	688	1	1.53
Rnaseh2b	NP_080277.1	472	5	11.16	Zfp575	XP_006539509.1	196	7	37.64
Gucy1b2	XP_011243363.2	836	4	5.04	Ethe1	NP_075643.1	415	9	22.86
Fam124a	NP_001230786.1	55	3	57.49	Phldb3	XP_017177646.1	68	6	92.99
Serpine3	NP_001186874.1	132	1	7.98	Lypd3	NP_598504.1	344	9	27.57
Ints6	NP_032741.2	500	4	8.43	Tex101	NP_064365.1	352	10	29.94
Wdfy2	NP_780755.2	1735	2	1.21	Lypd10	XP_006539905.1	107	8	78.80
Defb48	NP_001032840.1	75	0	0.00	Lypd11	NP_001349080.1	105	7	70.26
Defb47	XP_017171607.1	65	3	48.64	Cd177	NP_081138.2	372	6	17.00
Defb43	NP_001034210.1	89	1	11.84	Ceacam10	NP_031701.3	249	2	8.47
Defb30	NP_001034655.1	83	3	38.09	Lypd4	NP_877586.1	293	4	14.39
Defb42	XP_030103767.1	31	1	34.00	Dmrtc2	XP_006540421.1	231	4	18.25
Ctsb	NP_031824.1	1356	4	3.11	Rps19	NP_001347045.1	1219	1	0.86
Fdft1	XP_036014350.1	959	6	6.59	Cd79a	NP_031681.2	586	3	5.40
Neil2	XP_006519264.1	230	5	22.91	Arhgef1	XP_017177492.1	797	2	2.64
Gata4	NP_032118.2	1855	5	2.84	Rabac1	NP_034391.1	619	2	3.41
Blk	NP_031575.2	1208	3	2.62	Atp1a3	NP_001277398.1	1164	3	2.72
Fam167a	NP_808296.2	214	5	24.62	Grik5	XP_030098002.1	593	0	0.00
Tdh	NP_067455.5	416	1	2.53	Zfp574	XP_006539913.1	249	6	25.40
Mtmr9	NP_808262.1	427	9	22.21	Pou2f2	XP_006539719.1	507	3	6.24
Xkr6	NP_775569.2	165	4	25.55	Dedd2	NP_997560.3	272	0	0.00
Pinx1	NP_082504.1	660	5	7.98	Zfp526	XP_006539776.1	292	8	28.87
Sox7	NP_035576.1	566	5	9.31	Gsk3a	XP_006540322.1	2778	3	1.14
4930578I06Ril	NP_080635.2	117	4	36.03	Erf	NP_034285.3	346	0	0.00
Rp1l1	NP_666358.2	298	4	14.15	Cic	XP_036009331.1	213	4	19.79
Prss55	XP_036014741.1	261	2	8.08	Pafah1b3	XP_017177516.1	372	11	31.16
Prss51	XP_006518369.1	126	0	0.00	Prr19	XP_017177922.1	24	5	219.56

Prss52	NP_082801.2	119	0	0.00	Tmem145	NP_899134.2	629	10	16.76
Msra	NP_001240643.1	679	5	7.76	Megf8	NP_001153872.1	301	11	38.51
Kif13b	NP_001074646.1	894	1	1.18	Cnfn	NP_082495.1	264	8	31.94
Hmbox1	XP_011243339.1	222	0	0.00	Lipe	XP_006539633.2	767	7	9.62
Ints9	NP_700463.3	469	0	0.00	Cxcl17	NP_705804.2	179	0	0.00
Extl3	NP_001347314.1	254	0	0.00	Ceacam1	NP_036056.2	603	7	12.23
Fzd3	NP_067433.1	654	2	3.22	Ceacam2	NP_001106840.1	361	7	20.44
Fbxo16	NP_056610.1	444	2	4.75	Erich4	NP_001034332.3	21	0	0.00
Zfp395	NP_950194.2	196	2	10.75	Dmac2	NP_001277416.1	233	4	18.09
Pnoc	NP_001192004.1	537	1	1.96	B3gnt8	NP_666296.1	88	1	11.98
Elp3	NP_083087.1	836	2	2.52	Bckdha	NP_031559.3	677	6	9.34
Nuggc	XP_006518365.2	45	0	0.00	Exosc5	NP_613052.1	1071	2	1.97
Scara5	XP_036014744.1	343	1	3.07	Tmem91	XP_006540136.1	110	5	47.90
Pbk	NP_075698.1	1844	2	1.14	B9d2	NP_001349083.1	428	1	2.46
Esco2	NP_082315.3	812	3	3.89	Tgfb1	NP_035707.1	1447	4	2.91
Ccdc25	NP_666056.1	147	2	14.34	Ccdc97	NP_083047.1	142	5	37.11
Scara3	NP_766192.1	332	1	3.17	Hnrnpul1	NP_659171.1	769	3	4.11
Clu	XP_006518566.1	1288	2	1.64	Axl	XP_006540056.1	1552	2	1.36
Gulo	NP_848862.1	784	1	1.34	Cyp2s1	NP_083051.1	326	9	29.10
Adam2	XP_006518502.1	458	0	0.00	Cyp2b10	NP_034129.1	736	9	12.89
Ephx2	NP_031966.2	1411	2	1.49	Cyp2b13	NP_031839.2	539	5	9.78
Chrna2	NP_659052.1	649	3	4.87	Cyp2b9	NP_034130.1	690	7	10.69
Ptk2b	NP_766086.2	1162	3	2.72	Vmn1r184	NP_001161012.1	9	5	585.50
Trim35	NP_084255.2	442	2	4.77	Cyp2a4	NP_034127.2	652	13	21.01
Stmn4	NP_001297451.1	1364	4	3.09	Nlrp9c	XP_017177801.1	203	13	67.49
Adra1a	XP_017171291.1	698	4	6.04	Nlrp4a	NP_001349085.1	131	14	112.63
Dpysl2	NP_001365696.1	883	8	9.55	Nlrp9a	NP_001289721.1	295	14	50.02
Pnma2	NP_780707.1	445	6	14.21	Vmn1r185	NP_598992.1	61	14	241.88
Bnip3l	NP_033891.1	529	1	1.99	Cyp2b23	XP_036008976.1	302	4	13.96
Ppp2r2a	NP_001192117.1	1322	7	5.58	Cyp2b19	NP_031840.1	297	6	21.29
Ebf2	NP_001347131.1	674	5	7.82	Cyp2g1	NP_038837.1	490	7	15.06
Cdca2	XP_030103464.1	506	0	0.00	Cyp2a5	NP_031838.2	785	7	9.40
Kctd9	NP_001104498.1	174	2	12.11	Cyp2a22	NP_001094937.1	515	7	14.32
Gnrh1	NP_032171.1	1017	4	4.15	Cyp2a12	NP_598418.1	731	7	10.09
Dock5	NP_808448.2	628	5	8.39	Cyp2f2	NP_031843.2	794	4	5.31
Nefl	NP_035040.1	1415	3	2.23	Cyp2t4	NP_001093654.1	76	0	0.00
Nefm	NP_032717.2	1201	3	2.63	Egln2	NP_001344696.1	565	1	1.87
Adam7	NP_031428.2	415	3	7.62	Rab4b	NP_083667.1	1717	9	5.52
Adamdec1	NP_067450.1	295	4	14.29	Mia	NP_062267.2	164	1	6.43
Adam28	NP_001041640.1	383	3	8.26	Snrpa	NP_056597.3	1149	0	0.00
Stc1	NP_033311.3	679	7	10.86	BC024978	NP_001277442.1	46	3	68.73
Nkx2-6	NP_035050.2	461	1	2.29	Itpkc	NP_853624.1	318	3	9.94
Nkx3-1	NP_035051.1	610	2	3.46	Coq8b	NP_598531.2	424	2	4.97
Slc25a37	NP_080607.2	409	3	7.73	Numbl	NP_035080.2	843	4	5.00
Synb	XP_030103659.1	195	4	21.62	Ltbp4	XP_006539521.1	443	2	4.76
Entpd4	NP_080450.1	235	5	22.42	Shkbp1	XP_036008690.1	313	1	3.37
Gm16867	XP_030103971.1	407	3	7.77	Sptbn4	XP_006540500.1	1309	5	4.03
Gm21451	XP_036014915.1	441	5	11.95	Blvrb	NP_659172.1	331	0	0.00
Entpd4b	NP_001346088.1	235	5	22.42	Sertad3	NP_573473.1	138	3	22.91
Loxl2	XP_006519807.1	441	5	11.95	Sertad1	XP_006540280.1	217	4	19.43
R3hcc1	NP_001288580.1	227	1	4.64	Prx	NP_932165.2	228	0	0.00
Chmp7	NP_598839.2	107	5	49.25	Hipk4	NP_001028487.2	768	3	4.12
Tnfrsf10b	NP_064671.2	984	1	1.07	Pld3	XP_006539705.1	372	3	8.50
Rhobtb2	XP_036014493.1	1664	11	6.97	2310022A10F	NP_001361721.1	49	8	172.07
Pebp4	NP_082836.2	572	3	5.53	Akt2	NP_001318038.1	2215	1	0.48
Egr3	NP_061251.1	785	4	5.37	Ttc9b	NP_082693.1	546	7	13.51
Bin3	NP_067303.1	832	5	6.33	Map3k10	NP_001277457.1	1275	7	5.79
Ccar2	XP_006518960.1	457	1	2.31	Zfp607b	NP_940809.2	157	0	0.00
9930012K11R	NP_001106206.1	11	0	0.00	Zfp60	NP_083807.2	179	0	0.00
Pdlim2	XP_030103571.1	735	1	1.43	Zfp626	XP_017167792.1	158	3	20.01
Sorbs3	XP_006518799.1	743	1	1.42	Zfp59	NP_035892.2	217	0	0.00
Ppp3cc	XP_036014408.1	3100	4	1.36	Zfp607a	XP_036009147.1	150	0	0.00
Slc39a14	XP_006518849.1	357	0	0.00	Zfp974	XP_030098880.1	171	3	18.49
Piwil2	NP_001351250.1	720	1	1.46	Zfp780b	NP_001074490.1	172	3	18.38
Polr3d	NP_080221.3	634	2	3.32	Zfp850	NP_001241880.1	153	0	0.00
Phyhip	NP_666093.1	925	3	3.42	Psmc4	NP_036004.2	1488	3	2.12

Bmp1	NP_001346950.1	838	2	2.52	Fcgbp	NP_001116075.1	509	0	0.00
Sftpc	NP_035489.2	388	2	5.43	9530053A07F	NP_001158127.1	489	0	0.00
Lgi3	XP_030103578.1	562	3	5.63	Fbl	XP_030097991.1	1924	3	1.64
Reep4	XP_006519661.1	1038	0	0.00	Dyrk1b	NP_001258299.1	1014	0	0.00
Hr	NP_001366408.1	248	0	0.00	Eid2	NP_940817.1	96	0	0.00
Nudt18	NP_694776.2	129	0	0.00	Eid2b	NP_001170898.1	61	0	0.00
Fam160b2	XP_036014483.1	21	1	50.19	Selenov	NP_778198.2	100	0	0.00
Dmtn	NP_001346958.1	408	6	15.50	Dll3	NP_031892.2	467	0	0.00
Fgf17	NP_001347037.1	671	2	3.14	Timm50	NP_079892.1	831	4	5.07
Npm2	NP_851990.2	461	4	9.14	Supt5	XP_006539765.1	1424	4	2.96
Xpo7	NP_075532.1	586	2	3.60	Rps16	NP_038675.2	1592	2	1.32
Dok2	NP_034201.1	615	3	5.14	Plekhg2	NP_001077381.1	229	3	13.81
Gfra2	NP_032141.2	439	1	2.40	Zfp36	NP_035886.1	821	0	0.00
Fndc3a	XP_030103717.1	373	7	19.78	Med29	NP_080318.2	260	3	12.16
Cysltr2	XP_030103861.1	360	8	23.42	Paf1	XP_036009151.1	602	3	5.25
Rcbtb2	XP_036014266.1	592	7	12.46	Samd4b	NP_778186.2	441	1	2.39
Rb1	NP_033055.2	1322	3	2.39	Gmfg	NP_071307.1	523	1	2.02
Lpar6	NP_780325.1	429	9	22.11	Lrfn1	XP_030098980.1	587	0	0.00
Itm2b	NP_032436.1	455	5	11.58	Ifnl2	XP_006540184.1	244	0	0.00
Med4	NP_080395.1	569	6	11.11	Ifnl3	XP_006540193.1	290	0	0.00
Nudt15	NP_001347413.1	143	7	51.59	Sycn	NP_080992.3	176	1	5.99
Sucla2	NP_035636.1	1130	0	0.00	Nccrp1	NP_001074584.2	396	2	5.32
Htr2a	NP_766400.1	764	3	4.14	Pak4	XP_017167785.1	441	1	2.39
Esd	NP_001272352.1	644	11	18.00	Acp7	XP_006539510.1	327	0	0.00
Lrch1	XP_017171556.2	741	2	2.84	Fbxo27	XP_006539925.1	396	2	5.32
Rubcnl	NP_941044.3	261	1	4.04	Fbxo17	XP_006540232.1	461	5	11.43
Lrrc63	NP_081857.1	411	0	0.00	Mrps12	NP_001347181.1	1215	1	0.87
Lcp1	XP_036014400.1	1634	1	0.64	Sars2	NP_076126.2	1011	4	4.17
Cpb2	NP_062749.2	986	2	2.14	Ccer2	NP_001182020.1	45	1	23.42
Zc3h13	XP_036014719.1	793	3	3.99	Nfkbib	NP_035038.2	1527	1	0.69
Siah3	NP_001121565.1	492	1	2.14	Sirt2	NP_071877.3	1166	2	1.81
Cby2	NP_001157612.1	393	0	0.00	Rinl	XP_006540161.3	261	2	8.08
Erich6b	NP_001355951.1	0	0	#DIV/0!	Hnrnpl	XP_006539620.1	970	0	0.00
Cog3	NP_796355.2	528	0	0.00	Ech1	NP_058052.1	659	3	4.80
Slc25a30	XP_006519519.1	270	3	11.71	Lgals4	NP_034836.1	1939	2	1.09
Tpt1	NP_033455.1	1030	5	5.12	Lgals7	NP_032522.2	353	0	0.00
Gtf2f2	NP_081092.1	814	3	3.88	Capn12	NP_001104277.1	271	3	11.67
Kctd4	NP_080490.3	227	0	0.00	Actn4	NP_001347478.1	1458	4	2.89
Gpalpp1	NP_080453.2	95	0	0.00	Eif3k	NP_001272871.1	898	2	2.35
Nufip1	NP_038773.1	299	0	0.00	Map4k1	NP_032305.2	436	3	7.25
Tsc22d1	XP_017171431.1	447	3	7.07	Ryr1	XP_036008712.1	964	4	4.37
Serp2	XP_030103906.1	350	2	6.02	Rasgrp4	XP_011248827.1	556	2	3.79
Lacc1	NP_766076.1	191	1	5.52	Fam98c	XP_006540456.1	110	2	19.16
Ccdc122	NP_780578.1	39	2	54.05	Spred3	XP_030097822.1	247	0	0.00
Enox1	XP_030103660.1	176	1	5.99	Ggn	XP_006540007.1	235	1	4.48
Dnajc15	NP_079660.1	605	0	0.00	Psmd8	NP_080821.3	1099	4	3.84
Epsti1	NP_083771.1	458	0	0.00	Catsperg1	XP_006540153.1	101	4	41.74
Fam216b	NP_808297.1	29	0	0.00	Kcnk6	NP_001028697.2	253	3	12.50
Tnfsf11	NP_035743.2	1236	4	3.41	Yif1b	NP_084163.2	402	1	2.62
Akap11	XP_030103631.1	351	2	6.01	2200002D01F	NP_082455.1	42	2	50.19
Dgkh	XP_011243402.1	342	5	15.41	Spint2	NP_001076017.1	604	6	10.47
Vwa8	NP_082182.1	671	6	9.42	Ppp1r14a	NP_081007.2	344	4	12.25
Zfp957	NP_001028387.1	837	7	8.81	Dpf1	XP_006540095.1	336	0	0.00
Rgcc	NP_079703.2	423	4	9.97	Sipa1l3	XP_006540468.1	369	1	2.86
Naa16	NP_080108.1	346	8	24.37	Catsperg2	XP_036009443.1	178	3	17.76
Mtrf1	XP_036014423.1	530	6	11.93	Zfp84	NP_076239.1	276	0	0.00
Kbtbd7	NP_001019306.2	505	1	2.09	Zfp30	NP_001347136.1	279	0	0.00
Kbtbd6	NP_001030054.3	400	1	2.63	Zfp790	XP_036008844.1	202	0	0.00
Wbp4	NP_061235.2	618	1	1.71	Zfp940	XP_030098313.1	158	0	0.00
Elf1	NP_001273340.1	265	0	0.00	Zfp420	XP_036008846.1	267	0	0.00
Sugt1	NP_080750.1	1832	2	1.15	Zfp27	XP_017177621.1	225	0	0.00
Cnmd	XP_036014395.1	341	1	3.09	Zfp383	NP_001230837.1	218	0	0.00
Pcdh8	NP_001036191.1	802	8	10.51	Zfp74	NP_848471.2	316	0	0.00
Olfm4	NP_001338876.1	601	2	3.51	Zfp568	NP_001028527.2	251	0	0.00
Pcdh17	XP_006518968.1	775	8	10.88	Zfp14	NP_001345789.1	249	1	4.23
Diaph3	XP_011243425.1	771	5	6.83	Zfp82	NP_001239448.1	188	0	0.00

Tdrd3	NP_766193.3	577	7	12.79	Zfp566	NP_690027.1	191	0	0.00
Pcdh20	XP_036014467.1	518	7	14.24	Zfp260	NP_036111.2	335	0	0.00
Pcdh9	NP_001258728.1	1068	6	5.92	Zfp382	NP_001074476.1	237	0	0.00
Spertl	XP_017171619.1	420	6	15.06	Zfp146	NP_001344486.1	293	0	0.00
Klhl1	NP_444335.2	587	6	10.77	Cox7a1	NP_034074.1	463	7	15.93
Dach1	XP_036014326.1	632	2	3.34	Capns1	NP_033925.2	589	7	12.53
Mzt1	NP_780454.1	274	1	3.85	Tbcb	NP_079824.2	604	2	3.49
Bora	NP_780474.1	599	2	3.52	Polr2i	NP_081535.1	1190	8	7.09
Dis3	NP_082591.2	1361	2	1.55	Ovol3	XP_036009090.1	122	8	69.11
Pibf1	NP_083596.2	153	2	13.78	Wdr62	XP_006539944.1	446	0	0.00
Klf5	NP_033899.2	864	2	2.44	Clip3	NP_001074583.1	1000	9	9.49
Klf12	XP_011243272.1	473	1	2.23	Alkbh6	NP_932144.2	88	3	35.93
Prr30	NP_083956.1	189	1	5.58	Syne4	NP_705805.1	112	0	0.00
Tbc1d4	NP_001074747.2	491	3	6.44	Sdhaf1	NP_001028312.2	261	10	40.38
Commd6	NP_001162064.1	295	5	17.86	Lrfn3	NP_780687.1	629	12	20.11
Uchl3	XP_006519309.1	725	5	7.27	Tyrobp	NP_035792.1	989	4	4.26
Lmo7	XP_036014597.1	1261	8	6.69	Hcst	NP_035957.2	451	3	7.01
Kctd12	NP_808383.3	316	5	16.68	Nfkbid	NP_001347835.1	1208	12	10.47
Acod1	NP_032418.1	570	1	1.85	Aplp1	NP_031493.2	1514	11	7.66
Cln5	NP_001028414.1	281	5	18.75	Kirrel2	XP_036008988.1	299	10	35.25
Fbxl3	NP_001334529.1	1047	5	5.03	Nphs1	XP_036009153.1	860	3	3.68
Mycbp2	XP_017171274.1	918	6	6.89	Prodh2	XP_006540285.1	728	6	8.69
Scel	XP_006519418.1	745	1	1.41	Arhgap33	NP_001276599.1	259	3	12.21
Slain1	NP_001348568.1	118	2	17.86	Proser3	NP_001361572.1	97	3	32.59
Ednrb	NP_001129533.1	904	7	8.16	Hspb6	XP_006540020.1	472	4	8.93
Pou4f1	NP_035273.3	676	3	4.68	Lin37	NP_083653.1	209	8	40.34
Obi1	NP_080323.3	55	1	19.16	Psenen	NP_079774.1	424	7	17.40
Rbm26	XP_030103935.1	420	0	0.00	U2af1l4	NP_739566.1	568	1	1.86
Ndfip2	NP_083837.2	469	3	6.74	Igflr1	NP_663555.2	92	8	91.64
Spry2	XP_006519079.1	762	1	1.38	Kmt2b	NP_083550.2	1457	1	0.72
Trim52	NP_941003.1	330	1	3.19	Zbtb32	XP_011248965.1	426	3	7.42
Slitrk1	NP_951020.1	1108	1	0.95	Upk1a	NP_081091.1	200	4	21.08
Slitrk6	NP_780708.1	417	1	2.53	Cox6b1	NP_079904.1	754	4	5.59
Slitrk5	NP_001365697.1	720	2	2.93	Etv2	XP_006539605.1	393	0	0.00
Gpc5	XP_017171234.1	548	3	5.77	Rbm42	NP_598454.2	336	0	0.00
Gpc6	NP_001073313.1	537	3	5.89	Haus5	NP_082275.1	373	1	2.83
Dct	NP_034154.2	615	0	0.00	2200002J24R	XP_017167770.1	0	0	#DIV/0!
Tgds	NP_083854.3	362	8	23.29	Atp4a	NP_001277556.1	1155	1	0.91
Gpr180	NP_067409.2	303	6	20.87	Tmem147	NP_081491.2	427	3	7.40
Sox21	NP_808421.1	555	3	5.70	Gapdhs	NP_001277560.1	1771	1	0.60
Gm9376	NP_001095079.1	9	0	0.00	Sbsn	XP_006540092.1	240	3	13.17
Abcc4	NP_001157148.1	803	3	3.94	Dmkn	NP_001159645.1	140	2	15.06
Cldn10	NP_076367.2	365	7	20.21	Krtdap	NP_001350939.1	288	2	7.32
Dzip1	XP_030103796.1	252	10	41.82	Ffar2	NP_666299.1	436	4	9.67
Dnajc3	NP_032955.2	955	6	6.62	Ffar3	NP_001028488.1	376	4	11.21
Uggt2	NP_001074721.2	438	6	14.44	Ffar1	NP_918946.2	410	3	7.71
Hs6st3	XP_036014643.1	413	8	20.41	Cd22	XP_006539552.1	1010	4	4.17
Oxgr1	NP_001001490.1	452	5	11.66	Mag	NP_001333018.1	1046	2	2.02
Mbnl2	NP_001347309.1	715	7	10.32	Hamp2	NP_899080.2	530	3	5.97
Rap2a	NP_083795.2	1643	4	2.57	Hamp	NP_115930.1	845	3	3.74
Ipo5	XP_030103878.1	1386	3	2.28	Usf2	XP_006539796.1	614	3	5.15
Farp1	NP_598843.3	360	4	11.71	Lsr	NP_001157657.1	347	1	3.04
Stk24	NP_663440.1	844	4	4.99	Fam187b	NP_780449.2	53	0	0.00
Slc15a1	NP_444309.2	576	2	3.66	Fxyd5	NP_001104543.1	235	2	8.97
Dock9	XP_036014248.1	471	9	20.14	Fxyd7	NP_071290.1	351	5	15.01
Ubac2	NP_081137.2	327	3	9.67	Fxyd1	XP_011248951.1	402	5	13.11
Gpr18	NP_877958.1	561	4	7.51	Lgi4	XP_036008989.1	205	5	25.70
Gpr183	XP_036014569.1	661	4	6.38	Fxyd3	NP_032583.1	257	4	16.40
Timm8a2	XP_006518982.1	337	1	3.13	Hpn	NP_032307.2	859	5	6.13
Tm9sf2	NP_542123.3	457	4	9.71	Scn1b	NP_035452.1	473	6	13.37
Clybl	XP_036014733.1	327	4	14.32	Gramd1a	XP_006540248.1	111	2	18.99
Zic5	NP_075363.1	521	4	9.52	Scgb2b1	NP_001297589.1	150	0	0.00
Zic2	NP_033600.3	920	3	4.30	Scgb1b2	NP_065588.1	136	1	7.75
Pcca	NP_659093.2	546	1	2.57	Scgb2b2	NP_997145.2	429	6	14.74
Ggact	XP_036014472.1	138	0	0.00	Scgb2b3	NP_001257470.1	71	0	0.00
Tmtc4	XP_017171671.1	861	0	0.00	Scgb1b3	NP_001243002.1	90	1	11.71

Nalcn	XP_036014577.1	1262	1	1.39	Scgb2b7	NP_001185800.1	67	0	0.00
Itgbl1	NP_663442.2	638	1	3.00	Scgb1b7	NP_001257471.1	101	1	10.43
Fgf14	NP_997550.1	1101	1	1.91	Scgb2b10	NP_001365615.1	33	0	0.00
Selenop	NP_001036078.1	422	1	4.99	Scgb1b10	XP_006540554.1	63	1	16.73
Ccdc152	XP_036014939.1	67	2	57.20	Scgb2b11	XP_006540556.1	261	0	0.00
Ghr	NP_034414.2	475	1	3.70	Scgb2b12	XP_036009257.1	35	0	0.00
Fbxo4	NP_598860.2	452	0	0.00	Scgb1b12	XP_030098761.1	396	1	2.66
AW549877	NP_666042.2	102	0	0.00	Scgb1b29	NP_001242995.1	98	1	10.75
Oxct1	NP_077150.1	518	1	2.71	Scgb2b15	NP_001268452.1	31	0	0.00
Plcxd3	NP_796329.2	267	2	9.87	Scgb1b15	NP_001357808.1	278	0	0.00
C6	NP_057913.2	550	2	4.51	Scgb2b17	NP_001268453.1	0	0	#DIV/0!
Mroh2b	XP_030104374.1	138	0	0.00	Scgb1b17	NP_001357807.1	279	0	0.00
C7	NP_001230766.1	218	4	20.36	Scgb2b18	XP_006540561.1	427	0	0.00
Card6	XP_006520050.2	227	9	41.78	Scgb2b19	XP_036009149.1	76	0	0.00
Rpl37	NP_080345.1	930	2	2.27	Scgb1b19	XP_030098618.1	626	0	0.00
Prkaa1	XP_036014946.1	1810	7	4.08	Scgb2b20	XP_036009124.1	136	0	0.00
Ttc33	NP_001342549.1	868	9	10.93	Scgb1b20	NP_001257472.1	329	0	0.00
Ptger4	NP_032991.1	852	6	7.42	Scgb2b21	XP_036009211.1	179	0	0.00
Dab2	XP_017171913.1	938	5	5.62	Scgb2b24	NP_803229.1	283	0	0.00
C9	NP_001355349.1	687	3	4.60	Scgb1b24	NP_001092799.1	499	0	0.00
Fyb	XP_011243630.1	652	5	8.08	Scgb2b26	NP_840093.1	94	0	0.00
Rictor	XP_006520066.1	1050	2	2.01	Scgb2b27	NP_001093934.1	227	0	0.00
Osmr	NP_001297398.1	376	8	22.42	Scgb1b27	NP_033726.1	148	0	0.00
Lifr	NP_001345522.1	470	5	11.21	Scgb1b30	NP_001092800.1	19	0	0.00
Egflam	XP_017172117.1	1244	5	4.24	Wtip	NP_997095.1	825	5	6.39
Gdnf	NP_034405.1	971	3	3.26	Uba2	NP_057891.1	1868	5	2.82
Wdr70	NP_001074871.1	178	6	35.52	Pdcd2l	NP_080825.1	418	3	7.56
Nup155	NP_573490.3	960	6	6.59	Gpi1	NP_032181.2	1252	5	4.21
Cplane1	XP_036015509.1	95	4	44.37	Garre1	XP_030098318.1	67	5	78.65
Nipbl	NP_081983.2	1248	6	5.07	Lsm14a	XP_017167743.1	609	5	8.65
Slc1a3	NP_683740.1	1088	6	5.81	Kctd15	NP_001347749.1	381	3	8.30
Ranbp3l	XP_036015211.1	218	4	19.34	Chst8	NP_780349.3	282	1	3.74
Nadk2	NP_001078879.1	146	2	14.44	Pepd	NP_032846.2	739	4	5.70
Skp2	XP_011243661.1	1498	3	2.11	Cebpg	NP_034014.1	488	3	6.48
Lmbrd2	NP_796152.2	46	1	22.91	Cebpa	NP_001274443.1	1453	2	1.45
Ugt3a1	NP_997099.2	602	3	5.25	Slc7a10	XP_006540260.1	395	3	8.00
Ugt3a2	NP_659094.1	741	3	4.27	Lrp3	XP_006540228.1	238	1	4.43
Capsl	NP_083617.2	558	5	9.44	Gpatch1	NP_080457.1	314	0	0.00
Il7r	NP_032398.3	822	0	0.00	Rhpn2	NP_082173.3	633	4	6.66
Spef2	NP_001291971.1	307	6	20.60	Faap24	NP_848758.1	382	0	0.00
Prlr	XP_030104285.1	560	3	5.65	Cep89	NP_082396.1	280	4	15.06
Agxt2	NP_001027021.1	1075	4	3.92	Slc7a9	NP_067266.1	430	1	2.45
Dnajc21	NP_084322.2	895	4	4.71	Tdrd12	XP_030098861.1	1580	1	0.67
Brix1	NP_080672.3	1325	3	2.39	Nudt19	NP_149071.2	638	3	4.96
Rad1	NP_001276377.1	578	2	3.65	Rgs9bp	NP_665839.1	193	2	10.92
Ttc23l	NP_001361701.1	19	0	0.00	Ankrd27	NP_663608.3	1238	2	1.70
Rai14	XP_006520255.1	1009	3	3.13	Pdcd5	NP_062720.1	700	3	4.52
C1qtnf3	XP_036015535.1	192	1	5.49	Dpy19l3	NP_848819.2	228	0	0.00
Amacr	NP_032563.2	544	1	1.94	Zfp507	XP_006540361.1	494	1	2.13
Slc45a2	NP_444307.1	468	0	0.00	Tshz3	NP_758502.1	493	2	4.28
Rxfp3	NP_848832.1	362	0	0.00	Zfp536	XP_036008998.1	405	5	13.01
Adamts12	NP_780710.2	388	1	2.72	Uri1	XP_006539739.2	296	3	10.68
Tars	NP_149065.2	1284	4	3.28	Ccne1	NP_031659.2	2542	2	0.83
Npr3	NP_001034270.1	718	2	2.94	1600014C10R	NP_001078854.1	162	4	26.02
Sub1	XP_006520105.1	850	4	4.96	Plekhf1	NP_077724.2	328	4	12.85
Zfr	XP_006520120.1	775	6	8.16	Pop4	NP_079666.1	451	4	9.35
Mtmr12	NP_766546.1	199	5	26.48	Gm6818	XP_006540568.1	28	0	0.00
Golph3	NP_079949.1	767	7	9.62	Gm6605	XP_006540569.1	20	0	0.00
Pdzd2	XP_006520230.1	1413	10	7.46	Gm5591	NP_001013832.1	28	0	0.00
6030458C11R	NP_084274.1	108	1	9.76	Gm4454	XP_001480278.2	20	0	0.00
Drosha	NP_081075.3	3039	3	1.04	Gm21115	XP_003688880.1	20	0	0.00
Cdh6	NP_031692.2	537	10	19.63	Gm21129	XP_006540570.1	28	0	0.00
Cdh9	XP_011243639.1	661	10	15.94	Gm21136	XP_006540571.1	20	1	52.70
Cdh10	NP_033995.1	601	13	22.80	Gm6833	XP_003688883.1	28	0	0.00
Acot10	NP_073727.2	211	11	54.94	Gm1988	XP_001472012.1	28	0	0.00
Cdh12	XP_017172034.1	512	15	30.88	Gm6124	XP_001472050.1	20	1	52.70

Hnrnpa1l2-ps2	NP_001159443.1	2288	4	1.84	Gm29258	XP_006541389.1	28	0	0.00
Cdh18	NP_001074768.1	510	14	28.93	Gm9271	XP_001004349.3	20	1	52.70
Basp1	NP_081671.1	467	8	18.05	Gm5114	NP_808558.2	20	1	52.70
Myo10	NP_062345.2	1300	16	12.97	Zfp619	XP_036009324.1	134	0	0.00
Retreg1	NP_001030023.1	344	14	42.89	Vstm2b	XP_006541094.1	562	0	0.00
Zfp622	NP_653106.1	871	15	18.15	4930433I11Ri	NP_997131.2	110	0	0.00
Marchf11	NP_808265.2	371	14	39.77	Gm4884	XP_006540878.1	25	6	252.94
Fbxl7	NP_795933.2	966	15	16.36	Gm2128	XP_001472451.1	20	1	52.70
Ank	NP_065065.3	272	0	0.00	Gm6866	XP_001002281.1	28	0	0.00
Otulin	NP_001013814.2	236	6	26.79	Gm5592	NP_001028954.1	28	1	37.64
Otulinl	NP_938043.1	242	8	34.84	AI987944	NP_898990.1	132	0	0.00
Trio	XP_006520114.1	1250	1	0.84	Vmn2r57	NP_808432.2	38	0	0.00
Dnah5	XP_006520077.1	890	6	7.10	AW146154	NP_001028702.1	127	0	0.00
Ctnnd2	NP_032755.2	1361	5	3.87	Gm6871	NP_001365582.1	176	0	0.00
Dap	NP_666169.1	335	0	0.00	Zfp788	NP_001347607.1	202	0	0.00
Ankrd33b	XP_036015442.1	19	0	0.00	Vmn2r58	NP_001098525.1	25	0	0.00
Ropn1l	NP_665851.2	341	1	3.09	Vmn2r59	NP_001098526.1	32	0	0.00
Marchf6	NP_766194.2	540	1	1.95	Vmn2r60	NP_001098527.1	33	0	0.00
Cmbl	NP_853619.1	727	2	2.90	Vmn2r61	NP_001098528.1	33	0	0.00
Cct5	NP_031663.1	2114	4	1.99	4933421I07Ri	NP_001357701.1	110	0	0.00
Atpsckmt	NP_080822.1	444	2	4.75	Zfp141	XP_030098597.1	233	0	0.00
Tas2r119	NP_065249.3	380	1	2.77	Zfp977	NP_001349041.1	133	0	0.00
Sema5a	NP_033180.2	719	3	4.40	Zfp976	XP_017177564.1	149	0	0.00
Sdc2	NP_032330.1	646	2	3.26	Zfp975	XP_030098598.1	152	0	0.00
Cpq	NP_061225.2	342	4	12.33	Vmn2r62	NP_001098529.1	12	0	0.00
Tspyl5	NP_001078890.1	883	6	7.16	Vmn2r63	NP_001098530.1	28	0	0.00
Mtdh	NP_001344855.1	441	3	7.17	Zfp936	XP_030098763.1	200	0	0.00
Laptm4b	NP_277056.1	380	5	13.87	EU599041	NP_001170996.1	177	0	0.00
Matn2	NP_001345709.1	808	1	1.30	Zfp715	XP_030098837.1	186	0	0.00
9430069I07Ril	NP_001243090.1	0	0	#DIV/0!	Siglecf	XP_036008858.1	467	2	4.51
Rpl30	NP_001156957.1	1165	3	2.71	Siglecg	XP_006540923.1	311	4	13.55
Erich5	NP_775597.1	54	0	0.00	Gm38999	NP_001357781.1	0	0	#DIV/0!
Rida	NP_032313.2	524	0	0.00	Lim2	NP_808361.1	253	2	8.33
Pop1	NP_690854.1	259	0	0.00	Nkg7	NP_077215.2	376	2	5.61
Nipal2	XP_006520118.1	262	0	0.00	Cldnd2	XP_030098889.1	64	1	16.47
Kcns2	NP_851834.1	994	3	3.18	Etfb	NP_080971.2	731	2	2.88
Stk3	NP_062609.2	701	0	0.00	Vsig10l	NP_001355810.1	45	0	0.00
Osr2	NP_001355594.1	658	1	1.60	Iglon5	NP_001157990.1	265	0	0.00
Vps13b	NP_796125.2	585	2	3.60	4931406B18R	XP_017167829.1	0	0	#DIV/0!
Cox6c	NP_444301.1	573	2	3.68	Siglecl1	NP_001365379.1	448	7	16.47
Rgs22	XP_036015426.1	254	1	4.15	Cd33	NP_001104528.1	637	5	8.27
Fbxo43	NP_001355587.1	182	0	0.00	Zfp658	NP_001008549.1	192	0	0.00
Polr2k	NP_001034457.1	1007	6	6.28	Zfp719	NP_001344698.1	181	0	0.00
Spag1	XP_017172123.1	1089	1	0.97	Zfp819	XP_017167842.1	266	1	3.96
Rnf19a	NP_038951.1	736	3	4.30	Ceacam18	XP_017167815.1	81	4	52.04
Ankrd46	NP_001344935.1	878	5	6.00	Siglece	XP_006541383.1	448	5	11.76
Snx31	XP_006520210.1	140	0	0.00	Ctu1	NP_663557.1	682	7	10.82
Pabpc1	NP_032800.2	1574	7	4.69	Klk14	XP_006541019.1	217	6	29.14
Ywhaz	NP_001343498.1	3447	10	3.06	Klk13	NP_001034131.2	281	7	26.25
Zfp706	NP_080797.1	219	6	28.87	Klk12	XP_017167777.1	208	5	25.33
Grhl2	XP_017172111.1	439	8	19.21	Klk11	NP_001170844.1	325	8	25.94
Ncald	XP_017172182.1	1345	11	8.62	Klk10	NP_598473.1	404	16	41.74
4930447A16R	XP_017172263.1	0	0	#DIV/0!	Klk9	NP_082936.2	252	7	29.28
Rrm2b	NP_955770.1	1101	2	1.91	Klk8	NP_001311327.1	388	7	19.01
Ubr5	NP_001106192.1	1330	8	6.34	Klk7	NP_036002.1	469	10	22.47
Odf1	NP_032783.2	500	0	0.00	Klk6	XP_030098097.1	488	8	17.28
Klf10	NP_001276400.1	634	3	4.99	Klk5	NP_081082.1	430	8	19.61
Azin1	XP_030104521.1	731	7	10.09	Klk4	NP_064312.1	508	8	16.60
Atp6v1c1	NP_079770.2	657	6	9.62	Klk15	NP_777354.1	210	6	30.11
Baalc	NP_542371.1	591	5	8.92	Klk1b8	NP_032483.1	404	5	13.04
Fzd6	XP_017171919.1	583	3	5.42	Klk1b1	NP_034775.1	317	2	6.65
Cthrc1	NP_081054.1	348	3	9.09	Klk1b9	NP_034246.1	450	3	7.03
Slc25a32	XP_036015474.1	367	3	8.61	Klk1b11	NP_034770.1	163	2	12.93
Dcaf13	NP_941008.2	1081	4	3.90	Klk1b21	XP_017177490.1	158	0	0.00
Rims2	NP_444501.1	979	7	7.54	Klk1b26	NP_034774.1	177	1	5.95
Dcstamp	NP_083698.1	253	3	12.50	Klk1b27	NP_064664.1	157	1	6.71

Dpys	NP_073559.3	907	3	3.49	Klk1b22	NP_034244.1	230	0	0.00
Lrp12	NP_001333965.1	164	6	38.56	Klk1b16	XP_017177488.1	143	0	0.00
9330182O14R	NP_001242985.1	0	0	#DIV/0!	Klk1b24	NP_034773.1	129	1	8.17
Zfpm2	NP_035896.1	668	4	6.31	Klk1b3	NP_032719.1	294	2	7.17
Oxr1	NP_001345906.1	744	7	9.92	Klk1b4	NP_035045.2	600	4	7.03
Abra	NP_780665.1	524	5	10.06	Klk1b5	NP_032482.1	497	2	4.24
Angpt1	NP_001272991.1	964	4	4.37	Klk1	NP_034769.4	418	7	17.65
Rspo2	NP_001344885.1	880	5	5.99	2410002F23R	XP_017167726.1	103	1	10.23
Eif3e	NP_032414.1	1367	6	4.63	Acp4	XP_006540593.1	311	5	16.94
Emc2	NP_080012.1	501	8	16.83	Clec11a	NP_033157.1	311	0	0.00
Tmem74	XP_006520971.2	152	8	55.47	Shank1	XP_030098435.1	2140	4	1.97
Trhr	NP_038724.1	521	8	16.18	1700008O03F	XP_017167775.1	12	0	0.00
Nudcd1	NP_001107026.1	246	6	25.70	Syt3	XP_017177576.1	600	8	14.05
Eny2	NP_778174.1	563	4	7.49	Lrrc4b	NP_937893.1	1247	2	1.69
Pkhd1l1	NP_619615.2	350	6	18.07	Aspdh	NP_080966.1	538	1	1.96
Ebag9	NP_001344620.1	294	7	25.09	Josd2	XP_006541134.1	198	0	0.00
Sybu	XP_006521131.3	491	8	17.17	Emc10	NP_932108.3	164	5	32.13
Gm5471	XP_036015556.1	875	4	4.82	Fam71e1	XP_036009410.1	26	1	40.53
Kcnv1	NP_080476.2	971	5	5.43	Mybpc2	NP_666301.2	373	5	14.13
Csmd3	XP_030104400.1	586	3	5.40	Spib	XP_030098488.1	540	3	5.86
Trps1	XP_006521626.1	974	8	8.66	Pold1	XP_030098080.1	1548	4	2.72
Eif3h	XP_036015452.1	1014	9	9.35	Nr1h2	NP_001272447.1	926	2	2.28
Utp23	NP_084408.1	641	7	11.51	Napsa	NP_032463.1	767	0	0.00
Rad21	NP_033035.3	1549	3	2.04	Kcnc3	XP_006540717.1	1046	3	3.02
Aard	NP_780712.2	106	3	29.83	Myh14	XP_030098856.1	1369	4	3.08
Slc30a8	NP_766404.1	546	1	1.93	Izumo2	XP_017167850.1	120	2	17.57
Med30	NP_081488.1	404	9	23.48	Zfp473	XP_036009007.1	261	3	12.11
Ext1	NP_034292.2	516	7	14.30	Vrk3	NP_598706.1	748	1	1.41
Samd12	XP_011243962.1	146	7	50.53	Atf5	NP_109618.1	479	1	2.20
Tnfrsf11b	NP_032790.3	717	3	4.41	Nup62	NP_444304.1	670	5	7.86
Colec10	NP_775598.2	677	4	6.23	Il4i1b	NP_001164495.1	870	1	1.21
Mal2	NP_849251.1	497	4	8.48	Il4i1	NP_034345.2	870	1	1.21
Ccn3	NP_035060.1	239	0	0.00	Tbc1d17	NP_001036120.1	669	8	12.60
Enpp2	XP_006520659.1	852	5	6.18	Akt1s1	NP_001277624.1	433	4	9.74
Taf2	XP_006521139.1	986	5	5.34	Pnkp	XP_006541101.2	566	2	3.72
Dscc1	NP_898912.2	701	8	12.03	Ptov1	XP_036009497.1	188	3	16.82
Deptor	NP_663445.2	333	4	12.66	Med25	XP_030098926.1	188	7	39.24
Col14a1	NP_001355351.1	665	8	12.68	Fuz	XP_006541255.1	302	10	34.90
Mrpl13	NP_081035.1	1475	8	5.72	Ap2a1	NP_001070732.1	1125	4	3.75
Mtbp	NP_598853.3	415	7	17.78	Tsks	XP_006540856.1	256	13	53.52
Sntb1	XP_006520734.1	474	10	22.23	Cpt1c	NP_710146.1	536	8	15.73
Has2	NP_032242.3	947	1	1.11	Prmt1	NP_001239406.1	1744	10	6.04
Slc22a22	NP_001344859.1	136	1	7.75	Bcl2l12	NP_083686.1	331	12	38.21
Zhx2	NP_955520.1	541	1	1.95	Irf3	XP_006541058.1	1012	12	12.50
Derl1	NP_077169.1	897	1	1.17	Scaf1	XP_030098326.1	364	12	34.74
Tbc1d31	XP_006520761.1	750	3	4.22	Rras	XP_036008706.1	1790	12	7.07
Fam83a	NP_776287.2	71	0	0.00	Prr12	NP_778187.2	230	12	54.99
9130401M01F	NP_083694.1	47	1	22.42	Prrg2	NP_075375.1	130	13	105.39
Zhx1	NP_001035903.1	302	1	3.49	Nosip	XP_017167721.1	359	13	38.16
Atad2	NP_081711.2	1523	6	4.15	Rcn3	NP_001341974.1	437	8	19.29
Wdyhv1	NP_084010.1	72	0	0.00	Fcgrt	NP_034319.2	471	6	13.43
Fbxo32	NP_080622.1	976	1	1.08	Rps11	NP_038753.1	1478	6	4.28
Klhl38	NP_808423.1	300	1	3.51	Rpl13a	NP_033464.2	1643	5	3.21
Anxa13	NP_081487.1	297	1	3.55	Flt3l	XP_036008569.1	488	1	2.16
Fam91a1	NP_666071.2	434	3	7.29	Aldh16a1	NP_666066.1	579	3	5.46
Fer1l6	NP_001357841.1	279	1	3.78	Pih1d1	XP_036009299.1	475	2	4.44
Tmem65	NP_780421.2	243	0	0.00	Slc17a7	NP_892038.2	1442	1	0.73
Trmt12	NP_080918.2	400	3	7.90	Gfy	NP_001182184.1	64	0	0.00
Rnf139	NP_780435.1	604	5	8.72	Pth2	XP_017177419.1	207	0	0.00
Tatdn1	NP_780360.1	359	4	11.74	Kash5	NP_958762.2	149	3	21.22
Ndufb9	NP_075661.1	702	7	10.51	Dkkl1	XP_006541048.1	237	3	13.34
Mtss1	XP_030104311.1	438	9	21.66	Tead2	NP_001366201.1	638	7	11.56
Sqle	NP_033296.1	1012	8	8.33	Cd37	NP_001277733.1	403	3	7.85
Washc5	NP_705776.2	319	7	23.13	Slc6a21	XP_017167865.1	51	0	0.00
Nsmce2	NP_001361697.1	785	7	9.40	Trpm4	XP_036009293.1	338	1	3.12
Trib1	NP_653132.1	1489	6	4.25	Hrc	NP_034603.2	583	0	0.00

4933412E24Ri	NP_081944.1	89	0	0.00	Ppfia3	XP_036009445.1	580	5	9.09
Lratd2	NP_001156398.1	253	8	33.32	Lin7b	NP_035828.1	833	3	3.80
A1bg	NP_001074536.1	402	1	2.62	Snrnp70	NP_033250.3	1179	1	0.89
Myc	NP_034979.3	3656	3	0.86	Kcna7	NP_034726.2	676	5	7.80
Gsdmc	NP_113555.1	229	7	32.22	Ntf5	NP_937833.1	606	5	8.70
Gsdmc2	XP_030104472.1	85	1	12.40	Lhb	XP_030098037.1	456	4	9.24
Gsdmc3	XP_017172127.1	64	1	16.47	Ruvbl2	XP_006540785.1	2033	3	1.56
Gsdmc4	XP_011244059.1	57	0	0.00	Gys1	NP_109603.2	1189	4	3.55
Cyrib	XP_030104330.1	292	4	14.44	Ftl1	NP_034370.2	673	3	4.70
Asap1	XP_036015014.1	809	4	5.21	Bax	XP_011249082.1	600	2	3.51
Adcy8	NP_001318004.1	1548	5	3.40	Dhdh	NP_082179.1	449	4	9.39
Efr3a	NP_598527.2	344	3	9.19	Nucb1	NP_032775.1	543	0	0.00
Oc90	XP_036015122.1	173	1	6.09	Tulp2	XP_006541080.2	213	2	9.90
Hhla1	NP_001138568.1	67	1	15.73	Ppp1r15a	NP_032680.1	541	3	5.84
Kcnq3	NP_690887.2	1096	3	2.88	Plekha4	XP_006541221.1	163	0	0.00
Lrrc6	NP_062330.1	901	3	3.51	Hsd17b14	NP_079606.3	418	9	22.69
Tmem71	NP_766102.1	109	3	29.01	Bcat2	XP_017177442.1	792	4	5.32
Phf20l1	XP_006520979.1	844	3	3.75	Fgf21	NP_064397.1	1064	4	3.96
Tg	XP_006520808.2	656	3	4.82	Fut1	XP_036008571.1	427	5	12.34
Sla	NP_001025012.1	352	3	8.98	Izumo1	XP_036009362.1	225	4	18.74
Ccn4	NP_061353.1	554	5	9.51	Rasip1	NP_082820.1	1108	4	3.80
Ndrg1	NP_032707.2	744	5	7.08	Mamstr	XP_006541295.1	63	0	0.00
St3gal1	XP_036015179.1	364	2	5.79	Fut2	NP_061364.2	446	6	14.18
Zfat	XP_006521187.2	360	2	5.86	Sec1	XP_030098674.1	208	7	35.47
Khdrbs3	NP_034288.2	920	2	2.29	Ntn5	XP_006540948.1	182	5	28.95
Fam135b	NP_808487.2	541	4	7.79	Car11	NP_033930.1	321	9	29.55
Col22a1	XP_036015470.1	705	4	5.98	Dbp	NP_058670.2	341	4	12.36
Kcnk9	XP_036015221.1	922	5	5.72	Sphk2	XP_036009169.1	662	0	0.00
Trappc9	NP_850993.2	818	4	5.15	Rpl18	XP_030098107.1	1214	1	0.87
Chrac1	NP_444298.1	715	4	5.90	Fam83e	NP_001028342.2	105	3	30.11
Ago2	NP_694818.3	1555	3	2.03	Spaca4	NP_081331.2	284	6	22.27
Ptk2	XP_030104190.1	1356	1	0.78	Sult2b1	NP_059493.2	436	1	2.42
Dennd3	NP_001074535.1	295	1	3.57	Lmtk3	XP_030098591.1	585	2	3.60
Slc45a4	XP_017171852.1	241	1	4.37	Cyth2	NP_001106171.1	566	1	1.86
Gpr20	NP_775541.1	250	1	4.22	Kcnj14	XP_036008765.1	198	4	21.29
Ptp4a3	NP_001159862.1	473	4	8.91	Grwd1	NP_700468.2	924	1	1.14
Mroh5	NP_001028537.1	34	1	31.00	Grin2d	XP_036008583.1	550	2	3.83
Ndufb4c	NP_001348976.1	468	0	0.00	Kdelr1	NP_598711.1	698	0	0.00
Gm6569	XP_006521675.2	0	0	#DIV/0!	Syngr4	NP_067457.2	108	2	19.52
Adgrb1	XP_006520332.1	330	2	6.39	Tmem143	XP_006541252.1	100	0	0.00
Mroh4	XP_011244026.1	180	1	5.86	Emp3	NP_034259.2	491	2	4.29
Arc	NP_001263613.1	389	0	0.00	Ccdc114	XP_006540827.1	239	3	13.23
Jrk	NP_032441.4	283	1	3.72	Abcc6	NP_061265.2	1215	7	6.07
Psca	NP_082492.1	388	9	24.45	Nomo1	NP_694697.3	275	2	7.66
Them6	NP_941009.1	65	0	0.00	Kcnj11	NP_034732.1	744	8	11.33
Slurp1	NP_065265.1	211	11	54.94	Abcc8	NP_001344467.1	1112	9	8.53
Lypd2	NP_080947.1	278	9	34.12	Ush1c	NP_710143.2	936	6	6.76
Slurp2	NP_001075430.1	3	3	1053.90	Otog	NP_038652.2	394	9	24.07
Lynx1	NP_035968.1	201	8	41.95	Myod1	NP_034996.2	1430	6	4.42
Ly6d	NP_034872.1	306	9	31.00	Kcnc1	XP_006540710.1	1357	11	8.54
Ly6k	NP_083903.1	281	11	41.26	Sergef	XP_036009044.1	501	9	18.93
Gml	XP_006521296.1	175	11	66.25	Tph1	XP_036008805.1	696	7	10.60
Gml2	XP_006520515.1	104	10	101.34	Saal1	NP_084509.1	74	2	28.48
Cyp11b1	NP_001028401.2	291	0	0.00	Saa3	NP_035445.1	455	8	18.53
Cyp11b2	NP_034121.4	367	1	2.87	Saa4	NP_035446.1	429	4	9.83
Ly6m	NP_080205.1	45	10	234.20	Saa1	XP_006540788.1	722	4	5.84
Ly6e	XP_036015094.1	487	11	23.80	Saa2	NP_001344420.1	644	4	6.55
Ly6i	NP_001344934.1	90	10	117.10	Hps5	NP_001161336.1	229	1	4.60
Ly6a	NP_001258345.1	493	12	25.65	Gtf2h1	NP_001347004.1	785	1	1.34
Ly6c1	NP_001238985.1	513	8	16.44	Ldha	NP_001129541.2	1633	7	4.52
Ly6c2	NP_001344622.1	497	5	10.60	Ldhc	NP_001355290.1	1202	7	6.14
Ly6g	NP_001361159.1	510	2	4.13	Tsg101	NP_001335017.1	1136	3	2.78
Ly6f	XP_006520585.1	104	7	70.94	Uevld	NP_001035785.1	1488	2	1.42
Ly6l	NP_001333978.1	50	3	63.23	Spty2d1	NP_780527.2	224	0	0.00
Ly6h	XP_030104394.1	579	6	10.92	Tmem86a	NP_080712.1	186	0	0.00
Gpihbp1	NP_001348223.1	498	10	21.16	Ptpn5	XP_017177535.1	1416	0	0.00

Zfp41	NP_001038183.1	212	0	0.00	Mrgpra9	NP_001275730.1	26	0	0.00
Top1mt	NP_001342518.1	876	2	2.41	Mrgpra1	NP_694735.2	65	0	0.00
Rhpn1	XP_006520510.1	568	0	0.00	Mrgpra2a	NP_001166059.1	39	0	0.00
Mafa	NP_919331.1	263	0	0.00	Mrgpra2b	NP_694741.2	39	0	0.00
Zc3h3	XP_017172040.1	612	2	3.44	Mrgpra3	NP_694707.2	118	1	8.93
Gsdmd	NP_081236.1	470	2	4.48	Mrgpra4	NP_705744.2	33	1	31.94
Mroh6	XP_006520833.1	73	0	0.00	Mrgprx1	NP_997423.1	116	3	27.26
Naprt	NP_766195.2	539	1	1.96	Mrgprb5	NP_997421.1	50	0	0.00
Eef1d	XP_006521332.1	1029	5	5.12	Mrgprb4	NP_991364.1	92	1	11.46
Pycrl	NP_079688.2	654	3	4.83	Mrgprb8	NP_997422.1	40	0	0.00
Gfus	NP_001343934.1	651	1	1.62	Mrgprb1	NP_991379.3	22	0	0.00
Zfp623	XP_030104683.1	132	0	0.00	Mrgprx2	NP_001030040.2	63	0	0.00
Zfp707	NP_001348393.1	188	1	5.61	Mrgprb2	NP_780740.2	45	0	0.00
Ccdc166	NP_666171.2	46	0	0.00	Mrgprb3	NP_997420.1	35	0	0.00
Mapk15	XP_006521162.1	2081	1	0.51	Zdhhc13	NP_082307.1	538	1	1.96
Fam83h	XP_006520299.3	193	3	16.38	Csrp3	NP_001185770.1	1026	1	1.03
Iqank1	NP_001357849.1	955	3	3.31	E2f8	NP_001347741.1	670	1	1.57
Scrib	XP_006520301.1	1459	4	2.89	Nav2	XP_006541360.1	305	2	6.91
Puf60	XP_030104578.1	696	5	7.57	Dbx1	NP_001005232.1	425	3	7.44
Nrbp2	NP_001345288.1	94	2	22.42	Htatip2	XP_006541052.1	575	2	3.67
BC024139	NP_001136440.1	407	1	2.59	Prmt3	NP_598501.1	1252	0	0.00
Plec	NP_035247.2	2108	5	2.50	Slc6a5	XP_006540609.1	514	3	6.15
Parp10	NP_001157047.1	278	4	15.16	Nell1	NP_001032995.1	981	6	6.45
Grina	NP_075657.1	540	4	7.81	4933405O20F	NP_766489.1	777	2	2.71
Spatc1	XP_017172251.1	98	1	10.75	Ano5	XP_036008861.1	279	2	7.55
Smpd5	XP_006520290.1	169	4	24.94	Slc17a6	NP_543129.3	1126	4	3.74
Oplah	XP_006521571.1	285	0	0.00	Fancf	NP_001108559.1	226	1	4.66
Exosc4	NP_780608.1	1131	8	7.45	Gas2	XP_017177468.1	800	4	5.27
Gpaa1	NP_034461.1	239	11	48.51	Svip	NP_001153817.1	345	1	3.05
Cyc1	NP_079843.1	1081	8	7.80	1700015G11F	NP_001182530.1	151	1	6.98
Sharpin	XP_011243700.1	556	11	20.85	Luzp2	NP_848820.4	565	13	24.25
Maf1	NP_001158079.1	463	3	6.83	Siglech	XP_011249160.1	448	5	11.76
Hgh1	NP_067530.2	515	13	26.60	Tubgcp5	NP_666302.2	281	13	48.76
Tssk5	XP_017172248.1	987	3	3.20	Cyfip1	NP_001158134.1	882	13	15.53
Mroh1	XP_036015225.1	167	16	100.97	Nipa2	NP_076136.2	447	14	33.01
Bop1	NP_038509.1	1149	16	14.68	Nipa1	NP_705806.1	396	15	39.92
Scx	NP_942588.1	483	8	17.46	Herc2	NP_001347009.1	1012	14	14.58
Hsf1	NP_001318081.1	1235	12	10.24	Oca2	NP_068679.1	661	14	22.32
Dgat1	NP_034176.1	1075	16	15.69	Gabrg3	NP_032100.2	481	16	35.06
Scrt1	NP_570963.1	965	13	14.20	Gabra5	NP_001349091.1	1108	17	16.17
Fbxl6	NP_038937.2	565	17	31.71	Gabrb3	NP_001033790.1	1342	16	12.57
Slc52a2	NP_083919.1	196	13	69.90	Atp10a	NP_033858.2	771	16	21.87
Adck5	NP_766548.2	233	15	67.85	Ube3a	XP_017177614.1	1969	16	8.56
Cpsf1	NP_001157645.1	1142	12	11.07	Snurf	NP_001336624.1	1770	16	9.53
Slc39a4	XP_006521490.1	377	9	25.16	Ndn	NP_035012.2	661	16	25.51
Vps28	NP_080118.1	1525	12	8.29	Magel2	NP_038807.4	367	15	43.07
Tonsl	NP_898914.3	1525	11	7.60	Mkrn3	NP_035876.2	152	7	48.53
Cyhr1	XP_006521232.1	385	13	35.59	Peg12	NP_038816.1	324	12	39.03
Kifc2	NP_034760.2	1251	5	4.21	Chrna7	NP_031416.3	725	15	21.80
Foxh1	XP_006520499.1	844	3	3.75	Otud7a	XP_030098045.1	669	11	17.33
Ppp1r16a	NP_001345862.1	995	10	10.59	Klf13	NP_067341.2	515	8	16.37
Gpt	NP_877957.1	1321	0	0.00	E030018B13R	NP_001243240.1	0	0	#DIV/0!
Mfsd3	NP_081398.2	442	2	4.77	Trpm1	NP_001034193.2	524	10	20.11
Recql4	NP_478121.2	1077	9	8.81	Mtmr10	NP_766330.2	149	12	84.88
Lrrc14	XP_017172047.1	138	3	22.91	Fan1	NP_808561.2	346	5	15.23
Lrrc24	NP_932787.1	524	0	0.00	Mphosph10	NP_080759.2	820	1	1.29
C030006K11R	NP_663447.1	525	1	2.01	Mcee	NP_082902.1	930	8	9.07
Arhgap39	XP_030104354.1	279	9	34.00	Apba2	XP_030097866.1	1131	10	9.32
Zfp251	NP_001007569.1	218	7	33.84	Fam189a1	XP_006541260.1	416	2	5.07
Zfp7	NP_001345745.1	249	7	29.63	Nsmce3	NP_075728.1	232	4	18.17
Commd5	XP_036015432.1	107	3	29.55	Tjp1	XP_006540849.1	1572	5	3.35
Rpl8	NP_036183.1	2481	3	1.27	Tarsl2	NP_758514.2	1091	2	1.93
Zfp647	XP_030104410.1	181	2	11.65	Tm2d3	NP_835157.1	115	5	45.82
1110038F14Ri	NP_001334469.1	14	0	0.00	Pcsk6	NP_035178.1	1015	5	5.19
Mb	NP_038621.2	995	0	0.00	Snrpa1	NP_067311.4	1464	6	4.32
Apol6	NP_082286.1	278	12	45.49	Selenos	NP_077759.3	309	5	17.05

Rbfox2	NP_001273346.1	765	9	12.40	Chsy1	NP_001074632.1	352	11	32.93
Apol7a	NP_083695.2	434	6	14.57	Lrrk1	NP_666303.3	2644	5	1.99
Apol9a	NP_776147.1	359	5	14.68	Aldh1a3	NP_444310.3	870	2	2.42
Apol7b	NP_001020019.2	192	1	5.49	Asb7	NP_536691.2	1340	9	7.08
Apol10a	XP_017172100.1	191	4	22.07	Lins1	NP_690028.2	453	5	11.63
Apol11a	NP_001171004.1	143	2	14.74	Cers3	XP_006541064.1	413	3	7.66
Apol7c	NP_780600.1	245	4	17.21	Adamts17	NP_001029049.2	196	3	16.13
Apol10b	NP_808488.2	177	4	23.82	Lysmd4	NP_001177980.1	71	0	0.00
Apol11b	NP_001137158.1	170	3	18.60	Mef2a	NP_001278120.1	1479	7	4.99
Apol7e	NP_001128274.1	204	5	25.83	Lrrc28	XP_006541193.1	466	0	0.00
Apol9b	XP_030104641.1	405	1	2.60	Ttc23	XP_017167738.1	93	1	11.33
Apol8	XP_006520997.3	206	12	61.39	Synm	NP_997546.2	335	0	0.00
Myh9	NP_071855.2	1967	12	6.43	Igf1r	XP_006540704.1	2474	5	2.13
Txn2	NP_064297.1	1371	4	3.07	Pgpep1l	XP_006541378.1	78	2	27.02
Foxred2	NP_001017983.2	174	2	12.11	Arrdc4	NP_001036057.1	643	6	9.83
Eif3d	NP_061219.2	1219	2	1.73	Nr2f2	NP_899084.2	923	5	5.71
Cacng2	NP_031609.1	984	2	2.14	Mctp2	XP_030098441.1	289	8	29.17
Ift27	NP_080207.1	377	0	0.00	Rgma	NP_808408.2	362	7	20.38
Pvalb	NP_001317615.1	2095	2	1.01	Chd2	NP_001074814.2	1586	9	5.98
Ncf4	NP_032703.2	1098	7	6.72	Fam174b	NP_001156004.1	116	3	27.26
Csf2rb2	NP_001274318.1	614	7	12.02	St8sia2	XP_006540794.1	520	4	8.11
Csf2rb	NP_001345783.1	675	6	9.37	Slco3a1	NP_001033732.1	306	4	13.78
Tex33	NP_082798.1	249	3	12.70	Sv2b	NP_001347501.1	1020	1	1.03
Tst	NP_033463.1	804	4	5.24	Akap13	NP_083608.1	869	1	1.21
Mpst	XP_036015306.1	654	1	1.61	Klhl25	NP_083928.1	579	3	5.46
Kctd17	XP_036015504.1	365	4	11.55	Agbl1	NP_001186153.1	158	1	6.67
Tmprss6	XP_006521480.1	933	2	2.26	Ntrk3	NP_032772.3	2019	3	1.57
Il2rb	XP_006520540.1	813	7	9.07	Mrpl46	NP_075820.1	725	1	1.45
C1qtnf6	XP_006521515.1	213	5	24.74	Mrps11	NP_080774.2	1032	1	1.02
Sstr3	NP_001343890.1	919	2	2.29	Det1	XP_036009431.1	556	1	1.90
Rac2	NP_033034.1	2505	9	3.79	Aen	NP_001347638.1	670	2	3.15
Cyth4	NP_082471.2	697	6	9.07	Isg20	XP_030098687.1	786	2	2.68
Elfn2	NP_001345621.1	672	3	4.70	Acan	NP_001348429.1	1097	2	1.92
Mfng	NP_032621.1	423	3	7.47	Hapln3	XP_036009276.1	358	5	14.72
Card10	NP_570929.2	661	1	1.59	Mfge8	NP_001038954.1	584	1	1.80
Cdc42ep1	NP_081495.1	191	1	5.52	Abhd2	XP_036009150.1	452	3	6.99
Lgals2	NP_079898.2	240	1	4.39	Rlbp1	XP_030098106.1	521	3	6.07
Gga1	NP_666041.1	751	2	2.81	Fanci	NP_666058.2	596	3	5.30
Sh3bp1	NP_001303614.1	300	2	7.03	Polg	XP_006540771.1	950	5	5.55
Pdxp	NP_064667.2	300	2	7.03	Rhcg	NP_062773.2	419	4	10.06
Lgals1	NP_032521.1	900	2	2.34	Ticrr	NP_084111.1	309	4	13.64
Nol12	NP_598561.2	447	2	4.72	Kif7	XP_006540729.1	876	1	1.20
Triobp	XP_006520347.1	378	1	2.79	Plin1	NP_783571.2	464	4	9.09
H1f0	NP_032223.2	1327	4	3.18	Pex11a	NP_035198.1	505	0	0.00
Gcat	NP_001155184.1	797	1	1.32	Wdr93	XP_030098714.1	77	4	54.75
Galr3	NP_056553.2	797	1	1.32	Mesp1	NP_032614.2	181	1	5.82
Ankrd54	NP_001365919.1	1060	2	1.99	Mesp2	NP_032615.2	365	3	8.66
Eif3l	NP_660121.2	683	0	0.00	Anpep	XP_036008613.1	1339	2	1.57
Micall1	XP_036015335.1	528	0	0.00	Ap3s2	NP_033812.3	430	2	4.90
1700088E04R	NP_001297505.1	62	0	0.00	Arpin	NP_081696.1	63	4	66.91
Polr2f	NP_081507.1	2017	5	2.61	Zfp710	NP_780642.3	294	0	0.00
Sox10	NP_035567.1	1356	0	0.00	Idh2	NP_766599.2	1269	1	0.83
Pick1	NP_032863.2	713	1	1.48	Sema4b	NP_038687.2	344	0	0.00
Slc16a8	NP_065262.1	307	0	0.00	Cib1	NP_001278205.1	1446	1	0.73
Baiap2l2	XP_006520742.1	249	1	4.23	Gdpgp1	NP_848867.2	69	1	15.27
Pla2g6	XP_017172187.1	641	4	6.58	Ttll13	XP_006540981.1	145	0	0.00
Maff	NP_001291760.1	481	2	4.38	Ngrn	NP_113552.3	139	1	7.58
Tmem184b	XP_006520868.1	146	3	21.66	Vps33b	NP_835171.2	592	2	3.56
Csnk1e	XP_017172146.1	1181	1	0.89	Prc1	NP_001361553.1	818	3	3.87
Kcnj4	NP_032453.3	824	1	1.28	Rccd1	XP_006540993.1	493	4	8.55
Kdelr3	NP_598851.2	698	5	7.55	Unc45a	XP_011249077.1	749	3	4.22
Ddx17	NP_001035277.1	2055	1	0.51	Hddc3	NP_081088.1	477	6	13.26
Dmc1	XP_006520471.1	1447	0	0.00	Man2a2	XP_036008560.1	381	3	8.30
Fam227a	XP_017172268.1	48	5	109.78	Fes	NP_034324.2	1469	2	1.43
Cby1	XP_006521547.1	357	1	2.95	Furin	NP_001074923.1	1849	2	1.14
Tomm22	NP_766197.2	747	1	1.41	Blm	NP_031576.4	1001	1	1.05

Josd1	NP_083068.1	181	3	17.47	Crtc3	XP_006541258.1	272	0	0.00
Gtpbp1	NP_038846.2	381	1	2.77	Iqgap1	NP_057930.2	1311	1	0.80
Sun2	XP_030104362.1	649	4	6.50	Zscan2	NP_001369444.1	304	1	3.47
Dnal4	NP_059498.2	390	1	2.70	Wdr73	NP_082302.2	1573	3	2.01
Npcd	NP_001013378.2	360	3	8.78	Nmb	NP_001278209.1	352	4	11.98
Nptxr	NP_109614.2	598	1	1.76	Sec11a	XP_036009168.1	980	2	2.15
Cbx6	NP_083039.2	520	4	8.11	Zfp592	XP_036008876.1	420	4	10.04
Apobec3	XP_017172295.1	677	6	9.34	Alpk3	NP_473426.2	258	4	16.34
Cbx7	XP_011243992.1	594	3	5.32	Slc28a1	NP_001004184.2	318	3	9.94
Pdgfb	NP_035187.2	757	1	1.39	Pde8a	NP_032829.1	350	0	0.00
Rpl3	NP_038790.2	1594	1	0.66	Rps17	NP_033118.1	1105	2	1.91
Syngr1	NP_997591.1	591	3	5.35	Cpeb1	NP_001239455.1	769	4	5.48
Tab1	NP_079885.2	1286	2	1.64	Ap3b2	NP_067467.2	1276	6	4.96
Mgat3	XP_006520616.1	273	2	7.72	BC048679	NP_001344969.1	125	2	16.86
Mief1	NP_001344588.1	275	1	3.83	Fsd2	NP_766492.2	411	4	10.26
Atf4	NP_033846.2	1150	1	0.92	Whamm	NP_001004185.3	188	3	16.82
Rps19bp1	NP_780318.1	431	1	2.45	Homer2	NP_001157558.1	485	6	13.04
Cacna1i	XP_006521012.1	697	3	4.54	Ramac	NP_080273.1	127	0	0.00
Enthd1	XP_017172174.1	354	0	0.00	3110040N11F	NP_001357699.1	37	0	0.00
Grap2	NP_001276371.1	1196	1	0.88	Btbd1	NP_666305.2	583	2	3.62
Fam83f	NP_666098.2	123	0	0.00	Tm6sf1	NP_663350.2	209	1	5.04
Tnrc6b	NP_659061.2	385	0	0.00	Hdgfl3	XP_036009054.1	984	5	5.36
Adsl	NP_033764.2	1188	1	0.89	Bnc1	NP_031588.2	329	3	9.61
Sgsm3	XP_006520307.1	606	4	6.96	Sh3gl3	NP_059096.3	618	3	5.12
Mrtfa	NP_001076005.2	308	3	10.27	Adamtsl3	NP_001177303.1	355	2	5.94
Mchr1	NP_660114.1	933	3	3.39	Saxo2	NP_808562.3	26	0	0.00
Slc25a17	NP_035529.1	428	2	4.92	Efl1	NP_780526.2	1694	0	0.00
St13	NP_598487.1	646	1	1.63	Mex3b	NP_780575.2	292	1	3.61
Xpnpep3	NP_001334004.1	611	1	1.72	A530021J07R	XP_030098555.1	0	0	#DIV/0!
Dnajb7	NP_067292.2	498	6	12.70	Tmc3	NP_808363.3	154	7	47.90
Rbx1	NP_062686.1	1632	4	2.58	Stard5	NP_075866.2	154	1	6.84
Gm5218	XP_036015591.1	530	0	0.00	Il16	XP_006507448.1	1073	4	3.93
Ep300	XP_006521152.1	2751	5	1.92	Cfap161	NP_083611.2	93	5	56.66
L3mbtl2	NP_001276640.1	615	4	6.85	Tlnrd1	NP_109630.1	402	4	10.49
Chadl	NP_001157792.1	452	3	6.99	Mesd	NP_001361019.1	268	4	15.73
Rangap1	NP_001345551.1	1207	2	1.75	Cemip	NP_109653.3	277	1	3.80
Zc3h7b	XP_036015171.1	172	4	24.51	Abhd17c	NP_598483.2	191	0	0.00
Tef	XP_011243860.1	651	4	6.48	Arnt2	XP_006507302.1	712	2	2.96
Tob2	NP_065253.1	262	4	16.09	Fah	NP_034306.2	672	1	1.57
Phf5a	NP_081013.1	1150	6	5.50	Zfand6	NP_001347511.1	414	1	2.55
Aco2	NP_542364.1	1189	3	2.66	Olfr291	NP_666527.1	15	0	0.00
Polr3h	NP_084505.2	591	4	7.13	Olfr290	NP_666528.2	125	0	0.00
Csdc2	NP_663448.2	570	0	0.00	Vmn2r65	NP_001098650.1	33	0	0.00
Pmm1	NP_001268970.1	543	4	7.76	Vmn2r66	NP_001029050.3	27	0	0.00
1700029P11R	NP_079779.2	398	1	2.65	Vmn2r67	NP_001096049.1	7	0	0.00
Desi1	NP_001343470.1	297	2	7.10	Vmn2r68	NP_001098651.1	7	0	0.00
Xrcc6	XP_006520507.1	1477	4	2.85	Vmn2r69	NP_001098652.1	51	0	0.00
Snu13	NP_035612.2	1746	6	3.62	Vmn2r70	NP_001371843.1	16	0	0.00
4930407I10Ril	NP_001159947.1	108	0	0.00	Vmn2r71	NP_001371877.1	7	0	0.00
Mei1	XP_006521557.1	206	1	5.12	Vmn2r72	NP_001371844.1	27	0	0.00
Ccdc134	NP_766016.2	89	0	0.00	Vmn2r73	NP_001098656.1	8	0	0.00
Srebf2	NP_150087.1	865	0	0.00	Vmn2r74	NP_001371841.1	7	0	0.00
Shisa8	NP_001193950.1	142	0	0.00	Vmn2r75	NP_001096048.1	7	0	0.00
Tnfrsf13c	NP_001344687.1	647	1	1.63	Vmn2r76	NP_001371859.1	40	0	0.00
Cenpm	XP_030104557.1	530	2	3.98	Olfr310	NP_001011520.2	23	0	0.00
Septin3	XP_030104428.1	1059	1	1.00	Olfr309	NP_001011866.1	148	0	0.00
Wbp2nl	XP_017172261.1	180	0	0.00	Olfr308	NP_666832.1	76	0	0.00
Naga	NP_032695.3	368	1	2.86	Olfr307	NP_666828.1	22	0	0.00
Pheta2	NP_001344646.1	52	0	0.00	Olfr305	NP_666827.2	99	0	0.00
Smdt1	XP_036015460.1	428	1	2.46	Olfr304	NP_001011828.1	22	0	0.00
Ndufa6	NP_080263.1	729	4	5.78	Olfr303	NP_666830.1	117	0	0.00
Cyp2d22	NP_062797.3	402	1	2.62	Olfr301	NP_997601.1	23	0	0.00
Cyp2d11	NP_001098001.1	352	2	5.99	Olfr299	NP_001011767.1	34	0	0.00
Cyp2d10	XP_006520454.1	763	2	2.76	Olfr298	NP_001011751.1	42	0	0.00
Cyp2d9	NP_034136.2	694	1	1.52	Olfr297	NP_666829.2	38	0	0.00
Tdg-ps	XP_006521693.1	567	0	0.00	Olfr295	NP_667062.2	28	0	0.00

Cyp2d12	NP_958748.1	399	0	0.00	Olfr294	NP_001011750.2	49	0	0.00
Cyp2d34	NP_663449.2	582	0	0.00	Olfr293	NP_001011752.1	37	0	0.00
Cyp2d40	NP_076112.2	649	0	0.00	Olfr292	NP_666831.2	40	0	0.00
Cyp2d26	NP_083838.1	744	0	0.00	Folh1	XP_006508086.1	630	1	1.67
Tcf20	NP_038864.3	886	5	5.95	Vmn2r77	NP_001098658.1	24	0	0.00
Nfam1	NP_001258340.1	373	0	0.00	Vmn2r78	NP_001098659.1	7	0	0.00
Serhl	NP_075964.1	252	5	20.91	Vmn2r79	NP_001098660.1	20	2	105.39
Rrp7a	NP_083377.3	566	1	1.86	Nox4	XP_006508075.1	732	3	4.32
Poldip3	NP_001334011.1	706	3	4.48	Tyr	NP_035791.1	836	5	6.30
Cyb5r3	XP_030104133.1	697	2	3.02	Grm5	XP_006507247.1	1198	3	2.64
A4galt	XP_036015294.1	253	4	16.66	Ctsc	NP_034112.3	1013	1	1.04
Arfgap3	NP_001344686.1	852	2	2.47	Rab38	NP_082514.4	464	4	9.09
Pacsin2	NP_001345572.1	536	5	9.83	Tmem135	XP_030098876.1	119	2	17.71
Ttll1	NP_001344882.1	376	6	16.82	Fzd4	NP_032081.3	807	4	5.22
Bik	XP_006520417.1	276	0	0.00	Prss23	XP_036009434.1	514	2	4.10
Mcat	NP_001025185.1	1093	3	2.89	Me3	NP_852072.2	532	1	1.98
Tspo	NP_033905.3	652	1	1.62	Ccdc81	NP_001156451.1	91	0	0.00
Ttll12	NP_898838.2	468	3	6.76	Hikeshi	NP_001278215.1	393	4	10.73
Scube1	NP_001258402.1	466	2	4.52	Eed	NP_068676.1	1696	1	0.62
Mpped1	XP_017172062.1	788	3	4.01	Picalm	NP_666306.2	1081	3	2.92
Efcab6	XP_006521594.2	1079	1	0.98	Ccdc83	NP_899116.1	43	0	0.00
Sult4a1	NP_038901.3	1042	4	4.05	Sytl2	NP_001355807.1	325	2	6.49
Pnpla5	NP_083703.1	225	1	4.68	Ccdc89	NP_081574.1	138	0	0.00
Pnpla3	XP_036014984.1	527	3	6.00	Crebzf	NP_660133.2	340	0	0.00
Samm50	NP_848729.1	563	3	5.62	Tmem126a	NP_079736.1	471	3	6.71
Parvb	NP_573395.1	602	4	7.00	Tmem126b	NP_081010.1	213	3	14.84
Parvg	NP_001155972.1	620	1	1.70	Dlg2	XP_006507830.1	1772	1	0.59
Shisal1	NP_001334472.1	92	0	0.00	Ccdc90b	NP_079791.2	193	3	16.38
Rtl6	NP_808298.2	111	0	0.00	Ankrd42	NP_082941.2	1436	3	2.20
Prr5	NP_666173.4	376	1	2.80	Pcf11	NP_083354.3	835	0	0.00
Arhgap8	XP_006521531.1	307	2	6.87	Rab30	XP_030098934.1	302	2	6.98
Phf21b	XP_006521097.1	1035	5	5.09	Ddias	NP_001342055.1	252	0	0.00
Nup50	XP_036015118.1	496	0	0.00	Prcp	NP_082519.1	459	3	6.89
5031439G07R	NP_001028445.2	147	0	0.00	Tenm4	XP_036008945.1	417	0	0.00
Upk3a	NP_075967.2	292	1	3.61	Nars2	NP_705819.3	562	8	15.00
Fam118a	NP_001365769.1	108	0	0.00	Gab2	NP_034378.2	345	2	6.11
Smc1b	NP_536718.1	1274	2	1.65	Usp35	XP_006507886.1	530	13	25.85
Ribc2	NP_080633.2	226	1	4.66	Kctd21	NP_001034128.2	185	5	28.48
Fbln1	NP_034310.2	782	1	1.35	Alg8	NP_950200.2	289	12	43.76
Atxn10	NP_058539.2	432	1	2.44	Ndufc2	NP_077182.1	637	12	19.85
7530416G11R	XP_017172158.1	0	0	#DIV/0!	Thrsp	NP_033407.1	558	9	17.00
Wnt7b	NP_033554.3	876	2	2.41	Kctd14	XP_006507670.2	178	10	59.21
Ppara	XP_030104281.1	1568	0	0.00	Ints4	NP_081532.1	302	12	41.88
Cdpf1	NP_001158097.1	0	0	#DIV/0!	Aamdc	NP_001171417.1	156	9	60.80
Pkdrej	NP_035235.2	312	0	0.00	Rsf1	NP_001074736.1	614	13	22.31
Ttc38	NP_001028509.2	174	1	6.06	Clns1a	NP_076160.1	770	10	13.69
Gtse1	NP_001162143.1	706	1	1.49	Aqp11	NP_780314.1	285	9	33.28
Trmu	NP_082339.1	624	0	0.00	Pak1	NP_001344291.1	1371	2	1.54
Celsr1	XP_030104170.1	643	3	4.92	Gdpd4	NP_808364.2	356	5	14.80
Gramd4	XP_006520884.2	120	4	35.13	Myo7a	NP_032689.2	1525	6	4.15
Cerk	XP_011243890.1	562	4	7.50	Capn5	XP_006507338.1	398	3	7.94
Tbc1d22a	XP_036015258.1	366	3	8.64	Omp	NP_035140.1	363	3	8.71
Tafa5	XP_006520325.1	278	2	7.58	B3gnt6	NP_001074636.1	188	0	0.00
Zdhhc25	NP_081582.1	409	0	0.00	Acer3	NP_079684.2	378	8	22.30
Brd1	XP_030104370.1	732	3	4.32	Tsku	XP_036009011.1	685	8	12.31
Zbed4	XP_030104371.1	313	6	20.20	Gucy2d	NP_001124165.1	1058	5	4.98
Alg12	NP_001135829.1	291	5	18.11	Lrrc32	NP_001106850.1	662	2	3.18
Creld2	NP_083996.1	337	3	9.38	Emsy	XP_011240038.1	301	2	7.00
Pim3	NP_663453.1	1365	5	3.86	Thap12	NP_082686.1	145	0	0.00
Ttll8	XP_036015296.1	208	0	0.00	Wnt11	NP_001272721.1	950	0	0.00
Mlc1	NP_001351784.1	486	2	4.34	Uvrag	NP_848750.3	505	0	0.00
Mov10l1	XP_006521619.1	737	4	5.72	Dgat2	NP_080660.1	769	6	8.22
Panx2	XP_006521202.1	712	4	5.92	Mogat2	NP_803231.1	582	4	7.24
Trabd	NP_001365686.1	75	10	140.52	Map6	NP_034967.2	485	5	10.86
Selenoo	NP_082181.2	146	3	21.66	Serpinh1	NP_033955.2	936	2	2.25
Tubgcp6	NP_001156791.1	611	11	18.97	Gdpd5	NP_001349096.1	437	4	9.65

Hdac10	XP_006520606.1	1548	7	4.77	Klhl35	NP_082421.1	224	0	0.00
Mapk12	XP_036015350.1	2419	6	2.61	Rps3	NP_036182.1	2010	5	2.62
Mapk11	NP_035291.4	2631	6	2.40	Arrb1	NP_796205.1	2110	2	1.00
Plxnb2	XP_030104178.1	505	6	12.52	Tpbgl	NP_001182458.1	442	0	0.00
Dennd6b	XP_030104604.1	104	15	152.00	Slco2b1	NP_001239459.1	574	4	7.34
Ppp6r2	XP_006521461.1	618	9	15.35	Olfr520	NP_667274.2	68	1	15.50
Sbf1	NP_001074499.2	643	7	11.47	Olfr521	NP_666468.2	70	1	15.06
Adm2	NP_891558.1	312	1	3.38	Neu3	XP_030098621.1	268	7	27.53
Miox	NP_064361.2	367	6	17.23	Spcs2	XP_006508180.1	353	7	20.90
Lmf2	NP_849250.1	115	9	82.48	Xrra1	XP_017177834.1	517	7	14.27
Ncaph2	NP_001258530.1	377	4	11.18	Rnf169	XP_006507248.1	107	0	0.00
Sco2	NP_001104758.1	591	2	3.57	Chrdl2	NP_598470.3	213	8	39.58
Tymp	NP_612175.1	482	3	6.56	Pold3	NP_598453.1	528	9	17.96
Odf3b	XP_030104622.1	181	6	34.94	Lipt2	NP_080286.2	257	0	0.00
Klhdc7b	NP_001153650.2	133	4	31.70	Kcne3	NP_065599.1	235	7	31.39
Syce3	NP_001156352.1	166	2	12.70	Pgm2l1	NP_081905.1	919	0	0.00
Cpt1b	NP_034078.2	674	3	4.69	P4ha3	XP_006508007.1	368	0	0.00
Chkb	NP_031718.1	738	7	10.00	Ppme1	NP_082568.1	556	10	18.96
Mapk8ip2	NP_068740.3	1131	6	5.59	C2cd3	NP_001017985.2	326	3	9.70
Arsa	NP_033843.2	569	6	11.11	Ucp3	NP_033490.1	728	4	5.79
Shank3	NP_067398.3	2170	9	4.37	Ucp2	XP_030098236.1	878	3	3.60
Acr	NP_038483.1	499	2	4.22	Dnajb13	NP_705755.2	731	7	10.09
Rabl2	XP_006521389.1	1739	8	4.85	Coa4	NP_899093.1	69	0	0.00
Syt10	XP_011243998.1	593	5	8.89	Mrpl48	NP_942128.2	487	0	0.00
Alg10b	NP_001028613.1	265	3	11.93	Rab6a	NP_001157135.1	1039	2	2.03
Cpne8	NP_080091.1	332	5	15.87	Plekhb1	NP_001156654.1	364	0	0.00
Kif21a	XP_036015083.1	1163	7	6.34	Fam168a	XP_030098530.1	118	0	0.00
Abcd2	NP_001345896.1	546	2	3.86	Relt	NP_001345843.1	246	4	17.14
CN725425	XP_030104725.1	1	0	0.00	Arhgef17	NP_001074585.1	326	4	12.93
Slc2a13	NP_001028805.2	2919	9	3.25	P2ry6	XP_036008888.1	544	4	7.75
Lrrk2	NP_080006.3	3226	9	2.94	P2ry2	NP_032799.2	401	4	10.51
Smgc	NP_945121.1	21	0	0.00	Fchsd2	NP_950177.2	362	6	17.47
Cntn1	XP_017171911.1	1207	2	1.75	Atg16l2	XP_006508289.1	284	1	3.71
Pdzrn4	NP_001158065.1	864	4	4.88	Stard10	NP_001347389.1	304	6	20.80
Gxylt1	NP_001343935.1	116	8	72.68	Arap1	XP_006508249.1	681	4	6.19
Yaf2	NP_077151.3	432	5	12.20	Pde2a	NP_001137320.1	630	1	1.67
Zcrb1	NP_001334026.1	1304	10	8.08	Art2b	NP_064299.2	143	0	0.00
Pphln1	XP_006520913.1	128	7	57.64	Clpb	NP_001350920.1	1592	5	3.31
Prickle1	NP_001351775.1	1009	7	7.31	Phox2a	NP_032913.1	377	4	11.18
Adamts20	XP_011243896.1	294	7	25.09	Inppl1	NP_034697.2	1069	10	9.86
Pus7l	NP_766025.1	489	8	17.24	Folr2	NP_032061.1	490	2	4.30
Irak4	NP_084202.2	1330	3	2.38	Folr1	NP_032060.2	684	3	4.62
Twf1	XP_036015163.1	491	5	10.73	Anapc15	NP_001278277.1	197	3	16.05
Tmem117	NP_848904.1	35	1	30.11	Tomt	XP_017167892.1	326	5	16.16
Nell2	NP_001343880.1	1396	4	3.02	Lamtor1	NP_079881.2	476	0	0.00
Dbx2	XP_036015266.1	355	1	2.97	Lrrc51	NP_001344513.1	594	2	3.55
Ano6	NP_001240742.1	411	1	2.56	Numa1	XP_030097808.1	624	3	5.07
Arid2	NP_780460.3	932	2	2.26	Il18bp	NP_001350911.1	329	1	3.20
Scaf11	XP_011244038.1	562	1	1.88	Rnf121	XP_006508338.1	156	1	6.76
Slc38a1	XP_030104117.1	404	1	2.61	Xndc1	NP_001273618.1	409	4	10.31
Slc38a2	XP_036015447.1	501	2	4.21	Xntrpc	NP_035774.2	409	5	12.88
Slc38a4	NP_001344988.1	698	1	1.51	Trpc2	NP_001103367.1	409	6	15.46
Amigo2	NP_001158035.1	338	3	9.35	Art5	XP_030097884.1	199	10	52.96
Pced1b	XP_030104421.1	177	0	0.00	Art1	XP_036008500.1	273	2	7.72
Rpap3	NP_082279.1	916	1	1.15	Chrna10	NP_001074893.1	459	10	22.96
Endou	NP_001162164.1	167	0	0.00	Nup98	XP_030098484.1	1317	11	8.80
Rapgef3	XP_030104384.1	652	0	0.00	Pgap2	NP_001369532.1	291	12	43.46
Slc48a1	NP_080629.1	178	1	5.92	Rhog	NP_062512.1	1811	9	5.24
Hdac7	XP_030104529.1	1611	6	3.93	Stim1	NP_001360987.1	729	14	20.24
Vdr	NP_033530.2	1120	2	1.88	Rrm1	NP_033129.2	1962	10	5.37
Tmem106c	NP_001343437.1	74	1	14.24	Olfr543	NP_001011782.2	72	7	102.46
Col2a1	NP_001106987.2	973	3	3.25	Olfr544	NP_064685.2	112	6	56.46
Senp1	NP_001366502.1	626	1	1.68	Olfr545	NP_667051.1	97	5	54.32
Pfkm	XP_006520664.1	1258	1	0.84	Olfr547	NP_667290.2	242	1	4.35
Asb8	XP_030104679.1	1038	2	2.03	Olfr549	NP_667312.2	22	2	95.81
Ccdc184	NP_808384.2	183	0	0.00	Trim21	NP_033303.3	1098	4	3.84

Olfr288	NP_001011733.2	58	0	0.00	Olfr550	XP_006507941.1	10	0	0.00
Olfr287	NP_001011780.1	74	0	0.00	Olfr551	NP_666966.1	39	0	0.00
Olfr286	NP_001011779.1	27	0	0.00	Olfr552	NP_667313.2	72	0	0.00
H1f7	NP_081580.2	464	0	0.00	Olfr553	NP_997504.1	61	0	0.00
Zfp641	XP_017172093.1	181	0	0.00	Olfr554	NP_666437.2	61	0	0.00
Olfr285	NP_001011778.1	72	0	0.00	Olfr555	NP_667314.2	192	1	5.49
Olfr284	NP_666393.1	16	0	0.00	Olfr556	NP_666965.2	76	0	0.00
Olfr283	NP_667247.1	25	0	0.00	Trim68	XP_006507229.1	441	0	0.00
Olfr282	NP_666668.2	10	0	0.00	Olfr557	NP_666473.2	42	0	0.00
Olfr281	NP_666392.1	34	0	0.00	Olfr558	NP_667304.1	179	0	0.00
Lalba	XP_006520580.1	227	1	4.64	Olfr33	NP_667284.1	18	0	0.00
Olfr279	XP_036015322.1	38	0	0.00	Olfr559	NP_667323.1	19	0	0.00
Kansl2	XP_036015461.1	189	1	5.58	Olfr78	NP_570936.1	292	0	0.00
Ccnt1	NP_033963.1	1038	2	2.03	Olfr560	NP_667324.2	60	0	0.00
4930415O20R	NP_001188251.1	0	0	#DIV/0!	Olfr561	NP_667303.1	10	0	0.00
Adcy6	XP_006520370.1	1051	3	3.01	Olfr564	NP_666471.2	60	0	0.00
Cacnb3	XP_017171907.1	382	1	2.76	Olfr566	NP_001011536.2	34	0	0.00
Ddx23	NP_001074450.1	1401	0	0.00	Olfr568	NP_667302.2	75	0	0.00
Rnd1	NP_766200.1	1680	4	2.51	Olfr569	NP_667299.1	80	0	0.00
Ccdc65	NP_705738.1	233	0	0.00	Olfr570	NP_667321.1	11	0	0.00
Fkbp11	NP_077131.2	986	1	1.07	Olfr571	NP_667296.2	99	1	10.65
Arf3	NP_001342439.1	1861	7	3.96	Olfr572	NP_667300.1	122	0	0.00
Wnt10b	XP_006520955.1	679	4	6.21	Olfr574	NP_666472.2	79	0	0.00
Wnt1	NP_067254.1	1533	4	2.75	Olfr575	NP_667325.1	30	0	0.00
Ddn	NP_001013763.1	666	2	3.16	Olfr577	NP_667320.1	161	4	26.18
Prkag1	NP_058061.2	1309	5	4.03	Olfr578	NP_667326.1	134	4	31.46
Kmt2d	XP_017172165.1	1422	9	6.67	Olfr582	NP_667264.1	187	3	16.91
Rhebl1	XP_030104599.1	1462	8	5.77	Olfr583	NP_666968.1	46	1	22.91
Dhh	NP_031883.1	765	6	8.27	Olfr584	NP_667265.1	46	1	22.91
Lmbr1l	NP_083374.1	193	8	43.68	Olfr585	NP_667298.2	34	0	0.00
Tuba1b	NP_035784.1	1947	4	2.17	Olfr586	NP_667322.1	86	0	0.00
Tuba1a	NP_035783.1	2058	4	2.05	Olfr589	NP_667263.1	348	1	3.03
Tuba1c	NP_033474.1	1795	4	2.35	Olfr591	NP_001011847.1	127	2	16.60
Prph	XP_006520695.1	203	3	15.57	Olfr592	NP_997439.2	96	0	0.00
Troap	NP_084435.1	503	4	8.38	Olfr593	NP_666492.1	144	1	7.32
C1ql4	XP_030104424.1	85	2	24.80	Olfr594	NP_997026.1	77	0	0.00
Dnajc22	NP_789805.1	775	3	4.08	Usp17ld	NP_001001559.2	278	0	0.00
Spats2	NP_631879.1	58	0	0.00	Olfr596	NP_001177310.1	50	0	0.00
Kcnh3	XP_006520546.1	592	1	1.78	Olfr597	NP_001011845.2	34	0	0.00
Mcrs1	XP_030104500.1	426	0	0.00	Olfr598	NP_001011793.1	83	0	0.00
Fam186b	XP_006521235.1	13	6	486.42	Olfr599	NP_666942.1	136	0	0.00
Prpf40b	NP_001357561.1	656	3	4.82	Olfr600	NP_667257.2	170	0	0.00
Fmnl3	NP_001297552.1	379	2	5.56	Olfr601	NP_666426.2	166	0	0.00
Tmbim6	XP_036014961.1	591	1	1.78	Olfr603	NP_667281.2	50	0	0.00
Nckap5l	XP_006521185.1	101	3	31.30	Usp17lc	NP_034219.3	191	0	0.00
Bcdin3d	NP_083512.2	257	3	12.30	Olfr605	NP_001011854.2	105	0	0.00
Faim2	NP_001033747.1	1347	9	7.04	Olfr606	NP_667305.1	65	0	0.00
Aqp2	NP_033829.3	772	3	4.10	Olfr607	NP_001357824.1	14	0	0.00
Aqp5	NP_033831.1	698	3	4.53	Olfr608	NP_666967.2	110	0	0.00
Aqp6	NP_780296.1	389	4	10.84	Olfr609	NP_667293.2	64	0	0.00
Racgap1	XP_036015328.1	1050	4	4.01	Olfr610	NP_667292.2	91	0	0.00
Asic1	XP_006520360.1	588	5	8.96	Olfr611	NP_666938.2	72	0	0.00
Smarcd1	NP_114030.2	811	3	3.90	Olfr612	NP_001186956.1	63	0	0.00
Gpd1	NP_034401.1	1004	7	7.35	Olfr613	NP_667311.3	19	0	0.00
Cox14	NP_899079.1	324	1	3.25	Olfr615	NP_667291.1	341	1	3.09
Cers5	NP_082291.1	404	4	10.43	Olfr616	NP_667310.2	421	0	0.00
Lima1	NP_001107017.1	628	0	0.00	Olfr617	NP_667052.1	64	0	0.00
Fam186a	XP_030104645.1	45	1	23.42	Olfr618	NP_667258.2	92	2	22.91
Larp4	NP_001074417.1	1040	5	5.07	Olfr619	NP_667287.2	123	0	0.00
Dip2b	XP_011243931.1	425	4	9.92	Olfr620	NP_667023.2	81	0	0.00
Atf1	XP_006520414.1	471	0	0.00	Olfr622	NP_667294.1	64	0	0.00
Tmprss12	NP_898932.2	159	0	0.00	Olfr623	NP_667333.2	69	0	0.00
Mettl7a1	XP_006521452.1	739	4	5.70	Olfr624	NP_001011865.2	154	0	0.00
Mettl7a3	XP_011244014.1	512	2	4.12	Olfr243	NP_001020557.1	396	2	5.32
Mettl7a2	NP_955771.2	548	3	5.77	Olfr628	NP_667308.2	55	0	0.00
Methig1	NP_001019843.1	572	0	0.00	Olfr629	NP_667032.2	19	0	0.00

Higd1c	NP_001002900.1	97	1	10.86	Olfr630	NP_667309.1	80	1	13.17
Slc11a2	XP_011243789.1	880	0	0.00	Olfr69	NP_038649.2	117	3	27.02
Letmd1	NP_598854.1	124	0	0.00	Olfr68	NP_038648.2	113	1	9.33
Csrnp2	NP_700456.1	148	0	0.00	Olfr67	NP_038647.2	62	1	17.00
Tfcp2	XP_017172027.1	384	0	0.00	Hbb-bt	NP_032246.2	499	4	8.45
Pou6f1	XP_030104278.1	400	0	0.00	Hbb-bs	NP_001188320.1	617	6	10.25
Dazap2	NP_036003.2	310	0	0.00	Hbb-bh2	XP_006508068.1	286	2	7.37
Smagp	NP_778157.2	139	0	0.00	Hbb-bh1	NP_032245.1	627	10	16.81
Bin2	NP_001355595.1	807	0	0.00	Hbb-y	NP_032247.1	457	11	25.37
Cela1	NP_291090.2	410	0	0.00	Olfr66	NP_038646.2	94	2	22.42
Galnt6	XP_030104297.1	298	2	7.07	Olfr64	NP_038645.2	73	0	0.00
Slc4a8	NP_067505.2	409	2	5.15	Olfr65	NP_038644.2	48	2	43.91
Scn8a	XP_017172005.1	1178	1	0.89	Olfr631	XP_017177762.1	46	4	91.64
Fignl2	NP_001201840.1	654	1	1.61	Olfr632	NP_667330.1	104	2	20.27
Ankrd33	NP_659039.1	64	0	0.00	Olfr633	NP_666466.1	66	0	0.00
Acvrl1	NP_001264184.1	1285	2	1.64	Olfr635	NP_667329.2	39	2	54.05
Acvr1b	NP_031421.1	1507	2	1.40	Olfr638	NP_667331.1	93	3	34.00
Grasp	NP_062391.3	944	3	3.35	Olfr639	NP_667295.1	136	0	0.00
Nr4a1	XP_017171925.1	942	4	4.48	Olfr640	NP_667033.2	91	0	0.00
Atg101	NP_080842.1	362	0	0.00	Olfr641	NP_667283.1	48	0	0.00
Krt80	NP_083046.2	295	1	3.57	Olfr642	NP_666441.1	186	0	0.00
Krt7	NP_149064.1	615	2	3.43	Olfr643	NP_667288.1	12	0	0.00
Krt87	NP_001003668.2	272	10	38.75	Olfr644	NP_667332.1	10	0	0.00
Krt88	NP_079763.1	46	1	22.91	Olfr645	NP_997027.1	14	0	0.00
Krt81	NP_001159629.1	281	2	7.50	Olfr646	XP_017177763.1	96	2	21.96
Krt86	NP_034797.1	219	2	9.62	Ubqln5	NP_081910.1	1999	2	1.05
Krt83	NP_001188252.1	189	2	11.15	Ubqln3	NP_001371113.1	2082	3	1.52
Krt84	NP_032500.2	357	3	8.86	Ubqlnl	NP_941026.2	2139	7	3.45
Krt82	NP_444479.2	246	2	8.57	Olfm5	NP_766493.1	173	0	0.00
Krt90	NP_808385.2	101	2	20.87	Olfr648	NP_666962.1	51	5	103.32
Krt75	NP_579935.1	274	2	7.69	Olfr649	NP_667266.1	42	6	150.56
Gm5414	NP_001003670.1	131	17	136.77	Trim6	XP_006508427.1	443	8	19.03
Krt6b	NP_034799.2	293	2	7.19	Trim34a	NP_001344608.1	473	7	15.60
Krt6a	NP_032502.3	330	1	3.19	Trim5	XP_036009254.1	466	6	13.57
Krt5	NP_081287.1	738	1	1.43	Trim12a	NP_001355682.1	305	1	3.46
Krt71	NP_064340.1	869	0	0.00	Trim34b	NP_001230845.1	304	4	13.87
Krt72	NP_998893.1	335	2	6.29	Trim12c	NP_783608.3	320	8	26.35
Krt73	NP_997650.1	258	2	8.17	Gm38525	XP_006508442.1	237	1	4.45
Krt2	XP_030104217.1	402	3	7.86	Trim30b	NP_783579.1	237	1	4.45
Krt1	NP_032499.2	550	2	3.83	Trim30c	NP_001365622.1	322	6	19.64
Krt77	XP_006521203.1	304	2	6.93	Trim30a	NP_033125.2	717	6	8.82
Krt76	NP_001028349.1	258	2	8.17	Trim30d	NP_954597.2	436	4	9.67
Krt4	NP_032501.2	425	1	2.48	Olfr651	NP_667024.1	13	0	0.00
Krt79	NP_666175.1	272	1	3.87	Olfr652	NP_667259.2	41	5	128.52
Krt78	XP_011243982.1	232	1	4.54	Olfr653	NP_667285.2	54	0	0.00
Krt8	NP_112447.2	1022	1	1.03	Olfr654	NP_666491.1	27	7	273.23
Krt18	NP_034794.2	980	19	20.43	Olfr655	NP_667031.2	24	0	0.00
Eif4b	NP_663600.2	1905	9	4.98	Olfr656	NP_667286.1	63	0	0.00
Tns2	XP_030104299.1	1125	12	11.24	Olfr657	NP_666424.2	75	0	0.00
Spryd3	XP_006520949.1	346	13	39.60	Olfr658	NP_667260.1	44	0	0.00
Igfbp6	NP_032370.2	395	12	32.02	Olfr659	NP_667261.1	150	1	7.03
Soat2	NP_666176.1	662	13	20.70	Olfr661	NP_666959.1	151	0	0.00
Csad	XP_030104441.1	588	12	21.51	Olfr663	NP_001011757.1	152	1	6.93
Zfp740	XP_030104592.1	361	14	40.87	Usp17le	XP_011240180.1	368	0	0.00
Itgb7	XP_006520545.1	945	14	15.61	Usp17lb	NP_001345851.1	322	0	0.00
Rarg	NP_035374.3	728	16	23.16	Usp17la	NP_031913.1	232	0	0.00
Mfsd5	NP_598861.1	143	18	132.66	Olfr665	NP_667025.1	19	0	0.00
Espl1	NP_001343241.1	1421	16	11.87	Olfr666	NP_667307.1	85	0	0.00
Pfdn5	NP_081320.2	1089	18	17.42	Olfr667	NP_667271.2	35	0	0.00
Myg1	NP_068359.1	556	13	24.64	Olfr668	NP_667270.1	92	0	0.00
Aaas	NP_700465.2	329	5	16.02	Olfr669	NP_667254.1	41	0	0.00
Sp7	XP_006520581.1	847	5	6.22	Olfr670	NP_997029.1	123	0	0.00
Sp1	NP_038700.2	1102	3	2.87	Olfr671	XP_017177759.1	110	0	0.00
Amhr2	NP_001343504.1	1026	5	5.14	Olfr672	NP_666971.1	78	2	27.02
Prr13	NP_079661.1	119	9	79.71	Olfr675	NP_001011848.1	112	2	18.82
Pcbp2	XP_006520645.1	1125	9	8.43	Olfr676	XP_017177766.1	142	2	14.84

Map3k12	XP_017172113.1	444	7	16.62	Olfr677	NP_666470.1	165	1	6.39
Tarbp2	NP_033345.2	476	5	11.07	Olfr678	NP_666969.1	190	4	22.19
Npff	NP_061257.1	333	4	12.66	Olfr679	NP_667255.1	91	1	11.58
Atf7	NP_001296999.1	479	0	0.00	Olfr681	NP_997440.2	110	2	19.16
Atp5g2	NP_080744.1	614	2	3.43	Olfr683	NP_667256.1	176	3	17.96
Calcoco1	NP_080468.1	187	2	11.27	Olfr684	NP_997132.1	301	5	17.51
Hoxc13	NP_034594.1	460	11	25.20	Olfr685	NP_001011857.1	220	4	19.16
Hoxc12	NP_034593.1	345	8	24.44	Olfr686	NP_667280.1	105	1	10.04
Hoxc11	NP_001020013.1	332	8	25.40	Olfr687	XP_011240257.2	20	1	52.70
Hoxc10	NP_034592.2	413	8	20.41	Olfr688	NP_001011533.2	31	1	34.00
Hoxc9	XP_036015057.1	457	8	18.45	Olfr689	NP_666961.1	82	1	12.85
Hoxc8	NP_034596.1	488	9	19.44	Olfr690	NP_064686.2	54	1	19.52
Hoxc6	XP_006520531.1	469	8	17.98	Olfr691	NP_667272.1	111	1	9.49
Hoxc5	NP_783857.1	345	10	30.55	Olfr692	NP_666467.1	98	1	10.75
Hoxc4	NP_038581.2	427	10	24.68	Gm5901	NP_001369348.1	1	0	0.00
Smug1	XP_017172241.1	450	1	2.34	Fam160a2	XP_030098895.1	79	0	0.00
Cbx5	NP_001070257.1	1168	3	2.71	Cnga4	XP_017177672.1	415	9	22.86
Hnrnpa1	NP_001034218.1	2288	3	1.38	Cckbr	NP_031653.1	871	1	1.21
Nfe2	XP_036015117.1	464	3	6.81	Cavin3	NP_082720.1	329	1	3.20
Copz1	NP_062791.1	701	2	3.01	Smpd1	NP_035551.1	643	0	0.00
Gpr84	XP_006521617.1	482	2	4.60	Apbb1	XP_017177435.1	725	2	2.91
Zfp385a	NP_038894.2	189	1	6.20	Hpx	NP_059067.2	992	0	0.00
Itga5	NP_034707.5	1003	0	0.00	Trim3	NP_001272802.1	712	1	1.48
Gtsf1	NP_083073.1	419	1	3.14	Arfip2	XP_006508380.1	453	0	0.00
Gtsf2	NP_808299.1	192	1	7.32	Timm10b	NP_001346981.1	735	2	2.87
Nckap1l	NP_705725.1	867	1	1.74	Dnhd1	NP_001357732.1	618	1	1.71
Pde1b	NP_032826.1	505	1	3.21	Rrp8	NP_598712.1	806	1	1.31
Ppp1r1a	NP_067366.1	461	0	0.00	Ilk	NP_034692.2	1565	2	1.35
Glycam1	NP_032160.1	208	0	0.00	Taf10	NP_064408.2	666	2	3.16
Mucl2	NP_033293.2	87	0	0.00	Tpp1	NP_034036.1	264	0	0.00
Olfr161	NP_667071.1	24	0	0.00	Dchs1	XP_006507718.1	493	1	2.14
Mefv	XP_006522424.1	803	4	9.55	Mrpl17	NP_079577.1	868	2	2.43
Zfp263	NP_683726.2	453	2	7.75	Gvin2	XP_017177385.1	211	0	0.00
Olfr15	NP_032788.2	84	0	0.00	Gvin1	XP_017167845.1	191	0	0.00
Zfp174	XP_011244247.1	241	2	12.49	Olfr693	NP_666664.1	51	0	0.00
Zfp597	NP_001028331.2	233	1	6.03	Olfr694	NP_666663.2	27	0	0.00
Naa60	NP_083366.1	386	2	6.83	Olfr695	NP_666809.1	60	0	0.00
1700037C18R	NP_082760.2	35	0	0.00	Olfr697	NP_666810.1	27	0	0.00
Cluap1	XP_017172646.2	272	1	4.31	Olfr698	NP_666813.2	32	0	0.00
Nlrc3	XP_011244205.1	377	1	2.94	Olfr699	NP_001011862.1	71	0	0.00
Slx4	XP_036015901.1	323	0	0.00	Olfr700	NP_666811.1	112	0	0.00
Dnase1	NP_001344072.1	2134	4	1.98	Olfr701	XP_017167724.1	27	1	39.03
Trap1	NP_080784.1	2353	6	2.69	Olfr702	NP_666808.1	22	1	47.90
Crebbp	XP_006521817.1	3043	6	2.08	Olfr703	NP_666807.1	51	0	0.00
Adcy9	NP_033754.2	965	3	3.28	Olfr704	NP_001011749.1	32	0	0.00
Srl	NP_001334091.1	508	2	4.15	Olfr705	NP_667243.2	113	0	0.00
Tfap4	NP_112459.1	365	3	8.66	Olfr706	NP_666465.1	61	0	0.00
Glis2	XP_036016081.1	703	3	4.50	Olfr707	NP_001005570.2	10	0	0.00
Pam16	NP_079847.1	400	1	2.63	Olfr1532-ps1	NP_001011542.1	39	0	0.00
Coro7	NP_084481.3	224	0	0.00	Olfr710	NP_666812.1	116	0	0.00
Vasn	NP_647468.2	689	0	0.00	Olfr6	NP_996780.2	82	0	0.00
Dnaja3	NP_001128584.1	713	2	2.96	Olfr711	NP_667246.2	14	0	0.00
Nmral1	NP_001277690.1	440	0	0.00	Olfr2	NP_035113.1	145	0	0.00
Hmox2	NP_001365928.1	701	0	0.00	Olfr713	NP_667245.1	116	0	0.00
Cdip1	NP_001345010.1	240	2	8.78	Olfr714	NP_667244.2	114	0	0.00
4930562C15R	NP_084468.1	29	0	0.00	Olfr17	NP_065623.2	91	0	0.00
Ubald1	NP_663334.1	78	0	0.00	Olfr715b	NP_001156412.1	158	0	0.00
Mgrn1	NP_001365942.1	582	2	3.62	Olfr715	NP_666991.1	80	0	0.00
Nudt16l1	NP_080115.1	135	0	0.00	Olfr716	NP_666815.1	86	0	0.00
Anks3	XP_030105150.1	1035	1	1.02	Nlrp14	NP_001002894.2	324	0	0.00
Septin12	NP_001360874.1	543	2	3.88	Rbmxl2	NP_083936.1	399	0	0.00
Smim22	NP_001240731.1	53	0	0.00	Syt9	XP_006508162.1	570	0	0.00
Rogdi	NP_573448.2	175	0	0.00	Olfml1	NP_766495.1	303	3	10.43
Glyr1	XP_030105164.1	1409	0	0.00	Ppfibp2	XP_030098095.1	195	3	16.21
Ubn1	XP_036015690.1	429	2	4.91	Cyb5r2	XP_017177792.1	465	3	6.80
Ppl	NP_032935.2	1193	2	1.77	Ovch2	NP_766496.2	260	3	12.16

Sec14l5	NP_001121197.1	416	3	7.60	Olfr467	NP_001005488.1	193	1	5.46
Nagpa	NP_038824.2	233	1	4.52	Olfr469	NP_666637.1	41	0	0.00
AU021092	XP_036015807.1	49	0	0.00	Olfr470	NP_666636.1	22	0	0.00
Alg1	XP_036015739.1	757	2	2.78	Olfr472	NP_666985.1	13	0	0.00
Eef2kmt	NP_081722.1	258	2	8.17	Olfr473	NP_666986.1	63	0	0.00
Rbfox1	XP_036015827.1	1668	6	3.79	Olfr474	NP_666706.1	56	0	0.00
Tmem114	NP_083346.1	97	4	43.46	Olfr476	NP_667135.1	45	0	0.00
Mettl22	NP_666359.1	129	1	8.17	Olfr477	NP_667137.1	109	0	0.00
Abat	NP_766549.2	716	4	5.89	Olfr478	NP_666945.1	37	0	0.00
Tmem186	NP_079984.2	62	8	135.99	Olfr479	NP_001011742.1	59	0	0.00
Pmm2	NP_058577.1	510	3	6.20	Olfr480	NP_064687.2	171	1	6.16
Carhsp1	XP_006522410.1	626	5	8.42	Olfr481	NP_667136.1	53	0	0.00
Usp7	XP_006522201.1	1630	3	1.94	Gm4199	XP_036009543.1	127	0	0.00
1810013L24Ri	NP_001074869.1	60	6	105.39	Olfr482	NP_666944.1	73	0	0.00
Grin2a	NP_032196.2	1345	1	0.78	Olfr483	NP_666946.1	23	0	0.00
Rpl39l	NP_080870.1	963	0	0.00	Olfr484	NP_666710.1	22	0	0.00
Atf7ip2	XP_011244341.1	99	0	0.00	Olfr485	NP_001011810.2	45	0	0.00
Emp2	NP_031955.2	348	1	3.03	Olfr486	NP_666707.1	23	0	0.00
Tekt5	NP_001277930.1	201	3	15.73	Olfr487	NP_001011811.1	45	0	0.00
Nubp1	NP_036085.1	740	4	5.70	Olfr488	NP_666943.1	60	0	0.00
Tvp23a	NP_001345998.1	92	0	0.00	Olfr490	NP_666709.1	22	0	0.00
Ciita	NP_001289547.1	899	5	5.86	Olfr491	NP_666947.1	75	0	0.00
Dexi	NP_067403.2	183	5	28.80	Olfr493	NP_666422.1	117	0	0.00
Clec16a	XP_006522723.1	544	6	11.62	Olfr494	NP_666948.1	25	0	0.00
Socs1	NP_001258532.1	1253	7	5.89	Olfr495	NP_666476.1	22	0	0.00
Tnp2	NP_038722.3	560	4	7.53	Olfr497	NP_666949.1	59	0	0.00
Prm3	NP_038666.2	182	2	11.58	Olfr498	NP_666419.2	17	0	0.00
Prm2	NP_032959.1	574	2	3.67	Olfr502	NP_666950.1	59	0	0.00
Prm1	NP_038665.1	348	2	6.06	Olfr503	NP_001011527.1	17	1	61.99
Rmi2	NP_001156404.1	314	4	13.43	Olfr504	NP_001011858.1	171	1	6.16
Litaf	XP_030105082.1	481	3	6.57	Olfr506	NP_001011871.1	22	0	0.00
Snn	NP_033249.1	228	3	13.87	Olfr507	NP_666954.1	22	0	0.00
Txndc11	XP_006521722.1	1149	3	2.75	Olfr508	NP_666984.1	62	0	0.00
Zc3h7a	XP_006521727.2	145	2	14.54	Olfr509	NP_666484.1	69	0	0.00
Rsl1d1	NP_079822.1	1206	2	1.75	Olfr510	NP_666423.1	28	0	0.00
Gspt1	NP_001123480.1	1646	2	1.28	Olfr512	NP_666935.1	73	0	0.00
Tnfrsf17	NP_035738.1	361	0	0.00	Olfr513	NP_666934.1	57	0	0.00
Snx29	XP_006522729.1	263	1	4.01	Olfr514	NP_666937.1	108	0	0.00
Cpped1	NP_666179.2	422	1	2.50	Olfr516	NP_666936.1	173	0	0.00
Shisa9	XP_036016033.1	440	2	4.79	Olfr517	NP_001011846.1	36	0	0.00
Ercc4	NP_056584.2	766	0	0.00	Olfr518	NP_666418.1	73	0	0.00
Mrtfb	XP_030104964.1	369	1	2.86	Olfr519	NP_997043.1	87	0	0.00
Parn	NP_001345382.1	495	0	0.00	Nlrp10	NP_780741.1	64	0	0.00
Bfar	XP_030105108.1	310	2	6.80	Eif3f	NP_079620.2	1521	1	0.69
Pla2g10	NP_036117.1	287	2	7.34	Tub	NP_068685.1	1170	3	2.70
Rrn3	NP_001034610.1	461	7	16.00	Ric3	NP_001298090.1	244	4	17.28
Ntan1	NP_001333037.1	285	6	22.19	Lmo1	NP_001289134.1	604	5	8.72
Pdxdc1	XP_011244357.1	180	11	64.41	Stk33	XP_017177431.1	722	5	7.30
Mpv17l	NP_001276491.1	237	9	40.02	Trim66	NP_001164383.1	431	2	4.89
Ifitm7	NP_001257647.1	63	0	0.00	Rpl27a	NP_036105.2	1323	5	3.98
Bmerb1	XP_036015989.1	866	7	8.52	Denn2b	NP_084087.2	254	4	16.60
Marf1	XP_006522081.1	185	10	56.97	Akip1	NP_065641.1	124	1	8.50
Nde1	NP_001107557.1	810	11	14.31	BC051019	NP_001035790.1	11	0	0.00
Myh11	NP_001155247.1	1818	13	7.54	Ascl3	NP_064435.1	231	0	0.00
Cep20	XP_006522513.1	196	8	43.02	Tmem9b	NP_064434.1	124	0	0.00
Abcc1	NP_032602.1	908	8	9.29	Nrip3	NP_065635.1	877	0	0.00
Snai2	NP_035545.1	1385	4	3.04	Scube2	NP_064436.2	400	2	5.27
Efcab1	XP_006522537.1	832	2	2.53	Dennd5a	NP_001298068.1	295	2	7.15
Ube2v2	NP_001152823.1	1021	5	5.16	Tmem41b	XP_030098359.1	313	0	0.00
Mcm4	NP_032591.3	1691	7	4.36	Ipo7	NP_852658.2	1443	1	0.73
Prkdc	NP_035289.2	2677	7	2.76	Zfp143	XP_006507593.2	823	2	2.56
Mzt2	NP_001345932.1	167	1	6.31	Wee1	NP_033542.2	1358	0	0.00
Spidr	NP_666180.2	129	3	24.51	Swap70	NP_033328.3	292	0	0.00
Pkp2	NP_080439.1	681	2	3.10	Sbf2	XP_030098533.1	507	1	2.08
Yars2	NP_937889.1	1144	3	2.76	Adm	NP_033757.1	583	2	3.62
Dnm1l	NP_001021118.1	1890	5	2.79	Ampd3	NP_001359370.1	616	1	1.71

Fgd4	NP_001288746.1	537	0	0.00	Rnf141	XP_030098781.1	164	1	6.43
Olfr19	NP_666447.1	49	0	0.00	Lyve1	NP_444477.2	525	1	2.01
Spag6l	XP_017172552.1	1101	1	0.96	Mrvi1	NP_034956.2	284	1	3.71
Rpl31-ps12	NP_001245387.1	1072	0	0.00	Ctr9	NP_033457.2	1360	1	0.77
Igll1	NP_001177254.1	416	2	5.07	Eif4g2	NP_001035221.1	1488	3	2.12
Vpreb1	NP_058678.1	451	3	7.01	Galnt18	NP_001347832.1	328	1	3.21
Top3b	NP_001313505.1	872	4	4.83	Usp47	XP_006508336.1	1023	3	3.09
Ppm1f	XP_017172613.1	1301	7	5.67	Dkk3	NP_001347186.1	815	3	3.88
Mapk1	NP_001344044.1	3937	6	1.61	Mical2	NP_001180234.1	761	2	2.77
Ypel1	NP_001277982.1	224	6	28.23	Micalcl	NP_001368791.1	113	0	0.00
Ppil2	XP_006522510.1	1058	4	3.98	Parva	NP_065631.3	764	1	1.38
2610318N02R	NP_001345184.1	32	0	0.00	Tead1	NP_001160056.2	771	3	4.10
Sdf2l1	NP_071719.1	457	5	11.53	Rassf10	NP_780488.2	109	2	19.34
Ccdc116	NP_001360877.1	142	0	0.00	Arntl	XP_006507308.1	825	3	3.83
Ydjc	NP_001365723.1	91	1	11.58	Btbd10	NP_598461.1	242	3	13.06
Ube2l3	XP_006522067.1	1647	8	5.12	Pth	XP_017177534.1	928	5	5.68
Rimbp3	NP_001028510.2	245	1	4.30	Far1	NP_001347597.1	552	0	0.00
Hic2	NP_849253.2	386	5	13.65	Spon1	NP_663559.1	643	1	1.64
Tmem191c	NP_803424.2	46	2	45.82	Rras2	NP_080122.2	1966	7	3.75
Pi4ka	NP_001360884.1	2276	9	4.17	Copb1	NP_203534.1	1424	6	4.44
Serpind1	NP_001317976.1	772	8	10.92	Psma1	NP_036095.1	1566	7	4.71
Snap29	NP_075837.3	739	10	14.26	Pde3b	NP_035185.2	470	6	13.45
Crkl	NP_031790.2	1018	9	9.32	Cyp2r1	XP_006507904.1	399	8	21.13
Aifm3	XP_006522657.1	710	10	14.84	Calca	XP_030097892.1	925	3	3.42
Lztr1	NP_080084.2	275	11	42.16	Calcb	NP_473425.2	257	3	12.30
Thap7	XP_036016000.1	155	11	74.79	Insc	XP_006507722.1	419	5	12.58
Lrrc74b	NP_001138907.1	89	0	0.00	Sox6	XP_036008736.1	1005	1	1.05
P2rx6	NP_035158.2	128	10	82.34	1110004F10R	NP_062746.1	224	4	18.82
Slc7a4	NP_001346821.1	555	12	22.79	Plekha7	NP_001292114.1	808	3	3.91
Smpd4	XP_006522783.1	187	0	0.00	Rps13	NP_080809.1	1646	6	3.84
Ccdc74a	NP_001159636.1	90	0	0.00	Pik3c2a	NP_035213.2	2177	7	3.39
Med15	XP_017172661.1	482	11	24.05	Nucb2	NP_001347304.1	422	4	9.99
Klhl22	NP_001365953.1	527	8	16.00	Xylt1	NP_783576.2	289	1	3.65
Scarf2	XP_036015777.1	382	8	22.07	Rps15a	NP_733769.1	1355	4	3.11
Car15	NP_085035.1	188	2	11.21	Arl6ip1	NP_062292.1	387	2	5.45
Dgcr2	NP_001103220.1	214	7	34.47	Smg1	XP_006507740.1	2566	6	2.46
Tssk1	NP_033461.2	1374	1	0.77	Syt17	XP_006507265.3	352	0	0.00
Tssk2	NP_033462.2	1301	0	0.00	Itpripl2	NP_001028552.1	97	0	0.00
Ess2	NP_001075102.1	543	11	21.35	Coq7	NP_001291687.1	469	3	6.74
Gsc2	NP_083745.2	290	12	43.61	Tmc7	NP_766064.2	90	1	11.71
Slc25a1	NP_694790.1	650	10	16.21	Tmc5	NP_001098722.1	225	0	0.00
Vpreb2	XP_017172433.1	467	9	20.31	Gde1	NP_062526.1	392	2	5.38
Dgcr6	NP_034177.1	260	12	48.64	Ccp110	NP_001347723.1	539	0	0.00
Prodh	NP_035302.2	853	15	18.53	Vps35l	NP_082091.3	71	1	14.84
Rtn4r	NP_075358.1	1093	13	12.53	Knop1	NP_001161691.1	178	3	17.76
Ccdc188	XP_006522872.1	0	0	#DIV/0!	Iqck	NP_001361534.1	184	3	17.18
Zdhhc8	NP_001365948.1	342	16	49.31	Gprc5b	NP_001182703.1	416	2	5.07
Ranbp1	NP_035369.2	1490	17	12.02	Gpr139	NP_001019309.1	330	1	3.19
Trmt2a	NP_001074468.1	633	17	28.30	Gp2	XP_006508184.1	421	0	0.00
Dgcr8	XP_030105215.1	684	17	26.19	Umod	NP_033496.1	492	2	4.28
Tango2	XP_017172520.1	285	15	55.47	Pdilt	XP_030098854.1	1565	5	3.37
Arvcf	XP_036015643.1	543	16	31.05	Acsm5	XP_006507967.1	977	3	3.24
Comt	NP_031770.2	1103	18	17.20	Acsm2	NP_001171448.1	500	0	0.00
Txnrd2	NP_038739.2	1018	15	15.53	Acsm1	XP_006507277.1	1141	3	2.77
Gnb1l	XP_011244130.1	801	17	22.37	Acsm4	NP_848501.1	436	0	0.00
Tbx1	NP_001360867.1	603	14	24.47	Thumpd1	NP_663560.1	292	0	0.00
Gp1bb	NP_034457.1	581	7	12.70	Ppp1ccb	NP_001357876.1	3082	2	0.68
Septin5	NP_998779.2	1322	14	11.16	Acsm3	XP_017177552.1	1024	4	4.12
Cldn5	NP_038833.2	612	12	20.66	Eri2	NP_081974.3	222	1	4.75
Cdc45	NP_001155095.1	1136	13	12.06	Rexo5	XP_036009116.1	409	0	0.00
Ufd1	NP_035802.3	960	12	13.17	Dcun1d3	XP_036008898.1	321	0	0.00
2510002D24R	XP_036016030.1	40	4	105.39	Lyrm1	XP_036009372.1	54	0	0.00
Mrpl40	NP_035052.2	957	9	9.91	Dnah3	NP_001357735.1	693	0	0.00
Hira	NP_034565.2	865	4	4.87	Tmem159	XP_030098368.1	36	0	0.00
Olfr164	NP_666662.1	16	0	0.00	Zp2	NP_001361560.1	477	0	0.00
Olfr165	XP_017172473.1	12	0	0.00	Anks4b	NP_082361.2	1110	2	1.90

Olfr166	NP_667279.1	87	0	0.00	Crym	NP_057878.1	598	0	0.00
Olfr167	NP_667146.1	55	0	0.00	Abca14	XP_017167760.1	244	0	0.00
Olfr168	NP_666469.1	96	0	0.00	Abca15	NP_796187.2	240	0	0.00
Olfr169	NP_001011855.1	107	0	0.00	Abca16	XP_006507746.1	253	0	0.00
Olfr170	NP_667168.1	25	0	0.00	Uqcrc2	NP_080175.1	1207	2	1.75
Olfr171	NP_667169.2	106	0	0.00	Pdzd9	XP_006508214.1	55	6	114.97
Lamp3	NP_796330.2	401	0	0.00	Mosmo	XP_006507752.1	148	3	21.36
B3gnt5	XP_030104776.1	293	5	17.98	Vwa3a	XP_030098369.1	80	5	65.87
Klhl6	XP_036015812.1	631	8	13.36	Eef2k	NP_001254639.1	468	6	13.51
Klhl24	XP_036016067.1	846	9	11.21	Polr3e	NP_001366217.1	482	4	8.75
Yeats2	NP_001139402.1	764	12	16.55	Cdr2	NP_031698.2	475	7	15.53
Map6d1	NP_941001.2	129	12	98.04	Mfsd13b	XP_017167844.1	22	0	0.00
Parl	NP_001005767.1	611	11	18.97	Gm9234	XP_036009546.1	1921	1	0.55
Cyp2ab1	XP_017172442.1	145	3	21.80	Mettl9	XP_006508156.1	95	1	11.09
Abcc5	XP_006522271.1	707	9	13.42	Igsf6	NP_109616.1	531	1	1.98
Eif2b5	NP_758469.1	934	15	16.93	Otoa	XP_017177756.1	295	0	0.00
Dvl3	NP_001334105.1	1013	6	6.24	Hs3st2	XP_036008695.1	439	10	24.01
Ap2m1	NP_033809.1	1048	10	10.06	Usp31	NP_001028345.1	404	1	2.61
Abcf3	NP_038880.1	975	18	19.46	Scnn1g	NP_035456.1	556	11	20.85
Vwa5b2	XP_030105017.1	148	18	128.18	Scnn1b	NP_001258952.1	489	10	21.55
Alg3	NP_666051.2	383	16	44.03	Cog7	XP_030098374.1	355	12	35.62
Eef1akmt4-ec	NP_808810.1	395	8	21.34	Gga2	NP_083034.1	653	12	19.37
Eef1akmt4	NP_079738.2	395	7	18.68	Ears2	NP_080416.1	1067	13	12.84
Camk2n2	NP_082696.1	411	5	12.82	Ubfd1	NP_613055.2	633	5	8.32
Ece2	NP_647454.2	395	7	18.68	Ndufab1	NP_001347673.1	1942	12	6.51
Psmd2	XP_030104899.1	1167	12	10.84	Palb2	NP_001276774.1	478	11	24.25
Eif4g1	XP_006521998.1	1561	11	7.43	Dctn5	NP_067621.3	444	13	30.86
Fam131a	NP_598539.1	302	13	45.37	Plk1	NP_035251.3	2673	5	1.97
Clcn2	NP_034030.2	465	8	18.13	Ern2	XP_011240121.1	1099	11	10.55
Polr2h	NP_663607.1	1267	2	1.66	Chp2	NP_081639.1	1046	3	3.02
Thpo	XP_006522050.1	502	3	6.30	Prkcb	NP_001303601.1	1359	8	6.20
Chrd	XP_011244116.1	809	5	6.51	Cacng3	NP_062303.2	897	10	11.75
Gm6551	NP_001357823.1	14	0	0.00	Rbbp6	XP_036008696.1	1035	7	7.13
Gm6557	NP_001357822.1	14	0	0.00	Tnrc6a	XP_011240059.1	442	1	2.38
Teddm3	NP_079910.1	14	0	0.00	Slc5a11	NP_666310.1	344	0	0.00
Ephb3	XP_006521851.1	1417	1	0.74	Arhgap17	NP_001116113.1	439	2	4.80
Vps8	NP_001074835.1	443	1	2.38	Lcmt1	NP_079580.2	378	2	5.58
2510009E07R	NP_001001881.1	37	2	56.97	Aqp8	NP_031500.1	1030	3	3.07
Ehhadh	NP_076226.2	1353	2	1.56	Zkscan2	NP_001074798.1	260	2	8.11
Map3k13	XP_006522645.1	401	4	10.51	Hs3st4	NP_001239001.1	351	2	6.01
Tmem41a	NP_079969.1	112	3	28.23	4930571K23R	NP_001139231.1	47	0	0.00
Liph	NP_700453.1	244	4	17.28	Kdm8	NP_084118.1	544	1	1.94
Senp2	XP_006522751.1	336	3	9.41	Nsmce1	NP_001347588.2	309	6	20.46
Igf2bp2	NP_898850.2	584	3	5.41	Il4ra	NP_001008700.1	751	6	8.42
Tra2b	NP_001317483.1	1735	3	1.82	Il21r	NP_068687.1	570	4	7.40
Etv5	NP_001345357.1	664	2	3.17	Gtf3c1	NP_997122.1	254	4	16.60
Dgkg	NP_001333602.1	566	7	13.03	Katnip	XP_011240073.1	255	6	24.80
Crygs	NP_034097.1	189	1	5.58	Gsg1l	NP_001345814.1	199	3	15.89
Tbccd1	XP_006522629.1	104	1	10.13	Xpo6	NP_083092.2	1648	3	1.92
Dnajb11	NP_080676.3	741	1	1.42	Sbk1	XP_036008449.1	261	3	12.11
Ahsg	NP_038493.1	1179	8	7.15	Lat	XP_006507459.3	483	2	4.36
Fetub	NP_067539.1	810	6	7.81	Spns1	NP_076201.2	375	8	22.48
Hrg	NP_444406.2	869	5	6.06	Nfatc2ip	XP_006507493.1	1801	10	5.85
Kng2	NP_001095879.1	1256	6	5.03	Cd19	NP_001344020.1	1341	4	3.14
Kng1	NP_075614.1	1320	6	4.79	Rabep2	NP_085043.2	443	14	33.31
Eif4a2	NP_001334108.1	2129	1	0.50	Atp2a1	NP_031530.2	1341	10	7.86
Rfc4	NP_663455.1	1673	1	0.63	Sh2b1	XP_006507541.1	404	13	33.91
Adipoq	NP_033735.3	1364	4	3.09	Tufm	NP_766333.1	1554	12	8.14
St6gal1	NP_666045.1	479	2	4.40	Atxn2l	XP_006507788.1	813	8	10.37
Rtp1	NP_001004151.1	839	0	0.00	Eif3c	NP_666312.1	1219	5	4.32
Masp1	XP_036015702.1	467	6	13.54	Cln3	NP_001316718.1	425	8	19.84
Rtp4	XP_030105116.1	957	0	0.00	Apobr	XP_036008620.1	282	11	41.11
Sst	XP_030104869.1	1659	3	1.91	Il27	NP_663611.1	150	2	14.05
Rtp2	NP_001008231.1	770	0	0.00	Nupr1	NP_062712.1	284	0	0.00
Bcl6	NP_001334955.1	1283	5	4.11	Sgf29	XP_017167854.1	515	8	16.37
Lpp	XP_030104888.1	1166	6	5.42	Sult1a1	NP_598431.2	709	5	7.43

Tprg	NP_780374.1	96	2	21.96	Slx1b	NP_083696.2	338	5	15.59
Trp63	NP_001120731.1	881	4	4.79	Bola2	NP_780312.1	553	8	15.25
P3h2	NP_775555.1	347	8	24.30	Coro1a	XP_006507347.1	1304	11	8.89
Cldn1	NP_057883.1	683	5	7.72	Mapk3	NP_036082.1	4929	10	2.14
Cldn16	NP_444471.1	251	8	33.59	Gdpd3	NP_077190.2	385	11	30.11
Tmem207	NP_001095110.1	124	6	51.00	Ypel3	NP_081151.2	290	14	50.88
Il1rap	NP_001152789.1	288	0	0.00	Tbx6	NP_035668.2	583	15	27.12
Gmnc	NP_001272847.1	143	3	22.11	Ppp4c	NP_001347393.1	2500	16	6.74
Ostn	XP_036015814.1	239	5	22.05	Aldoa	NP_001170778.1	1162	15	13.60
Uts2b	NP_937809.1	257	5	20.50	Tlcd3b	XP_036009300.1	240	19	83.43
Ccdc50	XP_011244304.1	208	7	35.47	4930451I11Ri	XP_006508410.1	74	11	156.66
Fgf12	NP_034329.1	1428	6	4.43	Doc2a	NP_001355287.1	304	19	65.87
Mb21d2	NP_808386.2	207	3	15.27	Ino80e	XP_006507794.1	307	20	68.66
Plaat1	XP_017172511.1	234	1	4.50	Hirip3	NP_766334.1	478	19	41.89
Atp13a5	XP_036015848.1	624	0	0.00	Taok2	XP_030098581.1	315	19	63.57
Atp13a4	XP_017172444.1	688	0	0.00	Tmem219	NP_001361542.1	67	19	298.87
Opa1	XP_006522717.2	1046	2	2.02	Kctd13	NP_766335.1	394	20	53.50
Hes1	NP_032261.1	573	1	1.84	Asphd1	NP_001334586.1	139	20	151.64
Cpn2	NP_082180.2	896	4	4.70	Sez6l2	NP_659175.1	842	19	23.78
Lrrc15	XP_036016065.1	584	2	3.61	Cdipt	NP_001366189.1	1036	16	16.28
Gp5	NP_032174.2	727	2	2.90	Mvp	NP_542369.2	433	5	12.17
Atp13a3	XP_011244181.1	799	7	9.23	Pagr1a	NP_084516.1	159	15	99.42
Tmem44	XP_030104940.1	39	3	81.07	Prrt2	NP_001096033.1	719	15	21.99
Lsg1	NP_835170.1	1073	3	2.95	Maz	NP_001359450.1	635	13	21.58
Fam43a	NP_808300.1	99	3	31.94	Kif22	NP_663563.1	1328	13	10.32
Xxylt1	NP_941028.2	115	1	9.16	Zg16	NP_081194.1	182	10	57.91
Acap2	NP_084414.1	642	2	3.28	AI467606	NP_849232.1	84	9	112.92
Ppp1r2	NP_080076.1	496	3	6.37	Qprt	NP_598447.1	543	9	17.47
Apod	NP_001288282.1	519	3	6.09	Spn	XP_011240018.1	668	1	1.58
Bdh1	NP_780386.3	467	1	2.26	Cd2bp2	NP_001344746.1	577	0	0.00
Dlg1	XP_006521824.1	1219	4	3.46	Tbc1d10b	NP_653105.3	437	2	4.82
Meltf	XP_006522295.1	454	3	6.96	Mylpf	NP_058034.1	1287	0	0.00
Pigz	NP_766410.2	168	6	37.64	Septin1	NP_059489.2	499	0	0.00
Ncbp2	NP_080830.1	1467	3	2.16	Zfp553	NP_001347841.1	248	0	0.00
Senp5	XP_006522303.1	417	4	10.11	Zfp771	NP_796336.1	208	0	0.00
Pak2	NP_796300.1	1253	1	0.84	Dctpp1	NP_075692.1	523	0	0.00
Pigx	NP_001104495.1	54	1	19.52	Sephs2	NP_033292.2	322	1	3.27
Cep19	NP_080168.1	104	6	60.80	Itgal	NP_001240801.1	1104	5	4.77
Nrros	NP_001334111.1	758	7	9.73	Zfp768	NP_666314.1	264	1	3.99
Bex6	NP_001028711.1	35	1	30.11	Zfp747	NP_780769.1	149	0	0.00
Fbxo45	NP_775615.2	305	8	27.64	9130019O22R	NP_084502.2	163	1	6.47
Wdr53	NP_001172091.1	103	10	102.32	E430018J23Ri	XP_006507226.1	162	1	6.51
Smco1	NP_899106.2	26	11	445.88	Zfp764	NP_666315.3	146	0	0.00
Rnf168	NP_081631.2	556	1	1.90	Zfp688	XP_006508242.1	157	0	0.00
Ubxn7	NP_808301.3	2575	6	2.46	Zfp689	NP_780372.2	203	1	5.19
Tm4sf19	XP_006522280.1	125	10	84.31	Prr14	XP_030098393.1	102	1	10.33
Gm20056	XP_036016102.1	1251	0	0.00	Fbrs	NP_034313.2	114	1	9.24
Tctex1d2	NP_079605.1	214	2	9.85	Srcap	NP_001291195.1	1486	4	2.84
Pcyt1a	NP_001156631.1	674	11	17.20	Phkg2	NP_081164.2	400	0	0.00
Slc51a	NP_666044.1	446	11	25.99	Ccdc189	NP_001369335.1	44	0	0.00
Zdhhc19	NP_955013.1	137	12	92.31	Rnf40	XP_006507812.1	990	5	5.32
Tfrc	NP_035768.1	1876	7	3.93	Zfp629	XP_006508015.1	339	2	6.22
Tnk2	XP_006522398.1	728	10	14.48	Bcl7c	NP_001334581.1	261	2	8.08
Muc4	NP_536705.4	431	9	22.01	Ctf1	XP_030097931.1	295	2	7.15
Muc20	NP_001139346.1	249	9	38.09	Ctf2	NP_942155.1	93	1	11.33
Smbd1	NP_001243238.1	0	0	#DIV/0!	Fbxl19	XP_036008913.1	2470	8	3.41
Rubcn	NP_766203.1	447	8	18.86	Orai3	NP_940816.1	271	0	0.00
Fyttd1	NP_081502.2	459	2	4.59	Setd1a	NP_821172.2	1713	6	3.69
Lrch3	NP_001074724.1	553	8	15.25	Hsd3b7	XP_036008429.1	549	0	0.00
Iqcg	NP_848465.1	224	0	0.00	Stx1b	NP_077725.1	1379	4	3.06
Rpl35a	NP_067313.2	1002	2	2.10	Stx4a	XP_006507602.1	743	2	2.84
Lmln	NP_766411.1	157	6	40.28	Zfp668	XP_017177743.1	228	5	23.11
Osbpl11	NP_789810.2	355	1	2.97	Zfp646	XP_017177704.1	182	2	11.58
Snx4	NP_542124.1	658	1	1.60	Prss53	NP_001074737.1	212	5	24.86
Zfp148	XP_006522161.1	675	3	4.68	Vkorc1	NP_848715.1	188	2	11.21
Slc12a8	NP_599012.2	360	5	14.64	Bckdk	NP_033869.1	847	5	6.22

Heg1	XP_006522778.1	202	3	15.65	Kat8	NP_080646.1	1009	3	3.13
Muc13	NP_001369763.1	326	4	12.93	Prss8	NP_579929.1	466	3	6.78
Itgb5	NP_034710.2	921	4	4.58	Prss36	NP_001306076.1	121	1	8.71
Umps	NP_033497.1	1692	4	2.49	Fus	NP_631888.1	1073	0	0.00
Kalrn	XP_011244257.1	806	1	1.31	Pycard	NP_075747.3	728	2	2.90
Ropn1	NP_109669.1	466	0	0.00	Trim72	NP_001073401.1	640	1	1.65
Ccdc14	NP_766412.2	193	1	5.46	Itgam	NP_001076429.1	2056	3	1.54
Mylk	NP_647461.3	1047	7	7.05	Itgax	NP_067309.1	1622	4	2.60
Hacd2	NP_076076.2	423	5	12.46	Itgad	XP_036009093.1	347	2	6.07
Adcy5	NP_001012783.3	1489	3	2.12	Cox6a2	NP_034073.2	618	1	1.71
Sec22a	XP_006522298.1	682	4	6.18	9130023H24F	NP_795975.1	198	0	0.00
Pdia5	NP_082571.1	1038	2	2.03	Armc5	NP_666317.2	155	0	0.00
Sema5b	XP_006521932.1	488	1	2.16	Tgfb1i1	XP_036008777.1	1060	1	0.99
Slc49a4	NP_705778.1	274	2	7.69	Slc5a2	NP_573517.1	475	1	2.22
Hspbap1	XP_006522534.1	491	3	6.44	Rusf1	NP_663565.2	54	1	19.52
Parp14	NP_001034619.2	644	3	4.91	Rgs10	XP_006508202.1	509	1	2.07
Dtx3l	NP_001013389.2	534	2	3.95	Tial1	NP_001334569.1	1296	1	0.81
Parp9	XP_030105196.1	541	2	3.90	Bag3	NP_038891.4	1467	4	2.87
Kpna1	NP_032491.2	1102	0	0.00	Inpp5f	NP_848756.2	396	3	7.98
Wdr5b	NP_081389.1	805	1	1.31	Mcmbp	NP_666067.1	469	2	4.49
Fam162a	NP_081618.1	258	9	36.76	Sec23ip	NP_001025153.2	485	0	0.00
Ccdc58	NP_941047.1	356	9	26.64	Plpp4	NP_001074432.1	352	1	2.99
Csta1	NP_001028411.1	194	10	54.32	Wdr11	NP_758459.2	230	3	13.75
Stfa2l1	NP_776294.1	137	9	69.23	Fgfr2	NP_001334567.1	1714	5	3.07
Cstdc4	NP_001076016.1	64	9	148.20	Ate1	NP_001258272.1	326	4	12.93
Gm10913	XP_036016113.1	742	0	0.00	Nsmce4a	NP_001156327.1	250	2	8.43
Csta3	NP_001076011.1	56	11	207.02	Tacc2	XP_006508127.1	322	4	13.09
Csta2	NP_084009.1	56	11	207.02	Btbd16	NP_001074507.2	50	3	63.23
Stfa1	NP_001076012.1	84	9	112.92	Plekha1	XP_030097795.1	258	3	12.25
Cstdc3	XP_156070.1	68	6	92.99	Htra1	NP_062510.2	882	2	2.39
Cstdc6	NP_001001332.2	52	9	182.41	Dmbt1	XP_006507361.2	190	2	11.09
Cstdc5	NP_001076015.1	86	8	98.04	4933402N03F	NP_775585.1	18	0	0.00
Stfa2	NP_001076014.1	95	6	66.56	Cdcp3	NP_001357754.1	149	1	7.07
Stfa3	NP_079564.1	75	6	84.31	Cuzd1	NP_032437.3	151	1	6.98
Casr	NP_038831.2	879	0	0.00	Fam24b	XP_006508244.2	4	0	0.00
Cd86	NP_062261.3	1286	2	1.64	Fam24a	NP_899095.1	38	0	0.00
Ildr1	NP_001272717.1	299	0	0.00	2310057M21I	NP_080931.1	26	3	121.60
Slc15a2	NP_067276.3	541	0	0.00	Pstk	NP_001351090.1	174	1	6.06
Eaf2	NP_001106872.1	342	0	0.00	Ikzf5	XP_036009269.1	371	1	2.84
Iqcb1	XP_030105013.1	1137	1	0.93	Acadsb	NP_080102.1	663	5	7.95
Golgb1	XP_017172452.1	620	1	1.70	Hmx3	NP_032283.3	448	5	11.76
Hcls1	NP_032251.2	896	1	1.18	Hmx2	NP_001355289.1	344	5	15.32
Fbxo40	NP_001345146.1	583	2	3.62	Bub3	NP_033904.2	1348	6	4.69
Polq	NP_084253.1	613	2	3.44	Gpr26	NP_775586.1	646	9	14.68
Stxbp5l	NP_001108083.1	1138	4	3.70	Cpxm2	XP_006508097.1	241	10	43.73
Gtf2e1	NP_001342656.1	643	0	0.00	Chst15	NP_001347697.1	298	5	17.68
Rabl3	NP_001035964.1	208	0	0.00	Oat	NP_058674.1	994	4	4.24
Hgd	XP_030104838.1	1025	3	3.08	Nkx1-2	NP_033149.1	261	5	20.19
Ndufb4	NP_080886.1	468	1	2.25	Lhpp	NP_083885.1	351	4	12.01
Fstl1	NP_032073.2	708	0	0.00	Fam53b	NP_001368787.1	171	0	0.00
Lrrc58	NP_796067.2	524	2	4.02	Eef1akmt2	NP_082371.1	193	2	10.92
Gpr156	NP_700443.2	157	1	6.71	Abraxas2	NP_932134.3	123	6	51.41
Gsk3b	NP_001334161.1	4136	3	0.76	Zranb1	NP_001349128.1	455	2	4.63
Nr1i2	NP_001091874.1	1219	4	3.46	Ctbp2	XP_030097924.1	1176	2	1.79
Maats1	NP_001074494.1	189	1	5.58	Tex36	NP_082930.1	128	7	57.64
Cox17	XP_006521813.1	564	1	1.87	Edrf1	NP_835216.3	49	0	0.00
Popdc2	XP_030105086.1	273	0	0.00	Mmp21	NP_694423.1	146	1	7.22
Pla1a	XP_036016082.1	248	3	12.75	Uros	XP_030098239.1	389	4	10.84
Adprh	NP_031440.1	162	0	0.00	Bccip	NP_079668.2	1119	3	2.83
Cd80	XP_036015651.1	1124	0	0.00	Dhx32	NP_001344614.1	1073	8	7.86
Timmdc1	NP_077235.2	264	2	7.98	Gm15483	XP_036009550.1	1646	0	0.00
Poglut1	NP_759012.1	188	3	16.82	Fank1	NP_080126.1	1163	9	8.16
Tmem39a	NP_080683.2	57	1	18.49	Adam12	NP_031426.2	622	8	13.55
Arhgap31	NP_064656.2	298	1	3.54	D7Ertd443e	XP_011240205.1	90	1	11.71
B4galt4	XP_011244279.1	272	0	0.00	Dock1	NP_001028592.1	842	7	8.76
Upk1b	XP_017172432.1	320	0	0.00	Insyn2a	XP_006508166.1	63	2	33.46

Tex55	XP_006522739.1	201	0	0.00	Nps	NP_001157083.1	1206	1	0.87
Igsf11	NP_733548.2	291	0	0.00	Foxi2	NP_899016.2	537	4	7.85
Lsamp	XP_030104997.1	962	4	4.38	Clrn3	NP_848784.1	140	0	0.00
Gap43	NP_032109.1	1501	3	2.11	Ptpre	NP_001303607.1	749	5	7.04
Zbtb20	XP_036015933.1	661	5	7.97	Mki67	XP_006507476.1	2274	3	1.39
Tigit	NP_001139797.1	240	1	4.39	Mgmt	XP_006507469.1	893	6	7.08
Drd3	XP_006521840.1	742	3	4.26	Ebf3	XP_030097955.1	614	4	6.87
Qtrt2	NP_083404.2	471	0	0.00	9430038I01Ri	XP_036009451.1	0	0	#DIV/0!
Ccdc191	XP_006522021.1	4	0	0.00	Glrx3	NP_075629.2	833	6	7.59
Zdhhc23	XP_030105026.1	263	7	28.05	Tcerg1l	NP_899112.2	805	6	7.86
Gramd1c	XP_006521957.1	107	6	59.10	Mapk1ip1	NP_001038948.1	162	0	0.00
Atp6v1a	NP_001345132.1	1344	7	5.49	Ppp2r2d	NP_080667.1	1131	10	9.32
Naa50	NP_001334168.1	974	6	6.49	Bnip3	NP_033890.1	736	6	8.59
Usf3	XP_006521961.1	159	0	0.00	Jakmip3	XP_036009374.1	452	6	13.99
Sidt1	XP_017172523.1	247	6	25.60	Dpysl4	NP_036123.3	508	9	18.67
Spice1	XP_017172412.1	301	6	21.01	Stk32c	NP_067277.2	1337	4	3.15
Cfap44	NP_001028419.1	619	0	0.00	Lrrc27	XP_006536325.1	363	0	0.00
Boc	NP_766094.1	492	0	0.00	Pwwp2b	NP_001028378.1	44	0	0.00
Nepro	NP_666084.1	71	0	0.00	Inpp5a	NP_898967.2	297	8	28.39
Gtpbp8	NP_001152801.1	253	6	24.99	Nkx6-2	XP_036008587.1	536	0	0.00
Cd200r1	NP_067300.1	389	3	8.13	Cfap46	NP_001289475.1	82	1	12.85
Cd200r4	NP_997127.1	118	3	26.79	Adgra1	XP_006536266.1	670	9	14.16
Cd200r2	NP_996258.1	47	1	22.42	Kndc1	NP_796235.4	333	7	22.15
Cd200r3	XP_011244333.1	105	2	20.07	Utf1	NP_033508.1	490	2	4.30
Ccdc80	NP_080715.2	389	0	0.00	Spef1l	NP_001028637.1	8	0	0.00
Slc35a5	XP_006522711.1	314	1	3.36	Adam8	NP_001277995.1	733	2	2.88
Atg3	NP_080678.1	631	0	0.00	Tubgcp2	NP_598516.2	779	3	4.06
Btla	NP_001032808.2	201	2	10.49	Zfp511	NP_081477.1	55	4	76.65
Cd200	NP_034948.3	546	6	11.58	Msx3	NP_034966.1	287	1	3.67
Gm609	NP_001005854.2	13	3	243.21	Caly	XP_006536298.1	939	3	3.37
Slc9c1	NP_932774.3	590	1	1.79	Prap1	NP_033501.2	122	2	17.28
Gcsam	NP_001152769.1	180	1	5.86	Fuom	NP_001273146.1	56	2	37.64
BC016579	NP_663364.2	44	0	0.00	Echs1	NP_444349.1	1151	4	3.66
Tmprss7	XP_017172397.1	158	0	0.00	Paox	XP_011248143.3	559	3	5.66
Tagln3	NP_062728.1	1299	1	0.81	Mtg1	NP_955005.2	828	4	5.09
Abhd10	NP_001258999.1	291	0	0.00	Sprn	NP_898970.1	626	8	13.47
Phldb2	XP_030104880.1	260	1	4.05	Olfr522	NP_667163.1	33	2	63.87
Plcxd2	NP_001127952.1	161	1	6.55	Olfr523	NP_666729.1	77	2	27.37
Cd96	NP_115854.2	313	3	10.10	Olfr524	XP_006536253.1	60	2	35.13
Gm4737	NP_001291457.1	1255	1	0.84	Cd163l1	XP_017177746.1	148	5	35.60
Nectin3	NP_067471.1	472	1	2.23	5830411N06F	NP_001121618.1	135	1	7.81
Dppa4	NP_082886.2	498	1	2.12	Gm6314	XP_036009541.1	277	0	0.00
Dppa2	NP_082891.1	357	1	2.95	Olfr525	NP_667167.1	102	0	0.00
Morc1	NP_034946.1	303	7	24.35	Olfr527	NP_001011776.1	42	0	0.00
Trat1	XP_006522793.1	588	6	10.75	Olfr60	NP_667166.1	35	0	0.00
Retnlb	NP_076370.3	175	0	0.00	Olfr530	NP_666730.1	26	0	0.00
Retnla	NP_065255.3	341	1	3.09	Olfr531	NP_667164.1	41	0	0.00
Retnlg	NP_853627.2	135	0	0.00	Olfr532	NP_667237.1	151	0	0.00
Dzip3	NP_081617.1	736	6	8.59	Olfr533	NP_001011815.1	113	0	0.00
Cip2a	NP_766204.2	503	5	10.48	Olfr535	NP_667165.1	70	0	0.00
Myh15	NP_001159682.1	1000	6	6.32	Olfr536	NP_666731.1	45	0	0.00
Ift57	NP_082956.2	507	6	12.47	Olfr538	NP_001011867.1	42	0	0.00
Cd47	NP_034711.1	847	3	3.73	Olfr46	NP_667145.1	54	0	0.00
Bbx	XP_036016019.1	286	3	11.05	Olfr61	NP_667175.1	19	0	0.00
Ccdc54	NP_081322.1	308	1	3.42	Olfr53	NP_667171.1	23	0	0.00
Cblb	XP_006522010.1	955	1	1.10	Olfr539	NP_667172.1	44	0	0.00
Alcam	NP_001318039.1	686	1	1.54	Olfr45	NP_667174.1	38	1	27.73
Zpld1	NP_848835.1	155	3	20.40	Olfr541	NP_667173.1	15	0	0.00
Nfkbiz	NP_085115.1	1504	2	1.40	Cyp2e1	NP_067257.1	1010	2	2.09
Nxpe3	XP_030105027.1	62	0	0.00	Syce1	NP_001137237.1	237	1	4.45
Cep97	NP_083091.1	862	6	7.34	Zfp941	NP_001001180.2	244	0	0.00
Rpl24	NP_077180.1	959	0	0.00	Urah	XP_030098952.1	580	0	0.00
Zbtb11	NP_766614.2	342	0	0.00	Scgb1c1	NP_001093212.1	116	0	0.00
Pcnp	XP_036016068.1	219	2	9.62	Odf3	NP_081295.2	198	3	15.97
Trmt10c	NP_083368.1	745	1	1.41	Bet1l	NP_061212.3	490	4	8.60
Senp7	XP_036015979.1	569	2	3.70	Ric8a	NP_444424.1	424	5	12.43

Impg2	NP_777365.2	255	3	12.40	Sirt3	XP_006536285.1	1195	5	4.41
Abi3bp	XP_036015888.1	203	0	0.00	Psmd13	NP_036005.1	950	4	4.44
Tfg	NP_001239372.1	402	1	2.62	Cox8b	NP_031777.1	465	0	0.00
Adgrg7	NP_766413.2	360	3	8.78	Nlrp6	NP_598707.2	516	3	6.13
Tmem45a	XP_030105056.1	300	0	0.00	Pgghg	XP_017177594.1	200	8	42.16
Tmem45a2	NP_001345030.1	73	0	0.00	Ifitm5	NP_444318.1	241	3	13.12
Tomm70a	NP_613065.2	893	1	1.18	Ifitm2	NP_109619.1	333	4	12.66
Nit2	NP_075664.1	619	1	1.70	Ifitm1	NP_001347656.1	312	4	13.51
Tbc1d23	NP_080530.2	357	0	0.00	Ifitm3	NP_079654.1	667	6	9.48
Tmem30c	XP_030105143.1	366	2	5.76	Ifitm6	NP_001028804.1	285	7	25.89
Cmss1	NP_079875.2	551	3	5.74	B4galnt4	NP_808565.2	164	7	44.98
Filip1l	NP_001035487.2	293	2	7.19	Pkp3	NP_001156396.1	563	4	7.49
Col8a1	NP_031765.2	577	4	7.31	Sigirr	NP_075546.2	280	6	22.58
Dcbld2	NP_082799.2	273	1	3.86	Ano9	NP_848468.2	155	6	40.80
St3gal6	XP_036015919.1	331	1	3.18	Ptdss2	NP_038810.2	270	3	11.71
Ftdc1	NP_001028576.1	131	1	8.05	Rnh1	NP_001165572.1	431	2	4.89
Ftdc2	NP_778176.1	151	1	6.98	Hras	NP_001123915.1	4684	7	1.58
Cpox	NP_031783.2	849	1	1.24	Lrrc56	XP_006536303.1	657	4	6.42
Gpr15	NP_001156427.1	329	3	9.61	Lmntd2	NP_082326.1	222	1	4.75
Cldnd1	NP_001239379.1	108	1	9.76	Rassf7	NP_080162.3	275	9	34.49
Olfr172	NP_667212.2	24	0	0.00	Phrf1	XP_006536200.1	398	6	15.89
Olfr173	NP_667211.2	32	0	0.00	Irf7	XP_006536277.1	1075	3	2.94
Olfr175	NP_667213.2	104	0	0.00	Cdhr5	NP_082345.1	696	5	7.57
Olfr177	XP_030104978.1	115	0	0.00	Sct	NP_035458.1	471	3	6.71
Olfr178	NP_667208.2	96	0	0.00	Drd4	NP_031904.1	744	5	7.08
Olfr180	NP_001011662.2	90	0	0.00	Deaf1	XP_006536269.1	395	6	16.01
Olfr181	NP_667210.2	98	0	0.00	Tmem80	NP_001360949.1	136	2	15.50
Olfr183	XP_017172474.1	23	0	0.00	Eps8l2	NP_573454.2	468	5	11.26
Olfr186	NP_666433.1	23	0	0.00	Taldo1	NP_035658.1	1233	0	0.00
Olfr187	NP_666434.2	104	0	0.00	Gatd1	XP_006536234.2	77	6	82.12
Olfr190	NP_666509.2	74	0	0.00	Cend1	NP_067291.1	961	0	0.00
Olfr191	NP_001011807.2	157	0	0.00	Slc25a22	NP_001347652.1	423	1	2.49
Olfr193	NP_001011791.1	25	0	0.00	Pidd1	NP_073145.1	2482	0	0.00
Olfr194	NP_001005524.2	52	0	0.00	Rplp2	NP_001347586.1	1090	1	0.97
Olfr195	NP_667209.1	94	0	0.00	Pnpla2	NP_001157161.1	836	1	1.26
Olfr196	XP_017172475.1	98	0	0.00	Cracr2b	XP_036008773.1	105	2	20.07
Olfr198	NP_001011808.1	74	0	0.00	Cd151	NP_033972.2	494	1	2.13
Olfr199	NP_997433.2	13	0	0.00	Polr2l	NP_079869.1	1100	1	0.96
Olfr201	NP_667205.2	64	0	0.00	Tspan4	XP_006536287.1	374	2	5.64
Olfr202	NP_667206.1	33	0	0.00	Chid1	NP_080798.3	91	3	34.74
Olfr203	NP_666697.2	61	0	0.00	Ap2a2	NP_001343997.1	1142	3	2.77
Olfr204	NP_667203.2	42	0	0.00	Muc6	NP_001355882.2	409	5	12.88
Olfr205	NP_001011736.1	20	0	0.00	Muc2	NP_076055.4	796	4	5.30
Olfr206	NP_667202.1	12	0	0.00	Muc5ac	XP_006508562.1	692	3	4.57
Olfr209	NP_997434.2	10	0	0.00	Muc5b	NP_083077.2	633	4	6.66
Gabrr3	NP_001074659.1	494	1	2.13	Tollip	NP_076253.1	647	2	3.26
Riox2	NP_080186.3	218	2	9.67	Brsk2	NP_001263692.1	1534	2	1.37
Crybg3	NP_777273.3	56	1	18.82	Mob2	NP_082584.1	365	5	14.44
Arl6	NP_001334173.1	1443	3	2.19	Dusp8	XP_006508572.1	952	6	6.64
Epha6	NP_031964.2	1618	3	1.95	Gm4553	NP_001365612.1	80	5	65.87
Nsun3	XP_006521734.1	902	0	0.00	Krtap5-2	NP_082120.3	156	5	33.78
Arl13b	XP_006522568.1	1817	4	2.32	Krtap5-3	NP_076349.1	84	0	0.00
Stx19	NP_080864.1	478	2	4.41	Krtap5-5	NP_001032911.1	88	2	23.95
Pros1	NP_035303.1	438	1	2.41	Gm4559	NP_001186238.1	118	5	44.66
Epha3	NP_001349381.1	1516	4	2.78	Krtap5-1	NP_056623.1	118	2	17.86
Csnka2ip	NP_776286.2	247	1	4.27	Krtap5-4	NP_056624.1	136	2	15.50
4930453N24R	XP_006522552.1	152	4	27.73	Ifitm10	XP_011240304.1	128	4	32.93
Zfp654	NP_001334174.1	301	6	21.01	Ctsd	NP_034113.1	1727	5	3.05
Cggbp1	NP_001344345.1	260	2	8.11	Syt8	XP_036009154.1	393	4	10.73
Htr1f	NP_032336.1	443	2	4.76	Tnni2	NP_033431.1	637	3	4.96
Pou1f1	NP_032875.1	507	3	6.24	Lsp1	NP_001129543.1	528	2	3.99
Chmp2b	NP_081155.1	449	4	9.39	Tnnt3	NP_001157137.1	676	4	6.24
Vgll3	NP_001355689.1	382	4	11.04	Mrpl23	XP_017177539.1	713	8	11.82
Cadm2	NP_001139449.1	1050	5	5.02	Igf2	NP_034644.2	1598	14	9.23
Speer2	NP_775092.3	66	1	15.97	Ins2	NP_001172012.1	2243	13	6.11
Gbe1	NP_083079.1	848	4	4.97	Th	NP_033403.1	1342	5	3.93

Robo1	XP_006523023.1	974	3	3.25	Ascl2	NP_032580.2	476	11	24.35
Robo2	NP_001345419.1	1212	2	1.74	Tspan32	XP_006508682.1	188	11	61.66
Lipi	NP_001239442.1	231	6	27.37	Cd81	NP_598416.1	768	11	15.09
Rbm11	NP_938044.1	347	6	18.22	Tssc4	XP_017177874.1	358	13	38.27
Hspa13	NP_084477.1	1966	13	6.97	Trpm5	NP_064673.2	403	9	23.54
Samsn1	NP_075869.2	628	11	18.46	Kcnq1	XP_006508555.1	1236	15	12.79
Nrip1	XP_017172509.1	634	7	11.64	Cdkn1c	XP_030097904.1	992	14	14.87
Usp25	XP_006523118.1	1191	11	9.73	Slc22a18	XP_006508573.1	886	15	17.84
Cxadr	XP_006522947.1	364	7	20.27	Phlda2	XP_006508660.1	260	16	64.86
Btg3	NP_001284676.1	447	7	16.50	Nap1l4	XP_030098063.1	1164	14	12.68
D16Ertd472e	NP_080243.3	147	9	64.52	Cars	NP_001239522.1	1718	9	5.52
Chodl	NP_624360.2	383	7	19.26	Tnfrsf26	NP_783580.1	209	0	0.00
Tmprss15	NP_849186.2	381	7	19.36	Tnfrsf22	NP_001298074.1	288	3	10.98
Ncam2	NP_001106679.1	1041	3	3.04	Tnfrsf23	NP_077252.2	297	4	14.19
Mrpl39	NP_059100.3	1079	4	3.91	Osbpl5	NP_001371092.1	454	13	30.18
Jam2	NP_076333.3	239	2	8.82	Mrgprg	NP_987077.1	56	7	131.74
Atp5j	NP_001345428.1	931	7	7.92	Mrgpre	XP_036009019.1	166	2	12.70
Gabpa	XP_006522968.1	796	7	9.27	Nadsyn1	NP_084497.1	432	2	4.88
App	XP_006522936.1	2949	3	1.07	Dhcr7	XP_030097935.1	734	4	5.74
Cyyr1	NP_659102.1	122	2	17.28	Acte1	XP_006508732.1	1208	0	0.00
Adamts1	NP_033751.3	805	4	5.24	Shank2	XP_006508582.1	2055	11	5.64
Adamts5	NP_035912.2	594	1	1.77	Cttn	NP_001344045.1	1120	10	9.41
N6amt1	NP_080642.1	515	4	8.19	Ppfia1	XP_006508674.1	603	11	19.23
Ltn1	NP_001074537.1	840	4	5.02	Fadd	NP_034305.1	765	9	13.05
Rwdd2b	NP_058620.2	186	2	11.33	Ano1	XP_006508526.1	529	13	28.78
Usp16	NP_077220.2	655	3	4.83	Fgf3	NP_032033.2	849	10	14.60
Cct8	NP_033970.3	1537	3	2.06	Fgf4	NP_034332.2	1099	9	10.79
Map3k7cl	NP_659103.1	86	1	12.25	Fgf15	NP_032029.1	882	10	15.93
Bach1	XP_006522942.1	270	0	0.00	LTO1	NP_001334466.1	202	11	81.99
Grik1	XP_017172369.1	854	1	1.23	Ccnd1	NP_031657.1	2824	9	5.17
Cldn17	NP_852467.1	221	1	4.77	Tpcn2	XP_036008920.1	472	9	33.49
Cldn8	NP_061248.1	368	1	2.86	Mrgprf	NP_663354.1	237	6	48.51
Krtap24-1	NP_001156613.1	203	4	20.77	Mrgprd	NP_987075.1	206	2	20.46
2310079G19R	NP_081449.1	25	6	252.94	Insr	XP_006508764.1	1982	0	0.00
Krtap26-1	NP_081381.1	209	4	20.17	Arhgef18	XP_030099070.1	557	1	3.44
Krtap27-1	NP_001156577.1	222	6	28.48	Pex11g	NP_082870.1	350	1	5.02
2310061N02R	NP_081431.1	94	10	112.12	Tex45	XP_006508984.1	12	0	0.00
Krtap13-1	NP_899012.1	182	2	11.58	Zfp358	NP_536709.2	177	1	8.51
Krtap13	NP_034801.1	12	4	351.30	Mcoln1	NP_444407.1	461	1	3.05
2310034C09R	NP_473441.1	10	2	210.78	Pnpla6	XP_036010050.1	951	2	2.77
2310057N15R	NP_081446.1	128	7	57.64	Rps23rg1	NP_001019899.1	44	0	0.00
Gm5965	XP_030105222.1	11	3	287.43	Camsap3	NP_001350168.1	304	3	11.56
Krtap14	NP_038735.2	184	13	74.46	Xab2	NP_080432.1	1032	11	11.82
Krtap15	NP_038741.1	180	7	40.99	Pet100	NP_001182173.1	235	8	35.88
Krtap19-1	NP_570946.2	90	6	70.26	Pcp2	NP_001123275.1	513	8	16.44
Krtap19-2	NP_570940.1	153	8	55.11	Stxbp2	XP_006508806.3	885	13	15.48
Krtap19-3	NP_570927.1	96	8	87.83	Retn	NP_075360.1	821	9	11.55
Krtap19-4	XP_036015699.1	96	7	76.85	Mcemp1	NP_081261.1	387	8	21.79
Krtap19-5	NP_034806.2	168	9	56.46	Trappc5	NP_079977.3	449	15	35.21
Krtap19-9b	NP_579937.1	104	14	141.87	Fcer2a	NP_001240676.1	465	8	18.13
Krtap16-3	NP_899119.1	119	16	141.70	Clec4g	NP_083741.1	751	10	14.03
Krtap22-2	NP_001177947.1	92	11	126.01	Cd209a	NP_573501.1	741	6	8.53
Gm10229	NP_001186263.1	101	20	208.69	Cd209e	NP_570975.1	407	5	12.95
Krtap6-1	NP_034802.1	97	12	130.38	Cd209d	NP_570974.1	407	6	15.54
Gm10228	NP_001257416.1	100	18	189.70	Cd209b	NP_001032889.3	486	7	15.18
Krtap6-5	NP_570926.1	131	16	128.72	Cd209c	XP_017168079.1	321	1	3.28
1110025L11Ri	NP_001263207.1	100	17	179.16	Cd209f	XP_006508930.1	242	4	17.42
Krtap6-3	NP_034804.1	108	14	136.62	Cd209g	XP_006508948.1	190	3	16.64
Gm38490	XP_006523181.1	60	15	263.48	Evi5l	NP_001355721.1	189	0	0.00
Gm33726	XP_006523182.1	62	17	288.97	Prr36	XP_017168469.1	52	4	81.07
Gm33798	XP_006523183.1	62	17	288.97	Lrrc8e	NP_082451.2	464	1	2.27
Gm33888	XP_006523184.1	60	14	245.91	Map2k7	NP_001036022.1	1048	0	0.00
Gm6358	XP_006523185.1	62	16	271.97	Tgfbr3l	XP_036009587.1	18	0	0.00
Gm10061	XP_006523186.1	6	6	1053.90	Snapc2	NP_598729.1	127	1	8.30
Gm9789	NP_001344928.1	72	13	190.29	Ctxn1	NP_899138.1	273	0	0.00
Gm7735	XP_006523187.1	41	0	0.00	Timm44	NP_035722.2	743	1	1.42

Gm34396	XP_006523188.1	60	14	245.91	Elavl1	XP_030099161.1	1416	0	0.00
Krtap20-2	NP_001157087.1	54	8	156.13	Ccl25	XP_006508799.1	547	2	3.85
Gm34733	XP_006523192.1	72	12	175.65	Cers4	NP_080334.3	447	1	2.36
Gm34826	XP_006523193.1	4	4	1053.90	Zfp958	NP_663566.1	179	1	5.89
Gm35004	XP_006523195.1	72	10	146.38	Shcbp1	NP_035499.1	535	0	0.00
Gm35174	XP_006523197.1	72	9	131.74	Slc10a2	NP_035518.1	455	1	2.32
Gm35342	XP_006523199.1	62	13	220.98	Efnb2	NP_001355228.1	801	3	3.95
Krtap21-1	NP_082897.2	119	6	53.14	Arglu1	XP_006508823.1	270	4	15.61
Krtap6-2	NP_034803.2	103	2	20.46	Fam155a	NP_775622.1	580	6	10.90
Krtap8-1	NP_034805.1	268	5	19.66	Lig4	NP_001363971.1	1327	7	5.56
Krtap7-1	NP_082047.1	203	5	25.96	Abhd13	NP_001074588.1	174	2	12.11
Krtap11-1	NP_001106877.1	228	4	18.49	Tnfsf13b	NP_296371.1	831	1	1.27
Tiam1	XP_017172425.1	954	6	6.63	Myo16	XP_006508841.1	3167	6	2.00
Sod1	NP_035564.1	2383	5	2.21	Irs2	NP_001074681.1	937	3	3.37
Scaf4	XP_006523077.1	488	3	6.48	Col4a1	NP_034061.2	943	2	2.24
Hunk	NP_056570.1	1214	8	6.94	Col4a2	NP_034062.3	899	7	8.21
Mis18a	NP_079918.1	348	5	15.14	Rab20	NP_035357.1	932	6	6.78
Mrap	NP_084120.1	838	5	6.29	Naxd	NP_081271.2	359	0	0.00
Urb1	XP_006523040.1	452	5	11.66	Cars2	XP_030099635.1	789	4	5.34
Eva1c	NP_001186139.1	384	4	10.98	Ing1	NP_036049.2	833	4	5.06
Cfap298	NP_080778.2	224	5	23.52	Ankrd10	NP_598732.2	1072	8	7.86
Synj1	XP_030104764.1	1123	13	12.20	1700016D06F	NP_077233.1	0	0	#DIV/0!
Paxbp1	NP_080386.3	511	12	24.75	Arhgef7	XP_036010059.1	765	5	6.89
4932438H23R	XP_030105174.1	32	7	230.54	Tex29	XP_006508983.1	51	0	0.00
Olig2	XP_036015893.1	914	9	10.38	A230072I06R	XP_030099756.1	0	0	#DIV/0!
Olig1	NP_058664.2	763	12	16.58	Sox1	NP_033259.2	751	0	0.00
Ifnar2	NP_034639.2	267	10	39.47	Spaca7	NP_077241.1	36	0	0.00
Il10rb	NP_032375.2	521	11	22.25	Tubgcp3	NP_932148.1	689	5	7.65
Ifnar1	NP_034638.2	782	11	14.82	Atp11a	NP_056619.1	1010	5	5.22
Ifngr2	NP_032364.1	440	16	38.32	Mcf2l	NP_001357972.1	538	3	5.88
Tmem50b	XP_036016074.1	164	16	102.82	F7	NP_034302.2	714	2	2.95
Dnajc28	NP_001093208.1	166	1	6.35	F10	NP_001229297.1	685	3	4.62
Gart	NP_001344280.1	2749	15	5.75	Proz	NP_080110.1	658	2	3.20
Son	NP_849211.3	609	8	13.84	Pcid2	NP_848823.2	472	2	4.47
Donson	NP_068366.1	412	17	43.49	Cul4a	NP_666319.2	1626	7	4.54
Cryzl1	NP_081270.1	442	14	33.38	Lamp1	NP_001304282.1	1710	4	2.47
Itsn1	XP_006522994.1	1469	14	10.04	Grtp1	NP_080044.2	815	9	11.64
Atp5o	NP_613063.1	1269	10	8.30	Adprhl1	XP_036009777.1	371	5	14.20
Mrps6	NP_536704.1	842	12	15.02	Dcun1d2	NP_001036115.1	401	8	21.03
Slc5a3	NP_059087.2	368	11	31.50	Tmco3	NP_758486.1	391	3	8.09
Kcne2	NP_001345301.1	336	5	15.68	Tfdp1	XP_017168146.1	941	5	5.60
Smim11	XP_030105120.1	126	9	75.28	Atp4b	NP_033854.1	418	0	0.00
Fam243	NP_083528.1	126	0	0.00	Grk1	NP_036011.3	633	0	0.00
Kcne1	NP_001349385.1	478	5	11.02	Tmem255b	XP_006508878.1	76	4	55.47
Rcan1	NP_001075018.1	629	15	25.13	Gas6	NP_062394.2	681	7	10.83
Clic6	NP_766057.1	678	8	12.44	1700029H14F	NP_079877.1	26	0	0.00
Runx1	NP_001104492.1	939	3	3.37	Rasa3	NP_033051.2	548	5	9.62
Setd4	NP_663457.2	414	9	22.91	Cfap97d2	XP_006508884.1	7	0	0.00
Cbr1	NP_031646.2	704	13	19.46	Cdc16	NP_081552.2	1372	4	3.07
Cbr3	NP_766635.1	566	12	22.34	Upf3a	NP_080200.1	525	2	4.01
Dop1b	XP_011244445.1	491	14	30.05	AF366264	NP_694733.3	242	2	8.71
Morc3	NP_001038994.2	415	9	22.86	Champ1	NP_001350384.1	172	1	6.13
Chaf1b	NP_082359.1	998	11	11.62	Coprs	NP_001345988.1	175	0	0.00
Cldn14	XP_036015927.1	614	13	22.31	Fbxo25	NP_001345990.1	302	6	20.94
Sim2	XP_006523029.1	481	14	30.67	Tdrp	XP_011240385.1	83	5	63.49
Hlcs	XP_006522915.1	632	14	23.35	Erich1	NP_001030034.2	119	6	53.14
Ripply3	NP_573492.2	177	10	59.54	Dlgap2	NP_001139437.1	1154	7	6.39
Pigp	NP_001153092.1	233	19	85.94	Cln8	XP_036009958.1	357	3	8.86
Ttc3	XP_006523047.1	872	19	22.96	Arhgef10	XP_017168158.1	316	6	20.01
Vps26c	NP_031860.1	229	16	73.63	Kbtbd11	XP_036010237.1	148	1	7.12
Dyrk1a	NP_001106860.1	1502	19	13.33	Myom2	XP_006508790.1	482	4	8.75
Kcnj6	NP_001020755.1	936	17	19.14	Csmd1	NP_444401.2	752	3	4.20
Kcnj15	XP_006523013.1	394	12	32.10	Mcph1	XP_011240359.1	1048	2	2.01
Erg	XP_006522966.1	529	1	1.99	Angpt2	NP_031452.2	847	2	2.49
Ets2	NP_035939.3	939	15	16.84	Agpat5	NP_081068.1	455	0	0.00
Psmg1	NP_062410.1	568	17	31.54	Xkr5	XP_006508881.1	58	3	54.51

Brwd1	NP_660107.2	1150	14	12.83	Defb40	NP_898860.3	47	0	0.00
Hmgn1	NP_032277.3	391	15	40.43	Defb37	NP_859011.1	36	0	0.00
Get1	NP_997184.1	386	10	27.30	Defb38	NP_898857.1	20	0	0.00
Lca5l	XP_006523124.1	77	10	136.87	Defb39	NP_898859.2	28	0	0.00
Sh3bgr	NP_056640.1	346	14	42.64	Defb12	NP_690015.1	74	7	99.69
B3galt5	NP_001345319.1	373	14	39.56	Defb34	XP_017168358.1	177	1	5.95
Igsf5	XP_006523158.1	515	5	10.23	Spag11b	NP_001273422.1	129	5	40.85
Itgb2l	NP_032431.2	937	4	4.50	Spag11a	NP_694755.1	129	5	40.85
Pcp4	NP_032817.1	735	9	13.58	Defb14	NP_898847.1	148	3	21.36
Dscam	NP_112451.1	1150	12	12.22	Defb4	NP_062702.1	163	3	19.40
Bace2	NP_062390.3	667	11	20.45	Defb6	NP_473415.1	77	0	0.00
Fam3b	NP_065647.1	256	8	41.17	Defb46	NP_001020522.1	204	0	0.00
Tmprss2	NP_056590.2	750	9	16.86	Defb5	NP_109659.2	41	0	0.00
Ripk4	NP_076152.2	3966	11	4.18	Defb3	NP_038784.1	75	0	0.00
Prdm15	XP_030104790.1	306	9	47.69	Defb8	NP_694748.3	107	3	29.55
C2cd2	XP_011244411.1	210	7	58.55	Defb7	NP_631966.1	35	0	0.00
Zbtb21	XP_030104787.1	366	9	47.12	4930467E23R	XP_017168417.1	983	0	0.00
B230307C23R	NP_001363937.1	126	1	16.73	Gm15319	NP_001170879.1	958	0	0.00
Scaf8	NP_598884.2	544	3	11.62	Gm21119	NP_001257482.1	919	0	0.00
Tiam2	XP_030105627.1	711	6	16.17	Defb33	NP_001034208.1	279	2	7.55
Tfb1m	NP_666186.1	1095	2	3.21	Gm15056	XP_011240405.1	0	0	#DIV/0!
Cldn20	NP_001095030.1	115	4	56.40	Gm6040	NP_001020524.1	44	9	215.57
Nox3	NP_945196.2	344	2	8.75	AY761185	NP_001012658.1	49	2	43.02
Arid1b	XP_006523284.2	757	3	5.57	Defa21	NP_899076.1	55	2	38.32
Tmem242	NP_081733.3	61	3	64.79	Defa23	NP_001012307.1	105	3	30.11
Ldhal6b	NP_780558.1	914	0	0.00	Defa35	NP_001170952.1	28	3	112.92
Zdhhc14	XP_036016401.1	254	4	18.44	Defa25	NP_031875.1	155	1	6.80
Snx9	NP_079940.2	652	5	8.51	Defa38	NP_001170953.1	67	3	47.19
Synj2	XP_006523266.1	893	7	8.26	Defa30	NP_001170956.1	98	3	32.26
Serac1	XP_030105707.1	293	5	17.98	Defa22	NP_997541.2	62	2	34.00
Gtf2h5	NP_001344733.1	449	10	23.47	Defa31	NP_031874.2	43	0	0.00
Tulp4	XP_030105887.1	423	4	9.97	Defa41	NP_001170957.1	29	3	109.02
Tmem181a	XP_030106001.1	251	10	41.99	Defa40	NP_001170958.1	42	2	50.19
Dynlt1a	NP_001160101.1	441	3	7.17	Defa3	NP_031876.1	203	14	72.68
Dynlt1b	NP_033368.1	441	3	7.17	Defa5	NP_031877.2	42	14	351.30
Gm2808	XP_017173259.1	423	4	9.97	Defa27	NP_001164426.1	17	2	123.99
Dynlt1c	NP_001160102.1	441	4	9.56	Defa29	NP_031870.2	75	2	28.10
Dynlt1f	NP_001186877.1	441	4	9.56	Defa2	NP_001182563.2	43	0	0.00
Sytl3	NP_113572.1	226	10	46.63	Defa33	NP_001257484.1	43	0	0.00
Ezr	NP_033536.2	1474	2	1.43	Defa36	NP_001257542.1	40	0	0.00
Rsph3b	NP_001077414.1	321	5	16.42	Defa42	NP_001170989.1	26	2	81.07
Tagap1	NP_671511.2	304	5	17.33	Defa20	NP_899091.2	12	2	175.65
Rnaset2b	NP_080887.1	465	4	9.07	Defa32	NP_001170992.1	13	2	162.14
Gm1604b	NP_001028614.1	0	0	#DIV/0!	Defa37	NP_001170993.1	34	2	61.99
Rps6ka2	XP_006523262.1	618	7	11.94	Defa28	NP_001170994.1	96	2	21.96
Tcp10a	NP_035683.2	409	4	10.31	Defa43	NP_001170997.1	28	0	0.00
Ttll2	XP_011244473.1	267	7	27.63	Defa26	NP_001073402.1	49	0	0.00
Unc93a2	XP_030105852.1	135	7	54.65	Defa17	NP_001161262.1	193	1	5.46
Fndc1	XP_036016663.1	395	4	10.67	Defa34	NP_001170999.1	29	0	0.00
Tagap	NP_666080.1	431	4	9.78	Defa39	NP_001007583.1	62	0	0.00
Rsph3a	NP_080065.4	333	3	9.49	Defa24	NP_001019396.1	122	1	8.64
Rnaset2a	NP_001351881.1	465	8	18.13	Defb1	NP_031869.1	382	9	24.83
Cep43	XP_030105984.1	394	4	10.70	Defb50	NP_951022.1	51	1	20.66
Ccr6	NP_001177267.1	887	5	5.94	Defb2	NP_034160.1	170	1	6.20
Mpc1	NP_061289.1	400	6	15.81	Defb10	NP_631971.1	24	0	0.00
Sft2d1	NP_598875.2	187	8	45.09	Defb9	NP_631965.1	38	1	27.73
Prr18	XP_036016537.1	279	8	30.22	Defb11	NP_631967.1	54	0	0.00
T	XP_036016337.1	821	0	0.00	Defb15	NP_631968.1	178	2	11.84
Pde10a	XP_017172949.1	972	9	9.76	Defb35	NP_631970.1	137	2	15.39
1700010I14Ril	NP_001334473.1	49	7	150.56	Defb13	NP_631969.1	78	1	13.51
Gm17748	XP_036016760.1	1472	0	0.00	Fam90a1a	NP_001075131.1	27	0	0.00
Pabpc6	NP_001157308.1	1305	0	0.00	Ccdc70	NP_001344153.1	418	4	10.09
Qk	XP_006523363.1	837	3	3.78	Atp7b	NP_031537.2	1162	1	0.91
Gm46608	XP_017173263.2	8	1	131.74	Alg11	NP_001230090.1	692	1	1.52
Pacrg	NP_081308.1	472	8	17.86	Nek5	NP_001334247.1	190	2	11.09
Prkn	XP_006523399.1	1569	4	2.69	Nek3	NP_035978.2	287	3	11.02

Agpat4	XP_006523408.3	447	1	2.36	Ckap2	NP_001004140.2	603	3	5.24
Map3k4	NP_001344651.1	437	1	2.41	Vps36	NP_081614.1	478	1	2.20
Plg	NP_032903.3	1866	5	2.82	Thsd1	XP_006509236.1	150	0	0.00
Slc22a3	NP_035525.1	557	5	9.46	Tpte	NP_954866.2	738	1	1.43
Slc22a2	NP_038695.1	636	4	6.63	Slc25a15	NP_001345900.1	271	0	0.00
Slc22a1	NP_033228.2	849	2	2.48	Mrps31	NP_065585.1	451	0	0.00
Igf2r	NP_034645.2	948	10	11.12	Smim19	NP_001012685.2	19	2	110.94
Mas1	XP_017172759.1	233	1	4.52	Slc20a2	NP_035524.2	725	2	2.91
Mrgprh	NP_109651.1	112	4	37.64	Vdac3	NP_035826.1	1119	2	1.88
Pnldc1	XP_006523388.1	353	3	8.96	Dkk4	NP_663567.1	330	0	0.00
Mrpl18	XP_006523407.1	594	2	3.55	Polb	NP_035260.1	399	0	0.00
Tcp1	NP_038714.2	1946	5	2.71	Ikbkb	NP_001153246.1	1676	1	0.63
Acat3	NP_694791.2	871	4	4.84	Plat	NP_032898.2	788	1	1.34
Acat2	NP_033364.2	756	3	4.18	Ap3m2	NP_083781.2	444	5	11.87
Gm15946	XP_036016772.1	735	3	4.30	Kat6a	NP_001074618.1	1119	2	1.88
Wtap	NP_001107005.1	1050	0	0.00	Ank1	XP_006509063.1	1939	3	1.63
Sod2	NP_038699.2	2713	2	0.78	Nkx6-3	NP_083278.1	213	2	9.90
Gpr31b	NP_001013854.2	445	3	7.10	Gpat4	NP_061213.2	500	3	6.32
Tcp10b	NP_033367.2	345	1	3.05	Gins4	NP_077202.1	468	2	4.50
Unc93a	NP_954860.1	142	3	22.27	Golga7	NP_065610.2	251	5	20.99
Smok2a	NP_038769.1	172	4	24.51	Sfrp1	NP_038862.2	855	2	2.47
Smok2b	NP_001161385.1	155	4	27.20	Zmat4	XP_030099470.1	360	2	5.86
Tcp10c	XP_006523340.1	408	1	2.58	Tcim	NP_081207.1	157	0	0.00
Tcte2	XP_036016362.1	1	0	0.00	Ido2	XP_006509109.1	517	0	0.00
Afdn	XP_006523815.1	866	6	7.30	Ido1	NP_032350.1	804	0	0.00
Gm7168	XP_006524826.1	95	2	22.19	Adam18	NP_034214.2	213	7	34.64
Gm7356	NP_001365198.1	132	2	15.97	Adam3	NP_033749.2	416	3	7.60
Gm7358	XP_978062.3	132	2	15.97	Adam5	NP_001258987.1	208	5	25.33
4930488N24R	XP_011245038.1	132	2	15.97	Adam32	NP_700446.2	275	11	42.16
Dact2	XP_006524290.1	300	11	38.64	Adam9	NP_031430.2	661	2	3.19
Smoc2	XP_006524840.1	540	11	21.47	Tm2d2	NP_081470.2	187	14	78.90
Thbs2	NP_035711.2	906	12	13.96	Htra4	XP_036010000.1	432	13	31.71
Wdr27	XP_006524992.1	611	14	24.15	Plekha2	NP_112547.1	227	11	51.07
1600012H06R	NP_001077350.1	135	10	78.07	Tacc1	XP_006509209.1	309	9	30.70
Phf10	NP_077212.3	341	16	49.45	Fgfr1	NP_034336.2	1579	8	5.34
Gm3417	NP_001347590.1	335	13	40.90	Letm2	XP_030099450.1	325	15	48.64
9030025P20R	NP_001116842.1	87	14	169.59	Nsd3	NP_001074738.1	1192	12	10.61
Gm3448	NP_001347591.1	335	13	40.90	Plpp5	NP_082276.1	397	11	29.20
Gm3435	NP_001116844.1	87	14	169.59	Ddhd2	XP_011240462.1	367	12	34.46
Tcte3	NP_932772.1	335	13	40.90	Bag4	NP_080397.1	395	11	29.35
Ermard	XP_036016552.1	87	13	157.48	Lsm1	NP_080308.1	575	11	20.16
Dll1	NP_001365971.1	910	13	15.06	Star	NP_035615.2	496	3	6.37
Fam120b	NP_001344751.1	156	13	87.83	Ash2l	NP_035921.2	794	9	11.95
Psmb1	NP_035315.1	1500	12	8.43	Kcnu1	XP_006509087.1	440	2	4.79
Tbp	NP_038712.3	2172	11	5.34	Hgsnat	NP_084160.1	343	3	9.22
Pdcd2	XP_036016290.1	659	13	20.79	Pomk	NP_083313.1	224	3	14.11
Prdm9	XP_036016347.1	637	4	6.62	Fnta	NP_032059.1	575	6	11.00
Chd1	NP_031716.2	1820	6	3.47	Hook3	XP_006509213.1	326	5	16.16
Rgmb	NP_001359187.1	290	4	14.54	Rnf170	NP_001344221.1	358	4	11.78
Zfp960	NP_001157391.1	152	0	0.00	Thap1	NP_950243.1	520	3	6.08
Zfp97	XP_030105624.1	145	0	0.00	Zfp703	NP_001094972.1	307	6	20.60
Riok2	NP_080210.1	1820	6	3.47	Erlin2	NP_001351406.1	470	9	20.18
Lix1	NP_079957.2	320	4	13.17	Plpbp	NP_001350408.1	387	8	21.79
Lnpep	NP_766415.1	814	4	5.18	Adgra2	XP_006509286.3	824	6	7.67
Vmn2r90	NP_001098009.1	34	0	0.00	Brf2	NP_079962.1	454	2	4.64
Spaca6	XP_011244994.1	82	0	0.00	Rab11fip1	NP_083699.2	346	6	18.28
Has1	NP_032241.1	662	0	0.00	Got1l1	XP_006509274.1	489	8	17.24
Fpr1	NP_038549.1	768	3	4.12	Adrb3	XP_030099106.1	550	2	3.83
Fpr2	NP_032065.1	859	2	2.45	Eif4ebp1	NP_031944.3	981	2	2.15
Fpr3	NP_032068.2	766	2	2.75	Tex24	NP_001013627.1	106	0	0.00
Fpr-rs4	NP_032067.2	440	3	7.19	Chrnb3	NP_775304.1	560	2	3.76
Vmn2r124	NP_001258812.1	11	0	0.00	Chrna6	NP_067344.2	715	2	2.95
Vmn2r91	NP_001372053.1	25	0	0.00	Poteg	XP_006509255.1	910	2	2.32
Vmn2r92	NP_001372034.1	7	0	0.00	Unc5d	NP_001334427.1	369	1	2.86
Vmn2r94	NP_001098013.1	7	0	0.00	Dusp26	NP_001344152.1	1149	6	5.50
Vmn2r93	NP_001098012.1	25	0	0.00	Rnf122	NP_001355304.1	388	4	10.86

Vmn2r95	NP_001096051.1	32	0	0.00	Tti2	NP_001186917.1	150	4	28.10
Vmn2r96	NP_001372010.1	46	0	0.00	Mak16	NP_080729.1	1008	5	5.23
Vmn2r97	NP_001372007.1	25	0	0.00	Fut10	XP_030099189.1	111	2	18.99
Vmn2r98	NP_001098020.1	46	0	0.00	Nrg1	XP_006509131.1	951	0	0.00
Vmn2r99	NP_001098021.2	26	0	0.00	Wrn	XP_036009773.1	1337	2	1.58
Vmn2r100	NP_001098032.1	9	0	0.00	Purg	XP_036010239.1	170	1	6.20
Vmn2r101	NP_001098033.1	7	0	0.00	Tex15	XP_006509038.1	237	0	0.00
Vmn2r102	NP_001098034.1	29	1	36.34	Ppp2cb	NP_059070.1	2613	5	2.02
Vmn2r103	NP_001098035.1	11	0	0.00	Ubxn8	XP_006509043.1	586	1	1.80
Vmn2r104	NP_001098036.1	44	1	23.95	Gsr	NP_034474.4	1450	1	0.73
Fpr-rs7	XP_030106054.1	322	0	0.00	Gtf2e2	XP_006509248.1	705	3	4.48
Fpr-rs6	NP_796290.2	406	1	2.60	Smim18	NP_001193778.1	7	0	0.00
Vmn2r105	NP_001356161.1	38	1	27.73	Rbpms	NP_001273097.1	488	2	4.32
Vmn2r106	NP_001372089.1	24	1	43.91	Dctn6	NP_035852.1	455	1	2.32
Vmn2r107	NP_001098039.1	52	0	0.00	Mboat4	XP_006509169.1	318	1	3.31
Vmn1r224	NP_001160207.1	100	6	63.23	Leprotl1	NP_080885.1	202	2	10.43
Vmn2r108	NP_001098040.1	28	1	37.64	Saraf	NP_080708.4	365	1	2.89
Vmn1r225	NP_598955.1	511	2	4.12	Dusp4	NP_795907.1	988	2	2.13
Vmn2r109	NP_001098041.1	7	0	0.00	Tnks	NP_780300.2	1503	2	1.40
Vmn2r110	NP_001098042.1	26	1	40.53	Ppp1r3b	XP_017168286.1	625	4	6.74
Fpr-rs3	NP_032066.2	390	1	2.70	Eri1	NP_080343.4	442	0	0.00
Vmn1r226	NP_598952.1	3	0	0.00	Mfhas1	NP_001074748.1	474	3	6.67
Vmn1r228	NP_598953.1	86	0	0.00	Cldn23	NP_082274.1	215	2	9.80
Vmn1r229	NP_598951.1	38	0	0.00	Rps12-ps24	XP_036010305.1	1285	1	0.82
Vmn1r230	NP_598958.1	77	0	0.00	Prag1	NP_766499.2	129	1	8.17
Vmn1r231	NP_598957.1	112	0	0.00	Lonrf1	NP_001074619.2	306	3	10.33
Vmn1r232	NP_598954.2	108	1	9.76	Trmt9b	NP_001371032.1	682	6	9.27
Ppp2r1a	NP_058587.1	1751	0	0.00	Dlc1	NP_001181869.1	360	6	17.57
Vmn1r233	NP_598963.1	183	0	0.00	AI429214	NP_001034309.1	18	4	234.20
Zfp160	NP_663458.2	470	5	11.21	Sgcz	NP_665840.2	265	10	39.77
Vmn1r234	NP_598959.1	41	0	0.00	Tusc3	NP_084530.3	579	4	7.28
Vmn1r235	NP_598960.3	37	0	0.00	Msr1	XP_006509374.1	620	1	1.70
Vmn1r236	NP_598962.2	84	0	0.00	Fgf20	NP_085113.2	640	3	4.94
Vmn1r237	NP_598961.1	180	1	5.86	Micu3	NP_084386.1	218	5	24.17
Zfp677	XP_030105480.1	199	2	10.59	Zdhhc2	NP_848482.1	414	4	10.18
Zfp54	NP_001366288.1	308	2	6.84	Cnot7	XP_036009724.1	606	1	1.74
Zfp51	NP_033584.3	392	9	24.20	Vps37a	NP_291038.2	298	0	0.00
Zfp53	XP_036016503.1	289	2	7.29	Mtmr7	NP_001035789.1	386	2	5.46
Zfp52	NP_653098.2	224	2	9.41	Adam24	NP_034216.3	333	1	3.16
Zfp948	NP_001344276.1	206	2	10.23	Adam25	NP_035911.2	201	1	5.24
Zfp983	XP_017173158.1	127	0	0.00	Adam20	XP_006509516.1	321	3	9.85
Gm10509	NP_001341682.1	127	0	0.00	Adam39	NP_001020551.3	220	1	4.79
Zfp760	XP_030105629.1	251	1	4.20	Slc7a2	XP_006509315.1	524	2	4.02
Zfp229	XP_030105720.1	200	1	5.27	Pdgfrl	NP_081116.3	548	3	5.77
Zfp820	NP_083557.2	167	1	6.31	Mtus1	XP_036009594.1	289	1	3.65
Zfp995	XP_030105902.1	126	0	0.00	Fgl1	NP_663569.2	653	2	3.23
Zfp942	XP_030105932.1	224	1	4.70	Pcm1	XP_036009708.1	734	2	2.87
Zfp943	NP_001020544.1	152	0	0.00	Asah1	XP_017168016.1	530	0	0.00
Zfp947	XP_030105481.1	203	0	0.00	Frg1	XP_017168059.1	247	3	12.80
Zfp994	XP_030105634.1	192	0	0.00	Triml1	XP_017168292.1	332	5	15.87
Zfp944	XP_036016531.1	126	0	0.00	Triml2	XP_017168415.1	315	5	16.73
Zfp758	XP_036016404.1	290	0	0.00	Zfp42	NP_033582.2	633	2	3.33
Zfp946	NP_001318124.1	172	0	0.00	Adam26b	NP_001009547.1	129	0	0.00
Vmn2r111	NP_001098043.1	28	0	0.00	Adam26a	NP_034215.2	382	1	2.76
Vmn2r112	NP_001098045.1	31	0	0.00	Adam34l	NP_001020411.1	192	0	0.00
Gm9805	NP_001363972.2	126	0	0.00	Adam34	NP_665688.2	185	0	0.00
Zfp945	NP_001103724.1	209	0	0.00	Gm5347	NP_001073400.1	193	1	5.46
Vmn2r113	NP_001098048.1	7	0	0.00	Fat1	XP_030099147.1	841	7	8.77
Zfp40	XP_017172945.1	242	0	0.00	Mtnr1a	NP_032665.1	591	5	8.92
Vmn2r114	NP_001096054.1	14	0	0.00	F11	NP_082342.1	881	4	4.79
Vmn2r115	NP_001098049.1	32	0	0.00	Klkb1	NP_032481.2	746	3	4.24
Vmn2r116	NP_001098050.1	109	0	0.00	Cyp4v3	NP_598730.1	744	8	11.33
Vmn2r117	NP_001098051.1	16	0	0.00	Fam149a	NP_705763.1	104	11	111.47
Zfp213	NP_001028668.2	190	1	5.55	Tlr3	NP_001344245.1	1552	2	1.36
Zfp13	NP_035877.1	241	0	0.00	Sorbs2	XP_011240500.1	952	10	11.07
Zscan10	NP_001028597.2	498	3	6.35	Pdlim3	NP_058078.1	1007	6	6.28

Mmp25	NP_001028511.1	575	1	1.83	Ccdc110	NP_001028418.1	78	4	54.05
Bicdl2	XP_030105482.1	109	0	0.00	1700029J07R	NP_001346042.1	8	0	0.00
Thoc6	NP_001344309.1	503	0	0.00	Ufsp2	XP_017168107.1	284	1	3.71
Hcfc1r1	NP_861542.1	118	1	8.93	Ankrd37	XP_030099570.2	1215	7	6.07
Tnfrsf12a	NP_001155218.1	558	0	0.00	Lrp2bp	NP_080554.1	464	7	15.90
Cldn6	XP_006524704.1	379	2	5.56	Snx25	NP_001359272.1	442	10	23.84
Cldn9	NP_064689.2	248	0	0.00	Cfap97	NP_001344145.1	251	7	29.39
Pkmyt1	XP_006524373.1	1202	2	1.75	Slc25a4	NP_031476.3	1390	9	6.82
Paqr4	NP_076313.1	210	3	15.06	Helt	NP_776150.2	336	11	34.50
Kremen2	XP_006525046.3	382	0	0.00	Acsl1	XP_017168052.1	1092	8	7.72
Flywch1	NP_001344923.1	35	1	30.11	Cenpu	NP_082249.1	518	4	8.14
Flywch2	NP_001344069.1	44	0	0.00	Primpol	XP_036010045.1	279	5	18.89
Srrm2	NP_001355616.1	980	0	0.00	Casp3	XP_017168032.1	3496	5	1.51
Elob	NP_080581.1	1217	2	1.73	Irf2	XP_011240477.1	585	5	9.01
Prss33	XP_017173005.1	209	2	10.09	Enpp6	NP_796278.1	336	2	6.27
Prss41	NP_081920.1	222	5	23.74	Stox2	XP_030099626.1	136	9	69.74
Prss32	NP_081496.2	189	1	5.58	Trappc11	NP_796214.2	319	0	0.00
Prss21	NP_065233.2	663	3	4.77	Rwdd4a	NP_987103.1	290	1	3.63
Dcpp1	XP_017172711.1	90	0	0.00	Ing2	NP_075992.2	758	5	6.95
Dcpp2	XP_017173087.1	22	0	0.00	Cdkn2aip	NP_765995.1	134	0	0.00
Dcpp3	XP_030105796.1	5	0	0.00	Cldn24	NP_001104788.1	50	0	0.00
Sbp	NP_001371027.1	129	1	8.17	Cldn22	NP_083659.1	151	0	0.00
Sbpl	NP_001070889.1	50	1	21.08	Wwc2	NP_598552.2	734	2	2.87
Prss30	NP_038949.2	134	0	0.00	Dctd	XP_036009996.1	422	2	4.99
Prss22	XP_006524968.4	251	1	4.20	Tenm3	XP_036009897.1	435	9	21.80
Prss27	NP_780649.1	288	2	7.32	Aga	NP_001005847.1	457	1	2.31
Kctd5	NP_081284.2	313	0	0.00	Neil3	NP_666320.1	608	4	6.93
Pdpk1	NP_001074242.1	916	1	1.15	Vegfc	NP_033532.1	788	1	1.34
Amdhd2	NP_766523.2	274	0	0.00	Spcs3	NP_083977.1	437	9	21.71
Atp6v0c	NP_001348460.1	1129	2	1.87	Asb5	NP_083845.1	1411	9	6.72
Tbc1d24	NP_775278.3	433	2	4.87	Spata4	NP_598472.2	385	11	30.11
Ntn3	NP_035077.1	235	1	4.48	Wdr17	XP_036009921.1	1225	11	9.46
Tedc2	NP_082332.1	57	1	18.49	Gpm6a	NP_001240683.1	1236	10	8.53
Ccnf	NP_031660.3	1959	2	1.08	Adam29	NP_787953.2	409	8	20.61
Abca17	XP_017173025.1	294	1	3.58	Glra3	NP_536686.3	450	11	25.76
Abca3	XP_006524433.1	596	2	3.54	Hpgd	NP_032304.2	724	6	8.73
Rnps1	NP_001073597.1	1012	1	1.04	Cep44	XP_017168359.1	121	2	17.42
Eci1	NP_034153.2	965	3	3.28	Fbxo8	NP_056606.2	797	8	10.58
Dnase1l2	NP_079994.2	175	1	6.02	Hand2	NP_034532.3	1078	2	1.96
E4f1	NP_031919.2	482	1	2.19	Scrg1	XP_017168111.1	362	5	14.56
Pgp	NP_080230.2	511	1	2.06	Sap30	NP_068560.1	401	4	10.51
Bricd5	NP_783613.1	0	0	#DIV/0!	Hmgb2	NP_001350372.1	1275	3	2.48
Mlst8	XP_006524742.1	889	2	2.37	Galnt7	NP_653332.3	279	2	7.55
Caskin1	XP_006524376.1	1350	5	3.90	Galntl6	XP_030099456.1	228	4	18.49
Traf7	NP_001165584.1	1381	3	2.29	BC030500	XP_006509453.1	43	0	0.00
Rab26	NP_796349.1	309	3	10.23	Aadat	NP_035964.1	862	7	8.56
Pkd1	XP_036016298.1	499	3	6.34	Mfap3l	NP_001171352.1	120	1	8.78
Tsc2	XP_017172881.1	1698	4	2.48	Hpf1	NP_001365956.1	308	1	3.42
Nthl1	NP_032769.2	683	3	4.63	Clcn3	NP_776299.1	572	0	0.00
Slc9a3r2	NP_001344725.1	964	3	3.28	Nek1	NP_001280566.1	531	3	5.95
Npw	NP_001093134.1	361	1	2.92	Sh3rf1	XP_011240533.1	689	7	10.71
Zfp598	NP_898972.1	359	0	0.00	Gm10015	XP_036010320.1	1647	2	1.28
Syngr3	NP_035652.2	881	4	4.79	Cbr4	NP_663570.2	573	1	1.84
Gfer	NP_001365967.1	817	4	5.16	Palld	XP_006509566.1	581	5	9.07
Noxo1	NP_001344765.1	186	3	17.00	Ddx60	NP_001280712.1	2001	1	0.53
Tbl3	NP_663371.2	860	5	6.13	Anxa10	NP_001129561.1	347	3	9.11
Rnf151	NP_080481.1	364	0	0.00	Spock3	XP_006509579.1	762	3	4.15
Rps2	NP_032529.2	1326	2	1.59	Sgo2b	NP_001182616.1	216	3	14.64
Ndufb10	NP_080960.1	685	1	1.54	Tll1	NP_033416.2	451	4	9.35
Rpl3l	NP_001157417.1	1491	6	4.24	Cpe	NP_038522.2	909	1	1.16
Msrb1	NP_001333597.1	311	0	0.00	Msmo1	NP_079712.1	722	1	1.46
Hs3st6	NP_001012402.1	180	1	5.86	Klhl2	NP_848748.2	655	2	3.22
Meiob	NP_083473.1	995	2	2.12	Tmem192	NP_082703.1	16	0	0.00
Fahd1	NP_075969.1	437	1	2.41	Trim75	NP_001028601.1	581	3	5.44
Hagh	NP_077246.2	575	5	9.16	Trim60	NP_694737.1	524	4	8.05
Igfals	NP_001351824.1	1369	7	5.39	Trim61	NP_694750.3	275	1	3.83

Nubp2	NP_001365981.1	589	5	8.95	Apela	NP_001284483.1	276	2	7.64
Spsb3	XP_006525228.1	320	3	9.88	Marchf1	XP_017168467.1	572	1	1.84
Eme2	NP_001156574.1	116	0	0.00	Tma16	NP_079741.1	145	1	7.27
Mrps34	NP_075749.1	564	5	9.34	Tktl2	NP_083203.2	948	1	1.11
Nme3	NP_062704.2	642	5	8.21	Npy5r	NP_001345887.1	666	1	1.58
Mapk8ip3	NP_001156923.1	668	5	7.89	Npy1r	NP_001345884.1	669	2	3.15
Jpt2	NP_945175.1	103	0	0.00	Naf1	NP_001157036.1	453	0	0.00
Cramp1l	XP_006524762.1	120	3	26.35	Nat1	NP_032699.1	183	1	5.76
Ift140	NP_598887.3	417	2	5.05	Nat2	XP_006509655.1	199	1	5.30
Tmem204	NP_001001183.1	211	2	9.99	Nat3	NP_032700.1	196	2	10.75
Telo2	NP_082156.2	354	2	5.95	Psd3	XP_036009814.1	526	1	2.00
Ptx4	NP_081023.1	192	2	10.98	Sh2d4a	NP_082458.1	216	1	4.88
Clcn7	NP_036060.1	438	2	4.81	Csgalnact1	NP_001351185.1	384	0	0.00
Ccdc154	XP_006524012.1	15	0	0.00	Ints10	XP_006509804.1	275	0	0.00
BC003965	NP_898973.2	48	0	0.00	Lpl	NP_032535.2	891	0	0.00
Unkl	NP_001183953.1	541	1	1.95	Slc18a1	XP_011240564.1	402	1	2.62
Gnptg	NP_766117.2	450	1	2.34	Atp6v1b2	NP_031535.2	930	1	1.13
Tsr3	XP_036016652.1	224	0	0.00	Lzts1	NP_001357900.1	312	0	0.00
Baiap3	NP_001156742.1	317	2	6.65	Zfp930	NP_001013397.2	558	0	0.00
Ube2i	NP_001344623.1	2689	3	1.18	D130040H23I	NP_001371053.1	127	0	0.00
Prss34	NP_848459.1	176	2	11.98	Gm10033	NP_001361529.1	126	0	0.00
Prss28	NP_444489.2	227	1	4.64	Gm9495	XP_017168526.1	983	0	0.00
Prss29	NP_444490.2	198	0	0.00	Gm7697	XP_983008.3	958	0	0.00
Tpsb2	NP_034911.3	343	2	6.15	Zfp868	NP_766342.2	136	0	0.00
Tpsg1	XP_006524421.1	182	2	11.58	Zfp964	NP_001170998.1	140	0	0.00
Cacna1h	NP_067390.4	797	1	1.32	Zfp869	NP_001346015.1	161	0	0.00
Tekt4	XP_006525011.1	380	2	5.55	Zfp963	NP_001186952.1	135	1	7.81
Sstr5	NP_001177937.1	533	4	7.91	Zfp866	NP_808567.2	144	1	7.32
Sox8	NP_035577.1	713	3	4.43	Atp13a1	NP_573487.2	820	9	11.57
Lmf1	NP_083900.3	334	5	15.78	Gmip	XP_030099717.2	404	10	26.09
Gng13	NP_071867.1	1908	7	3.87	Lpar2	NP_064412.2	671	6	9.42
Chtf18	NP_663384.2	816	1	1.29	Pbx4	NP_001020125.1	400	11	28.98
Rpusd1	XP_036016143.1	343	1	3.07	Cilp2	NP_081094.2	251	12	50.39
Mslnl	NP_808490.1	253	7	29.16	Yjefn3	NP_001340859.1	382	15	41.38
Msln	NP_001343215.1	624	4	6.76	Ndufa13	NP_075801.1	490	2	4.30
Ciao3	NP_080514.3	297	0	0.00	Tssk6	NP_114393.1	1271	15	12.44
Haghl	NP_001258364.1	204	3	15.50	Gatad2a	NP_001273379.1	437	16	38.59
Ccdc78	XP_017173031.1	160	7	46.11	Mau2	NP_001161411.1	187	14	78.90
Antkmt	NP_663385.2	483	3	6.55	Sugp1	XP_030099615.1	521	15	30.34
Metrn	NP_598480.1	251	5	20.99	Tm6sf2	NP_001280724.1	469	14	31.46
Fbxl16	NP_001366323.1	1510	8	5.58	Hapln4	NP_808568.1	420	11	27.60
Wdr24	NP_776102.1	217	1	4.86	Ncan	NP_031815.2	935	12	13.53
Jmjd8	NP_082377.3	86	0	0.00	Nr2c2ap	XP_006509840.1	105	12	120.45
Stub1	NP_062693.1	1760	4	2.40	Rfxank	NP_001020760.1	1087	13	12.60
Rhbdl1	NP_659065.1	287	1	3.67	Borcs8	NP_001139024.1	123	3	25.70
Rhot2	NP_666111.1	1634	7	4.51	Mef2b	NP_001296984.1	824	4	5.12
Wdr90	NP_001157238.1	1421	4	2.97	Tmem161a	XP_011240598.1	155	2	13.60
Mcrip2	NP_080909.1	217	1	4.86	Slc25a42	NP_001007571.1	317	6	19.95
Mettl26	NP_080962.1	106	2	19.88	Armc6	NP_598733.2	100	0	0.00
Wfikkn1	NP_001093924.1	220	0	0.00	Sugp2	XP_036009839.1	374	2	5.64
Rab40c	NP_631893.1	685	1	1.54	Homer3	XP_006509744.1	302	1	3.49
Pigq	NP_001344521.1	307	0	0.00	Ddx49	NP_001020093.2	1755	5	3.00
Nhlrc4	NP_001034127.1	198	0	0.00	Cope	NP_067513.1	862	1	1.22
A930017K11R	NP_001344948.1	84	0	0.00	Cers1	NP_619588.1	352	0	0.00
Capn15	XP_006524668.1	325	0	0.00	Upf1	NP_109605.2	1470	4	2.87
Rab11fip3	XP_036016360.1	515	3	6.14	Comp	NP_057894.2	476	1	2.21
Decr2	NP_036063.1	614	3	5.15	Crtc1	XP_006509763.1	394	1	2.67
Nme4	XP_006524740.1	832	3	3.80	Klhl26	NP_001116302.1	190	1	5.55
Pgap6	NP_068565.1	226	6	27.98	Tmem59l	NP_001355296.1	1086	1	0.97
Mrpl28	NP_077189.2	564	1	1.87	Crlf1	NP_001365728.1	381	5	13.83
Axin1	NP_033863.2	1467	7	5.03	Rex1bd	NP_079853.1	209	3	15.13
Pdia2	XP_006524935.1	1245	8	6.77	Uba52	NP_001335156.1	2840	2	0.74
Arhgdig	NP_032139.1	573	5	9.20	Kxd1	NP_083642.1	644	1	1.64
Rgs11	NP_001074538.1	485	6	13.04	Fkbp8	NP_001104536.1	1329	2	1.59
Fam234a	XP_030105273.1	149	6	42.44	Ell	NP_031950.2	493	4	8.55
Luc7l	NP_082466.2	770	7	9.58	Isyna1	NP_076116.1	870	5	6.06

Neurl1b	NP_001075125.1	715	0	0.00	Ssbp4	NP_001359404.1	147	6	43.02
Dusp1	NP_038670.1	1479	0	0.00	Lrrc25	XP_036009747.1	309	6	20.46
Ergic1	XP_006524886.1	669	3	4.73	Gdf15	NP_001317616.1	840	2	2.51
Atp6v0e	NP_079548.1	267	3	11.84	Pgpep1	XP_006509782.1	207	7	35.64
Crebrf	XP_017173212.1	204	3	15.50	Lsm4	NP_056631.2	1225	6	5.16
Bnip1	NP_742161.4	444	7	16.62	Jund	NP_034722.1	908	1	1.16
Nkx2-5	XP_006523860.1	1053	2	2.00	Gm3336	NP_001182182.1	21	7	351.30
Kifc5b	NP_444403.2	1224	5	4.31	Pde4c	XP_011240550.1	473	0	0.00
Phf1	NP_033369.2	538	5	9.79	Rab3a	NP_001314976.1	1343	2	1.57
Cuta	XP_030105873.1	340	4	12.40	Mpv17l2	NP_898993.1	355	4	11.87
Syngap1	XP_006524303.1	1134	5	4.65	Ifi30	NP_075552.2	530	4	7.95
Zbtb9	NP_001005916.1	202	5	26.09	Pik3r2	NP_032867.2	1060	2	1.99
Ggnbp1	NP_001238810.1	415	0	0.00	Mast3	XP_006509774.2	391	2	5.39
Bak1	NP_031549.2	698	3	4.53	Il12rb1	XP_006509620.1	424	4	9.94
Itpr3	XP_006523775.1	1073	3	2.95	Arrdc2	NP_081836.1	626	0	0.00
Uqcc2	NP_001351873.1	411	3	7.69	Kcnn1	XP_036010288.1	340	5	15.50
Ip6k3	NP_766615.1	858	2	2.46	Ccdc124	XP_036009842.1	327	2	6.45
Lemd2	NP_666187.2	167	1	6.31	Slc5a5	XP_006509600.1	385	4	10.95
Grm4	NP_001013403.1	938	6	6.74	Rpl18a	NP_084027.1	983	0	0.00
Hmga1	NP_001160018.1	489	8	17.24	Map1s	NP_766601.2	509	1	2.07
AI413582	NP_001002895.2	5	0	0.00	Gm10654	NP_001344584.1	0	0	#DIV/0!
Nudt3	NP_062811.1	827	5	6.37	Haus8	NP_083897.2	297	1	3.55
Rps10	NP_001351863.1	941	15	16.80	Myo9b	XP_006509629.1	1456	6	4.34
Pacsin1	NP_848142.1	1143	9	8.30	Use1	XP_036010138.1	322	0	0.00
Spdef	NP_001344657.1	440	9	21.56	Ocel1	NP_084141.2	110	2	19.16
Ilrun	NP_001258440.1	178	11	65.13	Nr2f6	NP_034280.2	446	6	14.18
Snrpc	NP_035562.1	953	10	11.06	Ushbp1	XP_006509719.1	239	5	22.05
Uhrf1bp1	NP_001074238.1	295	13	46.44	Babam1	XP_017168436.1	315	8	26.77
Taf11	NP_001366297.1	470	10	22.42	Ankle1	NP_766344.2	1252	6	5.05
Anks1	XP_006524150.1	1036	14	14.24	Abhd8	XP_030099565.1	353	8	23.88
Tcp11	NP_001344610.1	457	9	20.76	Mrpl34	NP_444392.1	477	6	13.26
Scube3	XP_006524386.1	412	13	33.25	Dda1	XP_011240619.1	150	1	7.03
Zfp523	XP_017172898.1	404	11	28.70	Ano8	XP_017168362.1	408	0	0.00
Def6	NP_081461.2	180	0	0.00	Gtpbp3	NP_115933.2	580	2	3.63
Ppard	XP_036016302.1	916	8	9.20	Plvap	NP_115774.2	257	2	8.20
Fance	NP_001157291.1	239	8	35.28	Bst2	NP_932763.1	681	2	3.10
Rpl10a	NP_035417.2	1578	3	2.00	Mvb12a	NP_082893.1	226	4	18.65
Tead3	NP_001091696.2	586	8	14.39	Tmem221	NP_001093932.1	52	0	0.00
Tulp1	XP_017172883.1	418	6	15.13	Nxnl1	NP_663573.1	271	4	15.56
Fkbp5	NP_034350.1	1630	2	1.29	Slc27a1	XP_030099434.1	530	3	5.97
Armc12	NP_080566.2	149	5	35.37	Pgls	NP_079672.1	791	2	2.66
Clpsl2	NP_001030043.1	5	0	0.00	Niban3	XP_006509593.1	137	1	7.69
Clps	NP_001303994.1	472	3	6.70	Colgalt1	NP_666323.2	244	2	8.64
Lhfpl5	NP_080847.2	415	2	5.08	Unc13a	XP_030099506.1	936	0	0.00
Srpk1	NP_058075.2	896	9	10.59	Jak3	NP_001177759.1	1639	3	1.93
Slc26a8	XP_011244689.1	272	8	31.00	Insl3	NP_038592.3	381	1	2.77
Mapk14	NP_036081.1	4069	8	2.07	B3gnt3	NP_082465.3	208	1	5.07
Mapk13	NP_036080.2	2397	7	3.08	Fcho1	XP_011240633.1	691	1	1.53
Brpf3	NP_001074784.1	654	3	4.83	Zfp709	NP_663599.3	191	0	0.00
Pnpla1	NP_001030057.1	268	0	0.00	Zfp882	NP_001160117.1	127	0	0.00
Bnip5	NP_766038.1	52	1	20.27	Zfp617	NP_579936.2	325	0	0.00
Pxt1	NP_700439.1	97	3	32.59	Zfp961	NP_001158053.1	154	0	0.00
Kctd20	NP_001343227.1	265	3	11.93	Cyp4f18	NP_077764.2	390	0	0.00
Stk38	NP_001344102.1	1914	7	3.85	Olfr372	NP_997438.1	16	0	0.00
Srsf3	NP_038691.1	1115	4	3.78	Olfr373	NP_666750.2	16	0	0.00
Cdkn1a	NP_001104569.1	1894	3	1.67	Olfr374	NP_666450.2	33	0	0.00
Rab44	NP_001351777.1	540	2	3.90	Tpm4	NP_001001491.1	1119	3	2.83
Cpne5	NP_694806.1	268	1	3.93	Rab8a	NP_075615.2	1264	2	1.67
Ppil1	NP_081121.1	1291	2	1.63	Hsh2d	NP_922935.1	193	0	0.00
BC004004	NP_001366007.1	122	0	0.00	Cib3	XP_006509731.1	867	3	3.65
Pi16	NP_076223.3	146	0	0.00	Fam32a	NP_080731.1	62	0	0.00
Mtch1	NP_001334264.1	469	3	6.74	Ap1m1	NP_031482.1	682	1	1.55
Fgd2	XP_036016509.1	352	0	0.00	Klf2	NP_032478.2	1039	1	1.01
Pim1	NP_032868.2	1506	3	2.10	Eps15l1	NP_001276788.1	1033	2	2.04
Tmem217	XP_011244909.1	94	0	0.00	Calr3	XP_006509824.1	486	0	0.00
Tbc1d22b	NP_941049.1	308	2	6.84	1700030K09R	XP_011240630.1	0	0	#DIV/0!

Rnf8	NP_067394.1	1034	1	1.02	Cherp	NP_613051.3	430	3	7.35
Cmtr1	NP_083067.1	124	0	0.00	Slc35e1	XP_006509747.2	207	3	15.27
Ccdc167	NP_001157213.1	20	0	0.00	Med26	NP_081761.2	693	0	0.00
Mdga1	NP_001074629.1	314	1	3.36	Smim7	NP_765984.1	18	1	58.55
Zfand3	NP_001343194.1	334	2	6.31	Tmem38a	NP_653117.1	425	1	2.48
Btbd9	XP_030105556.1	320	3	9.88	Nwd1	XP_006531149.1	1575	2	1.34
Glo1	NP_079650.3	1148	3	2.75	Sin3b	NP_033214.2	1803	6	3.51
Dnah8	XP_036016186.1	878	5	6.00	F2rl3	NP_032001.2	363	0	0.00
Glp1r	NP_067307.2	617	3	5.12	Large1	NP_034817.1	279	0	0.00
Umodl1	NP_803416.2	364	7	20.27	Isx	NP_001281207.1	390	3	8.11
Abcg1	NP_033723.1	844	10	12.49	Hmgxb4	NP_821136.1	269	3	11.75
Tff3	NP_035705.1	429	9	22.11	Tom1	NP_001129731.1	500	5	10.54
Tff2	NP_033389.2	333	12	37.98	Hmox1	NP_034572.1	1243	0	0.00
Tff1	XP_006524093.1	700	11	16.56	Mcm5	NP_032592.2	1825	7	4.04
Tmprss3	NP_001157248.1	373	15	42.38	Rasd2	NP_083458.1	1793	4	2.35
Ubash3a	NP_808491.2	528	15	29.94	Iqcm	NP_082220.1	3	0	0.00
Rsph1	NP_001351845.1	524	15	30.17	Nr3c2	NP_001077375.1	891	3	3.55
Slc37a1	XP_036016419.1	426	14	34.64	Arhgap10	XP_017168504.1	347	0	0.00
Pde9a	NP_032830.3	523	16	32.24	Prmt9	NP_001074709.1	480	3	6.59
Wdr4	XP_030105793.1	615	17	29.13	Tmem184c	NP_001351374.1	121	3	26.13
Ndufv3	NP_084363.2	436	13	31.42	Ednra	NP_034462.1	672	4	6.27
Pknox1	NP_057879.2	532	13	25.75	Ttc29	NP_898919.3	95	1	11.09
Cbs	NP_835742.1	2051	13	6.68	Pou4f2	NP_620394.2	556	2	3.79
U2af1	NP_077149.2	803	13	17.06	Rbmxl1	NP_001345903.1	747	2	2.82
Cryaa	NP_001265498.1	509	12	24.85	Slc10a7	NP_084012.1	302	0	0.00
Sik1	NP_034961.2	499	3	6.34	Lsm6	NP_084421.1	1151	0	0.00
Hsf2bp	NP_083178.1	191	6	33.11	Zfp827	XP_006531309.1	248	1	4.25
Rrp1b	NP_082520.2	864	8	9.76	1700011L22R	NP_080591.1	11	5	479.05
Notch3	NP_032742.1	1563	3	2.02	Mmaa	NP_001350399.1	176	1	5.99
Ephx3	NP_001334270.1	304	5	17.33	Smad1	NP_032565.2	964	0	0.00
Brd4	XP_006524754.1	2003	5	2.63	Otud4	NP_001242962.1	512	4	8.23
Akap8	NP_062748.2	349	4	12.08	Abce1	NP_056566.2	1536	12	8.23
Akap8l	XP_011244836.1	198	1	5.32	Anapc10	NP_001344164.1	905	10	11.65
Wiz	XP_030105530.1	406	6	15.57	Hhip	XP_006530765.1	768	8	10.98
Rasal3	NP_848900.2	493	2	4.28	Gypa	NP_034499.3	317	4	13.30
Pglyrp2	NP_001258407.1	616	2	3.42	Frem3	NP_001161370.1	329	7	22.42
Cyp4f39	NP_796281.1	247	0	0.00	Smarca5	NP_444354.2	2141	8	3.94
Cyp4f17	NP_001094915.1	181	0	0.00	Gab1	NP_067331.2	444	8	18.99
Cyp4f16	NP_001343232.1	235	1	4.48	Usp38	NP_081830.2	592	7	12.46
Cyp4f37	NP_001093657.1	223	0	0.00	Inpp4b	XP_017168218.1	400	10	26.35
Cyp4f40	XP_006524820.1	175	0	0.00	Il15	XP_006530777.1	1118	4	3.77
Cyp4f15	NP_598888.1	588	1	1.79	Zfp330	NP_001355272.1	205	2	10.28
Zfp871	NP_766046.3	244	0	0.00	Rnf150	XP_017168354.1	426	2	4.95
Zfp811	NP_899000.2	142	2	14.84	Tbc1d9	NP_001104774.1	639	4	6.60
Zfp799	XP_011244744.1	135	0	0.00	Ucp1	NP_033489.1	763	4	5.53
Zfp870	NP_997128.1	149	2	14.15	Elmod2	XP_036009926.1	223	3	14.18
Cyp4f14	NP_071879.1	729	5	7.23	Mgat4d	NP_080509.2	82	2	25.70
Cyp4f13	NP_570952.1	320	1	3.29	Clgn	XP_011246598.1	1001	4	4.21
Zfp472	NP_694703.2	187	0	0.00	Scoc	XP_011246750.1	299	2	7.05
Zfp952	NP_001039024.1	134	0	0.00	Olfr370	NP_666382.2	78	2	27.02
Zfp763	NP_082819.1	184	0	0.00	Ndufb7	NP_080119.1	715	2	2.95
Zfp563	NP_001020121.2	477	0	0.00	Tecr	NP_081455.1	668	5	7.89
Morc2b	NP_001347132.1	168	3	18.82	Dnajb1	NP_061278.1	1147	4	3.68
Olfr55	NP_035128.2	66	0	0.00	Gipc1	XP_030099599.1	615	3	5.14
Olfr239	NP_997058.2	66	0	0.00	Ptger1	XP_006530834.1	411	5	12.82
Olfr1564	NP_001185991.1	28	0	0.00	Pkn1	NP_796236.2	1270	3	2.49
Zfp955a	XP_006525185.1	134	0	0.00	Ddx39a	NP_001350045.1	2268	5	2.32
Olfr63	NP_667148.1	49	0	0.00	Adgre5	NP_001156503.1	646	5	8.16
Zfp955b	NP_001136429.1	135	0	0.00	Adgrl1	XP_036010006.1	761	3	4.15
Zfp81	NP_997424.1	190	0	0.00	Asf1b	NP_077146.1	1386	7	5.32
Zfp101	NP_033568.2	189	0	0.00	Prkaca	NP_032880.1	3772	7	1.96
Actl9	NP_899105.2	1422	3	2.22	Samd1	NP_001074884.1	724	7	10.19
Adamts10	NP_766207.2	277	2	7.61	1700067K01R	XP_006531466.1	25	15	632.34
Myo1f	NP_444444.2	1437	4	2.93	Misp3	NP_001278221.1	73	2	28.87
Zfp414	NP_080988.1	216	3	14.64	Palm3	NP_083153.1	161	7	45.82
Pram1	NP_001002842.2	144	0	0.00	Il27ra	XP_030099528.1	315	1	3.35

Hnrnpm	NP_001103383.1	1361	2	1.55	Rln3	NP_775276.1	484	7	15.24
Marchf2	NP_663461.2	494	0	0.00	Rfx1	XP_036009732.1	768	3	4.12
Rab11b	NP_033023.1	1961	6	3.22	Dcaf15	NP_001344251.1	127	11	91.28
Angptl4	NP_065606.2	1092	4	3.86	Podnl1	XP_011246679.1	545	11	21.27
Kank3	NP_109622.1	975	7	7.57	Cc2d1a	NP_001368800.1	477	6	13.26
Rps28	NP_001342313.1	1060	9	8.95	4930432K21R	NP_083321.2	63	12	200.74
Ndufa7	NP_075691.1	713	3	4.43	Nanos3	XP_036009928.1	553	3	5.72
Cd320	NP_062294.3	178	6	35.52	Zswim4	NP_766091.2	154	6	41.06
Kifc1	NP_001182227.1	1388	12	9.11	D8Ertd738e	XP_017167984.1	144	13	95.14
Daxx	NP_031855.3	1016	9	9.34	Mri1	NP_080699.3	358	5	14.72
Zbtb22	NP_065650.1	445	10	23.68	Ccdc130	NP_080626.1	506	5	10.41
Tapbp	NP_001020484.1	504	13	27.18	Cacna1a	XP_011246587.1	1297	0	0.00
Rgl2	NP_033085.2	366	17	48.95	Ier2	NP_034629.3	284	6	22.27
Pfdn6	NP_001172111.1	1020	16	16.53	Nacc1	XP_036010137.1	544	7	13.56
Wdr46	XP_006524760.1	868	15	18.21	Trmt1	NP_001158032.1	742	13	18.46
B3galt4	NP_062293.1	184	6	34.37	Lyl1	NP_032561.2	444	8	18.99
Rps18	NP_035426.1	1290	15	12.25	Nfix	XP_036009691.1	598	9	15.86
Vps52	NP_001366339.1	658	14	22.42	Dand5	NP_957679.1	192	4	21.96
H2-K1	NP_001334275.1	1099	8	7.67	Gadd45gip1	NP_899202.3	448	12	28.23
Ring1	NP_033092.3	797	16	21.16	Rad23a	NP_033036.2	2434	11	4.76
H2-Ke6	NP_038571.2	642	18	29.55	Calr	NP_031617.1	1691	4	2.49
Slc39a7	NP_001071177.1	408	17	43.91	Farsa	NP_079924.2	674	12	18.76
Rxrb	NP_001192143.1	664	14	22.22	Syce2	NP_001161718.1	334	12	37.86
Col11a2	XP_006523622.1	556	15	28.43	Gcdh	XP_036009971.1	519	13	26.40
H2-Oa	NP_032232.2	369	10	28.56	Klf1	NP_034765.3	695	12	18.20
Brd2	XP_030105359.1	1787	19	11.21	Dnase2a	NP_034192.1	246	10	42.84
H2-DMa	XP_017172749.1	591	10	17.83	Mast1	XP_006531280.1	2324	2	0.91
H2-DMb2	NP_034518.1	244	14	60.47	Rtbdn	NP_001344255.1	125	0	0.00
H2-DMb1	NP_034517.2	344	11	33.70	Rnaseh2a	NP_081463.1	995	2	2.12
Psmb9	NP_038613.1	1192	20	17.68	Prdx2	NP_035693.3	1647	4	2.56
Tap1	NP_038711.2	667	14	22.12	Junb	NP_032442.1	1102	3	2.87
Psmb8	NP_034854.2	1454	19	13.77	Hook2	NP_001359286.1	285	4	14.79
Tap2	NP_035660.3	563	16	29.95	Best2	NP_001123666.1	349	0	0.00
H2-Ob	NP_034519.2	337	11	34.40	Get3	NP_062626.1	752	1	1.40
H2-Ab1	NP_996988.2	978	12	12.93	Trir	NP_081036.2	152	0	0.00
H2-Aa	NP_034508.2	768	12	16.47	Tnpo2	NP_001350960.1	902	2	2.34
H2-Eb1	NP_034512.2	608	9	15.60	Fbxw9	NP_081067.2	662	1	1.59
H2-Eb2	NP_001029150.1	479	16	35.20	Gm5741	NP_001182460.1	391	0	0.00
Btnl2	NP_524574.1	240	18	79.04	Dhps	NP_001034603.1	554	3	5.71
Btnl1	NP_001104564.1	222	16	75.96	Wdr83	NP_080675.2	489	1	2.16
BC051142	XP_030105748.1	133	10	79.24	Wdr83os	NP_001001493.1	271	1	3.89
Btnl4	NP_109671.1	105	1	10.04	Man2b1	XP_006530810.1	553	2	3.81
Btnl6	NP_109672.1	65	2	32.43	Zfp791	NP_001344295.1	200	0	0.00
Notch4	XP_006523861.1	793	14	18.61	Cks1brt	NP_001033011.1	588	0	0.00
Gpsm3	NP_598877.1	634	11	18.29	Olfr371	NP_667070.1	12	0	0.00
Pbx2	NP_059491.1	582	14	25.35	Vps35	NP_075373.1	1356	7	5.44
Ager	NP_031451.2	363	14	40.65	Orc6	NP_062690.2	748	7	9.86
Rnf5	NP_062276.1	558	14	26.44	Mylk3	XP_006530888.1	681	2	3.10
Agpat1	XP_030105775.1	589	15	26.84	4921524J17R	NP_079998.1	117	0	0.00
Egfl8	XP_036016745.1	324	17	55.30	Gpt2	NP_776291.1	937	7	7.87
Ppt2	XP_006524697.1	556	13	24.64	Dnaja2	NP_062768.1	1220	9	7.77
Prrt1	NP_001355658.1	606	13	22.61	Neto2	XP_036010231.1	292	7	25.26
Fkbpl	NP_063926.1	879	14	16.79	Itfg1	NP_082283.2	249	12	50.79
Atf6b	NP_059102.2	456	17	39.29	Phkb	XP_006530594.1	425	1	2.48
Tnxb	XP_006525248.1	982	19	20.39	Abcc12	NP_766500.3	494	9	19.20
C4b	XP_006523595.1	1050	6	6.02	Lonp2	NP_080103.1	684	2	3.08
Cyp21a1	NP_034125.2	495	14	29.81	Siah1a	XP_006530847.3	730	0	0.00
Stk19	NP_062315.1	232	18	81.77	N4bp1	XP_006531580.1	336	7	21.96
Dxo	NP_291091.2	361	15	43.79	Cbln1	NP_062600.2	706	12	17.91
Skiv2l	NP_067312.2	1299	17	13.79	4933402J07R	NP_808569.1	213	5	24.74
Nelfe	NP_613046.2	478	14	30.87	Zfp423	XP_030099736.1	701	9	13.53
Cfb	NP_032224.2	1219	15	12.97	Cnep1r1	NP_083350.2	207	7	35.64
C2	NP_038512.2	371	9	25.57	Heatr3	NP_766345.3	356	7	20.72
Zbtb12	NP_942589.3	314	14	46.99	Tent4b	XP_006530893.1	791	9	11.99
Ehmt2	XP_006523545.1	1739	10	6.06	Adcy7	XP_030099104.1	1083	9	8.76
Slc44a4	XP_006524948.1	330	11	35.13	Brd7	NP_001363945.1	492	11	23.56

Neu1	NP_035023.3	313	8	26.94	Nkd1	NP_081556.3	560	10	18.82
Hspa1b	NP_034608.2	3513	10	3.00	Snx20	XP_011246805.1	430	7	17.16
Hspa1a	NP_034609.2	2438	8	3.46	Nod2	XP_006531091.1	865	2	2.44
Hspa1l	NP_038586.2	2287	10	4.61	Cyld	NP_001121642.1	661	3	4.78
Lsm2	NP_001191203.1	1404	13	9.76	Sall1	NP_067365.2	708	10	14.89
Vars	XP_017172884.1	2253	14	6.55	Gm6625	XP_036010327.1	247	5	21.33
Vwa7	XP_017172986.1	406	9	23.36	Tox3	XP_006531052.1	812	6	7.79
Sapcd1	XP_036016738.1	80	8	105.39	Chd9	XP_017168006.1	1525	8	5.53
Msh5	XP_030105392.1	677	13	20.24	Rbl2	NP_035380.3	1064	7	6.93
Clic1	NP_254279.1	921	8	9.15	Aktip	XP_030099154.1	712	7	10.36
Ddah2	NP_001177378.1	328	14	44.98	Rpgrip1l	XP_036009932.1	538	7	13.71
Mpig6b	NP_001177941.1	185	12	68.36	Fto	NP_036066.2	627	8	13.45
Ly6g6c	NP_075952.1	257	15	61.51	Irx3	NP_001240751.1	695	7	10.61
Ly6g6d	NP_001357956.1	227	10	46.43	Irx5	NP_061296.1	522	6	12.11
Ly6g6e	XP_030105903.1	164	9	57.84	Irx6	NP_071873.2	456	3	6.93
Ly6g6f	NP_001156664.1	100	8	84.31	Mmp2	XP_006530814.1	1213	0	0.00
Abhd16a	NP_848707.1	327	17	54.79	Lpcat2	NP_766602.1	410	2	5.14
Ly6g5c	NP_683749.1	173	16	97.47	Capns2	NP_081388.1	239	1	4.41
Ly6g5b	NP_683741.1	160	15	98.80	Slc6a2	NP_033235.3	552	1	1.91
Csnk2b	NP_001290405.1	1906	15	8.29	Ces1a	NP_001013786.2	168	0	0.00
Gpank1	XP_030106017.1	130	10	81.07	Ces1b	NP_001074841.1	748	1	1.41
D17H6S53E	NP_258438.1	103	10	102.32	Ces1c	NP_031980.2	729	1	1.45
Apom	NP_061286.1	694	9	13.67	Ces1d	NP_444430.2	987	4	4.27
Bag6	XP_006524181.1	703	11	16.49	Ces1e	XP_006530714.1	748	1	1.41
Prrc2a	XP_030105760.1	491	17	36.49	Ces1f	NP_659179.1	625	1	1.69
Aif1	NP_001348431.1	1949	9	4.87	Ces1g	NP_067431.2	573	0	0.00
Lst1	NP_034864.2	302	7	24.43	Ces1h	NP_001357759.1	144	1	7.32
Ltb	NP_032544.1	826	11	14.03	Ces5a	NP_001003951.1	362	1	2.91
Tnf	NP_001265530.1	3908	14	3.78	Gnao1	NP_034438.1	1977	1	0.53
Lta	XP_006523794.1	793	6	7.97	Amfr	NP_035917.2	917	1	1.15
Nfkbil1	NP_035039.1	282	8	29.90	Nudt21	NP_080899.1	857	0	0.00
Atp6v1g2	NP_075668.1	1154	5	4.57	Ogfod1	NP_808435.3	371	1	2.84
Ddx39b	NP_001239386.1	1992	7	3.70	Bbs2	NP_080392.1	427	0	0.00
H2-D1	NP_034510.3	528	11	21.96	Mt4	NP_032657.1	235	1	4.48
H2-Q1	XP_011244603.1	315	2	6.69	Mt3	NP_038631.1	820	3	3.86
H2-Q2	NP_001355669.1	335	2	6.29	Mt2	NP_032656.1	405	1	2.60
H2-Q4	NP_001137161.1	276	2	7.64	Mt1	XP_006530815.1	319	0	0.00
H2-Q6	NP_001355670.1	348	3	9.09	Nup93	NP_001344195.1	790	3	4.00
H2-Q7	NP_001185489.1	346	2	6.09	Slc12a3	XP_017168114.1	473	1	2.23
H2-Q9	XP_036016156.1	346	1	3.05	Herpud1	NP_071726.1	452	3	6.99
H2-Q10	NP_034521.1	408	3	7.75	Nlrc5	NP_001028379.2	608	1	1.73
Pou5f1	NP_038661.2	2190	7	3.37	Cpne2	NP_705727.1	128	1	8.23
Tcf19	XP_030105298.1	550	11	21.08	Psme3ip1	NP_001350385.1	248	2	8.50
Cchcr1	XP_011244747.1	225	6	28.10	Rspry1	NP_001346022.1	285	2	7.40
Psors1c2	XP_036016600.1	200	5	26.35	Arl2bp	NP_077153.1	195	3	16.21
Cdsn	NP_001342204.1	375	7	19.67	Pllp	NP_080661.1	338	0	0.00
2300002M23F	NP_780357.1	138	3	22.91	Ccl22	NP_033163.1	553	2	3.81
Muc21	NP_001231583.1	109	0	0.00	Cx3cl1	NP_033168.2	1037	2	2.03
Mucl3	NP_001028538.1	38	3	83.20	Ccl17	NP_035462.2	534	2	3.95
Sfta2	NP_001156666.1	100	7	73.77	Ciapin1	NP_598902.1	467	0	0.00
Vars2	NP_780346.3	941	12	13.44	Coq9	NP_080728.1	464	0	0.00
Gtf2h4	XP_006523731.1	684	10	15.41	Polr2c	NP_033116.2	1949	1	0.54
Ddr1	NP_766550.1	454	1	2.32	Dok4	NP_444476.1	330	0	0.00
Ier3	NP_598423.1	494	9	19.20	Ccdc102a	NP_001028705.2	169	0	0.00
Flot1	XP_006523719.1	728	1	1.45	Adgrg5	XP_017168373.1	306	2	6.89
Tubb5	NP_035785.1	1930	9	4.91	Adgrg1	XP_006530743.1	673	3	4.70
Mdc1	NP_001010833.2	1152	6	5.49	Adgrg3	NP_766624.3	492	2	4.28
Nrm	NP_598883.1	216	10	48.79	Drc7	NP_001036180.2	230	1	4.58
Ppp1r18	NP_001365818.1	289	8	29.17	Katnb1	XP_006531473.1	305	3	10.37
Dhx16	XP_006524936.1	1295	14	11.39	Kifc3	XP_006530786.1	1101	2	1.91
2310061I04Ril	NP_001028802.1	38	6	166.41	Cngb1	NP_001182342.1	514	2	4.10
Atat1	NP_001334307.1	236	3	13.40	Tepp	XP_036010209.1	90	1	11.71
Mrps18b	NP_001334309.1	564	9	16.82	Zfp319	NP_077787.3	171	1	6.16
Ppp1r10	NP_001344669.1	325	9	29.18	Usb1	XP_017167985.1	218	0	0.00
Abcf1	NP_038882.1	964	7	7.65	Mmp15	NP_032635.1	591	2	3.57
Prr3	XP_030105982.1	107	10	98.50	Cfap20	XP_036009655.1	299	1	3.52

Gnl1	XP_036016217.1	937	8	9.00	Csnk2a2	XP_006530704.1	2220	1	0.47
H2-T24	NP_032233.2	126	5	41.82	Ccdc113	NP_766502.1	200	3	15.81
H2-T23	NP_034528.1	355	7	20.78	Prss54	XP_006531428.1	278	0	0.00
H2-T22	NP_001334311.1	175	5	30.11	Gins3	NP_084474.1	435	4	9.69
Gm6034	NP_001030081.2	38	4	110.94	Ndrg4	NP_001359350.1	987	4	4.27
Gm11127	NP_001186896.1	174	10	60.57	Setd6	XP_030099571.1	286	2	7.37
H2-T-ps	NP_001257934.1	348	4	12.11	Cnot1	NP_001192155.1	1329	5	3.97
Gm7030	NP_001170938.1	194	13	70.62	Sap18b	NP_001135913.1	432	0	0.00
Gm8909	XP_030105856.1	174	14	84.80	Slc38a7	NP_766346.1	316	3	10.01
H2-T3	NP_032234.3	274	4	15.39	Got2	NP_034455.1	1271	0	0.00
Rpp21	NP_080584.1	374	2	5.64	Cdh8	NP_001272842.1	854	9	11.11
Trim39	NP_077788.2	768	2	2.74	Cdh11	NP_033996.4	769	3	4.11
H2-M10.2	NP_808591.1	86	6	73.53	Cdh5	NP_033998.2	1369	2	1.54
H2-M10.1	NP_001347427.1	399	3	7.92	Bean1	NP_001135394.1	133	8	63.39
H2-M10.3	NP_963902.1	105	5	50.19	Tk2	NP_066356.3	375	8	22.48
H2-M10.4	NP_808302.1	117	3	27.02	Cklf	XP_030099686.1	126	5	41.82
H2-M11	NP_808303.1	199	2	10.59	Cmtm2a	NP_081298.1	235	10	44.85
H2-M9	NP_001355246.1	777	2	2.71	Cmtm1	NP_871790.2	114	2	18.49
H2-M1	XP_011244702.1	216	1	4.88	Cmtm2b	NP_082800.1	253	8	33.32
H2-M10.5	NP_808305.2	94	2	22.42	Cmtm3	NP_077179.1	299	7	24.67
H2-M10.6	NP_963905.1	86	2	24.51	Cmtm4	NP_705810.2	214	7	34.47
Trim26	NP_001020770.2	518	12	24.41	Dync1li2	XP_006530945.1	592	7	12.46
Trim15	NP_001171343.1	432	5	12.20	Terb1	XP_011246706.1	120	7	61.48
Trim10	XP_017172848.1	436	6	14.50	Nae1	XP_006530947.1	1552	1	0.68
Trim40	XP_017172847.1	410	7	17.99	Car7	NP_444300.1	243	0	0.00
Trim31	NP_666189.1	500	5	10.54	Pdp2	XP_006531223.1	918	0	0.00
Rnf39	NP_001093102.1	400	6	15.81	Cdh16	NP_001239557.1	436	0	0.00
Ppp1r11	NP_083908.1	491	8	17.17	Rrad	NP_062636.2	1676	0	0.00
Znrd1	NP_001342351.1	939	9	10.10	Ciao2b	NP_081029.1	516	1	2.04
Znrd1as	NP_083878.1	24	3	131.74	Ces2a	NP_598721.1	723	3	4.37
2410137M14R	NP_084023.1	42	0	0.00	Ces2b	NP_937814.1	210	0	0.00
H2-M5	NP_001108547.1	191	1	5.52	Ces2c	NP_663578.1	728	2	2.90
Zfp57	XP_036016475.1	508	3	6.22	Ces2e	NP_001157228.1	751	3	4.21
Mog	NP_034944.2	795	9	11.93	Ces2f	NP_001073334.2	180	0	0.00
Gabbr1	NP_062312.3	1099	2	1.92	Ces2g	NP_932116.2	295	1	3.57
Olfr90	NP_666688.2	62	1	17.00	Ces2h	NP_001258974.1	547	5	9.63
Olfr91	XP_017172966.1	37	0	0.00	Ces3a	XP_011246729.1	815	6	7.76
Olfr92	NP_666667.2	62	0	0.00	Ces3b	XP_006530717.1	718	3	4.40
Olfr93	NP_001011813.1	34	0	0.00	Ces4a	NP_666325.2	302	6	20.94
Ubd	NP_075626.1	3081	3	1.03	Cbfb	NP_071704.3	386	0	0.00
Olfr94	NP_001011518.1	11	0	0.00	D230025D16R	NP_001366027.1	190	0	0.00
Olfr95	NP_666724.1	38	0	0.00	B3gnt9	NP_849210.1	84	0	0.00
Olfr96	NP_666725.1	38	0	0.00	Tradd	NP_001028333.1	544	1	1.94
Olfr97	NP_666723.1	26	0	0.00	Fbxl8	NP_056636.2	320	1	3.29
Olfr98	NP_666721.1	14	0	0.00	Hsf4	NP_036069.1	734	1	1.44
H2-M3	NP_038847.1	439	2	4.80	Nol3	NP_084428.1	287	2	7.34
Olfr99	NP_666726.1	37	0	0.00	4931428F04R	XP_017168480.1	2	0	0.00
Olfr101	NP_667045.1	40	0	0.00	Exoc3l	NP_808456.1	463	6	13.66
Olfr102	NP_001011721.2	40	0	0.00	E2f4	NP_683754.1	1204	3	2.63
Olfr103	NP_667044.1	132	0	0.00	Elmo3	XP_030099358.1	392	5	13.44
Olfr107	NP_666722.2	33	0	0.00	Lrrc29	NP_001355338.1	479	6	13.20
Olfr108	NP_666676.1	14	0	0.00	Tmem208	NP_001365895.1	388	0	0.00
Olfr109	NP_667046.1	42	0	0.00	Fhod1	NP_808367.2	566	4	7.45
H2-M2	NP_001334316.1	242	1	4.35	Slc9a5	XP_006531130.1	464	0	0.00
Olfr110	XP_036016506.1	138	1	7.64	Plekhg4	NP_001351335.1	470	8	17.94
Olfr111	XP_036016580.1	99	1	10.65	Kctd19	NP_001288102.1	1728	7	4.27
Olfr112	NP_001013593.2	2	0	0.00	Lrrc36	NP_001164259.1	67	0	0.00
Olfr113	NP_666401.1	11	0	0.00	Tppp3	NP_080757.1	321	2	6.57
Olfr114	NP_666399.1	36	0	0.00	Zdhhc1	NP_001344181.1	168	4	25.09
Olfr115	NP_001011753.2	31	0	0.00	Hsd11b2	NP_032315.2	471	8	17.90
Olfr116	NP_666843.1	38	0	0.00	Atp6v0d1	NP_038505.2	792	7	9.31
Olfr117	NP_997038.2	38	0	0.00	Agrp	NP_001258735.1	458	2	4.60
Olfr118	NP_998886.2	21	0	0.00	Ripor1	XP_011246833.2	146	1	7.22
Olfr119	NP_001011830.2	100	0	0.00	Ctcf	XP_030099140.1	1508	2	1.40
Olfr120	NP_666842.1	27	0	0.00	Carmil2	XP_017168228.1	188	1	5.61
Olfr121	NP_666840.2	65	0	0.00	Acd	NP_001012656.1	249	5	21.16

Olfr122	NP_666400.3	57	0	0.00	Pard6a	NP_001040901.1	662	3	4.78
Olfr123	NP_666841.1	62	0	0.00	Enkd1	XP_036009598.1	141	0	0.00
Olfr124	NP_667273.1	196	0	0.00	4933405L10R	NP_081931.1	0	0	#DIV/0!
Olfr125	NP_666402.2	87	0	0.00	Gfod2	XP_017168447.1	284	4	14.84
Olfr126	NP_667101.1	33	0	0.00	Ranbp10	XP_030099673.1	515	5	10.23
Olfr127	NP_666489.1	15	0	0.00	Tsnaxip1	NP_077765.1	101	1	10.43
Olfr128	NP_996552.1	157	0	0.00	Cenpt	NP_796124.1	449	3	7.04
Olfr761	NP_001011829.1	27	0	0.00	Thap11	NP_067488.1	242	1	4.35
Olfr129	XP_030105671.1	65	0	0.00	Nutf2	NP_001344158.1	718	2	2.94
Olfr130	NP_666698.1	243	0	0.00	Edc4	XP_006530964.1	345	0	0.00
Olfr131	NP_667078.1	10	0	0.00	Nrn1l	NP_778189.1	132	0	0.00
Olfr132	NP_001005481.1	23	0	0.00	Pskh1	NP_775608.1	575	3	5.50
Olfr133	NP_667042.1	18	0	0.00	Ctrl	NP_075671.1	704	2	2.99
Olfr134	NP_667043.1	13	0	0.00	Psmb10	NP_038668.2	1163	9	8.16
Olfr135	NP_666444.1	64	0	0.00	Lcat	NP_032516.2	1042	5	5.06
Olfr138	NP_570938.1	23	0	0.00	Slc12a4	XP_006530855.1	416	3	7.60
Olfr137	NP_666699.1	171	1	6.16	Dpep3	NP_082236.1	230	0	0.00
Olfr136	NP_667018.1	29	0	0.00	Dpep2	XP_006531144.1	295	1	3.57
Esp36	NP_001171058.1	39	1	27.02	Ddx28	NP_082314.2	1625	7	4.54
Esp34	NP_001171056.1	47	1	22.42	Dus2	NP_079794.1	529	7	13.95
Esp31	NP_001171057.1	14	0	0.00	Nfatc3	NP_001355726.1	571	4	7.38
Esp24	NP_001242979.1	54	0	0.00	Esrp2	XP_006531555.1	459	3	6.89
Esp23	NP_001171053.1	0	0	#DIV/0!	Pla2g15	NP_598553.1	599	8	14.08
Esp18	NP_001231692.1	46	0	0.00	Slc7a6	XP_006531199.1	406	5	12.98
Esp16	NP_001242906.1	1	1	1053.90	Slc7a6os	NP_001007568.1	283	1	3.72
Esp15	NP_001231580.1	93	6	67.99	Prmt7	NP_663379.1	792	6	7.98
Esp38	NP_001242980.1	868	2	2.43	Smpd3	NP_067466.1	339	0	0.00
Pgk2	NP_112467.2	1854	3	1.71	Zfp90	NP_001355257.1	360	7	20.49
Crisp3	NP_033769.1	486	1	2.17	Cdh3	NP_001032898.1	929	3	3.40
Crisp1	NP_033768.3	506	1	2.08	Cdh1	NP_033994.1	2737	2	0.77
Esp8	NP_001171055.1	56	1	18.82	Tango6	NP_766625.1	212	2	9.94
Esp6	NP_001171000.1	12	0	0.00	Has3	NP_001317977.1	576	0	0.00
Esp5	NP_001274123.1	12	0	0.00	Chtf8	NP_001365305.1	71	0	0.00
Esp4	NP_001171054.1	56	1	18.82	Utp4	NP_001345911.1	891	4	4.73
Esp3	NP_001238845.1	48	1	21.96	Sntb2	NP_001355245.1	360	2	5.86
Esp1	NP_001033589.1	104	1	10.13	Vps4a	NP_569053.1	527	1	2.00
Crisp2	NP_001191000.1	499	1	2.11	Pdf	NP_080789.2	576	1	1.83
Rhag	NP_035399.1	373	2	5.65	Cog8	NP_631975.3	380	2	5.55
9130008F23Ri	NP_082110.1	104	1	10.13	Nip7	NP_079667.2	1249	3	2.53
Glyatl3	NP_001138532.1	88	0	0.00	Tmed6	NP_079734.1	219	5	24.06
Cenpq	NP_114069.2	284	1	3.71	Terf2	XP_036009763.1	412	3	7.67
Mmut	NP_032676.2	654	0	0.00	Cyb5b	NP_079834.2	1042	1	1.01
Ptchd4	XP_036016615.1	200	0	0.00	Nfat5	NP_001273189.1	851	5	6.19
Opn5	NP_861418.2	199	1	5.30	Nqo1	XP_036009703.1	871	2	2.42
Adgrf4	XP_017173214.1	284	5	18.55	Nob1	NP_080553.1	790	5	6.67
Adgrf2	XP_017173054.1	290	3	10.90	Wwp2	NP_080106.1	1255	5	4.20
Cd2ap	XP_011244565.1	754	2	2.80	Psmd7	NP_034947.1	1343	4	3.14
Tnfrsf21	NP_848704.1	393	3	8.05	Zfhx3	NP_031522.2	613	7	12.03
Adgrf1	XP_006525183.1	352	5	14.97	Pmfbp1	XP_030099553.1	105	6	60.22
Adgrf5	XP_006524187.1	503	5	10.48	Dhx38	XP_006531324.1	1145	11	10.12
Mep1a	NP_032611.2	371	6	17.04	Txnl4b	NP_783577.2	766	11	15.13
Ankrd66	NP_001241882.1	890	3	3.55	Hp	NP_059066.1	1135	1	0.93
Pla2g7	NP_038765.2	512	7	14.41	Dhodh	NP_064430.1	1082	8	7.79
Tdrd6	NP_001344914.1	975	4	4.32	Pkd1l3	NP_001273383.1	267	1	3.95
Slc25a27	NP_001344051.1	233	1	4.52	Ist1	XP_036010193.1	594	11	19.52
Cyp39a1	NP_061375.1	445	3	7.10	Zfp821	NP_001350311.1	222	11	52.22
Rcan2	NP_001344671.1	522	2	4.04	Atxn1l	NP_001074399.1	165	10	63.87
Enpp5	NP_114392.1	425	5	12.40	Ap1g1	NP_033807.2	834	8	10.11
Enpp4	XP_017172927.1	281	6	22.50	Phlpp2	NP_001116066.2	1918	6	3.30
Clic5	XP_006524198.1	627	5	8.40	Marveld3	NP_997612.2	181	7	40.76
Runx2	NP_001139510.1	1483	6	4.26	Tat	XP_030099366.1	1084	4	3.89
Supt3	XP_006523540.1	599	8	14.08	Chst4	NP_036128.3	146	1	7.22
Cdc5l	NP_690023.1	3263	11	3.55	Zfp612	XP_006530972.1	244	0	0.00
Spats1	NP_001344761.1	54	7	136.62	Tle7	NP_001357768.1	317	0	0.00
Aars2	NP_941010.2	1099	7	6.71	Calb2	NP_001355223.1	1997	2	1.06
Tcte1	NP_038716.2	590	9	16.08	Cmtr2	NP_666327.2	159	0	0.00

Tmem151b	NP_001013771.1	449	0	0.00	Hydin	XP_030099412.1	400	1	2.63
Nfkbie	NP_001291885.1	1468	3	2.15	Vac14	NP_666328.2	401	5	13.14
Slc35b2	NP_001344034.1	588	3	5.38	Mtss2	XP_006531074.1	405	2	5.20
Hsp90ab1	NP_032328.2	4032	7	1.83	Il34	XP_006531548.1	305	1	3.46
Slc29a1	NP_075018.1	530	1	1.99	Sf3b3	NP_598714.1	1245	5	4.23
Mymx	NP_001170941.1	110	0	0.00	Cog4	NP_598734.1	431	6	14.67
Capn11	NP_001013789.1	245	5	21.51	Fcsk	NP_758487.2	261	5	20.19
Tmem63b	XP_030105594.1	279	5	18.89	St3gal2	XP_017168113.1	286	6	22.11
Mrpl14	NP_081008.1	506	1	2.08	Ddx19a	NP_031942.2	685	2	3.08
1600014C23R	NP_082440.1	0	0	#DIV/0!	Ddx19b	NP_001177715.1	592	2	3.56
Vegfa	NP_001303970.1	2360	3	1.34	Aars	NP_666329.2	1326	9	7.15
Mrps18a	NP_081044.1	616	3	5.13	Exosc6	NP_082550.1	1326	9	7.15
Rsph9	XP_006525138.1	322	1	3.27	Clec18a	XP_011246720.1	264	0	0.00
Mad2l1bp	NP_079925.2	221	1	4.77	Pdpr	NP_938050.1	419	2	5.03
Gtpbp2	XP_006524714.1	315	2	6.69	Glg1	NP_033175.1	283	1	3.72
Polh	NP_109640.1	692	1	1.52	Rfwd3	NP_666330.2	267	1	3.95
Xpo5	NP_082474.1	2080	3	1.52	Mlkl	XP_036010235.1	1157	2	1.82
Polr1c	NP_033111.2	1893	1	0.56	Fa2h	NP_835187.2	1750	2	1.20
Yipf3	XP_036016527.1	222	2	9.49	Wdr59	XP_006531146.1	1569	2	1.34
Lrrc73	NP_001104612.1	65	0	0.00	Znrf1	NP_001350417.1	454	0	0.00
Tjap1	NP_083027.1	263	0	0.00	Ldhd	XP_036010057.1	470	1	2.24
Dlk2	NP_001272942.1	310	0	0.00	Zfp1	XP_030099278.1	265	1	3.98
Abcc10	NP_733780.1	385	2	5.47	Ctrb1	NP_079859.2	387	2	5.45
Zfp318	NP_067321.2	136	0	0.00	Bcar1	NP_034084.2	703	4	6.00
Crip3	XP_006523552.1	524	4	8.05	Cfdp1	NP_001355267.1	572	2	3.68
Slc22a7	NP_659105.2	736	3	4.30	Tmem170	XP_006531354.2	70	2	30.11
Ttbk1	XP_036016144.1	1124	5	4.69	Chst5	NP_064334.1	152	6	41.60
Dnph1	NP_997044.2	194	2	10.86	Tmem231	NP_001028493.1	206	3	15.35
Cul9	XP_006525199.1	376	1	2.80	Gabarapl2	NP_080969.2	985	4	4.28
Srf	XP_006524020.1	1559	1	0.68	Adat1	XP_006531138.1	229	5	23.01
Ptk7	XP_006524987.1	2146	3	1.47	Kars	NP_444322.1	1537	2	1.37
Klc4	XP_036016710.1	988	5	5.33	Terf2ip	NP_065609.2	328	3	9.64
Mrpl2	XP_017172985.1	2050	0	0.00	Cntnap4	NP_569724.2	682	5	7.73
Cul7	NP_079887.3	560	1	1.88	Mon1b	NP_001041608.1	469	2	4.49
Rrp36	NP_659106.2	442	3	7.15	Syce1l	NP_001041610.1	68	1	15.50
Klhdc3	NP_082186.2	248	3	12.75	Adamts18	NP_766054.2	426	6	14.84
Mea1	NP_001264238.1	262	4	16.09	Nudt7	NP_077757.2	726	6	8.71
Ppp2r5d	NP_033384.2	1094	6	5.78	Vat1l	NP_766604.2	680	5	7.75
Pex6	NP_663463.1	934	2	2.26	Clec3a	NP_001007224.1	350	6	18.07
Gnmt	NP_001351819.1	919	2	2.29	Wwox	NP_062519.2	686	6	9.22
Cnpy3	NP_082341.1	205	3	15.42	Maf	NP_001020748.2	1218	2	1.73
Ptcra	NP_035325.1	352	0	0.00	Dynlrb2	NP_083573.1	470	5	11.21
2310039H08R	NP_080242.1	66	0	0.00	Cdyl2	NP_083717.1	2136	5	2.47
Rpl7l1	NP_079709.2	1255	3	2.52	Cmc2	NP_081120.2	207	5	25.46
Bicral	XP_011244648.3	26	0	0.00	Cenpn	NP_082407.1	712	2	2.96
Tbcc	NP_848472.2	221	0	0.00	Atmin	NP_808368.3	351	6	18.02
Prph2	NP_032964.1	416	2	5.07	1700030J22R	NP_001344182.1	24	4	175.65
Ubr2	NP_001170845.1	1293	0	0.00	Gcsh	NP_080848.1	586	8	14.39
Mrps10	NP_001139684.1	1192	3	2.65	Pkd1l2	XP_036010263.1	222	3	14.24
Guca1b	NP_666191.1	976	1	1.08	Bco1	NP_067461.2	311	4	13.55
Guca1a	NP_032215.2	981	1	1.07	Gan	NP_001074620.1	661	0	0.00
1700001C19R	XP_030105987.1	49	0	0.00	Cmip	NP_001156734.1	311	5	16.94
AI661453	NP_663464.2	107	0	0.00	Plcg2	NP_758489.1	1088	1	0.97
Taf8	NP_001343219.1	492	1	2.14	Sdr42e1	NP_001344201.1	685	4	6.15
Ccnd3	NP_001075104.1	938	3	3.37	Hsd17b2	NP_032316.2	869	3	3.64
Bysl	NP_058555.3	1109	7	6.65	Mphosph6	NP_001344162.1	336	1	3.14
Med20	NP_064432.1	228	6	27.73	Cdh13	NP_062681.2	805	2	2.62
Usp49	NP_940813.1	468	6	13.51	Hsbp1	NP_077181.1	296	4	14.24
Tomm6	NP_079641.1	240	0	0.00	Mlycd	XP_017168411.1	578	6	10.94
Prickle4	NP_001277266.1	538	6	11.75	Osgin1	NP_082226.1	488	5	10.80
Frs3	NP_659188.1	330	6	19.16	Necab2	NP_473436.1	326	6	19.40
Pgc	NP_080249.2	854	4	4.94	Slc38a8	NP_001009950.1	168	7	43.91
Tfeb	XP_006524067.1	695	3	4.55	Mbtps1	XP_036010086.1	832	1	1.27
Mdfi	NP_034913.2	275	0	0.00	Hsdl1	NP_001365946.1	479	6	13.20
Foxp4	NP_083043.2	722	8	11.68	Dnaaf1	NP_080924.1	83	0	0.00
1700122O11R	NP_083965.1	18	0	0.00	Taf1c	NP_067416.2	244	2	8.64

9830107B12R	NP_001171368.1	62	6	101.99	Adad2	NP_083704.1	164	4	25.70
A530064D06R	NP_848911.2	122	6	51.83	Kcng4	NP_080010.2	783	1	1.35
Trem1	XP_006524792.1	448	9	21.17	Wfdc1	NP_075884.1	204	1	5.17
Trem3	NP_067382.1	196	8	43.02	Atp2c2	NP_081198.1	982	1	1.07
Treml4	XP_006524211.1	300	7	24.59	Meak7	XP_030099677.1	111	0	0.00
Treml2	NP_001028577.2	188	9	50.45	Cotl1	NP_082347.1	821	3	3.85
B430306N03R	XP_006524457.1	111	7	66.46	Klhl36	NP_666331.1	169	1	6.24
Trem2	NP_001259007.1	615	10	17.14	Usp10	NP_033488.1	1064	3	2.97
Treml1	NP_082039.1	122	7	60.47	Crispld2	XP_006531571.1	336	1	3.14
Nfya	NP_001334331.1	802	7	9.20	Zdhhc7	NP_598728.1	225	4	18.74
Oard1	NP_997102.2	103	0	0.00	6430548M08I	NP_758490.3	143	0	0.00
Apobec2	NP_033824.1	387	0	0.00	Cibar2	NP_001029152.2	51	1	20.66
Tspo2	XP_017173120.1	162	0	0.00	Gse1	XP_006531208.3	279	9	34.00
Unc5cl	XP_017173206.1	113	1	9.33	Gins2	NP_849187.1	703	10	14.99
Lrfn2	NP_081728.2	1274	5	4.14	Gm27021	NP_001295378.1	151	3	20.94
Mocs1	NP_064426.2	437	2	4.82	1190005I06Ri	NP_932105.2	64	7	115.27
Daam2	XP_036016720.1	1171	4	3.60	Emc8	NP_035056.1	390	12	32.43
Kif6	NP_796026.2	1188	2	1.77	Cox4i1	NP_034071.2	1267	11	9.15
Rftn1	NP_852062.1	234	1	4.50	Irf8	NP_032346.1	1174	9	8.08
Dazl	NP_001264792.1	1835	5	2.87	Foxf1	NP_034556.2	897	11	12.92
Plcl2	NP_038908.2	549	0	0.00	Mthfsd	NP_001359318.1	260	8	32.43
Tbc1d5	NP_082438.3	761	4	5.54	Foxc2	NP_038547.2	1053	11	11.01
Satb1	XP_036016317.1	688	4	6.13	Foxl1	NP_032050.2	739	12	17.11
Kcnh8	XP_036016335.1	394	5	13.37	1700018B08R	XP_006531538.1	107	5	49.25
Efhb	XP_006524046.1	103	1	10.23	Fbxo31	XP_006531541.1	433	6	14.60
Rab5a	NP_080163.1	1867	2	1.13	Map1lc3b	NP_001351287.1	1354	3	2.34
Pp2d1	NP_775625.1	888	1	1.19	Zcchc14	XP_006530730.1	723	12	17.49
Kat2b	NP_064389.2	2479	4	1.70	Jph3	NP_065630.1	876	7	8.42
Sgo1	NP_082508.1	855	1	1.23	Klhdc4	NP_663580.2	302	4	13.96
Sult1c2	NP_081211.3	577	1	1.83	Slc7a5	NP_035534.2	636	3	4.97
Sult1c1	NP_061221.2	451	0	0.00	Car5a	NP_031634.2	253	3	12.50
Slc5a7	XP_036016630.1	534	2	3.95	Banp	XP_017168388.1	270	4	15.61
St6gal2	NP_001334332.1	413	1	2.55	Zfp469	NP_001349812.1	288	0	0.00
Vmn2r118	NP_001372145.1	59	2	35.73	Zfpm1	XP_006530914.1	548	2	3.85
Pot1b	XP_030105924.1	245	0	0.00	Trhr2	XP_030099174.1	248	0	0.00
Adgre4	NP_631877.2	413	0	0.00	Zc3h18	XP_006531532.1	273	0	0.00
Zfp119a	NP_653129.1	213	1	4.95	Il17c	XP_036009870.1	239	0	0.00
Zfp959	NP_663465.1	271	1	3.89	Cyba	NP_001288213.1	743	3	4.26
Zfp119b	XP_011244758.1	132	1	7.98	Mvd	NP_619597.2	461	4	9.14
Ebi3	NP_056581.1	254	1	4.15	Snai3	XP_006531137.1	280	3	11.29
Yju2	NP_082657.1	533	1	1.98	Rnf166	NP_001028314.1	110	5	47.90
Shd	NP_001152995.1	201	0	0.00	Ctu2	NP_722470.2	395	1	2.67
Fsd1	NP_899001.1	416	0	0.00	Piezo1	NP_001032375.1	331	0	0.00
Stap2	XP_036016146.1	181	0	0.00	Cdt1	NP_080290.3	1126	1	0.94
Mpnd	NP_080806.4	971	1	1.09	Aprt	NP_033828.2	1050	6	6.02
Sh3gl1	NP_038692.1	558	1	1.89	Galns	NP_001180574.1	355	4	11.87
Chaf1a	NP_038761.1	985	1	1.07	Trappc2l	NP_067477.1	373	0	0.00
Ubxn6	NP_077752.1	386	1	2.73	Pabpn1l	NP_001007463.1	665	0	0.00
Hdgfl2	NP_001291713.1	1048	1	1.01	Cbfa2t3	NP_033954.2	458	1	2.30
Plin4	NP_065593.2	471	3	6.71	Acsf3	NP_659181.2	760	2	2.77
Plin5	XP_006524868.1	361	3	8.76	Cdh15	NP_031688.2	514	4	8.20
Lrg1	NP_084072.1	1155	2	1.82	Ankrd11	NP_001074848.2	383	2	5.50
Sema6b	NP_001123928.1	353	0	0.00	Spg7	NP_694816.3	1196	6	5.29
Tnfaip8l1	XP_030105845.1	214	0	0.00	Rpl13	NP_058018.2	1025	1	1.03
Mydgf	NP_543027.1	221	0	0.00	Cpne7	NP_733785.1	200	8	42.16
Dpp9	NP_001347213.1	221	0	0.00	Sult5a1	NP_065589.2	263	0	0.00
Fem1a	NP_034322.3	1044	0	0.00	Dpep1	XP_017168047.1	492	9	19.28
Ticam1	NP_778154.1	335	0	0.00	Chmp1a	NP_663581.1	485	10	21.73
Plin3	NP_080112.1	427	2	4.94	Spata33	NP_796253.2	83	12	152.37
Arrdc5	NP_084075.1	373	1	2.83	Cdk10	NP_919428.1	628	13	21.82
Uhrf1	NP_035061.3	1096	2	1.92	Spata2l	NP_001344229.1	79	14	186.77
Kdm4b	XP_030105461.1	1521	3	2.08	Vps9d1	XP_006531453.1	1034	1	1.02
Ptprs	NP_001239384.1	1006	5	5.24	Zfp276	XP_006531290.1	233	16	72.37
Znrf4	NP_035613.2	405	2	5.20	Fanca	XP_006530724.1	586	14	25.18
Safb2	XP_036016447.1	564	5	9.34	Spire2	NP_758491.1	290	16	58.15
Safb	NP_001361548.1	627	4	6.72	Tcf25	XP_006531360.1	353	14	41.80

Micos13	NP_694792.1	269	1	3.92	Mc1r	NP_032585.2	460	13	29.78
Rpl36	NP_061200.2	950	4	4.44	Tubb3	NP_075768.1	2683	13	5.11
Lonp1	NP_083058.2	921	4	4.58	Def8	XP_036009874.1	163	12	77.59
Catsperd	XP_006523493.1	81	2	26.02	Afg3l1	NP_473411.2	919	11	12.61
Ranbp3	NP_001239395.1	292	0	0.00	Dbndd1	NP_001164447.2	157	10	67.13
Vmac	NP_849257.1	51	0	0.00	Gas8	NP_061343.2	350	8	24.09
Ndufa11	NP_081520.1	617	4	6.83	Rhou	NP_598716.1	1794	2	1.17
Nrtn	XP_006523864.1	277	2	7.61	Rab4a	NP_033029.2	1653	2	1.28
Dus3l	XP_036016453.1	538	5	9.79	Ccsap	NP_082812.1	139	0	0.00
Prr22	NP_001182602.1	14	3	225.84	Acta1	NP_033736.1	2888	2	0.73
Rfx2	NP_033082.1	768	4	5.49	Nup133	NP_758492.2	909	0	0.00
Acsbg2	NP_001034203.1	620	3	5.10	Abcb10	NP_062425.1	543	3	5.82
Acsbg3	NP_084417.1	284	1	3.71	Taf5l	NP_001344244.1	1139	3	2.78
Mllt1	XP_006524841.1	1042	3	3.03	Urb2	NP_001355343.1	212	1	4.97
Acer1	NP_783858.1	331	2	6.37	Galnt2	NP_644678.2	346	2	6.09
Clpp	NP_059089.1	1412	3	2.24	Pgbd5	XP_030099239.1	601	2	3.51
Alkbh7	NP_079814.1	282	4	14.95	Cog2	NP_084022.2	618	4	6.82
Pspn	NP_032980.2	177	2	11.91	Agt	XP_030099108.1	1775	2	1.19
Gtf2f1	NP_598562.1	841	4	5.01	Capn9	XP_006531467.1	378	0	0.00
Khsrp	NP_034743.3	764	2	2.76	2310022B05R	NP_780358.3	46	0	0.00
Slc25a41	NP_780542.1	194	4	21.73	Ttc13	NP_001344285.1	404	1	2.61
Slc25a23	NP_001365998.1	447	0	0.00	Arv1	NP_001355301.1	223	1	4.73
Crb3	NP_001334337.1	249	0	0.00	Fam89a	NP_001074589.1	49	1	21.51
Dennd1c	XP_006524962.1	321	4	13.13	Trim67	XP_006531202.1	302	2	6.98
Tubb4a	NP_033477.2	2433	2	0.87	2810004N23R	NP_079891.2	139	0	0.00
Tnfsf9	NP_033430.1	395	3	8.00	Gnpat	NP_034452.3	470	1	2.24
Cd70	NP_035747.1	432	3	7.32	Exoc8	NP_932771.1	662	0	0.00
Tnfsf14	NP_062291.1	404	4	10.43	Sprtn	NP_001104611.1	447	0	0.00
C3	NP_033908.2	1423	2	1.48	Egln1	NP_444437.2	734	3	4.31
Trip10	XP_006523464.1	652	5	8.08	Tsnax	NP_058605.1	349	2	6.04
Gpr108	NP_001295001.1	340	1	3.10	Disc1	NP_777279.2	714	2	2.95
Vav1	NP_035821.3	1368	8	6.16	Sipa1l2	XP_011246691.1	470	1	2.24
Adgre1	NP_001342651.1	1298	4	3.25	Map10	NP_083184.1	110	0	0.00
Vmn2r120	NP_001372110.1	52	0	0.00	Ntpcr	NP_079912.1	241	0	0.00
Cntnap5c	XP_006524804.1	402	0	0.00	Pcnx2	NP_780770.2	114	0	0.00
Pdzph1	NP_081277.1	491	1	2.15	Map3k21	NP_663583.2	979	1	1.08
Nudt12	NP_001355322.1	1490	3	2.12	Kcnk1	XP_006530779.1	563	1	1.87
Efna5	NP_034239.1	755	2	2.79	Slc35f3	XP_030099242.1	240	1	4.39
Fbxl17	NP_056609.1	535	3	5.91	Coa6	NP_778152.1	411	0	0.00
Fer	XP_036016207.1	1166	4	3.62	Tarbp1	NP_001153379.1	520	2	4.27
Pja2	NP_659108.1	928	2	2.27	Irf2bp2	NP_001158070.1	390	2	6.01
Man2a1	NP_032575.2	404	1	2.61	Tomm20	NP_077176.1	1312	2	1.89
4930583I09Ri	NP_084307.1	0	0	#DIV/0!	Rbm34	NP_001361729.1	1510	1	0.87
Tmem232	XP_030105731.1	85	0	0.00	Pard3	XP_006531583.1	1096	3	3.85
Vapa	NP_038961.2	1472	9	6.44	Nrp1	XP_030099209.1	828	1	1.82
Txndc2	NP_001139474.1	1255	8	6.72	Itgb1	NP_034708.1	1760	2	1.84
Rab31	NP_598446.2	1110	7	6.65	Ccdc7a	NP_083337.1	48	0	0.00
Ppp4r1	NP_666193.2	863	7	8.55	Ccdc7b	NP_001355957.1	32	0	0.00
Ralbp1	XP_006523978.1	404	6	15.65	2610044O15R	NP_722475.1	293	0	0.00
Twsg1	NP_075540.1	473	7	15.60	Alkbh8	XP_006509942.1	1109	3	5.70
Ankrd12	XP_030105279.1	327	10	32.23	Cwf19l2	NP_081821.1	653	4	11.74
Ndufv2	NP_082664.1	957	6	6.61	Gucy1a2	NP_001028494.1	919	3	5.73
Washc1	XP_030105881.1	191	0	0.00	Aasdhppt	XP_006509940.1	714	2	4.54
Ddx11	NP_001335221.1	945	0	0.00	Kbtbd3	XP_030100419.1	117	5	64.34
Mtcl1	XP_036016661.1	241	0	0.00	Msantd4	NP_663584.1	81	3	52.04
Rab12	NP_077768.2	2124	2	0.99	Gria4	NP_062665.4	1279	1	1.03
Themis3	NP_083274.1	23	0	0.00	Casp1	NP_033937.2	1606	5	3.86
Ptprm	NP_033010.2	893	9	10.62	Casp4	XP_036010472.1	1003	5	5.84
Lrrc30	NP_001028512.2	595	12	21.26	Casp12	NP_033938.3	747	2	2.97
Lama1	NP_032506.2	725	6	8.72	Pdgfd	XP_011240672.1	477	2	4.42
Arhgap28	XP_036016523.1	285	8	29.58	Ddi1	NP_082218.1	519	1	2.03
L3mbtl4	NP_796252.2	412	4	10.23	Dync2h1	XP_006509890.1	895	1	1.18
Tmem200c	NP_001193590.1	77	2	27.37	Dcun1d5	NP_001344412.1	418	1	2.52
Epb41l3	XP_030105338.1	589	12	21.47	Mmp13	NP_032633.1	1046	10	10.08
Zbtb14	NP_001343211.1	404	14	36.52	Mmp12	NP_032631.3	810	9	11.71
Akain1	XP_006524478.1	0	0	#DIV/0!	Mmp1b	NP_114396.3	395	6	16.01

Dlgap1	XP_006524231.1	1202	11	9.64	Mmp3	NP_034939.1	1184	11	9.79
Tgif1	NP_001157547.1	633	6	9.99	Mmp1a	NP_114395.1	415	6	15.24
Myl12b	NP_075891.1	1376	12	9.19	Mmp10	NP_062344.1	598	8	14.10
Myl12a	NP_001344749.1	1255	4	3.36	Mmp8	NP_032637.3	775	3	4.08
Myom1	NP_034997.2	288	8	29.28	Mmp27	NP_001297646.1	999	3	3.16
Lpin2	NP_001158357.1	769	10	13.70	Mmp20	NP_038931.1	422	1	2.50
Emilin2	NP_001344266.1	142	6	44.53	Mmp7	NP_034940.2	943	10	11.18
Smchd1	NP_083163.3	618	8	13.64	Tmem123	NP_598500.1	148	4	28.48
Ndc80	NP_075783.2	1030	4	4.09	Birc2	XP_017168600.1	1263	7	5.84
Spdya	NP_083530.1	468	3	6.76	Birc3	XP_006509892.1	1364	7	5.41
Wdr43	NP_783570.1	912	0	0.00	Yap1	NP_033560.1	1697	5	3.11
Togaram2	XP_006524459.2	391	2	5.39	Cfap300	NP_950182.1	75	2	28.10
Pcare	XP_006524250.1	164	0	0.00	Cep126	NP_001038989.1	163	4	25.86
Clip4	XP_030106006.1	1402	5	3.76	Trpc6	XP_011240662.2	615	3	5.14
Alk	NP_031465.2	2088	1	0.50	Pgr	NP_032855.2	1603	9	5.92
Ypel5	NP_001344300.1	397	5	13.27	Arhgap42	NP_082099.1	443	6	14.27
Lbh	NP_084275.3	143	1	7.37	Cntn5	XP_036010845.1	791	4	5.33
Lclat1	XP_036016461.1	422	2	4.99	Jrkl	NP_001028353.1	200	2	10.54
Capn13	NP_001028616.1	402	5	13.11	Ccdc82	XP_017169000.1	138	0	0.00
Galnt14	NP_082140.2	313	1	3.37	Maml2	NP_001013835.2	198	1	5.32
Ehd3	NP_065603.2	722	3	4.38	Mtmr2	XP_036011299.1	416	1	2.53
Xdh	NP_035853.2	1091	0	0.00	Cep57	NP_001297650.1	485	1	2.17
Srd5a2	NP_444418.1	472	5	11.16	Fam76b	XP_011240914.1	531	2	3.97
Memo1	XP_036016724.1	315	5	16.73	Sesn3	NP_084537.2	318	0	0.00
Dpy30	NP_077746.3	975	4	4.32	Endod1	NP_082289.2	289	0	0.00
Spast	NP_001156342.1	1277	4	3.30	Kdm4d	NP_775609.2	1250	1	0.84
Slc30a6	NP_001239407.1	294	4	14.34	Cwc15	NP_075642.1	787	2	2.68
Nlrc4	XP_017172980.1	588	2	3.58	Amotl1	XP_006510730.1	314	1	3.36
Yipf4	NP_080693.2	272	0	0.00	Piwil4	NP_001355761.1	543	0	0.00
Birc6	XP_006523581.1	2462	3	1.28	Fut4	NP_034372.1	793	0	0.00
Ttc27	NP_690030.3	637	4	6.62	1700012B09R	NP_083582.2	32	0	0.00
Ltbp1	NP_064303.2	740	1	1.42	Ankrd49	NP_001335189.1	940	1	1.12
Rasgrp3	NP_001347032.1	739	3	4.28	Mre11a	XP_006510111.1	1180	0	0.00
Fam98a	NP_598508.2	288	1	3.66	Gpr83	XP_030099918.1	482	0	0.00
Crim1	NP_056615.1	531	2	3.97	Izumo1r	XP_017168992.1	337	0	0.00
Fez2	NP_001272878.1	351	1	3.00	Panx1	NP_062355.2	377	0	0.00
Vit	NP_083089.1	253	1	4.17	Hephl1	NP_001158269.1	425	0	0.00
Strn	NP_035630.2	736	4	5.73	Vstm5	NP_081231.1	35	0	0.00
Heatr5b	NP_001074648.1	273	1	3.86	Med17	NP_001351462.1	469	0	0.00
Gpatch11	NP_857632.4	145	2	14.54	4931406C07R	NP_001346187.1	221	0	0.00
Eif2ak2	XP_006523926.1	977	0	0.00	Taf1d	XP_036011262.1	428	1	2.46
Sult6b1	NP_001157097.1	147	1	7.17	Cep295	XP_011240850.1	258	1	4.08
Cebpzos	XP_030105878.1	83	0	0.00	Smco4	XP_006510106.1	72	0	0.00
Cebpz	NP_001019977.1	738	0	0.00	Deup1	XP_006510228.1	163	0	0.00
Ndufaf7	NP_082887.2	172	0	0.00	Slc36a4	NP_758493.2	319	2	6.61
Prkd3	NP_083515.2	343	0	0.00	Mtnr1b	NP_663758.2	627	2	3.36
Qpct	NP_081731.1	311	1	3.39	Fat3	XP_006510435.1	881	2	2.39
Cdc42ep3	NP_080790.1	318	0	0.00	Chordc1	NP_080120.1	386	0	0.00
Rmdn2	XP_036016564.1	127	1	8.30	Naalad2	NP_001344329.1	352	0	0.00
Cyp1b1	NP_001351818.1	783	3	4.04	Ubtfl1	NP_001347145.1	536	0	0.00
Atl2	NP_062691.3	450	0	0.00	Mbd3l2	NP_659183.3	369	2	5.71
Hnrnpll	NP_659051.3	727	1	1.45	Zfp558	XP_017169091.1	198	2	10.65
Galm	NP_795937.1	601	0	0.00	Mbd3l1	NP_001344347.1	487	2	4.33
Srsf7	XP_011244714.1	989	1	1.07	Muc16	NP_001365177.1	347	2	6.07
Ttc39d	NP_080627.2	21	0	0.00	Olfr24	NP_666817.1	84	0	0.00
Gemin6	NP_001342387.1	538	0	0.00	Olfr828	NP_666816.1	135	0	0.00
Dhx57	NP_945180.2	1045	3	3.03	Olfr829	NP_667278.1	55	0	0.00
Morn2	NP_001347369.1	92	0	0.00	Olfr830	NP_666777.1	121	0	0.00
Arhgef33	XP_006524572.1	86	0	0.00	Olfr832	NP_001011824.1	65	0	0.00
Sos1	NP_033257.2	1151	2	1.83	Olfr834	NP_001011823.1	143	0	0.00
Cdkl4	XP_006524592.1	153	1	6.89	Olfr835	NP_001012266.1	261	0	0.00
Map4k3	XP_036016468.1	463	2	4.55	Olfr836	NP_666775.1	26	0	0.00
Tmem178	NP_080792.2	304	2	6.93	Olfr837	NP_666776.1	26	0	0.00
Thumpd2	NP_082414.1	185	1	5.70	Olfr843	NP_666778.2	29	0	0.00
Slc8a1	NP_001273613.1	622	1	1.69	Olfr845	NP_997028.1	75	1	14.05
Pkdcc	NP_598878.2	1197	3	2.64	Olfr846	NP_666394.1	79	0	0.00

Eml4	XP_006525218.1	953	3	3.32	Olfr847	NP_666736.1	21	0	0.00
Cox7a2l	NP_033213.1	279	1	3.78	Olfr849	NP_666738.1	218	1	4.83
Kcng3	XP_006524263.1	668	3	4.73	Olfr850	NP_666734.1	49	0	0.00
Mta3	NP_001366318.1	350	1	3.01	Olfr851	XP_017168884.1	88	0	0.00
Haao	XP_030105301.1	792	2	2.66	Olfr853	NP_667117.1	87	0	0.00
Zfp36l2	NP_001001806.1	812	2	2.60	Olfr854	NP_666733.1	357	2	5.90
Thada	NP_898842.2	635	2	3.32	Olfr855	NP_666735.2	80	0	0.00
Plekhh2	XP_017172870.1	151	0	0.00	Zfp317	XP_017168854.1	261	0	0.00
Dync2li1	NP_758460.1	333	3	9.49	Olfr857	NP_001012265.1	31	0	0.00
Abcg5	NP_114090.1	1126	3	2.81	Olfr58	NP_035131.1	47	0	0.00
Abcg8	NP_080456.1	1126	3	2.81	Olfr859	NP_666737.2	22	0	0.00
Lrpprc	NP_082509.2	777	5	6.78	Olfr860	NP_666739.1	45	0	0.00
Ppm1b	XP_030105453.1	1150	6	5.50	Olfr862	XP_030100252.1	35	0	0.00
Slc3a1	NP_033231.2	982	8	8.59	Olfr77	NP_666451.1	61	0	0.00
Prepl	NP_001157094.1	356	4	11.84	Olfr866	NP_666769.1	64	0	0.00
Camkmt	NP_082852.1	314	6	20.14	Olfr867	NP_001011748.1	86	0	0.00
Six3	XP_017172856.1	717	1	1.47	Olfr868	NP_666770.1	33	0	0.00
Six2	XP_011244637.1	646	2	3.26	Olfr869	XP_017168879.1	15	0	0.00
Srbd1	NP_084409.3	887	0	0.00	Olfr870	NP_667115.1	56	0	0.00
Prkce	NP_035234.1	1707	5	3.09	Olfr871	NP_667114.2	74	0	0.00
Epas1	NP_034267.3	1090	2	1.93	Olfr872	NP_666771.2	23	0	0.00
Tmem247	NP_001264909.1	197	1	5.35	Olfr39	NP_667036.2	42	0	0.00
Atp6v1e2	NP_083397.3	420	0	0.00	Olfr873	NP_666772.1	39	0	0.00
Rhoq	NP_663466.2	1785	7	4.13	Olfr18	NP_666774.1	27	0	0.00
Pigf	XP_036016296.1	107	4	39.40	Zfp560	NP_001004190.2	192	0	0.00
Cript	NP_064320.1	224	4	18.82	Zfp26	NP_001344738.1	368	0	0.00
Socs5	NP_062628.2	501	7	14.73	Zfp426	NP_001297671.1	237	1	4.45
Mcfd2	XP_030105467.1	396	6	15.97	Zfp266	NP_001075954.1	251	1	4.20
Ttc7	XP_006524264.1	240	6	26.35	Zfp846	NP_766507.2	157	0	0.00
Stpg4	XP_006525135.1	19	1	55.47	Fbxl12	NP_001273458.1	892	5	5.91
Calm2	NP_031615.1	2215	6	2.85	Ubl5	NP_001297675.1	642	3	4.92
Epcam	NP_032558.2	1165	3	2.71	Pin1	NP_075860.1	962	1	1.10
Msh2	NP_032654.1	1206	9	7.86	Olfm2	NP_001344568.1	546	4	7.72
Kcnk12	NP_954859.1	287	6	22.03	Col5a3	NP_001304317.1	493	3	6.41
Msh6	NP_034960.1	1551	3	2.04	Rdh8	NP_001025461.1	344	1	3.06
Fbxo11	NP_001366232.1	884	9	10.73	Shfl	NP_783618.1	17	1	61.99
Foxn2	NP_001342671.1	783	5	7.08	Angptl6	NP_660136.1	406	1	2.60
Ppp1r21	NP_082934.3	111	2	21.10	Ppan	NP_663585.2	1085	3	2.91
Ston1	NP_084134.2	442	8	22.44	Eif3g	NP_058572.2	1195	5	4.41
Gtf2a1l	NP_076119.2	359	5	18.35	Dnmt1	XP_006510051.1	2161	2	0.98
Lhcgr	XP_011244612.1	629	8	17.87	S1pr2	NP_034463.2	574	4	7.34
Fshr	NP_038551.3	681	7	15.48	Mrpl4	NP_075656.2	1487	7	4.96
Nrxn1	XP_036016276.1	1047	3	4.65	Icam1	NP_034623.1	1559	6	4.06
Adcyap1	NP_001302433.1	832	4	8.44	Icam4	NP_076381.1	204	4	20.66
Mettl4	NP_001344067.1	177	1	10.83	Icam5	NP_032345.2	641	5	8.22
Gm20939	NP_001366374.1	128	0	0.00	Zglp1	NP_001096638.1	596	3	5.30
Vmn1r238	NP_001161011.1	2	0	0.00	Fdx2	NP_001034913.1	642	2	3.28
Crem	NP_001104328.1	907	1	2.11	Raver1	NP_082187.1	968	2	2.18
Cul2	NP_001347758.1	1825	0	0.00	Tyk2	XP_006510555.1	1421	3	2.22
Bambi	XP_030106445.1	366	0	0.00	Cdc37	NP_058022.1	779	1	1.35
Lyzl1	NP_080368.1	365	3	12.37	Pde4a	NP_899668.1	637	2	3.31
Map3k8	NP_031772.1	1045	2	2.69	Keap1	NP_001103775.1	1266	4	3.33
Mtpap	NP_080433.1	610	1	2.16	S1pr5	NP_444420.1	418	1	2.52
Jcad	NP_001075432.1	134	3	27.76	Atg4d	NP_705811.8	831	4	5.07
Svil	XP_036016964.1	595	2	3.94	Kri1	NP_663391.2	681	1	1.55
Zfp438	NP_001347376.1	203	1	5.46	Cdkn2d	NP_034008.2	1232	2	1.71
Zeb1	NP_035676.1	1156	2	1.82	Ap1m2	XP_006510013.1	638	0	0.00
Arhgap12	XP_030106483.1	553	1	1.91	Slc44a2	NP_001186115.1	547	6	11.56
Kif5b	NP_032474.2	1780	3	1.78	Ilf3	XP_011240705.1	961	5	5.48
Epc1	NP_031961.1	557	1	1.89	Qtrt1	NP_068688.2	611	3	5.17
4921524L21Ri	NP_081874.1	115	2	18.33	Dnm2	NP_001240823.1	1867	6	3.39
Rab18	NP_001265376.1	886	2	2.38	Tmed1	NP_034874.2	154	1	6.84
Mkx	NP_808263.2	442	4	9.54	AB124611	NP_001185723.1	117	1	9.01
Armc4	XP_006526376.1	302	1	3.49	Carm1	NP_067506.2	1251	9	7.58
Mpp7	NP_001347789.1	623	2	3.38	Yipf2	NP_001346228.1	191	0	0.00
Wac	XP_006525866.1	1070	4	3.94	Timm29	NP_848734.1	94	0	0.00

Fzd8	NP_032084.2	684	3	4.62	Smarca4	XP_011240739.1	2487	4	1.70
Gjd4	NP_694726.2	131	3	24.14	Ldlr	NP_034830.2	1493	12	8.47
Ccny	NP_080760.2	321	2	6.57	Spc24	NP_080558.1	511	1	2.06
Cetn1	NP_031619.3	1487	6	4.25	Kank2	NP_663586.3	1077	9	8.81
Colec12	NP_569716.2	397	2	5.31	Dock6	XP_036010951.1	431	9	22.01
Thoc1	NP_705780.1	702	10	15.01	Angptl8	NP_001074409.1	123	5	42.84
Usp14	NP_001033678.1	1087	6	5.82	Rab3d	NP_001311460.1	601	8	14.03
Rock1	XP_006525788.1	2152	10	4.90	Tmem205	NP_001240796.1	218	9	43.51
Greb1l	XP_006526131.1	228	0	0.00	Ccdc159	NP_001158086.1	21	8	401.49
Esco1	XP_006526416.1	809	5	6.51	Plppr2	NP_001277228.1	235	7	31.39
Snrpd1	NP_033252.1	1585	1	0.66	Swsap1	NP_080146.1	133	8	63.39
Abhd3	NP_598891.1	501	6	12.62	Epor	NP_034279.3	492	1	2.14
Mib1	NP_001351926.1	2717	5	1.94	Rgl3	XP_036011193.1	271	0	0.00
Gata6	NP_034388.2	1581	8	5.33	Ccdc151	XP_011240928.1	132	0	0.00
Rbbp8	NP_001074692.1	778	6	8.13	Prkcsh	XP_006510158.1	549	0	0.00
Cables1	NP_001139759.1	357	9	26.57	Elavl3	XP_030099924.1	1470	0	0.00
Tmem241	XP_030106378.1	230	1	4.58	Zfp653	NP_796292.1	218	1	4.83
Riok3	NP_077144.2	612	0	0.00	Ecsit	XP_011240841.1	483	1	2.18
Rmc1	XP_006526404.1	103	0	0.00	Cnn1	XP_011240690.2	730	0	0.00
Npc1	XP_017173338.1	815	1	1.29	Elof1	NP_001344242.1	275	0	0.00
Ankrd29	XP_017173360.1	1044	3	3.03	Acp5	NP_001095874.1	795	0	0.00
Lama3	XP_006525748.1	606	5	8.70	Pigyl	NP_001076001.1	68	0	0.00
Ttc39c	NP_082617.2	213	3	14.84	Zfp872	NP_001028985.1	136	0	0.00
Cabyr	NP_859420.2	281	1	3.75	Zfp809	XP_006510251.1	239	0	0.00
Osbpl1a	XP_006526207.1	546	5	9.65	Zfp599	NP_852084.2	126	0	0.00
Impact	NP_032404.1	195	1	5.40	Zfp810	NP_663587.1	168	0	0.00
Hrh4	NP_694727.1	434	2	4.86	Anln	NP_082666.2	931	0	0.00
Zfp521	NP_001347733.1	600	4	7.03	9530077C05R	XP_006510631.2	24	0	0.00
Ss18	XP_030106329.1	359	0	0.00	Rp9	NP_061209.1	224	1	4.70
Psma8	NP_001157081.1	1201	3	2.63	Bbs9	NP_001347188.1	463	2	4.55
Taf4b	NP_001093919.1	512	0	0.00	Bmper	NP_082748.1	475	1	2.22
Kctd1	NP_598873.2	271	2	7.78	Npsr1	XP_017168906.1	1500	1	0.70
Aqp4	NP_001304658.1	1366	0	0.00	Dpy19l1	NP_001346877.1	406	1	2.60
Chst9	NP_951010.1	185	2	11.39	Dpy19l2	XP_006510490.1	305	0	0.00
Cdh2	NP_031690.3	1927	3	1.64	Cypt4	NP_775588.2	263	0	0.00
Dsc3	NP_031908.3	416	8	20.27	Tbx20	NP_919239.1	563	0	0.00
Dsc2	NP_001304294.1	662	9	14.33	Herpud2	NP_065611.1	250	0	0.00
Dsc1	NP_001278733.1	308	8	27.37	Septin7	XP_006510253.1	1217	1	0.87
Dsg1c	NP_859008.1	482	8	17.49	Eepd1	NP_080465.3	903	1	1.17
Dsg1a	NP_034209.2	430	8	19.61	B3gat1	NP_001297695.1	1123	1	0.94
Dsg1b	NP_859010.1	233	9	40.71	Glb1l2	NP_001297697.1	262	0	0.00
Dsg4	NP_853543.1	388	8	21.73	Glb1l3	NP_001106794.1	283	1	3.72
Dsg3	NP_085099.2	429	8	19.65	Gm1110	XP_006510524.1	134	0	0.00
Dsg2	NP_031909.2	636	9	14.91	Acad8	NP_080138.2	595	0	0.00
Ttr	XP_030106272.1	1463	4	2.88	Thyn1	XP_006510751.1	197	5	26.75
B4galt6	NP_062711.1	334	1	3.16	Vps26b	NP_821170.1	634	2	3.32
Trappc8	XP_030106491.1	801	4	5.26	Ncapd3	XP_017169187.1	645	3	4.90
Rnf125	NP_001351902.1	259	3	12.21	Jam3	NP_075766.1	392	0	0.00
Rnf138	NP_997506.1	521	3	6.07	Igsf9b	NP_001313631.1	477	1	2.21
Mep1b	NP_032612.2	449	6	14.08	Spata19	NP_083575.1	255	0	0.00
Garem1	NP_001028617.2	98	6	64.52	Opcml	XP_030100283.1	1025	3	3.08
Klhl14	NP_001074872.1	206	3	15.35	Ntm	NP_758494.2	958	3	3.30
Ccdc178	NP_081892.2	85	6	74.39	Snx19	XP_030099808.1	194	3	16.30
Asxl3	XP_006525803.1	291	3	10.86	Adamts15	NP_001019310.1	202	1	5.22
Nol4	NP_001334439.1	510	0	0.00	Adamts8	NP_038934.2	309	2	6.82
Dtna	XP_036016865.1	600	7	12.30	Zbtb44	XP_006510278.1	382	0	0.00
Mapre2	NP_001156413.1	927	5	5.68	St14	NP_035306.2	409	1	2.58
Zscan30	XP_036017071.1	330	0	0.00	Aplp2	NP_033821.1	1091	4	3.86
Zfp397	NP_081283.2	292	6	21.66	Prdm10	NP_001355774.1	1426	0	0.00
Zfp35	XP_036017028.1	218	1	4.83	Nfrkb	XP_006510281.1	349	1	3.02
Zfp24	NP_001344377.1	400	2	5.27	Tmem45b	NP_659185.1	115	1	9.16
Ino80c	NP_766213.1	308	1	3.42	Barx2	NP_038828.2	276	2	7.64
Galnt1	NP_001348129.1	325	5	16.21	Arhgap32	XP_036010972.1	819	2	2.57
2700062C07R	NP_080805.1	70	8	120.45	Kcnj5	XP_017168642.1	474	1	2.22
Rprd1a	NP_001347736.1	406	8	20.77	Kcnj1	NP_001161826.1	480	0	0.00
Slc39a6	NP_631882.2	270	5	19.52	Fli1	XP_036010504.1	522	1	2.02

Elp2	NP_067423.2	600	5	8.78	Ets1	NP_001359463.1	1275	3	2.48
Mocos	NP_081055.1	452	5	11.66	Kirrel3	XP_036011097.1	630	2	3.35
Fhod3	XP_006525889.1	403	1	2.62	St3gal4	XP_006510171.1	284	0	0.00
Tpgs2	NP_001136170.1	174	1	6.06	Dcps	NP_081306.1	518	0	0.00
AW554918	XP_006525896.3	194	2	10.86	Tirap	XP_030099888.1	389	0	0.00
Celf4	NP_001349222.1	1046	6	6.05	Foxred1	XP_017168798.1	296	0	0.00
Pik3c3	XP_030106284.1	2490	4	1.69	Srpr	NP_080406.1	915	2	2.30
Rit2	NP_033091.1	2353	7	3.14	Fam118b	XP_030099865.1	75	0	0.00
Syt4	NP_033334.2	1296	2	1.63	Rpusd4	NP_082316.1	421	2	5.01
Slc25a46	NP_080441.1	237	3	13.34	Cdon	NP_067314.2	275	1	3.83
Sap130	XP_006526012.1	298	4	14.15	Ddx25	NP_038960.2	774	7	9.53
Ammecr1l	XP_036016993.1	162	2	13.01	Hyls1	NP_084038.1	142	1	7.42
Polr2d	NP_081278.1	1159	3	2.73	Pus3	NP_075781.3	698	2	3.02
Wdr33	NP_083142.2	1226	5	4.30	Pate4	NP_064660.2	219	6	28.87
Sft2d3	NP_080282.1	42	0	0.00	Pate3	NP_001161064.1	60	9	158.09
Lims2	NP_659111.1	785	2	2.69	Pate2	NP_001028593.1	95	10	110.94
Gpr17	NP_001020552.1	601	2	3.51	Pate1	NP_001186882.1	98	9	96.79
Myo7b	NP_115770.2	1143	4	3.69	Pate10	NP_001161060.1	124	7	59.49
Iws1	NP_775617.1	647	4	6.52	Pate7	NP_001161145.1	70	6	90.33
Proc	NP_032960.3	951	0	0.00	Pate6	NP_080869.1	192	7	38.42
Map3k2	XP_036017049.1	1479	3	2.14	Pate5	NP_084139.1	77	8	109.50
Ercc3	NP_598419.1	987	5	5.34	Pate13	NP_001365151.1	55	5	95.81
Bin1	XP_006526041.1	1267	3	2.50	Gm5916	XP_006510553.1	12	5	439.13
Gypc	NP_001334539.1	338	0	0.00	Gm3867	NP_001347990.1	64	14	230.54
Tslp	NP_067342.1	534	1	1.97	Pate12	NP_001161058.1	76	5	69.34
Wdr36	NP_001103486.1	1027	2	2.05	Pate11	NP_001121982.1	62	4	67.99
Camk4	XP_006525606.1	1403	3	2.25	Pate9	NP_001028955.1	65	2	32.43
Stard4	NP_598535.1	231	2	9.12	Pate8	NP_001161056.1	41	2	51.41
Nrep	NP_001103458.1	203	1	5.19	Pate14	NP_001028497.1	95	2	22.19
Epb41l4a	XP_006525690.1	413	1	2.55	Acrv1	NP_031417.2	305	9	31.10
Apc	XP_006525592.1	926	4	4.55	Chek1	NP_031717.2	1975	1	0.53
Srp19	NP_079803.2	811	4	5.20	Stt3a	NP_032434.3	767	1	1.37
Reep5	NP_031900.3	1128	2	1.87	Ei24	NP_001186423.2	215	1	4.90
Pkd2l2	XP_017173433.1	183	2	11.52	Fez1	NP_001344539.1	796	1	1.32
Fam13b	XP_036017002.1	198	6	31.94	Pknox2	XP_030100024.1	401	0	0.00
Wnt8a	NP_033316.2	667	7	11.06	Tmem218	NP_001297025.1	106	0	0.00
Nme5	NP_542368.2	803	9	11.81	Slc37a2	NP_064654.3	569	0	0.00
Brd8dc	NP_001345230.1	868	2	2.43	Ccdc15	XP_030100249.1	101	1	10.43
Brd8	NP_001276535.1	1345	9	7.05	Hepacam	NP_780398.2	429	1	2.46
Kif20a	NP_001159878.1	1544	2	1.37	Robo4	XP_006510707.1	681	2	3.10
Cdc23	XP_006526147.1	1431	13	9.57	Robo3	XP_011240726.1	698	1	1.51
Gfra3	NP_034410.3	249	10	42.33	Msantd2	XP_006510293.1	22	0	0.00
Cdc25c	XP_006525614.1	1399	5	3.77	Esam	NP_081378.1	424	5	12.43
Fam53c	NP_001344385.1	188	17	95.30	Vsig2	NP_065264.2	219	4	19.25
Kdm3b	NP_001074725.1	400	12	31.62	Nrgn	NP_071312.1	741	4	5.69
Reep2	NP_659114.2	1356	15	11.66	Spa17	XP_011240741.2	412	4	10.23
Egr1	NP_031939.1	2152	7	3.43	Siae	NP_035864.2	253	4	16.66
Etf1	NP_659115.3	1495	13	9.16	Tbrg1	NP_079565.2	974	1	1.08
Hspa9	NP_034611.2	2646	12	4.78	Panx3	NP_766042.2	222	1	4.75
Ctnna1	XP_030106159.1	988	11	11.73	Olfr160	NP_085030.2	113	0	0.00
Lrrtm2	NP_821072.1	903	13	15.17	Olfr874	XP_017168883.1	37	0	0.00
Sil1	NP_001347743.1	1518	11	7.64	Olfr875	NP_666960.2	32	0	0.00
Matr3	NP_034901.2	917	9	10.34	Olfr876	NP_667094.2	32	0	0.00
Paip2	XP_017173462.1	365	6	17.32	Olfr877	NP_666529.1	22	1	47.90
Slc23a1	XP_017173340.2	528	6	11.98	Olfr145	XP_017168878.1	71	0	0.00
Mzb1	NP_081498.2	233	1	4.52	Olfr878	NP_667009.1	41	1	25.70
Prob1	NP_001257575.1	39	2	54.05	Olfr881	NP_666530.1	28	0	0.00
Spata24	NP_083761.1	79	2	26.68	Olfr883	NP_666531.1	99	0	0.00
Dnajc18	NP_083945.1	515	4	8.19	Olfr884	XP_030100251.1	13	0	0.00
Ecscr	NP_001355581.1	128	0	0.00	Olfr885	NP_001011739.1	13	0	0.00
1700066B19R	XP_006526353.1	0	0	#DIV/0!	Olfr887	NP_666634.1	44	0	0.00
Sting1	NP_001276520.1	524	0	0.00	Olfr888	NP_666635.1	36	0	0.00
Ube2d2a	NP_064296.1	2115	1	0.50	Olfr889	NP_666693.1	13	0	0.00
Cxxc5	NP_001344387.1	281	0	0.00	Olfr890	NP_666692.2	14	0	0.00
Psd2	XP_036017188.1	1027	0	0.00	Olfr891	NP_666689.2	48	0	0.00
Nrg2	XP_006525525.1	357	1	2.95	Olfr893	NP_666448.2	13	0	0.00

Pura	XP_006525787.1	588	1	1.79	Olfr894	NP_667079.1	109	0	0.00
Igip	NP_001254725.1	588	1	1.79	Olfr143	NP_667017.2	22	0	0.00
Cystm1	NP_001355566.1	174	1	6.06	Olfr895	NP_667086.2	45	0	0.00
Pfdn1	NP_080303.1	454	1	2.32	Olfr25	NP_667081.2	14	0	0.00
Hbegf	NP_034545.1	741	2	2.84	Olfr898	NP_667082.2	33	0	0.00
Slc4a9	XP_011245222.1	286	3	11.05	Olfr250	NP_997435.2	44	0	0.00
Ankhd1	NP_780584.2	1549	5	3.40	Olfr251	NP_997436.2	188	0	0.00
Eif4ebp3	NP_957708.1	212	2	9.94	Olfr147	NP_667080.1	29	0	0.00
Sra1	NP_079567.2	447	1	2.36	Olfr901	NP_001011806.1	25	0	0.00
Apbb3	NP_666197.1	447	1	2.36	Olfr902	NP_667013.2	85	0	0.00
Slc35a4	NP_080680.2	295	0	0.00	Olfr904	XP_017168881.1	12	0	0.00
E230025N22R	XP_017173388.1	7	0	0.00	Olfr905	NP_667015.2	12	0	0.00
Cd14	NP_033971.1	961	2	2.19	Olfr906	NP_667014.2	38	0	0.00
Tmco6	NP_082312.3	4	0	0.00	Olfr907	NP_667016.2	10	0	0.00
Ndufa2	NP_035015.2	762	0	0.00	Olfr911-ps1	NP_667084.2	10	0	0.00
Ik	NP_036009.1	490	1	2.15	Olfr910	XP_017168882.1	12	0	0.00
Wdr55	NP_080740.2	430	10	24.51	Olfr912	NP_667021.2	34	0	0.00
Dnd1	NP_775559.2	396	2	5.32	Olfr913	NP_001011523.2	82	0	0.00
Hars	NP_032240.3	1200	3	2.63	Olfr914	NP_666997.2	56	0	0.00
Hars2	NP_542367.1	957	2	2.20	Olfr916	NP_666995.1	26	0	0.00
Zmat2	NP_079870.1	465	14	31.73	Olfr917	NP_001011864.1	23	0	0.00
Pcdha1	NP_473413.1	458	2	4.60	Olfr918	NP_666487.2	37	0	0.00
Pcdha2	NP_932785.1	458	2	4.60	Olfr919	NP_666651.1	189	0	0.00
Pcdha4	NP_031792.1	458	2	4.60	Vwa5a	NP_766355.3	254	0	0.00
Pcdha5	NP_034089.1	458	2	4.60	Olfr920	NP_666998.2	50	0	0.00
Pcdha9	NP_619602.1	458	2	4.60	Olfr921	NP_666993.2	29	0	0.00
Pcdha10	NP_034091.1	458	2	4.60	Olfr922	NP_666992.1	22	0	0.00
Pcdha11	NP_034090.1	458	1	2.30	Olfr923	NP_667027.2	33	0	0.00
Pcdha12	NP_619604.1	458	1	2.30	Olfr924	NP_997443.1	34	0	0.00
Pcdhac1	NP_001003671.1	458	1	2.30	Olfr26	NP_666994.2	39	0	0.00
Pcdhac2	NP_001003672.1	458	1	2.30	Olfr926	NP_667026.1	78	0	0.00
Pcdhb1	NP_444356.1	278	0	0.00	Olfr930	NP_666384.1	27	0	0.00
Pcdhb2	NP_444357.1	212	0	0.00	Olfr933	NP_666652.1	30	0	0.00
Pcdhb3	NP_444358.2	231	0	0.00	Olfr934	NP_666653.1	61	0	0.00
Pcdhb4	NP_444359.3	165	0	0.00	Olfr935	NP_666957.1	49	0	0.00
Pcdhb5	NP_444360.1	196	0	0.00	Olfr146	NP_666958.1	105	0	0.00
Pcdhb6	NP_444361.1	247	0	0.00	Olfr936	NP_997022.1	55	0	0.00
Pcdhb7	NP_444362.3	236	0	0.00	Olfr937	NP_666650.1	55	0	0.00
Pcdhb8	NP_444363.1	240	1	4.39	Olfr938	NP_666649.1	136	0	0.00
Pcdhb9	NP_444364.3	246	0	0.00	Olfr27	NP_667040.2	22	0	0.00
Pcdhb10	NP_444365.1	191	1	5.52	Olfr943	NP_666438.2	64	0	0.00
Pcdhb11	NP_444366.3	190	0	0.00	Olfr944	NP_666718.1	44	0	0.00
Pcdhb12	NP_444367.1	241	0	0.00	Olfr1537	NP_997548.1	41	0	0.00
Pcdhb13	NP_444368.1	267	1	3.95	Olfr945	NP_666717.1	57	0	0.00
Pcdhb14	NP_444369.3	298	0	0.00	Olfr948	NP_001011756.1	65	0	0.00
Pcdhb15	NP_444370.1	309	2	6.82	Olfr951	NP_001011812.1	44	0	0.00
Pcdhb16	NP_444371.3	285	1	3.70	Olfr952	NP_666714.1	153	0	0.00
Pcdhb17	NP_444372.2	262	1	4.02	Olfr954	NP_666443.1	122	0	0.00
Pcdhb18	NP_444373.1	281	6	22.50	Olfr955	NP_997024.1	105	0	0.00
Pcdhb19	NP_444374.2	220	2	9.58	Olfr44	NP_667041.2	68	0	0.00
Pcdhb20	NP_444375.2	280	1	3.76	Olfr957	NP_666956.2	12	0	0.00
Pcdhb21	NP_444376.1	239	0	0.00	Olfr958	NP_666442.1	111	0	0.00
Pcdhb22	NP_444377.3	234	1	4.50	Olfr959	NP_666719.1	116	0	0.00
Slc25a2	NP_001152747.1	118	15	133.97	AW551984	XP_006510368.1	114	0	0.00
Taf7	NP_786964.1	669	10	15.75	Olfr148	NP_666716.1	110	0	0.00
Pcdhga3	NP_291064.1	395	2	5.34	Olfr960	NP_666391.1	72	0	0.00
Pcdhga8	NP_291069.1	395	2	5.34	Olfr961	NP_666715.1	68	0	0.00
Pcdhga9	NP_291070.1	395	2	5.34	Olfr963	NP_001011827.1	174	0	0.00
Pcdhgb6	NP_291056.1	395	2	5.34	Olfr149	NP_997021.1	72	0	0.00
Pcdhga11	NP_291072.1	395	3	8.00	Olfr965	NP_001011859.1	55	0	0.00
Pcdhga12	NP_291073.1	395	3	8.00	Olfr150	NP_666820.2	62	0	0.00
Pcdhgc3	NP_291059.1	395	4	10.67	Olfr967	NP_001011826.1	54	0	0.00
Pcdhgc4	NP_291060.1	395	4	10.67	Olfr968	NP_666823.1	37	0	0.00
Pcdhgc5	NP_291061.1	395	4	10.67	Olfr969	NP_667037.1	45	0	0.00
Diaph1	NP_031884.1	773	1	1.36	Olfr970	NP_666822.1	118	0	0.00
Hdac3	NP_034541.2	2524	1	0.42	Olfr971	NP_666825.1	67	0	0.00

Rell2	NP_722488.2	186	0	0.00	Olfr972	NP_666821.1	18	0	0.00
Fchsd1	NP_783615.2	337	1	3.13	Olfr229	NP_666824.1	11	0	0.00
Arap3	XP_036016812.1	596	3	5.30	Olfr974	XP_017168885.1	36	0	0.00
Pcdh1	XP_006526383.1	415	7	17.78	Olfr975	NP_667039.1	59	0	0.00
Dele1	NP_077141.2	449	0	0.00	Olfr976	NP_666479.1	198	0	0.00
Pcdh12	NP_059074.2	400	6	15.81	Olfr978	NP_667316.2	82	0	0.00
Rnf14	XP_017173445.1	681	3	4.64	Olfr979	NP_667319.1	57	0	0.00
Gnpda1	NP_036067.2	523	1	2.02	Olfr980	NP_667317.1	30	0	0.00
Ndfip1	NP_001342678.1	390	2	5.40	Olfr981	NP_666398.1	94	0	0.00
Spry4	XP_036017041.1	512	2	4.12	Olfr982	NP_667065.1	125	0	0.00
Arhgap26	XP_036017178.1	594	9	15.97	Olfr983	NP_667038.1	25	0	0.00
Fgf1	XP_011245143.1	967	8	8.72	Olfr984	NP_666819.1	35	0	0.00
Gm5820	NP_001028961.1	594	10	17.74	Olfr985	NP_667066.1	42	0	0.00
Nr3c1	NP_032199.3	1886	8	4.47	Tmem225	NP_083655.1	159	0	0.00
Pabpc2	NP_035163.1	1188	1	0.89	Olfr986	NP_666826.1	58	0	0.00
Yipf5	NP_075800.1	469	16	35.95	Zfp202	NP_109638.2	369	1	2.86
Kctd16	NP_001357968.1	378	14	39.03	Scn3b	NP_001346629.1	552	3	5.73
Prelid2	NP_084218.1	63	13	217.47	Gramd1b	XP_030100160.1	164	1	6.43
Grxcr2	NP_001028598.1	154	16	109.50	Clmp	NP_598494.2	309	0	0.00
Sh3rf2	NP_001139771.1	358	14	41.21	Hspa8	NP_112442.2	3248	0	0.00
Plac8l1	NP_081348.1	72	17	248.84	Bsx	XP_036010875.1	342	3	9.24
Lars	NP_598898.2	1255	11	9.24	Jhy	XP_006510664.1	28	2	75.28
Rbm27	XP_006525926.1	348	14	42.40	Crtam	NP_062338.3	272	2	7.75
Pou4f3	NP_620395.2	538	9	17.63	Ubash3b	XP_006510695.1	524	0	0.00
Tcerg1	XP_006526173.1	1235	12	10.24	Sorl1	NP_035566.2	944	1	1.12
Gpr151	NP_853521.1	297	13	46.13	Sc5d	NP_766357.1	626	2	3.37
Ppp2r2b	NP_082668.1	1821	12	6.94	Tecta	NP_001311477.1	372	2	5.67
Stk32a	NP_848864.1	968	12	13.06	Tbcel	NP_001344586.1	158	1	6.67
Dpysl3	NP_001278384.1	719	3	4.40	Grik4	XP_036010454.1	582	1	1.81
Eif3j2	NP_001242984.1	309	0	0.00	Arhgef12	XP_036011146.1	822	2	2.56
Jakmip2	XP_006526401.1	551	7	13.39	Tlcd5	XP_017168819.1	124	3	25.50
Spink1	NP_033284.1	423	3	7.47	Pou2f3	NP_035269.2	412	1	2.56
Scgb3a2	NP_001276572.1	242	0	0.00	Oaf	NP_848759.1	130	1	8.11
Gm94	NP_001028452.1	121	1	8.71	Trim29	XP_006510679.1	303	2	6.96
Spink5	NP_001074649.1	427	5	12.34	Nectin1	NP_067399.2	434	6	14.57
Spink14	NP_001034307.2	91	2	23.16	Thy1	NP_033408.1	1642	6	3.85
Spink6	NP_001013819.1	128	3	24.70	Usp2	NP_932760.2	835	5	6.31
Spink12	NP_084337.2	183	1	5.76	C1qtnf5	NP_667337.1	470	5	11.21
Gm10267	NP_001268399.1	0	0	#DIV/0!	Rnf26	NP_717095.2	256	12	49.40
Spinkl	NP_898946.1	39	2	54.05	Mcam	NP_075548.2	680	3	4.65
Spink11	NP_001041682.3	27	0	0.00	Cbl	XP_006510026.1	1142	6	5.54
Npy6r	NP_035065.1	383	1	2.75	Ccdc153	XP_036010921.1	87	0	0.00
Myot	XP_006526194.1	443	0	0.00	Pdzd3	NP_573489.2	595	9	15.94
Dcp2	NP_081766.1	972	5	5.42	Nlrx1	NP_848507.2	375	3	8.43
Mcc	NP_001078842.1	148	2	14.24	Abcg4	NP_620405.3	816	10	12.92
Ythdc2	NP_001156485.1	2054	3	1.54	Hinfp	XP_006509960.1	446	9	21.27
Kcnn2	XP_030106191.1	467	1	2.26	C2cd2l	NP_082185.2	163	6	38.79
Trim36	NP_849203.2	895	2	2.36	Dpagt1	XP_006510054.1	716	12	17.66
Pggt1b	NP_766215.1	354	3	8.93	H2ax	NP_034566.1	2039	3	1.55
Ccdc112	NP_001153871.1	98	5	53.77	Hmbs	NP_038579.2	1036	6	6.10
Fem1c	NP_775599.1	1135	2	1.86	Vps11	NP_082165.1	803	8	10.50
Ticam2	XP_036017008.1	364	1	2.90	Hyou1	NP_067370.3	1731	5	3.04
Tmed7	NP_079974.1	508	3	6.22	Slc37a4	NP_001344659.1	556	15	28.43
Eif1a	NP_001361583.1	941	1	1.12	Trappc4	NP_068561.1	394	8	21.40
Cdo1	NP_149026.1	561	0	0.00	Rps25	NP_077228.1	1067	8	7.90
Atg12	NP_080493.2	721	1	1.46	Ccdc84	NP_958760.2	67	7	110.11
Ap3s1	XP_036016836.1	517	2	4.08	Foxr1	NP_001028641.1	590	5	8.93
Lvrn	NP_083284.1	530	1	1.99	Upk2	NP_033502.2	604	9	15.70
Arl14epl	XP_006526129.1	81	0	0.00	Bcl9l	XP_017169189.1	346	10	30.46
Commd10	XP_006526287.1	314	6	20.14	Cxcr5	NP_031577.2	921	2	2.29
Sema6a	NP_001298026.1	573	3	5.52	Ddx6	NP_001344632.1	2253	9	4.21
Eno1b	NP_001020559.1	1137	0	0.00	Treh	NP_067456.1	812	10	12.98
Dtwd2	XP_006526277.1	147	5	35.85	Phldb1	XP_030099838.2	172	7	42.89
Dmxl1	XP_006525979.1	795	4	5.30	Arcn1	NP_666097.3	887	10	11.88
Tnfaip8	NP_001171231.1	362	6	17.47	Ift46	XP_036011297.1	366	0	0.00
Hsd17b4	NP_032318.2	925	4	4.56	Tmem25	XP_030100472.1	125	3	25.29

Fam170a	XP_006525932.1	305	5	17.28	Ttc36	NP_620401.1	634	0	0.00
Pudp	NP_080384.2	588	3	5.38	Kmt2a	XP_011240745.1	1613	3	1.96
Prr16	NP_001074693.1	312	3	10.13	Atp5l	XP_036010936.1	528	1	2.00
Ftmt	NP_080562.2	535	9	17.73	Ube4a	NP_663375.3	998	9	9.50
Srfbp1	NP_080316.3	237	0	0.00	Cd3g	NP_033980.1	758	6	8.34
Lox	NP_034858.2	1538	3	2.06	Cd3d	XP_006510028.1	746	5	7.06
Zfp474	NP_001347913.1	59	3	53.59	Cd3e	XP_006510029.1	640	5	8.23
1700034E13R	NP_084373.1	162	2	13.01	Mpzl2	NP_001344656.1	371	4	11.36
Gykl1	NP_034423.2	1033	5	5.10	Mpzl3	NP_001087218.1	196	9	48.39
Sncaip	NP_001186080.1	1213	3	2.61	Jaml	XP_006510445.4	179	10	58.88
Snx2	NP_001344392.1	874	4	4.82	Scn2b	NP_001014761.1	773	7	9.54
Snx24	XP_011245297.1	302	1	3.49	Scn4b	NP_001013408.1	568	7	12.99
Ppic	NP_032934.1	1392	1	0.76	Tmprss4	NP_663378.1	458	6	13.81
Prdm6	XP_017173378.1	369	2	5.71	BC049352	XP_036010992.1	0	0	#DIV/0!
Cep120	XP_036017011.1	393	4	10.73	Il10ra	NP_001311415.1	923	6	6.85
Csnk1g3	XP_006526293.1	1081	2	1.95	Tmprss13	XP_006510195.1	256	5	20.58
Zfp608	NP_786927.2	343	4	12.29	Fxyd6	XP_030100346.1	285	7	25.89
Gramd3	NP_001347846.1	199	8	42.37	Fxyd2	NP_439888.1	290	4	14.54
Aldh7a1	NP_613066.2	997	4	4.23	Dscaml1	XP_006510010.1	702	2	3.00
Phax	NP_001156461.1	752	6	8.41	Cep164	XP_036010714.1	467	1	2.26
Tex43	XP_006526238.1	32	9	296.41	Bace1	NP_035922.4	1227	5	4.29
Lmnb1	NP_034851.2	1461	5	3.61	Rnf214	NP_001348043.1	82	1	12.85
Marchf3	NP_796089.1	513	10	20.54	Pcsk7	NP_032820.2	877	3	3.61
C330018D20R	NP_084185.1	87	8	96.91	Tagln	NP_035656.1	1021	2	2.06
Megf10	NP_001001979.1	483	3	6.55	Sidt2	NP_758461.1	226	10	46.63
Prrc1	NP_082723.2	233	8	36.19	Pafah1b2	NP_001344167.1	366	6	17.28
Ctxn3	NP_001128169.1	253	6	24.99	Sik3	NP_081774.3	275	9	34.49
Ccdc192	XP_017173492.1	0	0	#DIV/0!	Apoa1	NP_033822.2	1732	9	5.48
Slc12a2	NP_033220.2	780	5	6.76	Apoc3	NP_001276684.1	1003	8	8.41
Fbn2	XP_017173317.1	1056	6	5.99	Apoa4	NP_031494.2	1309	7	5.64
Slc27a6	NP_001074541.1	478	6	13.23	Apoa5	NP_001335024.1	992	7	7.44
Isoc1	NP_079754.2	357	9	26.57	Zpr1	NP_035882.1	783	7	9.42
Adamts19	XP_006525982.1	273	6	23.16	Bud13	NP_666112.1	633	8	13.32
Minar2	NP_001161397.2	266	7	27.73	4931429L15R	NP_898927.2	0	0	#DIV/0!
Chsy3	NP_001074797.1	210	4	20.07	Gm4791	NP_001230187.1	0	0	#DIV/0!
Gm4951	XP_006525988.1	65	2	32.43	Cadm1	NP_001297770.1	797	0	0.00
Gm4841	NP_001030031.2	66	0	0.00	Nxpe2	XP_006510756.1	110	2	19.16
F830016B08R	NP_001094945.1	68	0	0.00	Nxpe4	XP_036010876.1	68	0	0.00
Iigp1	XP_030106417.1	314	3	10.07	Rexo2	NP_001344430.1	416	0	0.00
Smim3	NP_598894.1	82	8	102.82	Rbm7	NP_659197.2	489	0	0.00
Dctn4	NP_001344391.1	590	10	17.86	Gm5617	NP_001004191.2	53	0	0.00
Rbm22	NP_080052.1	715	7	10.32	Nnmt	XP_006510140.1	668	0	0.00
Myoz3	NP_579941.2	201	7	36.70	Zbtb16	NP_001351472.1	1367	3	2.31
Synpo	XP_006525535.1	504	10	20.91	Htr3a	XP_011240700.1	819	5	6.43
Ndst1	XP_036016886.1	355	12	35.62	Htr3b	NP_064670.1	556	6	11.37
Rps14	NP_065625.2	1453	13	9.43	Usp28	XP_006510322.1	1078	4	3.91
Cd74	NP_001036070.1	1147	10	9.19	Cldn25	NP_001371194.1	108	0	0.00
Tcof1	XP_011245177.1	508	9	18.67	Zw10	NP_036169.1	553	3	5.72
Arsi	NP_001033588.1	225	10	46.84	Tmprss5	NP_001346389.1	160	6	39.52
Camk2a	XP_006525604.1	1621	3	1.95	Gm4894	NP_808369.1	34	0	0.00
Slc6a7	NP_958741.1	1064	3	2.97	Drd2	NP_034207.2	1555	7	4.74
Cdx1	NP_034010.3	771	8	10.94	Ankk1	NP_766510.1	987	5	5.34
Pdgfrb	NP_001139740.1	1700	7	4.34	Ttc12	XP_011240812.1	1039	6	6.09
Csf1r	NP_001032948.2	1557	11	7.45	Ncam1	NP_001074914.1	1928	5	2.73
Hmgxb3	NP_598895.2	78	0	0.00	Plet1	NP_083915.2	268	0	0.00
Slc26a2	NP_031911.1	587	6	10.77	Pts	NP_035350.1	670	0	0.00
Pde6a	NP_666198.1	542	2	3.89	Bco2	NP_001346539.1	469	4	8.99
Ppargc1b	NP_573512.1	561	3	5.64	Tex12	NP_079963.1	162	1	6.51
Arhgef37	NP_808496.2	359	2	5.87	Il18	XP_017168626.1	1254	2	1.68
Csnk1a1	NP_001344427.1	1145	2	1.84	Sdhd	NP_080124.1	806	3	3.92
Il17b	NP_062381.1	236	2	8.93	Timm8b	NP_038925.1	774	1	1.36
Pcyox1l	NP_766420.1	262	3	12.07	Nkapd1	XP_030100262.1	145	4	29.07
Grpel2	NP_067271.1	870	0	0.00	Pih1d2	XP_006510690.1	253	5	20.83
Afap1l1	NP_849259.2	186	5	28.33	Dlat	NP_663589.3	1062	5	4.96
Ablim3	XP_030106367.1	411	2	5.13	Dixdc1	XP_017168928.1	250	3	12.65
Sh3tc2	XP_006525944.1	224	1	4.70	2310030G06F	NP_080141.2	45	2	46.84

Adrb2	NP_031446.2	974	3	3.25	Hspb2	NP_001158180.1	1250	1	0.84
Gm9949	XP_036017013.1	0	0	#DIV/0!	Cryab	XP_030099896.1	995	4	4.24
Htr4	NP_001351886.1	367	3	8.61	1110032A03R	NP_001348037.1	60	5	87.83
Fbxo38	NP_001348017.1	182	2	11.58	Fdxacb1	NP_941077.2	769	0	0.00
Spink7	NP_001001803.2	158	3	20.01	Alg9	XP_006509964.1	307	8	27.46
Spink13	XP_006525523.2	217	1	4.86	Ppp2r1b	XP_006510705.3	1269	5	4.15
Spink10	XP_006526092.1	26	0	0.00	Sik2	XP_006510330.1	324	9	29.28
Apcdd1	NP_573500.2	198	1	5.32	Layn	XP_006510392.1	290	2	7.27
Napg	XP_011245108.1	402	1	2.62	Hoatz	XP_030100186.1	26	0	0.00
Piezo2	XP_006526232.1	284	2	7.42	Btg4	XP_017168979.1	530	2	3.98
Txnl1	NP_058072.2	627	2	3.36	Pou2af1	NP_035266.1	488	3	6.48
Wdr7	NP_001014981.1	800	1	1.32	Colca2	NP_001182610.1	60	7	122.96
St8sia3	XP_030106227.1	890	1	1.18	1810046K07R	NP_001359485.1	254	5	20.75
Onecut2	NP_919244.2	492	0	0.00	Arhgap20	NP_780744.2	372	4	11.33
Fech	NP_032024.2	1031	1	1.02	Fdx1	NP_032022.1	721	1	1.46
Nars	NP_081626.3	918	1	1.15	Rdx	NP_033067.2	1231	1	0.86
Atp8b1	NP_001001488.2	788	1	1.34	Zc3h12c	XP_006510396.1	285	0	0.00
Nedd4l	XP_036017211.1	1617	0	0.00	AI593442	NP_849237.2	441	1	2.39
Alpk2	NP_001032371.1	154	6	41.06	Ddx10	NP_084212.2	2003	6	3.16
Malt1	NP_001351948.1	340	1	3.10	Exph5	NP_789816.2	168	10	62.73
Zfp532	NP_001347982.1	430	7	17.16	Poglut3	NP_997610.1	154	11	75.28
Oacyl	XP_036017063.1	196	0	0.00	4930550C14R	NP_001363873.1	37	11	313.32
Sec11c	NP_079744.1	749	6	8.44	Atm	NP_031525.3	2459	7	3.00
Grp	XP_006525950.1	544	5	9.69	Npat	NP_001074621.1	397	12	31.86
Rax	NP_038861.2	458	2	4.60	Acat1	NP_659033.1	903	5	5.84
Cplx4	NP_663468.1	217	6	29.14	Cul5	NP_001155090.2	1604	10	6.57
Lman1	NP_081676.1	681	8	12.38	Rab39	NP_780771.1	1216	0	0.00
Ccbe1	NP_848908.1	307	0	0.00	Slc35f2	XP_006511524.1	319	10	33.04
Pmaip1	NP_067426.1	601	0	0.00	Sln	NP_079816.1	278	9	34.12
Mc4r	NP_058673.2	779	4	5.41	Elmod1	NP_808437.2	1153	10	9.14
Gnal	NP_796111.2	2370	6	2.67	Tnfaip8l3	NP_001028707.1	145	10	72.68
Chmp1b	NP_077152.1	499	6	12.67	Cyp19a1	NP_001335101.1	1101	6	5.74
Mppe1	XP_011245213.1	267	5	19.74	Gldn	NP_796324.1	398	6	15.89
Impa2	NP_444491.1	710	5	7.42	Dmxl2	XP_006511148.2	551	8	15.30
Cidea	NP_031728.2	510	2	4.13	Sh2d7	NP_776139.2	29	0	0.00
Tubb6	NP_080749.2	1384	5	3.81	Cib2	NP_062660.1	980	0	0.00
Afg3l2	NP_081406.1	1074	6	5.89	Idh3a	NP_083849.1	1128	1	0.93
Prelid3a	XP_006525958.1	179	5	29.44	Acsbg1	NP_444408.1	629	1	1.68
Spire1	XP_006526270.1	324	5	16.26	Dnaja4	NP_001344804.1	998	4	4.22
Gm17669	XP_036017248.1	558	0	0.00	Wdr61	NP_075680.1	968	1	1.09
Cep76	NP_001074542.1	297	8	28.39	Crabp1	NP_038524.1	609	0	0.00
Psmg2	NP_598899.1	513	5	10.27	Ireb2	NP_073146.2	1258	8	6.70
Ptpn2	XP_011245160.1	1061	5	4.97	Hykk	NP_796325.2	151	5	34.90
Seh1l	NP_001347863.1	654	6	9.67	AY074887	NP_660264.1	55	0	0.00
Cep192	XP_006526304.1	443	4	9.52	Psma4	XP_006511261.1	1515	7	4.87
Ldlrad4	XP_030106396.1	207	2	10.18	Chrna5	NP_789814.2	679	6	9.31
Fam210a	XP_030106132.1	97	3	32.59	Chrna3	NP_660111.3	887	7	8.32
Rnmt	NP_080716.1	473	4	8.91	Chrnb4	NP_683746.1	803	7	9.19
Mc5r	NP_038624.3	413	4	10.21	Ube2q2	NP_850931.2	470	1	2.24
Mc2r	NP_001258645.1	490	3	6.45	Fbxo22	NP_082325.2	526	2	4.01
Tcf4	NP_001348056.1	1005	3	3.15	Nrg4	XP_017169193.1	154	0	0.00
Ccdc68	XP_006526133.1	146	7	50.53	Tmem266	NP_766511.1	30	2	70.26
Rab27b	NP_085031.3	480	5	10.98	Etfa	NP_663590.3	985	2	2.14
Dynap	NP_083622.1	5	0	0.00	Isl2	XP_006510819.1	633	8	13.32
4930503L19Ri	NP_001366359.1	82	3	38.56	Scaper	NP_001344581.1	151	5	34.90
Stard6	NP_083295.1	132	12	95.81	4930563M21I	XP_017169163.1	13	0	0.00
Poli	XP_011245235.1	1140	0	0.00	Rcn2	NP_036122.2	383	1	2.75
Mbd2	NP_034903.2	437	2	4.82	Pstpip1	XP_011240979.1	859	6	7.36
Dcc	XP_006525651.1	660	6	9.58	Tspan3	NP_062767.3	569	8	14.82
Mex3c	NP_001034303.3	706	11	16.42	Peak1	NP_766512.2	177	5	29.77
Smad4	NP_001351897.1	1803	11	6.43	Hmg20a	NP_080088.1	728	1	1.45
Elac1	NP_444485.2	563	11	20.59	Lingo1	XP_006511149.1	1168	3	2.71
Me2	XP_006525559.1	588	7	12.55	Odf3l1	NP_001361610.1	60	0	0.00
Mro	NP_001347902.1	213	6	29.69	Cspg4	NP_620570.2	616	2	3.42
Mapk4	XP_030106306.1	2209	9	4.29	Snx33	XP_006511154.1	378	1	2.79
Ska1	NP_079857.3	675	5	7.81	Imp3	NP_598737.1	1306	1	0.81

Cxxc1	NP_001365773.1	752	7	9.81	Snupn	NP_848461.1	238	3	13.28
Mbd1	NP_001344352.1	173	2	12.18	Ptpn9	NP_062625.2	530	6	11.93
Cfap53	NP_083224.2	150	3	21.08	Sin3a	NP_001103820.1	2195	1	0.48
Myo5b	XP_006525777.1	1500	11	7.73	Man2c1	XP_006511557.1	404	4	10.43
Acaa2	NP_803421.1	896	1	1.18	Neil1	NP_001344338.1	275	1	3.83
Lipg	NP_034850.3	469	5	11.24	Commd4	XP_006511406.1	459	1	2.30
Rpl17	XP_017173407.1	1125	1	0.94	Trcg1	NP_001014420.2	866	1	1.22
BC031181	NP_001345235.1	91	1	11.58	1700017B05R	NP_083096.1	24	0	0.00
Dym	XP_030106454.1	153	6	41.33	Ppcdc	XP_030100363.1	1217	2	1.73
Smad7	NP_001036125.1	1141	6	5.54	Scamp5	NP_001288563.1	421	4	10.01
Ctif	XP_030106343.1	295	7	25.01	Rpp25	NP_598743.1	293	3	10.79
Zbtb7c	XP_036016934.1	277	3	11.41	Cox5a	NP_031773.2	806	5	6.54
Smad2	NP_034884.2	1852	4	2.28	Fam219b	XP_006511621.3	37	1	28.48
Skor2	NP_001103213.1	156	8	54.05	Mpi	NP_080113.1	685	3	4.62
Ier3ip1	NP_079685.1	251	6	25.19	Scamp2	NP_001365857.1	416	3	7.60
Hdhd2	NP_001348156.1	260	5	20.27	Ulk3	NP_082171.1	649	5	8.12
Katnal2	NP_001355585.1	906	5	5.82	Cplx3	NP_666335.1	257	4	16.40
Pias2	XP_006526486.1	870	7	8.48	Lman1l	XP_036010794.1	295	6	21.44
St8sia5	XP_006526492.1	413	8	20.41	Csk	XP_030099897.1	1479	3	2.14
Loxhd1	XP_006526497.1	219	3	14.44	Cyp1a2	NP_034123.1	1272	5	4.14
Rnf165	NP_001157976.1	399	2	5.28	Cyp1a1	NP_001129531.1	1009	7	7.31
8030462N17R	NP_001355688.1	65	3	48.64	Edc3	XP_036010982.1	596	3	5.30
Haus1	NP_001344460.1	577	0	0.00	Clk3	XP_036010394.1	722	8	11.68
Atp5a1	NP_031531.1	1929	0	0.00	Arid3b	NP_001366282.1	567	8	14.87
Pstpip2	XP_036016927.1	700	0	0.00	Ubl7	NP_081362.2	1872	10	5.63
Epg5	NP_001182562.1	163	0	0.00	Sema7a	NP_035482.1	448	5	11.76
Siglec15	XP_030106421.1	131	1	8.05	Cyp11a1	NP_062753.3	621	6	10.18
Slc14a1	NP_001164481.1	316	1	3.34	Ccdc33	NP_001159754.1	150	8	56.21
Slc14a2	NP_997534.2	363	1	2.90	Stra6	XP_017168751.1	433	3	7.30
Setbp1	NP_444329.2	1122	2	1.88	Islr	XP_006511267.1	757	6	8.35
Pard6g	NP_444347.3	602	3	5.25	Islr2	NP_001155007.1	767	5	6.87
Adnp2	NP_778193.1	164	2	12.85	Pml	XP_006510924.1	783	0	0.00
Rbfa	NP_954667.1	104	0	0.00	Stoml1	NP_081218.3	696	3	4.54
Gm16286	NP_001371105.1	443	0	0.00	Loxl1	NP_034859.2	512	4	8.23
Txnl4a	NP_079575.1	771	1	1.37	Tbc1d21	NP_001344384.1	601	1	1.75
Hsbp1l1	NP_001129653.1	13	0	0.00	Insyn1	NP_795895.1	244	1	4.32
Slc66a2	XP_030106435.1	136	2	15.50	Cd276	NP_598744.1	565	3	5.60
Kcng2	XP_017173393.1	627	0	0.00	Nptn	XP_006510945.1	311	2	6.78
Ctdp1	NP_080571.2	733	8	11.50	Rec114	XP_036011226.1	143	1	7.37
Nfatc1	NP_001157582.1	1006	3	3.14	Hcn4	NP_001074661.1	650	1	1.62
Atp9b	NP_056620.2	383	7	19.26	Neo1	XP_036010572.1	756	5	6.97
Sall3	NP_840064.2	424	5	12.43	Adpgk	NP_082397.1	432	8	19.52
Galr1	NP_032108.1	488	10	21.60	Bbs4	NP_001346487.1	1233	6	5.13
Mbp	XP_030106216.1	1391	2	1.52	Arih1	NP_064311.2	713	7	10.35
Zfp236	NP_808500.2	336	5	15.68	Tmem202	NP_848475.1	32	5	164.67
Zfp516	XP_030106376.1	346	6	18.28	Hexa	NP_034551.2	274	1	3.85
Tshz1	NP_001074769.1	337	8	25.02	Celf6	XP_006511603.1	456	3	6.93
Zadh2	NP_666202.2	360	7	20.49	Parp6	XP_017169016.1	163	7	45.26
Zfp407	NP_001028513.2	323	10	32.63	Pkm	XP_011240976.2	2055	5	2.56
Cndp1	NP_803233.2	659	3	4.80	Gramd2	XP_036011013.1	149	6	42.44
Cndp2	XP_030106430.1	855	3	3.70	Senp8	NP_001165542.1	260	1	4.05
Dipk1c	XP_006526536.1	110	4	38.32	Myo9a	XP_006511271.1	1392	4	3.03
Cyb5a	NP_080073.1	1258	3	2.65	Nr2e3	XP_006511212.1	602	3	5.25
Fbxo15	XP_011245388.1	638	2	3.67	Thsd4	XP_036010652.1	491	1	2.15
Timm21	NP_080245.1	311	2	7.97	Lrrc49	NP_001139518.1	746	3	4.24
Neto1	XP_036017044.1	872	5	7.55	Larp6	NP_080511.2	579	4	7.28
Cbln2	NP_001348071.1	494	1	2.84	Uaca	XP_017169098.1	1050	1	1.00
Socs6	XP_030106401.1	529	2	5.69	Tle3	NP_001077396.1	942	4	4.48
Rttn	NP_780751.2	266	5	30.48	Rplp1	NP_061341.1	885	1	1.19
Cd226	XP_006526524.1	442	3	11.92	Kif23	XP_030100479.1	1737	7	4.25
Dok6	NP_001034262.1	382	4	20.06	Paqr5	NP_083024.1	203	1	5.19
Tmx3	NP_938037.2	893	3	7.08	Glce	NP_001355233.1	219	1	4.81
Ighmbp2	NP_033238.2	835	2	5.05	Spesp1	NP_079997.1	176	0	0.00
Mrpl21	NP_758456.2	849	4	9.03	Anp32a	NP_033802.2	770	0	0.00
Cpt1a	XP_011246904.1	839	6	12.56	Coro2b	NP_780693.2	969	3	3.26
Tesmin	NP_001034746.1	322	2	10.07	Itga11	NP_795896.4	468	3	6.76

Gal	NP_001316596.1	650	1	2.32	Fem1b	NP_034323.1	1167	3	2.71
Ppp6r3	XP_036017520.1	539	3	7.82	Cln6	NP_001028347.1	208	2	10.13
Lrp5	NP_032539.2	870	6	9.09	Calml4	XP_006511592.1	1084	3	2.92
1810055G02R	NP_001345186.1	39	2	63.58	Pias1	NP_062637.2	1005	6	6.29
Kmt5b	XP_017173633.1	600	2	3.90	Skor1	NP_001157229.1	253	8	33.32
Chka	XP_006531709.1	608	3	5.47	Map2k5	XP_006511209.1	1271	4	3.32
Tcirg1	NP_001129563.1	726	6	8.71	2300009A05F	NP_081366.1	34	2	61.99
Ndufs8	NP_001258372.1	1106	10	9.53	Iqch	XP_006511616.1	113	5	46.63
Aldh3b1	NP_080592.2	1014	8	8.31	Aagab	NP_080133.1	123	2	17.14
Unc93b1	NP_062322.2	646	2	3.26	Smad3	NP_058049.3	1871	4	2.25
Aldh3b3	XP_006531905.1	623	4	6.77	Smad6	NP_032568.3	876	7	8.42
Aldh3b2	NP_001170909.1	686	3	4.61	Lctl	NP_665834.1	301	1	3.50
Acy3	NP_001289408.1	383	3	8.26	Zwilch	NP_080783.3	677	1	1.56
Tbx10	NP_001001320.1	404	1	2.61	Rpl4	XP_036011111.1	1679	3	1.88
Nudt8	NP_079805.1	508	6	12.45	Snapc5	NP_899139.2	155	4	27.20
Doc2g	NP_068563.2	105	0	0.00	Map2k1	NP_032953.1	2203	8	3.83
Ndufv1	NP_598427.1	1025	7	7.20	Uchl4	NP_291085.1	489	1	2.16
Gstp1	NP_038569.1	864	7	8.54	Tipin	NP_001311485.1	973	3	3.25
Gstp2	NP_861461.1	400	4	10.54	Dis3l	NP_001001295.2	845	5	6.24
Gstp3	NP_659118.1	357	5	14.76	Megf11	XP_036010678.1	314	1	3.36
Cabp2	XP_006531826.1	1043	0	0.00	Rab11a	NP_059078.2	2738	4	1.54
Cdk2ap2	NP_080649.1	267	2	7.89	Dennd4a	XP_030099796.1	256	4	16.47
Pitpnm1	NP_032877.1	537	1	1.96	Slc24a1	XP_030100076.1	317	1	3.32
Aip	XP_030106569.1	367	2	5.74	Ints14	NP_780362.2	154	9	61.59
Cabp4	NP_653115.1	1140	2	1.85	Hacd3	NP_067320.2	407	3	7.77
Gpr152	NP_996856.1	143	1	7.37	Dpp8	XP_036011253.1	222	4	18.99
Coro1b	XP_006531804.1	867	5	6.08	Igdcc4	XP_036011045.1	350	6	18.07
Ptprcap	NP_058629.2	232	5	22.71	Igdcc3	NP_001344186.1	867	1	1.22
Rps6kb2	NP_067460.1	1276	2	1.65	Parp16	NP_803411.3	199	5	26.48
Carns1	NP_598909.2	590	2	3.57	Cilp	NP_775561.1	317	0	0.00
Tbc1d10c	NP_848765.2	538	4	7.84	Clpx	NP_035932.2	922	5	5.72
Ppp1ca	NP_114074.1	3064	10	3.44	Pdcd7	NP_057897.2	170	0	0.00
Rad9a	NP_035367.1	586	1	1.80	Ubap1l	NP_001104615.1	0	0	#DIV/0!
Clcf1	NP_064336.1	258	0	0.00	Kbtbd13	NP_083250.1	430	1	2.45
Pold4	NP_081472.1	218	0	0.00	Rasl12	NP_001156395.1	1405	4	3.00
Ssh3	NP_001343549.1	660	2	3.19	Slc51b	NP_849264.1	196	0	0.00
Ankrd13d	NP_080996.2	116	0	0.00	Mtfmt	NP_081410.2	562	1	1.88
Grk2	XP_036017294.1	1996	1	0.53	Spg21	NP_001344742.1	307	0	0.00
Kdm2a	NP_001001984.2	1718	3	1.84	Ankdd1a	NP_001357800.1	1071	2	1.97
Rhod	NP_031511.1	1946	6	3.25	Plekho2	NP_694759.1	109	1	9.67
Syt12	NP_598925.1	407	0	0.00	Pif1	XP_036010658.1	862	1	1.22
2010003K11R	NP_081513.1	329	1	3.20	Rbpms2	NP_082306.2	394	0	0.00
Pcx	NP_001156418.1	1264	1	0.83	Oaz2	NP_035082.1	193	0	0.00
Lrfn4	XP_017173631.1	800	3	3.95	Zfp609	NP_766124.2	228	0	0.00
Rce1	NP_001355641.1	284	1	3.71	Trip4	NP_001344801.1	330	3	9.58
Gm960	XP_036017510.1	65	0	0.00	Pclaf	NP_080791.2	700	1	1.51
Sptbn2	NP_067262.1	1373	3	2.30	Csnk1g1	XP_030100085.1	758	4	5.56
Rbm4b	XP_030106890.1	463	1	2.28	Ppib	NP_035279.2	1627	0	0.00
Rbm4	NP_001277053.1	444	2	4.75	Snx22	NP_001366265.1	235	1	4.48
Gm21992	NP_001277057.1	434	2	4.86	Snx1	NP_062701.2	838	5	6.29
Rbm14	NP_063922.2	675	1	1.56	Ciao2a	NP_080911.1	298	0	0.00
Ccs	NP_058588.1	443	0	0.00	Dapk2	NP_034149.2	702	1	1.50
Ccdc87	NP_997151.2	17	0	0.00	Herc1	XP_006511166.1	882	4	4.78
Ctsf	NP_063914.1	481	0	0.00	Fbxl22	XP_036011252.1	777	3	4.07
Actn3	NP_038484.1	1262	2	1.67	Usp3	NP_001289045.1	650	4	6.49
Zdhhc24	NP_081752.2	101	0	0.00	Car12	XP_006511611.1	432	1	2.44
Bbs1	NP_001028300.1	600	1	1.76	Aph1b	NP_808251.1	229	1	4.60
Dpp3	NP_001347640.1	431	2	4.89	Aph1c	NP_080950.1	217	1	4.86
Peli3	NP_001334351.1	219	0	0.00	Rab8b	NP_775589.1	969	6	6.53
Mrpl11	XP_006531884.1	985	1	1.07	Rps27l	NP_080743.1	1064	2	1.98
Npas4	XP_030106732.1	497	1	2.12	Lactb	NP_109642.1	374	4	11.27
Slc29a2	NP_031880.2	369	1	2.86	Tpm1	NP_077745.2	1471	3	2.15
B4gat1	NP_780592.1	125	0	0.00	Tln2	XP_011241115.1	757	1	1.39
Brms1	NP_001360976.1	431	5	12.23	C2cd4b	NP_001074783.1	133	1	7.92
Rin1	XP_006531773.1	380	5	13.87	C2cd4a	NP_001156615.1	141	1	7.47
Cd248	NP_473383.1	644	0	0.00	Vps13c	NP_796158.2	628	3	5.03

Tmem151a	NP_001001885.1	454	1	2.32	Rora	NP_038674.1	846	2	2.49
Yif1a	NP_080829.1	406	3	7.79	Ice2	XP_036011313.1	473	1	2.23
Cnih2	NP_034050.1	771	5	6.83	Anxa2	XP_006510859.1	1326	5	3.97
Rab1b	NP_083852.1	1150	9	8.25	Foxb1	NP_071773.2	561	0	0.00
Klc2	NP_032477.2	1146	3	2.76	Bnip2	XP_006510854.2	243	0	0.00
Pacs1	NP_694769.2	555	10	18.99	Gtf2a2	NP_001269013.1	592	2	3.56
Sf3b2	NP_001349383.1	1127	2	1.87	Gcnt3	NP_082363.2	313	2	6.73
Gal3st3	NP_001019888.1	66	8	127.75	Fam81a	XP_006511613.1	153	4	27.55
Catsper1	NP_647462.1	668	6	9.47	Myo1e	NP_851417.2	1263	8	6.68
Cst6	NP_082899.1	249	6	25.40	Ccnb2	NP_031656.2	2470	1	0.43
Banf1	NP_001273537.1	1302	3	2.43	Rnf111	XP_006511644.1	786	1	1.34
Eif1ad	XP_030106933.1	343	2	6.15	Sltm	XP_006511412.1	595	2	3.54
Sart1	NP_058578.3	1190	6	5.31	Mindy2	NP_001361615.1	154	4	27.37
Tsga10ip	NP_001360907.1	12	0	0.00	Adam10	NP_031425.2	1237	3	2.56
Drap1	NP_077138.1	405	2	5.20	Lipc	NP_001311401.1	956	5	5.51
AI837181	NP_598910.2	87	1	12.11	Aqp9	NP_071309.1	999	4	4.22
Fosl1	NP_034365.1	1074	6	5.89	Aldh1a2	NP_033048.2	1129	1	0.93
Ccdc85b	NP_941018.1	162	1	6.51	Polr2m	NP_848717.1	142	1	7.42
Fibp	NP_001240761.1	258	3	12.25	Myzap	NP_001028380.1	86	1	12.25
Ctsw	NP_034115.2	405	2	5.20	Cgnl1	XP_036011116.1	328	0	0.00
Efemp2	XP_006531870.1	672	3	4.70	Tcf12	XP_006511061.1	1000	4	4.22
Mus81	NP_082153.3	703	9	13.49	Zfp280d	NP_666336.3	522	4	8.08
Cfl1	NP_031713.1	2021	5	2.61	Mns1	XP_036010534.1	333	3	9.49
Snx32	XP_011246919.1	257	5	20.50	Tex9	XP_006511085.1	131	1	8.05
Nscme3l	NP_076270.3	157	1	6.71	Rfx7	XP_006511286.1	580	3	5.45
Ovol1	NP_064319.1	431	7	17.12	Nedd4	NP_035020.2	1891	3	1.67
Ap5b1	NP_001348975.1	253	1	4.17	Prtg	NP_780694.3	274	1	3.85
Rnaseh2c	NP_080892.1	266	2	7.92	Pygo1	NP_082392.1	286	1	3.68
Kat5	NP_001186176.1	1589	5	3.32	Dnaaf4	NP_080590.3	995	5	5.30
Rela	NP_033071.1	1663	8	5.07	Ccpg1os	NP_001185718.1	0	0	#DIV/0!
Sipa1	XP_030106688.1	449	8	18.78	Ccpg1	XP_036011209.1	161	0	0.00
Pcnx3	XP_030106542.1	191	4	22.07	Pigb	XP_006511350.1	203	0	0.00
Map3k11	XP_030106811.1	1194	13	11.47	Rab27a	NP_076124.1	665	3	4.75
Kcnk7	NP_034739.2	205	12	61.69	Khdc3	XP_006511437.1	289	0	0.00
Ehbp1l1	NP_001108067.1	916	10	11.51	Rsl24d1	NP_941011.1	1057	1	1.00
Fam89b	NP_075655.2	99	10	106.45	Unc13c	XP_006510997.1	713	2	2.96
Znrd2	NP_065237.3	515	11	22.51	Wdr72	NP_001028672.2	621	3	5.09
Ltbp3	XP_006531731.1	540	11	21.47	Onecut1	NP_032288.1	731	0	0.00
Scyl1	XP_030107005.1	326	2	6.47	Fam214a	NP_001346745.1	129	4	32.68
Frmd8	NP_080445.1	296	11	39.17	Arpp19	NP_001136127.1	372	3	8.50
Slc25a45	NP_001348902.1	233	11	49.75	Myo5a	XP_006510890.1	1687	9	5.62
Tigd3	NP_001348974.1	125	10	84.31	Myo5c	XP_006510998.1	1282	8	6.58
Dpf2	NP_035392.1	419	11	27.67	Gnb5	NP_034443.1	1557	5	3.38
Cdc42ep2	NP_081048.1	192	10	54.89	Bcl2l10	NP_038507.1	761	0	0.00
Pola2	NP_032919.2	704	2	2.99	Mapk6	XP_011241069.1	2248	7	3.28
Slc22a20	NP_941052.1	262	1	4.02	Leo1	NP_001034611.1	1204	2	1.75
Capn1	NP_001103974.1	706	2	2.99	Tmod3	NP_058659.1	492	5	10.71
Syvn1	NP_083045.4	857	1	1.23	Tmod2	XP_030100304.1	862	5	6.11
Mrpl49	NP_080522.1	510	1	2.07	Lysmd2	NP_081585.2	34	7	216.98
Fau	NP_001153711.1	3273	8	2.58	Scg3	XP_017168709.1	967	3	3.27
Znhit2	NP_038887.2	424	2	4.97	Bmp5	NP_031581.2	736	6	8.59
Tm7sf2	NP_082730.2	704	3	4.49	Hmgcll1	NP_776092.1	578	2	3.65
Vps51	NP_001074510.1	383	1	2.75	Gfral	NP_995316.2	149	2	14.15
Tmem262	XP_006531849.1	2	0	0.00	Hcrtr2	NP_945200.1	335	0	0.00
Zfpl1	NP_001343923.1	217	0	0.00	Fam83b	NP_001038983.1	147	2	14.34
Cdca5	NP_080686.1	673	2	3.13	Tinag	XP_006511264.1	310	8	27.20
Naaladl1	NP_001009546.1	290	1	3.63	Mlip	NP_001355917.1	339	5	15.54
Sac3d1	NP_598439.3	670	1	1.57	Lrrc1	XP_030100078.1	771	5	6.83
Snx15	NP_081188.1	521	1	2.02	Klhl31	NP_766513.2	568	4	7.42
Arl2	NP_062696.2	384	0	0.00	Gclc	NP_034425.1	818	9	11.60
Batf2	NP_083243.1	577	1	1.83	Elovl5	XP_030100414.1	667	9	14.22
Majin	NP_001159391.1	36	0	0.00	Gcm1	NP_032129.2	433	3	7.30
Gpha2	NP_569720.1	224	1	4.70	Fbxo9	NP_001074959.1	647	1	1.63
Ppp2r5b	XP_036017413.1	934	7	7.90	Cilk1	NP_064371.2	517	6	12.23
Atg2a	XP_006531835.1	480	2	4.39	Gsta4	NP_034487.2	710	4	5.94
Ehd1	NP_034249.1	674	3	4.69	Gsta1	NP_032207.3	480	3	6.59

Cdc42bpg	NP_001028514.1	1106	6	5.72	Gm3776	NP_001230021.1	480	3	6.59
Men1	NP_001161960.1	736	5	7.16	Gm10639	NP_001116132.1	1465	7	5.04
Map4k2	NP_033032.1	331	5	15.92	Omt2a	NP_001157995.1	209	0	0.00
Sf1	XP_006531800.1	911	4	4.63	Omt2b	NP_991391.2	218	1	4.83
Pygm	NP_035354.1	1033	5	5.10	Gsta2	NP_032208.2	644	2	3.27
Rasgrp2	NP_035372.2	700	5	7.53	Dppa5a	NP_079550.2	299	3	10.57
Nrxn2	NP_001356292.1	1107	4	3.81	Ooep	NP_080756.1	189	4	22.30
Slc22a12	NP_033229.3	310	2	6.80	Ddx43	NP_001177973.1	1339	6	4.72
Rps6ka4	NP_064308.2	445	3	7.10	Cgas	NP_775562.2	386	3	8.19
Ccdc88b	XP_036017624.1	232	1	4.54	Mto1	NP_080934.2	688	1	1.53
Prdx5	NP_036151.1	742	3	4.26	Eef1a1	NP_034236.2	2110	5	2.50
Trmt112	NP_080582.3	752	3	4.20	Slc17a5	NP_001263381.1	425	3	7.44
Esrra	XP_006527174.1	654	3	4.83	Cd109	NP_694738.1	610	3	5.18
Catsperz	NP_001034583.1	56	2	37.64	Col12a1	XP_006510863.1	781	5	6.75
Kcnk4	XP_006526780.1	273	2	7.72	Cox7a2	NP_034075.2	623	2	3.38
Gpr137	NP_001170832.1	251	2	8.40	Tmem30a	NP_598479.1	825	2	2.55
Bad	NP_031548.1	426	1	2.47	Filip1	XP_006511506.1	193	4	21.84
Plcb3	XP_006526830.1	916	2	2.30	Senp6	NP_001298039.1	804	6	7.86
Ppp1r14b	NP_032915.2	461	2	4.57	Myo6	XP_006510898.1	1435	3	2.20
Fkbp2	NP_001347217.1	1228	2	1.72	Impg1	NP_071299.3	197	6	32.10
Vegfb	NP_001172093.1	398	4	10.59	Htr1b	XP_030099923.1	682	3	4.64
Dnajc4	XP_030106867.1	290	3	10.90	Mei4	NP_001344406.1	28	1	37.64
Nudt22	NP_080951.1	118	2	17.86	Irak1bp1	NP_075362.3	229	6	27.61
Trpt1	XP_036017277.1	92	1	11.46	Phip	XP_006511627.1	1351	6	4.68
Fermt3	NP_001349328.1	877	1	1.20	Hmgn3	NP_080398.1	208	3	15.20
Stip1	NP_058017.1	1362	1	0.77	Lca5	NP_083710.2	309	2	6.82
Macrod1	NP_001365848.1	410	2	5.14	Sh3bgrl2	NP_766095.1	148	7	49.85
Flrt1	NP_958813.1	755	0	0.00	Elovl4	NP_683743.2	789	3	4.01
Otub1	NP_598911.1	492	0	0.00	Ttk	XP_006511121.1	1041	1	1.01
Cox8a	NP_031776.1	477	2	4.42	Bckdhb	NP_001292864.1	790	2	2.67
Naa40	XP_006527399.1	236	1	4.47	Tent5a	NP_001153850.1	457	2	4.61
Rcor2	XP_006526614.1	845	0	0.00	Ibtk	XP_006510828.1	519	2	4.06
Mark2	NP_001073857.1	479	3	6.60	Tpbg	XP_017168769.1	666	0	0.00
Spindoc	NP_001028311.2	107	3	29.55	Ube2cbp	XP_006511495.1	342	9	27.73
2700081O15R	XP_011245417.1	172	3	18.38	Dop1a	XP_017168922.1	425	10	24.80
Rtn3	NP_444306.1	684	3	4.62	Pgm3	XP_036010453.1	815	13	16.81
Atl3	XP_006526667.1	311	2	6.78	Rwdd2a	NP_081376.2	411	11	28.21
Plaat3	NP_001349354.1	349	0	0.00	Me1	NP_032641.2	709	2	2.97
Lgals12	XP_006527284.1	162	0	0.00	Prss35	NP_848853.2	170	13	80.59
Plaat5	NP_001355625.1	300	0	0.00	Snap91	NP_001264914.1	1663	12	7.60
Slc22a19	XP_006526877.1	261	1	4.04	Ripply2	NP_001366172.1	100	12	126.47
Slc22a26	NP_666344.1	460	2	4.58	Cyb5r4	NP_077157.2	1378	14	10.71
Slc22a27	XP_017173565.1	403	2	5.23	Mrap2	NP_001346884.1	240	11	48.30
Slc22a28	XP_030106844.1	379	1	2.78	Cep162	NP_955020.2	328	14	44.98
Slc22a29	XP_006527123.1	345	5	15.27	Tbx18	NP_076303.3	570	9	16.64
Slc22a30	XP_006527198.1	460	2	4.58	Nt5e	NP_035981.1	969	8	8.70
Slc22a8	NP_112471.3	398	2	5.30	Snx14	NP_766514.3	485	10	21.73
Slc22a6	NP_032792.2	419	3	7.55	Syncrip	NP_001271257.1	1344	8	6.27
Chrm1	NP_001106167.1	845	3	3.74	Zfp949	XP_017169085.1	161	1	6.55
Slc3a2	NP_001154885.1	1015	5	5.19	Trim43a	NP_001344616.1	453	0	0.00
Wdr74	NP_598900.1	852	2	2.47	Mthfsl	XP_006510793.1	133	2	15.85
1700092M07F	NP_001170818.1	0	0	#DIV/0!	Bcl2a1d	NP_031562.1	561	1	1.88
Stx5a	XP_030106858.1	700	5	7.53	Trim43c	NP_001171329.1	317	0	0.00
Nxf1	NP_058093.2	995	2	2.12	Bcl2a1a	NP_033872.1	758	3	4.17
Tmem223	NP_080067.1	995	2	2.12	Trim43b	NP_001164355.1	256	0	0.00
Tmem179b	NP_080601.2	343	1	3.07	Bcl2a1b	NP_031560.2	770	2	2.74
Taf6l	NP_001171269.1	343	1	3.07	Mthfs	NP_081105.1	176	2	11.98
Polr2g	NP_080605.1	1261	5	4.18	Minar1	XP_017168754.1	103	2	20.46
Zbtb3	NP_001091707.1	338	7	21.83	Tmed3	NP_079636.2	461	1	2.29
Ttc9c	NP_081688.2	213	3	14.84	Ankrd34c	NP_997143.1	64	2	32.93
Hnrnpul2	NP_001074665.1	547	8	15.41	Rasgrf1	NP_035375.1	1440	5	3.66
Gng3	NP_034446.1	1209	3	2.62	Ctsh	NP_031827.2	731	4	5.77
Bscl2	NP_001129536.1	402	10	26.22	Morf4l1	NP_077751.1	956	2	2.20
Lrrn4cl	NP_001347557.1	0	0	#DIV/0!	Adamts7	NP_001313280.1	400	1	2.63
Ubxn1	NP_001348973.1	454	2	4.64	Tbc1d2b	NP_919315.2	804	2	2.62
Uqcc3	NP_001153828.1	208	1	5.07	Zic1	NP_001363870.1	1020	2	2.07

Lbhd1	NP_001273032.1	8	0	0.00	Zic4	XP_036010741.1	651	1	1.62
1810009A15R	NP_079739.1	218	0	0.00	Plscr5	NP_001182622.1	216	0	0.00
Ints5	NP_789813.1	313	10	33.67	Plscr1	NP_035766.2	477	3	6.63
Ganab	NP_032086.1	760	5	6.93	Plscr2	XP_006510920.1	299	1	3.52
B3gat3	NP_077218.1	293	1	3.60	1700057G04R	XP_011241139.1	292	1	3.61
Rom1	NP_033099.3	382	5	13.79	Plscr4	NP_848826.1	312	4	13.51
Eml3	XP_036017433.1	692	5	7.61	Plod2	NP_001136388.1	659	8	12.79
Mta2	NP_035972.3	435	0	0.00	Dipk2a	NP_001028317.2	128	1	8.23
Tut1	NP_932110.1	484	2	4.35	Slc9a9	NP_808577.3	666	8	12.66
Eef1g	NP_080283.3	2117	7	3.48	Chst2	NP_061233.2	311	4	13.55
Ahnak	NP_001035048.1	707	6	8.94	U2surp	NP_001344904.1	769	7	9.59
Scgb1a1	NP_035811.1	580	3	5.45	Paqr9	NP_940806.2	300	0	0.00
Asrgl1	NP_079886.2	401	2	5.26	Pcolce2	NP_083896.1	243	4	17.35
Scgb2a2	NP_001365339.1	155	0	0.00	Trpc1	XP_006511116.1	595	3	5.31
Incenp	XP_006526771.2	786	2	2.68	Pls1	XP_006510809.1	1127	7	6.55
Fth1	NP_034369.1	850	8	9.92	Atr	NP_063917.1	2668	2	0.79
Best1	NP_036043.2	475	6	13.31	Xrn1	NP_036046.2	1460	0	0.00
Rab3il1	XP_006527477.1	416	7	17.73	Gk5	NP_796326.1	796	0	0.00
Fads3	NP_068690.3	405	7	18.22	Tfdp2	XP_006511024.1	658	5	8.01
Fads2	NP_062673.1	721	9	13.16	Atp1b3	NP_001344141.1	432	1	2.44
Fads1	NP_666206.1	687	9	13.81	Rnf7	NP_035409.1	1154	3	2.74
Fen1	NP_001258543.1	1703	7	4.33	Rasa2	NP_444498.2	492	1	2.14
Tmem258	NP_081195.1	241	9	39.36	Zbtb38	XP_006511252.1	494	3	6.40
Myrf	XP_036017438.1	339	7	21.76	Pxylp1	XP_017168835.1	264	0	0.00
Dagla	XP_017173702.1	419	8	20.12	Spsb4	NP_660116.1	565	1	1.87
Syt7	XP_006527267.1	649	6	9.74	Slc25a36	NP_620095.1	502	4	8.40
Lrrc10b	NP_001104610.1	503	2	4.19	Trim42	NP_084495.2	430	0	0.00
Ppp1r32	XP_017173770.1	88	0	0.00	Clstn2	NP_071714.2	784	2	2.69
Sdhaf2	NP_079609.2	324	0	0.00	Nmnat3	NP_001344380.1	361	3	8.76
Cpsf7	NP_758506.3	550	0	0.00	Rbp1	NP_035384.1	332	1	3.17
Tmem216	NP_001264789.1	341	1	3.09	Rbp2	NP_033060.3	318	1	3.31
Tmem138	NP_082687.1	136	2	15.50	Copb2	NP_056642.1	948	3	3.34
Cyb561a3	XP_017173636.1	212	1	4.97	Mrps22	NP_079761.1	722	3	4.38
Tkfc	XP_006527004.1	372	1	2.83	Prr23a1	NP_001128132.1	40	1	26.35
Ddb1	XP_006526702.1	1593	1	0.66	Prr23a2	NP_001128133.1	30	0	0.00
Vwce	NP_082189.1	1029	2	2.05	Prr23a3	NP_082309.1	27	0	0.00
Pga5	NP_067428.2	479	0	0.00	7420426K07R	NP_001029155.1	201	0	0.00
Vps37c	NP_001348890.1	279	1	3.78	Foxl2	NP_036150.1	888	3	3.56
Cd5	XP_006526684.1	819	4	5.15	Faim	NP_001116323.1	205	0	0.00
Cd6	XP_036017308.1	341	2	6.18	Gm1123	NP_001074245.1	136	0	0.00
Slc15a3	NP_075531.2	633	2	3.33	Pik3cb	NP_083370.2	2108	2	1.00
Tmem132a	NP_598565.2	316	1	3.34	Faiml	NP_001231691.1	79	1	13.34
Tmem109	NP_598903.1	189	2	11.15	Cep70	NP_076362.2	416	0	0.00
Prpf19	NP_001240772.1	1727	2	1.22	Esyt3	NP_808443.2	323	2	6.53
Zp1	NP_033606.2	259	1	4.07	Mras	XP_030099940.1	1623	3	1.95
Ptgdr2	XP_006526758.1	468	1	2.25	Armc8	XP_036011248.1	384	3	8.23
Ccdc86	NP_076220.2	332	1	3.17	Dbr1	NP_113580.2	402	0	0.00
Ms4a10	NP_001366456.1	5	1	210.78	A4gnt	XP_006511307.3	134	2	15.73
Ms4a15	XP_006527275.1	43	3	73.53	Dzip1l	XP_006511535.1	164	2	12.85
Ms4a18	XP_006527492.1	33	2	63.87	Cldn18	NP_001181850.1	286	2	7.37
Ms4a8a	NP_071875.2	136	6	46.50	Sox14	NP_035570.1	446	6	14.18
Ms4a20	XP_006527388.1	1	0	0.00	Il20rb	NP_001028715.2	243	3	13.01
Ms4a19	NP_081350.1	131	2	16.09	Nck1	NP_035008.2	945	2	2.23
Ms4a13	NP_001334364.1	230	1	4.58	Slc35g2	NP_001094953.1	118	2	17.86
Ms4a12	XP_011245716.1	142	5	37.11	Stag1	XP_030100032.1	858	1	1.23
Ms4a1	NP_031667.1	474	10	22.23	Pccb	XP_036011089.1	738	3	4.28
Ms4a5	NP_899013.1	100	10	105.39	Msl2	NP_001093921.1	317	0	0.00
Ms4a14	XP_006527573.1	94	13	145.75	Ppp2r3a	XP_017168836.1	664	3	4.76
Ms4a7	NP_001263327.1	223	10	47.26	Ephb1	NP_001161768.1	1666	6	3.80
Ms4a4a	NP_001297260.1	210	7	35.13	Ky	NP_077253.3	423	2	4.98
Ms4a4c	NP_083775.1	389	5	13.55	Cep63	XP_017168902.1	351	1	3.00
Ms4a4b	NP_068364.1	148	7	49.85	Anapc13	XP_036011127.1	609	1	1.73
Ms4a6c	NP_082871.2	376	6	16.82	Amotl2	XP_006511835.1	706	1	1.49
Gm8369	XP_036017547.1	0	0	#DIV/0!	Ryk	NP_038677.3	925	7	7.98
Ms4a6b	NP_081485.2	427	7	17.28	Slco2a1	NP_201571.2	432	1	2.44
Ms4a4d	NP_079934.2	124	3	25.50	Rab6b	XP_030100263.1	1511	8	5.58

Ms4a6d	NP_081111.1	409	8	20.61	Srprb	NP_033301.1	554	6	11.41
Ms4a2	XP_017173549.1	165	3	19.16	Trf	NP_598738.1	789	2	2.67
Ms4a3	NP_573509.1	255	9	37.20	1300017J02R	NP_082194.1	581	2	3.63
Oosp2	NP_001032723.1	244	3	12.96	Topbp1	XP_006511769.3	1190	7	6.20
Oosp1	NP_579931.1	387	2	5.45	Cdv3	NP_787027.1	257	2	8.20
Oosp3	NP_001028455.2	227	1	4.64	Bfsp2	NP_001002896.1	257	4	16.40
Cblif	NP_032144.2	275	2	7.66	Tmem108	NP_001346394.1	149	5	35.37
Mrpl16	NP_079882.2	1403	1	0.75	Nphp3	NP_082997.3	978	2	2.16
Stx3	NP_035632.1	783	2	2.69	Uba5	NP_079968.2	1005	0	0.00
Olfr1417	NP_667147.1	69	0	0.00	Acad11	NP_780533.2	369	3	8.57
Olfr1418	NP_001011524.1	12	0	0.00	Ackr4	NP_663746.2	255	0	0.00
Olfr1419	NP_001011775.1	11	0	0.00	Dnajc13	NP_001156498.1	732	2	2.88
Olfr1420	NP_666522.1	26	0	0.00	Acpp	NP_997551.1	511	1	2.06
Patl1	NP_766223.2	655	0	0.00	Cpne4	NP_001344368.1	583	1	1.81
Osbp	NP_001028346.1	685	2	3.08	Mrpl3	NP_444389.2	1388	2	1.52
Olfr1423	NP_666891.1	51	0	0.00	Nudt16	XP_030100551.1	191	1	5.52
Olfr1424	NP_666892.1	49	0	0.00	Nudt16l2	NP_082838.2	383	1	2.75
Olfr1425	NP_001011853.1	49	0	0.00	Nek11	NP_766049.2	238	0	0.00
Olfr1426	NP_667020.1	80	0	0.00	Aste1	NP_079927.2	65	1	16.21
Olfr1427	NP_666890.1	45	0	0.00	Atp2c1	NP_001240760.1	988	2	2.13
Olfr1428	NP_666889.2	70	0	0.00	Pik3r4	XP_036011263.1	789	2	2.67
Olfr76	NP_666893.1	38	1	27.73	Col6a6	NP_001096077.1	336	2	6.27
Olfr1431	NP_666526.1	63	0	0.00	Col6a5	XP_030100352.2	316	1	3.34
Olfr262	NP_666899.1	22	0	0.00	Col6a4	XP_036011120.1	223	2	9.45
Olfr235	NP_666897.2	21	0	0.00	Glyctk	XP_006511778.1	405	2	5.20
Olfr1434	NP_666896.2	22	0	0.00	Wdr82	NP_084172.1	794	3	3.98
Olfr1436	NP_666898.2	74	0	0.00	Ppm1m	NP_080723.3	105	1	10.04
Olfr1437	NP_001011839.1	23	0	0.00	Twf2	NP_036006.1	477	5	11.05
Olfr1440	NP_666895.1	21	0	0.00	Tlr9	NP_112455.2	1486	1	0.71
Olfr1441	NP_666894.1	60	1	17.57	Alas1	NP_065584.2	828	4	5.09
Pfpl	NP_062413.2	219	0	0.00	Poc1a	NP_081630.2	377	5	13.98
Mpeg1	NP_034951.2	592	1	1.78	Dusp7	NP_703189.3	734	3	4.31
Dtx4	NP_766030.3	410	0	0.00	Rpl29	NP_001311463.1	742	1	1.42
Fam111a	NP_080916.1	289	1	3.65	Acy1	NP_079647.1	746	2	2.83
Gm4952	NP_001013784.2	588	2	3.58	Abhd14a	XP_006511866.1	252	1	4.18
Glyat	NP_666047.1	427	2	4.94	Abhd14b	XP_017169172.1	369	1	2.86
Olfr1442	NP_666908.2	22	0	0.00	Pcbp4	XP_030100340.1	674	1	1.56
Olfr1443	NP_666909.1	49	2	43.02	Gpr62	NP_001153124.1	229	1	4.60
Keg1	NP_083826.1	623	4	6.77	Parp3	NP_663594.2	591	0	0.00
Cntf	NP_740756.1	605	3	5.23	Rrp9	NP_663595.1	962	0	0.00
Zfp91	NP_443735.2	454	2	4.64	Iqcf1	NP_083119.1	66	1	15.97
Lpxn	NP_598913.1	1027	3	3.08	Iqcf5	NP_083576.1	68	1	15.50
Olfr1444	NP_666913.1	41	0	0.00	Iqcf3	XP_011241205.1	103	1	10.23
Olfr1445	NP_666910.1	23	0	0.00	Iqcf4	NP_080366.2	331	4	12.74
Olfr1446	NP_666915.1	21	0	0.00	Iqcf6	NP_001095098.2	276	1	3.82
Olfr1447	NP_666914.1	49	2	43.02	Grm2	NP_001153825.1	796	1	1.32
Olfr1448	NP_666912.1	31	0	0.00	Tex264	NP_001075123.1	211	0	0.00
Olfr1449	NP_666415.1	16	0	0.00	Rad54l2	NP_109655.2	965	2	2.18
Olfr1450	NP_666483.1	12	0	0.00	Dcaf1	XP_006511816.1	708	2	2.98
Olfr1451	NP_666916.1	26	0	0.00	Rbm15b	NP_780611.3	422	0	0.00
Olfr1453	NP_666911.1	21	0	0.00	Manf	NP_083379.2	486	0	0.00
Olfr1454	NP_666903.1	161	0	0.00	Dock3	XP_030100037.1	1105	3	2.86
Olfr1457	NP_666786.1	74	0	0.00	Mapkapk3	NP_849238.1	395	4	10.67
Olfr1458	XP_030107007.1	67	0	0.00	Cish	NP_034025.1	991	3	3.19
Olfr1459	NP_666900.1	79	0	0.00	Hemk1	XP_036011140.1	305	1	3.46
Olfr1461	NP_666414.1	147	1	7.17	6430571L13R	NP_780695.1	52	0	0.00
Olfr1462	NP_666904.1	34	0	0.00	Cacna2d2	XP_017168985.1	556	8	15.16
Olfr1463	NP_001011840.1	45	0	0.00	Tmem115	NP_062678.1	301	1	3.50
Olfr1465	NP_001011841.1	90	0	0.00	Cyb561d2	XP_030100321.1	47	10	224.23
Olfr1466	XP_030106807.1	69	0	0.00	Nprl2	NP_061367.1	343	9	27.65
Olfr1467	NP_666902.1	175	1	6.02	Zmynd10	XP_006511693.1	253	6	24.99
Olfr1469	NP_666906.1	44	0	0.00	Rassf1	NP_001230677.1	680	8	12.40
Olfr1471	NP_997015.2	140	0	0.00	Tusc2	NP_062716.1	115	7	64.15
Olfr1472	NP_666901.2	89	0	0.00	Hyal2	XP_036010506.1	318	8	26.51
Olfr1474	NP_001011842.1	58	0	0.00	Hyal1	NP_001318090.2	317	9	29.92
Olfr1475	NP_666413.1	196	0	0.00	Naa80	NP_062724.1	66	0	0.00

Olfr1477	NP_666907.2	13	0	0.00	Hyal3	NP_821139.2	163	3	19.40
Olfr1480	NP_997458.1	10	0	0.00	Ifrd2	NP_080179.1	270	4	15.61
Olfr1484	NP_666403.1	10	0	0.00	Sema3b	XP_017168714.1	393	9	24.14
Olfr1487	NP_666847.1	11	0	0.00	Gnai2	NP_032164.2	1479	6	4.28
Olfr1489	NP_666846.1	12	0	0.00	Slc38a3	XP_030100556.1	771	3	4.10
Olfr1490	NP_001011832.1	25	0	0.00	Gnat1	NP_032166.1	1355	1	0.78
Olfr1491	NP_666457.1	50	0	0.00	Sema3f	NP_001366425.1	359	5	14.68
Olfr1494	NP_667201.1	22	0	0.00	Rbm5	NP_001346453.1	869	2	2.43
Olfr1495	NP_666456.1	23	0	0.00	Rbm6	NP_035381.2	455	2	4.63
Olfr1496	NP_667200.2	61	0	0.00	Mon1a	NP_082645.1	437	8	19.29
Olfr1497	NP_666952.1	66	0	0.00	Mst1r	XP_006511717.1	1741	12	7.26
Olfr1499	NP_667007.1	23	0	0.00	Actl11	NP_080614.1	1446	9	6.56
Olfr1500	NP_001011831.1	45	0	0.00	Camkv	NP_663596.1	1326	10	7.95
Olfr1501	NP_666844.1	12	0	0.00	Traip	NP_035764.2	777	9	12.21
Olfr1502	NP_667008.1	63	0	0.00	Uba7	NP_076227.1	1398	10	7.54
Olfr1504	NP_666845.1	40	0	0.00	Inka1	NP_080873.2	29	1	36.34
Olfr1505	NP_001011850.1	85	0	0.00	Cdhr4	NP_001365320.1	171	9	55.47
Tle4	XP_006526976.1	944	4	4.47	Ip6k1	NP_038813.2	820	9	11.57
Psat1	NP_803155.1	1075	6	5.88	Gmppb	NP_001344611.1	737	3	4.29
Cep78	XP_006526886.1	328	2	6.43	Rnf123	XP_017169196.1	955	9	9.93
Gnaq	NP_032165.3	1322	6	4.78	Amigo3	NP_796249.1	88	1	11.98
Gna14	NP_032163.3	1011	4	4.17	Mst1	NP_032269.3	651	2	3.24
Vps13a	NP_766616.2	616	3	5.13	Apeh	NP_666338.2	535	7	13.79
Foxb2	NP_032049.1	366	2	5.76	Bsn	NP_031593.2	1302	3	2.43
Prune2	XP_006527207.1	332	1	3.17	Dag1	XP_030099900.1	625	6	10.12
Gcnt1	XP_017173551.1	412	4	10.23	Nicn1	NP_001351434.1	160	1	6.59
Rfk	NP_062310.1	692	3	4.57	Amt	NP_001013836.1	541	3	5.84
Pcsk5	XP_006526822.1	1059	1	1.00	Tcta	NP_001298085.1	146	2	14.44
Ostf1	NP_059071.1	298	4	14.15	Rhoa	NP_058082.2	3360	2	0.63
Nmrk1	NP_663472.1	861	9	11.02	Gpx1	NP_032186.2	1109	0	0.00
Carnmt1	NP_080396.2	305	3	10.37	Usp4	XP_006511759.1	886	3	3.57
D030056L22R	NP_001361059.1	57	2	36.98	1700102P08R	XP_006511691.1	12	2	175.65
Trpm6	XP_017173641.1	386	5	13.65	Iho1	XP_011241197.1	125	4	33.72
Rorb	NP_001036819.1	613	3	5.16	BC048562	NP_001004192.1	135	0	0.00
Anxa1	NP_034860.2	1470	4	2.87	Klhdc8b	NP_001346357.1	101	2	20.87
Aldh1a1	NP_001348434.1	1495	5	3.52	Ccdc71	NP_001365859.1	71	0	0.00
Aldh1a7	NP_036051.1	1134	2	1.86	Lamb2	NP_032509.2	569	1	1.85
Tmc1	NP_083229.1	344	6	18.38	Usp19	NP_001161843.1	708	4	5.95
Zfand5	XP_006527121.1	438	5	12.03	Qars	NP_598555.2	1213	2	1.74
Gda	NP_034396.1	388	0	0.00	Qrich1	NP_780352.2	135	0	0.00
1110059E24R	NP_079699.1	84	3	37.64	Impdh2	NP_001365850.1	1531	2	1.38
Abhd17b	NP_666208.2	196	3	16.13	Ndufaf3	NP_075736.1	450	3	7.03
Cemip2	NP_001028931.1	278	5	18.96	Dalrd3	XP_017169029.1	665	4	6.34
Trpm3	XP_006527042.1	526	3	6.01	Wdr6	NP_113569.1	433	2	4.87
Klf9	NP_034768.2	699	5	7.54	P4htm	NP_083220.3	346	1	3.05
Smc5	XP_006527044.1	1594	1	0.66	Arih2	NP_001344212.1	595	1	1.77
Mamdc2	NP_777282.1	222	3	14.24	Slc25a20	NP_065266.1	659	3	4.80
1700028P14R	NP_080464.1	58	1	18.17	Prkar2a	NP_032950.1	1142	3	2.77
Ptar1	XP_006527424.1	120	0	0.00	Ip6k2	NP_001366530.1	805	3	3.93
Apba1	XP_030106827.1	880	6	7.19	Nckipsd	NP_109654.2	363	3	8.71
Fam189a2	XP_006527219.1	71	3	44.53	Celsr3	NP_001346501.1	892	3	3.54
Tjp2	XP_030106721.1	770	6	8.21	Slc26a6	XP_036010526.1	435	2	4.85
Fxn	NP_032070.1	787	2	2.68	Tmem89	NP_081342.1	17	3	185.98
Pip5k1b	XP_006526826.1	873	5	6.04	Uqcrc1	NP_079683.2	1287	3	2.46
Fam122a	NP_080796.1	137	2	15.39	Col7a1	NP_031764.2	591	2	3.57
Tmem252	NP_898983.1	79	1	13.34	Ucn2	NP_659543.1	132	0	0.00
Pgm5	NP_778178.3	798	2	2.64	Pfkfb4	XP_036010930.1	588	1	1.79
Foxd4	NP_032048.1	540	5	9.76	Shisa5	NP_001271261.1	438	0	0.00
Cbwd1	NP_666209.1	351	0	0.00	Trex1	NP_035767.4	461	0	0.00
Dock8	NP_083061.2	652	5	8.08	Atrip	NP_766362.2	366	1	2.88
Ndufb4b	XP_001478493.1	468	0	0.00	Tma7	NP_899073.1	332	1	3.17
Kank1	XP_030106551.1	1207	7	6.11	Ccdc51	NP_079965.2	38	1	27.73
Dmrt1	NP_056641.2	576	7	12.81	Plxnb1	XP_030100205.1	557	0	0.00
Dmrt3	NP_796334.2	388	5	13.58	Fbxw21	NP_796043.1	356	0	0.00
Dmrt2	NP_665830.1	425	6	14.88	Fbxw13	NP_808266.1	423	0	0.00
Smarca2	XP_006527344.1	2073	4	2.03	Fbxw20	NP_001008428.1	382	0	0.00

Gm815	NP_001028579.1	128	0	0.00	Fbxw14	XP_006512245.1	381	0	0.00
Vldlr	XP_030106725.1	849	3	3.72	Fbxw28	NP_001170890.1	381	0	0.00
Kcnv2	NP_899002.1	858	2	2.46	Fbxw22	XP_006512244.1	457	0	0.00
Pum3	NP_803425.1	1243	1	0.85	Fbxw16	NP_796044.2	431	0	0.00
Rfx3	XP_017173576.1	786	6	8.05	Fbxw19	XP_011241263.1	319	0	0.00
Glis3	NP_780668.3	691	5	7.63	Fbxw15	XP_006512232.1	443	0	0.00
Slc1a1	NP_033225.1	762	5	6.92	Fbxw24	NP_001013798.2	458	0	0.00
4430402I18Ril	XP_036017513.1	40	0	0.00	Fbxw25	XP_030100627.1	478	0	0.00
Plpp6	NP_083198.1	172	3	18.38	Fbxw18	NP_001028966.1	432	0	0.00
Cdc37l1	NP_001289309.1	632	1	1.67	Fbxw26	NP_941076.1	457	0	0.00
Ak3	XP_006527285.1	849	1	1.24	Spink8	NP_898959.2	173	0	0.00
Rcl1	NP_067500.1	879	3	3.60	Nme6	XP_036011011.1	771	0	0.00
Jak2	NP_001041642.1	2387	3	1.32	Camp	NP_034051.2	481	2	4.38
Insl6	NP_038782.1	146	2	14.44	Cdc25a	XP_006511992.1	1463	2	1.44
Rln1	NP_035402.2	286	2	7.37	Map4	XP_036010539.1	707	1	1.49
Plgrkt	XP_030106918.1	109	6	58.01	Dhx30	XP_017169103.1	1209	1	0.87
Cd274	NP_068693.1	994	5	5.30	Smarcc1	NP_033237.2	1368	3	2.31
Pdcd1lg2	NP_067371.1	535	4	7.88	Cspg5	NP_001159745.1	693	2	3.04
Ric1	NP_001074788.1	466	5	11.31	Elp6	NP_001074850.1	196	0	0.00
Ermp1	NP_001074682.1	136	3	23.25	Scap	NP_001001144.2	439	1	2.40
Mlana	NP_084269.1	426	2	4.95	Ptpn23	NP_001074512.1	1426	3	2.22
9930021J03Ri	XP_006527135.1	32	3	98.80	Ngp	NP_032720.2	123	1	8.57
Ranbp6	NP_808389.2	987	6	6.41	Klhl18	NP_808439.2	271	5	19.44
Il33	NP_001347654.1	689	2	3.06	Kif9	XP_006512005.1	871	6	7.26
Trpd52l3	NP_080017.1	143	1	7.37	Setd2	XP_036010808.1	1838	4	2.29
Uhrf2	NP_659122.2	548	3	5.77	Nradd	NP_080288.1	117	2	18.02
Gldc	NP_613061.1	715	1	1.47	Nbeal2	NP_899099.2	384	3	8.23
Mbl2	NP_001351987.1	871	1	1.21	Ccdc12	XP_030100495.1	367	4	11.49
Dkk1	NP_034181.2	983	2	2.14	Pth1r	XP_006512051.1	649	2	3.25
Prkg1	XP_006526831.1	1395	5	3.78	Myl3	NP_001351413.1	1419	0	0.00
Cstf2t	NP_112539.2	1305	1	0.81	Prss42	NP_694739.1	107	2	19.70
A1cf	NP_001074543.1	1027	1	1.03	Prss44	NP_683742.2	116	1	9.09
Asah2	NP_061300.1	309	1	3.41	Prss43	NP_955765.1	87	2	24.23
Sgms1	NP_001349352.1	367	2	5.74	Prss45	NP_694812.1	111	1	9.49
Minpp1	NP_034929.1	249	4	16.93	Prss46	NP_898926.2	111	1	9.49
Papss2	XP_006527127.1	665	4	6.34	Prss50	NP_666339.2	252	1	4.18
Atad1	NP_080763.2	1062	5	4.96	Tmie	XP_036010653.1	188	2	11.21
Pten	NP_032986.1	3768	5	1.40	Als2cl	XP_017168843.1	191	3	16.55
Lipo4	NP_001310315.1	139	0	0.00	Fam240a	XP_036010812.1	0	0	#DIV/0!
Lipo3	NP_001013792.2	147	0	0.00	Tdgf1	NP_035692.2	700	1	1.51
Gm8978	NP_001310180.1	187	1	5.64	Lrrc2	XP_006512427.1	692	4	6.09
Lipo2	NP_001309267.1	130	0	0.00	Rtp3	NP_694740.2	812	1	1.30
Lipo1	NP_001309994.1	147	1	7.17	Ltf	NP_032548.2	869	0	0.00
Lipf	XP_017173769.1	502	4	8.40	Ccrl2	NP_059494.2	683	0	0.00
Lipk	NP_001192278.1	258	5	20.42	Lrrfip2	XP_011241302.1	511	5	10.31
Lipn	NP_081616.1	304	3	10.40	Mlh1	NP_081086.2	1089	1	0.97
Lipm	NP_076392.1	407	4	10.36	Epm2aip1	NP_780475.1	118	4	35.73
Ankrd22	NP_077166.4	1240	7	5.95	Trank1	XP_006512226.1	522	4	8.08
Stambpl1	NP_001347645.1	1073	7	6.88	Dclk3	NP_766516.2	741	3	4.27
Acta2	NP_031418.1	2655	3	1.19	Stac	XP_006512079.1	166	0	0.00
Fas	NP_032013.2	1184	5	4.45	Arpp21	XP_017169153.1	997	3	3.17
Ch25h	NP_034020.1	787	16	21.43	Pdcd6ip	NP_035182.2	847	1	1.24
Lipa	NP_067435.3	473	4	8.91	Clasp2	XP_006512447.1	566	3	5.59
Ifit2	NP_001342191.1	1259	4	3.35	Ubp1	XP_006512113.3	323	3	9.79
Ifit3	XP_011245455.1	1245	5	4.23	Fbxl2	NP_848739.1	335	3	9.44
Ifit1bl1	NP_001095075.1	877	6	7.21	Susd5	NP_001094980.1	105	4	40.15
Ifit3b	XP_006527327.1	865	7	8.53	Gm29825	NP_001365617.1	0	0	#DIV/0!
Ifit1bl2	NP_001349059.1	897	7	8.22	4921528I07Ri	XP_034952818.2	0	0	#DIV/0!
Ifit1	NP_032357.2	1327	6	4.77	Bcl2a1c	NP_031561.2	510	0	0.00
Slc16a12	NP_766426.1	595	10	17.71	Crtap	NP_064306.2	341	7	21.63
Pank1	XP_006527490.1	386	6	16.38	Glb1	NP_033882.1	488	3	6.48
Kif20b	NP_898867.1	1561	8	5.40	Tmppe	NP_001186931.1	29	6	218.05
Htr7	NP_032341.2	599	1	1.76	Ccr4	XP_006511995.1	485	1	2.17
Rpp30	NP_062301.3	557	1	1.89	Trim71	NP_001035968.1	833	3	3.80
Ankrd1	NP_038496.2	1626	4	2.59	Cnot10	XP_036011304.1	237	3	13.34
Pcgf5	NP_001355619.1	423	1	2.49	Dync1li1	NP_666341.1	803	5	6.56

Hectd2	NP_001156943.1	932	2	2.26	Cmtm6	NP_080312.1	280	3	11.29
Ppp1r3c	NP_058550.1	592	2	3.56	Cmtm7	NP_001239408.1	215	6	29.41
Tnks2	NP_001157107.1	1607	4	2.62	Cmtm8	NP_081570.1	209	5	25.21
Fgfbp3	NP_082539.2	204	1	5.17	Gpd1l	NP_780589.3	968	2	2.18
Btaf1	NP_001074175.1	879	1	1.20	Osbpl10	NP_683761.2	369	1	2.86
Cpeb3	XP_030106701.1	381	0	0.00	Rps27rt	NP_001177187.1	723	0	0.00
Marchf5	NP_001365726.1	333	0	0.00	Stt3b	NP_077184.2	572	1	1.84
Ide	XP_036017339.1	707	4	5.96	Gadl1	XP_036011234.1	513	1	2.05
Kif11	NP_034745.1	1926	5	2.74	Tgfbr2	NP_083851.3	1910	6	3.31
Hhex	NP_032271.1	833	3	3.80	Rbms3	NP_001165597.1	499	5	10.56
Exoc6	XP_017173533.1	758	4	5.56	Azi2	XP_006512210.1	256	4	16.47
Cyp26c1	XP_017173748.1	418	7	17.65	Cmc1	NP_080718.1	123	3	25.70
Cyp26a1	NP_031837.2	763	5	6.91	Eomes	NP_034266.2	1087	4	3.88
Myof	NP_001093104.1	671	6	9.42	Golga4	XP_036010998.1	522	3	6.06
Cep55	NP_001347592.1	726	2	2.90	Itga9	NP_598482.2	609	4	6.92
Ffar4	NP_861413.1	536	1	1.97	Ctdspl	NP_598471.3	652	6	9.70
Rbp4	NP_001152959.1	718	4	5.87	Vill	XP_006512122.1	423	6	14.95
Pde6c	NP_291092.1	514	6	12.30	Plcd1	NP_062650.1	733	8	11.50
Fra10ac1	XP_017173780.1	118	2	17.86	Dlec1	NP_796091.2	221	7	33.38
Lgi1	NP_064674.1	772	5	6.83	Acaa1b	NP_666342.1	1163	7	6.34
Slc35g1	NP_780716.1	223	2	9.45	Slc22a14	XP_011241282.1	213	3	14.84
Plce1	XP_006527457.1	811	6	7.80	Slc22a13	NP_598741.2	369	3	8.57
Noc3l	NP_067290.2	896	4	4.70	Oxsr1	NP_001346511.1	353	7	20.90
Tbc1d12	NP_666064.3	468	5	11.26	Myd88	NP_034981.1	1496	1	0.70
Hells	NP_032260.2	1931	3	1.64	Acaa1a	XP_030099878.1	806	4	5.23
Cyp2c55	NP_082365.1	476	10	22.14	Xylb	NP_001028381.1	667	6	9.48
Cyp2c65	NP_082467.2	287	9	33.05	Acvr2b	NP_031423.1	1276	4	3.30
Cyp2c66	NP_001011707.1	215	3	14.71	Exog	NP_766044.1	276	4	15.27
Cyp2c29	NP_031841.3	766	13	17.89	Scn5a	XP_006512059.1	926	5	5.69
Cyp2c39	NP_001360866.1	665	3	4.75	Scn10a	NP_033160.2	723	3	4.37
Cyp2c38	XP_017173543.1	689	3	4.59	Scn11a	XP_017168851.1	465	2	4.53
Cyp2c67	XP_011245593.1	675	2	3.12	Wdr48	NP_080512.1	863	1	1.22
Cyp2c68	NP_001034644.2	747	4	5.64	Gorasp1	NP_083252.1	613	1	1.72
Cyp2c40	NP_034134.3	615	3	5.14	Ttc21a	NP_083011.2	175	2	12.04
Cyp2c69	NP_001097995.1	342	1	3.08	Csrnp1	NP_001344490.1	287	1	3.67
Cyp2c37	NP_034131.2	822	3	3.85	Xirp1	XP_030100108.2	505	3	6.26
Cyp2c54	XP_006527235.1	772	2	2.73	Cx3cr1	NP_034117.3	921	2	2.29
Cyp2c50	NP_598905.2	772	1	1.37	Ccr8	NP_031746.1	556	1	1.90
Cyp2c70	NP_663474.2	771	1	1.37	Slc25a38	NP_659042.1	317	2	6.65
Pdlim1	NP_058557.2	902	2	2.34	Rpsa	NP_035159.3	1656	4	2.55
Sorbs1	XP_030106687.1	996	5	5.29	Mobp	NP_032640.1	579	3	5.46
Aldh18a1	NP_705782.2	1990	3	1.59	Myrip	NP_653140.1	514	1	2.05
Tctn3	NP_080536.2	208	1	5.07	Eif1b	NP_081168.1	684	2	3.08
Entpd1	NP_001291650.1	743	3	4.26	Entpd3	NP_848791.3	367	1	2.87
Ccnj	XP_017173689.1	170	0	0.00	Rpl14	NP_080250.1	1198	3	2.64
Zfp518a	NP_082595.1	222	0	0.00	Ctnnb1	NP_001159374.1	3480	3	0.91
Blnk	NP_001351983.1	723	2	2.92	Ulk4	XP_030100042.1	430	5	12.25
Dntt	NP_033371.2	1242	3	2.55	Trak1	NP_780323.2	287	1	3.67
Opalin	NP_705740.1	526	1	2.00	Cck	XP_017168605.1	1315	5	4.01
Tll2	NP_036034.1	316	3	10.01	Lyzl4	NP_001369764.1	113	0	0.00
Tm9sf3	NP_579930.1	518	2	4.07	Vipr1	NP_035833.2	498	1	2.12
Pik3ap1	NP_113553.1	634	1	1.66	Sec22c	NP_848792.2	600	4	7.03
Lcor	NP_001357696.1	288	1	3.66	Ss18l2	NP_081070.1	357	3	8.86
Slit1	XP_036017388.1	1324	0	0.00	Nktr	XP_030099969.1	1379	3	2.29
Arhgap19	NP_001156967.1	545	0	0.00	Zfp651	NP_001160116.1	268	4	15.73
Frat2	NP_808271.1	358	0	0.00	Klhl40	NP_082478.1	330	2	6.39
Rrp12	NP_955518.1	937	2	2.25	Hhatl	XP_030100549.1	462	1	2.28
Pgam1	NP_075907.2	957	0	0.00	Ccdc13	XP_036010389.1	159	2	13.26
Exosc1	NP_001307160.1	687	1	1.53	Higd1a	NP_001344768.1	271	1	3.89
Zdhhc16	NP_001347605.1	214	2	9.85	Ackr2	NP_067622.2	557	1	1.89
Mms19	NP_001360875.1	615	3	5.14	Cyp8b1	NP_034142.3	988	1	1.07
Ubtd1	NP_663475.1	104	2	20.27	Gask1a	XP_006512195.1	64	1	16.47
Ankrd2	NP_064417.2	1261	0	0.00	Pomgnt2	XP_036010725.1	232	1	4.54
Hoga1	NP_080428.1	967	2	2.18	Snrk	NP_001344704.1	242	1	4.35
4933411K16R	NP_080028.1	270	1	3.90	Ano10	NP_001258802.1	284	3	11.13
Morn4	XP_030106757.1	187	3	16.91	Abhd5	NP_080455.1	578	2	3.65

Pi4k2a	NP_663476.1	662	3	4.78	Topaz1	NP_001186665.1	119	1	8.86
Avpi1	NP_081382.1	146	3	21.66	Tcaim	XP_006512234.1	55	1	19.16
Marveld1	NP_899018.1	214	4	19.70	Zfp445	NP_001346864.1	377	0	0.00
Zfyve27	NP_796293.2	272	1	3.87	Zkscan7	NP_001170976.1	298	1	3.54
Sfrp5	NP_061250.2	560	0	0.00	Zfp105	NP_033570.2	313	1	3.37
Golga7b	XP_030106936.1	497	2	4.24	1110059G10F	NP_079695.2	34	0	0.00
Crtac1	XP_006527435.1	563	3	5.62	Kif15	NP_034750.1	1194	0	0.00
R3hcc1l	XP_006527245.1	242	0	0.00	Tmem42	NP_001158295.1	79	0	0.00
Loxl4	XP_036017563.1	306	3	10.33	Tgm4	XP_017168931.1	229	1	4.60
Pyroxd2	NP_083287.2	499	0	0.00	Zdhhc3	NP_001359467.1	287	6	22.03
Hps1	XP_030106637.1	125	1	8.43	Exosc7	NP_001074657.1	892	5	5.91
Hpse2	XP_006527277.1	249	4	16.93	Clec3b	NP_035736.2	494	5	10.67
Cnnm1	XP_006527543.1	885	3	3.57	Cdcp1	XP_006511984.1	207	5	25.46
Got1	NP_034454.2	778	0	0.00	Tmem158	NP_001002267.2	155	5	34.00
Nkx2-3	NP_032725.1	633	2	3.33	Lars2	NP_001335096.1	1021	7	7.23
Slc25a28	NP_660138.1	331	6	19.10	Limd1	NP_038888.2	891	5	5.91
Entpd7	NP_444333.3	133	6	47.54	Sacm1l	NP_001344414.1	1151	3	2.75
Cox15	NP_659123.2	543	3	5.82	Slc6a20b	NP_035861.2	241	2	8.75
Cutc	NP_001107034.1	144	4	29.28	Slc6a20a	NP_631881.1	377	3	8.39
Abcc2	NP_038834.2	1197	3	2.64	Lztfl1	NP_201579.1	264	5	19.96
Dnmbp	XP_006527414.1	1053	0	0.00	Ccr9	NP_001160097.1	728	8	11.58
Cpn1	NP_109628.1	642	5	8.21	Fyco1	XP_017168648.1	424	4	9.94
Cyp2c23	NP_001001446.3	750	5	7.03	Cxcr6	NP_109637.3	638	8	13.91
Erlin1	NP_663477.3	469	5	11.24	Xcr1	NP_035928.2	603	8	15.54
Chuk	NP_031726.2	2008	5	2.62	Ccr1	NP_034042.3	1004	7	8.64
Cwf19l1	NP_001074546.1	282	4	14.95	Ccr1l1	NP_031744.3	493	7	18.71
Bloc1s2	NP_082883.1	425	5	12.40	Ccr3	XP_017168608.1	798	7	12.33
Pkd2l1	XP_036017505.1	458	10	23.01	Ccr2	XP_036010478.1	1206	7	8.74
Scd3	NP_077770.1	541	3	5.84	Ccr5	XP_036010482.1	1406	7	8.07
Scd2	NP_033154.2	735	3	4.30	2010315B03R	NP_001230048.1	126	0	0.00
Scd4	NP_899039.2	580	3	5.45	Nlrp4g	XP_036010820.1	317	0	0.00
Scd1	NP_033153.2	1151	4	3.66	Ppp2r3d	NP_001156887.1	627	0	0.00
Wnt8b	NP_035850.2	615	9	15.42	Btbd35f23	NP_001257596.1	120	0	0.00
Sec31b	NP_001028515.1	657	8	12.83	Btbd35f11	NP_001079020.1	37	0	0.00
Ndufb8	NP_001334376.1	682	6	9.27	Btbd35f18	NP_001079014.1	16	0	0.00
Hif1an	NP_795932.2	619	5	8.51	Btbd35f10	NP_001079021.1	29	0	0.00
Pax2	NP_001355673.1	1067	6	5.93	Spin2-ps1	NP_083382.2	98	0	0.00
Slf2	NP_001074694.1	144	2	14.64	Btbd35f16	NP_001229940.1	27	0	0.00
Sema4g	XP_036017494.1	312	2	6.76	Btbd35f3	NP_001229930.1	13	0	0.00
Mrpl43	NP_444394.1	733	3	4.31	Btbd35f28	NP_001079012.1	26	0	0.00
Twnk	NP_722491.2	476	4	8.86	Btbd35f20	NP_001079022.1	20	0	0.00
Lzts2	XP_030106758.1	241	0	0.00	Btbd35f17	NP_001079023.1	51	0	0.00
Pdzd7	XP_006526601.1	585	1	1.80	Btbd35f4	NP_001093113.1	13	0	0.00
Sfxn3	NP_001349310.1	289	2	7.29	Btbd35f27	NP_001229953.1	131	1	8.05
Kazald1	XP_017173531.1	58	0	0.00	Mycs	NP_034980.2	342	0	0.00
Tlx1	XP_006526978.1	537	9	17.66	Btbd35f7	NP_001078992.1	23	0	0.00
Lbx1	NP_034821.2	500	7	14.75	Nudt11	NP_067406.2	747	0	0.00
Btrc	NP_001347049.1	1274	7	5.79	Ezhip	NP_001028383.2	162	1	6.51
Poll	NP_001317435.1	390	10	27.02	Nudt10	NP_001026834.1	747	0	0.00
Dpcd	NP_766227.1	241	9	39.36	Bmp15	NP_033887.1	874	2	2.41
Fbxw4	XP_006527194.1	507	9	18.71	Shroom4	NP_001035549.1	188	0	0.00
Fgf8	NP_001159835.1	1214	10	8.68	Dgkk	NP_808582.3	239	0	0.00
Npm3	NP_032749.1	374	3	8.45	Ccnb3	XP_017173937.1	1801	3	1.76
Oga	XP_036017614.1	593	8	14.22	Akap4	NP_033781.2	661	2	3.19
Kcnip2	NP_663750.1	1272	7	5.80	Clcn5	XP_036017696.1	480	1	2.20
Armh3	XP_036017585.1	103	2	20.46	Usp27x	NP_062334.2	550	2	3.83
Hps6	NP_789742.2	204	5	25.83	Ppp1r3f	NP_613071.3	228	1	4.62
Ldb1	XP_006526786.3	607	10	17.36	Foxp3	NP_473380.1	1645	1	0.64
Pprc1	NP_001333730.1	613	4	6.88	Ccdc22	NP_613069.3	458	2	4.60
Nolc1	NP_001034441.1	1164	5	4.53	Cacna1f	XP_030107268.1	792	5	6.65
Elovl3	NP_031729.1	724	4	5.82	Syp	NP_033331.2	1821	3	1.74
Pitx3	XP_006526827.1	504	3	6.27	Prickle3	NP_001277553.1	651	3	4.86
Gbf1	XP_006526643.1	832	4	5.07	Plp2	NP_062729.1	259	1	4.07
Nfkb2	NP_001170841.1	1963	3	1.61	Magix	NP_061302.2	702	5	7.51
Psd	XP_030106967.1	914	2	2.31	Gpkow	NP_776108.2	409	1	2.58
Fbxl15	NP_001365702.1	797	7	9.26	Wdr45	XP_030107255.1	381	4	11.06

Cuedc2	XP_030106898.1	142	4	29.69	Praf2	NP_613068.1	230	3	13.75
Mfsd13a	NP_083462.2	121	2	17.42	Ccdc120	NP_997085.2	262	1	4.02
Actr1a	NP_058556.1	1662	6	3.80	Tfe3	NP_766060.2	639	2	3.30
Sufu	NP_056567.2	1169	1	0.90	Gripap1	XP_017174054.1	244	0	0.00
Trim8	NP_444330.2	637	4	6.62	Kcnd1	XP_036017720.1	779	0	0.00
Arl3	NP_062692.1	1530	7	4.82	Otud5	XP_017174053.1	423	1	2.49
Sfxn2	NP_444426.3	314	3	10.07	Pim2	NP_613072.1	1321	4	3.19
Wbp1l	NP_001171283.1	120	5	43.91	Slc35a2	XP_006527648.1	574	1	1.84
Cyp17a1	NP_031835.3	1019	6	6.21	Pqbp1	NP_001239458.1	606	3	5.22
Borcs7	NP_079839.1	65	7	113.50	Timm17b	NP_035721.1	784	2	2.69
As3mt	NP_065602.2	339	9	27.98	Pcsk1n	NP_038920.2	612	0	0.00
Cnnm2	NP_291047.2	615	10	17.14	Eras	NP_853526.1	1531	4	2.75
Nt5c2	NP_001157835.1	594	9	15.97	Hdac6	XP_017173878.1	2250	8	3.75
Ina	NP_666212.3	799	1	1.32	Gata1	NP_032115.1	1735	9	5.47
Pcgf6	NP_081930.1	574	2	3.67	Glod5	NP_081503.1	20	2	105.39
Taf5	NP_796316.2	1181	6	5.35	Suv39h1	NP_035644.1	1473	3	2.15
Atp5md	NP_075700.2	594	9	15.97	Was	NP_033541.1	1292	3	2.45
Pdcd11	NP_035183.2	1361	3	2.32	Wdr13	XP_036018003.1	598	4	7.05
Calhm2	NP_598507.3	82	5	64.26	Rbm3	NP_001159882.1	1607	4	2.62
Calhm1	NP_001074740.1	277	7	26.63	Tbc1d25	NP_001159909.1	620	8	13.60
Neurl1a	NP_067335.4	878	1	1.20	Ebp	NP_031924.1	238	1	4.43
Sh3pxd2a	XP_036017325.1	1848	3	1.71	Porcn	XP_036017908.1	328	0	0.00
Stn1	XP_030106555.1	867	1	1.22	Ftsj1	NP_001277359.1	941	3	3.36
Slk	NP_033315.2	370	1	2.85	Slc38a5	NP_766067.2	462	1	2.28
Col17a1	NP_031758.2	515	0	0.00	Ssxb10	NP_951019.1	11	0	0.00
Sfr1	NP_080653.2	166	0	0.00	Ssxb9	NP_951021.2	0	0	#DIV/0!
Cfap43	XP_011245405.1	94	0	0.00	Ssxb1	NP_080768.1	45	1	23.42
Gsto1	NP_034492.1	672	2	3.14	Ssxb2	NP_001001450.1	32	1	32.93
Gsto2	XP_030106922.1	404	1	2.61	Gm14459	XP_006527711.1	32	1	32.93
Itprip	NP_001001738.2	94	0	0.00	Ssxb6	NP_001192037.1	5	0	0.00
Cfap58	NP_001156739.1	47	0	0.00	Ssxb3	NP_942601.1	0	0	#DIV/0!
Sorcs3	NP_079972.1	670	2	3.15	Ssxb7	XP_017174170.1	0	0	#DIV/0!
Sorcs1	NP_001239430.1	690	2	3.05	Ssxb8	NP_001075034.3	0	0	#DIV/0!
Ins1	NP_032412.3	1982	3	1.60	Ssx9	NP_951018.1	47	2	44.85
Xpnpep1	XP_011245460.1	715	3	4.42	Ssxb5	NP_955023.1	0	0	#DIV/0!
Add3	XP_030106819.1	580	3	5.45	Gm6592	NP_001075033.1	0	0	#DIV/0!
Mxi1	NP_001008542.2	607	5	8.68	Fthl17b	NP_001078993.1	60	0	0.00
Smndc1	XP_006527503.1	611	1	1.72	Fthl17c	NP_001078994.1	60	0	0.00
Dusp5	NP_001078859.1	1072	3	2.95	Fthl17d	NP_001092796.1	184	1	5.73
Smc3	NP_031816.2	2099	1	0.50	Fthl17e	NP_112551.2	186	1	5.67
Rbm20	XP_011245673.1	345	3	9.16	Fthl17-ps1	NP_001264113.1	60	0	0.00
Pdcd4	NP_035180.2	767	0	0.00	Fthl17f	NP_001033786.1	60	0	0.00
Bbip1	XP_036017257.1	31	0	0.00	4930402K13R	NP_001257629.1	217	3	14.57
Shoc2	NP_001342171.1	794	5	6.64	Lancl3	NP_775590.2	136	3	23.25
Adra2a	NP_031443.4	720	1	1.46	Xk	NP_075989.1	323	4	13.05
Gpam	XP_036017329.1	578	8	14.59	H2al1m	NP_083864.1	1204	1	0.88
Tectb	XP_006526966.1	279	9	34.00	H2al1k	NP_001079006.1	1202	0	0.00
Gucy2g	NP_001074545.1	628	10	16.78	Cybb	NP_031833.3	1371	4	3.07
Acsl5	XP_006527241.1	859	12	14.72	H2al1o	NP_001078986.1	1376	1	0.77
Zdhhc6	XP_006527331.1	178	12	71.05	Dynlt3	NP_080251.3	475	3	6.66
Vti1a	NP_001280614.1	517	13	26.50	H2ap	NP_080372.1	59	1	17.86
Tcf7l2	XP_006526923.1	1416	4	2.98	H2al3	NP_083505.1	159	1	6.63
Habp2	NP_001316864.1	1313	14	11.24	Sytl5	XP_017173956.1	299	1	3.52
Nrap	NP_932307.2	436	2	4.83	Srpx	NP_001352016.1	245	2	8.60
Casp7	XP_006526679.3	1166	13	11.75	Rpgr	NP_035415.1	857	4	4.92
Plekhs1	NP_766229.2	108	12	117.10	Otc	NP_032795.1	1221	3	2.59
Dclre1a	NP_001347367.1	674	11	17.20	Tspan7	NP_062608.2	727	0	0.00
Nhlrc2	NP_080087.1	398	13	34.42	Mid1ip1	NP_080800.1	177	1	5.95
Adrb1	NP_031445.2	750	11	15.46	H2al1a	NP_001104507.1	846	0	0.00
Ccdc186	NP_739563.1	330	11	35.13	H2al1b	NP_001229876.1	1378	1	0.76
Tdrd1	XP_006527536.1	940	4	4.48	H2al1c	NP_001229878.1	746	0	0.00
Vwa2	NP_766428.2	165	5	31.94	H2al1d	NP_001229879.1	746	0	0.00
Afap1l2	XP_036017467.1	198	0	0.00	H2al1e	NP_001020431.1	1203	1	0.88
Ablim1	XP_030106777.1	510	7	14.47	H2al1f	NP_001229880.1	746	0	0.00
Fam160b1	NP_663480.3	145	6	43.61	H2al1g	NP_001229881.1	848	0	0.00
Trub1	NP_082391.1	1107	6	5.71	H2al1h	NP_001229882.1	754	0	0.00

Atrnl1	NP_852080.3	432	3	7.32	H2al1i	NP_001229883.1	746	0	0.00
Gfra1	XP_030106613.1	504	4	8.36	H2al1j	NP_001108001.1	1404	1	0.75
Ccdc172	XP_006527485.1	69	0	0.00	Bcor	XP_030107363.1	1149	4	3.67
Pnlip	NP_081201.2	430	4	9.80	Atp6ap2	NP_081715.1	640	1	1.65
Pnliprp1	NP_061362.1	322	4	13.09	1810030O07R	NP_780350.1	98	2	21.51
Pnliprp2	NP_035258.2	316	4	13.34	Med14	XP_006527692.1	646	1	1.63
1700019N19R	NP_080484.1	167	0	0.00	Usp9x	XP_030107164.1	1521	4	2.77
Hspa12a	NP_001314927.1	2149	9	4.41	Llph-ps2	XP_036018053.1	280	0	0.00
Eno4	NP_848804.2	981	1	1.07	Ddx3x	NP_034158.1	2262	5	2.33
Shtn1	NP_001107784.1	201	5	26.22	Nyx	XP_030107174.1	701	4	6.01
Vax1	NP_033527.1	466	2	4.52	Cask	NP_001271433.1	1540	6	4.11
Kcnk18	NP_997144.1	330	0	0.00	Gpr34	NP_035953.3	246	3	12.85
Slc18a2	NP_766111.1	617	0	0.00	Gpr82	XP_017174011.1	179	3	17.66
Pdzd8	NP_001028394.2	142	2	14.84	H2al1n	NP_001029272.1	1378	5	3.82
Emx2	NP_034262.2	731	4	5.77	Cypt1	NP_080014.2	219	2	9.62
Rab11fip2	XP_011245688.1	431	1	2.45	Maoa	NP_776101.3	1089	7	6.77
Fam204a	NP_001345202.1	38	0	0.00	Maob	NP_766366.2	1247	6	5.07
Prlhr	NP_963909.2	348	2	6.38	Ndp	NP_035013.1	363	4	11.61
Cacul1	NP_001165568.1	1228	2	1.91	Efhc2	NP_083192.2	216	6	29.28
Nanos1	NP_848508.2	556	0	0.00	Fundc1	XP_011245798.1	347	5	15.19
Eif3a	NP_034253.3	1338	1	0.98	Dusp21	NP_082844.1	699	2	3.02
Dennd10	NP_001347549.1	75	0	0.00	Kdm6a	XP_006527657.1	3864	7	1.91
Sfxn4	XP_036017628.1	206	0	0.00	Dipk2b	NP_780437.1	100	0	0.00
Prdx3	NP_031478.1	1446	1	1.12	Tex13c3	NP_997407.1	2	0	0.00
Grk5	NP_061357.3	685	2	5.13	Chst7	NP_068361.1	213	6	29.69
Gm7102	NP_001170984.1	85	0	0.00	Slc9a7	XP_030107175.1	608	7	12.13
Csf2ra	XP_017173541.1	554	0	0.00	Rp2	NP_598430.1	181	1	5.82
Fam171a1	NP_001074630.1	112	1	18.82	Jade3	XP_011245778.1	468	9	20.27
Nmt2	NP_032734.1	578	3	9.95	Rgn	XP_006527635.1	1095	6	5.77
Rpp38	XP_030105367.1	1336	1	1.31	Ndufb11	NP_001345553.1	610	7	12.09
Acbd7	XP_030108104.1	325	3	14.97	Rbm10	NP_663602.1	666	10	15.82
Olah	XP_036018648.1	212	0	0.00	Uba1	NP_033483.2	1619	7	4.56
Meig1	NP_001342138.1	207	1	6.79	Cdk16	XP_006527633.1	2163	11	5.36
Dclre1c	XP_006497525.2	726	2	3.63	Usp11	NP_663603.3	817	3	3.87
Suv39h2	NP_073561.2	1308	4	3.79	Araf	XP_011245731.2	1436	11	8.07
Hspa14	NP_056580.2	1789	3	1.96	Syn1	NP_038708.3	1471	4	2.87
Cdnf	NP_808315.1	222	0	0.00	Timp1	NP_001037849.1	1409	6	4.49
Fam107b	XP_006497581.1	123	0	0.00	Cfp	NP_032849.2	1023	4	4.12
Frmd4a	XP_006497502.1	515	1	2.05	Elk1	NP_031948.4	287	1	3.67
Prpf18	NP_080321.2	631	1	1.67	Uxt	XP_006527665.1	825	4	5.11
Bend7	NP_848778.1	50	3	63.23	Zfp182	XP_030107225.1	204	3	15.50
Sephs1	NP_001349637.1	407	5	12.95	Spaca5	NP_001078862.1	141	3	22.42
Phyh	NP_034856.1	587	0	0.00	Zfp300	XP_006527691.1	182	3	17.37
Ucma	NP_001107030.1	110	0	0.00	Ssxa1	NP_001192015.1	125	0	0.00
Mcm10	XP_036018451.1	895	5	5.89	Gm5932	XP_036018055.1	143	0	0.00
Optn	NP_001343416.1	701	4	6.01	Agtr2	NP_031455.1	698	1	1.51
Ccdc3	NP_083080.1	188	6	33.64	Slc6a14	NP_064433.3	475	0	0.00
Camk1d	XP_006497534.1	1386	7	5.32	Klhl13	NP_080443.1	626	1	1.68
Cdc123	XP_030108123.1	550	2	3.83	Wdr44	NP_780389.2	200	0	0.00
Nudt5	XP_036018286.1	676	2	3.12	Tesl1	NP_001030036.2	571	0	0.00
Sec61a2	NP_001343340.1	1327	3	2.38	Gm5934	NP_001093914.1	0	0	#DIV/0!
Dhtkd1	NP_001074600.1	572	2	3.68	Gm4297	NP_001093916.1	0	0	#DIV/0!
Upf2	NP_001074601.1	843	2	2.50	Gm5935	NP_001075126.1	0	0	#DIV/0!
Proser2	NP_659132.2	12	2	175.65	Gm5169	NP_001035759.1	1	0	0.00
Echdc3	NP_077170.2	445	4	9.47	Gm1993	NP_001096147.1	13	0	0.00
Usp6nl	NP_001074017.1	671	1	1.57	Gm2003	XP_011247950.1	61	0	0.00
Celf2	XP_036014142.1	700	4	6.02	Gm5168	NP_001020778.1	0	0	#DIV/0!
Gata3	NP_001342039.1	2001	4	2.11	Gm2012	NP_001098416.1	0	0	#DIV/0!
Taf3	NP_082024.2	1193	3	2.65	Gm2030	NP_001093915.1	0	0	#DIV/0!
Atp5c1	NP_001361570.1	1207	4	3.49	Gm2036	XP_006535412.1	76	0	0.00
Kin	NP_079556.1	342	0	0.00	Slx	NP_001129948.1	61	0	0.00
Itih2	NP_034712.2	930	6	6.80	Gm14525	NP_001155836.1	61	1	17.28
Itih5	NP_766059.1	417	7	17.69	Gm6121	NP_001108226.1	33	1	31.94
Sfmbt2	NP_001185737.1	487	6	12.98	Gm10230	NP_001346647.1	5	1	210.78
Prkcq	NP_032885.1	1449	11	8.00	Gm10058	NP_001103439.1	18	6	351.30
Pfkfb3	NP_001171229.1	723	5	7.29	Gm4836	NP_033555.1	5	1	210.78

Rbm17	NP_690037.1	551	1	1.91	Gm10147	NP_001093389.1	4	2	526.95
Il2ra	NP_032393.3	861	4	4.90	Gm10096	NP_001096148.1	5	1	210.78
Il15ra	NP_032384.1	437	4	9.65	Gm10488	NP_001092795.1	157	2	13.43
Fbh1	NP_056607.1	996	7	7.41	Gm14632	NP_001094080.1	4	2	526.95
Ankrd16	NP_796242.2	977	4	4.31	Gm10487	NP_001094079.1	5	1	210.78
Itga8	NP_001001309.1	829	3	3.81	Btbd35f29	XP_003689009.1	26	0	0.00
Mindy3	NP_001342468.1	173	1	6.09	Spin2f	XP_017174160.1	98	0	0.00
Pter	XP_017171927.1	399	1	2.64	Btbd35f26	NP_001361652.1	13	0	0.00
C1ql3	NP_694795.1	274	1	3.85	Btbd35f19	XP_001474977.1	20	0	0.00
Rsu1	XP_006497468.1	703	1	1.50	Spin2e	XP_003689010.1	136	2	15.50
Cubn	NP_001074553.1	496	5	10.62	Btbd35f25	XP_003689011.1	36	0	0.00
Trdmt1	NP_034197.3	769	2	2.74	Gm21637	NP_001257614.1	98	0	0.00
Vim	NP_035831.2	1576	7	4.68	Btbd35f22	XP_003689013.1	71	0	0.00
St8sia6	NP_665837.1	213	0	0.00	Gm5923	XP_003945758.1	136	2	15.50
Hacd1	NP_038963.3	540	1	1.95	Btbd35f2	NP_001161806.1	26	0	0.00
Stam	NP_035614.1	604	0	0.00	Gm21466	XP_006536399.1	136	3	23.25
Tmem236	NP_001074779.1	61	1	17.28	Btbd35f1	NP_082231.2	75	4	56.21
Mrc1	NP_032651.2	1019	6	6.21	Gm5926	XP_017174159.1	98	0	0.00
Slc39a12	XP_006497544.1	463	6	13.66	Btbd35f21	NP_001257598.1	70	0	0.00
Cacnb2	NP_075605.1	700	10	15.06	Btbd35f5	NP_001161809.1	116	4	36.34
Nsun6	NP_083226.3	1004	8	8.40	Btbd35f12	NP_001092803.3	15	0	0.00
Arl5b	NP_083742.3	1476	8	5.71	Gm2854	XP_006536402.1	136	3	23.25
Malrd1	NP_001297384.1	967	3	3.27	Btbd35f15	NP_001229944.1	69	0	0.00
Plxdc2	NP_080438.2	283	6	22.34	Btbd35f13	NP_001268963.1	14	0	0.00
Nebl	NP_083033.1	544	5	9.69	Btbd35f14	NP_001138510.1	62	2	34.00
H2al2a	NP_080903.1	975	2	2.16	Btbd35f6	NP_001361649.1	17	0	0.00
Skida1	NP_082593.2	152	2	13.87	Btbd35f9	XP_003689016.1	37	0	0.00
Mllt10	XP_006497440.2	731	11	15.86	Spin2g	XP_017174162.1	98	0	0.00
Dnajc1	NP_001177746.1	936	7	7.88	Btbd35f8	XP_003689017.1	38	0	0.00
Commd3	NP_680087.1	379	4	11.12	Gm10486	NP_001103440.1	49	0	0.00
Bmi1	XP_036013664.1	1390	7	5.31	Gm14819	NP_001103720.1	4	0	0.00
Spag6	NP_001001334.2	1019	5	5.17	Dock11	XP_006541469.1	480	2	4.39
Pip4k2a	NP_001342076.1	689	11	16.83	Il13ra1	NP_598751.3	497	1	2.12
Armc3	XP_036018461.1	284	8	29.69	Zcchc12	XP_017174119.1	602	1	1.75
Msrb2	XP_017174809.1	419	6	15.09	Lonrf3	XP_006541467.1	1313	3	2.41
Ptf1a	NP_061279.2	504	8	16.73	Gm14569	NP_001357872.1	52	2	40.53
4921504E06Ri	NP_081876.2	25	5	210.78	Pgrmc1	NP_058063.2	957	5	5.51
Otud1	NP_081991.1	371	9	25.57	Akap17b	NP_001075425.1	127	3	24.90
Etl4	XP_036016008.1	256	7	28.82	Slc25a43	NP_001078966.1	240	8	35.13
Arhgap21	XP_036018467.1	439	4	9.60	Slc25a5	NP_031477.1	1401	8	6.02
Enkur	NP_082004.1	283	3	11.17	C330007P06R	XP_006541473.1	112	4	37.64
Thnsl1	NP_808256.1	314	3	10.07	Ube2a	NP_062642.1	2118	8	3.98
Gpr158	NP_001004761.1	1032	4	4.08	Nkrf	NP_084167.3	665	6	9.51
Myo3a	NP_680779.3	1488	2	1.42	Septin6	NP_001170794.1	564	4	7.47
Gad2	NP_032104.2	1448	3	2.18	Sowahd	NP_776140.1	49	0	0.00
Apbb1ip	NP_062329.2	618	2	3.41	Rpl39	NP_080331.1	1067	1	0.99
Pdss1	NP_062374.2	811	3	3.90	Upf3b	NP_080849.1	817	7	9.03
Abi1	NP_001070661.1	820	3	3.86	Nkap	NP_080213.3	396	5	13.31
Acbd5	NP_001342566.1	396	4	10.65	Akap14	NP_001028957.1	218	2	9.67
Mastl	NP_080255.3	668	3	4.73	Ndufa1	NP_062316.1	538	3	5.88
Yme1l1	NP_038799.1	1527	6	4.14	Rnf113a1	NP_705723.1	369	3	8.57
Potegl	NP_081911.1	927	0	0.00	Gm9	NP_001028406.1	49	1	21.51
Nxph2	NP_032778.1	194	2	10.86	Rhox1	XP_011249273.1	62	3	51.00
Spopl	XP_006498475.1	650	3	4.86	Rhox2a	NP_083479.1	1	1	1053.90
Hnmt	XP_006497733.1	345	2	6.11	Rhox3a	NP_918952.2	78	4	54.05
Tbpl2	NP_001276618.1	1214	0	0.00	Rhox4a	NP_001034777.1	228	1	4.62
Il1f8	NP_081439.1	375	5	14.05	Rhox3a2	XP_003689018.1	81	8	104.09
Il1f9	NP_705731.2	385	5	13.69	Rhox4a2	XP_003689019.1	230	4	18.33
Il1f6	NP_062323.1	425	5	12.40	Rhox2b	NP_001092786.1	1	1	1053.90
Il1f5	NP_062324.2	452	4	9.33	Rhox4b	NP_067275.1	238	1	4.43
Il1f10	XP_036016239.1	380	4	11.09	Rhox2c	NP_001092788.1	19	1	55.47
Il1rn	NP_112444.1	788	4	5.35	Rhox3c	NP_001095927.3	3	1	351.30
Psd4	XP_017172556.1	492	1	2.14	Rhox4c	NP_001034778.1	0	0	#DIV/0!
Pax8	NP_035170.1	844	0	0.00	Rhox2d	NP_001075138.2	183	0	0.00
Cacna1b	NP_031605.2	1292	1	0.82	Rhox4d	NP_001034784.1	0	0	#DIV/0!
Ehmt1	XP_036018609.1	1273	2	1.66	Rhox2e	NP_001078817.1	3	1	351.30

Arrdc1	XP_030105250.1	478	0	0.00	Rhox3e	NP_001171898.1	79	2	26.68
Zmynd19	NP_080297.1	134	0	0.00	Rhox4e	NP_957688.2	0	0	#DIV/0!
Dph7	NP_080320.1	147	0	0.00	Rhox2f	NP_001078825.1	182	0	0.00
Mrpl41	NP_001026978.2	377	0	0.00	Rhox3f	NP_001035178.3	66	1	15.97
Pnpla7	XP_036017642.1	844	2	2.50	Rhox4f	NP_001034785.1	182	0	0.00
Nsmf	NP_001034475.1	168	1	6.27	Rhox3g	NP_001138878.1	36	0	0.00
Entpd8	NP_082369.1	885	2	2.38	Rhox2g	NP_001107625.1	0	0	#DIV/0!
Noxa1	NP_001157098.1	560	3	5.65	Rhox4g	NP_001034787.1	223	0	0.00
Nrarp	NP_080256.2	1120	1	0.94	Rhox3h	NP_001107629.1	8	1	131.74
Tor4a	XP_036016545.1	192	0	0.00	Rhox2h	NP_001093935.1	183	0	0.00
Nelfb	NP_067368.3	401	0	0.00	Rhox5	NP_032844.2	187	11	61.99
Stpg3	XP_030108009.1	9	0	0.00	Rhox6	NP_032981.1	358	2	5.89
Fam166a	XP_006498328.1	118	0	0.00	Rhox7a	NP_001020257.2	39	2	54.05
Tubb4b	NP_666228.1	1795	3	1.76	Rhox8	XP_011249316.1	244	2	8.64
Slc34a3	XP_006497742.1	148	3	21.36	Rhox9	NP_076383.1	195	2	10.81
Rnf224	NP_001028582.1	1	1	1053.90	Btg1b	XP_006541575.1	184	0	0.00
Cysrt1	NP_080691.2	33	0	0.00	Btg1c	NP_001030028.1	192	0	0.00
Rnf208	NP_789804.2	403	2	5.23	Rhox10	NP_001020021.1	258	2	8.17
Ndor1	XP_006498518.1	513	2	4.11	Rhox11	NP_941000.1	384	2	5.49
Tmem203	NP_796318.2	331	2	6.37	Rhox12	NP_001020254.1	241	2	8.75
Tprn	NP_780495.2	248	4	17.00	Rhox13	NP_001171931.1	283	8	29.79
Ssna1	NP_075953.1	500	1	2.11	Zbtb33	NP_001072981.1	587	1	1.80
Anapc2	NP_780509.2	761	1	1.38	Tmem255a	NP_001276656.1	171	0	0.00
Tmem210	NP_084331.1	3	0	0.00	Atp1b4	NP_598451.1	315	0	0.00
Lrrc26	NP_666229.1	515	0	0.00	Lamp2	NP_001277414.1	1324	5	3.98
Grin1	NP_001171128.1	1818	2	1.16	Cul4b	NP_001103612.1	1809	6	3.50
Man1b1	NP_001025154.1	456	3	6.93	Mcts1	XP_036017981.1	664	3	4.76
Dpp7	NP_114031.2	389	3	8.13	C1galt1c1	NP_067525.1	543	4	7.76
Uap1l1	NP_001028465.1	631	1	1.67	6030498E09R	NP_898949.2	0	0	#DIV/0!
Sapcd2	NP_001074554.1	252	0	0.00	Cypt15	NP_001170851.1	190	1	5.55
Entpd2	NP_033979.2	360	1	2.93	Cypt14	NP_001177961.1	81	0	0.00
Npdc1	NP_032747.2	374	4	11.27	Gria3	XP_017174035.1	897	3	3.52
Fut7	XP_011237324.1	345	4	12.22	Thoc2	NP_001028594.1	1123	5	4.69
Abca2	NP_001355553.1	484	5	10.89	Xiap	XP_011249278.1	1321	5	3.99
Paxx	XP_030105477.1	98	1	10.75	Stag2	XP_030107136.1	1260	7	5.86
Clic3	NP_081361.1	456	5	11.56	Sh2d1a	NP_001300617.1	714	2	2.95
Ptgds	NP_032989.2	656	4	6.43	Tenm1	XP_006541554.1	455	3	6.95
Lcn12	NP_084234.1	170	2	12.40	Tex13c1	NP_001182200.1	266	2	7.92
C8g	NP_001258706.1	533	3	5.93	Dcaf12l2	NP_780748.1	69	4	61.10
Fbxw5	NP_038936.1	527	0	0.00	Dcaf12l1	NP_848854.3	159	4	26.51
Traf2	XP_006497913.1	940	0	0.00	Prr32	NP_081117.1	37	3	85.45
Edf1	XP_030107780.1	953	1	1.11	Actrt1	NP_082790.1	1575	6	4.01
Mamdc4	NP_001074668.2	131	1	8.05	Smarca1	NP_001345547.1	1735	4	2.43
Phpt1	XP_036018543.1	448	1	2.35	Ocrl	NP_796189.2	936	2	2.25
Ajm1	NP_001005424.2	399	0	0.00	Apln	NP_038940.1	335	0	0.00
Rabl6	NP_001019787.2	713	1	1.48	Xpnpep2	NP_001276658.1	856	1	1.23
Ccdc183	NP_084135.1	16	1	65.87	Sash3	NP_001300678.1	629	3	5.03
Tmem141	NP_001035219.2	15	0	0.00	Zdhhc9	XP_036017773.1	256	3	12.35
Fcna	XP_006497738.1	642	2	3.28	Utp14a	NP_082552.1	696	2	3.03
Lcn8	NP_149157.1	208	7	35.47	Bcorl1	NP_848897.3	908	3	3.48
Lcn5	XP_006497729.1	130	6	48.64	Elf4	NP_001368805.1	393	1	2.68
Lcn6	XP_006498274.1	71	5	74.22	Aifm1	NP_001277293.1	1309	1	0.81
Lcn10	NP_828875.1	113	8	74.61	Rab33a	XP_036017751.1	879	5	5.99
Obp2a	XP_006497931.1	172	7	42.89	Zfp280c	XP_030107141.1	294	5	17.92
Bmyc	NP_075815.2	914	1	1.15	Slc25a14	XP_006541509.1	300	2	7.03
Obp2b	XP_017174567.1	119	6	53.14	Gpr119	NP_861416.1	279	3	11.33
Lcn3	NP_034824.2	263	8	32.06	Rbmx2	NP_775552.1	484	3	6.53
Lcn11	NP_001093925.1	111	7	66.46	Fsip2l	XP_006541529.1	52	0	0.00
Glt6d1	NP_001034184.1	260	9	36.48	Enox2	XP_036017782.1	131	0	0.00
Lcn9	XP_017174829.1	255	5	20.66	Arhgap36	NP_001343323.1	510	6	12.40
Sohlh1	NP_001001714.1	294	6	21.51	Olfr1320	NP_997123.2	117	0	0.00
Kcnt1	NP_001138875.1	838	6	7.55	Olfr1321	NP_997514.1	170	0	0.00
Camsap1	NP_001263288.1	354	7	20.84	Igsf1	XP_030107148.1	263	6	24.04
Ubac1	NP_598596.2	520	8	16.21	Olfr1322	NP_001011794.1	48	0	0.00
Nacc2	XP_006498322.1	332	0	0.00	Olfr1323	NP_666502.1	160	5	32.93
Lhx3	NP_034841.2	580	1	1.82	Olfr1324	NP_666404.1	133	0	0.00

Qsox2	NP_705787.1	191	4	22.07	Stk26	NP_598490.1	801	7	9.21
Ccdc187	NP_808509.1	371	1	2.84	Frmd7	XP_006541570.1	398	6	15.89
Gpsm1	NP_700459.2	637	5	8.27	Rap2c	NP_766001.1	1758	4	2.40
Dnlz	NP_081104.1	288	2	7.32	Mbnl3	XP_030107096.1	392	8	21.51
Card9	XP_006498198.1	475	10	22.19	Hs6st2	NP_001277396.1	390	7	18.92
Snapc4	XP_006497966.1	2300	13	5.96	Usp26	XP_017174134.1	557	1	1.89
Entr1	XP_030107861.1	144	4	29.28	Magea14	XP_030107373.1	81	1	13.01
Pmpca	XP_036018387.1	764	8	11.04	Gpc4	NP_032176.2	644	4	6.55
Inpp5e	NP_149125.1	556	1	1.90	Gpc3	NP_057906.2	878	5	6.00
Sec16a	XP_011237375.1	510	1	2.07	Ccdc160	XP_006541574.1	21	1	50.19
Notch1	NP_032740.3	2361	6	2.68	Phf6	NP_081918.1	1147	3	2.76
Egfl7	NP_942017.3	528	2	3.99	Hprt	NP_038584.2	2195	3	1.44
Agpat2	NP_080488.1	899	0	0.00	Plac1	NP_001344731.1	259	5	20.35
Dipk1b	NP_062807.2	134	8	62.92	Fam122b	NP_001160055.1	106	0	0.00
Lcn4	NP_034825.1	180	0	0.00	Fam122c	NP_082947.1	199	0	0.00
Abo	NP_001277373.1	107	1	9.85	Mospd1	XP_030107356.1	677	5	7.78
Surf6	NP_033324.1	669	9	14.18	Etd	NP_780356.1	75	0	0.00
Med22	NP_001365717.1	236	9	40.19	4933416I08Ri	NP_081976.1	451	1	2.34
Rpl7a	NP_038749.1	1289	5	4.09	Rtl8c	NP_082651.1	112	1	9.41
Surf1	NP_001258653.2	585	5	9.01	Rtl8a	NP_077132.1	72	1	14.64
Surf2	NP_038706.1	111	4	37.98	Rtl8b	NP_001018073.1	50	1	21.08
Surf4	NP_035642.1	820	6	7.71	Ct55	NP_083418.1	495	1	2.13
Stkld1	XP_006498172.1	2056	2	1.03	1700013H16F	NP_001186942.1	77	2	27.37
Rexo4	NP_001349805.1	573	1	1.84	Zfp36l3	NP_001009549.1	687	0	0.00
Adamts13	NP_001001322.1	436	9	21.75	Xlr	NP_001278677.1	196	1	5.38
Cacfd1	NP_084138.1	57	1	18.49	Gm14596	NP_001346630.1	8	2	263.48
Slc2a6	NP_001171098.1	2339	1	0.45	Gm14594	XP_986693.2	109	11	106.36
Mymk	NP_079652.1	140	1	7.53	Gm16405	XP_017173821.1	8	3	395.21
Adamtsl2	XP_036018614.1	214	3	14.77	Gm16430	XP_017173822.1	8	3	395.21
Fam163b	NP_780636.1	623	3	5.07	Gm14595	NP_001346632.1	8	3	395.21
Dbh	NP_620392.2	497	2	4.24	Gm14590	NP_001346639.1	8	3	395.21
Sardh	NP_619606.1	778	4	5.42	Gm7950	XP_017174147.1	8	3	395.21
Vav2	XP_006497921.1	811	8	10.40	Gm7958	XP_017174141.1	8	3	395.21
Brd3	NP_001107045.1	1593	4	2.65	Slxl1	NP_083457.1	109	10	96.69
Wdr5	NP_543124.1	1466	10	7.19	Gm6660	XP_017174149.1	8	3	395.21
Rxra	NP_035435.1	1224	5	4.31	Gm14625	XP_030107322.1	8	3	395.21
Col5a1	NP_056549.2	943	5	5.59	Gm16404	NP_001207426.1	8	3	395.21
Fcnb	NP_034320.1	542	1	1.94	Gm6664	XP_011245997.1	109	9	87.02
Olfm1	NP_001033702.1	832	9	11.40	3830403N18F	XP_011245979.1	39	0	0.00
Ppp1r26	XP_017173526.1	55	0	0.00	Gm773	XP_006528162.1	57	0	0.00
1700007K13R	NP_081316.1	32	0	0.00	Zfp449	NP_084415.1	278	0	0.00
Mrps2	XP_006497688.1	1192	6	5.30	Smim10l2a	NP_898851.1	0	0	#DIV/0!
Gbgt1	XP_017172754.1	250	9	37.94	Ints6l	XP_006528039.1	391	3	8.09
Ralgds	XP_006497861.1	580	9	16.35	Gm648	XP_011245900.2	278	2	7.58
Cel	NP_034015.1	605	8	13.94	Mmgt1	NP_666346.1	276	0	0.00
Gtf3c5	NP_683730.1	313	11	37.04	Slc9a6	XP_036017816.1	790	4	5.34
Gfi1b	NP_032140.1	709	10	14.86	Fhl1	NP_001070830.1	850	1	1.24
Tsc1	XP_006498283.1	829	6	7.63	Map7d3	XP_006528152.1	160	4	26.35
Spaca9	NP_081559.1	79	6	80.04	Adgrg4	NP_001349814.1	344	2	6.13
Ak8	XP_030107901.1	910	8	9.27	Brs3	NP_033896.2	457	2	4.61
Gtf3c4	NP_766565.2	177	3	17.86	Htatsf1	NP_082518.1	484	2	4.35
Ddx31	NP_001028466.2	1649	8	5.11	Vgll1	NP_573514.1	196	1	5.38
Barhl1	NP_001342363.1	387	3	8.17	Cd40lg	NP_035746.2	1114	1	0.95
Cfap77	NP_001160177.1	26	0	0.00	Arhgef6	XP_006528378.1	627	4	6.72
Ttf1	XP_036016405.1	2161	3	1.46	Rbmx	XP_036017759.1	588	2	3.58
Setx	XP_030107510.1	1306	2	1.61	Gm364	XP_006528071.1	260	0	0.00
Ntng2	NP_598007.1	450	1	2.34	Gpr101	NP_001028532.1	477	4	8.84
Med27	NP_081172.2	229	1	4.60	Zic3	NP_033601.2	761	1	1.38
Rapgef1	XP_006497646.1	748	0	0.00	Magea13	NP_076263.1	143	0	0.00
Gm13547	XP_030107713.1	105	0	0.00	Fgf13	NP_001343264.1	1020	2	2.07
Trub2	XP_006497988.1	980	3	3.23	F9	NP_032005.1	734	1	1.44
Coq4	NP_848808.1	267	0	0.00	Gm5637	XP_036018060.1	737	1	1.43
Slc27a4	NP_036119.1	528	2	3.99	Mcf2	XP_030107052.1	462	0	0.00
Urm1	NP_080891.1	412	0	0.00	Atp11c	NP_001001798.1	697	2	3.02
Cercam	XP_006498538.1	282	1	3.74	Gm7073	NP_001369236.1	115	1	9.16
Odf2	XP_017171768.1	423	0	0.00	Sox3	NP_033263.2	735	5	7.17

Gle1	NP_083199.1	485	1	2.17	Cdr1	NP_001160130.1	436	0	0.00
Sptan1	XP_006497872.1	1234	1	0.85	Ldoc1	NP_001018097.1	169	3	18.71
Wdr34	NP_001008498.2	323	1	3.26	Magec2	NP_001186925.1	106	0	0.00
Set	NP_076360.1	1368	1	0.77	Gm6760	NP_001170848.1	202	3	15.65
Pkn3	XP_030107418.1	1084	1	0.97	Magea9	XP_030107357.1	76	0	0.00
Zdhhc12	NP_001032851.1	144	1	7.32	Slitrk4	NP_001352041.1	326	2	6.47
Zer1	XP_036016703.1	193	0	0.00	Ctag2	NP_081578.1	257	7	28.71
Tbc1d13	NP_666364.1	343	3	9.22	Slitrk2	XP_030107197.1	377	5	13.98
Endog	NP_031957.1	634	3	4.99	Gm1140	XP_036017890.1	4	0	0.00
Spout1	NP_766248.3	500	3	6.32	Gm14692	XP_036017967.1	4	0	0.00
Kyat1	XP_006498378.1	510	3	6.20	4933436I01Ri	NP_080039.1	219	7	33.69
Lrrc8a	NP_808393.1	669	1	1.58	Fmr1	NP_032057.2	1530	7	4.82
Phyhd1	NP_758471.1	185	0	0.00	Fmr1nb	NP_778158.2	193	4	21.84
Dolk	NP_808316.1	472	2	4.47	Gm6812	NP_001092312.1	13	1	81.07
Nup188	NP_938046.2	291	0	0.00	Aff2	XP_006527883.1	343	8	24.58
Sh3glb2	NP_001349817.1	269	0	0.00	1700020N15F	NP_083592.1	27	1	39.03
Miga2	XP_006497656.1	141	0	0.00	Ids	NP_034628.2	204	1	5.17
Dolpp1	NP_065062.1	311	2	6.78	Eola1	NP_081063.1	41	2	51.41
Crat	NP_031786.2	420	1	2.51	Tmem185a	NP_001028500.1	133	2	15.85
Ptpa	NP_001349662.1	862	2	2.45	Gm14725	NP_001074945.1	234	1	4.50
Ier5l	NP_084520.3	136	0	0.00	Mamld1	XP_017174020.1	276	2	7.64
Cstad	NP_084413.1	47	0	0.00	Mtm1	NP_001157662.1	444	6	14.24
1700001O22R	XP_006498439.1	32	0	0.00	Mtmr1	XP_036017901.1	382	7	19.31
Ntmt1	NP_001343362.1	287	0	0.00	Cd99l2	NP_612182.1	139	6	45.49
Asb6	NP_579924.1	1299	1	0.81	Hmgb3	XP_006527905.1	958	3	3.30
Prrx2	XP_006497869.1	658	5	8.01	Gpr50	NP_034470.1	285	7	25.89
Ptges	NP_071860.1	370	0	0.00	Vma21	XP_006528324.1	149	5	35.37
Tor1b	XP_017174461.1	267	4	15.79	Prrg3	NP_001074604.1	39	1	27.02
Tor1a	NP_659133.1	464	4	9.09	Cnga2	XP_036017697.1	590	1	1.79
BC005624	NP_659134.1	203	6	31.15	Magea4	NP_064676.2	218	1	4.83
Usp20	XP_030107995.1	688	5	7.66	Gabre	XP_011245825.1	338	2	6.24
Fnbp1	NP_001033789.1	681	7	10.83	Magea10	XP_017173961.1	290	0	0.00
Gpr107	NP_848875.2	314	1	3.36	Gabra3	NP_001344744.1	688	3	4.60
Ncs1	NP_062655.1	1343	1	0.78	Gabrq	NP_001277364.1	355	3	8.91
Hmcn2	XP_006498564.1	943	4	4.47	Cetn2	NP_062278.2	1761	2	1.20
Ass1	NP_031520.1	912	4	4.62	Nsdhl	NP_035071.3	631	1	1.67
Fubp3	NP_001277477.1	528	2	3.99	Zfp185	NP_033575.3	652	3	4.85
Prdm12	NP_001116834.1	215	2	9.80	Pnma5	NP_001093931.1	132	0	0.00
Exosc2	NP_659135.1	887	3	3.56	Pnma3	NP_694809.1	407	0	0.00
Abl1	XP_006497684.1	2276	7	3.24	Xlr4a	XP_017174029.1	72	0	0.00
Qrfp	NP_906269.1	284	2	7.42	Xlr3a	NP_001104254.1	101	0	0.00
Fibcd1	XP_006498531.1	567	3	5.58	Xlr5a	XP_011245956.1	31	1	34.00
Lamc3	NP_035966.2	537	6	11.78	Gm14685	XP_036017945.1	44	0	0.00
Aif1l	NP_660126.1	1153	1	0.91	DXBay18	NP_001020555.2	44	0	0.00
Nup214	XP_006498008.1	704	4	5.99	Xlr5b	NP_001104763.1	17	1	61.99
Fam78a	NP_780720.1	97	0	0.00	Spin2d	NP_001229931.1	136	0	0.00
Plpp7	NP_663496.1	242	1	4.35	Xlr3b	NP_001075112.1	12	0	0.00
Prrc2b	XP_030105757.1	320	2	6.59	Xlr4b	NP_067340.2	26	1	40.53
Pomt1	NP_660127.1	453	1	2.33	F8a	NP_032004.2	101	1	10.43
Uck1	NP_035805.2	959	2	2.20	Xlr4c	NP_898917.2	51	0	0.00
Swi5	NP_001277481.1	162	0	0.00	Xlr3c	XP_006528010.1	101	0	0.00
Golga2	XP_006498554.1	577	0	0.00	Xlr5c	XP_030107218.1	43	0	0.00
Dnm1	XP_006497717.2	2589	1	0.41	Zfp275	NP_113682.2	187	2	11.27
Ciz1	XP_006498334.1	269	1	3.92	Zfp92	XP_017173952.1	138	0	0.00
1110008P14R	NP_932118.1	59	0	0.00	Trex2	NP_036037.1	310	1	3.40
Lcn2	NP_032517.1	1022	1	1.03	Haus7	NP_996987.1	175	2	12.04
Ptges2	NP_598544.2	495	0	0.00	Bgn	XP_006527821.1	1404	3	2.25
Slc25a25	NP_001157829.1	412	0	0.00	Atp2b3	XP_036017881.1	1659	6	3.81
Naif1	NP_919316.1	149	1	7.07	Dusp9	NP_083628.3	796	1	1.32
Fam102a	NP_705788.1	196	1	5.38	Pnck	XP_030107390.1	1307	4	3.23
Dpm2	NP_034203.1	291	1	3.62	Slc6a8	NP_598748.1	528	6	11.98
Pip5kl1	XP_017172887.1	508	1	2.07	Bcap31	NP_036190.2	876	8	9.62
St6galnac4	NP_001263354.1	248	0	0.00	Abcd1	NP_031461.1	478	5	11.02
St6galnac6	NP_001020482.1	183	0	0.00	Plxnb3	NP_062533.2	548	12	23.08
Ak1	XP_030102669.1	933	1	1.13	Srpk3	XP_006528235.1	742	11	15.62
Eng	NP_031958.2	911	0	0.00	Idh3g	NP_001343285.1	1086	15	14.56

Fpgs	XP_030103361.1	1442	4	2.92	Ssr4	NP_001159952.1	604	8	13.96
Cdk9	NP_570930.1	996	0	0.00	Pdzd4	XP_006528085.1	580	6	10.90
Sh2d3c	NP_038809.1	349	2	6.04	L1cam	NP_001361623.1	884	8	9.54
6330409D20R	NP_081805.1	0	0	#DIV/0!	Avpr2	XP_006527819.1	944	11	12.28
Ttc16	NP_001277492.1	737	1	1.43	Arhgap4	NP_001155896.1	548	13	25.00
Tor2a	NP_001356151.1	136	0	0.00	Naa10	NP_063923.1	855	13	16.02
Ptrh1	NP_848710.1	299	2	7.05	Renbp	NP_001158176.1	254	11	45.64
Cfap157	NP_079895.1	69	0	0.00	Hcfc1	NP_032250.2	1005	9	9.44
Stxbp1	NP_033321.2	1575	3	2.01	Irak1	NP_001171447.1	1636	8	5.15
Niban2	NP_666231.1	161	0	0.00	Mecp2	NP_001075448.1	1196	13	11.46
Lrsam1	XP_036016776.1	967	1	1.09	Opn1mw	NP_032132.1	453	3	6.98
Rpl12	NP_033102.2	1130	0	0.00	Tex28	NP_001119960.1	106	6	59.65
Slc2a8	NP_062361.1	2334	7	3.16	Tktl1	NP_113556.2	958	8	8.80
Garnl3	XP_006498545.1	221	3	14.31	Flna	NP_034357.2	1879	11	6.17
Ralgps1	XP_030106885.1	605	4	6.97	Emd	XP_011245823.1	509	2	4.14
Angptl2	NP_036053.2	683	0	0.00	Rpl10	NP_443067.1	1219	7	6.05
Zbtb34	NP_001078976.1	246	1	4.28	Dnase1l1	XP_017174102.1	268	1	3.93
Zbtb43	NP_001342539.1	296	3	10.68	Taz	XP_011245964.1	536	5	9.83
Lmx1b	NP_034855.3	781	3	4.05	Atp6ap1	NP_061264.1	489	6	12.93
Mvb12b	XP_006498433.1	224	3	14.11	Gdi1	NP_034403.1	969	4	4.35
Pbx3	NP_058048.1	614	1	1.72	Fam50a	XP_036017663.1	211	5	24.97
Mapkap1	NP_001349848.1	539	2	3.91	Plxna3	NP_032909.2	547	2	3.85
Gapvd1	XP_036018369.1	1444	5	3.65	Lage3	NP_079686.1	351	1	3.00
Hspa5	NP_071705.3	3339	3	0.95	Ubl4a	NP_663380.1	3044	5	1.73
Rabepk	XP_036016799.1	542	2	3.89	Slc10a3	NP_663381.1	266	2	7.92
Fbxw2	XP_006498180.1	890	6	7.10	Fam3a	NP_079749.1	53	1	19.88
Psmd5	NP_542121.2	921	4	4.58	G6pdx	NP_032088.1	1084	5	4.86
Cutal	NP_084297.1	524	3	6.03	Ikbkg	NP_001129539.1	1137	3	2.78
Phf19	NP_082992.1	494	4	8.53	Ctag2l1	XP_006528290.1	11	0	0.00
Traf1	NP_001313530.1	1099	6	5.75	Olfr1325	NP_666510.1	234	0	0.00
Hc	XP_017171158.1	1125	4	3.75	Gm5640	NP_001092772.1	17	0	0.00
AI182371	NP_001230032.1	598	3	5.29	Ctag2l2	NP_001092776.1	0	0	#DIV/0!
Cntrl	NP_001366203.1	888	0	0.00	Gm5936	NP_001075139.1	17	0	0.00
Rab14	NP_080973.1	1594	4	2.64	Gab3	NP_853615.1	135	0	0.00
Gsn	XP_006498053.1	1270	8	6.64	Dkc1	NP_001346341.1	1746	2	1.21
Stom	NP_038543.1	947	7	7.79	Mpp1	NP_032647.1	604	3	5.23
4930568D16R	NP_083739.1	96	4	43.91	Smim9	XP_030107238.1	0	0	#DIV/0!
4930402F06Ri	NP_001074178.1	0	0	#DIV/0!	F8	XP_006527853.1	551	3	5.74
Ggta1	XP_006497783.1	245	4	17.21	Fundc2	NP_080402.3	166	4	25.40
Dab2ip	NP_001107597.1	658	7	11.21	Cmc4	NP_001281206.1	59	2	35.73
Ttll11	XP_036018534.1	242	5	21.77	Mtcp1	NP_001034462.1	32	0	0.00
Ndufa8	NP_080979.1	706	1	1.49	Brcc3	NP_001345666.1	1222	2	1.72
Morn5	NP_083585.1	144	2	14.64	Vbp1	NP_035822.2	1150	6	5.50
Lhx6	NP_001076594.1	597	1	1.77	Rab39b	NP_780331.1	1488	5	3.54
Rbm18	NP_001153107.1	80	1	13.17	Pls3	XP_006527794.1	1299	4	3.25
Mrrf	NP_080698.1	1018	0	0.00	Cldn34b3	NP_001013782.1	3	0	0.00
Ptgs1	XP_006497853.1	881	0	0.00	Cldn34b4	NP_001343325.1	1	0	0.00
Olfr338	NP_667158.1	14	0	0.00	Cldn34d	NP_001188324.1	50	0	0.00
Olfr339	NP_667160.1	25	0	0.00	Tbl1x	XP_030107159.1	1393	3	2.27
Olfr340	NP_667162.1	14	0	0.00	Prkx	XP_006527973.1	2318	3	1.36
Olfr341	NP_667161.1	15	0	0.00	Pbsn	NP_059499.1	274	1	3.85
Olfr342	XP_030107398.1	46	0	0.00	Gm14744	NP_001079013.1	106	3	29.83
Olfr344	NP_666839.1	128	0	0.00	5430402E10R	NP_082044.1	46	0	0.00
Olfr345	NP_667156.1	22	0	0.00	Obp1a	NP_032780.2	157	3	20.14
Olfr346	NP_667149.1	66	0	0.00	Gm5938	NP_001079003.1	102	1	10.33
Olfr347	NP_667154.1	25	0	0.00	Obp1b	NP_001297257.1	154	2	13.69
Olfr348	NP_667155.1	11	0	0.00	Gm14743	NP_001119793.1	65	2	32.43
Olfr50	NP_667157.1	14	0	0.00	4930480E11R	NP_001171437.1	195	2	10.81
Olfr3	NP_996786.1	195	0	0.00	Prrg1	XP_036017917.1	85	10	123.99
Olfr350	NP_666838.1	74	0	0.00	Fam47c	NP_001158211.1	384	6	16.47
Olfr351	NP_667153.1	81	0	0.00	Cfap47	NP_001355647.2	54	5	97.58
Olfr352	NP_667151.1	10	0	0.00	Mageb16	NP_082301.1	269	5	19.59
Olfr353	NP_667152.1	78	0	0.00	Tmem47	NP_620090.1	345	6	18.33
Olfr354	NP_667150.1	102	0	0.00	4930595M18I	NP_775611.2	714	1	1.48
Olfr355	NP_666836.1	181	0	0.00	Dmd	XP_006527831.1	1798	4	2.34
Olfr356	NP_666835.1	116	0	0.00	Tsga8	NP_068698.1	48	0	0.00

Olfr357	NP_666834.1	132	2	15.97	Fthl17a	NP_082322.1	75	1	14.05
Olfr358	NP_997118.1	74	1	14.24	Tab3	NP_001345376.1	533	3	5.93
Olfr360	NP_666833.1	10	0	0.00	Gk	NP_032220.1	1149	3	2.75
Olfr361	NP_666480.1	82	1	12.85	Tasl	XP_017174109.1	115	3	27.49
Olfr362	NP_667262.1	89	0	0.00	Samt3	NP_082830.2	45	1	23.42
Olfr365	XP_017173989.1	283	2	7.45	Nr0b1	NP_031456.1	872	7	8.46
Olfr366	NP_001005569.1	52	0	0.00	Mageb4	NP_001028664.2	131	4	32.18
Olfr368	NP_666486.1	44	0	0.00	Il1rapl1	NP_001153875.1	755	7	9.77
Pdcl	NP_080452.2	477	1	2.21	Dcaf8l	XP_017173915.1	157	2	13.43
Rc3h2	NP_001094061.1	468	5	11.26	4932429P05R	NP_001078980.1	404	2	5.22
Zbtb6	NP_666365.1	247	1	4.27	4930415L06R	NP_001029052.2	340	1	3.10
Zbtb26	NP_950190.2	353	3	8.96	Mageb11	NP_001094920.1	51	0	0.00
Rabgap1	XP_006498058.1	843	7	8.75	Mageb2	NP_112448.1	135	2	15.61
Gpr21	NP_796357.1	232	4	18.17	Mageb6b1	NP_001108150.1	96	2	21.96
Strbp	XP_030104850.1	454	7	16.25	Mageb5b	NP_001192197.1	324	4	13.01
Crb2	NP_001157038.1	1045	8	8.07	Mageb5	NP_083123.1	303	2	6.96
Dennd1a	XP_036016813.1	227	6	27.86	Gm5071	NP_001242933.1	96	2	21.96
Lhx2	XP_006497796.1	1025	5	5.14	Mageb1	NP_034889.1	157	1	6.71
Nek6	XP_006498272.1	542	5	9.72	Mageb18	NP_776144.1	112	1	9.41
Psmb7	NP_035317.1	1370	6	4.62	Arx	NP_031518.2	536	1	1.97
Nr5a1	NP_620639.1	974	4	4.33	Pola1	NP_032918.1	1432	4	2.94
Nr6a1	XP_011237327.1	676	4	6.24	Pcyt1b	NP_808214.1	586	4	7.19
Olfml2a	NP_766442.1	275	2	7.66	Pdk3	NP_663605.1	917	3	3.45
Wdr38	XP_036018565.1	1646	2	1.28	Zfx	XP_006528030.1	654	4	6.45
Rpl35	NP_079868.1	948	0	0.00	Eif2s3x	NP_036140.1	1207	7	6.11
Arpc5l	NP_083085.1	427	0	0.00	Klhl15	XP_030107182.1	270	8	31.23
Golga1	NP_001277578.1	457	0	0.00	Fam90a1b	NP_001349810.1	28	0	0.00
Scai	NP_848893.2	223	0	0.00	Apoo	NP_080949.2	337	3	9.38
Ppp6c	NP_077171.1	2349	5	2.24	Maged1	NP_062765.1	367	2	5.74
Lrp1b	XP_006498526.1	967	2	2.18	Gspt2	NP_032205.2	1402	2	1.50
Kynu	NP_081828.1	764	2	2.76	Zxdb	NP_001074942.1	252	1	4.18
Arhgap15	NP_001288760.1	779	5	6.76	Spin4	NP_848868.1	146	4	28.87
Gtdc1	NP_001349916.1	102	4	41.33	Arhgef9	XP_006528055.1	842	4	5.01
Zeb2	NP_001342220.1	932	3	3.39	Amer1	NP_780388.2	372	1	2.83
Acvr2a	XP_006497686.1	1273	6	4.97	Asb12	NP_543134.1	1370	1	0.77
Orc4	XP_030107441.1	934	6	6.77	Zc4h2	NP_001276626.1	218	4	19.34
Mbd5	XP_017170400.1	243	7	30.36	Zc3h12b	XP_011245944.1	254	1	4.15
Epc2	NP_766251.3	738	6	8.57	Las1l	XP_006528399.1	334	2	6.31
Kif5c	NP_032475.2	2099	11	5.52	Msn	NP_034963.2	1309	0	0.00
Lypd6b	XP_017174756.1	235	5	22.42	Vsig4	NP_808457.1	766	2	2.75
Lypd6	NP_796113.1	268	4	15.73	Hsf3	NP_001297683.1	570	1	1.85
Mmadhc	XP_006497659.1	339	5	15.54	Heph	NP_034547.2	578	3	5.47
Rnd3	NP_083086.1	1777	5	2.97	Gpr165	NP_083812.1	168	1	6.27
Tas2r134	NP_954609.1	118	0	0.00	Pgr15l	NP_001028533.1	229	1	4.60
Rbm43	NP_001342537.1	645	2	3.27	Eda2r	NP_780749.3	320	5	16.47
Nmi	NP_001135420.1	285	1	3.70	Ar	NP_038504.1	2296	4	1.84
Tnfaip6	NP_033424.1	659	2	3.20	Ophn1	NP_001300684.1	609	6	10.38
Rif1	NP_001342343.1	680	2	3.10	Yipf6	XP_006528400.1	355	4	11.87
Neb	XP_036015153.1	574	2	3.67	Stard8	XP_006528061.1	282	5	18.69
Arl5a	NP_892039.1	1407	3	2.25	Efnb1	NP_034240.1	724	4	5.82
Cacnb4	NP_001032176.1	814	6	7.77	Pja1	XP_030107116.1	641	4	6.58
Stam2	NP_062641.1	584	1	1.80	Tmem28	NP_001074752.1	181	2	11.65
Fmnl2	XP_006498393.1	448	4	9.41	Eda	NP_034229.1	544	2	3.87
Prpf40a	XP_006498256.2	1227	3	2.58	Awat2	NP_808414.2	256	8	32.93
Arl6ip6	NP_075365.3	76	3	41.60	Otud6a	NP_001156663.1	344	6	18.38
Rprm	NP_075885.1	251	0	0.00	Igbp1	NP_032810.4	682	7	10.82
Galnt13	XP_006498150.1	449	6	14.08	Dgat2l6	NP_001107556.1	195	8	43.24
Kcnj3	XP_030103612.1	727	2	2.90	Awat1	NP_001074605.1	234	8	36.03
Nr4a2	XP_036015278.1	922	3	3.43	P2ry4	NP_065646.1	455	5	11.58
Gpd2	NP_034404.3	1019	1	1.03	Arr3	XP_006527928.1	854	7	8.64
Ermn	XP_036018610.1	650	3	4.86	Pdzd11	NP_001343313.1	563	11	20.59
Galnt5	NP_766443.2	332	1	3.17	Kif4	NP_032472.2	1415	6	4.47
Cytip	NP_631939.1	986	2	2.14	Gdpd2	NP_001345436.1	375	10	28.10
Acvr1c	XP_006498149.1	1242	5	4.24	Dlg3	XP_006528186.1	1300	10	8.11
Acvr1	NP_001341977.1	1404	3	2.25	Tex11	NP_113561.2	352	3	8.98
Upp2	NP_001276589.1	818	3	3.87	Slc7a3	XP_036017684.1	663	3	4.77

Ccdc148	NP_001349322.1	68	3	46.50	Snx12	XP_011245947.2	501	5	10.52
Pkp4	XP_036016888.1	728	1	1.45	Foxo4	NP_061259.1	965	2	2.18
Dapl1	NP_083999.1	182	4	23.16	Gm614	NP_001028534.1	4	0	0.00
Tanc1	XP_017174689.1	1194	5	4.41	Il2rg	NP_038591.1	927	3	3.41
Wdsub1	NP_001366324.1	569	2	3.70	Med12	NP_067496.2	952	6	6.64
Baz2b	XP_036018259.1	1473	6	4.29	Nlgn3	NP_766520.2	1031	1	1.02
Marchf7	XP_036018318.1	1029	3	3.07	Gjb1	XP_030107087.1	754	0	0.00
Cd302	NP_079698.2	388	1	2.72	Zmym3	XP_006528231.1	443	7	16.65
Ly75	NP_038853.2	636	7	11.60	Nono	XP_030107241.1	1014	5	5.20
Pla2r1	XP_006498991.1	395	3	8.00	Itgb1bp2	XP_030107215.1	466	2	4.52
Itgb6	NP_067334.1	810	6	7.81	Taf1	XP_017174004.1	1279	6	4.94
Rbms1	XP_030107768.1	630	2	3.35	Ogt	NP_631883.2	2140	10	4.92
Tank	NP_035659.1	359	3	8.81	Cxcr3	NP_034040.1	1087	1	0.97
Psmd14	NP_067501.2	1795	2	1.17	8030474K03R	XP_017174169.1	43	0	0.00
Tbr1	NP_033348.2	854	2	2.47	Nhsl2	XP_011245801.1	103	0	0.00
Slc4a10	NP_001229307.1	1191	4	3.54	Rtl5	NP_001265463.1	157	0	0.00
Dpp4	NP_034204.1	1152	4	3.66	Pin4	NP_081457.1	947	4	4.45
Gcg	NP_032126.1	2196	8	3.84	Ercc6l	NP_666347.2	1397	3	2.26
Fap	NP_032012.1	602	4	7.00	Rps4x	NP_033120.1	1100	7	6.71
Ifih1	NP_001157949.1	1310	8	6.44	Cited1	NP_001263403.1	201	0	0.00
Gca	NP_663498.1	401	7	18.40	Hdac8	NP_081658.1	2192	6	2.88
Kcnh7	XP_011237617.1	496	9	19.12	Phka1	XP_036017745.1	361	2	5.84
Fign	NP_068362.1	842	4	5.01	Gm9112	NP_001170836.1	26	1	40.53
Grb14	NP_057928.1	601	4	7.01	Dmrtc1b	NP_001034205.2	159	1	6.63
Cobll1	XP_030107602.1	376	5	14.01	Dmrtc1c1	NP_001136163.1	15	0	0.00
Slc38a11	XP_036018189.1	151	4	27.92	Dmrtc1c2	NP_001136162.1	15	0	0.00
Scn3a	NP_061202.3	783	7	9.42	1700031F05R	XP_006528375.1	0	0	#DIV/0!
Scn2a	NP_001092768.1	1436	8	5.87	Dmrtc1a	XP_006528343.1	86	3	36.76
Csrnp3	XP_030108093.1	355	3	8.91	Nap1l2	NP_032697.2	1498	8	5.63
Galnt3	NP_056551.2	405	5	13.01	Cdx4	NP_031700.1	586	9	16.19
Ttc21b	XP_006500326.1	341	5	15.45	Chic1	XP_006527823.1	199	9	47.66
Scn1a	NP_061203.2	1150	7	6.42	Tsx	XP_030107161.1	97	8	86.92
Scn9a	NP_001277604.1	737	4	5.72	Zcchc13	NP_083434.1	535	8	15.76
Scn7a	NP_033161.2	486	3	6.51	Slc16a2	NP_033223.2	493	7	14.96
Xirp2	NP_001019789.1	377	3	8.39	Rlim	NP_001345134.1	934	12	13.54
B3galt1	XP_036018124.1	243	1	4.34	Nexmif	NP_001070822.2	246	11	47.13
Stk39	XP_030107736.1	501	1	2.10	Abcb7	NP_033722.1	758	7	9.73
4933409G03R	NP_808319.1	84	0	0.00	Uprt	NP_001074658.1	472	2	4.47
4932414N04R	XP_017174796.1	8	0	0.00	Zdhhc15	NP_780567.1	259	4	16.28
Cers6	NP_001334090.1	466	1	2.26	Magee2	NP_444436.1	209	2	10.09
Nostrin	NP_853525.3	211	1	4.99	Pbdc1	XP_006528333.1	342	6	18.49
Spc25	XP_036018344.1	656	0	0.00	Magee1	NP_444431.3	429	3	7.37
G6pc2	NP_067306.1	387	3	8.17	Cypt2	NP_775612.2	150	0	0.00
Abcb11	NP_066302.2	1302	3	2.43	Fgf16	NP_085117.2	656	2	3.21
Dhrs9	NP_780721.1	254	0	0.00	Atrx	NP_033556.2	2021	8	4.17
Lrp2	NP_001074557.1	968	0	0.00	Magt1	NP_001363932.1	550	2	3.83
Bbs5	XP_036018499.1	382	1	2.76	Cox7b	NP_079655.1	404	1	2.61
Klhl41	NP_001074556.1	656	2	3.21	Atp7a	NP_001103227.1	1165	4	3.62
Fastkd1	NP_796218.2	158	0	0.00	Tlr13	NP_991389.1	954	0	0.00
Ppig	NP_001074555.1	2032	2	1.04	Pgk1	NP_032854.2	2077	3	1.52
Ccdc173	NP_001071152.1	18	0	0.00	Taf9b	NP_001161460.1	671	0	0.00
Phospho2	NP_082797.1	125	1	8.43	Fnd3c2	NP_001028596.2	0	0	#DIV/0!
Klhl23	NP_001347324.1	187	2	11.27	Fndc3c1	XP_011245923.1	24	0	0.00
Ssb	NP_001103615.1	1274	4	3.31	Cysltr1	NP_067451.2	403	4	10.46
Mettl5	NP_083556.2	217	1	4.86	Gm5127	XP_011245919.1	0	0	#DIV/0!
Ubr3	XP_036018419.1	387	5	13.62	Rtl3	NP_955762.1	116	6	54.51
Myo3b	NP_796350.2	1385	4	3.04	Lpar4	NP_780480.2	343	4	12.29
Sp5	NP_071880.1	253	4	16.66	P2ry10	NP_001344739.1	452	5	11.66
Erich2	NP_080020.2	109	2	19.34	P2ry10b	NP_001116067.1	159	3	19.88
Gad1	NP_032103.2	1701	6	3.72	Gpr174	XP_017173946.1	262	6	24.14
Gorasp2	NP_081628.3	562	0	0.00	Itm2a	NP_032435.2	300	3	10.54
Tlk1	XP_006499252.1	615	1	1.71	Tbx22	NP_851836.2	444	4	9.49
Mettl8	NP_001103982.1	301	0	0.00	2610002M06I	NP_080197.2	499	5	10.56
Dcaf17	XP_036018544.1	183	1	5.76	Tent5d	NP_001156576.1	243	7	30.36
Cybrd1	NP_082869.2	281	0	0.00	Wmp	NP_001028424.1	451	5	11.68
Dync1i2	NP_001185801.1	908	2	2.32	Brwd3	XP_006528177.1	1013	11	11.44

Slc25a12	NP_766024.1	541	6	11.69	Hmgn5	NP_057919.2	360	8	23.42
Hat1	NP_080391.2	1014	6	6.24	Sh3bgrl	NP_064373.1	464	7	15.90
Metap1d	XP_017174675.1	966	6	6.55	Gm6377	NP_001033006.1	95	0	0.00
Dlx1	XP_036013950.1	920	8	9.16	Pou3f4	NP_032927.1	566	4	7.45
Dlx2	NP_034184.1	689	8	12.24	Cylc1	NP_080410.3	300	6	21.08
Itga6	NP_001264899.1	1071	5	4.92	Rps6ka6	XP_011246008.1	501	11	23.14
Pdk1	XP_011237746.1	828	2	2.55	Hdx	NP_001074018.1	245	5	21.51
Rapgef4	NP_001191094.1	762	10	13.83	Tex16	XP_017174133.1	270	0	0.00
Map3k20	NP_075544.1	865	1	1.22	4933403O08F	XP_011246010.1	186	0	0.00
Cdca7	NP_080142.1	680	5	7.75	Apool	NP_080841.1	365	12	34.65
Sp3	NP_001018052.1	977	2	2.16	Satl1	NP_082931.1	319	10	33.04
Ola1	NP_080218.1	999	4	4.22	2010106E10R	NP_001162062.1	19	0	0.00
Sp9	XP_036018234.1	386	8	21.84	Zfp711	NP_808415.2	477	8	17.68
Cir1	NP_080130.2	348	1	3.03	Pof1b	NP_853557.1	242	6	26.13
Scrn3	XP_006500376.1	262	0	0.00	Chm	XP_006528444.1	818	1	1.29
Gpr155	XP_006500167.1	274	1	3.85	Dach2	XP_030107383.1	331	8	25.47
Wipf1	XP_036016242.1	503	0	0.00	Klhl4	NP_766369.2	363	9	26.13
Chrna1	NP_031415.2	699	9	13.57	Ube2dnl1	NP_001263325.1	1455	3	2.17
Chn1	XP_006498621.2	1145	0	0.00	Ube2dnl2	NP_001075130.1	1558	5	3.38
Atf2	XP_030102713.1	1099	1	0.96	Gm7429	NP_001357882.1	1165	1	0.90
Atp5g3	XP_030106038.1	830	2	2.54	Cpxcr1	NP_001028643.2	155	6	40.80
Lnpk	XP_030107916.1	204	11	56.83	H2ab2	NP_001268459.1	1475	6	4.29
Evx2	XP_006498791.1	292	11	39.70	H2ab1	NP_001096135.1	1314	5	4.01
Hoxd13	NP_032301.2	484	12	26.13	Tgif2lx2	NP_001136222.1	563	6	11.23
Hoxd12	NP_032300.2	277	11	41.85	Tgif2lx1	NP_694749.1	563	6	11.23
Hoxd11	NP_032299.1	348	12	36.34	Pabpc5	NP_444344.1	906	8	9.31
Hoxd10	XP_006498852.1	470	11	24.67	Pcdh11x	XP_006528449.1	603	4	6.99
Hoxd9	NP_038583.1	418	12	30.26	H2ab3	NP_001268460.1	1306	5	4.03
Hoxd8	NP_032302.2	340	11	34.10	Nap1l3	NP_620081.1	1097	8	7.69
Hoxd4	NP_034599.2	448	11	25.88	Cldn34c1	NP_001297581.1	12	0	0.00
Hoxd3	NP_034598.2	389	12	32.51	Srsx	XP_011246026.1	111	2	18.99
Hoxd1	NP_034597.2	409	11	28.34	Gm2411	XP_006528483.1	12	0	0.00
Mtx2	NP_058084.3	411	8	20.51	Cldn34c2	XP_011246022.1	12	0	0.00
Hnrnpa3	NP_666242.2	1889	4	2.23	Cldn34c3	XP_011246023.1	3	0	0.00
Nfe2l2	NP_035032.1	1008	1	1.05	Gm5945	XP_011246024.1	3	0	0.00
Agps	NP_766254.2	500	2	4.22	Vmn2r121	XP_036017636.1	32	0	0.00
Ttc30b	NP_082511.1	265	2	7.95	Rps12-ps11	XP_036018062.1	1285	1	0.82
Ttc30a2	NP_001074697.1	162	3	19.52	Rps12-ps12	XP_036018063.1	1285	1	0.82
Ttc30a1	NP_084464.3	189	2	11.15	Rps12-ps15	XP_036018065.1	1285	1	0.82
Pde11a	XP_006499517.1	546	6	11.58	Gm21571	XP_036018066.1	1285	0	0.00
Cyct	NP_034119.1	1178	3	2.68	Rps12-ps13	XP_036018067.1	1285	0	0.00
Rbm45	NP_700454.1	375	2	5.62	Rps12-ps14	XP_036018068.1	1285	0	0.00
Osbpl6	XP_030108129.1	407	9	23.30	Rps12-ps16	XP_036018069.1	1285	0	0.00
Prkra	XP_030106794.1	432	2	4.88	4932411N23F	NP_808373.1	2074	3	1.52
Pjvk	NP_001074180.1	159	3	19.88	Gm382	NP_001028413.2	622	1	1.69
Fkbp7	NP_001342008.1	908	1	1.16	Cldn34c4	NP_001268466.1	25	0	0.00
Plekha3	NP_112546.1	188	2	11.21	Diaph2	XP_006528644.1	742	3	4.26
Ttn	NP_082280.2	1763	1	0.60	Pcdh19	XP_006528622.1	513	5	10.27
Ccdc141	XP_006499979.1	277	1	3.80	Tnmd	NP_071717.1	299	5	17.62
Sestd1	XP_030106040.1	382	0	0.00	Tspan6	XP_030107288.1	389	6	16.26
Zfp385b	NP_848838.2	404	1	2.61	Srpx2	XP_006528669.1	377	8	22.36
Cwc22	NP_001349593.1	782	1	1.35	Sytl4	XP_006528614.1	406	3	7.79
Ube2e3	NP_001343324.1	2079	1	0.51	Cstf2	NP_001345982.1	1305	7	5.65
Itga4	NP_034706.3	966	2	2.18	Nox1	XP_006528578.1	569	2	3.70
Cerkl	NP_001041641.1	593	4	7.11	Xkrx	NP_899142.2	121	0	0.00
Neurod1	NP_035024.1	1188	4	3.55	Arl13a	NP_083223.1	347	1	3.04
Itprid2	XP_036018457.1	163	0	0.00	Trmt2b	XP_006528577.1	555	0	0.00
Ppp1r1c	NP_001277672.1	147	2	14.34	Tmem35a	NP_080515.1	352	1	2.99
Pde1a	NP_001342069.1	477	4	8.84	Cenpi	XP_017173829.1	656	4	6.43
Prdx6b	NP_796230.1	844	1	1.25	Drp2	NP_034208.2	651	1	1.62
Dnajc10	NP_077143.2	2171	3	1.46	Taf7l	XP_006528673.1	633	1	1.66
Frzb	NP_035486.1	778	3	4.06	Timm8a1	NP_038926.1	926	5	5.69
Nckap1	NP_001342340.1	728	4	5.79	Btk	NP_038510.2	1828	3	1.73
Dusp19	NP_077758.1	574	2	3.67	Rpl36a	NP_063918.1	962	5	5.48
Nup35	NP_081367.1	448	2	4.70	Gla	NP_038491.2	510	3	6.20
Zfp804a	NP_780722.2	691	1	1.53	Hnrnph2	NP_001300646.1	1052	5	5.01

Fsip2	NP_001357195.1	157	1	6.71	Armcx4	NP_001189429.2	151	2	13.96
Zc3h15	NP_081210.2	893	0	0.00	Armcx1	XP_036018010.1	121	0	0.00
Itgav	NP_032428.2	1053	3	3.00	Armcx6	XP_036017867.1	80	0	0.00
Fam171b	NP_780723.1	405	0	0.00	Armcx3	XP_017174115.1	247	0	0.00
Zswim2	NP_082240.1	393	0	0.00	Armcx2	XP_036017972.1	525	1	2.01
Calcrl	XP_036018296.1	451	0	0.00	Nxf2	NP_112549.1	692	3	4.57
Tfpi	NP_001342202.1	779	1	1.35	Zmat1	NP_780655.2	175	0	0.00
Ctnnd1	XP_036013729.1	862	2	2.45	Gm15023	NP_001092791.1	14	1	75.28
Selenoh	NP_001028338.1	377	0	0.00	Tceal6	NP_079631.2	639	2	3.30
Tmx2	NP_001277680.1	227	0	0.00	Pramel3	XP_006528697.1	69	5	76.37
Med19	NP_080161.1	268	0	0.00	Gm5128	NP_899143.2	12	0	0.00
Zdhhc5	NP_659136.1	298	0	0.00	Gm7903	NP_001229866.1	12	0	0.00
Clp1	NP_598601.1	519	0	0.00	AV320801	NP_808586.1	14	1	75.28
Ypel4	XP_036017785.1	266	0	0.00	Nxf7	NP_001366606.1	646	3	4.89
Serping1	NP_033906.2	788	1	1.34	Pramex1	XP_017174123.1	187	0	0.00
Ube2l6	NP_064333.2	1721	0	0.00	Tcp11x2	NP_082228.2	415	3	7.62
Smtnl1	NP_077192.1	539	1	1.96	Tmsb15a	XP_006528691.1	121	0	0.00
Timm10	NP_038927.2	1005	0	0.00	Armcx5	NP_001009575.2	28	5	188.20
Slc43a1	XP_036018485.1	206	1	5.12	Gprasp1	NP_080357.4	346	10	30.46
Rtn4rl2	XP_006499657.1	653	0	0.00	Gprasp2	NP_001346300.1	593	8	14.22
Slc43a3	NP_067373.2	314	2	6.71	Bhlhb9	NP_001091692.1	94	7	78.48
Prg2	XP_036015513.1	288	1	3.66	Arxes2	NP_084099.1	363	6	17.42
Prg3	XP_036018284.1	319	0	0.00	Arxes1	NP_083817.1	363	6	17.42
P2rx3	NP_663501.2	337	0	0.00	Bex2	NP_033879.1	693	12	18.25
Ssrp1	NP_892035.2	1872	0	0.00	Nxf3	NP_001019312.1	559	6	11.31
Tnks1bp1	XP_006499274.1	347	0	0.00	Bex4	NP_001346499.1	65	3	48.64
Aplnr	NP_035914.1	563	0	0.00	Tceal8	NP_001162050.1	118	4	35.73
Lrrc55	XP_006499531.1	711	0	0.00	Tceal5	NP_001352047.1	488	6	12.96
Olfr987	NP_001011785.1	12	0	0.00	Bex1	NP_033078.2	311	13	44.05
Olfr988	NP_001011534.1	2	0	0.00	Tceal7	NP_001120641.1	185	4	22.79
Olfr992	NP_667076.1	68	0	0.00	Tceal9	NP_035842.1	190	4	22.19
Olfr993	NP_666646.1	56	0	0.00	Bex3	NP_001103703.1	282	11	41.11
Olfr994	NP_666644.1	54	0	0.00	Kir3dl2	XP_006528601.1	237	1	4.45
Olfr995	NP_666645.1	53	0	0.00	Kir3dl1	NP_808417.2	211	1	4.99
Fads2b	NP_001075133.1	285	1	3.70	Tceal3	NP_001346207.1	506	6	12.50
Olfr996	NP_666648.2	80	0	0.00	Tceal1	NP_001343296.1	180	5	29.28
Olfr998	NP_666647.2	46	0	0.00	Morf4l2	NP_062742.4	891	11	13.01
Olfr1000	NP_001011695.1	93	0	0.00	Glra4	NP_034427.2	298	6	21.22
Olfr1002	NP_666784.2	246	0	0.00	Plp1	NP_001346048.1	1084	6	5.83
Olfr154	NP_038756.2	370	2	5.70	Rab9b	NP_795945.1	1014	6	6.24
Olfr1006	NP_666781.2	104	2	20.27	H2bw2	NP_081343.1	812	1	1.30
Olfr1008	NP_667077.1	256	3	12.35	Tmsb15l	NP_997150.1	866	1	1.22
Olfr1009	NP_666783.1	109	0	0.00	Tmsb15b2	NP_001074436.1	52	0	0.00
Olfr1012	NP_666779.1	46	0	0.00	Tmsb15b1	NP_001075452.1	43	0	0.00
Olfr1013	NP_666973.2	87	1	12.11	Slc25a53	NP_001075881.1	70	0	0.00
Olfr1014	NP_666780.2	46	0	0.00	Zcchc18	NP_001345368.1	636	2	3.31
Olfr1015	NP_666782.2	76	1	13.87	Fam199x	NP_666373.1	53	0	0.00
Olfr1016	NP_001011758.2	45	0	0.00	Esx1	NP_031983.2	588	1	1.79
Olfr1018	NP_666797.2	81	1	13.01	Il1rapl2	NP_109613.1	525	1	2.01
Olfr1019	NP_667226.1	192	5	27.45	Tex13a	NP_080745.2	696	0	0.00
Olfr1020	NP_666791.1	39	0	0.00	Nrk	XP_006528613.1	182	1	5.79
Olfr1022	NP_666800.2	148	0	0.00	Serpina7	XP_006528642.1	801	2	2.63
Olfr1023	NP_666798.2	66	0	0.00	4930513O06F	NP_083450.1	2	1	526.95
Olfr1024	NP_001005230.2	104	0	0.00	4933428M09I	NP_001344785.1	0	0	#DIV/0!
Olfr1026	NP_666795.2	52	0	0.00	Pwwp3b	XP_017173988.1	137	2	15.39
Olfr1028	NP_001011774.1	78	1	13.51	Trap1a	NP_035765.1	83	0	0.00
Olfr1029	NP_001011852.2	26	0	0.00	Radx	NP_780535.1	81	2	26.02
Olfr1030	NP_666799.1	34	0	0.00	Rnf128	NP_001241690.1	349	0	0.00
Olfr1031	NP_001011759.1	51	0	0.00	Tbc1d8b	NP_001074968.1	649	6	9.74
Olfr1032	NP_666790.2	115	4	36.66	Ripply1	NP_001033004.1	87	3	36.34
Olfr1033	NP_666789.1	70	1	15.06	Cldn2	XP_006528549.1	736	5	7.16
Olfr1034	NP_001011872.2	41	1	25.70	Morc4	NP_001180238.1	122	4	34.55
Olfr1036	NP_997025.1	127	0	0.00	Rbm41	NP_001165619.1	172	2	12.25
Olfr1037	NP_001011532.1	564	5	9.34	Nup62cl	XP_017174010.1	280	4	15.06
Olfr1039	NP_001011784.2	73	1	14.44	Dnaaf6b	NP_808589.1	72	2	29.28
Olfr1040	NP_997444.2	167	2	12.62	Frmpd3	NP_001307875.1	449	0	0.00

Olfr1042	NP_001011777.1	152	1	6.93	Prps1	NP_067438.1	1169	4	3.61
Olfr1043	NP_666788.1	14	0	0.00	Tsc22d3	NP_001070832.1	479	0	0.00
Olfr1044	NP_667222.1	58	1	18.17	Mid2	NP_001345296.1	449	2	4.69
Olfr52	NP_666794.1	67	0	0.00	Tex13b	NP_113558.1	104	0	0.00
Olfr1045	NP_667228.2	55	1	19.16	Vsig1	NP_001346072.1	112	0	0.00
Olfr1046	XP_017173947.1	117	3	27.02	Psmd10	NP_058579.2	1286	1	0.82
Olfr1047	NP_667223.1	101	1	10.43	Atg4a	NP_001366610.1	930	2	2.27
Olfr1048	NP_667225.1	279	3	11.33	Col4a6	NP_444415.2	596	5	8.84
Olfr1049	NP_667227.2	120	2	17.57	Col4a5	NP_001156627.1	753	9	12.60
Olfr1051	NP_997445.1	93	1	11.33	Irs4	NP_034702.2	544	6	11.62
Olfr1052	NP_667221.2	76	0	0.00	Gucy2f	XP_036017858.1	858	10	12.28
Olfr1053	NP_001171328.1	328	7	22.49	Nxt2	NP_001277461.1	448	8	18.82
Olfr1054	NP_667230.1	253	6	24.99	Kcne1l	NP_067462.1	210	8	40.15
Olfr1055	NP_001297480.1	33	0	0.00	Acsl4	XP_030107239.1	1126	10	9.36
Olfr1056	NP_667229.2	60	0	0.00	Tmem164	NP_001186289.1	116	7	63.60
Olfr1057	NP_997446.1	194	2	10.86	Ammecr1	NP_062369.1	259	8	32.55
Olfr1058	NP_666503.2	66	1	15.97	Rtl9	NP_001035524.2	110	6	57.49
Olfr1061	NP_997017.1	90	0	0.00	Chrdl1	NP_001107857.1	335	3	9.44
Olfr1062	NP_667289.2	103	0	0.00	Pak3	NP_001181975.1	1570	3	2.01
Olfr1065	NP_666520.2	137	2	15.39	Capn6	NP_031629.3	406	1	2.60
Olfr1066	NP_001011735.2	51	0	0.00	Dcx	XP_030107074.1	798	3	3.96
Olfr228	NP_666517.2	270	3	11.71	Alg13	NP_080523.2	320	5	16.47
Olfr1076	NP_666518.2	54	0	0.00	Trpc5	NP_033454.1	534	7	13.82
Olfr1079	NP_666519.1	54	0	0.00	Rtl4	NP_001028967.2	121	5	43.55
Olfr1080	NP_666521.2	61	0	0.00	Lhfpl1	XP_030107192.1	191	4	22.07
Olfr1082	NP_997557.2	51	0	0.00	Amot	XP_006528927.1	524	4	8.05
Olfr1084	NP_997018.2	72	1	14.64	Htr2c	NP_032338.3	1363	2	1.55
Olfr1085	NP_666801.2	48	0	0.00	Il13ra2	XP_036017719.1	341	0	0.00
Olfr1086	NP_666803.1	185	2	11.39	Lrch2	XP_017173944.2	611	1	1.72
Olfr1087	NP_667057.2	70	1	15.06	Gm15128	XP_006529068.1	0	0	#DIV/0!
Olfr1089	NP_001011771.2	100	2	21.08	Gm15080	NP_001185917.1	0	0	#DIV/0!
Olfr1090	NP_667058.1	29	0	0.00	Gm15107	NP_001075117.1	1	1	1053.90
Olfr1093	NP_001355135.1	434	7	17.00	Gm15114	NP_001076435.1	0	0	#DIV/0!
Olfr141	NP_861539.2	48	0	0.00	Gm15127	NP_001107872.2	0	0	#DIV/0!
Olfr1094	NP_666477.1	85	1	12.40	Luzp4	XP_006528966.1	59	2	35.73
Olfr1095	NP_666941.2	93	0	0.00	Ott	NP_035152.1	4	4	1053.90
Olfr1097	NP_667054.2	140	1	7.53	Gm15093	NP_001093390.1	0	0	#DIV/0!
Olfr1098	NP_667056.2	161	2	13.09	Gm15085	NP_001116206.1	2	2	1053.90
Olfr1099	NP_666979.1	258	4	16.34	Gm10439	NP_001032805.2	0	0	#DIV/0!
Olfr1100	NP_666805.1	137	1	7.69	Gm15097	NP_001185916.2	0	0	#DIV/0!
Olfr1101	NP_666802.2	75	0	0.00	Gm15091	NP_001116207.1	9	1	117.10
Olfr1102	NP_997037.2	154	1	6.84	Tmem29	NP_001345968.1	30	1	35.13
Olfr1104	NP_666978.2	142	0	0.00	Apex2	XP_030107378.1	694	2	3.04
Olfr1105	NP_001011825.1	67	0	0.00	Alas2	XP_030107059.1	957	3	3.30
Olfr1106	NP_666963.2	265	1	3.98	Pfkfb1	XP_030107112.1	725	2	2.91
Olfr1107	NP_667055.2	110	1	9.58	Tro	NP_001277701.1	603	2	3.50
Olfr1109	XP_017174009.1	43	0	0.00	Maged2	XP_011246184.1	419	2	5.03
Olfr259	NP_666981.2	136	0	0.00	Gnl3l	NP_001162071.1	972	2	2.17
Olfr1110	NP_666980.1	398	4	10.59	Fgd1	NP_032027.2	516	4	8.17
Olfr1111	NP_666804.2	72	1	14.64	Tsr2	NP_780355.2	759	2	2.78
Olfr1112	NP_666872.2	16	0	0.00	Wnk3	XP_030107222.1	283	4	14.90
Olfr1113	NP_997448.1	153	0	0.00	Fam120c	NP_932773.2	190	5	27.73
Olfr1115	NP_666409.2	111	0	0.00	Phf8	NP_001106825.1	650	7	11.35
Olfr1116	NP_001011734.1	216	0	0.00	Huwe1	XP_030107294.1	1741	10	6.05
Olfr1118	NP_997515.2	217	0	0.00	Hsd17b10	NP_058043.3	807	6	7.84
Olfr1120	NP_667240.1	40	0	0.00	Ribc1	NP_079936.1	166	4	25.40
Olfr1121	NP_666460.2	183	0	0.00	Smc1a	NP_062684.2	1638	8	5.15
Olfr1122	NP_667242.1	70	0	0.00	Gm15266	XP_003085350.1	966	0	0.00
Olfr1123	NP_666462.2	88	1	11.98	Iqsec2	XP_006528905.1	572	8	14.74
Olfr1124	NP_667239.2	14	0	0.00	Kdm5c	XP_006528832.1	2145	8	3.93
Olfr1126	NP_667048.2	222	0	0.00	Tspyl2	NP_084112.1	838	7	8.80
Pramel7	NP_839981.1	88	0	0.00	Gpr173	NP_001346379.1	272	3	11.62
Pramel6	NP_001343474.1	84	0	0.00	Cldn34a	XP_006529023.1	3	0	0.00
Olfr153	NP_996559.1	36	0	0.00	Shroom2	NP_001277613.1	299	1	3.52
Olfr1128	NP_666461.2	30	0	0.00	Gpr143	NP_035081.3	360	1	2.93
Olfr1129	NP_001011836.2	270	0	0.00	Usp51	NP_001131019.1	438	2	4.81

Olfr1130	NP_667049.2	59	0	0.00	Mageh1	NP_076277.1	207	1	5.09
Olfr1131	NP_666869.2	194	0	0.00	Foxr2	XP_017174032.1	546	3	5.79
Olfr1132	NP_667047.1	202	0	0.00	Rragb	XP_006528913.1	402	2	5.24
Olfr1133	NP_666463.2	152	0	0.00	Klf8	XP_006528915.1	484	3	6.53
Olfr1134	NP_667241.2	347	0	0.00	Ubqln2	NP_061268.2	2348	2	0.90
Olfr1135	NP_666871.2	286	0	0.00	Cypt3	NP_001297645.1	38	0	0.00
Olfr1136	NP_666870.2	32	0	0.00	Kctd12b	NP_780638.1	224	1	4.70
Olfr1137	NP_001011833.1	75	0	0.00	Spin2c	NP_001005370.1	140	0	0.00
Olfr1138	NP_666850.1	95	0	0.00	Samt1	XP_017174127.1	112	4	37.64
Olfr1140	NP_666853.2	55	0	0.00	Samt1b	XP_017174095.1	112	5	47.05
Olfr1141	NP_666848.1	105	0	0.00	Samt1c	XP_017174077.1	112	5	47.05
Olfr152	NP_666857.2	124	0	0.00	Gm15143	XP_011246186.1	112	5	47.05
Olfr1143	NP_666405.1	174	0	0.00	Gm5947	NP_001361145.1	112	5	47.05
Olfr1145	NP_666432.2	40	0	0.00	Samt1d	XP_017174078.1	7	7	1053.90
Olfr1148	NP_001011519.1	181	0	0.00	Gm5645	NP_001369321.1	112	5	47.05
Olfr1151	NP_666849.1	98	0	0.00	Gm16445	NP_001229961.1	112	5	47.05
Olfr1152	NP_001011834.1	94	0	0.00	Samt2b	NP_001243188.1	130	8	64.86
Olfr1153	NP_666851.2	119	0	0.00	Samt4	NP_083475.1	139	9	68.24
Olfr1154	NP_666858.2	191	1	5.52	Samt2	NP_001032244.1	16	7	461.08
Olfr1155	NP_666854.1	98	0	0.00	Gm15142	XP_001475488.1	16	6	395.21
Olfr1156	NP_667028.2	92	0	0.00	Cldn34b1	NP_001091449.1	3	0	0.00
Olfr1157	XP_017174033.1	186	1	5.67	Magea6	XP_017173895.1	120	1	8.78
Olfr74	NP_473432.2	59	1	17.86	Magea3	NP_064401.2	261	1	4.04
Olfr1158	NP_666856.2	36	0	0.00	Magea8	XP_006528791.1	115	2	18.33
Olfr1160	NP_666860.2	98	0	0.00	Magea2	NP_064400.1	175	1	6.02
Olfr1161	NP_667059.2	77	0	0.00	Magea5	XP_006528788.1	135	4	31.23
Olfr73	NP_473431.1	200	1	5.27	Magea1	NP_064399.1	220	0	0.00
Olfr1162	NP_001011835.1	117	0	0.00	Cldn34b2	NP_082787.1	51	0	0.00
Olfr1163	NP_666855.1	46	0	0.00	Sat1	NP_033147.1	446	5	11.82
Olfr1164	NP_666852.2	136	0	0.00	Acot9	NP_062710.2	255	3	12.40
Olfr1166	NP_666861.2	40	0	0.00	Prdx4	NP_001300640.1	1202	5	4.38
Olfr1167	NP_666406.2	64	0	0.00	Ptchd1	NP_001087219.1	348	2	6.06
Olfr1168	NP_666742.2	88	0	0.00	Phex	NP_035207.1	587	4	7.18
Olfr1170	NP_666743.1	163	0	0.00	Sms	NP_033240.3	810	5	6.51
Olfr1173	NP_997449.1	74	0	0.00	Mbtps2	NP_758511.1	378	6	16.73
Olfr1176	NP_666982.1	55	0	0.00	Yy2	NP_001092193.1	633	1	1.66
Olfr1178	NP_001011868.1	86	1	12.25	Smpx	NP_001239520.1	553	4	7.62
Olfr1179	NP_667128.2	29	0	0.00	Klhl34	NP_001075136.1	505	2	4.17
Olfr1180	NP_667129.2	170	1	6.20	Cnksr2	XP_017173996.1	933	4	4.52
Olfr1181	NP_001011816.1	160	0	0.00	Rps6ka3	NP_683747.1	644	5	8.18
Olfr1182	NP_001011535.1	64	0	0.00	Eif1ax	NP_079713.2	807	3	3.92
Olfr1183	NP_666740.1	75	0	0.00	Map7d2	NP_001074593.1	441	3	7.17
Olfr1184	NP_667034.1	179	1	5.89	Bclaf3	NP_001028644.2	44	4	95.81
Olfr1186	NP_666741.2	205	0	0.00	Sh3kbp1	NP_001361630.1	778	1	1.35
Olfr1188	NP_667130.2	11	0	0.00	Map3k15	NP_001156557.2	433	2	4.87
Olfr1189	NP_666983.2	199	1	5.30	Pdha1	NP_032836.1	1176	6	5.38
Olfr1193	NP_001011517.2	165	7	44.71	Adgrg2	XP_006528869.1	477	0	0.00
Olfr1195	NP_666964.1	153	1	6.89	Phka2	XP_006528742.1	483	6	13.09
Olfr1196	NP_666675.2	10	0	0.00	Ppef1	NP_035277.1	1752	8	4.81
Olfr1197	NP_001005225.1	10	0	0.00	Rs1	XP_030107130.1	312	5	16.89
Olfr1198	NP_997450.1	264	1	3.99	Cdkl5	NP_001019795.1	471	8	17.90
Olfr1199	NP_001355141.1	262	2	8.05	Gja6	NP_001001496.1	262	0	0.00
Olfr1200	NP_001005227.2	37	0	0.00	Scml2	XP_006528733.1	1097	8	7.69
Olfr1201	NP_667106.1	23	0	0.00	Rai2	NP_940801.2	145	10	72.68
Olfr1202	XP_017173918.1	80	0	0.00	Scml1	NP_001361133.1	542	8	15.56
Olfr1205	NP_667107.2	44	1	23.95	Nhs	NP_001074521.1	109	10	96.69
Olfr1204	NP_666674.2	194	0	0.00	Reps2	XP_017173924.1	350	9	27.10
Olfr1206	NP_001001810.2	23	0	0.00	Rbbp7	NP_033057.3	2223	4	1.90
Olfr1208	NP_666989.1	72	0	0.00	Txlng	NP_001277705.1	320	2	6.59
Olfr1209	NP_666672.2	63	0	0.00	Syap1	NP_080208.2	289	7	25.53
Olfr1211	XP_011237863.1	122	2	17.28	Ctps2	NP_001345327.1	1288	8	6.55
Olfr1212	NP_997023.1	76	0	0.00	S100g	NP_033919.1	261	6	24.23
Olfr1213	NP_667109.2	50	0	0.00	Grpr	NP_032203.1	533	5	9.89
Olfr1214	NP_667108.2	89	0	0.00	Rnf138rt1	NP_083118.1	87	0	0.00
Olfr1215	NP_666670.2	137	0	0.00	Ap1s2	NP_001277308.1	664	7	11.11
Olfr1216	NP_667104.2	117	0	0.00	Zrsr2	NP_033479.2	465	2	4.53

Olfr1217	NP_667112.1	56	0	0.00	Car5b	NP_851832.2	223	8	37.81
Olfr1218	NP_667029.2	216	0	0.00	Siah1b	NP_033199.1	765	4	5.51
Olfr1219	NP_667110.2	82	0	0.00	Cltrn	NP_065651.1	279	1	3.78
Olfr1220	NP_667111.2	62	0	0.00	Ace2	NP_001123985.1	644	3	4.91
Olfr1221	NP_667113.2	95	0	0.00	Bmx	NP_033889.2	990	3	3.19
Olfr1222	NP_001011860.1	135	1	7.81	Pir	XP_036017982.1	352	0	0.00
Olfr1223	NP_667103.2	66	0	0.00	Vegfd	NP_034346.1	478	1	2.20
Olfr1225	NP_667102.2	155	1	6.80	Piga	XP_017173917.1	797	4	5.29
Olfr1226	NP_667178.1	131	1	8.05	Asb11	NP_001300666.1	1355	5	3.89
Olfr1228	NP_667182.1	35	0	0.00	Asb9	NP_081303.1	1317	3	2.40
Olfr1229	NP_001011761.1	84	1	12.55	Mospd2	NP_001277452.1	1221	4	3.45
Olfr1230	NP_667000.1	76	0	0.00	Fancb	NP_001139553.1	310	0	0.00
Olfr1231	NP_666665.2	100	2	21.08	Glra2	NP_001344029.1	595	2	3.54
Olfr1232	NP_666435.1	386	3	8.19	Gemin8	XP_036017838.1	212	0	0.00
Olfr1233	NP_667183.1	206	1	5.12	Gpm6b	XP_036017716.1	862	5	6.11
Olfr1234	XP_017174101.1	156	7	47.29	Ofd1	NP_803178.2	355	0	0.00
Olfr1238	NP_667001.1	104	4	40.53	Trappc2	NP_079708.2	331	2	6.37
Olfr1239	NP_667181.1	130	0	0.00	Rab9	XP_036017941.1	915	5	5.76
Olfr1240	NP_667019.1	96	2	21.96	Tceanc	NP_001007578.2	434	0	0.00
Olfr1241	XP_017173903.1	268	0	0.00	Egfl6	NP_062270.1	361	2	5.84
Olfr1242	NP_667179.1	232	3	13.63	Tmsb4x	NP_067253.1	502	4	8.40
Olfr1243	NP_667180.1	61	0	0.00	Tlr8	NP_573475.2	1320	1	0.84
Olfr1245	NP_666999.2	102	0	0.00	Tlr7	NP_573474.1	1623	1	0.72
Olfr1246	NP_667003.2	64	0	0.00	Prps2	NP_080938.1	929	5	6.67
Olfr1247	NP_667177.2	44	0	0.00	Frmpd4	NP_001356163.1	1069	4	4.93
Olfr1248	NP_667002.2	84	0	0.00	Msl3	NP_001357721.1	657	5	10.69
Olfr1249	NP_001011796.2	129	0	0.00	Arhgap6	XP_006528757.1	548	5	13.74
Olfr1250	NP_667176.1	35	0	0.00	Amelx	NP_001075447.1	396	5	20.47
Olfr1251	NP_001011529.1	23	0	0.00	Hccs	NP_001317978.1	603	6	17.48
Olfr1252	NP_997451.1	162	1	6.51	Mid1	NP_001277441.1	484	8	31.67
Olfr1253	NP_666485.1	48	0	0.00	Asmt	NP_001295417.1	396	4	21.29
Olfr1254	NP_666687.1	69	0	0.00	Zfy1	NP_033596.3	418	9	45.38
Olfr1255	NP_667188.2	121	1	8.71	Uba1y	XP_030107423.1	1230	11	17.14
Olfr1256	NP_667194.1	28	0	0.00	Kdm5d	NP_035549.1	2002	12	10.53
Olfr48	NP_035120.1	80	0	0.00	Eif2s3y	XP_036018085.1	1151	13	18.31
Olfr1257	XP_036018116.1	53	0	0.00	Uty	XP_011246887.1	3538	14	5.96
Olfr1258	XP_017174105.1	28	0	0.00	Ddx3y	XP_006531666.1	1867	15	11.29
Olfr1259	NP_666453.1	27	0	0.00	Usp9y	NP_683745.2	1014	15	19.49
Olfr1260	NP_667192.1	32	0	0.00	Zfy2	NP_033597.2	446	15	41.70
Olfr1261	NP_666685.1	44	0	0.00	H2al2c	NP_001171040.1	1857	14	8.83
Olfr1262	NP_667185.1	13	0	0.00	Sry	NP_035694.1	888	18	22.49
Olfr1263	NP_667005.1	49	0	0.00	H2al2b	NP_001106866.1	1857	15	8.51
Olfr1264	NP_067343.1	187	0	0.00	Gm4064	NP_001257447.1	249	10	42.33
Olfr1265	NP_666455.1	119	0	0.00	Rbmy	NP_035383.2	422	9	22.48
Olfr140	NP_065261.1	81	0	0.00	Gm10256	NP_001257439.1	357	6	17.71
Olfr1269	NP_666454.1	26	0	0.00	Gm10352	NP_001257440.1	357	5	14.76
Olfr32	NP_035110.1	76	0	0.00	Gm21677	NP_001257442.1	422	6	14.98
Olfr1270	NP_667196.1	116	0	0.00	Gm21693	NP_001257443.1	233	0	0.00
Olfr1506	XP_017173795.1	52	0	0.00	Gm21704	NP_001257444.1	422	4	9.99
Olfr142	NP_667195.1	94	0	0.00	Gm21708	NP_001257445.1	422	3	7.49
Olfr1271	NP_667004.1	281	0	0.00	Gm3376	NP_001257441.1	354	1	2.98
Olfr1272	NP_667191.1	75	0	0.00	Gm20918	XP_017174340.1	96	1	10.98
Olfr1565	XP_017169937.1	52	0	0.00	Gm20914	XP_017174358.1	145	0	0.00
Olfr1274	NP_666375.2	13	0	0.00	Gm20873	XP_011249331.1	105	0	0.00
Ptprj	NP_001129129.1	769	2	2.74	Gm20830	XP_017174335.1	95	0	0.00
Nup160	NP_067487.1	751	6	8.42	Gm21244	XP_017174369.1	271	0	0.00
Fnbp4	NP_061298.2	294	6	21.51	Gm28998	XP_017174245.1	0	0	#DIV/0!
Agbl2	XP_030107540.1	168	3	18.82	Gm20826	NP_001095093.1	271	0	0.00
Mtch2	NP_001304170.1	598	8	14.10	Gm28576	XP_017174244.1	0	0	#DIV/0!
C1qtnf4	NP_080437.2	241	8	34.98	Gm20825	XP_017174271.1	104	0	0.00
Ndufs3	NP_080964.1	998	6	6.34	Gm20824	NP_001365551.1	0	0	#DIV/0!
Kbtbd4	NP_001298045.1	196	5	26.89	Gm20815	NP_001017394.2	139	1	7.58
Celf1	XP_017170784.1	818	9	11.60	Gm21292	NP_001360820.1	95	0	0.00
Rapsn	NP_033049.2	298	5	17.68	Gm21310	XP_011249339.1	125	2	16.86
Psmc3	NP_032974.2	1680	4	2.51	Gm20834	XP_017174334.1	94	0	0.00
Slc39a13	XP_006500164.1	353	10	29.86	Gm20737	XP_011249341.1	136	1	7.75

Spi1	NP_001365827.1	1485	5	3.55	Gm20777	XP_017174219.1	135	1	7.81
Mybpc3	NP_032679.2	413	7	17.86	Gm20828	XP_001473675.1	135	1	7.81
Madd	XP_030106111.1	429	5	12.28	Gm20812	XP_017174310.1	97	0	0.00
Nr1h3	NP_038867.2	1234	4	3.42	Gm21379	NP_001279986.1	1466	10	7.19
Acp2	XP_011237559.1	467	5	11.28	Gm20807	XP_011249343.1	135	1	7.81
Ddb2	NP_001349634.1	766	3	4.13	Gm21425	XP_011249344.1	135	2	15.61
Pacsin3	NP_112019.2	560	4	7.53	Gm21828	NP_001360843.1	834	4	5.05
Arfgap2	NP_001159496.1	752	5	7.01	Gm21440	NP_001360822.1	137	4	30.77
1110051M20I	NP_001298073.1	135	4	31.23	Gm21454	NP_001359462.1	208	3	15.20
Lrp4	NP_766256.3	289	0	0.00	Gm20773	XP_011249347.1	875	2	2.41
Gm13770	XP_036018667.1	272	0	0.00	Gm20836	XP_011249348.1	247	4	17.07
Ckap5	XP_030108029.1	1183	4	3.56	Gm20928	XP_011249349.1	136	3	23.25
F2	XP_030103259.1	2279	2	0.92	Gm30737	XP_011249350.1	3	0	0.00
Zfp408	NP_001334104.1	212	2	9.94	Gm20822	NP_001186260.1	136	3	23.25
Arhgap1	NP_001139374.1	537	3	5.89	Gm20877	NP_001186261.1	97	0	0.00
Atg13	XP_006499955.1	432	1	2.44	Rbm31y	NP_083246.1	1466	11	7.91
Harbi1	NP_001343447.1	104	0	0.00	Gm20833	XP_011249352.1	134	2	15.73
Ambra1	NP_766257.3	923	3	3.43	Gm20772	XP_011249353.1	134	2	15.73
Chrm4	XP_006498717.1	696	3	4.54	Srsy	NP_001358038.1	5	0	0.00
Mdk	NP_001278412.1	1166	1	0.90	Gm20831	NP_001096622.1	113	7	65.29
Dgkz	XP_006498604.1	379	2	5.56	Ssty1	NP_033246.2	262	1	4.02
Creb3l1	NP_036087.2	426	0	0.00	Gm20851	XP_011248182.1	874	1	1.21
Phf21a	NP_001349760.1	1358	1	0.78	Gm21660	XP_011248183.1	247	4	17.07
Large2	NP_001160105.1	173	0	0.00	Gm21678	XP_011248184.1	135	2	15.61
Pex16	NP_660104.2	287	0	0.00	Gm35134	XP_011249354.1	3	2	702.60
1700029I15Ri	NP_898935.1	8	0	0.00	Gm21763	NP_001360865.1	340	2	6.20
Mapk8ip1	NP_035292.2	780	2	2.70	Gm20910	XP_017174256.1	207	3	15.27
Cry2	XP_017170770.1	752	1	1.40	Gm20909	NP_001371163.1	130	4	32.43
Slc35c1	NP_997597.1	330	1	3.19	Gm20865	NP_001153613.1	132	3	23.95
Chst1	NP_001343481.1	492	1	2.14	Gm21065	XP_017174319.1	209	3	15.13
Syt13	NP_109650.1	663	1	1.59	Gm21366	XP_030107460.1	9	2	234.20
Prdm11	XP_030101794.1	138	1	7.64	Gm21469	NP_001360832.1	105	0	0.00
Trp53i11	XP_017174437.1	183	0	0.00	Gm21661	XP_017174279.1	925	0	0.00
Tspan18	NP_899003.1	123	1	8.57	Gm20925	XP_017174274.1	830	1	1.27
Cd82	NP_001129527.1	520	1	2.03	Gm20926	XP_017174352.1	190	1	5.55
Alx4	NP_031468.1	621	1	1.70	Gm20840	XP_017174294.1	762	1	1.38
Ext2	NP_001342004.1	381	2	5.53	Gm20809	NP_001153609.1	930	4	4.53
Accs	XP_036018215.1	415	0	0.00	Gm21412	XP_017174277.1	927	0	0.00
Accsl	NP_001028624.2	595	0	0.00	Gm21427	XP_017174276.1	927	0	0.00
Gm13889	NP_001138506.1	35	0	0.00	Gm21443	XP_017174278.1	927	0	0.00
Alkbh3	XP_030107903.1	232	1	4.54	Gm20895	XP_017174323.1	124	0	0.00
Hsd17b12	NP_062631.1	748	2	2.82	Gm20890	XP_030107466.1	0	0	#DIV/0!
Ttc17	NP_001342619.1	62	1	17.00	Gm20892	XP_017174217.1	143	0	0.00
Api5	NP_001292187.1	631	2	3.34	Gm21476	XP_017174324.1	123	0	0.00
Lrrc4c	NP_001349978.1	1248	3	2.53	Gm21488	XP_030107471.1	0	0	#DIV/0!
Iftap	NP_080868.1	186	2	11.33	Gm21506	XP_017174218.1	143	0	0.00
Rag2	XP_017172095.1	760	4	5.55	Gm20891	XP_017174213.1	143	0	0.00
Rag1	NP_033045.2	931	5	5.66	Gm20894	XP_030107462.1	9	1	117.10
Traf6	NP_033450.2	1592	8	5.30	Gm21642	XP_017174214.1	143	0	0.00
Prr5l	XP_036018491.1	147	2	14.34	Gm20810	XP_017174353.1	291	1	3.62
Commd9	NP_083911.1	320	5	16.47	Gm21184	XP_017174364.1	291	1	3.62
Ldlrad3	XP_036017810.1	181	6	34.94	Gm21201	XP_017174303.1	929	0	0.00
Trim44	NP_064663.2	182	6	34.74	Gm20853	XP_017174311.1	204	5	25.83
Fjx1	NP_034348.2	263	7	28.05	Gm20855	XP_030107464.1	9	1	117.10
Pamr1	NP_776110.3	330	9	28.74	Gm20798	XP_017174349.1	929	0	0.00
Slc1a2	NP_001070982.1	1280	5	4.12	Gm20896	XP_017174242.1	9	1	117.10
Cd44	NP_001034240.1	2173	6	2.91	Gm20898	XP_030107478.1	767	0	0.00
Pdhx	NP_780303.1	965	10	10.92	Gm20835	XP_030107451.1	0	0	#DIV/0!
Apip	NP_062709.3	416	11	27.87	Gm20905	XP_030107452.1	0	0	#DIV/0!
Ehf	XP_006498758.1	594	4	7.10	Gm20738	NP_997045.1	151	9	62.82
Elf5	NP_034255.2	553	4	7.62	Gm20897	XP_030107455.1	9	1	117.10
Cat	NP_033934.2	2940	2	0.72	Gm21865	XP_017174192.1	3	0	0.00
Abtb2	NP_849221.2	253	0	0.00	Gm20793	XP_017174212.1	134	1	7.86
Nat10	NP_694766.1	1004	3	3.15	Gm20795	XP_011249372.1	248	1	4.25
Caprin1	NP_001104760.1	735	5	7.17	Gm20827	XP_011249373.1	134	1	7.86
Lmo2	NP_001135807.1	925	6	6.84	Gm21783	NP_001360853.1	121	0	0.00

4931422A03R	NP_780370.2	0	0	#DIV/0!	Gm21247	XP_017174361.1	291	1	3.62
Fbxo3	NP_997598.1	339	3	9.33	Gm21170	XP_017174194.1	1	1	1053.90
Cd59b	XP_006499864.1	637	4	6.62	Gm21281	XP_017174332.1	291	1	3.62
Cd59a	NP_001104530.1	376	1	2.80	Gm28553	XP_017174283.1	0	0	#DIV/0!
A930018P22R	NP_080910.1	5	0	0.00	Gm21308	NP_001360827.1	111	0	0.00
D430041D05R	XP_006499550.1	318	7	23.20	Gm21209	XP_017174196.1	47	2	44.85
Hipk3	NP_001139296.1	1147	9	8.27	Gm21316	NP_001360825.1	96	0	0.00
Cstf3	NP_663504.1	1261	10	8.36	Gm20880	XP_017174197.1	121	0	0.00
Tcp11l1	NP_796164.1	142	8	59.37	Gm20881	XP_017174216.1	142	0	0.00
Pin1rt1	XP_006499551.1	691	0	0.00	Gm20883	XP_017174237.1	9	0	0.00
Depdc7	NP_659053.1	232	10	45.43	Gm21117	XP_017174289.1	11	0	0.00
Qser1	XP_006500522.1	169	10	62.36	Gm20884	XP_017174281.1	929	2	2.27
Prrg4	XP_030106194.1	145	8	58.15	Gm21387	XP_030107437.1	929	2	2.27
Ccdc73	NP_808268.2	49	8	172.07	Gm21396	XP_017174215.1	142	1	7.42
Eif3m	NP_663355.1	1046	12	12.09	Gm20920	XP_017174249.1	9	1	117.10
Wt1	NP_659032.3	1444	15	10.95	Gm21245	XP_011249374.1	142	1	7.42
Them7	NP_083023.1	483	0	0.00	Gm21249	XP_017174360.1	97	1	10.86
Rcn1	NP_033063.1	578	9	16.41	Gm21258	NP_001360814.1	65	5	81.07
Pax6	NP_001231127.1	1865	9	5.09	Gm21268	XP_017174329.1	929	1	1.13
Elp4	NP_076365.2	204	7	36.16	Gm21275	XP_017174293.1	765	1	1.38
Immp1l	NP_082536.1	489	8	17.24	Gm21285	XP_017174348.1	127	1	8.30
Dnajc24	NP_081268.1	585	7	12.61	Gm20747	NP_001020412.1	130	10	81.07
Mpped2	NP_001343483.1	273	10	38.60	Gm20929	XP_030107469.1	9	2	234.20
Arl14ep	NP_001349793.1	152	2	13.87	Gm21302	NP_001360824.1	247	5	21.33
Fshb	NP_032071.1	474	8	17.79	Gm20927	XP_017174297.1	929	1	1.13
Kcna4	NP_067250.2	1015	5	5.19	Gm20930	XP_017174260.1	111	2	18.99
Mettl15	NP_084066.2	337	1	3.13	Gm20931	NP_001360810.1	9	2	234.20
Kif18a	XP_006499322.1	1316	5	4.00	Gm21330	NP_001360833.1	290	2	7.27
Bdnf	NP_031566.4	2084	6	3.03	Gm21340	XP_030107482.1	246	5	21.42
Lin7c	NP_035829.1	746	6	8.48	Gm20913	XP_017174261.1	111	3	28.48
Lgr4	NP_766259.2	999	5	5.27	Sly	NP_963288.2	0	0	#DIV/0!
Ccdc34	XP_011238057.1	233	6	27.14	Gm21943	NP_001017393.1	298	11	38.90
Bbox1	NP_569719.1	1119	4	3.77	Gm21573	XP_017174331.1	289	1	3.65
Fibin	NP_080547.1	220	2	9.58	Gm20803	XP_030107475.1	107	1	9.85
Slc5a12	NP_001171095.1	373	0	0.00	Gm20804	XP_017174231.1	140	2	15.06
Ano3	NP_001121575.1	627	2	3.36	Gm21518	XP_017174201.1	52	2	40.53
Muc15	NP_766567.1	335	1	3.15	Gm20823	NP_001153615.1	139	10	75.82
A26c3	NP_001365522.1	920	1	1.15	Gm21530	XP_017174202.1	120	2	17.57
Olfr1275	XP_017173815.1	98	0	0.00	Gm21617	XP_017174221.1	142	3	22.27
Olfr1276	NP_666507.1	51	0	0.00	Gm21627	XP_017174247.1	9	0	0.00
Olfr1277	NP_666508.1	109	0	0.00	Gm21634	XP_017174347.1	110	1	9.58
Olfr1278	NP_666506.1	39	0	0.00	Gm20885	XP_017174288.1	11	0	0.00
Olfr1279	NP_666505.1	57	0	0.00	Gm21654	XP_017174282.1	929	2	2.27
Olfr1280	NP_667119.1	20	0	0.00	Gm20882	XP_030107436.1	929	2	2.27
Olfr1281	NP_001005568.1	64	0	0.00	Gm20907	XP_011249384.1	145	4	29.07
Olfr1282	NP_667118.2	167	0	0.00	Gm20908	XP_017174248.1	9	2	234.20
Olfr1283	NP_997119.1	199	0	0.00	Gm20921	XP_011249386.1	144	4	29.28
Olfr1284	NP_666493.1	103	0	0.00	Gm21171	XP_017174290.1	51	4	82.66
Olfr1285	NP_001357854.1	199	0	0.00	Gm20924	NP_001371165.1	102	4	41.33
Olfr1286	NP_997137.1	11	0	0.00	Gm20736	NP_001032837.2	16	0	0.00
Olfr1287	NP_001011773.1	91	0	0.00	Gm20936	XP_017174328.1	929	2	2.27
Olfr1288	NP_666512.2	174	0	0.00	Gm20934	XP_011249388.1	765	2	2.76
Olfr1289	NP_666516.1	40	0	0.00	Gm20933	XP_017174350.1	127	3	24.90
Olfr1290	NP_666374.2	11	0	0.00	Gm20852	NP_001153601.1	255	9	37.20
Olfr1294	NP_667096.1	11	0	0.00	Ssty2	NP_076035.3	211	9	44.95
Olfr1295	NP_666515.1	188	0	0.00	Gm20874	XP_017174203.1	51	4	82.66
Olfr1297	NP_667099.1	60	0	0.00	Gm20935	XP_017174330.1	99	0	0.00
Olfr1298	NP_667097.1	29	0	0.00	Gm20937	XP_017174204.1	2	0	0.00
Olfr1299	NP_667095.2	118	0	0.00	Gm21127	XP_017174230.1	140	4	30.11
Olfr1301	NP_667098.1	97	0	0.00	Gm20816	NP_001153616.1	116	8	72.68
Olfr1302	NP_667100.1	63	0	0.00	Gm20818	XP_017174363.1	289	3	10.94
Olfr1303	NP_666514.2	65	0	0.00	Gm21151	XP_017174298.1	929	2	2.27
Olfr1305	NP_666513.2	116	0	0.00	Gm21160	XP_017174304.1	203	3	15.57
Olfr1306	NP_001011803.2	54	0	0.00	Gm20932	XP_017174299.1	930	2	2.27
Olfr1307	NP_001011787.1	69	0	0.00	Gm21524	XP_017174295.1	248	3	12.75
Olfr1308	NP_997034.1	78	0	0.00	Gm21672	XP_030107473.1	110	0	0.00

Olfr1309	NP_666658.1	25	0	0.00	Gm21118	XP_011249392.1	198	0	0.00
Olfr1310	NP_666660.1	35	0	0.00	Gm20888	XP_017174205.1	5	0	0.00
Olfr1311	NP_666386.1	39	0	0.00	Gm20866	XP_017174206.1	120	1	8.78
Olfr1312	NP_666474.1	101	0	0.00	1700040F15R	XP_017174246.1	9	2	234.20
Olfr1313	NP_997033.1	55	0	0.00	Gm20842	XP_011249395.1	139	0	0.00
Olfr1314	NP_666661.2	42	0	0.00	Gm20843	XP_017174207.1	51	6	123.99
Olfr1316	NP_666953.1	19	0	0.00	Gm20844	XP_017174308.1	96	0	0.00
Olfr1317	NP_666659.1	11	0	0.00	Gm20846	XP_030107485.1	928	1	1.14
Olfr1318	NP_001011802.1	11	0	0.00	Gm20847	XP_017174321.1	767	1	1.37
Lpcat4	NP_997089.1	256	4	16.47	Gm20848	XP_017174320.1	767	1	1.37
Nutm1	NP_766109.1	164	2	12.85	Gm20800	XP_017174351.1	126	0	0.00
Nop10	NP_079679.1	980	2	2.15	Gm21683	XP_017174326.1	122	0	0.00
Slc12a6	NP_598410.2	487	3	6.49	Gm20902	XP_017174252.1	140	0	0.00
Emc4	NP_080795.1	444	2	4.75	Gm20903	XP_030107467.1	9	0	0.00
Katnbl1	NP_077216.1	233	0	0.00	Gm20805	XP_017174253.1	141	0	0.00
Emc7	NP_598510.1	356	3	8.88	Gm21076	XP_017174370.1	928	3	3.41
Chrm5	NP_991352.2	599	9	15.83	Gm20813	XP_017174371.1	112	1	9.41
Aven	NP_083120.2	203	2	10.38	Gm20814	XP_017174372.1	9	0	0.00
Ryr3	XP_017172194.1	815	8	10.35	Gm21111	XP_017174269.1	113	2	18.65
Tmco5b	XP_006500389.1	72	0	0.00	Gm20850	XP_030107472.1	9	0	0.00
Fmn1	NP_034360.2	1035	5	5.09	Gm21155	XP_017174300.1	930	2	2.27
Grem1	NP_035954.1	662	6	9.55	Gm21163	XP_017174359.1	291	3	10.86
Scg5	XP_036015750.1	1195	8	7.06	Gm21180	XP_030107484.1	110	2	19.16
Arhgap11a	NP_852081.2	906	6	6.98	Gm20859	XP_017174243.1	143	2	14.74
Gjd2	XP_017171134.1	629	3	5.03	Gm21638	XP_017174208.1	52	4	81.07
Actc1	NP_033738.1	2395	5	2.20	Gm21650	XP_017174209.1	52	4	81.07
Aqr	NP_033832.2	938	2	2.25	Gm20867	NP_001153614.1	116	9	81.77
Zfp770	XP_036017232.1	178	1	5.92	Gm20808	XP_017174301.1	930	2	2.27
Dph6	NP_079951.1	188	5	28.03	Gm20806	NP_001153607.2	148	9	64.09
Cdin1	XP_036018243.1	36	4	117.10	Gm20919	XP_017174306.1	930	2	2.27
Meis2	XP_030104081.1	778	4	5.42	Gm20917	NP_001153608.1	106	2	19.88
Tmco5	XP_011238048.1	217	4	19.43	Gm20916	XP_030107468.1	9	3	351.30
Spred1	NP_277059.1	549	5	9.60	Gm21242	NP_001360813.1	211	12	59.94
Fam98b	NP_080896.2	182	5	28.95	Gm21257	XP_017174318.1	204	4	20.66
Rasgrp1	NP_035376.1	997	6	6.34	Gm20841	XP_017174254.1	141	2	14.95
Thbs1	NP_035710.2	1415	3	2.23	Gm21282	XP_017174356.1	128	3	24.70
Fsip1	XP_006500238.1	169	4	24.94	Gm20911	XP_030107430.1	0	0	#DIV/0!
Gpr176	NP_958755.1	402	3	7.86	Gm20912	XP_017174355.1	128	3	24.70
Eif2ak4	NP_038747.2	1168	2	1.80	Gm20801	XP_017174327.1	122	4	34.55
Srp14	NP_033299.1	1119	5	4.71	Gm21317	XP_030107474.1	9	4	468.40
Bmf	XP_036014892.1	307	3	10.30	Gm21333	XP_017174312.1	247	7	29.87
Bub1b	XP_006498685.1	1676	3	1.89	Gm21344	XP_017174307.1	930	0	0.00
Pak6	NP_001028426.2	1151	3	2.75	Gm20862	XP_017174263.1	114	1	9.24
Ankrd63	NP_001075440.1	963	0	0.00	Gm20863	XP_017174368.1	930	0	0.00
Plcb2	NP_001277719.1	1092	7	6.76	Gm21380	XP_017174259.1	115	0	0.00
Ccdc9b	NP_001001982.2	37	0	0.00	Gm21394	NP_001365341.1	119	3	26.57
Phgr1	NP_001139116.1	38	0	0.00	Gm21409	XP_030107479.1	9	2	246.53
Disp2	NP_733481.1	780	1	1.35	Gm20854	NP_001153603.1	202	9	52.17
Knstrn	NP_080688.2	481	2	4.38	Gm21435	XP_017174270.1	116	0	0.00
Ivd	NP_062800.1	642	3	4.92	Gm20861	XP_017174367.1	930	0	0.00
Bahd1	XP_006499328.1	342	5	15.41	Gm21462	XP_017174366.1	930	0	0.00
Chst14	NP_082393.3	255	0	0.00	Gm21470	XP_017174365.1	144	2	20.91
Ccdc32	NP_955014.2	242	0	0.00	Gm21477	XP_030107483.1	9	1	180.15
Rpusd2	NP_775626.1	460	10	22.91	Gm20906	XP_017174264.1	9	1	195.17
Knl1	NP_083893.2	712	8	11.84	Gm21287	XP_017174354.1	131	1	14.63
Rad51	NP_035364.1	2095	5	2.52	Gm21996	XP_011249410.1	0	0	#DIV/0!

Drosophila melanogaster

**Locus**	**Protein product**	**Co-expressions and co-interactions**		**Co-localization likelihood fold-change (FC)**	**Locus**	**Protein product**	**Co-expressions and co-interactions**		**Co-localization likelihood fold-change (FC)**
		**Total**	**witihin ±10 neighbor genes**				**Total**	**witihin ±10 neighbor genes**	
l(2)gl	NP_722583.1	458	1	2.95	CG4328	NP_648567.2	184	2	7.35
Ir21a	NP_001097043.1	83	1	14.81	Lmx1a	NP_729801.1	187	2	7.23
Cda5	NP_001245807.1	83	1	13.57	CG10418	NP_648570.1	826	0	0.00

dbr	NP_608486.1	64	8	129.98	CrzR	NP_001261755.1	137	0	0.00
galectin	NP_001245813.1	101	0	0.00	Ncc69	NP_648572.1	174	0	0.00
CG11374	NP_608488.3	126	9	64.37	CG32104	NP_648573.2	379	0	0.00
net	NP_524820.2	83	0	0.00	Obp69a	NP_524039.2	96	0	0.00
Zir	NP_608489.2	303	11	28.87	Smyd4-2	NP_648574.1	276	0	0.00
CG11377	NP_001259790.1	110	13	88.75	SCaMC	NP_729802.1	232	0	0.00
Nhe1	NP_608491.3	386	13	23.96	ArfGAP1	NP_001261761.1	457	1	1.48
Sam-S	NP_524923.1	1025	15	9.89	toe	NP_524041.2	116	1	5.83
CG13694	NP_608492.1	28	14	337.95	eyg	NP_524042.2	192	1	3.52
CG4822	NP_722602.1	64	15	158.41	CG32102	NP_729804.1	0	0	#DIV/0!
CG3164	NP_722605.1	152	16	71.15	CG10616	NP_001246734.1	42	0	0.00
Gs1	NP_476570.1	873	1	0.77	CG10663	NP_001097592.1	72	0	0.00
CG31975	NP_722607.1	30	14	315.42	CG33725	NP_001287049.1	0	0	#DIV/0!
CG31974	NP_722609.2	36	13	244.08	CG10660	NP_001261763.1	0	0	#DIV/0!
CG11454	NP_608497.1	157	14	60.27	CG10657	NP_648579.3	76	0	0.00
CG42399	NP_001259801.1	407	13	21.59	CG10638	NP_729808.1	211	0	0.00
CG3709	NP_608500.1	256	12	31.68	CG10681	NP_648580.1	415	1	1.63
ND-15	NP_722612.1	408	11	18.22	CG10646	NP_648581.1	111	0	0.00
CG3436	NP_608501.1	602	0	0.00	vih	NP_648582.1	868	3	2.34
CG33635	NP_001027215.1	171	9	35.57	CG10654	NP_648583.2	89	0	0.00
spen	NP_722615.1	341	9	17.84	sti	NP_648584.1	913	2	1.48
mRpL10	NP_523440.1	215	0	0.00	tral	NP_001261775.1	687	0	0.00
CG11617	NP_608502.1	123	0	0.00	Gcn5	NP_648586.2	956	0	0.00
Tbc1d15-17	NP_608503.1	365	0	0.00	ver	NP_648587.1	78	0	0.00
kis	NP_001245819.1	1202	2	1.12	eIF2beta	NP_524043.1	713	2	1.90
CG13693	NP_608505.1	580	2	2.33	nst	NP_648588.1	520	1	1.30
Rpp30	NP_524749.1	337	0	0.00	Tsf2	NP_524044.1	237	0	0.00
CG3645	NP_608507.1	431	0	0.00	CG34242	NP_001097595.1	0	0	#DIV/0!
CG17075	NP_608508.1	61	0	0.00	Pmm2	NP_648589.1	477	1	1.42
CG3345	NP_608509.1	723	1	0.93	CG4069	NP_648590.1	343	0	0.00
mbm	NP_608511.1	184	1	3.67	sowah	NP_729816.4	431	4	6.27
CG11555	NP_608510.2	176	1	3.84	ara	NP_524045.2	249	3	8.14
CG17078	NP_001259811.1	13	0	0.00	caup	NP_524046.2	256	3	7.92
smo	NP_523443.1	462	0	0.00	mirr	NP_001261778.1	309	3	6.56
CG11601	NP_608513.1	28	0	0.00	SmD1	NP_524774.1	1037	1	0.65
CG3625	NP_001097046.1	41	0	0.00	Ptp69D	NP_524048.2	391	1	1.73
Amnionless	NP_608515.1	97	0	0.00	CG32112	NP_001261779.1	15	0	0.00
CG11562	NP_608516.1	36	0	0.00	CG32109	NP_729822.1	28	0	0.00
U2af38	NP_477208.1	504	2	2.68	Klc	NP_001261781.1	667	3	3.04
Stip1	NP_477354.1	802	0	0.00	CG10984	NP_729823.2	82	0	0.00
Pi3K21B	NP_477270.2	424	2	3.19	CG10973	NP_001034002.1	774	0	0.00
Plc21C	NP_995605.1	329	2	4.11	Vps13D	NP_729825.2	146	0	0.00
CG11912	NP_608517.1	45	0	0.00	Hip1	NP_648597.2	574	2	2.36
CG11911	NP_608518.1	213	1	3.17	CG32106	NP_001246736.1	26	0	0.00
CG33127	NP_787956.1	72	1	9.39	CG10969	NP_648598.1	33	1	20.48
CG43755	NP_722626.2	293	1	2.31	CG17666	NP_648599.1	704	0	0.00
CG31921	NP_722627.1	361	1	1.87	Atg1	NP_648601.1	516	3	3.93
Ent1	NP_722628.1	126	0	0.00	CG42709	NP_648600.1	162	2	8.34
al	NP_722629.1	307	1	2.20	Sap130	NP_729834.1	184	1	3.67
CG4213	NP_608520.1	3	0	0.00	CG32110	NP_729837.1	235	0	0.00
RpI135	NP_476708.1	1404	2	0.96	Sf3a2	NP_648603.1	681	3	2.98
AP-2alpha	NP_476819.2	649	3	3.12	CG42588	NP_648604.2	91	0	0.00
ebi	NP_477329.1	1052	3	1.93	CG10960	NP_648605.1	375	2	3.60
CG13690	NP_608521.1	799	1	0.85	CG10948	NP_729844.1	72	0	0.00
RpLP1	NP_476630.1	646	0	0.00	Wbp2	NP_648607.3	139	0	0.00
CG11885	NP_608522.1	260	0	0.00	Ent3	NP_648608.2	99	0	0.00
CG13692	NP_608523.1	445	1	1.52	CG32107	NP_001261789.2	0	0	#DIV/0!
BBS8	NP_608524.1	340	2	3.98	CG10943	NP_648609.1	6	0	0.00
ex	NP_476840.2	179	0	0.00	CG14120	NP_648610.1	49	0	0.00
crq	NP_787957.1	390	1	1.73	CG43894	NP_648611.2	6	0	0.00
shv	NP_608525.1	370	2	3.65	CG14118	NP_648612.1	4	0	0.00
CG4133	NP_608526.1	16	0	0.00	CG12520	NP_001097596.1	82	1	8.24
CG4297	NP_608527.2	0	0	#DIV/0!	CG32117	NP_729853.1	241	2	5.61
MED15	NP_608528.1	308	1	2.19	CG32115	NP_729854.1	0	0	#DIV/0!
cbt	NP_722636.1	268	1	2.52	CG10752	NP_648614.1	127	0	0.00
ush	NP_001259828.1	139	1	4.86	Or69a	NP_996070.1	100	0	0.00

lwr	NP_476978.1	1180	2	1.15	CG10748	NP_648615.1	1098	3	1.85
Spp	NP_523444.1	270	1	2.50	CG10749	NP_648616.1	1542	4	1.75
nAChRbeta3	NP_525098.1	184	0	0.00	CG11262	NP_648617.2	747	3	2.71
Ets21C	NP_523445.1	185	1	3.65	CG11263	NP_648618.1	110	0	0.00
rempA	NP_608530.2	263	2	5.14	Cul6	NP_648620.1	685	1	0.99
Tspo	NP_608531.1	254	1	2.66	MICAL-like	NP_648621.1	244	0	0.00
CG11835	NP_608532.1	332	1	2.04	CG11267	NP_648622.1	1121	6	3.62
CG2794	NP_608533.1	78	0	0.00	mRpL20	NP_524051.1	792	4	3.41
Nle	NP_477294.2	657	0	0.00	ste14	NP_648623.1	281	0	0.00
Sf3b1	NP_001259837.1	872	0	0.00	AdenoK	NP_648624.1	321	4	8.42
Ipk2	NP_608535.1	638	1	1.06	RpS12	NP_729866.1	1049	3	1.93
cold	NP_608536.1	165	0	0.00	Zmynd10	NP_648625.1	114	0	0.00
Ptth	NP_001285550.1	181	0	0.00	CG17672	NP_001036603.1	2	0	0.00
Pph13	NP_477330.1	182	0	0.00	Srrm1	NP_648627.1	588	0	0.00
Gsc	NP_001137762.2	241	0	0.00	RpS4	NP_729871.1	929	4	2.91
CG13689	NP_608538.2	12	0	0.00	Syx13	NP_524054.1	404	0	0.00
lectin-21Cb	NP_608539.2	60	0	0.00	CG11279	NP_648628.1	110	1	6.14
lectin-21Ca	NP_652642.2	35	0	0.00	CG14115	NP_648629.1	0	0	#DIV/0!
CG2839	NP_608540.1	24	0	0.00	CG34428	NP_001097597.2	0	0	#DIV/0!
ds	NP_523446.2	296	0	0.00	CG34429	NP_001097598.1	0	0	#DIV/0!
Hsp60B	NP_524925.1	1652	1	0.41	CG17300	NP_648631.1	1081	2	1.25
Eaat2	NP_001162844.1	377	0	0.00	CG11251	NP_648632.1	826	2	1.64
GABA-B-R3	NP_523447.2	95	0	0.00	trn	NP_001261796.1	254	3	7.98
CG12506	NP_608541.1	0	0	#DIV/0!	CG33262	NP_996071.2	11	3	184.34
CG13946	NP_652360.1	24	0	0.00	snky	NP_648633.2	218	2	6.20
CG13947	NP_608542.1	89	1	7.59	CG32119	NP_729879.1	395	3	5.13
Gr21a	NP_001259841.1	221	1	3.06	caps	NP_001303391.1	257	2	5.26
CG3544	NP_608543.1	463	1	1.46	Sfp70A4	NP_001163437.1	0	0	#DIV/0!
Pkg21D	NP_477213.1	753	1	0.90	CG42481	NP_001163438.1	14	2	96.56
Nnf1b	NP_722652.1	39	0	0.00	CG43147	NP_001246741.1	39	2	34.66
Dbp21E2	NP_608544.1	1023	4	2.64	SP	NP_524057.2	271	3	7.48
Saf6	NP_001259843.1	59	0	0.00	CG14113	NP_652431.1	209	1	3.23
Pex12	NP_608546.1	248	0	0.00	CG17687	NP_001368937.1	80	0	0.00
CG15880	NP_608547.2	61	0	0.00	Nplp2	NP_001303387.1	116	1	5.83
PGAP2	NP_608548.1	118	0	0.00	CG14111	NP_648636.2	1	0	0.00
Clp	NP_477156.1	783	3	2.59	SNCF	NP_648635.1	48	0	0.00
CG3662	NP_608549.2	122	0	0.00	CG14107	NP_648637.2	14	1	48.28
CG3862	NP_608550.1	236	1	2.86	ImpL1	NP_729885.1	61	1	11.08
dock	NP_722657.1	311	1	2.17	CG14110	NP_648638.1	7	0	0.00
drongo	NP_477239.2	449	0	0.00	CG10171	NP_648639.1	33	0	0.00
CG4291	NP_608551.1	258	2	5.24	Poc1	NP_001261799.1	219	0	0.00
kraken	NP_477265.1	24	0	0.00	sens	NP_524818.1	340	0	0.00
CG13949	NP_608552.1	0	0	#DIV/0!	CG10222	NP_648641.2	539	0	0.00
CG13950	NP_001334728.1	20	0	0.00	flr	NP_648642.1	318	0	0.00
aru	NP_722663.1	166	0	0.00	CG32121	NP_001097599.1	0	0	#DIV/0!
dbe	NP_477240.1	904	1	0.75	CG33263	NP_996072.1	0	0	#DIV/0!
PNUTS	NP_001259850.1	130	0	0.00	CG14106	NP_648643.1	131	0	0.00
ninaA	NP_476656.1	518	1	1.30	CG14105	NP_648644.1	61	0	0.00
CG15824	NP_608555.2	215	0	0.00	CG10713	NP_648645.1	0	0	#DIV/0!
Lsp1beta	NP_476624.1	115	0	0.00	CG10154	NP_001189089.1	17	1	39.76
GluRIIC	NP_608557.4	383	0	0.00	CG10725	NP_648647.1	144	1	4.69
CG4341	NP_608558.1	469	0	0.00	CG10140	NP_648648.2	23	0	0.00
IA-2	NP_608562.4	388	0	0.00	CG14109	NP_648649.1	7	0	0.00
CG4375	NP_722673.1	745	0	0.00	cmb	NP_729901.1	188	0	0.00
CG4415	NP_608561.2	458	0	0.00	JMJD7	NP_648651.1	175	2	7.72
S	NP_523450.1	121	1	5.59	CG10116	NP_648652.1	145	0	0.00
ast	NP_523451.2	53	1	12.75	CG10738	NP_729905.2	248	0	0.00
Atg4a	NP_608563.1	713	1	0.95	CG10089	NP_648654.1	680	3	2.98
CG4629	NP_722676.3	470	1	1.44	stv	NP_001261804.1	168	0	0.00
mtRNApol	NP_608565.1	449	1	1.51	Abp1	NP_648657.1	412	0	0.00
CG14339	NP_608566.1	32	0	0.00	Tgi	NP_648658.1	43	1	15.72
CG14340	NP_608567.2	0	0	#DIV/0!	Spt20	NP_001287062.1	214	4	12.63
Pino	NP_722679.1	80	0	0.00	Vps36	NP_648660.1	270	0	0.00
TBC1D23	NP_608569.1	136	2	9.94	Liprin-beta	NP_648661.1	60	0	0.00
Iris	NP_608570.1	21	2	64.37	CG10710	NP_648662.1	2	0	0.00
CG4577	NP_001259855.1	62	2	21.80	bru3	NP_001369050.1	492	2	2.75

MFS3	NP_608572.1	112	1	6.03	CG43184	NP_001246744.1	64	0	0.00
l(2)10685	NP_608573.1	675	1	1.00	CG8757	NP_648664.2	54	0	0.00
Tfb4	NP_608574.2	302	0	0.00	CG8750	NP_652429.1	123	0	0.00
Vps29	NP_608575.1	467	0	0.00	Tsp68C	NP_729926.1	27	1	25.03
capt	NP_001033870.1	782	0	0.00	Hml	NP_524060.2	357	1	1.89
Tango14	NP_608577.2	303	1	2.23	CG8745	NP_648665.1	293	0	0.00
CG5080	NP_608578.1	46	1	14.69	dysc	NP_001261810.1	276	0	0.00
IntS14	NP_608579.1	145	0	0.00	CG13737	NP_648666.1	1	0	0.00
CG14341	NP_608580.2	7	0	0.00	Rgl	NP_001261814.1	199	0	0.00
Plap	NP_524666.2	412	2	3.28	CG8833	NP_648669.1	246	0	0.00
CG31922	NP_722687.1	332	2	4.07	DCTN1-p150	NP_524061.1	473	1	1.43
Charon	NP_608581.2	26	0	0.00	CG32137	NP_729935.1	108	1	6.26
CG4887	NP_608582.2	450	2	3.00	Meics	NP_001261815.1	176	0	0.00
CG4896	NP_608583.5	362	3	5.60	Nxf3	NP_648672.1	394	0	0.00
CG5126	NP_608584.1	4	0	0.00	CG13738	NP_648673.1	2	0	0.00
Tgt	NP_608585.1	511	1	1.32	Hsc70-1	NP_524063.1	1421	0	0.00
CG5001	NP_001245832.1	280	1	2.41	CG17364	NP_001261816.1	1	0	0.00
CG5139	NP_608587.1	751	0	0.00	CG17362	NP_648676.2	14	0	0.00
CG43348	NP_001245834.1	0	0	#DIV/0!	CG9040	NP_648677.1	3	0	0.00
CG43349	NP_001245835.1	0	0	#DIV/0!	26-29-p	NP_620470.1	163	0	0.00
CG5011	NP_652358.1	28	0	0.00	CG17361	NP_648678.3	104	1	6.50
CG14342	NP_608588.2	1	0	0.00	CG17359	NP_648679.1	153	1	4.42
CG42329	NP_608590.2	39	0	0.00	Nprl3	NP_648680.2	188	0	0.00
CG5397	NP_608591.1	31	0	0.00	upSET	NP_648681.1	457	1	1.48
robo3	NP_001368960.1	264	0	0.00	Ptip	NP_729947.2	529	1	1.28
a5	NP_476998.1	281	0	0.00	endos	NP_648684.1	195	1	3.47
CG5440	NP_608594.2	763	1	0.89	CG6650	NP_648685.1	175	1	3.86
CG33923	NP_001027216.2	0	0	#DIV/0!	CG6661	NP_648686.3	736	0	0.00
CG33922	NP_001027217.1	0	0	#DIV/0!	Hsc70Cb	NP_001189103.1	1450	3	1.40
Cdlc2	NP_477408.1	1105	4	2.45	Neurl4	NP_648688.2	101	3	20.08
CG5556	NP_608595.2	828	1	0.82	CG6833	NP_648689.1	536	4	5.04
CG5561	NP_608596.1	691	1	0.98	CG13484	NP_648690.1	17	4	159.04
CG31924	NP_722701.2	558	0	0.00	Frl	NP_729954.2	184	4	14.69
CG5565	NP_608598.1	869	1	0.78	Pex1	NP_652016.1	329	3	6.16
CG31659	NP_722702.1	80	0	0.00	btl	NP_729956.1	420	1	1.61
NLaz	NP_001259867.1	185	0	0.00	CG8100	NP_648693.2	28	1	24.14
CG14346	NP_608599.1	12	0	0.00	Fbp1	NP_001261826.1	168	3	12.07
robo2	NP_001259868.1	266	0	0.00	Sox21a	NP_001261827.1	268	1	2.52
CG43401	NP_001245837.1	0	0	#DIV/0!	Sox21b	NP_001261829.1	265	2	5.10
CG43402	NP_001245838.1	0	0	#DIV/0!	D	NP_524066.1	238	1	2.84
CG31928	NP_722705.1	263	0	0.00	nan	NP_648696.2	256	1	2.64
CG33128	NP_787961.1	192	0	0.00	nuf	NP_001246749.1	326	1	2.07
CG31926	NP_722706.1	256	0	0.00	saturn	NP_729963.1	475	0	0.00
CG31661	NP_722707.1	227	0	0.00	CG7768	NP_648697.1	961	0	0.00
CG18131	NP_722710.1	48	0	0.00	CG7924	NP_648698.1	158	2	8.56
CG7420	NP_001259871.1	200	0	0.00	CG34244	NP_001097604.1	0	0	#DIV/0!
CG18132	NP_608603.1	400	0	0.00	CG7906	NP_001261835.1	60	2	22.53
halo	NP_001137765.1	129	1	5.24	fz	NP_001261836.1	487	0	0.00
Or22a	NP_523453.1	154	1	4.39	CG13482	NP_648700.1	13	0	0.00
Or22b	NP_477425.1	139	0	0.00	CG13481	NP_001097605.1	37	0	0.00
CG10869	NP_608605.1	758	2	1.78	CG43120	NP_001246754.1	22	0	0.00
CG31935	NP_608608.2	256	3	7.92	CG3868	NP_648702.1	207	0	0.00
haf	NP_001356954.1	213	2	6.35	stwl	NP_524068.2	166	0	0.00
CG14352	NP_608609.1	35	0	0.00	CG3919	NP_001261840.1	0	0	#DIV/0!
RFeSP	NP_722715.1	924	2	1.46	bbg	NP_648705.2	344	2	3.93
chinmo	NP_001188680.1	150	0	0.00	CG9592	NP_648706.2	126	1	5.36
cpb	NP_477005.1	488	1	1.39	CG4613	NP_648707.2	122	0	0.00
CG17660	NP_608612.3	124	0	0.00	CG43246	NP_001246756.1	0	0	#DIV/0!
mRpL48	NP_608613.1	316	1	2.14	Mpcp2	NP_524069.2	610	1	1.11
frtz	NP_001259880.1	100	0	0.00	Gbs-70E	NP_648708.2	252	3	8.05
Rim2	NP_001245840.1	382	0	0.00	CG34039	NP_001034012.2	132	0	0.00
Eno	NP_722721.1	1366	1	0.49	Lk	NP_524893.2	140	0	0.00
Rrp40	NP_722725.1	689	1	0.98	CG42758	NP_001189106.1	239	3	8.48
CG31937	NP_608616.2	86	0	0.00	HGTX	NP_001368954.1	203	2	6.66
CG17652	NP_608617.1	564	1	1.20	shd	NP_001261843.1	131	0	0.00
CG17646	NP_608618.1	117	0	0.00	CG9628	NP_729982.1	8	0	0.00

CG17712	NP_608619.1	184	0	0.00	RecQ5	NP_729983.1	352	0	0.00
CG17648	NP_608620.1	7	0	0.00	dlp	NP_524071.2	245	0	0.00
Gr22f	NP_722729.2	88	0	0.00	CG43121	NP_001246758.1	51	0	0.00
CG17650	NP_608621.1	1	0	0.00	CG4914	NP_648711.1	200	0	0.00
Gr22e	NP_722731.1	105	0	0.00	ome	NP_001137955.1	166	0	0.00
Gr22d	NP_001259882.1	93	0	0.00	CG5048	NP_648712.1	766	4	3.53
Gr22c	NP_722732.2	96	0	0.00	goddard	NP_648713.1	851	4	3.18
Gr22b	NP_001014456.1	117	0	0.00	CG13474	NP_648714.1	0	0	#DIV/0!
Gr22a	NP_722733.1	82	0	0.00	CG17177	NP_648715.1	1	0	0.00
CG31933	NP_722734.1	56	0	0.00	CG13476	NP_996076.1	785	2	1.72
CG31664	NP_001356883.1	0	0	#DIV/0!	CG13473	NP_648717.1	602	0	0.00
c-cup	NP_608623.2	956	3	2.12	CG13471	NP_648718.1	640	2	2.11
wry	NP_722737.3	117	2	11.55	CG42507	NP_001163443.1	0	0	#DIV/0!
CG31663	NP_001245843.1	70	2	19.31	Tsen34	NP_001036605.2	135	1	5.01
CG7295	NP_608628.1	317	1	2.13	CG9384	NP_648720.3	114	1	5.93
CG15358	NP_608629.3	24	0	0.00	CG17173	NP_648721.1	112	1	6.03
Wdr62	NP_001097051.1	163	0	0.00	mop	NP_648722.2	823	0	0.00
CG15357	NP_001259888.1	6	0	0.00	bmm	NP_001163445.1	330	2	4.10
CG33673	NP_001027218.2	147	0	0.00	Tdrd3	NP_648724.1	352	1	1.92
erm	NP_001259891.2	302	0	0.00	Helz	NP_001261848.1	406	2	3.33
Der-1	NP_608632.1	368	0	0.00	NHP2	NP_651965.1	1418	0	0.00
CG15356	NP_608633.1	170	0	0.00	gnu	NP_648726.1	185	0	0.00
CG15362	NP_608634.1	81	0	0.00	CG17839	NP_996085.2	33	0	0.00
CG7289	NP_608635.1	59	0	0.00	CG34245	NP_001189107.1	0	0	#DIV/0!
Got2	NP_722744.1	678	0	0.00	DCX-EMAP	NP_001261850.1	643	0	0.00
Npc2a	NP_608637.1	201	2	6.73	CG13465	NP_525000.1	41	4	65.94
Obp22a	NP_722746.2	133	0	0.00	BobA	NP_525087.2	75	4	36.05
CG33543	NP_001014458.3	34	0	0.00	Tom	NP_524073.2	79	4	34.22
CG15353	NP_652353.2	1	1	675.90	Brd	NP_476889.1	120	4	22.53
Nplp4	NP_652352.1	112	1	6.03	Ocho	NP_536775.2	47	4	57.52
CG4238	NP_001097053.2	562	3	3.61	CG3349	NP_648730.2	11	0	0.00
CG42296	NP_652638.2	24	0	0.00	CG5114	NP_001246760.1	367	0	0.00
lectin-22C	NP_001137766.1	459	0	0.00	mnd	NP_524074.1	183	1	3.69
Su(dx)	NP_476753.1	735	2	1.84	Zip71B	NP_001097608.1	89	0	0.00
Kebab	NP_001259900.1	12	0	0.00	mRpL39	NP_524075.2	957	0	0.00
Or22c	NP_523454.2	40	0	0.00	Prosbeta2	NP_524076.2	707	2	1.91
dpr3	NP_001014459.2	21	0	0.00	Reck	NP_648733.2	91	0	0.00
CG12674	NP_608644.1	300	0	0.00	Sytbeta	NP_652427.2	94	0	0.00
CG4259	NP_608645.1	61	0	0.00	CG32148	NP_730009.1	600	1	1.13
GlyP	NP_722762.1	916	1	0.74	Rpn12R	NP_996086.1	1227	2	1.10
tho2	NP_722763.1	655	1	1.03	ind	NP_996087.2	140	0	0.00
CG11723	NP_608647.1	56	0	0.00	CG43678	NP_001261854.1	0	0	#DIV/0!
TBCD	NP_608648.1	336	1	2.01	CG18581	NP_648736.1	0	0	#DIV/0!
AIF	NP_722765.2	530	2	2.55	CG12310	NP_648737.1	22	2	61.45
CG15382	NP_608650.1	3	0	0.00	CG43679	NP_001261855.1	0	0	#DIV/0!
aop	NP_722766.1	330	0	0.00	CG13461	NP_648738.1	3	1	225.30
CG10874	NP_722768.2	62	0	0.00	CG43680	NP_001261857.1	0	0	#DIV/0!
CG34172	NP_001097054.1	131	0	0.00	CG18649	NP_648739.2	81	4	33.38
CG31668	NP_001097055.2	15	0	0.00	EAChm	NP_648740.1	65	3	31.20
CG33124	NP_001097056.3	15	0	0.00	CG13460	NP_648741.2	15	2	90.12
CG15385	NP_608654.1	44	0	0.00	Plp	NP_001246761.1	432	1	1.56
Uch	NP_001259915.1	450	0	0.00	Or71a	NP_001246763.1	73	0	0.00
CG34174	NP_001097057.1	64	0	0.00	sstn	NP_648745.1	202	2	6.69
daed	NP_001259916.1	22	0	0.00	GlyRS	NP_730022.1	1126	3	1.80
CG15386	NP_652351.1	96	0	0.00	CTPsyn	NP_730023.1	1077	1	0.63
mio	NP_608656.1	162	0	0.00	CG13458	NP_648748.1	32	0	0.00
papi	NP_608657.1	154	0	0.00	Cep135	NP_001189108.1	205	2	6.59
CG15387	NP_001097058.1	2	0	0.00	CG16959	NP_001189109.1	5	1	135.18
sau	NP_608658.1	308	1	2.19	CG32847	NP_730026.1	347	1	1.95
Sec24CD	NP_001259918.1	484	1	1.40	CG13457	NP_648751.1	274	1	2.47
Tengl3	NP_608660.3	458	1	1.48	CG12316	NP_648752.1	41	0	0.00
Tengl2	NP_722779.1	129	0	0.00	Pdi	NP_524079.1	1064	4	2.54
Tengl1	NP_722780.1	422	1	1.60	CG32147	NP_730035.1	97	0	0.00
CG4267	NP_608661.1	23	0	0.00	Pex3	NP_648753.1	281	1	2.41
Ser12	NP_523456.1	92	1	7.35	FucTA	NP_648754.2	122	1	5.54
CG17239	NP_722784.1	340	0	0.00	Msh6	NP_648755.1	780	1	0.87

CG17234	NP_722785.1	82	1	8.24	Prp31	NP_648756.1	1022	2	1.32
Send1	NP_608662.1	137	1	4.93	CG7011	NP_648758.1	422	1	1.60
CG4270	NP_608663.1	912	5	3.71	CG6878	NP_648757.1	351	1	1.93
CG34049	NP_001033871.2	36	0	0.00	CG6888	NP_648759.1	1258	8	4.30
CG17242	NP_722787.1	22	0	0.00	Tollo	NP_524757.1	412	5	8.20
CG4271	NP_608665.1	15	0	0.00	Best4	NP_001097609.1	49	3	41.38
CG31681	NP_722789.1	34	1	19.88	Best3	NP_730039.1	58	0	0.00
CG42658	NP_001188682.1	0	0	#DIV/0!	CG7255	NP_001036608.1	124	4	21.80
CG17237	NP_608666.1	1081	3	1.88	Toll-6	NP_524081.1	399	2	3.39
robl22E	NP_608667.1	461	3	4.40	CG33259	NP_996090.1	0	0	#DIV/0!
CG31949	NP_722791.1	628	3	3.23	cocoon	NP_648764.1	396	1	1.71
CG16995	NP_608668.2	23	0	0.00	Ran-like	NP_524082.1	1608	3	1.26
CG9967	NP_608669.1	12	0	0.00	CG12355	NP_001097610.1	82	0	0.00
eys	NP_001259924.2	388	1	1.74	CG13455	NP_648765.1	21	0	0.00
Rab5	NP_523457.1	1217	1	0.56	CG7276	NP_996092.1	391	1	1.73
Axud1	NP_608673.1	86	1	7.86	CG7275	NP_648767.1	777	0	0.00
CG3597	NP_608674.1	137	1	4.93	CG7272	NP_648768.1	36	0	0.00
CG3609	NP_608675.1	174	1	3.88	CG7857	NP_648769.2	241	0	0.00
CG15390	NP_608676.2	125	0	0.00	CG7841	NP_648770.1	81	0	0.00
Atxn7	NP_608677.1	235	0	0.00	Z600	NP_524083.2	55	1	12.29
Eogt	NP_608678.1	144	0	0.00	gdl-ORF39	NP_001027126.1	23	0	0.00
PIG-Wa	NP_608679.1	92	0	0.00	MsrA	NP_001261879.1	456	2	2.96
CG3557	NP_001259936.1	517	1	1.31	CG13454	NP_648771.1	2	0	0.00
CG43750	NP_001259937.1	0	0	#DIV/0!	mex1	NP_001189110.1	117	1	5.78
VGlut	NP_001245848.1	329	1	2.05	yellow-k	NP_648772.1	16	2	84.49
CG18641	NP_608682.3	10	0	0.00	CG7945	NP_648773.1	213	1	3.17
CG31948	NP_722807.1	891	2	1.52	CG33986	NP_001034017.2	36	0	0.00
CG34448	NP_001097059.1	1	0	0.00	CG33985	NP_001034018.1	0	0	#DIV/0!
CG34447	NP_001097060.1	39	0	0.00	CG42729	NP_001034019.2	0	0	#DIV/0!
CG9886	NP_608684.1	148	1	4.57	CG42728	NP_001189111.1	0	0	#DIV/0!
dpp	NP_477311.1	892	0	0.00	obst-H	NP_001034020.1	0	0	#DIV/0!
Slh	NP_001137769.2	335	1	2.02	CG43248	NP_001246767.1	0	0	#DIV/0!
oaf	NP_787963.2	79	2	17.11	CrebA	NP_524087.3	165	1	4.10
CG3528	NP_608686.1	170	0	0.00	AGO2	NP_648775.1	703	2	1.92
CG3515	NP_608687.2	384	1	1.76	CG7739	NP_648776.1	115	2	11.75
Cyp309a1	NP_608688.3	90	0	0.00	SCCRO	NP_648777.1	205	1	3.30
Cyp309a2	NP_608689.2	104	0	0.00	dop	NP_648778.1	324	0	0.00
Rad1	NP_477440.1	326	0	0.00	CG16979	NP_648779.1	140	0	0.00
insv	NP_608690.2	158	1	4.28	mrn	NP_648780.1	393	0	0.00
Elba2	NP_608691.1	171	1	3.95	CG12301	NP_648781.1	699	0	0.00
ND-B17.2	NP_001137770.1	404	1	1.67	AIMP2	NP_648782.2	408	0	0.00
Arpc5	NP_608693.1	500	1	1.35	CG7656	NP_730059.1	791	1	0.85
Drp1	NP_608694.2	1055	2	1.28	RhoGAP71E	NP_730060.1	139	1	4.86
CG15394	NP_001097062.2	49	0	0.00	CG7650	NP_648786.1	133	0	0.00
CG15395	NP_608696.3	88	0	0.00	CG13449	NP_648787.2	38	0	0.00
CG43403	NP_001245849.1	0	0	#DIV/0!	comm3	NP_648791.2	32	0	0.00
CG9962	NP_608697.1	161	0	0.00	Phs	NP_648789.3	282	1	2.40
CG43851	NP_001259948.1	0	0	#DIV/0!	CG43083	NP_001246772.1	0	0	#DIV/0!
CG33125	NP_787964.2	161	0	0.00	Eig71Ea	NP_524088.2	27	0	0.00
Prosbeta4R1	NP_608698.2	902	4	3.00	Eig71Eb	NP_524089.2	3	0	0.00
Or23a	NP_523458.3	88	1	7.68	CG43084	NP_001246773.1	1	0	0.00
Prosbeta4R2	NP_001259950.1	1186	3	1.71	Eig71Ec	NP_524090.2	84	0	0.00
Trf4	NP_608699.1	474	6	8.56	Eig71Ed	NP_524091.1	23	0	0.00
Gr23a	NP_523459.3	79	0	0.00	Eig71Ee	NP_524092.2	97	0	0.00
CG31690	NP_995615.2	434	0	0.00	Eig71Ef	NP_524093.2	16	0	0.00
Trf5	NP_608700.2	333	3	6.09	Eig71Eg	NP_524094.2	21	0	0.00
CG31689	NP_001259954.1	58	0	0.00	Eig71Eh	NP_648794.3	19	0	0.00
Hrs	NP_722830.2	552	0	0.00	Eig71Ei	NP_648795.2	53	0	0.00
CG9961	NP_608704.2	1452	5	2.33	Eig71Ej	NP_648796.1	0	0	#DIV/0!
Cwc25	NP_608705.1	504	0	0.00	Eig71Ek	NP_648797.1	1	0	0.00
Pgk	NP_476676.1	1279	5	2.64	CG43082	NP_001246774.1	0	0	#DIV/0!
Bacc	NP_608706.1	95	0	0.00	CG7304	NP_996098.1	28	2	48.28
HemK2	NP_001027221.1	260	0	0.00	CG7579	NP_648799.1	28	2	48.28
Tnpo-SR	NP_001259961.1	271	0	0.00	Pgant8	NP_648800.1	27	2	50.07
Snx21	NP_608709.1	102	1	6.63	CG34452	NP_001097611.2	136	3	14.91
aph-1	NP_608710.1	123	2	10.99	CG34451	NP_001369052.1	117	3	17.33

Taf10b	NP_477418.1	314	1	2.15	comm2	NP_648801.2	64	1	10.56
Taf10	NP_477463.1	485	2	2.79	CG42571	NP_001163448.2	0	0	#DIV/0!
colt	NP_477221.1	248	1	2.73	CG42570	NP_001163449.1	18	0	0.00
HINT1	NP_608711.3	579	1	1.17	comm	NP_524095.1	146	2	9.26
CG15399	NP_608712.1	9	0	0.00	CG6244	NP_996099.1	80	1	8.45
Syt1	NP_722838.1	609	1	1.11	CG13445	NP_648803.1	0	0	#DIV/0!
daw	NP_523461.1	230	0	0.00	fwe	NP_648804.1	75	0	0.00
CG2964	NP_608713.2	1156	0	0.00	CkIIalpha-i1	NP_524096.2	44	0	0.00
G6P	NP_001097063.1	226	0	0.00	DCP2	NP_648805.2	579	1	1.17
Duox	NP_608715.2	426	2	3.17	dbo	NP_001261892.1	345	2	3.92
CG3123	NP_608716.1	0	0	#DIV/0!	CG12713	NP_648806.1	34	0	0.00
CG3119	NP_608717.2	133	0	0.00	CG18081	NP_648807.1	94	1	7.19
Cpr23B	NP_608718.1	13	0	0.00	CG15715	NP_648808.1	64	2	21.12
CG2975	NP_608719.1	134	0	0.00	meru	NP_730077.1	24	1	28.16
CG18558	NP_608720.2	121	0	0.00	pHCl-1	NP_001356981.1	119	0	0.00
CG18557	NP_608721.1	27	0	0.00	sff	NP_001261901.1	513	1	1.32
CG3117	NP_608722.2	15	0	0.00	Pka-C3	NP_730083.2	642	2	2.11
CG2983	NP_608723.2	141	0	0.00	GXIVsPLA2	NP_648815.1	76	1	8.89
CG3104	NP_608724.1	353	0	0.00	elgi	NP_648816.1	372	2	3.63
RpS21	NP_722853.1	662	1	1.02	CG17032	NP_648817.1	22	0	0.00
CG2991	NP_608725.1	236	0	0.00	l(3)72Ab	NP_648818.3	902	6	4.50
CG31694	NP_722858.1	155	2	8.72	CG10516	NP_648819.2	102	7	46.39
CG8813	NP_608727.1	680	0	0.00	brm	NP_001261906.1	1240	9	4.91
CG8814	NP_608729.1	181	1	3.73	CG17026	NP_648820.1	131	5	25.80
Prx6005	NP_523463.2	539	3	3.76	CG17029	NP_648821.1	175	6	23.17
Sbat	NP_722859.1	758	1	0.89	CG17028	NP_648822.1	124	7	38.16
betaggt-II	NP_524894.1	238	0	0.00	CG17027	NP_648823.1	126	8	42.91
NTPase	NP_722860.1	271	2	4.99	Arl1	NP_524098.2	734	11	10.13
lilli	NP_001334729.1	342	1	1.98	DNApol-delta	NP_524099.2	782	11	9.51
Rbp9	NP_001259974.1	893	2	1.51	Hip14	NP_001261910.1	377	5	8.96
Ts	NP_477367.1	1174	5	2.88	hzg	NP_648825.1	240	1	2.82
Rrp1	NP_476841.1	1532	8	3.53	mRpS31	NP_524100.1	236	0	0.00
gammaTub23(	NP_476804.1	618	2	2.19	mib1	NP_648826.2	1426	4	1.90
CG9641	NP_608731.1	2	0	0.00	Notum	NP_730096.2	377	2	3.59
CG3165	NP_608732.1	295	2	4.58	CG42717	NP_001189115.1	44	9	138.25
CG9643	NP_608733.1	191	0	0.00	CG42716	NP_001189116.1	44	8	122.89
Chd1	NP_001259975.1	1101	4	2.46	CG42538	NP_001163451.1	44	7	107.53
Bem46	NP_477372.1	116	2	11.65	CG5895	NP_648828.3	20	1	33.80
okr	NP_476661.1	994	4	2.72	Diap1	NP_524101.2	623	1	1.08
CG3558	NP_001137777.1	99	1	6.83	Mbs	NP_001246785.1	682	6	5.95
Ccdc85	NP_608734.2	97	5	34.84	CG33258	NP_001034027.2	34	6	119.28
CG17224	NP_722867.1	226	4	11.96	CG13075	NP_648831.1	133	6	30.49
CG17264	NP_608736.2	14	4	193.11	Tasp1	NP_648832.1	148	5	22.83
alpha4GT1	NP_608737.1	58	4	46.61	CG5235	NP_648833.1	215	0	0.00
CG3542	NP_608738.1	635	6	6.39	ClC-c	NP_730105.3	229	0	0.00
Sf3b2	NP_608739.1	707	1	0.96	CG13074	NP_648835.1	8	0	0.00
CG17219	NP_608740.2	198	0	0.00	ATPsynbetaL	NP_648836.2	1320	2	1.02
GABPI	NP_608741.1	90	1	7.51	IleRS-m	NP_648837.2	689	2	1.96
CG17258	NP_001259980.1	1	0	0.00	IntS9	NP_648838.3	456	0	0.00
SerRS	NP_608743.2	654	3	3.10	CG43295	NP_001246788.1	11	0	0.00
CG17260	NP_608744.1	5	0	0.00	Zn72D	NP_788510.1	337	6	12.03
cnir	NP_608745.1	189	0	0.00	Taf4	NP_001137958.1	392	2	3.45
CG17221	NP_608746.1	379	3	5.35	Strump	NP_648840.1	143	0	0.00
CG17261	NP_608747.2	345	0	0.00	Pgm1	NP_524675.1	872	1	0.78
FASN2	NP_001259986.1	346	1	1.95	SsRbeta	NP_524103.2	230	0	0.00
FASN1	NP_001137778.2	450	2	3.00	Trs20	NP_648841.1	236	8	22.91
toc	NP_001259988.1	25	0	0.00	elg1	NP_996103.1	692	3	2.93
PpD6	NP_524947.1	836	0	0.00	CG5157	NP_648843.1	277	0	0.00
CG15403	NP_608749.2	613	0	0.00	CG32152	NP_648844.1	631	1	1.07
ND-B14.5B	NP_608750.1	382	1	1.77	CG33796	NP_001027128.1	34	9	178.91
Mad	NP_001259992.1	642	1	1.05	CG33795	NP_001027129.2	34	9	178.91
gkt	NP_523465.2	241	1	2.80	CG33687	NP_001027130.2	68	7	69.58
Hydr2	NP_608751.2	176	1	3.84	CG33688	NP_001027131.1	32	8	168.98
CG44002	NP_608754.1	74	0	0.00	CG33689	NP_001027132.2	32	8	168.98
CG31698	NP_722883.1	12	1	56.33	CG33690	NP_001368948.1	32	8	168.98
CG15404	NP_608756.1	12	1	56.33	CG13073	NP_648845.1	34	11	218.67

CG34393	NP_608753.2	60	0	0.00	l(3)72Dn	NP_648846.2	690	11	10.78
CG3347	NP_722887.2	256	0	0.00	CG5027	NP_648847.3	245	0	0.00
OtopLb	NP_722888.1	32	0	0.00	PDCD-5	NP_648848.1	440	0	0.00
CG9664	NP_001245853.1	53	0	0.00	MED10	NP_648849.3	286	10	23.63
CG9663	NP_608760.2	80	0	0.00	Hsc20	NP_001097616.1	267	0	0.00
CG15406	NP_608761.2	444	5	7.61	l(3)72Dp	NP_730119.1	198	8	27.31
CG3277	NP_608762.3	51	0	0.00	l(3)72Dr	NP_730120.1	208	6	19.50
Fign	NP_608763.2	513	0	0.00	CG13055	NP_648851.1	26	0	0.00
CG8837	NP_608764.1	305	5	11.08	CG13054	NP_648852.1	29	0	0.00
CG33281	NP_995620.1	315	4	8.58	CG13053	NP_652421.1	0	0	#DIV/0!
CG33282	NP_001097067.1	334	4	8.09	CG34247	NP_001097617.1	1	0	0.00
CG3285	NP_608766.1	384	5	8.80	CG13071	NP_001189117.1	64	0	0.00
CG15408	NP_608767.1	323	4	8.37	CG13070	NP_648854.2	1	0	0.00
E23	NP_995622.1	109	0	0.00	CG13051	NP_001097618.1	0	0	#DIV/0!
CG8838	NP_608769.2	706	1	0.96	CG13069	NP_652418.1	5	0	0.00
GramD1B	NP_608770.4	98	0	0.00	CG4950	NP_648855.2	276	0	0.00
CG15412	NP_001097072.1	0	0	#DIV/0!	CG13068	NP_648856.1	20	2	67.59
CG9662	NP_001259997.1	187	0	0.00	CG34248	NP_001189118.1	8	2	168.98
Ptpa	NP_523466.2	231	0	0.00	CG13067	NP_648857.1	10	1	67.59
Sfp23F	NP_001162856.1	0	0	#DIV/0!	CG13066	NP_652417.2	43	4	62.87
CG42460	NP_001162857.1	0	0	#DIV/0!	CG13065	NP_648858.1	20	1	33.80
CG34175	NP_001097073.1	9	0	0.00	CG13050	NP_648859.1	0	0	#DIV/0!
Pgant2	NP_995625.2	158	1	4.28	CG13064	NP_996106.1	0	0	#DIV/0!
CG8840	NP_608774.1	683	1	0.99	CG13049	NP_730133.1	72	1	9.39
CG31952	NP_608775.1	0	0	#DIV/0!	CG13048	NP_648861.1	5	0	0.00
CG2772	NP_608776.1	110	0	0.00	CG13047	NP_648862.2	15	2	90.12
Snx1	NP_608777.1	575	0	0.00	CG13046	NP_648863.2	0	0	#DIV/0!
CG12795	NP_001097074.1	135	1	5.01	CG13045	NP_648864.1	6	1	112.65
Cog3	NP_608779.1	236	2	5.73	CG4962	NP_648865.1	86	5	39.30
Sec5	NP_608780.1	426	1	1.59	CG4982	NP_648866.1	24	0	0.00
CG3246	NP_608781.2	53	0	0.00	CG13044	NP_648867.1	39	4	69.32
msl-2	NP_523467.1	193	0	0.00	CG43249	NP_001246791.1	0	0	#DIV/0!
ND-PDSW	NP_651972.1	573	0	0.00	CG13043	NP_648868.2	82	4	32.97
Pif1	NP_608782.1	678	2	1.99	CG13063	NP_648869.1	7	3	289.67
CG31776	NP_722909.2	27	0	0.00	CG13042	NP_648870.1	1	0	0.00
Pgant4	NP_001137779.1	64	0	0.00	CG13062	NP_648871.3	7	0	0.00
Thor	NP_477295.1	470	1	1.44	Nplp3	NP_652710.1	119	1	5.68
CG15414	NP_608784.1	6	1	112.65	CG13041	NP_648872.1	54	3	37.55
tim	NP_001334730.1	555	1	1.22	CG13060	NP_648873.1	171	6	23.72
CG31954	NP_722915.1	54	0	0.00	CG42718	NP_001189119.1	0	0	#DIV/0!
LeuRS	NP_787968.1	821	0	0.00	CG13059	NP_648874.2	63	5	53.64
CG17593	NP_608786.2	68	0	0.00	CG13040	NP_648875.1	4	0	0.00
CG3213	NP_608787.1	841	1	0.80	CG13039	NP_648876.1	334	0	0.00
CG8851	NP_722917.2	738	1	0.92	CG13038	NP_652415.2	0	0	#DIV/0!
Sr-CIV	NP_608789.1	32	0	0.00	CG13058	NP_648877.1	0	0	#DIV/0!
CG8852	NP_608790.2	137	0	0.00	CG33061	NP_001097620.1	23	0	0.00
Spindly	NP_608791.2	148	0	0.00	CG33060	NP_788513.2	267	0	0.00
IFT57	NP_608792.1	266	0	0.00	retinin	NP_524995.2	174	2	7.77
drm	NP_001285590.1	113	3	17.94	CG13056	NP_652414.2	0	0	#DIV/0!
CG43815	NP_001259999.1	0	0	#DIV/0!	CG4998	NP_001036609.1	69	0	0.00
sob	NP_476882.1	143	2	9.45	CG33257	NP_996107.1	0	0	#DIV/0!
odd	NP_722922.1	220	2	6.14	mRpS34	NP_524104.2	429	1	1.58
Ugt36A1	NP_524686.3	52	0	0.00	Golgin104	NP_648879.1	133	0	0.00
CG15418	NP_001334671.1	72	0	0.00	Tcs3	NP_648880.1	538	1	1.26
for	NP_477487.1	1561	3	1.30	Pdh	NP_730153.1	91	1	7.43
Sfp24Ba	NP_001162859.1	70	2	19.31	ringer	NP_648881.1	76	0	0.00
Sfp24Bd	NP_001162860.1	11	1	61.45	Cpr72Ea	NP_648882.1	36	0	0.00
Sfp24Bb	NP_001162861.1	31	1	21.80	Cpr72Eb	NP_648883.1	70	0	0.00
Sfp24Bc	NP_001162862.1	0	0	#DIV/0!	Cpr72Ec	NP_648884.1	129	0	0.00
Drgx	NP_001097075.2	112	2	12.07	CG4842	NP_648885.1	40	0	0.00
CG16704	NP_608797.1	133	2	10.16	CG18814	NP_652673.2	256	0	0.00
CG3513	NP_608798.1	40	0	0.00	roq	NP_648886.1	209	0	0.00
Acp24A4	NP_001036330.1	134	2	10.09	Agpat4	NP_648887.1	361	2	3.74
CG43165	NP_001245858.1	19	0	0.00	Agpat3	NP_001246793.1	265	2	5.10
CG16713	NP_608799.1	173	4	15.63	RAF2	NP_730163.1	100	0	0.00
IM33	NP_608800.1	158	3	12.83	CG44836	NP_730167.2	0	0	#DIV/0!

CG3604	NP_608801.1	121	1	5.59	Gagr	NP_648891.1	38	0	0.00
CG10031	NP_608802.2	131	2	10.32	fax	NP_001014587.1	153	0	0.00
CG42464	NP_001162863.1	25	0	0.00	TMS1	NP_648893.1	147	2	9.20
CG42465	NP_001162864.1	0	0	#DIV/0!	GluRS-m	NP_648894.2	693	2	1.95
Sfp24C1	NP_001162865.1	0	0	#DIV/0!	TyrRS	NP_648895.1	1398	4	1.93
CG42467	NP_001162866.1	0	0	#DIV/0!	CG33158	NP_788515.1	1020	2	1.33
CG43145	NP_001245859.1	0	0	#DIV/0!	dsx-c73A	NP_730169.1	64	0	0.00
CG2816	NP_608803.2	18	0	0.00	aos	NP_524107.2	180	0	0.00
CG31778	NP_722932.1	286	0	0.00	CG42514	NP_730171.1	24	0	0.00
CG31777	NP_722933.1	2	0	0.00	st	NP_524108.1	168	0	0.00
cutlet	NP_787969.1	457	3	4.44	CG43373	NP_001097622.2	209	0	0.00
CG31955	NP_001188688.1	288	0	0.00	CG13033	NP_648902.1	5	0	0.00
CG2818	NP_608804.1	115	0	0.00	CG4229	NP_648903.1	38	0	0.00
lectin-24A	NP_652637.1	89	1	7.59	Mipp1	NP_524109.2	228	0	0.00
Shaw	NP_001137782.1	510	4	5.30	CG43206	NP_001246794.1	1	0	0.00
CG10019	NP_001368961.1	43	3	47.16	mbf1	NP_001261952.1	462	1	1.46
CG43707	NP_001260003.1	209	4	12.94	nxf2	NP_001261953.1	333	1	2.03
CG43703	NP_001260009.1	0	0	#DIV/0!	Smn	NP_524112.1	398	0	0.00
bowl	NP_476883.1	114	2	11.86	Rpn12	NP_648904.1	658	2	2.05
CG31960	NP_722942.1	632	2	2.14	UQCR-C2	NP_648905.1	649	3	3.12
bark	NP_001245864.1	14	0	0.00	Syx8	NP_524113.1	241	0	0.00
Reph	NP_722945.2	33	1	20.48	CG32163	NP_730181.1	0	0	#DIV/0!
CG3407	NP_608809.1	125	2	10.81	l(3)73Ah	NP_524933.1	245	2	5.52
slp1	NP_476730.1	402	3	5.04	tra	NP_524114.1	140	1	4.83
slp2	NP_476834.1	358	3	5.66	spd-2	NP_648906.1	305	0	0.00
TTLL4B	NP_722946.1	471	0	0.00	Apl	NP_730182.1	682	5	4.96
Cep97	NP_608811.2	433	0	0.00	Arts	NP_001261957.1	342	2	3.95
CG31957	NP_722949.1	216	0	0.00	CG4098	NP_660192.1	90	1	7.51
capu	NP_722950.2	643	0	0.00	Prosbeta6	NP_524115.1	975	5	3.47
fred	NP_001245866.1	334	1	2.02	Su(P)	NP_524116.2	138	3	14.69
CG31773	NP_722953.1	893	3	2.27	CG4101	NP_648908.1	22	0	0.00
CG43797	NP_001260012.1	24	0	0.00	Mo25	NP_524117.1	477	2	2.83
CG34176	NP_001097076.1	11	1	61.45	Abl	NP_001261965.1	850	2	1.59
CG15422	NP_608814.1	40	2	33.80	Baldspot	NP_648909.1	247	2	5.47
CG15423	NP_608815.1	168	1	4.02	Galphaf	NP_524118.1	309	0	0.00
CG43773	NP_001137789.1	12	2	112.65	zetaCOP	NP_648910.1	387	2	3.49
CG43774	NP_608816.2	1	0	0.00	CG13032	NP_648911.1	374	0	0.00
ed	NP_523469.2	212	2	6.38	CG43954	NP_001261966.1	0	0	#DIV/0!
Sr-CIII	NP_524747.1	214	1	3.16	CG9692	NP_788516.1	5	0	0.00
Sr-CI	NP_477102.1	245	1	2.76	Dab	NP_001261967.1	324	3	6.26
CG2955	NP_608817.1	954	2	1.42	CG13031	NP_648914.1	0	0	#DIV/0!
Or24a	NP_523470.3	96	0	0.00	CG13026	NP_648915.2	15	3	135.18
TBCC	NP_722959.1	136	0	0.00	PGRP-SB2	NP_001261970.1	93	3	21.80
lectin-24Db	NP_652641.1	464	2	2.91	PGRP-SB1	NP_648917.1	197	3	10.29
Naprt	NP_001260015.1	279	0	0.00	Dbp73D	NP_476833.1	900	5	3.76
ft	NP_477497.1	175	1	3.86	CG9701	NP_648918.1	331	0	0.00
CG3702	NP_608819.1	123	1	5.50	CG42852	NP_001189120.1	0	0	#DIV/0!
RpL40	NP_476776.1	1722	3	1.18	Nrt	NP_476798.1	197	1	3.43
CG15425	NP_608820.1	425	1	1.59	CG13025	NP_648919.1	126	1	5.36
Art2	NP_608821.1	654	0	0.00	CG9705	NP_648920.1	554	1	1.22
tutl	NP_001260019.1	194	3	10.45	CG9706	NP_001356982.1	379	0	0.00
bdl	NP_608822.1	46	1	14.69	eIF3e	NP_477385.1	698	1	0.97
Atet	NP_001097079.1	166	2	8.14	CG9674	NP_648922.1	667	1	1.01
CG15429	NP_608823.2	632	4	4.28	nudC	NP_648923.1	520	2	2.60
Traf4	NP_001097080.2	408	1	1.66	CG13024	NP_001246806.1	11	0	0.00
CG17612	NP_608824.1	73	1	9.26	TSG101	NP_524120.1	507	1	1.33
Vps53	NP_608825.2	261	0	0.00	kud	NP_648925.1	210	0	0.00
Psf2	NP_608826.4	297	2	4.55	Zcchc7	NP_648926.3	531	0	0.00
Tps1	NP_608827.1	444	2	3.04	CG32161	NP_730205.1	574	1	1.18
CG3652	NP_608828.2	149	0	0.00	Rh4	NP_476701.1	466	5	7.25
l(2)k05819	NP_722974.1	235	0	0.00	sina	NP_476725.1	333	1	2.03
CG33003	NP_001137790.1	10	0	0.00	sinah	NP_648927.1	1034	3	1.96
Dim1	NP_608830.3	618	0	0.00	CG13029	NP_648928.1	52	3	38.99
CG15431	NP_608831.1	0	0	#DIV/0!	CG9951	NP_648929.1	150	0	0.00
CG43055	NP_001245867.1	81	0	0.00	Lmpt	NP_648930.1	249	2	5.43
CG34177	NP_001097081.1	0	0	#DIV/0!	CG32170	NP_730211.1	0	0	#DIV/0!

CG43056	NP_001245868.1	0	0	#DIV/0!	CG13028	NP_996114.1	0	0	#DIV/0!
Sfp24F	NP_001162870.1	61	0	0.00	CG13023	NP_648935.1	4	0	0.00
CG15432	NP_608832.1	46	0	0.00	CG11905	NP_001097625.1	56	0	0.00
morgue	NP_608833.1	688	0	0.00	CG13022	NP_648937.2	6	0	0.00
Elp3	NP_608834.1	777	2	1.74	Cpr73D	NP_001097627.1	30	0	0.00
MFS18	NP_608835.1	47	0	0.00	Obp73a	NP_001334711.1	13	1	51.99
CG15439	NP_608836.1	410	1	1.65	Nc73EF	NP_788520.2	473	0	0.00
Secp43	NP_608837.2	671	2	2.01	Exn	NP_001189123.1	399	0	0.00
ND-B8	NP_652341.1	362	1	1.87	CG3764	NP_001261977.1	73	0	0.00
Gs1l	NP_477229.3	123	2	10.99	CG6664	NP_648944.1	111	0	0.00
RpL27A	NP_476963.1	996	3	2.04	CG7728	NP_648945.1	813	0	0.00
mRpL27	NP_787971.1	828	4	3.27	CG6652	NP_648946.3	642	0	0.00
CG15435	NP_608838.1	26	1	26.00	a10	NP_524121.2	83	1	8.14
CG15443	NP_608839.1	4	0	0.00	Fit2	NP_648947.1	205	0	0.00
CG15436	NP_608840.1	134	0	0.00	CG7730	NP_788521.2	32	0	0.00
FIG4	NP_608841.1	285	0	0.00	Ilp8	NP_648949.2	58	0	0.00
ine	NP_477012.1	149	0	0.00	beg	NP_648950.1	690	2	1.96
dpy	NP_001245879.1	795	1	0.85	scaf6	NP_001014589.1	699	0	0.00
HP6	NP_608842.1	362	1	1.87	Papst2	NP_648954.1	83	0	0.00
BG642163	NP_001036331.1	25	1	27.04	Pop5	NP_648955.1	440	0	0.00
CG15635	NP_608846.2	25	1	27.04	TpnC73F	NP_524122.2	448	0	0.00
CG43798	NP_001260043.1	0	0	#DIV/0!	rogdi	NP_730244.1	62	0	0.00
CG3355	NP_608848.1	136	0	0.00	CG7724	NP_648957.1	481	1	1.41
CG11929	NP_608849.1	456	3	4.45	Rbp6	NP_001261979.1	360	0	0.00
Bsg25A	NP_523472.2	80	2	16.90	CG6512	NP_730248.2	687	1	0.98
Elba3	NP_608850.1	63	2	21.46	CG7707	NP_648962.1	826	3	2.45
CG3251	NP_608851.1	183	0	0.00	CG6497	NP_648963.1	636	3	3.19
Taf12L	NP_608852.1	270	0	0.00	CG13723	NP_648964.1	1	0	0.00
CG34351	NP_001260044.1	17	0	0.00	ND-24L	NP_648965.1	858	3	2.36
CG15631	NP_608855.2	795	2	1.70	CG13724	NP_648966.1	0	0	#DIV/0!
fipi	NP_787975.1	71	0	0.00	CG13725	NP_648967.1	857	3	2.37
CG42523	NP_001162872.1	451	1	1.50	Or74a	NP_524123.1	64	0	0.00
CG3294	NP_608857.2	289	0	0.00	CG6479	NP_001261981.1	211	0	0.00
slf	NP_608858.3	163	0	0.00	CG13727	NP_648969.1	0	0	#DIV/0!
CG15629	NP_608859.1	93	0	0.00	brv2	NP_001261982.1	99	0	0.00
CG3225	NP_608860.1	682	1	0.99	Mip	NP_648971.1	112	0	0.00
CG15628	NP_001260047.1	2	0	0.00	CG7692	NP_648972.2	16	0	0.00
Fnta	NP_608862.1	295	0	0.00	tap	NP_524124.1	51	0	0.00
Ir25a	NP_608863.2	159	0	0.00	Cad74A	NP_648973.1	105	0	0.00
tank	NP_608864.1	54	0	0.00	CG13728	NP_648974.3	0	0	#DIV/0!
CG12194	NP_001162873.1	65	0	0.00	CG13731	NP_730262.3	33	0	0.00
mxt	NP_001260054.1	61	1	11.08	blot	NP_524125.2	118	0	0.00
CG11927	NP_608867.1	14	3	144.84	Sec3	NP_648976.2	221	0	0.00
mRpS2	NP_523473.2	957	4	2.83	CG7630	NP_652413.1	137	2	9.87
Mon1	NP_608868.1	310	3	6.54	CG33051	NP_788522.1	157	1	4.31
smog	NP_001245881.1	71	3	28.56	Gorab	NP_788523.1	173	0	0.00
Cf2	NP_001350854.1	171	0	0.00	frc	NP_648977.3	240	0	0.00
CG3008	NP_608871.1	415	1	1.63	CG32176	NP_730271.1	35	0	0.00
CG15625	NP_608872.1	33	0	0.00	CG12229	NP_648980.1	1257	2	1.08
CG3036	NP_608873.1	68	0	0.00	CG18265	NP_648982.1	17	0	0.00
snsl	NP_001097086.1	5	0	0.00	QIL1	NP_648983.1	116	2	11.65
hoe1	NP_608876.1	77	0	0.00	SecCl	NP_001261986.1	55	0	0.00
hoe2	NP_608878.1	49	0	0.00	UQCR-Q	NP_648985.1	476	3	4.26
Sgs1	NP_523475.3	134	2	10.09	CG34250	NP_001287094.1	0	0	#DIV/0!
CG14044	NP_608879.1	53	0	0.00	CG13733	NP_648986.1	773	2	1.75
mRpL24	NP_523476.1	938	1	0.72	qjt	NP_648987.1	194	0	0.00
betaggt-I	NP_001260061.1	162	0	0.00	CG6333	NP_730277.1	41	1	16.49
jet	NP_001036332.1	550	0	0.00	edin	NP_730278.1	120	0	0.00
Jon25Biii	NP_608881.1	333	3	6.09	Oatp74D	NP_648989.1	62	0	0.00
Jon25Bii	NP_608882.1	326	3	6.22	U4-U6-60K	NP_648990.1	673	0	0.00
Jon25Bi	NP_001033873.1	291	2	4.65	CG7564	NP_648991.1	468	2	2.89
Marcal1	NP_608883.1	666	1	1.01	Edc3	NP_648992.1	501	0	0.00
CG34124	NP_001036333.1	427	0	0.00	Nedd4	NP_001261990.1	801	2	1.69
CG34125	NP_001036334.1	4	0	0.00	Jon74E	NP_001189126.2	247	1	2.74
eIF3i	NP_523478.1	798	3	2.54	CG7542	NP_648995.3	172	1	3.93
Scox	NP_608884.1	428	0	0.00	CycT	NP_524127.2	501	2	2.70

CG3756	NP_608885.1	1178	3	1.72	Eip74EF	NP_730286.3	206	1	3.28
l(2)05714	NP_001260062.1	39	0	0.00	Vps60	NP_648997.1	268	0	0.00
CG14043	NP_608886.1	54	0	0.00	anchor	NP_001261995.1	28	0	0.00
mRpL28	NP_608887.1	300	2	4.51	CG7497	NP_648999.2	146	0	0.00
Gmd	NP_608888.2	258	0	0.00	Pep	NP_524858.1	171	1	3.95
CG3792	NP_608889.1	110	0	0.00	CG43085	NP_001246818.1	0	0	#DIV/0!
CG8891	NP_608890.1	727	2	1.86	Ndfip	NP_730291.1	157	1	4.31
CG8892	NP_608891.1	242	0	0.00	Krn	NP_524129.1	67	0	0.00
CG34126	NP_001260066.1	152	0	0.00	CG7484	NP_649000.1	174	0	0.00
Rtnl1	NP_787983.1	481	0	0.00	Crtc	NP_001261997.1	165	0	0.00
CG31917	NP_608895.1	447	2	3.02	CG34251	NP_001097634.1	0	0	#DIV/0!
SelT	NP_608897.1	179	0	0.00	Ccn	NP_730294.1	189	0	0.00
vkg	NP_477190.1	350	0	0.00	CheB74a	NP_001027134.1	19	0	0.00
Col4a1	NP_723044.1	849	0	0.00	CG6052	NP_649002.3	30	0	0.00
CG14042	NP_001036336.1	9	0	0.00	CG7460	NP_649004.1	212	0	0.00
CG31650	NP_001260075.1	207	1	3.27	CG6034	NP_649005.1	211	0	0.00
CG33995	NP_001033875.1	588	1	1.15	Adgf-B	NP_649006.2	848	1	0.80
CG9121	NP_608901.1	559	1	1.21	Adgf-A	NP_524130.1	376	2	3.60
eIF3h	NP_524834.2	632	3	3.21	Adgf-A2	NP_525020.2	608	3	3.34
senju	NP_608902.1	124	0	0.00	CG32182	NP_730299.1	0	0	#DIV/0!
CG14036	NP_608903.1	187	0	0.00	CG32181	NP_730300.2	38	1	17.79
His3.3A	NP_523479.1	820	1	0.82	CG42815	NP_001189127.1	38	0	0.00
Nepl3	NP_608904.1	35	0	0.00	CG42816	NP_730301.3	39	0	0.00
Rpn11	NP_608905.1	1104	5	3.06	NUCB1	NP_649008.2	188	0	0.00
qtc	NP_523480.2	45	0	0.00	CG5589	NP_649009.1	811	3	2.50
RpL37A	NP_524781.1	744	2	1.82	CG32187	NP_730303.1	64	1	10.56
Pgant5	NP_001036338.1	144	0	0.00	CG32191	NP_730304.2	220	1	3.07
CG5828	NP_608907.1	106	0	0.00	Cln3	NP_649011.1	173	2	7.81
CG4230	NP_001260087.1	2	0	0.00	Ir75a	NP_649012.2	79	0	0.00
ND-13A	NP_608909.1	490	0	0.00	Ir75b	NP_001137966.2	95	0	0.00
Msp300	NP_001260088.1	474	0	0.00	Ir75c	NP_649013.3	113	0	0.00
Cyp28d2	NP_608911.1	113	3	17.94	Grd	NP_524131.1	122	1	5.54
Cyp28d1	NP_608912.1	93	2	14.54	CG32188	NP_730308.1	0	0	#DIV/0!
CG7742	NP_608913.1	571	0	0.00	CG32189	NP_730309.1	0	0	#DIV/0!
CG14034	NP_001036339.1	27	0	0.00	CG5577	NP_649014.1	89	0	0.00
TpnC25D	NP_608915.2	384	0	0.00	CG5567	NP_649015.2	135	1	5.01
tkv	NP_787989.1	701	1	0.96	MED19	NP_649016.1	102	0	0.00
Cyp4ac1	NP_608916.1	140	1	4.83	CG7430	NP_649017.1	902	1	0.75
Cyp4ac2	NP_608917.3	140	2	9.66	TrpRS-m	NP_649018.2	561	1	1.20
Cyp4ac3	NP_608918.1	145	2	9.32	CG5535	NP_649019.2	136	3	14.91
Bsg25D	NP_001260092.1	136	0	0.00	CG34002	NP_001034029.2	23	0	0.00
Bub1	NP_608919.1	764	0	0.00	CG7408	NP_649020.1	261	1	2.59
vri	NP_477191.1	259	1	2.61	CG5506	NP_649021.1	139	1	4.86
CG14024	NP_608920.1	82	0	0.00	CG16775	NP_649022.2	51	1	13.25
Ncoa6	NP_001097088.3	191	0	0.00	CG7402	NP_649023.1	237	1	2.85
cype	NP_536787.1	327	0	0.00	Tsp74F	NP_524132.1	26	2	51.99
CG14022	NP_608922.1	28	0	0.00	Prestin	NP_649024.1	212	3	9.56
TotM	NP_523482.1	136	0	0.00	Wdr92	NP_649025.1	74	2	18.27
fusl	NP_608923.1	33	1	20.48	CG5290	NP_649026.1	572	0	0.00
CG12512	NP_608924.1	252	0	0.00	Eip75B	NP_730321.1	286	0	0.00
nompC	NP_001303309.1	702	0	0.00	CG32192	NP_730325.1	295	0	0.00
H15	NP_608926.2	177	2	7.64	CG42393	NP_001137967.1	0	0	#DIV/0!
mid	NP_608927.2	271	2	4.99	CG44005	NP_001262003.1	62	0	0.00
CG14020	NP_001260097.1	85	2	15.90	CG34253	NP_001097636.1	8	0	0.00
CG7382	NP_608929.1	158	0	0.00	CG13698	NP_649027.1	0	0	#DIV/0!
cl	NP_608930.1	126	0	0.00	mRpS26	NP_524134.1	335	0	0.00
Vps52	NP_608931.1	374	1	1.81	RpIIIC53	NP_649028.3	535	0	0.00
CG6907	NP_608932.1	108	0	0.00	mus304	NP_524135.3	113	0	0.00
Cap-D3	NP_608935.2	254	2	5.32	CG32195	NP_730328.1	82	2	16.49
CG31648	NP_723086.1	288	0	0.00	CG7341	NP_649029.1	22	1	30.72
CG31915	NP_723087.1	279	0	0.00	CG42853	NP_001189128.1	0	0	#DIV/0!
Coq6	NP_608934.1	487	1	1.39	Cyp312a1	NP_649030.1	559	2	2.42
tomb	NP_608936.1	242	1	2.79	geko	NP_524980.1	74	0	0.00
CG14015	NP_608937.1	67	0	0.00	CG7330	NP_649032.1	30	0	0.00
Hel25E	NP_723089.1	1684	1	0.40	CG13699	NP_649033.1	2	0	0.00
Lam	NP_476616.1	762	2	1.77	hid	NP_524136.2	160	4	16.90

Oscillin	NP_001245895.1	418	1	1.62	CG7320	NP_649034.3	34	0	0.00
CG14014	NP_608939.2	14	1	48.28	CheA75a	NP_649035.2	79	1	8.56
CG18269	NP_608940.1	8	1	84.49	CG5103	NP_649036.1	1293	3	1.57
CG14013	NP_608941.1	766	4	3.53	CG13700	NP_001262012.1	820	4	3.30
CG14017	NP_608942.1	207	2	6.53	grim	NP_524137.2	160	5	21.12
GluRIIA	NP_523484.2	428	0	0.00	rpr	NP_524138.1	339	3	5.98
GluRIIB	NP_523485.3	362	0	0.00	skl	NP_524139.1	148	3	13.70
CG14011	NP_608943.1	373	2	3.62	CG32196	NP_001262014.1	70	0	0.00
CG7251	NP_608944.1	825	3	2.46	CG4306	NP_649038.1	120	0	0.00
CG18266	NP_608945.1	759	3	2.67	Met75Cb	NP_651980.1	50	0	0.00
DIP-eta	NP_608946.3	40	0	0.00	Met75Ca	NP_730336.1	2	0	0.00
DIP-theta	NP_723103.1	82	0	0.00	AstC-R2	NP_001303398.1	172	2	7.86
Hsp60C	NP_608948.2	1564	4	1.73	CG32198	NP_730339.1	36	1	18.78
CG12511	NP_608949.2	104	0	0.00	AstC-R1	NP_649040.2	153	0	0.00
CG7236	NP_001285635.1	124	0	0.00	CG7271	NP_649041.1	49	0	0.00
CG14007	NP_001097090.2	0	0	#DIV/0!	term	NP_524882.2	19	0	0.00
CG34011	NP_001033878.3	14	0	0.00	CG13380	NP_730342.2	0	0	#DIV/0!
CG11149	NP_001162889.1	47	0	0.00	CG18234	NP_649043.3	31	0	0.00
sip1	NP_524725.2	266	0	0.00	CG18233	NP_649044.3	47	0	0.00
CG11030	NP_608956.1	605	0	0.00	CG18231	NP_649045.2	63	3	32.19
CG14006	NP_608953.1	110	0	0.00	CG32201	NP_730346.2	41	0	0.00
CG11147	NP_608954.1	76	0	0.00	CG32199	NP_730347.1	131	0	0.00
CG11029	NP_608955.1	41	0	0.00	not	NP_524140.2	487	2	2.78
obst-E	NP_723116.1	156	0	0.00	bora	NP_649048.1	318	0	0.00
CG9171	NP_723117.1	14	0	0.00	MYPT-75D	NP_649049.1	345	0	0.00
CG31913	NP_723119.1	318	0	0.00	CG42495	NP_001163464.1	0	0	#DIV/0!
CG14005	NP_001260107.1	9	0	0.00	Capr	NP_652671.1	339	0	0.00
CG7239	NP_608960.2	36	0	0.00	GNBP2	NP_524141.1	86	1	7.86
CG11034	NP_608961.2	162	0	0.00	GNBP1	NP_524142.2	114	1	5.93
CG43307	NP_001245900.1	0	0	#DIV/0!	Sgf11	NP_001097638.1	195	2	6.93
CG31644	NP_001285639.1	672	0	0.00	CG32202	NP_730354.2	315	0	0.00
TrissinR	NP_001097092.1	99	0	0.00	Chmp1	NP_649051.3	270	0	0.00
rau	NP_608965.1	125	0	0.00	Cyp12c1	NP_649052.1	107	0	0.00
Sfp26Ac	NP_001162892.1	0	0	#DIV/0!	CG6893	NP_001369058.1	444	0	0.00
CG43185	NP_001245901.2	0	0	#DIV/0!	Dic4	NP_649054.1	143	0	0.00
CG9029	NP_001285645.1	155	2	8.72	CG12477	NP_649055.1	190	0	0.00
Acp26Ab	NP_476645.1	155	1	4.36	CG6885	NP_649056.1	27	1	25.03
Acp26Aa	NP_476644.1	418	2	3.23	MED11	NP_649057.1	123	1	5.50
CG9021	NP_608967.1	24	0	0.00	Nufip	NP_649058.1	190	0	0.00
bchs	NP_608968.2	687	0	0.00	Atg3	NP_649059.1	371	0	0.00
CG14000	NP_608969.1	11	0	0.00	ftz-f1	NP_524143.2	301	0	0.00
CG9016	NP_608970.2	477	0	0.00	CG14073	NP_649060.1	349	0	0.00
dsf	NP_477140.1	260	0	0.00	HipHop	NP_649061.1	173	0	0.00
Sfp26Ad	NP_001162893.1	11	1	61.45	Cat	NP_536731.1	1281	3	1.58
Gpdh1	NP_476566.1	699	0	0.00	Indy	NP_730363.2	202	1	3.35
CG9044	NP_001260114.1	103	0	0.00	CG6865	NP_001246825.1	70	0	0.00
CG13999	NP_608972.1	41	0	0.00	CG14074	NP_001246826.1	39	0	0.00
CG13998	NP_652551.1	47	1	14.38	Dysb	NP_649064.1	266	1	2.54
Vm26Ab	NP_476784.1	140	2	9.66	Grx1	NP_649065.1	376	1	1.80
Vm26Ac	NP_608973.1	132	2	10.24	Blos3	NP_001097640.1	15	1	45.06
Vm26Aa	NP_476783.1	116	3	17.48	Rad9	NP_649066.1	316	0	0.00
psd	NP_001260116.1	21	4	128.74	Uggt	NP_524151.2	366	0	0.00
CG13992	NP_608975.1	148	5	22.83	CG3961	NP_730369.1	521	3	3.89
Ucp4C	NP_608976.1	573	1	1.18	NijB	NP_649068.1	6	0	0.00
Ucp4B	NP_608977.1	105	2	12.87	CG3902	NP_649069.2	302	2	4.48
chic	NP_477016.1	709	2	1.91	RpL26	NP_649070.1	969	1	0.70
eIF4A	NP_476595.1	1185	2	1.14	arx	NP_649071.1	181	0	0.00
ifc	NP_476594.1	264	2	5.12	CG6843	NP_649072.1	218	2	6.20
lid	NP_523486.1	1273	0	0.00	CSN1b	NP_524152.2	288	2	4.69
Tsp26A	NP_001097093.1	198	1	3.41	CG6841	NP_649073.1	719	2	1.88
Gal	NP_001260120.1	324	1	2.09	Ir75d	NP_649074.2	96	0	0.00
CG9098	NP_608979.2	97	0	0.00	CG14077	NP_649075.1	633	0	0.00
H2.0	NP_523488.2	146	0	0.00	CG6839	NP_649076.1	210	2	6.44
CG13996	NP_608980.1	0	0	#DIV/0!	CG18223	NP_649077.1	141	0	0.00
CG9107	NP_608981.1	543	0	0.00	CG3819	NP_649078.1	207	1	3.27
CG9109	NP_608982.2	167	0	0.00	CG32204	NP_001369059.1	0	0	#DIV/0!

mtm	NP_523489.1	198	1	3.41	CG43049	NP_001246829.1	0	0	#DIV/0!
CG9117	NP_608983.2	69	1	9.80	CG6836	NP_649079.2	20	0	0.00
CG31643	NP_723145.1	78	1	8.67	CG14075	NP_649080.1	30	0	0.00
Pfas	NP_477212.1	535	1	1.26	CG11619	NP_649081.1	107	0	0.00
CG34179	NP_001097094.1	1	0	0.00	CG34256	NP_001097642.2	0	0	#DIV/0!
IPIP	NP_608984.2	101	0	0.00	CG18135	NP_788524.1	170	2	7.95
CG9135	NP_608985.1	556	1	1.22	CG3808	NP_649083.2	689	0	0.00
CG13995	NP_608986.1	98	0	0.00	SmydA-2	NP_649084.1	259	0	0.00
CG34180	NP_001097096.1	0	0	#DIV/0!	nkd	NP_524788.2	208	2	6.50
ND-51	NP_608987.1	457	0	0.00	Acp76A	NP_001246831.1	229	0	0.00
CG13994	NP_608988.1	190	1	3.56	CG43407	NP_001246832.1	0	0	#DIV/0!
Fbw5	NP_608989.1	273	0	0.00	Ugt316A1	NP_649085.1	45	0	0.00
CG9147	NP_608990.1	0	0	#DIV/0!	Sfxn2	NP_649086.2	139	1	4.86
CG9150	NP_608991.2	58	1	11.65	Mkp3	NP_001262031.1	374	2	3.61
Pfdn1	NP_608992.1	413	3	4.91	MESR6	NP_001262033.1	63	0	0.00
CG9154	NP_608993.2	75	0	0.00	CNPYb	NP_649089.2	146	2	9.26
Kr-h2	NP_523490.1	189	0	0.00	Naxd	NP_649090.1	310	1	2.18
CG9175	NP_608995.2	225	0	0.00	CG14079	NP_649091.1	35	0	0.00
stai	NP_723156.1	298	0	0.00	Gem2	NP_649092.1	199	0	0.00
CG31642	NP_723159.1	209	0	0.00	CG14082	NP_001262035.1	0	0	#DIV/0!
Arpc4	NP_608996.1	395	0	0.00	Spn75F	NP_001036614.1	132	0	0.00
WDR79	NP_001285665.1	423	0	0.00	fz2	NP_001262037.1	277	0	0.00
B9d2	NP_608998.2	110	0	0.00	CG33647	NP_001027136.1	0	0	#DIV/0!
CG9222	NP_608999.2	1274	3	1.59	mRpL21	NP_649095.2	797	0	0.00
CG13991	NP_609000.2	408	2	3.31	rept	NP_524156.1	1171	0	0.00
Muc26B	NP_652552.1	67	0	0.00	nes	NP_524157.2	185	0	0.00
CG31639	NP_723162.1	638	2	2.12	Bet1	NP_649096.1	287	0	0.00
CG13989	NP_609001.2	662	3	3.06	Max	NP_649097.1	138	0	0.00
smal	NP_723165.2	108	0	0.00	CG42374	NP_001137972.1	20	0	0.00
Ugt37B1	NP_525008.2	80	0	0.00	Aldh7A1	NP_649099.1	323	0	0.00
AANATL2	NP_609005.1	40	1	16.90	CG14085	NP_649101.2	5	0	0.00
CG13983	NP_609006.1	51	1	13.25	CG14088	NP_649100.2	3	0	0.00
Ddr	NP_001285666.1	92	1	7.35	CG14086	NP_649102.3	0	0	#DIV/0!
CG43922	NP_001260125.1	0	0	#DIV/0!	CG14089	NP_649103.1	1	0	0.00
CG13984	NP_609010.1	3	0	0.00	Gbs-76A	NP_649104.1	128	0	0.00
PDZ-GEF	NP_001137797.1	243	1	2.78	fal	NP_001137974.1	137	0	0.00
Pez	NP_609013.1	261	1	2.59	pip	NP_524158.2	94	0	0.00
Cpr	NP_477158.1	587	0	0.00	CG32206	NP_788538.2	25	1	27.04
CG9497	NP_609014.3	82	5	41.21	ms(3)76Ba	NP_649109.3	22	1	30.72
CG9498	NP_609015.2	78	2	17.33	CG33062	NP_788539.1	0	0	#DIV/0!
ppk7	NP_609016.2	73	2	18.52	Chd3	NP_649111.1	823	0	0.00
ppk14	NP_609017.2	64	2	21.12	CG14095	NP_649112.1	32	0	0.00
CG9500	NP_609018.3	183	1	3.69	CG14096	NP_649113.1	34	0	0.00
Nepl4	NP_609019.1	34	0	0.00	CG32214	NP_730409.2	8	0	0.00
slam	NP_609020.3	57	0	0.00	825-Oak	NP_730410.2	8	0	0.00
DIP-epsilon	NP_001137799.1	10	0	0.00	CG12519	NP_001262046.1	57	2	23.72
Nepl5	NP_609022.1	45	2	30.04	CG18294	NP_649115.2	10	1	67.59
Nepl6	NP_609023.3	12	0	0.00	CG32213	NP_730413.2	7	0	0.00
CG42369	NP_609024.1	10	0	0.00	brv1	NP_649116.1	103	0	0.00
CG13982	NP_609025.1	4	0	0.00	CG32212	NP_730416.1	9	1	75.10
Tig	NP_477033.1	188	1	3.60	CG9449	NP_001137976.1	75	2	18.02
Fic	NP_609026.1	96	1	7.04	CG9451	NP_649118.1	74	1	9.13
CG9527	NP_001260131.1	272	1	2.48	CG9452	NP_649119.2	75	1	9.01
retm	NP_001260132.1	216	1	3.13	Cpr76Ba	NP_649120.1	8	0	0.00
frj	NP_609029.1	123	1	5.50	Cpr76Bb	NP_649121.1	6	0	0.00
Rchy1	NP_609030.1	443	1	1.53	Cpr76Bc	NP_001097644.1	25	0	0.00
HemK1	NP_609031.2	186	0	0.00	Cpr76Bd	NP_001262052.1	151	4	17.90
mmy	NP_609032.1	439	2	3.08	Ltn1	NP_730427.1	443	4	6.10
CG9536	NP_609033.1	98	2	13.79	CG9300	NP_649127.1	277	4	9.76
Sec61alpha	NP_609034.1	1063	4	2.54	Shal	NP_001262055.1	564	4	4.79
Daxx	NP_609035.2	423	0	0.00	CG9231	NP_649128.1	69	4	39.18
KFase	NP_609036.1	258	4	10.48	CG9330	NP_649129.1	655	0	0.00
epsilonCOP	NP_609037.1	473	3	4.29	CG14100	NP_649130.2	480	1	1.41
Phf5a	NP_609038.1	807	0	0.00	ash1	NP_524160.2	605	1	1.12
CG31638	NP_001097099.1	55	0	0.00	Taf6	NP_524161.2	327	0	0.00
CG9547	NP_609040.1	202	2	6.69	CG9368	NP_730432.1	5	0	0.00

CG9550	NP_609042.1	392	1	1.72	lush	NP_524162.1	147	1	4.60
CG31637	NP_609043.1	11	2	122.89	CG9372	NP_649132.1	181	0	0.00
eya	NP_523492.1	453	1	1.49	Lon	NP_649133.1	726	1	0.93
IFT52	NP_609045.2	185	1	3.65	PIG-F	NP_649134.3	69	0	0.00
COX5B	NP_609046.1	513	1	1.32	l(3)76BDm	NP_649135.2	360	0	0.00
COX5BL	NP_609047.1	836	2	1.62	asf1	NP_524163.1	1132	1	0.60
CG9596	NP_995638.1	639	1	1.06	ms(3)76Cc	NP_649136.1	480	0	0.00
Ent2	NP_609049.1	138	1	4.90	wnd	NP_649137.3	310	0	0.00
CG13766	NP_609050.1	73	1	9.26	Rnf146	NP_649138.2	188	0	0.00
CG11319	NP_609051.1	142	0	0.00	Oat	NP_649139.1	606	0	0.00
CG11050	NP_609052.1	133	0	0.00	tey	NP_001262059.1	121	2	11.17
DIP-iota	NP_001097100.1	21	0	0.00	CG8765	NP_001262061.1	38	0	0.00
CG34345	NP_001097101.1	77	0	0.00	Gbeta76C	NP_523720.2	503	0	0.00
Oatp26F	NP_609055.2	74	0	0.00	verm	NP_730442.2	183	1	3.69
CG31635	NP_001097102.1	58	0	0.00	serp	NP_730444.1	210	4	12.87
Tango1	NP_609058.2	132	0	0.00	Trpml	NP_649145.1	128	2	10.56
CG31633	NP_723199.1	87	1	7.77	CG42637	NP_524165.2	194	2	6.97
CG31636	NP_723200.1	36	0	0.00	CG42529	NP_001163471.1	34	1	19.88
CG11070	NP_609060.1	544	0	0.00	CG42638	NP_001163472.1	127	1	5.32
CG13771	NP_609061.2	101	0	0.00	Gyc76C	NP_001163473.1	195	3	10.40
CG34310	NP_523493.3	0	0	#DIV/0!	ms(3)76Ca	NP_649146.1	776	1	0.87
Galt	NP_609062.2	220	1	3.07	SREBP	NP_730449.1	607	1	1.11
Spn27A	NP_652024.1	215	1	3.14	Ir76a	NP_001097647.3	95	0	0.00
cort	NP_523494.1	791	2	1.71	CG14102	NP_649147.2	1	0	0.00
Nse1	NP_609063.1	339	1	1.99	TORIP	NP_649149.1	10	1	67.59
Nhe3	NP_001097105.3	402	0	0.00	CG34116	NP_001036616.1	5	1	135.18
CG11327	NP_609065.1	476	3	4.26	Ccdc58	NP_730454.2	370	0	0.00
Tsp	NP_001137802.1	183	3	11.08	CG14104	NP_652408.2	46	0	0.00
AkhR	NP_995639.1	423	0	0.00	Kap-alpha1	NP_524167.1	585	0	0.00
Aatf	NP_609066.1	537	1	1.26	Cyp305a1	NP_649151.1	139	0	0.00
homer	NP_001036341.2	230	0	0.00	cyc	NP_524168.2	392	1	1.72
Liprin-alpha	NP_001260155.1	316	0	0.00	Fibp	NP_996123.3	94	0	0.00
TTLL3B	NP_609068.2	560	3	3.62	Deaf1	NP_524169.1	181	0	0.00
TTLL3A	NP_609069.1	151	0	0.00	trc	NP_524170.2	963	1	0.70
meng	NP_609070.1	39	0	0.00	CG32221	NP_730456.2	67	0	0.00
CG31910	NP_723211.1	612	4	4.42	kto	NP_524786.1	391	2	3.46
CG11322	NP_609071.2	86	0	0.00	Papss	NP_730457.1	485	0	0.00
LUBEL	NP_001260160.1	156	0	0.00	Rab8	NP_524172.1	640	2	2.11
CG17378	NP_609073.2	38	0	0.00	Usp32	NP_001262074.2	572	1	1.18
nrv1	NP_477167.1	215	0	0.00	Mi-2	NP_649154.2	807	3	2.51
nrv2	NP_477168.1	355	1	1.90	Su(Tpl)	NP_001163477.1	276	1	2.45
CG17376	NP_652707.1	713	2	1.90	Prp3	NP_649156.1	639	2	2.12
CG17377	NP_723219.2	796	2	1.70	Mtr3	NP_649157.1	679	0	0.00
CG11236	NP_609075.1	319	0	0.00	Rpn1	NP_649158.1	818	2	1.65
CG17375	NP_609076.3	579	1	1.17	Su(z)12	NP_730465.1	365	1	1.85
sens-2	NP_723223.1	146	0	0.00	Pfdn6	NP_649159.1	561	1	1.20
Rca1	NP_477214.1	259	1	2.61	Grasp65	NP_649160.1	388	2	3.48
l(2)k09022	NP_609079.2	754	4	3.59	CG8004	NP_649161.1	94	0	0.00
Nha1	NP_723224.2	221	1	3.06	RhoGDI	NP_649162.1	385	0	0.00
Nlg2	NP_523496.1	192	1	3.52	Ac76E	NP_001246838.1	484	0	0.00
CG13773	NP_609081.1	727	4	3.72	CG32219	NP_730469.1	10	0	0.00
Rat1	NP_609082.1	870	4	3.11	CG34258	NP_001097648.1	0	0	#DIV/0!
Wee1	NP_477035.1	590	1	1.15	CG33710	NP_001027137.1	0	0	#DIV/0!
x16	NP_001260168.1	556	5	6.08	CG42655	NP_001189133.2	0	0	#DIV/0!
CG32829	NP_723227.1	0	0	#DIV/0!	CG42654	NP_001189134.2	0	0	#DIV/0!
nop5	NP_477412.1	1188	8	4.55	CG42263	NP_001137983.2	0	0	#DIV/0!
Hrb27C	NP_476869.1	571	3	3.55	CG14182	NP_649163.1	16	0	0.00
CG43232	NP_001245920.1	0	0	#DIV/0!	CG17732	NP_001189135.1	40	0	0.00
CG18304	NP_001260169.1	57	0	0.00	hale	NP_649165.1	620	1	1.09
Fgop2	NP_609084.1	84	1	8.05	schuy	NP_649166.1	897	2	1.51
ihog	NP_609085.1	108	1	6.26	Csas	NP_730474.2	121	0	0.00
Gas41	NP_609086.1	563	2	2.40	fln	NP_524174.1	178	0	0.00
SA	NP_477268.2	353	1	1.91	CG7646	NP_649167.1	319	1	2.12
CG13775	NP_609087.1	72	0	0.00	Nca	NP_788543.1	406	1	1.66
CG3430	NP_542438.1	568	2	2.38	Gabat	NP_649168.2	404	1	1.67
Atac1	NP_609088.1	553	4	4.89	Tom20	NP_649169.3	624	1	1.08

Hmgcl	NP_609089.1	612	1	1.10	CG7668	NP_649170.1	101	0	0.00
sip2	NP_723234.2	57	0	0.00	kug	NP_001027138.2	378	0	0.00
Coprox	NP_524777.1	445	1	1.52	CG14183	NP_649172.3	796	1	0.85
smt3	NP_477411.1	1530	0	0.00	CG14184	NP_649173.1	270	1	2.50
snRNP-U1-70k	NP_477205.1	669	4	4.04	sNPF-R	NP_524176.1	203	1	3.33
uif	NP_001137804.1	196	1	3.45	CG14186	NP_649174.1	80	0	0.00
CG43321	NP_001245925.1	3	0	0.00	CG14185	NP_649175.1	309	0	0.00
CG43322	NP_609092.1	92	1	7.35	Ir76b	NP_649176.1	184	1	3.67
Gart	NP_523497.2	1544	5	2.19	CG14187	NP_001262087.1	69	1	9.80
Pcp	NP_476673.1	83	1	8.14	CG7365	NP_649178.1	43	0	0.00
CG31908	NP_723239.1	43	0	0.00	gig	NP_524177.1	555	1	1.22
MME1	NP_609093.1	213	0	0.00	obst-J	NP_649179.2	27	2	50.07
Rab30	NP_609094.1	208	0	0.00	CG7335	NP_649180.1	668	0	0.00
Caper	NP_609095.1	417	1	1.62	CG7328	NP_649181.1	37	0	0.00
milt	NP_723249.1	197	1	3.43	CG42674	NP_001189137.1	226	0	0.00
Mnn1	NP_001036342.1	287	0	0.00	Ssk	NP_649184.1	84	0	0.00
Mst27D	NP_723253.2	609	0	0.00	CG42833	NP_001189138.1	0	0	#DIV/0!
Sem1	NP_652555.1	428	0	0.00	obst-F	NP_649186.1	121	4	22.34
Nuf2	NP_609096.2	192	0	0.00	CG7298	NP_649187.3	197	6	20.59
Ugt307A1	NP_609097.2	21	0	0.00	CG7290	NP_649188.1	154	5	21.94
Pvf2	NP_523499.2	91	0	0.00	CG6996	NP_649189.2	2	0	0.00
Pvf3	NP_001285706.1	39	0	0.00	CG7017	NP_649190.1	77	3	26.33
MICU1	NP_609100.1	54	0	0.00	CG6933	NP_649191.1	232	5	14.57
CG4496	NP_609101.1	55	0	0.00	CG17145	NP_649192.2	160	4	16.90
CG4497	NP_609102.1	57	1	11.86	CG17147	NP_001262099.1	0	0	#DIV/0!
CG4502	NP_609103.1	105	1	6.44	Su(var)3-3	NP_649194.1	896	0	0.00
CG13784	NP_001260176.1	110	1	6.14	CG13813	NP_649195.1	2	0	0.00
mEFG1	NP_609105.1	1328	3	1.53	CG17233	NP_730499.1	140	0	0.00
Ndae1	NP_001260177.1	217	3	9.34	Clc	NP_524178.2	605	0	0.00
CG43799	NP_001260184.1	13	0	0.00	CG6951	NP_649197.1	298	0	0.00
CG43800	NP_001260185.1	11	0	0.00	rdgC	NP_536738.3	526	1	1.28
CG13786	NP_609106.2	7	2	193.11	CG32228	NP_730504.1	3	0	0.00
Wnt4	NP_001260187.1	938	3	2.16	gogo	NP_649200.2	56	0	0.00
CG31909	NP_723266.1	196	0	0.00	CG17122	NP_730506.1	377	0	0.00
wg	NP_523502.1	1289	3	1.57	FRG1	NP_649202.1	207	1	3.27
Wnt6	NP_609108.3	182	3	11.14	Pex23	NP_730508.1	167	0	0.00
Wnt10	NP_609109.3	148	3	13.70	SCCRO4	NP_649204.2	188	1	3.60
ninaC	NP_523503.2	723	2	1.87	CG32225	NP_730511.1	209	0	0.00
CG5149	NP_609110.1	45	0	0.00	polo	NP_524179.2	1322	2	1.02
gudu	NP_609111.1	764	0	0.00	alphaSnap	NP_524180.1	410	0	0.00
CG5160	NP_609112.1	309	1	2.19	Spn77Ba	NP_649205.3	109	0	0.00
CG5171	NP_001188709.1	341	2	3.96	Spn77Bb	NP_649206.1	128	0	0.00
CG5177	NP_609114.1	392	1	1.72	Spn77Bc	NP_649207.1	125	0	0.00
CG5181	NP_609115.1	137	0	0.00	Rbbp5	NP_649209.1	343	2	3.94
CG15818	NP_609116.1	60	1	11.27	eRF1	NP_001262105.1	922	0	0.00
chm	NP_609117.4	596	1	1.13	CG5618	NP_649211.1	270	0	0.00
muc	NP_609118.1	753	4	3.59	kin17	NP_649212.2	189	1	3.58
CG5958	NP_609119.2	81	4	33.38	CG5665	NP_001246844.1	103	0	0.00
CG5973	NP_609120.1	70	4	38.62	DNApol-alpha	NP_652001.2	689	2	1.96
santa-maria	NP_723277.3	302	5	11.19	Toll-9	NP_001246845.1	436	0	0.00
Gr28a	NP_523504.2	96	0	0.00	in	NP_788548.1	145	1	4.66
Gr28b	NP_647614.2	146	0	0.00	CG13247	NP_649215.2	0	0	#DIV/0!
CG6055	NP_609122.1	71	0	0.00	Ide	NP_524182.3	233	0	0.00
Ziz	NP_001260200.1	216	5	15.65	RhoBTB	NP_001262107.1	764	3	2.65
CG6441	NP_609124.3	631	0	0.00	CG5498	NP_730525.2	59	1	11.46
Obp28a	NP_523505.1	116	0	0.00	fbl	NP_597678.1	257	0	0.00
RapGAP1	NP_001097114.2	239	1	2.83	CG33969	NP_001034035.2	119	1	5.68
Spn28B	NP_609128.2	103	1	6.56	trbl	NP_524672.1	596	0	0.00
Cyp4d21	NP_609129.2	130	0	0.00	CG13248	NP_649219.1	148	1	4.57
CG6739	NP_609130.1	26	0	0.00	zye	NP_649220.1	43	0	0.00
Slob	NP_609131.2	94	0	0.00	CG5282	NP_649221.1	122	0	0.00
Myo28B1	NP_723294.1	365	0	0.00	CG5274	NP_649222.1	23	0	0.00
CG18585	NP_609132.1	234	2	5.78	CG5262	NP_649223.1	56	0	0.00
CG7025	NP_609133.2	250	2	5.41	Las	NP_524183.2	464	0	0.00
CG43235	NP_001245931.1	12	0	0.00	Psn	NP_524184.1	674	0	0.00
Tep2	NP_523506.1	456	3	4.45	mRpL15	NP_524185.1	860	0	0.00

Tep3	NP_523507.2	168	0	0.00	CtIP	NP_649224.1	21	0	0.00
Ntl	NP_609135.1	264	1	2.56	Hpd	NP_730536.1	320	2	4.22
CG33296	NP_995644.2	89	0	0.00	CG5910	NP_649226.1	0	0	#DIV/0!
CG13793	NP_609136.2	123	0	0.00	CG5199	NP_649227.2	133	0	0.00
CG13794	NP_609137.2	93	0	0.00	atk	NP_649228.1	188	0	0.00
CG13795	NP_609138.3	102	0	0.00	mag	NP_649229.1	264	0	0.00
CG13796	NP_723308.2	77	0	0.00	CG42764	NP_001189141.1	0	0	#DIV/0!
CG31904	NP_723310.1	76	0	0.00	CG5955	NP_649230.1	220	2	6.14
Acp1	NP_477115.1	115	2	11.75	CG32428	NP_001189142.1	1	0	0.00
CG7214	NP_609141.1	111	2	12.18	CG5969	NP_788549.1	68	0	0.00
ATPsynGL	NP_609142.1	981	3	2.07	isoQC	NP_788550.1	58	1	11.65
CG7203	NP_609143.2	70	2	19.31	ZnT77C	NP_649233.2	193	1	3.50
CG46025	NP_609145.2	308	2	4.39	ND-39	NP_649234.1	650	2	2.08
CG7191	NP_609146.3	6	0	0.00	CG32425	NP_001097649.1	134	1	5.04
CG7179	NP_723315.3	0	0	#DIV/0!	CG18281	NP_649236.1	110	0	0.00
Uro	NP_476779.1	277	0	0.00	CG17637	NP_649237.1	122	0	0.00
CG7164	NP_609147.1	788	2	1.72	CG5078	NP_649238.3	38	0	0.00
CG7154	NP_609148.1	459	0	0.00	CG5059	NP_649239.1	320	1	2.11
CG7149	NP_001285717.1	193	1	3.50	mTerf3	NP_649240.1	266	1	2.54
LKRSDH	NP_609150.2	420	1	1.61	CG5104	NP_649241.1	160	0	0.00
Herp	NP_723316.1	138	0	0.00	l(3)77CDf	NP_001262118.1	143	0	0.00
r2d2	NP_609152.1	181	0	0.00	CG4858	NP_649243.1	380	0	0.00
cdc14	NP_609153.1	659	2	2.05	Rcd2	NP_649244.3	48	0	0.00
LanB1	NP_476618.1	253	1	2.67	CG13250	NP_649245.1	0	0	#DIV/0!
CG7115	NP_001260215.1	493	2	2.74	knrl	NP_788552.1	107	0	0.00
Rack1	NP_477269.1	1347	3	1.51	CG13251	NP_649246.1	33	0	0.00
mts	NP_476805.1	1239	3	1.64	kni	NP_001287130.1	179	0	0.00
CG14537	NP_609155.1	545	0	0.00	cmpy	NP_001097652.1	71	0	0.00
CG14535	NP_609156.1	320	0	0.00	CG34260	NP_001262120.1	6	0	0.00
lectin-28C	NP_652636.2	88	0	0.00	CG13252	NP_649248.3	25	0	0.00
TwdlE	NP_609157.3	73	0	0.00	Gr77a	NP_730560.1	106	0	0.00
Proc	NP_609158.2	136	0	0.00	CG33912	NP_001027139.1	0	0	#DIV/0!
spz3	NP_609160.2	63	0	0.00	CG43251	NP_001246850.1	0	0	#DIV/0!
CG7102	NP_787994.1	97	0	0.00	CG4074	NP_649249.2	111	0	0.00
SmE	NP_609162.1	1140	1	0.59	CG4186	NP_652281.1	105	0	0.00
CG34132	NP_001036344.1	524	2	2.58	CG4042	NP_649250.2	103	0	0.00
Spn28Da	NP_001036345.1	100	0	0.00	Pitslre	NP_649251.2	268	0	0.00
Spn28Db	NP_001356884.1	75	0	0.00	Pex16	NP_649252.1	223	1	3.03
Snoo	NP_001097115.1	143	0	0.00	Pex14	NP_649253.1	165	2	8.19
CG7231	NP_723327.1	110	0	0.00	CG11399	NP_649254.2	150	0	0.00
pes	NP_001137808.1	276	1	2.45	CG11396	NP_649255.1	62	0	0.00
CG7227	NP_609169.1	256	1	2.64	Six4	NP_649256.1	196	0	0.00
Sirup	NP_609170.1	110	0	0.00	CG3698	NP_649257.1	5	0	0.00
Wwox	NP_609171.1	204	0	0.00	tzn	NP_730568.1	313	0	0.00
Spn28Dc	NP_609172.1	142	1	4.76	Spc105R	NP_001262123.1	272	0	0.00
CG12560	NP_001285736.1	1	0	0.00	CG3634	NP_649260.1	56	0	0.00
Glyat	NP_001033883.2	10	0	0.00	HIPP1	NP_649261.1	285	1	2.37
Tg	NP_609174.1	229	2	5.90	CG3618	NP_649262.1	27	2	50.07
CG7367	NP_001162906.1	5	0	0.00	Pka-R1	NP_001189147.1	791	2	1.71
baf	NP_609176.1	517	0	0.00	Mst77F	NP_524188.1	736	3	2.76
Cka	NP_001245936.1	468	0	0.00	CG3288	NP_649264.1	1	0	0.00
CCDC53	NP_609178.1	95	1	7.11	CG13255	NP_652282.1	0	0	#DIV/0!
RpL36A	NP_609179.2	679	0	0.00	CSN3	NP_524190.2	362	0	0.00
Megf8	NP_609180.3	108	0	0.00	CG11456	NP_649265.1	41	0	0.00
poe	NP_476986.1	616	2	2.19	CG32432	NP_001262131.1	90	0	0.00
MED20	NP_001260223.1	149	0	0.00	Sfp77F	NP_001163485.1	0	0	#DIV/0!
Trf	NP_476939.1	393	0	0.00	CG43931	NP_001262132.1	0	0	#DIV/0!
Mcr	NP_524688.1	393	3	5.16	CG11458	NP_652283.1	29	0	0.00
Bsg	NP_723346.2	286	0	0.00	fng	NP_524191.1	272	0	0.00
mon2	NP_001033884.2	284	4	9.52	CG10589	NP_649269.1	699	4	3.87
CG8673	NP_609182.1	32	0	0.00	Sems	NP_649270.1	375	4	7.21
CG12375	NP_609183.1	274	0	0.00	CG10588	NP_649271.1	267	0	0.00
CG8668	NP_609184.1	82	0	0.00	CG11037	NP_649272.1	471	4	5.74
PAPLA1	NP_001285741.1	343	2	3.94	CG10587	NP_649273.1	156	2	8.67
CG42762	NP_001188716.1	0	0	#DIV/0!	CG12983	NP_001137992.1	790	5	4.28
CG42763	NP_001188717.1	0	0	#DIV/0!	CG33287	NP_001137993.1	7	1	96.56

CG31606	NP_723348.1	0	0	#DIV/0!	CG33286	NP_001097653.1	7	1	96.56
CG43394	NP_001245938.1	24	0	0.00	CG12984	NP_649276.2	1	0	0.00
Rbsn-5	NP_609186.1	343	1	1.97	skd	NP_001262134.1	573	0	0.00
Acbp1	NP_609187.1	244	2	5.54	Pdss2	NP_649277.1	409	1	1.65
Piezo	NP_001260226.1	121	1	5.59	CG10584	NP_649278.1	162	0	0.00
CG34134	NP_001036347.1	21	0	0.00	Sin	NP_524192.2	304	2	4.45
CG8475	NP_609189.2	86	0	0.00	CG10581	NP_649279.2	186	2	7.27
CG8460	NP_609190.2	75	0	0.00	siz	NP_730594.1	272	0	0.00
PGAP5	NP_609191.1	120	0	0.00	CG43072	NP_001246854.1	0	0	#DIV/0!
SLC5A11	NP_609192.1	30	0	0.00	ko	NP_524193.2	323	2	4.19
CG8419	NP_609193.1	222	0	0.00	CG33285	NP_996130.1	0	0	#DIV/0!
Su(var)205	NP_476755.1	903	1	0.75	CG43938	NP_001262136.1	0	0	#DIV/0!
Ssb-c31a	NP_477136.1	457	2	2.96	CG33284	NP_996131.1	571	0	0.00
CG8372	NP_723362.1	160	0	0.00	ICA69	NP_649283.1	185	1	3.65
CG8360	NP_609195.1	412	0	0.00	CG10565	NP_649284.1	642	1	1.05
CG8353	NP_609196.1	411	0	0.00	Ac78C	NP_001262138.1	260	2	5.20
CG8349	NP_609197.2	934	1	0.72	AsnS	NP_996132.1	433	1	1.56
CG8292	NP_609198.1	132	1	5.12	chb	NP_524651.2	315	1	2.15
Snx6	NP_609199.1	276	0	0.00	CG42337	NP_649286.2	183	0	0.00
Pvr	NP_787997.2	510	1	1.33	park	NP_730600.1	636	1	1.06
Spn28F	NP_524957.2	474	0	0.00	CG34261	NP_001097656.1	42	0	0.00
CG14277	NP_609200.1	5	0	0.00	CG33054	NP_788553.1	36	0	0.00
CG43057	NP_001245940.1	0	0	#DIV/0!	CG33056	NP_788555.1	45	0	0.00
CG8086	NP_001260238.1	46	0	0.00	CG10512	NP_649289.2	21	0	0.00
Btk29A	NP_001097121.1	585	1	1.16	CG10510	NP_649291.2	623	1	1.08
wol	NP_609202.1	511	2	2.65	CG10508	NP_001246859.1	2	0	0.00
Scgalpha	NP_001260246.1	103	0	0.00	CG12975	NP_649293.1	530	0	0.00
CG7840	NP_609203.1	246	2	5.50	Ilk	NP_525001.2	539	1	1.25
Ostgamma	NP_609204.2	411	1	1.64	CG43219	NP_001246861.1	0	0	#DIV/0!
Mettl14	NP_609205.1	316	1	2.14	Eip78C	NP_524195.2	484	0	0.00
CG7810	NP_609206.1	115	0	0.00	CG43218	NP_001246864.1	0	0	#DIV/0!
CG7806	NP_609207.1	148	1	4.57	CG9391	NP_649294.1	206	0	0.00
mtsh	NP_609208.2	39	0	0.00	CG9389	NP_649295.1	790	1	0.86
strat	NP_609209.2	84	0	0.00	AcCoAS	NP_730611.1	715	1	0.95
CG7781	NP_609210.1	5	0	0.00	ppl	NP_524197.1	354	2	3.82
CG14275	NP_609211.1	3	0	0.00	CG12974	NP_730613.2	50	0	0.00
CG14274	NP_609212.2	1	0	0.00	pzg	NP_649297.1	341	0	0.00
CG14273	NP_609213.2	0	0	#DIV/0!	Cpr78Ca	NP_649298.2	0	0	#DIV/0!
CG7778	NP_609214.1	37	0	0.00	Cpr78Cb	NP_001262144.1	25	0	0.00
Mur29B	NP_723377.1	47	0	0.00	Edg78E	NP_524198.1	54	1	12.52
CG7627	NP_001245943.1	367	2	3.68	CG43702	NP_001262145.1	0	0	#DIV/0!
RpS13	NP_476938.1	1144	2	1.18	Cpr78Cc	NP_649300.1	52	1	13.00
CSN8	NP_723378.2	352	0	0.00	CG11309	NP_730617.1	87	0	0.00
Pp2A-29B	NP_001245944.1	1023	3	1.98	CG7632	NP_649302.1	19	0	0.00
CG17292	NP_609216.1	30	0	0.00	Pc	NP_524199.1	558	0	0.00
Wdr82	NP_609217.1	486	2	2.78	Rab26	NP_649303.2	304	0	0.00
CG13390	NP_609218.1	397	2	3.41	Tsr1	NP_649304.1	873	0	0.00
CG17294	NP_609219.1	141	1	4.79	CG7324	NP_001262153.1	412	0	0.00
AlaRS	NP_523511.2	729	1	0.93	CG32436	NP_730622.1	629	0	0.00
CG13392	NP_609220.1	6	0	0.00	ebd2	NP_649307.1	9	0	0.00
Rcd4	NP_609221.1	69	0	0.00	CG12971	NP_001262154.1	0	0	#DIV/0!
Dad1	NP_609222.1	398	0	0.00	CG32437	NP_730625.1	286	1	2.36
CG13384	NP_723383.1	69	0	0.00	CG32440	NP_730626.1	344	2	3.93
CG42819	NP_001188728.1	0	0	#DIV/0!	sa	NP_649311.1	295	0	0.00
CG42820	NP_995659.1	4	0	0.00	rgn	NP_001246866.1	69	1	9.80
Acp29AB	NP_523512.2	300	2	4.51	barc	NP_649313.1	246	0	0.00
lectin-29Ca	NP_652640.1	114	1	5.93	CG33288	NP_996136.2	277	0	0.00
Peritrophin-15	NP_001260248.1	104	0	0.00	Aef1	NP_001262157.1	122	0	0.00
Peritrophin-15	NP_524983.1	226	0	0.00	CG5664	NP_649314.2	27	0	0.00
CG13385	NP_609225.1	703	1	0.96	Alp1	NP_649315.1	94	1	7.19
CG13386	NP_609226.1	0	0	#DIV/0!	Syx7	NP_730632.1	469	1	1.44
CG31898	NP_723390.1	3	0	0.00	croc	NP_524202.1	321	2	4.21
fy	NP_477500.1	214	0	0.00	Neu2	NP_001262160.1	62	1	10.90
emb	NP_723391.2	1351	0	0.00	CG7202	NP_649317.1	669	0	0.00
CG13397	NP_652045.1	148	0	0.00	CG7519	NP_649318.1	355	0	0.00
CG13398	NP_609227.1	139	1	4.86	Hr78	NP_524203.2	223	0	0.00

Akap200	NP_787998.1	109	1	6.20	Glg1	NP_788563.1	136	3	14.91
grk	NP_476568.2	289	0	0.00	Est-Q	NP_649321.1	123	0	0.00
D12	NP_609228.2	602	2	2.25	M6	NP_649323.2	123	1	5.50
Chrac-14	NP_476646.2	608	2	2.22	SAK	NP_649324.1	957	1	0.71
mus201	NP_001027230.1	631	2	2.14	Cdk12	NP_649325.2	738	1	0.92
CG31897	NP_723396.1	0	0	#DIV/0!	CG7611	NP_649326.1	218	0	0.00
Aasdh	NP_609230.2	867	1	0.78	Arv1	NP_730651.1	105	0	0.00
Agpat2	NP_609231.2	355	0	0.00	Mkrn1	NP_524704.1	247	0	0.00
prg	NP_609232.2	102	0	0.00	ppk5	NP_996138.2	49	0	0.00
d	NP_001188730.1	625	1	1.08	Rpn10	NP_524204.1	842	0	0.00
CheA29a	NP_788004.1	1	0	0.00	COX8	NP_649328.1	200	1	3.38
CG43796	NP_001260254.1	539	0	0.00	VhaM9.7-b	NP_649327.2	253	1	2.67
CG13088	NP_609234.2	242	0	0.00	CG33290	NP_996139.1	7	0	0.00
CG43400	NP_001245945.1	0	0	#DIV/0!	Wnk	NP_001262174.3	230	1	2.94
Dh31	NP_523514.1	166	0	0.00	CG7173	NP_649330.1	3	0	0.00
Bace	NP_609235.1	236	0	0.00	CapaR	NP_996140.1	157	0	0.00
CG13096	NP_609236.2	793	1	0.85	CG7634	NP_649332.1	667	0	0.00
CG13097	NP_609237.1	733	1	0.92	CRIF	NP_649333.1	263	0	0.00
PIG-U	NP_609238.1	91	0	0.00	SMC5	NP_996141.2	649	0	0.00
mRpL51	NP_609239.1	303	0	0.00	EMC10	NP_730661.2	519	0	0.00
Uba4	NP_609240.2	412	1	1.64	TfAP-2	NP_001262180.1	197	0	0.00
Sgp	NP_609241.1	156	0	0.00	S1P	NP_649337.2	396	0	0.00
Acer	NP_477195.1	189	1	3.58	CG11307	NP_001163490.1	26	0	0.00
lmgA	NP_001097124.1	554	1	1.22	CG7166	NP_649339.2	5	0	0.00
CG17834	NP_609242.2	2	0	0.00	Alg11	NP_649340.1	363	0	0.00
Sema1a	NP_001260265.1	273	0	0.00	MED1	NP_649341.1	276	0	0.00
Glt	NP_477504.1	155	1	4.36	ORMDL	NP_730669.1	238	0	0.00
CG9287	NP_609244.1	46	0	0.00	CG32444	NP_730670.1	165	0	0.00
CG9289	NP_609245.1	56	4	48.28	CG32445	NP_730671.1	967	1	0.70
PrBP	NP_609246.1	211	3	9.61	Atox1	NP_001262184.1	254	0	0.00
Trs23	NP_609247.1	278	3	7.29	CG43980	NP_996142.1	129	0	0.00
Hnf4	NP_476887.2	355	4	7.62	Cpr78E	NP_649345.1	86	0	0.00
CG9314	NP_609248.1	1555	2	0.87	CG11249	NP_649346.1	960	2	1.41
raw	NP_524789.2	88	0	0.00	Als2	NP_649347.1	436	0	0.00
CG12438	NP_609249.1	650	2	2.08	CG11248	NP_649348.1	0	0	#DIV/0!
LManIII	NP_609250.2	186	0	0.00	Sfp78E	NP_001163491.1	0	0	#DIV/0!
LManIV	NP_609251.2	56	0	0.00	CG32447	NP_001262188.1	32	0	0.00
LManV	NP_609252.1	269	0	0.00	CG11247	NP_001097659.1	61	0	0.00
LManVI	NP_609253.1	318	1	2.13	Rpb8	NP_649352.1	962	1	0.70
CG42710	NP_001188734.1	0	0	#DIV/0!	CG14570	NP_649353.1	5	1	135.18
SoxN	NP_001260269.1	522	1	1.29	CG14569	NP_649354.1	31	2	43.61
CG32986	NP_788006.1	0	0	#DIV/0!	CG14568	NP_649355.1	47	2	28.76
CG32987	NP_788007.1	0	0	#DIV/0!	CG14573	NP_649356.1	11	4	245.78
CG32988	NP_788008.1	7	1	96.56	CG14567	NP_649357.1	17	1	39.76
CG32983	NP_788009.1	0	0	#DIV/0!	CG14566	NP_649358.1	3	1	225.30
CG9483	NP_609255.1	461	0	0.00	CG14572	NP_649359.1	18	5	187.75
CG42713	NP_001188736.1	12	0	0.00	CG14565	NP_649360.1	38	2	35.57
CG34398	NP_001097128.1	38	0	0.00	Syn1	NP_730686.3	357	0	0.00
Tsp29Fa	NP_523515.1	301	2	4.49	CG14564	NP_649361.1	21	2	64.37
Tsp29Fb	NP_523516.1	158	1	4.28	CG7370	NP_649363.1	0	0	#DIV/0!
Argl	NP_609257.3	398	1	1.70	CG43895	NP_001262194.1	0	0	#DIV/0!
CG9515	NP_001285769.1	130	1	5.20	eg	NP_524206.1	134	0	0.00
C1GalTA	NP_609258.1	241	0	0.00	CycH	NP_524207.1	615	1	1.10
CG31886	NP_001097129.1	1	0	0.00	CG7407	NP_649364.1	42	0	0.00
CG9525	NP_001260270.1	0	0	#DIV/0!	CG7414	NP_649365.1	607	1	1.11
CG32985	NP_788013.2	13	0	0.00	CG32448	NP_730691.2	17	0	0.00
CG32984	NP_788014.2	0	0	#DIV/0!	CG7148	NP_649366.1	68	0	0.00
CG18088	NP_609262.1	0	0	#DIV/0!	Nopp140	NP_730693.1	788	2	1.72
CG9541	NP_609263.2	409	0	0.00	P5CDh1	NP_730695.1	233	2	5.80
CG13101	NP_609264.1	0	0	#DIV/0!	CG14563	NP_649369.1	0	0	#DIV/0!
CG18662	NP_652561.1	832	1	0.81	mub	NP_001246876.1	699	0	0.00
rost	NP_723435.1	43	2	31.44	SLC22A	NP_649370.1	50	1	13.52
CG9555	NP_609265.1	0	0	#DIV/0!	DNApol-eta	NP_649371.2	660	2	2.05
CG17906	NP_609266.1	0	0	#DIV/0!	CG14562	NP_649372.1	290	0	0.00
alien	NP_523517.1	724	1	0.93	CG7458	NP_649374.1	16	0	0.00
CG18661	NP_652562.2	34	0	0.00	Chs2	NP_524209.3	538	2	2.51

Try29F	NP_523518.2	75	1	9.01	CG7470	NP_649375.1	1246	7	3.80
CG9568	NP_609267.1	121	1	5.59	Act79B	NP_524210.1	1156	3	1.75
CG13102	NP_609268.2	92	2	14.69	CG7140	NP_649376.3	1343	2	1.01
Rcd-1r	NP_609269.1	163	2	8.29	CG43392	NP_001246878.1	44	0	0.00
CG9582	NP_609270.1	741	2	1.82	Oct-TyrR	NP_524419.2	180	1	3.76
DIP-zeta	NP_001188744.1	23	0	0.00	CG14561	NP_649377.1	38	0	0.00
CG12439	NP_609274.2	0	0	#DIV/0!	CG7139	NP_649378.1	182	1	3.71
Toll-4	NP_523519.2	406	0	0.00	CG7133	NP_649379.1	390	2	3.47
CG31609	NP_723444.2	45	0	0.00	CG7130	NP_649380.1	164	1	4.12
Or30a	NP_523520.2	47	0	0.00	RpLP0	NP_524211.1	1267	6	3.20
CG32982	NP_001260277.1	11	0	0.00	Sfp79B	NP_001163495.1	0	0	#DIV/0!
CG13108	NP_609276.1	0	0	#DIV/0!	msopa	NP_524859.2	46	0	0.00
CG9586	NP_609277.1	65	0	0.00	olf413	NP_730711.3	98	1	6.90
tai	NP_001245949.1	300	2	4.51	Rich	NP_649385.1	308	0	0.00
aust	NP_609278.1	52	4	51.99	Ddx1	NP_524212.2	770	2	1.76
borr	NP_609279.1	339	3	5.98	CG11523	NP_649386.1	48	0	0.00
lectin-30A	NP_652635.2	108	0	0.00	Csp	NP_730713.1	537	3	3.78
Ggamma30A	NP_001285776.1	157	0	0.00	Srpk79D	NP_001246881.1	371	2	3.64
CG17005	NP_609280.2	44	1	15.36	Hem	NP_524214.1	267	1	2.53
Cks30A	NP_476947.1	652	4	4.15	IleRS	NP_730716.1	881	2	1.53
Eaat1	NP_001369123.1	450	1	1.50	Ten-m	NP_001262211.1	330	0	0.00
CG3748	NP_609281.1	389	1	1.74	CG32450	NP_730717.1	298	0	0.00
CG43350	NP_001245951.1	0	0	#DIV/0!	CG11449	NP_649390.1	63	1	10.73
CG13110	NP_609282.1	859	9	7.08	laza	NP_649391.1	132	0	0.00
CG34181	NP_001097130.1	1	0	0.00	CG11438	NP_649392.1	132	1	5.12
Shawl	NP_609284.3	526	2	2.57	CG11437	NP_649393.1	92	0	0.00
Aldh	NP_609285.1	691	1	0.98	CG11426	NP_649394.1	211	1	3.20
CG4438	NP_609286.1	502	1	1.35	CG11425	NP_649395.1	153	0	0.00
Mco1	NP_609287.3	221	3	9.18	CG43312	NP_001246883.1	0	0	#DIV/0!
Fbp2	NP_523522.1	124	0	0.00	CG43331	NP_001246884.1	66	1	10.24
CG42847	NP_001188756.1	0	0	#DIV/0!	CG43330	NP_001246885.1	3	0	0.00
FucTB	NP_609288.4	113	0	0.00	CG43329	NP_001246886.1	3	0	0.00
scat	NP_524905.1	254	1	2.66	CG11404	NP_649397.1	525	1	1.29
CG3769	NP_609289.1	180	0	0.00	Trxr-2	NP_524216.1	887	1	0.76
RpS28-like	NP_001097132.1	75	0	0.00	CG14459	NP_001356911.1	21	0	0.00
CG33723	NP_001027235.1	0	0	#DIV/0!	ND-B14.5AL	NP_649399.1	923	3	2.20
numb	NP_523523.2	409	1	1.65	CG33766	NP_001027140.1	0	0	#DIV/0!
Gdi	NP_523524.2	708	0	0.00	CG33767	NP_001027141.1	0	0	#DIV/0!
CG33298	NP_995666.1	413	1	1.64	CG33768	NP_001027142.2	0	0	#DIV/0!
CG31883	NP_609292.2	230	0	0.00	CG33769	NP_001027143.1	0	0	#DIV/0!
Oatp30B	NP_723463.1	92	1	7.35	CG33770	NP_001027144.2	0	0	#DIV/0!
CG17855	NP_609294.1	13	0	0.00	CG33771	NP_001027145.3	0	0	#DIV/0!
Cpr30B	NP_609295.1	23	0	0.00	CG33772	NP_001027146.4	0	0	#DIV/0!
CG13113	NP_609296.1	57	0	0.00	CG14457	NP_001014600.1	18	0	0.00
CG13114	NP_609297.2	72	0	0.00	CG14455	NP_649402.2	0	0	#DIV/0!
brwl	NP_609298.1	41	0	0.00	CG14456	NP_001137998.1	1	0	0.00
Mtpalpha	NP_609299.1	454	0	0.00	CG14454	NP_649404.1	1	0	0.00
CG31709	NP_723472.1	569	1	1.19	CG12546	NP_649405.2	1	0	0.00
gcm	NP_477108.1	266	0	0.00	CG14453	NP_649406.2	3	0	0.00
CG3841	NP_609300.1	88	0	0.00	CG14452	NP_649407.1	0	0	#DIV/0!
CG4382	NP_609301.2	25	0	0.00	CG32453	NP_730733.1	0	0	#DIV/0!
gcm2	NP_609302.1	123	0	0.00	CG11370	NP_649408.1	28	0	0.00
Trx-2	NP_523526.1	623	0	0.00	ArfGAP3	NP_001262216.1	472	0	0.00
GlcAT-S	NP_723476.1	176	0	0.00	CG32452	NP_730736.1	23	0	0.00
CG31882	NP_723477.1	482	1	1.40	CG14451	NP_649410.1	31	0	0.00
Apoltp	NP_995670.2	354	0	0.00	mael	NP_788566.1	261	0	0.00
CG33299	NP_995671.2	1	0	0.00	CG14450	NP_649411.1	4	0	0.00
Pka-C1	NP_476977.1	860	1	0.79	CG11367	NP_649412.1	240	0	0.00
hoip	NP_524714.1	1443	4	1.87	CG32454	NP_730740.1	386	0	0.00
pelo	NP_476982.1	330	2	4.10	CG11241	NP_730741.2	302	0	0.00
CG31710	NP_723480.1	27	3	75.10	l(3)04053	NP_649414.1	50	0	0.00
CG4364	NP_609305.1	813	2	1.66	CG7369	NP_649415.2	20	0	0.00
Nckx30C	NP_001260296.1	112	0	0.00	SPoCk	NP_730742.1	490	0	0.00
CG13116	NP_609306.2	0	0	#DIV/0!	CG14448	NP_649418.1	732	1	0.92
CG13117	NP_609307.1	1	0	0.00	jim	NP_001262221.1	55	0	0.00
Cyp4e3	NP_523527.1	161	0	0.00	CG11226	NP_649420.2	100	0	0.00

zf30C	NP_477301.1	208	0	0.00	CG32462	NP_730750.1	4	0	0.00
Taf11	NP_723484.1	313	0	0.00	CG32459	NP_730752.1	523	1	1.29
und	NP_477373.1	947	2	1.43	CG13239	NP_649422.1	2	0	0.00
ppk18	NP_609308.3	50	1	13.52	CG33169	NP_001137999.1	11	0	0.00
CG4017	NP_609309.1	49	0	0.00	CG33170	NP_001138000.1	9	0	0.00
CG17633	NP_609310.2	314	0	0.00	EMC4	NP_649424.1	270	0	0.00
ppk11	NP_001334673.1	126	1	5.36	Arf79F	NP_001097667.1	1010	0	0.00
IP3K1	NP_609313.2	314	0	0.00	CG11109	NP_730761.1	736	1	0.92
CG4036	NP_609314.1	39	1	17.33	Chro	NP_649426.3	201	0	0.00
CG13123	NP_609315.1	38	0	0.00	Ssl1	NP_649427.1	380	0	0.00
nAChRalpha6	NP_609316.4	184	0	0.00	slif	NP_649428.1	225	0	0.00
Fkbp59	NP_524895.2	673	1	1.00	CG11131	NP_649429.1	6	0	0.00
CG34183	NP_001097134.1	279	0	0.00	BoYb	NP_649430.1	889	3	2.28
CG4537	NP_001097135.1	61	0	0.00	CG12768	NP_649431.1	6	1	112.65
mtDNA-helica	NP_609318.1	356	1	1.90	Mes2	NP_730768.1	75	1	9.01
Bka	NP_524867.2	769	3	2.64	CG32461	NP_730770.1	3	0	0.00
RpS2	NP_476874.1	1155	4	2.34	CG32457	NP_730771.1	27	0	0.00
Muc30E	NP_995676.4	32	0	0.00	nrm	NP_001246891.1	89	0	0.00
CG13124	NP_001137811.1	100	0	0.00	RpL10	NP_651954.3	720	1	0.94
Etl1	NP_609320.2	461	1	1.47	CkIIalpha	NP_001036624.1	1126	4	2.40
Srp54	NP_477347.1	416	1	1.62	CG33217	NP_996147.1	145	2	9.32
yip2	NP_523528.1	552	3	3.67	Vps11	NP_730776.1	461	3	4.40
CG5885	NP_609321.1	237	0	0.00	CG34031	NP_001246894.1	52	0	0.00
CG4598	NP_609322.1	485	3	4.18	nAChRalpha4	NP_001097669.2	333	4	8.12
CG4594	NP_609323.1	264	3	7.68	alpha-Cat	NP_524219.1	428	3	4.74
CG4592	NP_001260306.1	283	3	7.17	ND-MLRQ	NP_730777.1	194	0	0.00
TbCMF46	NP_609325.2	394	0	0.00	AGO3	NP_001036627.2	268	0	0.00
CG42366	NP_001260307.1	240	0	0.00	CG17454	NP_001138001.1	356	4	7.59
CG42367	NP_723502.2	241	0	0.00	CG40298	NP_001036631.1	0	0	#DIV/0!
Cpr30F	NP_723504.1	85	1	7.95	CG17698	NP_001036633.2	364	0	0.00
CG31712	NP_609327.3	94	1	7.19	CG43968	NP_001262238.1	177	2	7.64
Apf	NP_723505.2	585	1	1.16	CG40470	NP_001036635.1	115	2	11.75
CG4619	NP_001162929.1	2	0	0.00	CG40045	NP_001036640.1	789	2	1.71
CG5853	NP_788019.1	48	0	0.00	Snap25	NP_001036641.1	566	3	3.58
CG13126	NP_609330.1	409	3	4.96	nvd	NP_001097670.1	173	0	0.00
CG4709	NP_609331.1	78	0	0.00	mRpS5	NP_001036652.1	1023	0	0.00
CG5846	NP_609333.1	439	0	0.00	scro	NP_001015473.1	226	3	8.97
CG4658	NP_788020.1	2	0	0.00	CG40472	NP_001163845.1	212	2	6.38
CG5850	NP_001260310.1	27	0	0.00	ND-AGGG	NP_001015476.1	233	3	8.70
Dref	NP_523529.1	464	1	1.46	Set1	NP_001104406.1	1174	1	0.58
RpL13	NP_523530.1	854	4	3.17	MED21	NP_001015223.1	412	2	3.28
bib	NP_001260313.1	282	2	4.79	UQCR-11	NP_001263163.1	429	2	3.15
CG31875	NP_723509.1	1037	3	1.96	lovit	NP_001303415.1	59	0	0.00
SoYb	NP_609335.3	904	4	2.99	FASN3	NP_001015405.3	279	2	4.85
Ndf	NP_001188767.1	405	1	1.67	l(3)80Fg	NP_001015188.2	242	1	2.94
Trp1	NP_477008.1	377	1	1.79	CG40160	NP_001015213.3	39	0	0.00
CG13131	NP_609337.2	1	0	0.00	CG41284	NP_001104417.3	1	0	0.00
CG13127	NP_609338.1	7	0	0.00	CG42598	NP_001163857.2	1	0	0.00
obst-B	NP_609339.1	79	0	0.00	CG40228	NP_001263167.1	143	1	6.30
CG33301	NP_001285793.1	76	0	0.00	vtd	NP_001015132.1	705	3	4.11
Cpr31A	NP_995678.1	33	0	0.00	RpL15	NP_001104430.1	885	4	4.70
Pen	NP_477041.1	766	0	0.00	Dbp80	NP_001263168.1	520	2	4.33
Spn31A	NP_609341.1	56	0	0.00	eIF4B	NP_001015319.1	1029	4	4.78
CG44153	NP_001097136.2	45	0	0.00	l(3)80Fj	NP_001015316.1	764	4	7.08
CG13133	NP_609343.1	42	0	0.00	Myo81F	NP_001303492.1	543	1	2.49
CG34109	NP_001036351.2	23	0	0.00	Pzl	NP_001303493.1	24	0	0.00
CG13135	NP_723521.1	0	0	#DIV/0!	spok	NP_001104460.2	133	0	0.00
CG4839	NP_609349.1	355	0	0.00	Parp	NP_001104452.1	783	3	3.98
CG13137	NP_609350.2	103	0	0.00	Alg-2	NP_001104459.2	190	0	0.00
Sur	NP_001334732.1	938	1	0.72	Tim17b	NP_001015401.1	354	2	5.09
Fum4	NP_723527.1	560	0	0.00	CG41128	NP_001015402.3	182	1	4.64
Ripalpha	NP_609351.1	19	0	0.00	CG41099	NP_001104451.1	2600	4	1.22
RpL7	NP_523531.1	933	0	0.00	CG42402	NP_001287642.1	215	0	0.00
CG34159	NP_001097137.1	43	0	0.00	Gfat1	NP_001015157.2	518	2	2.75
Snx17	NP_609353.1	133	0	0.00	CG40198	NP_001015164.3	5	0	0.00
CG5731	NP_609354.1	109	0	0.00	CG12581	NP_649435.2	42	0	0.00

CG5727	NP_609355.1	9	0	0.00	Dsk	NP_524845.2	132	0	0.00
CG4901	NP_001334733.1	758	2	1.78	beta-Man	NP_649436.2	282	5	11.98
Npc1a	NP_001285795.1	301	1	2.25	CG14636	NP_001036655.1	0	0	#DIV/0!
Prosalpha6	NP_523532.1	852	2	1.59	CG31516	NP_730784.1	0	0	#DIV/0!
CG4908	NP_609358.1	638	0	0.00	aux	NP_649438.1	2263	3	0.90
CG5708	NP_609359.1	149	3	13.61	DhpD	NP_649439.1	263	0	0.00
ova	NP_609360.2	117	3	17.33	CG14641	NP_649440.3	492	0	0.00
eEF1delta	NP_609361.1	790	2	1.71	abs	NP_524220.1	355	0	0.00
mthl15	NP_723538.3	16	0	0.00	Gel	NP_524865.2	439	1	1.54
me31B	NP_523533.2	1383	7	3.42	CG14642	NP_730791.1	62	0	0.00
chico	NP_723540.1	271	1	2.49	CG34305	NP_001097671.1	0	0	#DIV/0!
bsk	NP_723541.1	1207	2	1.12	TwdlF	NP_649443.1	97	0	0.00
CG31717	NP_723543.1	118	0	0.00	TwdlG	NP_649444.1	71	0	0.00
Ror	NP_476962.1	457	5	7.39	CG14644	NP_649445.3	606	0	0.00
CG5676	NP_609362.1	136	3	14.91	TwdlV	NP_649446.1	73	0	0.00
Pten	NP_477424.1	1407	5	2.40	Or82a	NP_730794.1	80	0	0.00
Rsf1	NP_477001.2	311	0	0.00	CG1092	NP_730797.2	26	0	0.00
REPTOR-BP	NP_609363.1	94	0	0.00	CG9766	NP_001097672.1	476	0	0.00
Mob3	NP_609364.1	162	1	4.17	cpx	NP_525019.2	473	0	0.00
CG4953	NP_609365.3	70	0	0.00	Nepl11	NP_649449.1	110	0	0.00
CG4957	NP_609366.2	266	0	0.00	MP1	NP_001138002.1	88	0	0.00
Mulk	NP_723549.1	203	0	0.00	Vps24	NP_649451.1	212	0	0.00
trk	NP_476767.2	104	1	6.50	Suv3	NP_649452.3	234	0	0.00
Utx	NP_001188773.1	2175	5	1.55	Syt14	NP_001262249.1	156	0	0.00
CG34043	NP_001033893.2	3	0	0.00	CG9795	NP_730812.3	38	0	0.00
Ufd4	NP_609369.1	492	3	4.12	CG9776	NP_649455.1	24	0	0.00
Hand	NP_609370.2	137	0	0.00	CG14645	NP_652325.2	6	0	0.00
CYLD	NP_723554.1	233	3	8.70	Skp2	NP_730815.2	814	0	0.00
CG13138	NP_609372.1	7	0	0.00	CG1103	NP_001036660.1	8	0	0.00
nmd	NP_609373.1	383	3	5.29	hkb	NP_524221.1	188	0	0.00
CG5390	NP_609374.1	58	0	0.00	CG1090	NP_649459.4	110	0	0.00
CG4968	NP_609375.1	189	2	7.15	Sfxn1-3	NP_730823.2	128	0	0.00
Cdk5alpha	NP_523535.2	221	0	0.00	Cont	NP_649461.2	284	0	0.00
l(2)SH0834	NP_609376.1	4	0	0.00	tub	NP_001189164.1	52	0	0.00
Usp14	NP_609377.1	524	4	5.16	CG14646	NP_649462.1	31	0	0.00
CG4972	NP_609378.1	207	1	3.27	CG34112	NP_001246902.1	0	0	#DIV/0!
CG5381	NP_001260324.1	9	0	0.00	CG9855	NP_649463.2	30	0	0.00
CG4995	NP_609380.1	439	0	0.00	CG9853	NP_001097680.1	180	1	3.76
RnrL	NP_477027.1	1316	4	2.05	CG14647	NP_649465.6	91	0	0.00
Schip1	NP_001334735.1	59	0	0.00	lost	NP_649466.1	239	0	0.00
CG5037	NP_609382.1	615	2	2.20	CG32944	NP_001036666.2	430	0	0.00
GATAd	NP_609383.2	449	1	1.51	CG31528	NP_730833.1	1282	2	1.05
SamDC	NP_477223.2	337	0	0.00	srl	NP_730836.3	401	0	0.00
CG31715	NP_723568.1	275	0	0.00	CG31525	NP_730837.1	20	0	0.00
Sps2	NP_477478.4	187	2	7.23	eIF3a	NP_649470.2	785	1	0.86
CG5022	NP_609384.1	125	0	0.00	CG1074	NP_649471.3	448	0	0.00
CG34367	NP_001097140.1	167	2	8.09	CG9804	NP_649472.1	165	0	0.00
CG5367	NP_609387.1	212	2	6.38	CG14650	NP_649473.1	236	2	5.73
ClpP	NP_609388.1	847	2	1.60	CG31522	NP_730841.2	64	0	0.00
Cand1	NP_609389.1	545	3	3.72	CG31523	NP_649474.1	66	0	0.00
pim	NP_476678.1	233	1	2.90	CG14651	NP_649475.3	50	0	0.00
lft	NP_609390.1	166	0	0.00	Fip1	NP_649476.1	523	1	1.29
CG5056	NP_609391.1	22	0	0.00	CG34357	NP_001356914.1	369	1	1.83
rho-5	NP_995679.1	696	2	1.94	CG34425	NP_001097683.1	3	0	0.00
CG33303	NP_001260335.1	385	2	3.51	Mur82C	NP_649481.2	0	0	#DIV/0!
dpr19	NP_609392.1	9	0	0.00	CG32945	NP_788575.1	153	0	0.00
gny	NP_609393.1	153	2	8.84	5-HT2A	NP_730859.1	187	1	3.61
CG5096	NP_723576.1	353	1	1.91	CG9775	NP_649482.2	184	1	3.67
Cdk1	NP_476797.1	2173	4	1.24	MED31	NP_001287159.1	280	1	2.41
mRpS7	NP_523537.1	1060	4	2.55	slx1	NP_649484.3	165	1	4.10
da	NP_001356956.1	524	0	0.00	Dlip2	NP_524224.2	48	0	0.00
Mdh1	NP_609394.1	981	2	1.38	rpk	NP_001246909.1	176	0	0.00
Cnot4	NP_001260343.1	499	1	1.35	Karybeta3	NP_524226.1	803	1	0.84
CG17768	NP_001188778.1	674	3	3.01	spartin	NP_649485.1	162	0	0.00
Ir31a	NP_001260346.1	75	0	0.00	CG33927	NP_001027147.1	0	0	#DIV/0!
lp259	NP_477379.1	931	4	2.90	smash	NP_001246912.1	546	1	1.24

RpS27A	NP_476778.1	1814	6	2.24	eIF3f1	NP_649489.1	832	2	1.62
CG5355	NP_609397.2	166	0	0.00	Nep2	NP_524227.1	138	0	0.00
Klp31E	NP_609398.2	544	2	2.48	tacc	NP_001262261.1	370	0	0.00
RfC3	NP_609399.1	889	5	3.80	CG44098	NP_001262265.1	19	0	0.00
pie	NP_524740.2	758	1	0.89	atms	NP_649493.1	381	0	0.00
Wdfy2	NP_609400.1	486	2	2.78	CG14655	NP_649494.1	76	1	8.89
SmB	NP_001188780.1	1280	3	1.58	CG17387	NP_649495.1	434	0	0.00
KdelR	NP_477296.1	432	1	1.56	ctrip	NP_001287163.1	1257	3	1.61
CG5188	NP_609401.2	712	1	0.95	Hus1-like	NP_477426.1	241	2	5.61
Bug22	NP_609402.1	203	1	3.33	CG1129	NP_649498.1	350	0	0.00
TfIIB	NP_476888.1	624	2	2.17	BBS5	NP_649499.1	169	0	0.00
TBC1D16	NP_609403.2	160	1	4.22	CG14657	NP_649501.1	2	0	0.00
holn1	NP_609404.2	377	2	3.59	CG14658	NP_649502.1	784	0	0.00
STUB1	NP_477441.1	590	1	1.15	CG14659	NP_649503.1	0	0	#DIV/0!
Nse4	NP_609405.2	149	2	9.07	opa	NP_001368991.1	234	1	2.89
loh	NP_609406.2	139	0	0.00	laf	NP_649504.1	237	0	0.00
ppk10	NP_001033894.1	91	0	0.00	Gnf1	NP_001014605.1	505	2	2.68
LManI	NP_609407.1	68	0	0.00	Cdep	NP_001036676.1	110	1	6.14
LManII	NP_609408.1	120	0	0.00	Ubc6	NP_524230.2	1111	2	1.22
RluA-1	NP_723592.1	380	1	1.78	CG14661	NP_649507.1	6	0	0.00
RluA-2	NP_609411.1	502	1	1.35	CG2016	NP_649508.1	50	1	13.52
Grip75	NP_609412.1	178	0	0.00	CG1124	NP_649509.1	58	1	11.65
Cog4	NP_609413.1	209	0	0.00	CG14662	NP_649510.2	0	0	#DIV/0!
CG6144	NP_001033895.1	21	0	0.00	plh	NP_649511.2	99	0	0.00
CG13144	NP_609415.1	15	0	0.00	corto	NP_524231.2	134	0	0.00
Myo31DF	NP_523538.1	579	1	1.17	CG43131	NP_001246924.1	0	0	#DIV/0!
CG6094	NP_609416.1	702	2	1.93	CG12007	NP_649512.1	166	0	0.00
CG7384	NP_609417.1	12	3	168.98	CG12589	NP_652322.1	4	0	0.00
Fatp1	NP_001162940.3	248	5	13.63	dpr16	NP_001287169.1	9	0	0.00
Lrr47	NP_523539.2	572	2	2.36	CG12590	NP_649513.1	0	0	#DIV/0!
Lip4	NP_001260356.1	106	1	6.38	cno	NP_730891.1	269	0	0.00
CG18301	NP_609419.1	54	2	25.03	Prosbeta2R2	NP_649515.3	367	1	1.84
CG18302	NP_609420.1	55	2	24.58	CG1116	NP_730894.2	112	1	6.03
Trim9	NP_001188786.1	351	1	1.93	Sccpdh1	NP_649517.1	95	0	0.00
CG6138	NP_723601.1	634	3	3.20	CG14664	NP_001246925.1	3	0	0.00
w-cup	NP_001188787.1	551	8	9.81	Vps37B	NP_649518.1	144	1	4.69
CG34160	NP_001097142.1	0	0	#DIV/0!	Kat60	NP_524997.2	532	2	2.54
CG34161	NP_001162942.1	370	2	3.65	Mms19	NP_649519.1	372	2	3.63
CG7329	NP_609425.1	88	2	15.36	rtp	NP_649520.1	150	1	4.51
CG31872	NP_723603.1	146	4	18.52	CG12163	NP_649521.1	289	0	0.00
CG18284	NP_609428.1	160	3	12.67	CG33293	NP_996150.1	188	1	3.60
CG17097	NP_609429.1	310	6	13.08	CG1113	NP_649522.1	119	1	5.68
dbf	NP_609430.1	763	5	4.43	CG12173	NP_649523.1	221	0	0.00
CG31871	NP_723607.1	86	4	31.44	Hph	NP_730906.1	400	1	1.69
CG17104	NP_609433.1	63	3	32.19	Tim17a2	NP_649524.1	242	0	0.00
CG17105	NP_609434.1	0	0	#DIV/0!	mtd	NP_001138008.1	294	0	0.00
CG17107	NP_609435.1	0	0	#DIV/0!	CG31538	NP_730917.1	507	2	2.67
CG7299	NP_609436.1	2	0	0.00	Tim17b1	NP_649526.2	248	0	0.00
CG7296	NP_609437.2	96	0	0.00	CG11999	NP_649527.1	241	1	2.80
CG7294	NP_609438.1	0	0	#DIV/0!	CG1161	NP_649528.2	48	0	0.00
CG17108	NP_609439.1	12	0	0.00	Prosbeta7	NP_649529.1	734	3	2.76
CG7300	NP_609440.2	0	0	#DIV/0!	Cerk	NP_730923.1	251	2	5.39
Lip1	NP_523540.1	55	1	12.29	CG31542	NP_730924.1	389	1	1.74
Lip2	NP_524667.1	107	0	0.00	RpII18	NP_524910.1	1162	2	1.16
CG6415	NP_609441.1	405	0	0.00	hd	NP_649531.1	392	1	1.72
CG6431	NP_001188790.1	1	0	0.00	CG14667	NP_001014607.1	49	0	0.00
CG6443	NP_609443.1	82	2	16.49	7B2	NP_001014608.1	254	1	2.66
CG17118	NP_723611.1	189	0	0.00	kkv	NP_730928.2	524	1	1.29
EMC3	NP_609444.1	160	2	8.45	CG14668	NP_649533.1	457	1	1.48
Dpy-30L1	NP_609445.1	329	2	4.11	CG1172	NP_649534.1	43	0	0.00
Nup107	NP_609446.1	777	1	0.87	Or83a	NP_524234.2	31	0	0.00
Vha16-5	NP_609447.1	402	0	0.00	CG2663	NP_730931.1	58	0	0.00
CG12299	NP_609448.1	151	0	0.00	Orco	NP_001097687.1	369	0	0.00
CG6729	NP_609449.1	173	0	0.00	CG12147	NP_649536.1	652	1	1.04
CG17124	NP_609450.1	209	1	3.23	CG14669	NP_649537.5	425	3	4.77
CG6495	NP_609451.1	131	1	5.16	PEK	NP_649538.1	430	1	1.57

Ubc2	NP_477137.1	873	2	1.55	RpL35A	NP_649539.1	686	0	0.00
CG6724	NP_001260363.1	988	2	1.37	POLDIP2	NP_730934.1	172	0	0.00
CG17127	NP_001137813.1	18	0	0.00	mRpL44	NP_649541.1	898	2	1.51
CG31869	NP_001137815.1	28	0	0.00	Snr1	NP_730935.1	899	1	0.75
CG31870	NP_723620.1	532	1	1.27	Hcs	NP_001287175.1	310	1	2.18
Nos	NP_523541.2	610	0	0.00	ksr	NP_524236.2	501	2	2.70
CG6508	NP_609457.1	210	1	3.22	CG31550	NP_001262285.1	329	0	0.00
CG17134	NP_609458.1	106	0	0.00	NKCC	NP_730938.1	92	0	0.00
CG6700	NP_609459.1	113	0	0.00	Nmdar1	NP_730940.1	731	3	2.77
CG17139	NP_001033896.1	423	2	3.20	Itpr	NP_730941.1	590	2	2.29
Porin2	NP_609462.1	931	3	2.18	CG2519	NP_001262286.1	34	0	0.00
porin	NP_476813.1	711	2	1.90	mia	NP_001138010.1	269	1	2.51
SmydA-3	NP_001245962.1	263	0	0.00	Pcmt	NP_536756.1	324	0	0.00
Dnz1	NP_477449.1	89	1	7.59	Snm1	NP_649548.1	628	1	1.08
aurB	NP_477336.1	1046	1	0.65	Pi4KIIalpha	NP_730944.2	512	0	0.00
Stam	NP_477448.1	335	0	0.00	CG14671	NP_649550.1	135	1	5.01
CG12517	NP_609465.1	9	1	75.10	CG12746	NP_649551.4	155	0	0.00
CG14071	NP_609466.1	394	1	1.72	CG2931	NP_649552.1	225	1	3.00
CG14070	NP_609467.1	227	0	0.00	Rheb	NP_730950.2	717	2	1.89
CG7309	NP_609468.4	620	0	0.00	CRMP	NP_730954.2	360	1	1.88
CG14069	NP_609469.1	7	1	96.56	CG2926	NP_649554.1	353	1	1.91
dpr2	NP_001162945.3	27	0	0.00	Rev7	NP_649555.1	193	1	3.50
CG14072	NP_609472.1	0	0	#DIV/0!	noi	NP_477114.1	854	3	2.37
CG33129	NP_788023.1	123	0	0.00	Vha26	NP_524237.1	361	1	1.87
YL-1	NP_609475.1	337	1	2.01	kra	NP_524238.1	321	1	2.11
CG16743	NP_001260367.1	30	0	0.00	SecS	NP_649556.3	235	1	2.88
abo	NP_524784.1	318	1	2.13	asl	NP_649557.2	276	2	4.90
ATPsynG	NP_001036353.1	583	2	2.32	Rga	NP_524239.2	366	1	1.85
SCAR	NP_001260369.1	246	1	2.75	Atu	NP_524240.1	363	2	3.72
piwi	NP_476875.1	509	0	0.00	Spec2	NP_730968.1	112	1	6.03
CG12253	NP_652056.2	67	0	0.00	CG2911	NP_001097689.1	272	1	2.48
TTLL4A	NP_723642.2	104	0	0.00	RpL13A	NP_649560.1	1065	1	0.63
aub	NP_476734.1	301	0	0.00	CG31551	NP_730971.1	0	0	#DIV/0!
lectin-33A	NP_001097145.1	68	0	0.00	CG31549	NP_730972.2	260	2	5.20
RpL9	NP_477161.1	1019	3	1.99	CG12171	NP_649563.1	306	4	8.84
Nup154	NP_477287.1	789	2	1.71	CG31546	NP_730973.1	579	1	1.17
Art8	NP_609478.1	730	2	1.85	CG31548	NP_730974.1	287	2	4.71
dUTPase	NP_609479.1	1211	3	1.67	CG12170	NP_649565.1	597	4	4.53
Samuel	NP_525101.2	107	2	12.63	MTA1-like	NP_001262293.1	250	1	2.70
Acp32CD	NP_477328.2	176	0	0.00	Sec23	NP_001246932.1	805	1	0.84
CG14913	NP_001097146.1	40	0	0.00	elm	NP_649568.1	448	1	1.51
CG18666	NP_652565.1	1	0	0.00	MED27	NP_649569.1	130	0	0.00
CG14915	NP_609482.1	10	0	0.00	CG2182	NP_649570.1	72	1	9.39
CG43725	NP_001260374.1	0	0	#DIV/0!	Wdr33	NP_730982.1	766	2	1.76
AstC	NP_001162948.1	137	1	4.93	Xe7	NP_649572.4	89	1	7.59
AstCC	NP_609483.2	79	1	8.56	Atg17	NP_649573.2	269	2	5.03
CG16854	NP_001245974.1	6	0	0.00	Rab23	NP_649574.1	210	0	0.00
Wdr59	NP_995682.1	370	4	7.31	plx	NP_001163511.1	182	1	3.71
Ge-1	NP_609486.2	243	3	8.34	CG2104	NP_649575.1	248	0	0.00
Reps	NP_001245975.1	241	2	5.61	cas	NP_001262300.1	87	0	0.00
l(2)gd1	NP_001245976.1	246	0	0.00	CG1239	NP_649577.1	237	0	0.00
CG6201	NP_609489.1	62	0	0.00	CG2100	NP_649578.1	358	1	1.89
Gr32a	NP_523543.3	154	0	0.00	CG1236	NP_649579.2	292	1	2.31
CG6230	NP_609490.1	483	0	0.00	Sym	NP_649580.1	579	1	1.17
CG14921	NP_609491.1	593	1	1.14	Madm	NP_649581.1	110	0	0.00
Csl4	NP_609492.1	593	1	1.14	CG2091	NP_649582.1	401	3	5.06
Nup160	NP_609493.1	313	1	2.16	Sec8	NP_730996.2	334	3	6.07
RfC38	NP_609494.1	1068	4	2.53	CG2082	NP_001246935.1	101	2	13.38
CG4751	NP_609495.1	591	0	0.00	jagn	NP_649585.1	100	1	6.76
hgo	NP_523544.2	358	0	0.00	kat-60L1	NP_001163522.1	497	3	4.08
Gyc32E	NP_001285834.1	224	0	0.00	Hat1	NP_649587.1	614	3	3.30
CG31867	NP_723660.1	76	0	0.00	Rpn5	NP_649588.1	806	2	1.68
CG6287	NP_609496.1	570	0	0.00	CG1218	NP_649589.1	218	1	3.10
Ca-beta	NP_995684.2	212	0	0.00	PSMG1	NP_649590.1	93	1	7.27
Vm32E	NP_477103.1	112	1	6.03	CG10979	NP_649591.1	129	0	0.00
CG4788	NP_609499.1	40	1	16.90	CG11000	NP_001097690.1	87	0	0.00

ab	NP_476562.1	80	0	0.00	CG42675	NP_649593.1	117	0	0.00
cmet	NP_001260382.1	675	2	2.00	Hpr1	NP_649594.1	343	0	0.00
cana	NP_001027247.1	556	1	1.22	Sec20	NP_649595.1	182	0	0.00
CG33695	NP_001027248.1	56	0	0.00	dgrn	NP_649596.1	255	0	0.00
Ppt2	NP_609500.1	217	2	6.23	glob3	NP_649597.3	9	0	0.00
mre11	NP_523547.1	624	1	1.08	CG1208	NP_649598.1	335	1	2.02
Osi21	NP_609501.1	35	0	0.00	CG1213	NP_649599.1	379	1	1.78
CG14926	NP_609502.1	851	1	0.79	CG14676	NP_649600.1	408	0	0.00
salr	NP_523548.1	243	2	5.56	disp	NP_524734.2	162	0	0.00
salm	NP_723670.2	289	0	0.00	CG34287	NP_001097693.1	0	0	#DIV/0!
CG43355	NP_001245986.1	0	0	#DIV/0!	ECSIT	NP_649602.2	297	0	0.00
Cog8	NP_609503.2	187	0	0.00	CG2017	NP_649603.3	158	0	0.00
spz4	NP_609504.2	64	0	0.00	side-III	NP_001036682.1	100	0	0.00
Dlg5	NP_001260385.1	784	0	0.00	CG11373	NP_652319.1	279	0	0.00
CG4970	NP_001027253.1	1	0	0.00	Obp83a	NP_001287190.1	105	3	19.31
CG14929	NP_609507.1	0	0	#DIV/0!	Obp83b	NP_524242.2	86	3	23.58
CG14930	NP_609508.1	3	0	0.00	Rm62	NP_524243.2	1121	0	0.00
CG43153	NP_001245989.1	0	0	#DIV/0!	CG10280	NP_649607.1	16	0	0.00
CG31705	NP_609509.2	74	1	9.13	CG11459	NP_649608.1	260	0	0.00
CG31706	NP_723675.1	9	0	0.00	CG15580	NP_649610.1	237	0	0.00
CG34162	NP_001097149.2	1	0	0.00	Or83c	NP_524244.2	84	2	16.09
CG43438	NP_001245990.1	0	0	#DIV/0!	Gasp	NP_001097694.1	167	3	12.14
Mst33A	NP_001245991.1	836	4	3.23	Obp83cd	NP_649612.1	105	5	32.19
Acp33A	NP_523550.3	52	0	0.00	Obp83ef	NP_731042.1	131	5	25.80
spag4	NP_609510.2	223	0	0.00	Obp83g	NP_731043.1	160	3	12.67
kek2	NP_523551.1	264	0	0.00	Vha14-2	NP_001246943.1	272	0	0.00
CG6614	NP_609511.3	134	2	10.09	CG31559	NP_731045.1	45	0	0.00
CG12307	NP_609512.1	756	4	3.58	CG1077	NP_649617.1	74	0	0.00
CG4983	NP_609513.1	859	6	4.72	Osi1	NP_649618.1	99	2	13.65
CG43167	NP_001245992.1	0	0	#DIV/0!	NPFR	NP_001246947.1	254	7	18.63
CG4988	NP_609514.1	856	4	3.16	Osi24	NP_649620.2	33	9	184.34
Vha100-5	NP_609515.1	490	0	0.00	Osi2	NP_649621.1	24	5	140.81
CG42470	NP_001162953.1	0	0	#DIV/0!	Osi3	NP_649622.2	70	6	57.93
CG42471	NP_001162954.1	0	0	#DIV/0!	Osi4	NP_649623.2	18	7	262.85
Sfp33A1	NP_001162955.1	0	0	#DIV/0!	Osi5	NP_649624.1	17	8	318.07
CG14931	NP_001260389.1	4	0	0.00	Osi6	NP_649625.2	127	1	5.32
CG14932	NP_001162956.1	20	1	33.80	Osi7	NP_649626.1	115	2	11.75
Tsp33B	NP_523552.1	371	3	5.47	Osi8	NP_649627.1	98	3	20.69
CG14937	NP_609518.1	68	0	0.00	Osi9	NP_649628.2	123	9	49.46
crol	NP_477244.1	218	1	3.10	Osi10b	NP_001368993.1	22	14	430.12
CycY	NP_609519.1	139	0	0.00	Osi11	NP_649630.1	18	9	337.95
Pde1c	NP_001245994.1	290	0	0.00	Osi12	NP_649631.1	21	8	257.49
esc	NP_477431.1	897	1	0.75	CG15597	NP_649632.2	21	11	354.04
Sfp33A4	NP_001162957.1	0	0	#DIV/0!	CG15594	NP_731062.1	21	10	321.86
Sfp33A2	NP_001162958.1	0	0	#DIV/0!	Osi13	NP_649634.1	18	6	225.30
Sfp33A3	NP_001162959.1	18	0	0.00	Osi14	NP_649635.1	160	8	33.80
CG31704	NP_723689.1	17	0	0.00	Osi15	NP_649636.1	115	4	23.51
CG46426	NP_001285852.1	11	0	0.00	Osi16	NP_731065.1	3	1	225.30
CG42486	NP_001162960.1	0	0	#DIV/0!	CG31560	NP_731067.1	0	0	#DIV/0!
Mal-B1	NP_609522.1	166	0	0.00	Osi17	NP_001262307.1	18	4	150.20
Mal-B2	NP_723693.3	162	0	0.00	Osi18	NP_649639.1	127	1	5.32
CG42751	NP_001188792.1	19	0	0.00	Osi19	NP_649640.1	128	1	5.28
Or33a	NP_523553.1	99	0	0.00	Osi20	NP_649641.1	156	8	34.66
Or33b	NP_523554.1	85	0	0.00	CG17917	NP_649642.1	283	0	0.00
Or33c	NP_523555.1	71	0	0.00	CG10298	NP_649643.1	643	0	0.00
Crys	NP_476906.1	198	0	0.00	CG17919	NP_649644.1	281	0	0.00
CG16964	NP_609526.1	32	0	0.00	SmD2	NP_649645.1	1168	2	1.16
CG16965	NP_609528.3	125	0	0.00	CG18048	NP_649646.3	219	0	0.00
CG6686	NP_609529.2	703	0	0.00	Hr83	NP_649647.1	127	0	0.00
CG34163	NP_001097150.1	14	2	96.56	Pak	NP_001262310.1	608	1	1.11
zuc	NP_609530.1	458	2	2.95	CG42724	NP_001189184.1	616	3	3.29
dgt2	NP_609531.1	160	2	8.45	CG10286	NP_649649.1	339	0	0.00
escl	NP_723702.1	714	2	1.89	CRAT	NP_649650.2	192	0	0.00
Ada1-2	NP_723703.1	76	2	17.79	CG42564	NP_649651.2	60	0	0.00
CG18789	NP_723704.1	126	1	5.36	CG42537	NP_001163530.1	26	0	0.00
Qtzl	NP_723706.1	397	2	3.41	CG1024	NP_001262315.1	39	0	0.00

Ada1-1	NP_723707.1	89	0	0.00	TfIIFalpha	NP_524246.1	549	2	2.46
CG18787	NP_652664.2	126	1	5.36	gzl	NP_001097695.1	197	0	0.00
Nfs1	NP_609533.1	483	2	2.80	Dmtn	NP_001163531.1	153	0	0.00
Hacd1	NP_609534.1	375	0	0.00	gpp	NP_731085.2	473	0	0.00
Rh5	NP_477096.1	422	0	0.00	CG9727	NP_001262317.1	270	0	0.00
Wdr81	NP_609535.1	197	0	0.00	Zif	NP_649657.1	236	0	0.00
dmGlut	NP_620115.2	45	0	0.00	Neurochondri	NP_649658.1	110	0	0.00
CG18788	NP_652665.3	106	0	0.00	CG15186	NP_001246950.1	17	0	0.00
Tom70	NP_609536.1	390	2	3.47	CG15185	NP_649660.1	3	0	0.00
CG6766	NP_609537.1	449	0	0.00	dpr11	NP_788586.1	89	0	0.00
CG6785	NP_609538.2	23	0	0.00	Pif2	NP_731093.2	246	3	8.24
CG6770	NP_609539.1	119	0	0.00	CG1137	NP_649665.2	719	7	6.58
Plzf	NP_609540.2	114	0	0.00	CG2336	NP_731094.3	1055	10	6.41
PLCXD	NP_609541.1	23	0	0.00	CG1138	NP_649667.1	589	6	6.89
JhI-21	NP_609542.1	153	0	0.00	CG31482	NP_731097.1	654	8	8.27
CG34164	NP_001097151.1	748	2	1.81	glob2	NP_001262321.1	324	5	10.43
Rab3-GAP	NP_723718.2	240	1	2.82	sowi	NP_649671.2	1200	8	4.51
firl	NP_001033900.2	77	0	0.00	CG15177	NP_001262323.1	1159	9	5.25
CG14947	NP_609546.1	19	0	0.00	sunz	NP_649673.1	992	10	6.81
prd	NP_723721.1	522	1	1.29	CG31286	NP_731103.1	465	8	11.63
Pex19	NP_609547.2	280	0	0.00	Ass	NP_649674.1	509	2	2.66
CG6712	NP_477479.1	893	2	1.51	Taf1	NP_001246952.1	802	2	1.69
Mt2	NP_001036355.1	667	2	2.03	CG1307	NP_649675.2	344	4	7.86
Ced-12	NP_609548.1	260	0	0.00	twr	NP_649676.1	584	4	4.63
Patsas	NP_001356894.1	213	5	15.87	agt	NP_477366.1	349	2	3.87
ThrRS	NP_723725.1	964	5	3.51	lab	NP_476613.1	119	1	5.68
Rab6	NP_477172.1	618	5	5.47	Edg84A	NP_524247.1	76	0	0.00
Phae1	NP_609550.1	147	3	13.79	Ccp84Ag	NP_649677.1	185	0	0.00
Phae2	NP_609551.2	151	3	13.43	Ccp84Af	NP_649678.1	147	0	0.00
CG17211	NP_609552.1	32	0	0.00	Ccp84Ae	NP_649679.1	215	0	0.00
rho-6	NP_788038.1	113	1	5.98	Ccp84Ad	NP_649680.1	140	0	0.00
Gr33a	NP_525102.4	125	0	0.00	Ccp84Ac	NP_649681.1	271	2	4.99
CG31760	NP_723730.2	157	0	0.00	Ccp84Ab	NP_649682.1	198	0	0.00
CG31861	NP_723731.1	45	0	0.00	Ccp84Aa	NP_649683.1	231	0	0.00
ZnT33D	NP_723732.2	315	1	2.15	pb	NP_996161.1	128	6	31.68
CG17217	NP_609555.1	3	0	0.00	zen2	NP_476794.1	52	2	26.00
CG6583	NP_609556.2	15	2	90.12	CG34297	NP_001097696.1	339	0	0.00
crok	NP_609557.1	85	5	39.76	zen	NP_476793.1	190	5	17.79
atilla	NP_609558.3	69	2	19.59	bcd	NP_476825.1	377	7	12.55
CG31759	NP_723735.2	254	1	2.66	Ama	NP_731114.2	224	5	15.09
bru1	NP_723737.1	298	4	9.07	Dfd	NP_477201.1	217	6	18.69
CG31862	NP_723741.1	25	3	81.11	Scr	NP_001368995.1	214	4	12.63
CG43813	NP_001260409.1	0	0	#DIV/0!	ftz	NP_477498.1	265	4	10.20
bru2	NP_001036356.1	211	4	12.81	Sodh-1	NP_477348.1	521	0	0.00
CG17010	NP_609560.2	1178	1	0.57	CG1979	NP_649685.1	426	3	4.76
Pkd2	NP_609561.2	287	0	0.00	dj	NP_731118.3	764	3	2.65
vir-1	NP_001260412.1	147	0	0.00	djl	NP_649686.2	746	3	2.72
CG6405	NP_609564.1	16	0	0.00	Mst84B	NP_788591.1	487	3	4.16
CG5446	NP_001260413.1	129	0	0.00	CG1105	NP_649688.1	106	0	0.00
CG6388	NP_609566.1	676	1	1.00	CG1965	NP_649689.1	192	0	0.00
CG5435	NP_609567.2	74	0	0.00	Ufl1	NP_649690.1	270	0	0.00
SC35	NP_652612.1	540	2	2.50	CG1943	NP_649691.1	144	0	0.00
eRF3	NP_477259.1	1019	1	0.66	Ref1	NP_651968.1	588	2	2.30
Oatp33Ea	NP_001260417.1	32	0	0.00	Dpck	NP_649692.2	290	0	0.00
CG5421	NP_609569.1	86	0	0.00	Tailor	NP_649693.3	189	0	0.00
Oatp33Eb	NP_609570.1	15	0	0.00	alphaTub84B	NP_476772.1	995	0	0.00
CG5418	NP_609571.1	76	0	0.00	e(y)2b	NP_652310.1	169	0	0.00
bun	NP_525103.2	254	0	0.00	Alh	NP_001097697.2	435	4	6.22
t-cup	NP_723757.1	276	0	0.00	Mlp84B	NP_477122.1	461	0	0.00
nub	NP_001097153.1	442	2	3.06	CG10098	NP_649694.1	134	4	20.18
Ref2	NP_609574.1	392	3	5.17	CG10068	NP_649695.1	50	0	0.00
pdm2	NP_723763.1	236	3	8.59	CG31248	NP_649696.1	0	0	#DIV/0!
Nepl7	NP_609577.2	33	3	61.45	CG31493	NP_731135.2	18	0	0.00
CCT4	NP_609579.1	1300	1	0.52	Fer1	NP_001262334.1	152	0	0.00
CG6153	NP_609580.1	57	0	0.00	CG14610	NP_649698.2	24	3	84.49
IFT43	NP_609581.3	91	0	0.00	CG2656	NP_649699.1	469	3	4.32

PICK1	NP_001285881.1	279	0	0.00	gfzf	NP_001262335.1	263	0	0.00
CG5781	NP_609583.1	513	1	1.32	Sas-4	NP_649701.2	483	0	0.00
CG17036	NP_609584.1	57	0	0.00	MAGE	NP_649702.2	379	0	0.00
CG5776	NP_609585.1	850	1	0.80	sas	NP_731141.1	44	0	0.00
spict	NP_001260421.1	397	3	5.11	CG10055	NP_649703.1	145	0	0.00
CG15484	NP_609587.1	0	0	#DIV/0!	lap	NP_001262339.1	522	4	5.18
CG6180	NP_609588.1	336	0	0.00	CG14609	NP_649704.3	558	2	2.42
CG5787	NP_609589.1	70	0	0.00	CG1288	NP_649705.2	850	3	2.39
Pih1D1	NP_609590.1	309	0	0.00	CG14606	NP_001334690.1	304	2	4.45
MRP	NP_001303319.1	346	2	3.91	CG14605	NP_731145.1	1094	3	1.85
CG15483	NP_609592.1	604	6	6.71	thw	NP_001097699.2	57	1	11.86
kek1	NP_523559.3	265	0	0.00	CG14607	NP_649709.2	1	0	0.00
ACXC	NP_609593.1	820	8	6.59	CG1234	NP_649710.1	783	0	0.00
ACXB	NP_620474.2	440	5	7.68	CG10053	NP_649711.1	60	0	0.00
ACXA	NP_620475.2	1157	6	3.51	CG1227	NP_649712.1	140	1	4.83
ACXE	NP_652601.3	172	7	27.51	CG10050	NP_649713.1	34	0	0.00
CG16800	NP_609594.2	14	0	0.00	Obp84a	NP_001097700.2	127	0	0.00
Vha68-2	NP_652004.2	596	2	2.27	Gld	NP_001350861.1	272	0	0.00
Vha68-3	NP_609595.1	1064	7	4.45	Syt4	NP_477464.1	440	2	3.07
Yip1d1	NP_609596.1	176	0	0.00	rn	NP_001262343.1	210	4	12.87
Vha68-1	NP_523560.2	431	3	4.70	RacGAP84C	NP_476704.1	392	1	1.72
Tor	NP_001260427.1	1298	2	1.04	CG42544	NP_001163539.1	0	0	#DIV/0!
CG9934	NP_609597.1	532	4	5.08	CG10029	NP_649716.1	425	1	1.59
A16	NP_609598.1	16	0	0.00	CG31496	NP_731155.2	210	0	0.00
CG9932	NP_001260428.1	145	0	0.00	nxf4	NP_731156.1	369	1	1.83
Ttc30	NP_001097155.2	116	0	0.00	CG43290	NP_001246961.1	0	0	#DIV/0!
CG43050	NP_001246017.1	0	0	#DIV/0!	CG17944	NP_731158.1	840	6	4.83
CG5122	NP_609601.1	847	1	0.80	Mst84Dd	NP_524253.1	847	3	2.39
CG9928	NP_609602.2	34	0	0.00	Mst84Dc	NP_001097701.1	272	2	4.97
CG16978	NP_652569.2	6	0	0.00	Mst84Db	NP_524255.2	285	3	7.11
Pk34A	NP_609603.1	864	2	1.56	Mst84Da	NP_524256.1	553	2	2.44
kmg	NP_609604.1	105	0	0.00	MstProx	NP_649719.2	285	0	0.00
DnaJ-H	NP_001260431.1	521	2	2.59	CG42650	NP_001189191.1	21	0	0.00
Sirt1	NP_477351.1	1244	2	1.09	Ir84a	NP_649720.2	117	0	0.00
Sfmbt	NP_609606.2	477	0	0.00	wa-cup	NP_649721.2	804	7	5.88
CG43925	NP_001260433.1	0	0	#DIV/0!	CG14598	NP_649722.1	3	0	0.00
CG5439	NP_609607.1	242	0	0.00	CG1142	NP_788592.1	446	0	0.00
CG5287	NP_609608.1	360	1	1.88	alpha-Est10	NP_001246962.1	76	0	0.00
CG31849	NP_723787.1	38	0	0.00	CG42656	NP_001189192.1	0	0	#DIV/0!
Rsph1	NP_609609.1	319	0	0.00	alpha-Est9	NP_524258.2	49	0	0.00
CG16974	NP_609610.2	200	2	6.76	Mics1	NP_649725.2	700	3	2.90
Edem2	NP_609611.1	247	2	5.47	alpha-Est8	NP_524259.2	62	0	0.00
CG16972	NP_609612.2	97	0	0.00	Ubc84D	NP_524260.1	1181	2	1.14
Pect	NP_723789.2	259	1	2.61	alpha-Est7	NP_524261.1	102	1	6.63
CG15482	NP_609614.2	7	1	96.56	alpha-Est6	NP_524262.1	179	0	0.00
kek4	NP_609615.2	182	1	3.71	Rtnl2	NP_524263.2	305	2	4.43
CG9426	NP_609616.1	142	1	4.76	alphaTub84D	NP_524264.1	784	1	0.86
mRF1	NP_609617.1	539	0	0.00	alpha-Est5	NP_524265.3	69	0	0.00
Ski6	NP_609618.2	668	1	1.01	alpha-Est4	NP_524266.1	151	1	4.48
CG16812	NP_609619.1	11	1	61.45	alpha-Est3	NP_524267.2	49	0	0.00
CG15480	NP_609620.1	18	0	0.00	alpha-Est2	NP_524268.3	182	2	7.43
CG16813	NP_609621.1	16	0	0.00	alpha-Est1	NP_524269.3	32	0	0.00
CG16815	NP_609622.2	43	0	0.00	Nlg3	NP_001036685.2	119	1	5.68
Prosalpha6T	NP_609623.1	1275	6	3.18	Nlg1	NP_731172.2	107	1	6.32
Mabi	NP_609624.1	11	0	0.00	CG2616	NP_649731.1	549	0	0.00
CG5867	NP_609625.2	55	1	12.29	CG43254	NP_001246967.1	41	0	0.00
CG5945	NP_609626.1	29	1	23.31	pyd3	NP_649732.1	251	0	0.00
CG16820	NP_609627.1	0	0	#DIV/0!	CG10919	NP_649733.2	162	0	0.00
l(2)k05911	NP_723797.3	70	0	0.00	Sp7	NP_649734.2	188	0	0.00
Naa20B	NP_723798.1	172	1	3.93	CG11286	NP_649735.1	57	0	0.00
CG31730	NP_723799.1	369	1	1.83	CG18747	NP_652650.2	2	0	0.00
CG44085	NP_001097157.1	0	0	#DIV/0!	CG18745	NP_652648.1	156	0	0.00
CG31848	NP_723803.2	5	0	0.00	CG18748	NP_001097702.1	95	0	0.00
Drep4	NP_609631.1	77	0	0.00	CG18746	NP_652649.1	93	0	0.00
Uvrag	NP_609632.1	219	0	0.00	CG10086	NP_649736.3	139	0	0.00
CG31729	NP_609634.1	204	0	0.00	CG2641	NP_649737.1	96	0	0.00

CG16824	NP_001097158.1	82	0	0.00	CG18744	NP_652647.2	255	0	0.00
CG43778	NP_001369129.1	0	0	#DIV/0!	CG3014	NP_649738.1	93	0	0.00
CG43779	NP_001162971.1	0	0	#DIV/0!	CG18268	NP_649739.1	91	0	0.00
CG16825	NP_609636.2	275	0	0.00	CG2993	NP_001262347.1	95	0	0.00
CG16826	NP_609638.1	35	0	0.00	sgll	NP_731186.2	200	3	10.14
CG9395	NP_609639.2	40	0	0.00	CG31473	NP_731188.1	129	2	10.48
CG9377	NP_609640.1	47	0	0.00	CG34384	NP_001246969.1	145	1	4.66
Nnp-1	NP_651976.1	778	0	0.00	CG17816	NP_001097705.1	44	0	0.00
CG6523	NP_609641.1	459	0	0.00	ArgRS-m	NP_649745.3	572	0	0.00
Tap42	NP_723811.1	386	0	0.00	mRpS9	NP_524270.2	685	2	1.97
CG31855	NP_001137822.1	64	0	0.00	wtrw	NP_731193.1	241	0	0.00
ND-B22	NP_609642.1	440	1	1.54	EMC1	NP_001287216.1	248	1	2.73
Bdp1	NP_001285890.1	236	1	2.86	Taf7	NP_649748.1	472	0	0.00
CG6565	NP_609644.1	137	0	0.00	Prat	NP_524271.2	564	2	2.40
CG9302	NP_609645.2	415	1	1.63	CG2846	NP_649749.2	390	3	5.20
beta'COP	NP_524836.2	397	0	0.00	CG2678	NP_649750.1	130	0	0.00
loqs	NP_609646.1	211	0	0.00	CG10445	NP_001287219.1	373	1	1.81
Ing5	NP_723814.1	334	0	0.00	lds	NP_524850.2	538	1	1.26
CG7099	NP_609648.1	134	1	5.04	dsx	NP_001262353.1	319	1	2.12
RpL24	NP_609649.1	831	2	1.63	CD98hc	NP_001262354.2	98	1	6.90
CG16957	NP_609650.1	464	3	4.37	Os-C	NP_001163543.1	55	0	0.00
CG10859	NP_609651.1	413	2	3.27	Sfp84E	NP_001163544.1	0	0	#DIV/0!
CG7110	NP_609652.3	6	0	0.00	ELOVL	NP_649754.1	136	0	0.00
Vm34Ca	NP_476785.1	150	0	0.00	PIG-H	NP_649755.1	92	1	7.35
CG16848	NP_609653.1	305	1	2.22	Kmn2	NP_001163546.2	28	1	24.14
CG16956	NP_609654.1	21	0	0.00	CG2698	NP_001246976.1	118	0	0.00
CG43850	NP_001260444.1	0	0	#DIV/0!	CG2767	NP_649757.1	198	0	0.00
Tehao	NP_477438.1	349	0	0.00	CG11052	NP_652181.1	85	0	0.00
Hacd2	NP_609655.2	279	0	0.00	CG3223	NP_649758.1	1136	2	1.19
Hsp60D	NP_609656.1	1334	4	2.03	CG10435	NP_649759.1	107	0	0.00
CG42809	NP_001188797.1	0	0	#DIV/0!	Pbp95	NP_649760.1	1357	5	2.49
CG42810	NP_609657.2	8	0	0.00	CG2747	NP_001163547.1	246	0	0.00
B4	NP_477325.2	41	0	0.00	ImpE3	NP_524908.1	5	0	0.00
CG33640	NP_001027255.2	0	0	#DIV/0!	CG10903	NP_649762.1	697	2	1.94
CG33641	NP_001027256.1	0	0	#DIV/0!	CG11035	NP_649763.1	155	2	8.72
CG33642	NP_001027257.1	0	0	#DIV/0!	unc-45	NP_524796.1	439	4	6.16
CG33643	NP_001027258.1	0	0	#DIV/0!	CG43060	NP_001246979.1	0	0	#DIV/0!
CG33644	NP_001027259.1	0	0	#DIV/0!	CG43618	NP_001246981.1	0	0	#DIV/0!
CG33645	NP_001027260.3	0	0	#DIV/0!	CG43061	NP_001246982.1	56	0	0.00
CG15638	NP_609660.1	201	2	6.73	mAChR-B	NP_649764.1	117	1	5.78
CG16852	NP_609661.3	0	0	#DIV/0!	CG7910	NP_649765.1	199	2	6.79
CG9263	NP_609662.1	334	5	10.12	CG7900	NP_001097707.1	151	0	0.00
CG16853	NP_609663.2	22	2	61.45	puc	NP_524273.1	602	0	0.00
kuz	NP_001097160.2	499	0	0.00	CG7878	NP_649767.1	548	0	0.00
CG9254	NP_609664.1	572	3	3.54	CG9667	NP_649768.2	616	2	2.19
ics	NP_609665.2	285	0	0.00	Arl8	NP_649769.1	566	1	1.19
CG18507	NP_001260447.1	2	0	0.00	grn	NP_001262366.1	656	1	1.03
DIP-kappa	NP_001285904.1	30	0	0.00	CheA84a	NP_001027150.1	0	0	#DIV/0!
Gpo3	NP_609667.2	1125	4	2.40	CG7800	NP_649770.1	229	2	5.90
CG9014	NP_609668.2	1073	5	3.15	CG18249	NP_001163549.1	166	2	8.14
nur	NP_723832.1	3	0	0.00	DNApol-iota	NP_649772.1	505	2	2.68
TM9SF4	NP_609669.1	208	2	6.50	Ada2b	NP_001027151.1	521	1	1.30
CG43376	NP_001246024.1	0	0	#DIV/0!	CG9636	NP_649774.1	41	0	0.00
CG9008	NP_609670.1	103	1	6.56	CG18749	NP_001287229.1	40	0	0.00
p38b	NP_477361.1	1361	2	0.99	CG15864	NP_652183.2	40	0	0.00
CG16890	NP_609671.1	58	2	23.31	mtg	NP_731221.2	105	0	0.00
Eato	NP_001036360.1	66	0	0.00	RpA-70	NP_524274.1	661	2	2.05
CG16863	NP_001036361.1	44	0	0.00	Mcm2	NP_477121.1	1075	5	3.14
Sec71	NP_609675.2	688	1	0.98	CG9630	NP_649777.1	1072	3	1.89
CAH1	NP_523561.1	149	0	0.00	ato	NP_731223.1	379	0	0.00
Adat1	NP_609676.1	169	0	0.00	Nazo	NP_649778.1	189	0	0.00
CG16865	NP_609677.1	40	0	0.00	CG11672	NP_649779.1	25	0	0.00
CG16888	NP_609678.1	2	0	0.00	CG31464	NP_731226.2	0	0	#DIV/0!
Sos	NP_476597.2	598	1	1.13	CG31463	NP_731227.1	115	0	0.00
b	NP_476788.1	362	2	3.73	CG9626	NP_001262369.1	2	0	0.00
tam	NP_476821.1	667	4	4.05	mRpL1	NP_524275.1	930	4	2.91

Arpc1	NP_476596.1	396	3	5.12	nac	NP_649782.1	124	0	0.00
Orc5	NP_477132.1	736	5	4.59	Sgt1	NP_649783.2	332	0	0.00
DNApol-gamm	NP_001027262.1	42	5	80.46	tex	NP_649784.1	353	2	3.83
RpII33	NP_477419.1	1634	3	1.24	CG11693	NP_001246985.1	0	0	#DIV/0!
mRpS23	NP_723847.1	320	0	0.00	CG11694	NP_649786.1	7	0	0.00
CenG1A	NP_723849.1	323	1	2.09	CG11698	NP_649787.2	2	0	0.00
CG33307	NP_995705.1	54	4	50.07	CG7483	NP_649788.2	1252	2	1.08
CG33306	NP_995706.1	17	0	0.00	Coq2	NP_649789.1	423	2	3.20
CG8997	NP_609681.1	124	4	21.80	Ctr1B	NP_649790.1	207	0	0.00
CG7916	NP_609682.1	113	4	23.93	Poxm	NP_001036687.1	238	0	0.00
CG7953	NP_609683.1	91	4	29.71	Or85a	NP_524277.1	81	0	0.00
CG7968	NP_609684.1	88	3	23.04	CG7443	NP_649791.1	1	0	0.00
Smg5	NP_609685.1	356	0	0.00	COX7A	NP_652184.2	288	1	2.35
Ance	NP_477046.1	177	0	0.00	Arl2	NP_476886.1	189	1	3.58
Ance-2	NP_788042.1	152	0	0.00	CG9601	NP_649792.3	482	3	4.21
Acyp	NP_523563.1	267	1	2.53	COX7AL	NP_649793.1	653	0	0.00
Ance-3	NP_001285917.1	154	1	4.39	DppIII	NP_731237.1	281	0	0.00
Vajk1	NP_001188800.1	112	0	0.00	CG7352	NP_649795.1	131	0	0.00
CG42711	NP_001188801.2	0	0	#DIV/0!	Atg13	NP_649796.1	205	0	0.00
Vajk2	NP_609689.3	144	0	0.00	stck	NP_731241.1	306	0	0.00
Vajk3	NP_609690.1	167	0	0.00	CG9684	NP_649797.2	16	0	0.00
NimA	NP_609691.6	168	2	8.05	CG7963	NP_649798.3	30	0	0.00
NimB1	NP_001260452.1	112	1	6.03	CG45263	NP_001246988.1	2	0	0.00
NimB2	NP_723857.1	128	2	10.56	Obp85a	NP_001334691.1	66	0	0.00
NimB3	NP_001033905.1	120	3	16.90	Or85b	NP_524279.2	117	0	0.00
NimB4	NP_788046.1	124	3	16.35	Or85c	NP_524280.2	106	0	0.00
NimB5	NP_609692.3	76	3	26.68	CG11737	NP_649803.1	50	0	0.00
He	NP_652734.2	110	4	24.58	CG31454	NP_731250.1	21	0	0.00
NimC1	NP_609693.1	217	6	18.69	CG31259	NP_731251.1	23	0	0.00
Cyp28a5	NP_609694.1	151	0	0.00	CG11741	NP_001097709.1	0	0	#DIV/0!
NimC2	NP_788047.1	159	4	17.00	Or85d	NP_524281.1	66	0	0.00
rk	NP_476702.1	370	0	0.00	pch2	NP_524282.4	871	0	0.00
CG42692	NP_001188804.1	0	0	#DIV/0!	mRpS18A	NP_731252.1	528	2	2.56
bgm	NP_524698.1	401	0	0.00	CG31460	NP_731253.1	268	1	2.52
NimC3	NP_524928.2	75	0	0.00	Cenp-C	NP_731254.2	214	1	3.16
hll	NP_609696.1	368	0	0.00	5-HT2B	NP_001262373.1	85	0	0.00
CG18095	NP_001188805.1	253	0	0.00	Or85e	NP_001262374.1	85	0	0.00
NimC4	NP_609698.2	99	1	6.83	Cyp313b1	NP_649807.1	45	0	0.00
nAChRalpha5	NP_001356885.1	177	1	3.82	Mkk4	NP_477353.1	236	0	0.00
CG46301	NP_001334737.1	55	0	0.00	SLIRP2	NP_649808.1	619	3	3.28
CG15293	NP_609702.1	139	1	4.86	Dhod	NP_477224.1	744	2	1.82
CG31769	NP_723866.2	18	1	37.55	CG11753	NP_649809.1	159	0	0.00
CG43332	NP_001246028.1	0	0	#DIV/0!	CG42489	NP_001163552.1	0	0	#DIV/0!
CG43333	NP_788051.1	71	0	0.00	bel	NP_536783.1	1029	1	0.66
Dyrk2	NP_001260455.1	693	1	0.98	p	NP_649810.1	146	0	0.00
wb	NP_723870.1	273	2	4.95	CG8032	NP_649811.1	257	0	0.00
CG32971	NP_001137823.1	112	0	0.00	CG8036	NP_649812.2	792	1	0.85
ms(2)34Fe	NP_609705.1	40	0	0.00	mRpL19	NP_524284.1	769	2	1.76
CG33090	NP_788055.2	352	2	3.84	CG9773	NP_649813.1	135	0	0.00
CG15286	NP_609706.2	1018	0	0.00	CG8043	NP_649814.1	279	1	2.42
Send2	NP_609708.2	25	0	0.00	CG11755	NP_649815.1	1	0	0.00
mTTF	NP_609709.1	78	0	0.00	CG33325	NP_996182.2	0	0	#DIV/0!
l(2)34Fc	NP_609710.1	120	2	11.27	hb	NP_731267.1	357	0	0.00
CG43052	NP_001246030.1	0	0	#DIV/0!	CG8112	NP_731269.2	406	1	1.66
l(2)34Fd	NP_609711.1	609	2	2.22	Cks85A	NP_649817.1	467	0	0.00
Rab14	NP_788056.1	477	1	1.42	TMEM216	NP_649818.1	113	0	0.00
Pgant35A	NP_652069.2	126	3	16.09	CG11760	NP_649819.1	57	0	0.00
spel1	NP_523565.2	864	3	2.35	CG8136	NP_731272.1	706	1	0.96
ppk	NP_477232.1	266	3	7.62	nom	NP_649821.2	91	1	7.43
elB	NP_995712.1	208	3	9.75	ouib	NP_649822.2	81	1	8.34
Pburs	NP_609712.1	66	2	20.48	CG8159	NP_649823.2	75	2	18.02
Cpr35B	NP_609713.1	65	0	0.00	ranshi	NP_649824.1	89	3	22.78
CG15283	NP_652574.3	129	1	5.24	M1BP	NP_649825.1	197	1	3.43
noc	NP_476917.1	71	4	38.08	CG8202	NP_649826.3	38	0	0.00
CG33648	NP_001285933.1	0	0	#DIV/0!	CG9801	NP_649827.2	73	0	0.00
CG4218	NP_609714.1	759	3	2.67	CG34135	NP_001262385.1	0	0	#DIV/0!

CG3473	NP_609715.1	1598	3	1.27	CG8223	NP_649828.1	794	4	3.41
CG42680	NP_001188810.1	0	0	#DIV/0!	CG8236	NP_649830.2	469	1	1.44
osp	NP_723879.3	161	2	8.40	CG13318	NP_649831.1	58	0	0.00
Adh	NP_001027266.1	553	3	3.67	CG34301	NP_001097710.1	6	0	0.00
CG15282	NP_609716.2	0	0	#DIV/0!	Pif1A	NP_001262386.1	58	0	0.00
CG34165	NP_001097161.1	0	0	#DIV/0!	CG33191	NP_788598.1	596	2	2.27
CG42685	NP_609717.5	15	0	0.00	CG33189	NP_788599.1	258	1	2.62
CG31835	NP_723881.1	507	5	6.67	Ir85a	NP_649833.1	36	0	0.00
CG31775	NP_723882.1	3	0	0.00	CCT7	NP_649835.1	1218	2	1.11
CG42585	NP_001162977.1	507	5	6.67	CG9839	NP_649836.1	30	0	0.00
CG42586	NP_001162978.1	3	0	0.00	hng2	NP_649837.1	23	1	29.39
CG34166	NP_001097162.1	49	0	0.00	CG9837	NP_649838.5	0	0	#DIV/0!
CG42681	NP_001188812.1	0	0	#DIV/0!	aqz	NP_788602.1	116	0	0.00
CG42587	NP_001162981.1	0	0	#DIV/0!	CG8369	NP_652187.1	3	0	0.00
CG4691	NP_609719.1	866	7	5.46	IscU	NP_649840.1	505	1	1.34
CSN1a	NP_609720.1	943	8	5.73	CG8379	NP_996185.1	55	1	12.29
CG4701	NP_609721.1	1266	9	4.80	pyd	NP_001246998.1	433	1	1.56
CG4650	NP_609722.2	3	0	0.00	osk	NP_731295.1	413	3	4.91
CG18636	NP_995714.2	3	0	0.00	ScsbetaA	NP_001163558.1	636	3	3.19
CG18420	NP_995715.1	3	0	0.00	CG11964	NP_649847.1	475	4	5.69
CG33308	NP_995716.2	652	7	7.26	ich	NP_649848.2	70	3	28.97
CG33309	NP_995717.2	400	9	15.21	CAHbeta	NP_649849.1	439	0	0.00
CG42682	NP_001188813.2	9	5	375.50	RagA-B	NP_649850.1	331	0	0.00
CG15279	NP_609726.1	86	4	31.44	CG11970	NP_649851.1	130	0	0.00
CG4480	NP_001188814.1	697	10	9.70	E(var)3-9	NP_649852.2	332	1	2.04
CG15278	NP_001285944.2	419	8	12.91	CG11975	NP_649853.1	191	0	0.00
ProtA	NP_477050.1	1030	7	4.59	CG31100	NP_001262395.1	302	0	0.00
ProtB	NP_477051.1	578	4	4.68	CG11977	NP_649855.3	0	0	#DIV/0!
side-II	NP_001014485.3	254	1	2.66	CG11980	NP_649857.1	520	1	1.30
CG3491	NP_609731.1	97	0	0.00	Prosbeta3	NP_649858.1	775	3	2.62
mol	NP_001036362.1	141	2	9.59	Iru	NP_649859.1	363	0	0.00
DCTN5-p25	NP_723893.1	241	4	11.22	CG11983	NP_649860.1	4	0	0.00
l(2)35Be	NP_609735.1	417	4	6.48	Kcmf1	NP_731305.2	269	1	2.51
NC2beta	NP_609736.1	273	0	0.00	Sf3b5	NP_652189.1	759	2	1.78
THG	NP_609737.1	98	4	27.59	CG11986	NP_649862.1	66	1	10.24
Rnmt	NP_523568.2	294	5	11.49	tgo	NP_731308.1	307	1	2.20
GABA-B-R1	NP_523569.3	219	2	6.17	neur	NP_731309.1	440	0	0.00
CG33310	NP_995718.2	80	1	8.45	hyx	NP_649863.1	425	1	1.59
CG31832	NP_723894.2	202	1	3.35	Nmdmc	NP_476929.1	645	2	2.10
yellow-c	NP_523570.3	69	2	19.59	Rel	NP_477094.1	1090	4	2.48
CIAPIN1	NP_524938.1	297	3	6.83	Mst85C	NP_477476.1	13	3	155.98
Su(H)	NP_476868.1	533	5	6.34	Kdm2	NP_649864.2	1152	3	1.76
ck	NP_523571.1	501	2	2.70	beag	NP_649865.1	178	5	18.99
CG33679	NP_001027273.1	0	0	#DIV/0!	Ada	NP_649866.1	426	2	3.17
TfIIS	NP_476967.1	641	0	0.00	pum	NP_731314.1	746	0	0.00
vig	NP_523572.1	316	3	6.42	CG11997	NP_649867.1	0	0	#DIV/0!
solo	NP_001027274.2	842	1	0.80	D1	NP_524286.1	91	0	0.00
CG15270	NP_001246034.1	151	1	4.48	Vps15	NP_649868.1	321	2	4.21
CG15269	NP_609739.2	44	3	46.08	CG8420	NP_649869.1	29	0	0.00
stc	NP_476599.1	165	4	16.39	St2	NP_649870.1	171	0	0.00
CG4168	NP_609740.4	288	3	7.04	CG16734	NP_649872.2	28	0	0.00
Sfp35C	NP_001162982.1	0	0	#DIV/0!	CG16736	NP_649873.1	123	0	0.00
CG43230	NP_001246035.1	0	0	#DIV/0!	Ibf2	NP_649874.1	159	0	0.00
ZnT35C	NP_609741.3	260	4	10.40	Ibf1	NP_649875.1	79	0	0.00
dao	NP_609742.1	282	3	7.19	ATP6AP2	NP_649876.1	291	0	0.00
Pol32	NP_609743.1	228	3	8.89	sage	NP_524287.1	50	0	0.00
Uxt	NP_609744.2	411	3	4.93	TrpRS	NP_524826.1	575	4	4.70
yuri	NP_723903.3	114	0	0.00	Vps16A	NP_649877.1	322	5	10.50
Cul3	NP_523573.1	1039	1	0.65	CG8861	NP_001163567.1	40	4	67.59
UK114	NP_609747.1	177	0	0.00	HP1e	NP_649878.1	318	4	8.50
CG15263	NP_609748.1	1	0	0.00	CG12951	NP_649880.1	65	1	10.40
CG15260	NP_609749.2	744	4	3.63	CG16749	NP_649881.1	301	1	2.25
ms(2)35Ci	NP_723912.1	192	3	10.56	Aduk	NP_731331.1	310	0	0.00
CG15262	NP_609750.1	300	2	4.51	pasi2	NP_649883.1	34	0	0.00
nht	NP_523574.4	291	1	2.32	CG45050	NP_001262407.1	300	4	9.01
esg	NP_476600.1	396	0	0.00	CG43675	NP_001027160.2	0	0	#DIV/0!

CG15258	NP_609751.1	698	3	2.91	CG18473	NP_731339.1	113	0	0.00
wor	NP_476601.1	207	0	0.00	Srr	NP_649886.1	341	0	0.00
CG4161	NP_609753.1	800	5	4.22	FER	NP_731341.1	514	0	0.00
sna	NP_476732.1	394	0	0.00	Or85f	NP_524289.1	35	0	0.00
Tim17b2	NP_524746.2	932	5	3.63	CG34117	NP_001036690.1	55	0	0.00
lace	NP_476614.1	444	0	0.00	RpL34b	NP_649887.1	603	0	0.00
Skadu	NP_001162985.1	15	0	0.00	CG8132	NP_649888.1	256	0	0.00
CG15256	NP_609754.2	435	2	3.11	CG9356	NP_649889.1	171	0	0.00
kek3	NP_523575.1	278	0	0.00	BBIP1	NP_001163568.1	21	0	0.00
CG15255	NP_609755.1	207	1	3.27	betaTub85D	NP_524290.2	1276	2	1.06
Semp1	NP_609756.1	116	0	0.00	Task7	NP_649891.1	110	1	6.14
CG15254	NP_609757.1	69	0	0.00	alpha-Man-IIa	NP_001262413.1	230	1	2.94
CG15253	NP_609758.1	36	0	0.00	ps	NP_001036692.1	285	1	2.37
CG11865	NP_609759.1	84	0	0.00	GstZ1	NP_649894.1	267	0	0.00
Or35a	NP_723916.1	100	0	0.00	GstZ2	NP_731358.1	268	1	2.52
CG7631	NP_609760.1	19	0	0.00	CG16779	NP_649896.2	392	1	1.72
CG18480	NP_609761.1	296	1	2.28	phu	NP_649897.4	566	2	2.39
CG4587	NP_001097163.3	73	0	0.00	RhoL	NP_001247003.1	552	3	3.67
CG18477	NP_609763.1	21	0	0.00	CG8149	NP_649898.1	295	2	4.58
CG18478	NP_609764.1	5	0	0.00	rump	NP_649899.1	709	4	3.81
CG43923	NP_001260469.1	1	0	0.00	Ras85D	NP_476699.1	1388	6	2.92
CG31780	NP_723920.1	21	0	0.00	Rlb1	NP_477099.1	72	2	18.78
CG31827	NP_723923.1	5	0	0.00	mRpL47	NP_788610.1	583	1	1.16
CG43924	NP_001260471.1	1	0	0.00	JHDM2	NP_788611.1	190	1	3.56
CycE	NP_001246037.1	1103	0	0.00	CG8176	NP_001097723.1	604	5	5.60
ND-B17	NP_609765.1	320	0	0.00	by	NP_649901.1	452	2	2.99
Mtr4	NP_524929.1	1038	0	0.00	mura	NP_649902.2	415	1	1.63
Gli	NP_476602.1	168	0	0.00	RnpS1	NP_649903.1	766	3	2.65
CG3793	NP_001246038.1	188	0	0.00	CG9386	NP_649904.2	84	0	0.00
wek	NP_524930.1	193	0	0.00	CG8199	NP_649905.1	334	1	2.02
Ku80	NP_609767.2	235	0	0.00	AP-1mu	NP_649906.1	507	3	4.00
CG31826	NP_001260473.1	17	0	0.00	MBD-like	NP_001262421.1	234	0	0.00
CG43760	NP_001260474.1	0	0	#DIV/0!	CG9393	NP_649908.1	215	0	0.00
CG18109	NP_723933.1	464	1	1.46	Vps45	NP_649909.1	352	2	3.84
CG42876	NP_001188818.1	0	0	#DIV/0!	CG16789	NP_001262423.1	338	2	4.00
CG18518	NP_609768.1	1	0	0.00	CG16790	NP_649911.1	96	3	21.12
CG13243	NP_609769.2	841	2	1.61	CG9396	NP_649912.1	148	1	4.57
Tektin-A	NP_523577.1	836	3	2.43	CG9399	NP_649913.1	167	1	4.05
CG42792	NP_001188820.1	0	0	#DIV/0!	Kap-alpha3	NP_731377.1	610	0	0.00
CG7653	NP_001356886.1	400	1	1.69	Son	NP_649914.1	169	0	0.00
CG44475	NP_001285967.1	0	0	#DIV/0!	CG8301	NP_649915.1	47	0	0.00
Tep1	NP_523578.1	217	1	3.11	CG33654	NP_001027165.1	0	0	#DIV/0!
CG31735	NP_723938.1	720	9	8.45	P58IPK	NP_649916.1	377	1	1.79
CG31823	NP_723939.1	93	0	0.00	bocks	NP_001014611.1	89	0	0.00
CG31821	NP_609771.2	93	0	0.00	CG8312	NP_649918.1	66	0	0.00
beat-Ib	NP_523579.1	45	0	0.00	CG9427	NP_001262429.1	61	1	11.08
CG13244	NP_609772.1	359	3	5.65	CG8319	NP_649920.2	130	0	0.00
CG4793	NP_723941.2	21	1	32.19	Calr	NP_524293.2	775	4	3.49
CG12448	NP_723942.2	295	3	6.87	SpdS	NP_731384.1	622	3	3.26
CG18063	NP_723944.2	202	4	13.38	Scm	NP_731385.1	370	0	0.00
CG10839	NP_609776.1	790	2	1.71	Dh44	NP_649922.2	145	0	0.00
CG34167	NP_001097165.1	9	0	0.00	Fst	NP_524294.2	147	0	0.00
CG13245	NP_001260478.1	818	8	6.61	CG9492	NP_001247008.1	275	0	0.00
CG31820	NP_723947.1	604	7	7.83	Nepl12	NP_649924.1	4	0	0.00
beat-Ic	NP_523580.1	76	0	0.00	p23	NP_649925.1	835	2	1.62
BicC	NP_001285973.1	481	1	1.41	nmdyn-D7	NP_649926.2	524	1	1.29
beat-Ia	NP_001285975.1	104	0	0.00	CG9444	NP_649927.2	128	0	0.00
CG4891	NP_609777.1	460	2	2.94	eca	NP_788616.1	327	3	6.20
CG4892	NP_609778.3	413	3	4.91	CG18542	NP_649928.1	63	0	0.00
CG34168	NP_001097166.1	826	10	8.18	p24-2	NP_731389.2	312	2	4.33
Ca-alpha1D	NP_001260480.2	335	2	4.04	CG32939	NP_788618.1	63	0	0.00
CG31819	NP_723954.1	590	4	4.58	Unc-115a	NP_001287257.1	131	1	5.16
Ca-Ma2d	NP_723955.2	59	3	34.37	trbd	NP_649931.1	268	0	0.00
CG42818	NP_001188821.1	34	3	59.64	dmt	NP_524295.1	99	0	0.00
EndoGI	NP_609781.1	63	0	0.00	hyd	NP_524296.2	660	1	1.02
twe	NP_476633.1	614	1	1.10	FoxP	NP_001247011.1	371	1	1.82

CG4935	NP_609782.2	230	0	0.00	alphaTub85E	NP_524297.1	793	1	0.85
crp	NP_476605.1	346	3	5.86	side-VII	NP_649933.1	31	0	0.00
pkaap	NP_609783.1	60	0	0.00	Pnn	NP_001097727.1	401	0	0.00
CG17329	NP_609784.2	69	1	9.80	CG34409	NP_001097728.2	79	1	8.56
CG13258	NP_609785.1	0	0	#DIV/0!	CG31415	NP_731395.1	59	0	0.00
Cyp303a1	NP_652070.1	135	0	0.00	CG12948	NP_649936.1	7	0	0.00
heix	NP_523581.1	230	0	0.00	PpD3	NP_524946.1	1038	0	0.00
CG17328	NP_001260494.1	102	1	6.63	CG16908	NP_649937.1	155	0	0.00
GMF	NP_609787.2	278	2	4.86	Rpt3R	NP_649938.2	640	1	1.06
c(2)M	NP_609788.1	71	0	0.00	CG8412	NP_649939.1	122	1	5.54
CG5861	NP_609789.1	216	2	6.26	Mpi	NP_649940.1	438	1	1.54
Syx5	NP_599132.2	368	1	1.84	Snap24	NP_524298.1	484	1	1.40
cni	NP_477068.1	265	3	7.65	CG8478	NP_001163576.1	112	0	0.00
fzy	NP_477501.1	1141	1	0.59	MED6	NP_001163577.1	259	1	2.61
cact	NP_476942.1	872	1	0.78	Naa80	NP_649942.1	24	0	0.00
CG4278	NP_609790.1	205	0	0.00	CG9471	NP_001097729.1	127	1	5.32
mRpL4	NP_524939.1	1114	1	0.61	Whamy	NP_001262436.1	250	1	2.70
CG4440	NP_652577.1	58	2	23.31	topi	NP_649945.1	90	0	0.00
CG31817	NP_723962.2	34	1	19.88	RpS29	NP_649946.1	864	1	0.78
CG42266	NP_001137827.1	319	0	0.00	CG12947	NP_001097731.1	26	2	51.99
CG42231	NP_001137828.1	3	0	0.00	MtnA	NP_524299.1	240	2	5.63
CG4455	NP_609791.1	26	0	0.00	CG8500	NP_649948.1	395	4	6.84
CaBP1	NP_609792.1	479	1	1.41	CG12945	NP_001262439.1	1	0	0.00
jhamt	NP_609793.2	155	1	4.36	CG8507	NP_649950.1	192	0	0.00
CG5888	NP_609794.1	206	1	3.28	CG8516	NP_649951.1	32	0	0.00
Idgf1	NP_477258.1	153	1	4.42	CG9467	NP_001369000.1	124	0	0.00
Idgf2	NP_477257.2	93	0	0.00	CG8526	NP_649953.1	281	0	0.00
Idgf3	NP_477256.1	88	1	7.68	FBXO11	NP_649954.1	406	1	1.66
dac	NP_001014486.1	337	1	2.01	CG8534	NP_649955.1	44	0	0.00
Nepl8	NP_609795.2	4	0	0.00	eloF	NP_649956.1	150	0	0.00
Tpr2	NP_523584.1	330	1	2.05	CG16904	NP_649957.1	74	2	18.27
CG5953	NP_609796.1	52	0	0.00	CG9459	NP_649958.2	50	1	13.52
CG31816	NP_723977.1	247	0	0.00	CG9458	NP_731419.1	70	1	9.66
CG5968	NP_609797.1	566	1	1.19	CG42857	NP_001189206.1	1	0	0.00
mtgo	NP_001137829.1	231	0	0.00	CG34302	NP_001097733.2	0	0	#DIV/0!
CG31815	NP_723979.1	0	0	#DIV/0!	Teh1	NP_001262442.1	51	0	0.00
CG4631	NP_609799.1	551	2	2.45	CG46467	NP_001262444.1	99	0	0.00
Trpgamma	NP_001137830.2	148	0	0.00	Art4	NP_649963.1	946	2	1.43
her	NP_476572.1	57	0	0.00	Gr85a	NP_731426.1	90	0	0.00
squ	NP_001246044.1	222	0	0.00	Spn85F	NP_649965.2	52	0	0.00
CG31807	NP_723984.1	1	0	0.00	CG5359	NP_649966.1	171	0	0.00
CG33552	NP_001014488.1	0	0	#DIV/0!	Mical	NP_788620.1	262	0	0.00
grp	NP_477011.1	992	3	2.04	CG31407	NP_731435.1	601	0	0.00
CG31808	NP_001188822.1	17	1	39.76	CG3909	NP_649969.1	519	1	1.30
Cyt-c-d	NP_477164.1	1634	3	1.24	CG11722	NP_649970.1	181	0	0.00
Cyt-c-p	NP_477176.1	1013	1	0.67	mtTFB2	NP_649971.1	76	0	0.00
VhaSFD	NP_523585.2	333	0	0.00	CG12811	NP_649972.1	42	0	0.00
Tsen2	NP_723994.2	205	0	0.00	ohgt	NP_649973.1	90	0	0.00
CG17996	NP_609803.1	190	0	0.00	Fancl	NP_001287266.1	209	4	12.94
Prosbeta4	NP_609804.1	793	1	0.85	Npc2e	NP_731439.2	145	2	9.32
CG17904	NP_609805.1	386	0	0.00	Npc2d	NP_649975.1	272	3	7.45
glu	NP_723996.1	1210	2	1.12	Npc2c	NP_649976.1	81	2	16.69
ChLD3	NP_609806.1	23	0	0.00	CG33630	NP_001027166.1	0	0	#DIV/0!
LSm7	NP_609807.1	810	1	0.83	CG33631	NP_001027167.1	0	0	#DIV/0!
BuGZ	NP_001260513.1	308	0	0.00	CG33783	NP_001027168.1	0	0	#DIV/0!
yellow-b	NP_523586.1	64	0	0.00	CG33784	NP_001027169.1	0	0	#DIV/0!
ppk17	NP_609809.1	58	0	0.00	nerfin-2	NP_001262446.1	108	0	0.00
Mhc	NP_723999.1	920	2	1.47	Alp13	NP_649978.2	58	0	0.00
Cyt-b5-r	NP_477154.1	285	2	4.74	Fmr1	NP_001247017.1	688	2	1.96
CG17928	NP_609810.1	180	2	7.51	CAH7	NP_649979.1	32	0	0.00
Ugt37C2	NP_652629.1	96	0	0.00	gammaSnap2	NP_649980.1	130	0	0.00
Ugt37E1	NP_652628.2	89	0	0.00	ATPsynepsilor	NP_001097736.1	307	0	0.00
Ugt37D1	NP_652627.2	92	1	7.35	knk	NP_649981.1	90	0	0.00
CG13280	NP_609812.1	525	0	0.00	tws	NP_476880.1	934	1	0.72
CG13272	NP_609811.1	22	0	0.00	TAF1B	NP_649982.1	140	0	0.00
mdy	NP_609813.1	567	2	2.38	CG42759	NP_001189207.1	0	0	#DIV/0!

Cse1	NP_523588.2	742	1	0.91	Invadolysin	NP_652072.1	106	1	6.38
AspRS-m	NP_001260520.2	422	5	8.01	CG34303	NP_001356898.1	11	1	61.45
CG13282	NP_609814.1	1	0	0.00	CG6254	NP_649983.2	117	0	0.00
Nepl9	NP_609815.1	0	0	#DIV/0!	Best1	NP_652603.1	193	7	24.51
CG13284	NP_001260522.1	182	1	3.71	CG12814	NP_649984.3	27	11	275.37
CG31810	NP_724023.1	189	1	3.58	Syn	NP_731459.2	492	8	10.99
CG31809	NP_001097168.1	243	1	2.78	Timp	NP_731461.1	178	9	34.17
CG6012	NP_609817.1	192	1	3.52	CG12420	NP_649985.1	60	13	146.45
CG32832	NP_724026.1	712	2	1.90	CG12817	NP_001036701.1	63	13	139.47
CG31743	NP_001097169.1	93	0	0.00	CG34107	NP_001036703.1	188	14	50.33
CG6115	NP_652578.1	50	0	0.00	CG42795	NP_001287271.1	357	15	28.40
tweek	NP_001286002.1	208	1	3.25	CG3999	NP_649989.1	314	17	36.59
CG42556	NP_001162994.1	212	0	0.00	Sirt6	NP_649990.2	599	13	14.67
CG31805	NP_001246052.1	6	0	0.00	pont	NP_652608.1	1268	14	7.46
CG12288	NP_609822.1	1009	0	0.00	Nuak1	NP_001369002.1	162	15	62.58
ApepP	NP_477409.1	386	1	1.75	CG6293	NP_649994.1	218	15	46.51
beat-IIIb	NP_788071.3	41	0	0.00	Bruce	NP_001262462.1	1218	1	0.55
CG31806	NP_724032.1	511	3	3.97	CG12818	NP_649996.1	61	13	144.04
CG6304	NP_609826.1	832	4	3.25	sle	NP_731472.1	91	1	7.43
CG15136	NP_609827.1	514	3	3.94	CG12592	NP_731474.1	70	11	106.21
CG12620	NP_001260525.1	855	5	3.95	CG18545	NP_649998.2	49	9	124.14
CG31784	NP_724035.2	205	3	9.89	CG31406	NP_731476.1	270	9	22.53
CG34106	NP_001188828.1	0	0	#DIV/0!	jumu	NP_524302.1	505	9	12.05
CG34169	NP_001097170.1	0	0	#DIV/0!	Rfx	NP_001097738.2	416	0	0.00
Gr36a	NP_724038.2	90	0	0.00	cwo	NP_524775.1	118	0	0.00
Gr36b	NP_724039.1	85	0	0.00	mthl11	NP_731479.3	139	0	0.00
Gr36c	NP_724040.1	111	0	0.00	CG6325	NP_650000.1	50	0	0.00
CG31750	NP_724041.1	34	0	0.00	Irp-1B	NP_524303.2	494	2	2.74
beat-IIIc	NP_609830.2	85	0	0.00	Rrp46	NP_650001.2	835	1	0.81
CG15140	NP_001097171.2	0	0	#DIV/0!	CG6345	NP_650002.1	330	2	4.10
CG6380	NP_609833.1	1113	5	3.04	Cyp12e1	NP_650003.4	103	0	0.00
CG34170	NP_001097172.1	0	0	#DIV/0!	hth	NP_476578.3	479	1	1.41
CG31804	NP_724045.1	478	5	7.07	CG6465	NP_650004.1	137	1	4.93
Lrch	NP_724046.1	238	2	5.68	CG14688	NP_001097740.1	280	1	2.41
CLIP-190	NP_001368962.1	438	3	4.63	Skeletor	NP_001247027.1	56	0	0.00
Rpb11	NP_609836.1	1204	1	0.56	pug	NP_731489.2	591	1	1.14
CG15141	NP_609837.1	224	0	0.00	CG14683	NP_001369010.1	174	4	15.54
Rsph9	NP_724049.1	378	2	3.58	CG31391	NP_731491.2	183	2	7.39
Dif	NP_523589.2	276	0	0.00	CG31467	NP_731492.1	1	0	0.00
CG5043	NP_609838.1	658	3	3.08	CG14684	NP_650007.1	3	0	0.00
CG33928	NP_001027276.1	0	0	#DIV/0!	CG31278	NP_731494.1	285	1	2.37
dl	NP_724052.1	248	0	0.00	CG31373	NP_731495.1	337	1	2.01
CG5050	NP_609839.2	546	3	3.71	CG14689	NP_650009.1	767	2	1.76
SPH93	NP_609840.1	119	1	5.68	CG4073	NP_650010.1	549	3	3.69
CG18563	NP_609841.2	48	1	14.08	tomboy20	NP_650011.1	1265	3	1.60
BicD	NP_001260531.1	319	3	6.36	TkR86C	NP_524304.2	302	0	0.00
Sgt	NP_609842.1	698	3	2.91	CG14691	NP_650012.2	12	0	0.00
cass	NP_477454.1	244	4	11.08	CG31272	NP_650013.3	427	1	1.58
fws	NP_523590.1	245	4	11.04	MED7	NP_731500.1	520	1	1.30
CG5110	NP_609843.1	273	4	9.90	Cap-H2	NP_650014.2	243	1	2.78
GCS2beta	NP_609844.1	301	0	0.00	TfIIFbeta	NP_524305.2	503	6	8.06
CG5131	NP_609845.1	259	0	0.00	CG45076	NP_650015.3	92	0	0.00
CG43221	NP_001334741.1	1	0	0.00	Desi	NP_650016.1	50	0	0.00
CG43220	NP_001260533.1	0	0	#DIV/0!	CG42633	NP_001163583.1	0	0	#DIV/0!
qua	NP_724060.1	462	0	0.00	mRpL37	NP_524306.3	220	0	0.00
CG15142	NP_609846.1	28	0	0.00	Rbp1	NP_524307.1	495	2	2.73
mEFTs	NP_609847.1	662	1	1.02	Art1	NP_650017.1	1163	3	1.74
Dhc36C	NP_001369131.1	423	2	3.20	Mcm5	NP_524308.2	1393	4	1.94
CG15143	NP_609848.1	109	0	0.00	CG14687	NP_650018.1	94	1	7.19
CG15144	NP_609849.2	148	0	0.00	CG14692	NP_650019.6	359	2	3.77
CG15145	NP_609850.2	165	1	4.10	CG14693	NP_650020.2	585	0	0.00
CG7094	NP_609851.2	969	4	2.79	TTLL15	NP_650021.1	48	0	0.00
Oli	NP_523592.1	148	2	9.13	Cad86C	NP_001247033.1	96	0	0.00
CG6870	NP_609852.1	375	2	3.60	CG42394	NP_001138035.1	1	0	0.00
Sytalpha	NP_001188832.1	92	2	14.69	CG4565	NP_001097743.1	429	2	3.15
CG15147	NP_609854.1	553	1	1.22	CG6567	NP_001097742.1	160	0	0.00

CG43054	NP_001246068.1	0	0	#DIV/0!	CG4511	NP_650026.1	342	1	1.98
CadN	NP_724068.1	467	0	0.00	CG4570	NP_650027.1	172	1	3.93
CG43231	NP_001246069.1	0	0	#DIV/0!	CG14694	NP_650028.1	58	0	0.00
CadN2	NP_001036368.2	174	0	0.00	CG6574	NP_650029.1	71	3	28.56
CG42830	NP_001188834.1	79	2	17.11	SelR	NP_731522.1	406	6	9.99
CG43271	NP_001246070.1	0	0	#DIV/0!	Tsp86D	NP_001262468.1	60	3	33.80
btv	NP_001036369.3	368	5	9.18	Fdh	NP_524310.1	523	4	5.17
CG5674	NP_609856.4	28	5	120.70	CG4596	NP_650031.1	27	0	0.00
CG5681	NP_609858.1	6	0	0.00	Sodh-2	NP_524311.1	504	1	1.34
Prosbeta5R2	NP_724081.1	640	1	1.06	CG14695	NP_650032.1	212	2	6.38
CG5693	NP_652582.1	477	8	11.34	Tpc1	NP_650034.1	139	0	0.00
CG42659	NP_001188835.1	304	3	6.67	CG14696	NP_650035.1	136	0	0.00
CG42634	NP_001163002.1	0	0	#DIV/0!	Leash	NP_650037.2	125	2	10.81
CG31740	NP_724084.1	499	7	9.48	Adk3	NP_524312.1	615	3	3.30
elfless	NP_001163003.1	421	8	12.84	CG6621	NP_001189210.1	70	0	0.00
rdo	NP_001286024.1	276	6	14.69	Tengl4	NP_650038.2	786	2	1.72
Arr1	NP_476681.1	476	4	5.68	side-VI	NP_001036705.1	101	0	0.00
ninaD	NP_724087.1	259	2	5.22	CG43449	NP_001247038.1	0	0	#DIV/0!
CG31741	NP_724088.2	213	2	6.35	CG6629	NP_650041.1	1149	2	1.18
CG15153	NP_609863.1	3	0	0.00	Ugt35C1	NP_001097744.1	165	0	0.00
Slc25A46b	NP_609864.1	914	4	2.96	mAcon2	NP_650042.2	1184	3	1.71
CG15152	NP_609865.1	55	0	0.00	CheA86a	NP_001027174.1	13	0	0.00
CG5758	NP_609866.1	16	0	0.00	Ugt302K1	NP_652620.1	125	0	0.00
CG31785	NP_724093.2	0	0	#DIV/0!	Ugt302E1	NP_652625.2	114	0	0.00
kel	NP_476589.4	349	0	0.00	Ugt302C1	NP_652626.1	77	1	8.78
Socs36E	NP_523593.5	373	0	0.00	CG4757	NP_650043.1	129	0	0.00
CG17681	NP_724098.2	20	0	0.00	Ugt35E1	NP_652622.3	105	0	0.00
CG15155	NP_609868.1	32	0	0.00	Ugt35E2	NP_652623.2	28	0	0.00
CG5783	NP_609869.1	8	0	0.00	Ugt35B1	NP_524313.2	158	0	0.00
JMJD4	NP_609870.1	74	0	0.00	Ugt35A1	NP_524314.2	120	1	5.63
CG42750	NP_001286032.1	641	0	0.00	Ugt304A1	NP_652619.1	91	0	0.00
Ptp36E	NP_609872.1	224	0	0.00	Ugt303A1	NP_652621.1	105	1	6.44
CG31802	NP_724103.1	1246	3	1.63	CG18577	NP_650044.1	0	0	#DIV/0!
CG31788	NP_724104.1	571	3	3.55	cu	NP_731540.2	357	0	0.00
CG31787	NP_724105.1	798	3	2.54	SdhC	NP_001262472.1	546	0	0.00
CG43362	NP_001246076.1	0	0	#DIV/0!	Tctp	NP_650048.1	614	2	2.20
Sfp36F	NP_001163004.1	0	0	#DIV/0!	ZnT86D	NP_650049.1	302	0	0.00
CG5790	NP_609876.2	1256	4	2.15	RpS25	NP_524315.2	710	2	1.90
CG43354	NP_001246078.1	0	0	#DIV/0!	CG4820	NP_001097748.1	67	0	0.00
Fas3	NP_001286038.1	265	0	0.00	CG6689	NP_650051.1	63	0	0.00
Acp36DE	NP_523594.1	406	1	1.66	CG6693	NP_650052.1	407	2	3.32
CG34171	NP_001097175.2	3	0	0.00	RpL3	NP_524316.1	1102	2	1.23
RpS26	NP_523595.1	641	0	0.00	scpr-C	NP_650053.1	378	0	0.00
ncm	NP_609877.2	798	1	0.85	CG17726	NP_650054.2	143	0	0.00
bsf	NP_523596.2	359	1	1.88	GCC88	NP_650055.1	158	0	0.00
Ntf-2r	NP_609878.1	418	0	0.00	CG17187	NP_650056.1	282	2	4.79
CG10283	NP_001163006.1	108	0	0.00	CG14701	NP_650057.1	152	1	4.45
grnd	NP_609880.2	35	0	0.00	Arfip	NP_650058.1	217	0	0.00
kon	NP_609881.3	187	0	0.00	CG17721	NP_001247041.1	3	0	0.00
Ugt301D1	NP_609882.1	105	0	0.00	CG31441	NP_731558.1	57	0	0.00
CG10211	NP_609883.1	270	0	0.00	CG31388	NP_650060.1	35	0	0.00
Pde11	NP_001097177.1	441	1	1.53	scpr-B	NP_650061.1	60	0	0.00
CG15160	NP_001286047.1	314	1	2.15	scpr-A	NP_650062.1	58	0	0.00
amos	NP_477446.1	383	0	0.00	CG17734	NP_650063.1	164	0	0.00
CG10413	NP_609887.1	122	0	0.00	CG5214	NP_650064.1	550	3	3.69
CG31789	NP_724118.1	16	0	0.00	KP78a	NP_650065.1	91	0	0.00
CG10333	NP_609888.2	708	1	0.95	pros	NP_001247045.1	223	1	3.03
Atac2	NP_609889.1	332	2	4.07	mRpL40	NP_524318.1	565	2	2.39
IFT46	NP_609890.2	154	0	0.00	mgr	NP_650067.1	580	2	2.33
MESR3	NP_001260541.1	55	0	0.00	Irbp	NP_536773.1	875	2	1.54
Cyp310a1	NP_609891.1	75	0	0.00	Ranbp9	NP_650068.1	391	1	1.73
CG43338	NP_001246080.1	50	0	0.00	CG6723	NP_650069.2	51	0	0.00
CG43339	NP_001163009.2	0	0	#DIV/0!	ninaG	NP_001247047.1	25	0	0.00
Mst36Fb	NP_001163010.1	21	0	0.00	CG5270	NP_731570.2	147	0	0.00
Mst36Fa	NP_724121.3	0	0	#DIV/0!	Dtd	NP_650072.2	218	0	0.00
CG31751	NP_001334743.1	75	1	9.01	RpL24-like	NP_650073.1	828	1	0.82

tos	NP_477145.1	1059	4	2.55	CG5276	NP_650074.1	268	1	2.52
msl-1	NP_476896.1	208	1	3.25	Exd2	NP_650075.1	229	1	2.95
CG10336	NP_609895.2	511	2	2.65	CG5281	NP_650076.1	96	0	0.00
CG10383	NP_609896.1	154	0	0.00	sals	NP_001247050.1	56	0	0.00
CG10338	NP_609897.2	13	0	0.00	PGRP-LB	NP_001247052.1	230	0	0.00
CG10341	NP_609898.1	339	1	1.99	CG17230	NP_650077.1	70	0	0.00
CG10376	NP_609899.1	512	2	2.64	dpr5	NP_650080.3	29	0	0.00
CG10343	NP_609900.1	152	0	0.00	dpr4	NP_001014616.2	22	0	0.00
Jwa	NP_609901.5	202	0	0.00	CG14708	NP_650082.1	169	0	0.00
Faf2	NP_477388.1	398	0	0.00	CG10898	NP_650083.1	34	0	0.00
Grip71	NP_609902.3	265	2	5.10	fabp	NP_001027175.1	438	1	1.54
Irk3	NP_609903.2	101	0	0.00	Mrp4	NP_650086.2	345	1	1.96
CG10348	NP_609904.2	124	1	5.45	CG6790	NP_650087.2	772	0	0.00
CG42752	NP_001188839.1	0	0	#DIV/0!	CG5342	NP_650088.2	107	0	0.00
CG15167	NP_609906.1	367	0	0.00	wkd	NP_650089.1	186	0	0.00
ham	NP_001260545.2	175	1	3.86	CG6791	NP_731594.1	51	0	0.00
CG43814	NP_001260546.1	0	0	#DIV/0!	Jupiter	NP_001262475.1	58	1	11.65
CG10570	NP_652585.1	24	1	28.16	CG14710	NP_650092.4	122	0	0.00
CG17325	NP_001137837.1	71	1	9.52	CG6808	NP_731602.1	39	0	0.00
Ugt36F1	NP_609909.1	97	0	0.00	CG14711	NP_650094.1	99	0	0.00
Ugt36D1	NP_609910.1	105	0	0.00	CG6813	NP_001163590.1	34	0	0.00
Ugt36E1	NP_609911.1	98	0	0.00	CG18764	NP_652712.2	51	0	0.00
CG17597	NP_609912.1	231	0	0.00	CG14712	NP_650096.1	58	0	0.00
ScpX	NP_524715.2	407	0	0.00	Csk	NP_650097.2	552	2	2.45
CG10600	NP_001260547.1	81	0	0.00	Elp1	NP_001262478.1	506	0	0.00
CG31752	NP_724136.3	0	0	#DIV/0!	CG42327	NP_001036706.2	280	1	2.41
CG33120	NP_788073.1	0	0	#DIV/0!	CG14715	NP_650101.1	741	2	1.82
CG17321	NP_609915.1	4	0	0.00	CG18476	NP_731615.1	34	0	0.00
CG10602	NP_609916.5	364	1	1.86	Taf12	NP_524320.1	320	1	2.11
mRpL13	NP_523598.2	1059	2	1.28	Ho	NP_524321.1	484	0	0.00
tup	NP_476775.1	355	1	1.90	CG6830	NP_650103.1	49	0	0.00
ssp3	NP_001286069.1	37	1	18.27	CG6834	NP_650104.2	59	1	11.46
CG10428	NP_609918.1	339	0	0.00	CG6908	NP_650105.1	63	1	10.73
Nedd8	NP_609919.1	1544	2	0.88	CG18765	NP_001097750.1	121	0	0.00
CG10621	NP_609920.1	215	0	0.00	Fer3	NP_524322.1	57	0	0.00
CG10623	NP_609921.1	205	0	0.00	CG14717	NP_650106.1	348	2	3.88
Nak	NP_477165.1	620	2	2.18	CG14718	NP_650107.1	1192	3	1.70
Tango6	NP_609922.2	163	1	4.15	I-t	NP_524323.2	704	2	1.92
L2HGDH	NP_609923.2	266	0	0.00	CG14720	NP_650108.2	0	0	#DIV/0!
CG10431	NP_609924.2	136	0	0.00	CG12594	NP_650109.1	39	0	0.00
Swip-1	NP_609925.1	159	0	0.00	CG14721	NP_650110.3	194	0	0.00
EMC5	NP_609926.1	167	2	8.09	Blm	NP_524319.2	600	0	0.00
CG15170	NP_001286080.1	2	0	0.00	CG14722	NP_650111.3	326	1	2.07
CG15169	NP_609928.1	1	0	0.00	CG6923	NP_001262484.1	433	1	1.56
CG10650	NP_609929.2	26	0	0.00	CG43062	NP_001247057.1	0	0	#DIV/0!
CG31792	NP_724148.2	264	2	5.12	Lk6	NP_651986.2	184	0	0.00
CG31793	NP_609930.4	355	2	3.81	l(3)neo38	NP_001262485.1	137	1	4.93
RpL30	NP_524687.1	801	1	0.84	prd1	NP_001262486.1	69	2	19.59
robl37BC	NP_609931.1	188	0	0.00	HisCl1	NP_731632.1	104	1	6.50
CG15172	NP_609932.1	372	0	0.00	Sbf	NP_731633.2	241	2	5.61
Sidpn	NP_523599.1	79	1	8.56	ClC-a	NP_001097751.2	189	0	0.00
hook	NP_476573.1	102	0	0.00	glo	NP_650120.1	439	0	0.00
CG31800	NP_724152.1	23	0	0.00	COX5A	NP_477217.1	575	0	0.00
mib2	NP_609933.1	1581	5	2.14	Kyat	NP_650121.1	560	0	0.00
Catsup	NP_524931.1	272	4	9.94	CG6959	NP_001138037.1	173	2	7.81
Ttc19	NP_609934.3	422	0	0.00	sad	NP_650123.1	111	0	0.00
Acn	NP_609935.1	308	1	2.19	CG6962	NP_650124.1	221	2	6.12
CG10470	NP_609936.1	346	4	7.81	CG31368	NP_996198.2	685	3	2.96
l(2)37Bb	NP_536791.1	138	4	19.59	mthl5	NP_650126.2	126	3	16.09
Rpn3	NP_477300.1	811	5	4.17	Dnali1	NP_650128.1	694	0	0.00
CG10492	NP_001097179.1	37	1	18.27	Cad87A	NP_731649.2	102	2	13.25
Phlpp	NP_609938.1	561	1	1.20	CG34307	NP_001097753.2	0	0	#DIV/0!
CG10495	NP_609939.1	430	0	0.00	CG14731	NP_650130.1	0	0	#DIV/0!
CG17343	NP_609940.1	2	0	0.00	CG31211	NP_788641.1	252	4	10.73
CG10702	NP_724157.1	431	0	0.00	Hsp70Aa	NP_731651.1	1546	2	0.87
CG17572	NP_609941.2	76	2	17.79	Hsp70Ab	NP_524798.2	1538	2	0.88

CG10700	NP_609942.1	301	0	0.00	CG3281	NP_650132.1	92	5	36.73
Lim3	NP_001260559.1	181	0	0.00	aurA	NP_476749.1	1051	1	0.64
CG17344	NP_001260560.1	845	0	0.00	CG12213	NP_650133.2	63	0	0.00
amd	NP_724162.1	315	5	10.73	GC1	NP_650134.1	165	0	0.00
CG10561	NP_476608.1	339	3	5.98	GC2	NP_731657.2	1009	0	0.00
Ddc	NP_724163.1	504	2	2.68	Tango9	NP_650136.1	150	0	0.00
l(2)37Cc	NP_724165.1	688	2	1.96	CG10005	NP_650137.3	29	0	0.00
l(2)37Cb	NP_609946.1	772	1	0.88	CG18547	NP_650138.1	100	0	0.00
l(2)37Cd	NP_609947.1	284	2	4.76	CG12224	NP_650139.3	86	1	7.86
AsnRS	NP_609948.1	499	1	1.35	CG3397	NP_650140.1	141	1	4.79
DCTN6-p27	NP_609949.1	255	4	10.60	mfas	NP_788643.1	147	2	9.20
l(2)37Cg	NP_001260562.1	870	2	1.55	Tk	NP_650141.2	180	0	0.00
brat	NP_001188842.2	689	1	0.98	Ect3	NP_650142.1	384	1	1.76
CG17568	NP_609951.2	94	0	0.00	KLHL18	NP_650143.1	136	0	0.00
CG42688	NP_001188843.1	1	0	0.00	GCC185	NP_001097755.1	513	0	0.00
gammaTub37(	NP_476922.1	752	0	0.00	DCAF12	NP_650145.1	79	1	8.56
pigeon	NP_476946.1	36	0	0.00	Spt3	NP_650146.1	268	0	0.00
drl	NP_001260566.1	261	1	2.59	CG42504	NP_001163593.1	1	0	0.00
CG31797	NP_724173.1	108	0	0.00	ssp5	NP_650149.1	846	4	3.20
CG17564	NP_609954.1	696	2	1.94	CG14736	NP_731666.2	415	3	4.89
CG10750	NP_001286084.1	579	2	2.33	CG14739	NP_650151.1	1083	5	3.12
dnt	NP_477341.2	225	1	3.00	CG14740	NP_650152.1	1651	5	2.05
CG13086	NP_609956.1	24	0	0.00	ATP8B	NP_001287294.1	393	2	3.44
CG17349	NP_609957.2	983	3	2.06	dpr17	NP_731670.1	149	2	9.07
CG17350	NP_609958.1	18	1	37.55	CG4702	NP_650156.1	95	1	7.11
fon	NP_724177.1	338	0	0.00	dpr15	NP_731672.1	64	0	0.00
CG17549	NP_724178.1	18	0	0.00	Cpn	NP_731673.1	116	0	0.00
CG17544	NP_609961.2	224	0	0.00	eIF3d2	NP_731675.1	1133	5	2.98
CG31798	NP_724182.1	39	0	0.00	CG43450	NP_001247073.1	0	0	#DIV/0!
Pax	NP_724183.1	584	1	1.16	CG3809	NP_731676.2	1145	6	3.54
Lectin-galC1	NP_477200.1	146	1	4.63	CG4830	NP_650160.1	190	1	3.56
lectin-37Da	NP_001188846.1	82	0	0.00	CG14742	NP_001247074.1	3	0	0.00
lectin-37Db	NP_001188847.1	25	1	27.04	Cog1	NP_650162.1	216	1	3.13
Alp12	NP_609963.1	96	0	0.00	CG4860	NP_650163.2	196	1	3.45
CG13085	NP_609964.1	79	0	0.00	Rsbp15	NP_650164.1	537	3	3.78
RtcB	NP_609965.1	231	1	2.93	CG17404	NP_650165.2	13	0	0.00
Rab9	NP_609966.1	460	0	0.00	CG12256	NP_650166.2	57	0	0.00
CG10237	NP_609967.1	71	4	38.08	CG3916	NP_650167.1	4	0	0.00
Hs2st	NP_477339.1	97	4	27.87	Pglym87	NP_652041.2	1056	3	1.92
RanGAP	NP_476712.1	708	4	3.82	Cyp313a5	NP_650168.1	39	0	0.00
Top2	NP_476760.1	1733	6	2.34	Cyp313a2	NP_650169.3	102	0	0.00
CG10026	NP_609968.3	76	4	35.57	Cyp313a3	NP_650170.2	76	0	0.00
tj	NP_001260582.1	308	0	0.00	CG3942	NP_650171.2	828	3	2.45
CG10195	NP_609970.4	254	0	0.00	CG17738	NP_650172.1	5	0	0.00
CG13084	NP_609971.1	117	0	0.00	Sfp87B	NP_001163595.1	11	0	0.00
CG13083	NP_995727.1	69	0	0.00	svp	NP_001369011.1	417	1	1.62
CG10194	NP_609973.1	263	0	0.00	CG4066	NP_650174.1	22	0	0.00
CG18094	NP_001260584.1	253	0	0.00	CG10013	NP_650175.2	43	0	0.00
CG10189	NP_609975.1	262	0	0.00	CG10038	NP_650176.3	53	1	12.75
swm	NP_001260586.1	176	1	3.84	CG10041	NP_650177.2	4	0	0.00
Top3alpha	NP_523602.2	745	1	0.91	MBD-R2	NP_731688.2	735	0	0.00
cyst	NP_001260588.1	336	0	0.00	CG4115	NP_650179.1	124	0	0.00
Hasp	NP_609978.4	216	0	0.00	Tim17a1	NP_650180.1	677	1	1.00
CG10132	NP_609979.2	232	0	0.00	GstD10	NP_652713.1	399	0	0.00
CG10166	NP_609980.1	486	0	0.00	GstD9	NP_650181.1	306	0	0.00
CG10137	NP_609981.1	81	0	0.00	GstD1	NP_524326.1	442	0	0.00
CG10165	NP_609982.1	89	1	7.59	GstD2	NP_524912.1	304	0	0.00
CG33116	NP_788074.1	179	0	0.00	GstD3	NP_788656.1	294	0	0.00
CG31697	NP_724209.1	146	0	0.00	GstD4	NP_524913.1	262	0	0.00
CG13082	NP_609984.2	9	0	0.00	GstD5	NP_524914.3	359	0	0.00
CG13081	NP_609985.1	0	0	#DIV/0!	GstD6	NP_524915.1	301	0	0.00
ref(2)P	NP_476700.1	685	1	0.99	GstD7	NP_525114.1	265	0	0.00
CG10337	NP_609986.1	19	0	0.00	GstD8	NP_524916.1	318	0	0.00
Tep4	NP_001260590.1	203	0	0.00	CG10035	NP_650182.1	69	0	0.00
CG13078	NP_609989.1	5	0	0.00	GstD11	NP_001138040.1	215	0	0.00
CG13077	NP_609990.1	5	0	0.00	CG34402	NP_001097756.1	244	0	0.00

spi	NP_476909.2	196	0	0.00	CG33098	NP_788657.1	230	1	2.94
msb1l	NP_609991.1	143	0	0.00	CG10096	NP_001027183.1	157	0	0.00
CG10268	NP_609992.1	13	0	0.00	DNAlig3	NP_650187.2	574	0	0.00
Hakai	NP_788075.2	278	0	0.00	Cyp9f2	NP_650189.1	215	0	0.00
PCNA2	NP_609994.1	903	2	1.50	Sccpdh2	NP_650190.1	95	0	0.00
CG10366	NP_609995.1	65	0	0.00	CG5196	NP_650191.1	71	0	0.00
scw	NP_524863.3	203	0	0.00	Dip-C	NP_650192.1	330	0	0.00
CG10462	NP_001246096.1	92	0	0.00	pps	NP_001247075.1	307	2	4.40
Nf-YB	NP_609997.1	670	1	1.01	Scgbeta	NP_524327.1	80	1	8.45
CG10631	NP_609998.1	85	0	0.00	CG17202	NP_650194.1	83	1	8.14
CG10628	NP_609999.2	378	2	3.58	Pp1-87B	NP_524937.1	1081	4	2.50
CG10463	NP_610000.1	515	1	1.31	NANS	NP_650195.1	241	0	0.00
fbp	NP_724223.4	661	0	0.00	CG5641	NP_650196.1	629	2	2.15
bsh	NP_477350.2	276	0	0.00	CG5245	NP_650197.1	114	0	0.00
CdGAPr	NP_001163018.1	203	0	0.00	Prosalpha2	NP_524328.1	981	3	2.07
CG10466	NP_610003.1	243	0	0.00	Aos1	NP_650198.1	962	3	2.11
fs(2)ltoPP43	NP_524940.1	120	0	0.00	Srlp	NP_650199.1	443	2	3.05
sick	NP_001260603.1	240	0	0.00	Desat2	NP_650201.1	470	2	2.88
CG13962	NP_610004.1	1	0	0.00	CG17207	NP_001369012.1	162	1	4.17
CG10481	NP_995731.1	47	0	0.00	Desat1	NP_652731.1	531	6	7.64
TotF	NP_536780.3	29	0	0.00	vrs	NP_650202.1	707	1	0.96
Victoria	NP_788077.2	18	0	0.00	CG18549	NP_650203.3	99	0	0.00
COX4	NP_610013.1	579	0	0.00	CG5509	NP_650204.1	131	1	5.16
CG34051	NP_001188852.1	4	1	168.98	trus	NP_650205.1	278	0	0.00
CG42866	NP_001188853.1	0	0	#DIV/0!	CG5961	NP_650206.1	361	0	0.00
CG13965	NP_610014.1	22	2	61.45	CG12267	NP_650207.1	486	5	6.95
CG16772	NP_610015.2	68	1	9.94	CG5608	NP_650208.1	196	0	0.00
CG10680	NP_610016.1	15	0	0.00	Hsp70Ba	NP_731716.1	1478	4	1.83
OS9	NP_652666.2	345	1	1.96	Hsp70Bbb	NP_788663.1	1458	2	0.93
Hf	NP_788078.1	58	1	11.65	Hsp70Bb	NP_524927.2	1478	3	1.37
CG43861	NP_001260615.1	15	0	0.00	Hsp70Bc	NP_650209.1	1478	2	0.91
AANATL3	NP_610018.1	118	0	0.00	Octbeta3R	NP_001034043.2	195	1	3.47
sNPF	NP_724239.1	252	0	0.00	Hug	NP_524329.1	126	0	0.00
barr	NP_477106.1	747	2	1.81	Octbeta2R	NP_001163596.2	219	1	3.09
lok	NP_477219.1	1423	4	1.90	Vha55	NP_476908.1	716	5	4.72
vls	NP_610019.2	1045	4	2.59	Snx3	NP_650214.1	416	0	0.00
bwa	NP_001260616.1	329	0	0.00	Not10	NP_650215.1	106	0	0.00
CG10730	NP_610021.3	2	0	0.00	CG18530	NP_652714.2	64	0	0.00
pr	NP_724244.1	222	0	0.00	CG11598	NP_001036710.1	70	0	0.00
neb	NP_476817.1	456	4	5.93	CG11600	NP_650217.2	35	0	0.00
fok	NP_001260617.1	4	0	0.00	CG11608	NP_650218.3	73	0	0.00
CG10747	NP_610022.3	78	0	0.00	CG6753	NP_650219.2	36	0	0.00
Pyroxd1	NP_610023.1	180	0	0.00	Dtg	NP_650220.2	191	0	0.00
Taf13	NP_610024.1	385	4	7.02	CG6225	NP_001097759.1	302	1	2.24
nesd	NP_610025.1	221	3	9.18	CG14395	NP_650222.2	0	0	#DIV/0!
mRpS18B	NP_523606.1	360	2	3.76	Gnmt	NP_650223.1	185	0	0.00
Kua	NP_610026.1	85	0	0.00	CG43630	NP_001247079.1	0	0	#DIV/0!
CG13970	NP_610027.1	0	0	#DIV/0!	mbo	NP_524330.2	285	0	0.00
CG10651	NP_610029.2	18	0	0.00	Cyp313a4	NP_650224.3	99	0	0.00
CG17571	NP_610109.1	317	0	0.00	kar	NP_652025.2	108	1	6.26
CG17570	NP_610110.1	63	1	10.73	Su(fu)	NP_536750.2	302	1	2.24
CG12617	NP_652589.1	757	0	0.00	Arp1	NP_524331.2	1104	0	0.00
spir	NP_724254.1	264	1	2.56	CG31347	NP_731735.1	1	0	0.00
La	NP_477014.1	769	1	0.88	CG14391	NP_650225.2	619	0	0.00
RtGEF	NP_001097185.2	366	1	1.85	NijC	NP_001097760.1	65	0	0.00
Ugt37A1	NP_523607.2	21	0	0.00	Past1	NP_731737.1	320	0	0.00
CG16798	NP_610030.1	4	0	0.00	CG12279	NP_650227.1	148	0	0.00
CG10947	NP_610031.1	158	1	4.28	DNApol-theta	NP_524333.1	494	0	0.00
CG10949	NP_610032.1	71	0	0.00	Men	NP_524880.2	534	2	2.53
Arpc2	NP_610033.1	525	0	0.00	Ugt37A2	NP_650228.1	53	0	0.00
CG15130	NP_610034.2	40	0	0.00	Ugt37A3	NP_650229.2	137	0	0.00
CG31688	NP_610035.4	23	0	0.00	beat-Vc	NP_001356887.1	38	0	0.00
CG31683	NP_001260626.1	305	1	2.22	CG31345	NP_731744.1	310	2	4.36
CG31687	NP_001163022.1	663	0	0.00	beat-Va	NP_001189214.1	50	0	0.00
CG18858	NP_652700.1	248	1	2.73	CG10126	NP_788664.2	291	1	2.32
Cdc23	NP_610036.2	715	0	0.00	d-cup	NP_650235.1	405	2	3.34

CG2493	NP_610037.1	186	2	7.27	CG10909	NP_650236.1	975	0	0.00
CG43233	NP_001246106.1	0	0	#DIV/0!	beat-Vb	NP_731748.1	37	0	0.00
CG34007	NP_001286104.1	0	0	#DIV/0!	grsm	NP_731749.3	269	1	2.51
CG31677	NP_724270.1	0	0	#DIV/0!	Spc25	NP_650239.1	181	0	0.00
CG15475	NP_610039.1	765	2	1.77	Cyp304a1	NP_731751.1	129	0	0.00
CG17472	NP_610040.1	241	2	5.61	CG14384	NP_650241.1	293	0	0.00
CG31680	NP_724273.1	78	1	8.67	CG7381	NP_001262514.1	83	0	0.00
[truncated: 1,637,797 more chars]
